# Global, regional, and national under-5 mortality, adult mortality, age-specific mortality, and life expectancy, 1970–2016: a systematic analysis for the Global Burden of Disease Study 2016

**DOI:** 10.1016/S0140-6736(17)31833-0

**Published:** 2017-09-16

**Authors:** GBD 2016 Mortality Collaborators, Amanuel Alemu Abajobir, Kalkidan Hassen Abate, Cristiana Abbafati, Kaja M Abbas, Foad Abd-Allah, Semaw Ferede Abera, Haftom Niguse Abraha, Laith J Abu-Raddad, Niveen M E Abu-Rmeileh, Isaac Akinkunmi Adedeji, Rufus Adesoji Adedoyin, Ifedayo Morayo O Adetifa, Olatunji Adetokunboh, Ashkan Afshin, Rakesh Aggarwal, Anurag Agrawal, Sutapa Agrawal, Aliasghar Ahmad Kiadaliri, Muktar Beshir Ahmed, Miloud Taki Eddine Aichour, Amani Nidhal Aichour, Ibthiel Aichour, Sneha Aiyar, Ali Shafqat Akanda, Tomi F Akinyemiju, Nadia Akseer, Faris Hasan Al Lami, Samer Alabed, Fares Alahdab, Ziyad Al-Aly, Khurshid Alam, Noore Alam, Deena Alasfoor, Robert William Aldridge, Kefyalew Addis Alene, Ayman Al-Eyadhy, Samia Alhabib, Raghib Ali, Reza Alizadeh-Navaei, Syed M Aljunid, Juma M Alkaabi, Ala'a Alkerwi, François Alla, Shalini D Allam, Peter Allebeck, Rajaa Al-Raddadi, Ubai Alsharif, Khalid A Altirkawi, Nelson Alvis-Guzman, Azmeraw T Amare, Emmanuel A Ameh, Erfan Amini, Walid Ammar, Yaw Ampem Amoako, Nahla Anber, Catalina Liliana Andrei, Sofia Androudi, Hossein Ansari, Mustafa Geleto Ansha, Carl Abelardo T Antonio, Palwasha Anwari, Johan Ärnlöv, Megha Arora, Al Artaman, Krishna Kumar Aryal, Hamid Asayesh, Solomon Weldegebreal Asgedom, Rana Jawad Asghar, Reza Assadi, Ashagre Molla Assaye, Tesfay Mehari Atey, Sachin R Atre, Leticia Avila-Burgos, Euripide Frinel G Arthur Avokpaho, Ashish Awasthi, Tesleem Kayode Babalola, Umar Bacha, Alaa Badawi, Kalpana Balakrishnan, Shivanthi Balalla, Aleksandra Barac, Ryan M Barber, Miguel A Barboza, Suzanne L Barker-Collo, Till Bärnighausen, Simon Barquera, Lars Barregard, Lope H Barrero, Bernhard T Baune, Shahrzad Bazargan-Hejazi, Neeraj Bedi, Ettore Beghi, Yannick Béjot, Bayu Begashaw Bekele, Michelle L Bell, Aminu K Bello, Derrick A Bennett, James R Bennett, Isabela M Bensenor, Jennifer Benson, Adugnaw Berhane, Derbew Fikadu Berhe, Eduardo Bernabé, Mircea Beuran, Addisu Shunu Beyene, Neeraj Bhala, Anil Bhansali, Soumyadeep Bhaumik, Zulfiqar A Bhutta, Burcu Kucuk Bicer, Hassan Haghparast Bidgoli, Boris Bikbov, Charles Birungi, Stan Biryukov, Donal Bisanzio, Habtamu Mellie Bizuayehu, Peter Bjerregaard, Christopher D Blosser, Dube Jara Boneya, Soufiane Boufous, Rupert R A Bourne, Alexandra Brazinova, Nicholas J K Breitborde, Hermann Brenner, Traolach S Brugha, Gene Bukhman, Lemma Negesa Bulto Bulto, Blair Randal Bumgarner, Michael Burch, Zahid A Butt, Leah E Cahill, Lucero Cahuana-Hurtado, Ismael Ricardo Campos-Nonato, Josip Car, Mate Car, Rosario Cárdenas, David O Carpenter, Juan Jesus Carrero, Austin Carter, Carlos A Castañeda-Orjuela, Franz F Castro, Ruben Estanislao Castro, Ferrán Catalá-López, Honglei Chen, Peggy Pei-Chia Chiang, Mirriam Chibalabala, Vesper Hichilombwe Chisumpa, Abdulaal A Chitheer, Jee-Young Jasmine Choi, Hanne Christensen, Devasahayam Jesudas Christopher, Liliana G Ciobanu, Massimo Cirillo, Aaron J Cohen, Samantha M Colquhoun, Josef Coresh, Michael H Criqui, Elizabeth A Cromwell, John A Crump, Lalit Dandona, Rakhi Dandona, Paul I Dargan, José das Neves, Gail Davey, Dragos V Davitoiu, Kairat Davletov, Barbora de Courten, Diego De Leo, Louisa Degenhardt, Selina Deiparine, Robert P Dellavalle, Kebede Deribe, Amare Deribew, Don C Des Jarlais, Subhojit Dey, Samath D Dharmaratne, Mukesh K Dherani, Cesar Diaz-Torné, Eric L Ding, Priyanka Dixit, Shirin Djalalinia, Huyen Phuc Do, David Teye Doku, Christl Ann Donnelly, Kadine Priscila Bender dos Santos, Dirk Douwes-Schultz, Tim R Driscoll, Leilei Duan, Manisha Dubey, Bruce Bartholow Duncan, Laxmi Kant Dwivedi, Hedyeh Ebrahimi, Charbel El Bcheraoui, Christian Lycke Ellingsen, Ahmadali Enayati, Aman Yesuf Endries, Sergey Petrovich Ermakov, Setegn Eshetie, Babak Eshrati, Sharareh Eskandarieh, Alireza Esteghamati, Kara Estep, Fanuel Belayneh Bekele Fanuel, André Faro, Maryam S Farvid, Farshad Farzadfar, Valery L Feigin, Seyed-Mohammad Fereshtehnejad, Jefferson G Fernandes, João C Fernandes, Tesfaye Regassa Feyissa, Irina Filip, Florian Fischer, Nataliya Foigt, Kyle J Foreman, Tahvi Frank, Richard C Franklin, Maya Fraser, Joseph Friedman, Joseph J Frostad, Nancy Fullman, Thomas Fürst, Joao M Furtado, Neal D Futran, Emmanuela Gakidou, Ketevan Gambashidze, Amiran Gamkrelidze, Fortuné Gbètoho Gankpé, Alberto L Garcia-Basteiro, Gebremedhin Berhe Gebregergs, Tsegaye Tewelde Gebrehiwot, Kahsu Gebrekirstos Gebrekidan, Mengistu Welday Gebremichael, Amha Admasie Gelaye, Johanna M Geleijnse, Bikila Lencha Gemechu, Kasiye Shiferaw Gemechu, Ricard Genova-Maleras, Hailay Abrha Gesesew, Peter W Gething, Katherine B Gibney, Paramjit Singh Gill, Richard F Gillum, Ababi Zergaw Giref, Bedilu Weji Girma, Giorgia Giussani, Shifalika Goenka, Beatriz Gomez, Philimon N Gona, Sameer Vali Gopalani, Alessandra Carvalho Goulart, Nicholas Graetz, Harish Chander Gugnani, Prakash C Gupta, Rahul Gupta, Rajeev Gupta, Tanush Gupta, Vipin Gupta, Juanita A Haagsma, Nima Hafezi-Nejad, Alex Hakuzimana, Yara A Halasa, Randah Ribhi Hamadeh, Mitiku Teshome Hambisa, Samer Hamidi, Mouhanad Hammami, Jamie Hancock, Alexis J Handal, Graeme J Hankey, Yuantao Hao, Hilda L Harb, Habtamu Abera Hareri, Sivadasanpillai Harikrishnan, Josep Maria Haro, Mohammad Sadegh Hassanvand, Rasmus Havmoeller, Roderick J Hay, Simon I Hay, Fei He, Ileana Beatriz Heredia-Pi, Claudiu Herteliu, Esayas Haregot Hilawe, Hans W Hoek, Nobuyuki Horita, H Dean Hosgood, Sorin Hostiuc, Peter J Hotez, Damian G Hoy, Mohamed Hsairi, Aung Soe Htet, Guoqing Hu, John J Huang, Hsiang Huang, Kim Moesgaard Iburg, Ehimario Uche Igumbor, Bogdan Vasile Ileanu, Manami Inoue, Asnake Ararsa Irenso, Caleb M S Irvine, Sheikh Mohammed Shariful Islam, Nazrul Islam, Kathryn H Jacobsen, Thomas Jaenisch, Nader Jahanmehr, Mihajlo B Jakovljevic, Mehdi Javanbakht, Achala Upendra Jayatilleke, Panniyammakal Jeemon, Paul N Jensen, Vivekanand Jha, Ye Jin, Denny John, Oommen John, Sarah Charlotte Johnson, Jost B Jonas, Mikk Jürisson, Zubair Kabir, Rajendra Kadel, Amaha Kahsay, Yogeshwar Kalkonde, Ritul Kamal, Haidong Kan, André Karch, Corine Kakizi Karema, Seyed M Karimi, Ganesan Karthikeyan, Amir Kasaeian, Nigussie Assefa Kassaw, Nicholas J Kassebaum, Anshul Kastor, Srinivasa Vittal Katikireddi, Anil Kaul, Norito Kawakami, Konstantin Kazanjan, Peter Njenga Keiyoro, Sefonias Getachew Kelbore, Andrew Haddon Kemp, Andre Pascal Kengne, Andre Keren, Maia Kereselidze, Chandrasekharan Nair Kesavachandran, Ezra Belay Ketema, Yousef Saleh Khader, Ibrahim A Khalil, Ejaz Ahmad Khan, Gulfaraz Khan, Young-Ho Khang, Sahil Khera, Abdullah Tawfih Abdullah Khoja, Mohammad Hossein Khosravi, Getiye Dejenu Kibret, Christian Kieling, Yun Jin Kim, Cho-il Kim, Daniel Kim, Pauline Kim, Sungroul Kim, Ruth W Kimokoti, Yohannes Kinfu, Sami Kishawi, Niranjan Kissoon, Mika Kivimaki, Ann Kristin Knudsen, Yoshihiro Kokubo, Jacek A Kopec, Soewarta Kosen, Parvaiz A Koul, Ai Koyanagi, Michael Kravchenko, Kristopher J Krohn, Barthelemy Kuate Defo, Ernst J Kuipers, Xie Rachel Kulikoff, Veena S Kulkarni, G Anil Kumar, Pushpendra Kumar, Fekede Asefa Kumsa, Michael Kutz, Carl Lachat, Abraham K Lagat, Anton Carl Jonas Lager, Dharmesh Kumar Lal, Ratilal Lalloo, Nkurunziza Lambert, Qing Lan, Van C Lansingh, Heidi J Larson, Anders Larsson, Dennis Odai Laryea, Pablo M Lavados, Avula Laxmaiah, Paul H Lee, James Leigh, Janni Leung, Ricky Leung, Miriam Levi, Yongmei Li, Yu Liao, Misgan Legesse Liben, Stephen S Lim, Shai Linn, Steven E Lipshultz, Shiwei Liu, Rakesh Lodha, Giancarlo Logroscino, Alan D Lopez, Scott A Lorch, Stefan Lorkowski, Paulo A Lotufo, Rafael Lozano, Raimundas Lunevicius, Ronan A Lyons, Stefan Ma, Erlyn RachelleKing Macarayan, Isis Eloah Machado, Mark T Mackay, Mohammed Magdy Abd El Razek, Carlos Magis-Rodriguez, Mahdi Mahdavi, Marek Majdan, Reza Majdzadeh, Azeem Majeed, Reza Malekzadeh, Rajesh Malhotra, Deborah Carvalho Malta, Lorenzo G Mantovani, Tsegahun Manyazewal, Chabila C Mapoma, Laurie B Marczak, Guy B Marks, Elena Alvarez Martin, Jose Martinez-Raga, Francisco Rogerlândio Martins-Melo, João Massano, Pallab K Maulik, Bongani M Mayosi, Mohsen Mazidi, Colm McAlinden, Stephen Theodore McGarvey, John J McGrath, Martin McKee, Suresh Mehata, Man Mohan Mehndiratta, Kala M Mehta, Toni Meier, Tefera Chane Mekonnen, Kidanu Gebremariam Meles, Peter Memiah, Ziad A Memish, Walter Mendoza, Melkamu Merid Mengesha, Mubarek Abera Mengistie, Desalegn Tadese Mengistu, Geetha R Menon, Bereket Gebremichael Menota, George A Mensah, Tuomo J Meretoja, Atte Meretoja, Haftay Berhane Mezgebe, Renata Micha, Joseph Mikesell, Ted R Miller, Edward J Mills, Shawn Minnig, Mojde Mirarefin, Erkin M Mirrakhimov, Awoke Misganaw, Shiva Raj Mishra, Karzan Abdulmuhsin Mohammad, Alireza Mohammadi, Kedir Endris Mohammed, Shafiu Mohammed, Murali B V Mohan, Sanjay K Mohanty, Ali H Mokdad, Sarah K Mollenkopf, Mariam Molokhia, Lorenzo Monasta, Julio Cesar Montañez Hernandez, Marcella Montico, Meghan D Mooney, Ami R Moore, Maziar Moradi-Lakeh, Paula Moraga, Lidia Morawska, Rintaro Mori, Shane D Morrison, Kalayu Birhane Mruts, Ulrich O Mueller, Erin Mullany, Kate Muller, Christopher J L Murray, Gudlavalleti Venkata Satyanarayana Murthy, Srinivas Murthy, Kamarul Imran Musa, Jean B Nachega, Chie Nagata, Gabriele Nagel, Mohsen Naghavi, Kovin S Naidoo, Lipika Nanda, Vinay Nangia, Bruno Ramos Nascimento, Gopalakrishnan Natarajan, Ionut Negoi, Cuong Tat Nguyen, Quyen Le Nguyen, Trang Huyen Nguyen, Grant Nguyen, Dina Nur Anggraini Ningrum, Muhammad Imran Nisar, Marika Nomura, Vuong Minh Nong, Ole F Norheim, Bo Norrving, Jean Jacques N Noubiap, Luke Nyakarahuka, Martin J O'Donnell, Carla Makhlouf Obermeyer, Felix Akpojene Ogbo, In-Hwan Oh, Anselm Okoro, Olanrewaju Oladimeji, Andrew Toyin Olagunju, Bolajoko Olubukunola Olusanya, Jacob Olusegun Olusanya, Eyal Oren, Alberto Ortiz, Aaron Osgood-Zimmerman, Erika Ota, Mayowa O Owolabi, Abayomi Samuel Oyekale, Mahesh PA, Rosana E Pacella, Smita Pakhale, Adrian Pana, Basant Kumar Panda, Songhomitra Panda-Jonas, Eun-Kee Park, Mahboubeh Parsaeian, Tejas Patel, Scott B Patten, George C Patton, Deepak Paudel, David M Pereira, Rogelio Perez-Padilla, Fernando Perez-Ruiz, Norberto Perico, Aslam Pervaiz, Konrad Pesudovs, Carrie Beth Peterson, William Arthur Petri, Max Petzold, Michael Robert Phillips, Frédéric B Piel, David M Pigott, Farhad Pishgar, Dietrich Plass, Suzanne Polinder, Svetlana Popova, Maarten J Postma, Richie G Poulton, Farshad Pourmalek, Narayan Prasad, Manorama Purwar, Mostafa Qorbani, Beatriz Paulina Ayala Quintanilla, Rynaz H S Rabiee, Amir Radfar, Anwar Rafay, Afarin Rahimi-Movaghar, Vafa Rahimi-Movaghar, Mohammad Hifz Ur Rahman, Sajjad Ur Rahman, Mahfuzar Rahman, Rajesh Kumar Rai, Sasa Rajsic, Usha Ram, Saleem M Rana, Chhabi Lal Ranabhat, Paturi Vishnupriya Rao, Salman Rawaf, Sarah E Ray, Maria Albertina Santiago Rego, Jürgen Rehm, Robert C Reiner, Giuseppe Remuzzi, Andre M N Renzaho, Serge Resnikoff, Satar Rezaei, Mohammad Sadegh Rezai, Antonio L Ribeiro, Jacqueline Castillo Rivas, Mohammad Bagher Rokni, Luca Ronfani, Gholamreza Roshandel, Gregory A Roth, Dietrich Rothenbacher, Ambuj Roy, Enrico Rubagotti, George Mugambage Ruhago, Soheil Saadat, Yogesh Damodar Sabde, Perminder S Sachdev, Nafis Sadat, Mahdi Safdarian, Sare Safi, Saeid Safiri, Rajesh Sagar, Ramesh Sahathevan, Amirhossein Sahebkar, Mohammad Ali Sahraian, Joseph Salama, Payman Salamati, Joshua A Salomon, Sundeep Santosh Salvi, Abdallah M Samy, Juan Ramon Sanabria, Maria Dolores Sanchez-Niño, Itamar S Santos, Milena M Santric Milicevic, Rodrigo Sarmiento-Suarez, Benn Sartorius, Maheswar Satpathy, Monika Sawhney, Sonia Saxena, Mete I Saylan, Maria Inês Schmidt, Ione J C Schneider, Sam Schulhofer-Wohl, Aletta E Schutte, David C Schwebel, Falk Schwendicke, Soraya Seedat, Abdulbasit Musa Seid, Sadaf G Sepanlou, Edson E Servan-Mori, Katya Anne Shackelford, Amira Shaheen, Saeid Shahraz, Masood Ali Shaikh, Mansour Shamsipour, Morteza Shamsizadeh, Jayendra Sharma, Rajesh Sharma, Jun She, Jiabin Shen, Balakrishna P Shetty, Peilin Shi, Kenji Shibuya, Girma Temam Shifa, Mika Shigematsu, Rahman Shiri, Ivy Shiue, Mark G Shrime, Inga Dora Sigfusdottir, Donald H Silberberg, Naris Silpakit, Diego Augusto Santos Silva, João Pedro Silva, Dayane Gabriele Alves Silveira, Shireen Sindi, Jasvinder A Singh, Prashant Kumar Singh, Abhishek Singh, Virendra Singh, Dhirendra Narain Sinha, Katarzyna A Kissimova- Skarbek, Eirini Skiadaresi, Amber Sligar, David L Smith, Badr H A Sobaih, Eugene Sobngwi, Samir Soneji, Joan B Soriano, Chandrashekhar T Sreeramareddy, Vinay Srinivasan, Vasiliki Stathopoulou, Nicholas Steel, Dan J Stein, Caitlyn Steiner, Heidi Stöckl, Mark Andrew Stokes, Mark Strong, Muawiyyah Babale Sufiyan, Rizwan Abdulkader Suliankatchi, Bruno F Sunguya, Patrick J Sur, Soumya Swaminathan, Bryan L Sykes, Cassandra E I Szoeke, Rafael Tabarés-Seisdedos, Santosh Kumar Tadakamadla, Fentaw Tadese, Nikhil Tandon, David Tanne, Musharaf Tarajia, Mohammad Tavakkoli, Nuno Taveira, Arash Tehrani-Banihashemi, Tesfalidet Tekelab, Dejen Yemane Tekle, Mohamad-Hani Temsah, Abdullah Sulieman Terkawi, Cheru Leshargie Tesema, Belay Tesssema, Andrew Theis, Nihal Thomas, Alex H Thompson, Alan J Thomson, Amanda G Thrift, Tenaw Yimer Tiruye, Ruoyan Tobe-Gai, Marcello Tonelli, Roman Topor-Madry, Fotis Topouzis, Miguel Tortajada, Bach Xuan Tran, Thomas Truelsen, Ulises Trujillo, Nikolaos Tsilimparis, Kald Beshir Tuem, Emin Murat Tuzcu, Stefanos Tyrovolas, Kingsley Nnanna Ukwaja, Eduardo A Undurraga, Olalekan A Uthman, Benjamin S Chudi Uzochukwu, Job F M van Boven, Yuri Y Varakin, Santosh Varughese, Tommi Vasankari, Ana Maria Nogales Vasconcelos, Ilais Moreno Velasquez, Narayanaswamy Venketasubramanian, Ramesh Vidavalur, Francesco S Violante, Abhishek Vishnu, Sergey K Vladimirov, Vasiliy Victorovich Vlassov, Stein Emil Vollset, Theo Vos, Jillian L Waid, Tolassa Wakayo, Haidong Wang, Yuan-Pang Wang, Scott Weichenthal, Elisabete Weiderpass, Robert G Weintraub, Andrea Werdecker, Joshua Wesana, Tissa Wijeratne, James D Wilkinson, Charles Shey Wiysonge, Belete Getahun Woldeyes, Charles D A Wolfe, Abdulhalik Workicho, Shimelash Bitew Workie, Denis Xavier, Gelin Xu, Mohsen Yaghoubi, Bereket Yakob, Ayalnesh Zemene Yalew, Lijing L Yan, Yuichiro Yano, Mehdi Yaseri, Pengpeng Ye, Hassen Hamid Yimam, Paul Yip, Biruck Desalegn Yirsaw, Naohiro Yonemoto, Seok-Jun Yoon, Marcel Yotebieng, Mustafa Z Younis, Zoubida Zaidi, Maysaa El Sayed Zaki, Hajo Zeeb, Zerihun Menlkalew Zenebe, Taddese Alemu Zerfu, Anthony Lin Zhang, Xueying Zhang, Sanjay Zodpey, Liesl Joanna Zuhlke

## Abstract

**Background:**

Detailed assessments of mortality patterns, particularly age-specific mortality, represent a crucial input that enables health systems to target interventions to specific populations. Understanding how all-cause mortality has changed with respect to development status can identify exemplars for best practice. To accomplish this, the Global Burden of Diseases, Injuries, and Risk Factors Study 2016 (GBD 2016) estimated age-specific and sex-specific all-cause mortality between 1970 and 2016 for 195 countries and territories and at the subnational level for the five countries with a population greater than 200 million in 2016.

**Methods:**

We have evaluated how well civil registration systems captured deaths using a set of demographic methods called death distribution methods for adults and from consideration of survey and census data for children younger than 5 years. We generated an overall assessment of completeness of registration of deaths by dividing registered deaths in each location-year by our estimate of all-age deaths generated from our overall estimation process. For 163 locations, including subnational units in countries with a population greater than 200 million with complete vital registration (VR) systems, our estimates were largely driven by the observed data, with corrections for small fluctuations in numbers and estimation for recent years where there were lags in data reporting (lags were variable by location, generally between 1 year and 6 years). For other locations, we took advantage of different data sources available to measure under-5 mortality rates (U5MR) using complete birth histories, summary birth histories, and incomplete VR with adjustments; we measured adult mortality rate (the probability of death in individuals aged 15–60 years) using adjusted incomplete VR, sibling histories, and household death recall. We used the U5MR and adult mortality rate, together with crude death rate due to HIV in the GBD model life table system, to estimate age-specific and sex-specific death rates for each location-year. Using various international databases, we identified fatal discontinuities, which we defined as increases in the death rate of more than one death per million, resulting from conflict and terrorism, natural disasters, major transport or technological accidents, and a subset of epidemic infectious diseases; these were added to estimates in the relevant years. In 47 countries with an identified peak adult prevalence for HIV/AIDS of more than 0·5% and where VR systems were less than 65% complete, we informed our estimates of age-sex-specific mortality using the Estimation and Projection Package (EPP)-Spectrum model fitted to national HIV/AIDS prevalence surveys and antenatal clinic serosurveillance systems. We estimated stillbirths, early neonatal, late neonatal, and childhood mortality using both survey and VR data in spatiotemporal Gaussian process regression models. We estimated abridged life tables for all location-years using age-specific death rates. We grouped locations into development quintiles based on the Socio-demographic Index (SDI) and analysed mortality trends by quintile. Using spline regression, we estimated the expected mortality rate for each age-sex group as a function of SDI. We identified countries with higher life expectancy than expected by comparing observed life expectancy to anticipated life expectancy on the basis of development status alone.

**Findings:**

Completeness in the registration of deaths increased from 28% in 1970 to a peak of 45% in 2013; completeness was lower after 2013 because of lags in reporting. Total deaths in children younger than 5 years decreased from 1970 to 2016, and slower decreases occurred at ages 5–24 years. By contrast, numbers of adult deaths increased in each 5-year age bracket above the age of 25 years. The distribution of annualised rates of change in age-specific mortality rate differed over the period 2000 to 2016 compared with earlier decades: increasing annualised rates of change were less frequent, although rising annualised rates of change still occurred in some locations, particularly for adolescent and younger adult age groups. Rates of stillbirths and under-5 mortality both decreased globally from 1970. Evidence for global convergence of death rates was mixed; although the absolute difference between age-standardised death rates narrowed between countries at the lowest and highest levels of SDI, the ratio of these death rates—a measure of relative inequality—increased slightly. There was a strong shift between 1970 and 2016 toward higher life expectancy, most noticeably at higher levels of SDI. Among countries with populations greater than 1 million in 2016, life expectancy at birth was highest for women in Japan, at 86·9 years (95% UI 86·7–87·2), and for men in Singapore, at 81·3 years (78·8–83·7) in 2016. Male life expectancy was generally lower than female life expectancy between 1970 and 2016, and the gap between male and female life expectancy increased with progression to higher levels of SDI. Some countries with exceptional health performance in 1990 in terms of the difference in observed to expected life expectancy at birth had slower progress on the same measure in 2016.

**Interpretation:**

Globally, mortality rates have decreased across all age groups over the past five decades, with the largest improvements occurring among children younger than 5 years. However, at the national level, considerable heterogeneity remains in terms of both level and rate of changes in age-specific mortality; increases in mortality for certain age groups occurred in some locations. We found evidence that the absolute gap between countries in age-specific death rates has declined, although the relative gap for some age-sex groups increased. Countries that now lead in terms of having higher observed life expectancy than that expected on the basis of development alone, or locations that have either increased this advantage or rapidly decreased the deficit from expected levels, could provide insight into the means to accelerate progress in nations where progress has stalled.

**Funding:**

Bill & Melinda Gates Foundation, and the National Institute on Aging and the National Institute of Mental Health of the National Institutes of Health.

**Research in context**

**Evidence before this study**

Three organisations periodically report on some dimensions of all-cause mortality: the UN Population Division (UNPD) produces revised estimates of age-specific mortality for 5-year intervals every 2 years; the United States Census Bureau reports periodically on life expectancy; and WHO produces estimates of life expectancy on a 2-year cycle, although these estimates are substantially based on those from the UNPD. The Global Burden of Diseases, Injuries, and Risk Factors Study (GBD) produces the only annual assessment of trends in age-specific mortality for all locations with a population over 50 000 from 1970 to the present that is compliant with the Guidelines for Accurate and Transparent Health Estimates Reporting (GATHER) standard.

**Added value of this study**

This study improves on the GBD 2015 assessment in 11 substantial ways. First, new data have been incorporated; at the national level we included 171 new location-years of vital registration data, 41 new survey sources for under-5 mortality, eight new survey sources for adult mortality, and 15 667 new empirical life tables. New prevalence data were used to revise HIV/AIDS estimates and the fatal discontinuities database was updated. Second, we incorporated a new systematic analysis of data on educational attainment in reproductive-aged women, which is an important covariate for the estimation of under-5 mortality, and for educational attainment in the population older than 15 years, which is a covariate for adult mortality models. The new systematic analysis improved estimates, particularly for census and survey data that reported on categories of educational attainment such as primary school completion. Third, in previous GBD studies we used UNPD estimates of total fertility rate (TFR) and births. For this study, we did a systematic analysis of fertility data to estimate time series of TFR for each country and subnational location in the GBD study. Birth numbers used to compute the number of child deaths for GBD 2016 were estimated on the basis of TFR. These modifications led to substantial changes in estimated birth numbers in some countries and at the global level. Fourth, for the analysis of expected death rates based on the Socio-demographic Index (SDI), we updated SDI estimates and extended the SDI time series back to 1970 and used Gaussian process regression to fit the expected death rate for each age-sex group. Fifth, new subnational assessments for Indonesia by province and local government areas in England were included in the analysis. Sixth, in the modelling of HIV/AIDS, we replaced an assumed antiretroviral therapy (ART) allocation to those most in need with an empirical pattern derived from household surveys. This captured the allocation of ART in some cases to individuals who do not necessarily qualify in national guidelines. Seventh, given the interest in civil registration and vital statistics, we reported our estimated completeness of vital registration data for each location and year. Globally, completeness in the registration of deaths increased from 28% in 1970 to a peak of 45% in 2013. Eighth, since GBD 2010, we have estimated all-cause mortality from 1970 to the most recent estimation year. In this study, we present the full time series of these results for the first time. Ninth, given the rising interest in adverse trends in mortality for selected age groups—such as the increase in mortality in middle age in some locations—we focused on presenting age-specific trends in addition to summary measures of mortality such as life expectancy. Tenth, we used the time series of age-specific mortality rates to assess whether there has been convergence or divergence in either absolute or relative mortality rates. Finally, we formally assessed which countries had higher observed life expectancy than expected on the basis of their development status alone. These countries can potentially serve as exemplars on how to accelerate declines in mortality.

**Implications of all the available evidence**

The empirical basis for assessing age-specific mortality has improved; nearly 45% of deaths are now registered through civil registration and vital statistics and survey data provide measurements for child and adult mortality in other settings. These data show that there have been substantial improvements in life expectancy over the past 47 years in nearly all locations assessed by GBD. From our analysis, a new set of countries emerged as exemplars for achieving better than expected life expectancy for their level of development, including Ethiopia and Peru.

## Introduction

Mortality, particularly at younger ages, is a key measure of population health. Avoiding premature mortality from any cause is a crucial goal for every health system, and targets for mortality reduction are central in the development agenda for improving health.^1^, ^2^ In the era of the Millennium Development Goals (MDGs), reducing mortality rates among children was one of eight overall goals.^3^ In the current era of Sustainable Development Goals (SDGs), reducing neonatal and under-5 mortality remains a priority, accompanied by attention to reducing premature deaths among adults from non-communicable causes, road injuries, natural disasters, and other causes.^4^ As the global health agenda broadens, the need for up-to-date and accurate measurement of overall mortality continues to grow. Global interest in the convergence between death rates in countries with lower levels of development and those in countries at higher levels of development also adds value to the monitoring of age-specific mortality rates over the long term.^5^ Evidence of stagnation or reversals in mortality rates in specific age-sex groups in countries such as the USA and Mexico has also heightened interest in acquiring timely assessments of levels and trends in all-cause mortality.^6^, ^7^, ^8^

Age-specific mortality from all causes can be measured annually in locations with vital statistics from civil registration systems that capture more than 95% of all deaths. Incomplete civil registration data can also be used to monitor mortality if the completeness of reporting can be quantified. For countries with very incomplete or non-existent civil registration systems, age-specific mortality must be estimated from surveys, censuses, surveillance systems, and sample registration systems. Several regional groups regularly attempt to collate available mortality data, including Eurostat, the Organisation for Economic Co-operation and Development (OECD), and the Human Mortality Database. Fewer efforts attempt to estimate age-specific mortality rates based on some of the available data; these include the UN Population Division (UNPD),^9^ WHO,^10^ the United States Census Bureau (USCB),^11^ and the Global Burden of Diseases, Injuries, and Risk Factors Study (GBD). The UNPD provides updated demographic estimates, for 5-year intervals, every 2 years; WHO provides annual life tables for 194 countries for the years 2000–15 with episodic updates; currently the USCB provides demographic estimates and projections up to the year 2050 for 193 countries. In addition to these efforts to measure mortality across all age groups, the United Nations Interagency Group for Child Mortality (IGME) produces periodic assessments of mortality in children younger than 5 years for 195 countries.

Of these estimation efforts, the GBD study is unique. This study (GBD 2016) provides an annual update of the full time series from 1970 to the present for 195 countries or territories and for first administrative level disaggregations for countries with a population greater than 200 million, covering age-specific death rates and life table measures up to the age group 95 years or older. Estimates are based on statistical methods that yield 95% uncertainty intervals (UIs) for all age-specific mortality rates and summary life table measures. The GBD study is also the only effort that fulfils the Guidelines for Accurate and Transparent Health Estimates Reporting (GATHER) requirements for transparent and accurate reporting.^12^ In contrast to the UNPD, WHO, and USCB estimates, in the GBD study, mortality among adult age groups in many locations without civil registration is not estimated solely on the basis of mortality levels for children younger than 5 years. Finally, the GBD study is based on the application of a set of standardised methods to all locations in a consistent manner, enabling comparisons between locations and over time, whereas other efforts at mortality estimation frequently use different methods or approaches in different countries.^13^, ^14^, ^15^, ^16^

The primary objective of this study was to estimate all-cause mortality by age, sex, and location from 1970 to 2016. Compared with GBD 2015, the main changes that are reflected in this study include updates to data, methods, and presentation (Research in context panel). We use the time trend to 2016 to explore patterns by age and location, assess the convergence of absolute and relative mortality rates, and examine which countries have higher than expected life expectancy on the basis of their level of development using consistent methods and a comprehensively updated database.^17^ Because we re-estimate the entire time series from 1970 to 2016 for all-cause mortality, additions to data and revisions to methods mean that results from this study supersede all prior GBD results for all-cause mortality.

## Methods

### Overview

The goal of this analysis was to use all available data sources that met quality criteria to estimate mortality rates with 95% UIs for 23 age groups, by sex, for 195 locations from 1970 to 2016 with subnational disaggregation for the five countries with a population greater than 200 million in 2016. The estimation process was complex because of the diversity of data types that provide relevant information on death rates in different age groups. Here we provide a broad explanation of the GBD 2016 mortality analysis with an emphasis on the challenges these methods address, while the appendix provides detailed descriptions of each step in the analytical process.

In general, locations can be divided into two groups: 80 countries and territories with a civil registration system or sample registration system that captures more than 95% of all deaths (complete vital registration [VR]) and the remaining 115 countries or territories. For countries with complete VR, there are two main measurement challenges: dealing with problems of small numbers for some age-sex groups, and lags in the reporting of VR data that mean generated estimates for the most recent year must be estimated from data reported 1–5 years previously. To account for lags in data, we used models with covariates and spatiotemporal effects to estimate the years since the last measurement. In the remaining 115 countries and territories, our modelling process took advantage of the greater volume of survey and census data available for measuring under-5 mortality rate (U5MR) compared with the lower volumes of data, primarily from sibling histories and incomplete VR, for mortality in adults aged 15 to 60 years (45q15). We used the available data for U5MR, 45q15, and covariates to generate a best estimate with uncertainty for these quantities in each location-year. Building on a decades-long tradition in demographic estimation, we estimate age-sex specific death rates for a location-year using information on under-5 child mortality, adult mortality, crude death rate due to HIV, and a set of expected associations with death rates in each age-sex group—called a model life table.^18^, ^19^, ^20^ In previous analyses, the GBD model life tables have been shown to perform better in predicting age-specific mortality than have other model life table systems.^20^

The modelling approach for countries without complete VR was modified to deal with two classes of events that were not well captured by the demographic process of estimating under-5 and adult mortality by use of model life tables: fatal discontinuities and locations with large HIV/AIDS epidemics. Fatal discontinuities are abrupt changes in death rates related to conflicts and terrorism, disasters, or acute epidemics such as Ebola virus disease. We use data from various databases tracking these mortality events to modify estimates of death rates made from data excluding these events. Second, in the 47 countries with VR systems that are less than 65% complete, and where the peak prevalence of the HIV/AIDS epidemic reached more than 0·5%, the rapid increases in death rates from HIV/AIDS, particularly in younger adults (aged 15–49 years), were not well-captured by the standard demographic estimation model. For these countries, we used a modelling process that also uses information on the prevalence of HIV/AIDS from surveys and surveillance as a further input.

As with the previous iteration of the GBD study, this analysis adheres to GATHER standards developed by WHO and others.^12^ A table detailing our mechanism for adhering to GATHER is included in section 8 of the appendix (p 77); statistical code used in the entire process is available through an online repository. Analyses were done with Python versions 2.5.4 and 2.7.3, Stata version 13.1, or R version 3.1.2.

### Geographic units and time periods

The GBD study organises geographic units, or locations, by use of a set of hierarchical categories, beginning with seven super-regions; 21 regions are nested within those super-regions; and 195 countries or territories within the 21 regions (appendix section 1, p 4). For GBD 2016, new subnational assessments were added for Indonesia by province and England by local government areas. In this Global Health Metrics paper, we present data from subnational assessments for the five countries with a population greater than 200 million in 2016: Brazil, China, India, Indonesia, and the USA. Detailed subnational assessments will be reported in separate studies or reports; appendix section 1 (p 4) provides a description of all subnational assessments included in the analytical phase for GBD 2016. All-cause mortality covers the period 1970 to 2016; online data visualisation tools are available that provide results for each year of estimation in addition to what is presented here and in the appendix (p 4).

### Completeness of VR

Many countries operate civil registration systems to register births and deaths, with causes of death certified by a medical doctor; individual records are tabulated to produce annual vital statistics on births and deaths from these civil registration systems. VR data thus refers to data sourced from civil registration and vital statistics systems; India, Pakistan, and Bangladesh operate sample registration systems that collect data from a representative sample of communities in those countries. For all VR systems and sample registration systems, we have evaluated how well these systems have captured deaths in adults using a set of demographic methods called death distribution methods (DDM).^21^, ^22^ There are several well-described variants in DDM methods, each with particular advantages and limitations; in simulation studies, we found no real advantage for one method over the others.^21^ Additional details of our use of DDM are available in appendix section 2 (p 25). The completeness of registration systems in tabulating deaths for children younger than 5 years was based on consideration of survey and census data for the same populations. We generated an overall assessment of completeness of registration for all age groups combined by dividing registered deaths in each location-year by our estimate of all-age deaths generated from our overall estimation process.

### New data sources in GBD 2016

GBD 2016 estimated mortality from a comprehensive database that included both data from prior years (ie, 1970–2014) that were not available in previous GBD assessments and the most recent data sources, which might not yet have been publicly available. New data sources for GBD 2016 supplied an additional 171 location-years of VR data at the national level and 6902 location-years of VR and 45 sample registration years including all subnational locations, 13 complete birth history sources at the national level and three complete birth histories added for subnational locations, 28 national and 45 subnational summary birth history data sources, and eight national and six subnational sibling history surveys. The all-cause mortality databases used in GBD 2016 included a total of 165 674 point estimates of U5MR, 47 279 point estimates of 45q15, and 32 174 empirical life tables. The availability of data by year is summarised in appendix section 8 (p 159); data sources by location can also be identified with an online source tool.

### Estimating educational attainment, total fertility rate, and births

For GBD 2016, we substantially revised the systematic analysis of educational attainment. The new estimation is based on 2160 unique location-years of data for educational attainment. The method for estimating average years of schooling for categorical responses (such as primary school) was revised to reflect national and regional variation in school duration. Appendix section 4 (p 55) provides details on how educational attainment was estimated from these data sources, including the cross-validation of the modelling approach.

For GBD 2016, we did a systematic analysis of data on the total fertility rate (TFR); using surveys, census, and civil registration data, we identified 16 847 location-years of data for TFR. We used spatiotemporal Gaussian process regression (ST-GPR) to estimate the time trend of TFR in each location. Details of data and methods used in this systematic analysis are available in appendix section 3 (p 53). We estimated births for each location-year on the basis of the estimated TFR using the age patterns of fertility produced by the UNPD. Since births are an important input to under-5 mortality and stillbirth estimation, this change of method impacted the all-cause mortality and stillbirth estimates.

### Stillbirths, early neonatal, late neonatal, post-neonatal, and childhood mortality

The numbers of location-years for which any data from VR systems, surveys, and censuses were available to estimate the overall level of under-5 mortality between 1970 and 2016 are presented in the appendix (section 8 p 143). Point estimates of U5MR were generated with both direct and indirect estimation methods applied to survey responses of mothers; additional details of location-specific and year-specific measurements are available in appendix section 2 (p 7). We used ST-GPR to generate the full time series of estimates of U5MR for each location included in GBD 2016 after the application of a bias adjustment process to standardise across disparate data sources. This estimation process is described in detail in appendix section 2 (p 11).

We modelled the ratio of the stillbirth rate to the neonatal death rate using ST-GPR. This ratio was modelled as a function of educational attainment of women of reproductive age, a non-linear function of the neonatal death rate, location random effects, and random effects for specific data source types nested within each location. In the source data collated for our database, stillbirth was variously defined as fetal death after 20, 22, 24, 26, and 28 weeks' gestation, or weighing at least 500 g or 1000 g. Additionally, our database contained 1066 location-years for which no stillbirth definition was provided. We accounted for variation in stillbirth definitions in the original data, including no definition, by adjusting the data with scalars developed by Blencowe and colleagues.^23^ Further details of data source and definition adjustments and the development and use of covariates in the modelling process for stillbirth estimation are provided in appendix section 2 (p 21).

### Adult mortality estimation

Our estimates of adult mortality were developed using data from VR systems, censuses, and household surveys of the survival histories of siblings. The number of years for which data were available for adult mortality estimation by location—an indication of data completeness—are shown in appendix section 8 (p 143). Although sibling survival data have known biases, including selection bias, zero reporter bias, and recall bias,^24^, ^25^ they are one of the most important, and sometimes only, sources of information on the levels and trends of adult mortality rate in some locations. We used an improved sibling survival method to account for these biases as detailed by Obermeyer and colleagues.^25^ We applied this method to each new data source that contains sibling histories. We used ST-GPR with lag-distributed income per capita, educational attainment, and the estimated HIV/AIDS death rate as covariates to estimate adult mortality for each location.

### Age-specific mortality from GBD model life table system

Age-specific mortality among age groups older than 5 years was estimated from U5MR, 45q15, crude death rate due to HIV in corresponding age groups, and a location-year standard in the GBD model life table system. The location-year standard was selected from the database of 15 221 empirical life tables that met strict quality inclusion criteria (appendix section 2 p 39). The selection of the standard was designed to capture location-specific differences in the relative pattern of mortality over different ages.^17^ In locations with complete VR, the GBD model life table system standard was driven almost exclusively by the observed age pattern of mortality in that location. In locations without complete VR, the standard was derived from locations with high-quality life tables that had similar levels of U5MR and adult mortality. To capture regional differences in age patterns of mortality that might be driven by different causes of death, the selection of the standard gives preference to life tables that are proximate in space and time. The availability of empirical age patterns of mortality in the GBD database is summarised in appendix section 6 (p 80); the development of a standard age pattern of mortality from these data is summarised in appendix section 2 (p 7).

### Fatal discontinuities

In the GBD study a fatal discontinuity is defined as conflict and terrorism, a natural disaster, a major transport or technological accident, or one of a subset of epidemic infectious diseases that results in an abrupt increase in mortality greater than one death per million for all ages or that caused more than 100 deaths. We identified data for these discontinuities from a range of international databases;^26^, ^27^, ^28^, ^29^ specific sources are listed in appendix section 5 (p 59) and in the online source tool. Events in locations for which we do subnational assessments were geolocated to the appropriate subnational unit. When mortality from a fatal discontinuity was only available as a point estimate rather than as a range, we used the regional cause-specific UI to estimate uncertainty for that event. To supplement the temporal lags in these databases, we used additional searches of internet sources to find information on fatal discontinuities occurring in the most recent year. If conflicting data sources were identified for a single event, we used estimates sourced from VR systems over alternative estimates identified from internet searches. Ebola virus disease, cholera, and meningococcal meningitis were the subset of epidemic infectious diseases included as fatal discontinuities. Cholera and meningococcal meningitis were added as cause-specific fatal discontinuities for GBD 2016 because their current modelling strategy did not optimally capture epidemic mortality levels and trends, and they have contributed to substantial total fatalities in a given location-year. More information on these methods is listed in appendix section 5 (p 58).

### Estimating mortality in locations with high HIV/AIDS prevalence and without complete VR

In 47 countries with VR completeness less than 65% and where the peak adult prevalence of HIV/AIDS exceeded 0·5%, we modified our estimation approach to account for the specific temporal patterns of the HIV/AIDS epidemic and the concentration of mortality in younger adult age groups (ages 15–49 years). First, an HIV/AIDS-free age pattern of mortality (assuming zero deaths due to HIV/AIDS) was estimated using the estimation methods already described and setting the HIV/AIDS crude death rate to zero. We then add on to the HIV-free age pattern of mortality the excess mortality due to HIV/AIDS by using the age pattern of the relative risk of dying from HIV estimated in the UNAIDS Spectrum model (Spectrum).^30^ This step provides the implied HIV/AIDS-related mortality based on demographic sources. Second, we used a combination of the Estimation and Projection Package (EPP)^31^ and a modification of Spectrum^30^ to estimate the HIV/AIDS-related death rate using data on HIV/AIDS prevalence, prevention of mother-to-child transmission, ART coverage, and assumptions about the natural history of the disease embedded in the Spectrum model. For GBD 2016, to capture the allocation of ART to individuals who do not necessarily qualify in national guidelines, we replaced the prior assumption of ART allocation to those most in need with an empirical pattern derived from household surveys. For two countries, Myanmar and Cambodia, we used the UNAIDS estimates of incidence derived from the Asian Epidemic Model because the underlying prevalence data were not available to model with EPP-Spectrum. Third, our final estimate of HIV/AIDS-related mortality in these 47 countries was the average of the demographic source estimate and the HIV/AIDS natural history model estimate. We used both approaches because of the inconsistency in some countries between these sources that results from the large uncertainty associated with data for adult mortality derived only from sibling histories and the sensitivity of the EPP-Spectrum estimates of mortality to assumptions on progression of disease and death rates and scale-up of ART. Details of this multistep process, including safeguards to ensure that the HIV/AIDS-free estimate of mortality is not artificially depressed by overestimation of HIV/AIDS-related mortality, are described in appendix section 2 (p 46).

### Socio-demographic Index and expected mortality analysis

To move beyond binary descriptions such as developed and developing countries and assessments of development status based solely on income per capita, a Socio-demographic Index (SDI) was developed for GBD 2015. GBD 2015 used the Human Development Index method^32^ to compute SDI. SDI was calculated as the geometric mean of the rescaled values of lag-distributed income per capita (LDI), average years of education in the population older than 15 years, and TFR. The rescaling of each component variable was based on the minimum and maximum values observed for each component during the examined time period.^17^ Alternative approaches to equal weighting, such as principal components analysis, yielded results that were correlated (Pearson correlation 0·994, p<0·0001; more detail on the correlation used is listed in appendix section 6, p 62). In response to the addition of more subnational locations for GBD 2016—with further expansion anticipated in subsequent iterations—a fixed scale was developed for the rescaling of each component of SDI in GBD 2016. For each component, an index score of zero for a component represents the level below which we have not observed GDP per capita or educational attainment or above which we have not observed the TFR in known datasets. Maximum scores for educational attainment and LDI represent the maximum levels of the plateau in the relationship between each of the two components and the selected health outcomes, suggesting no additional benefit. Analogously, the maximum score for TFR represents the minimum level at which the relationship with the selected health outcomes plateaued. Detail for the development of these fixed-scale restrictions on SDI components is shown in appendix section 4 (p 55). The final SDI score for each location in each year was calculated as the geometric mean of the component scores for that location. The correlation between the SDI computed for GBD 2016 with these updated methods and that calculated for GBD 2015 was 0·977 (p<0·0001). Aggregate results are reported for the GBD 2016 study by locations grouped into quintiles; thresholds defining quintiles were selected on the basis of the distribution of SDI for the year 2016 for national-level GBD locations with populations greater than 1 million. The classification of locations into these quintiles is shown in appendix section 8 (p 98). Additional details of the development of this index are provided in appendix section 4 (p 57).

For GBD 2015, we characterised the relationship between SDI and death rates for every age-sex combination using first-order basis splines. For GBD 2016 we have improved the robustness and replicability of the estimation of this relationship. We used Gaussian process regression (GPR) with a linear prior for the mean function to estimate expected all-cause mortality rates for each age-sex group on the basis of SDI alone using data from 1970 to 2016. We examined the expected age-sex-specific mortality rates by SDI to confirm that mortality rates were consistent with known relationships (eg, Gompertz–Makeham law) and that there was no overlap in age-sex-specific mortality rates estimated across SDI levels. The set of expected age and sex mortality rates was used to generate a complete expected life table based on SDI. Finally, we made draw-level comparisons between observed life expectancy at birth (E_0_) and expected E_0_ based on SDI to identify location-years where this difference was statistically significant. These comparisons between expected values and observed levels for age-sex-specific mortality rates and life expectancy at birth were used to identify locations where improvements in life expectancy were greater than anticipated on the basis of SDI alone. We examined age-specific and sex-specific correlations between starting levels of mortality and annualised rates of change in mortality rate and the absolute change in the mortality rate to assess available evidence for either relative or absolute convergence in death rates, respectively.

### Uncertainty analysis

We have systematically estimated uncertainty throughout the all-cause mortality estimation process. For U5MR, completeness synthesis, and adult mortality rate estimation, uncertainty comes from sampling error by data source and non-sampling error. For the model life table step and the determination of HIV/AIDS-specific mortality, uncertainty comes from the sampling error and regression parameters in EPP and from uncertainty in the life table standard. We generated 1000 draws of each all-cause mortality metric including U5MR, adult mortality rate, age-specific mortality rates, overall mortality, and life expectancy. All analytical steps are linked at the draw level and uncertainty of all key mortality metrics are propagated throughout the all-cause mortality estimation process. The 95% uncertainty intervals were computed using the 2·5th and 97·5th percentile of the draw level values.

### Role of the funding source

The funders of the study had no role in study design, data collection, data analysis, data interpretation, or writing of the report. All authors had full access to the data in the study and had final responsibility for the decision to submit for publication.

## Results

### Civil registration and vital statistics completeness

**Figure 1:**
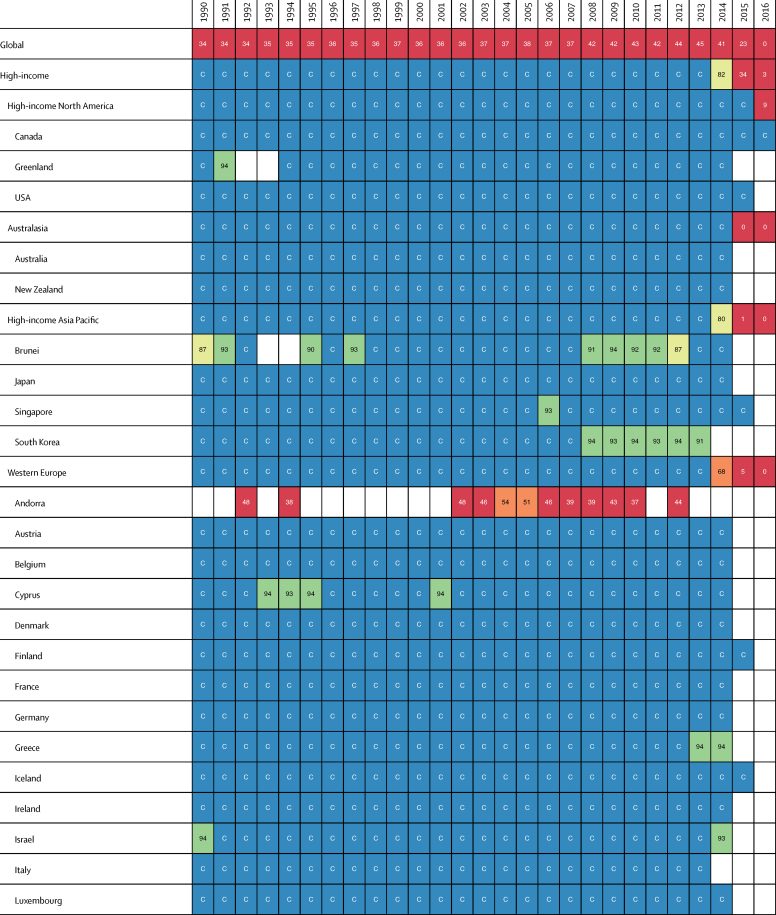

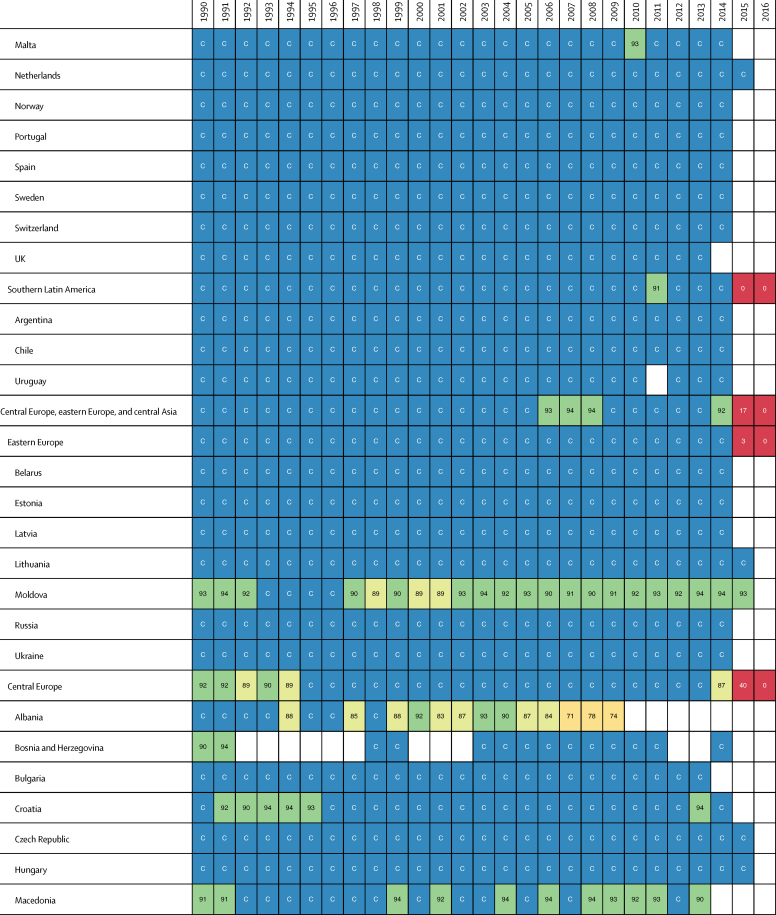

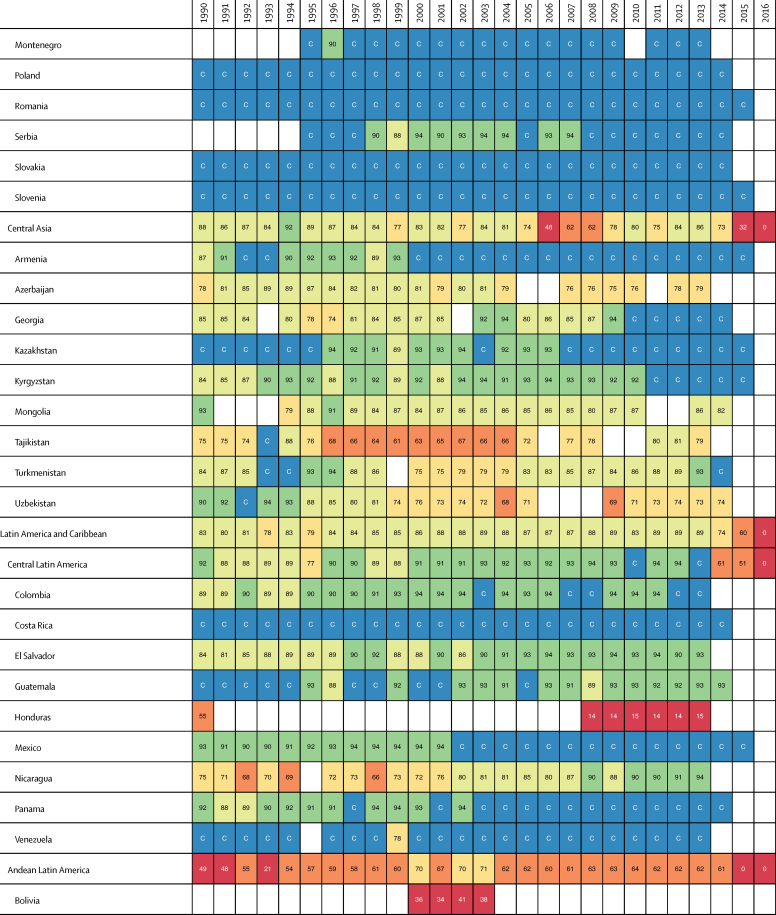

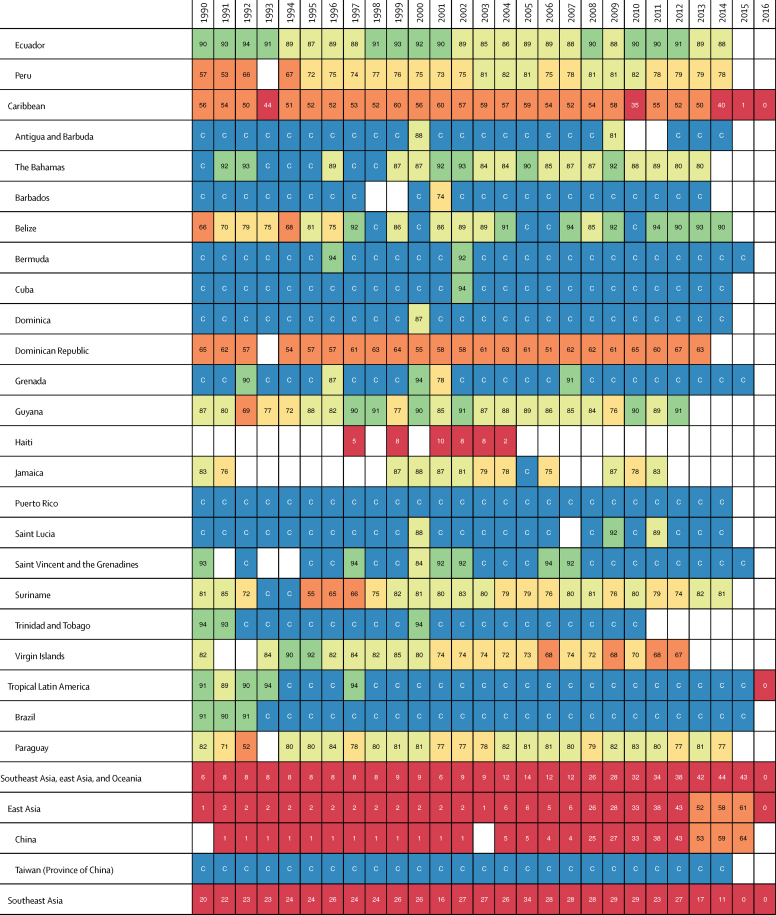

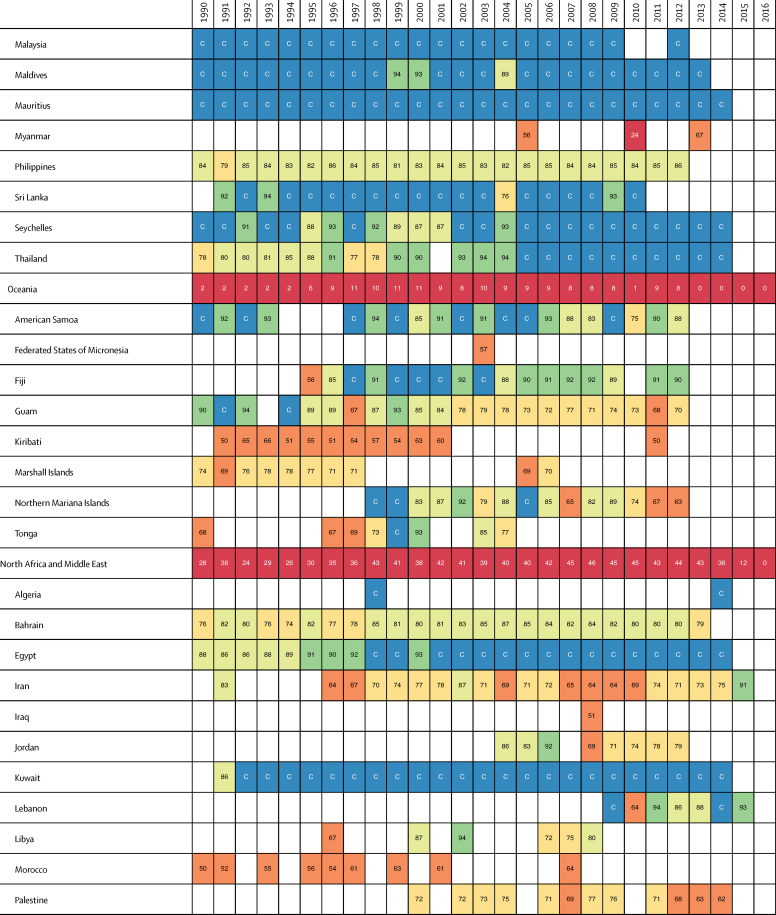

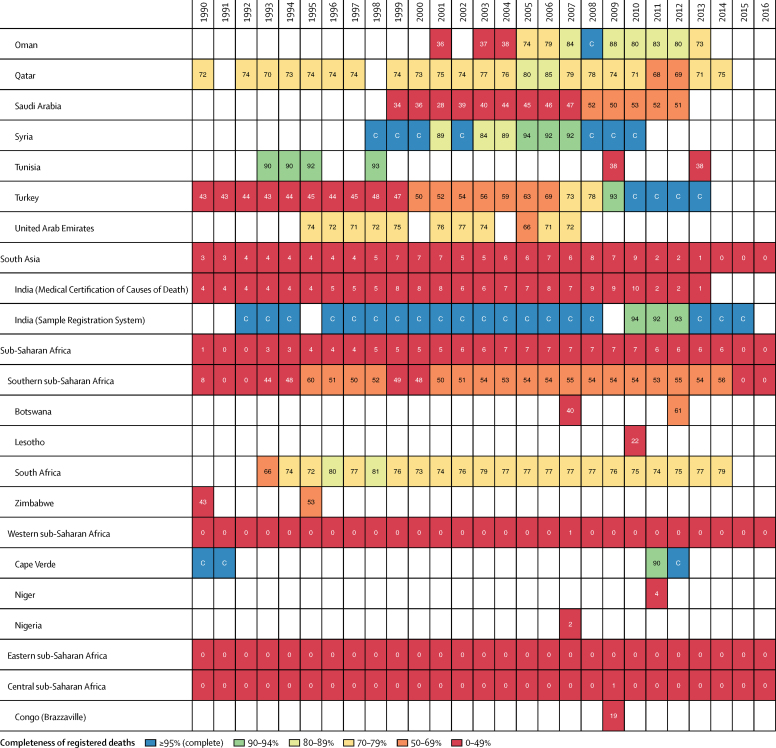
Estimated completeness of death registration, 1990–2016. Each square represents one location-year. Location-years in blue show complete vital registration systems. Shades of green show 80–95% completeness, whereas yellow, orange, and red show lower levels of completeness. Blank white squares indicate location-years without vital registration data in the GBD 2016 mortality database. Countries that are not shown have 0 years of VR data in the GBD 2016 mortality database.

At the global level, registration of deaths increased from 28% in 1970 to a peak of 45% in 2013. Death registration completeness declined after 2013 because of lags in reporting. Completeness of registration increased steadily, although slowly, at 0·35 percentage points per year on average through to 2008. The improvement since 2008 was largely driven by substantial increases in the registration of deaths in China, which reached 64% by 2015. Figure 1 shows the completeness of registration as a time series by location for 1990–2016. Registration was deemed complete (ie, more than 95%) in nearly all countries in western Europe, central Europe, eastern Europe, Australasia, and North America. Completeness was more variable in Latin America and the Caribbean, where several countries, such as Peru and Ecuador, have maintained completeness levels in the range of 70–94% since 1995, whereas others, such as Costa Rica, Cuba, and Argentina, have had complete systems for many years. Completeness was highly variable across countries in north Africa and the Middle East and across countries in southeast Asia. Of note, the Indian Sample Registration System completeness ranged from 92% to complete. Recent improvements include the increase in completeness in Iran from 64% in 1996 to 91% in 2015, an increase in Nicaragua from 75% in 1990 to 94% in 2013, and an increase in Thailand from 78% in 1990 to complete registration from 2005 to 2014. A few settings have seen declines in completeness including Albania, Uzbekistan, Guam, Northern Mariana Islands, and the Bahamas.

### Long-term trends in global mortality

**Figure 2:**
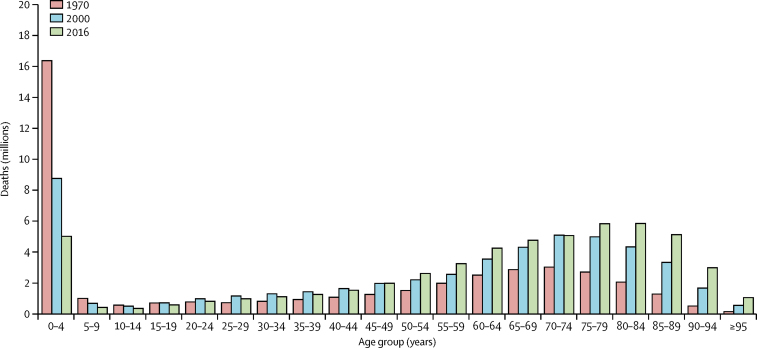
Global deaths by age group, 1970, 2000, and 2016 Each bar represents the total number of deaths in the given year in the specified age group.

The total number of deaths in the world per year increased from 42·8 million (95% UI 42·3 million to 43·3 million) in 1970 to 46·5 million (46·2 million to 46·9 million) in 1990 and 54·7 million (54·0 million to 55·5 million) in 2016. These changes reflect interplay between mortality rates, population totals, and the ageing of the world's populations. Figure 2 shows the change in the global number of deaths by age group estimated for the years 1970, 2000, and 2016. The number of under-5 deaths decreased from 16·4 million (16·1 million to 16·7 million) in 1970 to 8·7 million (8·5 million to 9·0 million) in 2000, and to 5·0 million (4·8 million to 5·2 million) in 2016. Decreases between time periods were also evident, although at a lower magnitude, for ages 5–24 years. By contrast, the number of adult deaths generally increased relative to 1970. Deaths among younger adults (25–49 years) increased from 4·8 million (4·7 million to 4·9 million) in 1970 to 7·5 million (7·4 million to 7·6 million) in 2000, but decreased to 6·9 million (6·7 million to 7·0 million) in 2016. The rate of increase in deaths for older adults (50–74 years) has been steady, increasing from 11·8 million (11·7 million to 12·0 million) in 1970 to 17·7 million (17·5 million to 17·8 million) in 2000, and to 20·0 million (19·6 million to 20·2 million) in 2016. Increases in adult deaths were largest in age groups older than 75 years; there were 6·7 million (6·6 million to 6·7 million) deaths among people 75 years and older in 1970, increasing to 14·7 million (14·6 million to 14·8 million) in 2000, and to 20·8 million (20·5–21·1 million) in 2016.

From 1970 to 2016, global mortality rates decreased for both men and women (appendix section 8 p 358). Age-standardised death rates for women decreased from 1367·4 per 100 000 (95% UI 1351·5 to 1384·2) in 1970 to 1036·9 per 100 000 (1026·9 to 1,047·4) in 1990 and 690·5 per 100 000 (678·2 to 706·3) in 2016, an annualised decrease of 1·49% during the period 1970 to 2016. The male age-standardised death rate declined from 1724·7 per 100 000 (1698·5 to 1751·8) in 1970 to 1407·5 per 100 000 (1394·7 to 1421·3) in 1990 and 1002·4 per 100 000 (985·1 to 1020·8) in 2016, an annualised decrease of 1·18% per year from 1970 to 2016. Over the same period, global life expectancy at birth for both sexes combined increased from 58·4 years (95% UI 57·9–58·9) in 1970 to 65·1 years (64·9–65·3) in 1990 and 72·5 years (72·1–72·8) in 2016 (appendix section 8 p 279). Life expectancy remains higher for women than for men on a global scale, with an estimated life expectancy at birth in 2016 of 75·3 years (75·0–75·6) for women and 69·8 years (69·3–70·2) for men; the absolute increase in life expectancy at birth was 14·8 years (14·1–15·4) for women (60·5 years [60·2–60·9] in 1970), but 13·5 years (12·3–14·6) for men (56·3 years [55·6–57·0] in 1970). The rate of increase in female life expectancy at birth was greater than that for men, rising by 0·32 years per year between 1970 and 2016 while the annualised rate for global male life expectancy at birth rose by 0·29 years per year over the same period. The difference in life expectancy at birth between men and women globally increased to 5·5 years in 2016 from 4·2 years in 1970. Life expectancy at age 65 years increased in 189 of 195 countries between 1970 and 2016.

**Figure 3:**
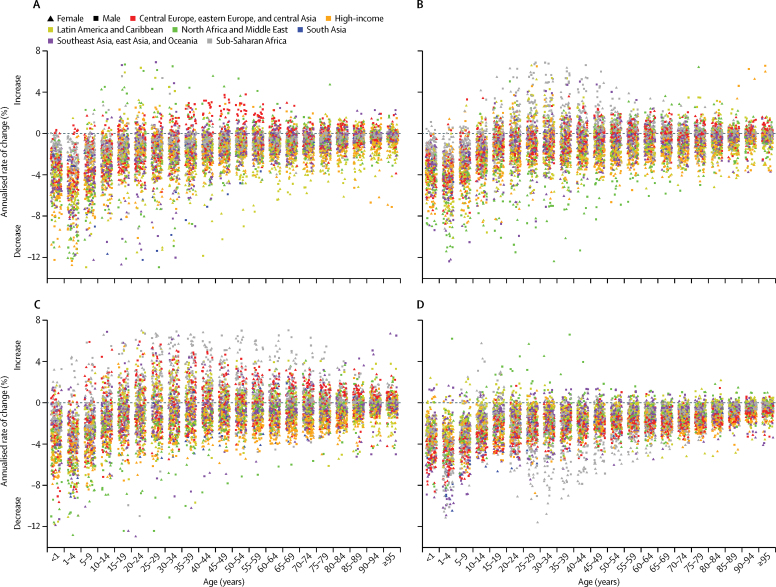
Annualised rates of change in age-specific mortality rates for 195 countries and territories Each point represents the annualised rate of change for a location grouped by age group and sex for (A) 1970–80, (B) 1980–90, (C) 1990–2000, and (D) 2000–16.

Figure 3 shows the distribution of annualised rates of change in mortality rates by age group and sex for locations grouped within GBD super-regions. From 1970 to 1980 (figure 3A), age-specific mortality rates decreased in the most locations for both sexes. Increases in annualised mortality rates did occur in many locations, notably across most age groups for locations in the super-region of central Europe, eastern Europe, and central Asia. The largest annualised increases occurred for adolescent and younger adult males (aged 15–34 years) in north Africa and the Middle East; southeast Asia, east Asia, and Oceania; and Latin America and the Caribbean. By contrast, the largest decreases in rates of change occurred for children younger than 5 years, particularly in the GBD super-regions of the high-income countries, Latin America and the Caribbean, and north Africa and the Middle East, while decreasing rates also occurred in young people aged 5–19 years in the super-regions of southeast Asia, east Asia, and Oceania and south Asia. Between 1980 and 1990 (figure 3B), rates notably increased in adolescent age groups in sub-Saharan Africa and in older adult age groups (older than 70 years) in the high-income super-region. Decreases in annualised rates of change occurred across most age groups and for both sexes in north Africa and the Middle East, with large decreases for children younger than 5 years. From 1990 to 2000 (figure 3C), annualised increases occurred in more locations than in the previous decades, particularly for locations in sub-Saharan Africa, but also for locations in central Europe, eastern Europe, and central Asia, and for locations in Latin America and the Caribbean. Increased annualised rates of change also occurred for adults of both sexes older than 70 years in the super-region of southeast Asia, east Asia, and Oceania. The distribution of annualised rates of change in age-specific mortality was visibly different over the period 2000 to 2016 compared with previous periods, with fewer instances of increasing annualised rates of change. Most annualised rates of change in age-specific mortality rates decreased, particularly for young adults (25–49 years) in sub-Saharan Africa and for children younger than 5 years in almost all GBD locations. However, notable exceptions included adolescents and younger adults in some locations in north Africa and the Middle East and adolescents in some locations in sub-Saharan Africa. Smaller increases were scattered across locations and age groups within other super-regions. Annualised rates of change in mortality rates between 2000 and 2016 were greater than 5·0% in 15 age-sex-location groups and greater than 10·0% in Syria for males aged 15–19 years (10·5%), 20–24 years (12·9%), and 25–29 years (11·2%) and females aged 10–14 years (10·2%).

**Figure 4:**
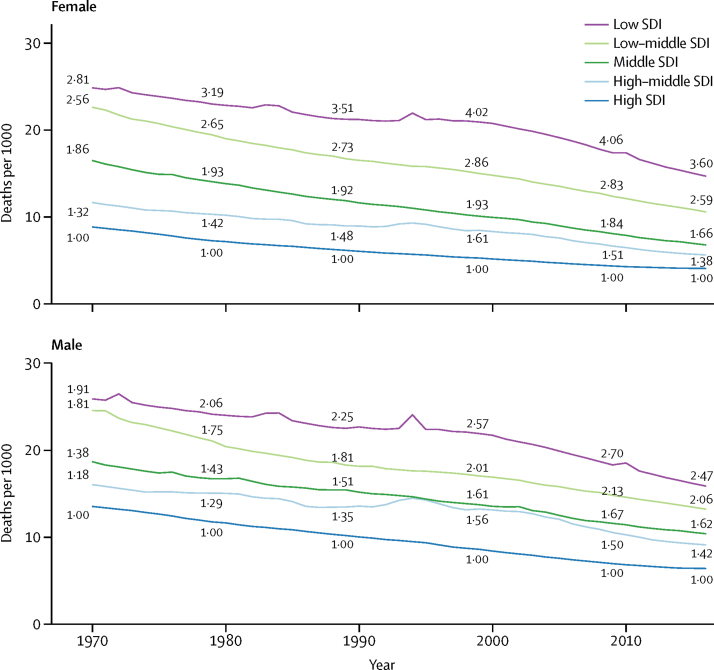
Age-standardised mortality rates, 1970–2016 Each line represents the trend in age-standardised mortality rates from 1970 to 2016 by SDI quintile. Values shown above the lines are ratios between the given SDI quintile and high SDI.

**Figure 5:**
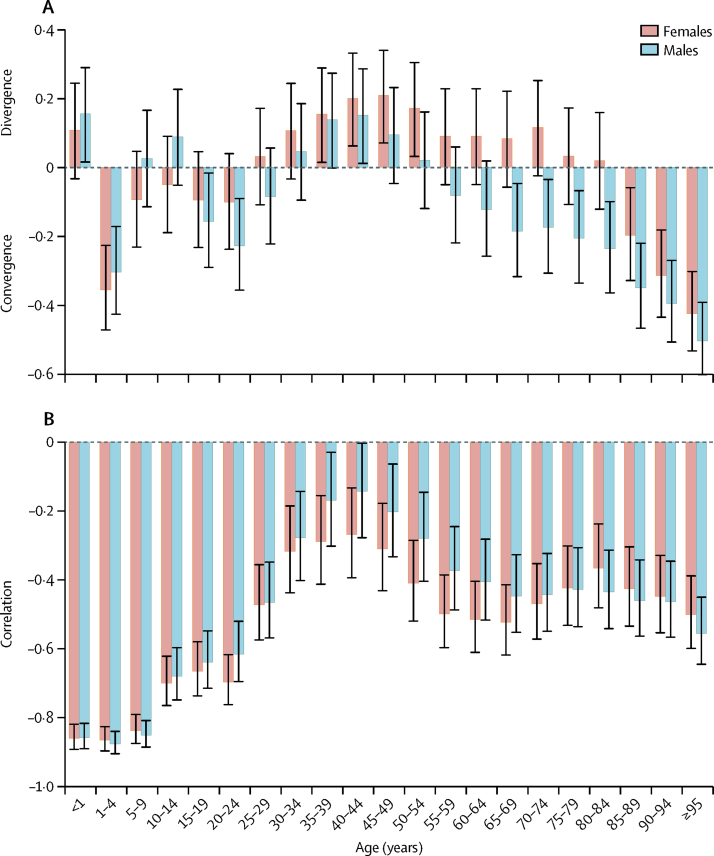
Correlation between the log of age-specific mortality rates in 1970 and (A) annualised (relative) rates of change and (B) absolute change, 1970–2016 Each bar represents the correlation between the log of age-specific mortality rate in 1970 and the change in the age-specific mortality rate from 1970 to 2016, for 195 countries and territories, by sex. Black lines represent 95% uncertainty intervals.

Figure 4 shows that the absolute difference between the age-standardised death rate for locations in the lowest SDI quintile and highest SDI quintile (countries classified by their 2016 level of SDI) narrowed between 1970 and 2016. However, the ratio of death rates in the lowest SDI quintile to those in the highest SDI quintile, a measure of relative inequality, increased over the same period. Whether this pattern is interpreted as convergence or divergence in death rates depends on which metric—the ratio of death rates or the absolute difference in death rates—is evaluated. Relative convergence can also be assessed by correlating annualised rates of change between time periods with starting levels of mortality. A positive correlation between the rate of change by age and the starting level of death rate indicates that countries with higher starting levels of mortality in an age group also had slower rates of decline or even increases, suggesting divergence in mortality rates; a negative correlation would indicate convergence. Figure 5A shows these correlations by age and sex. There was more evidence of divergence by age group over the period 1970 to 2016 for women (positive correlations) with the exceptions of ages 1–4 years and older than 85 years. Correlations were negative for females aged 5–9 years, 10–14 years, 15–19 years, and 20–24 years; however, the UIs for these correlations included zero. For men, evidence of convergence was clearer, with negative correlations between starting levels of mortality in 1970 and subsequent rates of change occurring for ages 1–4 years, 15–19 years, 20–24 years, and for each 5-year age group older than 65 years; negative correlations were also estimated for males aged 25–29 years, 55–59 years, and 60–64 years, although UIs for these correlations included zero. Correlations between the absolute change in age-sex-specific mortality rates between 1970 and 2016 and starting levels of mortality in 1970 (figure 5B) suggest convergence in mortality rates across all age groups for both men and women. Because small rates of change might nevertheless produce large magnitude differences when starting levels are high, negative correlations from absolute measures—apparent convergence in levels—might effectively mask evidence of diverging mortality rates.

### Stillbirths and child mortality

Numbers and rates of stillbirths across locations in 2016 are presented in table 1. In 2016, there were 1·7 million (95% UI 1·6 million to 1·8 million) stillbirths worldwide, a decrease of 65·3% since 1970. This decrease occurred against a background increase in the number of livebirths worldwide, which rose from 114·1 million in 1970 to 128·8 million in 2016. Rates of stillbirth decreased by 68·4%, from 41·5 deaths per 1000 livebirths (38·0–45·6) in 1970 to 13·1 deaths per 1000 livebirths (12·5–13·9) in 2016. The lowest rate of stillbirths in 2016 was 1·1 per 1000 (1·0–1·2) in Finland; stillbirth rates were highest in South Sudan at 43·4 per 1000 (42·4–44·5).

**Table 1:** Stillbirth rate, neonatal, post–neonatal, child, and under–5 mortality rates, number of stillbirths, and total number of under–5 deaths in 2016 and annualised rate of change in under–5 mortality for 2000–16, for global, SDI groups, GBD regions and super–regions, countries, and territories, both sexes combined

			**Deaths per 1000 livebirths**					**Total stillbirths (thousands)**	**Total under–5 deaths (thousands)**	**Annualised rate of change in under–5 deaths (%)**
			Stillbirths	Neonatal (0–27 days)	Post–neonatal (28 days to 1 year)	Child (1–4 years)	Under 5			
**Global**			**13·1 (12·5 to 13·9)**	**16·7 (14·9 to 18·9)**	**11·7 (10·6 to 12·9)**	**10·5 (9·3 to 11·9)**	**38·4 (34·5 to 43·1)**	**1716·2 (1634·4 to 1811·0)**	**4999.8 (4766.3 to 5252.6)**	**–3·7% (−4·3 to −3·0)**
High SDI			2·6 (2·5 to 2·8)	2·7 (2·5 to 2·9)	1·4 (1·3 to 1·5)	0·8 (0·7 to 0·9)	4·9 (4·6 to 5·3)	36·7 (34·9 to 38·8)	53·3 (51·7 to 55·2)	–2·1% (−2·6 to −1·6)
High–middle SDI			6·4 (6·0 to 6·8)	6·2 (5·3 to 7·2)	3·1 (2·7 to 3·6)	2·1 (1·7 to 2·4)	11·3 (9·8 to 13·2)	145·3 (136·2 to 155·9)	163·2 (143·0 to 195·6)	–4·9% (−5·8 to −3·9)
Middle SDI			8·9 (8·5 to 9·4)	9·9 (8·5 to 11·5)	5·8 (5·1 to 6·6)	3·6 (3·1 to 4·3)	19·3 (16·7 to 22·2)	276·8 (264·2 to 292·4)	600·3 (566·2 to 638·2)	–4·6% (−5·5 to −3·7)
Low–middle SDI			20·5 (19·3 to 21·9)	23·2 (20·8 to 26·1)	14·2 (13·0 to 15·6)	13·0 (11·5 to 14·7)	49·6 (44·8 to 55·2)	847·9 (797·6 to 904·4)	2275·9 (2104·1 to 2454·7)	–4·0% (−4·6 to −3·4)
Low SDI			19·1 (18·5 to 19·7)	24·3 (21·1 to 28·1)	23·2 (20·4 to 26·5)	24·4 (20·8 to 28·5)	70·2 (60·9 to 80·7)	409·5 (396·4 to 424·3)	1904·7 (1762·9 to 2056·6)	–4·3% (−5·1 to −3·5)
**High income**			**2·7 (2·5 to 3·0)**	**3·0 (2·8 to 3·2)**	**1·6 (1·5 to 1·7)**	**0·9 (0·8 to 1·0)**	**5·4 (5·1 to 5·8)**	**31·9 (29·3 to 35·2)**	**62·8 (60·7 to 64·9)**	**–2·1% (−2·5 to −1·7)**
High–income North America			2·7 (2·5 to 3·0)	3·8 (3·6 to 4·0)	1·9 (1·7 to 2·1)	1·0 (0·9 to 1·1)	6·7 (6·5 to 6·9)	12·2 (11·2 to 13·4)	29·6 (28·8 to 30·5)	–1·2% (−1·4 to −0·9)
	Canada		2·0 (2·0 to 2·1)	3·1(3·1 to 3·2)	1·5 (1·5 to 1·5)	0·7 (0·6 to 0·9)	5·4 (5·2 to 5·6)	0·8 (0·8 to 0·8)	2·2 (1·8 to 2·6)	–0·9% (−1·1 to −0·6)
	Greenland		5·6 (5·2 to 6·0)	7·7 (6·6 to 8·9)	3·7 (3·2 to 4·4)	2·8 (2·1 to 3·7)	14·1 (11·9 to 16·9)	<0·1 (<0·1 to <0·1)	<0·1 (<0·1 to <0·1)	–2·0% (−3·2 to −0·7)
	USA		2·8 (2·6 to 3·1)	3·9 (3·7 to 4·1)	1·9 (1·8 to 2·1)	1·0 (1·0 to 1·1)	6·8 (6·7 to 7·0)	11·4 (10·4 to 12·6)	27·5 (26·7 to 28·2)	–1·1% (−1·3 to −1·0)
	Australasia		3·1 (2·7 to 3·5)	2·4 (2·2 to 2·5)	1·1 (1·0 to 1·2)	0·7 (0·6 to 0·8)	4·2 (3·8 to 4·5)	1·1 (1·0 to 1·2)	1·5 (1·3 to 1·7)	–2·9% (−3·4 to −2·3)
		Australia	3·0 (2·6 to 3·4)	2·2 (2·1 to 2·4)	1·0 (0·9 to 1·1)	0·7 (0·6 to 0·8)	3·9 (3·6 to 4·2)	0·9 (0·8 to 1·0)	1·2 (1·0 to 1·4)	–3·1% (−3·6 to −2·5)
		New Zealand	3·3 (3·1 to 3·6)	3·1 (2·9 to 3·3)	1·6 (1·5 to 1·7)	0·9 (0·8 to 1·1)	5·6 (5·2 to 6·0)	0·2 (0·2 to 0·2)	0·3 (0·3 to 0·4)	–1·9% (−2·4 to −1·4)
High–income Asia Pacific			1·8 (1·5 to 2·1)	1·1 (1·0 to 1·2)	1·1 (1·0 to 1·2)	0·7 (0·7 to 0·8)	2·9 (2·6 to 3·2)	2·5 (2·1 to 2·9)	4·1 (3·7 to 4·5)	–3·8% (−4·4 to −3·1)
	Brunei		2·6 (2·3 to 3·0)	4·3 (3·8 to 4·8)	3·4 (3·1 to 3·8)	1·5 (1·2 to 1·9)	9·2 (8·0 to 10·5)	<0·1 (<0·1 to <0·1)	0·1 (0·1 to 0·1)	0·2% (−0·7 to 1·2)
	Japan		1·7 (1·4 to 2·1)	0·9 (0·8 to 1·0)	1·0 (0·9 to 1·1)	0·7 (0·6 to 0·8)	2·6 (2·4 to 2·9)	1·6 (1·3 to 2·0)	2·6 (2·4 to 2·7)	–3·4% (−3·9 to −2·7)
	Singapore		1·9 (1·9 to 2·0)	1·0 (0·9 to 1·1)	0·8 (0·7 to 0·9)	0·5 (0·4 to 0·6)	2·3 (2·0 to 2·5)	0·1 (0·1 to 0·1)	0·1 (0·1 to 0·1)	–3·3% (−4·0 to −2·5)
	South Korea		1·9 (1·8 to 2·0)	1·5 (1·2 to 1·9)	1·2 (1·0 to 1·5)	0·8 (0·6 to 1·0)	3·5 (2·8 to 4·4)	0·8 (0·7 to 0·8)	1·4 (1·1 to 1·8)	–4·3% (−5·8 to −2·9)
Western Europe			2·3 (2·2 to 2·4)	2·0 (1·8 to 2·3)	1·0 (0·9 to 1·2)	0·6 (0·5 to 0·7)	3·7 (3·3 to 4·2)	10·0 (9·6 to 10·4)	16·4 (15·1 to 17·7)	–2·7% (−3·5 to −1·9)
	Andorra		2·7 (2·7 to 2·8)	1·2 (0·9 to 1·5)	0·7 (0·5 to 0·8)	1·5 (1·1 to 2·0)	3·3 (2·6 to 4·3)	<0·1 (<0·1 to <0·1)	<0·1 (<0·1 to <0·1)	0·3% (−1·1 to 1·9)
	Austria		1·8 (1·7 to 1·9)	1·9 (1·6 to 2·2)	0·9 (0·8 to 1·1)	0·6 (0·5 to 0·7)	3·4 (2·8 to 4·0)	0·1 (0·1 to 0·2)	0·3 (0·2 to 0·4)	–3·3% (−4·5 to −2·0)
	Belgium		1·8 (1·4 to 2·3)	2·0 (1·8 to 2·1)	1·2 (1·1 to 1·3)	0·8 (0·6 to 0·9)	3·9 (3·6 to 4·3)	0·2 (0·2 to 0·3)	0·5 (0·4 to 0·6)	–2·5% (−3·0 to −1·9)
	Cyprus		2·1 (2·0 to 2·2)	1·7 (1·4 to 2·1)	1·0 (0·8 to 1·2)	0·6 (0·4 to 0·8)	3·3 (2·8 to 3·8)	<0·1 (<0·1 to <0·1)	<0·1 (<0·1 to <0·1)	–5·7% (−6·7 to −4·6)
	Denmark		1·3 (1·1 to 1·5)	2·5 (2·2 to 2·8)	1·0 (0·9 to 1·1)	0·6 (0·5 to 0·8)	4·1 (3·5 to 4·7)	0·1 (0·1 to 0·1)	0·2 (0·2 to 0·3)	–2·0% (−3·0 to −1·0)
	Finland		1·1 (1·1 to 1·2)	1·2 (1·0 to 1·4)	0·6 (0·5 to 0·7)	0·4 (0·3 to 0·6)	2·2 (1·8 to 2·7)	0·1 (0·1 to 0·1)	0·1 (0·1 to 0·2)	–4·1% (−5·5 to −2·7)
	France		3·3 (3·3 to 3·4)	2·1 (1·7 to 2·5)	1·1 (0·9 to 1·4)	0·7 (0·5 to 0·8)	3·8 (3·2 to 4·6)	2·7 (2·6 to 2·7)	3·0 (2·4 to 3·9)	–2·2% (−3·3 to −1·0)
	Germany		1·5 (1·5 to 1·5)	1·9 (1·6 to 2·3)	1·1 (0·9 to 1·3)	0·6 (0·5 to 0·8)	3·6 (3·0 to 4·4)	1·0 (1·0 to 1·0)	2·5 (2·0 to 3·2)	–2·5% (−3·7 to −1·2)
	Greece		1·9 (1·8 to 1·9)	2·2 (2·0 to 2·4)	1·1 (1·0 to 1·2)	0·6 (0·5 to 0·8)	3·9 (3·6 to 4·3)	0·2 (0·2 to 0·2)	0·4 (0·3 to 0·5)	–3·0% (−3·7 to −2·4)
	Iceland		1·2 (1·1 to 1·4)	1·0 (0·8 to 1·1)	0·6 (0·5 to 0·7)	0·7 (0·5 to 0·9)	2·2 (1·9 to 2·6)	<0·1 (<0·1 to <0·1)	<0·1 (<0·1 to <0·1)	–3·8% (−5·0 to −2·5)
	Ireland		2·1 (2·0 to 2·3)	2·2 (1·9 to 2·6)	1·1 (0·9 to 1·2)	0·6 (0·5 to 0·7)	3·9 (3·4 to 4·5)	0·1 (0·1 to 0·1)	0·3 (0·2 to 0·3)	–3·9% (−4·9 to −2·8)
	Israel		2·4 (2·1 to 2·7)	1·8 (1·6 to 2·0)	1·1 (1·0 to 1·2)	0·7 (0·6 to 0·9)	3·6 (3·1 to 4·1)	0·4 (0·4 to 0·5)	0·6 (0·5 to 0·7)	–4·3% (−5·1 to −3·4)
	Italy		1·5 (1·3 to 1·6)	1·9 (1·5 to 2·3)	0·8 (0·7 to 1·0)	0·5 (0·4 to 0·7)	3·2 (2·5 to 4·0)	0·7 (0·7 to 0·8)	1·6 (1·1 to 2·2)	–3·2% (−4·7 to −1·8)
	Luxembourg		1·8 (1·5 to 2·2)	1·1 (0·9 to 1·3)	0·6 (0·5 to 0·7)	0·5 (0·4 to 0·5)	2·2 (1·8 to 2·6)	<0·1 (<0·1 to <0·1)	<0·1 (<0·1 to <0·1)	–4·8% (−5·9 to −3·7)
	Malta		2·5 (2·5 to 2·6)	4·4 (3·8 to 5·1)	1·1 (0·9 to 1·3)	0·7 (0·6 to 0·9)	6·3 (5·4 to 7·3)	<0·1 (<0·1 to <0·1)	<0·1 (<0·1 to <0·1)	–1·3% (−2·4 to −0·2)
	Netherlands		1·8 (1·4 to 2·3)	2·3 (2·2 to 2·5)	0·9 (0·8 to 1·0)	0·6 (0·5 to 0·7)	3·8 (3·4 to 4·2)	0·3 (0·2 to 0·4)	0·7 (0·5 to 0·8)	–3·2% (−3·8 to −2·5)
	Norway		1·5 (1·4 to 1·6)	1·5 (1·3 to 1·7)	0·7 (0·6 to 0·8)	0·5 (0·4 to 0·6)	2·7 (2·3 to 3·2)	0·1 (0·1 to 0·1)	0·2 (0·1 to 0·2)	–3·8% (−4·9 to −2·6)
	Portugal		1·6 (1·6 to 1·7)	1·5 (1·5 to 1·6)	0·9 (0·9 to 1·0)	0·6 (0·6 to 0·7)	3·1 (2·9 to 3·4)	0·1 (0·1 to 0·1)	0·3 (0·2 to 0·3)	–5·1% (−5·5 to −4·6)
	Spain		1·3 (1·2 to 1·4)	1·7 (1·5 to 1·9)	0·9 (0·8 to 1·1)	0·6 (0·5 to 0·8)	3·3 (2·8 to 3·8)	0·5 (0·5 to 0·6)	1·4 (1·0 to 1·9)	–3·0% (−3·9 to −2·2)
	Sweden		2·0 (1·7 to 2·5)	1·4 (1·1 to 1·7)	0·8 (0·6 to 1·0)	0·5 (0·4 to 0·6)	2·6 (2·2 to 3·2)	0·2 (0·2 to 0·3)	0·3 (0·2 to 0·4)	–2·5% (−3·8 to −1·2)
	Switzerland		1·7 (1·6 to 1·9)	2·6 (2·3 to 3·0)	0·8 (0·7 to 0·9)	0·5 (0·3 to 0·8)	3·9 (3·3 to 4·7)	0·1 (0·1 to 0·2)	0·3 (0·3 to 0·4)	–2·4% (−3·5 to −1·3)
	UK		3·5 (3·4 to 3·6)	2·6 (2·4 to 2·8)	1·3 (1·2 to 1·4)	0·7 (0·6 to 0·8)	4·6 (4·2 to 5·0)	2·8 (2·7 to 2·9)	3·6 (3·5 to 3·7)	–2·3% (−2·9 to −1·8)
		England	3·2 (3·1 to 3·3)	2·6 (2·4 to 2·8)	1·3 (1·2 to 1·4)	0·7 (0·7 to 0·8)	4·6 (4·3 to 4·9)	2·2 (2·1 to 2·2)	3·1 (3·0 to 3·1)	–2·3% (−2·8 to −1·8)
		Northern Ireland	5·4 (5·3 to 5·5)	3·0 (2·0 to 4·2)	1·2 (0·8 to 1·7)	0·7 (0·5 to 1·0)	4·8 (3·3 to 6·8)	0·1 (0·1 to 0·1)	0·1 (0·1 to 0·2)	–1·9% (−4·4 to 0·5)
		Scotland	5·4 (5·2 to 5·5)	2·2 (1·8 to 2·8)	1·2 (0·9 to 1·5)	0·6 (0·5 to 0·8)	4·1 (3·2 to 5·1)	0·3 (0·3 to 0·3)	0·2 (0·2 to 0·3)	–2·9% (−4·4 to −1·4)
		Wales	5·2 (5·0 to 5·3)	2·4 (2·0 to 3·0)	1·3 (1·0 to 1·5)	0·7 (0·5 to 0·8)	4·4 (3·5 to 5·3)	0·2 (0·2 to 0·2)	0·1 (0·1 to 0·2)	–2·4% (−3·7 to −1·1)
Southern Latin America			6·1 (4·6 to 7·9)	6·3 (6·0 to 6·6)	3·2 (2·9 to 3·4)	1·6 (1·5 to 1·7)	11·0 (10·5 to 11·6)	6·2 (4·7 to 8·1)	11·2 (9·9 to 12·7)	–3·0% (−3·3 to −2·7)
	Argentina		5·4 (4·4 to 6·6)	6·9 (6·6 to 7·1)	3·6 (3·4 to 3·8)	1·8 (1·7 to 1·9)	12·2 (11·7 to 12·7)	3·9 (3·2 to 4·8)	8·9 (7·6 to 10·3)	–3·2% (−3·5 to −3·0)
	Chile		8·5 (5·5 to 12·5)	4·8 (4·6 to 5·1)	1·9 (1·8 to 2·1)	1·1 (1·0 to 1·2)	7·8 (7·4 to 8·3)	2·1 (1·3 to 3·0)	1·9 (1·5 to 2·3)	–2·1% (−2·5 to −1·8)
	Uruguay		4·6 (4·1 to 5·2)	4·5 (3·5 to 5·6)	3·0 (2·4 to 3·8)	1·3 (0·8 to 1·9)	8·8 (6·8 to 11·3)	0·2 (0·2 to 0·3)	0·4 (0·3 to 0·6)	–3·8% (−5·4 to −2·1)
**Central Europe, eastern Europe, and central Asia**			**4·5 (4·3 to 4·6)**	**7·0 (5·7 to 8·6)**	**4·2 (3·3 to 5·4)**	**2·4 (1·9 to 3·2)**	**13·6 (10·9 to 17·1)**	**25·3 (24·7 to 26·0)**	**77·6 (67·2 to 91·3)**	**–4·5% (−6·0 to −3·1)**
Eastern Europe			2·9 (2·8 to 2·9)	4·4 (4·0 to 4·8)	2·4 (2·1 to 2·8)	1·6 (1·4 to 1·8)	8·4 (7·6 to 9·4)	7·5 (7·3 to 7·6)	21·7 (19·0 to 25·0)	–5·4% (−6·1 to −4·6)
	Belarus		2·8 (2·7 to 2·8)	2·7 (2·1 to 3·4)	1·6 (1·1 to 2·3)	1·3 (1·0 to 1·7)	5·5 (4·2 to 7·4)	0·3 (0·3 to 0·3)	0·6 (0·5 to 0·9)	–6·7% (−8·5 to −4·7)
	Estonia		1·5 (1·4 to 1·6)	1·3 (1·0 to 1·7)	1·0 (0·8 to 1·4)	0·8 (0·6 to 1·0)	3·1 (2·4 to 4·1)	<0·1 (<0·1 to <0·1)	<0·1 (<0·1 to 0·1)	–8·0% (−9·8 to −6·3)
	Latvia		2·3 (2·3 to 2·4)	2·5 (1·8 to 3·4)	1·4 (0·9 to 2·0)	0·9 (0·6 to 1·4)	4·8 (3·3 to 6·8)	0·1 (0·1 to 0·1)	0·1 (0·1 to 0·2)	–6·3% (−8·7 to −4·0)
	Lithuania		1·9 (1·9 to 2·0)	2·2 (1·9 to 2·5)	1·6 (1·4 to 1·8)	0·9 (0·7 to 1·1)	4·7 (4·1 to 5·3)	0·1 (0·1 to 0·1)	0·1 (0·1 to 0·2)	–5·5% (−6·4 to −4·6)
	Moldova		4·1 (3·9 to 4·4)	7·7 (5·1 to 11·2)	2·4 (1·6 to 3·4)	1·6 (1·0 to 2·3)	11·6 (7·7 to 16·8)	0·2 (0·2 to 0·2)	0·5 (0·3 to 0·8)	–6·2% (−9·2 to −3·6)
	Russia		2·8 (2·8 to 2·9)	4·2 (4·1 to 4·3)	2·5 (2·2 to 2·8)	1·7 (1·6 to 1·8)	8·4 (8·1 to 8·7)	5·4 (5·2 to 5·5)	15·8 (14·2 to 17·6)	–5·4% (−5·6 to −5·1)
	Ukraine		3·0 (3·0 to 3·1)	5·2 (3·5 to 7·5)	2·4 (1·3 to 4·2)	1·6 (0·9 to 2·6)	9·2 (5·7 to 14·1)	1·4 (1·4 to 1·5)	4·5 (2·5 to 7·5)	–5·1% (−8·4 to −2·1)
Central Europe			2·9 (2·7 to 3·1)	3·3 (2·8 to 4·1)	1·9 (1·6 to 2·3)	1·0 (0·8 to 1·3)	6·2 (5·1 to 7·6)	3·2 (3·0 to 3·4)	6·9 (5·9 to 8·0)	–5·0% (−6·2 to −3·6)
	Albania		4·1 (4·1 to 4·2)	7·2 (4·9 to 10·4)	4·2 (2·8 to 6·1)	2·3 (1·4 to 3·8)	13·7 (9·1 to 20·2)	0·2 (0·2 to 0·2)	0·5 (0·3 to 0·8)	–4·3% (−7·2 to −1·7)
	Bosnia and Herzegovina		3·6 (3·2 to 4·0)	3·5 (2·9 to 4·2)	0·9 (0·9 to 0·9)	0·8 (0·7 to 0·9)	5·2 (4·5 to 6·0)	0·1 (0·1 to 0·1)	0·2 (0·1 to 0·2)	–4·7% (−5·7 to −3·7)
	Bulgaria		3·9 (3·4 to 4·5)	4·0 (2·7 to 5·7)	2·9 (2·0 to 4·1)	1·5 (1·0 to 2·2)	8·3 (5·7 to 12·0)	0·3 (0·2 to 0·3)	0·5 (0·3 to 0·9)	–4·3% (−6·7 to −2·0)
	Croatia		2·3 (2·2 to 2·4)	2·8 (2·3 to 3·2)	1·0 (0·9 to 1·2)	0·7 (0·6 to 0·8)	4·5 (3·8 to 5·3)	0·1 (0·1 to 0·1)	0·2 (0·1 to 0·2)	–4·4% (−5·5 to −3·3)
	Czech Republic		1·7 (1·5 to 1·8)	1·4 (1·2 to 1·6)	0·9 (0·8 to 1·1)	0·5 (0·4 to 0·7)	2·8 (2·4 to 3·3)	0·2 (0·2 to 0·2)	0·3 (0·2 to 0·4)	–4·0% (−5·2 to −2·9)
	Hungary		2·4 (2·3 to 2·5)	3·0 (2·4 to 3·6)	1·8 (1·5 to 2·3)	0·8 (0·6 to 1·1)	5·6 (4·5 to 6·9)	0·2 (0·2 to 0·2)	0·5 (0·4 to 0·7)	–4·0% (−5·4 to −2·6)
	Macedonia		7·7 (6·7 to 8·9)	6·5 (4·1 to 10·0)	2·4 (1·5 to 3·7)	1·2 (0·7 to 1·7)	10·1 (6·4 to 15·3)	0·2 (0·1 to 0·2)	0·2 (0·1 to 0·3)	–2·2% (−5·1 to 0·3)
	Montenegro		3·3 (3·0 to 3·6)	3·0 (2·3 to 3·9)	1·4 (1·1 to 1·8)	0·7 (0·5 to 0·9)	5·1 (3·9 to 6·6)	<0·1 (<0·1 to <0·1)	<0·1 (<0·1 to <0·1)	–7·5% (−9·2 to −5·8)
	Poland		2·0 (1·7 to 2·4)	2·6 (1·8 to 3·8)	1·2 (0·8 to 1·7)	0·7 (0·4 to 1·0)	4·5 (3·0 to 6·5)	0·7 (0·6 to 0·9)	1·7 (1·0 to 2·7)	–4·7% (−7·2 to −2·4)
	Romania		3·9 (3·9 to 3·9)	4·5 (3·8 to 5·3)	3·4 (2·8 to 4·2)	1·5 (1·3 to 1·8)	9·5 (8·2 to 11·0)	0·7 (0·7 to 0·7)	1·7 (1·3 to 2·1)	–5·6% (−6·6 to −4·7)
	Serbia		4·7 (4·4 to 5·1)	4·5 (3·7 to 5·3)	2·3 (2·0 to 2·8)	1·1 (0·9 to 1·3)	7·9 (6·6 to 9·3)	0·4 (0·4 to 0·4)	0·6 (0·5 to 0·8)	–4·1% (−5·6 to −2·5)
	Slovakia		2·3 (2·2 to 2·4)	3·0 (2·3 to 3·9)	2·0 (1·5 to 2·6)	1·1 (0·8 to 1·4)	6·1 (4·6 to 8·0)	0·1 (0·1 to 0·1)	0·3 (0·2 to 0·5)	–3·1% (−4·9 to −1·3)
	Slovenia		1·9 (1·8 to 2·1)	1·5 (1·3 to 1·7)	0·4 (0·3 to 0·5)	0·5 (0·4 to 0·6)	2·4 (2·0 to 2·8)	<0·1 (<0·1 to <0·1)	<0·1 (<0·1 to 0·1)	–5·2% (−6·3 to −4·0)
Central Asia			7·4 (7·2 to 7·6)	12·4 (9·3 to 16·3)	7·9 (5·6 to 10·7)	4·4 (3·1 to 6·3)	24·5 (17·9 to 32·9)	14·7 (14·3 to 15·1)	48·9 (39·2 to 62·1)	–4·5% (−6·5 to −2·5)
	Armenia		6·8 (6·1 to 7·6)	6·1 (4·5 to 8·2)	4·1 (3·0 to 5·5)	1·9 (1·4 to 2·5)	12·1 (8·9 to 16·1)	0·3 (0·3 to 0·3)	0·5 (0·4 to 0·7)	–5·8% (−7·9 to −3·7)
	Azerbaijan		9·4 (9·1 to 9·8)	16·7 (12·4 to 22·0)	9·6 (6·4 to 14·2)	3·9 (2·7 to 5·5)	30·0 (21·4 to 41·1)	1·8 (1·7 to 1·9)	5·7 (3·8 to 8·4)	–4·6% (−7·0 to −2·4)
	Georgia		5·3 (4·9 to 5·8)	9·6 (7·1 to 12·8)	3·5 (2·6 to 4·8)	2·3 (1·7 to 3·1)	15·4 (11·4 to 20·4)	0·3 (0·3 to 0·4)	1·0 (0·7 to 1·4)	–5·6% (−7·7 to −3·7)
	Kazakhstan		5·2 (5·0 to 5·4)	6·6 (5·0 to 8·6)	4·2 (3·0 to 5·8)	2·7 (1·8 to 3·7)	13·5 (9·9 to 18·0)	2·0 (2·0 to 2·1)	5·3 (3·8 to 7·3)	–6·3% (−8·5 to −4·0)
	Kyrgyzstan		9·9 (9·5 to 10·3)	16·7 (14·1 to 19·5)	8·2 (6·6 to 10·0)	4·4 (3·6 to 5·2)	28·9 (24·2 to 34·4)	1·5 (1·5 to 1·6)	4·4 (3·6 to 5·4)	–3·4% (−4·6 to −2·2)
	Mongolia		7·1 (6·9 to 7·4)	11·2 (9·5 to 13·0)	6·1 (4·8 to 7·6)	4·1 (2·5 to 6·3)	21·2 (16·7 to 26·6)	0·6 (0·5 to 0·6)	1·6 (1·2 to 2·2)	–6·2% (−7·9 to −4·7)
	Tajikistan		8·9 (8·7 to 9·2)	16·9 (12·7 to 21·9)	14·1 (10·1 to 19·1)	7·1 (4·4 to 10·7)	37·6 (27·1 to 50·8)	2·3 (2·2 to 2·3)	9·4 (5·8 to 14·4)	–4·3% (−6·5 to −2·2)
	Turkmenistan		7·7 (7·6 to 7·9)	17·1 (11·6 to 24·2)	12·5 (8·4 to 18·1)	7·9 (5·2 to 11·6)	37·0 (25·2 to 52·6)	0·9 (0·9 to 1·0)	4·5 (2·9 to 6·6)	–4·9% (−7·6 to −2·3)
	Uzbekistan		7·1 (6·9 to 7·4)	12·0 (8·5 to 16·4)	7·2 (5·1 to 9·8)	4·5 (3·2 to 6·3)	23·5 (16·7 to 32·1)	4·9 (4·7 to 5·1)	16·4 (8·7 to 28·0)	–3·7% (−6·1 to −1·5)
**Latin America and Caribbean**			**6·3 (5·9 to 6·8)**	**9·3 (7·3 to 11·7)**	**6·2 (4·8 to 7·9)**	**3·4 (2·6 to 4·4)**	**18·7 (14·7 to 23·8)**	**62·6 (58·6 to 67·7)**	**185·2 (174·7 to 198·3)**	**–3·6% (−5·0 to −2·1)**
Central Latin America			5·4 (5·1 to 5·7)	8·3 (7·0 to 9·9)	5·3 (4·4 to 6·4)	3·2 (2·7 to 3·8)	16·7 (14·0 to 20·1)	24·5 (23·2 to 26·0)	75·7 (71·4 to 80·6)	–3·4% (−4·5 to −2·3)
	Colombia		8·5 (7·7 to 9·5)	9·0 (7·5 to 10·7)	3·6 (3·0 to 4·3)	3·0 (2·2 to 4·1)	15·5 (12·6 to 19·0)	5·8 (5·3 to 6·5)	10·6 (8·5 to 13·4)	–3·4% (−4·9 to −2·0)
	Costa Rica		5·9 (5·5 to 6·4)	6·5 (5·2 to 8·2)	2·6 (2·0 to 3·3)	1·5 (1·0 to 2·2)	10·6 (8·1 to 13·7)	0·3 (0·3 to 0·4)	0·6 (0·4 to 0·9)	–2·4% (−4·1 to −0·5)
	El Salvador		4·5 (4·3 to 4·6)	5·4 (4·3 to 6·7)	4·2 (3·4 to 5·3)	2·3 (1·9 to 2·9)	11·9 (9·5 to 14·8)	0·5 (0·4 to 0·5)	1·2 (0·9 to 1·8)	–5·6% (−7·3 to −4·1)
	Guatemala		6·6 (5·9 to 7·3)	10·8 (8·8 to 13·2)	9·7 (7·9 to 11·9)	6·5 (6·3 to 6·7)	26·7 (22·9 to 31·2)	2·7 (2·5 to 3·0)	10·9 (9·0 to 13·3)	–4·1% (−5·0 to −3·2)
	Honduras		8·4 (8·3 to 8·4)	11·6 (9·6 to 14·2)	5·5 (4·9 to 6·3)	4·4 (3·8 to 5·2)	21·4 (18·3 to 25·4)	1·7 (1·6 to 1·7)	4·2 (3·3 to 5·2)	–3·4% (−4·3 to −2·4)
	Mexico		4·0 (3·9 to 4·2)	7·3 (5·8 to 9·2)	5·4 (4·2 to 7·0)	2·8 (2·2 to 3·6)	15·5 (12·1 to 19·7)	9·3 (8·9 to 9·8)	35·6 (33·2 to 38·2)	–3·6% (−5·1 to −2·1)
	Nicaragua		5·4 (5·3 to 5·5)	7·4 (6·2 to 8·9)	4·9 (4·2 to 6·0)	2·8 (2·4 to 3·4)	15·1 (12·7 to 18·2)	0·6 (0·6 to 0·6)	1·8 (1·5 to 2·2)	–5·5% (−6·5 to −4·4)
	Panama		4·8 (4·6 to 5·0)	7·1 (5·7 to 8·9)	5·5 (4·4 to 7·0)	4·0 (3·2 to 5·1)	16·6 (13·1 to 20·9)	0·3 (0·3 to 0·3)	1·1 (0·8 to 1·6)	–2·1% (−3·6 to −0·5)
	Venezuela		5·6 (5·2 to 6·1)	10·0 (9·6 to 10·4)	4·3 (4·1 to 4·6)	2·6 (2·3 to 2·9)	16·8 (16·0 to 17·7)	3·2 (3·0 to 3·5)	9·6 (8·1 to 11·3)	–1·5% (−1·8 to −1·2)
Andean Latin America			6·2 (5·8 to 6·7)	9·5 (7·6 to 11·8)	6·6 (5·3 to 8·2)	4·5 (3·4 to 5·8)	20·4 (16·3 to 25·4)	8·7 (8·1 to 9·5)	28·2 (24·1 to 32·8)	–4·9% (−6·3 to −3·6)
	Bolivia		8·9 (7·4 to 10·9)	15·2 (11·8 to 19·4)	11·7 (9·0 to 15·0)	7·3 (5·6 to 9·3)	33·8 (26·3 to 43·2)	2·6 (2·2 to 3·2)	9·7 (7·4 to 12·7)	–4·8% (−6·3 to −3·3)
	Ecuador		5·3 (4·8 to 5·8)	8·2 (7·0 to 9·4)	5·3 (4·5 to 6·1)	3·9 (2·8 to 5·4)	17·3 (14·5 to 20·7)	2·0 (1·8 to 2·1)	6·3 (5·0 to 8·0)	–4·0% (−5·2 to −2·9)
	Peru		5·6 (5·0 to 6·3)	7·8 (6·2 to 9·9)	5·2 (4·1 to 6·7)	3·6 (2·8 to 4·6)	16·6 (13·2 to 20·8)	4·2 (3·7 to 4·7)	12·1 (9·4 to 15·5)	–5·2% (−6·7 to −3·8)
Caribbean			15·8 (13·8 to 18·3)	15·8 (11·4 to 21·8)	11·3 (7·9 to 16·0)	6·7 (4·6 to 9·5)	33·5 (23·6 to 46·8)	12·6 (11·0 to 14·6)	27·2 (19·7 to 37·9)	–2·5% (−4·8 to −0·4)
	Antigua and Barbuda		8·1 (6·6 to 10·2)	5·7 (3·7 to 8·6)	2·8 (1·8 to 4·2)	2·7 (1·9 to 3·8)	11·2 (7·4 to 16·5)	<0·1 (<0·1 to <0·1)	<0·1 (<0·1 to <0·1)	–2·4% (−5·0 to 0·1)
	The Bahamas		12·1 (9·1 to 15·9)	5·3 (2·8 to 9·7)	3·5 (1·8 to 6·6)	2·2 (1·1 to 4·2)	11·0 (5·9 to 20·3)	0·1 (0·1 to 0·1)	0·1 (<0·1 to 0·2)	–1·5% (−5·8 to 3·0)
	Barbados		9·4 (7·6 to 11·7)	9·9 (5·4 to 17·2)	3·5 (1·9 to 5·9)	1·4 (0·7 to 2·7)	14·7 (7·9 to 25·7)	<0·1 (<0·1 to <0·1)	<0·1 (<0·1 to 0·1)	–1·2% (−6·0 to 3·4)
	Belize		9·6 (7·8 to 12·1)	8·3 (4·9 to 13·5)	4·0 (2·3 to 6·5)	2·8 (1·7 to 4·7)	15·1 (8·8 to 24·6)	0·1 (0·1 to 0·1)	0·2 (0·1 to 0·3)	–3·9% (−7·4 to −0·5)
	Bermuda		5·9 (5·5 to 6·3)	1·7 (1·2 to 2·3)	0·8 (0·5 to 1·2)	1·4 (0·7 to 2·4)	3·9 (2·5 to 5·8)	<0·1 (<0·1 to <0·1)	<0·1 (<0·1 to <0·1)	–0·7% (−4·2 to 3·0)
	Cuba		10·4 (7·6 to 13·8)	2·4 (2·2 to 2·6)	1·7 (1·4 to 2·0)	1·2 (1·0 to 1·6)	5·3 (5·0 to 5·7)	1·1 (0·8 to 1·5)	0·6 (0·5 to 0·7)	–3·1% (−3·4 to −2·6)
	Dominica		15·1 (12·3 to 18·9)	18·2 (11·7 to 27·3)	4·8 (3·1 to 7·3)	2·1 (0·9 to 4·1)	25·0 (15·7 to 38·5)	<0·1 (<0·1 to <0·1)	<0·1 (<0·1 to <0·1)	1·8% (−1·4 to 4·9)
	Dominican Republic		11·9 (10·7 to 13·2)	17·6 (14·0 to 22·1)	6·3 (5·0 to 8·0)	3·7 (2·9 to 4·6)	27·4 (21·7 to 34·5)	2·2 (2·0 to 2·5)	5·1 (3·6 to 6·9)	–1·9% (−3·5 to −0·4)
	Grenada		10·5 (8·5 to 13·2)	9·8 (5·5 to 17·1)	3·2 (1·8 to 5·7)	2·4 (1·2 to 4·9)	15·4 (8·4 to 27·9)	<0·1 (<0·1 to <0·1)	<0·1 (<0·1 to <0·1)	0·0% (−4·3 to 4·1)
	Guyana		12·0 (11·2 to 13·1)	16·4 (13·1 to 20·5)	5·7 (4·4 to 7·2)	3·0 (2·4 to 3·8)	24·9 (19·7 to 31·2)	0·2 (0·2 to 0·2)	0·3 (0·2 to 0·4)	–2·8% (−4·5 to −1·3)
	Haiti		22·4 (18·2 to 28·0)	22·1 (15·0 to 31·5)	21·3 (14·3 to 30·8)	13·0 (8·7 to 19·0)	55·4 (37·4 to 79·2)	7·5 (6·1 to 9·4)	18·0 (11·0 to 28·2)	–3·7% (−6·3 to −1·3)
	Jamaica		12·8 (11·8 to 13·8)	11·4 (7·2 to 17·5)	5·2 (3·0 to 8·7)	3·6 (2·1 to 5·8)	20·1 (12·2 to 31·7)	0·7 (0·7 to 0·8)	1·1 (0·6 to 1·9)	–1·3% (−5·0 to 2·2)
	Puerto Rico		5·5 (5·3 to 5·8)	4·4 (3·9 to 4·8)	1·6 (1·4 to 1·8)	0·6 (0·4 to 0·9)	6·6 (5·8 to 7·5)	0·2 (0·2 to 0·2)	0·3 (0·2 to 0·3)	–4·0% (−4·9 to −3·2)
	Saint Lucia		13·7 (12·3 to 15·5)	13·0 (7·0 to 22·5)	2·7 (1·4 to 4·6)	1·8 (0·7 to 4·2)	17·5 (9·1 to 31·2)	0·0 (0·0 to 0·0)	<0·1 (<0·1 to 0·1)	–0·2% (−5·0 to 4·3)
	Saint Vincent and the Grenadines		11·7 (9·3 to 14·6)	10·6 (6·9 to 16·5)	4·0 (2·6 to 6·3)	2·0 (0·9 to 4·3)	16·6 (10·4 to 26·8)	<0·1 (<0·1 to <0·1)	<0·1 (<0·1 to <0·1)	–2·1% (−5·4 to 1·2)
	Suriname		15·7 (13·9 to 17·6)	20·4 (15·7 to 26·2)	10·7 (8·3 to 13·8)	4·0 (3·0 to 5·3)	34·8 (26·8 to 44·8)	0·1 (0·1 to 0·2)	0·3 (0·2 to 0·5)	–1·7% (−3·1 to −0·3)
	Trinidad and Tobago		12·9 (11·1 to 14·9)	15·9 (9·4 to 25·6)	2·7 (1·5 to 4·4)	2·5 (1·4 to 4·2)	20·9 (12·3 to 34·2)	0·2 (0·1 to 0·2)	0·3 (0·1 to 0·6)	–1·7% (−5·0 to 1·6)
	Virgin Islands		6·2 (5·0 to 7·8)	5·9 (4·8 to 7·1)	2·0 (1·6 to 2·5)	1·2 (0·9 to 1·6)	9·1 (7·4 to 11·2)	<0·1 (<0·1 to <0·1)	<0·1 (<0·1 to <0·1)	–2·7% (−4·0 to −1·4)
Tropical Latin America			5·3 (4·8 to 5·8)	8·8 (6·7 to 11·5)	5·9 (4·5 to 7·8)	2·4 (1·8 to 3·2)	17·1 (12·9 to 22·4)	16·8 (15·3 to 18·4)	54·1 (49·9 to 58·2)	–3·8% (−5·7 to −1·9)
	Brazil		5·3 (4·9 to 5·8)	8·7 (6·6 to 11·4)	5·9 (4·4 to 7·8)	2·4 (1·8 to 3·2)	16·9 (12·8 to 22·1)	16·1 (14·7 to 17·6)	51·7 (47·6 to 55·8)	–3·9% (−5·8 to −1·9)
	Paraguay		5·2 (4·1 to 6·7)	10·6 (8·1 to 13·9)	5·7 (4·3 to 7·6)	2·6 (2·0 to 3·5)	18·9 (14·3 to 24·7)	0·7 (0·5 to 0·9)	2·4 (1·8 to 3·2)	–2·7% (−4·6 to −0·8)
**Southeast Asia, east Asia, and Oceania**			**6·7 (6·4 to 7·0)**	**8·9 (7·8 to 10·1)**	**5·2 (4·6 to 5·8)**	**3·5 (2·9 to 4·1)**	**17·5 (15·3 to 19·9)**	**158·5 (152·4 to 165·7)**	**422·4 (395·2 to 451·2)**	**–5·4% (−6·3 to −4·5)**
East Asia			5·6 (5·2 to 6·0)	6·2 (5·4 to 7·2)	3·7 (3·3 to 4·3)	2·5 (2·0 to 3·2)	12·4 (10·6 to 14·6)	67·2 (62·3 to 72·6)	154·5 (140·7 to 170·4)	–6·8% (−8·1 to −5·6)
	China		5·5 (5·1 to 6·0)	5·9 (5·2 to 6·8)	3·5 (3·1 to 4·0)	2·4 (2·0 to 3·0)	11·8 (10·2 to 13·7)	62·2 (57·4 to 67·4)	137·5 (125·4 to 151·0)	–6·8% (−7·9 to −5·7)
	North Korea		7·0 (6·1 to 8·2)	12·9 (10·2 to 16·5)	8·5 (6·8 to 10·9)	5·0 (3·5 to 7·3)	26·2 (20·4 to 34·3)	4·3 (3·7 to 5·0)	16·1 (10·4 to 25·0)	–7·6% (−11·1 to −2·7)
	Taiwan (province of China)		4·0 (3·8 to 4·2)	2·2 (2·0 to 2·4)	1·5 (1·4 to 1·7)	1·0 (0·8 to 1·3)	4·7 (4·2 to 5·4)	0·8 (0·7 to 0·8)	0·9 (0·8 to 1·1)	–3·8% (−4·5 to −2·9)
Southeast Asia			7·5 (7·4 to 7·8)	11·5 (9·9 to 13·4)	6·5 (5·7 to 7·5)	4·4 (3·7 to 5·2)	22·3 (19·2 to 25·8)	85·8 (83·5 to 88·3)	254·4 (231·9 to 278·7)	–4·6% (−5·6 to −3·7)
	Cambodia		10·8 (10·5 to 11·2)	15·7 (12·7 to 19·3)	11·5 (9·3 to 14·0)	5·0 (4·3 to 5·8)	31·9 (26·1 to 38·6)	4·3 (4·1 to 4·4)	12·5 (9·9 to 15·4)	–6·8% (−8·2 to −5·5)
	Indonesia		8·7 (8·5 to 9·0)	13·5 (11·0 to 16·6)	7·4 (5·9 to 9·4)	4·5 (3·6 to 5·6)	25·2 (20·5 to 31·0)	38·7 (37·7 to 39·9)	112·7 (102·3 to 124·2)	–4·8% (−6·1 to −3·4)
	Laos		15·8 (15·5 to 16·0)	25·9 (18·3 to 35·4)	23·9 (17·0 to 33·1)	9·5 (6·0 to 14·4)	58·2 (40·9 to 80·4)	4·0 (4·0 to 4·1)	14·9 (7·5 to 25·6)	–5·5% (−8·0 to −3·2)
	Malaysia		3·5 (3·2 to 3·7)	2·9 (2·5 to 3·3)	1·9 (1·6 to 2·2)	1·3 (1·1 to 1·5)	6·0 (5·2 to 6·9)	1·8 (1·6 to 1·9)	3·1 (2·6 to 3·6)	–2·8% (−3·7 to −1·9)
	Maldives		4·8 (4·2 to 5·4)	4·6 (4·0 to 5·3)	1·3 (1·0 to 1·6)	1·6 (1·4 to 1·8)	7·5 (6·6 to 8·6)	<0·1 (<0·1 to <0·1)	<0·1 (<0·1 to 0·1)	–8·9% (−9·7 to −8·1)
	Mauritius		7·7 (7·1 to 8·4)	8·8 (8·0 to 9·6)	3·4 (3·0 to 3·9)	1·8 (1·4 to 2·3)	13·9 (12·5 to 15·6)	0·1 (0·1 to 0·1)	0·2 (0·1 to 0·2)	–1·6% (−2·3 to −0·9)
	Myanmar		7·6 (7·5 to 7·7)	15·0 (11·3 to 20·3)	8·0 (6·1 to 10·6)	5·0 (3·6 to 7·1)	27·7 (20·8 to 37·5)	7·1 (7·0 to 7·2)	26·2 (15·5 to 44·2)	–6·9% (−8·9 to −4·8)
	Philippines		7·1 (7·0 to 7·2)	11·6 (9·8 to 13·7)	6·6 (5·6 to 7·8)	6·1 (5·2 to 7·3)	24·1 (20·4 to 28·6)	17·3 (17·1 to 17·6)	58·0 (47·9 to 69·7)	–3·0% (−4·0 to −1·9)
	Sri Lanka		3·7 (3·5 to 3·9)	4·0 (3·0 to 5·2)	1·6 (1·2 to 2·0)	1·2 (0·9 to 1·8)	6·8 (5·1 to 9·0)	1·0 (1·0 to 1·1)	1·9 (1·2 to 2·9)	–5·7% (−7·5 to −3·9)
	Seychelles		6·8 (6·3 to 7·4)	7·8 (6·4 to 9·4)	1·7 (1·4 to 2·1)	1·9 (1·5 to 2·5)	11·4 (9·3 to 13·9)	<0·1 (<0·1 to <0·1)	<0·1 (<0·1 to <0·1)	–1·0% (−2·4 to 0·4)
	Thailand		3·5 (3·2 to 3·8)	3·3 (2·6 to 4·1)	1·6 (1·3 to 2·0)	1·6 (1·2 to 2·0)	6·4 (5·1 to 8·0)	1·9 (1·8 to 2·1)	3·7 (2·6 to 5·0)	–5·6% (−7·1 to −3·9)
	Timor–Leste		13·1 (13·0 to 13·2)	15·1 (8·1 to 25·4)	13·8 (8·7 to 21·1)	6·7 (4·3 to 10·3)	35·3 (21·1 to 55·4)	0·4 (0·4 to 0·4)	1·2 (0·6 to 1·9)	–6·5% (−9·8 to −3·6)
	Vietnam		6·1 (5·7 to 6·5)	7·0 (5·8 to 8·5)	3·1 (2·5 to 3·9)	3·1 (2·6 to 3·8)	13·1 (10·9 to 16·1)	9·1 (8·5 to 9·7)	19·7 (14·9 to 26·4)	–4·7% (−5·8 to −3·5)
Oceania			18·6 (17·1 to 20·4)	18·9 (10·7 to 31·5)	15·1 (8·2 to 25·6)	11·6 (6·6 to 19·6)	45·0 (26·0 to 73·8)	5·6 (5·1 to 6·1)	13·5 (8·0 to 22·2)	–2·2% (−5·6 to 0·7)
	American Samoa		4·5 (4·1 to 4·9)	3·8 (3·0 to 4·7)	3·0 (2·4 to 3·7)	1·8 (1·3 to 2·4)	8·5 (6·7 to 10·7)	<0·1 (<0·1 to <0·1)	<0·1 (<0·1 to <0·1)	–3·1% (−4·7 to −1·6)
	Federated States of Micronesia		8·2 (7·5 to 9·0)	9·0 (5·7 to 13·9)	4·8 (2·8 to 7·5)	3·6 (2·3 to 5·5)	17·3 (11·2 to 26·1)	<0·1 (<0·1 to <0·1)	<0·1 (<0·1 to 0·1)	–4·4% (−7·9 to −1·1)
	Fiji		12·3 (11·9 to 12·7)	17·0 (10·6 to 26·3)	11·8 (6·7 to 19·2)	9·4 (4·7 to 17·3)	37·8 (22·4 to 61·0)	0·1 (0·1 to 0·1)	0·4 (0·2 to 0·6)	1·3% (−2·4 to 4·7)
	Guam		9·9 (9·6 to 10·3)	7·4 (5·6 to 9·6)	4·6 (3·4 to 6·1)	1·8 (1·2 to 2·5)	13·7 (10·2 to 18·1)	<0·1 (<0·1 to <0·1)	<0·1 (<0·1 to 0·1)	1·2% (−0·7 to 3·2)
	Kiribati		16·7 (15·4 to 18·4)	19·8 (10·1 to 35·7)	16·3 (8·0 to 29·6)	13·1 (6·7 to 23·7)	48·4 (25·3 to 85·4)	<0·1 (<0·1 to 0·1)	0·1 (0·1 to 0·2)	–1·9% (−6·1 to 2·0)
	Marshall Islands		9·0 (8·3 to 9·9)	10·5 (5·5 to 18·0)	5·8 (2·9 to 10·8)	6·0 (3·1 to 10·6)	22·2 (11·8 to 37·9)	<0·1 (<0·1 to <0·1)	<0·1 (<0·1 to 0·1)	–3·7% (−8·6 to 0·8)
	Northern Mariana Islands		2·4 (2·2 to 2·6)	1·2 (0·6 to 2·0)	0·6 (0·3 to 0·9)	0·7 (0·4 to 1·2)	2·5 (1·4 to 4·1)	<0·1 (<0·1 to <0·1)	<0·1 (<0·1 to <0·1)	–3·8% (−8·0 to −0·3)
	Papua New Guinea		20·6 (18·9 to 22·6)	20·6 (11·3 to 35·3)	17·0 (9·0 to 29·2)	13·1 (7·3 to 22·4)	49·9 (28·3 to 83·0)	4·9 (4·5 to 5·5)	11·6 (6·3 to 19·9)	–2·5% (−6·0 to 0·6)
	Samoa		5·0 (4·6 to 5·5)	4·7 (2·1 to 9·2)	2·7 (1·2 to 5·5)	3·0 (1·5 to 5·7)	10·4 (4·8 to 20·2)	<0·1 (<0·1 to <0·1)	0·1 (<0·1 to 0·1)	–2·6% (−7·0 to 1·9)
	Solomon Islands		12·0 (11·0 to 13·2)	12·4 (7·9 to 19·1)	7·5 (4·4 to 11·7)	5·8 (3·7 to 8·6)	25·5 (16·7 to 37·9)	0·2 (0·2 to 0·2)	0·4 (0·3 to 0·7)	–2·1% (−4·9 to 0·6)
	Tonga		8·7 (8·0 to 9·5)	9·9 (5·1 to 17·6)	5·0 (2·4 to 9·3)	3·7 (1·9 to 6·7)	18·6 (9·8 to 32·9)	<0·1 (<0·1 to <0·1)	<0·1 (<0·1 to 0·1)	–1·5% (−4·9 to 1·7)
	Vanuatu		12·9 (11·9 to 14·2)	14·5 (11·4 to 18·0)	10·5 (5·1 to 20·3)	6·9 (4·3 to 11·0)	31·6 (20·7 to 48·6)	0·1 (0·1 to 0·1)	0·3 (0·2 to 0·4)	–1·4% (−4·5 to 1·5)
**North Africa and Middle East**			**10·4 (9·6 to 11·5)**	**13·4 (11·3 to 16·0)**	**8·8 (7·4 to 10·5)**	**6·0 (4·9 to 7·5)**	**27·9 (23·4 to 33·6)**	**132·8 (121·7 to 146·6)**	**357·1 (295·8 to 423·9)**	**–3·7% (−4·8 to −2·7)**
Afghanistan			15·7 (13·6 to 18·1)	25·9 (20·2 to 33·9)	24·0 (19·4 to 30·4)	18·3 (14·0 to 24·0)	66·6 (52·9 to 85·7)	18·5 (16·0 to 21·4)	74·8 (49·2 to 104·3)	–4·5% (−5·9 to −3·0)
Algeria			12·2 (10·9 to 13·6)	10·9 (9·0 to 13·3)	5·0 (3·5 to 7·5)	2·4 (1·8 to 3·3)	18·2 (14·2 to 23·9)	10·7 (9·6 to 12·0)	16·0 (10·4 to 24·3)	–3·7% (−5·4 to −2·0)
Bahrain			5·1 (5·0 to 5·2)	2·7 (2·2 to 3·2)	2·8 (2·2 to 3·5)	1·4 (1·0 to 2·0)	6·9 (5·4 to 8·7)	0·1 (0·1 to 0·1)	0·1 (0·1 to 0·2)	–3·6% (−5·3 to −2·0)
Egypt			9·1 (7·8 to 10·9)	9·2 (6·3 to 13·4)	6·6 (4·5 to 9·5)	3·6 (2·5 to 5·2)	19·3 (13·2 to 27·8)	19·5 (16·6 to 23·3)	41·7 (28·5 to 60·7)	–5·1% (−7·6 to −2·8)
Iran			7·2 (6·6 to 7·8)	10·9 (7·7 to 15·0)	4·3 (3·0 to 6·0)	2·7 (1·9 to 3·8)	17·8 (12·6 to 24·5)	11·1 (10·3 to 12·1)	27·9 (11·0 to 59·2)	–5·2% (−7·8 to −2·8)
Iraq			11·3 (9·7 to 13·5)	14·6 (12·0 to 18·3)	8·2 (6·5 to 10·5)	5·9 (4·0 to 9·0)	28·5 (22·5 to 36·5)	19·0 (16·1 to 22·7)	46·4 (21·8 to 70·5)	–2·5% (−3·9 to −0·9)
Jordan			6·5 (6·0 to 7·2)	10·1 (8·3 to 12·6)	4·1 (3·6 to 4·7)	3·1 (2·3 to 4·1)	17·2 (14·2 to 21·3)	1·3 (1·2 to 1·4)	3·4 (2·7 to 4·3)	–2·8% (−4·1 to −1·4)
Kuwait			5·4 (4·7 to 6·3)	5·1 (3·9 to 6·5)	2·9 (2·2 to 3·7)	1·5 (1·1 to 2·0)	9·4 (7·2 to 12·2)	0·3 (0·3 to 0·3)	0·5 (0·4 to 0·7)	–1·9% (−3·7 to −0·2)
Lebanon			5·9 (5·7 to 6·1)	4·9 (3·6 to 6·5)	3·0 (2·2 to 4·0)	1·6 (1·1 to 2·4)	9·5 (7·0 to 12·5)	0·4 (0·4 to 0·4)	0·6 (0·4 to 0·9)	–4·1% (−6·6 to −1·7)
Libya			4·9 (4·2 to 5·9)	6·4 (4·5 to 8·8)	3·4 (2·4 to 4·8)	4·2 (2·7 to 6·5)	13·9 (9·7 to 19·7)	0·4 (0·3 to 0·5)	1·1 (0·7 to 1·6)	–4·1% (−6·1 to −2·2)
Morocco			9·5 (8·9 to 10·2)	13·4 (10·1 to 17·7)	5·6 (4·2 to 7·5)	2·8 (2·1 to 3·7)	21·7 (16·4 to 28·6)	4·3 (4·0 to 4·5)	9·7 (7·1 to 13·0)	–4·6% (−6·2 to −3·2)
Palestine			5·3 (4·7 to 6·1)	8·8 (6·1 to 12·3)	5·1 (3·4 to 7·2)	2·9 (2·0 to 4·1)	16·7 (12·0 to 22·8)	1·2 (1·1 to 1·4)	3·9 (1·8 to 6·3)	–3·0% (−5·3 to −0·9)
Oman			6·4 (6·1 to 6·8)	4·2 (3·7 to 4·8)	2·3 (2·0 to 2·6)	1·5 (1·3 to 1·7)	8·0 (7·0 to 9·2)	0·6 (0·6 to 0·6)	0·7 (0·5 to 1·0)	–4·2% (−5·5 to −3·0)
Qatar			4·5 (3·5 to 6·0)	4·8 (3·2 to 7·1)	2·8 (1·8 to 4·1)	1·8 (1·2 to 2·7)	9·4 (6·2 to 13·8)	0·1 (0·1 to 0·2)	0·2 (0·2 to 0·4)	–3·5% (−6·9 to −0·3)
Saudi Arabia			6·8 (6·0 to 7·8)	3·4 (2·3 to 5·1)	2·0 (1·4 to 2·9)	1·2 (0·8 to 1·8)	6·6 (4·4 to 9·8)	3·1 (2·7 to 3·5)	3·1 (2·5 to 3·9)	–8·0% (−9·9 to −6·0)
Sudan			18·1 (16·4 to 19·9)	26·6 (21·1 to 33·9)	20·4 (15·2 to 28·1)	15·8 (12·7 to 20·0)	61·6 (48·3 to 79·8)	16·1 (14·6 to 17·8)	55·0 (31·1 to 94·1)	–2·7% (−4·3 to −1·0)
Syria			4·8 (4·1 to 5·7)	6·5 (5·1 to 7·9)	4·6 (2·9 to 7·0)	16·1 (5·6 to 32·6)	27·0 (13·7 to 46·9)	1·4 (1·2 to 1·7)	8·9 (4·4 to 13·4)	2·5% (−1·7 to 6·0)
Tunisia			8·8 (7·2 to 10·7)	7·4 (5·9 to 9·2)	3·0 (2·4 to 3·8)	2·2 (1·7 to 2·8)	12·5 (10·0 to 15·7)	1·4 (1·1 to 1·6)	2·0 (1·5 to 2·6)	–4·7% (−6·0 to −3·4)
Turkey			7·5 (6·4 to 8·9)	8·8 (6·2 to 12·3)	4·1 (2·8 to 5·7)	2·9 (2·0 to 4·1)	15·7 (11·0 to 22·1)	9·0 (7·6 to 10·7)	18·9 (13·0 to 26·9)	–5·9% (−8·4 to −3·4)
United Arab Emirates			2·8 (2·5 to 3·0)	2·7 (1·4 to 4·9)	1·7 (0·9 to 3·0)	1·3 (0·7 to 2·4)	5·7 (3·0 to 10·3)	0·4 (0·4 to 0·5)	0·9 (0·5 to 1·6)	–3·8% (−9·2 to 1·5)
Yemen			14·5 (13·6 to 15·6)	19·8 (15·5 to 25·6)	15·3 (13·8 to 17·0)	9·1 (6·7 to 12·4)	43·6 (36·1 to 53·8)	13·9 (13·0 to 14·9)	41·0 (18·3 to 65·8)	–4·4% (−5·7 to −3·1)
**South Asia**			**17·4 (16·7 to 18·1)**	**23·2 (20·4 to 26·7)**	**10·6 (9·5 to 11·9)**	**7·4 (6·4 to 8·6)**	**40·6 (35·9 to 46·6)**	**534·2 (513·2 to 556·0)**	**1240·5 (1161·0 to 1324·1)**	**–4·6% (−5·4 to −3·8)**
Bangladesh			16·1 (15·6 to 16·6)	21·0 (18·4 to 23·8)	7·5 (6·4 to 8·8)	5·9 (5·2 to 6·6)	34·0 (30·3 to 38·2)	45·9 (44·5 to 47·4)	96·5 (81·9 to 114·0)	–5·8% (−6·6 to −5·1)
Bhutan			14·1 (13·7 to 14·6)	19·8 (16·7 to 24·0)	8·0 (6·8 to 9·5)	5·4 (4·4 to 6·8)	32·9 (27·6 to 39·8)	0·2 (0·2 to 0·2)	0·5 (0·4 to 0·6)	–6·0% (−6·8 to −4·9)
India			15·8 (15·1 to 16·6)	21·8 (17·8 to 27·1)	10·7 (9·5 to 12·2)	7·2 (6·0 to 8·7)	39·2 (33·0 to 47·3)	350·2 (333·9 to 367·3)	865·6 (816·2 to 919·5)	–4·7% (−5·7 to −3·6)
Nepal			15·2 (14·7 to 15·8)	18·5 (16·0 to 21·3)	7·1 (6·2 to 8·1)	4·0 (3·2 to 5·1)	29·4 (25·8 to 33·5)	12·8 (12·3 to 13·3)	24·3 (17·5 to 32·4)	–6·3% (−7·1 to −5·6)
Pakistan			25·9 (25·1 to 26·8)	31·8 (27·8 to 36·4)	12·6 (10·8 to 14·5)	9·9 (8·6 to 11·4)	53·4 (46·9 to 61·1)	125·1 (121·0 to 129·5)	253·6 (195·9 to 319·9)	–3·3% (−4·1 to −2·5)
**Sub–Saharan Africa**			**21·3 (20·2 to 22·6)**	**25·9 (23·1 to 29·3)**	**24·5 (22·2 to 27·2)**	**28·3 (25·2 to 32·0)**	**76·7 (68·9 to 85·6)**	**770·8 (730·9 to 819·1)**	**2654·3 (2453·0 to 2884·2)**	**–4·0% (−4·6 to −3·4)**
Southern sub–Saharan Africa			13·8 (12·5 to 15·4)	17·6 (14·6 to 21·4)	18·8 (16·1 to 22·2)	10·8 (9·0 to 13·0)	46·4 (39·2 to 55·5)	26·0 (23·5 to 29·1)	86·5 (73·5 to 102·4)	–3·1% (−4·2 to −1·9)
	Botswana		5·8 (4·9 to 6·8)	7·2 (5·3 to 10·1)	3·3 (2·2 to 4·9)	4·5 (3·6 to 5·5)	14·9 (11·1 to 20·4)	0·3 (0·3 to 0·4)	0·8 (0·5 to 1·3)	–9·1% (−10·9 to −7·1)
	Lesotho		17·0 (15·6 to 18·7)	27·9 (22·1 to 35·0)	25·7 (20·4 to 32·3)	14·5 (11·3 to 18·4)	66·6 (53·2 to 83·0)	1·0 (0·9 to 1·1)	3·7 (2·7 to 4·9)	–2·5% (−4·0 to −1·1)
	Namibia		8·8 (7·3 to 10·9)	15·9 (10·5 to 23·3)	12·3 (8·0 to 18·3)	8·7 (5·7 to 12·9)	36·5 (24·0 to 53·3)	0·6 (0·5 to 0·8)	2·6 (1·6 to 3·8)	–3·5% (−6·2 to −0·9)
	South Africa		9·8 (8·9 to 10·7)	15·2 (11·6 to 19·8)	19·6 (14·8 to 25·3)	9·2 (7·0 to 12·0)	43·4 (33·2 to 55·9)	10·6 (9·7 to 11·6)	46·3 (35·2 to 60·6)	–3·5% (−5·3 to −1·7)
	Swaziland		7·8 (6·7 to 9·1)	15·9 (13·2 to 19·1)	23·6 (18·5 to 29·7)	10·5 (8·0 to 13·5)	49·2 (39·2 to 61·1)	0·4 (0·3 to 0·4)	2·2 (1·6 to 2·8)	–4·0% (−5·6 to −2·5)
	Zimbabwe		23·1 (19·5 to 27·8)	23·5 (19·7 to 28·4)	19·6 (16·1 to 24·3)	14·4 (12·0 to 17·6)	56·5 (47·3 to 68·6)	13·1 (11·0 to 15·8)	30·9 (25·2 to 38·1)	–2·0% (−3·1 to −0·7)
Western sub–Saharan Africa			26·1 (23·9 to 28·5)	31·6 (27·9 to 35·8)	28·4 (26·2 to 31·0)	40·7 (36·3 to 46·1)	97·3 (87·8 to 108·7)	394·6 (361·9 to 432·2)	1403·8 (1257·0 to 1564·1)	–3·5% (−4·1 to −2·8)
	Benin		15·7 (15·2 to 16·2)	25·1 (21·6 to 29·4)	22·3 (17·9 to 28·4)	26·6 (22·2 to 32·5)	72·2 (60·5 to 87·5)	7·0 (6·8 to 7·2)	30·8 (24·9 to 38·0)	–4·1% (−5·2 to −3·0)
	Burkina Faso		13·1 (12·7 to 13·5)	28·7 (24·5 to 34·3)	33·6 (29·9 to 38·0)	50·6 (43·4 to 60·0)	108·8 (94·7 to 126·7)	9·8 (9·5 to 10·1)	77·8 (54·0 to 101·9)	–3·1% (−3·9 to −2·2)
	Cameroon		17·1 (16·0 to 18·4)	27·2 (21·6 to 34·0)	27·9 (22·0 to 35·2)	35·7 (27·9 to 45·2)	88·1 (70·1 to 110·1)	15·2 (14·2 to 16·5)	75·7 (59·6 to 96·0)	–2·7% (−4·2 to −1·3)
	Cape Verde		7·4 (6·4 to 8·6)	8·9 (7·3 to 10·6)	5·0 (4·7 to 5·3)	3·3 (2·9 to 3·8)	17·0 (14·9 to 19·5)	0·1 (0·1 to 0·1)	0·3 (0·2 to 0·3)	–6·1% (−7·2 to −4·9)
	Chad		28·6 (27·7 to 29·6)	30·8 (26·9 to 35·6)	37·1 (31·9 to 43·7)	49·6 (42·3 to 59·2)	113·1 (97·9 to 132·3)	18·8 (18·2 to 19·5)	69·1 (59·3 to 81·5)	–3·0% (−3·8 to −2·1)
	Côte d'Ivoire		19·3 (18·8 to 19·8)	32·1 (27·1 to 37·9)	27·8 (23·5 to 32·8)	28·0 (23·6 to 33·3)	85·3 (72·5 to 100·4)	16·2 (15·8 to 16·6)	68·6 (51·0 to 88·3)	–3·0% (−4·0 to −1·9)
	The Gambia		21·0 (20·4 to 21·8)	21·3 (18·2 to 25·4)	12·2 (10·4 to 14·5)	16·0 (13·5 to 19·1)	48·7 (41·5 to 57·9)	1·7 (1·7 to 1·8)	3·9 (3·1 to 4·7)	–3·6% (−4·6 to −2·6)
	Ghana		15·4 (13·9 to 17·2)	23·4 (20·0 to 27·7)	13·5 (11·1 to 16·9)	16·2 (13·4 to 20·1)	52·3 (44·0 to 63·2)	14·6 (13·2 to 16·3)	48·3 (38·1 to 60·4)	–4·1% (−5·3 to −2·9)
	Guinea		16·8 (16·2 to 17·4)	32·1 (26·2 to 39·9)	29·1 (24·3 to 35·5)	39·6 (32·4 to 49·3)	97·5 (80·6 to 119·6)	8·0 (7·7 to 8·3)	44·5 (36·8 to 54·6)	–3·6% (−4·7 to −2·3)
	Guinea–Bissau		20·9 (19·5 to 22·5)	29·0 (25·3 to 33·1)	18·6 (16·1 to 21·4)	26·0 (22·0 to 30·5)	71·8 (62·0 to 82·7)	1·5 (1·4 to 1·6)	4·9 (4·2 to 5·6)	–4·9% (−5·8 to −4·0)
	Liberia		15·3 (14·4 to 16·6)	22·4 (20·1 to 25·3)	23·4 (21·1 to 26·2)	20·9 (16·2 to 27·3)	65·3 (56·3 to 76·7)	2·5 (2·3 to 2·7)	10·1 (8·4 to 12·2)	–6·0% (−7·0 to −5·0)
	Mali		28·9 (27·1 to 31·2)	38·0 (32·0 to 45·8)	34·3 (29·9 to 39·6)	49·6 (39·0 to 64·2)	117·1 (97·6 to 142·4)	22·0 (20·6 to 23·8)	84·2 (69·7 to 102·4)	–3·1% (−4·3 to −1·8)
	Mauritania		14·9 (14·6 to 15·4)	23·9 (20·4 to 28·0)	14·0 (12·1 to 16·4)	13·2 (11·2 to 15·5)	50·3 (43·1 to 58·8)	1·7 (1·6 to 1·7)	5·6 (4·7 to 6·5)	–3·8% (−4·7 to −2·9)
	Niger		20·2 (19·5 to 20·9)	26·3 (21·2 to 33·1)	29·2 (25·0 to 34·6)	56·6 (47·4 to 68·6)	108·2 (90·9 to 130·6)	18·3 (17·7 to 19·0)	91·7 (76·4 to 111·0)	–4·6% (−5·6 to −3·4)
	Nigeria		34·3 (30·2 to 39·0)	35·7 (28·8 to 43·5)	30·5 (27·5 to 33·8)	46·6 (39·5 to 54·8)	108·7 (93·0 to 126·2)	238·4 (209·2 to 272·4)	717·2 (577·1 to 868·7)	–3·3% (−4·3 to −2·3)
	São Tomé and Príncipe		9·1 (8·5 to 9·8)	12·8 (10·7 to 15·2)	9·8 (8·3 to 11·6)	8·1 (6·8 to 9·7)	30·4 (25·6 to 36·1)	0·1 (0·1 to 0·1)	0·2 (0·2 to 0·3)	–5·5% (−6·7 to −4·4)
	Senegal		14·2 (11·7 to 17·0)	20·1 (17·8 to 23·0)	13·9 (11·8 to 16·5)	16·2 (14·4 to 18·4)	49·4 (43·9 to 56·3)	8·1 (6·6 to 9·7)	27·1 (23·2 to 31·8)	–5·3% (−6·0 to −4·5)
	Sierra Leone		24·8 (23·2 to 26·8)	34·8 (31·0 to 39·0)	42·9 (37·9 to 48·4)	42·6 (36·2 to 50·0)	115·6 (101·5 to 131·2)	6·2 (5·8 to 6·7)	27·5 (24·1 to 31·2)	–3·9% (−4·7 to −3·1)
	Togo		18·4 (17·7 to 19·1)	25·1 (21·5 to 29·7)	19·6 (17·6 to 22·0)	25·9 (21·9 to 31·3)	69·0 (59·7 to 80·8)	4·6 (4·4 to 4·8)	16·5 (12·8 to 20·6)	–3·6% (−4·4 to −2·6)
Eastern sub–Saharan Africa			16·9 (16·4 to 17·5)	21·8 (18·7 to 25·8)	20·8 (18·3 to 24·2)	18·1 (15·5 to 21·5)	59·6 (51·6 to 69·7)	239·7 (232·6 to 247·8)	813·3 (762·1 to 865·6)	–5·0% (−5·9 to −4·0)
	Burundi		16·1 (15·9 to 16·4)	27·3 (17·6 to 42·5)	27·4 (19·1 to 39·8)	28·4 (20·9 to 39·2)	80·8 (56·5 to 116·7)	8·1 (8·0 to 8·2)	38·8 (27·1 to 56·2)	–4·4% (−6·8 to −2·1)
	Comoros		19·0 (18·0 to 20·2)	26·4 (21·6 to 32·1)	16·8 (10·6 to 26·3)	10·4 (8·0 to 14·0)	52·6 (39·9 to 70·6)	0·4 (0·4 to 0·4)	1·0 (0·7 to 1·4)	–3·9% (−5·8 to −1·9)
	Djibouti		19·4 (19·0 to 19·8)	18·0 (14·4 to 22·9)	17·2 (14·5 to 20·5)	13·6 (10·8 to 17·6)	48·0 (39·2 to 59·8)	0·7 (0·7 to 0·7)	1·8 (1·1 to 2·6)	–4·7% (−5·8 to −3·6)
	Eritrea		13·9 (13·2 to 14·8)	17·7 (13·4 to 23·8)	16·4 (13·6 to 20·1)	18·0 (14·1 to 23·5)	51·2 (40·5 to 65·8)	2·3 (2·2 to 2·5)	8·4 (6·2 to 11·1)	–3·7% (−5·2 to −2·3)
	Ethiopia		11·6 (11·3 to 11·9)	18·8 (15·7 to 23·0)	13·4 (11·5 to 15·6)	11·9 (10·2 to 14·0)	43·4 (37·0 to 51·7)	40·1 (39·1 to 41·3)	144·8 (121·9 to 173·1)	–7·3% (−8·3 to −6·3)
	Kenya		18·2 (17·2 to 19·3)	18·0 (15·0 to 21·3)	15·0 (12·4 to 18·3)	11·1 (9·3 to 13·2)	43·4 (36·9 to 51·2)	25·8 (24·3 to 27·4)	59·0 (55·9 to 62·6)	–4·6% (−5·7 to −3·4)
	Madagascar		20·0 (18·9 to 21·3)	26·6 (21·8 to 33·2)	29·2 (23·4 to 36·9)	26·1 (19·6 to 35·4)	79·7 (63·4 to 101·9)	18·1 (17·1 to 19·3)	69·2 (53·9 to 88·9)	–2·4% (−4·0 to −0·8)
	Malawi		15·7 (15·4 to 16·1)	24·2 (19·5 to 30·6)	23·8 (19·1 to 30·2)	22·6 (17·8 to 29·3)	69·0 (55·3 to 87·4)	11·5 (11·2 to 11·8)	48·7 (39·2 to 61·9)	–5·5% (−6·9 to −4·0)
	Mozambique		19·6 (18·5 to 20·9)	24·6 (21·6 to 28·2)	30·6 (26·0 to 36·6)	22·9 (19·6 to 27·3)	76·1 (65·9 to 89·2)	22·1 (20·9 to 23·5)	82·9 (67·9 to 99·9)	–4·7% (−5·6 to −3·7)
	Rwanda		12·7 (11·8 to 13·6)	18·0 (14·9 to 22·0)	16·5 (12·8 to 21·6)	15·6 (12·4 to 20·0)	49·2 (39·6 to 62·3)	5·1 (4·8 to 5·5)	19·5 (15·2 to 24·9)	–7·2% (−8·7 to −5·7)
	Somalia		24·0 (22·7 to 25·5)	28·8 (24·5 to 34·4)	34·8 (29·6 to 41·3)	37·0 (29·3 to 46·9)	97·2 (81·3 to 117·8)	7·4 (7·0 to 7·9)	28·5 (20·3 to 37·6)	–2·9% (−4·0 to −1·9)
	South Sudan		43·4 (42·4 to 44·5)	30·7 (23·0 to 40·3)	34·6 (28·8 to 41·1)	31·3 (23·4 to 41·3)	93·5 (73·4 to 117·4)	30·9 (30·2 to 31·7)	61·5 (46·7 to 80·0)	–2·7% (−4·2 to −1·1)
	Tanzania		15·3 (14·9 to 15·8)	21·0 (18·4 to 24·4)	19·6 (16·2 to 23·9)	16·2 (13·6 to 19·6)	55·7 (48·2 to 65·3)	30·8 (29·9 to 31·8)	108·5 (85·9 to 134·3)	–5·0% (−5·9 to −4·0)
	Uganda		15·1 (15·0 to 15·3)	22·3 (19·6 to 25·8)	22·3 (19·9 to 25·3)	19·1 (16·6 to 22·4)	62·4 (55·0 to 71·8)	26·0 (25·7 to 26·3)	103·1 (90·3 to 118·4)	–4·8% (−5·6 to −3·9)
	Zambia		16·1 (15·3 to 17·2)	20·9 (16·5 to 26·3)	20·7 (16·3 to 25·7)	19·4 (15·4 to 24·2)	59·7 (47·7 to 73·9)	10·5 (9·9 to 11·2)	37·3 (29·3 to 46·7)	–5·4% (−6·9 to −4·0)
Central sub–Saharan Africa			22·3 (21·7 to 23·1)	23·6 (17·8 to 30·8)	25·7 (19·3 to 33·8)	27·5 (20·4 to 36·4)	74·9 (56·5 to 97·4)	110·5 (107·3 to 114·4)	350·7 (238·9 to 483·4)	–4·4% (−6·2 to −2·7)
	Angola		18·3 (17·5 to 19·3)	18·1 (13·4 to 23·9)	18·6 (13·5 to 24·5)	18·7 (13·6 to 25·2)	54·5 (40·4 to 71·8)	20·7 (19·8 to 21·8)	59·2 (39·0 to 85·1)	–6·4% (−8·4 to −4·4)
	Central African Republic		42·2 (39·7 to 45·3)	39·5 (29·9 to 53·0)	46·7 (34·1 to 64·8)	50·4 (36·5 to 70·6)	130·5 (97·2 to 176·9)	7·9 (7·4 to 8·5)	22·8 (16·8 to 31·3)	–2·0% (−4·2 to 0·0)
	Congo (Brazzaville)		17·7 (16·6 to 19·0)	18·9 (13·4 to 26·6)	16·2 (11·6 to 23·3)	18·7 (13·3 to 27·0)	52·8 (37·8 to 74·9)	2·9 (2·7 to 3·1)	8·4 (5·9 to 12·0)	–4·2% (−6·5 to −2·0)
	Democratic Republic of the Congo		22·9 (22·4 to 23·4)	24·9 (18·2 to 33·3)	27·7 (20·0 to 37·4)	30·1 (21·6 to 40·9)	80·5 (58·5 to 107·7)	77·7 (76·0 to 79·5)	256·8 (148·4 to 384·4)	–4·0% (−6·1 to −2·0)
	Equatorial Guinea		19·2 (18·1 to 20·6)	20·2 (14·5 to 28·5)	19·6 (13·6 to 28·5)	18·3 (11·1 to 30·4)	57·0 (38·6 to 85·0)	0·4 (0·4 to 0·5)	1·3 (0·8 to 1·9)	–5·9% (−8·1 to −3·7)
	Gabon		17·7 (16·7 to 19·0)	19·2 (15·4 to 24·5)	13·4 (10·9 to 16·6)	11·7 (9·5 to 14·4)	43·7 (35·5 to 54·6)	1·0 (0·9 to 1·0)	2·3 (1·6 to 3·1)	–2·9% (−4·2 to −1·6)

Data in parentheses are 95% uncertainty intervals. To download the data in this table, please visit the Global Health Data Exchange (GHDx).

Regionally, stillbirth rates were highest among the countries of central sub-Saharan Africa, where rates exceeded 23 per 1000 in 2016. Rates were highly variable across south and southeast Asia, spanning 3·5 per 1000 (3·2–3·7) in Malaysia to 25·9 per 1000 (25·1–26·8) in Pakistan. Only six countries in western Europe had stillbirth rates below 1·5 per 1000 in 2016. Across the Americas, no country had a stillbirth rate below 1·5 in 2016. For 114 of 195 countries, decreases in stillbirth rates were most rapid in the most recent decades; annualised stillbirth rates in these countries decreased faster in the years after 2000 than in the period 1990–2000.

Rates of mortality for children younger than 5 years decreased globally between 2000 and 2016, from 69·4 per 1000 livebirths (67·2–71·8) to 38·4 per 1000 livebirths (34·5–43·1); since 2000, U5MR has decreased in 189 of 195 countries. Table 1 also shows the variation in levels of U5MR in 2016, which ranged from 2·2 per 1000 livebirths (1·8–2·6) in Luxembourg to 130·6 per 1000 livebirths (97·2–176·9) in the Central African Republic. Not only were levels highly variable, but there was considerable variation in rates of change over the period 2000–16. The largest annualised change for this time period was estimated for Botswana, with a decrease of 9·1% (7·1–10·9). In other locations, rates of change ranged from an annualised decrease of 8·9% (8·1–9·7) in the Maldives to an annualised increase of 2·5% (–1·7 to 6·0) in Syria. In the SDG era, the target for U5MR has been set as 25 deaths per 1000 livebirths by 2030 with a target for neonatal mortality of 12 deaths per 1000 livebirths. As of 2016, the SDG target for U5MR had been met or exceeded in 121 countries and the SDG target for neonatal death rate had been met or exceeded by 118 countries.

Age-specific mortality rates for children younger than 5 years varied across locations (table 1). Neonatal mortality was greater than mortality in the 1–4 year age group in 19 of the 21 GBD regions; for example, in southern sub-Saharan Africa, rates of mortality for neonates in 2016 (17·6 deaths per 1000 livebirths, 95% UI 14·6–21·4) were 1·6 times greater than those for the 1–4 year age group (10·8 per 1000 livebirths, 9·0–13·0). Mortality rates were highest for children aged 1–4 years in the GBD regions of western sub-Saharan Africa and central sub-Saharan Africa (18·1 per 1000 livebirths, 15·5–21·5) and central sub-Saharan Africa (27·5 per 1000 livebirths, 20·4–36·4). The range of mortality rates across countries was also largest within this age group, from 0·4 deaths per 1000 (0·3–0·6) in Finland to 56·6 per 1000 (46·4–68·6) in Niger in 2016.

Regionally, 24·8% of under-5 deaths in 2016 occurred in South Asia (1·2 million deaths, 95% UI 1·2 million to 1·3 million), with a further 28·1% in western sub-Saharan Africa (1·4 million deaths, 1·3 million to 1·6 million), and 16·3% in eastern sub-Saharan Africa (0·8 million, 0·8 million to 0·9 million). In absolute terms, the largest number of under-5 deaths nationally in 2016 occurred in India at 0·9 million (0·8 million to 0·9 million) followed by Nigeria (0·7 million, 0·6 million to 0·9 million) and the Democratic Republic of the Congo (0·3 million, 0·1 million to 0·4 million).

### Adult mortality and life expectancy in 2016

Summary measures of adult mortality, life expectancy at birth, and life expectancy at age 65 years in 2016 are presented in table 2. Among countries with populations greater than 1 million in 2016, mortality rates were highest in the countries of sub-Saharan Africa, where 16 of 46 countries had mortality rates in excess of 1500 deaths per 100 000, including the highest global age-standardised mortality rate of 2470·7 per 100 000 in the Central African Republic. The lowest age-standardised mortality rates in 2016 were in countries in the high-income Asia Pacific region: the lowest rate globally was in Japan at 379·0 per 100 000.

**Table 2:** Life expectancy at birth and at age 65 years, age-standardised death rates, and total deaths by sex for global, SDI groups, GBD regions and super-regions, countries, and territories in 2016

			**Life expectancy at birth**		**Life expectancy at age 65 years**		**Age-standardised death rate (per 100 000)**		**Total deaths (thousands)**	
			Male	Female	Male	Female	Male	Female	Male	Female
**Global**			**69·8 (69·3 to 70·2)**	**75·3 (75·0 to 75·6)**	**15·7 (15·6 to 15·8)**	**18·6 (18·4 to 18·7)**	**1002·4 (985·1 to 1020·8)**	**690·5 (678·2 to 706·3)**	**30 003·0 (29 540·0 to 30 525·8)**	**24 710·4 (24 267·8 to 25 275·7)**
High SDI			78·1 (77·8 to 78·3)	83·4 (83·2 to 83·6)	18·4 (18·2 to 18·5)	21·7 (21·5 to 21·8)	636·0 (624·5 to 648·7)	400·0 (392·3 to 407·7)	4783·7 (4694·8 to 4881·3)	4692·7 (4606·4 to 4779·7)
High-middle SDI			73·1 (72·1 to 74·1)	79·9 (78·7 to 80·8)	15·8 (15·4 to 16·3)	19·2 (18·4 to 19·9)	898·6 (842·8 to 960·5)	550·1 (503·8 to 611·2)	4720·7 (4395·0 to 5105·6)	3930·5 (3577·7 to 4385·3)
Middle SDI			71·1 (70·7 to 71·4)	77·3 (77·0 to 77·6)	15·0 (14·9 to 15·2)	18·2 (18·0 to 18·4)	1013·2 (993·5 to 1034·1)	653·6 (640·6 to 667·4)	9131·3 (8946·0 to 9326·2)	6450·8 (6317·5 to 6595·9)
Low-middle SDI			66·2 (65·6 to 66·8)	70·3 (69·7 to 70·7)	13·9 (13·8 to 14·1)	15·5 (15·3 to 15·7)	1251·8 (1219·8 to 1284·3)	989·5 (962·3 to 1018·9)	8176·3 (7946·4 to 8398·5)	6735·7 (6541·1 to 6936·5)
Low SDI			61·6 (60·7 to 62·5)	64·1 (63·3 to 64·8)	13·2 (12·9 to 13·5)	13·3 (13·0 to 13·6)	1492·6 (1437·1 to 1553·8)	1380·5 (1330·5 to 1436·1)	3168·5 (3055·4 to 3297·8)	2879·9 (2766·4 to 2994·4)
**High income**			**78·3 (78·0 to 78·5)**	**83·5 (83·3 to 83·7)**	**18·5 (18·3 to 18·6)**	**21·8 (21·7 to 21·9)**	**625·5 (614·2 to 637·6)**	**394·7 (387·4 to 402·7)**	**4841·9 (4751·9 to 4937·8)**	**4774·3 (4688·3 to 4865·5)**
High-income North America			76·8 (76·5 to 77·0)	81·5 (81·3 to 81·7)	18·2 (18·1 to 18·3)	20·7 (20·6 to 20·9)	680·7 (667·3 to 693·8)	469·0 (460·0 to 478·3)	1543·5 (1512·0 to 1574·5)	1502·9 (1474·4 to 1532·4)
	Canada		79·8 (79·3 to 80·2)	83·9 (83·5 to 84·3)	19·1 (18·8 to 19·4)	22·0 (21·7 to 22·3)	554·0 (531·8 to 576·9)	379·1 (364·1 to 394·3)	137·5 (131·7 to 143·2)	135·3 (130·0 to 140·5)
	Greenland		67·8 (64·7 to 70·6)	72·8 (70·4 to 75·6)	11·9 (10·6 to 13·3)	14·2 (12·8 to 15·9)	1422·9 (1165·6 to 1746·0)	1027·3 (815·2 to 1247·7)	0·3 (0·2 to 0·3)	0·1 (0·1 to 0·2)
	USA		76·5 (76·2 to 76·7)	81·2 (81·0 to 81·5)	18·1 (17·9 to 18·2)	20·6 (20·4 to 20·8)	696·1 (681·7 to 710·5)	479·9 (470·0 to 490·1)	1405·3 (1375·1 to 1435·7)	1367·1 (1339·6 to 1395·9)
Australasia			80·3 (79·6 to 81·0)	84·4 (83·8 to 85·0)	19·4 (18·9 to 19·8)	22·1 (21·7 to 22·6)	536·4 (503·3 to 571·2)	365·0 (342·1 to 388·2)	101·7 (95·4 to 108·5)	96·5 (90·5 to 102·5)
	Australia		80·5 (79·7 to 81·2)	84·6 (83·8 to 85·3)	19·5 (18·9 to 20·0)	22·2 (21·7 to 22·7)	529·8 (495·9 to 569·9)	358·1 (332·3 to 386·9)	85·0 (79·1 to 91·6)	80·7 (75·1 to 86·9)
	New Zealand		79·5 (78·3 to 80·8)	83·4 (82·3 to 84·6)	18·9 (18·1 to 19·8)	21·5 (20·7 to 22·4)	571·3 (508·4 to 638·1)	401·3 (353·3 to 449·6)	16·7 (14·8 to 18·8)	15·8 (13·9 to 17·6)
High-income Asia Pacific			80·1 (79·1 to 80·9)	86·4 (85·6 to 87·1)	19·2 (18·8 to 19·6)	23·8 (23·3 to 24·2)	548·3 (510·7 to 592·1)	292·6 (271·3 to 318·7)	879·8 (823·6 to 945·6)	822·0 (771·6 to 881·9)
	Brunei		74·6 (72·5 to 77·3)	79·5 (77·8 to 81·7)	15·9 (15·1 to 17·4)	18·9 (18·2 to 20·2)	849·1 (691·4 to 955·0)	574·0 (472·5 to 651·0)	1·0 (0·8 to 1·2)	0·7 (0·5 to 0·8)
	Japan		80·8 (80·6 to 81·1)	86·9 (86·7 to 87·2)	19·5 (19·4 to 19·7)	24·2 (24·1 to 24·4)	516·3 (504·8 to 529·2)	276·1 (269·4 to 282·9)	687·0 (671·5 to 704·3)	657·4 (641·6 to 672·4)
	Singapore		81·3 (78·8 to 83·7)	86·1 (83·9 to 88·4)	19·7 (17·9 to 21·5)	23·3 (21·6 to 25·2)	490·0 (390·9 to 610·3)	309·2 (237·5 to 384·8)	10·3 (8·1 to 12·9)	9·1 (6·9 to 11·4)
	South Korea		77·7 (74·3 to 81·5)	84·2 (81·2 to 87·1)	17·5 (15·5 to 20·1)	21·7 (19·4 to 23·9)	676·1 (481·2 to 887·5)	374·9 (269·4 to 509·6)	181·6 (124·3 to 244·8)	154·8 (109·1 to 213·2)
Western Europe			79·2 (78·9 to 79·5)	84·1 (83·8 to 84·4)	18·5 (18·3 to 18·7)	21·8 (21·6 to 22·0)	597·7 (582·8 to 613·9)	378·8 (368·0 to 389·5)	2071·0 (2017·7 to 2128·0)	2122·2 (2063·4 to 2181·0)
	Andorra		79·3 (77·4 to 81·9)	85·8 (83·0 to 87·7)	18·5 (17·4 to 20·1)	23·1 (20·9 to 24·5)	596·0 (471·1 to 694·4)	315·7 (252·4 to 426·5)	0·4 (0·3 to 0·4)	0·3 (0·3 to 0·5)
	Austria		79·1 (78·5 to 79·9)	83·9 (83·2 to 84·5)	18·4 (18·0 to 18·9)	21·5 (21·0 to 21·9)	605·1 (566·3 to 638·2)	392·9 (365·6 to 420·7)	39·3 (36·7 to 41·5)	43·2 (40·2 to 46·1)
	Belgium		78·4 (77·2 to 79·5)	83·4 (82·3 to 84·5)	18·0 (17·2 to 18·7)	21·4 (20·6 to 22·2)	642·4 (582·4 to 710·9)	407·8 (361·7 to 452·8)	55·8 (50·4 to 61·9)	57·2 (50·9 to 63·3)
	Cyprus		78·1 (77·5 to 78·8)	82·8 (82·3 to 83·4)	17·4 (17·0 to 17·9)	20·2 (19·7 to 20·6)	663·7 (623·8 to 702·3)	441·5 (417·2 to 467·6)	3·7 (3·5 to 3·9)	3·5 (3·3 to 3·7)
	Denmark		78·8 (77·3 to 80·1)	82·8 (81·5 to 84·2)	18·0 (17·0 to 18·9)	20·8 (19·8 to 21·8)	628·0 (556·1 to 714·8)	429·5 (372·6 to 489·1)	26·7 (23·5 to 30·5)	26·1 (22·7 to 29·7)
	Finland		78·9 (78·0 to 79·8)	84·6 (83·9 to 85·4)	18·4 (17·8 to 19·0)	22·0 (21·4 to 22·6)	615·9 (569·7 to 664·7)	363·4 (331·7 to 393·1)	25·9 (23·9 to 28·1)	25·9 (23·6 to 27·9)
	France		79·2 (78·6 to 79·8)	85·4 (84·9 to 86·0)	19·2 (18·9 to 19·6)	23·2 (22·8 to 23·7)	579·9 (549·8 to 610·6)	326·3 (305·9 to 345·3)	295·5 (280·2 to 311·0)	292·2 (274·9 to 308·1)
	Germany		78·5 (77·4 to 79·5)	83·3 (82·5 to 84·3)	17·9 (17·3 to 18·6)	21·1 (20·5 to 21·8)	641·1 (584·5 to 700·5)	413·3 (373·8 to 451·9)	449·3 (408·7 to 492·4)	469·0 (423·8 to 512·5)
	Greece		78·4 (77·5 to 79·5)	83·5 (82·8 to 84·3)	18·3 (17·7 to 19·0)	21·0 (20·4 to 21·5)	625·0 (572·1 to 677·9)	409·9 (377·8 to 443·4)	64·5 (59·2 to 70·0)	63·1 (58·4 to 68·2)
	Iceland		80·6 (79·7 to 81·4)	84·0 (82·9 to 85·0)	19·0 (18·4 to 19·6)	21·3 (20·5 to 22·1)	542·5 (501·3 to 590·4)	387·5 (346·7 to 433·1)	1·2 (1·1 to 1·3)	1·1 (1·0 to 1·2)
	Ireland		79·0 (77·5 to 80·4)	83·3 (82·0 to 84·7)	18·1 (17·1 to 19·1)	21·0 (20·0 to 22·1)	614·7 (539·7 to 702·4)	410·5 (354·7 to 471·3)	16·2 (14·1 to 18·7)	14·9 (12·8 to 17·1)
	Israel		80·0 (77·8 to 82·3)	84·1 (82·2 to 85·9)	18·9 (17·4 to 20·5)	21·5 (20·0 to 23·0)	551·1 (444·7 to 674·8)	378·6 (311·2 to 464·6)	23·3 (18·8 to 28·5)	22·7 (18·7 to 27·7)
	Italy		79·9 (79·1 to 80·7)	84·6 (84·0 to 85·3)	18·6 (18·1 to 19·1)	21·9 (21·4 to 22·5)	571·7 (532·1 to 614·2)	361·4 (335·8 to 387·7)	318·7 (296·4 to 343·1)	348·6 (323·8 to 373·5)
	Luxembourg		80·3 (79·1 to 81·4)	83·9 (82·8 to 85·3)	18·9 (18·2 to 19·7)	21·5 (20·6 to 22·5)	552·8 (496·1 to 614·2)	390·0 (336·3 to 438·1)	2·0 (1·8 to 2·2)	2·1 (1·8 to 2·4)
	Malta		79·0 (77·1 to 81·0)	83·8 (82·1 to 85·8)	18·0 (16·8 to 19·4)	21·3 (19·9 to 22·9)	614·1 (511·6 to 726·2)	389·6 (313·9 to 468·9)	1·9 (1·5 to 2·2)	1·6 (1·3 to 1·9)
	Netherlands		79·6 (78·6 to 80·6)	83·6 (82·6 to 84·5)	18·1 (17·4 to 18·8)	21·4 (20·7 to 22·1)	597·5 (545·8 to 660·4)	399·5 (363·1 to 442·0)	74·0 (67·4 to 82·2)	73·9 (67·3 to 81·7)
	Norway		80·1 (79·0 to 81·3)	84·1 (82·9 to 85·3)	18·7 (17·9 to 19·5)	21·6 (20·7 to 22·5)	567·5 (506·0 to 627·3)	381·8 (333·3 to 432·7)	20·6 (18·3 to 22·9)	21·3 (18·7 to 24·0)
	Portugal		77·8 (76·9 to 78·7)	84·0 (83·4 to 84·8)	17·8 (17·3 to 18·4)	21·5 (21·0 to 22·1)	672·0 (623·0 to 719·0)	384·6 (355·1 to 413·3)	56·5 (52·3 to 60·5)	55·3 (51·2 to 59·5)
	Spain		80·3 (79·7 to 80·8)	85·6 (85·1 to 86·0)	19·2 (18·8 to 19·6)	22·8 (22·5 to 23·2)	544·1 (516·7 to 574·8)	323·7 (307·2 to 340·5)	208·9 (198·0 to 220·4)	209·6 (199·4 to 220·3)
	Sweden		80·1 (78·8 to 81·4)	84·0 (82·6 to 85·2)	18·6 (17·7 to 19·5)	21·4 (20·4 to 22·3)	569·7 (505·4 to 642·7)	389·3 (340·9 to 447·7)	46·6 (41·1 to 52·6)	47·5 (41·7 to 54·4)
	Switzerland		81·0 (78·1 to 83·6)	85·2 (82·6 to 87·6)	19·5 (17·5 to 21·3)	22·5 (20·5 to 24·4)	517·0 (399·3 to 680·6)	337·3 (254·5 to 446·5)	32·7 (25·0 to 43·4)	34·3 (26·1 to 45·4)
	UK		78·9 (78·7 to 79·1)	82·9 (82·6 to 83·1)	18·3 (18·1 to 18·4)	20·9 (20·7 to 21·0)	611·5 (600·8 to 622·5)	426·7 (418·3 to 435·9)	305·5 (300·0 to 311·3)	306·9 (301·1 to 312·8)
		England	79·2 (79·1 to 79·4)	83·1 (83·0 to 83·3)	18·4 (18·3 to 18·5)	21·1 (20·9 to 21·2)	596·1 (588·2 to 603·9)	416·7 (410·5 to 423·0)	251·8 (248·4 to 255·1)	253·6 (249·9 to 257·4)
		Northern Ireland	77·9 (76·1 to 79·7)	82·4 (80·8 to 84·0)	17·8 (16·7 to 18·9)	20·6 (19·5 to 21·8)	666·9 (569·8 to 773·1)	446·5 (381·2 to 520·4)	8·1 (6·9 to 9·4)	8·0 (6·8 to 9·3)
		Scotland	76·9 (75·4 to 78·4)	81·2 (79·7 to 82·6)	17·2 (16·2 to 18·1)	19·7 (18·7 to 20·7)	718·4 (634·8 to 812·3)	500·6 (435·9 to 575·0)	28·7 (25·3 to 32·6)	29·0 (25·3 to 33·3)
		Wales	77·9 (76·4 to 79·4)	82·2 (80·8 to 83·5)	17·7 (16·7 to 18·7)	20·4 (19·4 to 21·4)	663·0 (582·9 to 754·8)	454·8 (399·2 to 520·3)	16·8 (14·7 to 19·2)	16·2 (14·3 to 18·5)
Southern Latin America			74·4 (73·3 to 75·4)	80·9 (79·9 to 81·8)	16·1 (15·5 to 16·8)	20·2 (19·5 to 20·9)	833·1 (768·7 to 905·7)	494·4 (452·7 to 540·9)	245·9 (225·7 to 268·3)	230·8 (211·0 to 252·4)
	Argentina		73·3 (72·3 to 74·4)	80·0 (79·1 to 80·9)	15·6 (14·9 to 16·2)	19·7 (19·1 to 20·4)	907·0 (836·0 to 980·8)	534·1 (490·0 to 578·4)	171·6 (157·4 to 186·1)	163·4 (149·9 to 177·2)
	Chile		77·3 (74·0 to 80·5)	83·2 (80·2 to 86·1)	17·9 (15·9 to 20·0)	21·6 (19·4 to 23·9)	659·5 (504·1 to 852·3)	402·3 (298·2 to 533·7)	57·4 (43·2 to 75·2)	51·0 (37·6 to 67·9)
	Uruguay		73·4 (72·6 to 74·4)	81·1 (80·2 to 81·9)	15·5 (15·0 to 16·1)	20·4 (19·9 to 21·0)	898·7 (839·2 to 960·6)	485·6 (449·7 to 524·0)	16·8 (15·7 to 18·0)	16·4 (15·2 to 17·6)
**Central Europe, eastern Europe, and central Asia**			**68·2 (66·1 to 70·2)**	**77·2 (75·0 to 78·9)**	**14·1 (13·2 to 15·0)**	**17·8 (16·5 to 18·9)**	**1201·9 (1059·5 to 1362·6)**	**681·6 (588·3 to 807·2)**	**2462·2 (2142·2 to 2822·5)**	**2381·1 (2038·8 to 2834·4)**
Eastern Europe			66·1 (62·6 to 69·7)	76·6 (73·2 to 79·7)	13·3 (11·9 to 14·9)	17·4 (15·5 to 19·3)	1364·8 (1084·6 to 1675·8)	723·5 (556·7 to 940·8)	1449·3 (1131·2 to 1808·2)	1459·6 (1116·6 to 1898·0)
	Belarus		68·2 (65·6 to 70·8)	78·8 (76·6 to 81·0)	13·3 (12·0 to 14·6)	18·1 (16·7 to 19·7)	1273·2 (1063·7 to 1509·9)	625·1 (507·3 to 756·7)	60·5 (49·9 to 72·5)	60·6 (49·0 to 73·2)
	Estonia		73·0 (71·3 to 74·7)	81·8 (80·7 to 83·5)	15·7 (14·9 to 16·6)	20·1 (19·4 to 21·4)	925·3 (814·4 to 1032·8)	477·0 (402·7 to 527·4)	7·4 (6·5 to 8·3)	8·5 (7·2 to 9·3)
	Latvia		70·0 (68·1 to 72·0)	79·8 (78·2 to 81·3)	14·2 (13·3 to 15·3)	19·0 (18·0 to 20·1)	1126·2 (976·5 to 1273·1)	567·4 (491·6 to 651·3)	13·4 (11·5 to 15·2)	15·0 (13·0 to 17·2)
	Lithuania		69·7 (68·6 to 70·9)	80·3 (79·4 to 81·3)	14·7 (14·1 to 15·3)	19·6 (19·0 to 20·3)	1104·7 (1019·2 to 1193·1)	534·7 (489·3 to 580·5)	19·7 (18·1 to 21·3)	20·3 (18·5 to 22·0)
	Moldova		68·3 (66·6 to 70·3)	76·1 (74·6 to 77·8)	13·3 (12·4 to 14·4)	16·3 (15·3 to 17·5)	1229·7 (1070·0 to 1384·9)	756·3 (658·3 to 857·4)	22·6 (19·4 to 25·9)	20·5 (17·7 to 23·4)
	Russia		65·4 (60·8 to 70·7)	76·2 (71·4 to 80·7)	13·2 (11·3 to 15·7)	17·3 (14·5 to 20·1)	1402·2 (1002·0 to 1838·5)	741·2 (507·5 to 1072·7)	1000·4 (695·7 to 1336·4)	996·7 (680·9 to 1443·9)
	Ukraine		67·0 (63·3 to 71·4)	77·0 (73·5 to 80·5)	13·3 (11·6 to 15·5)	17·2 (15·2 to 19·5)	1325·9 (989·2 to 1671·2)	718·1 (531·3 to 950·3)	325·4 (237·3 to 415·8)	338·0 (247·4 to 446·1)
Central Europe			73·5 (72·9 to 74·0)	80·3 (79·8 to 80·7)	15·4 (15·1 to 15·7)	18·9 (18·6 to 19·2)	916·7 (879·6 to 954·2)	550·6 (526·8 to 574·6)	672·3 (643·8 to 701·3)	646·5 (619·0 to 676·2)
	Albania		74·7 (73·1 to 76·4)	80·7 (79·4 to 82·1)	16·1 (14·9 to 17·3)	19·6 (18·6 to 20·6)	825·5 (720·1 to 944·9)	511·5 (447·2 to 581·0)	12·8 (11·0 to 14·8)	9·6 (8·3 to 11·0)
	Bosnia and Herzegovina		74·9 (73·0 to 76·9)	80·2 (78·4 to 82·1)	15·6 (14·4 to 16·8)	18·6 (17·2 to 19·9)	832·2 (712·3 to 966·2)	548·3 (460·6 to 653·2)	20·3 (17·1 to 23·8)	18·4 (15·3 to 22·1)
	Bulgaria		71·7 (69·6 to 73·9)	78·5 (76·5 to 80·4)	14·3 (13·1 to 15·6)	17·6 (16·2 to 18·9)	1062·6 (897·6 to 1240·4)	658·2 (550·9 to 789·4)	56·3 (47·1 to 66·0)	53·4 (44·5 to 64·4)
	Croatia		74·2 (72·6 to 76·0)	80·5 (79·0 to 82·1)	15·1 (14·2 to 16·3)	18·6 (17·5 to 19·8)	901·5 (778·7 to 1021·2)	552·0 (470·0 to 636·6)	26·9 (23·1 to 30·6)	28·8 (24·4 to 33·1)
	Czech Republic		76·2 (75·5 to 76·9)	81·9 (81·3 to 82·5)	16·3 (15·9 to 16·8)	19·7 (19·2 to 20·1)	779·9 (734·9 to 828·5)	488·7 (459·1 to 519·2)	54·1 (50·8 to 57·7)	54·2 (50·8 to 57·6)
	Hungary		72·2 (70·7 to 73·9)	79·1 (77·7 to 80·3)	14·7 (13·9 to 15·7)	18·3 (17·5 to 19·2)	1009·2 (892·5 to 1131·6)	610·8 (544·5 to 687·4)	63·4 (55·9 to 71·3)	67·4 (60·1 to 75·5)
	Macedonia		72·4 (71·5 to 73·3)	77·4 (76·6 to 78·1)	13·7 (13·2 to 14·1)	16·3 (15·8 to 16·8)	1079·2 (1006·8 to 1149·0)	738·2 (687·6 to 786·6)	11·6 (10·8 to 12·4)	10·2 (9·5 to 10·9)
	Montenegro		74·4 (73·1 to 75·5)	79·7 (78·7 to 81·2)	15·7 (14·9 to 16·6)	18·5 (17·6 to 19·7)	872·7 (790·5 to 966·4)	580·0 (502·5 to 641·3)	3·2 (2·8 to 3·5)	2·8 (2·4 to 3·1)
	Poland		74·1 (72·8 to 75·4)	81·7 (80·6 to 82·8)	16·0 (15·3 to 16·8)	20·1 (19·3 to 20·9)	862·6 (780·8 to 947·1)	479·8 (431·2 to 532·2)	199·9 (180·2 to 220·3)	189·9 (170·7 to 211·0)
	Romania		71·7 (70·2 to 73·1)	78·9 (77·7 to 80·1)	14·8 (13·9 to 15·6)	18·1 (17·3 to 18·9)	1010·3 (905·9 to 1128·2)	614·7 (550·7 to 682·6)	131·6 (117·4 to 147·5)	122·8 (109·7 to 136·5)
	Serbia		73·0 (72·2 to 73·9)	78·8 (78·1 to 79·4)	15·0 (14·6 to 15·4)	17·9 (17·5 to 18·2)	958·1 (897·0 to 1014·5)	628·0 (596·9 to 663·9)	54·7 (51·0 to 57·9)	52·1 (49·6 to 55·0)
	Slovakia		73·4 (71·9 to 75·0)	80·4 (79·1 to 81·8)	15·1 (14·2 to 16·0)	18·8 (17·8 to 19·8)	939·4 (830·2 to 1059·0)	556·2 (484·8 to 631·5)	27·5 (24·1 to 31·3)	26·5 (23·0 to 30·2)
	Slovenia		77·8 (76·2 to 79·5)	83·8 (82·5 to 85·3)	17·5 (16·5 to 18·6)	21·3 (20·3 to 22·4)	676·9 (584·5 to 777·0)	396·1 (337·5 to 454·0)	10·1 (8·6 to 11·6)	10·6 (9·0 to 12·1)
Central Asia			67·5 (66·4 to 68·7)	75·1 (74·1 to 76·0)	13·5 (13·1 to 14·0)	16·8 (16·3 to 17·3)	1252·9 (1168·5 to 1333·4)	776·6 (728·3 to 831·2)	340·6 (316·5 to 365·5)	275·0 (256·6 to 295·3)
	Armenia		72·0 (70·5 to 73·5)	79·3 (78·0 to 80·7)	14·8 (14·0 to 15·6)	18·3 (17·4 to 19·3)	985·9 (883·3 to 1093·3)	584·4 (514·3 to 655·0)	13·9 (12·4 to 15·4)	13·3 (11·7 to 15·0)
	Azerbaijan		68·4 (65·7 to 71·2)	75·8 (73·5 to 77·9)	13·8 (12·5 to 15·4)	17·3 (15·7 to 18·6)	1195·1 (982·0 to 1413·0)	731·2 (617·5 to 885·6)	40·5 (32·6 to 48·9)	29·5 (24·4 to 36·0)
	Georgia		69·1 (66·2 to 72·2)	78·8 (76·6 to 81·4)	13·8 (12·6 to 15·1)	18·5 (17·1 to 20·3)	1187·5 (973·1 to 1417·6)	600·5 (473·3 to 726·3)	25·7 (20·9 to 30·9)	22·2 (17·4 to 26·6)
	Kazakhstan		67·1 (64·1 to 70·0)	76·2 (73·9 to 78·5)	13·3 (11·9 to 14·7)	16·9 (15·5 to 18·4)	1289·6 (1064·5 to 1559·2)	742·2 (613·0 to 896·3)	73·9 (59·8 to 90·2)	60·6 (49·2 to 73·9)
	Kyrgyzstan		67·5 (66·1 to 69·0)	74·9 (73·8 to 76·1)	13·5 (12·9 to 14·3)	16·6 (15·8 to 17·4)	1253·9 (1138·6 to 1371·7)	790·0 (720·2 to 869·4)	20·2 (18·0 to 22·2)	15·8 (14·4 to 17·5)
	Mongolia		63·6 (61·4 to 65·8)	73·0 (71·1 to 75·1)	12·3 (11·5 to 13·2)	15·6 (14·6 to 16·7)	1562·3 (1365·7 to 1771·6)	920·4 (789·0 to 1061·2)	13·1 (11·1 to 15·1)	8·3 (7·1 to 9·8)
	Tajikistan		69·4 (67·1 to 71·5)	74·3 (72·4 to 76·1)	14·9 (13·6 to 15·8)	16·9 (15·7 to 17·9)	1067·0 (938·1 to 1244·9)	789·8 (698·1 to 915·3)	23·9 (20·4 to 28·2)	17·4 (14·8 to 20·5)
	Turkmenistan		66·5 (65·1 to 67·8)	74·0 (72·7 to 75·1)	13·7 (13·3 to 14·2)	16·9 (16·3 to 17·5)	1251·7 (1179·3 to 1327·8)	790·0 (737·8 to 850·2)	18·6 (17·3 to 20·4)	14·2 (13·1 to 15·4)
	Uzbekistan		67·1 (65·3 to 68·8)	73·5 (71·9 to 75·1)	13·1 (12·4 to 13·8)	15·8 (14·9 to 16·7)	1309·6 (1186·8 to 1454·7)	889·1 (786·6 to 999·8)	110·7 (97·6 to 125·8)	93·6 (81·9 to 106·4)
**Latin America and Caribbean**			**72·8 (72·2 to 73·2)**	**78·9 (78·4 to 79·3)**	**16·8 (16·6 to 16·9)**	**19·5 (19·3 to 19·7)**	**860·7 (840·3 to 883·0)**	**565·3 (550·1 to 581·0)**	**1804·6 (1762·2 to 1851·2)**	**1425·2 (1391·4 to 1463·0)**
Central Latin America			73·6 (72·9 to 74·2)	79·4 (79·0 to 79·9)	17·3 (17·0 to 17·6)	19·8 (19·5 to 20·1)	806·4 (775·0 to 840·6)	541·4 (520·7 to 562·3)	715·7 (687·6 to 747·9)	556·2 (535·0 to 578·3)
	Colombia		75·4 (74·2 to 76·7)	81·0 (80·0 to 82·1)	17·6 (16·9 to 18·3)	20·2 (19·5 to 21·0)	732·3 (664·9 to 804·2)	483·6 (437·5 to 532·3)	122·3 (109·8 to 135·1)	97·6 (87·8 to 108·3)
	Costa Rica		78·5 (77·4 to 79·6)	83·6 (82·6 to 84·5)	19·5 (18·9 to 20·2)	22·3 (21·6 to 22·9)	584·4 (535·8 to 634·1)	382·3 (349·1 to 416·7)	12·1 (11·0 to 13·2)	9·2 (8·3 to 10·0)
	El Salvador		71·5 (69·3 to 73·4)	79·1 (77·6 to 80·5)	18·0 (17·1 to 18·9)	19·6 (18·6 to 20·5)	880·9 (774·9 to 995·9)	568·9 (502·0 to 642·3)	20·9 (18·3 to 23·8)	17·1 (15·0 to 19·4)
	Guatemala		69·4 (65·4 to 73·9)	76·0 (72·4 to 79·8)	16·9 (15·1 to 19·0)	18·8 (16·6 to 21·2)	993·8 (744·0 to 1256·7)	678·2 (504·9 to 888·6)	48·3 (36·6 to 61·1)	36·2 (27·0 to 47·1)
	Honduras		71·6 (67·3 to 75·8)	73·7 (70·4 to 77·7)	15·7 (13·5 to 17·9)	16·0 (14·3 to 18·6)	952·3 (711·8 to 1265·1)	870·1 (630·2 to 1102·8)	22·9 (16·8 to 30·4)	22·2 (16·1 to 28·3)
	Mexico		73·7 (73·2 to 74·2)	79·1 (78·7 to 79·6)	17·2 (17·0 to 17·4)	19·5 (19·2 to 19·7)	805·8 (782·4 to 830·0)	557·7 (539·7 to 575·9)	367·7 (357·5 to 378·5)	292·0 (282·3 to 301·0)
	Nicaragua		75·2 (72·9 to 77·7)	81·2 (79·2 to 83·2)	18·4 (17·1 to 19·7)	21·0 (19·6 to 22·4)	729·2 (606·4 to 861·8)	472·6 (390·4 to 562·4)	13·4 (11·2 to 16·0)	10·5 (8·7 to 12·5)
	Panama		76·0 (74·0 to 78·1)	82·0 (80·4 to 83·6)	18·8 (17·6 to 19·9)	21·7 (20·6 to 22·8)	673·7 (576·6 to 781·9)	431·6 (372·7 to 497·1)	10·8 (9·1 to 12·6)	7·7 (6·6 to 9·0)
	Venezuela		71·3 (68·2 to 73·8)	79·8 (77·5 to 81·8)	16·7 (15·3 to 18·0)	20·2 (18·7 to 21·6)	916·2 (775·5 to 1109·1)	524·7 (438·5 to 632·9)	97·3 (81·6 to 119·7)	63·7 (52·5 to 77·3)
Andean Latin America			76·0 (74·6 to 77·5)	79·8 (78·3 to 81·2)	18·4 (17·5 to 19·2)	20·1 (19·2 to 21·1)	688·6 (614·5 to 764·2)	525·1 (465·7 to 593·6)	144·5 (128·9 to 161·4)	128·1 (113·8 to 144·5)
	Bolivia		72·2 (69·7 to 75·0)	74·3 (71·2 to 77·0)	16·6 (15·5 to 18·0)	16·9 (15·0 to 18·6)	878·0 (728·1 to 1026·2)	797·9 (649·2 to 1004·9)	34·2 (28·4 to 40·1)	34·2 (28·3 to 42·8)
	Ecuador		75·4 (74·1 to 76·5)	80·4 (79·4 to 81·3)	18·7 (18·0 to 19·3)	20·8 (20·2 to 21·4)	701·8 (647·0 to 764·6)	495·1 (456·2 to 537·3)	42·1 (38·6 to 46·0)	33·2 (30·6 to 36·1)
	Peru		77·8 (75·3 to 80·3)	81·6 (79·2 to 84·0)	18·9 (17·4 to 20·5)	21·1 (19·4 to 22·8)	618·6 (501·9 to 750·9)	453·7 (361·5 to 561·6)	68·2 (54·6 to 83·3)	60·7 (48·2 to 75·3)
Caribbean			71·0 (69·7 to 72·3)	75·4 (74·0 to 76·6)	16·5 (16·1 to 16·9)	18·6 (18·0 to 19·2)	922·0 (865·1 to 984·6)	692·7 (640·1 to 746·5)	185·7 (174·7 to 198·7)	165·2 (152·6 to 178·4)
	Antigua and Barbuda		74·7 (72·7 to 76·4)	79·9 (78·3 to 81·6)	16·8 (15·9 to 17·8)	19·7 (18·7 to 20·9)	794·4 (699·6 to 905·5)	538·1 (463·8 to 615·1)	0·3 (0·2 to 0·3)	0·2 (0·2 to 0·3)
	The Bahamas		71·3 (69·7 to 72·9)	76·3 (74·6 to 78·0)	15·7 (15·1 to 16·5)	17·6 (16·6 to 18·8)	978·9 (880·4 to 1079·3)	718·7 (625·8 to 817·6)	1·5 (1·3 to 1·7)	1·4 (1·2 to 1·6)
	Barbados		74·4 (72·8 to 75·9)	78·7 (77·3 to 80·1)	16·7 (16·0 to 17·5)	19·4 (18·6 to 20·2)	815·4 (736·4 to 903·0)	584·9 (523·1 to 650·0)	1·3 (1·2 to 1·5)	1·4 (1·2 to 1·5)
	Belize		69·1 (67·1 to 71·4)	74·9 (73·1 to 76·8)	14·1 (13·1 to 15·2)	16·6 (15·6 to 17·7)	1161·9 (1006·8 to 1318·0)	807·7 (696·4 to 923·9)	1·1 (0·9 to 1·3)	0·7 (0·6 to 0·9)
	Bermuda		75·7 (74·1 to 77·2)	82·4 (80·7 to 84·5)	16·3 (15·3 to 17·2)	20·4 (19·1 to 22·1)	802·1 (708·4 to 914·1)	451·9 (362·1 to 536·1)	0·2 (0·2 to 0·3)	0·2 (0·1 to 0·2)
	Cuba		76·7 (75·6 to 77·7)	81·3 (80·3 to 82·4)	17·3 (16·6 to 18·0)	20·2 (19·5 to 21·0)	712·3 (652·5 to 775·6)	483·4 (438·5 to 528·2)	52·8 (48·2 to 57·7)	43·6 (39·5 to 47·8)
	Dominica		70·2 (67·9 to 72·4)	75·8 (73·9 to 77·4)	14·6 (13·7 to 15·8)	17·3 (16·0 to 18·2)	1075·3 (929·4 to 1231·2)	739·5 (654·2 to 862·5)	0·3 (0·3 to 0·4)	0·3 (0·2 to 0·3)
	Dominican Republic		72·9 (71·2 to 74·9)	78·6 (76·9 to 80·6)	17·2 (16·3 to 18·5)	19·6 (18·5 to 21·2)	838·5 (726·1 to 933·4)	569·3 (476·4 to 651·4)	32·7 (28·1 to 36·5)	24·2 (20·0 to 27·9)
	Grenada		68·8 (66·7 to 71·1)	74·4 (72·3 to 76·7)	14·0 (13·1 to 14·9)	16·3 (15·2 to 17·6)	1181·4 (1030·2 to 1330·2)	839·0 (709·7 to 978·5)	0·4 (0·4 to 0·5)	0·4 (0·4 to 0·5)
	Guyana		64·7 (63·0 to 66·4)	71·2 (69·6 to 72·9)	13·0 (12·3 to 13·7)	15·2 (14·3 to 16·2)	1416·0 (1279·2 to 1560·8)	996·3 (880·4 to 1110·2)	3·5 (3·1 to 3·9)	2·4 (2·1 to 2·8)
	Haiti		63·6 (60·2 to 66·9)	64·4 (61·4 to 67·5)	13·8 (12·4 to 15·2)	13·0 (11·7 to 14·5)	1377·6 (1149·7 to 1647·1)	1414·9 (1163·8 to 1691·5)	45·7 (37·1 to 55·8)	50·1 (40·6 to 60·3)
	Jamaica		73·1 (70·4 to 75·5)	76·8 (74·5 to 79·1)	16·3 (14·8 to 17·6)	17·9 (16·5 to 19·4)	874·8 (735·5 to 1051·7)	682·4 (567·9 to 816·1)	11·5 (9·7 to 13·9)	10·5 (8·8 to 12·6)
	Puerto Rico		75·0 (73·8 to 76·4)	82·4 (81·3 to 83·5)	17·6 (16·9 to 18·3)	21·1 (20·3 to 22·0)	754·1 (684·1 to 825·9)	435·5 (389·5 to 481·5)	16·1 (14·6 to 17·6)	14·0 (12·5 to 15·5)
	Saint Lucia		73·0 (71·6 to 74·3)	79·3 (78·1 to 80·4)	17·2 (16·6 to 17·7)	20·0 (19·5 to 20·6)	832·3 (776·2 to 894·1)	543·3 (502·0 to 583·3)	0·7 (0·6 to 0·7)	0·5 (0·5 to 0·6)
	Saint Vincent and the Grenadines		68·8 (67·4 to 70·0)	74·8 (73·7 to 76·1)	14·0 (13·1 to 14·6)	16·4 (15·7 to 17·3)	1173·3 (1091·4 to 1292·6)	820·5 (732·4 to 889·7)	0·5 (0·5 to 0·6)	0·4 (0·4 to 0·4)
	Suriname		68·4 (67·1 to 69·7)	74·4 (73·1 to 75·4)	14·5 (13·8 to 15·3)	17·1 (16·4 to 17·7)	1139·5 (1044·9 to 1220·6)	784·6 (731·7 to 852·7)	2·2 (2·0 to 2·4)	1·8 (1·7 to 2·0)
	Trinidad and Tobago		69·3 (67·8 to 70·8)	77·0 (75·6 to 78·2)	14·4 (13·8 to 15·0)	18·7 (18·1 to 19·4)	1144·3 (1046·7 to 1238·5)	650·1 (596·7 to 709·8)	6·3 (5·7 to 6·9)	4·7 (4·2 to 5·1)
	Virgin Islands		70·6 (68·8 to 72·9)	78·8 (77·5 to 80·1)	14·6 (13·8 to 15·7)	18·5 (17·9 to 19·1)	1070·2 (920·2 to 1208·9)	608·7 (551·2 to 669·4)	0·7 (0·6 to 0·8)	0·6 (0·5 to 0·6)
Tropical Latin America			71·6 (71·0 to 72·1)	78·9 (78·4 to 79·4)	16·0 (15·7 to 16·2)	19·3 (19·0 to 19·6)	954·6 (925·2 to 986·6)	573·1 (552·7 to 593·7)	758·7 (734·2 to 785·7)	575·7 (555·4 to 597·8)
	Brazil		71·6 (70·9 to 72·1)	79·0 (78·5 to 79·5)	16·0 (15·8 to 16·2)	19·3 (19·1 to 19·6)	955·7 (925·5 to 988·1)	570·1 (550·1 to 591·3)	738·3 (713·8 to 764·4)	560·1 (539·9 to 581·7)
	Paraguay		72·1 (70·3 to 73·5)	77·1 (75·7 to 78·5)	15·7 (14·7 to 16·5)	17·9 (17·0 to 18·9)	937·7 (849·7 to 1066·4)	674·0 (598·0 to 753·4)	20·4 (18·3 to 23·4)	15·6 (13·8 to 17·5)
**Southeast Asia, east Asia, and Oceania**			**72·1 (71·8 to 72·4)**	**78·4 (78·1 to 78·6)**	**15·1 (14·9 to 15·2)**	**18·5 (18·3 to 18·6)**	**970·4 (951·0 to 991·6)**	**612·7 (600·1 to 626·2)**	**8441·6 (8263·4 to 8635·6)**	**5832·2 (5695·8 to 5971·6)**
East Asia			73·3 (73·0 to 73·6)	79·8 (79·5 to 80·1)	15·2 (15·0 to 15·3)	19·0 (18·8 to 19·2)	928·4 (906·9 to 949·7)	556·2 (542·1 to 571·3)	6139·1 (5986·3 to 6297·2)	3934·5 (3825·0 to 4051·8)
	China		73·4 (73·0 to 73·7)	79·9 (79·6 to 80·2)	15·1 (15·0 to 15·3)	19·0 (18·8 to 19·2)	929·9 (908·6 to 951·5)	552·4 (537·5 to 567·3)	5924·9 (5774·8 to 6081·4)	3741·3 (3632·0 to 3853·4)
	North Korea		67·9 (66·7 to 69·1)	73·6 (72·5 to 74·7)	13·5 (13·1 to 14·0)	15·9 (15·4 to 16·5)	1240·1 (1160·0 to 1318·5)	873·0 (807·1 to 943·7)	110·9 (101·7 to 120·5)	122·1 (111·9 to 132·6)
	Taiwan (province of China)		76·7 (75·0 to 78·5)	82·8 (81·4 to 84·4)	17·9 (16·9 to 19·0)	21·0 (20·0 to 22·2)	693·8 (598·3 to 790·5)	423·8 (361·5 to 484·8)	103·3 (88·8 to 118·1)	71·1 (60·2 to 81·8)
Southeast Asia			70·0 (69·3 to 70·6)	75·5 (75·0 to 76·0)	14·8 (14·5 to 15·2)	17·1 (16·9 to 17·4)	1058·4 (1014·9 to 1101·4)	751·2 (726·0 to 778·0)	2249·7 (2154·6 to 2347·7)	1849·3 (1782·2 to 1917·1)
	Cambodia		65·7 (64·5 to 67·0)	71·6 (70·5 to 72·7)	13·1 (12·6 to 13·7)	15·1 (14·6 to 15·6)	1364·3 (1257·7 to 1463·4)	991·9 (923·4 to 1063·1)	53·1 (48·3 to 57·6)	45·4 (41·6 to 49·2)
	Indonesia		69·8 (68·8 to 70·7)	73·6 (73·0 to 74·1)	14·6 (14·0 to 15·3)	15·8 (15·5 to 16·1)	1081·5 (1007·7 to 1154·2)	884·1 (848·4 to 921·9)	820·1 (768·4 to 872·2)	717·7 (687·2 to 751·4)
	Laos		64·8 (62·9 to 66·4)	69·7 (68·0 to 71·1)	13·7 (13·3 to 14·2)	15·2 (14·6 to 15·9)	1313·9 (1237·5 to 1397·8)	1031·5 (954·1 to 1107·1)	26·5 (22·1 to 32·8)	22·0 (18·4 to 26·8)
	Malaysia		73·2 (72·4 to 73·9)	78·0 (77·5 to 78·6)	15·3 (14·8 to 15·8)	16·9 (16·5 to 17·2)	927·3 (871·1 to 980·1)	699·6 (667·3 to 735·2)	91·0 (85·3 to 96·7)	60·0 (56·8 to 63·4)
	Maldives		77·6 (75·0 to 80·3)	81·3 (78·6 to 83·9)	17·2 (15·5 to 19·2)	19·4 (17·4 to 21·5)	673·6 (530·3 to 837·6)	493·1 (380·2 to 636·1)	0·7 (0·5 to 0·9)	0·4 (0·3 to 0·6)
	Mauritius		71·4 (69·6 to 73·4)	77·8 (76·0 to 79·6)	14·8 (13·9 to 15·9)	18·0 (16·8 to 19·1)	1021·5 (888·9 to 1157·6)	650·9 (560·1 to 757·1)	5·6 (4·8 to 6·4)	4·6 (3·9 to 5·4)
	Myanmar		66·7 (65·2 to 67·9)	73·4 (72·4 to 74·3)	13·4 (12·8 to 13·9)	15·8 (15·4 to 16·3)	1299·3 (1224·0 to 1405·3)	888·9 (832·5 to 943·4)	210·4 (195·2 to 235·2)	163·6 (151·6 to 177·3)
	Philippines		66·6 (64·6 to 68·7)	73·9 (72·1 to 75·8)	12·6 (11·6 to 13·6)	16·0 (14·9 to 17·1)	1454·1 (1264·7 to 1663·7)	898·2 (771·1 to 1034·3)	367·0 (315·4 to 427·4)	259·9 (220·0 to 302·5)
	Sri Lanka		73·7 (70·5 to 76·9)	81·1 (78·7 to 83·8)	16·2 (14·4 to 18·1)	19·7 (17·9 to 21·7)	842·8 (667·3 to 1056·6)	493·0 (381·0 to 614·2)	72·3 (56·3 to 91·9)	50·4 (38·1 to 63·4)
	Seychelles		70·3 (68·2 to 72·0)	77·4 (76·0 to 78·9)	14·4 (13·6 to 15·4)	17·7 (17·0 to 18·5)	1096·0 (972·0 to 1248·8)	675·9 (604·1 to 751·6)	0·4 (0·4 to 0·5)	0·3 (0·3 to 0·4)
	Thailand		74·6 (72·9 to 76·2)	80·9 (79·6 to 82·0)	18·3 (17·4 to 19·2)	20·3 (19·5 to 21·1)	748·4 (668·8 to 836·6)	492·8 (446·1 to 550·1)	255·9 (226·6 to 288·7)	196·5 (176·3 to 221·1)
	Timor-Leste		71·7 (68·7 to 74·7)	73·7 (70·6 to 76·6)	16·1 (14·6 to 17·7)	16·5 (14·8 to 18·1)	917·5 (750·8 to 1111·3)	835·3 (679·8 to 1035·6)	2·9 (2·3 to 3·6)	2·6 (2·1 to 3·4)
	Vietnam		70·9 (69·1 to 72·7)	78·1 (76·6 to 79·1)	14·5 (13·9 to 15·1)	18·1 (17·3 to 18·5)	1062·9 (956·8 to 1172·7)	639·9 (596·5 to 715·8)	339·5 (298·0 to 382·9)	321·9 (301·2 to 359·0)
Oceania			60·7 (58·2 to 62·9)	63·8 (61·4 to 66·0)	11·5 (10·9 to 12·1)	12·0 (11·2 to 12·8)	1759·4 (1600·0 to 1930·0)	1556·8 (1392·4 to 1741·6)	52·7 (46·2 to 59·6)	48·4 (42·2 to 55·1)
	American Samoa		70·4 (67·8 to 72·7)	74·4 (72·0 to 77·0)	14·4 (13·3 to 15·5)	16·0 (14·6 to 17·5)	1099·4 (935·8 to 1286·0)	861·2 (702·7 to 1037·1)	0·2 (0·1 to 0·2)	0·2 (0·1 to 0·2)
	Federated States of Micronesia		63·6 (60·7 to 66·3)	67·6 (64·5 to 70·5)	12·0 (11·0 to 13·0)	12·7 (11·4 to 14·2)	1596·6 (1373·7 to 1884·1)	1357·8 (1108·9 to 1645·0)	0·4 (0·4 to 0·5)	0·4 (0·3 to 0·5)
	Fiji		63·3 (59·2 to 68·0)	67·7 (63·5 to 71·9)	11·7 (10·2 to 13·6)	13·5 (11·5 to 15·7)	1622·3 (1214·3 to 2035·4)	1255·9 (945·3 to 1628·2)	4·6 (3·3 to 6·0)	3·8 (2·7 to 5·1)
	Guam		69·0 (66·9 to 71·3)	76·1 (74·3 to 78·1)	13·8 (13·0 to 14·8)	17·1 (16·2 to 18·1)	1182·1 (1025·2 to 1349·6)	745·4 (639·1 to 851·7)	0·8 (0·7 to 1·0)	0·6 (0·5 to 0·7)
	Kiribati		58·2 (55·4 to 61·1)	65·6 (62·4 to 68·3)	10·6 (9·9 to 11·3)	12·8 (11·8 to 13·9)	2023·5 (1795·5 to 2252·2)	1391·5 (1196·0 to 1605·3)	0·6 (0·5 to 0·7)	0·5 (0·4 to 0·5)
	Marshall Islands		62·7 (60·1 to 65·4)	67·3 (64·4 to 70·1)	11·8 (10·9 to 12·8)	12·7 (11·4 to 14·1)	1656·8 (1436·7 to 1900·2)	1367·0 (1128·6 to 1650·9)	0·3 (0·2 to 0·3)	0·2 (0·2 to 0·3)
	Northern Mariana Islands		73·9 (71·5 to 76·2)	77·4 (74·9 to 79·9)	15·8 (14·8 to 16·8)	17·4 (15·9 to 19·0)	886·7 (759·7 to 1037·1)	700·0 (560·9 to 858·0)	0·2 (0·1 to 0·2)	0·1 (0·1 to 0·1)
	Papua New Guinea		59·5 (56·5 to 62·3)	62·2 (59·4 to 64·9)	11·1 (10·3 to 12·0)	11·3 (10·3 to 12·2)	1870·3 (1629·5 to 2103·2)	1724·3 (1491·6 to 1984·8)	35·5 (29·5 to 41·7)	33·6 (28·0 to 39·6)
	Samoa		69·9 (67·9 to 72·2)	74·0 (72·0 to 76·1)	14·1 (13·4 to 15·4)	15·9 (14·8 to 17·1)	1133·1 (965·5 to 1260·2)	880·5 (746·7 to 1025·3)	0·6 (0·5 to 0·7)	0·6 (0·5 to 0·7)
	Solomon Islands		61·9 (59·3 to 64·3)	64·2 (61·5 to 66·9)	11·5 (10·7 to 12·3)	11·5 (10·3 to 12·6)	1724·9 (1523·6 to 1990·5)	1640·4 (1391·9 to 1933·7)	2·5 (2·2 to 2·9)	2·2 (1·8 to 2·6)
	Tonga		67·5 (65·2 to 70·1)	73·3 (70·9 to 75·6)	13·3 (12·5 to 14·3)	15·7 (14·4 to 17·0)	1278·7 (1106·5 to 1440·7)	907·4 (766·8 to 1075·9)	0·4 (0·3 to 0·5)	0·4 (0·3 to 0·5)
	Vanuatu		62·2 (59·7 to 64·7)	65·7 (62·8 to 68·2)	11·7 (10·9 to 12·5)	12·3 (11·0 to 13·4)	1682·7 (1494·3 to 1922·5)	1467·0 (1255·2 to 1759·2)	1·3 (1·1 to 1·5)	1·1 (0·9 to 1·3)
**North Africa and Middle East**			**70·9 (70·0 to 71·8)**	**75·6 (74·9 to 76·3)**	**15·7 (15·2 to 16·1)**	**17·7 (17·3 to 18·2)**	**968·6 (922·5 to 1019·2)**	**714·1 (678·7 to 753·1)**	**1658·9 (1568·4 to 1753·7)**	**1281·7 (1214·8 to 1355·8)**
Afghanistan			56·8 (54·5 to 59·1)	59·2 (57·5 to 60·8)	10·9 (10·4 to 11·5)	10·7 (10·2 to 11·3)	2007·8 (1826·7 to 2184·0)	1919·1 (1760·8 to 2091·9)	162·2 (140·4 to 186·7)	141·9 (125·4 to 158·9)
Algeria			76·4 (75·1 to 77·7)	78·4 (77·5 to 79·3)	17·8 (17·0 to 18·6)	18·6 (18·1 to 19·0)	696·7 (629·5 to 774·0)	610·5 (571·6 to 654·0)	93·0 (83·2 to 103·4)	85·0 (78·9 to 92·3)
Bahrain			76·0 (73·4 to 78·5)	77·8 (75·7 to 80·1)	15·5 (13·9 to 17·2)	16·6 (15·2 to 18·3)	820·7 (657·1 to 1015·9)	698·6 (562·7 to 843·1)	2·2 (1·7 to 2·8)	1·4 (1·1 to 1·7)
Egypt			69·4 (67·6 to 71·5)	75·0 (73·2 to 77·0)	14·0 (13·0 to 15·1)	16·7 (15·5 to 18·0)	1142·7 (988·8 to 1293·1)	777·9 (665·3 to 894·2)	290·8 (248·6 to 332·3)	226·9 (193·1 to 261·8)
Iran			73·8 (71·2 to 76·2)	78·4 (76·6 to 80·6)	16·3 (14·9 to 17·7)	18·1 (16·9 to 19·6)	855·1 (713·7 to 1034·3)	628·1 (512·4 to 733·2)	218·9 (178·7 to 266·5)	141·8 (113·4 to 171·1)
Iraq			64·8 (61·5 to 69·1)	70·5 (67·4 to 73·5)	13·2 (12·1 to 15·7)	14·9 (13·4 to 16·4)	1390·8 (1052·0 to 1656·0)	1046·8 (848·3 to 1277·9)	128·6 (99·1 to 158·8)	98·9 (78·0 to 122·1)
Jordan			74·7 (71·4 to 78·2)	78·0 (74·9 to 81·0)	16·9 (14·7 to 19·2)	18·4 (16·5 to 20·4)	789·7 (595·3 to 1023·0)	627·6 (483·1 to 807·2)	14·5 (10·8 to 18·6)	11·3 (8·9 to 14·6)
Kuwait			80·0 (76·4 to 83·6)	79·5 (76·6 to 82·7)	19·8 (17·4 to 22·4)	17·3 (15·0 to 20·0)	525·6 (377·5 to 705·3)	616·1 (438·6 to 827·1)	4·2 (2·9 to 5·9)	2·5 (1·7 to 3·5)
Lebanon			78·8 (77·1 to 80·3)	81·4 (79·9 to 82·9)	18·6 (17·3 to 19·7)	20·1 (18·9 to 21·2)	601·7 (524·9 to 702·6)	481·7 (416·8 to 554·3)	14·0 (12·1 to 16·5)	11·8 (10·0 to 13·7)
Libya			72·6 (71·0 to 74·7)	77·6 (76·2 to 78·7)	15·4 (14·7 to 16·8)	18·1 (17·3 to 18·7)	937·3 (810·2 to 1039·2)	653·2 (603·6 to 722·8)	15·4 (13·2 to 17·4)	11·6 (10·5 to 13·1)
Morocco			73·5 (72·5 to 74·5)	76·4 (75·1 to 77·7)	16·5 (16·0 to 16·9)	17·5 (16·6 to 18·4)	846·3 (795·2 to 897·7)	707·3 (636·0 to 790·1)	95·5 (88·6 to 102·1)	89·1 (79·9 to 99·8)
Palestine			70·2 (69·5 to 71·0)	73·5 (72·8 to 74·2)	12·9 (12·6 to 13·3)	13·6 (13·2 to 14·1)	1355·8 (1283·2 to 1420·7)	1063·3 (994·9 to 1124·1)	11·4 (10·1 to 12·8)	9·5 (8·4 to 10·6)
Oman			75·3 (74·5 to 76·3)	79·9 (79·2 to 80·8)	16·0 (15·7 to 16·5)	19·0 (18·6 to 19·5)	816·5 (761·7 to 861·5)	553·6 (512·7 to 584·8)	8·8 (7·9 to 9·7)	3·7 (3·4 to 4·0)
Qatar			79·0 (75·4 to 82·6)	81·8 (78·9 to 85·2)	18·6 (16·1 to 21·3)	20·3 (18·4 to 22·9)	592·7 (424·8 to 806·0)	467·2 (329·3 to 601·8)	2·6 (1·8 to 3·7)	0·8 (0·5 to 1·1)
Saudi Arabia			76·0 (74·9 to 77·0)	78·7 (77·8 to 79·6)	16·2 (15·6 to 16·8)	16·8 (16·1 to 17·5)	779·2 (718·1 to 843·5)	665·6 (607·6 to 719·3)	54·8 (49·9 to 60·1)	35·8 (32·5 to 39·1)
Sudan			66·4 (64·9 to 67·7)	70·3 (68·9 to 71·5)	14·7 (14·2 to 15·1)	15·9 (15·1 to 16·5)	1181·3 (1115·6 to 1245·7)	964·7 (905·5 to 1047·4)	118·5 (104·2 to 139·7)	97·6 (85·1 to 115·0)
Syria			63·3 (56·4 to 71·3)	73·6 (69·1 to 78·2)	15·0 (14·3 to 15·9)	18·1 (17·6 to 18·9)	1308·8 (979·2 to 1654·7)	766·6 (622·8 to 920·5)	75·1 (45·9 to 105·5)	42·5 (30·0 to 55·2)
Tunisia			74·6 (72·1 to 77·2)	80·5 (78·9 to 82·7)	16·1 (14·6 to 18·0)	19·4 (18·3 to 21·0)	836·2 (670·4 to 1004·6)	526·8 (424·9 to 605·2)	35·7 (28·4 to 43·5)	26·7 (21·2 to 31·0)
Turkey			75·8 (73·7 to 78·1)	82·3 (80·4 to 84·3)	17·3 (15·9 to 18·7)	21·1 (19·6 to 22·6)	730·0 (615·7 to 858·9)	430·3 (354·7 to 517·6)	202·6 (166·6 to 240·8)	162·2 (132·1 to 196·4)
United Arab Emirates			74·5 (72·1 to 76·8)	78·6 (76·2 to 80·6)	16·0 (14·8 to 17·0)	18·4 (17·1 to 19·4)	851·3 (724·9 to 1008·1)	619·4 (527·9 to 749·3)	21·1 (15·7 to 27·1)	4·3 (3·4 to 5·6)
Yemen			65·5 (63·7 to 67·2)	67·9 (66·6 to 69·2)	14·5 (13·6 to 15·4)	14·1 (13·6 to 14·5)	1241·3 (1127·9 to 1378·4)	1179·3 (1106·2 to 1273·3)	87·6 (73·4 to 104·5)	75·4 (63·9 to 88·1)
**South Asia**			**67·1 (66·5 to 67·7)**	**70·6 (70·1 to 71·1)**	**13·8 (13·6 to 14·0)**	**15·2 (14·9 to 15·5)**	**1237·9 (1202·8 to 1275·5)**	**997·7 (965·9 to 1029·2)**	**6714·3 (6501·4 to 6931·4)**	**5396·1 (5220·8 to 5572·2)**
Bangladesh			70·5 (68·8 to 72·0)	75·1 (73·5 to 76·4)	15·3 (14·5 to 16·1)	17·6 (16·6 to 18·3)	1007·3 (908·2 to 1129·0)	741·3 (671·6 to 835·8)	498·2 (444·7 to 558·1)	349·7 (313·6 to 396·5)
Bhutan			72·2 (70·1 to 74·4)	75·9 (73·8 to 77·4)	16·3 (15·2 to 17·4)	18·1 (16·9 to 19·0)	896·0 (772·9 to 1028·7)	694·5 (616·8 to 813·2)	2·4 (2·0 to 2·8)	1·5 (1·3 to 1·8)
India			66·9 (66·2 to 67·6)	70·3 (69·6 to 71·0)	13·6 (13·4 to 13·8)	15·0 (14·8 to 15·3)	1265·5 (1228·0 to 1306·5)	1016·9 (981·6 to 1054·3)	5414·9 (5253·2 to 5587·3)	4380·4 (4234·6 to 4539·2)
Nepal			69·7 (68·5 to 71·0)	71·9 (71·1 to 72·8)	14·7 (14·0 to 15·6)	15·2 (14·8 to 15·7)	1075·3 (978·8 to 1169·8)	971·7 (910·2 to 1025·0)	92·2 (83·2 to 101·9)	89·5 (83·2 to 95·4)
Pakistan			66·4 (64·3 to 68·4)	68·9 (66·9 to 71·2)	14·1 (13·2 to 14·9)	14·8 (13·6 to 16·3)	1229·3 (1102·4 to 1385·5)	1078·8 (908·9 to 1249·6)	706·6 (619·5 to 804·0)	574·9 (486·3 to 662·0)
**Sub-Saharan Africa**			**61·2 (60·3 to 62·0)**	**64·6 (63·9 to 65·3)**	**13·7 (13·4 to 14·0)**	**14·4 (14·1 to 14·7)**	**1472·2 (1423·2 to 1524·6)**	**1270·3 (1227·5 to 1311·8)**	**4079·6 (3938·8 to 4245·9)**	**3619·8 (3491·3 to 3761·4)**
Southern sub-Saharan Africa			58·4 (57·4 to 59·3)	64·9 (63·8 to 66·0)	13·4 (13·1 to 13·8)	16·7 (16·3 to 17·2)	1693·6 (1619·9 to 1763·5)	1163·5 (1102·4 to 1224·5)	407·5 (388·1 to 426·3)	351·1 (331·7 to 369·9)
	Botswana		61·7 (58·4 to 67·0)	69·2 (64·5 to 78·5)	12·8 (11·3 to 15·9)	15·8 (12·7 to 23·0)	1639·5 (1175·7 to 1995·2)	1073·6 (532·8 to 1516·0)	10·0 (7·2 to 12·4)	7·0 (3·9 to 9·8)
	Lesotho		47·1 (44·6 to 49·7)	53·7 (49·9 to 58·2)	9·4 (8·6 to 10·4)	12·1 (10·1 to 15·7)	2987·2 (2579·6 to 3385·1)	2083·0 (1542·1 to 2590·4)	17·5 (15·0 to 19·9)	15·1 (11·6 to 18·6)
	Namibia		60·4 (57·8 to 63·3)	69·3 (65·5 to 75·2)	12·7 (11·7 to 14·2)	16·8 (14·4 to 21·5)	1665·9 (1417·4 to 1893·3)	990·5 (651·1 to 1278·6)	10·5 (8·9 to 12·1)	7·5 (5·4 to 9·5)
	South Africa		59·2 (57·9 to 60·3)	65·5 (64·2 to 66·7)	14·2 (13·8 to 14·5)	17·6 (17·3 to 18·1)	1597·9 (1526·4 to 1675·1)	1101·0 (1040·3 to 1165·8)	283·6 (268·6 to 299·7)	250·1 (234·9 to 265·2)
	Swaziland		53·3 (50·2 to 57·1)	62·2 (57·4 to 68·3)	11·1 (9·9 to 13·4)	14·7 (11·7 to 19·1)	2263·0 (1776·1 to 2661·7)	1406·5 (943·9 to 1928·8)	7·9 (6·4 to 9·3)	5·6 (4·1 to 7·4)
	Zimbabwe		56·7 (54·4 to 59·3)	61·9 (59·2 to 65·2)	11·6 (10·5 to 13·1)	13·2 (11·8 to 15·6)	1955·3 (1651·7 to 2236·6)	1521·3 (1201·7 to 1805·2)	78·1 (68·3 to 87·9)	65·7 (55·4 to 76·0)
Western sub-Saharan Africa			61·9 (60·6 to 63·1)	64·8 (63·7 to 65·9)	14·6 (14·0 to 15·1)	15·0 (14·5 to 15·6)	1349·2 (1272·5 to 1432·1)	1206·7 (1132·0 to 1276·1)	1655·0 (1558·4 to 1764·7)	1439·7 (1355·2 to 1526·9)
	Benin		62·5 (60·9 to 64·0)	66·3 (64·8 to 68·1)	13·3 (13·0 to 13·8)	14·3 (13·4 to 15·4)	1433·4 (1346·0 to 1529·0)	1205·1 (1069·3 to 1338·4)	43·3 (39·4 to 47·6)	38·0 (33·9 to 41·8)
	Burkina Faso		59·5 (58·0 to 60·8)	62·1 (61·0 to 63·0)	13·2 (12·9 to 13·5)	13·3 (12·9 to 13·8)	1548·5 (1481·2 to 1619·8)	1429·4 (1358·6 to 1495·3)	82·1 (68·9 to 95·4)	78·3 (67·8 to 88·6)
	Cameroon		58·3 (56·0 to 60·6)	62·0 (59·3 to 65·2)	12·9 (12·1 to 13·8)	14·0 (12·4 to 16·1)	1667·7 (1497·4 to 1849·4)	1402·1 (1149·0 to 1660·9)	116·4 (103·6 to 130·9)	102·2 (86·7 to 117·1)
	Cape Verde		68·8 (66·6 to 71·1)	78·5 (77·2 to 80·2)	14·0 (13·2 to 15·0)	18·8 (18·1 to 20·1)	1175·6 (1016·9 to 1336·3)	601·6 (515·1 to 665·8)	1·7 (1·5 to 2·0)	1·1 (1·0 to 1·2)
	Chad		58·3 (56·4 to 60·3)	61·4 (59·6 to 63·2)	13·1 (12·6 to 14·0)	14·3 (13·2 to 15·2)	1612·6 (1481·2 to 1736·5)	1379·2 (1251·4 to 1534·9)	71·9 (65·5 to 79·2)	64·7 (59·1 to 70·5)
	Côte d'Ivoire		57·7 (55·8 to 59·5)	62·3 (60·5 to 64·0)	12·6 (11·7 to 13·1)	13·0 (12·2 to 14·0)	1725·7 (1608·4 to 1913·3)	1485·2 (1320·4 to 1636·2)	120·5 (107·7 to 135·7)	87·6 (77·7 to 98·6)
	The Gambia		65·5 (64·0 to 66·9)	69·2 (67·6 to 71·3)	14·5 (13·8 to 15·1)	14·9 (14·0 to 16·3)	1253·2 (1157·4 to 1371·8)	1074·7 (922·3 to 1199·0)	6·2 (5·6 to 6·9)	4·9 (4·3 to 5·5)
	Ghana		64·5 (62·8 to 66·1)	67·5 (66·0 to 69·7)	13·9 (13·2 to 14·6)	14·3 (13·6 to 15·7)	1323·7 (1218·6 to 1457·9)	1183·9 (1012·0 to 1298·3)	98·6 (89·0 to 109·6)	90·6 (79·5 to 100·4)
	Guinea		59·8 (57·3 to 62·1)	61·6 (59·5 to 64·0)	13·1 (12·5 to 13·9)	13·0 (12·0 to 14·4)	1559·1 (1395·7 to 1707·1)	1490·4 (1277·6 to 1684·0)	59·6 (52·9 to 67·4)	56·0 (49·4 to 62·7)
	Guinea-Bissau		56·3 (54·5 to 58·2)	61·4 (59·4 to 63·3)	11·1 (10·4 to 12·2)	12·4 (11·6 to 13·3)	2008·6 (1775·3 to 2215·9)	1600·7 (1430·6 to 1783·7)	10·3 (9·3 to 11·3)	8·3 (7·5 to 9·2)
	Liberia		64·0 (62·8 to 65·2)	64·8 (63·6 to 66·1)	13·9 (13·5 to 14·3)	13·4 (12·9 to 14·0)	1337·8 (1258·8 to 1411·3)	1351·9 (1243·9 to 1448·1)	16·3 (15·0 to 17·8)	16·1 (14·9 to 17·3)
	Mali		61·0 (58·1 to 63·8)	62·7 (60·0 to 65·5)	14·7 (13·4 to 16·0)	14·3 (12·7 to 15·7)	1356·4 (1178·3 to 1577·1)	1316·4 (1126·1 to 1559·7)	81·2 (70·5 to 93·7)	74·4 (65·0 to 84·2)
	Mauritania		70·3 (67·8 to 73·2)	70·2 (67·6 to 73·3)	16·1 (14·8 to 17·9)	15·1 (13·8 to 17·1)	954·8 (779·4 to 1123·7)	1022·8 (812·5 to 1211·5)	9·6 (8·0 to 11·2)	10·4 (8·5 to 12·3)
	Niger		60·6 (58·2 to 62·9)	62·8 (60·2 to 65·5)	13·5 (12·7 to 14·5)	14·1 (12·6 to 15·5)	1477·2 (1307·2 to 1644·6)	1326·9 (1142·7 to 1572·6)	94·2 (83·2 to 106·8)	83·4 (72·9 to 94·5)
	Nigeria		63·7 (61·2 to 66·2)	66·4 (64·3 to 69·1)	16·3 (15·0 to 17·8)	16·8 (15·6 to 18·6)	1158·6 (1001·4 to 1333·4)	1036·2 (869·4 to 1173·6)	731·5 (641·9 to 829·3)	622·5 (543·5 to 706·5)
	São Tomé and Príncipe		69·0 (66·7 to 71·5)	72·1 (70·5 to 73·9)	14·6 (13·2 to 16·0)	15·2 (14·5 to 16·2)	1108·8 (937·9 to 1306·2)	967·6 (845·4 to 1076·1)	0·5 (0·4 to 0·6)	0·5 (0·4 to 0·5)
	Senegal		64·6 (63·3 to 66·0)	67·8 (66·6 to 68·8)	13·6 (13·0 to 14·3)	13·9 (13·3 to 14·3)	1342·8 (1237·8 to 1451·8)	1209·3 (1139·5 to 1302·7)	48·9 (45·2 to 53·1)	46·0 (42·8 to 49·9)
	Sierra Leone		58·1 (56·4 to 59·8)	60·2 (58·4 to 62·1)	12·9 (12·4 to 13·5)	12·9 (12·0 to 14·2)	1641·6 (1510·2 to 1760·8)	1539·6 (1355·1 to 1713·1)	32·0 (29·4 to 34·9)	29·5 (26·7 to 32·2)
	Togo		60·1 (58·2 to 61·7)	65·1 (63·6 to 66·8)	12·8 (11·9 to 13·4)	13·8 (13·1 to 15·1)	1606·0 (1478·1 to 1790·3)	1301·4 (1138·1 to 1420·2)	30·2 (27·1 to 33·8)	25·0 (22·3 to 27·5)
Eastern sub-Saharan Africa			61·7 (60·6 to 62·8)	65·1 (64·1 to 66·1)	13·2 (12·7 to 13·6)	13·7 (13·2 to 14·2)	1504·8 (1423·2 to 1588·4)	1323·8 (1242·3 to 1402·0)	1514·2 (1442·3 to 1592·0)	1351·4 (1284·8 to 1425·0)
	Burundi		59·2 (56·0 to 62·3)	61·5 (58·7 to 64·1)	12·3 (11·0 to 13·3)	12·1 (11·2 to 13·0)	1685·1 (1463·0 to 1968·3)	1613·5 (1415·4 to 1848·9)	53·6 (44·7 to 64·5)	49·4 (42·4 to 58·5)
	Comoros		66·6 (64·4 to 68·8)	68·5 (66·8 to 70·9)	14·3 (13·4 to 15·1)	14·3 (13·6 to 15·9)	1208·7 (1077·8 to 1345·3)	1136·1 (954·7 to 1257·6)	2·2 (1·9 to 2·5)	2·1 (1·8 to 2·3)
	Djibouti		64·7 (62·5 to 66·7)	68·8 (66·1 to 71·8)	13·9 (12·9 to 15·0)	15·3 (13·9 to 17·5)	1325·9 (1177·3 to 1508·0)	1070·7 (859·9 to 1267·6)	3·8 (3·2 to 4·4)	3·1 (2·5 to 3·7)
	Eritrea		62·9 (60·6 to 65·1)	64·5 (62·8 to 66·6)	12·8 (11·7 to 13·7)	12·5 (11·7 to 13·4)	1497·8 (1320·2 to 1715·1)	1475·1 (1294·9 to 1633·9)	17·1 (14·9 to 19·6)	17·7 (15·3 to 19·8)
	Ethiopia		64·7 (62·1 to 67·6)	66·5 (64·2 to 68·9)	13·3 (12·0 to 14·8)	13·3 (12·2 to 14·3)	1371·4 (1150·8 to 1618·0)	1320·7 (1132·7 to 1527·4)	346·1 (288·6 to 403·1)	331·2 (285·0 to 382·4)
	Kenya		64·7 (63·9 to 65·6)	69·0 (68·2 to 69·9)	14·1 (13·8 to 14·5)	15·4 (14·9 to 15·8)	1314·7 (1261·5 to 1367·4)	1063·2 (1007·1 to 1116·5)	147·9 (142·4 to 152·9)	121·7 (116·6 to 126·5)
	Madagascar		61·5 (58·4 to 64·8)	63·9 (60·8 to 67·8)	13·0 (11·5 to 14·3)	13·1 (11·7 to 15·5)	1499·9 (1264·0 to 1814·0)	1399·0 (1058·5 to 1685·9)	102·8 (85·4 to 121·3)	94·7 (76·6 to 112·9)
	Malawi		57·9 (55·3 to 60·5)	62·6 (59·8 to 65·9)	12·9 (11·4 to 14·1)	14·0 (12·4 to 16·4)	1717·7 (1504·1 to 2013·4)	1398·4 (1119·5 to 1657·3)	85·0 (74·9 to 96·2)	73·9 (62·8 to 85·5)
	Mozambique		57·0 (54·9 to 59·2)	62·9 (60·4 to 65·4)	13·0 (11·8 to 14·0)	14·8 (13·2 to 16·5)	1740·5 (1558·2 to 1981·1)	1319·2 (1129·1 to 1543·1)	140·3 (125·8 to 157·4)	121·8 (106·5 to 139·0)
	Rwanda		66·0 (63·9 to 67·9)	69·3 (67·2 to 71·5)	14·3 (13·2 to 15·2)	15·0 (13·9 to 16·4)	1240·1 (1107·2 to 1405·4)	1065·1 (908·4 to 1214·1)	34·2 (29·9 to 39·3)	33·9 (29·4 to 38·7)
	Somalia		56·6 (54·1 to 59·1)	57·7 (55·8 to 59·4)	11·9 (10·8 to 12·7)	10·8 (10·1 to 11·5)	1830·6 (1644·9 to 2089·5)	1943·5 (1761·7 to 2164·6)	50·1 (43·2 to 57·8)	51·4 (45·9 to 57·6)
	South Sudan		58·7 (56·1 to 61·5)	60·7 (57·9 to 63·9)	12·9 (12·0 to 13·9)	12·9 (11·6 to 14·7)	1633·9 (1439·2 to 1842·4)	1555·2 (1284·4 to 1809·5)	72·9 (62·7 to 84·5)	67·8 (57·7 to 79·0)
	Tanzania		62·6 (60·5 to 64·5)	66·0 (64·3 to 68·3)	13·6 (12·4 to 14·4)	14·1 (13·3 to 16·0)	1435·4 (1300·4 to 1642·0)	1250·9 (1043·4 to 1385·9)	207·9 (187·6 to 233·9)	181·0 (158·9 to 201·0)
	Uganda		59·8 (58·0 to 61·4)	64·7 (62·9 to 67·0)	12·8 (11·7 to 13·5)	13·6 (12·7 to 15·0)	1630·9 (1488·9 to 1829·4)	1341·1 (1139·9 to 1502·5)	164·8 (151·9 to 180·7)	134·9 (120·5 to 148·0)
	Zambia		55·6 (52·5 to 59·3)	61·9 (58·2 to 66·8)	11·0 (9·9 to 12·9)	13·0 (11·1 to 16·0)	2083·2 (1662·9 to 2440·6)	1532·5 (1106·8 to 1936·8)	84·5 (70·7 to 99·1)	66·0 (51·1 to 81·1)
Central sub-Saharan Africa			60·6 (58·9 to 62·3)	62·8 (61·2 to 64·4)	12·9 (12·4 to 13·3)	12·8 (12·3 to 13·2)	1564·5 (1478·8 to 1660·5)	1489·9 (1398·6 to 1576·9)	502·7 (437·8 to 574·6)	477·7 (421·6 to 539·9)
	Angola		63·9 (60·3 to 67·1)	65·4 (61·9 to 68·7)	13·6 (12·0 to 15·0)	13·3 (11·7 to 15·1)	1371·2 (1154·7 to 1700·3)	1349·0 (1089·6 to 1683·7)	85·4 (68·9 to 105·3)	82·3 (66·3 to 102·4)
	Central African Republic		47·9 (44·5 to 51·6)	52·6 (48·8 to 56·6)	9·4 (8·6 to 10·8)	10·7 (9·3 to 12·6)	2748·4 (2280·0 to 3165·5)	2225·2 (1767·0 to 2693·6)	42·0 (34·9 to 49·5)	36·9 (29·7 to 44·4)
	Congo (Brazzaville)		63·6 (60·1 to 67·3)	62·4 (59·6 to 65·6)	13·9 (12·4 to 15·5)	12·4 (11·0 to 13·9)	1370·9 (1117·8 to 1664·2)	1591·8 (1304·1 to 1893·2)	17·8 (14·5 to 21·3)	20·5 (16·8 to 24·2)
	Democratic Republic of the Congo		60·4 (58·3 to 62·4)	62·7 (60·8 to 64·5)	12·9 (12·4 to 13·3)	12·8 (12·3 to 13·3)	1562·1 (1463·9 to 1669·2)	1484·0 (1383·7 to 1586·4)	347·3 (286·9 to 417·1)	328·8 (275·4 to 387·5)
	Equatorial Guinea		64·5 (60·2 to 69·1)	66·6 (62·3 to 71·4)	15·3 (13·3 to 18·0)	15·7 (13·1 to 19·3)	1233·1 (946·5 to 1567·4)	1135·4 (812·0 to 1507·1)	3·2 (2·4 to 4·0)	2·6 (2·0 to 3·5)
	Gabon		64·9 (61·4 to 68·4)	68·3 (65·9 to 71·1)	14·3 (12·6 to 16·0)	14·2 (13·1 to 16·0)	1295·8 (1057·9 to 1599·9)	1168·8 (946·7 to 1371·8)	7·1 (5·8 to 8·7)	6·6 (5·5 to 7·8)

Data in parentheses are 95% uncertainty intervals. Age-standardised rates are standardised with the GBD world population standard. To download the data in this table, please visit the Global Health Data Exchange (GHDx). GBD=Global Burden of Disease. SDI=Socio-demographic Index.

On a global level, life expectancy at birth increased overall by 13·5 years for men and 14·8 years for women from 1970 to 2016. In 2016 there was a 1·7-times difference between the lowest overall life expectancy of 50·2 years (95% UI 47·3–53·3) in the Central African Republic to the highest of 83·9 years (83·8–84·1) in Japan. The highest life expectancy was in Singapore for men, at 81·3 years (78·8–83·7), and in Japan for women, at 86·9 years (86·7–87·2). In 2016, the lowest life expectancy at birth worldwide was in Lesotho for men, at 47·1 years (44·6–49·7), and in the Central African Republic for women, at 52·6 years (48·8–56·6). Life expectancy at birth was greater than 80 years for women in 57 countries and in just 10 countries for men. Life expectancy at birth was less than 50 years for men and less than 55 in women in Lesotho and Central African Republic. Geographic patterns in life expectancy at age 65 years were similarly variable. In 2016, female life expectancy at age 65 years was highest in Japan (24·2 years, 24·1–24·4), Singapore (23·3 years, 21·6–25·2), and France (23·2 years, 22·8–23·7), whereas male life expectancy at age 65 years was highest in Kuwait (19·8 years, 17·4–22·4), Singapore (19·7 years, 17·9–21·5), and Japan (19·5 years, 19·4–19·7). Life expectancy at age 65 years was less than 10 years for men in Lesotho and the Central African Republic.

### Observed versus expected life expectancy

**Figure 6:**
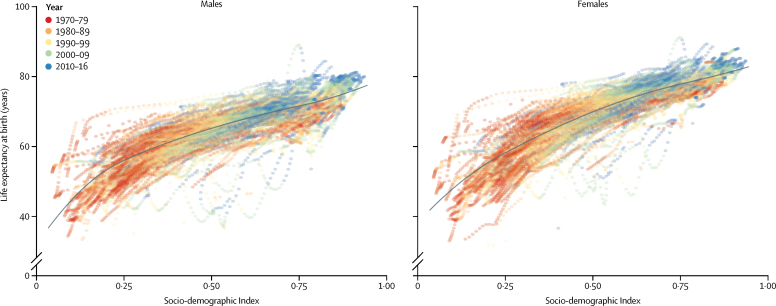
Life expectancy at birth, by sex, and fit of expected value based on SDI, 1970–2016 Each point represents life expectancy at birth in a single location-year by that location's SDI in the given year, coloured by decade. SDI in most locations has increased year on year, so points from earlier years are associated with lower SDI in most cases. The black lines indicate expected values based on SDI. SDI=Socio-demographic Index.

The entire GBD dataset of life expectancy at birth from 1970 to 2016 for the 195 countries and territories, as well as the expected value of life expectancy as a function of SDI is shown in figure 6. The expected value is based on fitting of the expected pattern of age-specific mortality and using the expected death rates to calculate expected life expectancy. In addition to the strong relationship between life expectancy at birth and SDI (correlation of 0·83 for men and 0·87 for women), the figure also shows the strong temporal shift over the decades towards higher life expectancies. This shift is most evident at high SDI and not evident at levels of SDI from 0 to 0·7 (ie, in most low-income and middle-income countries; appendix section 8 p 113).

**Figure 7:**
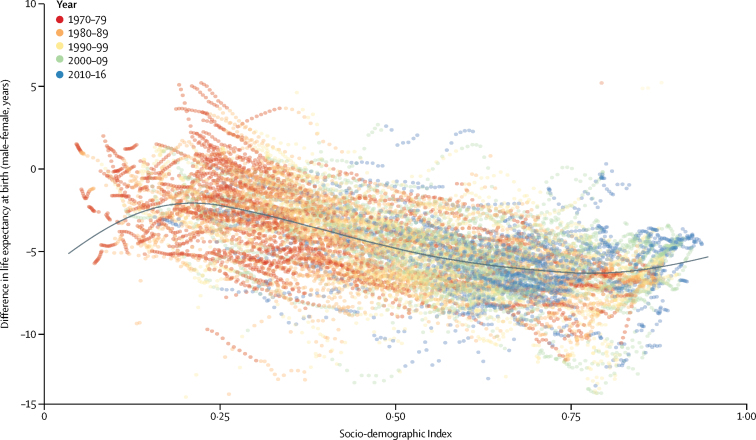
Differences between male and female life expectancy at birth by SDI, 1970–2016 Each point represents the gap in life expectancy at birth between males and females in a single location and year. The black line shows the global trend by SDI. SDI=Socio-demographic Index.

In the most location-years, male life expectancy at birth was less than female life expectancy at birth (figure 7). The GBD estimation of sex differences in life expectancy shows a clear pattern of an increasing negative difference between male and female life expectancy as countries achieve higher levels of SDI. The expected difference between male and female life expectancy reaches a maximum of 6·3 years at an SDI of 0·78; above this level of SDI, the difference in life expectancy between men and women is narrower. At high SDI there were generally smaller differences in life expectancy in the most recent time periods. During the entire period of estimation and across all levels of SDI, there were 324 location-years of negative difference in which male life expectancy was more than 10 years lower than female life expectancy, including for Latvia, Estonia, and Lithuania at high SDI. Conversely, during the entire estimation period, there were 1725 location-years with a positive difference whereby male life expectancy was higher than female life expectancy, although only 351 such location-years occurred in the most recent period, 2000–16. In 2016, the largest negative difference between male and female life expectancy at birth at the national level was in Russia, where male life expectancy (65·4 years, 60·8–70·7) was 10·8 years lower than female life expectancy (76·2 years, 71·4–80·7). In 2016, only three locations at the national level had an estimated male life expectancy that was higher than female life expectancy: Congo (Brazzaville), Kuwait, and Mauritania. Around the observed patterns of increasing and decreasing difference in life expectancy between men and women, there was also large variation in the sex differences in life expectancy within the same level of SDI.

**Figure 8:**
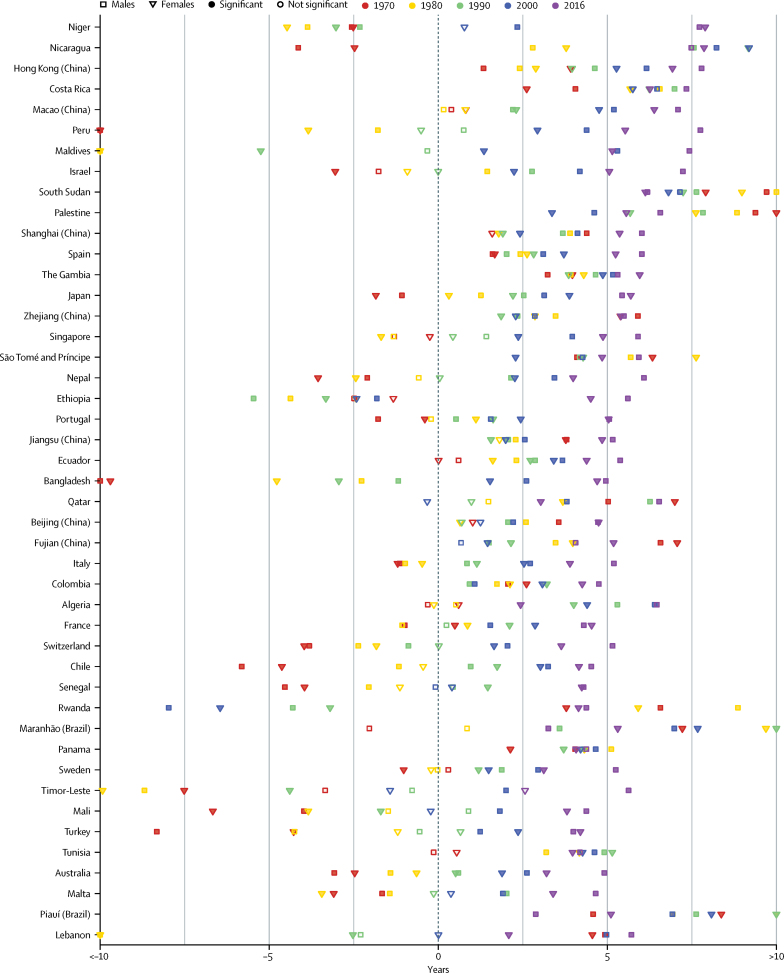

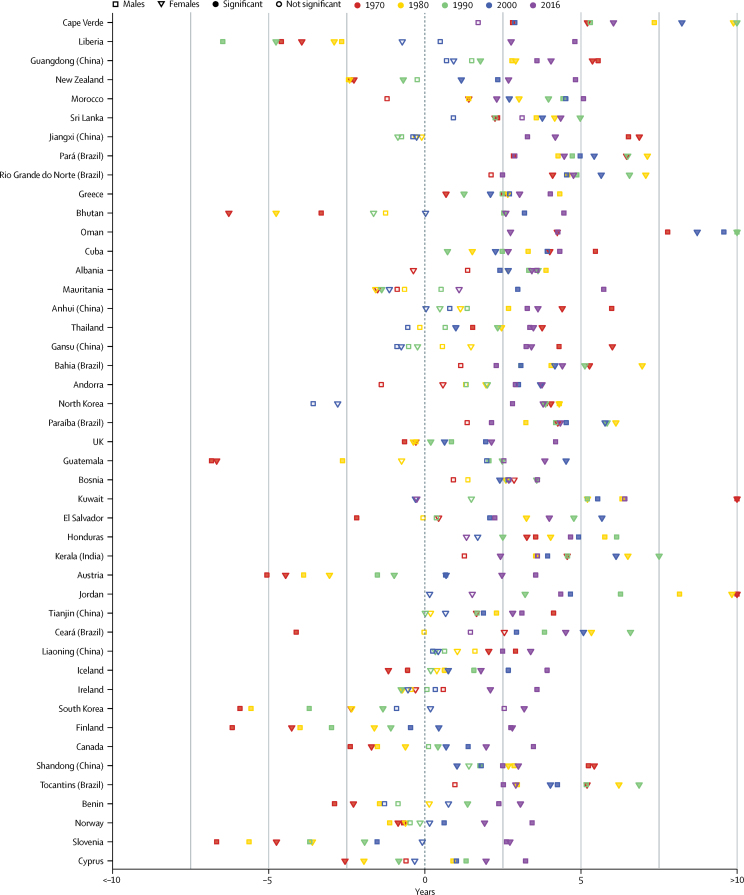

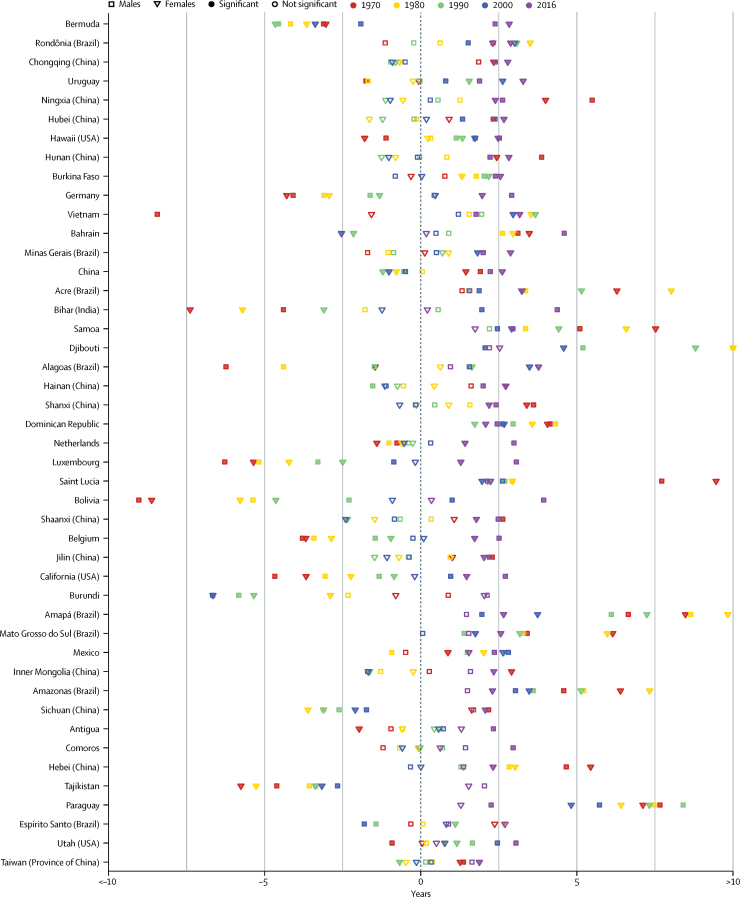

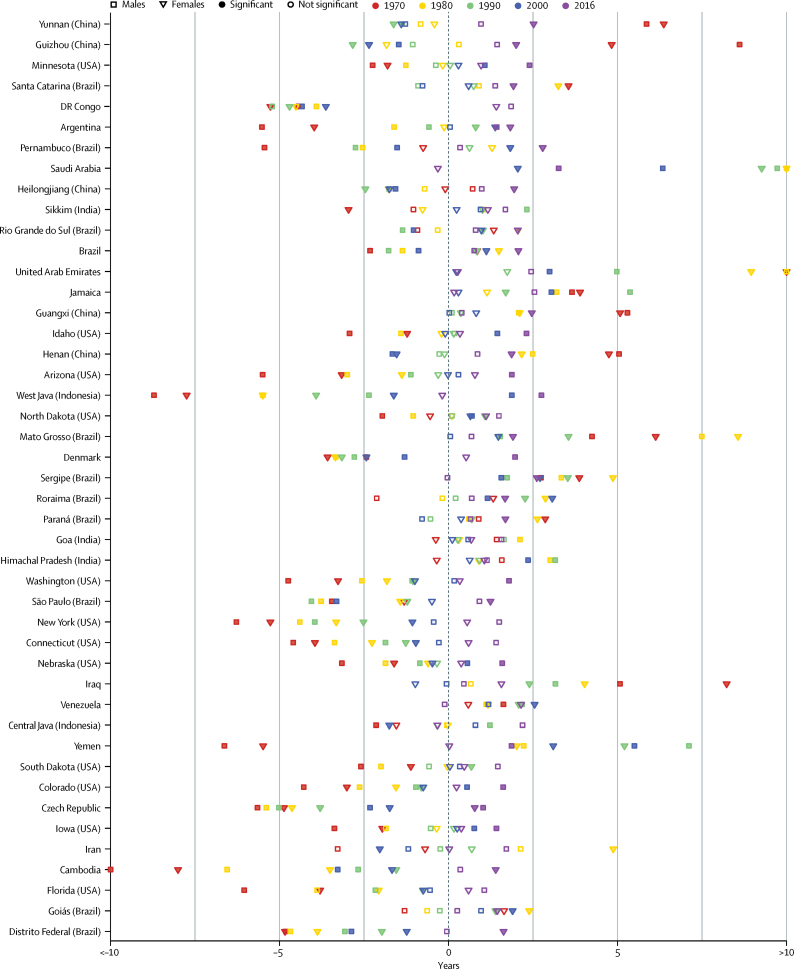

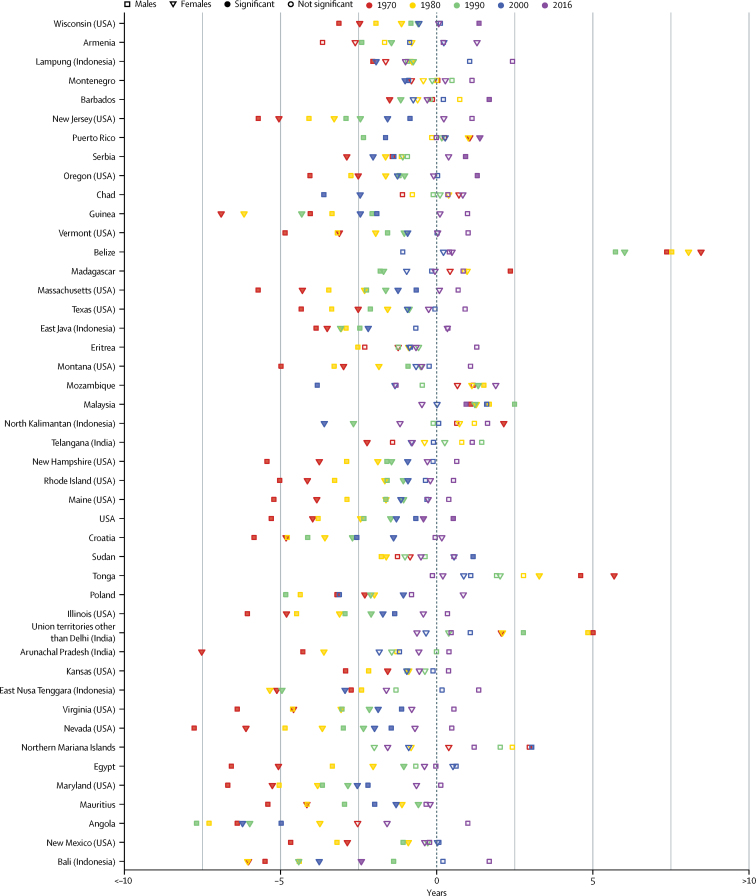

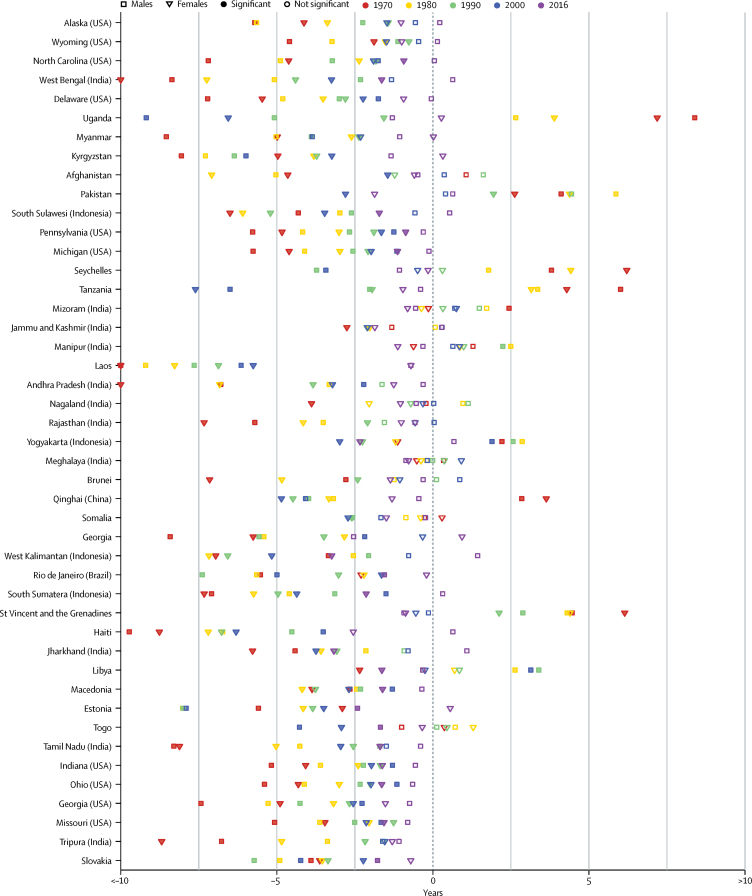

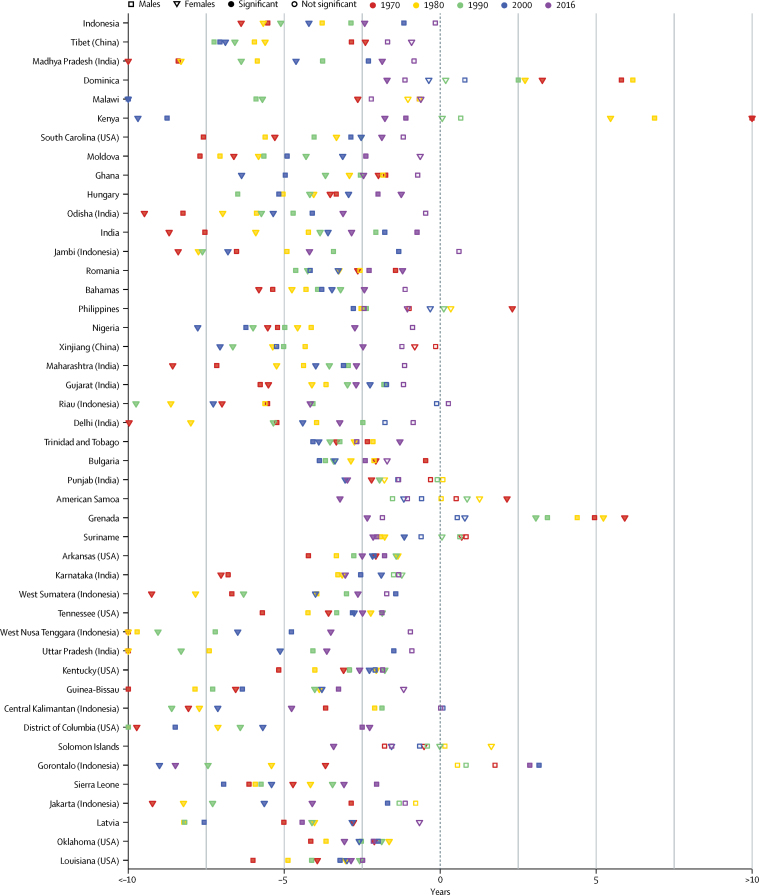

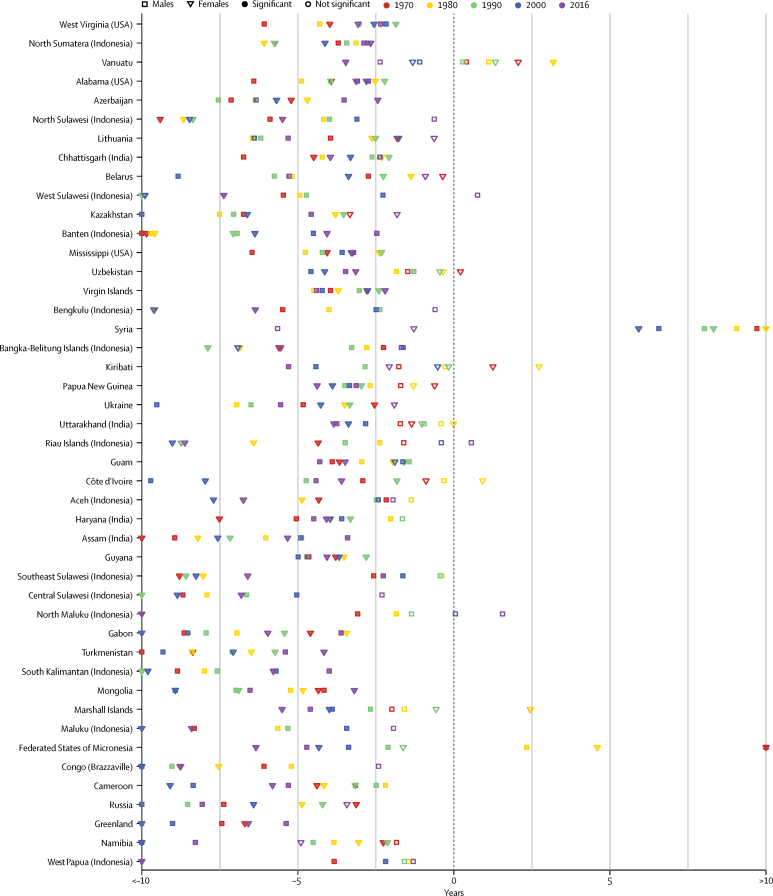

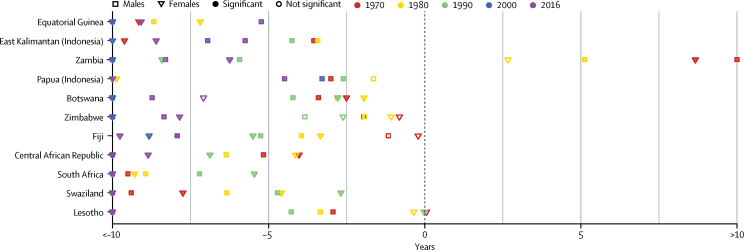
Difference between observed and expected life expectancy at birth on the basis of SDI alone for countries and territories, and subnational units in Brazil, China, India, Indonesia, and the USA, by sex, 1970–2016 Each point represents the observed minus the expected life expectancy for each location in the given year, by sex. Points are colour-coded by range of years with squares representing males and triangles representing females. The 0 line represents no difference between observed life expectancy and the value expected on the basis of SDI. Solid points represent significant differences and hollow points represent differences that are not significant. Locations are in decreasing order by average male and female difference between observed and expected life expectancy at birth in 2016. SDI=Socio-demographic Index.

Figure 8 shows the difference in observed life expectancy at birth and life expectancy at birth anticipated on the basis of SDI for men and women in 1970, 1980, 1990, 2000, and 2016, with locations ordered by the average value for men and women in 2016. This difference quantifies how much a location exceeds the value expected on the basis of SDI alone. In 2016, higher than expected life expectancies occurred in many regions; at the national level, Niger, Nicaragua, Costa Rica, Peru, and the Maldives had the largest positive differences between observed life expectancy and that expected in 2016. Negative differences—where observed life expectancy was below that expected on the basis of SDI—were largest in Lesotho, Swaziland, South Africa, the Central African Republic, and Fiji. Figure 8 also shows how the difference between observed and expected life expectancy at birth has changed over time. For example, the general trend in Costa Rica has been for increases in this difference, from an average difference for both sexes of 3·3 years in 1970, the positive difference increased and life expectancy has remained 6 or more years higher than expected since 1980; whereas, in 1970, Cuba had a life expectancy 4·7 years higher than expected, which subsequently decreased to 3·5 years higher than expected in 2016.

The largest increases in positive differences between observed and expected life expectancy during the period 2000–16 were achieved by Niger (7·1 years for women and 5·3 years for men), Senegal (3·8 years for women and 4·4 years for men), Timor-Leste (4·0 years for women and 3·6 years for men), and Mali (4·0 years for women and 2·6 years for men). Reductions in the negative difference between observed and expected life expectancy also represent improvement. From 2000 to 2016, notable improvements of this type occurred in many locations in sub-Saharan Africa, such as Botswana, Zimbabwe, Malawi, and Zambia, where the negative difference in observed and expected life expectancy at birth shrank by 10 or more years. Outside of sub-Saharan Africa, the largest decreases in negative difference over that period were by 6·0 years in Kazakhstan, 5·2 years in Laos, and 4·8 years in Estonia. National locations notable for progressing from having observed life expectancy below the level anticipated on the basis of SDI in the year 2000 to observed life expectancy greater than expected in 2016 included: Rwanda (from 7·2 years less than expected to 4·3 years greater than expected), Burundi (from 6·7 years less than expected to 2·0 years greater than expected), and Ethiopia (from 6·7 years less than expected to 5·0 years greater than expected). By contrast, the so-called 4C countries—China, Costa Rica, Cuba, and Chile—noted by the *Lancet* Commission on Investing in Health^5^ as exemplar locations for improvements in health outcomes in previous time periods, were notable for their comparatively slower success in reducing the difference from expected life expectancy at birth during the most recent period. From 2000 to 2016, the positive difference in observed and expected life expectancy grew slowly, increasing for women by 0·5 years in Costa Rica, 0·4 years in Cuba, and 1·1 years in Chile, and increasing for men by 0·9 years in Costa Rica, 0·4 years in Cuba, and 1·3 years in Chile. Contrasting with this slower progression, the most recent improvements in China remained notable. From 1990 to 2000, the negative difference in observed and expected life expectancy decreased by just 0·2 years for women and 0·1 years for men; however, from 2000 to 2016, observed life expectancy in China exceeded the level expected on the basis of SDI for the first time since the 1970s, by 2·6 years for women and 2·2 years for men.

## Discussion

### Main findings

Mortality rates decreased for most age groups from 2000 to 2016, with particularly notable decreases for adolescents and younger adults in several world regions. From 1970 to 2016, life expectancy at birth overall increased by 13·5 years for men and 14·8 years for women. Over the past 47 years, life expectancy at birth increased in 194 of 195 locations included in this analysis; the only decline observed was related to conflict in Syria. Globally, U5MR declined from 2000 to 2016, decreasing from 69·4 per 1000 livebirths (67·2–71·8) to 38·4 per 1000 livebirths (34·5–43·1), giving an average decrease of 3·7% per year (3·0–4·3); of 195 countries and territories, 189 had declines in U5MR since 2000. In many countries, major increases in mortality have been followed by faster rates of decline in subsequent years or decades,^17^ leading to some degree of mortality rate catch-up declines for locations that had mortality increases. More recent increases in mortality in some countries^8^—possibly linked to drug use, self-harm, homicide,^7^ tobacco use,^33^ and obesity^34^, ^35^, ^36^—are still occurring. As mortality has declined, the capacity to monitor detailed trends through VR systems has improved globally: in 1970, 28% of deaths were registered, increasing to 34% in 1990, with a peak of 45% in 2013. Male life expectancy was generally lower than female life expectancy in the most locations and time periods. Niger, Nicaragua, Costa Rica, Peru, and the Maldives had the highest levels of observed life expectancy relative to the level expected in 2016 on the basis of SDI.

### Stillbirths and under-5 mortality rate

Enormous global progress has been made in reducing the stillbirth rate over the past 47 years, but the stillbirth rate varies considerably across countries and even across countries with similar neonatal mortality rates. The global trend toward reduced stillbirth rates and numbers could be further accelerated by addressing common risks—low access to antenatal car or skilled birth attendence and detection and treatment of maternal disorders during pregnancy, for example—particularly in countries with the highest burden of stillbirths. Although we provide an overall assessment of stillbirth rates, our understanding of trends in stillbirths is hampered by the inconsistent definitions used in many studies and national reporting. Standardised reporting in VR systems and in other studies would enable the future tracking of stillbirths.

The number of deaths among children younger than 5 years has declined substantially over the past 47 years, with rates of change accelerating in many countries since 2000, particularly compared with the previous decade, 1990–2000. In 2016, the number of under-5 deaths dropped below 5 million for the first time. This finding is continued evidence that progress is being made in tackling the key causes of child death, which is probably linked to successful strategies and trends, including increased educational levels of mothers,^37^, ^38^ rising incomes per capita,^39^, ^40^ declining levels of fertility,^41^, ^42^, ^43^ scale-up of vaccination programme,^44^, ^45^ mass distribution of insecticide-treated bednets,^46^, ^47^, ^48^ improved water and sanitation,^49^, ^50^, ^51^ and the impact of a wide array of other health programmes funded by expanded provision of development assistance for health.^52^ The multiple global development initiatives that have prioritised maternal and child health over the past four decades have also raised and maintained global and national policy attention on child mortality.^3^, ^53^, ^54^, ^55^, ^56^ However, trends in the decline of child mortality in the past decade suggest that the ambitious SDG targets—setting absolute levels of the U5MR at below 25 deaths per 1000 livebirths and a neonatal death rate equal to or lower than 12 deaths per 1000 livebirths by 2030—can only be achieved if the pace of progress is accelerated, particularly in countries in sub-Saharan African such as the Central African Republic, Chad, Mali, and Sierra Leone. If trends seen in the period 2000–16 were to continue through to the year 2030, 148 of the 195 countries in the GBD study would achieve the SDG U5MR target and 47 countries would not. Similarly, although 157 countries could achieve the SDG neonatal mortality target by 2030 on the basis of current trajectories, 38 countries would not reach this target. The shift in the SDGs from relative targets of a two-thirds reduction in the starting U5MR to an absolute target requires a major shift in how resources are allocated. Increased funding in general and prioritisation of support for countries that are farthest from these targets will be needed to accelerate progress. Enhanced funding of health programmes, expansion of female education, and continued innovation in interventions for child mortality reduction will likely be key parts of the strategy to achieve the SDG targets for child and neonatal mortality.

### Adolescents

The GBD 2016 results showed great variation in the health profiles of 10–24 year olds. Although annualised rates of change in mortality rates for adolescents decreased for most locations from 2000 to 2016, this age group also included some of the largest increases estimated for this time period, most notably for adolescent girls in Syria and adolescent boys in Syria, Yemen, Iraq, and Libya. It is during adolescence that inequities related to gender, poverty, and social disadvantage have their greatest effects,^57^ and this is reflected in the distribution of increasing annualised rates of change in mortality rates for this age group. Adolescence is a formative life stage for health and wellbeing.^57^, ^58^ Investment in adolescent health will result in benefits during the adolescent and young adult years, healthier trajectories across the adult life course, and the healthiest possible start for the next generation, given that adolescents are the next generation to be parents. Unfortunately, relative to other age groups, adolescent health has attracted little policy attention, although adolescent health is central to nearly every major agenda in global health, including HIV/AIDS, mental health, injury, maternal, newborn, and child health, and non-communicable diseases.

### Younger adults

Our analysis shows that during 1970–2016, mortality rates in men aged 25–49 years increased in 32 (16%) of 195 countries; for women in this age range, mortality rates increased in 18 (9%) countries. Increased mortality rates were more common in the earlier periods analysed, particularly for men; in this age group, mortality rate increased in 62 (32%) countries from 1980 to 1990 and increased in 100 (51%) countries from 1990 to 2000. For another subset of countries—27 (14%) countries for men and 55 (28%) countries for women—mortality in younger adults declined by at least 2% per year from 1970 to 2016.

The enormous variability in trends in this age group reflects the impact of many factors that have disproportionate effects on younger adults, such as conflict and terrorism, HIV/AIDS,^59^ injuries, and alcohol-related or drug-related mortality.^17^ However, these increased mortality rates occurred not only among countries heavily affected by the HIV/AIDS epidemic and other public health crises such as drug,^7^ tobacco,^33^ and alcohol use,^60^ but also in countries such as the USA, Portugal, Nicaragua, Guam, Jamaica, and Venezuela in different decades, where sources of mortality increases, including obesity,^36^ diabetes,^61^ chronic kidney disease,^62^ smoking,^33^ ischaemic heart disease, or cirrhosis, might be additional factors.^63^ Mortality in these age groups has been studied far less than mortality in children, adolescents, and the elderly. In light of our observations, more study and policy attention is justified for these age groups.

### Older adults

Whereas improvement in mortality among children younger than 5 years and young adults has been substantial (figure 3A), changes in mortality among people older than 65 years have been relatively small. A consequence of the improvements in survival at younger age groups over the past five decades is that more people are living to older adult ages. Understanding the levels and trends of mortality in older age groups has increased relevance for countries at high SDI with greater life expectancy and is rapidly becoming more relevant among countries lower on the SDI scale because of decreases in mortality and a decline in fertility over the past five decades. Life expectancy at age 65 years increased in almost all countries from 1970 to 2016, and these increases typically occurred across successive decades, a trend with significant implications for health care, health insurance, and social security systems across all levels of SDI. Given the large fraction of the world's population that will probably survive into these age groups, more research and policy attention on the determinants of mortality and health at these ages is warranted.

### Differences between male and female mortality

For the first time, we have characterised how sex difference in life expectancy (and age-specific mortality) compare with the patterns expected on the basis of development status. The characteristic U-shaped pattern that we saw, in which males fall progressively behind females with increasing SDI until high levels of SDI where the gap narrows, deserves attention. Throughout the development process, mortality rates decline more slowly for men than for women across a range of SDI levels, from low SDI to upper-middle SDI. At high SDI, male progress is faster, especially in middle-aged and older adults, leading to a narrowing difference in life expectancy; the temporal shifts shown in figure 7 also suggest that this U-shaped curve has a more marked upturn over time at high SDI. The extent to which this narrowing gap is due to shifts in male versus female behaviours in settings with much more equal labour force participation versus risk exposure to factors such as tobacco deserves further analysis.

### Compensating mortality change

In analysing the rates of change in age-specific mortality, both over the long-term from 1970 to 2016 and by decade, it is notable that the two GBD super-regions in which many locations had increasing mortality in the 1980s and 1990s, sub-Saharan Africa and central Europe, eastern Europe, and central Asia, had large decreases in 2000–16. This catch-up phenomenon can partly be explained as a regression to the mean, in which exceptional circumstances in a location result in increases or fast rates of decline in one time period that revert back to more typical patterns in later time periods. In addition to this phenomenon, however, we have found clear compensating patterns after unique mortality increases: for example, the rise and subsequent fall in mortality as a consequence of ART scale-up in countries with large HIV/AIDS epidemics,^59^, ^64^, ^65^, ^66^ as well as the rise and then fall of alcohol-related mortality in eastern Europe and central Asia.^67^, ^68^, ^69^, ^70^ Additionally, for many locations, such as Rwanda or Liberia, episodes of major conflict and terrorism have been followed by rapid declines in mortality in the subsequent decade.^71^, ^72^ Regression to the mean and compensating rates of change in mortality after major increases result in greater consistency in long-term rates of change for age-specific mortality than is seen in any given year or decade. These compensating rates of change can be driven by concerted societal response to major mortality reversals. As new types of adverse mortality events arise, such as increased mortality related to drug use, obesity, or chronic kidney diseases,^17^, ^62^, ^73^, ^74^ the likely consequence is that, over a longer period of observation, there will be a concerted societal response to counter these effects. This likely mechanism will also be an important factor in assessing the long-term impact of emerging mortality threats such as food insecurity stemming from climate change.

### Convergence

The *Lancet* Commission on Investing in Health^5^ called for a “grand convergence” in health, through the elimination of global inequalities in mortality rates. This vision was instrumental in the call by the governments of the USA, India, and Ethiopia to end preventable maternal and child death in a generation. These efforts were central to the development of the absolute targets for U5MR and neonatal mortality rate set in SDG goal 3. More generally, however, assessments of convergence in the research literature on mortality inequalities have focused on relative measures, such as the ratio of the death rates in the top quintile of SDI to those in the bottom quintile.^5^, ^75^, ^76^, ^77^, ^78^ In order for ratios of death rates between the worst off and best off to narrow, the worst off need to have faster, not slower, rates of decline. We found that convergence, when measured as the relative change in death rates, has occurred for children aged 1–4 years, adolescents, and young people, and for men older than 55 years; however, there has been relative divergence for women and men aged 30–54 years, as well as for women through to age 84 years. When convergence is assessed in terms of the absolute difference in death rates, the findings are much more positive, showing clear convergence in all age groups. Although relative convergence is a high bar, it is not impossible, as shown by the progress achieved in some age groups. The potential for mortality to be reduced through access to high-quality personal health care and through population-level modification of risk factors means that this divergence is not necessarily inevitable.^79^, ^80^, ^81^, ^82^, ^83^, ^84^, ^85^ More attention on how even faster progress can be made in the worst-off places will be needed to achieve convergence in relative death rates.

### New generation of exemplars

In the 1980s, the book *Good Health at Low Cost*^86^ called attention to the notable levels of health achieved in several lower-income locations: China, Cuba, Costa Rica, Sri Lanka, and the Indian state Kerala. Nearly 30 years later, this assessment was updated by Balabanova and colleagues,^87^ who highlighted Bangladesh, Ethiopia, Kyrgyzstan, Thailand, and the Indian state Tamil Nadu, while the *Lancet* Commission on Investing in Health^5^ called attention to what has been called the 4C countries (China, Cuba, Costa Rica, and Chile). Identification of exemplars is important because examination of these countries can identify successful policy strategies or leadership approaches. In our analysis comparing observed life expectancy to that expected on the basis of SDI alone, some of the locations previously identified as success stories do not stand out to the same degree in 2016. Across 371 locations, ranked by the difference between observed and expected life expectancy, Cuba ranked 58th, China 104th, Kyrgyzstan 233rd, Thailand 62nd, Kerala 74th, and Tamil Nadu 264th. By contrast, countries such as Niger, Nicaragua, Peru, The Gambia, Nepal, and Ethiopia are low-SDI to middle-SDI nations that rank 1st, 2nd, 6th, 13th, 18th, and 19th, respectively. When the assessment is extended to look at which countries have improved the gap between observed and expected life expectancy the most since the year 2000, the top five countries include Botswana, Zimbabwe, Rwanda, Malawi, and Zambia; in four of these countries, the scale-up of ART played a crucial role in the recent progress. Among countries where observed life expectancy minus expected life expectancy was greater than 5 years in 2016, the locations with the largest gains since 2000 were, in order, Ethiopia, Niger, Portugal, Peru, and the Maldives. The policies and leadership strategies of these countries should be studied in more depth to see whether consistent messages that are relevant to other countries can be identified. Our quantified approach of directly comparing expected life expectancy with that anticipated on the basis of SDI provides a more rigorous assessment of which countries have achieved the most impressive improvements in life expectancy that are not explained by income per capita, educational attainment, or fertility decline. Some of the differences in our assessment from past efforts such as *Good Health at Low Cost* might be related to the use of SDI instead of GDP per capita because it removes from the assessment countries with higher life expectancy related to early investment in educational attainment. Education, especially education of young girls,^88^, ^89^, ^90^ is a powerful driver of health improvement, and our identification of countries that have performed well while controlling for SDI should not be taken as evidence to the contrary.

### Measurement challenges

In our analysis, we estimated that 80 countries have civil registration systems that capture more than 95% of deaths in the most recent year with available data. The number of countries with VR systems capturing more than 95% of deaths increased from 57 in 1970 to a peak of 76 in 2012. The fraction of global deaths that are registered has increased from 28% to a peak of 45% over the past 47 years. Progress made on VR has been larger than previously appreciated.^91^ The increased coverage of death registration is good news, and suggests that further accelerated progress might be possible. The rapid increase in coverage in China is particularly encouraging. However, some countries have stagnated, with VR completeness remaining stubbornly in the range of 70–90%. Several new initiatives have been launched to strengthen civil registration, such as the Bloomberg Data for Health Initiative and the World Bank Global Financing Facility for Maternal and Child Health, which include VR systems to monitor progress.^92^ These efforts to strengthen registration will hopefully improve the precision of age-specific mortality estimates by reducing the dependency of the GBD study and various national monitoring efforts on model life tables and other statistical modelling. The annual assessment of VR completeness from the GBD studies can provide a mechanism to evaluate progress on this important global agenda.

In the GBD assessment, we have not used U5MR to predict adult mortality rates or age-specific mortality rates directly in places without empirical mortality data from civil registration. Instead, we have depended on sibling history data collected through household surveys and Bayesian statistical models that incorporate key covariates, including lag-distributed income per capita, educational attainment, and HIV/AIDS mortality, which affect all-cause mortality. The relationship between under-5 mortality and adult mortality in our results has remained generally weak over the past five decades. This generally weak relationship should not be surprising, especially in the past two decades, given the focused investments in child health and the development of effective interventions for child health. More importantly, this weak relationship highlights the problems of using child death rates as the main driver to predict adult mortality, and thus the full age pattern of mortality, as is the practice for UNPD^15^ and USCB^11^ estimation.^93^

Our estimates of mortality in locations with ongoing conflict and terrorism, such as in Syria or Yemen, are limited by data that are generally not validated and are based on eyewitness recall and by the sparseness of data on indirect sources of mortality during conflict and terrorism, such as poor access to health care, famine, or epidemic diseases that affect civilian populations. The direction of the bias for these types of sources is unknown. In the GBD we have made substantial use of the Uppsala Conflict Data Program (UCPD) database, which records the range of estimates for many conflicts. The UCPD database, however, does not provide detailed information on the source of the estimate for each event. In future work, we hope to collaborate with groups that track conflicts and disasters to more precisely characterise the nature of the source for each event.

### Comparison of GBD 2016 to other estimates

The Research in context panel summarises the main changes between GBD 2015 and GBD 2016. Substantial correlation exists between GBD 2016 stillbirth results and those published by the Stillbirth Epidemiology Investigator Group (SEIG);^94^, ^95^ where results differed the most, the probable explanation is related to variation in modelling strategies. The unified modelling strategy used by GBD, which includes methods developed to account for non-sampling biases from different data sources, supports the estimation of an internally consistent time series of levels and rates for stillbirths across locations; a comparison between modelling strategies for SEIG and GBD and resulting estimates is shown in the appendix (section 8 p 74).

**Figure 9:**
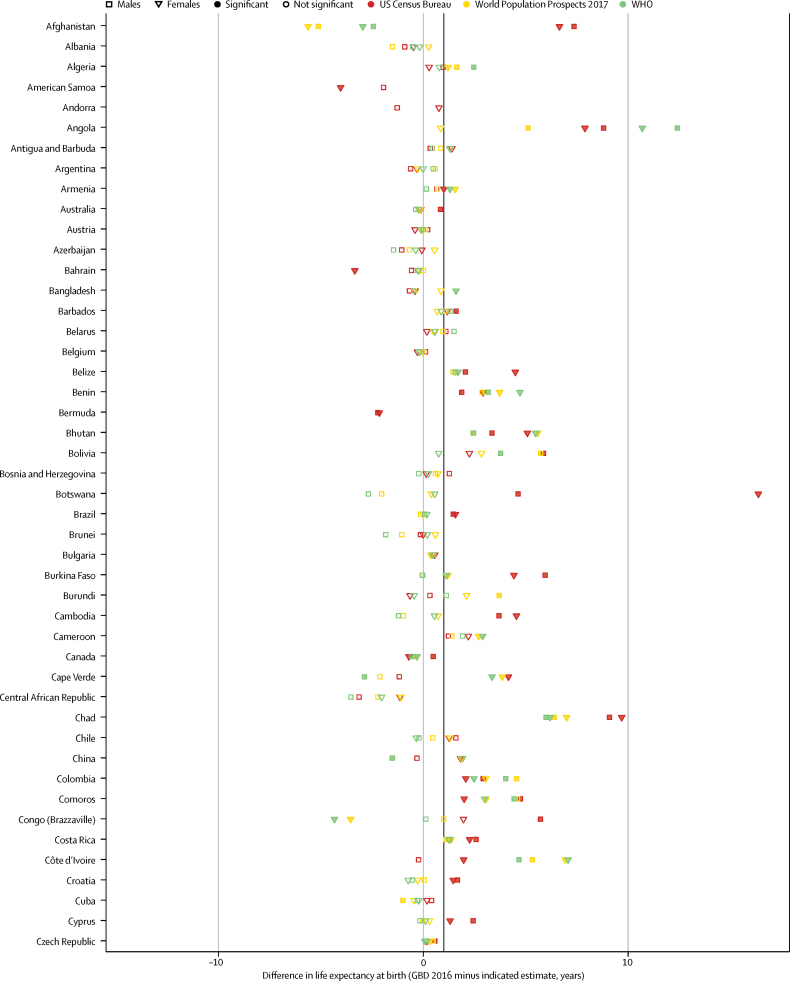

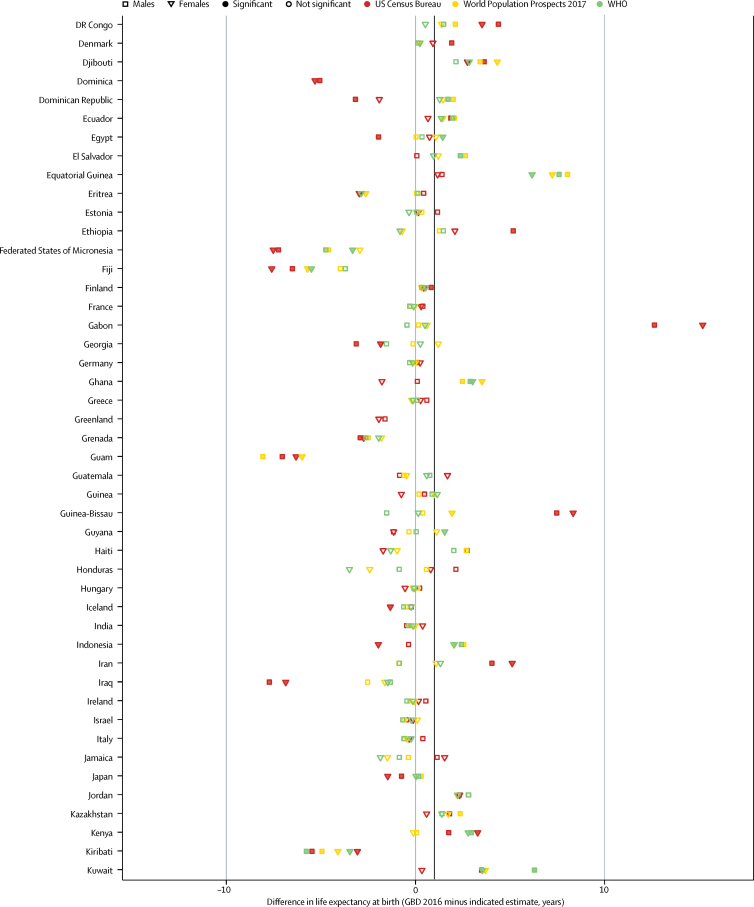

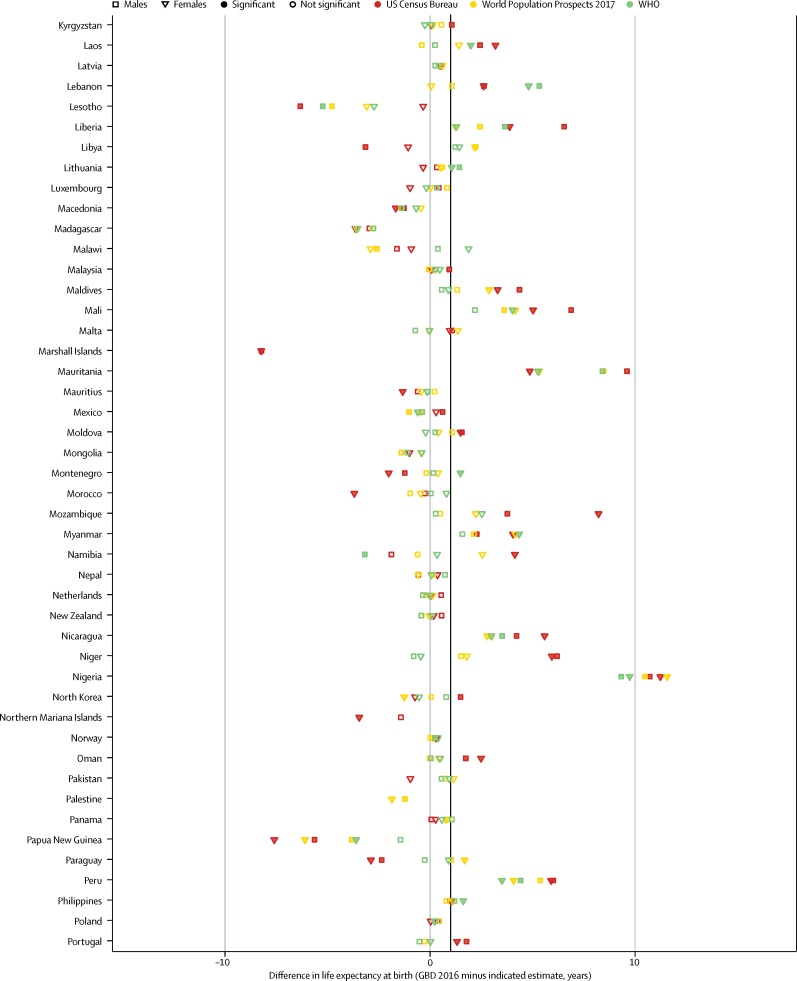

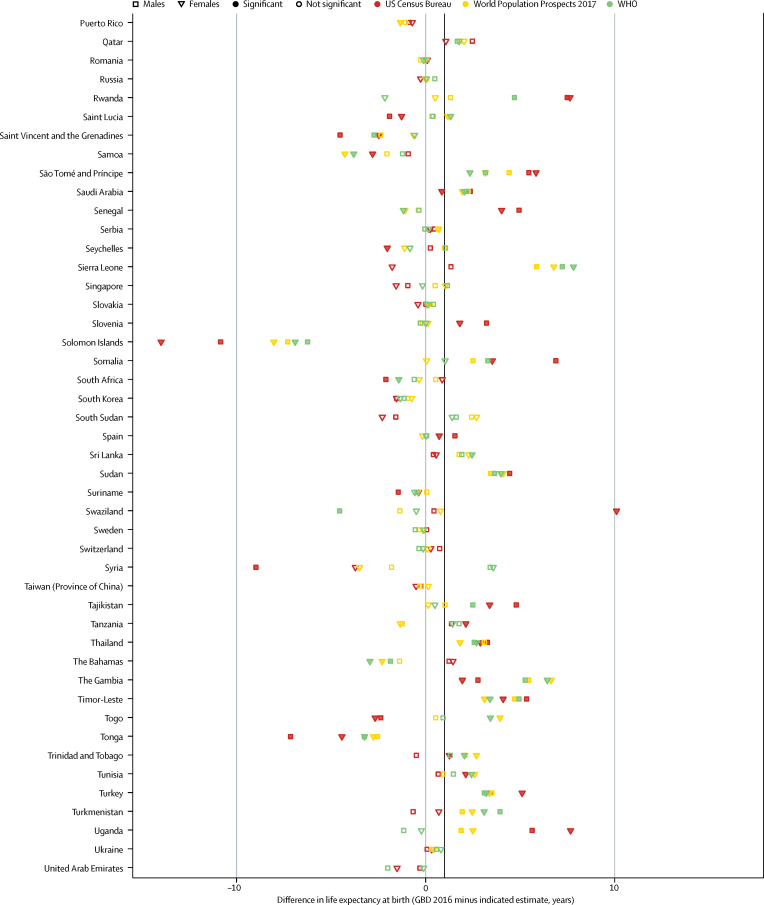

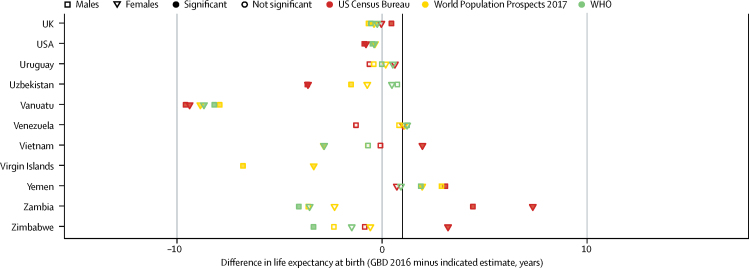
Difference in estimates of life expectancy at birth between GBD 2016 and other sources, 2015 Each point represents the difference between GBD 2016 estimates of life expectancy at birth minus the life expectancy at birth estimated by the indicated sources for each country in 2015, the most recent year with estimates by all sources, by sex. Points are colour-coded by source with squares representing males and triangles representing females. The line at 0 represents no difference between the life expectancy at birth calculated in GBD 2016 and that calculated by the indicated source.

Previous estimates of U5MR from the UN Interagency Group for Child Mortality (IGME) have been broadly similar to those produced by GBD,^94^ and the most recent estimates from IGME are strongly correlated (Pearson correlation 0·982, p<0·0001) with those of GBD 2016 (appendix section 8 p 72). However, country-level disparities in estimation are evident and this results in notable differences in the assessment of progress toward achieving SDG targets.^96^, ^97^ Figure 9 shows substantial differences in life expectancy at birth as assessed for 2015 by the GBD 2016 study compared with estimates from WHO, UNPD, and USCB. For higher SDI countries, particularly those with complete VR systems, the differences are less notable. The WHO estimates of life expectancy differed from GBD estimates by more than 5 years for 28 countries; UNPD estimates differed by more than 5 years from the GBD 2016 estimates in 23 countries for men and 21 countries for women; USCB estimates differed from those of GBD 2016 by more than 5 years in 35 countries for men and 31 countries for women. Such large differences in a summary measure of mortality suggest even more greater differences for age-specific mortality. The USCB and WHO release summary measures such as life expectancy at birth, U5MR, and adult mortality rates, but not for more detailed disaggregation of age groups over a long time series. WHO, UNPD, and USCB do not follow GATHER for their analyses of all-cause mortality, which limits the ability to understand the basis of these differences. The UNPD and USCB predict adult mortality from under-5 mortality in many lower-SDI countries, which might be a contributing factor. The UNPD and USCB use model life table systems based on a set of life tables collected before 1980; there are many reasons to expect that these mortality patterns are not relevant to the current period.^96^, ^97^

### Limitations

Although this study includes many methodological advances, it also has limitations. First, the accuracy of the estimates depends crucially on the available data sources and density of data by time period. For child mortality, at least one year of data was available for all countries. For adults, however, there were 12 countries with no data; in these cases, estimates depend critically on covariates and the ST-GPR statistical model. Additionally, for country-years with input data, data quality, as determined by both sampling and non-sampling errors, varies across locations and over time within the same location. This adds to the uncertainty in comparing the same metric from different locations, even though we have made every effort to systematically propagate uncertainty throughout our estimation process. Second, for many countries with limited or absent VR systems, particularly in sub-Saharan Africa, we use sibling history data to estimate levels and trends in adult mortality. Sibling history data have several known biases.^17^, ^22^, ^98^ In settings outside of sub-Saharan Africa we found no net biases in our estimates based on sibling histories when compared with equivalent estimates derived from other sources of information such as VR systems. Although differences in adoption practices in parts of sub-Saharan Africa create the potential for sibling histories to perform differently than in other settings, Obermeyer and colleagues,^25^ Helleringer and colleagues,^98^ and Masquelier^99^ did not find a consistent direction of bias in sibling history data in these settings. Third, our assessment of mortality depends on the validity of our modelling of HIV/AIDS epidemics, particularly in settings such as in eastern and southern sub-Saharan Africa, which have large generalised epidemics. While there are relatively robust data available on the prevalence of HIV/AIDS from population-based surveys in many of these countries, such data are often available only for selected years, and the data on HIV/AIDS-specific mortality and CD4 progression rates, both on and off ART, are far more scarce. Death rates on ART by CD4 count are also confounded by other indications for ART such as the presence of opportunistic infections.^100^, ^101^, ^102^ All combined, there is a much higher level of uncertainty in the HIV/AIDS-specific mortality estimates than in all-cause mortality estimation and these are used as a key covariate in the estimation of all-cause mortality. Fourth, there is significant challenge in the synthesis of stillbirth mortality across countries, because the definition of stillbirth varies over time and across countries. We include a fixed effect on the definition of stillbirth with a no definition category. Bias adjustment for data from this definition category is done with the adjustment factor used by Blencowe and colleagues.^23^ Although this situation is not ideal, it does help us to generate estimates for countries in central Asia where no definition of stillbirth is provided in the data that we have. Fifth, in this assessment we use the UNPD estimates of population by age, sex, and year in 150 countries. However, in years far from a census, these estimates of population are affected by the UNPD estimates of mortality, which differ from the GBD estimates; where UNPD mortality estimates are lower than those from GBD, population estimates will be larger and GBD mortality might be underestimated. In future assessments, it would be preferable to develop estimates of population that are fully consistent with GBD estimates of age-specific mortality and the GBD 2016 assessment of age-specific fertility. Sixth, estimating the mortality envelope for the 95 years and older age group presents a challenge. Our estimated population in this age group might be biased due to the potential underestimation or overestimation of the number of 79 year olds in the interpolated UNPD population estimates; the estimated mortality rate itself might be biased depending on the population structure in this age group; and availability of empirical data on mortality in this age group may be low. A more rigorous analytical framework of population estimation needs to be applied in the future to improve accuracy. Seventh, we used ST-GPR in our current study to estimate TFR with all available data on TFR from surveys, censuses, and civil registration systems. By using informative covariates including income and education, our current estimates are likely to reflect the level and trends of fertility in periods for which we have sparse data and that are further away from available censuses. However, our current model for TFR is separate from the demographic estimation process of mortality, migration, and population, and it is likely to have induced internal inconsistency among the key components of the demographic balancing equation. Combined with use of the age pattern of fertility from World Population Prospects 2015 revision, our current birth estimates might be biased in data-sparse locations, even though we provide a 95% UI. Eighth, data availability assessed for GBD 2016 peaks in 2013. Fewer data are available for subsequent years because of lags in reporting of vital statistics and lags in the collation, analysis, and publication of household surveys. Estimates for more recent years are increasingly model-dependent. Ninth, we have yet to propagate uncertainty of estimated completeness of VR system into our all-cause mortality estimation process because of constraints in computation. Currently, we only adjust mortality rate from VR systems if the estimated completeness is below 95%. This dichotomous approach, however, could generate rather substantial changes in the age-specific mortality if the estimated completeness is just under the 95% threshold. In the case of South Korea, for example, our estimated completeness is 0·1% below 95%, which results in a 5·4% increase in the age-specific mortality that we have used. Such instability needs to be eliminated in future iterations of GBD. Tenth, migration, both domestic and international, should have a substantial impact on estimated mortality in certain locations. However, our input data on mortality, fertility, and population might not have fully considered the impact of migration. VR systems and disease surveillance systems are likely to omit deaths from migrants, domestic or international. Given the quality and quantity of available data on migration, long-term investment to improve the quality of VR and civil registration systems is essential. Eleventh, our assessment of SDI is based on mean levels of income per capita, educational attainment, and the total fertility rate. We do not take into account the distribution of each of these quantities within each country or location. In future work, it would be desirable to explore distribution-sensitive measures of SDI. Finally, our current study only provides subnational estimates for countries with populations higher than 200 million. Further detailed subnational analysis will be incorporated in future iterations of GBD as necessary data become available.

### Future directions

For this GBD 2016, we used a systematic analysis of fertility data to estimate TFR and annual birth numbers; our assessment has, however, continued to use the UNPD estimates of relative age pattern of fertility by the age of the mother. We plan to explore the empirical evidence in each age group and location for maternal age patterns of fertility. More importantly, for this study we used the UNPD estimates of population by age and sex. These estimates, especially in years that are further away from a high-quality census, are dependent on their estimates of age-specific mortality, fertility in previous years, and migration. GBD estimates of mortality are quite different, and in some locations our fertility estimates were also quite different, leading to potential inconsistencies between UNPD population estimates and the implied GBD estimates of population. For GBD 2017, we plan to develop estimates of age-specific population that are internally consistent with GBD estimates of fertility and mortality.

## Conclusion

Understanding comparative mortality levels in different population subgroups, and how they are changing, has enormous implications for policy. Our results tracking mortality rates in 195 countries and territories over 47 years, suggest that progress is being made in improving survival rates; however, in some populations, that progress has been slow. This slow progress is particularly the case for young adult males, for whom the leading causes of death are largely preventable, but it is also evident at older adult ages where the return on the comprehensive public health and disease control strategies of the past few decades is less evident. Moreover, although massive declines in the risk of child mortality since 1970 have led to substantial gains in life expectancy at birth, they have done little to accelerate the relative convergence of mortality levels in populations, which is now increasingly driven by premature adult death. Our findings also make evident the fact that some of the exemplar countries that were at the vanguard of mortality declines over the last 25 years of the 20th century, particularly in terms of child mortality, have been surpassed in terms of recent successes. In 2016, survival prospects in populations as diverse as Ethiopia, Niger, Portugal, Peru, and the Maldives have increased substantially, and these countries now top the list in terms of the magnitude of overall improvement since 2000. Learning from such successes, making the detection changes in mortality patterns more reliable, and use of that information to more extensively guide health and social policy should accelerate further declines in mortality, particularly in populations where progress remains modest.

Correspondence to: Prof Christopher J L Murray, 2301 5th Avenue, Suite 600, Seattle, WA 98121, USA cjlm@uw.edu

For the **online data visualisation tools** see https://vizhub.healthdata.org/gbd-compare

For the **online source tool** see http://ghdx.healthdata.org

For the **specific sources** see http://ghdx.healthdata.org

For the **online repository of the statistical code for this study** see https://github.com/ihmeuw/ihme-modeling

For the **Global Health Data Exchange** see http://ghdx.healthdata.org/node/308322
