# Department of Error

**DOI:** 10.1016/S0140-6736(17)32645-4

**Published:** 2017-10-28

*GBD 2016 Mortality Collaborators. Global, regional, and national under-5 mortality, adult mortality, age-specific mortality, and life expectancy, 1970–2016: a systematic analysis for the Global Burden of Disease Study 2016.* Lancet *2017; **390**: 1084–150.* The full-text version of this Article has been updated so that the list of authors is displayed in the correct order, in line with the pdf version, rather than in alphabetical order. This correction has been made to the online version as of Oct 12, 2017.

