# Supplementary material for: Global, regional, and national under-5 mortality, adult mortality, age-specific mortality, and life expectancy, 1970–2016: a systematic analysis for the Global Burden of Disease Study 2016
Source: Lancet. 2017 Sep 16;390(10100):1084–150. doi: 10.1016/S0140-6736(17)31833-0 (PMC5605514; doi:10.1016/S0140-6736(17)31833-0)
Supplement: Supplementary appendix [file mmc1.pdf]

THE LANCET

Supplementary appendix

This appendix formed part of the original submission and has been peer reviewed. We post it as supplied by the authors.

Supplement to: GBD 2016 Mortality Collaborators. Global, regional, and national under-5 mortality, adult mortality, age-specific mortality, and life expectancy, 1970–2016: a systematic analysis for the Global Burden of Disease Study 2016. *Lancet* 2017; **390:** 1084–150.

Appendix to “Global, regional, and national life expectancy and all-cause mortality, 1970-2016: a systematic review for the Global Burden of Disease Study 2016”

This appendix provides further methodological detail, supplemental figures, and more detailed results for “Global, regional, and national life expectancy and all-cause mortality, 1970-2016: a systematic review for the Global Burden of Disease Study 2016.” This appendix is organized into sections that follow the structure of the main paper.

Preamble

This appendix provides further methodological detail and more detailed results for “Global, regional, and national life expectancy and all-cause mortality, 1970-2016: a systematic review for the Global Burden of Disease Study 2016.” This study complies with the Guidelines for Accurate and Transparent Health Estimates Reporting (GATHER) recommendations. It includes detailed tables and information on data in an effort to maximize transparency in our estimation processes and provide a comprehensive description of analytical steps. We intend this appendix to be a living document, to be updated with each iteration of the Global Burden of Disease Study.

Table of Contents

Author Contributions......i
Section 1: GBD Overview......4
1.1 Geographic locations of analysis...... 4
1.2 Time period of analysis......4
1.3 Statement of GATHER compliance......4
1.4 List of abbreviations......4
1.5 GBD results overview......5
1.6 Data input sources overview......6
1.7 Funding sources......6
Section 2: GBD 2016 All-Cause Mortality and HIV Estimation Process......7
2.1 Overview......7
2.2 Child mortality......7
2.3 Stillbirth estimation......21
2.4 Adult mortality......24
2.5 Model life table system...... 38
2.6 HIV/AIDS estimation...... 46
2.7 Age-specific mortality estimation for all GBD age-groups: with and without HIV......51
Section 3: Fertility and Birth Estimation Methods......53
3.1 Input data......53
3.2 Modeling strategy......53
Section 4: Socio-demographic Index (SDI) Analysis......55
4.1 Case definition......55
4.2 Modeling strategy......55
4.3 Age-sex-specific relationships between SDI and death rates......56
4.4 SDI quintiles......57
Section 5: Fatal Discontinuities Estimation......58
5.1 Input data......58
5.2 Modeling strategy......60
Section 6: Additional Methods Information......61
6.1 Creation of the GBD world population standard......61
6.2 Generating population estimates in older age groups......61
6.3 Special population definitions......61
6.4 Estimating correlation......62
6.5 Calculating annualised rates of change......62
6.6 Causes of death data star rating calculation......62
Section 7: References.......65
Section 8: Figures and Tables......68

List of Appendix Figures and Tables

Appendix Figure 1. Components of GBD 2016 all-cause mortality and HIV estimation and their relations

Appendix Figure 2. Sibling history correction for zero-survivorship, by sex

Appendix Figure 3. Comparison of life expectancy at birth by location in GBD 2015 vs GBD 2016

Appendix Figure 4. Comparison of estimates of under-5 mortality from GBD 2016 and IGME 2015 for all countries included in both analyses, 2015

Appendix Figure 5. Ratio of stillbirth rates to neonatal mortality rates by GBD super region, 1970-2016

Appendix Figure 6. Comparison of stillbirth rates from GBD 2016 and SEIG for all countries included in both analyses, 2015

Appendix Figure 7. Ratio of estimated number of live births by location between GBD 2016 and UN Population Division, 1970, 1980, 1990, 2000, 2016

Appendix Figure 8. Estimated completeness of death registration, 1970-1989

Appendix Table 1. GATHER checklist of information that should be included in reports of global health estimates, with description of compliance and location of information for GBD 2016 mortality capstone

Appendix Table 2. Distribution of empirical life tables by GBD super-region and decade, 1950-2016

Appendix Table 3. GBD 2016 geography hierarchy with levels

Appendix Table 4. Socio-demographic Index groupings by geography, based on 2016 values

Appendix Table 5. Socio-demographic Index values by location and year

Appendix Table 6. GBD world population age standard

Appendix Table 7. Number of all-cause mortality data sources by type and location, 1950-2016

Appendix Table 8. Number of all-cause mortality data sources by type and year, 1950-2016

Appendix Table 9. Life expectancy at birth in 2015 from UN, WHO, census, and GBD 2016

Appendix Table 10. Under-5 mortality reference sources by source date and location, 1950-2016

Appendix Table 11, A-C. Under-5 mortality rates by location, sex, and year for 1970, 1975, 1980, 1985, 1990, 1995, 2000, 2005, 2010, 2016

Appendix Table 12, A-C. Under-5 death numbers by location, sex, and year for 1970, 1975, 1980, 1985, 1990, 1995, 2000, 2005, 2010, 2016

Appendix Table 13, A-C. Estimates of life expectancy at birth by location, sex, and year for 1970, 1975, 1980, 1985, 1990, 1995, 2000, 2005, 2010, 2016

Appendix Table 14, A-C. Estimates of life expectancy at age 65 by location, sex, and year for 1970, 1975, 1980, 1985, 1990, 1995, 2000, 2005, 2010, 2016

Appendix Table 15, A-C. Live births (in thousands) by location, sex, and year for 1970, 1975, 1980, 1985, 1990, 1995, 2000, 2005, 2010, 2016

Appendix Table 16, A-C. Under-5 population (in thousands) by location, sex, and year for 1970, 1975, 1980, 1985, 1990, 1995, 2000, 2005, 2010, 2016

Appendix Table 17, A-D. Stillbirth rates (per 1,000 live births) by location, 1970-1979, 1980-1995, 1996-2005, 2006-2016

Appendix Table 18, A-D. Stillbirths (in thousands) by location, for 1980-1995, 1996-2005, 2006-2016

Appendix Table 19, A-C. Age-standardised mortality rates by location, sex, and year for 1970, 1975, 1980, 1985, 1990, 1995, 2000, 2005, 2010, 2016

Authors' Contributions

Managing the estimation process

Ryan Barber, Blair Bumgarner, Austin Carter, Dirk Douwes-Schultz, Tahvi Frank, Maya Fraser, Joseph Friedman, Emmanuela Gakidou, Fei He, Xie Rachel Kulikoff, Michael Kutz, Christopher Murray, Mohsen Naghavi, Grant Nguyen, Amber Sligar, Vinay Srinivasan, Alexander Thompson, and Haidong Wang.

Writing the first draft of the manuscript

Laurie B Marczak, Christopher J L Murray, and Haidong Wang.

Providing data or critical feedback on data sources

Cristiana Abbafati, Semaw Ferede Abera, Niveen M E Abu -Rmeileh, Laith J Abu-Raddad, Isaac Akinkunmi Adedeji, Rufus Adesoji Adesoji Adedoyin, Ifedayo Morayo O Adetifa, Olatunji Adetokunboh, Ashkan Afshin, Rakesh Aggarwal, Sutapa Agrawal, Aliasghar Ahmad Kiadaliri, Muktar Beshir Ahmed, Amani Nidhal Aichour, Ibtihel Aichour, Miloud Taki Eddine Aichour, Tomi F Akinyemiju, Nadia Akseer, Fares Alahdab, Khurshid Alam, Deena Alasfoor, Robert William Aldridge, Ayman Al-Eyadhy, Samia Alhabib, Raghib Ali, Ala'a Alkerwi, François Alla, Peter Allebeck, Ubai Alsharif, Khalid A Altirkawi, Azmeraw T Amare, Erfan Amini, Walid Ammar, Nahla Anber, Catalina Liliana Andrei, Hossein Ansari, Carl Abelardo T Antonio, Palwasha Anwari, Al Artaman, Krishna Kumar Aryal, Hamid Asayesh, Solomon Weldegebreal Asgedom, Rana Jawad Ashgar, Ashish Awasthi, Kalpana Balakrishnan, Aleksandra Barac, Miguel A Barboza, Till Bärnighausen, Simon Barquera, Lars Barregard, Lope H Barrero, Neeraj Bedi, Yannick Béjot, Bayu Begashaw Bekele, Derrick A Bennett, James R Bennett, Isabela M Bensenor, Mircea Beuran, Addisu Shunu Beyene, Anil Bhansali, Boris Bikbov, Charles Birungi, Donal Bisanzio, Dube Jara Boneya, Rupert R A Boourne, Lemma Negesa Bulto Bulto, Lucero Cahuana-Hurtado, Mate Car, Juan Jesus Carrero, Carlos A Castañeda-Orjuela, Franz F Castro, Ferrán Catalá-López, Honglei Chen, Peggy Pei-Chia Pei-Chia Chiang, Mirriam Chibalabala, Abdulaal A Chitheer, Jee-Young J Choi, Michael H Criqui, Lalit Dandona, Rakhi Dandona, Paul I Dargan, José das Neves, Gail Davey, Kebede Deribe, Amare Deribew, Subhojit Dey, Samath D Dharmaratne, Cesar Diaz-Torne, Priyanka Dixit, Shirin Djalalinia, Christl Ann Donnelly, Dirk Douwes-Schultz, Tim R Driscoll, Manisha Dubey, Laxmi Kant Dwivedi, Charbel El Bcheraoui, Sergey Petrovich Ermakov, Babak Eshrati, Sharareh Eskandarieh, Alireza Esteghamati, Fanuel Belayneh Bekele Fanuel, Andre Faro, Farshad Farzadfar, Valery L Feigin, Seyed-Mohammad Fereshtehnejad, João C Fernandes, Tesfaye R Regassa Feyissa, Irina Filip, Florian Fischer, Richard C Franklin, Nancy Fullman, Thomas Fürst, João M Furtado, Neal D Futran, Ketevan Gambashidze, Fortuné Gbètoho Gankpé, Gebremedhin Berhe Gebregergs, Tsegaye Tewelde Gebrehiwot, Johanna M Geleijnse, Hailay Abrha Gesesew, Peter Gething, Katherine B Gibney, Beatriz Gomez, Philimon Gona, Harish Cahnder Gugnani, Prakash Gupta, Nima Hafezi-Nejad, Yara A Halasa-Rappel, Mitiku Teshome Hambisa, Graeme J Hankey, Hilda L Harb, Habtamu Abera Hareri, Josep Maria Haro, Mohammad Sadegh Hassanvand, Rasmus Havmoeller, Roderick J Hay, Simon I Hay, Ileana Beatriz Heredia-Pi, Hans W Hoek, Nobuyuki Horita, H Dean Hosgood, Damian G Hoy, Aung Soe Htet, Guoqing Hu, John J Huang, Kim Moesgaard Iburg, Ehimario Uche Igumbor, Bogdan Vasile Ileanu, Manami Inoue, Asnake Ararsa Irenso, Kathryn H Jacobsen, Mihajlo B Jakovljevic, Mehdi Javanbakht, Panniyammakal Jeemon, Paul N Jensen, Sarah Charlotte Johnson, Jost B Jonas, Mikk Jürisson, Zubair Kabir, Rajendra Kadel, Ritul Kamal, Haidong Kan, André Karch, Corine Kakizi Karema, Seyed M Karimi, Amir Kasaeian, Nicholas J Kassebaum, Srinivasa Vittal Katikireddi, Anil Kaul, Norito Kawakami, Konstantin Kazanjan, Andre Keren, Maia Kereselidze,

Chandrasekharan Nair Kesavachandran, Yousef Saleh Khader, Ibrahim A Khalil, Abdullah Tawfih Abdullah Khoja, Christian Kieling, Yun Jin Kim, Ruth W Kimokoti, Yohannes Kinfu, Mika Kivimaki, Yoshihiro Kokubo, Soewarta Kosen, Parvaiz A Koul, Michael Kravchenko, Kristopher J Krohn, Barthelemy Kuate Defo, Burcu Kucuk Bicer, G Anil Kumar, Pushpendra Kumar, Carl Lachat, Anton Carl Jonas Lager, Dharmesh Kumar Lal, Nkurunziza Lambert, Qing Lan, Anders Larsson, James Leigh, Janni Leung, Ricky Leung, Miriam Levi, Misgan Legesse Liben, Shiwei Liu, Giancarlo Logroscino, Alan D Lopez, Stefan Lorkowski, Stefan Ma, Carlos Magis-Rodriguez, Marek Majdan, Azeem Majeed, Reza Malekzadeh, Lorenzo G Mantovani, Tsegahun Manyazewal, Guy B Marks, Mohsen Mazidi, Colm McAlinden, John J McGrath, Suresh Mehata, Tefera Chane Mekonnen, Kidanu Gebremariam Meles, Ziad A Memish, Walter Mendoza, Melkamu Merid Mengesha, Desalegn Tadese Mengistu, Bereket Gebremichael Menota, Atte Meretoja, Tuomo J Meretoja, Haftay Berhane Mezgebe, Ted R Miller, Shiva Raj Mishra, Shafiu Mohammed, Sanjay K Mohanty, Ali H Mokdad, Mariam Molokhia, Lorenzo Monasta, Meghan D Mooney, Ami R Moore, Maziar Moradi-Lakeh, Ilais Moreno Velasquez, Rintaro Mori, Ulrich O Mueller, Christopher J L Murray, Kamarul Imran Musa, Jean B Nachega, Gabriele Nagel, Mohsen Naghavi, Bruno Ramos Nascimento, Ionut Negoi, Marika Nomura, Bo Norrving, Luke Nyakarahuka, Felix Akpojene Ogbo, Olanrewaju Oladimeji, Andrew Toyin Olagunju, Bolajoko Olubukunola Olusanya, Jacob Olusegun Olusanya, Alberto Ortiz, Mayowa O Owolabi, Mahesh PA, Smita Pakhale, Adrian Pana, Songhomitra Panda-Jonas, Scott Patten, Deepak Paudel, David M Pereira, Fernando Perez-Ruiz, Norberto Perico, William Arthur Petri, Frédéric B Piel, David M Pigott, Dietrich Plass, Suzanne Polinder, Svetlana Popova, Farshad Pourmalek, Narayan Prasad, Mostafa Qorbani, Rynaz H S Rabiee, Amir Radfar, Anwar Rafay, Afarin Rahimi-Movaghar, Vafa Rahimi-Movaghar, Mahfuzar Rahman, Rajesh Kumar Rai, Sasa Rajsic, Usha Ram, Chhabi Lal Ranabhat, Salman Rawaf, Jürgen Rehm, Giuseppe Remuzzi, Mohammad Sadegh Rezai, Antonio Luiz Ribeiro, Luca Ronfani, Gholamreza Roshandel, Gregory A Roth, Dietrich Rothenbacher, Ambuj Roy, George Mugambage Ruhago, Soheil Saadat, Nafis Sadat, Saeid Safiri, Mohammad Ali Sahraian, Payman Salamati, Abdallah M Samy, Itamar S Santos, Milena Santric Milicevic, Benn Sartorius, Maheswar Satpathy, Monika Sawhney, Sonia Saxena, Mete I Saylan, Falk Schwendicke, Soraya Seedat, Sadaf G Sepanlou, Edson E Servan-Mori, Saeid Shahraz, Masood Ali Ali Shaikh, Marina Shakhnazarova, Morteza Shamsizadeh, Sheikh Mohammed Shariful Islam, Jayendra Sharma, Jiabin Shen, Peilin Shi, Kenji Shibuya, Mark G Shrime, Diego Augusto Santos Silva, Abhishek Singh, Jasvinder Singh, Virendra Singh, Badr H A Sobaih, Eugene Sobngwi, Chandrashekhar T Sreeramareddy, Vasiliki Stathopoulou, Heidi Stöckl, Mark Andrew Stokes, Muawiyyah Babale Sufiyan, Rizwan Abdulkader Suliankatchi, Patrick J Sur, Bryan L Sykes, Cassandra E I Szoeke, Rafael Tabarés-Seisdedos, David Tanne, Musharaf Tarajia, Mohammad Tavakkoli, Nuno Taveira, Girma Temam Shifa, Mohamad-Hani Temsah, Abdullah Sulieman Terkawi, Andrew Theis, Amanda G Thrift, Ruoyan Tobe-Gai, Marcello Tonelli, Bach Xuan Tran, Thomas Truelsen, Kingsley Nnanna Ukwaja, Eduardo Undurraga, Job FM van Boven, Yurii Y Varakin, Santosh Varughese, Tommi Vasankari, Ana Maria Nogales Vasconcelos, Narayanaswamy Venketasubramanian, Ramesh Vidavalur, Francesco S Violante, Vasiliy Victorovich Vlassov, Stein Emil Vollset, Tolassa Wakayo, Haidong Wang, Yuan-Pang Wang, Elisabete Weiderpass, Robert G Weintraub, Andrea Werdecker, Tissa Wijeratne, Charles Shey Wiysonge, Charles D A Wolfe, Abdulhalik Workicho, Denis Xavier, Gelin Xu, Bereket Yakob, Lijing L Yan, Pengpeng Ye, Hassen Hamid Yimam, Biruck Desalegn Yirsaw, Naohiro Yonemoto, Marcel Yotebieng, Mustafa Z Younis, Zoubida Zaidi, Anthony Lin Zhang, Xueying Zhang, Sanjay Zodpey, and Liesl Joanna Zuhlke.

Developing methods or computational machinery

Ashkan Afshin, Dina Nur Anggraini Ningrum, Megha Arora, Solomon Weldegebreal Asgedom, James R Bennett, Stan Biryukov, Donal Bisanzio, Dube Jara Boneya, Lemma Negesa Bulto Bulto, Austin Carter,

Paul Dargan, Charbel El Bcheraoui, Babak Eshrati, Kyle J Foreman, Tahvi Frank, Maya Fraser, Joseph Friedman, Joseph J Frostad, Emmanuela Gakidou, Nicholas Graetz, Hassen Hamid Yimam, Xie Rachel Kulikoff, Michael Kutz, Ali H Mokdad, Christopher J L Murray, Mohsen Naghavi, Grant Nguyen, Aaron Osgood-Zimmerman, Robert Reiner, Enrico Rubagotti, Maheswar Satpathy, Naris Silpakit, David L Smith, Vinay Srinivasan, Alexander H Thompson, and Haidong Wang.

Applying analytical methods to produce estimates

Ashkan Afshin, Muktar Beshir Ahmed, Fares Alahdab, Azmeraw T Amare, Nahla Anber, Hossein Ansari, Mustafa Geleto Ansha, Solomon Weldegebreal Asgedom, Ryan M Barber, Adugnaw Berhane, Donal Bisanzio, Dube Jara Boneya, Lemma Negesa Bulto Bulto, Austin Carter, Elizabeth A Cromwell, Dirk Douwes-Schultz, Charbel El Bcheraoui, Aman Yesuf Endries, Babak Eshrati, Tahvi Frank, Maya Fraser, Joseph Friedman, Joseph J Frostad, Fei He, Bogdan Vasile Ileanu, Caleb M S Irvine, Yun Jin Kim, Xie Rachel Kulikoff, Qing Lan, Janni Leung, Misgan Kegesse Liben, Joseph Mikesell, Ali H Mokdad, Sarah K Mollenkopf, Christopher J L Murray, Mohsen Naghavi, Grant Nguyen, Dina Nur Anggraini Ningrum, Felix Akopjene Ogbo, Olanrewaju Oladimeji, Sarah E Ray, Robert Reiner, Mohammad Sadegh Rezai, Gregory A Roth, Enrico Rubagotti, Maheswar Satpathy, Vinay Srinivasan, Alexander H Thompson, Kingsley Nnanna Ukwaja, Tolassa Wakayo, Haidong Wang, Tissa Wijeratne, Hassen Hamid Yimam, and Mustafa Z Younis.

Providing critical feedback on methods or results

Amanuel Alemu Abajobir, Kalkidan Hassen Abate, Cristiana Abbafati, Kaja M Abbas, Semaw Ferede Abera, Haftom Niguse Abraha, Niveen M E Abu-Rmeileh, Isaac Akinkunmi Adedeji, Rufus Adesoji Adedoyin, Ifedayo Morayo O Adetifa, Olatunji Adetokunboh, Ashkan Afshin, Anurag Agrawal, Sutapa Agrawal, Aliasghar Ahmad Kiadaliri, Muktar Beshir Ahmed, Amani Nidhal Aichour, Ibtihel Aichour, Miloud Taki Eddine Aichour, Ali Shafqat Akanda, Tomi F Akinyemiju, Nadia Akseer, Faris Hasan Al Lami, Samer Alabed, Fares Alahdab, Ziyad Al-Aly, Khurshid Alam, Noore Alam, Deena Alasfoor, Robert William Aldridge, Kefyalew Addis Alene, Raghib Ali, Reza Alizadeh-Navaei, Shalini D Allam, Peter Allebeck, Ubai Alsharif, Khalid A Altirkawi, Nelson Alvis-Guzman, Azmeraw T Amare, Erfan Amini, Walid Ammar, Yaw Ampem Amoako, Catalina Liliana Andrei, Sofia Androudi, Mustafa Geleto Ansha, Carl Abelardo T Antonio, Palwasha Anwari, Johan Ärnlöv, Megha Arora, Al Artaman, Krishna Kumar Aryal, Hamid Asayesh, Solomon Weldegebreal Asgedom, Rana Jawad Asghar, Reza Assadi, Tesfay Mehari Atey, Sachin R Atre, Leticia Avila-Burgos, Euripide Frinel G Arthur Avokpaho, Ashish Awasthi, Beatriz Paulina Ayala Quintanilla, Tesleem Kayode Babalola, Umar Bacha, Alaa Badawi, Aleksandra Barac, Suzanne L Barker-Collo, Till Bärnighausen, Simon Barquera, Lars Barregard, Lope H Barrero, Bernhard T Baune, Shahrzad Bazargan-Hejazi, Neeraj Bedi, Ettore Beghi, Bayu Begashaw Bekele, Michelle L Bell, Derrick A Bennett, Isabela M Bensenor, Adugnaw Berhane, Derbew Fikadu Berhe, Eduardo Bernabe, Mircea Beuran, Addisu Shunu Shunu Beyene, Neeraj Bhala, Zulfiqar A Bhutta, Boris Bikbov, Charles Birungi, Donal Bisanzio, Christopher D Blosser, Dube Jara Boneya, Rupert R A Boourne, Nicholas J K Breitborde, Hermann Brenner, Gene Bukhman, Lemma Negesa Bulto Bulto, Zahid A Butt, Leah E Cahill, Ismael Ricardo Campos-Nonato, Mate Car, Rosario Cárdenas, David O Carpenter, Austin Carter, Carlos A Castañeda-Orjuela, Jacqueline Castillo-Rivas, Ferrán Catalá-López, Piggy Pei-Chia Chiang, Vesper Hichilombwe Chisumpa, Jee-Young J Choi, Hanne Christensen, Liliana G Ciobanu, Aaron J Cohen, Samantha M Colquhoun, Michael H Criqui, Elizabeth A Cromwell, John A Crump, Lalit Dandona, Rakhi Dandona, José das Neves, Kairat Davletov, Barbora de Courten, Diego De Leo, Louisa Degenhardt, Kebede Deribe, Amare Deribew, Subhojit Dey, Samath D Dharmaratne, Shirin Djalalinia, Huyen Phuc Do, David T Doku, Kadine Priscila Bender dos Santos, Dirk Douwes-Schultz, Tim R Driscoll, Manisha Dubey, Charbel El

Bcheraoui, Christian Lycke Lyche Ellingsen, Ahdalima Enayati, Aman Yesuf Endries, Setegn Eshetie, Babak Eshrati, Sharareh Eskandarieh, Alireza Esteghamati, Fanuel Belayneh Bekele Fanuel, André Faro, Farshad Farzadfar, Seyed-Mohammad Fereshtehnejad, Joao C Fernandes, Tesfaye Regassa Feyissa, Irina Filip, Florian Fischer, Nataliya Foigt, Kyle J Foreman, Richard C Franklin, Nancy Fullman, Thomas Fürst, João M Furtado, Neal D Futran, Emmanuela Gakidou, Ketevan Gambashidze, Amiran Gamkrelidze, Alberto L García-Basteiro, Gebremedhin Berhe Gebregergs, Tsegaye Tewelde Gebrehiwot, Mengistu Welday Gebremichael, Hailay Abrha Gesesew, Peter W Gething, Katherine B Gibney, Richard F Gillum, Bedilu Weji Girma, Giorgia Giussani, Philimon N Gona, Sameer Vali Gopalani, Alessandra Carvalho Goulart, Harish Chander Gugnani, Prakash C Gupta, Rajeev Gupta, Tanush Gupta, Vipin Gupta, Nima Hafezi-Nejad, Hassan Haghparast-Bidgoli, Alex Hakuzimana, Randah Ribhi Hamadeh, Mitiku Teshome Hambisa, Samer Hamidi, Yuantao Hao, Hilda L Harb, Habtamu Abera Hareri, Josep Maria Haro, Rasmus Havmoeller, Roderick Hay, Simon I Hay, Fei He, Ileana Beatriz Heredia-Pi, Nobuyuki Horita, H Dean Hosgood, Sorin Hostiuc, Damian G Hoy, Mohamed Hsairi, Aung Soe Htet, Guoqing Hu, Hsiang Huang, John J Huang, Kim Moesgaard Iburg, Ehimario Uche Igumbor, Manami Inoue, Asnake Ararsa Irenso, Kathryn H Jacobsen, Nader Jahanmehr, Mihajlo B Jakovljevic, Mehdi Javanbakht, Panniyammakal Jeemon, Paul N Jensen, Vivekanand Jha, Denny John, Oommen John, Jost B Jonas, Mikk Jürisson, Rajendra Kadel, Amaha Kahsay, Yogeshwar Kalkonde, Ritul Kamal, André Karch, Seyed M Karimi, Amir Kasaeian, Nigussie Assefa Kassaw, Nicholas J Kassebaum, Anshul Kastor, Srinivasa Vittal Katikireddi, Norito Kawakami, Konstantin Kazanjan, Peter Njenga Keiyoro, Andre Keren, Maia Kereselidze, Chandrasekharan Nair Kesavachandran, Ezra Belay Ketema, Yousef Saleh Khader, Young-Ho Khang, Sahil Khera, Mohammad Hossein Khosravi, Getiye Dejenu Kibret, Daniel Kim, Pauline Kim, Yun Jin Kim, Ruth W Kimokoti, Yohannes Kinfu, Sami Kishawi, Katarzyna A Kissimova-Skarbek, Niranjan Kissoon, Mika Kivimaki, Ann Kristin Knudsen, Jacek A Kopec, Parvaiz A Koul, Ai Koyanagi, Michael Kravchenko, Kristopher J Krohn, Barthelemy Kuate Defo, Ernst J Kuipers, G Anil Kumar, Pushpendra Kumar, Michael Kutz, Anton Carl Jonas Lager, Dharmesh Kumar Lal, Nkurunziza Lambert, Qing Lan, Van C Lansingh, Anders Larsson, Dennis Odai Laryea, Pablo M Lavados, Avula Laxmaiah, Paul H Lee, James Leigh, Janni Leung, Miriam Levi, Yongmei Li, Misgan Legesse Liben, Shai Linn, Rakesh Lodha, Alan D Lopez, Scott A Lorch, Stefan Lorkowski, Paulo A Lotufo, Ronan A Lyons, Erlyn Rachelle King Macarayan, Mahdi Mahdavi, Marek Majdan, Azeem Majeed, Reza Malekzadeh, Rajesh Malhotra, Deborah Carvalho Malta, Tsegahun Manyazewal, Chabila C Mapoma, Laurie Marczak, Guy B Marks, Jose Martinez-Raga, Pallab K Maulik, Mohsen Mazidi, Colm McAlinden, Stephen Theodore McGarvey, Martin McKee, Kala M' Mehta, Toni Meier, Tefera Chane Mekonnen, Kidanu Gebremariam Meles, Peter Memiah, Walter Mendoza, Melkamu Merid Mengesha, Mubarek Abera Mengistie, Desalegn Tadese Mengistu, Bereket Gebremichael Menota, George A Mensah, Atte Meretoja, Tuomo J Meretoja, Haftay Berhane Mezgebe, Joseph Mikesell, Ted R Miller, Shawn Minnig, Mojde Mirarefin, Erkin M Mirrakhimov, Shiva Raj Mishra, Karzan Abdulmuhsin Mohammad, Alireza Mohammadi, Kedir Endris Mohammed, Shafiu Mohammed, Ali H Mokdad, Sarah K Mollenkopf, Mariam Molokhia, Lorenzo G Monasta, Marcella Montico, Ami R Moore, Paula Moraga, Lidia Morawska, Kalayu Birhane Mruts, Ulrich O Mueller, Kate Muller, Christopher J L Murray, Gudlavalleti Venkata Satyanarayana Murthy, Srinivas Murthy, Kamarul Imran Musa, Jean B Nachega, Chie Nagata, Gabriele Nagel, Mohsen Naghavi, Vinay Nangia, Bruno Ramos Nascimento, Ionut Negoi, Cuong Tat Nguyen, Grant Nguyen, Quyen Le Nguyen, Trang Huyen Nguyen, Dina Nur Anggraini Ningrum, Vuong Minh Nong, Ole F Norheim, Bo Norrving, Jean Jacques N Noubiap, Felix Akpojene Ogbo, Anselm Okoro, Olanrewaju Oladimeji, Andrew Toyin Olagunju, Bolajoko Olubukunola Olusanya, Jacob Olusegun Olusanya, Eyal Oren, Alberto Ortiz, Erika Ota, Mayowa O

Owolabi, Mahesh PA, Smita Pakhale, Adrian Pana, Basant Kumar Panda, Songhomitra Panda-Jonas, Eun-Kee Park, George C Patton, Deepak Paudel, David M Pereira, Fernando Perez-Ruiz, Norberto Perico, Aslam Pervaiz, Konrad Pesudovs, Carrie Beth Peterson, Michael Robert Phillips, Frédéric B Piel, David M Pigott, Dietrich Plass, Suzanne Polinder, Maarten J Postma, Richie G Poulton, Farshad Pourmalek, Narayan Prasad, Mostafa Qorbani, Amir Radfar, Afarin Rahimi-Movaghar, Vafa Rahimi-Movaghar, Mahfuzar Rahman, Mohammad Hifz Ur Rahman, Rajesh Kumar Rai, Sasa Rajsic, Usha Ram, Chhabi Lal Lal Ranabhat, Salman Rawaf, Sarah E Ray, Maria Albertina Santiago Rego, Jürgen Rehm, Robert Reiner, Giuseppe Remuzzi, Andre M N Renzaho, Serge Resnikoff, Satar Rezaei, Mohammad Sadegh Rezai, Antonio L Ribeiro, Luca Ronfani, Gholamreza Roshandel, Gregory A Roth, Dietrich Rothenbacher, Ambuj Roy, George Mugambage Ruhago, Yogesh Damodar Sabde, Perminder S Sachdev, Mahdi Safdarian, Saeid Safiri, Rajesh Sagar, Mohammad Ali Sahraian, Payman Salamati, Joshua A Salomon, Abdallah M. M Samy, Maria Dolores Sanchez-Niño, Itamar S Santos, Milena M Santric Milicevic, Rodrigo Sarmiento-Suarez, Benn Sartorius, Maheswar Satpathy, Monika Sawhney, Sonia Saxena, Mete I Saylan, Ione J C Schneider, Aletta E Schutte, David C Schwebel, Falk Schwendicke, Abdulbasit Musa Seid, Sadaf G Sepanlou, Amira Shaheen, Saeid Shahraz, Masood Ali Ali Shaikh, Marina Shakhnazarova, Mansour Shamsipour, Morteza Shamsizadeh, Sheikh Mohammed Shariful Islam, Jayendra Sharma, Rajesh Sharma, Jun She, Jiabin Shen, Kenji Shibuya, Mika Shigematsu, Rahman Shiri, Ivy Shiue, Mark G Shrime, Inga Dora Sigfusdottir, Donald H Silberberg, Diego Augusto Santos Silva, Dayane Gabriele Alves Silveira, Jasvinder A Singh, Dhirendra Narain Sinha, Badr H A Sobaih, Eugene Sobngwi, Samir Soneji, Chandrashekhar T Sreeramareddy, Nicholas Steel, Mark Andrew Stokes, Mark Strong, Lela Sturua, Muawiyyah Babale Sufiyan, Rizwan Abdulkader Suliankatchi, Bruno F Sunguya, Patrick J Sur, Bryan L Sykes, Cassandra E I Szoeke, Rafael Tabarés-Seisdedos, Fentaw Tadese, Nikhil Tandon, Nuno Taveira, Tesfalidet Tekelab, Girma Temam Shifa, Mohamad-Hani Temsah, Abdullah Sulieman Terkawi, Cheru Leshargie Tesema, Belay Tessema, Alexander H Thompson, Alan J Thomson, Tenaw Yimer Tiruye, Ruoyan Tobe-Gai, Marcello Tonelli, Roman Topor-Madry, Miguel Tortajada-Girbés, Bach Xuan Tran, Thomas Truelsen, Kald Beshir Tuem, Emin Murat Tuzcu, Stefanos Tyrovolas, Kingsley Nnanna Ukwaja, Eduardo A Undurraga, Olalekan A Uthman, Benjamin S C Uzochukwu, Job F M van Boven, Santosh Varughese, Tommi Vasankari, Ana Maria Nogales Vasconcelos, Narayanaswamy Venketasubramanian, Ramesh Vidavalur, Francesco S Violante, Abhishek Vishnu, Sergey K Vladimirov, Vasiliy Victorovich Vlassov, Stein Emil Vollset, Theo Vos, Tolassa Wakayo, Haidong Wang, Yuan-Pang Wang, Scitt Weichenthal, Elisabete Weiderpass, Robert G Weintraub, Andrea Werdecker, Joshua Wesana, Tissa Wijeratne, Belete Getahun Woldeyes, Charles D A Wolfe, Abdulhalik Workicho, Denis Xavier, Gelin Xu, Mohsen Yaghoubi, Bereket Yakob, Lijing L Yan, Mehdi Yaseri, Hassen Hamid Yimam, Paul Yip, Biruck Desalegn Yirsaw, Naohiro Yonemoto, Marcel Yotebieng, Mustafa Z Younis, Zoubida Zaidi, Maysaa El Sayed Zaki, Zerihun Menlkalew Zenebe, Taddese Alemu Zerfu, Anthony Lin Zhang, Sanjay Zodpey, and Liesl Joanna Zuhlke.

Drafting the work or revising is critically for important intellectual content

Amanuel Alemu Abajobir, Kalkidan Hassen Abate, Cristiana Abbafati, Kaja M Abbas, Foad Abd-Allah, Semaw Ferede Abera, Isaac Akinkunmi Adedeji, Ifedayo Morayo O Adetifa, Olatunji Adetokunboh, Ashkan Afshin, Sutapa Agrawal, Aliasghar Ahmad Kiadaliri, Muktar Beshir Ahmed, Tomi F Akinyemiju, Fares Alahdab, Khurshid Alam, Noore Alam, Raghib Ali, Ala'a Alkerwi, Shalini D Allam, Peter Allebeck, Khalid A Altirkawi, Nelson Alvis-Guzman, Azmeraw T Amare, Emmanuel A Ameh, Erfan Amini, Walid Ammar, Yaw A Ampem Amoako, Nahla Anber, Sofia Androudi, Hossein Ansari, Mustafa Geleto Ansha, Palwasha Anwari, Johan Ärnlöv, Hamid Asayesh, Solomon Weldegebreal Asgedom, Reza Assadi, Ashagre Molla Assaye, Tesfay Mehari Atey, Leticia Avila-Burgos, Ashish Awasthi, Alaa Badawi, Aleksandra Barac,

Suzanne L Barker-Collo, Till Bärnighausen, Lope H Barrero, Bernhard T Baune, Neeraj Bedi, Ettore Beghi, Michelle L Bell, Isabela M Bensenor, Adugnaw Berhane, Eduardo Bernabé, Mircea Beuran, Addisu Shunu Beyene, Neeraj Bhala, Donal Bisanzio, Dube Jara Boneya, Hermann Brenner, Lemma Negesa Bulto Bulto, Zahid A Butt, Lucero Cahuana-Hurtado, Josip Car, Juan Jesus Carrero, Austin Carter, Carlos A Castañeda-Orjuela, Jacqueline Castillo-Rivas, Franz F Castro, Ferrán Catalá-López, Aaron J Cohen, Michael H Criqui, John A Crump, José das Neves, Gail Davey, Dragos V Davitoiu, Barbora de Courten, Louisa Degenhardt, Selina Deiparine, Robert P Dellavalle, Kebede Deribe, Don C Des Jarlais, Subhojit Dey, Samath D Dharmaratne, Huyen Phuc Do, Tim R Driscoll, Manisha Dubey, Bruce Bartholow Duncan, Hedyeh Ebrahimi, Charbel El Bcheraoui, Aman Yesuf Endries, Babak Eshrati, Sharareh Eskandarieh, Alireza Esteghamati, André Faro, Seyed-Mohammad Fereshtehnejad, João C Fernandes, Irina Filip, Florian Fischer, Nataliya Foigt, Nancy Fullman, Thomas Fürst, João M Furtado, Emmanuela Gakidou, Amiran Gamkrelidze, Alberto L García-Basteiro, Gebremedhin Berhe Gebregergs, Tsegaye Tewelde Gebrehiwot, Johanna M Geleijnse, Hailay Abrha Gesesew, Katherine B Gibney, Richard F Gillium, Philimon N Gona, Sameer Vali Gopalani, Alessandra Goulart, Rajeev Gupta, Tanush Gupta, Nima Hafezi-Nejad, Randah Ribhi Hamadeh, Mitiku Teshome Hambisa, Alexis J Handal, Graeme J Hankey, Hilda L Harb, Josep Maria Haro, Rasmus Havmoeller, Ileana Beatriz Heredia-Pi, Esayas Haregot Hilawe, Nobuyuki Horita, H Dean Hosgood, Sorin Hostiuc, Peter J Hotez, Damian G Hoy, Hsiang Huang, Ehimario Uche Igumbor, Manami Inoue, Asnake Ararsa Irenso, Sheikh Mohammed Shariful Islam, Kathryn H Jacobsen, Mihajlo Jakovljevic, Panniyammakal Jeemon, Paul N Jensen, Devasahayam Jesudas, Vivekanand Jha, Jost B Jonas, Mikk Jürisson, Rajendra Kadel, Ritul Kamal, André Karch, Seyed M Karimi, Amir Kasaeian, Srinivasa Vittal Katikireddi, Norito Kawakami, Peter Njenga Keiyoro, Andre Pascal Kengne, Chandrasekharan Nair Kesavachandran, Yousef Saleh Khader, Ejaz Ahmad Khan, Gulfaraz Khan, Young-Ho Khang, Sahil Khera, Abdullah Tawfih Abdullah Khoja, Mohammad Hossein Khosravi, Christian Kieling, Daniel Kim, Pauline Kim, Yun Jin Kim, Sami Kishawi, Katarzyna A Kissimova-Skarbek, Niranjan Kissoon, Mika B Kivimaki, Ann Kristin Knudsen, Yoshihiro Kokubo, Parvaiz A Koul, Ai Koyanagi, Kristopher J Krohn, Barthelemy Kuate Defo, Burcu Kucuk Bicer, Fekede Asefa Kumsa, Anton Carl Jonas Lager, Dharmesh Kumar Lal, Ratilal Lalloo, Van C Lansingh, Anders Larsson, Dennis Odai Laryea, Pablo M Lavados, Janni Leung, Miriam Levi, Misgan Legesse Liben, Rakesh Lodha, Giancarlo Logroscino, Alan D Lopez, Scott A Lorch, Paulo A Lotufo, Raimundas Lunevicius, Isis Eloah Machado, Mohammed Magdy Abd El Razek, Carlos Magis-Rodriguez, Marek Majdan, Reza Majdzadeh, Azeem Majeed, Reza Malekzadeh, Tsegahun Manyazewal, Laurie B Marczak, Jose Martinez-Raga, Francisco Rogerlândio Martins-Melo, João Massano, Mohsen Mazidi, Colm McAlinden, John J McGrath, Suresh Mehata, Kala M Mehta, Toni Meier, Kidanu Gebremariam Meles, Ziad A Memish, Walter Mendoza, Melkamu Merid Mengesha, Desalegn Tadese Mengistu, George A Mensah, Atte Meretoja, Tuomo J Meretoja, Ted R Miller, Mubarek Abera Mingistie, Vuong Minh Minh Nong, Shiva Raj Mishra, Karzan Abdulmuhsin Mohammad, Alireza Mohammadi, Shafiu Mohammed, Ali H Mokdad, Mariam Molokhia, Lorenzo Monasta, Maziar Moradi-Lakeh, Paula Moraga, Shane D Morrison, Ulrich O Mueller, Erin Mullany, Kate Muller, Christopher J L Murray, Kamarul Imran Musa, Jean Nachega, Chie Nagata, Mohsen Naghavi, Bruno Ramos Nascimento, Ionut Negoi, Cuong Tat Nguyen, Quyen Le Nguyen, Trang Huyen Nguyen, Ole F Norheim, Bo Norrving, Jean Jacques Noubiap, Martin J O'Donnell, Felix Akpojene Ogbo, In-Hwan Oh, Anselm Okoro, Olanrewaju Oladimeji, Andrew Toyin Olagunju, Bolajoko Olubukunola Olusanya, Jacob Olusegun Olusanya, Eyal Oren, Alberto Ortiz, Erika Ota, Mayowa O Owolabi, Mahesh PA, Smita Pakhale, Basant Kumar Panda, Songhomitra Panda-Jonas, Eun-Kee Park, George C Patton, David M Pereira, Fernando Perez-Ruiz, Konrad Pesudovs, Carrie Beth Peterson, Michael Robert Phillips, Farhad Pishgar, Dietrich Plass, Maarten J Postma, Richie G

Poulton, Mostafa Qorbani, Amir Radfar, Anwar Rafay, Vafa Rahimi-Movaghar, Mahfuzar Rahman, Rajesh Kumar Rai, Salman Rawaf, Sarah E Ray, Robert Reiner, Andre M N Renzaho, Satar Rezaei, Mohammad Sadegh Rezai, Antonio Luiz L Ribeiro, Luca Ronfani, Gholamreza Roshandel, Dietrich Rothenbacher, Yogesh Damodar Sabde, Perminder S Sachdev, Mahdi Safdarian, Saeid Safiri, Rajesh Sagar, Joshua A Salomon, Abdallah M Samy, Maria Dolores Sanchez-Niño, Itamar S Santos, Milena M Santric Milicevic, Maheswar Satpathy, Monika Sawhney, Sonia Saxena, Mete I Saylan, Maria Inês Schmidt, Aletta E Schutte, David C Schwebel, Falk Schwendicke, Soraya Seedat, Abdulbasit Musa Seid, Sadaf G Sepanlou, Masood Ali Shaikh, Morteza Shamsizadeh, Kenji Shibuya, Girma Shifa, Mika Shigematsu, Rahman Shiri, Mark G Shrime, Diego Augusto Santos Silva, João Pedro Silva, Jasvinder A Singh, Amber Sligar, Chandrashekhar T Sreeramareddy, Vasiliki Stathopoulou, Dan J Stein, Muawiyyah Babale Sufiyan, Bryan L Sykes, Cassandra E I Szoeke, Santosh Kumar Tadakamadla, Fentaw Tadese, Nuno Taveira, Arash Tehrani-Banihashemi, Mohamad-Hani Temsah, Belay Tessema, Alan J Thomson, Amanda G Thrift, Marcello Tonelli, Roman Topor-Madry, Miguel Tortajada-Girbés, Bach Xuan Tran, Emin Murat Tuzcu, Stefanos Tyrovolas, Kingsley Nnanna Ukwaja, Eduardo A Undurraga, Olalekan A Uthman, Job F M van Boven, Tommi Vasankari, Narayanaswamy Venketasubramanian, Vasiliy Victorovich Vlassov, Stein Emil Vollset, Tolassa Wakayo, Haidong Wang, Yuan-Pang Wang, Elisabete Weiderpass, Robert G Weintraub, Andrea Werdecker, Joshua Wesana, Tissa Wijeratne, Charles Shey Wiysonge, Denis Xavier, Gelin Xu, Bereket Yakob, Lijing L Yan, Mehdi Yaseri, Hassen Hamid Yimam, Naohiro Yonemoto, Seok-Jun Yoon, Mustafa Z Younis, Zouvida Zaidi, Maysaa El Sayed Zaki, Hajo Zeeb, Zerihun Menlkalew Zenebe, and Liesl Joanna Zuhlke.

Extracting, cleaning, or cataloging data; designing or coding figures and tables

Olatunji Adetokunboh, Muktar Beshir Ahmed, Sneha Aiyar, Megha Arora, Solomon Weldegebreal Asgedom, Reza Assadi, Ryan M Barber, James R Bennett, Jennifer Benson, Adugnaw Berhane, Donal Bisanzio, Lemma Negesa Bulton Bulto, Austin Carter, Dirk Douwes-Schultz, Sergey Petrovich Ermakov, Tahvi Frank, Maya Fraser, Joseph J Frostad, Nancy Fullman, Jamie Hancock, Guoqing Hu, Sarah Charlotte Johnson, Nicholas J Kassebaum, Yousef Saleh Khader, Young-Ho Khang, Kristopher J Krohn, Barthelemy Kuate Defo, Xie Rachel Kulikoff, Michael Kutz, Mohsen Mazidi, Melkamu Merid Mengesha, Joseph Mikesell, Alireza Mohammadi, Christopher J L Murray, Mohsen Naghavi, Ionut Negoi, Olanrewaju Oladimeji, Mahesh PA, Max Petzold, David M Pigott, Sarah E Ray, Jürgen Rehm, Gregory A Roth, Enrico Rubagotti, Mohammad Ali Sahraian, Payman Salamati, Peilin Shi, Naris Silpakit, Chandrashekhar T Sreeramareddy, Vinay Srinivasan, Patrick J Sur, Mohammad Tavakkoli, Alexander H Thompson, Haidong Wang, Tissa Wijeratne, Gelin Xu, Pengpeng Ye, and Naohiro Yonemoto.

Managing the overall research enterprise

Ashkan Afshin, Blair R Bumgarner, Charbel El Bcheraoui, Kara Estep, Simon I Hay, Laurie B Marczak, Ali H Mokdad, Meghan D Mooney, Erin Mullany, Kate Muller, Christopher J L Murray, Mohsen Naghavi, Joseph Salama, Katya A Shackelford, Amber Sligar, Caitlyn Steiner, and Haidong Wang.

Did not provide authorship contribution information

Syed M Aljunid, Juma M Alkaabi, Rajaa Al-Raddadi, Elena Alvarez Martin, Shivanthi Balalla, Aminu K Bello, Soumyadeep Bhaumik, Habtamu Mellie Bizuayehu, Peter Bjerregaard, Soufiane Boufous, Alexandra Brazinova, Michael Burch, Ruben Estanislao Castro, Massimo Cirillo, Jefferson G Fernandes, Kahsu Gebrekirstos Gebrekidan, Amha Admasie Gelaye, Bikila Lencha Gemechu, Kasiye Shiferaw Gemechu, Ricard Genova-Maleras, Shifalika Goenka, Rahul Gupta, Juanita A Haagsma, Sivadasanpillai

Harikrishnan, Claudiu Herteliu, Thomas Jaenisch, Achala Upendra Jayatilleke, Ye Jin, Ganesan Karthikeyan, Sefonias Getachew Kelbore, Andrew Haddon Kemp, Veena S Kulkarni, Steven E Lipshultz, Mark T Mackay, Geetha R Menon, Edward J Mills, Murali B V Mohan, Kovin S Naidoo, Lipika Nanda, Gopalakrishnan Natarajan, Muhammad Imran Nisar, Charles D Parry, Mahboubeh Parsaeian, Tejas Patel, Rogelio Perez-Padilla, Manorama Purwar, Sajjad Ur Rahman, Saleem M Rana, Paturi Vishnupriya Rao, Mohammad Bagher Rokni, Sare Safi, Amirhossein Sahebkar, Sundeep Santosh Salvi, Juan Ramon Sanabria, Sam Schulhofer-Wohl, Sheikh Mohammed Shariful Islam, Balakrishna P Shetty, Shireen Sindi, Prashant Kumar Singh, Eirini Skiadaresi, Joan B Soriano, Soumya Swaminathan, Dejen Yemane Tekle, Nihal Thomas, Fotis Topouzis, Ulises Trujillo, Nikolaos Tsilimparis, Jillian L Waid, James D Wilkinson, Shimelash Bitew Workie, Ayalnesh Zemene Yalew, and Yuichiro Yano.

Section 1. GBD Overview

Section 1.1. Geographic Locations of the Analysis

The geographic locations included in GBD 2016 have been arranged into a set of hierarchical categories composed of seven super-regions and a further nested set of 21 regions containing 195 countries and territories. The locations for which GBD estimated global, regional, and national life expectancy, all-cause mortality, and YLDs expanded following GBD 2015. For GBD 2016, subnational assessments are presented for China, India, the United States, and Brazil; results for other countries with a population greater than 200 million in 2016 and for those developed based on national policy interest are reported in separate studies or reports. Since GBD 2015, which included subnational assessments for Brazil, India, Japan, Kenya, Saudi Arabia, South Africa, Sweden, the United States, China, Mexico, and the United Kingdom, an additional two subnational assessments were undertaken. Subnational breakdowns for countries in GBD 2016 included: 26 states and one federal district for Brazil, 34 provinces and municipalities for China, 31 states and union territories of India, 34 provinces of Indonesia, and 51 states and districts for the United States. Combined, there are a total of 143 locations at the first subnational unit level. Included in subnational Level 1 locations are countries that have been subdivided into the first subnational level, such as states or provinces, for the GBD analysis; subnational Level 2 only applies to India and England. For this paper we present data at the national and territory level.

Section 1.2. Time Period of the Analysis

A complete set of life expectancy and all-cause deaths and rates were computed for the years 1970-2016.

All GBD 2016 mortality results and online data visualizations are available at http://vizhub.healthdata.org/mortality. Results for all GBD metrics available at http://www.healthdata.org/results/data-visualizations.

Section 1.3. Statement of GATHER Compliance

This study complies with the Guidelines for Accurate and Transparent Health Estimates Reporting (GATHER) recommendations. We have documented the steps involved in our analytical procedures and detailed the data sources used in compliance with the Guidelines for Accurate and Transparent Health Estimates Reporting (GATHER). See Appendix Table 1 for GATHER checklist.

The GATHER recommendations can be found here: http://gather-statement.org/

Section 1.4. List of abbreviations

5q0: probability of death from birth to age 5 years
45q15: probability of death from age 15 years to 60 years
ART: antiretroviral therapy
BTL: basic tabulation list
CBH: complete birth history
CD4: white blood cells (T-cells)
COD: causes of death
CODEm: Cause of Death Ensemble model
DALY: disability-adjusted life-year

DHS: Demographic and Health Surveys
DSP: Disease Surveillance Points
EPP: Estimation and Projection Package
GATHER: Guidelines for Accurate and Transparent Health Estimates Reporting
GBD: Global Burden of Disease
GEMS: Global Enteric Multicenter Study
HIV CDR: Crude death rate due to HIV/AIDS
ICD: International Classification of Disease
IPUMS: Integrated Public Use Microdata Series
LDI: lag-distributed income per capita
MCCD: Medical Certification of Causes of Death
MICS: Multiple Indicator Cluster Surveys
NCD: non-communicable disease
SBH: summary birth history
SDI: Socio-demographic Index
SEER: Surveillance, Epidemiology, and End Results Program
SRS: Sample Registration System
ST-GPR: Spatiotemporal Gaussian process regression
UI: uncertainty interval
UN: United Nations
U5MR: under-5 mortality rate
VR: vital registration
WFS: World Fertility Surveys
WHO: World Health Organization
YLD: years lived with disability
YLL: years of life lost

Section 1.5. GBD results overview

Results from the Global Burden of Disease Study (GBD 2016) are now measured in terabytes. Results are available in an interactive data downloading tool available in the Global Health Data exchange (GHDx). The tool contains the complete set of results from all summary papers; however, specialized tables from the papers are available as separate entries in the GHDx as were made available for GBD 2015.

The current version of the data download tool is available in the GHDx and contains core summary results for the Global Burden of Disease Study 2016 (GBD 2016): http://ghdx.healthdata.org/gbd-results-tool. The core summary results include deaths, YLLs, YLDs, and DALYs. It includes data for causes, risks, cause-risk attribution, etiologies, and impairments.

In the GBD 2016 version, the tool also contains measures such as prevalence and incidence as well as rate of change data. Data above a certain size cannot be viewed online but can be downloaded. Depending on the size of the download, users need to enter an email address and a download location will be sent to them when the files are prepared.

Section 1.6. Data input sources overview

GBD 2016 incorporated a large number and wide variety of input sources to estimate mortality, causes of death and illness, and risk factors for 195 countries and territories from 1990-2016. These input sources are accessible through an interactive citation tool available in IHME's GHDx. Users can retrieve citations for a specific GBD component, cause or risk, and location by choosing from the available selection boxes. They can then view and access GHDx records for input sources and export a CSV file that includes the GHDx metadata, citations, and information about where the data were used in GBD. Additional metadata for each input source are available through the citation tool, as required by the GATHER statement.

The citation tool is accessible through the GHDx at http://ghdx.healthdata.org/gbd-2016/data-input-sources

Section 1.7. Funding Sources

Funding for this research was provided by the Bill & Melinda Gates Foundation, and the National Institute on Aging and the National Institute of Mental Health of the National Institutes of Health.

Section 2. GBD 2016 All-Cause Mortality and HIV Estimation Process

Section 2.1. Overview

The goal of the all-cause mortality estimation process for the GBD is to produce the most accurate time series estimates for 1970 to 2016 for all-cause deaths and death rates broken down into the GBD age groups and by sex for all 591 locations in the GBD 2016. This task is necessarily complicated by the diversity of data sources available for all-cause mortality for different age groups, the known biases in some sources, and the powerful effects of the HIV epidemic in countries with large epidemics on the age pattern of mortality. In this appendix, we divide the analysis of all-cause mortality into five distinct but interconnected areas: child mortality (5q0), adult mortality between ages 15 and 60 (45q15), estimation of a complete set of age-specific death rates, estimation of HIV mortality, and final estimates of age-specific mortality including HIV and fatal discontinuities. While HIV is a cause of death, we present some information on the epidemiological modeling of HIV in this section because of the close interdependency between HIV mortality estimation and the all-cause mortality estimation process.

If all countries had complete vital registration (VR) systems recording the event of death and periodic censuses, the task of estimating all-cause mortality rates would be much easier. However, for many countries of lower-to-middle levels of sociodemographic development, information systems on mortality are incomplete and multiple sources must be used to infer levels, trends, and patterns of mortality. The analytical building blocks of the process, as shown in Appendix Figure 1, are repeated through two full iterations. The reason for the iterations is the interdependence between the HIV epidemiological models on estimates of HIV-free all-cause mortality and the use of estimated HIV crude death rates as an input to the 5q0, 45q15, and age-specific mortality models. In the following sections, we provide more detail on each analytical module identifying primary input data and analytical processes.

Section 2.2. Child mortality

Section 2.2.1. Data sources

Vital registration from Causes of Death team

Approximately 62% of deaths data from VR systems were used as input for our all-cause mortality modeling. These data were provided by the GBD causes of death (CoD) research team and were aggregated into total age-sex-specific all-cause mortality for each location-year. This aggregation occurred after the data were adjusted and mapped to the GBD cause list.

Data intended for use in causes of death modeling are assessed for quality with respect to consistency of cause fractions, diagnostic accuracy, and missing data, whereas for all-cause mortality modeling it is more important that data are fully representative of the given estimation area and are consistent with other all-cause mortality data sources. Thus, there are cases in which VR data prepared for cause-specific modeling cannot be used in all-cause modeling or must be adjusted based on degree of completeness before being used.

In our vetting of CoD VR data, we dropped points with a more than 1% difference from corresponding points in the WHO database. There were instances where VR data used in cause-specific mortality

analysis had been collapsed to Basic Tabulation List (BTL) format rather than in full cause classification list format (e.g., ICD9). In some of these cases, we elected to use WHO data instead.

Vital registration, sample registration systems, and Disease Surveillance Points from other sources

We endeavored to include all available data from VR systems as inputs in our all-cause mortality estimation process. To achieve this, we utilized a number of multi-country VR sources, including the WHO Mortality Database, the Human Mortality Database, United Nations Demographic Yearbooks, and OECD (Organisation for Economic Co-operation and Development) databases. These multi-country sources are regularly updated in our systems when new data are added. Beyond multi-country sources, for all ongoing national VR systems (for example, the USA National Vital Statistics System), where possible, we cataloged all data sources from each system.

Some countries that do not have a well-performing VR system implement sample registration systems that are incomplete by design. We made use of these data, paying close attention to the proper weighting of sampled data and consistency with other representative sources. We have systematically extracted data from the Sample Registration System Statistical Report series published by the Registrar General of India. For the Disease Surveillance Points (DSP) system of China, we obtained both national and provincial level DSP data through a data usage agreement with the Chinese Center for Disease Control and Prevention. Census data are systematically extracted from Demographic Yearbook series, Integrated Public Use Microdata Series (IPUMS), and statistical reports from the national statistical bureaus. Manuscript Figure 1 shows more detail of percent of completeness of registered deaths from 1990 to 2016.

Under-5 populations and live births

For most GBD locations, live births come from the World Population Prospects 2015 (WPP 2015). For subnational locations, we often use interpolated census birth numbers scaled to the national estimates. For locations not estimated in the WPP 2015, we use interpolated census birth counts.

Complete birth history microdata

Complete birth histories (CBHs), the preferred method for data collection on child mortality in the absence of VR, rely on administering surveys to mothers. The questionnaires ask about all living and deceased children, including date of birth, survival status, and date of death. These modules are included in many routine survey series, including the World Fertility Surveys (WFS), Demographic and Health Surveys (DHS), Multiple Indicator Cluster Surveys (MICS), and many national survey programs. When available, we download and use microdata that has individual-level survey responses as opposed to using tabulated results.

Complete birth history tabulated data

In some instances, tabulated records from reports become available before survey microdata, and we incorporate these data points into our database of 5q0 data as well. However, as microdata become available, we update with point estimates from our processed microdata rather than the tabulated report estimates.

Summary birth history microdata

Summary birth history (SBH) questionnaires are a shorter alternative to complete birth histories. Instead of asking in detail about each child, summary birth histories simply ask mothers how many children they have given birth to and how many of the children have died. The questionnaires are shorter and can be more easily attached to other surveys. Often, censuses and MICS surveys contain summary birth histories. For GBD, we have compiled all available SBH data with microdata that enables us to apply the updated SBH method that leads to more accurate and timely assessment of U5MR.[1]

Summary birth history tabulated data

In cases where we do not have access to the microdata on SBH modules from surveys and censuses, we utilize the reported estimates of U5MR from survey or census reports and outlier the first two data points based on mothers in ages 15-19 and 20-24.

Under-5 age-sex patterns from VR/SRS/DSP

VR systems are the primary source of data for the under-5 age pattern of mortality in high-income countries. Often, these data are classified into several age groups: early neonatal (0-6 days), late neonatal (7-27 days), post-neonatal (28-364 days), and 1 to 4 years. Some country-years of data have other age groupings with less specificity, with the early and late neonatal age groups combined, or all of the under-1 age groups combined. Sample Registration Systems (SRS) also provide data for the age-sex pattern of under-5 mortality in several countries (notably India and Bangladesh), as well as the DSP system in China.

Under-5 age-sex patterns from complete birth history

In many countries without VR systems, CBH surveys can be used to obtain age-sex patterns of mortality in under-5 age groups. These sources are described above in the "complete birth history microdata" section. For all CBH microdata sources, we apply direct estimation methods to obtain probabilities of death for each of the under-5 age groups. Within each survey, if each observation is a child recalled by a mother, observations are grouped into 5-year groups in time to provide a data point of probability of death for each of the under-5 age-sex groups. Recall is cut off 15 years before the survey, limiting data points estimated from the survey to the 15 years prior. All of these estimates are then put in the database of estimates for the age-sex pattern of under-5 mortality.

Section 2.2.2. Vital registration prioritization

Our continual evaluation of VR data sources has led us to develop a general hierarchy of preferred VR sources. When considering which of multiple sources to use for a given location-year, we first prefer to use WHO data from GBD cause-specific mortality estimation, then unadjusted WHO data, then Human Mortality Database (HMD) data, then UN Demographic Yearbook data. There were exceptions to this hierarchy where we had reason to believe that there were quality issues with a certain source. For instance, where available we preferred to use HMD VR over WHO data for Germany, Taiwan (Province of China), and Spain due to WHO data producing mortality rates that were inconsistent with previously established trends. Single-country VR sources were evaluated based on consistency with other data sources and also VR system documentation.

Section 2.2.3. Identify vital registration under-enumeration for bias correction

The approach to estimating the completeness of VR systems for deaths under age 5 is the same as that of the previous two GBD studies.

In many countries with VR systems to record deaths, CBH and/or SBH are also conducted. By comparing the under-5 death rates from these sources to the levels from VR or sample registration systems, we can assess the completeness of under-5 death registration. Completeness can evolve over time as seen with the likely declines in completeness in Central Asia in the 1990s or the increases in completeness in other settings.[2,3] We estimate VR completeness where VR data are available using a model that allows for completeness to vary over time. This assessment is undertaken in two steps: we first assess whether VR is biased, and then we assess time-varying completeness.

In the first step, a country-level regression of log10 (5q0) on year with a binary indicator variable for 5q0 estimates derived from VR systems is used to determine whether or not a VR system is biased (see equation). If the coefficient for the VR indicator variable is statistically significant at the 0.05 α-level, we deem the VR system to be biased.

$$log_{10}({}_5q_0)_t = \alpha + \beta_1 * t + \beta_2 * I_{VR} + \xi_t$$

where t is time (a continuous variable); I_{VR} is an indicator for 5q0 estimates derived from VR systems; and ξ_t is in error term.

Second, for all countries with biased VR systems, we estimate the bias, allowing for completeness to evolve over time. We first apply Loess regression to all non-VR 5q0 estimates in a given country; we then calculate the difference between the Loess predicted $\log_{10}$(5q0) and the observed $\log_{10}$(5q0) estimate from VR in a given year. Since we believe that completeness changes relatively slowly over time, the bias in any given year is defined as the mean difference between the predicted $\log_{10}$(5q0) and the observed $\log_{10}$(5q0) from VR systems over the adjacent five-year period. This allows for flexibility in the bias correction over time while still maintaining the premise that the completeness of VR systems does not change abruptly. Loess predictions can be unreliable out of sample, so for country-years outside of the range of non-VR data used to generate the predicted 5q0, we use the mean bias from the nearest five years of bias estimates from VR points that are within the timespan of the non-VR data. We then correct the VR estimates of 5q0 using the bias correction as shown in the equation:

$${}_5q_0^{corr.} = {}_5q_0^{obs.} * 10^{\widehat{bias}}$$

Where ${}_5q_0^{corr.}$ is the corrected estimate of 5q0; ${}_5q_0^{obs.}$ is the observed estimate of 5q0; and $\widehat{bias}$ is the bias estimate described above. Once the biased estimates have been adjusted, we also approximate the variance of the bias estimate. This variance is approximated using the median absolute deviation (MAD) comparing the biased VR estimates to the Loess-based estimate of $\log_{10}$(5q0). As with the bias estimation above, the MAD is estimated over a five-year time period. This MAD times 1.4826 is an approximation of standard deviation used to add variance to the biased VR data when included in our final Gaussian Process Regression (GPR) model described in detail later.

In addition to countries where there are both VR estimates and survey estimates of under-5 mortality, there are countries for which only VR data are available, and the VR systems are considered biased. This is a problem particularly in English-speaking Caribbean countries, so for these countries we have adjusted 5q0 estimates from VR using the regional average VR bias in a given year for those countries with both VR and survey 5q0 estimates. The countries for which VR systems have been adjusted using this method include Antigua and Barbuda, Bahamas, Barbados, Bermuda, Dominica, Grenada, Saint Lucia, and Saint Vincent and the Grenadines. While there is no direct evidence on the level of VR bias in these countries, assuming they are complete when similar countries in the region have under-registration seems unwarranted.

Section 2.2.4 Biennial 5q0 estimates

Computation of complete birth history 5q0

Microdata (individual-level survey data) from CBH yield direct calculation of death numbers and probabilities of death in the under-5 age group. Observations are grouped into two-year intervals such that biennial estimates of 5q0 are obtained from these survey data. In GBD 2016, we have unpooled surveys for our analysis, whereas surveys were pooled by series in GBD 2013.[4] Instead of grouping observations from all DHS complete birth history questionnaires from a country into one full set of observations and all MICS observations from multiple survey years into another full set of observations, we analyzed each survey separately by location (e.g., DHS 2012, DHS 1996, MICS 2002). This allowed for a greater ability to address known data quality issues in specific surveys. To compensate for the decreased sample size and to generate greater stability in the unpooled data points, we created two-year estimates of under-5 mortality, pooling observations over two-year periods instead of single years.

Processing of tabular complete birth history

In some instances, microdata from surveys were not available. If survey reports could be obtained but the microdata were not available for us to do our own calculations to obtain 5q0, we used report data point estimates. These estimates were added directly to the under-5 mortality database.

Section 2.2.5. Summary birth history time series method

Summary birth history method from microdata

Rajaratnam and colleagues developed an updated SBH method that is able to provide more accurate and timely estimates of U5MR from microdata on SBH from surveys and censuses.[1]

Analysis of summary birth history from tabular data

When only tabular data are available for the numbers of children ever born and number of children that have died by mother's age, we apply the Maternal Age Cohort model from the method developed by Rajaratnam and colleagues.[1]

Section 2.2.6. 5q0 data synthesis, model running, and bias correction

Data synthesis using ST-GPR and bias correction

We apply the child mortality estimation methodology as reported by Wang and colleagues.[4] Based on the under-5 mortality data synthesis model for the Global Burden of Disease Study 2010,[2,5] 2013,[4] and 2015,[6] we have incorporated data bias adjustment into the modeling process. Specifically, we have

included a fixed effect for source type across all locations to detect systematic differences in the level of child mortality, controlling for covariates for one source type versus another. The groups of sources used to make this adjustment are listed in the table below. In addition, we include a random effect for each country-source. By choosing a reference source country-by-country or using the mean of a set of sources, we can adjust on a country-by-country basis for the problem of compositional bias created by substantial source-specific non-sampling error. Reference sources are not adjusted, even if multiple sources are used as reference. Once the systematic difference in sources is removed, we are able to avoid estimating false trends due to partial overlap of sources with different levels of non-sampling variance. We then apply the combination of non-linear mixed effects model, spatiotemporal regression, and Gaussian process regression to synthesize raw child mortality data after data bias adjustment to obtain consistent time series estimates of mortality with 95% uncertainty intervals for every country.

Table: Source types used in child mortality bias correction

**Data Source Type**
Complete Birth History-Demographic and Health Survey
Complete Birth History-AIDS Indicator Survey and Malaria Indicators Survey
Complete Birth History-World Fertility Survey
Complete Birth History-Multiple Indicator Cluster Survey
Complete Birth History-Census
Complete Birth History-Other survey Series
Summary Birth History-Demographic and Health Survey
Summary Birth History-Multiple Indicator Cluster Survey
Summary Birth History-Other survey series
Summary Birth History-AIDS Indicator Survey and Malaria Indicators Survey
Summary Birth History-Census
Summary Birth History-World Fertility Survey
Vital Registration/Sample Registration/Surveillance- complete
Vital Registration/Sample Registration/Surveillance- incomplete
Household Death Recall-Other survey series
Household Death Recall-Census
Household Death Recall – incomplete Vital Registration/Sample Registration/Surveillance

Mixed effect non-linear model and the bias adjustment for raw U5MR sources

In this stage, we used a non-linear mixed effects regression to estimate data bias and provide first stage predictions.

The nonlinear mixed effects regression model is

$$ {}_5m_{0cys} = \exp[(\beta_1 + \gamma_{1c}) * \log\left(LDI_{cy}\right) + (\beta_2 + \gamma_{2c}) * education_{cy} + \gamma_c + \gamma_{cs} + \alpha_t] + \beta_3 * HIV_{cy} + \varepsilon_{cys} $$

where c is country, y is year, s is source, and t is source type; each source was categorized into one of 17 source types across all countries, as listed in the table above.

Additionally, ${}_5m_0$ is under-5 mortality rate; *LDI* is lag-distributed income per capita; *education* is mean years of education for women of reproductive age (15-49 years); *HIV* is death rate due to HIV in age groups 0-4; γ is a random effect; α is a fixed effect on source type across countries; β_i is a fixed covariate coefficient; and ε is the residual. For each country, we rely on expert opinion to choose a source, or combination of sources, which are believed to be the least biased. If a country has a VR system which we deem to be complete (described in detail in an earlier section), this is the reference source. If a country does not have complete VR system, but has DHS estimates from complete birth histories, these were chosen as the reference source. If a country has neither of these types of data or DHS estimates are deemed unreliable, we assigned the surveys conducted after 1970, in combination, as the reference (incomplete VR data are not included). Additionally, in many countries we chose other surveys as the reference. For accurate estimation, it is important to have local knowledge on specific data sources' accuracy. All-cause mortality experts draw from their familiarity with data quality to help us to choose the reference category.

Each data source has an associated random effect as well as a source type fixed effect. The values of these random and fixed effects for the reference sources are deemed to be the true deviation from unbiased mortality level. In countries with multiple high-quality sources, the mean of the random and fixed effects from these sources is taken as this true deviation. We adjusted all other sources by including these reference values for the random and fixed effects values instead of those estimated for each individual source, as shown below.

$$ adjusted_5m_{0,cys} = \exp[(\beta_1 + \gamma_{1c}) * \log\left(LDI_{cy}\right) + (\beta_2 + \gamma_{2c}) * education_{cy} + \gamma_c + \gamma_{ref,c} + \alpha_{ref,c}] + (\beta_3 + \gamma_{3c}) * HIV_{cy} + \varepsilon_{cys} $$

The exception to this correction is incomplete VR data, which was adjusted upwards using a five year rolling mean of the difference between incomplete vital registration and a Loess of the already-adjusted survey data, described above in section 2.2.3.

Spatiotemporal smoothing

The spatiotemporal stage smooths the residuals between the predicted time series of 5q0 and the adjusted raw data over time and across countries in the same GBD region. The predicted time series for this smoother was obtained from the equation below; no random effects or survey type fixed effects are included.

$$ predicted_5m_{0,cy} = \exp[\beta_1 * \log\left(LDI_{cy}\right) + \beta_2 * education_{cy} + \alpha_{intercept}] + \beta_3 * HIV_{cy} $$

We first found the residuals between the predicted time series, above, and the adjusted points. We then applied a combination of smoothing functions to these residuals. For each country-year, we weighted all

the data points in this region based on their proximity to this country-year in space and time. We gave 99% of the weight to in-country residuals, and 1% of the weight to out-of-country residuals. Additionally, we used a modified tricubic window, as specified below, to give more weight to points closer in time, and less weight to points further in time.

$$w_t = \left(1 - \left(\frac{|r_t - r_{est}|}{1 + \text{argmax}_t |r_t - r_{est}|}\right)^{\lambda}\right)^3$$

The r_t and r_{est} terms are, respectively, the year of interest and the year of the residual being weighted. The $\text{argmax}_t|r_t - r_{est}|$ term is the maximum distance between the year of interest and a residual within the region. The λ parameter in this weighting function dictates how quickly the weights fall off as the distance in time increases: a larger λ implies that the assigned weights will diminish slowly with time, while a smaller λ allows the weights to diminish more rapidly with time.

λ values were chosen using the parameter selection process described below. We then created one estimate of the smoothed residuals using a linear fit to this weighted data; this is similar to a Loess fit. Additionally, we created a second estimate of the smoothed residuals by calculating the weighted average of this data.

We then combined these two estimates for a final estimate of the smoothed residuals. In data-dense countries, more weight was given to the local linear fit; in data sparse countries, more weight was given to the weighted average. The equation for this is as follows.

$$final\ smoothed\ residual = k * linear\ estimate + (1 - k) * weighted\ average$$

where $k = \frac{number\ of\ in\ country\ data\ points}{number\ of\ in\ country\ data\ points + number\ of\ country\ years\ with\ no\ data}$

Finally, the smoothed residuals were added back to the predictions from above; this smoothed approximation to the adjusted data was used as the prior for GPR, described below.

Third stage: Gaussian process regression (GPR)

The output of the space-time smoothing step was used as a prior for GPR, which produced a final time series of point estimates, as well as confidence bounds. Parameters for GPR were chosen through cross-validation as described below.

The model for GPR is shown below, where μ_t is the true $\log_{10}(5q0)$ at time t, f(t) is the baseline mortality risk, and S_t captures excess mortality due to fatal discontinuities. S_t is estimated independently of f(t). M and C describe the Gaussian process, giving the mean and covariance, respectively.

$$\mu_t = f(t) + S_t$$

$$f(t) \sim GP(M, C)$$

We specified a prior distribution for f(t) from the spatiotemporal regression, and a likelihood function which describes the data generation process; the specified prior distributions and likelihood function are described below. We then used Markov Chain Monte Carlo (MCMC)[7] to approximate the posterior

distribution of f(t) which also incorporates information from the observed empirical estimates of adult mortality. An MCMC chain of length 5,000 was produced; the first 3,000 samples were discarded and the remaining 2000 were thinned by a factor of 2 for a total of 1,000 simulations retained. The reported best estimates and uncertainty intervals were generated from the mean and the 2.5th and 97.5th percentiles of the 1,000 samples, respectively.

The prior distribution of f(t) can be described in terms of the mean prior—the prior for M—and the covariance prior—the prior for C. We utilized the second stage predictions as the mean prior and used a Matern covariance function to describe the covariance prior. The parameters of the Matern covariance function were selected through cross-validation and are location-specific.

Likelihood

The likelihood describes the probability of observing the data given a particular set of parameters. As shown in the equation below, we used a normal model for describing the probability of observing a particular value of log(5q0) where the mean is given by f(t) and the variance by V_t, the data variance.

$$\log_{10}({}_5q_{0_t}) \sim \mathrm{Normal}(f(t), V_t)$$

Data variance was calculated for each empirical observation of 5q0 and incorporated both sampling and non-sampling variation. The method for calculating the data variance depended on the type of data:

1. For estimates derived from complete VR data we assumed that there was no non-sampling variance and included only sampling variance as computed from a binomial model. We set N equal to the national population aged 0 to 5 years and p equal to the mortality rate, ${}_5m_0$. We calculated the variance of ${}_5m_0$ from p(1-p)/N and then transformed this to the variance of $\log_{10}$(5q0) using the delta method.[8]
2. For estimates derived from incomplete VR data, we wanted to include not only sampling variance but also the non-sampling variance that arises from uncertainty in the completeness estimate. For these data, the total data variance was given by the sum of the sampling variance (calculated as for complete VR data) and the variance of the completeness estimate;
3. For estimates derived from CBHs we generate 1,000 simulations of 5q0, convert these estimates into $\log_{10}$ space and calculate the sampling variance from these 1,000 simulations;
4. For estimates derived from SBHs we use the standard error from the mean residuals;
5. For estimates not covered under the above four calculations the missing data variance is determined as the maximum standard error from non-VR points in the country, if the data variance is still missing it is calculated as the maximum standard error from non-VR data in the GBD region.
6. Finally, for each source type, we calculate the within-source-type variance of the source-specific random effect. This additional non-sampling variance is then converted to $\log_{10}$ space and added to the variance as calculated above for all data points not classified as complete VR.

Hyper-parameter selection for under-5 mortality rate ST-GPR

For GBD 2016, we used the same hyper-parameters that were selected for GBD 2015. For locations that were newly added in GBD 2016, we used for Indonesia national-level parameters for the provinces and the average parameter values for Japanese prefectures for the British local government areas.

In GBD 2015, we expanded the scope of our parameter selection to include variables used in space-time smoothing in addition to scale and amplitude used in GPR. We applied rigorous out-of-sample predictive validity testing to select space-time and GPR parameters, and the process was carried out in the following steps:

1. For space-time smoothing, we tested ζ space weight values of 0.7, 0.8, 0.9, and 0.99 and λ time weight values of 0.1 to 0.9 in increments of 0.1. We test five values of the scale—10, 15, 20, 25, 30 --- and five values of the squared amplitude—1, 1.5, 2, 2.5, and 3 times the mean squared error of the residuals from the second-stage prediction model. Because we tested combinations of both space-time and GPR parameters, this led to a total of 900 combinations tested in each process.

2. We divided the data into testing and training sets 100 times. Data were divided as follows: for each region, a number X between 10 and 20 was sampled and the most recent X years of data in that region were assigned to the testing set. Then a number N between 5 and 10 was sampled, a country from within the region was sampled, and a year where there is data in that country was sampled. All data within N years of the selected year in the selected country were assigned to the testing set. This was repeated as many times as there were countries in the region. Because iterations of this procedure were independent, the data selected for the testing set could overlap. Any data that were not selected for the testing set were included in the training set.

3. The space-time smoothing and GPR were fit on the training set using each set of parameters and estimates for every location are generated for the entire time period;

4. Within a given iteration we calculated the absolute relative error of the final GPR estimates compared to each empirical estimate in the testing set. We also classified each empirical estimate in the testing set as covered or not covered and calculated the percent of the data covered by the 95% uncertainty interval of the GPR estimates while considering the uncertainty of the data themselves. The determination of coverage was made by calculating total variance—the sum of the data variance and the variance of the GPR estimate—and then calculating a 95% uncertainty interval around the GPR estimate based on this total variance and assuming a normal distribution. If the empirical estimate was within this uncertainty interval, it was classified as covered, and otherwise not. For each combination of parameters, we calculated the mean absolute relative error and the mean coverage across all iterations from all countries within a particular group. The loss function described below was then calculated for each parameter combination, and the parameter combination with the lowest loss was selected for each group;

if coverage <=0.95, loss = absolute relative error + ((1-coverage)-0.05) / 5

if coverage > 0.95, loss = absolute relative error + (0.05 - (1-coverage))/1

5. For U5MR, parameter selection occurred at the location level, i.e. different parameters for each location. While there were data sparse locations, all locations had some data on U5MR for the time period we provide estimates for.

In some cases, we restricted the universe of possible parameters. Most of these restrictions occurred for the λ parameter. For many locations with complete VR, we knew that higher lambda values would result in a larger uncertainty interval than would be appropriate based on the quality of the available data. We also included a lower limit of λ for some locations with either incomplete VR or no VR, so that confidence intervals would reflect the uncertainty of these data. For example, in Western Europe, High-Income North America, High-Income Asia Pacific, and Australasia, we set the condition that λ must be less than 0.5 and the condition that ζ be 0.99. We also made some other manual exceptions to λ, ζ, and scale where results did not pass common sense tests.

We set the differentiability to 0.8 in countries with only complete VR data, except those in the Caribbean, Oceania, and the country of Mauritius, and to 2.0 in other all other locations. We used a lower differentiability in countries with complete VR data because in these countries we wanted the final estimates to follow the data closely even if the trend described was not smooth. In contrast, in countries where the data were less reliable we did not want the final estimates to be overly influenced by individual data points.

Section 2.2.7. Identify and remove outliers

There are several important quality-control steps in reviewing child mortality data and estimates. First, data points from years in which fatal discontinuities occurred are outliered, unless they are VR data points with sufficient information that the fatal discontinuities can simply be subtracted out of the VR data. The intent is to capture the underlying mortality risk rather than large stochastic variations. These fatal discontinuities are then added on in a later step (see section 2.7.3). Secondly, we outlier data sources with quality concerns such as the Afghanistan DHS from 2010. Our extensive collaborator network allows for review of sources, and collaborators can raise concerns over known issues with data sources about which they have expert knowledge.

Section 2.2.8. Rake subnational estimates to national level (excluding South Africa)

The estimation process for 5q0 does not enforce consistency between subnational estimates and national estimates. To ensure consistency throughout the GBD hierarchy, we rescaled the subnational estimates to the national level by population-weighting to get an implied national estimate from the subnational estimates, creating a scalar of the national-level estimate from GPR to the aggregated subnational estimates, and then multiplying all of the subnational estimates by this scalar to obtain the scaled estimates. In most cases, we considered national-level estimates to be more reliable, so we chose this strategy of subnational scaling. In locations with high-quality VR data, this scaling has a minimal effect, but the effect can be greater in locations with more subnational units and variable-quality data.

In South Africa, it was essential that the province-specific mortality patterns be consistent with HIV models, since such a large part of the trend was driven by deaths due to HIV/AIDS. In this case, instead of scaling provincial-level estimates to national-level GPR estimates, we aggregated province-level GPR estimates to generate the national-level estimates.

Section 2.2.9. Review estimates for quality

Estimates of 5q0 from the ST-GPR process were reviewed in comparison to UNICEF estimates from their 2015 revision and GBD 2015 results.[9] Any differences were traced to either changes in available data or changes induced by changes in hyper-parameters or input covariates. Revisions were made through this review process and through expert consultation with the GBD mortality collaborator network.

Section 2.2.10. Under-5 mortality rates with HIV

The 5q0 ST-GPR process generates U5MR for all GBD 2016 locations that is inclusive of the impact of all causes of death excluding fatal discontinuities, which are added in a separate step (see section 2.7.3).

Section 2.2.11. HIV-free 5q0

As a result of the non-linear mixed effects model, we are able to generate HIV-free 5q0 counterfactuals where the crude death rate due to HIV in age group 0-4 is set to zero. This is a crucial input to the GBD model life table system as described in section 2.5.

Section 2.2.12. Under-5 age and sex pattern model estimation

The process used to break down under-5 mortality into age- and sex- specific groups has been previously described.[7] The current process is largely similar but has been modified to improve the accuracy of predictions for countries affected by HIV/AIDS. As pointed out by Bradshaw et al.,[10] neonatal mortality tends to be overestimated if the all-cause under-5 mortality rate is used as the only predictor. We use a multi-stage modeling process to generate sex-specific estimates of early neonatal (days 0 to 6), late neonatal (days 7 to 27), post-neonatal (the remainder of the first year), under-1, and childhood (ages 1 to 4) mortality. First, the ratio of male to female under-5 probability of death is estimated, then age- and sex-specific mortality estimates are generated using this ratio. To fit models to obtain estimates, data from VR, sample VR, and CBHs are converted to mortality risks for specific age groups. Sources have differing levels of age specificity and at least include infant (composed of early neonatal, late neonatal, and post-neonatal) and child mortality, but can include all 4 smaller age groups. The two models – first the sex model, then the age-specific and sex specific model – are fit on the data.

The sex model first predicts the ratio of male probability of death under age 5 (5q0) to female 5q0 in rescaled logit space for each country *i* in region *j* in year *t*. The data are ordered by observed 5q0, and categorized into 20 evenly sized bins. We rescale the ratio data between 0 and 1 from between 0.8 and 1.5. Then the model is fit to the data as described in the equation below.

$$logit\left(\frac{\text{Male }{}_5q_0}{\text{Female }{}_5q_0}\right)_{\text{jit}} = \beta + \gamma_{{}_5q_0\text{ bin}} + \gamma_{\text{j}} + \gamma_{\text{i}} + \varepsilon_{\text{jit}}$$

The ratio is predicted by nested location and region random effects γ_i and γ_j, a random effect on the 5q0 bin, and an intercept term, β. A Loess regression is then used to smooth the estimated $\gamma_{{}_5q_0\,\mathrm{bin}}$ on 5q0, creating a continuous $\gamma'_{{}_5q_0\,\mathrm{bin}}$. Then, the equation below is used to predict the ratio of male to female ${}_5q_0$:

$$logit\left(\frac{\mathrm{Male}\ {}_5q_0}{\mathrm{Female}\ {}_5q_0}\right)_{\mathrm{jit}} = \hat{\beta} + \gamma'_{{}_5q_0\,\mathrm{bin}}\left({}_5q_{0_{\mathrm{jit}}}\right) + \hat{\gamma}_{\mathrm{j}} + \hat{\gamma}_{\mathrm{i}}$$

The predicted ratios are unscaled and inverse logited and used as the first stage prediction for ST-GPR as used for the estimation of under-5 mortality rate as described in this appendix. ST-GPR hyper-parameters were chosen based on location-specific data density. The male and female ${}_5q_0$ values are found using the system of equations that includes the prediction from the ST-GPR described above and equation below, where r_{birth} is the sex-ratio at birth.

$${}_5q_0 = \left(\frac{1}{1+\mathrm{r_{birth}}}\right) * (\mathrm{female}_5q_0) + \left(\frac{\mathrm{r_{birth}}}{1+\mathrm{r_{birth}}}\right) * (\mathrm{male}_5q_0)$$

Age-specific models are then fit for each age group on sex-specific data. A separate model is fit for each age group yielding five models for each sex: early neonatal, late neonatal, postneonatal, infant, and child. The log of the probability that an under-5 death occurs in a given age group conditioned on surviving to that age group is modeled instead of the mortality risk, simplifying the scaling process and restricting risks to be between 0 and 1. Because evidence suggests HIV has differential effects on different under-5 age groups,[11,12] the crude death rates from HIV/AIDS in the under-5 age group were included in the model for age groups after neonatal, since we make the assumption that all HIV deaths that occur in the first year of a child's life occur in the post-neonatal stage (after 28 days), since the literature on HIV in these age groups is still unclear but seems to indicate higher mortality in the post-neonatal stage, and there is no clear evidence to guide alternative methods of age-splitting under-1 deaths due to HIV.[12,13] We used crude death rate due to HIV from the GBD 2016 model (see section 2.2.6). The inclusion of this covariate improves both the fit and prediction of the model in countries with high HIV prevalence. In addition, in this version of GBD, we restricted the two new covariates added last round to the 1 to 4 age group, as they produced implausible directionality of correlation for the other age groups. That is, we included the maternal education covariate that is also used in the 5q0 first-stage model and the completeness of the source-specific 5q0 estimate for the data-point used in the regression for that 1 to 4 age group only. This completeness measure was calculated by taking the source-specific 5q0 point estimate and dividing by the final 5q0 estimate from GPR. The functional forms of the model are below.

$$\log(\Pr(\text{death at age enn}|\text{u5 death})_{\mathrm{jit}}) = \beta_1 + \gamma_{{}_5q_0\,\mathrm{bin}} + \gamma_{\mathrm{j}} + \gamma_{\mathrm{i}} + \varepsilon_{\mathrm{jit}}$$

$$\log(\Pr(\text{death at age y}|\text{u5 death})_{\mathrm{jit}}) = \beta_1 + \beta_2 * HIV_{it} + \gamma_{{}_5q_0\,\mathrm{bin}} + \gamma_{\mathrm{j}} + \gamma_{\mathrm{i}} + \varepsilon_{\mathrm{jit}}$$

$$\log(\Pr(\text{death in age } 1-4\ |\text{u5 death})_{\mathrm{jit}}) = \beta_1 + \beta_2 * HIV_{it} + \beta_3 * Mat.Ed._{it} + \beta_4 * Completeness_{sit} + \gamma_{{}_5q_0\,\mathrm{bin}} + \gamma_{\mathrm{j}} + \gamma_{\mathrm{i}} + \varepsilon_{\mathrm{jit}}$$

Where age y includes late-neonatal, post-neonatal, and under-1.

Similar to the sex model, the sex-specific age prediction uses 5q0 bins and smooths the random effect on the bin using 5q0. The prediction equation for age *y* in country in region *j* at time *t* for the age group 1 to 4 is seen below, with nested random effects on country ($\hat{\gamma}_{\mathrm{i}}$) and region ($\hat{\gamma}_{\mathrm{j}}$), an intercept term ($\hat{\beta}_1$), a smoothed random effect on 5q0 bin ($\hat{\gamma}'_{{}_5q_0\ \mathrm{bin}}({}_5q_{0_{\mathrm{jit}}})$), a coefficient on the under-5 crude death rate from HIV ($\hat{\beta}_2$), a coefficient on maternal education ($\hat{\beta}_3$), and a coefficient on completeness ($\hat{\beta}_4$):

$$\log(\Pr(\text{death in age } 1-4|\text{u5 death})_{\mathrm{jit}}) = \hat{\beta}_1 + \hat{\beta}_2 * HIV_{it} + \hat{\beta}_3 * Mat.Ed._{it} + \hat{\beta}_4 * 1 + \hat{\gamma}'_{{}_5q_0\ \mathrm{bin}}({}_5q_{0_{\mathrm{jit}}}) + \hat{\gamma}_{\mathrm{j}} + \hat{\gamma}_{\mathrm{i}}$$

Note that for prediction, the completeness coefficient gets multiplied by 1 instead of a source-specific completeness, as we seek to predict based on a hypothetically complete source. Similar the sex ratio model discussed earlier, these predictions are used as the first stage prediction for ST-GPR that produces sex and age specific probabilities of death.

Once each of these predictions is made by age group, they are rescaled such that the probabilities of death in the early neonatal, late neonatal, post-neonatal, and 1 to 4-year age groups aggregate to the 5q0 estimates from the under-5 model.

Section 2.2.13. Identify and remove outliers

There are several criteria for removing outliers for the under-5 age-sex pattern model. For the sex model, non-VR (survey) data points from high-quality VR locations (as determined by the GBD vital registration quality rating system) are outliered to ensure the model follows the highest quality data. Additionally, un-outliered sex ratio data were adjusted to be between 0.8 and 1.5. For the age model, the following outliering criteria were used. VR data that are considered incomplete are marked as outliers. To be considered incomplete, the 9-year rolling average of the VR data 5q0 value is compared to the 9-year rolling average of the 5q0 estimates. Then, for a given data-year, the value of 5q0 in the raw data is compared to our final 5q0 estimate. A value of 85% would be considered incomplete and outliered, unless the ratio of the 9-year rolling average above is above 85% complete. Any data that are chosen as outliers as part of the 5q0 analysis are also marked as outliers in the age pattern analysis. If a country has both VR and CBH data, they are typically both used, unless the two conflict, in which case the VR data are used. CBH data points more than 15 years before the survey are outliered. Lastly, some data points are manually outliered. For example, the definition of live birth changed in some Eastern European countries in the 1990s, leading to inconsistencies. In this case, age group data in ages that would include childbirth deaths (early neonatal, neonatal, and ages 1-4) are outliered if the definition of live birth contains a minimum weight, as it did in some of these locations.

Section 2.2.14. Under-5 age-sex splitting model application

The prediction method from the age-specific model is described above in section 2.2.12. First, the results of the sex model are applied, yielding sex-specific 5q0 estimates. Once age-sex-specific predictions of the log conditional probability of death are made, these are exponentiated and rescaled so that they sum to 1. First, the under-1 and 1-4 conditional probabilities are scaled to add to 1. Then, the early neonatal, late neonatal, and post-neonatal conditional probabilities are scaled to the under-1 conditional probability. Then, the probabilities of death can be calculated so that they properly

aggregate to the final 5q0 prediction. For example, to calculate the probability of death in the early neonatal age group, the rescaled conditional probability of early neonatal death given under-5 death is multiplied by the probability of under-5 death. Then, to obtain the probability of death in the late neonatal age group, the rescaled conditional probability of death in the late neonatal age group given under-5 death is multiplied by the probability of under-5 death and then divided by the probability of survival to the beginning of the age group, and so on. Equations below represent this process, where enn represents early neonatal and lnn represents late neonatal.

$$q_{enn} = \Pr(death\ in\ enn \mid u5\ death) * 5q0$$

$$q_{lnn} = \Pr(death\ in\ lnn \mid u5\ death) * 5q0/(1 - q_{enn})$$

The rest of the older age groups are also calculated in this manner, yielding probabilities of death in each of the under-5 age-sex groups.

Section 2.2.15. Update under-5 populations using fatal discontinuities

To obtain denominators for VR death numbers and to estimate death numbers for age groups under-5, we need to obtain age-specific populations for the under-5 age groups. Using final probability of death estimates including impacts of fatal discontinuities from the first run of the all-cause mortality process as the mortality risks, we take our input birth numbers and create person-year estimates of population as described in section 2.2.1. These person-year estimates are then the input as populations for the final run of the estimation process.

Section 2.2.16. Under-5 death number estimation

Assigning under-5 deaths to GBD age-sex groups

To estimate the number of under-5 deaths, we run an estimation process that ages birth cohorts through our estimated probabilities of death. This process separates our yearly birth numbers for each location into week-sized cohorts and ages each of these cohorts through our mortality estimates in week-long steps to estimate the number of person-years and deaths in each of the early neonatal, late neonatal, postneonatal, and 1-4 years age groups.

Section 2.3. Stillbirth Data and Estimation

Section 2.3.1. Data sources

Vital registration and government report data

Our search for stillbirth data from countries with vital registration systems began with GBD ICD-coded vital registration deaths database. In the GBD causes of death database, certain ICD codes are mapped to late foetal deaths and then discarded in the normal cause of death modelling process. We extracted those data, but found their coverage of stillbirths to be largely incomplete and lacking important information such as gestational age at time of foetal death. After the GBD causes of death VR data, our search continued with other multi-country sources, notably the WHO Europe Health for All database and UN Demographic Yearbooks. After extracting data from multi-country sources, we began sifting through single-country vital statistics reports published by national ministries of health or statistical offices. During the data seeking and extraction process, we were careful to note national differences and

changes in definition of stillbirth so as to adjust data to align with the international standard definition of foetal death after 28 weeks of gestation or at a foetal weight of greater than 1,000 grams.

The process of preparing stillbirth vital registration data for modelling is similar to that for other data-types and models. Depending on source and format of raw input data, a certain amount of data cleaning was required to extract usable stillbirth values and source-specific live births values. We also extracted source-specific neonatal mortality data from many sources.

Surveys

Stillbirth rates were extracted from USAID Demographic and Health Surveys, Reproductive Health Surveys, and UNICEF Multiple Indicator Cluster surveys.

Stillbirth rates were calculated for Demographic Health Surveys if microdata were available and if the microdata recorded contraceptive calendar information. Stillbirth rates from surveys were also calculated if a survey provided a complete birth history along with other stillbirth data, either in the form of a stillbirth option listed as an outcome in the complete birth history, or a total number of live births and stillbirths with a shared recall period.

If microdata were unavailable, stillbirth rates were instead extracted from survey reports where available.

Literature

We completed a literature review to supplement the literature sources listed in the Blencowe web appendix.[14] We modified the search string provided by Blencowe et al[14] to exclude the limitations on locations.

> ((stillbirth[MeSH Terms] OR stillbirth[All Fields] OR (still[All Fields] AND birth[All Fields]) OR still birth[All Fields]) OR (foetal death[All Fields] OR fetal death[MeSH Terms] OR (fetal[All Fields] AND death[All Fields]) OR fetal death[All Fields]) OR (perinatal mortality[MeSH Terms] OR (perinatal[All Fields] AND mortality[All Fields]) OR perinatal mortality[All Fields]) AND (timing[All Fields] OR rate[All Fields] OR epidemiology[Subheading] OR epidemiology[All Fields] OR prevalence[All Fields] OR prevalence[MeSH Terms] OR incidence[All Fields] OR incidence[MeSH Terms]))

We searched PubMed on March 3, 2016 and limited the results to January 20, 2015 through March 3, 2016 to bring the Blencowe search up to date. The initial query of PubMed returned 1,511 results which were reviewed based on their title and abstract. Of the 131 sources selected for full text review, 25 had usable data for stillbirth rate. We set broad exclusion criteria to include as many studies as possible. Sources were excluded if they covered a limited subpopulation (e.g., high risks mothers, or exclusively triplet pregnancies), if the study didn't provide information about stillbirth rate or was otherwise irrelevant (e.g., covered non-human populations), or if the study didn't contain primary data (e.g., meta-analyses). We extracted stillbirth rate and neonatal mortality rate from the 25 included sources.

Section 2.3.2. Stillbirth Modeling Strategy

We used a mixed effects generalized linear model to predict, in natural logarithmic space, the ratio of the stillbirth rate from the data sources described above to the neonatal mortality rate generated in the under-5 age pattern model discussed in section 2.2.12. The model included fixed effects on maternal education; random effects on location, location-source, and smoothed neonatal mortality classified into

20 bins; and dummy variables on stillbirth definition and data type in order to account for systematic differences in these variables. In order to account for a nonlinear relationship between neonatal mortality rate and the ratio between neonatal mortality rate and stillbirth rate, we ordered observations by neonatal mortality rate, split the observations into twenty bins with an equal number of observations, ran the model using the lme4 package in R, and put the resulting random effect estimates through a loess smoothing process to generate random effects for all neonatal mortality rates rounded to the hundredths place.

The model is below:

$$log\,(\frac{SBR}{NMR}) = (\beta_1 * education_{cy}) + \alpha_{definition} + \alpha_t + \gamma_{nmr} + \gamma_c + \gamma_{cs} + \epsilon_{cys}$$

Where c is country, y is year, s is source, and t is source type; each source was categorized into one of 5 source types across all countries; each observation was categorized into 8 definitions, as listed in the tables below. Additionally, *SBR* is stillbirth rate; *NMR* is neonatal mortality rate; *education* is mean years of education for women of reproductive age (15-49 years); *definition* is the stillbirth definition (see table below) β is a fixed covariate coefficient; α is a fixed effect dummy variable; γ is a random effect; and ε is the residual.

Table: Source classifications for stillbirth model

Stillbirth Data Source Type
Complete vital registration
Incomplete vital registration
Survey sources
Literature sources
Government reports

The completeness of VR systems was determined according to the child mortality completeness described in section 2.2.1.

Table: Definition classifications for stillbirth model

Stillbirth Definition	Adjustment factor – developed locations	Adjustment factor – other locations
Foetal death after 28 weeks of gestation	1	1
Foetal death after 26 weeks of gestation	0.86	0.89
Foetal death after 24 weeks of gestation	0.72	0.89
Foetal death after 22 weeks of gestation	0.68	0.89
Foetal death after 20 weeks of gestation	0.46	0.89
Foetal death where the foetus weighs at least 1,000 grams	1.15	1
Foetal death where the foetus weighs at least 500 grams	0.68	0.89
No definition given	0.68	0.89

For this analysis, we chose complete VR, if available, as the reference source in prediction. If no complete VR was available, we designated survey sources as reference, using the survey beta fixed effect and the average of the country-source random effects for each survey if there was more than one survey per location. For locations with neither complete VR data nor survey data, we used the average of complete VR and survey for the fixed effects and used a country-source random effect of 0. For locations with both complete VR and survey sources, we used the source for which we had the longer time-series of data as a reference in prediction. Going along with the WHO standard definition as well as the work of Blencowe et al.,[14] we designated 28 weeks as the reference definition.

As mentioned above, each data source has a source-specific random effect as well as a source-type fixed effect. The reference sources are designated above, and we adjust all the other sources by including these reference values for the random and fixed effects values instead of those estimated for each individual source. Going along with the work of Blencowe et. al.,[14] data are multiplied by an adjustment factor based on stillbirth definition designated in the table above. This first stage model is shown below.

$$\text{adjusted data} = \text{data} \times k_{def} + (re_{ref,cs} + fe_{ref,t} + fe_{ref}) - (re_{cs} + fe_t)$$

Where k is a definition – specific constant; fe is a fixed effect, re is a random effect; ref is the reference value of each fixed or random effect; def is the stillbirth definition; t is the source type; and cs is the country-source random effect. We use these adjusted data and the predictions without source type fixed effects or country source random effects as priors to input into our spatio-temporal smoothing, which is detailed in section 2.2.6. Briefly, for each country-year, we weighted data points in the region based on the proximity of those data points to the country-year in space and in time, giving a certain proportion of weight to in-country residuals and to in-region (out-of-country) residuals based on data density of each country. We designated λ, the parameter that designates how quickly the weights decrease with time, also based on data density of each location. To predict the residuals, we combined a Loess-like linear fit using weighted data and a weighted average of our data. The smoothed residuals are added back to the predictions generated without source type fixed effects or country source random effects in order to generate stage two predictions. We used Gaussian Process Regression (GPR) to generate the third stage final estimates. GPR was discussed in depth in section 2.2.6 of this appendix. The parameters used were selected based on data density of each country.

Section 2.4. Adult mortality

Section 2.4.1. Data sources

Adult population estimates

To calculate adult mortality rate using household death recall, age specific populations in age group 15 to 59 from the corresponding survey or census sources are used. This is also true in calculating adult mortality rate using reported deaths from Sample Registration System from India and Disease Surveillance Point system from China. For data from VR systems, we currently have two major sources for population in the corresponding age groups:

1. Population estimates from the World Population Prospect 2015 Revision by the United Nations Population Division. This provides majority of the population estimates used in GBD2015.
2. For the 37 countries covered by the Human Mortality Database, we use population exposure from this source instead of WPP2015.

For subnational locations, interpolation and extrapolation based on rate of change are used together with age specific population from censuses. Raking is applied to ensure consistency between subnational and national populations.

VR/SRS/DSP

See section 2.2 descriptions of VR sources for information on how VR, SRS, and DSP data are identified and prioritized.

Household recall of deaths

Household recall is ascertained from large survey series such as the Malawi 2010 DHS. A survey series must include a module asking about the number of deaths of household members within a given recall time period, along with a list of household members who have not died over this period of time. In addition, these survey series must be considered nationally representative, include survey weights (if applicable), and include the sex and age (either current or at death) of all household members.

Sibling survival histories

Data for sibling survival histories are primarily taken from large survey series in which respondents are asked about the status of their siblings, alive and dead. Some examples of sibling survival history sources include the Laos 2011-12 Multiple Indicator Cluster Survey and many DHS sources. To generate estimates of sibling survival, each questionnaire must contain a module with a full accounting of all siblings (children born to the same mother) of all respondents, along with data on the year of death (if applicable), sex, and age at death or year of birth. Additionally, the surveys must not have significant missingness in terms of responses to the sibling survival history module, as was the case in certain surveys such as the Mexico Health and Aging Study 2012.

Section 2.4.2 Completeness Assessment: Death Distribution Methods and completeness estimates synthesis

Vital registration systems may not capture all adult deaths. It is important to assess the quality, or in other words, the completeness, of available VR data. Demographers have long been applying a suite of death distribution methods (DDMs) including generalized growth balance (GGB), synthetic extinct generation (SEG), and a combined approach (GGBSEG) to assess completeness.[3,15–18] These methods compare the age distribution of the population recorded in two censuses with the age distribution of deaths recorded between these two censuses and attempt to estimate completeness of VR.
Recent modifications of these DDM methods provide estimates of completeness that, based on careful simulation studies, are more accurate and robust than traditional methods; nevertheless, these methods generate completeness estimates with substantial uncertainty intervals.[19]

For the GBD study, the process for estimating completeness of death registration for adults is based on estimates of adult completeness from three death distribution methods updated by Murray and colleagues in 2010 as well as information about child completeness.[19] These two sources of information are combined to generate a series of estimates of source- and country-specific adult death registration completeness from 1970 to 2015. The underlying assumption of this process is that completeness of systems will change gradually, and consequently, assessments of completeness for a given year should be informed by DDM estimates for prior and future years in that country. Further, completeness is likely to be similar among countries within a region, and we can inform estimates of completeness with levels of completeness estimated for countries in the same region or super-region by borrowing strength over space as well as time. To do this, we use a two-stage model, whereby we first predict adult completeness based on child completeness and then use a spatial-temporal regression model to incorporate information about adult completeness from the application of DDM methods.

Child completeness is calculated as the ratio of observed child mortality to estimated child mortality for a given source, country, and year. For a particular country-source, estimates of child completeness are only available for years where that data source is present, but a complete time series of child completeness estimates is produced based on a smoothing process. For country sources with no more than three years of data, a constant level of child completeness at the level of the mean of those years that are available is assumed. For country sources with more than three years of data, Loess regression is used to fill in the time series. In order to be conservative in our out-of-sample estimates of child completeness, instead of using the Loess predictions to forecast and backcast we simply hold child completeness constant before the first observation and after the last observation. Additionally, when there is a gap of more than five years we linearly interpolate between the observations on either side of this gap instead of using the Loess predictions to fill in these years.

Section 2.4.3. Sibling survival method

In countries where sources including vital registration and household death recall are scarce, sibling histories provide important information on the levels and trends of adult mortality rates. However, as studies have shown, estimates of adult mortality rate using sibling survival modules have significant biases resulting from the design and implementation of the method. Specifically, there are four different types of biases: selection bias (under representation of siblings from high mortality sibships), zero reporter bias (sibships not represented in the survey due to sex composition and/or mortality level of a sibship), sparse data, and recall bias (generally under reporting of death of siblings living in different places or have died a while back). For the Global Burden of Disease Study, we have greatly improved the methods used for sibling survival. The sibling survival technique employed in the current iteration of GBD is largely based on the work by Obermeyer et al.[20] with a few improvements to their methods: (1) use of appropriate survival weights that account for the study design; (2) implementation of a correction to account for the mortality experience of families not represented because none of the siblings were alive and eligible to respond to the survey; and (3) refinements for adjusting for recall bias. We validated these methodological developments in a range of simulation environments, and we have also developed ways of adjusting for recall bias and handling sparse data in survey designs where the age range of the respondents is narrower than the age range desired for estimation.[20]

Selection bias refers to the underrepresentation of high mortality sibships in the sample population—sibships with higher rates of mortality are less likely to be represented in the survey because fewer of them are likely to have survived to be selected into the sample. A method to correct for this underrepresentation, proposed by Gakidou and King,[21] incorporates a sibship-level weight, *Wj = Bj /Sj*, where *Bj* is the original sibship size and *Sj* is the number of siblings in sibship *j* who survive to the time of the survey. When each observation in the dataset being analyzed is at the sibship level, this Gakidou-King (GK) weight can be used to compute a weighted average of the proportions of siblings deceased as reported by each respondent. In the absence of any sibships where all siblings have died, this correction algebraically corrects for the underrepresentation of high-mortality sibships in the survey sample.

When the dataset is expanded to the sibling level (i.e., one observation for each sibling as opposed to sibship), the number of observations listed in the dataset for each sibship corresponds to the original sibship size, *Bj*, and so the numerator of *Wj* is already accounted for. The resulting sibling-level weight is therefore *Wi* = 1/*Sj* for sibling *i* in sibship *j*.[22,23]

Since the analysis reported here is carried out at the sibling level, we use *Wi* rather than *Wj*. This improves on previous applications of the method where the sibship-level weight was inappropriately applied to data that had been expanded to the sibling level.

Further, the number of surviving siblings in the family must also be tailored to the eligibility criteria for respondents of the given survey.[22] In applying Gakidou and King's elucidation of the survivorship correction, *Sj*/*Bj* represents the probability that a sibling in sibship *j* survives and is eligible to be selected in the survey. For Demographic and Health Surveys (DHS), respondents must be women between the ages of 15 and 49 and so the *Sj* in this case would be the number of surviving women in a sibship *j* who are between the ages of 15 and 49 at the time of the survey. In this analysis, the value of *Sj* has been chosen to be consistent with the eligibility criteria of each survey.

The sampled population excludes sibships in which there are not any eligible siblings to respond to the surveys; thus, we cannot report on the mortality experiences of these siblings. The zero-survivor correction estimates the number of sibling deaths that are missing from the sample by age and sibship size, and then adds these siblings to the observed sample before calculating age-specific mortality rates. This correction is applied to sibships with one or two females. The correction uses the relationship between the true number of sibships with one (or two) females and the cumulative probability of dying before the time of the survey to estimate the number of missing sibling deaths. For one-sibling sibships,

$$K^1_{obs} = K^1_{true} * (1 - {}_{a}q^1_0)$$

$$K^1_{miss} = K^1_{true} * {}_{a}q^1_0$$

Where K^1_{obs} is the number of sibships with one sister that are observed in the sampled population; K^1_{true} is the true number of sibships with one sister in the population; K^1_{miss} is the number of sibships with one sister that are not represented in the sampled population due to zero-survivor bias; ${}_{a}q^1_0$ is the

cumulative probability of death for five-year age-group *a*; and $(1 - {}_{a}q_{0}^{1})$ is the probability that the sister has survived to the time of the survey.

From these two equations, it follows that the number of sibships with only one sister that are not represented in the population due to zero-survivor bias is equal to:

$$K_{miss}^{1} = \frac{K_{obs}^{1}}{1 - {}_{a}q_{0}^{1}} * {}_{a}q_{0}^{1}$$

We multiply this estimate of the number of missing sibships by the number of females in the sibship (which in this case is one) to get an estimate of the number of females in each age group that are missing from the sample because they have died. We then expand this number so that we have one observation per missing sibling, assign birth and death dates to these missing siblings based on the distribution in the observed siblings, and append them to our existing dataset. This process is also carried out for families with two sisters:

$$K_{obs}^{2} = K_{true}^{2} * (1 - {}_{a}q_{0}^{1} * {}_{a}q_{0}^{2})$$

$$K_{miss}^{2} = K_{true}^{2} * {}_{a}q_{0}^{1} * {}_{a}q_{0}^{2}$$

$$\therefore K_{miss}^{2} = \frac{K_{obs}^{2}}{1 - {}_{a}q_{0}^{1} * {}_{a}q_{0}^{2}} * {}_{a}q_{0}^{1} * {}_{a}q_{0}^{2}$$

Where K_{obs}^{2} is the number of sibships with two sisters that are observed in the sampled population; K_{true}^{2} is the true number of sibships with two sisters in the population; K_{miss}^{2} is the number of sibships with two sisters that are not represented in the sampled population due to zero-survivor bias; ${}_{a}q_{0}^{1}$ is the cumulative probability of death for the first sister in five-year age-group *a*; ${}_{a}q_{0}^{2}$ is the cumulative probability of death for the second sister in five-year age-group *a*. If there is only one sister within the 15 to 49 age range, the equations are different than above because the second sister does not contribute to the probability of the sibship being observed in the sample:

$$K_{obs}^{2} = K_{true}^{2} * (1 - {}_{a}q_{0}^{1})$$

$$K_{miss}^{2} = K_{true}^{2} * {}_{a}q_{0}^{1} * {}_{a}q_{0}^{2} + K_{true}^{2} * {}_{a}q_{0}^{1} * (1 - {}_{a}q_{0}^{2})$$

$$\therefore K_{miss}^{2} = \frac{K_{obs}^{2}}{1 - {}_{a}q_{0}^{1}} * {}_{a}q_{0}^{1} * {}_{a}q_{0}^{2} * \frac{K_{obs}^{2}}{1 - {}_{a}q_{0}^{1}} * {}_{a}q_{0}^{1} * (1 - {}_{a}q_{0}^{2})$$

Both this analysis and Obermeyer et al. take into account time prior to the survey in the logistic regression to model mortality.[20] This current analysis, however, provides an updated method for recall bias adjustment. After 45q15 has been estimated for each of the four surveys, the estimates are combined and paired up for all periods where they overlap. This overlap occurred when there were at least two surveys carried out in the same country within 15 years of each other. In GBD 2013, we estimated adult mortality from sibling histories for three five-year periods prior to the survey date. This was changed in GBD 2015 – and continued for GBD 2016 – where single-year 45q15 estimates from sibling survival were generated using the same methodology to account for the changing level and

trends of 45q15 within the 15-year period covered by sibling survival module. This generates pairs of estimates in years where there are overlapping surveys. For each of these pairs, we calculate the difference in the years of recall as the interval between when the two surveys were conducted and we also calculate the magnitude of the difference between the two estimates of 45q15. We then estimate the linear regression model shown in the below equation to quantify the relationship between years of recall and level of mortality separately for each sex of sibling:

$$\Delta({}_{45}q_{15})_{i,j} = \beta * \Delta(\text{survey date})_{i,j} + \xi$$

Where $\Delta({}_{45}q_{15})_{i,j}$ is the difference in 45q15; and $\Delta(\text{survey date})_{i,j}$ is the difference in survey date for survey pair *j* in country *i*. Upper and lower uncertainty intervals were also derived. The coefficient on recall period represents the effect of recall bias and was used to adjust the 45q15 estimates to account for that bias.

Section 2.4.4. Completeness data synthesis

Once we have obtained a full series of under-5 completeness estimates for each country source we fit the model described in the equation:

$$\log_{10}\left(c_{i,s,t}^{adult}\right) = \alpha + \beta_1 * \log_{10}\left(c_{i,s,t}^{child}\right) + \gamma_1^{SR} + \gamma_2^{SR} * \log_{10}\left(c_{i,s,t}^{child}\right) + \gamma_1^{R} + \gamma_2^{R} * \log_{10}\left(c_{i,s,t}^{child}\right) + \eta_{i,s} + \xi_{i,s,t}$$

Where $c_{i,s,t}^{adult}$ is completeness of adult deaths registration in country *i*, source *s*, at time *t*; $c_{i,s,t}^{child}$is completeness of child deaths registration in country *i*, source *s*, at time *t*; γ terms are random effects at the region (R) and super-region (SR) level; $\eta_{i,s}$ is a random effect at the country and source level; and $\xi_{i,s,t}$ is an error term. This model relates adult completeness to under-5 completeness and includes super-region and region-level random effects to allow for differences in both the average level of adult completeness and the relationship between child and adult completeness at these levels. The country-source random effect captures the fundamental difference in level of completeness between different data sources.

A $\log_{10}$ transformation is employed to make over- and under-completeness symmetric (e.g., 50% complete and 200% complete are symmetric around 0 when $\log_{10}$ transformed) and to simplify calculation of the variance of completeness estimates in $\log_{10}$ space, which is needed for the adult mortality estimation process. Also, to avoid allowing outlying DDM-derived estimates of adult completeness from unduly influencing the predictions from the model in the equation above, for any given set of three DDM estimates (GGB, SEG, GGBSEG) calculated from a single pair of censuses, the estimate that is furthest from 1 (i.e., complete) is excluded.

For each country-source $\log_{10}(c_{i,s,t}^{adult})$ is predicted from coefficients estimated in the model above and child completeness. Not every country can be used to fit this model as DDM cannot be applied in some cases due to lack of appropriate census data. However, because the coefficients used for prediction are at the region and super-region level, predictions from this model can be generated for all countries where estimates of child completeness are available. We do not believe that the same relationship between adult and child completeness exists for registration-based sources as for recall-based sources,

so the above model is applied only to registration-based sources (primarily VR data but also sample registration systems). For sources that only include household death recall, we set an arbitrary value of 1 for the first stage values instead of making predictions from completeness from child age groups for the aforementioned reason. However, it should be noted that this set value by no means reflects the true completeness of adult age groups in the household death recall sources and it will change once we apply the spatial-temporal regression in the second-stage estimation.

In the second stage, we calculate the residuals from the first stage and apply spatial-temporal smoothing to these residuals. The predicted residuals are then added back onto the first-stage predictions, generating the second-stage predictions. Spatial-temporal smoothing is carried out in the same way as in the adult mortality estimation process with three modifications: the λ and ζ parameters are set to 2.0 and 0.95; only the fixed effect local regression variant is used; and the residuals are not held constant out of sample. The registration-based sources and the non-registration-based sources are handled separately in this step.

The variance of the completeness estimates must also be calculated, as this information is utilized in the adult mortality estimation process. To do this, we approximate the variance based on the median absolute deviation (MAD) compared to the second-stage estimates. We calculate variances at the regional level and do so separately for registration-based sources and other sources. Then, for each country-source-year, we generate 10,000 simulations from a normal distribution with mean equal to the second-stage prediction for that year and variance calculated as just described. For non-registration-based sources we believe that both under- and over-reporting are possible (over-reporting may occur due to telescoping of events outside of the recall period into the recall period), and so for these sources we now exponentiate the 10,000 simulations and find the mean, which serves as the final prediction for completeness. For registration-based sources we believe that only underreporting is possible, so for these sources we first truncate any simulations above 1 to 1 and then exponentiate the 10,000 simulations to find the truncated mean, which will serve as the final prediction for completeness. In both cases, before exponentiating the simulations, the variance of the simulations is calculated, and used as the variance of the completeness estimates in the adult mortality estimation process.

The final completeness estimates are used to adjust, where appropriate, the corresponding country-source-years before these data are used in the adult mortality estimation process. For countries in which we believe males and females have differential completeness, we carry out the above process separately by sex. For a small number of data points, completeness cannot be estimated using the procedure described above due to a lack of appropriate census data, and the original growth balance method is the only viable option.[12] In previous papers, we have included a selected number of data points derived from household recall of deaths to which the Brass growth balance method had been applied. Our simulation studies suggest this method is extremely imprecise, so we have excluded these points from the analysis. This accounts for why the number of household recall of deaths data points has decreased in this analysis compared to Lozano et al.[24]

Section 2.4.5. 45q15 data synthesis using non-linear mixed effects model and ST-GPR

Overview of adult (45q15) mortality estimation

For each country, we generated a time series of 45q15 estimates. We modeled the underlying mortality risk separately from excess mortality due to fatal discontinuities. To model the underlying mortality risk we relied on a three-stage process that incorporates all data in our database for each country after excluding data identified as outliers or that refer to years identified to contain mortality shocks from conflict or natural disaster. In the first stage, we applied a nonlinear mixed effects model which used covariates to explain variation in 45m15. In the second stage, we exploited spatial and temporal correlation in the residuals from the first stage regression by performing a smoothing process on these residuals. The smoothed residuals were then added back into the first stage regression predictions to produce an updated time-series of 45q15 for each country. In the third stage, we applied Gaussian process regression (GPR) which synthesizes information from the second stage predictions and the observed data. After applying this procedure to generate estimates of the underlying mortality risk, we modeled abrupt changes in mortality by estimating the excess risk of mortality in years identified as containing a conflict or natural disaster. This estimated excess mortality risk was then added to the underlying mortality risk to produce our final time series of 45q15 estimates. All analyses were carried out in Stata 13.1, R 3.0.2, and the PyMC package, version 2.0, in Python 2.5.4. A more complete description of the various stages of our modeling strategy is given below.

First stage nonlinear mixed effects regression

The first stage nonlinear regression models country/year/sex-specific adult mortality rate using key covariates: lag-distributed GDP per capita, mean years of education in age group 15 to 59, and crude death rate due to HIV/AIDS in age group 15 to 59. We use the nonlinear mixed effects model specified in the equation below. This model was fit separately for males and females.

$$ {}_{45}\mathrm{m}_{15}^{\mathrm{observed}} = \exp(\beta_1 + \beta_2 \cdot \mathrm{Edu} + \beta_3 \cdot \ln(\mathrm{LDI}) + \gamma_{\mathrm{country}}) + \beta_4 \cdot \mathrm{HIV} + \mathcal{E} $$

Edu is the mean years of education for the age group 15 to 59; LDI is lag-distributed income; $\gamma_{\mathrm{country}}$ is a country-level random effect; and HIV is the crude mortality rate from HIV for ages 15 to 59. We initialized the model with starting values for each of the β coefficients equal to 0. We tested our model with different starting values, including values from a hierarchical linear mixed effects model, and found that this model was not sensitive to starting values.

The final stage one predictions were based on predictions from the model, the above equation excluding the country random effect. We excluded the country random effect to facilitate modeling spatial trends in mortality in the second stage. The model predictions were then converted from 45m15 to 45q15 to be put into the second stage model described next.

Second stage spatial-temporal smoothing of residuals

The first stage regression model reflects the explanatory power of the set of covariates but, as might be expected, fails to explain all of the variation in 45q15. The residuals from the first stage regression are correlated in both time and space, indicating that 45q15 is correlated in time and space in ways that are not fully captured by the covariates included in this regression. We exploited this remaining pattern of

variation by applying a local regression to the residuals from the first stage regression, effectively smoothing across time and space.

The local regressions were fitted separately for each of the 21 GBD regions which have been constructed so that countries within each region share similar epidemiological profiles.[25] We applied two variations of local regression to the first stage residuals, both of which utilized the same weighting scheme to incorporate temporal and spatial relatedness. In both variants, a set of weighted linear regressions, one for each country-year of interest, were undertaken. When carrying out the regression for a given country-year of interest, all residuals in the dataset were weighted with respect to this country and year. We first weighted residuals with respect to time using a weighting function similar to that utilized in Loess regression:

$$w_t = \left(1 - \left(\frac{|r_t - r_{est}|}{1 + \text{argmax}_t|r_t - r_{est}|}\right)^{\lambda}\right)^3$$

The r_t and r_{est} terms are, respectively, the year of interest and the year of the residual being weighted. The $\text{argmax}_t|r_t - r_{est}|$ term is the maximum distance between the year of interest and a residual within the region. The λ parameter in this weighting function dictates how quickly the weights fall off as the distance in time increases: a larger λ implies that the assigned weights will diminish slowly with time, while a smaller λ allows the weights to diminish more rapidly with time.

We then weighted residuals with respect to space by modifying the time weights described below. Weights for residuals within the country of interest were multiplied by a factor of:

$$\frac{\zeta \sum_{i \notin c_{est}} w_i}{(1-\zeta) \sum_{i \in c_{est}} w_i}$$

where c_{est} is the country of interest and w_i are the time weights described above. As a result of this modification, 100·ζ % of the total weight was placed on residuals within the same country, and the remaining 100·(1-ζ) % of the weight was placed on residuals from other countries in the region. For countries with no residuals (i.e. countries with no data) the above factor is 0 and there was no re-weighting: all of the weight consequently remains, by necessity, in other countries in the region. We set λ and ζ based on data density of each location.

The first local regression variant, which we call linear local regression, is described below and is a weighted linear regression of the residuals on year and an indicator of the residual from the country currently being estimated (if this indicator cannot be estimated, i.e. because there are no residuals in a given country, it is dropped):

$$r_{est} = \beta_0 + \beta_1 t + \beta_2 c_{est} + \mathcal{E}$$

The second variant, which we call fixed effects local regression, used a weighted linear regression with no covariates. This is equivalent to a simple weighted average of the residuals.

Linear local regression incorporates information from covariates, in this case year, but extrapolation is heavily based on this covariate and in settings with sparse data this can result in implausible out-of-

sample predictions. In contrast, fixed effects local regression does not incorporate an explicit time-trend and does not suffer from this problem in extrapolation, but also is less adequate at fitting the data in countries with many observations. We therefore chose to combine the estimates from both variants. As described in the below equation, we calculated the data density, d_c, for each country we estimated, c_{est}, and then calculated a weighted average of the predictions from the linear local regression and fixed effects local regression where d_c% of the weight is assigned to the linear local regression and the rest to the fixed effects local regression. In this way, the final estimates for countries with more VR data are more heavily informed by the linear local regression and the final estimates for countries with less (or no) VR data are predominantly informed by the fixed effects local regression, as is appropriate given the strengths and weaknesses of these two variants.

$$\mathrm{d_c} = 100 * \left(\frac{\text{\# VR points in } \mathrm{c_{est}}}{\text{Maximum \# VR points in any country in the region}}\right)$$

Residuals were logit-transformed before undergoing this smoothing; once the final estimates of the smoothed residuals were obtained for every country-year, these estimates were added back into the logit transform of the first stage regression predictions. This sum was then reverse-logit transformed; by carrying out the first two stages in logit-space we restricted the predictions to between 0 and 1. These predictions are called the second-stage predictions.

Model

The third stage of our prediction method is a Gaussian process regression (GPR) based on the model given in below where μ_t is the true $\log_{10}$(45q15) at time *t*, *f(t)* is the baseline mortality risk, and S_t captures excess mortality due to war and disasters. S_t is estimated independently of *f(t)* as described in a later section. *M* and *C* describe the Gaussian process, giving the mean and covariance, respectively.

$$\mu_t = f(t) + S_t$$

$$f(t) \sim GP(M, C)$$

For the Dominican Republic, Peru, and Madagascar, a slightly different model, described in the equation below, is used. For these countries, measurements from sibling histories and from VR are at different levels and the direction of the bias in each source is unknown. We therefore used a model which includes a bias term for each source (β_S).

$$\mu_t = f(t) + \beta_s + S_{t,s}$$

$$\beta_s \sim \mathrm{Normal}(0, 0.01^2)$$

$$f(t) \sim GP(M, C)$$

Gaussian process regression is a method of Bayesian inference. We specified a prior distribution for *f(t)*, and a likelihood function which describes the data generation process; the specified prior distributions and likelihood function are described in subsequent sections. We then used Markov Chain Monte Carlo (MCMC) to approximate the posterior distribution of *f(t)* which also incorporates information from the observed empirical estimates of adult mortality. An MCMC chain of length 5,000 was produced; the first 3,000 samples were discarded and the remaining 2,000 were thinned by a factor of 2 for a total of 1,000

simulations retained. The reported best estimates and confidence intervals were generated from the mean and the 2.5th and 97.5th percentiles of the 1,000 samples, respectively.

Priors

The prior distribution of *f(t)* can be described in terms of the mean prior—the prior for *M*—and the covariance prior—the prior for *C*. We utilized the second stage predictions as the mean prior and used a Matérn covariance function to describe the covariance prior. This covariance function incorporates three parameters: the amplitude, which controls the amount by which realizations of the Gaussian process distribution can deviate from the mean function, the scale, which controls the distance over which the function is correlated, and the degree of differentiability, which influences the smoothness of the samples from the Gaussian process. These parameters are selected in the parameter selection process described below.

Likelihood

The likelihood describes the probability of observing the data given a particular set of parameters. As shown in the below equation, we used a normal model for describing the probability of observing a particular value of log(45q15) where the mean is given by *f(t)* and the variance by V_t, the data variance.

$$\log_{10}({}_{45}q_{15_t}) \sim \mathrm{Normal}(f(t), V_t)$$

Data variance was calculated for each empirical observation of 45q15 and incorporated both sampling and non-sampling variation. The method for calculating the data variance depended on the type of data. We computed sampling variance from a binomial model. We set *N* equal to the national population aged 15 to 59 years and *p* equal to the mortality rate, 45m15. We calculated the variance of 45m15 from *p(1-p)/N* and then transformed this to the variance of $\log_{10}$(45q15) using the delta method. For estimates derived from both complete and incomplete VR data, we wanted to include not only sampling variance but also the non-sampling variance that arises from uncertainty in the completeness estimate. For these data, the total data variance was given by the sum of the sampling variance (calculated as for complete VR data) and the variance of the completeness estimate (calculated as described in section 2.2.6).

For estimates derived from sibling history, census, or survey data, the median absolute deviation (MAD) estimator of the variance was calculated by source type with reference to the second stage predictions, as described in the equation, where *s* is the source type (sibling history, census, or survey):

$$\begin{aligned}\theta_s^2 &= (1.4826 \cdot \mathrm{MAD}_s)^2 \\ &= 1.4826 \\ &\cdot \mathrm{median}\left(\left|\left(\log_{10}\left({}_{45}q_{15}^{\mathrm{observed}}\right) - \log_{10}\left({}_{45}q_{15}^{\mathrm{predicted}}\right)\right)\right.\right. \\ &\left.\left. - \mathrm{median}\left(\log_{10}\left({}_{45}q_{15}^{\mathrm{observed}}\right) - \log_{10}\left({}_{45}q_{15}^{\mathrm{predicted}}\right)\right)\right|\right)^2\end{aligned}$$

Hyper-parameter selection for adult mortality rate ST-GPR

Similar to the new parameter selection process for U5MR, we have also expanded the scope of our parameter selection for adult mortality rate (45q15) to include variables used in space-time smoothing.

The out-of-sample predictive validity testing used to select space-time and GPR parameters for adult mortality rate follows.

For space-time smoothing, we used ζ values based on data density, assigning higher values to locations with more years of data. We tested λ values of 0.1 to 0.9 in increments of 0.1. We test five values of the scale—5, 7.5, 10, 12.5, 15, 17.5, 20 -- and five values of the squared amplitude—1, 1.5, 2, 2.5, and 3 times the mean squared error of the residuals from the second-stage prediction model.

We divided the data into testing and training sets 100 times. Data were divided as follows: for each region, a number X between 10 and 20 was sampled and the most recent X years of data in that region were assigned to the testing set. Then a number N between 5 and 10 was sampled, a country from within the region was sampled, and a year where there is data in that country was sampled. All data within N years of the selected year in the selected country were assigned to the testing set. This was repeated as many times as there are countries in the region; because iterations of this procedure were independent, the data selected for the testing set may overlap. Any data that were not selected for the testing set were included in the training set.

We then ran Space-time smoothing and Gaussian process regression with the training data set using each set of parameters being tested to generate estimates for each location and every year in both the training and testing data set.

Within a given iteration we calculate the absolute relative error of the GPR estimate compared to each empirical estimate in the testing set. We also classify each empirical estimate in the testing set as covered or not covered. The determination of coverage is made by calculating total variance—the sum of the data variance and the variance of the GPR estimate—and then calculating a 95% uncertainty interval around the GPR estimate based on this total variance and assuming a normal distribution. If the empirical estimate is within this uncertainty interval, it is classified as covered, and otherwise not. For each combination of parameters, we calculate the mean absolute relative error and the mean coverage across all iterations from all countries within a particular group. The loss function described below is then calculated for each parameter combination, and the parameter combination with the lowest loss is selected for each group;

$$\text{loss} = (0.95 - \text{coverage})/5 + (\text{absolute relative error})$$

Countries with at least 20 years of VR data were assigned their own parameters. Other locations were grouped based on how much and what type of data they had available. A set of parameters was then applied to all locations in that group. A summary of these groups is in the following table:

Table. Parameter Groups

Group	Conditions
Sparse data, with complete VR only	Only complete VR, more than 10 years of VR, with VR after 1970
Sparse data, VR only	At least some incomplete VR, more than 10 years VR, with VR after 1970
Sparse data, VR +	At least some incomplete VR, with either fewer than 10 years of VR or all VR before 1970
Sparse data, other	No VR or sibling data
Sibling data, small	No VR, less than 21 years of sibling data
Sibling data, large	No VR, more than 21 years of sibling data
No data	No data

For countries with no data, the highest of all selected scales and squared amplitudes from among all other countries was used.

In some cases, we restricted the universe of possible parameters. Most of these restrictions occurred for the λ parameter. For many locations with complete VR, we knew that higher lambda values would result in a larger confidence interval than would be appropriate. We also included a lower limit of λ for some locations with either incomplete VR or no VR, so that confidence intervals would reflect the uncertainty of these data. For example, in locations where we only had sibling history data, we restricted lambda to being greater than 0.5. We also made some other manual exceptions to λ, ζ, and scale where results did not pass the common sense test.

We set the differentiability to 0.8 in countries with only complete VR data, excepting those in the Caribbean, Oceania, and the country of Mauritius, and to 2.0 in other all other locations. We used a lower differentiability in countries with complete VR data because in these countries we want the final estimates to follow the data closely even if the trend described is not smooth. In contrast, in countries where the data are less reliable we don't want the final estimates to be overly influenced by individual data points.

Accounting for the uncertainty in HIV covariate in the first stage model for high HIV/AIDS burden locations

To account for the uncertainty in estimated crude death rate due to HIV, a key covariate for the first stage model described in section 2.6, we generated draw level crude death rate due to HIV and repeated the first stage model and ST-GPR processes described in section 2.4.5 250 times using 250 unique draw level crude death rate due to HIV derived from our EPP-Spectrum process. We repeated this process 250 times to balance between computation intensity and obtained increases in the estimated uncertainty interval for 45q15. For each one of the 250 ST-GPR runs, four draws were selected out of 1,000 to form the final 1,000 draws of 45q15 for each location over time.

Section 2.4.6. Identify and remove outliers

To arrive at sensible level and trends in adult mortality rate, certain outliers were excluded from our ST-GPR regression process. In general, we used the following process to outlier influential raw data points that otherwise lead to erroneous without fatal discontinuity 45q15 estimates:

1. Raw input 45q15 data points from years affected by war, natural disasters, and other fatal discontinuities as defined in GBD 2016 are excluded from the analysis described in section 2.4.
2. Examination of survey/registration data quality leads certain raw data to be outliered. Such examples include the Afghanistan 2010 Mortality Survey that was not nationally representative, and some of the VR data from Serbia that didn't include deaths from Kosovo.
3. Visual inspection of raw input data on 45q15 and estimated time series estimates of 45q15 from ST-GPR by GBD researchers and country experts through the GBD collaborator network. Data points are outliered when it is unexplainably different from other adjacent points from the similar source indicating a data reporting issue and compilation error in the direct sources where such data was obtained. Some subnational level single year 45q15 estimates from sibling survival methods are excluded for unreasonably high or low estimates due to small sample sizes.

Section 2.4.7. Rake subnational estimates to national level (excluding South Africa)

First, we randomize the order of the 1,000 subnational-level draws and the national draws separately to avoid any correlation between subnational and national draws that may have been introduced in prior processes.

GBD 2016 provides estimates of 45q15 for 754 locations at both subnational and national level. While it is absolutely essential to use input data from the subnational level in informing the level and trend of 45q15 for the corresponding location, it is often the case that we tend to have more robust data at the national level, more data sources and data sources that cover longer time periods. To have consistent estimates between aggregated subnational level estimates and the separately estimated national level estimates, we rake the subnational 45q15 to match our national level 45q15 estimate by using the following formulas:

$$ {}_{45}q_{15}^{s'} = 1 - e^{-45 \cdot {}_{45}M_{15}^{S} \cdot r} $$

and

$$ r = \frac{\ln(1 - {}_{45}q_{15}^{N}) / -45}{\sum_{s=1}^{n} \ln(1 - {}_{45}q_{15}^{s}) \cdot \frac{p_s}{P_N} / (-45)} $$

In the above equations, s refers to subnational locations within a country N, p is population in age group 15 to 59, and 45q15 are estimates of adult mortality rate from the ST-GPR process, and ${}_{45}q_{15}^{s'}$ is the post-raking 45q15 for subnational locations.

Section 2.4.8. Review estimates for quality

The preliminary estimates of adult mortality rates are reviewed by both the researchers who work on the demographic estimation process and the GBD all-cause mortality collaborator network. Concerns

regarding quality of certain survey and data points are raised and reviewed which lead to revision of the database where applicable.

Section 2.4.9. 45q15 estimates with HIV

The results of the adult mortality rate ST-GPR process are estimates of probability of death from all causes of death except fatal discontinuities for all locations covered in GBD 2016 for 1970 to 2015.

Section 2.4.10. HIV-free 45q15

As a result of the non-linear mixed effects model used in the first stage before ST-GPR, we also generate HIV-free counterfactual 45q15 by removing the impact of HIV, as specifically measured by crude death rate due to HIV/AIDS estimated in the EPP-Spectrum process, from all-cause 45q15 without fatal discontinuity.

Section 2.5. Model life table system

Section 2.5.1. Overview

For many purposes, estimates of age-specific mortality rates are useful. In settings without complete VR systems, data are often available for some summary measures of mortality such as under-5 mortality rate (henceforth 5q0) from complete or summary birth histories and mortality from ages 15 to 60 from sibling histories (45q15, and commonly described as adult mortality rate). Model life tables, which are structured relationships between levels of age-specific mortality at different ages, can be used to generate an estimate of age-specific mortality for detailed age-groups from summary measures of mortality on children and adults (5q0 and 45q15, respectively). Model life tables have many other applications as well, such as simplifying the task of mortality projection or updating life tables from the most recent empirically observed data. This is true even for countries with good VR systems due to the fact that compilation and publication of VR data may take years.

An ideal model life table system has several desirable attributes. First, a model life table system should be parsimonious and require only a few entry parameters to generate a full life table with age specific mortality rates. Second, it should adequately capture the range of age patterns of mortality observed in real populations and yield high predictive validity, not just measured by summary indices such as life expectancy at birth, but more importantly by age-specific mortality rates. Third, it should provide satisfactory estimates of age-specific mortality for countries with high levels of mortality, especially those with substantial HIV/AIDS epidemics. Finally, a model life table should generate age-specific mortality with a plausible time trend, and the partial derivative of age-specific mortality should be positive with respect to entry parameters such as 5q0 and 45q15.

A simple and somewhat flexible model life table system was proposed by Brass.[16] He observed that in general the logit of the *lx* (proportion of a hypothetical birth cohort still alive at age x) column in a life table could be represented as a linear transformation of the logit of the *lx* column of a reference standard life table. Murray et al. noted that as a population moved further away from the levels of mortality in the reference standard, the assumption of linearity in logit *lx* space was violated.[26] A series of age-specific modification factors were proposed that allowed for the characteristic bending of the logit *lx* function compared to the standard. Murray et al. also assessed the ability of this modified logit life table system to predict age-specific mortality rates. The modified logit life table system with its built-

in optional mechanism of predicting adult mortality (45q15) from child mortality (5q0) has been extensively used by the World Health Organization since the early 2000s.

This system, however, suffers from two major limitations. First, when adult mortality is very high relative to child mortality, such as in the presence of an HIV/AIDS epidemic, the age patterns of mortality generated do not fit well to the observed data. Second, when the modified logit life table system is applied to time series of 5q0 and 45q15, paradoxical trends can be generated where adult and child mortality are declining, but predicted age-specific death rates in some age groups are increasing. We have extended the modified logit life table system to deal with these two major limitations. Wang et al. provide a more complete discussion of the development of this approach.[2,4] We summarize these developments in brief here. There are four distinct steps.

Section 2.5.2. Building empirical model life table database: data sources and quality review

Relational model life table systems critically depend on the empirical database used to generate model life table standards and test the predictive validity of model life tables. For both all-cause mortality and cause-specific mortality analyses in GBD, we have amassed a comprehensive database on human mortality from censuses, VR systems, sample VR systems such as the SRS from India, the Disease Surveillance Point system from China, and the Human Mortality Database. These aforementioned data sources provide a total of 52,149 empirical life tables. After removing duplicated empirical life tables between Human Mortality Database and VR sources, we have closely examined every single empirical life table by applying the following quality control process to further remove life tables with irregular/impossible age pattern of mortality:

a. Empirical life tables with irregular age pattern of mortality such as probability of death in age group 1-4 higher than first year of year are dropped;
b. Empirical life tables where age pattern of mortality doesn’t conform to Gompertz law of mortality between age 40 and 80 are dropped.

Murray et al. used a database of 3,566 life tables covering 63 countries.[26] We have expanded this database to include 15,221 empirical life tables. Two major data sources provide about 97.0% of these life tables. A total of 4,130 life tables from the Human Mortality Database (HMD)[27] dating between 1950 and 2015 are included in our database. We excluded life tables of poor quality and those from years affected by wars and pandemic. The other major source of empirical life tables is the collection in which VR data have been evaluated using death distribution methods, as revised by Murray and colleagues (2010) and Wang et al.[4,19]

It is our view that accuracy of the age pattern of mortality in the raw data is to a certain degree correlated with the completeness of the data. We did make certain exceptions for the purpose of including age pattern of mortality from regions that otherwise won’t be represented in our database with the strict completeness threshold. In all cases, rigorous assessment of the quality of these data is conducted. In total, we included over 10, 000 life tables from various VR sources including subnational VR from Mexico, Brazil, and United States, Disease Surveillance Point system, and sample registration system that are adjusted by GBD’s completeness assessment process as detailed in section 2.1. in addition, in order to provide better fit to places with high quality VR data but age pattern of mortality

has suffered from the small number issues, we have included additional empirical life tables that are only used in estimating age pattern of mortality for the geographies the life tables are from. This adds another 16953 empirical life tables mostly for subnational locations with small population size.

In building the empirical life table database for the model life table system used by GBD, we examined additional data sources that we decided to exclude from our database. These included VR sources deemed incomplete and life tables with implausible patterns or high levels of stochastic fluctuation among age groups.

In addition, all life tables in our database with crude death rate due to HIV/AIDS in adult age groups over 0.1% are excluded in the process described in section 2.5.1.

Section 2.5.3. Extending age-specific mortality to age 100+

To extrapolate age-specific mortality beyond age 85, the Gompertz law of mortality and other functional model age pattern of mortality methods are generally used.[8,28] Here, we have developed a new model with better predictive validity than existing methods. Age-group dummies and probability of dying from age 80 to 84 in logarithmic scales are used to estimate the difference in age-specific probability of dying in logit scale between two consecutive age-groups, as described in the following equation:

$$\text{logit}\left({}_{5}q_{x}^{\mathrm{j,t,g}}\right) - \text{logit}\left({}_{5}q_{x+5}^{\mathrm{j,t,g}}\right) = \alpha^{\mathrm{g}} + \beta_{\mathrm{x}}^{\mathrm{g}} \cdot \text{age} + \gamma^{\mathrm{g}} \cdot \text{logit}\left({}_{5}q_{80}^{\mathrm{j,t,g}}\right) + \eta_{\mathrm{j}}^{\mathrm{g}} + \xi_{\mathrm{x}}^{\mathrm{j,t,g}}$$

Here, *j* refers to country, *g* refers to sex, and *t* refers to time. Parameters are estimated using data from selected countries in Human Mortality Database with high quality VR data in the oldest old age groups above age 80.[27] The parameters estimated from the above model are then used to generate age-specific probability of death from age 85 to 109.

Section 2.5.4. GBD relational model life table system with a flexible standard selection mechanism

The relational model life table system is based on the logic that in order to capture the very high levels of younger adult mortality seen in populations with high HIV prevalence, we needed to develop a model life table for a counterfactual population without HIV and then add on the effects of HIV by age and sex. This system is captured in three components. We first estimated counterfactual levels of 5q0 and 45q15 in the absence of HIV. The empirical approach taken for this step is explained below. Then a full set of age-specific death rates were generated using the model life table system from the counterfactual levels of child and adult mortality. Finally, the increase in mortality, specific to each age group, associated with HIV was estimated.

Model for populations free of HIV/AIDS

In terms of estimating a set of age-specific death rates from 5q0 and 45q15 using a relational model life table, we have undertaken several innovations. A key change from previous relational model life table systems is the shift to modeling q_x in logit space rather than the l_x in the same space. Modeling q_x enables us to more precisely capture the different impacts of changes from the two entry parameters (5q0 and 45q15) on different age-groups. The following equation provides the life table for populations not affected by HIV/AIDS.

$$\mathrm{logit}({}_{n}q_{x}^{c}) = \mathrm{logit}({}_{n}q_{x}^{s}) + \beta_{\mathrm{x}}^{1} \cdot \left(\mathrm{logit}({}_{5}q_{0}^{c}) - \mathrm{logit}({}_{5}q_{0}^{s})\right) + \beta_{\mathrm{x}}^{2} \cdot \left(\mathrm{logit}({}_{45}q_{15}^{c}) - \mathrm{logit}({}_{45}q_{15}^{s})\right) + \xi_{\mathrm{x}}$$

Where $\mathrm{logit}({}_{45}q_{15}^{s})$ is the logit transformation of the 45q15 in the standard life table (development of the standard life table is presented below); $\mathrm{logit}({}_{45}q_{15}^{c})$ is the logit transformation of the 45q15 value for a country without HIV or the counterfactual level of 45q15 in the absence of HIV in a country affected by HIV/AIDS; $\mathrm{logit}({}_{5}q_{0}^{s})$ is the logit transformation of the 5q0 in the standard population; $\mathrm{logit}({}_{5}q_{0}^{c})$ is the logit transformation of 5q0 for a country without HIV or the counterfactual level of 5q0 in the absence of HIV in a country affected by HIV/AIDS; $\mathrm{logit}({}_{n}q_{x}^{s})$ is the logit of the probability of death in the standard population from age x to x + n; $\mathrm{logit}({}_{n}q_{x}^{c})$ is the logit transformation of the probability of death from age x to x + n in a country without HIV or the counterfactual level of ${}_{n}q_{x}$ in the absence of HIV in a country affected by HIV/AIDS; and β_{x}^{1} and β_{x}^{2} are coefficients that vary by age x and which measure the impact of differences in child and adult mortality rates between a target life table and the standard life table on the estimated age pattern of mortality. In other words, both coefficients determine how much the estimated age pattern of mortality deviates from the standard by age and from linearity.

This equation proposes that the logit transformed age specific probability of dying in a target life table (*c*) can be represented as a function of the corresponding logit transformed age specific probability of dying in a standard life table (*s*) and the differences in probability of dying from age 0 to 5 in logit scale and the difference in probability of dying from age 15 to 60 in logit scale between a pairs of life tables: c and s. Life table *c* is the estimated HIV-free life table either for a country affected by the epidemic or not. The model is based on an empirical observation where the differences in age specific probabilities of dying in logit scale between two life tables are highly correlated with differences in 5q0 or 45q15 in logit scale when HIV/AIDS epidemic is not present.

Coefficients β_{x}^{1} and β_{x}^{2} are estimated using the following equation:

$$logit\ {}_{n}q_{x}^{c} - logit\ {}_{n}q_{x}^{s} = \beta_{x}^{1} * (logit\ {}_{5}q_{0}^{c} - logit\ {}_{5}q_{0}^{s}) + \beta_{x}^{2} * (logit_{45}q_{15}^{c} - logit_{45}q_{15}^{s}) + \xi_{x}$$

In estimating the parameters, we use country-time specific and region (i.e. global burden of disease region) specific standard for each life table not affected by HIV/AIDS in our database (other aggregated standard life tables by different geographical or epidemiological clustering criteria are also possible). Country-time specific standard life tables are used whenever an empirical life table from the same country within a 15-year time frame is available in our database. Region specific standard life tables are generated by collapsing all zero-HIV life tables in our database from the same global burden of disease region by sex. We then pair up all zero-HIV life tables in our database with the generated region specific life tables.

The estimated $\hat{\beta}_{x}^{1}$ and $\hat{\beta}_{x}^{2}$ are shown in the table below. We limit the effects of 5q0 and 45q15 to certain age groups to avoid implausible outputs when 5q0 and 45q15 from a population change in opposite directions. With the results in the table below, we can generate full life tables for populations

not affected by HIV/AIDS. The values of 5q0 and 45q15 serve as points of entry (or entry parameters) for this model life table system.

Table: Model life table coefficients

	Diff. in $_{5}q_{0}$ (logit scale)		Diff. in $_{45}q_{15}$ (logit scale)	
**Age**	**Male**	**Female**	**Male**	**Female**
0	0.993	0.982	--	--
1-4	1.005	1.047	--	--
5-9	0.823	0.766	--	--
10-14	0.468	0.368	0.386	0.507
15-19	0.134	0.147	0.823	0.776
20-24	0.042	0.136	0.828	0.865
25-29	0.029	0.121	0.775	0.925
30-34	0.018	0.094	0.784	0.935
35-39	--	--	0.871	1.064
40-44	--	--	0.906	0.990
45-49	--	--	0.924	0.921
50-54	--	--	0.903	0.878
55-59	--	--	0.855	0.860
60-64	--	--	0.792	0.838
65-69	--	--	0.786	0.866
70-74	--	--	0.801	0.911
75-79	--	--	0.799	0.914
80-84	--	--	0.709	0.832

Our procedure of standard life table computation takes into account empirical relationships between differences in age pattern of mortality, geography, and time. To generate a standard, we first calculated the Mahalanobis distance between the target life table and all zero-HIV empirical life tables of the same sex in our database based on 5q0 and 45q15 (in logit scale). The Mahalanobis distance between two sets of 5q0 and 45q15 are defined as:

$$D_M^i(Q^i) = \sqrt{(Q^i - O)^\tau S^{-1}(Q^i - O)}$$

Where *O* is a multivariate vector representing entry parameters 5q0 and 45q15 in logit scale. $Q^i = (logit({}_5q_0^i), logit({}_{45}q_{15}^i))$ is a multivariate vector that corresponds to an empirical life table *i* in our life table database. We chose Mahalanobis distance over Euclidean distance due to the fact that 5q0 and 45q15 are highly correlated in logit space (the correlation coefficients are 0.58 and 0.87 for males and females, respectively), and Mahalanobis distance takes the covariance matrix of 5q0 and 45q15 in logit scale into consideration when calculating the distance between any pair of life tables. We then keep the potentially most similar life tables as measured by the Mahalanohis distance. The number of life tables retained is based on the numbers of empirical life tables from the same country included in our

database. For example, for developed countries with empirical life tables for every year, only the 10 most similar life tables and all life tables from the same country are kept for additional analysis.

In the second step, instead of obtaining a simple arithmetic mean of all selected life tables, we applied empirical weights to each selected life table and computed the weighted average. We examined all possible pairs of life tables of the same sex in our empirical database by country, GBD region, and GBD super-region. For each pair of life tables, we computed the sum of difference in ${}_nq_x$ in logit space. We then obtained the mean of such values by sex, time difference in years of observation, and whether the pair of life tables are from the same country, same GBD region, or same GBD super-region. The reciprocals of the squared means were used as empirical weights. The figure below shows the empirical weights by lag in time and geographic region for males. This process gives more weight to life tables closer in terms of time and geographic location.

Figure. Empirical weights by lag in time and geographic region for males.

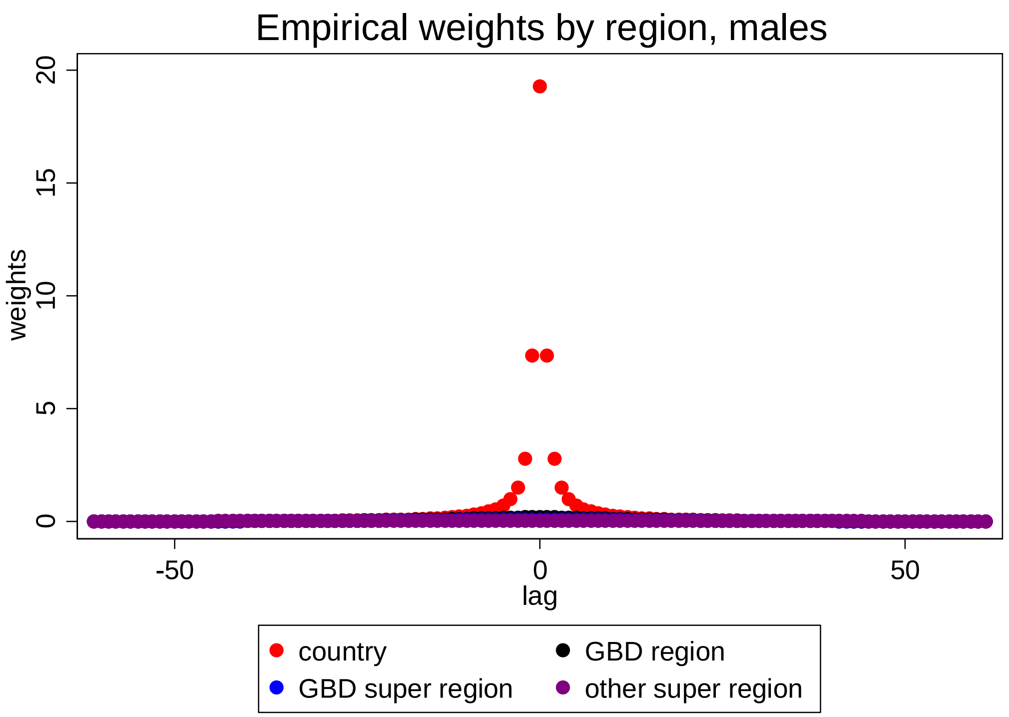

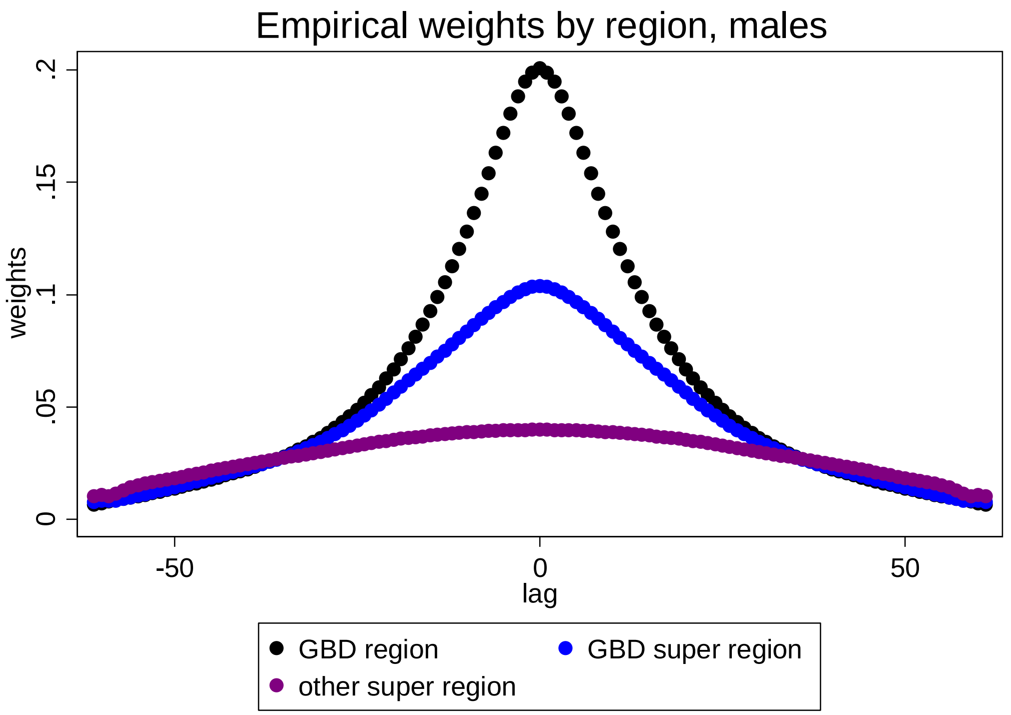

The counterfactual life table, *c*, or the final life table, *p*, when HIV/AIDS is non-existent in the population, is estimated using a standard life table, *s*, which is generated using the aforementioned procedure and coefficients $\hat{\beta}_x^{1,g}$ and $\hat{\beta}_x^{2,g}$ as shown in the model life table coefficients table.

Introducing AIDS

Currently, perhaps the most challenging issue for all existing model life table systems is providing plausible age specific mortality estimates given an HIV/AIDS epidemic. Both widely used model life table systems, the Coale-Demeny model life tables and the Modified Logit Life Table system, are largely based on empirical life tables from the pre-HIV era. In addition, they do not provide an integrated solution to the problem of incorporating HIV/AIDS.

In GBD 2016, we apply a two-step process where we first estimate HIV/AIDS counterfactual age pattern of mortality first using the method described in section 2.6 with HIV counterfactual 5q0 and 45q15 as entry parameters, and then in the second step add excess mortality due to HIV/AIDS in summary age group of under-5 and age 15 to 59 to HIV-free age specific mortality estimated from step 1. HIV counterfactual 5q0 and 45q15 are estimated using the first stages models employed in the Gaussian Process Regressions for 5q0 and 45q15 discussed in sections 2.2 and 2.4.

For GBD 2016, we apply an innovative way of adding excess mortality due to HIV to specific age groups by location estimated in the Spectrum model. In this step, we extract number of deaths due to HIV/AIDS in the spectrum model and constructed relative risk that is defined as the ratio HIV specific mortality in a specific age group and age group 40-44.

$$R_i = {}_{n}M_x^{HIV} \Big/ {}_{5}M_{40}^{HIV}$$

Where *i* indicates different age groups 0, 1-4, 4-9, 10-14, etc.

Since EPP/Spectrum is run at the draw level, we have 1000 draw of relative risks of dying from HIV/AIDS by location, sex and time. This is different from the relative risks used in previous iterations of GBD that were estimated using available VR data and by types of epidemic.

Estimating all-cause mortality for South Africa

For all locations except South Africa, the estimation of all-cause mortality follow the two step process where we generate HIV-free age pattern of mortality first, then we add on the excess mortality due to HIV/AIDS as described in the previous sections. However, since South Africa has a near complete vital registration system, we want to utilize the age pattern of all-cause mortality observed in the vital registration system to inform our all-cause mortality process. For this purpose, we used observed VR from South Africa and applied the two step model life table process as used for all other populations in reverse order. We first estimate with HIV mortality using observed VR with HIV included to generate the standard life table. Then excessive mortality due to HIV is removed from the estimated with HIV mortality using the relative risk of dying from HIV estimated in the Spectrum model.

The advantage of this process is that we are able to match the completeness adjusted vital registration data by age and the age pattern of HIV specific mortality matches what is estimated in the epidemiological model of EPP/Spectrum.

Uncertainty in the estimated life tables

One important attribute of this study is the inclusion of uncertainty in every single step of the GBD mortality estimation process. We have integrated uncertainty from the entry parameters (5q0 and 45q15) and the coefficients of each model discussed in this section based on the method illustrated by King et al.[29] Essentially, the model life table process described above is repeated 1,000 times based on randomly paired entry parameters (thus different standard life tables) and model coefficients. As a result of this process, in countries where uncertainties in estimated entry parameters are high, we can have wide uncertainty intervals in the estimated deaths, which is the final product of the GBD mortality process.

Section 2.5.5. Rake subnational life tables to national level (excluding South Africa)

Since data are scarcer for subnational units than it is at the national level in most countries, estimates of subnational mortality require adjustment to be consistent with national estimates. To generate consistent time series estimates of all-cause mortality including mortality rates and death numbers, we raked subnational-level estimates to separately-estimated national level estimates. For the mortality envelope, we applied a national scalar such that the sum of the subnational location mortality envelopes matched the mortality envelope separately estimated for the national level.

The only exception to this subnational-to-national raking process is South Africa, where we aggregate subnational level estimates as our national level estimates. Based on our observation in previous iterations of GBD, the high level of heterogeneity in the HIV/AIDS epidemic at the subnational level in South Africa introduced a challenge to accurate estimation of both all-cause and HIV cause-specific mortality in South Africa at the national level. Since our EPP-Spectrum model generated estimates at the province level for South Africa, we produced province-level all-cause mortality estimates using all available subnational sources of child and adult mortality rates and aggregated province-level results as our final national-level estimates for South Africa. The number of data sources were found to be comparable at the national and provincial levels.

Section 2.6. HIV/AIDS estimation

Section 2.6.1. Age-specific mortality (with and without HIV)

The with-HIV and without-HIV age-specific mortality rates that are inputs to the HIV/AIDS estimation are part of the model life table process described in section 2.5.

Section 2.6.3. HIV-free mortality rate

The age-specific mean HIV-free mortality rates produced by the model life table process are used as inputs to the estimation of survival on and off ART, a portion of the modeling of HIV/AIDS mortality.

Section 2.6.4. HIV-free survival rates (for Spectrum)

The age-specific HIV-free survival probabilities from the model life table process were used to create inputs for Spectrum. However, because the model life table process produces 5-year age group estimates and Spectrum models in single-year age bins, we interpolated 5-year age group survival into single-year age group survival. Life tables generally include a variable called lx, which represents a synthetic cohort, aged through the mortality observed in the table. At age 0, 100,000 people may be alive, and at each age group, the probability of survival is applied to the number who survive the previous age group. In order to interpolate single-year survival, we fit a spline to the 5-year lx values to interpolate single-year lx values. Then, we back-calculated the single-year probabilities of survival. This method was considered better than assuming constant probability of survival for each year in a 5-year age group and generated smoother single-year estimates. These single-year probabilities of HIV-free survival were then used as inputs to Spectrum.

Section 2.6.5. EPP and Spectrum

For the GBD analysis of HIV, we used variants of two tools developed by UNAIDS: a) the estimates and projections package (EPP) which is meant to generate a time series estimate of HIV incidence consistent with observed prevalence data in ages 15-49 and b) the Spectrum natural history model that uses an estimated time series of HIV incidence, demographic inputs including HIV-free mortality and population, assumptions about CD4 progression rates, and assumptions about on- and off-ART HIV death rates by age, sex, and CD4 rate to predict incidence, prevalence, and death by age, sex, and year. We modified both tools for use in the GBD.

Section 2.6.6. HIV/Mortality Reckoning

The Reckoning process is intended as a method of reconciling separate estimates of HIV mortality (and its resulting effect on estimates of HIV-free and all-cause mortality) due to two separate estimation processes within the GBD all-cause and HIV estimation framework: those from the model life table system as a way to capture the impact of HIV on age pattern of all-cause mortality, and the those from the natural history model of EPP-Spectrum as used by GBD and UNAIDS. In addition, we also utilize space-time GPR smoothed VR data on HIV-specific mortality for countries with good quality VR instead of using mortality estimates from Spectrum based on back-calculated incidence using case report data.

As part of the HIV/AIDS estimation process, all GBD 2016 locations were assigned to a modeling strategy group, depending on the level of HIV within the country and the availability and quality of HIV and VR data. Groups were used to determine which sources to use for HIV-specific mortality data and how to calculate final estimates of HIV and all-cause mortality.

Table: Locations by HIV estimation group

Group 1	Group 2A	Group 2B	Group 2C
Angola	Antigua and Barbuda	Albania	Afghanistan
Benin	Argentina	Algeria	American Samoa
Botswana	Armenia	Azerbaijan	Andorra
Burkina Faso	Australia	Bahrain	Bangladesh
Burundi	Austria	Bosnia and Herzegovina	Bolivia
Cambodia	The Bahamas	Brunei	Bhutan
Cameroon	Barbados	China	Comoros
Cape Verde	Belarus	Cyprus	Federated States of Micronesia
Central African Rep	Belgium	Egypt	Guam
Chad	Belize	El Salvador	Iraq
Cote D'Ivoire	Bermuda	Fiji	Laos
DR Congo	Brazil	FYR Macedonia	Lebanon
Djibouti	Bulgaria	Greenland	Libya
Dominican Republic	Canada	Honduras	Marshall Islands
Equatorial Guinea	Chile	Indonesia	Mauritania
Eritrea	Colombia	Iran	Mongolia
Ethiopia	Costa Rica	Jamaica	Morocco
Gabon	Croatia	Jordan	Nepal
Gambia	Cuba	Kiribati	North Korea
Ghana	Czech Republic	Malaysia	Northern Mariana Islands
Guinea	Denmark	Maldives	Pakistan
Guinea-Bissau	Dominica	Montenegro	Samoa

Group 1	Group 2A	Group 2B	Group 2C
Haiti	Ecuador	Myanmar	Sao Tome and Principe
Kenya	Estonia	Nicaragua	Saudi Arabia
Lesotho	Finland	Oman	Solomon Islands
Liberia	France	Palestine	Timor-Leste
Madagascar	Georgia	Paraguay	Tonga
Malawi	Germany	Peru	United Arab Emirates
Mali	Greece	Qatar	U.S. Virgin Islands
Mozambique	Grenada	Serbia	Vanuatu
Myanmar	Guatemala	Seychelles	Vietnam
Namibia	Guyana	Slovakia	Yemen
Niger	Hungary	South Korea	
Nigeria	Iceland	Sri Lanka	
Papua New Guinea	Ireland	Suriname	
Rep of the Congo	Israel	Syria	
Rwanda	Italy	Taiwan (Province of China)	
Senegal	Japan	Tajikistan	
Sierra Leone	Kazakhstan	Thailand	
South Africa	Kyrgyzstan	Tunisia	
South Sudan	Kuwait	Turkey	
Swaziland	Latvia	Vietnam	
Tanzania	Lithuania		
Togo	Luxembourg		
Uganda	Macao		
Zambia	Malta		
Zimbabwe	Mauritius		
India (1B)	Mexico		
Sudan (1B)	Moldova		
Somalia (1B)	Netherlands		
	Norway		
	New Zealand		
	Panama		
	Philippines		
	Poland		
	Puerto Rico		
	Portugal		
	Romania		
	Russia		
	Saint Lucia		

Group 1	Group 2A	Group 2B	Group 2C
	Saint Vincent and the Grenadines		
	Singapore		
	Slovenia		
	Spain		
	Sweden		
	Switzerland		
	Trinidad and Tobago		
	Turkmenistan		
	Ukraine		
	United Kingdom		
	United States of America		
	Uruguay		
	Uzbekistan		
	Venezuela		

Group 1 includes countries with greater than .25% adult prevalence of HIV and available HIV prevalence survey data and/or antenatal care (ANC) clinic data. We ran the EPP model using these available input data for all locations in Group 1. Demographic assessments for these locations depend substantially on sibling history data analysis, which have large uncertainty intervals and for which there may be local variation in biases in sibling history responses. India, Sudan, and Somalia were classified as Group 1B, since they had available ANC data but adult HIV prevalence less than .5%. In these locations, all-cause mortality estimates were taken from the model life table system while HIV-specific deaths were estimated using EPP-Spectrum.

Group 2A locations were classified as high quality (4 or 5 star) VR systems using the GBD VR quality rating system.[30] Since these locations have high-quality VR data, all-cause mortality estimates are driven by national data and the VR system is a robust source of data for HIV deaths.

Locations with lower quality VR make up Group 2B, and locations without any VR data are Group 2C.

For HIV-specific mortality estimates, Group 2A locations used mortality output from the ST-GPR process due to the high quality of their VR systems. Group 1A and 1B locations used Spectrum output, while Group 2B and 2C locations used output from cohort incidence bias adjusted (CIBA) deaths due to HIV/AIDS from the Spectrum model.[31]

Outputs were modeled to include an under-1 age group without the early-, late-, and post-neonatal groups. To attribute under-1 deaths from Spectrum to these neonatal groups, we make the assumption that all HIV deaths that occur in the first year of a child’s life occur in the post-neonatal stage (after 28 days), since the literature on HIV in these age groups is still unclear but seems to indicate higher mortality in the post-neonatal stage, and there is no clear evidence to guide alternative methods of age-splitting under-1 deaths due to HIV.[12,13]

Section 2.6.7. Envelope Calculation

In general, the all-cause and HIV-deleted envelopes were generated by synthesizing the results from the with-HIV and HIV-free life tables, along with selected HIV mortality from ST-GPR, Spectrum output, or cohort incidence bias adjusted Spectrum output (CIBA-Spectrum). We used with-HIV and HIV-free life tables in order to ascertain the implied HIV from the model life table system.

For age groups under age 5, we used the under-5 results from the age-sex process described in section 2.2.14. In Group 1A and 1B locations, we generated a scalar based on Spectrum results of HIV-specific and non-HIV deaths to generate HIV-deleted envelope deaths based on the all-cause results from the envelope. In other locations, we directly subtracted mortality from ST-GPR or Spectrum, after capping HIV at 90% of the all-cause envelope, to generate the HIV-deleted envelope. As mentioned previously, we made the assumption that all under-1 HIV-specific deaths occur in the post-neonatal stage.

In all groups except Group 1A, for all ages above 5 and under 15, we subtracted mortality from Spectrum or ST-GPR directly from the all-cause with-HIV envelope, after capping HIV at 90% of the all-cause envelope. In Group 1A, we took the HIV-free mortality and add mortality from Spectrum directly to generate the all-cause with-HIV envelope.

For ages above 15 and below 80, we applied separate approaches for Group 1A and 1B locations compared to Group 2A, 2B, and 2C locations. Group 1A and 1B locations used HIV mortality determined by an ensemble model where we averaged the implied HIV mortality from the model life table process and the HIV mortality output by Spectrum, which are intrinsically linked by the draw-level HIV-free mortality age pattern. Group 2A, 2B, and 2C locations used HIV mortality directly from ST-GPR and CIBA-Spectrum. For all locations except Group 1A, we subtracted the calculated HIV mortality from the all-cause envelope to generate HIV-deleted envelope deaths. In Group 1A, we added the HIV mortality to HIV-free mortality from the MLT process to calculate all-cause mortality, thus effectively allowing all-cause mortality from the demographic estimation process to be changed based on our ensemble HIV estimates. This reflects our view about the inherent uncertainty in all-cause mortality estimates largely based on sibling survival data in Group 1A locations and the various assumptions on mortality and program data required in both EPP and Spectrum.

For the age group 80 and above, we first approximated the over-80 mortality rate by using our life table output and divided lx by Tx from the age 80-84 values in the country-specific life tables. We then calculated a scalar from the approximated all-cause mortality rate from the life table to the all-cause mortality rate by taking the envelope deaths/populations. We applied this scalar to both the with-HIV and HIV-free life tables to rescale these numbers and the implied HIV death rate to the envelope space. Finally, HIV-deleted and all-cause mortality were determined by the same approaches as used for other ages above 15.

Section 2.6.8. Life Table Calculation

Generally, life tables were calculated using a consistent approach to those used in calculating the envelope. There are, however, a few differences in calculation, particularly related to sex and location aggregation.

For Group 1A locations, we took the HIV-free life table results and multiplied by the ratio to obtain all-cause life table results. In all other locations, we took the all-cause life table and divided by the ratio to obtain the HIV-free life table results, consistent with their handling in the envelope process.

For aggregate locations, we generated aggregated mx and ax values by weighting mx by population and ax by deaths (here, calculated by mx * population). From this, we population- and death-weighted regional mx and ax values, which we used to generate region-level life tables.

In the under-5 age groups, we applied a similar approach to calculating the envelope. However, when aggregating the all-cause life table for countries with subnational locations, we substituted the national-level under-5 results instead of using the aggregated mx and ax values from the subnational units. We did this in all aggregate countries except South Africa, where we aggregated the national results from subnational results, consistent with our approach in 5q0, 45q15, and the model life tables. We substituted in the under-5 national aggregates because we want to preserve the estimated 5q0 results from our 5q0 data synthesis and age/sex splitting modeling processes, which are not necessarily equivalent to those generated by the aggregated mx and ax values from the model life table process. For HIV-deleted life tables, we aggregated subnational units using the regular weighting scheme.

Section 2.6.9. HIV crude death rates for under-5 and ages 15-59

As a result of the envelope and life table calculation steps, the Reckoning produces final HIV death rates, based on the results of the Envelope Calculation portion of the Reckoning process.

Section 2.7. Age-specific mortality estimation for all GBD age-groups: with and without HIV

Section 2.7.1. Age-specific mortality without discontinuities (with HIV/AIDS)

Age-specific mortality rates without fatal discontinuities were generated using the model life table system (section 2.5), age-sex model (section 2.2.14), and HIV reckoning process (section 2.6.7) depending on the location groups detailed in section 2.6.6.

For mortality rates in age groups younger than age 5, age-specific mortality was taken from our age-sex model that split U5MR into mortality for age groups enn, lnn, pnn, and ages 1-4. For Group 1B, 2A, 2B, and 2C locations, age-specific mortality rates in ages 5 and older were generated using the GBD model life table system described in section 2.5. For Group 1A locations, age specific mortality with HIV was produced as the sum of HIV-free mortality rates generated in the first step of the model life table system described in section 2.5 and the results of the ensemble model for HIV-specific mortality described in section 2.6.

Section 2.7.2. HIV-deleted age-specific mortality

HIV-deleted age-specific mortality was used in cause of death analyses in GBD 2016 to avoid the spillover effect of HIV mortality into other causes, particularly for locations affected by high HIV burden. We used the difference between the age-specific mortality with HIV, without fatal discontinuities as described in section 5.1 and the HIV-specific mortality as a result of the HIV reckoning process described in section 2.6.7.

Section 2.7.3. Add fatal discontinuities

In our mortality estimation process, we excluded data from years with fatal discontinuities to ensure that these sudden idiosyncratic increases in mortality would not affect long-term trends in mortality for a given country. This section details how we added the deaths due to fatal discontinuities to the all-cause mortality envelope and life tables. For more information on how fatal discontinuity death numbers were estimated, please see Section 4.

To incorporate deaths due to fatal discontinuities into the mortality envelope, 1,000 draws of deaths due to fatal discontinuities were added pairwise to 1,000 draws of the with-HIV mortality envelope for each location, sex, and age group. Ninety-five percent uncertainty intervals were calculated as in other processes, taking the 97.5% and 2.5% quantiles of the summed draws.

To incorporate fatal discontinuity deaths into the life table, we created a ratio of deaths with fatal discontinuities to deaths without fatal discontinuities and applied the ratio as a scalar to the without-fatal-discontinuity mortality rate (${}_{n}m_{x}$) produced in the HIV Reckoning step, discussed in section 2.6.7. For under 1 age groups, we have scalars for early neonatal (enn), late neonatal (lnn) and post neonatal (pnn) ages. In order to calculate the probability of death for the under 1 age group (${}_{1}q_{0}$), we pulled results from the under-5 age and sex pattern of mortality process (section 2.2.14.), calculated with-fatal discontinuity mx for enn, lnn, and pnn, back-calculated qx for enn, lnn, and pnn, and then aggregated to ${}_{1}q_{0}$ using the equations in section 2.2.14. In addition to the granular under-1 age groups, we also used the ${}_{4}q_{1}$ generated by the age-sex model. Because we only had mortality and fatal discontinuity death numbers for the aggregate age group 80+, we applied the scalars for this group to the granular age groups of the life table up to 100 in order to calculate the life table with fatal discontinuities for older age groups.

Section 2.7.4. Age-specific deaths with discontinuities and HIV/AIDS

From the process described in section 2.7.3, we produced location-, sex-, year-, and age- specific death numbers including fatal discontinuities.

Section 2.7.5. Life tables with HIV/AIDS and fatal discontinuities

From the process described in section 2.7.4, we produced location-, sex-, and year-specific child mortality (5q0), adult mortality (45q15), and life expectancy at birth and age 50.

Section 3. Fertility and Birth Estimation Methods

Section 3.1. Input data

For developed countries with complete vital registration systems, we primarily utilized tabulated TFRs provided by the Human Fertility Database (HFD), a repository of rigorously-vetted empirical fertility data curated by the Max Planck Institute for Demographic Research (MPIDR). Also managed by MPIDR, the Human Fertility Collection (HFC) provided a plethora of additional data for developing countries and countries that lack complete vital registration systems. Though they are also vetted prior to inclusion in the database, not all the data from the HFC meet the complete quality standards of those from the HFD, nor are they all empirical in nature. Accordingly, from the HFC we excluded all research estimates applying methods beyond standard demographic techniques used to compute TFR from raw empirical data. We additionally maintained all HFC data from vital statistics, tabulated surveys, and official government statistical reports.

At the national level we extracted all surveys in the GHDx that fully met complete birth history requirements, applying standard direct estimation methods to generate age-specific fertility rates (ASFR), which we collapsed to TFR. Where microdata were unavailable, we extracted tabulated TFRs from the corresponding survey reports. At the subnational level, we re-utilized surveys that had been extracted for GBD 2015, which included both complete and summary birth histories. Additional data sources included provisions from international collaborators as well as tabulated reports from national statistics offices. In total, 20, 260 location years of data were included in our model over the 1950-2016 estimation period. For GBD 2017 we hope to expand our extractions to all available summary birth histories and registrations of live births, as well as hope to receive more data directly from collaborators.

Section 3.2. Modeling strategy

Section 3.2.1. Overview

In previous iterations of GBD, fertility estimates from UN WPP were used for all national locations for which they were available. For subnational locations and miscellaneous countries not covered by UN WPP we modeled total fertility rate (TFR) using spatiotemporal Gaussian process regression (ST-GPR). The specifics of the GBD 2015 ST-GPR methodology for these locations are described in the appendix to the GBD 2015 mortality and causes of death paper.[32]

To improve upon estimates used in the past and ensure consistency within our processes, we expanded the use of ST-GPR to model TFR for all 755 national and subnational locations included in GBD 2016.

Section 3.2.2. Trend estimation – Total Fertility Rate

We used ST-GPR to synthesize point estimates from multiple data sources and derive a complete time series for total fertility rate. This method has been used extensively in GBD and related studies, and accounts for uncertainty pertaining to each point estimate while borrowing strength across geographic space and time. Briefly, we assumed the Gaussian process was defined by a mean function *m*(•) and covariance function *Cov*(•).

We estimated the mean function using a two-step approach. Specifically, $m_c(t)$ can be expressed as:

$$m_c(t) = X\beta + h(r_{c,t})$$

where $X\beta$ is a linear model and $h(r_{c,t})$ is a smoothing function for the residuals; and $r_{c,t}$ is derived from the linear model. The following linear model was used for the estimation of TFR:

$$logit(tfr_{c,t}/9.5) = \beta_0 + (\beta_1 + \omega_{1R[c]})medu_{c,t} + (\beta_2 + \omega_{2R[c]})lnLDI_{c,t} + \alpha_c + \gamma_{R[c]} + \varepsilon_{c,t}$$

where $tfr_{c,t}$ is TFR for country c year t; $medu_{c,t}$ and $lnLDI_{c,t}$ are the level of maternal education and natural log of per capita lag-distributed income for country c and year t; α_c and $\gamma_{R[c]}$ are country and region random intercepts; and $\omega_{iR[c]}$ are region random slopes for the aforementioned covariates. These estimates were then modeled through ST-GPR.

The upper bound on TFR of 9.5 was informed by data from the 1981 Jordanian demographic survey, from which a TFR of 9.1 was calculated for 1972. This represented the maximum observed TFR from all available location-years of data.

Hyper-parameters for the spatiotemporal smoothing and Gaussian-process regression stages were dichotomized and selected based on the extent of vital registration completeness in a given country, as determined previously assessed for CoDEm.[32] Accordingly, countries were categorized as either data rich or not data rich. Data rich countries were assigned hyper-parameters that drew relatively less strength over space and time and yielded less variability in the mean function of the Gaussian process, such that our predictions would be more driven by high-quality, in-country data. The converse applied to countries not deemed data rich.

Random draws of 1,000 samples were obtained from the distributions above for every GBD location. Ninety-five percent uncertainty intervals were calculated by taking the ordinal 25 and 975th draws from the sample distribution.

Lastly, we scaled the outputs of the Gaussian process regression for subnational locations to national-level estimates, except in the cases of China (where Hong Kong and Macao do not exhibit trends in fertility reflective of the mainland) and the UK (where much of our high-quality data spanning the complete estimation period was at the level of the four member states). In these two cases, the national estimates were instead population-weighted aggregates of the first tier of subnational locations. Estimates for secondary, and, in the case of the UK, tertiary, tiers of subnational locations were still scaled to those of their respective geographic parents.

Section 3.2.3. Trend estimation – Age-specific fertility rate and live births

For all locations reported by UN WPP, we scaled interpolated UN WPP age-specific fertility rates (ASFR; 15-49) to GBD 2016 estimates of TFR. Interpolation was necessary, as UN WPP only produces estimates for fertility indicators in five-year intervals. For locations not covered by UN WPP—e.g. subnational units and several small countries— we generated age-patterns using the model fertility table method used in GBD 2015. Subnational age-patterns were scaled to national age-patterns in a manner similar to that for TFR above. Finally, we applied our estimated ASFR to GBD 2016 populations to produce full time series for estimates of live births.

Section 4. Socio-Demographic Index (SDI) analysis

Section 4.1. Case definition

The Socio-demographic Index (SDI) is a composite indicator of development status constructed for GBD 2015 whose components are strongly correlated with health outcomes. It is the geometric mean of 0 to 1 indices of total fertility rate, mean education for those aged 15 and older, and lag distributed income per capita.

Section 4.2. Modeling strategy

SDI was calculated using the Human Development Index (HDI) methodology, wherein an index value was determined for each of the covariate inputs (log LDI, mean educational attainment over age 15, and TFR). For GBD 2015 these indices were computed on the basis of a relative scale, in which the upper and lower bounds were established by the maximum and minimum observed values, respectively, for each input over the entire estimation period of 1980-2015.

Prompted by the observations that the scales (and by extension SDI) were sensitive to the addition of new subnational locations as GBD becomes more granular and to the length of the time period over which SDI is computed, for GBD 2016 we implemented fixed scales in determining individual indices. Thus, an index score of 0 now represents the minimum level of each covariate input past which selected health outcomes can get no worse. An index score of 1 represents the maximum level of each covariate input past which selected health outcomes cease to improve. As a composite, a location with an SDI of 0 would have a theoretical minimum level of development relevant to health, while a location with an SDI of 1 would have a theoretical maximum level of development relevant to health.

We selected the minima and maxima of the scales by examining the relationships each of the inputs had with life expectancy at birth and under-5 mortality and identifying points of limiting returns at both high and low values, if they occurred prior to theoretical limits (e.g., a TFR of 0). The final scales are summarized in the table below.

Input	Lower Bound	Upper Bound
TFR	1.5[a]	8
LDI per capita	250 USD (5.52 log USD)[b]	60,000 USD (11.00 log USD)
Mean educational attainment for ages 15 and older	0 years	17 years

[a] The low point of limiting returns for TFR was identified at 1 during GBD 2015; however, incorporating feedback with regard to accounting for a pattern of TFR rebound in highly developed countries, we instead set the lower limit of TFR at 1.5.

[b] The minimum for the LDI scale was originally set at the theoretical limit of 0 USD, as we did not observe an asymptotic relationship between log(LDI) and E_0 or 5q0 at lower values of log(LDI). Empirically, however, we also did not observe an LDI below 350 USD (5.86 log USD) for the estimation period 1970-2016. In log-space, this meant that approximately half of our scale was not being utilized, compressing the observed variation in LDI and diminishing its meaningful contribution to SDI. Accordingly, we set the lower limit on LDI to 250 USD (5.52 log USD) to ensure we were fully utilizing the range of the scale to capture its variation across space and time, as is the case with the other two inputs.

Using the limits on the scales described above, we computed the index scores underlying SDI analogously to GBD 2015 as follows:

$$I_{Cly} = {(C_{ly} - C_{low})}\Big/{(C_{high} - C_{low})}$$

Where I_{Cly} – the index for covariate *C*, location *l*, and year *y* – is equal to the difference between the value of that covariate in that location-year and the lower bound of the covariate divided by the difference between the upper and lower bounds for that covariate. If the values of input covariates fell outside the upper or lower bounds (e.g. LDI per capita greater than 60,000 USD), they were mapped to the respective upper or lower bounds. We also note that the index value for TFR was computed as $1 - I_{TFRly}$, as lower TFRs correspond to higher levels of development, and thus higher index scores. For GBD 2016 we expanded the computation of SDI to 755 national and subnational locations spanning the time period 1970-2016.

The composite Socio-demographic Index is the geometric mean of these three indices for a given location-year. The cutoff values used to determine quintiles for analysis were then computed using country-level estimates of SDI for the year 2016, excluding countries with populations less than 1 million.

We further aimed to validate the use of SDI by regressing it in a variety of forms against life expectancy at birth, 5q0, 35q15, and 20q50. We found that SDI generally is as capable of predicting these demographic indicators as the previous SDI, and also as the inputs. We also found that in incorporating year, we did not substantially reduce the coefficients for SDI. Additionally, in testing lags of 2-10 years, we found the version with no lag to be the most predictive.

Section 4.3. Age-sex-specific relationships between SDI and death rates

In order to evaluate the relationship between SDI and mortality, we fit a Gaussian process regression using a linear prior to the mean function within a stochastic partial differential equation (SPDE) framework.

We first assume the following:

$$\ln(Y_{ias}) \sim N(\mu_i, \sigma^2)$$

Where $Y_{,ias}$ is the all-cause mortality rate for a given level of SDI (*i*), age group (*a*), and sex (*s*).

We then specify a linear prior to the mean μ_i,:

$$\mu_i = \alpha + \beta(SDI) + z_i$$

Where

$$z_i \sim GP(0, \Sigma_M)$$

GP refers to a Gaussian process, and Σ_M refers to the Matern covariance function.

Using SPDE, we specified additional priors on the range, variance, and precision of the mean function, as well as selected the number of underlying bases. These hyperparameters were chosen empirically and

were identical for all age-sex combinations. Values for the selected hyperparameters are displayed in the table below.

Hyper-parameter	Value
Range	0.2
Variance	1
Precision	1×10^{10}
Number of bases	3 (mesh points at 0.25, 0.50, 0.75)

Regressions were run separately by age and sex, using observed all-cause mortality rates from all years 1970-2016 to produce 10,000 simulations per level of SDI from 0 to 1 in increments of .005. We fit models on observations from all countries estimated in GBD and included state and province level estimates in lieu of national estimates for Brazil, China, and India due to their large populations (> 200 million) and small number of state-level units modelled in GBD (BRA – 27, IND – 31, CHN – 33) relative to population. Though the United States and Indonesia also fall under the designation of large-population (> 200 million), we fit models on national-level observations instead of state/province-level observations for these two countries as a result of the undue influence from the relatively large number of state-level units modelled in GBD relative to population (USA – 51, IDN – 34). Country and region dummy variables used in GBD 2015 were no longer included in this analysis. All models were fit using the INLA package in R.

Section 4.4. SDI quintiles

All GBD 2016 locations were assigned to SDI quintiles based on their calculated SDI value in the year 2016. The quintiles (Low SDI, Low-middle SDI, Middle SDI, High-middle SDI, and High SDI) were generated using national-level SDI for the year 2016 from countries with populations greater than 1 million. The cutoffs represent each 20th percentile from the list of aforementioned values and were applied to the SDI values for the year 2016 for all GBD locations to assign them to specific groups.

Table: SDI quintile cutoffs

Quintile	Lower bound	Upper bound
Low	0	0.450402989
Low-middle	0.450402989	0.637800918
Middle	0.637800918	0.747342101
High-middle	0.747342101	0.849027407
High	0.849027407	1

Section 5. Fatal Discontinuities Estimation

5.1 Input data

Input data for fatal discontinuities are compiled from a range of sources, including country vital registration (VR) data; international databases that capture several cause-specific fatal discontinuities; and supplemental data in the presence of known issues with data quality or representativeness, or time lags in reporting.

Subnational locations and population splitting

In locations where we produced estimates at the subnational level for GBD 2016, deaths due to all fatal discontinuity causes were assigned to the relevant subnational location(s) when that information could be obtained either through country data sources (e.g., VR) or through additional online research. If no subnational location could be found, the deaths were split proportionally by population across all subnational locations.

In locations that have experienced boundary changes or split from other locations that we currently estimate (e.g., the former Yugoslavia, Czechoslovakia, the Soviet Union, Sudan and South Sudan), we split deaths due to events that occurred prior to boundary changes proportionally based on the populations residing within the boundaries of present-day locations unless we found documentation that clearly indicated whether the event and corresponding deaths occurred in one of the present-day GBD 2016 locations.

Locations with 4- or 5-star data quality ratings

For countries and territories assigned 4- or 5-star data quality ratings we prioritized data from country-specific vital registration.[30] VR data for fatal discontinuities was exclusively used in 4- and 5-star locations unless there was well-known data quality issues or discrepancies in the cause of death data reporting related to a particular event (e.g., supplemental death data for Louisiana was used for Hurricane Katrina because of established data reporting issues).

Locations with less than 4-star data quality ratings

For countries and territories assigned data quality ratings below 4 stars, we compared VR with data available from alternative sources for Exposure to forces of nature, taking the highest death estimate available from all sources. For other fatal discontinuity causes, we disregarded lower quality VR and used well-established databases by type of fatal discontinuity. Whenever specific events were identified that did not have corresponding data points within these databases, we used supplemental data sources, including scientific literature.

Major data sources other than country vital registration for each fatal discontinuity cause follow.

Conflict and terrorism. Data for conflict and terrorism come from the Uppsala Conflict Data Program (UCDP), International Institute for Strategic Studies, and Robert S. Strauss Center for International Security and Law. The table below provides details about the various datasets we utilized from these sources, the dates they were last accessed, and the years for which we used the data provided.

Data source name	Date accessed	Years of data downloaded	Type of data included
**Uppsala Conflict Data Program**[33]			
Battles	10/6/16	1989-2015	Armed conflict: incompatibility that concerns government and/or territory over which the use of armed force between the military forces of two parties, of which at least one is the government of a state, which resulted in deaths
Non-state	10/6/16	1989-2015	The use of armed force between two organized armed groups, neither of which is the government of a state, which results in deaths
One-sided	10/6/16	1989-2015	The use of armed force by the government of a state or by a formally organized group against civilians which results in deaths
Georeferenced Event Dataset	10/6/16	1989-2015	UCDP battles, non-state, and one-sided conflict deaths with the most disaggregated location information available
PRIO Battles Deaths Dataset	10/6/16	1970-1988	Armed conflict (civil wars, etc.)
**International Institute for Strategic Studies**			
Armed Conflict Dataset	10/6/16	1997-Present	Insurgency, Inter-state, Intra-state conflict deaths
**Robert S. Strauss Center For International Security And Law**			
Armed Conflict Location and Event Dataset (ACLED)	10/6/16	1997-2016	Actions of opposition groups, governments, and militias in selected locations in Africa and Asia, specifying the exact location and date of battle events, transfers of military control, headquarter establishment, civilian violence, and rioting
Social Conflict Analysis Database (SCAD)	10/6/16	1990-2016	Protests, riots, strikes, inter-communal conflict, government violence against civilians, and other forms of social conflict (covers Africa and Latin America)

Supplemental online research was conducted for recent conflicts where the databases above were not up-to-date. For country-years where multiple sources provided estimates, we prioritized sources in the following order: (1) country VR data, if death estimates were highest of all sources; (2) UCDP; (3) IISS; (4) country VR if death estimates were not the highest of all sources; (5) Robert Strauss Center; (6) online supplemental research.

Exposure to forces of nature, other injury causes, and protein-energy malnutrition. The Centre for Research on the Epidemiology of Disasters' International Disaster Database (EM-DAT) served as the primary non-VR source of fatal discontinuities due to exposure to forces of nature (i.e., natural disasters); other transport injuries (eg, plane, train, and boat accidents); poisonings; fire, heat, and hot substances; other exposure to mechanical forces (eg, building collapse); and protein-energy malnutrition (ie, famine or severe drought). Data from EM-DAT were last accessed March 29, 2017. Supplemental online research was conducted for events where EM-DAT was not up-to-date.

For country-years where multiple sources provided estimates, we prioritized sources in the following order: (1) country VR data, if data quality rating is 4 or 5 stars; (2) country VR data if data quality rating is

less than 4 stars and death estimates were highest of all sources; (3) EM-DAT; (4) online supplemental research. Exceptions were made where it was clear that VR systems had been compromised by the event being measured.

Epidemic infectious diseases. In GBD 2016, we included fatal discontinuities due to a subset of infectious diseases: meningococcal meningitis (or meningococcal infection), diarrheal disease caused by cholera, and Ebola virus disease. These diseases were included because (1) existing modelling strategies (for meningitis and cholera) did not optimally capture epidemic mortality levels and trends; and (2) they contributed to significant total fatalities in a given location-year. Other infectious diseases for which the latter is true – high death rates in the presence of an outbreak or epidemic – are currently modelled with alternative cause of death methods (eg, natural history models for measles and yellow fever), which allow for greater variation year-over-year if or when outbreaks occur. In future iterations of the GBD, we plan to revisit the inclusion criteria for infectious diseases as fatal discontinuities and develop more of an ensemble approach to modelling causes that can be both endemic (and thus result in more uniform levels and trends over time) and epidemic (and subsequently lead to rapid increases – and decreases – in deaths for a given location-year).

The Global Infectious Diseases and Epidemiology Network (GIDEON) served as the primary data source for collating cholera and meningococcal meningitis or meningococcal infection death reports.[34,35] For any year in which cholera or meningococcal meningitis deaths were recorded in a country or territory covered by the GBD, we directly extracted reported deaths from 1970 to 2016. When there were reporting gaps in cholera or meningococcal meningitis deaths over this period of time and the World Health Organization (WHO) annual cholera or meningitis reports had death reports for those years, we used the WHO reports.

The input data for deaths due to Ebola virus disease came in two forms: (i) modelled estimates for the West African outbreak from 2013 to 2016 provided by the World Health Organization (WHO) focused specifically on the three worst-affected countries (Liberia, Guinea, and Sierra Leone) and (ii) literature searches for reported deaths due to Ebola virus disease not captured by the West African dataset.

5.2 Modeling strategy

All input data for fatal discontinuity causes were run through the causes of death data formatting and mapping process[30], with the exception of deaths due to Ebola virus disease, where WHO data already contained age- and sex-specific information.

Uncertainty analysis for input and draw-level input to age-sex splitting

Uncertainty intervals for deaths due to conflict and terrorism were generated using UCDP high and low death estimates, except in the case of Iraq 2003-2016, as explained above. In cases where low and high estimates were not included in the available data, the regional average uncertainty interval was applied to the available death estimate across all fatal discontinuity causes.

We assumed a normal distribution using the mean deaths and standard deviation based on high and low estimates. The standard deviation was capped at the mean divided by 1.96 in order to ensure that 95% of the 3,000 draws generated were greater than zero. Non-positive draws were dropped, and 1,000 draws were sampled from the remaining set of positive draws. These 1,000 positive draws were used for final calculations of means and uncertainty intervals.

Section 6. Additional Methods Information

6.1 GBD world population age standard

Age-standardized populations in the GBD are calculated using the GBD world population age standard, which is calculated using methods detailed in Ahmad et al 2001.[36] Briefly, we used the age-specific proportional distributions of all national locations from the World Population Prospects 2012 revision for all years from 2010 to 2035 and generated a standard population structure by taking the non-weighted mean across all the aforementioned country-years. For consistency and comparability across recent iterations of the GBD, we used the same standard population structure as used in GBD 2013 and GBD 2015. The values used for the age standard are found in Appendix Table 6.

6.2 Generating population estimates in older age groups

GBD 2016 expands the estimated age groups from a terminal age group of 80+ to 95+. For some population data sources, 5-year age group populations were not available above age 80. In these cases, we used a model to age populations into the additional age groups: 80-84, 85-89, 90-94, and 95+. First we estimated survival for single-year age groups by sex and year for all locations included in GBD 2016, and then applied a cohort component model to age 79-year-olds forward to estimate population exposure by age and year in the detailed 80-plus age groups.

6.3 Categorising special populations

The GBD uses a *de facto* definition of populations in all GBD estimation. Since populations are primarily taken from UN Population Division's World Population Prospects, which uses a strict *de facto* definition, we strive to use the same definition in capturing deaths that occur where the deceased died in a country where they did not hold citizenship and/or residency. The table below describes the idea criteria used for some specific examples of these complex situations.

Though we strive to ensure that all mortality data used in GBD estimation follows these same *de facto* rules, we are limited by the collection methods and information provided in the data source itself. We have made adjustments in locations where we know that death sources did not use a de facto definition but are aware that there may be additional unknown cases where our deaths source and population source do not match in their definition of included populations.

Table: Preferred country of death assignments for non-citizen/resident deaths

Category of person	How do we assign country of death?
a legal permanent resident or temporary resident (student, worker assigned overseas by their domestic employer, migrant worker temporarily in the country)	Assign to the country of residence at the time of the death
someone on a tourist visa	Assign to the country he/she is visiting
an illegal immigrant/visitor	Assign to the country where he/she migrated to
a mercenary	Assign to the country where he/she worked
a soldier or other government official or contractor stationed overseas	Assign to the country where he/she is from
a person in a refugee camp just outside a country's border	Assign to the country where they are citizens
on the high seas or coastal waters/below walls of a country where refugees tried to flee	Assign to the country where they are citizens

6.4 Estimating correlation

For the estimation of correlation, we used the Pearson correlation coefficient as estimated by Stata version 14, which uses the following function[37]:

$$\hat{\rho} = \frac{\sum_{i=1}^{n} w_i (x_i - \bar{x})(y_i - \bar{y})}{\sqrt{\sum_{i-1}^{n} w_i (x_i - \bar{x})^2} \sqrt{\sum_{i-1}^{n} w_i (y_i - \bar{y})^2}}$$

where w_i are the weights, if specified, or $w_i = 1$ if weights are not specified. $\bar{x} = (\sum w_i x_i)/(\sum w_i)$ is the mean of x, and $\bar{y}$ is similarly defined.

6.5 Calculating annualised rates of change

Annualized rates of change were calculated by taking the log of 5q0 in a target year, represented as *y+t*, divided by the 5q0 at the baseline year *y*, and divided by the difference in years between the target and baseline years, represented as *t*. This is illustrated by the equation below.

$$aroc_{_5q_0} = \ln\left(\frac{_5q_{0\ y+t}}{_5q_{0\ y}}\right) / \text{(t)}$$

6.6 Causes of death data star rating calculation

GBD estimates are most accurate when computed with a full time series of complete vital registration with a low percentage of garbage codes. For GBD 2016, we developed a simple star-rating system from 0 to 5 to give a picture of the quality of data available in a given country over the full time series used in GBD estimates. Countries improve in the star rating as they increase availability, completeness, and detail of their mortality data and reduce the percentage of deaths coded to ill-defined garbage codes or highly aggregated causes.

To assign stars, we measure the proportion of deaths registered to a well-defined cause from 1980 to 2016. We call this proportion “percent well-certified.” We measure this proportion for each location-

year of vital registration and each verbal autopsy study separately, and then combine the yearly measurements into a percent well-certified for the full time series.

For each year of vital registration, percent well-certified is:

$$pct_{wellcertified} = completeness * (1 - pct_{majgarbage})$$

Where:

$$completeness = \frac{registered\ deaths}{GBD\ mortality\ envelope}$$

$$pct_{majgarbage} = \frac{deaths\ coded\ to\ level\ 1\ or\ 2\ garbage\ or\ highly\ aggregated\ cause}{registered\ deaths}$$

Simplifying this equation, one can see that in this case "percent well-certified" is simply the number of deaths that are registered to a well-defined cause (those codes which are not Level 1 or 2 garbage or highly aggregated) divided by the GBD mortality envelope. More information about ICD10 and ICD9 codes assigned to Level 1 or 2 garbage can be found in the GBD 2016 causes of death paper.[38]

For each verbal autopsy data source, percent well-certified is:

$$pct_{wellcertified} = VerbalAutopsyAdjustment * (1 - pct_{majgarbage})$$

Where:

$$VerbalAutopsyAdjustment = SubAdj * RegAdj * AgeSexCoverage$$

And:

SubAdj:

10% for subnationally representative studies, 100% for nationally representative studies. This adjustment, while arbitrary in its specific value, reflects the bias that can be associated with studies that only cover a potentially non-representative sample of a country's population.

RegAdj:

64% for all verbal autopsy data sources. This accounts for the inaccuracy of verbal autopsy in assigning cause of death compared to medically-verified vital registration. The specific multiplier 0·64 is based on the chance-corrected concordance of Physician Certified Verbal Autopsy (PCVA) versus medical certification by the Population Health Metrics Research Consortium.[39]

Age-Sex Coverage:

The number of deaths estimated in the GBD mortality envelope for the ages and sexes in the study for the country and year divided by the number of deaths estimated in the GBD mortality envelope for the country and year. Studies that only cover children under age 5 or maternal mortality, for example, will be highly discounted by this multiplier.

In the case of verbal autopsy, all garbage codes are considered ill-defined, as redistribution for verbal autopsy is highly imprecise. Causes such as "Injuries" or "Cancer" will also be included in major garbage percentage, as this percentage includes use of highly aggregated causes.

Once percent well-certified is calculated for each location-year of vital registration and each verbal autopsy study-year, we then combine these into one measurement for each five-year time interval and the full time series 1980–2016. For each five-year time interval, we take the maximum percent well-certified. Then for 1980–2016, we take the average of the maximum percentages well-certified for the seven five-year time intervals, including any five-year time interval where no data were available as a zero.

Once these values are calculated, we assign stars as follows:
5 stars: 85%–100% well-certified
4 stars: 65%–84% well-certified
3 stars: 35%–64% well-certified
2 stars: 10%–34% well-certified
1 star: >0%–9% well-certified
0 stars: No vital registration or verbal autopsy data available from 1980–2016

The GBD 2016 causes of death paper[38] provides more detail on the percent well-certified, stars, data sources, and underlying values for percent well-certified used for each country and time interval.

Section 7: References

1 Rajaratnam JK, Tran LN, Lopez AD, Murray CJL. Measuring Under-Five Mortality: Validation of New Low-Cost Methods. *PLOS Med* 2010; **7**: e1000253.

2 Wang H, Dwyer-Lindgren L, Lofgren KT, *et al.* Age-specific and sex-specific mortality in 187 countries, 1970–2010: a systematic analysis for the Global Burden of Disease Study 2010. *The Lancet* 2012; **380**: 2071–94.

3 Hill K. Estimating census and death registration completeness. *Asian Pac Popul Forum* 1987; **1**: 8–13, 23–4.

4 Wang H, Liddell CA, Coates MM, *et al.* Global, regional, and national levels of neonatal, infant, and under-5 mortality during 1990–2013: a systematic analysis for the Global Burden of Disease Study 2013. *The Lancet* 2014; **384**: 957–79.

5 Murray CJ, Ezzati M, Flaxman AD, *et al.* GBD 2010: design, definitions, and metrics. *The Lancet* 2012; **380**: 2063–6.

6 Wang H, Bhutta ZA, Coates MM, *et al.* Global, regional, national, and selected subnational levels of stillbirths, neonatal, infant, and under-5 mortality, 1980–2015: a systematic analysis for the Global Burden of Disease Study 2015. *The Lancet* 2016; **388**: 1725–74.

7 Gelman A, Carlin JB, Stern HS, Rubin DB. Bayesian Data Analysis, Second Edition, 2 edition. Boca Raton, Fla: Chapman and Hall/CRC, 2003.

8 Gompertz B. On one Uniform Law of Mortality from Birth to extreme Old Age, and on the Law of Sickness. *J Inst Actuar Assur Mag* 1871; **16**: 329–44.

9 The UN Inter-agency Group for Child Mortality Estimation. Child Mortality Estimates. http://www.childmortality.org/ (accessed May 20, 2017).

10 Bradshaw D, Dorrington R. Child mortality in South Africa - we have lost touch. *S Afr Med J* 2007; **97**: 582.

11 Kerber KJ, Lawn JE, Johnson LF, *et al.* South African child deaths 1990-2011: have HIV services reversed the trend enough to meet Millennium Development Goal 4? *AIDS Lond Engl* 2013; **27**: 2637–48.

12 Kim H-Y, Kasonde P, Mwiya M, *et al.* Pregnancy loss and role of infant HIV status on perinatal mortality among HIV-infected women. *BMC Pediatr* 2012; **12**: 138.

13 Brocklehurst P, French R. The association between maternal HIV infection and perinatal outcome: a systematic review of the literature and meta-analysis. *Br J Obstet Gynaecol* 1998; **105**: 836–48.

14 Blencowe H, Cousens S, Jassir FB, *et al.* National, regional, and worldwide estimates of stillbirth rates in 2015, with trends from 2000: a systematic analysis. *Lancet Glob Health* 2016; **4**: e98–108.

15 Bennett NG, Horiuchi S. Estimating the Completeness of Death Registration in a Closed Population. *Popul Index* 1981; **47**: 207–21.

16 Brass W, Coale AJ. Methods of Analysis and Estimation. In: Mathematical Demography. Springer Berlin Heidelberg, 1977: 307–13.

17 Preston SH. The changing relation between mortality and level of economic development. *Popul Stud* 1975; **29**: 231–48.

18 Preston S, Coale AJ, Trussell J, Weinstein M. Estimating the Completeness of Reporting of Adult Deaths in Populations That Are Approximately Stable. *Popul Index* 1980; **46**: 179–202.

19 Murray CJL, Rajaratnam JK, Marcus J, Laakso T, Lopez AD. What Can We Conclude from Death Registration? Improved Methods for Evaluating Completeness. *PLOS Med* 2010; **7**: e1000262.

20 Obermeyer Z, Rajaratnam JK, Park CH, *et al.* Measuring Adult Mortality Using Sibling Survival: A New Analytical Method and New Results for 44 Countries, 1974–2006. *PLOS Med* 2010; **7**: e1000260.

21 Gakidou E, King G. Death by survey: estimating adult mortality without selection bias from sibling survival data. *Demography* 2006; **43**: 569–85.

22 Masquelier B. Adult mortality from sibling survival data: a reappraisal of selection biases. *Demography* 2013; **50**: 207–28.

23 Rogers RG, Crimmins EM, editors. International Handbook of Adult Mortality. http://www.springer.com/gp/book/9789048199952 (accessed May 20, 2017).

24 Lozano R, Wang H, Foreman KJ, *et al.* Progress towards Millennium Development Goals 4 and 5 on maternal and child mortality: an updated systematic analysis. *The Lancet* 2011; **378**: 1139–65.

25 Schnuelle P, Lorenz D, Trede M, Van Der Woude FJ. Impact of renal cadaveric transplantation on survival in end-stage renal failure: evidence for reduced mortality risk compared with hemodialysis during long-term follow-up. *J Am Soc Nephrol JASN* 1998; **9**: 2135–41.

26 Murray CJL, Ferguson BD, Lopez AD, Guillot M, Salomon JA, Ahmad O. Modified Logit Life Table System: Principles, Empirical Validation, and Application. *Popul Stud* 2003; **57**: 165–82.

27 Shkolnikov V, Barbieri M, Wilmoth J. Human Mortality Database. http://www.mortality.org/ (accessed May 20, 2017).

28 Thatcher AR. The long-term pattern of adult mortality and the highest attained age. *J R Stat Soc Ser A Stat Soc* 1999; **162**: 5–43.

29 King G, Tomz M, Wittenberg J. Making the Most of Statistical Analyses: Improving Interpretation and Presentation. *Am J Polit Sci* 2000; **44**: 341–55.

30 Global Burden of Disease Causes of Death Collaboration. Global, regional, and national age-sex specific mortality for 264 causes of death, 1980–2016: a systematic analysis for the Global Burden of Disease Study 2016. *Lancet Rev* 2017.

31 Wang H, Wolock TM, Carter A, *et al.* Estimates of global, regional, and national incidence, prevalence, and mortality of HIV, 1980–2015: the Global Burden of Disease Study 2015. *Lancet HIV* 2016; **3**: e361–87.

32 Wang H, Naghavi M, Allen C, *et al.* Global, regional, and national life expectancy, all-cause mortality, and cause-specific mortality for 249 causes of death, 1980–2015: a systematic analysis for the Global Burden of Disease Study 2015. *The Lancet* 2016; **388**: 1459–544.

33 UCDP/PRIO Armed Conflict Dataset Codebook. Uppsala Conflict Data Program (UCDP); Centre for the Study of Civil Wars, International Peace Research Institute, Oslo (PRIO), 2013.

34 Inc GI, Berger DS. Cholera: Global Status: 2017 edition. GIDEON Informatics Inc, 2017.

35 Inc GI, Berger DS. Bacterial Meningitis: Global Status: 2017 edition. GIDEON Informatics Inc, 2017.

36 Ahmad OB, Boschi-Pinto C, Lopez AD, Murray CJL, Lozano R, Inoue M. Age standardization of rates: A new WHO standard. Geneva, Switzerland: World Health Organization, 2001 http://www.who.int/healthinfo/paper31.pdf (accessed May 20, 2017).

37 Functions Reference Manual. 2015. http://www.stata.com/manuals13/rcorrelate.pdf (accessed May 17, 2017).

38 Nagavi M. Global, regional, and national cause-specific mortality for 242 causes of death, 1980-2016: a systematic analysis for the Global Burden of Disease Study 2016. *The Lancet* 2017; published online Sept.

39 Lozano R, Lopez AD, Atkinson C, Naghavi M, Flaxman AD, Murray CJ. Performance of physician-certified verbal autopsies: multisite validation study using clinical diagnostic gold standards. *Popul Health Metr* 2011; **9**: 32.

Section 8. Figures and Tables

Appendix Figure 1. Analytical flowchart for the estimation of all-cause mortality by age and sex and HIV/AIDS incidence, prevalence, and mortality for GBD 2016.

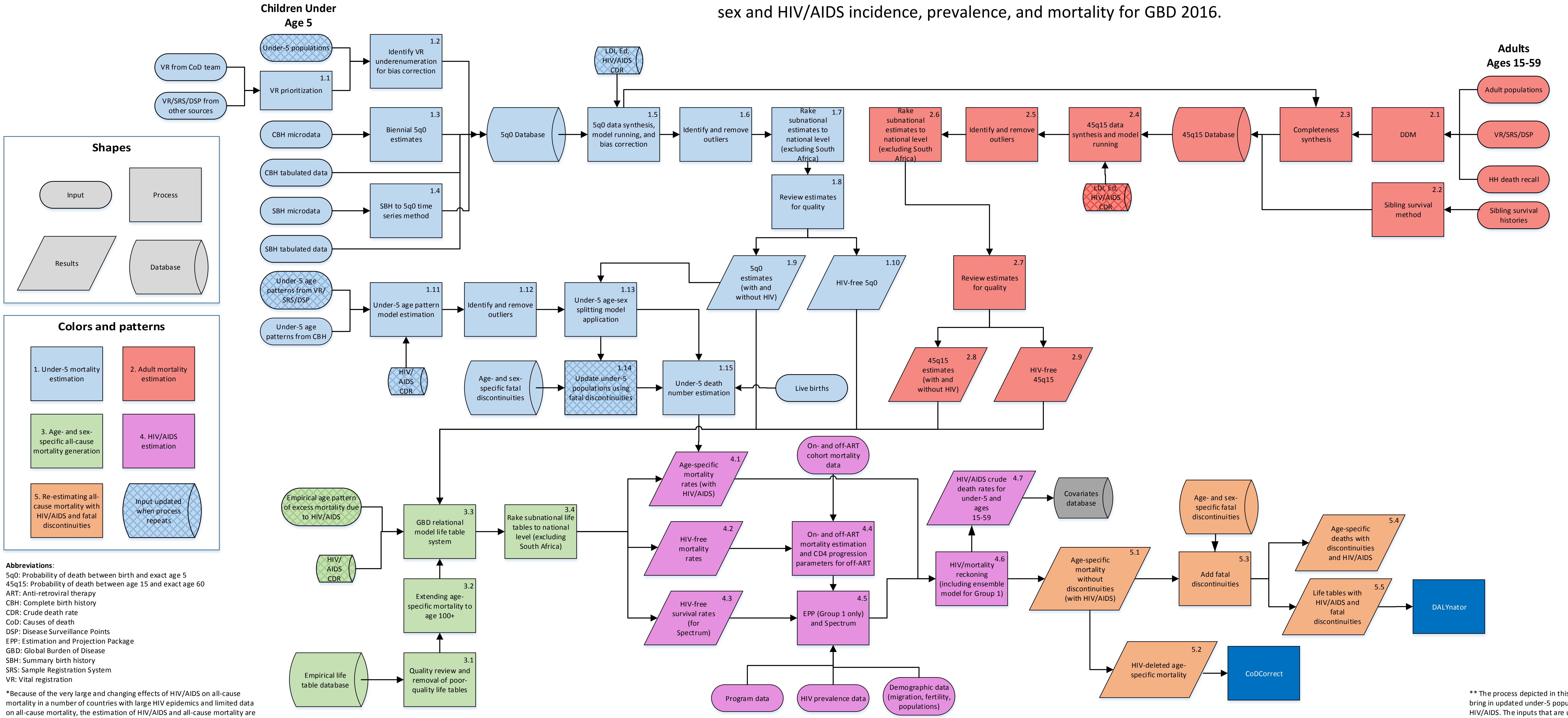

Abbreviations:
5q0: Probability of death between birth and exact age 5
45q15: Probability of death between age 15 and exact age 60
ART: Anti-retroviral therapy
CBH: Complete birth history
CDR: Crude death rate
CoD: Causes of death
DSP: Disease Surveillance Points
EPP: Estimation and Projection Package
GBD: Global Burden of Disease
SBH: Summary birth history
SRS: Sample Registration System
VR: Vital registration

*Because of the very large and changing effects of HIV/AIDS on all-cause mortality in a number of countries with large HIV epidemics and limited data on all-cause mortality, the estimation of HIV/AIDS and all-cause mortality are closely linked and presented jointly here.

** The process depicted in this flowchart is performed two times in order to bring in updated under-5 population estimates and crude death rates due to HIV/AIDS. The inputs that are updated in the second run of the process are shown using patterned boxes in this flowchart.

Appendix Figure 2: Sibling history correction for zero-survivorship, by sex

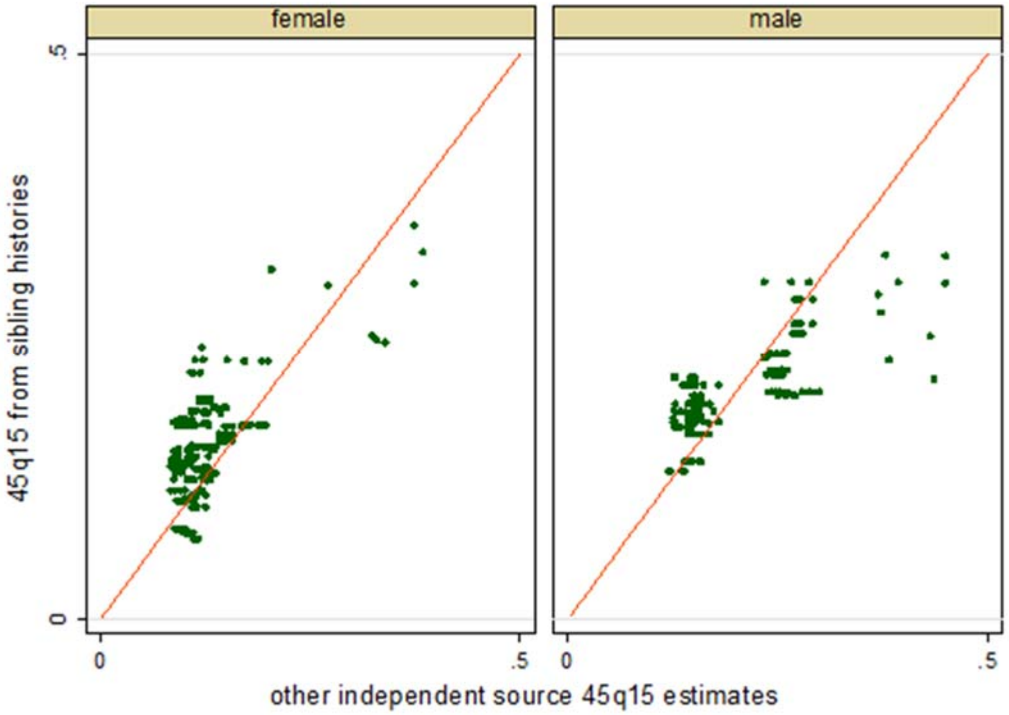

Appendix Figure 3: Comparison of life expectancy at birth by location in GBD 2015 vs GBD 2016

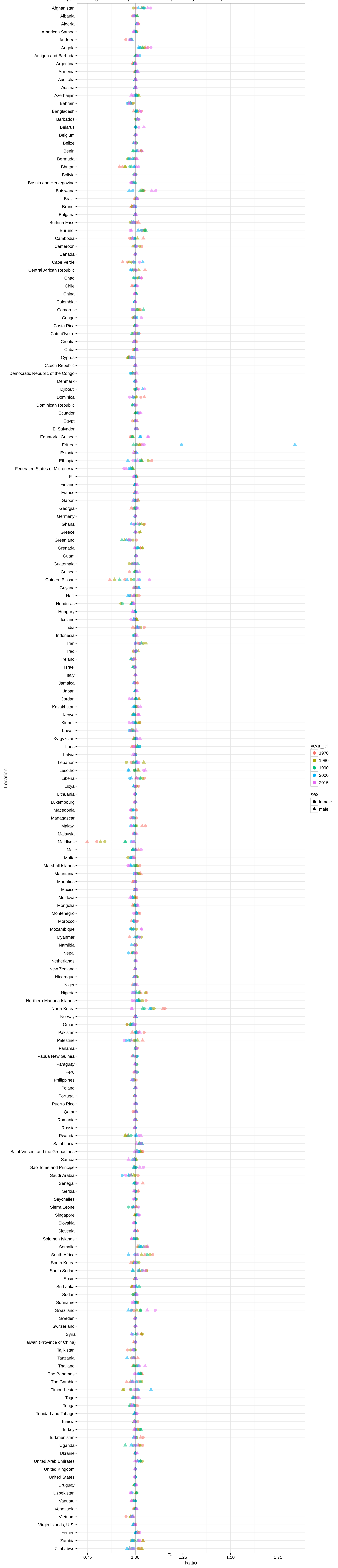

Appendix Figure 4. Comparison of estimates of under-5 mortality from GBD 2016 and IGME 2015 for all countries included in both analyses, 2015

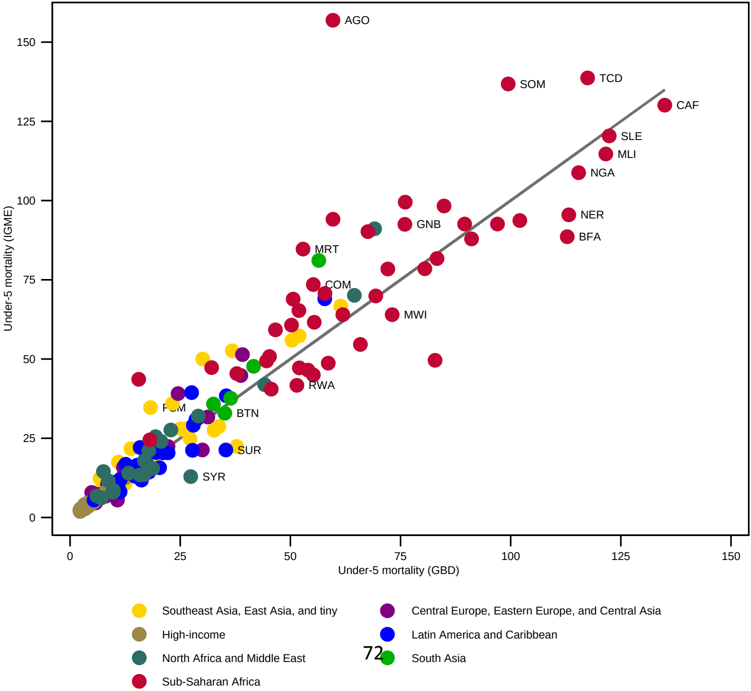

Appendix Figure 5. Ratio of stillbirth rates to neonatal mortality rates by GBD super region, 1970−2016

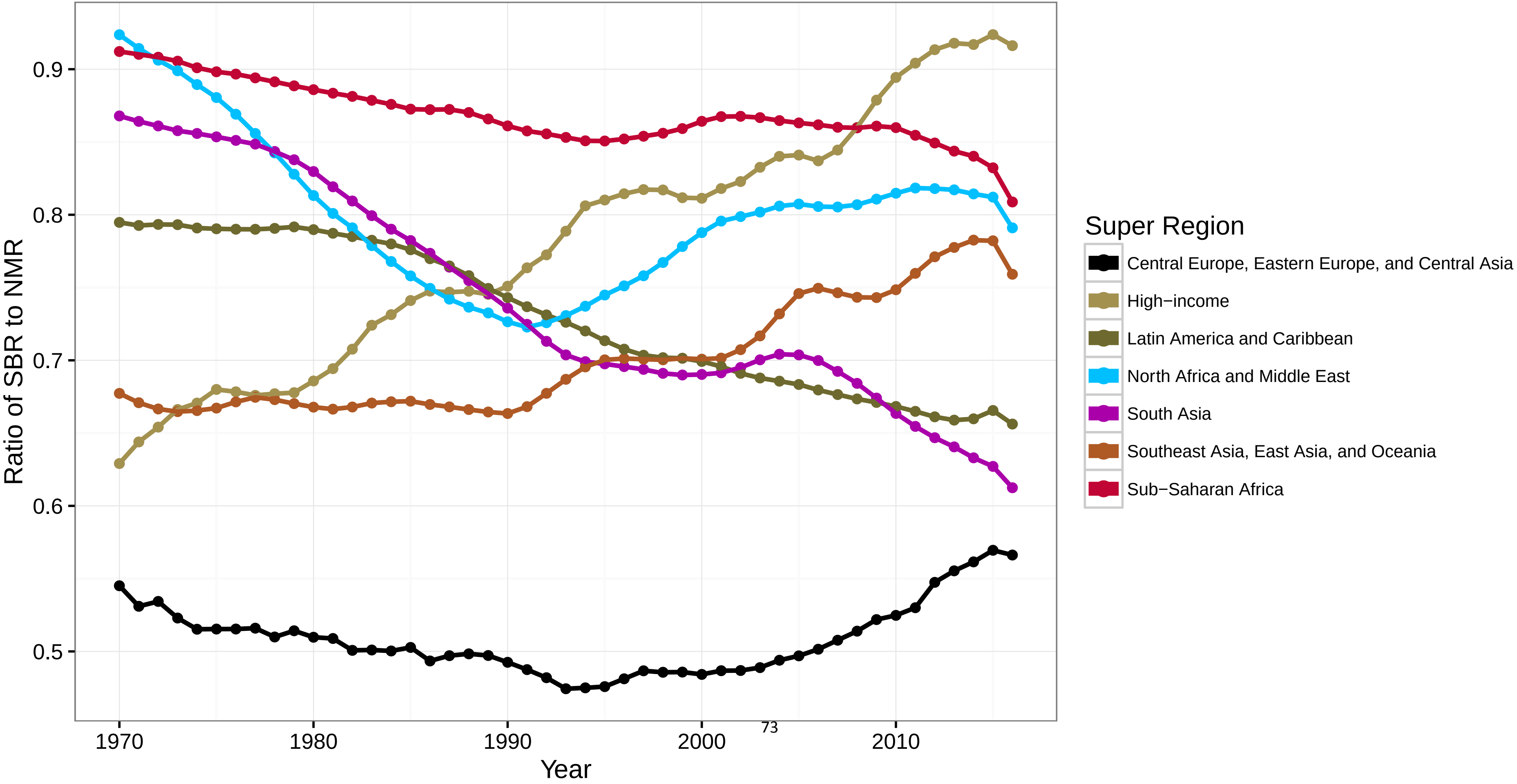

Appendix Figure 6. Comparison of stillbirth rates from GBD 2016 and SEIG for all countries included in both analyses, 2015

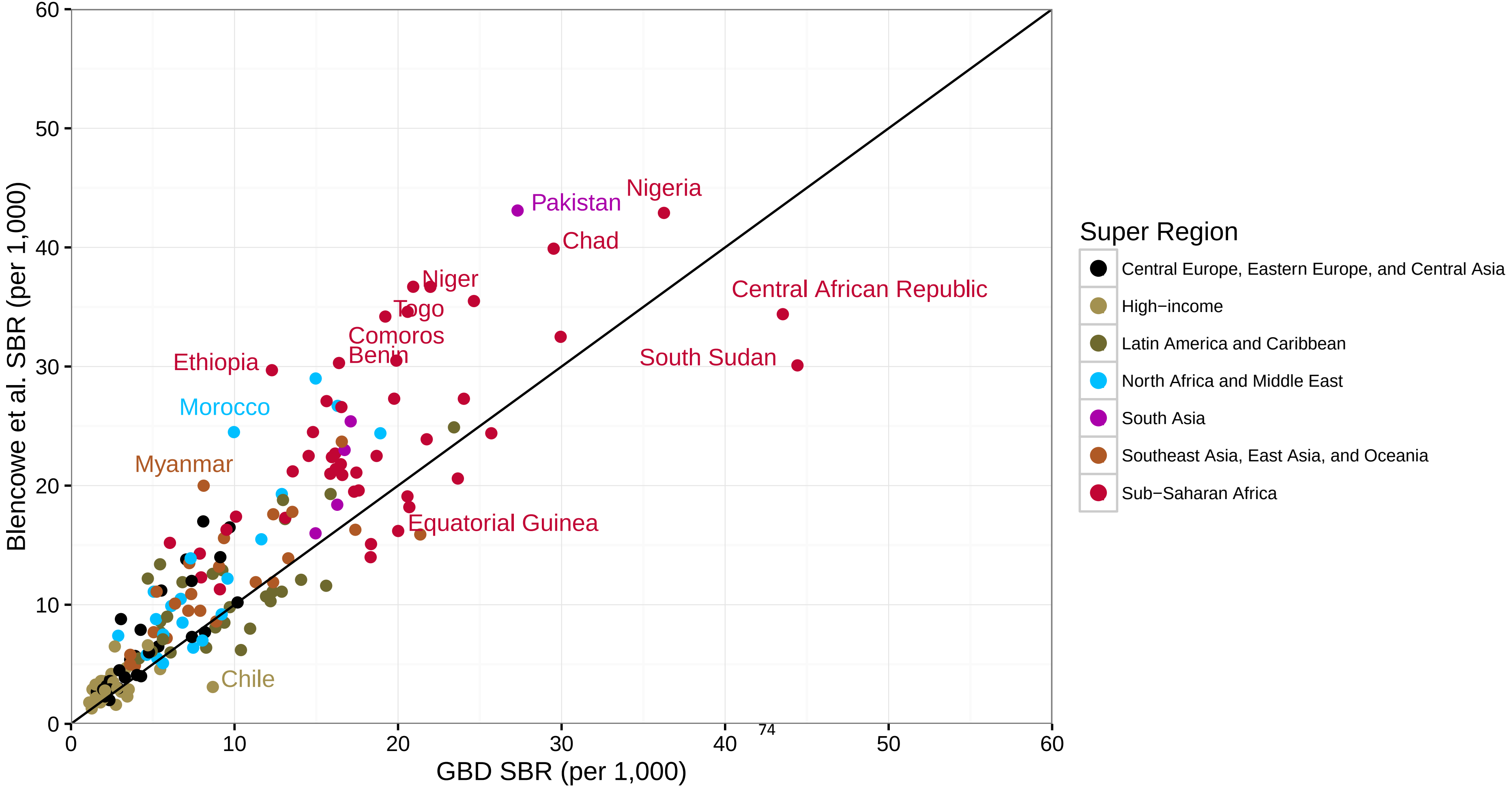

Appendix Figure 7. Ratio of estimated number of live births by location between GBD 2016 and UN Population Division, 1970, 1980, 1990, 2000, 2016

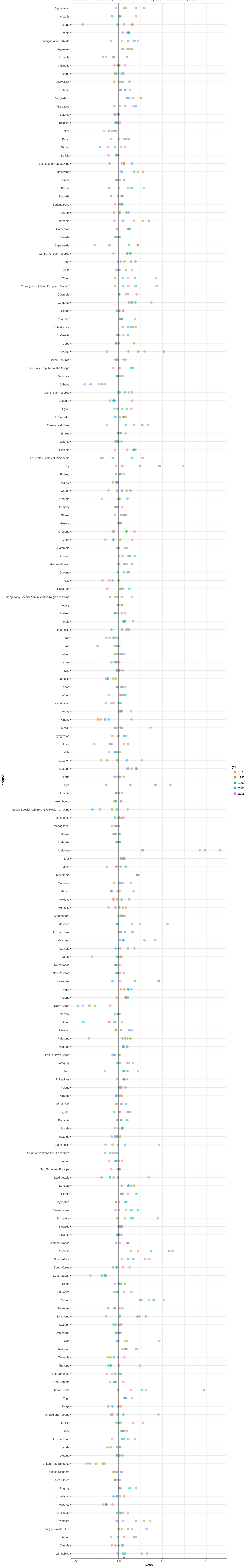

Appendix figure 8. Estimated completeness of death registration, 1970 to 1989

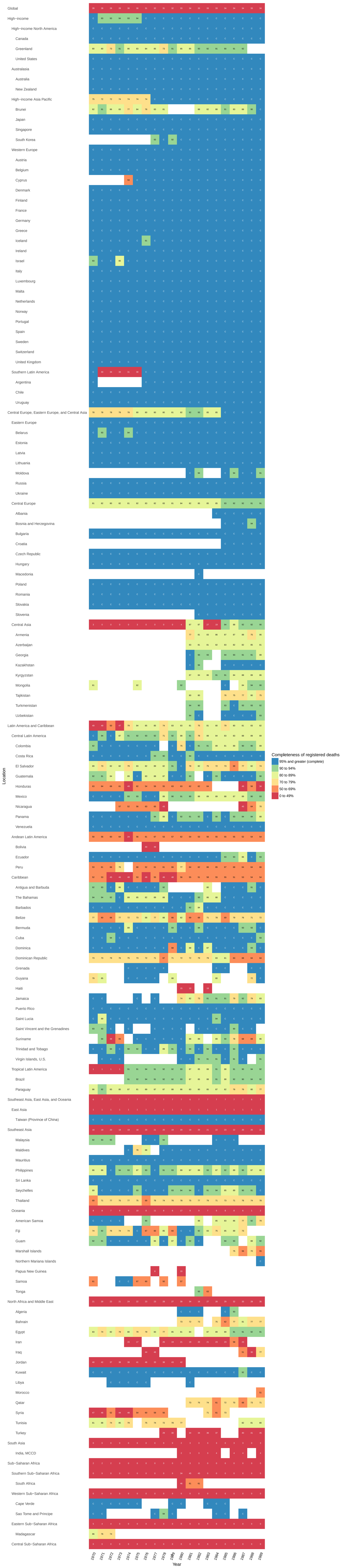

Appendix Table 1. GATHER checklist of information that should be included in reports of global health estimates, with description of compliance and location of information for GBD 2016. Global, regional, and national life expectancy and all-cause mortality, 1980-2016: a systematic review for the Global Burden of Disease Study 2016.			
**#**	**GATHER checklist item**	**Description of compliance**	**Reference**
**Objectives and funding**			
1	Define the indicators, populations, and time periods for which estimates were made.	Narrative provided in paper and methods appendix describing indicators, definitions, and populations	Main text (Methods—Overview, Geographic units and time periods) and methods appendix
2	List the funding sources for the work.	Funding sources listed in paper	Summary (Funding)
**Data Inputs**			
For all data inputs from multiple sources that are synthesized as part of the study:			
3	Describe how the data were identified and how the data were accessed.	Narrative description of data seeking methods provided	Main text (Methods) and methods appendix
4	Specify the inclusion and exclusion criteria. Identify all ad-hoc exclusions.	Narrative about inclusion and exclusion criteria by data type provided	Main text (Methods) and methods appendix
5	Provide information on all included data sources and their main characteristics. For each data source used, report reference information or contact name/institution, population represented, data collection method, year(s) of data collection, sex and age range, diagnostic criteria or measurement method, and sample size, as relevant.	An interactive, online data source tool that provides metadata for data sources by component, geography, cause, risk, or impairment has been developed	Online data citation tools
6	Identify and describe any categories of input data that have potentially important biases (e.g., based on characteristics listed in item 5).	Summary of known biases by cause included in methods appendix	Methods appendix
For data inputs that contribute to the analysis but were not synthesized as part of the study:			
7	Describe and give sources for any other data inputs.	Included in online data source tool	Online data citation tools
For all data inputs:			
8	Provide all data inputs in a file format from which data can be efficiently extracted (e.g., a spreadsheet as opposed to a PDF), including all relevant meta-data listed in item 5. For any data inputs that cannot be	Downloads of input data available through online tools, including data visualization	Online data visualization tools, data query tools, and the Global Health Data Exchange

	shared due to ethical or legal reasons, such as third-party ownership, provide a contact name or the name of the institution that retains the right to the data.	tools and data query tools; input data not available in tools will be made available upon request	
**Data analysis**			
9	Provide a conceptual overview of the data analysis method. A diagram may be helpful.	Flow diagrams of the overall methodological processes, as well as cause-specific modelling processes, have been provided	Main text (Methods, figures 1) and methods appendix
10	Provide a detailed description of all steps of the analysis, including mathematical formulae. This description should cover, as relevant, data cleaning, data pre-processing, data adjustments and weighting of data sources, and mathematical or statistical model(s).	Flow diagrams and corresponding methodological write-ups for each cause, as well as the demographics and causes of death databases and modelling processes, have been provided	Main text (Methods, figures 1) and methods appendix
11	Describe how candidate models were evaluated and how the final model(s) were selected.	Provided in the methodological write-ups	Methods appendix
12	Provide the results of an evaluation of model performance, if done, as well as the results of any relevant sensitivity analysis.	Provided in the methodological write-ups	Methods appendix
13	Describe methods for calculating uncertainty of the estimates. State which sources of uncertainty were, and were not, accounted for in the uncertainty analysis.	Provided in the methodological write-ups	Methods appendix
14	State how analytic or statistical source code used to generate estimates can be accessed.	Access statement provided	Code is provided in an online repository
**Results and Discussion**			
15	Provide published estimates in a file format from which data can be efficiently extracted.	GBD 2016 results are available through online data visualization tools, the Global Health Data Exchange, and the online data query tool	Main text, methods appendix, and online data tools (data visualization tools, data query tools, and the Global Health Data Exchange)
16	Report a quantitative measure of the uncertainty of the estimates (e.g. uncertainty intervals).	Uncertainty intervals are provided with all results	Main text, methods appendix, and online data tools (data visualization tools,

			data query tools, and the Global Health Data Exchange)
17	Interpret results in light of existing evidence. If updating a previous set of estimates, describe the reasons for changes in estimates.	Discussion of methodological changes between GBD rounds provided in the narrative of the Article and methods appendix	Main text (Methods and Discussion) and methods appendix
18	Discuss limitations of the estimates. Include a discussion of any modelling assumptions or data limitations that affect interpretation of the estimates.	Discussion of limitations provided in the narrative of the main paper, as well as in the methodological write-ups in the methods appendix	Main text (Limitations) and methods appendix

Appendix Table 2. Distribution of empirical life tables by GBD super-region, 1950-2016

Super Region	Number of Life Tables
Central Europe, Eastern Europe, and Central Asia	1599
High-income	9516
Latin America and Caribbean	3208
North Africa and Middle East	83
South Asia	196
Southeast Asia, East Asia, and Oceania	617
Sub-Saharan Africa	2
Total	15221

Appendix Table 3. GBD 2016 location hierarchy with levels

	Level
Global	0
Southeast Asia, East Asia, and Oceania	1
East Asia	2
China	3
Anhui	4
Beijing	4
Chongqing	4
Fujian	4
Gansu	4
Guangdong	4
Guangxi	4
Guizhou	4
Hainan	4
Hebei	4
Heilongjiang	4
Henan	4
Hong Kong Special Administrative Region of China	4
Hubei	4
Hunan	4
Inner Mongolia	4
Jiangsu	4
Jiangxi	4
Jilin	4
Liaoning	4
Macao Special Administrative Region of China	4
Ningxia	4
Qinghai	4
Shaanxi	4
Shandong	4
Shanghai	4
Shanxi	4
Sichuan	4
Tianjin	4
Tibet	4
Xinjiang	4
Yunnan	4
Zhejiang	4
North Korea	3
Taiwan (Province of China)	3
Southeast Asia	2
Cambodia	3
Indonesia	3
Aceh	4
Bali	4

Appendix Table 3. GBD 2016 location hierarchy with levels	
	**Level**
Bangka-Belitung Islands	4
Banten	4
Bengkulu	4
Gorontalo	4
Jakarta	4
Jambi	4
Jawa Barat (West Java)	4
Jawa Tengah (Central Java)	4
Jawa Timur (East Java)	4
Kalimantan Barat (West Kalimantan)	4
Kalimantan Selatan (South Kalimantan)	4
Kalimantan Tengah (Central Kalimantan)	4
Kalimantan Timur (East Kalimantan)	4
Kalimantan Utara (North Kalimantan)	4
Kepulauan Riau (Riau Islands)	4
Lampung	4
Maluku	4
Maluku Utara (North Maluku)	4
Nusa Tenggara Barat (West Nusa Tenggara)	4
Nusa Tenggara Timur (East Nusa Tenggara)	4
Papua	4
Papua Barat (West Papua)	4
Riau	4
Sulawesi Barat (West Sulawesi)	4
Sulawesi Selatan (South Sulawesi)	4
Sulawesi Tengah (Central Sulawesi)	4
Sulawesi Tenggara (Southeast Sulawesi)	4
Sulawesi Utara (North Sulawesi)	4
Sumatera Barat (West Sumatra)	4
Sumatera Selatan (South Sumatra)	4
Sumatera Utara (North Sumatra)	4
Yogyakarta	4
Laos	3
Malaysia	3
Maldives	3
Mauritius	3
Myanmar	3
Philippines	3
Sri Lanka	3
Seychelles	3
Thailand	3
Timor-Leste	3
Vietnam	3
Oceania	2

Appendix Table 3. GBD 2016 location hierarchy with levels	
	Level
Federated States of Micronesia	3
Fiji	3
Kiribati	3
Marshall Islands	3
Papua New Guinea	3
Samoa	3
Solomon Islands	3
Tonga	3
Vanuatu	3
Central Europe, Eastern Europe, and Central Asia	1
Central Asia	2
Armenia	3
Azerbaijan	3
Georgia	3
Kazakhstan	3
Kyrgyzstan	3
Mongolia	3
Tajikistan	3
Turkmenistan	3
Uzbekistan	3
Central Europe	2
Albania	3
Bosnia and Herzegovina	3
Bulgaria	3
Croatia	3
Czech Republic	3
Hungary	3
Macedonia	3
Montenegro	3
Poland	3
Romania	3
Serbia	3
Slovakia	3
Slovenia	3
Eastern Europe	2
Belarus	3
Estonia	3
Latvia	3
Lithuania	3
Moldova	3
Russia	3
Ukraine	3
High-income	1
High-income Asia Pacific	2

Appendix Table 3. GBD 2016 location hierarchy with levels	
	Level
Brunei	3
Japan	3
Aichi	4
Akita	4
Aomori	4
Chiba	4
Ehime	4
Fukui	4
Fukuoka	4
Fukushima	4
Gifu	4
Gunma	4
Hiroshima	4
Hokkaido	4
Hyogo	4
Ibaraki	4
Ishikawa	4
Iwate	4
Kagawa	4
Kagoshima	4
Kanagawa	4
Kochi	4
Kumamoto	4
Kyoto	4
Mie	4
Miyagi	4
Miyazaki	4
Nagano	4
Nagasaki	4
Nara	4
Niigata	4
Oita	4
Okayama	4
Okinawa	4
Osaka	4
Saga	4
Saitama	4
Shiga	4
Shimane	4
Shizuoka	4
Tochigi	4
Tokushima	4
Tokyo	4
Tottori	4

Appendix Table 3. GBD 2016 location hierarchy with levels	
	Level
Toyama	4
Wakayama	4
Yamagata	4
Yamaguchi	4
Yamanashi	4
South Korea	3
Singapore	3
Australasia	2
Australia	3
New Zealand	3
Western Europe	2
Andorra	3
Austria	3
Belgium	3
Cyprus	3
Denmark	3
Finland	3
France	3
Germany	3
Greece	3
Greenland	3
Iceland	3
Ireland	3
Israel	3
Italy	3
Luxembourg	3
Malta	3
Netherlands	3
Norway	3
Portugal	3
Spain	3
Sweden	3
Stockholm	4
Sweden except Stockholm	4
Switzerland	3
United Kingdom	3
England	4
East Midlands	5
Derby	6
Derbyshire	6
Leicester	6
Leicestershire	6
Lincolnshire	6
Northamptonshire	6

Appendix Table 3. GBD 2016 location hierarchy with levels	
	Level
Nottingham	6
Nottinghamshire	6
Rutland	6
East of England	5
Bedford	6
Cambridgeshire	6
Central Bedfordshire	6
Essex	6
Hertfordshire	6
Luton	6
Norfolk	6
Peterborough	6
Southend-on-Sea	6
Suffolk	6
Thurrock	6
Greater London	5
Barking and Dagenham	6
Barnet	6
Bexley	6
Brent	6
Bromley	6
Camden	6
Croydon	6
Ealing	6
Enfield	6
Greenwich	6
Hackney	6
Hammersmith and Fulham	6
Haringey	6
Harrow	6
Havering	6
Hillingdon	6
Hounslow	6
Islington	6
Kensington and Chelsea	6
Kingston upon Thames	6
Lambeth	6
Lewisham	6
Merton	6
Newham	6
Redbridge	6
Richmond upon Thames	6
Southwark	6
Sutton	6

Appendix Table 3. GBD 2016 location hierarchy with levels	
	Level
Tower Hamlets	6
Waltham Forest	6
Wandsworth	6
Westminster	6
North East England	5
County Durham	6
Darlington	6
Gateshead	6
Hartlepool	6
Middlesbrough	6
Newcastle upon Tyne	6
North Tyneside	6
Northumberland	6
Redcar and Cleveland	6
South Tyneside	6
Stockton-on-Tees	6
Sunderland	6
North West England	5
Blackburn with Darwen	6
Blackpool	6
Bolton	6
Bury	6
Cheshire East	6
Cheshire West and Chester	6
Cumbria	6
Halton	6
Knowsley	6
Lancashire	6
Liverpool	6
Manchester	6
Oldham	6
Rochdale	6
Salford	6
Sefton	6
St Helens	6
Stockport	6
Tameside	6
Trafford	6
Warrington	6
Wigan	6
Wirral	6
South East England	5
Bracknell Forest	6
Brighton and Hove	6

Appendix Table 3. GBD 2016 location hierarchy with levels	
	Level
Buckinghamshire	6
East Sussex	6
Hampshire	6
Isle of Wight	6
Kent	6
Medway	6
Milton Keynes	6
Oxfordshire	6
Portsmouth	6
Reading	6
Slough	6
Southampton	6
Surrey	6
West Berkshire	6
West Sussex	6
Windsor and Maidenhead	6
Wokingham	6
South West England	5
Bath and North East Somerset	6
Bournemouth	6
Bristol, City of	6
Cornwall	6
Devon	6
Dorset	6
Gloucestershire	6
North Somerset	6
Plymouth	6
Poole	6
Somerset	6
South Gloucestershire	6
Swindon	6
Torbay	6
Wiltshire	6
West Midlands	5
Birmingham	6
Coventry	6
Dudley	6
Herefordshire, County of	6
Sandwell	6
Shropshire	6
Solihull	6
Staffordshire	6
Stoke-on-Trent	6
Telford and Wrekin	6

Appendix Table 3. GBD 2016 location hierarchy with levels	
	Level
Walsall	6
Warwickshire	6
Wolverhampton	6
Worcestershire	6
Yorkshire and the Humber	5
Barnsley	6
Bradford	6
Calderdale	6
Doncaster	6
East Riding of Yorkshire	6
Kingston upon Hull, City of	6
Kirklees	6
Leeds	6
North East Lincolnshire	6
North Lincolnshire	6
North Yorkshire	6
Rotherham	6
Sheffield	6
Wakefield	6
York	6
Northern Ireland	4
Scotland	4
Wales	4
Southern Latin America	2
Argentina	3
Chile	3
Uruguay	3
High-income North America	2
Canada	3
United States	3
Alabama	4
Alaska	4
Arizona	4
Arkansas	4
California	4
Colorado	4
Connecticut	4
Delaware	4
District of Columbia	4
Florida	4
Georgia	4
Hawaii	4
Idaho	4
Illinois	4

Appendix Table 3. GBD 2016 location hierarchy with levels	
	Level
Indiana	4
Iowa	4
Kansas	4
Kentucky	4
Louisiana	4
Maine	4
Maryland	4
Massachusetts	4
Michigan	4
Minnesota	4
Mississippi	4
Missouri	4
Montana	4
Nebraska	4
Nevada	4
New Hampshire	4
New Jersey	4
New Mexico	4
New York	4
North Carolina	4
North Dakota	4
Ohio	4
Oklahoma	4
Oregon	4
Pennsylvania	4
Puerto Rico	4
Rhode Island	4
South Carolina	4
South Dakota	4
Tennessee	4
Texas	4
Utah	4
Vermont	4
Virginia	4
Washington	4
West Virginia	4
Wisconsin	4
Wyoming	4
Latin America and Caribbean	1
Caribbean	2
Antigua and Barbuda	3
The Bahamas	3
Barbados	3
Belize	3

Appendix Table 3. GBD 2016 location hierarchy with levels	
	Level
Bermuda	3
Cuba	3
Dominica	3
Dominican Republic	3
Grenada	3
Guyana	3
Haiti	3
Jamaica	3
Saint Lucia	3
Saint Vincent and the Grenadines	3
Suriname	3
Trinidad and Tobago	3
Andean Latin America	2
Bolivia	3
Ecuador	3
Peru	3
Central Latin America	2
Colombia	3
Costa Rica	3
El Salvador	3
Guatemala	3
Honduras	3
Mexico	3
Aguascalientes	4
Baja California	4
Baja California Sur	4
Campeche	4
Chiapas	4
Chihuahua	4
Coahuila	4
Colima	4
Distrito Federal	4
Durango	4
Guanajuato	4
Guerrero	4
Hidalgo	4
Jalisco	4
Mexico	4
Michoacan de Ocampo	4
Morelos	4
Nayarit	4
Nuevo Leon	4
Oaxaca	4
Puebla	4

Appendix Table 3. GBD 2016 location hierarchy with levels	
	Level
Queretaro	4
Quintana Roo	4
San Luis Potosi	4
Sinaloa	4
Sonora	4
Tabasco	4
Tamaulipas	4
Tlaxcala	4
Veracruz de Ignacio de la Llave	4
Yucatan	4
Zacatecas	4
Nicaragua	3
Panama	3
Venezuela	3
Tropical Latin America	2
Brazil	3
Acre	4
Alagoas	4
Amapa	4
Amazonas	4
Bahia	4
Ceara	4
Distrito Federal	4
Espirito Santo	4
Goias	4
Maranhao	4
Mato Grosso	4
Mato Grosso do Sul	4
Minas Gerais	4
Para	4
Paraiba	4
Parana	4
Pernambuco	4
Piaui	4
Rio de Janeiro	4
Rio Grande do Norte	4
Rio Grande do Sul	4
Rondonia	4
Roraima	4
Santa Catarina	4
Sao Paulo	4
Sergipe	4
Tocantins	4
Paraguay	3

Appendix Table 3. GBD 2016 location hierarchy with levels	
	Level
North Africa and Middle East	1
North Africa and Middle East	2
Afghanistan	3
Algeria	3
Bahrain	3
Egypt	3
Iran	3
Iraq	3
Jordan	3
Kuwait	3
Lebanon	3
Libya	3
Morocco	3
Palestine	3
Oman	3
Qatar	3
Sudan	3
Saudi Arabia	3
Syria	3
Tunisia	3
Turkey	3
United Arab Emirates	3
Yemen	3
South Asia	1
South Asia	2
Bangladesh	3
Bhutan	3
India	3
Andaman and Nicobar Islands, Rural	4
Andaman and Nicobar Islands, Urban	4
Andhra Pradesh, Rural	4
Andhra Pradesh, Urban	4
Arunachal Pradesh, Rural	4
Arunachal Pradesh, Urban	4
Assam, Rural	4
Assam, Urban	4
Bihar, Rural	4
Bihar, Urban	4
Chandigarh, Rural	4
Chandigarh, Urban	4
Chhattisgarh, Rural	4
Chhattisgarh, Urban	4
Dadra and Nagar Haveli, Rural	4
Dadra and Nagar Haveli, Urban	4

Appendix Table 3. GBD 2016 location hierarchy with levels	
	Level
Daman and Diu, Rural	4
Daman and Diu, Urban	4
Delhi, Rural	4
Delhi, Urban	4
Goa, Rural	4
Goa, Urban	4
Gujarat, Rural	4
Gujarat, Urban	4
Haryana, Rural	4
Haryana, Urban	4
Himachal Pradesh, Rural	4
Himachal Pradesh, Urban	4
Jammu and Kashmir, Rural	4
Jammu and Kashmir, Urban	4
Jharkhand, Rural	4
Jharkhand, Urban	4
Karnataka, Rural	4
Karnataka, Urban	4
Kerala, Rural	4
Kerala, Urban	4
Lakshadweep, Rural	4
Lakshadweep, Urban	4
Madhya Pradesh, Rural	4
Madhya Pradesh, Urban	4
Maharashtra, Rural	4
Maharashtra, Urban	4
Manipur, Rural	4
Manipur, Urban	4
Meghalaya, Rural	4
Meghalaya, Urban	4
Mizoram, Rural	4
Mizoram, Urban	4
Nagaland, Rural	4
Nagaland, Urban	4
Orissa, Rural	4
Orissa, Urban	4
Puducherry, Rural	4
Puducherry, Urban	4
Punjab, Rural	4
Punjab, Urban	4
Rajasthan, Rural	4
Rajasthan, Urban	4
Sikkim, Rural	4
Sikkim, Urban	4

Appendix Table 3. GBD 2016 location hierarchy with levels	
	Level
Tamil Nadu, Rural	4
Tamil Nadu, Urban	4
Telangana, Rural	4
Telangana, Urban	4
Tripura, Rural	4
Tripura, Urban	4
Uttar Pradesh, Rural	4
Uttar Pradesh, Urban	4
Uttarakhand, Rural	4
Uttarakhand, Urban	4
West Bengal, Rural	4
West Bengal, Urban	4
Nepal	3
Pakistan	3
Sub-Saharan Africa	1
Central Sub-Saharan Africa	2
Angola	3
Central African Republic	3
Congo (Brazzaville)	3
Democratic Republic of the Congo	3
Equatorial Guinea	3
Gabon	3
Eastern Sub-Saharan Africa	2
Burundi	3
Comoros	3
Djibouti	3
Eritrea	3
Ethiopia	3
Kenya	3
Baringo	4
Bomet	4
Bungoma	4
Busia	4
Elgeyo-Marakwet	4
Embu	4
Garissa	4
HomaBay	4
Isiolo	4
Kajiado	4
Kakamega	4
Kericho	4
Kiambu	4
Kilifi	4
Kirinyaga	4

Appendix Table 3. GBD 2016 location hierarchy with levels	
	Level
Kisii	4
Kisumu	4
Kitui	4
Kwale	4
Laikipia	4
Lamu	4
Machakos	4
Makueni	4
Mandera	4
Marsabit	4
Meru	4
Migori	4
Mombasa	4
Murang'a	4
Nairobi	4
Nakuru	4
Nandi	4
Narok	4
Nyamira	4
Nyandarua	4
Nyeri	4
Samburu	4
Siaya	4
TaitaTaveta	4
TanaRiver	4
TharakaNithi	4
TransNzoia	4
Turkana	4
UasinGishu	4
Vihiga	4
Wajir	4
WestPokot	4
Madagascar	3
Malawi	3
Mozambique	3
Rwanda	3
Somalia	3
South Sudan	3
Tanzania	3
Uganda	3
Zambia	3
Southern Sub-Saharan Africa	2
Botswana	3
Lesotho	3

Appendix Table 3. GBD 2016 location hierarchy with levels	
	Level
Namibia	3
South Africa	3
Eastern Cape	4
Free State	4
Gauteng	4
KwaZulu-Natal	4
Limpopo	4
Mpumalanga	4
North-West	4
Northern Cape	4
Western Cape	4
Swaziland	3
Zimbabwe	3
Western Sub-Saharan Africa	2
Benin	3
Burkina Faso	3
Cameroon	3
Cape Verde	3
Chad	3
Cote d'Ivoire	3
The Gambia	3
Ghana	3
Guinea	3
Guinea-Bissau	3
Liberia	3
Mali	3
Mauritania	3
Niger	3
Nigeria	3
Sao Tome and Principe	3
Senegal	3
Sierra Leone	3
Togo	3

Appendix Table 4. Socio-demographic Index groupings by geography, based on 2016 values	
**Location**	**SDI Level**
Aichi	High SDI
Akita	High SDI
Alabama	High SDI
Alaska	High SDI
Andorra	High SDI
Aomori	High SDI
Arizona	High SDI
Arkansas	High SDI
Australia	High SDI
Austria	High SDI
Barking and Dagenham	High SDI
Barnet	High SDI
Barnsley	High SDI
Bath and North East Somerset	High SDI
Bedford	High SDI
Belgium	High SDI
Bexley	High SDI
Birmingham	High SDI
Blackburn with Darwen	High SDI
Blackpool	High SDI
Bolton	High SDI
Bournemouth	High SDI
Bracknell Forest	High SDI
Bradford	High SDI
Brent	High SDI
Brighton and Hove	High SDI
Bristol, City of	High SDI
Bromley	High SDI
Brunei	High SDI
Buckinghamshire	High SDI
Bury	High SDI
Calderdale	High SDI
California	High SDI
Cambridgeshire	High SDI
Camden	High SDI
Canada	High SDI
Central Bedfordshire	High SDI
Cheshire East	High SDI
Cheshire West and Chester	High SDI
Chiba	High SDI
Colorado	High SDI
Connecticut	High SDI
Cornwall	High SDI
County Durham	High SDI
Coventry	High SDI
Croatia	High SDI

Appendix Table 4. Socio-demographic Index groupings by geography, based on 2016 values	
Croydon	High SDI
Cumbria	High SDI
Cyprus	High SDI
Czech Republic	High SDI
Darlington	High SDI
Delaware	High SDI
Denmark	High SDI
Derby	High SDI
Derbyshire	High SDI
Devon	High SDI
District of Columbia	High SDI
Doncaster	High SDI
Dorset	High SDI
Dudley	High SDI
Ealing	High SDI
East Riding of Yorkshire	High SDI
East Sussex	High SDI
Ehime	High SDI
Enfield	High SDI
Essex	High SDI
Estonia	High SDI
Finland	High SDI
Florida	High SDI
France	High SDI
Fukui	High SDI
Fukuoka	High SDI
Fukushima	High SDI
Gateshead	High SDI
Georgia	High SDI
Germany	High SDI
Gifu	High SDI
Gloucestershire	High SDI
Greece	High SDI
Greenwich	High SDI
Gunma	High SDI
Hackney	High SDI
Halton	High SDI
Hammersmith and Fulham	High SDI
Hampshire	High SDI
Haringey	High SDI
Harrow	High SDI
Hartlepool	High SDI
Havering	High SDI
Hawaii	High SDI
Herefordshire, County of	High SDI
Hertfordshire	High SDI
Hillingdon	High SDI

Appendix Table 4. Socio-demographic Index groupings by geography, based on 2016 values	
Hiroshima	High SDI
Hokkaido	High SDI
Hounslow	High SDI
Hyogo	High SDI
Ibaraki	High SDI
Iceland	High SDI
Idaho	High SDI
Illinois	High SDI
Indiana	High SDI
Iowa	High SDI
Ireland	High SDI
Ishikawa	High SDI
Isle of Wight	High SDI
Islington	High SDI
Italy	High SDI
Iwate	High SDI
Kagawa	High SDI
Kagoshima	High SDI
Kanagawa	High SDI
Kansas	High SDI
Kensington and Chelsea	High SDI
Kent	High SDI
Kentucky	High SDI
Kingston upon Hull, City of	High SDI
Kingston upon Thames	High SDI
Kirklees	High SDI
Knowsley	High SDI
Kumamoto	High SDI
Kyoto	High SDI
Kochi	High SDI
Lambeth	High SDI
Lancashire	High SDI
Latvia	High SDI
Leeds	High SDI
Leicester	High SDI
Leicestershire	High SDI
Lewisham	High SDI
Lincolnshire	High SDI
Lithuania	High SDI
Liverpool	High SDI
Louisiana	High SDI
Luton	High SDI
Luxembourg	High SDI
Maine	High SDI
Malta	High SDI
Manchester	High SDI
Maryland	High SDI

Appendix Table 4. Socio-demographic Index groupings by geography, based on 2016 values	
Massachusetts	High SDI
Medway	High SDI
Merton	High SDI
Michigan	High SDI
Middlesbrough	High SDI
Mie	High SDI
Milton Keynes	High SDI
Minnesota	High SDI
Mississippi	High SDI
Missouri	High SDI
Miyagi	High SDI
Miyazaki	High SDI
Montana	High SDI
Nagano	High SDI
Nagasaki	High SDI
Nara	High SDI
Nebraska	High SDI
Netherlands	High SDI
Nevada	High SDI
New Hampshire	High SDI
New Jersey	High SDI
New Mexico	High SDI
New York	High SDI
New Zealand	High SDI
Newcastle upon Tyne	High SDI
Newham	High SDI
Niigata	High SDI
Norfolk	High SDI
North Carolina	High SDI
North Dakota	High SDI
North East Lincolnshire	High SDI
North Lincolnshire	High SDI
North Somerset	High SDI
North Tyneside	High SDI
North Yorkshire	High SDI
Northamptonshire	High SDI
Northern Ireland	High SDI
Northumberland	High SDI
Norway	High SDI
Nottingham	High SDI
Nottinghamshire	High SDI
Ohio	High SDI
Okayama	High SDI
Okinawa	High SDI
Oklahoma	High SDI
Oldham	High SDI
Oregon	High SDI

Appendix Table 4. Socio-demographic Index groupings by geography, based on 2016 values	
Oxfordshire	High SDI
Pennsylvania	High SDI
Peterborough	High SDI
Plymouth	High SDI
Poland	High SDI
Poole	High SDI
Portsmouth	High SDI
Puerto Rico	High SDI
Reading	High SDI
Redbridge	High SDI
Redcar and Cleveland	High SDI
Rhode Island	High SDI
Richmond upon Thames	High SDI
Rochdale	High SDI
Rotherham	High SDI
Rutland	High SDI
Saga	High SDI
Saitama	High SDI
Salford	High SDI
Sandwell	High SDI
Scotland	High SDI
Sefton	High SDI
Sheffield	High SDI
Shiga	High SDI
Shimane	High SDI
Shizuoka	High SDI
Shropshire	High SDI
Singapore	High SDI
Slough	High SDI
Slovakia	High SDI
Slovenia	High SDI
Solihull	High SDI
Somerset	High SDI
South Carolina	High SDI
South Dakota	High SDI
South Gloucestershire	High SDI
South Korea	High SDI
South Tyneside	High SDI
Southampton	High SDI
Southend-on-Sea	High SDI
Southwark	High SDI
St Helens	High SDI
Staffordshire	High SDI
Stockholm	High SDI
Stockport	High SDI
Stockton-on-Tees	High SDI
Stoke-on-Trent	High SDI

Appendix Table 4. Socio-demographic Index groupings by geography, based on 2016 values	
Suffolk	High SDI
Sunderland	High SDI
Surrey	High SDI
Sutton	High SDI
Sweden except Stockholm	High SDI
Swindon	High SDI
Switzerland	High SDI
Taiwan (Province of China)	High SDI
Tameside	High SDI
Telford and Wrekin	High SDI
Tennessee	High SDI
Texas	High SDI
Thurrock	High SDI
Tochigi	High SDI
Tokushima	High SDI
Torbay	High SDI
Tottori	High SDI
Tower Hamlets	High SDI
Toyama	High SDI
Trafford	High SDI
Tokyo	High SDI
Utah	High SDI
Vermont	High SDI
Virgin Islands, U.S.	High SDI
Virginia	High SDI
Wakayama	High SDI
Wakefield	High SDI
Wales	High SDI
Walsall	High SDI
Waltham Forest	High SDI
Wandsworth	High SDI
Warrington	High SDI
Warwickshire	High SDI
Washington	High SDI
West Berkshire	High SDI
West Sussex	High SDI
West Virginia	High SDI
Westminster	High SDI
Wigan	High SDI
Wiltshire	High SDI
Windsor and Maidenhead	High SDI
Wirral	High SDI
Wisconsin	High SDI
Wokingham	High SDI
Wolverhampton	High SDI
Worcestershire	High SDI
Wyoming	High SDI

Appendix Table 4. Socio-demographic Index groupings by geography, based on 2016 values	
Yamagata	High SDI
Yamaguchi	High SDI
Yamanashi	High SDI
York	High SDI
Oita	High SDI
Osaka	High SDI
'Asir	High-middle SDI
Antigua and Barbuda	High-middle SDI
Argentina	High-middle SDI
Armenia	High-middle SDI
Azerbaijan	High-middle SDI
Bahah	High-middle SDI
Barbados	High-middle SDI
Beijing	High-middle SDI
Belarus	High-middle SDI
Bermuda	High-middle SDI
Bulgaria	High-middle SDI
Chile	High-middle SDI
Cuba	High-middle SDI
Delhi, Urban	High-middle SDI
Distrito Federal	High-middle SDI
Eastern Province	High-middle SDI
Georgia	High-middle SDI
Goa, Urban	High-middle SDI
Greenland	High-middle SDI
Guam	High-middle SDI
Guangdong	High-middle SDI
Ha'il	High-middle SDI
Himachal Pradesh, Urban	High-middle SDI
Hong Kong Special Administrative Region of China	High-middle SDI
Hungary	High-middle SDI
Iran	High-middle SDI
Israel	High-middle SDI
Jakarta	High-middle SDI
Jawf	High-middle SDI
Jiangsu	High-middle SDI
Jizan	High-middle SDI
Kalimantan Timur	High-middle SDI
Kalimantan Utara	High-middle SDI
Kazakhstan	High-middle SDI
Kepulauan Riau	High-middle SDI
Kuwait	High-middle SDI
Lebanon	High-middle SDI
Libya	High-middle SDI
Macao Special Administrative Region of China	High-middle SDI
Macedonia	High-middle SDI
Madinah	High-middle SDI

Appendix Table 4. Socio-demographic Index groupings by geography, based on 2016 values	
Makkah	High-middle SDI
Malaysia	High-middle SDI
Mauritius	High-middle SDI
Montenegro	High-middle SDI
Najran	High-middle SDI
Northern Borders	High-middle SDI
Northern Mariana Islands	High-middle SDI
Panama	High-middle SDI
Portugal	High-middle SDI
Qassim	High-middle SDI
Qatar	High-middle SDI
Rio de Janeiro	High-middle SDI
Riyadh	High-middle SDI
Romania	High-middle SDI
Russia	High-middle SDI
Serbia	High-middle SDI
Shanghai	High-middle SDI
Spain	High-middle SDI
Sao Paulo	High-middle SDI
Tabuk	High-middle SDI
The Bahamas	High-middle SDI
Tianjin	High-middle SDI
Trinidad and Tobago	High-middle SDI
Turkey	High-middle SDI
Turkmenistan	High-middle SDI
Ukraine	High-middle SDI
United Arab Emirates	High-middle SDI
Zhejiang	High-middle SDI
Aceh	Middle SDI
Acre	Middle SDI
Aguascalientes	Middle SDI
Albania	Middle SDI
Algeria	Middle SDI
Amapa	Middle SDI
Amazonas	Middle SDI
American Samoa	Middle SDI
Andhra Pradesh, Urban	Middle SDI
Anhui	Middle SDI
Assam, Urban	Middle SDI
Bahia	Middle SDI
Bahrain	Middle SDI
Baja California	Middle SDI
Baja California Sur	Middle SDI
Bali	Middle SDI
Bangka Belitung	Middle SDI
Banten	Middle SDI
Bengkulu	Middle SDI

Appendix Table 4. Socio-demographic Index groupings by geography, based on 2016 values	
Bosnia and Herzegovina	Middle SDI
Botswana	Middle SDI
Campeche	Middle SDI
Chhattisgarh, Urban	Middle SDI
Chiapas	Middle SDI
Chihuahua	Middle SDI
Chongqing	Middle SDI
Coahuila	Middle SDI
Colima	Middle SDI
Colombia	Middle SDI
Costa Rica	Middle SDI
Delhi, Rural	Middle SDI
Distrito Federal	Middle SDI
Dominica	Middle SDI
Dominican Republic	Middle SDI
Durango	Middle SDI
Eastern Cape	Middle SDI
Ecuador	Middle SDI
Egypt	Middle SDI
El Salvador	Middle SDI
Equatorial Guinea	Middle SDI
Espirito Santo	Middle SDI
Fiji	Middle SDI
Free State	Middle SDI
Fujian	Middle SDI
Gauteng	Middle SDI
Goa, Rural	Middle SDI
Goias	Middle SDI
Grenada	Middle SDI
Guanajuato	Middle SDI
Guangxi	Middle SDI
Guerrero	Middle SDI
Gujarat, Urban	Middle SDI
Guyana	Middle SDI
Hainan	Middle SDI
Haryana, Urban	Middle SDI
Hebei	Middle SDI
Heilongjiang	Middle SDI
Henan	Middle SDI
Hidalgo	Middle SDI
Himachal Pradesh, Rural	Middle SDI
Hubei	Middle SDI
Hunan	Middle SDI
Inner Mongolia	Middle SDI
Jalisco	Middle SDI
Jamaica	Middle SDI
Jambi	Middle SDI

Appendix Table 4. Socio-demographic Index groupings by geography, based on 2016 values	
Jammu and Kashmir, Urban	Middle SDI
Jawa Barat	Middle SDI
Jawa Timur	Middle SDI
Jharkhand, Urban	Middle SDI
Jiangxi	Middle SDI
Jilin	Middle SDI
Jordan	Middle SDI
Kalimantan Selatan	Middle SDI
Kalimantan Tengah	Middle SDI
Karnataka, Urban	Middle SDI
Kerala, Rural	Middle SDI
Kerala, Urban	Middle SDI
KwaZulu-Natal	Middle SDI
Lampung	Middle SDI
Liaoning	Middle SDI
Limpopo	Middle SDI
Madhya Pradesh, Urban	Middle SDI
Maharashtra, Urban	Middle SDI
Maldives	Middle SDI
Manipur, Urban	Middle SDI
Mato Grosso	Middle SDI
Mato Grosso do Sul	Middle SDI
Meghalaya, Urban	Middle SDI
Michoacan de Ocampo	Middle SDI
Minas Gerais	Middle SDI
Mizoram, Urban	Middle SDI
Moldova	Middle SDI
Mongolia	Middle SDI
Morelos	Middle SDI
Mpumalanga	Middle SDI
Mexico	Middle SDI
Nagaland, Urban	Middle SDI
Nayarit	Middle SDI
Ningxia	Middle SDI
North-West	Middle SDI
Northern Cape	Middle SDI
Nuevo Leon	Middle SDI
Oaxaca	Middle SDI
Oman	Middle SDI
Papua Barat	Middle SDI
Paraguay	Middle SDI
Parana	Middle SDI
Pernambuco	Middle SDI
Peru	Middle SDI
Philippines	Middle SDI
Puebla	Middle SDI
Punjab, Urban	Middle SDI

Appendix Table 4. Socio-demographic Index groupings by geography, based on 2016 values	
Qinghai	Middle SDI
Queretaro	Middle SDI
Quintana Roo	Middle SDI
Riau	Middle SDI
Rio Grande do Norte	Middle SDI
Rio Grande do Sul	Middle SDI
Rondonia	Middle SDI
Roraima	Middle SDI
Saint Lucia	Middle SDI
Saint Vincent and the Grenadines	Middle SDI
San Luis Potosi	Middle SDI
Santa Catarina	Middle SDI
Sergipe	Middle SDI
Seychelles	Middle SDI
Shaanxi	Middle SDI
Shandong	Middle SDI
Shanxi	Middle SDI
Sichuan	Middle SDI
Sikkim, Urban	Middle SDI
Sinaloa	Middle SDI
Sonora	Middle SDI
Sri Lanka	Middle SDI
Sulawesi Selatan	Middle SDI
Sulawesi Utara	Middle SDI
Sumatera Barat	Middle SDI
Sumatera Selatan	Middle SDI
Sumatera Utara	Middle SDI
Suriname	Middle SDI
Tabasco	Middle SDI
Tamaulipas	Middle SDI
Tamil Nadu, Urban	Middle SDI
Telangana, Urban	Middle SDI
Thailand	Middle SDI
Union Territories other than Delhi, Urban	Middle SDI
Tlaxcala	Middle SDI
Tocantins	Middle SDI
Tunisia	Middle SDI
Uruguay	Middle SDI
Uttarakhand, Urban	Middle SDI
Uzbekistan	Middle SDI
Venezuela	Middle SDI
Veracruz de Ignacio de la Llave	Middle SDI
Vietnam	Middle SDI
West Bengal, Urban	Middle SDI
Western Cape	Middle SDI
Xinjiang	Middle SDI
Yogyakarta	Middle SDI

Appendix Table 4. Socio-demographic Index groupings by geography, based on 2016 values	
Yucatan	Middle SDI
Yunnan	Middle SDI
Zacatecas	Middle SDI
Alagoas	Low-middle SDI
Andhra Pradesh, Rural	Low-middle SDI
Arunachal Pradesh, Rural	Low-middle SDI
Arunachal Pradesh, Urban	Low-middle SDI
Assam, Rural	Low-middle SDI
Bangladesh	Low-middle SDI
Baringo	Low-middle SDI
Belize	Low-middle SDI
Bhutan	Low-middle SDI
Bihar, Urban	Low-middle SDI
Bolivia	Low-middle SDI
Bomet	Low-middle SDI
Bungoma	Low-middle SDI
Busia	Low-middle SDI
Cambodia	Low-middle SDI
Cameroon	Low-middle SDI
Cape Verde	Low-middle SDI
Ceara	Low-middle SDI
Chhattisgarh, Rural	Low-middle SDI
Congo	Low-middle SDI
Elgeyo-Marakwet	Low-middle SDI
Embu	Low-middle SDI
Federated States of Micronesia	Low-middle SDI
Gabon	Low-middle SDI
Gansu	Low-middle SDI
Garissa	Low-middle SDI
Ghana	Low-middle SDI
Gorontalo	Low-middle SDI
Guatemala	Low-middle SDI
Guizhou	Low-middle SDI
Gujarat, Rural	Low-middle SDI
Haryana, Rural	Low-middle SDI
HomaBay	Low-middle SDI
Honduras	Low-middle SDI
Iraq	Low-middle SDI
Isiolo	Low-middle SDI
Jammu and Kashmir, Rural	Low-middle SDI
Jawa Tengah	Low-middle SDI
Jharkhand, Rural	Low-middle SDI
Kajiado	Low-middle SDI
Kakamega	Low-middle SDI
Kalimantan Barat	Low-middle SDI
Karnataka, Rural	Low-middle SDI
Kericho	Low-middle SDI

Appendix Table 4. Socio-demographic Index groupings by geography, based on 2016 values	
Kiambu	Low-middle SDI
Kilifi	Low-middle SDI
Kirinyaga	Low-middle SDI
Kisii	Low-middle SDI
Kisumu	Low-middle SDI
Kitui	Low-middle SDI
Kwale	Low-middle SDI
Kyrgyzstan	Low-middle SDI
Laikipia	Low-middle SDI
Lamu	Low-middle SDI
Laos	Low-middle SDI
Lesotho	Low-middle SDI
Machakos	Low-middle SDI
Madhya Pradesh, Rural	Low-middle SDI
Maharashtra, Rural	Low-middle SDI
Makueni	Low-middle SDI
Maluku	Low-middle SDI
Maluku Utara	Low-middle SDI
Mandera	Low-middle SDI
Manipur, Rural	Low-middle SDI
Maranhao	Low-middle SDI
Marsabit	Low-middle SDI
Marshall Islands	Low-middle SDI
Mauritania	Low-middle SDI
Meghalaya, Rural	Low-middle SDI
Meru	Low-middle SDI
Migori	Low-middle SDI
Mizoram, Rural	Low-middle SDI
Mombasa	Low-middle SDI
Morocco	Low-middle SDI
Murang'a	Low-middle SDI
Myanmar	Low-middle SDI
Nagaland, Rural	Low-middle SDI
Nairobi	Low-middle SDI
Nakuru	Low-middle SDI
Namibia	Low-middle SDI
Nandi	Low-middle SDI
Narok	Low-middle SDI
Nepal	Low-middle SDI
Nicaragua	Low-middle SDI
Nigeria	Low-middle SDI
North Korea	Low-middle SDI
Nusa Tenggara Barat	Low-middle SDI
Nusa Tenggara Timur	Low-middle SDI
Nyamira	Low-middle SDI
Nyandarua	Low-middle SDI
Nyeri	Low-middle SDI

Appendix Table 4. Socio-demographic Index groupings by geography, based on 2016 values	
Odisha, Rural	Low-middle SDI
Odisha, Urban	Low-middle SDI
Pakistan	Low-middle SDI
Papua	Low-middle SDI
Paraiba	Low-middle SDI
Para	Low-middle SDI
Piaui	Low-middle SDI
Punjab, Rural	Low-middle SDI
Rajasthan, Rural	Low-middle SDI
Rajasthan, Urban	Low-middle SDI
Samburu	Low-middle SDI
Samoa	Low-middle SDI
Siaya	Low-middle SDI
Sikkim, Rural	Low-middle SDI
Sudan	Low-middle SDI
Sulawesi Barat	Low-middle SDI
Sulawesi Tengah	Low-middle SDI
Sulawesi Tenggara	Low-middle SDI
Swaziland	Low-middle SDI
Syria	Low-middle SDI
TaitaTaveta	Low-middle SDI
Tajikistan	Low-middle SDI
Tamil Nadu, Rural	Low-middle SDI
TanaRiver	Low-middle SDI
Telangana, Rural	Low-middle SDI
TharakaNithi	Low-middle SDI
Union Territories other than Delhi, Rural	Low-middle SDI
Tibet	Low-middle SDI
Timor-Leste	Low-middle SDI
Tonga	Low-middle SDI
TransNzoia	Low-middle SDI
Tripura, Rural	Low-middle SDI
Tripura, Urban	Low-middle SDI
Turkana	Low-middle SDI
UasinGishu	Low-middle SDI
Uttar Pradesh, Rural	Low-middle SDI
Uttar Pradesh, Urban	Low-middle SDI
Uttarakhand, Rural	Low-middle SDI
Vanuatu	Low-middle SDI
Vihiga	Low-middle SDI
Wajir	Low-middle SDI
West Bengal, Rural	Low-middle SDI
WestPokot	Low-middle SDI
Zambia	Low-middle SDI
Zimbabwe	Low-middle SDI
Afghanistan	Low SDI
Angola	Low SDI

Appendix Table 4. Socio-demographic Index groupings by geography, based on 2016 values	
Benin	Low SDI
Bihar, Rural	Low SDI
Burkina Faso	Low SDI
Burundi	Low SDI
Central African Republic	Low SDI
Chad	Low SDI
Comoros	Low SDI
Cote d'Ivoire	Low SDI
Democratic Republic of the Congo	Low SDI
Djibouti	Low SDI
Eritrea	Low SDI
Ethiopia	Low SDI
Guinea	Low SDI
Guinea-Bissau	Low SDI
Haiti	Low SDI
Kiribati	Low SDI
Liberia	Low SDI
Madagascar	Low SDI
Malawi	Low SDI
Mali	Low SDI
Mozambique	Low SDI
Niger	Low SDI
Palestine	Low SDI
Papua New Guinea	Low SDI
Rwanda	Low SDI
Sao Tome and Principe	Low SDI
Senegal	Low SDI
Sierra Leone	Low SDI
Solomon Islands	Low SDI
Somalia	Low SDI
South Sudan	Low SDI
Tanzania	Low SDI
The Gambia	Low SDI
Togo	Low SDI
Uganda	Low SDI
Yemen	Low SDI

Appendix Table 5A. SDI Values by location, 1970-1989																				
**Location**	**1970**	**1971**	**1972**	**1973**	**1974**	**1975**	**1976**	**1977**	**1978**	**1979**	**1980**	**1981**	**1982**	**1983**	**1984**	**1985**	**1986**	**1987**	**1988**	**1989**
Global	0.4542	0.4623	0.4709	0.4794	0.4876	0.4952	0.5023	0.5089	0.5149	0.5204	0.5256	0.5306	0.5351	0.5397	0.5443	0.5490	0.5537	0.5586	0.5638	0.5691
Southeast Asia, East Asia, and Oceania	0.3162	0.3305	0.3449	0.3597	0.3737	0.3867	0.3984	0.4094	0.4199	0.4292	0.4375	0.4454	0.4532	0.4611	0.4697	0.4781	0.4865	0.4951	0.5039	0.5125
East Asia	0.3066	0.3233	0.3397	0.3562	0.3715	0.3854	0.3974	0.4084	0.4190	0.4282	0.4360	0.4430	0.4504	0.4582	0.4673	0.4767	0.4860	0.4958	0.5057	0.5149
China	0.2951	0.3113	0.3270	0.3429	0.3575	0.3712	0.3826	0.3930	0.4035	0.4126	0.4204	0.4276	0.4353	0.4436	0.4532	0.4632	0.4731	0.4834	0.4937	0.5031
North Korea	0.3879	0.4009	0.4123	0.4233	0.4328	0.4412	0.4487	0.4553	0.4611	0.4664	0.4714	0.4760	0.4805	0.4846	0.4882	0.4914	0.4942	0.4968	0.4993	0.5019
Taiwan (Province of China)	0.5024	0.5235	0.5415	0.5586	0.5744	0.5775	0.5873	0.6037	0.6122	0.6239	0.6356	0.6454	0.6572	0.6687	0.6812	0.6953	0.7060	0.7105	0.7181	0.7268
Hong Kong Special Administrative Region of China	0.5167	0.5269	0.5379	0.5506	0.5645	0.5776	0.5900	0.6014	0.6121	0.6225	0.6331	0.6427	0.6520	0.6617	0.6713	0.6773	0.6824	0.6881	0.6937	0.6988
Macao Special Administrative Region of China	0.5409	0.5521	0.5630	0.5734	0.5836	0.5937	0.6034	0.6133	0.6224	0.6303	0.6379	0.6463	0.6537	0.6604	0.6668	0.6725	0.6779	0.6837	0.6893	0.6948
China (without Hong Kong and Macao)	0.2900	0.3062	0.3217	0.3375	0.3519	0.3654	0.3765	0.3866	0.3968	0.4057	0.4132	0.4202	0.4280	0.4363	0.4461	0.4564	0.4665	0.4770	0.4874	0.4968
Anhui	0.2312	0.2479	0.2638	0.2798	0.2942	0.3076	0.3183	0.3279	0.3377	0.3460	0.3530	0.3596	0.3668	0.3753	0.3853	0.3958	0.4061	0.4168	0.4274	0.4373
Beijing	0.5218	0.5325	0.5429	0.5534	0.5634	0.5731	0.5817	0.5872	0.5936	0.5998	0.6057	0.6116	0.6179	0.6242	0.6311	0.6384	0.6455	0.6526	0.6592	0.6649
Chongqing	0.2204	0.2414	0.2614	0.2811	0.2988	0.3149	0.3271	0.3379	0.3489	0.3584	0.3668	0.3751	0.3843	0.3944	0.4066	0.4191	0.4311	0.4431	0.4544	0.4643
Fujian	0.2628	0.2770	0.2903	0.3043	0.3169	0.3290	0.3386	0.3474	0.3569	0.3651	0.3718	0.3781	0.3860	0.3957	0.4078	0.4203	0.4327	0.4454	0.4576	0.4687
Gansu	0.2191	0.2385	0.2579	0.2760	0.2921	0.3069	0.3186	0.3288	0.3384	0.3465	0.3526	0.3579	0.3643	0.3723	0.3817	0.3914	0.4009	0.4111	0.4213	0.4305
Guangdong	0.3331	0.3462	0.3589	0.3724	0.3850	0.3971	0.4073	0.4169	0.4272	0.4362	0.4437	0.4506	0.4583	0.4666	0.4764	0.4867	0.4966	0.5071	0.5175	0.5269
Guangxi	0.2462	0.2610	0.2748	0.2891	0.3020	0.3144	0.3244	0.3339	0.3442	0.3533	0.3612	0.3685	0.3764	0.3851	0.3952	0.4062	0.4168	0.4279	0.4387	0.4488
Guizhou	0.1183	0.1465	0.1706	0.1926	0.2112	0.2276	0.2402	0.2511	0.2621	0.2713	0.2791	0.2866	0.2946	0.3034	0.3141	0.3254	0.3364	0.3473	0.3580	0.3677
Hainan	0.3561	0.3667	0.3769	0.3877	0.3977	0.4071	0.4146	0.4212	0.4280	0.4337	0.4384	0.4428	0.4480	0.4539	0.4616	0.4696	0.4777	0.4863	0.4946	0.5024
Hebei	0.3169	0.3297	0.3415	0.3539	0.3650	0.3760	0.3855	0.3942	0.4037	0.4124	0.4198	0.4267	0.4346	0.4427	0.4524	0.4624	0.4721	0.4822	0.4921	0.5005
Heilongjiang	0.3745	0.3880	0.4011	0.4147	0.4273	0.4392	0.4489	0.4574	0.4653	0.4718	0.4772	0.4821	0.4881	0.4951	0.5038	0.5127	0.5216	0.5308	0.5399	0.5484
Henan	0.2545	0.2692	0.2825	0.2963	0.3085	0.3204	0.3302	0.3394	0.3498	0.3595	0.3677	0.3753	0.3841	0.3938	0.4054	0.4175	0.4291	0.4412	0.4529	0.4630
Hubei	0.2577	0.2786	0.2986	0.3181	0.3358	0.3518	0.3649	0.3765	0.3880	0.3980	0.4065	0.4145	0.4229	0.4317	0.4418	0.4525	0.4630	0.4737	0.4844	0.4943
Hunan	0.2320	0.2535	0.2743	0.2940	0.3116	0.3280	0.3412	0.3530	0.3649	0.3752	0.3835	0.3909	0.3994	0.4088	0.4196	0.4310	0.4420	0.4536	0.4653	0.4759
Inner Mongolia	0.2781	0.2972	0.3147	0.3324	0.3481	0.3621	0.3730	0.3827	0.3922	0.4004	0.4068	0.4132	0.4216	0.4312	0.4437	0.4560	0.4680	0.4804	0.4923	0.5025
Jiangsu	0.3200	0.3364	0.3515	0.3666	0.3803	0.3934	0.4043	0.4143	0.4251	0.4347	0.4430	0.4507	0.4593	0.4683	0.4786	0.4893	0.4997	0.5104	0.5209	0.5301
Jiangxi	0.1547	0.1898	0.2197	0.2465	0.2693	0.2891	0.3046	0.3174	0.3292	0.3389	0.3468	0.3542	0.3622	0.3713	0.3822	0.3936	0.4047	0.4163	0.4281	0.4389
Jilin	0.3492	0.3626	0.3753	0.3884	0.4003	0.4117	0.4209	0.4292	0.4376	0.4450	0.4512	0.4570	0.4638	0.4711	0.4804	0.4900	0.4995	0.5095	0.5193	0.5282
Liaoning	0.4334	0.4444	0.4549	0.4658	0.4759	0.4857	0.4938	0.5013	0.5089	0.5156	0.5214	0.5268	0.5329	0.5394	0.5472	0.5552	0.5632	0.5713	0.5793	0.5865
Ningxia	0.2262	0.2473	0.2677	0.2873	0.3049	0.3202	0.3328	0.3441	0.3546	0.3631	0.3699	0.3754	0.3817	0.3897	0.3982	0.4079	0.4184	0.4292	0.4402	0.4505
Qinghai	0.1865	0.2121	0.2362	0.2598	0.2816	0.3006	0.3159	0.3287	0.3396	0.3480	0.3543	0.3597	0.3662	0.3752	0.3857	0.3970	0.4082	0.4195	0.4311	0.4423
Shaanxi	0.2615	0.2789	0.2950	0.3112	0.3256	0.3391	0.3498	0.3595	0.3694	0.3781	0.3854	0.3925	0.4004	0.4089	0.4193	0.4301	0.4406	0.4514	0.4619	0.4713
Shandong	0.3002	0.3142	0.3270	0.3402	0.3521	0.3639	0.3739	0.3835	0.3944	0.4044	0.4131	0.4215	0.4306	0.4402	0.4511	0.4628	0.4740	0.4855	0.4967	0.5065
Shanghai	0.5669	0.5766	0.5860	0.5956	0.6048	0.6117	0.6168	0.6217	0.6271	0.6324	0.6375	0.6425	0.6477	0.6518	0.6562	0.6610	0.6657	0.6705	0.6751	0.6792
Shanxi	0.3003	0.3159	0.3309	0.3464	0.3606	0.3739	0.3849	0.3949	0.4046	0.4129	0.4198	0.4261	0.4336	0.4419	0.4518	0.4620	0.4720	0.4824	0.4927	0.5021
Sichuan	0.1488	0.1827	0.2120	0.2378	0.2604	0.2804	0.2961	0.3104	0.3250	0.3377	0.3488	0.3591	0.3693	0.3803	0.3924	0.4054	0.4178	0.4305	0.4428	0.4543
Tianjin	0.4742	0.4857	0.4968	0.5079	0.5185	0.5292	0.5387	0.5476	0.5564	0.5631	0.5693	0.5753	0.5819	0.5886	0.5960	0.6036	0.6109	0.6180	0.6247	0.6303
Tibet	0.2655	0.2749	0.2837	0.2933	0.3029	0.3120	0.3196	0.3265	0.3335	0.3393	0.3440	0.3482	0.3523	0.3570	0.3627	0.3693	0.3761	0.3831	0.3905	0.3981
Xinjiang	0.2654	0.2786	0.2910	0.3054	0.3195	0.3321	0.3434	0.3542	0.3640	0.3716	0.3774	0.3830	0.3898	0.3985	0.4104	0.4227	0.4350	0.4483	0.4619	0.4745
Yunnan	0.1374	0.1673	0.1933	0.2170	0.2376	0.2556	0.2698	0.2820	0.2936	0.3033	0.3114	0.3190	0.3269	0.3358	0.3464	0.3576	0.3687	0.3800	0.3913	0.4018
Zhejiang	0.2983	0.3136	0.3278	0.3423	0.3555	0.3683	0.3788	0.3888	0.3997	0.4096	0.4179	0.4256	0.4349	0.4450	0.4569	0.4693	0.4810	0.4931	0.5048	0.5147
Southeast Asia	0.3179	0.3263	0.3350	0.3443	0.3537	0.3629	0.3726	0.3823	0.3920	0.4017	0.4114	0.4214	0.4311	0.4406	0.4499	0.4586	0.4670	0.4752	0.4832	0.4911
Cambodia	0.2325	0.2361	0.2395	0.2424	0.2449	0.2471	0.2496	0.2514	0.2539	0.2559	0.2580	0.2607	0.2636	0.2670	0.2709	0.2750	0.2790	0.2841	0.2900	0.2958
Indonesia	0.3027	0.3112	0.3203	0.3301	0.3404	0.3505	0.3608	0.3712	0.3815	0.3919	0.4028	0.4141	0.4248	0.4355	0.4464	0.4568	0.4668	0.4760	0.4846	0.4930
Laos	0.1881	0.1928	0.1975	0.2024	0.2076	0.2129	0.2183	0.2237	0.2291	0.2342	0.2392	0.2454	0.2520	0.2588	0.2654	0.2722	0.2791	0.2855	0.2913	0.2973
Malaysia	0.3882	0.3967	0.4087	0.4209	0.4327	0.4437	0.4563	0.4693	0.4799	0.4876	0.4946	0.5021	0.5096	0.5153	0.5185	0.5231	0.5314	0.5403	0.5490	0.5568
Maldives	0.2758	0.2825	0.2893	0.2959	0.3027	0.3090	0.3149	0.3211	0.3279	0.3353	0.3443	0.3532	0.3625	0.3707	0.3794	0.3885	0.3973	0.4062	0.4151	0.4242
Myanmar	0.2138	0.2263	0.2364	0.2451	0.2532	0.2609	0.2684	0.2756	0.2823	0.2886	0.2949	0.3015	0.3081	0.3147	0.3214	0.3278	0.3337	0.3388	0.3422	0.3457
Philippines	0.3651	0.3746	0.3840	0.3937	0.4032	0.4123	0.4214	0.4304	0.4392	0.4478	0.4565	0.4652	0.4737	0.4819	0.4889	0.4946	0.4999	0.5049	0.5099	0.5148
Sri Lanka	0.3723	0.3795	0.3872	0.3955	0.4037	0.4107	0.4158	0.4193	0.4221	0.4252	0.4297	0.4366	0.4447	0.4534	0.4629	0.4733	0.4836	0.4923	0.4997	0.5062

Appendix Table 5A. SDI Values by location, 1970-1989

Location	1970	1971	1972	1973	1974	1975	1976	1977	1978	1979	1980	1981	1982	1983	1984	1985	1986	1987	1988	1989
Thailand	0.3055	0.3166	0.3278	0.3395	0.3512	0.3635	0.3771	0.3912	0.4059	0.4205	0.4348	0.4489	0.4623	0.4747	0.4864	0.4972	0.5073	0.5167	0.5259	0.5352
Timor-Leste	0.1369	0.1478	0.1579	0.1678	0.1773	0.1862	0.1959	0.2057	0.2158	0.2260	0.2362	0.2448	0.2531	0.2613	0.2695	0.2777	0.2863	0.2952	0.3044	0.3138
Vietnam	0.2908	0.2964	0.3022	0.3080	0.3137	0.3193	0.3259	0.3335	0.3407	0.3480	0.3540	0.3624	0.3697	0.3780	0.3866	0.3948	0.4027	0.4112	0.4206	0.4300
Mauritius	0.4456	0.4521	0.4656	0.4731	0.4739	0.4840	0.4951	0.5033	0.5098	0.5198	0.5317	0.5458	0.5589	0.5690	0.5785	0.5866	0.5897	0.5911	0.5955	0.6022
Seychelles	0.3200	0.3316	0.3475	0.3691	0.3968	0.4282	0.4538	0.4657	0.4715	0.4799	0.4922	0.5103	0.5241	0.5311	0.5383	0.5482	0.5601	0.5737	0.5856	0.5951
Aceh	0.2937	0.3068	0.3197	0.3326	0.3455	0.3581	0.3704	0.3825	0.3943	0.4060	0.4181	0.4305	0.4429	0.4557	0.4689	0.4816	0.4937	0.5053	0.5164	0.5269
Sumatera Utara	0.2991	0.3098	0.3204	0.3315	0.3428	0.3537	0.3645	0.3753	0.3857	0.3962	0.4071	0.4183	0.4289	0.4394	0.4501	0.4601	0.4695	0.4779	0.4852	0.4922
Sumatera Barat	0.2862	0.2959	0.3056	0.3159	0.3265	0.3366	0.3467	0.3569	0.3668	0.3767	0.3871	0.3977	0.4078	0.4181	0.4287	0.4388	0.4484	0.4577	0.4664	0.4748
Riau	0.3039	0.3180	0.3318	0.3453	0.3590	0.3724	0.3857	0.3990	0.4120	0.4248	0.4379	0.4515	0.4651	0.4791	0.4935	0.5074	0.5207	0.5332	0.5449	0.5559
Jambi	0.2590	0.2676	0.2764	0.2861	0.2962	0.3058	0.3153	0.3250	0.3345	0.3443	0.3547	0.3656	0.3760	0.3866	0.3976	0.4082	0.4185	0.4284	0.4378	0.4470
Sumatera Selatan	0.3192	0.3288	0.3384	0.3482	0.3583	0.3681	0.3779	0.3876	0.3970	0.4063	0.4160	0.4261	0.4357	0.4457	0.4561	0.4661	0.4759	0.4854	0.4945	0.5034
Bengkulu	0.2419	0.2512	0.2615	0.2723	0.2836	0.2944	0.3052	0.3161	0.3266	0.3371	0.3479	0.3589	0.3694	0.3801	0.3913	0.4022	0.4130	0.4233	0.4331	0.4427
Lampung	0.2407	0.2493	0.2588	0.2693	0.2804	0.2908	0.3013	0.3119	0.3223	0.3328	0.3439	0.3551	0.3654	0.3757	0.3861	0.3960	0.4053	0.4138	0.4215	0.4292
Bangka Belitung	0.3066	0.3153	0.3242	0.3336	0.3433	0.3526	0.3618	0.3709	0.3799	0.3890	0.3984	0.4080	0.4174	0.4269	0.4369	0.4467	0.4564	0.4659	0.4748	0.4833
Kepulauan Riau	0.3012	0.3150	0.3289	0.3429	0.3572	0.3713	0.3852	0.3989	0.4123	0.4256	0.4389	0.4531	0.4674	0.4823	0.4977	0.5129	0.5276	0.5416	0.5551	0.5681
Kalimantan Utara	0.3214	0.3349	0.3481	0.3612	0.3746	0.3880	0.4014	0.4148	0.4280	0.4411	0.4547	0.4690	0.4833	0.4981	0.5130	0.5273	0.5411	0.5541	0.5661	0.5774
Jakarta	0.4412	0.4520	0.4634	0.4752	0.4873	0.4988	0.5100	0.5213	0.5321	0.5430	0.5542	0.5656	0.5765	0.5874	0.5985	0.6090	0.6190	0.6286	0.6375	0.6461
Jawa Barat	0.3152	0.3233	0.3315	0.3404	0.3498	0.3589	0.3681	0.3774	0.3867	0.3962	0.4061	0.4164	0.4263	0.4363	0.4465	0.4561	0.4653	0.4737	0.4812	0.4885
Jawa Tengah	0.2676	0.2758	0.2844	0.2938	0.3037	0.3133	0.3230	0.3328	0.3426	0.3527	0.3632	0.3741	0.3844	0.3948	0.4054	0.4153	0.4247	0.4334	0.4415	0.4494
Yogyakarta	0.2750	0.2849	0.2956	0.3073	0.3196	0.3315	0.3436	0.3559	0.3678	0.3800	0.3925	0.4052	0.4171	0.4286	0.4402	0.4512	0.4619	0.4718	0.4810	0.4900
Jawa Timur	0.2906	0.2985	0.3067	0.3158	0.3255	0.3348	0.3443	0.3541	0.3637	0.3738	0.3843	0.3952	0.4056	0.4160	0.4266	0.4368	0.4467	0.4561	0.4648	0.4734
Banten	0.3113	0.3197	0.3281	0.3367	0.3457	0.3544	0.3630	0.3717	0.3802	0.3890	0.3981	0.4079	0.4175	0.4278	0.4387	0.4491	0.4591	0.4685	0.4775	0.4864
Bali	0.2899	0.2989	0.3080	0.3179	0.3283	0.3386	0.3491	0.3600	0.3708	0.3821	0.3939	0.4063	0.4182	0.4302	0.4424	0.4542	0.4657	0.4765	0.4865	0.4963
Nusa Tenggara Barat	0.1921	0.1997	0.2076	0.2168	0.2266	0.2358	0.2451	0.2548	0.2644	0.2745	0.2854	0.2967	0.3073	0.3177	0.3281	0.3377	0.3464	0.3540	0.3606	0.3673
Nusa Tenggara Timur	0.2101	0.2178	0.2262	0.2359	0.2462	0.2556	0.2649	0.2742	0.2832	0.2922	0.3014	0.3106	0.3187	0.3268	0.3353	0.3434	0.3513	0.3588	0.3660	0.3732
Kalimantan Barat	0.2272	0.2354	0.2437	0.2526	0.2619	0.2710	0.2803	0.2898	0.2993	0.3091	0.3194	0.3304	0.3414	0.3529	0.3650	0.3766	0.3876	0.3982	0.4085	0.4186
Kalimantan Tengah	0.3115	0.3203	0.3299	0.3401	0.3508	0.3610	0.3709	0.3809	0.3905	0.4001	0.4099	0.4199	0.4293	0.4391	0.4493	0.4592	0.4690	0.4786	0.4878	0.4968
Kalimantan Selatan	0.2992	0.3079	0.3169	0.3267	0.3370	0.3471	0.3573	0.3676	0.3778	0.3882	0.3990	0.4102	0.4209	0.4316	0.4424	0.4527	0.4626	0.4717	0.4798	0.4872
Kalimantan Timur	0.3275	0.3411	0.3545	0.3682	0.3821	0.3958	0.4093	0.4227	0.4360	0.4494	0.4631	0.4776	0.4921	0.5068	0.5218	0.5363	0.5502	0.5633	0.5755	0.5868
Sulawesi Utara	0.3454	0.3539	0.3625	0.3721	0.3821	0.3916	0.4010	0.4106	0.4200	0.4296	0.4397	0.4500	0.4595	0.4689	0.4783	0.4871	0.4954	0.5032	0.5102	0.5173
Sulawesi Tengah	0.2603	0.2695	0.2794	0.2902	0.3014	0.3118	0.3221	0.3324	0.3423	0.3523	0.3626	0.3731	0.3826	0.3922	0.4021	0.4113	0.4202	0.4287	0.4366	0.4444
Sulawesi Selatan	0.2529	0.2617	0.2709	0.2810	0.2915	0.3017	0.3120	0.3224	0.3328	0.3433	0.3544	0.3659	0.3767	0.3876	0.3986	0.4092	0.4195	0.4292	0.4382	0.4470
Sulawesi Tenggara	0.2233	0.2325	0.2419	0.2521	0.2630	0.2734	0.2839	0.2947	0.3053	0.3161	0.3274	0.3391	0.3502	0.3614	0.3730	0.3842	0.3950	0.4050	0.4142	0.4233
Gorontalo	0.2343	0.2420	0.2507	0.2607	0.2713	0.2809	0.2904	0.2999	0.3090	0.3183	0.3280	0.3375	0.3459	0.3540	0.3623	0.3701	0.3776	0.3848	0.3916	0.3989
Sulawesi Barat	0.2325	0.2395	0.2470	0.2553	0.2642	0.2728	0.2815	0.2904	0.2994	0.3087	0.3184	0.3286	0.3384	0.3483	0.3585	0.3684	0.3781	0.3874	0.3963	0.4051
Maluku	0.3580	0.3673	0.3764	0.3860	0.3957	0.4049	0.4137	0.4223	0.4302	0.4380	0.4455	0.4532	0.4603	0.4677	0.4756	0.4833	0.4910	0.4986	0.5058	0.5125
Maluku Utara	0.2682	0.2769	0.2858	0.2955	0.3057	0.3154	0.3251	0.3348	0.3443	0.3538	0.3635	0.3736	0.3832	0.3932	0.4036	0.4135	0.4231	0.4322	0.4408	0.4492
Papua Barat	0.3125	0.3216	0.3308	0.3405	0.3506	0.3602	0.3700	0.3802	0.3906	0.4016	0.4130	0.4249	0.4365	0.4483	0.4606	0.4725	0.4839	0.4947	0.5049	0.5148
Papua	0.3701	0.3775	0.3846	0.3922	0.3999	0.4072	0.4143	0.4214	0.4283	0.4351	0.4424	0.4500	0.4574	0.4653	0.4736	0.4813	0.4882	0.4944	0.5000	0.5051
Oceania	0.2806	0.2851	0.2896	0.2946	0.3001	0.3057	0.3112	0.3163	0.3214	0.3267	0.3320	0.3376	0.3429	0.3482	0.3534	0.3583	0.3632	0.3679	0.3727	0.3779
Fiji	0.4366	0.4446	0.4526	0.4613	0.4706	0.4799	0.4890	0.4980	0.5065	0.5156	0.5246	0.5336	0.5421	0.5497	0.5575	0.5647	0.5720	0.5785	0.5849	0.5919
Kiribati	0.2146	0.2182	0.2212	0.2229	0.2260	0.2310	0.2360	0.2411	0.2467	0.2538	0.2606	0.2672	0.2734	0.2789	0.2838	0.2880	0.2922	0.2954	0.2993	0.3031
Marshall Islands	0.3229	0.3296	0.3348	0.3421	0.3511	0.3597	0.3679	0.3758	0.3836	0.3917	0.3989	0.4061	0.4127	0.4196	0.4268	0.4324	0.4393	0.4462	0.4533	0.4596
Federated States of Micronesia	0.1044	0.1062	0.1202	0.1394	0.1620	0.1825	0.2026	0.2214	0.2393	0.2565	0.2741	0.2923	0.3104	0.3281	0.3456	0.3626	0.3790	0.3945	0.4088	0.4221
Papua New Guinea	0.2175	0.2211	0.2247	0.2286	0.2328	0.2369	0.2408	0.2444	0.2482	0.2521	0.2559	0.2597	0.2632	0.2670	0.2706	0.2743	0.2783	0.2827	0.2872	0.2917
Samoa	0.2867	0.2941	0.3011	0.3098	0.3191	0.3277	0.3368	0.3455	0.3536	0.3625	0.3717	0.3806	0.3891	0.3971	0.4048	0.4123	0.4200	0.4278	0.4353	0.4432
Solomon Islands	0.2234	0.2272	0.2307	0.2343	0.2396	0.2437	0.2484	0.2533	0.2579	0.2639	0.2692	0.2746	0.2796	0.2844	0.2893	0.2940	0.2984	0.3031	0.3078	0.3128
Tonga	0.3194	0.3259	0.3321	0.3385	0.3451	0.3517	0.3580	0.3644	0.3704	0.3757	0.3819	0.3890	0.3967	0.4031	0.4115	0.4195	0.4273	0.4349	0.4417	0.4482
Vanuatu	0.2240	0.2286	0.2326	0.2364	0.2424	0.2481	0.2538	0.2589	0.2644	0.2707	0.2765	0.2825	0.2882	0.2938	0.2997	0.3056	0.3117	0.3178	0.3235	0.3290

Appendix Table 5A. SDI Values by location, 1970-1989																				
**Location**	**1970**	**1971**	**1972**	**1973**	**1974**	**1975**	**1976**	**1977**	**1978**	**1979**	**1980**	**1981**	**1982**	**1983**	**1984**	**1985**	**1986**	**1987**	**1988**	**1989**
American Samoa	0.4512	0.4610	0.4709	0.4814	0.4923	0.5022	0.5120	0.5218	0.5316	0.5412	0.5506	0.5606	0.5691	0.5763	0.5830	0.5893	0.5954	0.6011	0.6064	0.6116
Guam	0.6312	0.6400	0.6484	0.6571	0.6662	0.6751	0.6839	0.6924	0.7009	0.7093	0.7178	0.7260	0.7329	0.7384	0.7419	0.7430	0.7427	0.7434	0.7460	0.7504
Northern Mariana Islands	0.5021	0.5124	0.5220	0.5319	0.5428	0.5543	0.5662	0.5775	0.5882	0.5988	0.6104	0.6219	0.6328	0.6430	0.6527	0.6623	0.6712	0.6801	0.6886	0.6970
Central Europe, Eastern Europe, and Central Asia	0.6597	0.6642	0.6690	0.6732	0.6772	0.6822	0.6876	0.6928	0.6977	0.7019	0.7057	0.7083	0.7097	0.7113	0.7139	0.7159	0.7175	0.7209	0.7265	0.7329
Central Asia	0.4934	0.5023	0.5107	0.5197	0.5288	0.5374	0.5459	0.5542	0.5622	0.5695	0.5761	0.5818	0.5865	0.5907	0.5945	0.5980	0.6023	0.6071	0.6124	0.6182
Armenia	0.5278	0.5374	0.5459	0.5552	0.5640	0.5717	0.5791	0.5859	0.5923	0.5979	0.6025	0.6065	0.6104	0.6146	0.6186	0.6221	0.6263	0.6307	0.6353	0.6400
Azerbaijan	0.4966	0.5075	0.5179	0.5295	0.5412	0.5522	0.5634	0.5746	0.5857	0.5962	0.6057	0.6144	0.6223	0.6299	0.6367	0.6425	0.6485	0.6539	0.6590	0.6640
Georgia	0.6176	0.6238	0.6296	0.6363	0.6431	0.6493	0.6559	0.6624	0.6687	0.6743	0.6792	0.6835	0.6875	0.6914	0.6950	0.6983	0.7021	0.7060	0.7103	0.7150
Kazakhstan	0.5381	0.5481	0.5576	0.5676	0.5775	0.5870	0.5964	0.6055	0.6142	0.6222	0.6295	0.6357	0.6408	0.6453	0.6495	0.6535	0.6581	0.6632	0.6689	0.6751
Kyrgyzstan	0.4575	0.4682	0.4786	0.4893	0.4999	0.5102	0.5204	0.5302	0.5396	0.5482	0.5559	0.5622	0.5670	0.5711	0.5747	0.5781	0.5820	0.5866	0.5919	0.5977
Mongolia	0.2833	0.2972	0.3105	0.3233	0.3354	0.3471	0.3583	0.3693	0.3800	0.3905	0.4030	0.4169	0.4309	0.4446	0.4582	0.4726	0.4888	0.5057	0.5225	0.5389
Tajikistan	0.3465	0.3584	0.3702	0.3825	0.3951	0.4074	0.4194	0.4311	0.4423	0.4526	0.4617	0.4689	0.4739	0.4777	0.4809	0.4841	0.4882	0.4939	0.5013	0.5100
Turkmenistan	0.4469	0.4550	0.4629	0.4713	0.4801	0.4888	0.4976	0.5062	0.5147	0.5228	0.5301	0.5362	0.5410	0.5450	0.5484	0.5517	0.5553	0.5596	0.5647	0.5705
Uzbekistan	0.4057	0.4148	0.4236	0.4329	0.4423	0.4515	0.4606	0.4694	0.4778	0.4856	0.4926	0.4984	0.5027	0.5064	0.5098	0.5134	0.5179	0.5233	0.5296	0.5366
Central Europe	0.6527	0.6584	0.6632	0.6651	0.6678	0.6732	0.6788	0.6847	0.6900	0.6953	0.7007	0.7051	0.7086	0.7112	0.7134	0.7165	0.7203	0.7244	0.7294	0.7356
Albania	0.4808	0.4876	0.4942	0.5006	0.5067	0.5127	0.5185	0.5241	0.5296	0.5350	0.5402	0.5455	0.5505	0.5554	0.5601	0.5642	0.5687	0.5729	0.5766	0.5810
Bosnia and Herzegovina	0.4186	0.4273	0.4361	0.4447	0.4537	0.4625	0.4707	0.4794	0.4877	0.4956	0.5024	0.5085	0.5136	0.5179	0.5222	0.5270	0.5319	0.5355	0.5393	0.5424
Bulgaria	0.6491	0.6568	0.6608	0.6611	0.6645	0.6709	0.6769	0.6837	0.6898	0.6969	0.7045	0.7103	0.7149	0.7195	0.7245	0.7284	0.7328	0.7382	0.7444	0.7521
Croatia	0.6904	0.6966	0.7024	0.7079	0.7137	0.7192	0.7244	0.7299	0.7355	0.7410	0.7459	0.7506	0.7548	0.7586	0.7627	0.7672	0.7717	0.7755	0.7789	0.7822
Czech Republic	0.7561	0.7570	0.7541	0.7492	0.7505	0.7559	0.7613	0.7652	0.7684	0.7769	0.7863	0.7903	0.7935	0.7963	0.7978	0.8002	0.8032	0.8045	0.8071	0.8099
Hungary	0.6617	0.6673	0.6711	0.6690	0.6654	0.6701	0.6790	0.6876	0.6957	0.7036	0.7114	0.7191	0.7266	0.7316	0.7334	0.7350	0.7390	0.7437	0.7479	0.7507
Macedonia	0.5953	0.6044	0.6127	0.6204	0.6275	0.6342	0.6406	0.6469	0.6530	0.6589	0.6641	0.6690	0.6735	0.6774	0.6815	0.6856	0.6905	0.6954	0.6997	0.7024
Montenegro	0.6755	0.6814	0.6873	0.6932	0.6989	0.7039	0.7087	0.7132	0.7174	0.7213	0.7249	0.7284	0.7315	0.7343	0.7370	0.7395	0.7418	0.7439	0.7460	0.7481
Poland	0.6616	0.6650	0.6694	0.6733	0.6775	0.6813	0.6867	0.6929	0.6967	0.6992	0.7021	0.7022	0.6991	0.6994	0.7028	0.7083	0.7143	0.7189	0.7240	0.7304
Romania	0.5865	0.5985	0.6091	0.6112	0.6133	0.6232	0.6302	0.6372	0.6456	0.6532	0.6608	0.6716	0.6842	0.6883	0.6874	0.6880	0.6889	0.6947	0.7028	0.7155
Serbia	0.6458	0.6494	0.6528	0.6561	0.6593	0.6623	0.6656	0.6690	0.6722	0.6752	0.6779	0.6800	0.6815	0.6828	0.6844	0.6864	0.6887	0.6912	0.6941	0.6972
Slovakia	0.7011	0.7046	0.7062	0.7084	0.7133	0.7190	0.7245	0.7297	0.7345	0.7415	0.7486	0.7531	0.7562	0.7591	0.7620	0.7658	0.7703	0.7741	0.7779	0.7814
Slovenia	0.7116	0.7174	0.7224	0.7273	0.7323	0.7370	0.7414	0.7464	0.7520	0.7588	0.7660	0.7739	0.7819	0.7886	0.7931	0.7974	0.8010	0.8034	0.8079	0.8126
Eastern Europe	0.6910	0.6950	0.6999	0.7052	0.7097	0.7146	0.7199	0.7249	0.7297	0.7336	0.7366	0.7383	0.7381	0.7391	0.7420	0.7434	0.7436	0.7467	0.7532	0.7603
Belarus	0.6529	0.6577	0.6628	0.6687	0.6747	0.6802	0.6856	0.6907	0.6955	0.6997	0.7032	0.7060	0.7083	0.7106	0.7131	0.7157	0.7189	0.7227	0.7275	0.7330
Estonia	0.7375	0.7417	0.7478	0.7534	0.7579	0.7613	0.7643	0.7682	0.7723	0.7747	0.7754	0.7758	0.7756	0.7757	0.7786	0.7804	0.7790	0.7781	0.7809	0.7881
Latvia	0.7169	0.7211	0.7255	0.7307	0.7360	0.7409	0.7461	0.7508	0.7547	0.7574	0.7589	0.7591	0.7585	0.7582	0.7590	0.7600	0.7619	0.7652	0.7702	0.7763
Lithuania	0.7130	0.7185	0.7262	0.7336	0.7389	0.7431	0.7479	0.7533	0.7589	0.7640	0.7683	0.7710	0.7715	0.7721	0.7743	0.7758	0.7773	0.7814	0.7872	0.7902
Moldova	0.5634	0.5691	0.5743	0.5800	0.5857	0.5910	0.5965	0.6017	0.6067	0.6109	0.6143	0.6169	0.6189	0.6207	0.6226	0.6247	0.6277	0.6316	0.6362	0.6414
Russia	0.7008	0.7044	0.7094	0.7145	0.7186	0.7233	0.7286	0.7336	0.7383	0.7421	0.7448	0.7460	0.7448	0.7453	0.7485	0.7494	0.7481	0.7507	0.7579	0.7657
Ukraine	0.6630	0.6678	0.6726	0.6781	0.6836	0.6888	0.6942	0.6994	0.7042	0.7085	0.7122	0.7150	0.7171	0.7191	0.7213	0.7239	0.7272	0.7314	0.7366	0.7424
High-income	0.7084	0.7170	0.7262	0.7343	0.7420	0.7488	0.7544	0.7598	0.7643	0.7682	0.7729	0.7777	0.7824	0.7873	0.7914	0.7954	0.7994	0.8027	0.8058	0.8090
High-income Asia Pacific	0.6613	0.6680	0.6762	0.6868	0.6987	0.7097	0.7190	0.7275	0.7331	0.7381	0.7446	0.7504	0.7562	0.7629	0.7694	0.7754	0.7812	0.7865	0.7928	0.7995
Brunei	0.5876	0.5947	0.6020	0.6095	0.6173	0.6253	0.6345	0.6441	0.6541	0.6642	0.6736	0.6807	0.6873	0.6937	0.7000	0.7062	0.7132	0.7209	0.7288	0.7367
Japan	0.7039	0.7101	0.7172	0.7261	0.7370	0.7469	0.7542	0.7596	0.7647	0.7703	0.7755	0.7792	0.7814	0.7838	0.7879	0.7931	0.7982	0.8034	0.8099	0.8166
South Korea	0.4634	0.4758	0.4913	0.5098	0.5286	0.5472	0.5671	0.5889	0.5995	0.6058	0.6176	0.6313	0.6488	0.6686	0.6836	0.6935	0.7042	0.7140	0.7227	0.7315
Singapore	0.5537	0.5611	0.5730	0.5948	0.6164	0.6281	0.6398	0.6525	0.6602	0.6682	0.6779	0.6859	0.6954	0.7044	0.7112	0.7171	0.7181	0.7111	0.7191	0.7340
Hokkaido	0.6991	0.7055	0.7127	0.7216	0.7321	0.7417	0.7489	0.7545	0.7598	0.7655	0.7710	0.7750	0.7776	0.7803	0.7844	0.7895	0.7943	0.7991	0.8044	0.8088
Aomori	0.6863	0.6925	0.6996	0.7085	0.7195	0.7296	0.7371	0.7429	0.7483	0.7542	0.7599	0.7639	0.7662	0.7686	0.7728	0.7784	0.7837	0.7891	0.7954	0.8012
Iwate	0.6780	0.6839	0.6909	0.6998	0.7110	0.7213	0.7288	0.7345	0.7398	0.7458	0.7516	0.7558	0.7584	0.7611	0.7655	0.7709	0.7758	0.7804	0.7862	0.7920
Miyagi	0.6883	0.6945	0.7017	0.7107	0.7219	0.7322	0.7398	0.7456	0.7511	0.7572	0.7631	0.7674	0.7703	0.7733	0.7779	0.7834	0.7887	0.7939	0.8004	0.8071
Akita	0.6865	0.6927	0.6997	0.7085	0.7192	0.7291	0.7364	0.7421	0.7475	0.7534	0.7592	0.7635	0.7661	0.7688	0.7728	0.7778	0.7823	0.7866	0.7921	0.7976
Yamagata	0.6759	0.6819	0.6888	0.6977	0.7089	0.7191	0.7267	0.7326	0.7381	0.7442	0.7502	0.7547	0.7574	0.7599	0.7637	0.7683	0.7725	0.7767	0.7825	0.7885
Fukushima	0.6799	0.6859	0.6930	0.7021	0.7136	0.7242	0.7319	0.7377	0.7431	0.7491	0.7548	0.7587	0.7610	0.7633	0.7676	0.7731	0.7782	0.7832	0.7896	0.7958

Appendix Table 5A. SDI Values by location, 1970-1989																				
Location	1970	1971	1972	1973	1974	1975	1976	1977	1978	1979	1980	1981	1982	1983	1984	1985	1986	1987	1988	1989
Ibaraki	0.6929	0.6990	0.7062	0.7153	0.7267	0.7372	0.7449	0.7508	0.7563	0.7623	0.7681	0.7721	0.7745	0.7771	0.7814	0.7869	0.7921	0.7974	0.8041	0.8109
Tochigi	0.6962	0.7025	0.7098	0.7191	0.7306	0.7411	0.7488	0.7547	0.7600	0.7657	0.7709	0.7742	0.7759	0.7777	0.7816	0.7870	0.7922	0.7976	0.8046	0.8117
Gunma	0.6938	0.6999	0.7070	0.7161	0.7272	0.7374	0.7449	0.7505	0.7558	0.7615	0.7669	0.7707	0.7729	0.7752	0.7791	0.7843	0.7892	0.7940	0.8004	0.8070
Saitama	0.6930	0.6991	0.7061	0.7150	0.7258	0.7355	0.7425	0.7478	0.7528	0.7582	0.7633	0.7669	0.7691	0.7716	0.7757	0.7809	0.7860	0.7911	0.7976	0.8043
Chiba	0.6951	0.7014	0.7087	0.7179	0.7289	0.7388	0.7461	0.7515	0.7565	0.7617	0.7665	0.7695	0.7713	0.7737	0.7783	0.7842	0.7900	0.7958	0.8029	0.8099
Tokyo	0.7595	0.7657	0.7728	0.7813	0.7914	0.8007	0.8074	0.8120	0.8163	0.8204	0.8244	0.8283	0.8319	0.8352	0.8385	0.8420	0.8455	0.8492	0.8533	0.8576
Kanagawa	0.7057	0.7117	0.7187	0.7275	0.7382	0.7479	0.7548	0.7600	0.7648	0.7702	0.7753	0.7791	0.7814	0.7839	0.7880	0.7930	0.7980	0.8030	0.8096	0.8166
Niigata	0.6876	0.6936	0.7006	0.7096	0.7208	0.7311	0.7386	0.7443	0.7497	0.7556	0.7612	0.7653	0.7676	0.7698	0.7735	0.7784	0.7830	0.7876	0.7937	0.8001
Toyama	0.6994	0.7055	0.7126	0.7216	0.7325	0.7426	0.7498	0.7553	0.7604	0.7659	0.7709	0.7743	0.7761	0.7781	0.7820	0.7872	0.7924	0.7977	0.8045	0.8114
Ishikawa	0.6929	0.6988	0.7059	0.7149	0.7260	0.7362	0.7437	0.7493	0.7546	0.7605	0.7662	0.7704	0.7729	0.7757	0.7801	0.7856	0.7906	0.7955	0.8020	0.8088
Fukui	0.6905	0.6965	0.7037	0.7129	0.7244	0.7350	0.7426	0.7483	0.7535	0.7592	0.7645	0.7681	0.7698	0.7718	0.7757	0.7810	0.7860	0.7910	0.7976	0.8044
Yamanashi	0.6892	0.6956	0.7030	0.7122	0.7233	0.7335	0.7410	0.7468	0.7522	0.7580	0.7634	0.7674	0.7696	0.7718	0.7755	0.7804	0.7851	0.7899	0.7962	0.8025
Nagano	0.6875	0.6934	0.7003	0.7092	0.7203	0.7305	0.7378	0.7435	0.7489	0.7549	0.7607	0.7652	0.7678	0.7703	0.7741	0.7788	0.7832	0.7877	0.7936	0.7998
Gifu	0.6909	0.6971	0.7043	0.7133	0.7244	0.7344	0.7418	0.7474	0.7527	0.7584	0.7639	0.7677	0.7699	0.7721	0.7761	0.7815	0.7866	0.7919	0.7986	0.8055
Shizuoka	0.7047	0.7108	0.7180	0.7270	0.7382	0.7484	0.7558	0.7612	0.7663	0.7716	0.7766	0.7798	0.7815	0.7834	0.7874	0.7927	0.7978	0.8030	0.8099	0.8170
Aichi	0.7152	0.7211	0.7280	0.7368	0.7479	0.7579	0.7651	0.7705	0.7755	0.7810	0.7862	0.7897	0.7916	0.7936	0.7974	0.8027	0.8079	0.8134	0.8204	0.8275
Mie	0.6966	0.7028	0.7099	0.7190	0.7301	0.7403	0.7477	0.7533	0.7586	0.7643	0.7697	0.7735	0.7757	0.7780	0.7822	0.7877	0.7930	0.7984	0.8052	0.8122
Shiga	0.6939	0.6999	0.7072	0.7165	0.7283	0.7391	0.7469	0.7528	0.7583	0.7642	0.7697	0.7732	0.7748	0.7768	0.7809	0.7866	0.7922	0.7978	0.8051	0.8125
Kyoto	0.7037	0.7100	0.7173	0.7264	0.7372	0.7470	0.7542	0.7598	0.7650	0.7705	0.7756	0.7790	0.7809	0.7829	0.7868	0.7921	0.7974	0.8029	0.8097	0.8157
Osaka	0.7190	0.7249	0.7318	0.7406	0.7512	0.7608	0.7677	0.7729	0.7777	0.7830	0.7879	0.7913	0.7932	0.7952	0.7990	0.8040	0.8090	0.8143	0.8211	0.8269
Hyogo	0.6936	0.6997	0.7069	0.7159	0.7268	0.7367	0.7438	0.7492	0.7542	0.7597	0.7647	0.7683	0.7704	0.7728	0.7771	0.7827	0.7883	0.7938	0.8008	0.8078
Nara	0.6761	0.6828	0.6905	0.6999	0.7108	0.7208	0.7282	0.7339	0.7391	0.7445	0.7494	0.7526	0.7544	0.7569	0.7616	0.7675	0.7733	0.7789	0.7858	0.7923
Wakayama	0.6847	0.6909	0.6980	0.7069	0.7179	0.7280	0.7353	0.7408	0.7460	0.7516	0.7569	0.7607	0.7630	0.7656	0.7699	0.7755	0.7810	0.7865	0.7934	0.8003
Tottori	0.6717	0.6779	0.6851	0.6943	0.7058	0.7164	0.7241	0.7300	0.7354	0.7414	0.7470	0.7508	0.7528	0.7550	0.7590	0.7642	0.7690	0.7737	0.7798	0.7861
Shimane	0.6683	0.6742	0.6812	0.6903	0.7019	0.7126	0.7204	0.7262	0.7317	0.7378	0.7436	0.7476	0.7499	0.7521	0.7561	0.7612	0.7659	0.7705	0.7768	0.7832
Okayama	0.6885	0.6946	0.7018	0.7109	0.7222	0.7325	0.7400	0.7456	0.7508	0.7565	0.7617	0.7653	0.7672	0.7693	0.7734	0.7789	0.7842	0.7897	0.7966	0.8036
Hiroshima	0.6977	0.7036	0.7107	0.7197	0.7309	0.7410	0.7484	0.7539	0.7590	0.7646	0.7698	0.7733	0.7752	0.7772	0.7812	0.7866	0.7919	0.7972	0.8040	0.8108
Yamaguchi	0.6952	0.7012	0.7082	0.7172	0.7281	0.7381	0.7455	0.7510	0.7562	0.7618	0.7671	0.7707	0.7728	0.7750	0.7789	0.7842	0.7893	0.7946	0.8012	0.8078
Tokushima	0.6875	0.6939	0.7011	0.7102	0.7213	0.7314	0.7390	0.7448	0.7502	0.7560	0.7615	0.7654	0.7677	0.7702	0.7744	0.7797	0.7848	0.7900	0.7964	0.8030
Kagawa	0.6908	0.6968	0.7039	0.7129	0.7240	0.7341	0.7416	0.7472	0.7526	0.7584	0.7641	0.7680	0.7703	0.7725	0.7762	0.7812	0.7860	0.7912	0.7979	0.8049
Ehime	0.6838	0.6900	0.6971	0.7060	0.7170	0.7271	0.7345	0.7402	0.7455	0.7514	0.7571	0.7613	0.7640	0.7668	0.7710	0.7762	0.7811	0.7860	0.7923	0.7986
Kochi	0.6730	0.6797	0.6873	0.6966	0.7076	0.7176	0.7251	0.7309	0.7360	0.7414	0.7462	0.7492	0.7509	0.7530	0.7572	0.7628	0.7683	0.7737	0.7803	0.7867
Fukuoka	0.6961	0.7025	0.7098	0.7189	0.7299	0.7399	0.7473	0.7530	0.7583	0.7640	0.7694	0.7730	0.7752	0.7775	0.7816	0.7870	0.7924	0.7977	0.8043	0.8107
Saga	0.6754	0.6817	0.6890	0.6982	0.7096	0.7201	0.7280	0.7339	0.7395	0.7456	0.7514	0.7553	0.7575	0.7598	0.7639	0.7694	0.7746	0.7799	0.7865	0.7929
Nagasaki	0.6751	0.6815	0.6888	0.6979	0.7092	0.7195	0.7271	0.7330	0.7383	0.7441	0.7494	0.7530	0.7551	0.7573	0.7615	0.7670	0.7722	0.7772	0.7835	0.7895
Kumamoto	0.6747	0.6811	0.6884	0.6976	0.7088	0.7190	0.7267	0.7327	0.7383	0.7443	0.7501	0.7541	0.7566	0.7590	0.7629	0.7682	0.7731	0.7781	0.7844	0.7907
Oita	0.6868	0.6929	0.6999	0.7088	0.7198	0.7299	0.7374	0.7432	0.7487	0.7547	0.7607	0.7651	0.7680	0.7709	0.7751	0.7802	0.7848	0.7894	0.7954	0.8015
Miyazaki	0.6737	0.6798	0.6869	0.6960	0.7074	0.7178	0.7256	0.7314	0.7369	0.7429	0.7486	0.7525	0.7549	0.7575	0.7619	0.7675	0.7726	0.7775	0.7838	0.7899
Kagoshima	0.6672	0.6736	0.6809	0.6902	0.7017	0.7123	0.7203	0.7264	0.7321	0.7383	0.7442	0.7483	0.7510	0.7540	0.7589	0.7650	0.7703	0.7754	0.7818	0.7882
Okinawa	0.6511	0.6568	0.6637	0.6730	0.6856	0.6972	0.7055	0.7116	0.7173	0.7237	0.7300	0.7342	0.7363	0.7385	0.7431	0.7496	0.7557	0.7618	0.7694	0.7767
Australasia	0.7137	0.7199	0.7333	0.7460	0.7568	0.7657	0.7716	0.7766	0.7812	0.7848	0.7873	0.7897	0.7927	0.7964	0.7992	0.8016	0.8051	0.8084	0.8111	0.8129
Australia	0.7139	0.7201	0.7336	0.7463	0.7569	0.7657	0.7716	0.7765	0.7812	0.7852	0.7874	0.7894	0.7922	0.7962	0.7991	0.8017	0.8059	0.8100	0.8132	0.8152
New Zealand	0.7113	0.7177	0.7306	0.7438	0.7558	0.7651	0.7709	0.7766	0.7802	0.7826	0.7864	0.7904	0.7943	0.7967	0.7989	0.8004	0.8004	0.7998	0.7999	0.8004
Western Europe	0.6881	0.6956	0.7042	0.7124	0.7202	0.7268	0.7326	0.7381	0.7434	0.7483	0.7537	0.7593	0.7651	0.7706	0.7751	0.7794	0.7837	0.7876	0.7919	0.7965
Andorra	0.8213	0.8238	0.8263	0.8288	0.8313	0.8337	0.8360	0.8384	0.8408	0.8431	0.8455	0.8478	0.8502	0.8525	0.8548	0.8571	0.8595	0.8617	0.8640	0.8660
Austria	0.7504	0.7579	0.7664	0.7732	0.7787	0.7856	0.7922	0.7968	0.7994	0.8007	0.8012	0.8029	0.8072	0.8121	0.8157	0.8176	0.8197	0.8218	0.8242	0.8268
Belgium	0.7474	0.7553	0.7655	0.7760	0.7852	0.7914	0.7964	0.8009	0.8051	0.8088	0.8130	0.8178	0.8230	0.8274	0.8311	0.8333	0.8352	0.8373	0.8395	0.8415
Cyprus	0.6340	0.6472	0.6592	0.6702	0.6784	0.6773	0.6759	0.6797	0.6824	0.6852	0.6916	0.6961	0.6988	0.7044	0.7123	0.7195	0.7257	0.7302	0.7338	0.7391
Denmark	0.8163	0.8187	0.8247	0.8307	0.8338	0.8394	0.8473	0.8519	0.8555	0.8605	0.8658	0.8673	0.8690	0.8709	0.8731	0.8756	0.8785	0.8798	0.8793	0.8790

Appendix Table 5A. SDI Values by location, 1970-1989																				
**Location**	**1970**	**1971**	**1972**	**1973**	**1974**	**1975**	**1976**	**1977**	**1978**	**1979**	**1980**	**1981**	**1982**	**1983**	**1984**	**1985**	**1986**	**1987**	**1988**	**1989**
Finland	0.7694	0.7790	0.7875	0.7921	0.7937	0.7958	0.7995	0.8040	0.8081	0.8120	0.8151	0.8164	0.8175	0.8209	0.8261	0.8316	0.8357	0.8370	0.8379	0.8396
France	0.6644	0.6711	0.6807	0.6932	0.7070	0.7180	0.7251	0.7309	0.7364	0.7397	0.7431	0.7490	0.7575	0.7647	0.7688	0.7726	0.7774	0.7825	0.7877	0.7931
Germany	0.7482	0.7583	0.7686	0.7769	0.7823	0.7856	0.7888	0.7919	0.7949	0.7976	0.8006	0.8042	0.8070	0.8094	0.8118	0.8142	0.8169	0.8196	0.8225	0.8256
Greece	0.6361	0.6440	0.6525	0.6579	0.6616	0.6670	0.6734	0.6796	0.6845	0.6905	0.6991	0.7075	0.7143	0.7219	0.7309	0.7387	0.7454	0.7510	0.7563	0.7607
Iceland	0.7482	0.7436	0.7457	0.7619	0.7733	0.7793	0.7914	0.8003	0.7996	0.7997	0.8081	0.8175	0.8230	0.8301	0.8411	0.8479	0.8475	0.8425	0.8422	0.8441
Ireland	0.6064	0.6095	0.6200	0.6305	0.6430	0.6544	0.6608	0.6664	0.6718	0.6759	0.6838	0.6942	0.7051	0.7173	0.7267	0.7328	0.7401	0.7500	0.7589	0.7638
Israel	0.6418	0.6484	0.6569	0.6658	0.6739	0.6797	0.6822	0.6813	0.6807	0.6842	0.6911	0.6982	0.7005	0.6998	0.7017	0.7078	0.7162	0.7229	0.7268	0.7306
Italy	0.6665	0.6725	0.6785	0.6845	0.6929	0.7021	0.7116	0.7213	0.7306	0.7398	0.7484	0.7554	0.7612	0.7674	0.7723	0.7764	0.7806	0.7849	0.7894	0.7939
Luxembourg	0.7975	0.8063	0.8182	0.8265	0.8306	0.8338	0.8385	0.8411	0.8438	0.8464	0.8480	0.8506	0.8534	0.8555	0.8579	0.8606	0.8642	0.8678	0.8709	0.8730
Malta	0.6349	0.6410	0.6512	0.6585	0.6610	0.6674	0.6782	0.6897	0.6993	0.7071	0.7166	0.7229	0.7277	0.7334	0.7378	0.7413	0.7434	0.7431	0.7433	0.7471
Netherlands	0.7497	0.7635	0.7779	0.7902	0.7993	0.8058	0.8111	0.8155	0.8191	0.8216	0.8246	0.8296	0.8331	0.8353	0.8376	0.8386	0.8402	0.8430	0.8461	0.8477
Norway	0.7511	0.7573	0.7670	0.7767	0.7859	0.7957	0.8047	0.8105	0.8142	0.8191	0.8243	0.8283	0.8326	0.8371	0.8403	0.8429	0.8449	0.8452	0.8447	0.8453
Portugal	0.5236	0.5328	0.5439	0.5536	0.5602	0.5643	0.5706	0.5814	0.5930	0.6022	0.6105	0.6185	0.6264	0.6343	0.6428	0.6513	0.6584	0.6651	0.6722	0.6796
Spain	0.5813	0.5869	0.5942	0.5998	0.6078	0.6160	0.6237	0.6344	0.6457	0.6577	0.6696	0.6797	0.6889	0.6978	0.7051	0.7131	0.7209	0.7273	0.7332	0.7395
Sweden	0.7557	0.7597	0.7654	0.7697	0.7753	0.7830	0.7891	0.7935	0.7957	0.7964	0.7993	0.8032	0.8063	0.8081	0.8081	0.8078	0.8085	0.8080	0.8070	0.8060
Switzerland	0.8365	0.8425	0.8492	0.8551	0.8608	0.8650	0.8671	0.8680	0.8681	0.8673	0.8669	0.8671	0.8677	0.8683	0.8686	0.8690	0.8694	0.8691	0.8686	0.8686
United Kingdom	0.6490	0.6568	0.6660	0.6754	0.6832	0.6898	0.6953	0.6992	0.7022	0.7058	0.7109	0.7167	0.7223	0.7274	0.7320	0.7370	0.7418	0.7464	0.7525	0.7583
Sweden except Stockholm	0.7254	0.7298	0.7362	0.7410	0.7472	0.7557	0.7623	0.7671	0.7697	0.7708	0.7741	0.7784	0.7819	0.7840	0.7843	0.7840	0.7850	0.7846	0.7839	0.7831
Stockholm	0.8430	0.8463	0.8494	0.8524	0.8551	0.8577	0.8600	0.8617	0.8633	0.8649	0.8664	0.8680	0.8696	0.8712	0.8731	0.8749	0.8769	0.8791	0.8805	0.8793
Northern Ireland	0.6732	0.6784	0.6845	0.6914	0.6976	0.7029	0.7076	0.7102	0.7100	0.7116	0.7182	0.7263	0.7324	0.7372	0.7418	0.7468	0.7523	0.7593	0.7678	0.7755
Scotland	0.6416	0.6507	0.6620	0.6715	0.6789	0.6853	0.6926	0.6974	0.6988	0.7011	0.7057	0.7117	0.7184	0.7238	0.7278	0.7322	0.7369	0.7415	0.7475	0.7525
Wales	0.6153	0.6231	0.6324	0.6421	0.6502	0.6570	0.6627	0.6671	0.6708	0.6751	0.6806	0.6865	0.6921	0.6974	0.7022	0.7076	0.7132	0.7185	0.7257	0.7325
England	0.6501	0.6579	0.6669	0.6763	0.6843	0.6909	0.6962	0.7000	0.7032	0.7071	0.7121	0.7179	0.7234	0.7285	0.7331	0.7381	0.7429	0.7475	0.7535	0.7592
Darlington	0.6292	0.6374	0.6471	0.6572	0.6656	0.6723	0.6778	0.6818	0.6853	0.6896	0.6952	0.7015	0.7077	0.7139	0.7194	0.7251	0.7305	0.7354	0.7409	0.7444
Northumberland	0.6175	0.6257	0.6354	0.6455	0.6539	0.6606	0.6661	0.6701	0.6737	0.6780	0.6834	0.6893	0.6951	0.7009	0.7066	0.7130	0.7196	0.7259	0.7328	0.7383
Stockton-on-Tees	0.6219	0.6309	0.6414	0.6524	0.6614	0.6688	0.6746	0.6787	0.6822	0.6864	0.6917	0.6976	0.7030	0.7084	0.7139	0.7206	0.7270	0.7331	0.7397	0.7446
Newcastle upon Tyne	0.6361	0.6446	0.6544	0.6646	0.6732	0.6803	0.6862	0.6908	0.6949	0.6998	0.7054	0.7114	0.7168	0.7218	0.7261	0.7312	0.7365	0.7427	0.7499	0.7563
North Tyneside	0.6146	0.6231	0.6329	0.6432	0.6517	0.6586	0.6643	0.6686	0.6724	0.6768	0.6820	0.6874	0.6923	0.6965	0.7006	0.7056	0.7114	0.7177	0.7252	0.7315
Redcar and Cleveland	0.5907	0.5994	0.6096	0.6203	0.6290	0.6361	0.6418	0.6458	0.6494	0.6536	0.6590	0.6649	0.6704	0.6756	0.6803	0.6855	0.6912	0.6972	0.7044	0.7103
County Durham	0.6062	0.6145	0.6243	0.6345	0.6429	0.6498	0.6554	0.6596	0.6633	0.6678	0.6733	0.6793	0.6850	0.6905	0.6957	0.7016	0.7075	0.7131	0.7192	0.7242
Gateshead	0.6211	0.6293	0.6389	0.6489	0.6572	0.6640	0.6695	0.6736	0.6772	0.6815	0.6867	0.6924	0.6977	0.7029	0.7078	0.7134	0.7191	0.7251	0.7322	0.7385
Middlesbrough	0.5937	0.6034	0.6147	0.6265	0.6362	0.6440	0.6502	0.6544	0.6579	0.6622	0.6679	0.6742	0.6802	0.6856	0.6900	0.6946	0.6994	0.7046	0.7125	0.7202
South Tyneside	0.5866	0.5954	0.6056	0.6162	0.6249	0.6321	0.6381	0.6427	0.6469	0.6515	0.6567	0.6618	0.6660	0.6692	0.6716	0.6751	0.6799	0.6859	0.6937	0.7008
Sunderland	0.6076	0.6163	0.6265	0.6371	0.6459	0.6530	0.6588	0.6629	0.6666	0.6709	0.6764	0.6825	0.6886	0.6944	0.6998	0.7059	0.7119	0.7178	0.7247	0.7308
Hartlepool	0.6017	0.6106	0.6210	0.6318	0.6408	0.6481	0.6540	0.6584	0.6622	0.6667	0.6720	0.6776	0.6827	0.6872	0.6910	0.6953	0.6995	0.7043	0.7110	0.7178
Cheshire East	0.6639	0.6718	0.6811	0.6908	0.6989	0.7055	0.7108	0.7147	0.7178	0.7215	0.7262	0.7316	0.7367	0.7419	0.7469	0.7528	0.7584	0.7636	0.7695	0.7743
Stockport	0.6388	0.6469	0.6564	0.6663	0.6746	0.6813	0.6866	0.6904	0.6934	0.6971	0.7021	0.7078	0.7136	0.7193	0.7244	0.7296	0.7343	0.7397	0.7469	0.7527
Trafford	0.6618	0.6701	0.6799	0.6899	0.6984	0.7054	0.7109	0.7149	0.7181	0.7218	0.7265	0.7317	0.7366	0.7411	0.7454	0.7504	0.7553	0.7604	0.7668	0.7724
Cheshire West and Chester	0.6509	0.6592	0.6688	0.6789	0.6873	0.6941	0.6995	0.7032	0.7062	0.7097	0.7145	0.7201	0.7256	0.7309	0.7360	0.7415	0.7466	0.7508	0.7560	0.7609
Sefton	0.6190	0.6270	0.6363	0.6460	0.6541	0.6608	0.6662	0.6701	0.6734	0.6772	0.6821	0.6876	0.6931	0.6985	0.7035	0.7092	0.7146	0.7195	0.7251	0.7299
Lancashire	0.6275	0.6362	0.6464	0.6570	0.6659	0.6731	0.6788	0.6828	0.6859	0.6896	0.6945	0.7000	0.7054	0.7106	0.7156	0.7212	0.7266	0.7317	0.7379	0.7436
Cumbria	0.6398	0.6477	0.6569	0.6665	0.6745	0.6811	0.6864	0.6903	0.6935	0.6973	0.7020	0.7074	0.7127	0.7180	0.7233	0.7295	0.7357	0.7413	0.7472	0.7514
Bolton	0.6132	0.6218	0.6318	0.6422	0.6510	0.6581	0.6636	0.6674	0.6703	0.6738	0.6785	0.6841	0.6897	0.6952	0.7006	0.7069	0.7131	0.7187	0.7251	0.7305
Wirral	0.6105	0.6190	0.6290	0.6393	0.6479	0.6549	0.6606	0.6647	0.6681	0.6720	0.6768	0.6822	0.6873	0.6919	0.6958	0.7003	0.7047	0.7095	0.7161	0.7221
Bury	0.6156	0.6244	0.6346	0.6451	0.6539	0.6611	0.6669	0.6711	0.6745	0.6783	0.6830	0.6880	0.6926	0.6965	0.6997	0.7040	0.7089	0.7140	0.7204	0.7266
St Helens	0.6091	0.6166	0.6255	0.6348	0.6425	0.6488	0.6540	0.6578	0.6611	0.6650	0.6698	0.6752	0.6806	0.6861	0.6911	0.6969	0.7029	0.7088	0.7151	0.7196
Warrington	0.6569	0.6649	0.6743	0.6841	0.6923	0.6990	0.7043	0.7080	0.7109	0.7143	0.7189	0.7242	0.7297	0.7351	0.7405	0.7468	0.7531	0.7593	0.7660	0.7708
Oldham	0.6029	0.6115	0.6216	0.6321	0.6409	0.6480	0.6536	0.6575	0.6605	0.6640	0.6686	0.6740	0.6791	0.6840	0.6885	0.6937	0.6990	0.7040	0.7101	0.7155
Rochdale	0.6005	0.6093	0.6197	0.6304	0.6394	0.6468	0.6525	0.6564	0.6594	0.6630	0.6677	0.6733	0.6785	0.6835	0.6885	0.6943	0.7001	0.7053	0.7115	0.7164

Appendix Table 5A. SDI Values by location, 1970-1989																				
**Location**	**1970**	**1971**	**1972**	**1973**	**1974**	**1975**	**1976**	**1977**	**1978**	**1979**	**1980**	**1981**	**1982**	**1983**	**1984**	**1985**	**1986**	**1987**	**1988**	**1989**
Wigan	0.6068	0.6150	0.6247	0.6347	0.6431	0.6499	0.6554	0.6593	0.6624	0.6661	0.6709	0.6763	0.6817	0.6871	0.6923	0.6982	0.7040	0.7096	0.7163	0.7223
Halton	0.6198	0.6288	0.6394	0.6503	0.6595	0.6671	0.6732	0.6775	0.6809	0.6848	0.6898	0.6953	0.7000	0.7040	0.7074	0.7110	0.7149	0.7199	0.7274	0.7343
Liverpool	0.6166	0.6257	0.6362	0.6471	0.6562	0.6638	0.6700	0.6745	0.6783	0.6825	0.6876	0.6930	0.6979	0.7020	0.7052	0.7089	0.7124	0.7160	0.7216	0.7286
Tameside	0.6061	0.6147	0.6246	0.6349	0.6436	0.6508	0.6565	0.6605	0.6638	0.6676	0.6725	0.6779	0.6830	0.6878	0.6922	0.6973	0.7023	0.7071	0.7131	0.7187
Salford	0.6237	0.6323	0.6423	0.6526	0.6613	0.6684	0.6742	0.6784	0.6818	0.6857	0.6906	0.6960	0.7013	0.7060	0.7095	0.7139	0.7186	0.7225	0.7275	0.7324
Blackburn with Darwen	0.5974	0.6071	0.6184	0.6300	0.6398	0.6478	0.6540	0.6580	0.6610	0.6646	0.6694	0.6750	0.6804	0.6852	0.6893	0.6938	0.6980	0.7022	0.7086	0.7149
Knowsley	0.5989	0.6077	0.6179	0.6286	0.6376	0.6448	0.6504	0.6541	0.6569	0.6604	0.6655	0.6717	0.6775	0.6834	0.6889	0.6945	0.6996	0.7042	0.7096	0.7149
Blackpool	0.5995	0.6077	0.6171	0.6270	0.6353	0.6423	0.6482	0.6527	0.6568	0.6615	0.6672	0.6735	0.6794	0.6849	0.6885	0.6903	0.6912	0.6935	0.6989	0.7041
Manchester	0.6451	0.6540	0.6641	0.6747	0.6837	0.6912	0.6974	0.7020	0.7058	0.7101	0.7154	0.7211	0.7261	0.7306	0.7344	0.7390	0.7441	0.7491	0.7555	0.7615
North Yorkshire	0.6461	0.6535	0.6622	0.6713	0.6789	0.6852	0.6903	0.6941	0.6974	0.7014	0.7066	0.7125	0.7183	0.7242	0.7301	0.7362	0.7419	0.7474	0.7539	0.7589
East Riding of Yorkshire	0.6324	0.6401	0.6490	0.6584	0.6663	0.6728	0.6781	0.6822	0.6858	0.6902	0.6958	0.7020	0.7082	0.7140	0.7195	0.7244	0.7267	0.7264	0.7299	0.7381
York	0.6613	0.6696	0.6791	0.6890	0.6974	0.7044	0.7103	0.7148	0.7189	0.7235	0.7289	0.7346	0.7400	0.7449	0.7487	0.7526	0.7560	0.7598	0.7658	0.7729
North East Lincolnshire	0.6249	0.6337	0.6440	0.6545	0.6635	0.6708	0.6768	0.6810	0.6845	0.6884	0.6932	0.6982	0.7025	0.7062	0.7093	0.7141	0.7207	0.7285	0.7360	0.7403
Calderdale	0.6223	0.6307	0.6405	0.6508	0.6594	0.6664	0.6720	0.6759	0.6792	0.6830	0.6879	0.6936	0.6990	0.7041	0.7094	0.7158	0.7224	0.7286	0.7356	0.7414
North Lincolnshire	0.6253	0.6337	0.6434	0.6535	0.6620	0.6690	0.6746	0.6785	0.6818	0.6857	0.6906	0.6961	0.7013	0.7060	0.7101	0.7149	0.7196	0.7241	0.7306	0.7363
Bradford	0.6000	0.6092	0.6199	0.6311	0.6404	0.6480	0.6539	0.6579	0.6609	0.6645	0.6694	0.6751	0.6806	0.6856	0.6902	0.6959	0.7017	0.7072	0.7142	0.7203
Kirklees	0.6105	0.6189	0.6288	0.6391	0.6477	0.6547	0.6603	0.6642	0.6673	0.6712	0.6763	0.6823	0.6882	0.6940	0.6994	0.7053	0.7109	0.7163	0.7229	0.7289
Leeds	0.6416	0.6497	0.6592	0.6690	0.6773	0.6842	0.6899	0.6941	0.6978	0.7020	0.7074	0.7133	0.7190	0.7243	0.7291	0.7340	0.7387	0.7433	0.7498	0.7565
Sheffield	0.6396	0.6470	0.6556	0.6646	0.6722	0.6786	0.6841	0.6883	0.6921	0.6966	0.7018	0.7074	0.7130	0.7182	0.7226	0.7274	0.7319	0.7366	0.7425	0.7481
Wakefield	0.6149	0.6229	0.6323	0.6421	0.6504	0.6572	0.6628	0.6669	0.6703	0.6744	0.6796	0.6853	0.6908	0.6958	0.7006	0.7058	0.7110	0.7163	0.7227	0.7284
Rotherham	0.6032	0.6113	0.6207	0.6306	0.6388	0.6456	0.6510	0.6549	0.6582	0.6621	0.6672	0.6729	0.6785	0.6838	0.6886	0.6941	0.6993	0.7042	0.7102	0.7155
Doncaster	0.5989	0.6070	0.6164	0.6263	0.6345	0.6413	0.6468	0.6507	0.6540	0.6578	0.6628	0.6684	0.6736	0.6788	0.6833	0.6887	0.6943	0.6999	0.7066	0.7121
Kingston upon Hull, City of	0.6056	0.6144	0.6246	0.6350	0.6439	0.6513	0.6574	0.6620	0.6659	0.6702	0.6752	0.6803	0.6846	0.6880	0.6902	0.6937	0.6998	0.7088	0.7176	0.7215
Barnsley	0.5964	0.6043	0.6136	0.6232	0.6313	0.6379	0.6433	0.6472	0.6506	0.6546	0.6597	0.6654	0.6708	0.6760	0.6812	0.6868	0.6920	0.6969	0.7027	0.7078
Northamptonshire	0.6333	0.6414	0.6506	0.6601	0.6681	0.6748	0.6804	0.6846	0.6883	0.6927	0.6983	0.7045	0.7105	0.7157	0.7200	0.7250	0.7298	0.7343	0.7404	0.7460
Leicestershire	0.6439	0.6514	0.6600	0.6690	0.6764	0.6827	0.6879	0.6919	0.6954	0.6997	0.7052	0.7113	0.7172	0.7227	0.7278	0.7335	0.7388	0.7441	0.7504	0.7557
Lincolnshire	0.6270	0.6343	0.6427	0.6514	0.6586	0.6647	0.6698	0.6736	0.6771	0.6814	0.6869	0.6931	0.6990	0.7049	0.7103	0.7162	0.7217	0.7269	0.7330	0.7379
Rutland	0.6444	0.6521	0.6609	0.6699	0.6774	0.6835	0.6884	0.6918	0.6946	0.6982	0.7032	0.7092	0.7154	0.7215	0.7283	0.7362	0.7436	0.7509	0.7596	0.7654
Derby	0.6260	0.6342	0.6436	0.6532	0.6613	0.6679	0.6730	0.6766	0.6795	0.6834	0.6891	0.6961	0.7032	0.7101	0.7163	0.7227	0.7284	0.7329	0.7382	0.7429
Derbyshire	0.6209	0.6286	0.6373	0.6463	0.6538	0.6600	0.6653	0.6692	0.6727	0.6770	0.6825	0.6886	0.6945	0.7000	0.7052	0.7108	0.7164	0.7216	0.7278	0.7329
Nottinghamshire	0.6194	0.6268	0.6353	0.6441	0.6514	0.6576	0.6627	0.6665	0.6701	0.6744	0.6799	0.6860	0.6919	0.6977	0.7031	0.7088	0.7141	0.7192	0.7254	0.7309
Nottingham	0.6365	0.6449	0.6543	0.6640	0.6723	0.6794	0.6852	0.6897	0.6937	0.6985	0.7045	0.7112	0.7178	0.7236	0.7291	0.7335	0.7371	0.7406	0.7460	0.7526
Leicester	0.6048	0.6137	0.6238	0.6341	0.6428	0.6501	0.6561	0.6603	0.6639	0.6682	0.6739	0.6805	0.6864	0.6917	0.6964	0.7019	0.7066	0.7109	0.7172	0.7243
Warwickshire	0.6513	0.6591	0.6681	0.6775	0.6855	0.6921	0.6975	0.7014	0.7048	0.7088	0.7139	0.7195	0.7251	0.7306	0.7355	0.7407	0.7457	0.7504	0.7562	0.7612
Herefordshire, County of	0.6286	0.6363	0.6454	0.6550	0.6631	0.6697	0.6751	0.6789	0.6822	0.6863	0.6915	0.6976	0.7036	0.7094	0.7149	0.7203	0.7246	0.7285	0.7335	0.7375
Solihull	0.6608	0.6686	0.6777	0.6871	0.6951	0.7017	0.7070	0.7110	0.7144	0.7184	0.7232	0.7284	0.7334	0.7379	0.7418	0.7463	0.7508	0.7554	0.7610	0.7651
Shropshire	0.6327	0.6403	0.6491	0.6584	0.6663	0.6727	0.6780	0.6819	0.6853	0.6892	0.6942	0.6997	0.7051	0.7103	0.7157	0.7216	0.7275	0.7327	0.7385	0.7429
Worcestershire	0.6253	0.6330	0.6421	0.6517	0.6597	0.6663	0.6717	0.6756	0.6789	0.6828	0.6879	0.6936	0.6992	0.7048	0.7101	0.7159	0.7214	0.7267	0.7327	0.7377
Staffordshire	0.6259	0.6338	0.6431	0.6528	0.6610	0.6677	0.6732	0.6772	0.6806	0.6845	0.6896	0.6952	0.7005	0.7057	0.7106	0.7161	0.7214	0.7267	0.7331	0.7389
Dudley	0.6150	0.6228	0.6318	0.6413	0.6492	0.6558	0.6611	0.6650	0.6683	0.6721	0.6770	0.6823	0.6877	0.6928	0.6975	0.7029	0.7084	0.7140	0.7207	0.7263
Coventry	0.6286	0.6374	0.6475	0.6580	0.6669	0.6743	0.6802	0.6844	0.6877	0.6916	0.6966	0.7020	0.7069	0.7113	0.7150	0.7193	0.7238	0.7286	0.7354	0.7424
Telford and Wrekin	0.6306	0.6392	0.6493	0.6598	0.6686	0.6759	0.6817	0.6857	0.6889	0.6928	0.6979	0.7039	0.7098	0.7155	0.7207	0.7262	0.7311	0.7359	0.7418	0.7473
Stoke-on-Trent	0.6049	0.6132	0.6227	0.6326	0.6411	0.6481	0.6537	0.6578	0.6612	0.6652	0.6702	0.6757	0.6809	0.6859	0.6912	0.6969	0.7013	0.7047	0.7098	0.7160
Walsall	0.6043	0.6123	0.6215	0.6312	0.6394	0.6461	0.6514	0.6552	0.6583	0.6619	0.6666	0.6718	0.6769	0.6816	0.6859	0.6911	0.6968	0.7024	0.7089	0.7140
Wolverhampton	0.6140	0.6220	0.6314	0.6411	0.6495	0.6563	0.6619	0.6660	0.6694	0.6733	0.6783	0.6837	0.6888	0.6935	0.6978	0.7028	0.7074	0.7117	0.7174	0.7229
Birmingham	0.6113	0.6202	0.6307	0.6415	0.6507	0.6582	0.6641	0.6682	0.6715	0.6753	0.6803	0.6858	0.6912	0.6959	0.7001	0.7049	0.7097	0.7141	0.7204	0.7266
Sandwell	0.6022	0.6103	0.6198	0.6297	0.6381	0.6450	0.6505	0.6544	0.6575	0.6611	0.6660	0.6714	0.6768	0.6817	0.6863	0.6914	0.6964	0.7015	0.7081	0.7141
Bedford	0.6509	0.6589	0.6680	0.6774	0.6852	0.6916	0.6966	0.7000	0.7028	0.7063	0.7113	0.7172	0.7227	0.7280	0.7332	0.7388	0.7446	0.7503	0.7574	0.7644
Central Bedfordshire	0.6461	0.6544	0.6638	0.6734	0.6815	0.6882	0.6935	0.6972	0.7002	0.7039	0.7089	0.7147	0.7200	0.7251	0.7296	0.7349	0.7407	0.7467	0.7536	0.7593

Appendix Table 5A. SDI Values by location, 1970-1989

Location	1970	1971	1972	1973	1974	1975	1976	1977	1978	1979	1980	1981	1982	1983	1984	1985	1986	1987	1988	1989
Suffolk	0.6425	0.6505	0.6595	0.6688	0.6765	0.6829	0.6880	0.6917	0.6947	0.6984	0.7034	0.7093	0.7150	0.7203	0.7253	0.7309	0.7358	0.7402	0.7459	0.7516
Hertfordshire	0.6700	0.6779	0.6868	0.6960	0.7037	0.7100	0.7151	0.7188	0.7219	0.7258	0.7310	0.7370	0.7429	0.7482	0.7527	0.7569	0.7604	0.7638	0.7692	0.7752
Essex	0.6392	0.6471	0.6561	0.6653	0.6730	0.6793	0.6845	0.6882	0.6914	0.6954	0.7006	0.7065	0.7122	0.7174	0.7218	0.7264	0.7303	0.7340	0.7395	0.7450
Cambridgeshire	0.6663	0.6743	0.6834	0.6927	0.7005	0.7070	0.7122	0.7161	0.7193	0.7233	0.7284	0.7341	0.7397	0.7447	0.7494	0.7550	0.7602	0.7647	0.7708	0.7767
Thurrock	0.6372	0.6459	0.6556	0.6654	0.6738	0.6808	0.6864	0.6904	0.6938	0.6976	0.7024	0.7075	0.7115	0.7145	0.7166	0.7195	0.7226	0.7261	0.7329	0.7410
Norfolk	0.6419	0.6496	0.6583	0.6672	0.6747	0.6810	0.6861	0.6899	0.6932	0.6972	0.7024	0.7082	0.7139	0.7190	0.7233	0.7279	0.7325	0.7372	0.7434	0.7492
Southend-on-Sea	0.6190	0.6272	0.6364	0.6459	0.6539	0.6606	0.6661	0.6704	0.6740	0.6782	0.6832	0.6885	0.6930	0.6965	0.6993	0.7031	0.7087	0.7158	0.7242	0.7306
Peterborough	0.6492	0.6576	0.6671	0.6768	0.6851	0.6919	0.6974	0.7013	0.7045	0.7083	0.7132	0.7187	0.7239	0.7281	0.7319	0.7369	0.7421	0.7471	0.7534	0.7588
Luton	0.6239	0.6332	0.6437	0.6544	0.6634	0.6707	0.6764	0.6802	0.6832	0.6869	0.6919	0.6978	0.7031	0.7073	0.7101	0.7126	0.7147	0.7175	0.7246	0.7332
Richmond upon Thames	0.7005	0.7073	0.7153	0.7236	0.7308	0.7357	0.7401	0.7447	0.7498	0.7545	0.7598	0.7656	0.7708	0.7752	0.7788	0.7829	0.7869	0.7913	0.7977	0.8054
Kensington and Chelsea	0.7483	0.7550	0.7604	0.7648	0.7690	0.7732	0.7777	0.7824	0.7875	0.7930	0.7984	0.8036	0.8090	0.8146	0.8204	0.8263	0.8320	0.8370	0.8398	0.8482
Barnet	0.6711	0.6778	0.6856	0.6938	0.7009	0.7069	0.7120	0.7159	0.7193	0.7235	0.7290	0.7355	0.7418	0.7476	0.7532	0.7590	0.7640	0.7689	0.7752	0.7823
Westminster	0.7662	0.7724	0.7791	0.7821	0.7852	0.7885	0.7920	0.7957	0.7995	0.8036	0.8079	0.8124	0.8166	0.8210	0.8252	0.8295	0.8307	0.8325	0.8372	0.8443
Bromley	0.6707	0.6774	0.6853	0.6935	0.7007	0.7065	0.7109	0.7157	0.7203	0.7246	0.7300	0.7358	0.7411	0.7455	0.7490	0.7527	0.7565	0.7610	0.7675	0.7739
Bexley	0.6410	0.6480	0.6560	0.6644	0.6717	0.6781	0.6834	0.6876	0.6913	0.6957	0.7011	0.7070	0.7127	0.7175	0.7215	0.7256	0.7296	0.7333	0.7386	0.7438
Redbridge	0.6441	0.6508	0.6585	0.6667	0.6738	0.6799	0.6850	0.6889	0.6923	0.6963	0.7017	0.7078	0.7131	0.7180	0.7229	0.7286	0.7339	0.7396	0.7464	0.7522
Merton	0.6623	0.6701	0.6789	0.6881	0.6961	0.7030	0.7087	0.7131	0.7168	0.7211	0.7264	0.7320	0.7367	0.7403	0.7432	0.7473	0.7518	0.7568	0.7646	0.7726
Brent	0.6643	0.6713	0.6795	0.6881	0.6955	0.7019	0.7072	0.7111	0.7143	0.7183	0.7237	0.7300	0.7358	0.7411	0.7464	0.7525	0.7588	0.7652	0.7728	0.7788
Hillingdon	0.6944	0.7017	0.7101	0.7189	0.7266	0.7332	0.7387	0.7428	0.7463	0.7504	0.7557	0.7618	0.7674	0.7721	0.7759	0.7801	0.7841	0.7880	0.7945	0.8013
Havering	0.6458	0.6523	0.6598	0.6676	0.6745	0.6805	0.6856	0.6895	0.6931	0.6974	0.7027	0.7088	0.7145	0.7196	0.7242	0.7284	0.7320	0.7353	0.7403	0.7453
Kingston upon Thames	0.6860	0.6929	0.7010	0.7095	0.7169	0.7233	0.7287	0.7329	0.7366	0.7409	0.7464	0.7524	0.7579	0.7623	0.7663	0.7700	0.7743	0.7793	0.7879	0.7962
Sutton	0.6505	0.6578	0.6662	0.6749	0.6826	0.6893	0.6950	0.6995	0.7035	0.7082	0.7138	0.7200	0.7257	0.7303	0.7336	0.7367	0.7394	0.7427	0.7490	0.7558
Harrow	0.6581	0.6653	0.6737	0.6823	0.6899	0.6963	0.7017	0.7058	0.7094	0.7137	0.7193	0.7256	0.7315	0.7366	0.7413	0.7453	0.7485	0.7514	0.7569	0.7636
Enfield	0.6554	0.6625	0.6707	0.6793	0.6868	0.6932	0.6986	0.7027	0.7063	0.7106	0.7159	0.7220	0.7275	0.7322	0.7364	0.7409	0.7450	0.7486	0.7549	0.7612
Croydon	0.6579	0.6653	0.6739	0.6829	0.6907	0.6974	0.7030	0.7073	0.7109	0.7152	0.7207	0.7268	0.7323	0.7371	0.7408	0.7451	0.7494	0.7534	0.7590	0.7651
Hammersmith and Fulham	0.7262	0.7329	0.7406	0.7486	0.7530	0.7572	0.7618	0.7667	0.7721	0.7779	0.7837	0.7893	0.7948	0.8006	0.8071	0.8140	0.8205	0.8259	0.8330	0.8406
Ealing	0.6728	0.6796	0.6876	0.6960	0.7032	0.7093	0.7142	0.7175	0.7203	0.7239	0.7293	0.7360	0.7428	0.7495	0.7571	0.7652	0.7727	0.7793	0.7863	0.7912
Greenwich	0.6388	0.6461	0.6546	0.6635	0.6713	0.6779	0.6835	0.6876	0.6912	0.6954	0.7010	0.7074	0.7133	0.7186	0.7228	0.7273	0.7316	0.7351	0.7405	0.7462
Wandsworth	0.6857	0.6928	0.7010	0.7096	0.7171	0.7235	0.7288	0.7327	0.7361	0.7402	0.7457	0.7521	0.7582	0.7641	0.7703	0.7771	0.7839	0.7909	0.7993	0.8076
Waltham Forest	0.6210	0.6282	0.6365	0.6453	0.6529	0.6593	0.6645	0.6683	0.6715	0.6755	0.6810	0.6874	0.6935	0.6992	0.7047	0.7103	0.7152	0.7197	0.7264	0.7327
Camden	0.7727	0.7779	0.7813	0.7843	0.7874	0.7908	0.7943	0.7980	0.8018	0.8059	0.8102	0.8146	0.8188	0.8231	0.8278	0.8331	0.8384	0.8433	0.8476	0.8524
Lambeth	0.6740	0.6817	0.6905	0.6997	0.7077	0.7146	0.7203	0.7245	0.7282	0.7326	0.7384	0.7451	0.7508	0.7559	0.7612	0.7663	0.7707	0.7734	0.7786	0.7866
Lewisham	0.6397	0.6470	0.6553	0.6640	0.6716	0.6781	0.6836	0.6879	0.6917	0.6963	0.7019	0.7082	0.7139	0.7189	0.7238	0.7284	0.7323	0.7358	0.7408	0.7466
Hounslow	0.6772	0.6856	0.6950	0.7048	0.7134	0.7209	0.7271	0.7319	0.7359	0.7402	0.7452	0.7499	0.7534	0.7548	0.7548	0.7571	0.7613	0.7657	0.7733	0.7841
Southwark	0.6910	0.6986	0.7073	0.7164	0.7243	0.7311	0.7365	0.7404	0.7436	0.7477	0.7534	0.7603	0.7664	0.7723	0.7775	0.7823	0.7866	0.7909	0.7977	0.8059
Newham	0.6253	0.6335	0.6428	0.6525	0.6609	0.6681	0.6738	0.6779	0.6810	0.6848	0.6899	0.6957	0.7006	0.7045	0.7077	0.7118	0.7162	0.7217	0.7285	0.7339
Barking and Dagenham	0.6302	0.6375	0.6459	0.6546	0.6623	0.6690	0.6747	0.6791	0.6829	0.6872	0.6923	0.6976	0.7021	0.7049	0.7072	0.7107	0.7150	0.7186	0.7238	0.7284
Haringey	0.6626	0.6706	0.6797	0.6892	0.6975	0.7047	0.7108	0.7156	0.7198	0.7246	0.7304	0.7363	0.7414	0.7448	0.7465	0.7482	0.7502	0.7521	0.7575	0.7653
Hackney	0.7065	0.7131	0.7210	0.7289	0.7360	0.7418	0.7459	0.7480	0.7490	0.7508	0.7549	0.7607	0.7662	0.7714	0.7771	0.7816	0.7843	0.7871	0.7937	0.8002
Islington	0.7295	0.7368	0.7451	0.7537	0.7613	0.7679	0.7733	0.7773	0.7795	0.7820	0.7862	0.7917	0.7968	0.8017	0.8070	0.8125	0.8173	0.8214	0.8266	0.8312
Tower Hamlets	0.6781	0.6857	0.6947	0.7036	0.7115	0.7178	0.7219	0.7234	0.7233	0.7239	0.7270	0.7321	0.7361	0.7404	0.7470	0.7569	0.7678	0.7771	0.7850	0.7902
Wokingham	0.7060	0.7128	0.7205	0.7285	0.7352	0.7407	0.7447	0.7480	0.7505	0.7537	0.7580	0.7630	0.7680	0.7733	0.7783	0.7843	0.7904	0.7963	0.8026	0.8064
Buckinghamshire	0.6865	0.6936	0.7019	0.7105	0.7176	0.7233	0.7277	0.7305	0.7327	0.7358	0.7405	0.7463	0.7523	0.7580	0.7633	0.7683	0.7727	0.7764	0.7812	0.7858
Surrey	0.6931	0.7003	0.7086	0.7172	0.7244	0.7303	0.7351	0.7385	0.7413	0.7448	0.7495	0.7547	0.7596	0.7636	0.7669	0.7701	0.7728	0.7754	0.7807	0.7867
Windsor and Maidenhead	0.7020	0.7095	0.7182	0.7271	0.7346	0.7407	0.7457	0.7492	0.7522	0.7558	0.7604	0.7656	0.7700	0.7736	0.7763	0.7792	0.7816	0.7830	0.7857	0.7904
West Berkshire	0.6961	0.7041	0.7133	0.7227	0.7306	0.7369	0.7418	0.7449	0.7473	0.7502	0.7547	0.7601	0.7652	0.7696	0.7745	0.7801	0.7860	0.7914	0.7976	0.8033
Hampshire	0.6694	0.6768	0.6853	0.6940	0.7013	0.7072	0.7117	0.7147	0.7170	0.7202	0.7250	0.7308	0.7366	0.7422	0.7472	0.7520	0.7561	0.7601	0.7657	0.7709
Bracknell Forest	0.6803	0.6874	0.6957	0.7043	0.7114	0.7171	0.7213	0.7238	0.7257	0.7285	0.7333	0.7397	0.7463	0.7532	0.7596	0.7657	0.7713	0.7766	0.7840	0.7911
West Sussex	0.6637	0.6709	0.6793	0.6879	0.6951	0.7010	0.7056	0.7088	0.7115	0.7150	0.7198	0.7257	0.7313	0.7366	0.7412	0.7459	0.7499	0.7534	0.7585	0.7636

Appendix Table 5A. SDI Values by location, 1970-1989

Location	1970	1971	1972	1973	1974	1975	1976	1977	1978	1979	1980	1981	1982	1983	1984	1985	1986	1987	1988	1989
Oxfordshire	0.6910	0.6980	0.7060	0.7143	0.7212	0.7268	0.7313	0.7343	0.7368	0.7402	0.7449	0.7507	0.7565	0.7621	0.7674	0.7731	0.7780	0.7815	0.7859	0.7901
Reading	0.6916	0.6997	0.7089	0.7183	0.7264	0.7330	0.7382	0.7418	0.7447	0.7483	0.7533	0.7590	0.7643	0.7687	0.7719	0.7752	0.7791	0.7834	0.7903	0.7978
Kent	0.6525	0.6599	0.6685	0.6774	0.6848	0.6909	0.6958	0.6992	0.7021	0.7057	0.7105	0.7160	0.7212	0.7255	0.7291	0.7326	0.7360	0.7395	0.7449	0.7500
Brighton and Hove	0.6686	0.6763	0.6850	0.6939	0.7015	0.7078	0.7130	0.7171	0.7208	0.7250	0.7299	0.7351	0.7397	0.7431	0.7453	0.7477	0.7508	0.7550	0.7631	0.7715
Medway	0.6272	0.6350	0.6441	0.6533	0.6611	0.6674	0.6722	0.6754	0.6778	0.6810	0.6858	0.6914	0.6966	0.7013	0.7056	0.7105	0.7154	0.7202	0.7266	0.7328
East Sussex	0.6414	0.6485	0.6566	0.6650	0.6720	0.6778	0.6823	0.6854	0.6880	0.6914	0.6962	0.7019	0.7076	0.7129	0.7180	0.7231	0.7276	0.7318	0.7370	0.7417
Portsmouth	0.6620	0.6704	0.6799	0.6897	0.6981	0.7051	0.7109	0.7152	0.7188	0.7230	0.7285	0.7345	0.7395	0.7436	0.7455	0.7481	0.7505	0.7541	0.7603	0.7683
Isle of Wight	0.6408	0.6478	0.6559	0.6642	0.6712	0.6768	0.6813	0.6844	0.6870	0.6903	0.6950	0.7005	0.7056	0.7102	0.7144	0.7191	0.7245	0.7303	0.7369	0.7415
Milton Keynes	0.6901	0.6990	0.7092	0.7195	0.7283	0.7356	0.7416	0.7461	0.7497	0.7534	0.7573	0.7608	0.7624	0.7620	0.7629	0.7681	0.7754	0.7826	0.7899	0.7947
Southampton	0.6651	0.6731	0.6823	0.6917	0.6997	0.7063	0.7117	0.7155	0.7188	0.7228	0.7280	0.7338	0.7387	0.7429	0.7455	0.7477	0.7489	0.7497	0.7539	0.7613
Slough	0.6830	0.6922	0.7026	0.7131	0.7221	0.7295	0.7351	0.7389	0.7416	0.7446	0.7487	0.7530	0.7560	0.7574	0.7585	0.7618	0.7660	0.7707	0.7790	0.7870
South Gloucestershire	0.6688	0.6767	0.6856	0.6947	0.7025	0.7090	0.7144	0.7184	0.7218	0.7258	0.7309	0.7365	0.7419	0.7469	0.7514	0.7564	0.7615	0.7668	0.7736	0.7791
Dorset	0.6431	0.6508	0.6595	0.6685	0.6760	0.6823	0.6876	0.6916	0.6950	0.6992	0.7043	0.7100	0.7154	0.7205	0.7256	0.7311	0.7366	0.7417	0.7474	0.7515
Wiltshire	0.6513	0.6593	0.6683	0.6777	0.6856	0.6921	0.6973	0.7011	0.7042	0.7081	0.7133	0.7192	0.7253	0.7309	0.7359	0.7409	0.7458	0.7503	0.7561	0.7611
North Somerset	0.6361	0.6435	0.6519	0.6607	0.6680	0.6740	0.6790	0.6826	0.6858	0.6897	0.6950	0.7014	0.7077	0.7142	0.7208	0.7274	0.7335	0.7396	0.7459	0.7505
Devon	0.6376	0.6455	0.6544	0.6636	0.6713	0.6779	0.6834	0.6876	0.6913	0.6956	0.7009	0.7067	0.7122	0.7171	0.7214	0.7263	0.7312	0.7362	0.7425	0.7481
Poole	0.6440	0.6523	0.6616	0.6712	0.6794	0.6862	0.6917	0.6957	0.6990	0.7030	0.7081	0.7138	0.7190	0.7240	0.7287	0.7345	0.7410	0.7476	0.7548	0.7611
Bath and North East Somerset	0.6581	0.6660	0.6747	0.6838	0.6915	0.6981	0.7025	0.7070	0.7120	0.7168	0.7221	0.7272	0.7320	0.7367	0.7404	0.7451	0.7502	0.7557	0.7622	0.7676
Gloucestershire	0.6541	0.6620	0.6709	0.6801	0.6879	0.6944	0.6998	0.7037	0.7071	0.7112	0.7164	0.7223	0.7279	0.7333	0.7382	0.7436	0.7489	0.7539	0.7600	0.7653
Somerset	0.6388	0.6466	0.6556	0.6648	0.6726	0.6791	0.6844	0.6883	0.6917	0.6958	0.7012	0.7075	0.7136	0.7194	0.7249	0.7302	0.7344	0.7381	0.7433	0.7481
Swindon	0.6720	0.6802	0.6894	0.6989	0.7071	0.7139	0.7195	0.7236	0.7270	0.7311	0.7362	0.7420	0.7476	0.7527	0.7567	0.7612	0.7656	0.7704	0.7772	0.7837
Torbay	0.6204	0.6282	0.6370	0.6461	0.6538	0.6603	0.6658	0.6700	0.6738	0.6783	0.6838	0.6900	0.6957	0.7010	0.7059	0.7109	0.7155	0.7207	0.7275	0.7336
Bristol, City of	0.6702	0.6786	0.6881	0.6978	0.7061	0.7133	0.7193	0.7240	0.7282	0.7328	0.7383	0.7440	0.7491	0.7532	0.7561	0.7593	0.7625	0.7657	0.7715	0.7785
Bournemouth	0.6424	0.6502	0.6587	0.6676	0.6752	0.6806	0.6849	0.6894	0.6944	0.6997	0.7049	0.7097	0.7148	0.7201	0.7259	0.7318	0.7371	0.7415	0.7466	0.7519
Cornwall	0.6215	0.6296	0.6387	0.6481	0.6560	0.6626	0.6679	0.6719	0.6753	0.6794	0.6848	0.6909	0.6969	0.7025	0.7078	0.7131	0.7180	0.7228	0.7293	0.7348
Plymouth	0.6326	0.6409	0.6502	0.6599	0.6681	0.6750	0.6807	0.6849	0.6885	0.6929	0.6986	0.7050	0.7113	0.7176	0.7234	0.7292	0.7356	0.7407	0.7471	0.7520
North East England	0.6128	0.6214	0.6314	0.6418	0.6505	0.6575	0.6632	0.6674	0.6711	0.6755	0.6810	0.6868	0.6923	0.6976	0.7024	0.7080	0.7137	0.7196	0.7265	0.7324
North West England	0.6272	0.6357	0.6456	0.6559	0.6646	0.6717	0.6773	0.6813	0.6846	0.6884	0.6933	0.6988	0.7041	0.7091	0.7137	0.7190	0.7241	0.7291	0.7352	0.7407
Yorkshire and the Humber	0.6245	0.6326	0.6421	0.6520	0.6603	0.6672	0.6728	0.6769	0.6803	0.6844	0.6896	0.6953	0.7008	0.7060	0.7107	0.7160	0.7211	0.7262	0.7326	0.7385
East Midlands	0.6280	0.6358	0.6447	0.6538	0.6616	0.6680	0.6733	0.6773	0.6808	0.6852	0.6907	0.6970	0.7031	0.7087	0.7139	0.7194	0.7246	0.7296	0.7357	0.7411
West Midlands	0.6227	0.6310	0.6406	0.6506	0.6590	0.6660	0.6716	0.6757	0.6790	0.6829	0.6880	0.6935	0.6989	0.7040	0.7087	0.7140	0.7191	0.7240	0.7301	0.7357
East of England	0.6490	0.6570	0.6661	0.6753	0.6831	0.6896	0.6948	0.6985	0.7017	0.7055	0.7107	0.7165	0.7221	0.7271	0.7315	0.7362	0.7405	0.7447	0.7506	0.7565
Greater London	0.6927	0.6998	0.7079	0.7164	0.7238	0.7301	0.7353	0.7391	0.7423	0.7463	0.7515	0.7574	0.7629	0.7679	0.7724	0.7771	0.7817	0.7860	0.7923	0.7994
South East England	0.6722	0.6797	0.6882	0.6970	0.7044	0.7104	0.7152	0.7185	0.7213	0.7247	0.7295	0.7351	0.7403	0.7450	0.7491	0.7534	0.7574	0.7611	0.7666	0.7720
South West England	0.6476	0.6555	0.6645	0.6737	0.6816	0.6881	0.6935	0.6976	0.7011	0.7053	0.7105	0.7164	0.7221	0.7273	0.7320	0.7370	0.7420	0.7467	0.7527	0.7581
Southern Latin America	0.5612	0.5634	0.5673	0.5715	0.5759	0.5798	0.5848	0.5896	0.5915	0.5945	0.5994	0.6037	0.6070	0.6126	0.6176	0.6208	0.6266	0.6306	0.6341	0.6372
Argentina	0.5727	0.5740	0.5769	0.5795	0.5811	0.5822	0.5846	0.5879	0.5878	0.5891	0.5953	0.6004	0.6022	0.6076	0.6134	0.6165	0.6237	0.6291	0.6335	0.6361
Chile	0.5323	0.5358	0.5426	0.5525	0.5640	0.5746	0.5853	0.5932	0.5982	0.6018	0.6033	0.6062	0.6130	0.6193	0.6232	0.6270	0.6300	0.6311	0.6329	0.6378
Uruguay	0.5551	0.5602	0.5644	0.5651	0.5700	0.5726	0.5772	0.5820	0.5894	0.6030	0.6103	0.6150	0.6186	0.6216	0.6237	0.6256	0.6290	0.6321	0.6350	0.6384
High-income North America	0.7785	0.7927	0.8050	0.8125	0.8179	0.8227	0.8256	0.8286	0.8317	0.8333	0.8360	0.8392	0.8419	0.8449	0.8468	0.8489	0.8514	0.8522	0.8518	0.8509
Canada	0.7506	0.7624	0.7720	0.7791	0.7843	0.7893	0.7950	0.8008	0.8057	0.8099	0.8148	0.8196	0.8232	0.8264	0.8300	0.8340	0.8387	0.8427	0.8445	0.8453
United States	0.7813	0.7957	0.8084	0.8159	0.8214	0.8262	0.8288	0.8315	0.8344	0.8357	0.8381	0.8412	0.8438	0.8469	0.8485	0.8504	0.8526	0.8531	0.8524	0.8514
Greenland	0.5906	0.6200	0.6462	0.6690	0.6880	0.7028	0.7144	0.7228	0.7271	0.7291	0.7327	0.7388	0.7453	0.7499	0.7516	0.7535	0.7553	0.7554	0.7539	0.7523
Alabama	0.7345	0.7507	0.7650	0.7739	0.7808	0.7871	0.7914	0.7958	0.8004	0.8035	0.8077	0.8124	0.8164	0.8204	0.8229	0.8256	0.8284	0.8295	0.8295	0.8295
Alaska	0.7688	0.7917	0.8105	0.8216	0.8315	0.8430	0.8506	0.8550	0.8562	0.8524	0.8485	0.8445	0.8429	0.8436	0.8432	0.8459	0.8502	0.8515	0.8501	0.8468
Arizona	0.7681	0.7859	0.8014	0.8105	0.8169	0.8216	0.8233	0.8249	0.8270	0.8275	0.8295	0.8321	0.8339	0.8359	0.8360	0.8369	0.8391	0.8399	0.8394	0.8376
Arkansas	0.7218	0.7388	0.7540	0.7642	0.7720	0.7788	0.7830	0.7874	0.7923	0.7951	0.7989	0.8034	0.8072	0.8111	0.8137	0.8164	0.8192	0.8200	0.8199	0.8198
California	0.8052	0.8188	0.8305	0.8367	0.8411	0.8448	0.8464	0.8480	0.8497	0.8498	0.8509	0.8525	0.8540	0.8557	0.8557	0.8558	0.8563	0.8541	0.8495	0.8436
Colorado	0.7974	0.8130	0.8270	0.8357	0.8422	0.8476	0.8505	0.8532	0.8559	0.8569	0.8588	0.8610	0.8627	0.8645	0.8651	0.8665	0.8687	0.8697	0.8697	0.8693

Appendix Table 5A. SDI Values by location, 1970-1989																				
**Location**	**1970**	**1971**	**1972**	**1973**	**1974**	**1975**	**1976**	**1977**	**1978**	**1979**	**1980**	**1981**	**1982**	**1983**	**1984**	**1985**	**1986**	**1987**	**1988**	**1989**
Connecticut	0.8218	0.8339	0.8445	0.8508	0.8541	0.8564	0.8589	0.8617	0.8647	0.8677	0.8702	0.8729	0.8751	0.8777	0.8792	0.8809	0.8833	0.8848	0.8859	0.8869
Delaware	0.8087	0.8211	0.8321	0.8383	0.8425	0.8461	0.8477	0.8493	0.8512	0.8518	0.8540	0.8569	0.8593	0.8618	0.8629	0.8646	0.8667	0.8672	0.8663	0.8656
District of Columbia	0.8348	0.8463	0.8566	0.8615	0.8643	0.8670	0.8699	0.8729	0.8759	0.8786	0.8806	0.8827	0.8844	0.8860	0.8874	0.8880	0.8888	0.8867	0.8818	0.8756
Florida	0.7841	0.7997	0.8135	0.8224	0.8290	0.8341	0.8366	0.8388	0.8411	0.8417	0.8434	0.8455	0.8470	0.8491	0.8499	0.8512	0.8529	0.8529	0.8519	0.8514
Georgia	0.7496	0.7657	0.7801	0.7890	0.7955	0.8010	0.8045	0.8082	0.8125	0.8154	0.8198	0.8246	0.8285	0.8323	0.8345	0.8370	0.8399	0.8410	0.8409	0.8407
Hawaii	0.7807	0.7999	0.8160	0.8253	0.8322	0.8378	0.8401	0.8417	0.8429	0.8422	0.8430	0.8438	0.8451	0.8481	0.8498	0.8521	0.8547	0.8551	0.8540	0.8528
Idaho	0.7421	0.7616	0.7786	0.7883	0.7957	0.8013	0.8036	0.8055	0.8076	0.8071	0.8092	0.8125	0.8164	0.8218	0.8252	0.8289	0.8326	0.8339	0.8335	0.8324
Illinois	0.7854	0.8000	0.8127	0.8201	0.8255	0.8303	0.8329	0.8357	0.8387	0.8401	0.8428	0.8462	0.8493	0.8524	0.8540	0.8557	0.8575	0.8575	0.8564	0.8549
Indiana	0.7798	0.7929	0.8044	0.8115	0.8162	0.8202	0.8226	0.8253	0.8285	0.8302	0.8332	0.8367	0.8396	0.8426	0.8445	0.8463	0.8484	0.8487	0.8477	0.8467
Iowa	0.7740	0.7886	0.8019	0.8106	0.8163	0.8214	0.8239	0.8267	0.8303	0.8317	0.8344	0.8382	0.8415	0.8451	0.8477	0.8504	0.8532	0.8543	0.8538	0.8532
Kansas	0.7720	0.7885	0.8031	0.8121	0.8184	0.8239	0.8266	0.8292	0.8318	0.8329	0.8353	0.8387	0.8422	0.8460	0.8482	0.8507	0.8539	0.8553	0.8556	0.8552
Kentucky	0.7373	0.7528	0.7663	0.7749	0.7815	0.7873	0.7910	0.7950	0.7990	0.8017	0.8053	0.8096	0.8138	0.8181	0.8213	0.8242	0.8269	0.8281	0.8285	0.8285
Louisiana	0.7177	0.7363	0.7521	0.7614	0.7687	0.7755	0.7798	0.7842	0.7888	0.7916	0.7960	0.8013	0.8062	0.8111	0.8141	0.8174	0.8205	0.8217	0.8219	0.8218
Maine	0.7753	0.7895	0.8019	0.8094	0.8151	0.8198	0.8230	0.8260	0.8287	0.8298	0.8318	0.8340	0.8361	0.8391	0.8411	0.8439	0.8476	0.8504	0.8526	0.8546
Maryland	0.8067	0.8217	0.8349	0.8432	0.8494	0.8544	0.8577	0.8605	0.8631	0.8639	0.8652	0.8665	0.8678	0.8698	0.8708	0.8724	0.8746	0.8754	0.8754	0.8754
Massachusetts	0.8178	0.8300	0.8407	0.8456	0.8486	0.8511	0.8537	0.8564	0.8594	0.8623	0.8652	0.8681	0.8707	0.8738	0.8772	0.8796	0.8824	0.8843	0.8856	0.8864
Michigan	0.7893	0.8027	0.8147	0.8219	0.8268	0.8309	0.8335	0.8367	0.8402	0.8421	0.8448	0.8474	0.8493	0.8515	0.8527	0.8545	0.8567	0.8569	0.8558	0.8545
Minnesota	0.7785	0.7920	0.8042	0.8119	0.8174	0.8223	0.8252	0.8288	0.8328	0.8354	0.8392	0.8438	0.8480	0.8524	0.8557	0.8593	0.8633	0.8657	0.8668	0.8677
Mississippi	0.7031	0.7221	0.7390	0.7495	0.7572	0.7637	0.7679	0.7722	0.7765	0.7793	0.7836	0.7889	0.7938	0.7990	0.8026	0.8063	0.8098	0.8113	0.8114	0.8112
Missouri	0.7667	0.7816	0.7946	0.8023	0.8076	0.8124	0.8150	0.8180	0.8212	0.8230	0.8259	0.8295	0.8329	0.8367	0.8392	0.8419	0.8450	0.8464	0.8464	0.8460
Montana	0.7669	0.7834	0.7985	0.8079	0.8147	0.8204	0.8230	0.8254	0.8282	0.8282	0.8295	0.8314	0.8336	0.8367	0.8382	0.8403	0.8432	0.8445	0.8443	0.8441
Nebraska	0.7710	0.7871	0.8013	0.8101	0.8160	0.8216	0.8240	0.8263	0.8292	0.8298	0.8316	0.8349	0.8384	0.8423	0.8451	0.8484	0.8518	0.8534	0.8536	0.8533
Nevada	0.8175	0.8296	0.8399	0.8450	0.8482	0.8507	0.8513	0.8523	0.8539	0.8538	0.8549	0.8566	0.8582	0.8601	0.8600	0.8599	0.8597	0.8573	0.8537	0.8500
New Hampshire	0.7958	0.8093	0.8212	0.8284	0.8334	0.8377	0.8403	0.8433	0.8468	0.8491	0.8524	0.8561	0.8591	0.8623	0.8639	0.8658	0.8687	0.8711	0.8733	0.8755
New Jersey	0.8038	0.8172	0.8289	0.8360	0.8412	0.8457	0.8483	0.8510	0.8538	0.8554	0.8580	0.8610	0.8637	0.8667	0.8684	0.8700	0.8720	0.8726	0.8726	0.8728
New Mexico	0.7418	0.7604	0.7765	0.7855	0.7923	0.7984	0.8015	0.8045	0.8074	0.8078	0.8094	0.8115	0.8141	0.8176	0.8194	0.8221	0.8251	0.8261	0.8255	0.8241
New York	0.8072	0.8200	0.8311	0.8375	0.8420	0.8460	0.8478	0.8497	0.8516	0.8522	0.8538	0.8560	0.8581	0.8607	0.8623	0.8638	0.8658	0.8663	0.8662	0.8661
North Carolina	0.7615	0.7759	0.7889	0.7975	0.8039	0.8094	0.8131	0.8166	0.8203	0.8224	0.8255	0.8290	0.8319	0.8351	0.8373	0.8395	0.8417	0.8422	0.8418	0.8415
North Dakota	0.7393	0.7587	0.7773	0.7926	0.8023	0.8097	0.8124	0.8145	0.8177	0.8170	0.8162	0.8185	0.8218	0.8266	0.8303	0.8345	0.8393	0.8421	0.8424	0.8434
Ohio	0.7855	0.7988	0.8105	0.8173	0.8223	0.8264	0.8286	0.8312	0.8340	0.8354	0.8380	0.8410	0.8436	0.8466	0.8484	0.8504	0.8526	0.8533	0.8529	0.8523
Oklahoma	0.7537	0.7712	0.7861	0.7951	0.8018	0.8076	0.8106	0.8135	0.8162	0.8174	0.8200	0.8236	0.8280	0.8328	0.8362	0.8398	0.8430	0.8437	0.8432	0.8422
Oregon	0.7914	0.8059	0.8186	0.8261	0.8317	0.8366	0.8395	0.8425	0.8457	0.8474	0.8499	0.8525	0.8544	0.8566	0.8572	0.8579	0.8590	0.8583	0.8568	0.8555
Pennsylvania	0.7860	0.7994	0.8112	0.8184	0.8239	0.8288	0.8318	0.8349	0.8380	0.8396	0.8420	0.8448	0.8473	0.8500	0.8514	0.8529	0.8549	0.8557	0.8558	0.8560
Rhode Island	0.7982	0.8107	0.8217	0.8282	0.8322	0.8343	0.8369	0.8396	0.8425	0.8449	0.8475	0.8506	0.8533	0.8565	0.8587	0.8608	0.8631	0.8643	0.8648	0.8652
South Carolina	0.7416	0.7584	0.7732	0.7827	0.7900	0.7962	0.8002	0.8039	0.8077	0.8100	0.8135	0.8176	0.8212	0.8252	0.8278	0.8302	0.8326	0.8329	0.8320	0.8314
South Dakota	0.7336	0.7522	0.7694	0.7812	0.7886	0.7948	0.7967	0.7994	0.8028	0.8041	0.8064	0.8103	0.8141	0.8185	0.8219	0.8254	0.8295	0.8318	0.8322	0.8320
Tennessee	0.7514	0.7660	0.7792	0.7877	0.7941	0.7996	0.8033	0.8071	0.8113	0.8140	0.8177	0.8218	0.8252	0.8288	0.8311	0.8333	0.8355	0.8363	0.8359	0.8354
Texas	0.7439	0.7619	0.7774	0.7866	0.7935	0.7999	0.8035	0.8069	0.8104	0.8121	0.8151	0.8191	0.8228	0.8266	0.8286	0.8310	0.8338	0.8344	0.8337	0.8321
Utah	0.7124	0.7361	0.7556	0.7643	0.7697	0.7743	0.7746	0.7753	0.7768	0.7758	0.7793	0.7858	0.7940	0.8031	0.8089	0.8142	0.8189	0.8203	0.8198	0.8194
Vermont	0.7863	0.8013	0.8144	0.8220	0.8273	0.8317	0.8342	0.8365	0.8392	0.8404	0.8427	0.8455	0.8481	0.8510	0.8529	0.8554	0.8588	0.8615	0.8635	0.8657
Virginia	0.7841	0.7991	0.8123	0.8207	0.8272	0.8328	0.8364	0.8400	0.8437	0.8458	0.8488	0.8520	0.8547	0.8577	0.8596	0.8617	0.8644	0.8657	0.8663	0.8666
Washington	0.8026	0.8164	0.8282	0.8349	0.8400	0.8447	0.8470	0.8495	0.8525	0.8540	0.8566	0.8595	0.8619	0.8646	0.8656	0.8669	0.8689	0.8691	0.8681	0.8668
West Virginia	0.7430	0.7579	0.7709	0.7787	0.7846	0.7901	0.7939	0.7979	0.8021	0.8049	0.8090	0.8134	0.8176	0.8217	0.8243	0.8270	0.8296	0.8306	0.8308	0.8305
Wisconsin	0.7760	0.7908	0.8038	0.8116	0.8173	0.8223	0.8251	0.8284	0.8317	0.8335	0.8364	0.8395	0.8422	0.8453	0.8473	0.8497	0.8530	0.8551	0.8560	0.8567
Wyoming	0.7474	0.7685	0.7864	0.7968	0.8049	0.8118	0.8150	0.8182	0.8215	0.8221	0.8242	0.8270	0.8305	0.8347	0.8372	0.8416	0.8474	0.8505	0.8511	0.8506
Latin America and Caribbean	0.3769	0.3846	0.3938	0.4031	0.4123	0.4214	0.4305	0.4395	0.4483	0.4568	0.4665	0.4767	0.4858	0.4944	0.5027	0.5110	0.5188	0.5259	0.5331	0.5398
Caribbean	0.4293	0.4336	0.4458	0.4600	0.4739	0.4846	0.4940	0.5052	0.5151	0.5240	0.5323	0.5382	0.5435	0.5509	0.5566	0.5627	0.5688	0.5727	0.5779	0.5830
Antigua and Barbuda	0.5185	0.5318	0.5447	0.5567	0.5670	0.5747	0.5807	0.5874	0.5944	0.6028	0.6111	0.6189	0.6266	0.6336	0.6399	0.6468	0.6544	0.6622	0.6701	0.6776
The Bahamas	0.5870	0.5976	0.6072	0.6159	0.6211	0.6243	0.6278	0.6328	0.6399	0.6501	0.6591	0.6663	0.6735	0.6810	0.6892	0.6972	0.7042	0.7094	0.7133	0.7165

Appendix Table 5A. SDI Values by location, 1970-1989																				
**Location**	**1970**	**1971**	**1972**	**1973**	**1974**	**1975**	**1976**	**1977**	**1978**	**1979**	**1980**	**1981**	**1982**	**1983**	**1984**	**1985**	**1986**	**1987**	**1988**	**1989**
Barbados	0.5739	0.5862	0.5978	0.6090	0.6191	0.6286	0.6361	0.6431	0.6494	0.6557	0.6618	0.6675	0.6728	0.6785	0.6849	0.6914	0.6986	0.7058	0.7128	0.7199
Belize	0.2780	0.2872	0.2951	0.3025	0.3110	0.3180	0.3235	0.3290	0.3337	0.3386	0.3437	0.3480	0.3531	0.3601	0.3689	0.3791	0.3907	0.4042	0.4187	0.4348
Cuba	0.4887	0.4886	0.5075	0.5302	0.5518	0.5664	0.5791	0.5969	0.6111	0.6219	0.6308	0.6341	0.6365	0.6451	0.6501	0.6578	0.6664	0.6683	0.6734	0.6788
Dominica	0.3727	0.3919	0.4138	0.4391	0.4650	0.4870	0.5032	0.5149	0.5239	0.5267	0.5290	0.5341	0.5408	0.5484	0.5560	0.5630	0.5704	0.5775	0.5844	0.5901
Dominican Republic	0.3311	0.3354	0.3427	0.3536	0.3665	0.3794	0.3912	0.4020	0.4120	0.4227	0.4351	0.4485	0.4626	0.4770	0.4896	0.4991	0.5065	0.5131	0.5189	0.5237
Grenada	0.3539	0.3629	0.3723	0.3820	0.3916	0.4011	0.4106	0.4194	0.4278	0.4355	0.4418	0.4478	0.4533	0.4583	0.4631	0.4683	0.4730	0.4790	0.4853	0.4924
Guyana	0.4284	0.4359	0.4424	0.4487	0.4555	0.4630	0.4697	0.4757	0.4817	0.4883	0.4959	0.5032	0.5088	0.5136	0.5165	0.5178	0.5187	0.5193	0.5189	0.5180
Haiti	0.2107	0.2144	0.2183	0.2228	0.2276	0.2323	0.2377	0.2425	0.2471	0.2520	0.2570	0.2608	0.2640	0.2675	0.2711	0.2748	0.2787	0.2838	0.2904	0.2968
Jamaica	0.4298	0.4435	0.4602	0.4772	0.4935	0.5088	0.5219	0.5334	0.5432	0.5508	0.5568	0.5626	0.5681	0.5734	0.5776	0.5801	0.5832	0.5870	0.5905	0.5945
Saint Lucia	0.2506	0.2750	0.2984	0.3209	0.3420	0.3621	0.3833	0.4037	0.4231	0.4398	0.4529	0.4635	0.4730	0.4825	0.4924	0.5024	0.5128	0.5230	0.5339	0.5445
Saint Vincent and the Grenadines	0.2981	0.3095	0.3255	0.3402	0.3527	0.3637	0.3758	0.3867	0.3976	0.4077	0.4175	0.4277	0.4382	0.4490	0.4601	0.4708	0.4803	0.4890	0.4988	0.5076
Suriname	0.4009	0.4125	0.4259	0.4392	0.4512	0.4630	0.4740	0.4853	0.4958	0.5039	0.5100	0.5152	0.5195	0.5236	0.5283	0.5346	0.5407	0.5463	0.5532	0.5603
Trinidad and Tobago	0.5176	0.5249	0.5333	0.5436	0.5555	0.5660	0.5753	0.5819	0.5860	0.5891	0.5922	0.5954	0.5993	0.6032	0.6082	0.6153	0.6240	0.6334	0.6419	0.6488
Bermuda	0.5775	0.5861	0.6024	0.6182	0.6302	0.6400	0.6502	0.6603	0.6681	0.6742	0.6805	0.6868	0.6925	0.6978	0.7026	0.7075	0.7131	0.7186	0.7239	0.7288
Puerto Rico	0.6130	0.6237	0.6354	0.6467	0.6554	0.6611	0.6649	0.6701	0.6781	0.6884	0.6991	0.7098	0.7190	0.7268	0.7335	0.7386	0.7434	0.7481	0.7534	0.7596
Virgin Islands, U.S.	0.5975	0.6092	0.6208	0.6326	0.6446	0.6572	0.6706	0.6843	0.6981	0.7114	0.7232	0.7320	0.7376	0.7429	0.7493	0.7554	0.7601	0.7637	0.7673	0.7715
Andean Latin America	0.3793	0.3858	0.3922	0.3987	0.4057	0.4128	0.4200	0.4273	0.4346	0.4420	0.4496	0.4574	0.4654	0.4729	0.4806	0.4881	0.4959	0.5037	0.5113	0.5178
Bolivia	0.2904	0.2960	0.3018	0.3076	0.3137	0.3199	0.3262	0.3329	0.3396	0.3466	0.3537	0.3610	0.3684	0.3756	0.3829	0.3904	0.3975	0.4046	0.4115	0.4183
Ecuador	0.3768	0.3832	0.3895	0.3963	0.4037	0.4110	0.4185	0.4263	0.4341	0.4420	0.4501	0.4583	0.4667	0.4752	0.4839	0.4927	0.5015	0.5091	0.5163	0.5232
Peru	0.4087	0.4152	0.4217	0.4282	0.4351	0.4422	0.4494	0.4565	0.4635	0.4706	0.4778	0.4853	0.4929	0.4998	0.5068	0.5137	0.5209	0.5289	0.5366	0.5426
Central Latin America	0.3732	0.3818	0.3913	0.4001	0.4084	0.4174	0.4267	0.4358	0.4452	0.4554	0.4658	0.4758	0.4849	0.4935	0.5021	0.5102	0.5175	0.5246	0.5315	0.5374
Colombia	0.3931	0.4016	0.4109	0.4206	0.4303	0.4398	0.4491	0.4583	0.4675	0.4764	0.4849	0.4927	0.4998	0.5063	0.5126	0.5190	0.5256	0.5319	0.5382	0.5443
Costa Rica	0.4440	0.4586	0.4718	0.4830	0.4913	0.4975	0.5028	0.5082	0.5138	0.5201	0.5266	0.5319	0.5352	0.5370	0.5387	0.5414	0.5464	0.5519	0.5573	0.5634
El Salvador	0.3037	0.3093	0.3150	0.3208	0.3267	0.3331	0.3407	0.3501	0.3609	0.3721	0.3826	0.3922	0.4007	0.4082	0.4142	0.4192	0.4239	0.4286	0.4336	0.4388
Guatemala	0.2594	0.2659	0.2726	0.2794	0.2861	0.2925	0.2991	0.3060	0.3127	0.3191	0.3252	0.3305	0.3347	0.3384	0.3418	0.3450	0.3482	0.3515	0.3554	0.3598
Honduras	0.1962	0.2029	0.2113	0.2203	0.2290	0.2382	0.2489	0.2607	0.2727	0.2841	0.2946	0.3043	0.3131	0.3213	0.3294	0.3372	0.3448	0.3530	0.3621	0.3716
Mexico	0.3569	0.3656	0.3749	0.3845	0.3942	0.4040	0.4138	0.4236	0.4342	0.4456	0.4576	0.4697	0.4808	0.4910	0.5012	0.5112	0.5205	0.5296	0.5382	0.5467
Nicaragua	0.3517	0.3581	0.3643	0.3710	0.3782	0.3847	0.3921	0.3997	0.4046	0.4065	0.4092	0.4121	0.4147	0.4178	0.4208	0.4239	0.4269	0.4300	0.4313	0.4327
Panama	0.4375	0.4499	0.4633	0.4764	0.4879	0.4980	0.5072	0.5156	0.5243	0.5329	0.5421	0.5516	0.5605	0.5677	0.5742	0.5809	0.5877	0.5936	0.5971	0.6000
Venezuela	0.4524	0.4616	0.4746	0.4794	0.4805	0.4883	0.4977	0.5046	0.5113	0.5219	0.5328	0.5402	0.5471	0.5571	0.5684	0.5774	0.5827	0.5877	0.5936	0.5917
Aguascalientes	0.3860	0.3953	0.4050	0.4151	0.4253	0.4355	0.4456	0.4557	0.4664	0.4778	0.4898	0.5016	0.5121	0.5212	0.5298	0.5384	0.5483	0.5582	0.5670	0.5764
Baja California	0.4303	0.4388	0.4476	0.4566	0.4656	0.4744	0.4831	0.4918	0.5013	0.5118	0.5234	0.5359	0.5479	0.5597	0.5722	0.5853	0.5974	0.6079	0.6167	0.6238
Baja California Sur	0.4474	0.4554	0.4637	0.4721	0.4804	0.4886	0.4969	0.5051	0.5141	0.5241	0.5353	0.5473	0.5591	0.5711	0.5836	0.5960	0.6050	0.6152	0.6271	0.6353
Campeche	0.3358	0.3446	0.3539	0.3636	0.3735	0.3834	0.3933	0.4032	0.4139	0.4253	0.4372	0.4491	0.4594	0.4683	0.4767	0.4849	0.4938	0.5028	0.5110	0.5203
Coahuila	0.3845	0.3948	0.4057	0.4170	0.4282	0.4395	0.4506	0.4618	0.4736	0.4860	0.4989	0.5117	0.5230	0.5332	0.5429	0.5522	0.5621	0.5723	0.5822	0.5933
Colima	0.3620	0.3719	0.3823	0.3930	0.4038	0.4145	0.4252	0.4360	0.4474	0.4594	0.4720	0.4845	0.4959	0.5065	0.5169	0.5277	0.5400	0.5527	0.5652	0.5785
Chiapas	0.1858	0.1950	0.2049	0.2153	0.2263	0.2374	0.2488	0.2604	0.2729	0.2860	0.2995	0.3125	0.3234	0.3317	0.3383	0.3425	0.3449	0.3453	0.3422	0.3393
Chihuahua	0.3823	0.3921	0.4023	0.4128	0.4234	0.4340	0.4445	0.4550	0.4662	0.4780	0.4903	0.5026	0.5133	0.5226	0.5314	0.5400	0.5491	0.5586	0.5677	0.5776
Distrito Federal	0.4739	0.4845	0.4957	0.5073	0.5190	0.5307	0.5423	0.5539	0.5661	0.5788	0.5920	0.6047	0.6154	0.6238	0.6306	0.6353	0.6383	0.6421	0.6489	0.6597
Durango	0.3593	0.3679	0.3770	0.3866	0.3962	0.4060	0.4157	0.4256	0.4360	0.4472	0.4588	0.4704	0.4801	0.4881	0.4951	0.5018	0.5091	0.5163	0.5222	0.5288
Guanajuato	0.2796	0.2887	0.2982	0.3081	0.3182	0.3284	0.3386	0.3489	0.3599	0.3717	0.3840	0.3963	0.4073	0.4174	0.4272	0.4370	0.4484	0.4607	0.4723	0.4846
Guerrero	0.2548	0.2630	0.2720	0.2813	0.2910	0.3007	0.3106	0.3206	0.3315	0.3431	0.3552	0.3672	0.3776	0.3866	0.3950	0.4036	0.4131	0.4233	0.4312	0.4371
Hidalgo	0.2839	0.2923	0.3012	0.3106	0.3201	0.3297	0.3394	0.3492	0.3598	0.3712	0.3832	0.3954	0.4064	0.4164	0.4263	0.4368	0.4487	0.4603	0.4701	0.4803
Jalisco	0.3575	0.3668	0.3767	0.3869	0.3972	0.4075	0.4178	0.4281	0.4391	0.4508	0.4630	0.4752	0.4861	0.4959	0.5052	0.5144	0.5245	0.5347	0.5439	0.5540
Mexico	0.3723	0.3791	0.3861	0.3929	0.3994	0.4057	0.4118	0.4182	0.4257	0.4352	0.4475	0.4628	0.4808	0.5024	0.5279	0.5539	0.5707	0.5820	0.5918	0.5952
Michoacan de Ocampo	0.2650	0.2739	0.2832	0.2930	0.3029	0.3129	0.3229	0.3331	0.3440	0.3555	0.3677	0.3799	0.3907	0.4005	0.4100	0.4196	0.4311	0.4430	0.4533	0.4642
Morelos	0.3691	0.3789	0.3893	0.4001	0.4109	0.4217	0.4325	0.4434	0.4548	0.4669	0.4794	0.4919	0.5031	0.5133	0.5230	0.5327	0.5432	0.5538	0.5641	0.5756
Nayarit	0.3101	0.3204	0.3312	0.3424	0.3539	0.3655	0.3772	0.3890	0.4015	0.4147	0.4284	0.4420	0.4538	0.4638	0.4731	0.4817	0.4910	0.5011	0.5110	0.5221
Nuevo Leon	0.4770	0.4867	0.4968	0.5071	0.5175	0.5279	0.5382	0.5485	0.5593	0.5706	0.5822	0.5936	0.6035	0.6117	0.6192	0.6256	0.6318	0.6380	0.6443	0.6517

Appendix Table 5A. SDI Values by location, 1970-1989

Location	1970	1971	1972	1973	1974	1975	1976	1977	1978	1979	1980	1981	1982	1983	1984	1985	1986	1987	1988	1989
Oaxaca	0.2667	0.2738	0.2814	0.2894	0.2975	0.3057	0.3139	0.3222	0.3312	0.3409	0.3512	0.3618	0.3712	0.3796	0.3877	0.3963	0.4063	0.4169	0.4265	0.4365
Puebla	0.3050	0.3136	0.3226	0.3320	0.3416	0.3513	0.3610	0.3707	0.3812	0.3923	0.4041	0.4158	0.4264	0.4358	0.4449	0.4541	0.4646	0.4752	0.4847	0.4945
Queretaro	0.3045	0.3171	0.3306	0.3446	0.3591	0.3737	0.3884	0.4028	0.4174	0.4317	0.4451	0.4564	0.4632	0.4647	0.4607	0.4527	0.4520	0.4698	0.4947	0.5148
Quintana Roo	0.3765	0.3864	0.3969	0.4078	0.4188	0.4300	0.4412	0.4525	0.4645	0.4772	0.4903	0.5031	0.5143	0.5237	0.5322	0.5397	0.5467	0.5533	0.5594	0.5669
San Luis Potosi	0.2525	0.2631	0.2743	0.2860	0.2979	0.3099	0.3219	0.3341	0.3471	0.3609	0.3753	0.3897	0.4026	0.4141	0.4251	0.4362	0.4485	0.4618	0.4743	0.4873
Sinaloa	0.3853	0.3944	0.4040	0.4141	0.4244	0.4348	0.4452	0.4557	0.4669	0.4788	0.4912	0.5034	0.5140	0.5231	0.5314	0.5393	0.5475	0.5561	0.5642	0.5726
Sonora	0.4175	0.4274	0.4379	0.4486	0.4595	0.4704	0.4813	0.4922	0.5037	0.5158	0.5283	0.5408	0.5515	0.5606	0.5689	0.5766	0.5844	0.5917	0.5983	0.6059
Tabasco	0.3153	0.3240	0.3334	0.3431	0.3531	0.3633	0.3737	0.3842	0.3954	0.4074	0.4198	0.4320	0.4427	0.4520	0.4607	0.4689	0.4780	0.4875	0.4963	0.5058
Tamaulipas	0.4030	0.4122	0.4218	0.4317	0.4417	0.4515	0.4613	0.4712	0.4816	0.4927	0.5042	0.5158	0.5260	0.5352	0.5440	0.5526	0.5621	0.5721	0.5822	0.5933
Tlaxcala	0.2698	0.2784	0.2871	0.2959	0.3045	0.3130	0.3213	0.3293	0.3381	0.3479	0.3591	0.3715	0.3839	0.3974	0.4132	0.4324	0.4549	0.4789	0.4992	0.5135
Veracruz de Ignacio de la Llave	0.3069	0.3155	0.3247	0.3342	0.3439	0.3538	0.3637	0.3737	0.3845	0.3960	0.4080	0.4200	0.4306	0.4400	0.4490	0.4579	0.4673	0.4765	0.4845	0.4927
Yucatan	0.3786	0.3862	0.3943	0.4027	0.4112	0.4198	0.4283	0.4369	0.4460	0.4558	0.4662	0.4766	0.4857	0.4935	0.5007	0.5075	0.5148	0.5219	0.5284	0.5361
Zacatecas	0.2732	0.2826	0.2924	0.3026	0.3131	0.3236	0.3341	0.3447	0.3560	0.3680	0.3807	0.3934	0.4048	0.4150	0.4249	0.4347	0.4460	0.4586	0.4710	0.4845
Tropical Latin America	0.3594	0.3677	0.3766	0.3863	0.3958	0.4051	0.4145	0.4234	0.4318	0.4391	0.4490	0.4607	0.4708	0.4800	0.4890	0.4983	0.5071	0.5154	0.5236	0.5316
Brazil	0.3591	0.3674	0.3764	0.3860	0.3956	0.4049	0.4143	0.4232	0.4316	0.4390	0.4489	0.4607	0.4710	0.4803	0.4895	0.4989	0.5079	0.5163	0.5246	0.5327
Paraguay	0.3577	0.3650	0.3725	0.3804	0.3884	0.3964	0.4047	0.4136	0.4225	0.4302	0.4371	0.4436	0.4480	0.4509	0.4536	0.4562	0.4586	0.4613	0.4647	0.4685
Acre	0.2521	0.2591	0.2668	0.2751	0.2834	0.2915	0.3000	0.3080	0.3157	0.3220	0.3321	0.3457	0.3577	0.3689	0.3802	0.3918	0.4040	0.4161	0.4277	0.4388
Alagoas	0.2355	0.2425	0.2503	0.2588	0.2673	0.2755	0.2838	0.2916	0.2991	0.3057	0.3142	0.3245	0.3336	0.3418	0.3500	0.3580	0.3676	0.3780	0.3877	0.3971
Amazonas	0.2801	0.2882	0.2969	0.3063	0.3155	0.3244	0.3334	0.3419	0.3499	0.3564	0.3670	0.3815	0.3939	0.4053	0.4164	0.4271	0.4389	0.4517	0.4638	0.4755
Amapa	0.2510	0.2600	0.2696	0.2798	0.2899	0.2998	0.3102	0.3202	0.3299	0.3397	0.3518	0.3660	0.3796	0.3926	0.4055	0.4186	0.4320	0.4448	0.4567	0.4678
Bahia	0.2737	0.2811	0.2892	0.2980	0.3068	0.3154	0.3239	0.3320	0.3399	0.3472	0.3556	0.3649	0.3734	0.3812	0.3891	0.3968	0.4065	0.4174	0.4276	0.4375
Ceara	0.2632	0.2708	0.2791	0.2882	0.2973	0.3062	0.3147	0.3228	0.3306	0.3382	0.3462	0.3546	0.3623	0.3692	0.3762	0.3822	0.3924	0.4057	0.4174	0.4285
Distrito Federal	0.4987	0.5077	0.5174	0.5276	0.5376	0.5472	0.5580	0.5682	0.5778	0.5866	0.5969	0.6075	0.6168	0.6254	0.6340	0.6433	0.6520	0.6591	0.6664	0.6735
Espirito Santo	0.3652	0.3738	0.3830	0.3930	0.4028	0.4125	0.4224	0.4318	0.4409	0.4490	0.4589	0.4703	0.4805	0.4898	0.4991	0.5086	0.5181	0.5273	0.5362	0.5448
Goias	0.2988	0.3077	0.3174	0.3277	0.3380	0.3481	0.3582	0.3678	0.3767	0.3839	0.3955	0.4115	0.4250	0.4374	0.4496	0.4619	0.4730	0.4827	0.4925	0.5021
Maranhao	0.2326	0.2391	0.2464	0.2543	0.2623	0.2702	0.2783	0.2862	0.2939	0.3019	0.3087	0.3140	0.3191	0.3237	0.3285	0.3332	0.3414	0.3520	0.3615	0.3707
Minas Gerais	0.3632	0.3713	0.3800	0.3895	0.3988	0.4079	0.4170	0.4256	0.4337	0.4406	0.4503	0.4625	0.4731	0.4829	0.4926	0.5029	0.5115	0.5182	0.5253	0.5323
Mato Grosso do Sul	0.3098	0.3182	0.3273	0.3370	0.3467	0.3561	0.3657	0.3748	0.3833	0.3898	0.4014	0.4178	0.4318	0.4446	0.4572	0.4706	0.4812	0.4889	0.4975	0.5061
Mato Grosso	0.3274	0.3356	0.3446	0.3542	0.3638	0.3732	0.3828	0.3920	0.4007	0.4082	0.4189	0.4326	0.4448	0.4561	0.4675	0.4796	0.4898	0.4980	0.5068	0.5155
Para	0.2846	0.2919	0.2999	0.3086	0.3172	0.3254	0.3338	0.3417	0.3491	0.3553	0.3642	0.3752	0.3847	0.3933	0.4019	0.4109	0.4196	0.4277	0.4357	0.4436
Paraiba	0.2412	0.2490	0.2576	0.2668	0.2760	0.2850	0.2937	0.3018	0.3095	0.3160	0.3252	0.3370	0.3471	0.3561	0.3648	0.3721	0.3838	0.3989	0.4120	0.4243
Parana	0.3488	0.3574	0.3668	0.3768	0.3868	0.3966	0.4064	0.4158	0.4247	0.4319	0.4431	0.4581	0.4709	0.4827	0.4944	0.5067	0.5168	0.5244	0.5326	0.5408
Pernambuco	0.2766	0.2848	0.2938	0.3033	0.3128	0.3221	0.3312	0.3398	0.3479	0.3547	0.3644	0.3765	0.3869	0.3961	0.4052	0.4137	0.4245	0.4367	0.4477	0.4582
Piaui	0.2432	0.2500	0.2575	0.2658	0.2740	0.2822	0.2903	0.2981	0.3056	0.3127	0.3206	0.3291	0.3368	0.3438	0.3509	0.3577	0.3670	0.3781	0.3882	0.3981
Rio de Janeiro	0.4394	0.4481	0.4576	0.4676	0.4774	0.4868	0.4965	0.5055	0.5137	0.5201	0.5308	0.5449	0.5566	0.5669	0.5768	0.5870	0.5954	0.6018	0.6084	0.6148
Rio Grande do Norte	0.2679	0.2761	0.2852	0.2949	0.3046	0.3142	0.3233	0.3319	0.3401	0.3472	0.3568	0.3693	0.3802	0.3901	0.3998	0.4094	0.4200	0.4311	0.4416	0.4516
Rondonia	0.2719	0.2797	0.2883	0.2975	0.3066	0.3155	0.3246	0.3332	0.3413	0.3476	0.3583	0.3734	0.3863	0.3982	0.4100	0.4219	0.4341	0.4458	0.4570	0.4675
Roraima	0.3131	0.3209	0.3294	0.3386	0.3478	0.3567	0.3658	0.3746	0.3832	0.3919	0.4012	0.4113	0.4210	0.4304	0.4401	0.4504	0.4614	0.4723	0.4829	0.4932
Rio Grande do Sul	0.4194	0.4275	0.4363	0.4457	0.4550	0.4639	0.4731	0.4817	0.4898	0.4969	0.5058	0.5162	0.5251	0.5332	0.5412	0.5495	0.5574	0.5648	0.5720	0.5790
Santa Catarina	0.3878	0.3961	0.4052	0.4149	0.4245	0.4337	0.4431	0.4520	0.4603	0.4676	0.4773	0.4890	0.4990	0.5082	0.5172	0.5264	0.5356	0.5445	0.5531	0.5614
Sergipe	0.2485	0.2566	0.2654	0.2749	0.2843	0.2936	0.3027	0.3114	0.3195	0.3263	0.3364	0.3500	0.3614	0.3717	0.3817	0.3906	0.4027	0.4171	0.4299	0.4419
Sao Paulo	0.4389	0.4472	0.4562	0.4658	0.4753	0.4845	0.4942	0.5033	0.5120	0.5194	0.5295	0.5415	0.5519	0.5613	0.5707	0.5807	0.5891	0.5956	0.6024	0.6092
Tocantins	0.2840	0.2924	0.3016	0.3117	0.3217	0.3316	0.3416	0.3513	0.3608	0.3701	0.3797	0.3891	0.3981	0.4064	0.4150	0.4239	0.4335	0.4430	0.4521	0.4609
North Africa and Middle East	0.2863	0.2948	0.3040	0.3136	0.3238	0.3336	0.3434	0.3531	0.3625	0.3722	0.3821	0.3918	0.4009	0.4097	0.4185	0.4272	0.4362	0.4455	0.4550	0.4648
North Africa and Middle East	0.2863	0.2948	0.3040	0.3136	0.3238	0.3336	0.3434	0.3531	0.3625	0.3722	0.3821	0.3918	0.4009	0.4097	0.4185	0.4272	0.4362	0.4455	0.4550	0.4648
Algeria	0.2540	0.2620	0.2708	0.2799	0.2899	0.3000	0.3106	0.3214	0.3326	0.3443	0.3559	0.3674	0.3787	0.3899	0.4009	0.4120	0.4227	0.4333	0.4433	0.4532
Bahrain	0.3458	0.3587	0.3721	0.3863	0.4013	0.4157	0.4323	0.4490	0.4654	0.4817	0.4979	0.5131	0.5264	0.5359	0.5399	0.5418	0.5496	0.5594	0.5648	0.5713
Egypt	0.2531	0.2610	0.2693	0.2780	0.2869	0.2960	0.3057	0.3157	0.3260	0.3363	0.3469	0.3577	0.3686	0.3797	0.3910	0.4022	0.4134	0.4245	0.4352	0.4462
Iran	0.2593	0.2734	0.2881	0.3031	0.3182	0.3307	0.3413	0.3512	0.3604	0.3699	0.3790	0.3874	0.3954	0.4037	0.4119	0.4215	0.4335	0.4488	0.4665	0.4870

Appendix Table 5A. SDI Values by location, 1970-1989																				
**Location**	**1970**	**1971**	**1972**	**1973**	**1974**	**1975**	**1976**	**1977**	**1978**	**1979**	**1980**	**1981**	**1982**	**1983**	**1984**	**1985**	**1986**	**1987**	**1988**	**1989**
Iraq	0.1971	0.2042	0.2110	0.2190	0.2276	0.2364	0.2465	0.2570	0.2676	0.2783	0.2894	0.2995	0.3097	0.3204	0.3312	0.3413	0.3510	0.3611	0.3715	0.3809
Jordan	0.1112	0.1126	0.1139	0.1152	0.1164	0.1175	0.1189	0.1435	0.1919	0.2300	0.2637	0.2940	0.3219	0.3465	0.3685	0.3890	0.4088	0.4282	0.4475	0.4660
Kuwait	0.2660	0.2995	0.3280	0.3498	0.3660	0.3790	0.3938	0.4117	0.4297	0.4453	0.4603	0.4764	0.4940	0.5151	0.5404	0.5663	0.5898	0.6108	0.6275	0.6421
Lebanon	0.3570	0.3725	0.3885	0.4047	0.4217	0.4385	0.4525	0.4674	0.4818	0.4962	0.5104	0.5251	0.5363	0.5475	0.5605	0.5750	0.5880	0.6019	0.6131	0.6206
Libya	0.4954	0.5041	0.5124	0.5204	0.5279	0.5344	0.5409	0.5471	0.5528	0.5586	0.5648	0.5698	0.5747	0.5794	0.5835	0.5877	0.5914	0.5947	0.5979	0.6013
Morocco	0.2128	0.2188	0.2251	0.2316	0.2386	0.2459	0.2538	0.2619	0.2700	0.2784	0.2868	0.2949	0.3034	0.3119	0.3206	0.3295	0.3391	0.3488	0.3595	0.3703
Palestine	0.1709	0.1875	0.2044	0.2206	0.2374	0.2536	0.2696	0.2851	0.3002	0.3141	0.3281	0.3406	0.3512	0.3602	0.3686	0.3762	0.3837	0.3905	0.3970	0.4036
Oman	0.0846	0.0873	0.0897	0.0917	0.0938	0.0960	0.0984	0.1007	0.1028	0.1047	0.1066	0.1087	0.1108	0.1128	0.1147	0.1355	0.1561	0.1755	0.1942	0.2135
Qatar	0.3437	0.3586	0.3744	0.3911	0.4085	0.4262	0.4448	0.4637	0.4825	0.5014	0.5203	0.5398	0.5583	0.5747	0.5873	0.5958	0.6007	0.6047	0.6091	0.6148
Saudi Arabia	0.1333	0.1364	0.1396	0.1424	0.1468	0.1538	0.1630	0.1742	0.1863	0.1981	0.2104	0.2253	0.2423	0.2602	0.2769	0.2926	0.3077	0.3227	0.3380	0.3531
Syria	0.1904	0.1966	0.2044	0.2124	0.2210	0.2305	0.2404	0.2501	0.2600	0.2699	0.2800	0.2904	0.3007	0.3110	0.3204	0.3297	0.3388	0.3476	0.3570	0.3661
Tunisia	0.2528	0.2609	0.2699	0.2789	0.2884	0.2985	0.3093	0.3199	0.3306	0.3415	0.3529	0.3644	0.3760	0.3878	0.3999	0.4123	0.4248	0.4373	0.4498	0.4625
Turkey	0.3908	0.3983	0.4063	0.4146	0.4232	0.4321	0.4416	0.4510	0.4605	0.4700	0.4791	0.4887	0.4981	0.5076	0.5171	0.5267	0.5365	0.5468	0.5569	0.5664
United Arab Emirates	0.1370	0.1392	0.1416	0.1440	0.1463	0.1546	0.2079	0.2481	0.2826	0.3125	0.3395	0.3647	0.3893	0.4129	0.4356	0.4581	0.4797	0.5007	0.5208	0.5394
Yemen	0.1188	0.1207	0.1234	0.1274	0.1313	0.1348	0.1386	0.1424	0.1467	0.1513	0.1553	0.1592	0.1627	0.1656	0.1675	0.1679	0.1676	0.1673	0.1682	0.1702
Afghanistan	0.1155	0.1174	0.1189	0.1206	0.1224	0.1241	0.1261	0.1278	0.1298	0.1318	0.1338	0.1361	0.1387	0.1416	0.1445	0.1474	0.1504	0.1526	0.1544	0.1561
Sudan	0.1896	0.1937	0.1976	0.2013	0.2056	0.2106	0.2165	0.2230	0.2290	0.2344	0.2397	0.2455	0.2518	0.2578	0.2633	0.2682	0.2736	0.2792	0.2851	0.2903
Ha'il	0.1296	0.1326	0.1356	0.1382	0.1424	0.1494	0.1587	0.1699	0.1816	0.1930	0.2051	0.2198	0.2356	0.2517	0.2674	0.2826	0.2971	0.3116	0.3263	0.3407
Qassim	0.0923	0.0942	0.0960	0.0979	0.0998	0.1017	0.1038	0.1060	0.1158	0.1336	0.1504	0.1694	0.1901	0.2110	0.2312	0.2505	0.2691	0.2877	0.3061	0.3242
Riyadh	0.1007	0.1027	0.1046	0.1066	0.1086	0.1105	0.1193	0.1368	0.1541	0.1698	0.1854	0.2033	0.2232	0.2436	0.2635	0.2828	0.3009	0.3191	0.3371	0.3533
Tabuk	0.1127	0.1145	0.1158	0.1159	0.1179	0.1259	0.1374	0.1516	0.1664	0.1801	0.1940	0.2103	0.2288	0.2480	0.2668	0.2850	0.3009	0.3162	0.3316	0.3467
Madinah	0.1196	0.1226	0.1256	0.1281	0.1323	0.1395	0.1494	0.1614	0.1742	0.1866	0.1994	0.2149	0.2324	0.2507	0.2685	0.2847	0.3002	0.3157	0.3314	0.3468
Makkah	0.1486	0.1526	0.1568	0.1609	0.1664	0.1741	0.1838	0.1950	0.2067	0.2184	0.2308	0.2456	0.2614	0.2774	0.2930	0.3082	0.3227	0.3372	0.3520	0.3667
Bahah	0.1471	0.1508	0.1548	0.1588	0.1639	0.1706	0.1789	0.1885	0.1984	0.2084	0.2190	0.2320	0.2458	0.2597	0.2734	0.2867	0.2994	0.3121	0.3251	0.3380
Northern Borders	0.1751	0.1800	0.1853	0.1906	0.1973	0.2058	0.2160	0.2275	0.2394	0.2513	0.2638	0.2782	0.2933	0.3084	0.3232	0.3374	0.3508	0.3642	0.3780	0.3917
Jawf	0.1414	0.1463	0.1514	0.1559	0.1615	0.1703	0.1815	0.1946	0.2085	0.2221	0.2362	0.2529	0.2716	0.2911	0.3108	0.3301	0.3472	0.3639	0.3808	0.3976
Jizan	0.1434	0.1467	0.1502	0.1536	0.1580	0.1634	0.1701	0.1777	0.1854	0.1932	0.2017	0.2121	0.2230	0.2339	0.2446	0.2549	0.2650	0.2751	0.2855	0.2957
'Asir	0.1062	0.1072	0.1076	0.1071	0.1083	0.1137	0.1221	0.1330	0.1445	0.1555	0.1669	0.1812	0.1971	0.2130	0.2286	0.2434	0.2579	0.2722	0.2865	0.3005
Najran	0.1324	0.1345	0.1366	0.1383	0.1410	0.1459	0.1526	0.1609	0.1696	0.1782	0.1875	0.1990	0.2116	0.2244	0.2370	0.2490	0.2610	0.2729	0.2850	0.2969
Eastern Province	0.1240	0.1276	0.1311	0.1343	0.1384	0.1463	0.1567	0.1693	0.1826	0.1955	0.2088	0.2246	0.2425	0.2612	0.2799	0.2975	0.3135	0.3295	0.3456	0.3616
South Asia	0.2411	0.2458	0.2504	0.2553	0.2599	0.2648	0.2695	0.2741	0.2790	0.2836	0.2882	0.2931	0.2980	0.3032	0.3085	0.3143	0.3204	0.3269	0.3339	0.3413
South Asia	0.2411	0.2458	0.2504	0.2553	0.2599	0.2648	0.2695	0.2741	0.2790	0.2836	0.2882	0.2931	0.2980	0.3032	0.3085	0.3143	0.3204	0.3269	0.3339	0.3413
Bangladesh	0.2168	0.2207	0.2245	0.2277	0.2313	0.2344	0.2379	0.2415	0.2453	0.2495	0.2538	0.2584	0.2633	0.2686	0.2745	0.2807	0.2870	0.2935	0.3000	0.3071
Bhutan	0.1881	0.1923	0.1967	0.2011	0.2061	0.2108	0.2157	0.2208	0.2260	0.2316	0.2367	0.2421	0.2474	0.2531	0.2591	0.2654	0.2715	0.2786	0.2859	0.2934
India	0.2481	0.2530	0.2579	0.2631	0.2678	0.2731	0.2781	0.2830	0.2882	0.2929	0.2976	0.3026	0.3075	0.3127	0.3180	0.3236	0.3295	0.3356	0.3424	0.3495
Nepal	0.1647	0.1671	0.1698	0.1725	0.1755	0.1785	0.1814	0.1843	0.1874	0.1910	0.1944	0.1980	0.2020	0.2056	0.2097	0.2143	0.2191	0.2240	0.2296	0.2357
Pakistan	0.1924	0.1950	0.1976	0.2003	0.2032	0.2059	0.2088	0.2112	0.2130	0.2144	0.2159	0.2175	0.2197	0.2227	0.2270	0.2337	0.2429	0.2546	0.2681	0.2824
Andhra Pradesh	0.2363	0.2403	0.2443	0.2485	0.2524	0.2569	0.2610	0.2650	0.2693	0.2729	0.2765	0.2809	0.2850	0.2898	0.2941	0.2987	0.3034	0.3086	0.3151	0.3219
Arunachal Pradesh	0.2182	0.2228	0.2273	0.2321	0.2365	0.2413	0.2459	0.2504	0.2551	0.2594	0.2633	0.2685	0.2742	0.2798	0.2857	0.2923	0.2997	0.3068	0.3145	0.3217
Assam	0.2396	0.2450	0.2503	0.2559	0.2610	0.2668	0.2723	0.2776	0.2831	0.2879	0.2924	0.2984	0.3044	0.3108	0.3179	0.3250	0.3321	0.3394	0.3460	0.3532
Bihar	0.2106	0.2144	0.2182	0.2223	0.2261	0.2305	0.2345	0.2384	0.2427	0.2464	0.2504	0.2548	0.2596	0.2641	0.2696	0.2749	0.2812	0.2870	0.2937	0.2998
Chhattisgarh	0.2172	0.2213	0.2253	0.2294	0.2332	0.2374	0.2413	0.2448	0.2486	0.2522	0.2561	0.2603	0.2644	0.2686	0.2730	0.2773	0.2821	0.2870	0.2923	0.2980
Delhi	0.3806	0.3913	0.4017	0.4121	0.4218	0.4315	0.4405	0.4488	0.4570	0.4644	0.4713	0.4778	0.4844	0.4901	0.4950	0.5000	0.5053	0.5102	0.5150	0.5199
Goa	0.3059	0.3148	0.3240	0.3335	0.3425	0.3516	0.3602	0.3687	0.3775	0.3855	0.3935	0.4008	0.4088	0.4159	0.4244	0.4318	0.4400	0.4481	0.4562	0.4647
Gujarat	0.2604	0.2663	0.2722	0.2783	0.2841	0.2904	0.2964	0.3022	0.3082	0.3138	0.3192	0.3257	0.3314	0.3384	0.3453	0.3515	0.3590	0.3649	0.3726	0.3804
Haryana	0.2554	0.2612	0.2671	0.2733	0.2793	0.2858	0.2921	0.2982	0.3046	0.3108	0.3172	0.3238	0.3310	0.3381	0.3450	0.3529	0.3609	0.3685	0.3773	0.3860
Himachal Pradesh	0.2269	0.2324	0.2381	0.2442	0.2500	0.2565	0.2626	0.2689	0.2756	0.2820	0.2885	0.2958	0.3029	0.3107	0.3178	0.3257	0.3343	0.3429	0.3530	0.3634
Jammu and Kashmir	0.2184	0.2240	0.2295	0.2353	0.2408	0.2470	0.2528	0.2585	0.2645	0.2702	0.2760	0.2821	0.2884	0.2947	0.3014	0.3080	0.3156	0.3218	0.3290	0.3360
Jharkhand	0.2255	0.2290	0.2324	0.2359	0.2392	0.2428	0.2461	0.2491	0.2524	0.2554	0.2588	0.2623	0.2658	0.2693	0.2730	0.2767	0.2808	0.2850	0.2897	0.2947

Appendix Table 5A. SDI Values by location, 1970-1989																				
**Location**	**1970**	**1971**	**1972**	**1973**	**1974**	**1975**	**1976**	**1977**	**1978**	**1979**	**1980**	**1981**	**1982**	**1983**	**1984**	**1985**	**1986**	**1987**	**1988**	**1989**
Karnataka	0.2433	0.2484	0.2535	0.2589	0.2640	0.2696	0.2749	0.2801	0.2857	0.2906	0.2956	0.3009	0.3061	0.3119	0.3181	0.3238	0.3304	0.3372	0.3444	0.3520
Kerala	0.2996	0.3073	0.3148	0.3226	0.3296	0.3371	0.3440	0.3507	0.3575	0.3632	0.3690	0.3740	0.3794	0.3851	0.3910	0.3964	0.4026	0.4089	0.4152	0.4219
Madhya Pradesh	0.2270	0.2311	0.2352	0.2396	0.2437	0.2483	0.2527	0.2570	0.2617	0.2661	0.2708	0.2755	0.2804	0.2860	0.2912	0.2972	0.3028	0.3094	0.3166	0.3236
Maharashtra	0.2706	0.2770	0.2836	0.2905	0.2971	0.3042	0.3110	0.3178	0.3250	0.3318	0.3388	0.3460	0.3531	0.3605	0.3677	0.3752	0.3828	0.3908	0.3990	0.4083
Manipur	0.2147	0.2212	0.2277	0.2346	0.2411	0.2480	0.2545	0.2607	0.2675	0.2740	0.2804	0.2878	0.2949	0.3024	0.3104	0.3182	0.3263	0.3358	0.3456	0.3548
Meghalaya	0.2370	0.2424	0.2476	0.2531	0.2582	0.2637	0.2687	0.2735	0.2785	0.2827	0.2869	0.2914	0.2957	0.3003	0.3053	0.3104	0.3161	0.3226	0.3282	0.3352
Mizoram	0.2642	0.2715	0.2787	0.2861	0.2930	0.3005	0.3076	0.3145	0.3216	0.3277	0.3339	0.3400	0.3459	0.3520	0.3592	0.3687	0.3781	0.3899	0.3990	0.4073
Nagaland	0.2750	0.2831	0.2909	0.2988	0.3060	0.3136	0.3203	0.3267	0.3330	0.3383	0.3431	0.3483	0.3542	0.3599	0.3658	0.3713	0.3766	0.3828	0.3892	0.3950
Odisha	0.2091	0.2133	0.2174	0.2217	0.2257	0.2302	0.2344	0.2385	0.2429	0.2467	0.2507	0.2551	0.2588	0.2643	0.2687	0.2741	0.2802	0.2853	0.2917	0.2992
Punjab	0.2590	0.2658	0.2727	0.2800	0.2870	0.2944	0.3015	0.3083	0.3156	0.3225	0.3294	0.3368	0.3444	0.3517	0.3592	0.3670	0.3750	0.3833	0.3915	0.4006
Rajasthan	0.2102	0.2144	0.2186	0.2231	0.2272	0.2320	0.2365	0.2410	0.2457	0.2499	0.2541	0.2588	0.2635	0.2696	0.2749	0.2802	0.2863	0.2918	0.2994	0.3062
Sikkim	0.2200	0.2249	0.2296	0.2345	0.2390	0.2442	0.2491	0.2539	0.2589	0.2635	0.2684	0.2732	0.2785	0.2836	0.2899	0.2966	0.3043	0.3125	0.3207	0.3289
Tamil Nadu	0.2562	0.2614	0.2667	0.2723	0.2775	0.2831	0.2884	0.2936	0.2992	0.3041	0.3089	0.3146	0.3195	0.3245	0.3304	0.3366	0.3434	0.3511	0.3589	0.3675
Telangana	0.2285	0.2323	0.2360	0.2400	0.2436	0.2477	0.2514	0.2551	0.2590	0.2622	0.2653	0.2694	0.2732	0.2776	0.2817	0.2863	0.2908	0.2960	0.3025	0.3093
Tripura	0.2645	0.2702	0.2758	0.2816	0.2868	0.2926	0.2978	0.3027	0.3077	0.3116	0.3154	0.3195	0.3238	0.3276	0.3315	0.3350	0.3388	0.3425	0.3479	0.3530
Uttar Pradesh	0.2239	0.2278	0.2317	0.2359	0.2397	0.2440	0.2481	0.2522	0.2566	0.2604	0.2646	0.2685	0.2731	0.2776	0.2823	0.2874	0.2932	0.2990	0.3055	0.3124
Uttarakhand	0.2161	0.2204	0.2246	0.2288	0.2328	0.2372	0.2413	0.2450	0.2490	0.2528	0.2571	0.2618	0.2667	0.2718	0.2772	0.2828	0.2889	0.2952	0.3019	0.3089
West Bengal	0.2786	0.2834	0.2881	0.2931	0.2976	0.3026	0.3073	0.3118	0.3164	0.3201	0.3239	0.3275	0.3312	0.3349	0.3393	0.3432	0.3471	0.3520	0.3563	0.3610
Union Territories other than Delhi	0.3009	0.3090	0.3170	0.3254	0.3333	0.3416	0.3495	0.3571	0.3648	0.3724	0.3803	0.3885	0.3966	0.4046	0.4126	0.4204	0.4285	0.4365	0.4445	0.4525
Andhra Pradesh, Urban	0.3344	0.3392	0.3439	0.3486	0.3528	0.3575	0.3617	0.3657	0.3700	0.3736	0.3771	0.3820	0.3868	0.3924	0.3975	0.4028	0.4078	0.4133	0.4199	0.4265
Andhra Pradesh, Rural	0.2054	0.2088	0.2121	0.2156	0.2188	0.2225	0.2259	0.2293	0.2328	0.2358	0.2386	0.2423	0.2456	0.2494	0.2529	0.2567	0.2606	0.2650	0.2708	0.2769
Arunachal Pradesh, Urban	0.3545	0.3601	0.3655	0.3711	0.3762	0.3818	0.3870	0.3920	0.3972	0.4017	0.4055	0.4107	0.4163	0.4215	0.4268	0.4327	0.4394	0.4452	0.4516	0.4570
Arunachal Pradesh, Rural	0.2109	0.2151	0.2192	0.2235	0.2274	0.2318	0.2359	0.2398	0.2439	0.2476	0.2510	0.2556	0.2606	0.2655	0.2706	0.2764	0.2829	0.2891	0.2959	0.3022
Assam, Urban	0.3766	0.3843	0.3918	0.3994	0.4062	0.4137	0.4205	0.4269	0.4332	0.4382	0.4427	0.4487	0.4544	0.4602	0.4667	0.4726	0.4783	0.4840	0.4886	0.4939
Assam, Rural	0.2246	0.2296	0.2344	0.2396	0.2444	0.2498	0.2550	0.2600	0.2652	0.2698	0.2742	0.2800	0.2858	0.2921	0.2990	0.3060	0.3131	0.3204	0.3269	0.3341
Bihar, Urban	0.3156	0.3213	0.3270	0.3330	0.3385	0.3445	0.3499	0.3550	0.3605	0.3654	0.3703	0.3766	0.3841	0.3910	0.3986	0.4056	0.4134	0.4202	0.4277	0.4343
Bihar, Rural	0.1952	0.1985	0.2020	0.2057	0.2091	0.2131	0.2168	0.2204	0.2243	0.2277	0.2313	0.2352	0.2393	0.2432	0.2481	0.2528	0.2584	0.2636	0.2697	0.2753
Chhattisgarh, Urban	0.3841	0.3919	0.3995	0.4072	0.4145	0.4221	0.4294	0.4363	0.4433	0.4497	0.4563	0.4623	0.4676	0.4725	0.4769	0.4809	0.4848	0.4886	0.4923	0.4961
Chhattisgarh, Rural	0.1743	0.1772	0.1801	0.1830	0.1858	0.1889	0.1917	0.1943	0.1971	0.1998	0.2029	0.2065	0.2102	0.2142	0.2185	0.2228	0.2277	0.2328	0.2383	0.2443
Delhi, Urban	0.3883	0.3990	0.4094	0.4199	0.4295	0.4392	0.4481	0.4564	0.4645	0.4717	0.4785	0.4848	0.4913	0.4967	0.5015	0.5065	0.5116	0.5165	0.5214	0.5263
Delhi, Rural	0.3070	0.3158	0.3244	0.3332	0.3414	0.3499	0.3580	0.3657	0.3735	0.3807	0.3878	0.3948	0.4022	0.4090	0.4152	0.4218	0.4289	0.4359	0.4430	0.4503
Goa, Urban	0.3769	0.3857	0.3946	0.4037	0.4121	0.4208	0.4288	0.4367	0.4449	0.4519	0.4586	0.4644	0.4709	0.4763	0.4831	0.4885	0.4949	0.5011	0.5071	0.5137
Goa, Rural	0.2861	0.2941	0.3025	0.3112	0.3194	0.3277	0.3354	0.3430	0.3509	0.3582	0.3655	0.3721	0.3795	0.3861	0.3940	0.4010	0.4089	0.4167	0.4244	0.4328
Gujarat, Urban	0.3380	0.3457	0.3533	0.3609	0.3680	0.3755	0.3824	0.3890	0.3957	0.4018	0.4075	0.4150	0.4222	0.4307	0.4386	0.4456	0.4536	0.4594	0.4669	0.4742
Gujarat, Rural	0.2237	0.2285	0.2333	0.2383	0.2431	0.2484	0.2534	0.2583	0.2635	0.2683	0.2729	0.2783	0.2828	0.2887	0.2945	0.2998	0.3065	0.3118	0.3190	0.3264
Haryana, Urban	0.3571	0.3642	0.3714	0.3790	0.3862	0.3940	0.4012	0.4081	0.4152	0.4217	0.4282	0.4356	0.4442	0.4523	0.4597	0.4679	0.4757	0.4826	0.4906	0.4982
Haryana, Rural	0.2293	0.2341	0.2389	0.2443	0.2495	0.2553	0.2610	0.2665	0.2723	0.2780	0.2838	0.2896	0.2958	0.3020	0.3080	0.3150	0.3223	0.3292	0.3374	0.3455
Himachal Pradesh, Urban	0.3665	0.3740	0.3813	0.3889	0.3957	0.4029	0.4093	0.4155	0.4220	0.4277	0.4332	0.4405	0.4487	0.4572	0.4643	0.4721	0.4801	0.4877	0.4965	0.5052
Himachal Pradesh, Rural	0.2150	0.2202	0.2255	0.2313	0.2370	0.2432	0.2493	0.2555	0.2621	0.2685	0.2750	0.2822	0.2891	0.2968	0.3037	0.3116	0.3201	0.3286	0.3386	0.3490
Jammu and Kashmir, Urban	0.3023	0.3091	0.3158	0.3227	0.3290	0.3358	0.3420	0.3479	0.3541	0.3599	0.3660	0.3736	0.3829	0.3921	0.4015	0.4106	0.4203	0.4281	0.4366	0.4444
Jammu and Kashmir, Rural	0.1941	0.1990	0.2039	0.2091	0.2141	0.2198	0.2252	0.2306	0.2363	0.2416	0.2471	0.2525	0.2579	0.2634	0.2692	0.2751	0.2819	0.2875	0.2941	0.3005
Jharkhand, Urban	0.3962	0.4020	0.4077	0.4137	0.4193	0.4254	0.4311	0.4367	0.4423	0.4474	0.4527	0.4575	0.4617	0.4656	0.4692	0.4724	0.4759	0.4793	0.4831	0.4872
Jharkhand, Rural	0.2044	0.2074	0.2103	0.2133	0.2161	0.2192	0.2221	0.2246	0.2274	0.2299	0.2327	0.2357	0.2386	0.2416	0.2447	0.2478	0.2513	0.2550	0.2590	0.2635
Karnataka, Urban	0.3277	0.3345	0.3412	0.3482	0.3544	0.3612	0.3674	0.3734	0.3797	0.3854	0.3910	0.3975	0.4045	0.4119	0.4196	0.4265	0.4340	0.4413	0.4487	0.4562
Karnataka, Rural	0.2091	0.2132	0.2173	0.2216	0.2256	0.2301	0.2344	0.2386	0.2432	0.2472	0.2513	0.2555	0.2596	0.2642	0.2693	0.2741	0.2799	0.2859	0.2924	0.2994
Kerala, Urban	0.3381	0.3466	0.3548	0.3631	0.3703	0.3778	0.3846	0.3910	0.3974	0.4026	0.4077	0.4122	0.4172	0.4224	0.4278	0.4324	0.4378	0.4432	0.4483	0.4539
Kerala, Rural	0.2919	0.2992	0.3065	0.3139	0.3205	0.3277	0.3343	0.3407	0.3472	0.3527	0.3584	0.3631	0.3683	0.3738	0.3796	0.3849	0.3911	0.3975	0.4038	0.4106
Madhya Pradesh, Urban	0.3499	0.3559	0.3618	0.3678	0.3731	0.3787	0.3838	0.3886	0.3938	0.3985	0.4033	0.4088	0.4152	0.4222	0.4284	0.4350	0.4409	0.4475	0.4546	0.4612
Madhya Pradesh, Rural	0.1933	0.1966	0.1998	0.2033	0.2065	0.2103	0.2138	0.2174	0.2212	0.2248	0.2286	0.2323	0.2361	0.2405	0.2446	0.2494	0.2540	0.2597	0.2660	0.2722

Appendix Table 5A. SDI Values by location, 1970-1989																				
Location	1970	1971	1972	1973	1974	1975	1976	1977	1978	1979	1980	1981	1982	1983	1984	1985	1986	1987	1988	1989
Maharashtra, Urban	0.3442	0.3526	0.3609	0.3693	0.3771	0.3851	0.3925	0.3998	0.4072	0.4141	0.4210	0.4284	0.4360	0.4436	0.4507	0.4579	0.4648	0.4716	0.4784	0.4858
Maharashtra, Rural	0.2294	0.2346	0.2399	0.2456	0.2511	0.2572	0.2632	0.2692	0.2757	0.2818	0.2883	0.2948	0.3011	0.3077	0.3144	0.3216	0.3289	0.3368	0.3451	0.3544
Manipur, Urban	0.2751	0.2834	0.2914	0.2995	0.3070	0.3147	0.3215	0.3277	0.3342	0.3400	0.3456	0.3531	0.3609	0.3691	0.3776	0.3855	0.3933	0.4022	0.4110	0.4191
Manipur, Rural	0.2051	0.2110	0.2169	0.2231	0.2291	0.2354	0.2414	0.2472	0.2534	0.2595	0.2654	0.2722	0.2784	0.2850	0.2921	0.2989	0.3061	0.3148	0.3238	0.3323
Meghalaya, Urban	0.3988	0.4067	0.4142	0.4219	0.4284	0.4351	0.4406	0.4454	0.4500	0.4531	0.4560	0.4586	0.4603	0.4618	0.4633	0.4646	0.4662	0.4683	0.4689	0.4711
Meghalaya, Rural	0.2049	0.2096	0.2141	0.2189	0.2233	0.2281	0.2326	0.2370	0.2416	0.2456	0.2496	0.2539	0.2583	0.2629	0.2680	0.2733	0.2792	0.2860	0.2919	0.2992
Mizoram, Urban	0.3076	0.3159	0.3239	0.3322	0.3398	0.3480	0.3556	0.3629	0.3703	0.3765	0.3827	0.3889	0.3949	0.4010	0.4081	0.4172	0.4262	0.4373	0.4457	0.4533
Mizoram, Rural	0.2316	0.2378	0.2439	0.2502	0.2561	0.2627	0.2689	0.2750	0.2813	0.2867	0.2923	0.2978	0.3030	0.3085	0.3154	0.3246	0.3340	0.3457	0.3548	0.3632
Nagaland, Urban	0.3853	0.3941	0.4023	0.4102	0.4170	0.4239	0.4296	0.4345	0.4393	0.4428	0.4459	0.4509	0.4583	0.4656	0.4731	0.4800	0.4864	0.4935	0.5006	0.5067
Nagaland, Rural	0.2636	0.2712	0.2785	0.2860	0.2927	0.2999	0.3063	0.3124	0.3184	0.3233	0.3279	0.3326	0.3379	0.3429	0.3481	0.3528	0.3574	0.3628	0.3685	0.3736
Odisha, Urban	0.3179	0.3237	0.3294	0.3353	0.3406	0.3463	0.3514	0.3562	0.3611	0.3654	0.3698	0.3756	0.3816	0.3895	0.3960	0.4032	0.4110	0.4172	0.4244	0.4322
Odisha, Rural	0.1958	0.1995	0.2030	0.2068	0.2103	0.2143	0.2181	0.2218	0.2257	0.2291	0.2328	0.2366	0.2398	0.2446	0.2484	0.2532	0.2587	0.2632	0.2690	0.2758
Punjab, Urban	0.3558	0.3644	0.3731	0.3821	0.3907	0.3999	0.4086	0.4170	0.4256	0.4335	0.4410	0.4487	0.4562	0.4632	0.4702	0.4772	0.4841	0.4913	0.4981	0.5056
Punjab, Rural	0.2259	0.2314	0.2372	0.2432	0.2491	0.2554	0.2613	0.2671	0.2734	0.2795	0.2857	0.2925	0.2998	0.3068	0.3141	0.3217	0.3296	0.3379	0.3461	0.3551
Rajasthan, Urban	0.3202	0.3256	0.3310	0.3366	0.3418	0.3475	0.3529	0.3583	0.3639	0.3688	0.3737	0.3789	0.3839	0.3903	0.3957	0.4009	0.4068	0.4119	0.4190	0.4251
Rajasthan, Rural	0.1803	0.1840	0.1875	0.1914	0.1949	0.1990	0.2029	0.2067	0.2108	0.2143	0.2179	0.2219	0.2261	0.2316	0.2363	0.2410	0.2465	0.2514	0.2585	0.2648
Sikkim, Urban	0.3070	0.3146	0.3216	0.3286	0.3350	0.3417	0.3480	0.3539	0.3599	0.3652	0.3706	0.3773	0.3860	0.3944	0.4041	0.4144	0.4258	0.4374	0.4483	0.4586
Sikkim, Rural	0.2118	0.2163	0.2206	0.2251	0.2294	0.2342	0.2388	0.2433	0.2481	0.2524	0.2569	0.2613	0.2662	0.2708	0.2765	0.2827	0.2898	0.2975	0.3051	0.3128
Tamil Nadu, Urban	0.3289	0.3355	0.3421	0.3490	0.3552	0.3619	0.3682	0.3743	0.3807	0.3862	0.3914	0.3975	0.4026	0.4076	0.4133	0.4191	0.4253	0.4322	0.4387	0.4458
Tamil Nadu, Rural	0.2206	0.2247	0.2288	0.2333	0.2374	0.2421	0.2464	0.2509	0.2557	0.2600	0.2643	0.2697	0.2744	0.2795	0.2854	0.2919	0.2990	0.3070	0.3150	0.3238
Telangana, Urban	0.3126	0.3190	0.3253	0.3317	0.3374	0.3436	0.3492	0.3546	0.3602	0.3652	0.3700	0.3762	0.3824	0.3894	0.3957	0.4024	0.4087	0.4155	0.4235	0.4316
Telangana, Rural	0.1985	0.2014	0.2042	0.2072	0.2098	0.2128	0.2156	0.2182	0.2211	0.2233	0.2254	0.2283	0.2309	0.2341	0.2370	0.2403	0.2437	0.2478	0.2531	0.2588
Tripura, Urban	0.3957	0.4026	0.4092	0.4158	0.4215	0.4276	0.4327	0.4373	0.4416	0.4444	0.4470	0.4499	0.4527	0.4546	0.4564	0.4576	0.4590	0.4601	0.4630	0.4654
Tripura, Rural	0.2475	0.2527	0.2578	0.2631	0.2678	0.2731	0.2779	0.2825	0.2871	0.2907	0.2942	0.2981	0.3022	0.3059	0.3096	0.3131	0.3169	0.3205	0.3259	0.3311
Uttar Pradesh, Urban	0.3187	0.3241	0.3293	0.3347	0.3394	0.3444	0.3491	0.3536	0.3583	0.3624	0.3668	0.3713	0.3768	0.3822	0.3874	0.3928	0.3986	0.4042	0.4104	0.4167
Uttar Pradesh, Rural	0.2039	0.2075	0.2109	0.2146	0.2179	0.2216	0.2252	0.2288	0.2327	0.2360	0.2397	0.2430	0.2469	0.2508	0.2548	0.2593	0.2644	0.2697	0.2757	0.2821
Uttarakhand, Urban	0.3403	0.3469	0.3533	0.3597	0.3658	0.3722	0.3782	0.3839	0.3899	0.3952	0.4009	0.4065	0.4117	0.4169	0.4221	0.4270	0.4322	0.4373	0.4425	0.4479
Uttarakhand, Rural	0.1910	0.1944	0.1977	0.2010	0.2041	0.2075	0.2106	0.2134	0.2164	0.2193	0.2226	0.2265	0.2307	0.2351	0.2399	0.2448	0.2504	0.2563	0.2626	0.2693
West Bengal, Urban	0.3872	0.3935	0.3995	0.4057	0.4112	0.4170	0.4223	0.4272	0.4321	0.4361	0.4399	0.4444	0.4496	0.4549	0.4607	0.4658	0.4706	0.4763	0.4811	0.4860
West Bengal, Rural	0.2347	0.2390	0.2431	0.2475	0.2515	0.2560	0.2603	0.2644	0.2685	0.2719	0.2754	0.2785	0.2814	0.2844	0.2881	0.2914	0.2948	0.2992	0.3031	0.3075
Union Territories other than Delhi, Rural	0.2419	0.2466	0.2515	0.2569	0.2620	0.2675	0.2728	0.2776	0.2826	0.2876	0.2930	0.2992	0.3058	0.3125	0.3192	0.3259	0.3331	0.3403	0.3476	0.3551
Union Territories other than Delhi, Urban	0.4021	0.4096	0.4174	0.4256	0.4334	0.4416	0.4493	0.4566	0.4638	0.4702	0.4767	0.4827	0.4880	0.4929	0.4974	0.5014	0.5057	0.5099	0.5141	0.5186
Sub-Saharan Africa	0.2122	0.2170	0.2217	0.2268	0.2321	0.2372	0.2424	0.2476	0.2527	0.2577	0.2627	0.2676	0.2724	0.2772	0.2820	0.2869	0.2909	0.2946	0.2984	0.3025
Central Sub-Saharan Africa	0.2187	0.2232	0.2275	0.2323	0.2372	0.2412	0.2450	0.2485	0.2516	0.2550	0.2587	0.2624	0.2664	0.2705	0.2752	0.2796	0.2833	0.2869	0.2907	0.2947
Angola	0.1863	0.1903	0.1942	0.1983	0.2021	0.2050	0.2076	0.2101	0.2125	0.2151	0.2178	0.2203	0.2228	0.2255	0.2286	0.2321	0.2353	0.2391	0.2433	0.2477
Central African Republic	0.1804	0.1837	0.1868	0.1902	0.1932	0.1962	0.1994	0.2031	0.2066	0.2098	0.2125	0.2162	0.2196	0.2223	0.2257	0.2295	0.2332	0.2362	0.2396	0.2433
Congo	0.2327	0.2388	0.2451	0.2518	0.2589	0.2658	0.2726	0.2786	0.2847	0.2916	0.2996	0.3081	0.3170	0.3262	0.3355	0.3440	0.3509	0.3577	0.3648	0.3723
Democratic Republic of the Congo	0.2126	0.2165	0.2201	0.2245	0.2291	0.2323	0.2349	0.2375	0.2397	0.2427	0.2459	0.2492	0.2530	0.2571	0.2624	0.2669	0.2707	0.2740	0.2774	0.2808
Equatorial Guinea	0.1929	0.1962	0.1989	0.2021	0.2055	0.2100	0.2168	0.2240	0.2304	0.2363	0.2423	0.2521	0.2604	0.2681	0.2745	0.2794	0.2819	0.2837	0.2862	0.2888
Gabon	0.2874	0.2952	0.3033	0.3119	0.3221	0.3326	0.3442	0.3541	0.3627	0.3715	0.3804	0.3891	0.3974	0.4057	0.4139	0.4218	0.4290	0.4353	0.4419	0.4497
Eastern Sub-Saharan Africa	0.1356	0.1389	0.1422	0.1458	0.1496	0.1537	0.1580	0.1625	0.1671	0.1716	0.1761	0.1806	0.1851	0.1900	0.1955	0.2011	0.2066	0.2118	0.2171	0.2225
Burundi	0.0859	0.0899	0.0935	0.0974	0.1016	0.1057	0.1102	0.1155	0.1207	0.1260	0.1311	0.1366	0.1420	0.1478	0.1537	0.1600	0.1662	0.1721	0.1778	0.1831
Comoros	0.1635	0.1676	0.1716	0.1756	0.1801	0.1846	0.1891	0.1935	0.1981	0.2030	0.2081	0.2134	0.2190	0.2251	0.2318	0.2388	0.2460	0.2531	0.2604	0.2675
Djibouti	0.0722	0.0734	0.0746	0.0758	0.0769	0.0780	0.0793	0.0804	0.0813	0.0822	0.0888	0.1001	0.1102	0.1214	0.1348	0.1479	0.1599	0.1708	0.1806	0.1899
Eritrea	0.1077	0.1110	0.1142	0.1175	0.1210	0.1244	0.1280	0.1316	0.1353	0.1392	0.1429	0.1465	0.1501	0.1539	0.1582	0.1628	0.1675	0.1722	0.1771	0.1822
Ethiopia	0.0969	0.0991	0.1016	0.1042	0.1064	0.1090	0.1116	0.1140	0.1166	0.1189	0.1217	0.1249	0.1282	0.1322	0.1363	0.1390	0.1419	0.1449	0.1477	0.1502
Kenya	0.0807	0.0917	0.1052	0.1179	0.1326	0.1485	0.1635	0.1780	0.1923	0.2062	0.2196	0.2325	0.2449	0.2572	0.2692	0.2804	0.2914	0.3020	0.3125	0.3234
Madagascar	0.1373	0.1425	0.1477	0.1531	0.1589	0.1649	0.1709	0.1771	0.1830	0.1891	0.1954	0.2013	0.2069	0.2128	0.2192	0.2257	0.2321	0.2381	0.2438	0.2493
Malawi	0.0909	0.0951	0.0998	0.1046	0.1096	0.1148	0.1201	0.1256	0.1313	0.1370	0.1429	0.1487	0.1545	0.1607	0.1674	0.1746	0.1816	0.1880	0.1938	0.1994

Appendix Table 5A. SDI Values by location, 1970-1989																				
**Location**	**1970**	**1971**	**1972**	**1973**	**1974**	**1975**	**1976**	**1977**	**1978**	**1979**	**1980**	**1981**	**1982**	**1983**	**1984**	**1985**	**1986**	**1987**	**1988**	**1989**
Mozambique	0.1035	0.1066	0.1096	0.1127	0.1157	0.1184	0.1212	0.1237	0.1260	0.1283	0.1307	0.1333	0.1353	0.1360	0.1370	0.1376	0.1381	0.1386	0.1396	0.1420
Rwanda	0.0534	0.0546	0.0558	0.0569	0.0580	0.0591	0.0603	0.0616	0.0627	0.0641	0.0654	0.0667	0.0678	0.0688	0.0699	0.0708	0.0886	0.1200	0.1436	0.1637
Somalia	0.1394	0.1412	0.1438	0.1459	0.1472	0.1499	0.1519	0.1548	0.1576	0.1594	0.1605	0.1622	0.1644	0.1660	0.1680	0.1706	0.1732	0.1764	0.1797	0.1833
Tanzania	0.1118	0.1172	0.1227	0.1288	0.1351	0.1416	0.1483	0.1550	0.1619	0.1689	0.1761	0.1832	0.1900	0.1970	0.2046	0.2128	0.2201	0.2265	0.2330	0.2391
Uganda	0.0642	0.0654	0.0667	0.0679	0.0690	0.0699	0.0708	0.0718	0.0779	0.0908	0.1012	0.1097	0.1174	0.1254	0.1338	0.1418	0.1492	0.1561	0.1629	0.1698
Zambia	0.0888	0.0901	0.0915	0.0928	0.0941	0.1160	0.1353	0.1520	0.1668	0.1804	0.1930	0.2051	0.2163	0.2275	0.2391	0.2506	0.2613	0.2714	0.2814	0.2905
South Sudan	0.0464	0.0471	0.0478	0.0485	0.0491	0.0498	0.0505	0.0511	0.0518	0.0525	0.0531	0.0538	0.0544	0.0551	0.0557	0.0564	0.0570	0.0623	0.0701	0.0767
Central	0.1580	0.1698	0.1815	0.1933	0.2066	0.2210	0.2353	0.2498	0.2644	0.2786	0.2920	0.3050	0.3177	0.3305	0.3434	0.3557	0.3678	0.3792	0.3903	0.4014
Coast	0.0721	0.0737	0.0753	0.0769	0.0786	0.0800	0.0815	0.0880	0.1110	0.1299	0.1456	0.1596	0.1725	0.1847	0.1955	0.2048	0.2138	0.2224	0.2312	0.2406
Eastern	0.0740	0.0757	0.0775	0.0833	0.1021	0.1208	0.1374	0.1530	0.1681	0.1827	0.1960	0.2088	0.2210	0.2331	0.2448	0.2557	0.2664	0.2768	0.2871	0.2977
Nairobi	0.2947	0.3066	0.3186	0.3306	0.3433	0.3560	0.3686	0.3820	0.3954	0.4080	0.4197	0.4308	0.4417	0.4527	0.4642	0.4754	0.4863	0.4964	0.5062	0.5158
North Eastern	0.0398	0.0407	0.0417	0.0426	0.0436	0.0445	0.0454	0.0464	0.0475	0.0485	0.0493	0.0502	0.0510	0.0518	0.0527	0.0535	0.0544	0.0553	0.0562	0.0571
Nyanza	0.0769	0.0786	0.0804	0.0934	0.1108	0.1288	0.1451	0.1606	0.1758	0.1904	0.2037	0.2165	0.2287	0.2408	0.2526	0.2635	0.2743	0.2847	0.2951	0.3058
Rift Valley	0.0807	0.0822	0.0838	0.0854	0.0869	0.0883	0.0896	0.1135	0.1367	0.1565	0.1732	0.1883	0.2023	0.2156	0.2277	0.2386	0.2491	0.2592	0.2693	0.2798
Western	0.0848	0.0954	0.1105	0.1243	0.1398	0.1563	0.1717	0.1866	0.2011	0.2151	0.2277	0.2397	0.2512	0.2626	0.2736	0.2837	0.2936	0.3030	0.3121	0.3216
Kiambu	0.1584	0.1729	0.1870	0.2010	0.2166	0.2333	0.2496	0.2659	0.2822	0.2980	0.3127	0.3270	0.3408	0.3546	0.3684	0.3816	0.3945	0.4065	0.4181	0.4292
Kirinyaga	0.1700	0.1789	0.1879	0.1972	0.2080	0.2197	0.2314	0.2437	0.2560	0.2682	0.2799	0.2915	0.3029	0.3144	0.3260	0.3371	0.3481	0.3585	0.3687	0.3787
Murang'a	0.1459	0.1578	0.1693	0.1810	0.1941	0.2084	0.2224	0.2366	0.2509	0.2647	0.2777	0.2903	0.3026	0.3151	0.3277	0.3398	0.3517	0.3628	0.3736	0.3843
Nyandarua	0.1391	0.1498	0.1603	0.1709	0.1831	0.1965	0.2097	0.2232	0.2368	0.2500	0.2627	0.2751	0.2872	0.2997	0.3125	0.3248	0.3369	0.3484	0.3598	0.3713
Nyeri	0.1592	0.1707	0.1822	0.1940	0.2071	0.2210	0.2349	0.2494	0.2640	0.2782	0.2920	0.3054	0.3184	0.3315	0.3448	0.3575	0.3703	0.3824	0.3942	0.4066
Kilifi	0.0708	0.0723	0.0738	0.0752	0.0767	0.0781	0.0794	0.0809	0.0823	0.0837	0.0849	0.0861	0.0872	0.0882	0.0892	0.0901	0.0978	0.1111	0.1225	0.1342
Kwale	0.0660	0.0675	0.0692	0.0707	0.0723	0.0738	0.0752	0.0887	0.1042	0.1186	0.1303	0.1411	0.1512	0.1611	0.1700	0.1774	0.1849	0.1923	0.2003	0.2094
Lamu	0.0724	0.0738	0.0753	0.0768	0.0783	0.0891	0.1081	0.1249	0.1404	0.1552	0.1683	0.1809	0.1929	0.2046	0.2150	0.2241	0.2328	0.2410	0.2488	0.2554
Mombasa	0.1240	0.1334	0.1433	0.1536	0.1656	0.1788	0.1919	0.2057	0.2198	0.2337	0.2463	0.2586	0.2707	0.2826	0.2941	0.3048	0.3155	0.3260	0.3366	0.3475
TaitaTaveta	0.0718	0.0735	0.0753	0.0770	0.0787	0.0803	0.0819	0.0836	0.1149	0.1380	0.1559	0.1718	0.1863	0.1999	0.2120	0.2226	0.2327	0.2425	0.2525	0.2633
TanaRiver	0.0493	0.0509	0.0525	0.0540	0.0556	0.0569	0.0582	0.0598	0.0613	0.0627	0.0640	0.0651	0.0662	0.0770	0.0845	0.0879	0.0905	0.0919	0.0933	0.0972
Embu	0.1114	0.1220	0.1325	0.1432	0.1555	0.1689	0.1821	0.1958	0.2098	0.2236	0.2368	0.2496	0.2620	0.2744	0.2866	0.2982	0.3096	0.3205	0.3313	0.3418
Isiolo	0.1407	0.1454	0.1502	0.1549	0.1605	0.1673	0.1742	0.1818	0.1897	0.1978	0.2050	0.2120	0.2190	0.2261	0.2331	0.2396	0.2460	0.2521	0.2581	0.2636
Kitui	0.0648	0.0664	0.0682	0.0698	0.0715	0.0730	0.0745	0.0917	0.1119	0.1294	0.1445	0.1582	0.1709	0.1834	0.1955	0.2065	0.2173	0.2273	0.2369	0.2458
Machakos	0.0798	0.0817	0.0835	0.0854	0.0872	0.0889	0.1013	0.1319	0.1557	0.1764	0.1940	0.2102	0.2253	0.2398	0.2535	0.2661	0.2783	0.2902	0.3020	0.3148
Makueni	0.0811	0.0827	0.0843	0.0859	0.0875	0.0889	0.0903	0.0919	0.0934	0.1279	0.1537	0.1747	0.1928	0.2098	0.2253	0.2392	0.2522	0.2644	0.2762	0.2879
Marsabit	0.1425	0.1458	0.1492	0.1525	0.1563	0.1603	0.1644	0.1691	0.1741	0.1793	0.1841	0.1887	0.1933	0.1980	0.2027	0.2072	0.2118	0.2162	0.2204	0.2242
Meru	0.0987	0.1067	0.1150	0.1239	0.1349	0.1476	0.1602	0.1728	0.1855	0.1979	0.2094	0.2209	0.2320	0.2432	0.2540	0.2640	0.2742	0.2842	0.2943	0.3053
TharakaNithi	0.0951	0.1031	0.1110	0.1194	0.1298	0.1419	0.1539	0.1665	0.1795	0.1925	0.2045	0.2159	0.2271	0.2384	0.2496	0.2605	0.2714	0.2820	0.2926	0.3032
Nairobi	0.2947	0.3066	0.3186	0.3306	0.3433	0.3560	0.3686	0.3820	0.3954	0.4080	0.4197	0.4308	0.4417	0.4527	0.4642	0.4754	0.4863	0.4964	0.5062	0.5158
Garissa	0.0397	0.0409	0.0421	0.0433	0.0446	0.0457	0.0468	0.0481	0.0494	0.0506	0.0517	0.0527	0.0538	0.0548	0.0558	0.0568	0.0578	0.0589	0.0600	0.0611
Mandera	0.0346	0.0356	0.0366	0.0377	0.0387	0.0397	0.0406	0.0417	0.0428	0.0439	0.0450	0.0460	0.0470	0.0480	0.0490	0.0500	0.0510	0.0521	0.0532	0.0543
Wajir	0.0400	0.0408	0.0416	0.0425	0.0433	0.0441	0.0449	0.0458	0.0466	0.0475	0.0482	0.0489	0.0496	0.0503	0.0510	0.0517	0.0524	0.0531	0.0538	0.0546
HomaBay	0.0761	0.0778	0.0795	0.0811	0.0827	0.0842	0.0886	0.1155	0.1370	0.1561	0.1727	0.1880	0.2021	0.2158	0.2288	0.2403	0.2512	0.2613	0.2710	0.2805
Kisii	0.0784	0.0802	0.0820	0.0877	0.1085	0.1286	0.1461	0.1624	0.1777	0.1924	0.2055	0.2181	0.2303	0.2422	0.2536	0.2642	0.2748	0.2853	0.2960	0.3078
Kisumu	0.0809	0.0826	0.0844	0.0873	0.1099	0.1314	0.1500	0.1672	0.1840	0.1998	0.2142	0.2277	0.2407	0.2536	0.2660	0.2779	0.2897	0.3014	0.3132	0.3260
Migori	0.0768	0.0785	0.0802	0.0819	0.0836	0.0851	0.1090	0.1297	0.1482	0.1653	0.1804	0.1944	0.2075	0.2205	0.2328	0.2439	0.2545	0.2644	0.2739	0.2833
Nyamira	0.1563	0.1645	0.1728	0.1815	0.1917	0.2035	0.2156	0.2286	0.2419	0.2552	0.2675	0.2797	0.2918	0.3040	0.3163	0.3281	0.3399	0.3513	0.3626	0.3735
Siaya	0.0740	0.0804	0.0872	0.0944	0.1032	0.1131	0.1230	0.1335	0.1445	0.1555	0.1659	0.1760	0.1860	0.1960	0.2059	0.2153	0.2249	0.2342	0.2436	0.2531
Baringo	0.0812	0.0826	0.0841	0.0855	0.0869	0.0882	0.0894	0.0908	0.1001	0.1295	0.1509	0.1692	0.1854	0.2002	0.2135	0.2251	0.2362	0.2469	0.2576	0.2683
Bomet	0.0834	0.0851	0.0869	0.0886	0.0903	0.0919	0.0934	0.0950	0.0967	0.0982	0.0994	0.1077	0.1434	0.1689	0.1888	0.2045	0.2184	0.2309	0.2425	0.2542
Elgeyo-Marakwet	0.0732	0.0748	0.0765	0.0782	0.0799	0.0814	0.0829	0.0846	0.1010	0.1285	0.1492	0.1670	0.1829	0.1975	0.2108	0.2226	0.2340	0.2449	0.2554	0.2661
Kajiado	0.0843	0.0858	0.0874	0.0890	0.0906	0.0920	0.1171	0.1411	0.1617	0.1803	0.1970	0.2126	0.2273	0.2413	0.2545	0.2665	0.2783	0.2897	0.3010	0.3126
Kericho	0.0773	0.0791	0.0809	0.0827	0.0845	0.0861	0.0877	0.0895	0.0912	0.0929	0.0943	0.0956	0.1079	0.1351	0.1545	0.1693	0.1828	0.1956	0.2084	0.2236

Appendix Table 5A. SDI Values by location, 1970-1989																				
**Location**	**1970**	**1971**	**1972**	**1973**	**1974**	**1975**	**1976**	**1977**	**1978**	**1979**	**1980**	**1981**	**1982**	**1983**	**1984**	**1985**	**1986**	**1987**	**1988**	**1989**
Laikipia	0.1063	0.1154	0.1247	0.1348	0.1472	0.1612	0.1750	0.1888	0.2026	0.2163	0.2288	0.2412	0.2532	0.2651	0.2763	0.2867	0.2970	0.3069	0.3170	0.3268
Nakuru	0.0867	0.0971	0.1074	0.1175	0.1295	0.1427	0.1556	0.1689	0.1825	0.1958	0.2084	0.2206	0.2324	0.2440	0.2552	0.2658	0.2766	0.2871	0.2978	0.3087
Nandi	0.0844	0.0859	0.0875	0.0891	0.0906	0.0921	0.0935	0.0950	0.0965	0.0980	0.0992	0.1242	0.1515	0.1732	0.1910	0.2057	0.2192	0.2317	0.2438	0.2562
Narok	0.0727	0.0741	0.0756	0.0770	0.0784	0.0797	0.0809	0.0823	0.0837	0.0849	0.1064	0.1253	0.1411	0.1555	0.1678	0.1781	0.1880	0.1978	0.2077	0.2186
Samburu	0.1219	0.1249	0.1279	0.1309	0.1350	0.1401	0.1454	0.1509	0.1567	0.1629	0.1692	0.1752	0.1809	0.1866	0.1918	0.1963	0.2007	0.2048	0.2089	0.2125
TransNzoia	0.0851	0.0867	0.0884	0.0900	0.0917	0.0931	0.0946	0.0961	0.0977	0.0992	0.1032	0.1373	0.1608	0.1804	0.1966	0.2096	0.2217	0.2335	0.2458	0.2605
Turkana	0.1416	0.1447	0.1477	0.1507	0.1545	0.1591	0.1639	0.1690	0.1745	0.1803	0.1852	0.1901	0.1949	0.1999	0.2047	0.2089	0.2130	0.2167	0.2201	0.2227
UasinGishu	0.0876	0.0893	0.0910	0.0927	0.0944	0.0959	0.0975	0.0991	0.1318	0.1608	0.1831	0.2024	0.2201	0.2368	0.2521	0.2657	0.2787	0.2912	0.3034	0.3156
WestPokot	0.0865	0.0904	0.0943	0.0986	0.1049	0.1126	0.1203	0.1282	0.1363	0.1447	0.1527	0.1604	0.1681	0.1758	0.1831	0.1896	0.1964	0.2033	0.2107	0.2195
Bungoma	0.0845	0.0861	0.0878	0.0894	0.0910	0.0924	0.1152	0.1415	0.1634	0.1828	0.1993	0.2143	0.2282	0.2417	0.2545	0.2659	0.2767	0.2866	0.2958	0.3052
Busia	0.1258	0.1349	0.1440	0.1530	0.1638	0.1760	0.1880	0.2001	0.2123	0.2243	0.2351	0.2455	0.2556	0.2658	0.2758	0.2852	0.2943	0.3031	0.3118	0.3206
Kakamega	0.0996	0.1125	0.1240	0.1356	0.1493	0.1641	0.1783	0.1920	0.2053	0.2183	0.2300	0.2413	0.2521	0.2628	0.2732	0.2828	0.2923	0.3015	0.3109	0.3208
Vihiga	0.1383	0.1482	0.1582	0.1683	0.1801	0.1931	0.2059	0.2190	0.2324	0.2455	0.2578	0.2696	0.2812	0.2927	0.3041	0.3147	0.3250	0.3345	0.3437	0.3524
Southern Sub-Saharan Africa	0.4474	0.4534	0.4595	0.4658	0.4722	0.4780	0.4836	0.4888	0.4940	0.4993	0.5052	0.5110	0.5164	0.5214	0.5266	0.5314	0.5359	0.5406	0.5462	0.5523
Botswana	0.2979	0.3084	0.3209	0.3340	0.3461	0.3569	0.3673	0.3768	0.3860	0.3951	0.4044	0.4136	0.4228	0.4319	0.4411	0.4507	0.4608	0.4710	0.4840	0.4973
Lesotho	0.2574	0.2608	0.2659	0.2737	0.2808	0.2862	0.2924	0.3006	0.3105	0.3172	0.3229	0.3281	0.3326	0.3365	0.3406	0.3448	0.3489	0.3527	0.3578	0.3639
Namibia	0.3176	0.3232	0.3288	0.3345	0.3403	0.3460	0.3514	0.3568	0.3623	0.3680	0.3743	0.3800	0.3854	0.3902	0.3949	0.3995	0.4045	0.4099	0.4158	0.4219
South Africa	0.4857	0.4917	0.4975	0.5037	0.5099	0.5156	0.5210	0.5260	0.5312	0.5365	0.5425	0.5483	0.5538	0.5589	0.5642	0.5692	0.5738	0.5787	0.5843	0.5901
Swaziland	0.3119	0.3197	0.3279	0.3353	0.3428	0.3513	0.3596	0.3668	0.3731	0.3787	0.3844	0.3913	0.3979	0.4042	0.4099	0.4151	0.4215	0.4291	0.4371	0.4456
Zimbabwe	0.2923	0.3001	0.3084	0.3164	0.3243	0.3312	0.3374	0.3423	0.3472	0.3519	0.3571	0.3637	0.3703	0.3773	0.3835	0.3903	0.3969	0.4033	0.4121	0.4224
Eastern Cape	0.4350	0.4417	0.4481	0.4550	0.4619	0.4679	0.4735	0.4784	0.4834	0.4887	0.4949	0.5010	0.5064	0.5112	0.5163	0.5206	0.5244	0.5285	0.5333	0.5384
Free State	0.4965	0.5017	0.5068	0.5122	0.5177	0.5227	0.5274	0.5317	0.5361	0.5407	0.5459	0.5509	0.5556	0.5599	0.5644	0.5685	0.5723	0.5764	0.5812	0.5864
Gauteng	0.6185	0.6225	0.6263	0.6304	0.6345	0.6382	0.6418	0.6452	0.6487	0.6523	0.6562	0.6601	0.6637	0.6672	0.6708	0.6742	0.6773	0.6806	0.6844	0.6883
KwaZulu-Natal	0.4668	0.4726	0.4783	0.4844	0.4905	0.4960	0.5011	0.5059	0.5107	0.5158	0.5215	0.5271	0.5321	0.5369	0.5418	0.5463	0.5506	0.5550	0.5603	0.5658
Limpopo	0.3845	0.3909	0.3972	0.4039	0.4109	0.4171	0.4230	0.4285	0.4342	0.4402	0.4472	0.4541	0.4606	0.4666	0.4730	0.4789	0.4844	0.4903	0.4970	0.5041
Mpumalanga	0.4367	0.4425	0.4483	0.4545	0.4608	0.4666	0.4723	0.4777	0.4832	0.4891	0.4954	0.5018	0.5078	0.5136	0.5197	0.5254	0.5310	0.5368	0.5434	0.5502
North-West	0.4676	0.4733	0.4789	0.4849	0.4910	0.4965	0.5018	0.5067	0.5118	0.5171	0.5230	0.5289	0.5343	0.5395	0.5449	0.5499	0.5546	0.5595	0.5651	0.5707
Northern Cape	0.4973	0.5023	0.5073	0.5125	0.5178	0.5226	0.5272	0.5314	0.5358	0.5403	0.5452	0.5502	0.5547	0.5590	0.5635	0.5677	0.5716	0.5756	0.5804	0.5852
Western Cape	0.6213	0.6248	0.6282	0.6318	0.6354	0.6386	0.6417	0.6444	0.6472	0.6502	0.6535	0.6567	0.6597	0.6624	0.6653	0.6677	0.6700	0.6724	0.6753	0.6785
Western Sub-Saharan Africa	0.1971	0.2024	0.2079	0.2135	0.2195	0.2253	0.2311	0.2373	0.2432	0.2488	0.2540	0.2587	0.2634	0.2678	0.2717	0.2762	0.2790	0.2807	0.2825	0.2846
Benin	0.1417	0.1448	0.1479	0.1511	0.1543	0.1569	0.1596	0.1623	0.1650	0.1678	0.1711	0.1746	0.1782	0.1823	0.1868	0.1919	0.1970	0.2019	0.2070	0.2118
Burkina Faso	0.0815	0.0832	0.0855	0.0876	0.0898	0.0918	0.0938	0.0959	0.0985	0.1011	0.1035	0.1057	0.1076	0.1093	0.1108	0.1128	0.1152	0.1173	0.1198	0.1226
Cameroon	0.2195	0.2252	0.2311	0.2371	0.2433	0.2501	0.2564	0.2630	0.2701	0.2767	0.2827	0.2892	0.2958	0.3024	0.3091	0.3156	0.3218	0.3272	0.3320	0.3367
Cape Verde	0.1902	0.1952	0.2000	0.2048	0.2097	0.2141	0.2187	0.2233	0.2286	0.2343	0.2411	0.2484	0.2557	0.2633	0.2710	0.2789	0.2867	0.2944	0.3021	0.3099
Chad	0.1344	0.1371	0.1396	0.1418	0.1442	0.1471	0.1499	0.1526	0.1553	0.1568	0.1580	0.1587	0.1595	0.1613	0.1633	0.1659	0.1688	0.1718	0.1754	0.1792
Cote d'Ivoire	0.1788	0.1838	0.1888	0.1937	0.1987	0.2037	0.2089	0.2139	0.2186	0.2228	0.2248	0.2259	0.2271	0.2283	0.2297	0.2338	0.2408	0.2486	0.2575	0.2675
The Gambia	0.1604	0.1641	0.1681	0.1723	0.1769	0.1816	0.1860	0.1907	0.1955	0.2004	0.2053	0.2100	0.2155	0.2211	0.2264	0.2317	0.2371	0.2421	0.2468	0.2513
Ghana	0.2523	0.2596	0.2661	0.2727	0.2796	0.2854	0.2905	0.2956	0.3006	0.3049	0.3089	0.3126	0.3153	0.3173	0.3196	0.3226	0.3262	0.3304	0.3375	0.3468
Guinea	0.1479	0.1506	0.1535	0.1565	0.1597	0.1631	0.1668	0.1703	0.1740	0.1775	0.1810	0.1843	0.1876	0.1908	0.1938	0.1970	0.2001	0.2032	0.2066	0.2101
Guinea-Bissau	0.1409	0.1444	0.1480	0.1515	0.1551	0.1588	0.1627	0.1658	0.1695	0.1732	0.1761	0.1788	0.1818	0.1845	0.1874	0.1901	0.1933	0.1967	0.2003	0.2042
Liberia	0.1643	0.1681	0.1718	0.1755	0.1791	0.1823	0.1857	0.1889	0.1922	0.1954	0.1983	0.2007	0.2031	0.2053	0.2073	0.2093	0.2112	0.2132	0.2157	0.2186
Mali	0.0913	0.0935	0.0956	0.0975	0.0993	0.1015	0.1040	0.1067	0.1091	0.1119	0.1143	0.1165	0.1184	0.1203	0.1220	0.1242	0.1265	0.1290	0.1315	0.1346
Mauritania	0.2215	0.2270	0.2323	0.2373	0.2424	0.2476	0.2530	0.2584	0.2633	0.2685	0.2735	0.2786	0.2834	0.2882	0.2927	0.2973	0.3021	0.3069	0.3118	0.3169
Niger	0.0988	0.1010	0.1030	0.1046	0.1064	0.1079	0.1097	0.1117	0.1137	0.1162	0.1186	0.1208	0.1232	0.1256	0.1271	0.1288	0.1310	0.1328	0.1346	0.1362
Nigeria	0.2175	0.2239	0.2304	0.2373	0.2447	0.2519	0.2592	0.2671	0.2748	0.2822	0.2893	0.2960	0.3026	0.3088	0.3143	0.3202	0.3229	0.3233	0.3228	0.3222
Sao Tome and Principe	0.1842	0.1890	0.1939	0.1988	0.2038	0.2089	0.2140	0.2198	0.2254	0.2316	0.2372	0.2427	0.2484	0.2540	0.2592	0.2647	0.2699	0.2746	0.2792	0.2841
Senegal	0.1645	0.1676	0.1709	0.1739	0.1770	0.1803	0.1837	0.1870	0.1901	0.1936	0.1968	0.2001	0.2039	0.2077	0.2115	0.2155	0.2201	0.2251	0.2302	0.2353
Sierra Leone	0.1541	0.1578	0.1614	0.1649	0.1684	0.1717	0.1747	0.1776	0.1804	0.1833	0.1860	0.1889	0.1920	0.1949	0.1980	0.2008	0.2037	0.2069	0.2101	0.2136
Togo	0.1685	0.1727	0.1767	0.1804	0.1848	0.1886	0.1923	0.1962	0.2002	0.2041	0.2081	0.2119	0.2154	0.2184	0.2216	0.2248	0.2284	0.2316	0.2360	0.2416

Appendix Table 5B. SDI Values by location, 1990-2016

Location	1990	1991	1992	1993	1994	1995	1996	1997	1998	1999	2000	2001	2002	2003	2004	2005	2006	2007	2008	2009	2010	2011	2012	2013	2014	2015	2016
Global	0.5746	0.5804	0.5860	0.5911	0.5957	0.6003	0.6048	0.6093	0.6135	0.6176	0.6219	0.6263	0.6304	0.6345	0.6388	0.6432	0.6477	0.6524	0.6572	0.6616	0.6662	0.6710	0.6759	0.6809	0.6860	0.6913	0.6958
Southeast Asia, East Asia, and Oceania	0.5209	0.5297	0.5387	0.5476	0.5563	0.5648	0.5730	0.5809	0.5874	0.5936	0.5999	0.6061	0.6125	0.6191	0.6261	0.6334	0.6414	0.6498	0.6580	0.6662	0.6745	0.6828	0.6908	0.6987	0.7058	0.7122	0.7185
East Asia	0.5238	0.5331	0.5428	0.5524	0.5618	0.5710	0.5796	0.5875	0.5943	0.6010	0.6077	0.6144	0.6214	0.6286	0.6362	0.6441	0.6526	0.6614	0.6697	0.6778	0.6858	0.6938	0.7014	0.7086	0.7155	0.7221	0.7288
China	0.5121	0.5214	0.5315	0.5416	0.5517	0.5616	0.5704	0.5782	0.5854	0.5925	0.5996	0.6067	0.6142	0.6218	0.6299	0.6382	0.6472	0.6565	0.6653	0.6738	0.6822	0.6905	0.6984	0.7059	0.7130	0.7198	0.7266
North Korea	0.5047	0.5076	0.5096	0.5114	0.5099	0.5064	0.5013	0.4953	0.4892	0.4834	0.4780	0.4735	0.4703	0.4685	0.4682	0.4688	0.4700	0.4714	0.4729	0.4742	0.4760	0.4783	0.4811	0.4842	0.4875	0.4910	0.4936
Taiwan (Province of China)	0.7337	0.7425	0.7492	0.7556	0.7623	0.7684	0.7750	0.7880	0.7983	0.7998	0.8082	0.8151	0.8197	0.8242	0.8286	0.8330	0.8373	0.8417	0.8455	0.8488	0.8528	0.8568	0.8605	0.8641	0.8677	0.8713	0.8751
Hong Kong Special Administrative Region of China	0.7035	0.7084	0.7133	0.7181	0.7227	0.7267	0.7305	0.7344	0.7371	0.7397	0.7428	0.7459	0.7492	0.7525	0.7563	0.7607	0.7654	0.7704	0.7751	0.7789	0.7829	0.7870	0.7908	0.7945	0.7981	0.8014	0.8053
Macao Special Administrative Region of China	0.7004	0.7054	0.7111	0.7164	0.7214	0.7251	0.7281	0.7306	0.7323	0.7337	0.7354	0.7383	0.7420	0.7468	0.7539	0.7609	0.7681	0.7761	0.7837	0.7890	0.7928	0.7961	0.7993	0.8024	0.8055	0.8085	0.8113
China (without Hong Kong and Macao)	0.5058	0.5152	0.5254	0.5358	0.5461	0.5563	0.5654	0.5735	0.5810	0.5883	0.5956	0.6030	0.6107	0.6187	0.6269	0.6354	0.6446	0.6541	0.6630	0.6717	0.6803	0.6887	0.6967	0.7043	0.7115	0.7184	0.7253
Anhui	0.4458	0.4547	0.4642	0.4738	0.4834	0.4929	0.5017	0.5101	0.5177	0.5249	0.5316	0.5381	0.5448	0.5517	0.5591	0.5670	0.5758	0.5851	0.5940	0.6029	0.6111	0.6187	0.6258	0.6326	0.6390	0.6451	0.6514
Beijing	0.6707	0.6763	0.6824	0.6888	0.6955	0.7024	0.7090	0.7153	0.7209	0.7263	0.7317	0.7375	0.7436	0.7500	0.7568	0.7640	0.7717	0.7797	0.7873	0.7947	0.8020	0.8092	0.8162	0.8229	0.8292	0.8354	0.8415
Chongqing	0.4740	0.4845	0.4957	0.5072	0.5189	0.5300	0.5393	0.5486	0.5571	0.5654	0.5736	0.5819	0.5906	0.5989	0.6073	0.6159	0.6250	0.6346	0.6434	0.6519	0.6602	0.6681	0.6755	0.6822	0.6885	0.6945	0.7004
Fujian	0.4797	0.4917	0.5045	0.5177	0.5309	0.5439	0.5547	0.5642	0.5727	0.5812	0.5895	0.5977	0.6063	0.6148	0.6234	0.6324	0.6420	0.6523	0.6618	0.6711	0.6802	0.6892	0.6976	0.7055	0.7131	0.7203	0.7274
Gansu	0.4384	0.4467	0.4556	0.4646	0.4734	0.4819	0.4901	0.4982	0.5058	0.5132	0.5201	0.5263	0.5327	0.5392	0.5459	0.5528	0.5602	0.5680	0.5754	0.5827	0.5899	0.5970	0.6036	0.6099	0.6159	0.6216	0.6276
Guangdong	0.5364	0.5462	0.5567	0.5674	0.5780	0.5884	0.5980	0.6071	0.6154	0.6234	0.6317	0.6398	0.6484	0.6576	0.6673	0.6775	0.6884	0.6997	0.7103	0.7198	0.7286	0.7372	0.7454	0.7532	0.7606	0.7677	0.7747
Guangxi	0.4585	0.4684	0.4790	0.4899	0.5007	0.5115	0.5214	0.5308	0.5391	0.5471	0.5546	0.5616	0.5690	0.5768	0.5849	0.5936	0.6030	0.6129	0.6222	0.6317	0.6415	0.6514	0.6609	0.6703	0.6790	0.6861	0.6933
Guizhou	0.3760	0.3848	0.3940	0.4034	0.4128	0.4221	0.4306	0.4389	0.4465	0.4540	0.4616	0.4692	0.4773	0.4854	0.4939	0.5026	0.5120	0.5218	0.5310	0.5402	0.5495	0.5587	0.5673	0.5749	0.5812	0.5873	0.5937
Hainan	0.5108	0.5198	0.5294	0.5391	0.5486	0.5578	0.5664	0.5748	0.5824	0.5898	0.5969	0.6037	0.6108	0.6177	0.6246	0.6319	0.6398	0.6483	0.6564	0.6647	0.6724	0.6795	0.6863	0.6928	0.6989	0.7048	0.7108
Hebei	0.5087	0.5175	0.5270	0.5370	0.5469	0.5564	0.5649	0.5732	0.5809	0.5883	0.5958	0.6033	0.6111	0.6189	0.6270	0.6353	0.6442	0.6536	0.6623	0.6708	0.6792	0.6874	0.6951	0.7024	0.7094	0.7159	0.7225
Heilongjiang	0.5566	0.5656	0.5748	0.5816	0.5875	0.5936	0.5997	0.6058	0.6114	0.6171	0.6225	0.6280	0.6336	0.6388	0.6441	0.6494	0.6553	0.6616	0.6676	0.6735	0.6793	0.6850	0.6904	0.6954	0.7001	0.7045	0.7092
Henan	0.4714	0.4805	0.4909	0.5020	0.5133	0.5247	0.5352	0.5454	0.5541	0.5622	0.5702	0.5784	0.5868	0.5953	0.6041	0.6132	0.6230	0.6332	0.6426	0.6518	0.6608	0.6695	0.6777	0.6854	0.6926	0.6994	0.7062
Hubei	0.5039	0.5138	0.5243	0.5348	0.5450	0.5532	0.5612	0.5690	0.5761	0.5831	0.5900	0.5967	0.6036	0.6108	0.6184	0.6263	0.6346	0.6433	0.6514	0.6593	0.6669	0.6744	0.6815	0.6881	0.6943	0.7002	0.7063
Hunan	0.4854	0.4949	0.5052	0.5157	0.5259	0.5360	0.5455	0.5545	0.5617	0.5687	0.5756	0.5825	0.5897	0.5971	0.6048	0.6129	0.6215	0.6305	0.6389	0.6470	0.6551	0.6630	0.6704	0.6775	0.6842	0.6905	0.6969
Inner Mongolia	0.5118	0.5222	0.5336	0.5453	0.5563	0.5656	0.5748	0.5843	0.5930	0.6016	0.6099	0.6182	0.6266	0.6338	0.6408	0.6478	0.6556	0.6642	0.6722	0.6803	0.6882	0.6959	0.7031	0.7098	0.7161	0.7218	0.7276
Jiangsu	0.5389	0.5484	0.5569	0.5649	0.5733	0.5823	0.5909	0.5992	0.6067	0.6141	0.6214	0.6292	0.6374	0.6458	0.6547	0.6639	0.6737	0.6838	0.6932	0.7023	0.7112	0.7199	0.7281	0.7359	0.7432	0.7502	0.7571
Jiangxi	0.4485	0.4584	0.4689	0.4795	0.4898	0.4995	0.5088	0.5178	0.5261	0.5341	0.5419	0.5495	0.5572	0.5649	0.5727	0.5808	0.5896	0.5989	0.6068	0.6145	0.6221	0.6294	0.6364	0.6430	0.6493	0.6553	0.6616
Jilin	0.5370	0.5467	0.5569	0.5640	0.5713	0.5789	0.5863	0.5936	0.6003	0.6069	0.6133	0.6198	0.6263	0.6327	0.6392	0.6459	0.6533	0.6611	0.6684	0.6755	0.6826	0.6895	0.6960	0.7021	0.7078	0.7132	0.7187
Liaoning	0.5916	0.5966	0.6021	0.6081	0.6144	0.6209	0.6273	0.6336	0.6393	0.6450	0.6505	0.6562	0.6620	0.6678	0.6737	0.6798	0.6864	0.6935	0.7001	0.7065	0.7129	0.7191	0.7249	0.7305	0.7357	0.7406	0.7457
Ningxia	0.4599	0.4693	0.4792	0.4890	0.4983	0.5072	0.5154	0.5233	0.5306	0.5377	0.5449	0.5517	0.5589	0.5664	0.5743	0.5827	0.5917	0.6011	0.6096	0.6171	0.6244	0.6317	0.6386	0.6453	0.6517	0.6578	0.6642
Qinghai	0.4524	0.4625	0.4726	0.4823	0.4916	0.5005	0.5087	0.5165	0.5237	0.5307	0.5373	0.5436	0.5504	0.5575	0.5644	0.5712	0.5784	0.5859	0.5930	0.6000	0.6069	0.6138	0.6203	0.6266	0.6327	0.6387	0.6450
Shaanxi	0.4803	0.4902	0.5007	0.5116	0.5224	0.5320	0.5404	0.5487	0.5563	0.5638	0.5711	0.5787	0.5866	0.5943	0.6021	0.6102	0.6189	0.6282	0.6368	0.6453	0.6537	0.6619	0.6696	0.6768	0.6836	0.6900	0.6964
Shandong	0.5155	0.5248	0.5350	0.5440	0.5530	0.5627	0.5718	0.5806	0.5886	0.5963	0.6040	0.6118	0.6201	0.6288	0.6379	0.6474	0.6575	0.6679	0.6776	0.6868	0.6960	0.7049	0.7132	0.7211	0.7285	0.7356	0.7425
Shanghai	0.6840	0.6887	0.6936	0.6986	0.7037	0.7090	0.7142	0.7189	0.7233	0.7275	0.7317	0.7364	0.7414	0.7468	0.7527	0.7589	0.7653	0.7720	0.7784	0.7846	0.7907	0.7969	0.8029	0.8088	0.8145	0.8201	0.8260
Shanxi	0.5109	0.5203	0.5301	0.5401	0.5499	0.5594	0.5684	0.5771	0.5850	0.5927	0.5998	0.6067	0.6137	0.6206	0.6277	0.6349	0.6427	0.6510	0.6587	0.6662	0.6737	0.6810	0.6878	0.6943	0.7005	0.7063	0.7123
Sichuan	0.4635	0.4724	0.4822	0.4923	0.5024	0.5114	0.5198	0.5278	0.5351	0.5421	0.5491	0.5560	0.5632	0.5711	0.5796	0.5885	0.5980	0.6077	0.6166	0.6252	0.6336	0.6419	0.6496	0.6568	0.6637	0.6702	0.6768
Tianjin	0.6356	0.6410	0.6469	0.6531	0.6595	0.6662	0.6727	0.6790	0.6848	0.6904	0.6960	0.7022	0.7087	0.7153	0.7221	0.7293	0.7369	0.7450	0.7527	0.7603	0.7677	0.7751	0.7820	0.7887	0.7951	0.8011	0.8073
Tibet	0.4060	0.4137	0.4216	0.4292	0.4366	0.4437	0.4503	0.4564	0.4622	0.4681	0.4744	0.4804	0.4874	0.4950	0.5031	0.5115	0.5187	0.5257	0.5322	0.5384	0.5445	0.5504	0.5558	0.5610	0.5661	0.5712	0.5769
Xinjiang	0.4864	0.4988	0.5115	0.5241	0.5362	0.5475	0.5582	0.5686	0.5783	0.5881	0.5972	0.6057	0.6142	0.6218	0.6290	0.6362	0.6440	0.6524	0.6605	0.6680	0.6755	0.6828	0.6895	0.6959	0.7018	0.7074	0.7132
Yunnan	0.4117	0.4217	0.4323	0.4430	0.4534	0.4637	0.4732	0.4825	0.4909	0.4992	0.5074	0.5152	0.5235	0.5319	0.5408	0.5500	0.5599	0.5702	0.5792	0.5874	0.5956	0.6035	0.6111	0.6184	0.6253	0.6320	0.6388
Zhejiang	0.5240	0.5336	0.5435	0.5520	0.5609	0.5703	0.5794	0.5882	0.5962	0.6040	0.6118	0.6199	0.6285	0.6374	0.6465	0.6560	0.6661	0.6765	0.6861	0.6954	0.7046	0.7136	0.7221	0.7302	0.7379	0.7453	0.7526
Southeast Asia	0.4991	0.5069	0.5147	0.5226	0.5306	0.5386	0.5467	0.5545	0.5604	0.5658	0.5713	0.5768	0.5822	0.5875	0.5929	0.5984	0.6041	0.6100	0.6163	0.6225	0.6293	0.6364	0.6437	0.6511	0.6585	0.6659	0.6723
Cambodia	0.3009	0.3062	0.3116	0.3165	0.3210	0.3261	0.3310	0.3364	0.3420	0.3491	0.3567	0.3648	0.3733	0.3821	0.3914	0.4015	0.4119	0.4226	0.4333	0.4429	0.4520	0.4611	0.4700	0.4788	0.4873	0.4957	0.5029
Indonesia	0.5013	0.5096	0.5177	0.5258	0.5336	0.5413	0.5491	0.5567	0.5623	0.5673	0.5721	0.5769	0.5816	0.5862	0.5910	0.5958	0.6010	0.6066	0.6131	0.6199	0.6271	0.6351	0.6433	0.6518	0.6603	0.6688	0.6762
Laos	0.3032	0.3089	0.3147	0.3208	0.3274	0.3353	0.3443	0.3537	0.3629	0.3723	0.3815	0.3908	0.4000	0.4091	0.4181	0.4261	0.4335	0.4411	0.4484	0.4559	0.4634	0.4713	0.4796	0.4878	0.4961	0.5045	0.5118
Malaysia	0.5636	0.5692	0.5745	0.5831	0.5933	0.6025	0.6132	0.6254	0.6324	0.6371	0.6476	0.6614	0.6719	0.6791	0.6866	0.6945	0.7012	0.7065	0.7119	0.7181	0.7260	0.7341	0.7423	0.7503	0.7582	0.7660	0.7727
Maldives	0.4331	0.4420	0.4509	0.4600	0.4693	0.4790	0.4894	0.5002	0.5113	0.5224	0.5336	0.5445	0.5547	0.5650	0.5755	0.5834	0.5923	0.6015	0.6115	0.6208	0.6305	0.6409	0.6510	0.6608	0.6704	0.6795	0.6871
Myanmar	0.3491	0.3520	0.3558	0.3605	0.3658	0.3721	0.3790	0.3861	0.3934	0.4014	0.4106	0.4203	0.4303	0.4412	0.4526	0.4649	0.4775	0.4901	0.5018	0.5129	0.5244	0.5357	0.5472	0.5583	0.5694	0.5803	0.5898
Philippines	0.5198	0.5246	0.5292	0.5337	0.5386	0.5436	0.5487	0.5536	0.5579	0.5618	0.5658	0.5697	0.5733	0.5771	0.5810	0.5850	0.5890	0.5935	0.5982	0.6028	0.6080	0.6134	0.6190	0.6249	0.6308	0.6368	0.6420
Sri Lanka	0.5124	0.5190	0.5257	0.5331	0.5413	0.5500	0.5592	0.5682	0.5757	0.5806	0.5844	0.5878	0.5925	0.5983	0.6041	0.6093	0.6145	0.6206	0.6280	0.6364	0.6457	0.6555	0.6657	0.6756	0.6853	0.6947	0.7030
Thailand	0.5445	0.5537	0.5629	0.5721	0.5814	0.5908	0.6001	0.6082	0.6148	0.6213	0.6278	0.6347	0.6418	0.6492	0.6568	0.6645	0.6722	0.6799	0.6859	0.6912	0.6970	0.7028	0.7088	0.7142	0.7191	0.7239	0.7287
Timor-Leste	0.3232	0.3325	0.3417	0.3509	0.3598	0.3683	0.3760	0.3827	0.3886	0.3896	0.3915	0.3943	0.3961	0.3949	0.4041	0.4157	0.4289	0.4368	0.4444	0.4524	0.4620	0.4746	0.4885	0.5020	0.5140	0.5252	0.5324
Vietnam	0.4393	0.4485	0.4579	0.4672	0.4763	0.4857	0.4951	0.5046	0.5136	0.5222	0.5304	0.5382	0.5456	0.5524	0.5591	0.5659	0.5726	0.5793	0.5859	0.5923	0.5991	0.6059	0.6128	0.6194	0.6261	0.6329	0.6390
Mauritius	0.6101	0.6156	0.6215	0.6297	0.6390	0.6469	0.6544	0.6624	0.6673	0.6715	0.6784	0.6838	0.6882	0.6930	0.6977	0.7035	0.7096	0.7155	0.7219	0.7264	0.7303	0.7345	0.7388	0.7430	0.7472	0.7515	0.7559
Seychelles	0.6028	0.6104	0.6180	0.6253	0.6343	0.6441	0.6537	0.6633	0.6709	0.6767	0.6819	0.6868	0.6907	0.6932	0.6944	0.6959	0.6971	0.6982	0.6997	0.7029	0.7056	0.7075	0.7108	0.7163	0.7227	0.7296	0.7358
Aceh	0.5373	0.5474	0.5568	0.5663	0.5747	0.5829	0.5909	0.5988	0.6045	0.6086	0.6120	0.6138	0.6157	0.6179	0.6198	0.6215	0.6249	0.6286	0.6328	0.6369	0.6416	0.6468	0.6529	0.6598	0.6670	0.6744	0.6809
Sumatera Utara	0.4993	0.5060	0.5131	0.5196	0.5266	0.5332	0.5397	0.5470	0.5521	0.5579	0.5624	0.5672	0.5722	0.5775	0.5826	0.5877	0.5932	0.5997	0.6073	0.6158	0.6251	0.6349	0.6446	0.6540	0.6632	0.6721	0.6797
Sumatera Barat	0.4831	0.4908	0.4978	0.5055	0.5129	0.5195	0.5259	0.5318	0.5364	0.5414	0.5457	0.5509	0.5568	0.5625	0.5683	0.5741	0.5803	0.5870	0.5944	0.6024	0.6112	0.6203	0.6293	0.6383	0.6471	0.6558	0.6633
Riau	0.5666	0.5767	0.5860	0.5945	0.6018	0.6087	0.6156	0.6225	0.6284	0.6324	0.6391	0.6455	0.6520	0.6563	0.6609	0.6653	0.6698	0.6750	0.6815	0.6881	0.6947	0.7024	0.7108	0.7195	0.7283	0.7369	0.7443
Jambi	0.4564	0.4652	0.4738	0.4828	0.4917	0.5007	0.5099	0.5189	0.5256	0.5319	0.5370	0.5424	0.5486	0.5550	0.5617	0.5684	0.5749	0.5826	0.5916	0.6002	0.6096	0.6198	0.6300	0.6399	0.6494	0.6586	0.6666
Sumatera Selatan	0.5125	0.5215	0.5301	0.5372	0.5436	0.5501	0.5567	0.5632	0.5686	0.5722	0.5752	0.5785	0.5812	0.5842	0.5874	0.5916	0.5964	0.6018	0.6084	0.6147	0.6216	0.6293	0.6376	0.6462	0.6547	0.6632	0.6706
Bengkulu	0.4522	0.4616	0.4708	0.4796	0.4888	0.4979	0.5066	0.5153	0.5209	0.5259	0.5298	0.5336	0.5377	0.5426	0.5478	0.5542	0.5606	0.5675	0.5747	0.5818	0.5893	0.5981	0.6071	0.6161	0.6249	0.6335	0.6411

Appendix Table 5B. SDI Values by location, 1990-2016																											
**Location**	**1990**	**1991**	**1992**	**1993**	**1994**	**1995**	**1996**	**1997**	**1998**	**1999**	**2000**	**2001**	**2002**	**2003**	**2004**	**2005**	**2006**	**2007**	**2008**	**2009**	**2010**	**2011**	**2012**	**2013**	**2014**	**2015**	**2016**
Lampung	0.4369	0.4443	0.4528	0.4603	0.4687	0.4785	0.4886	0.4983	0.5074	0.5163	0.5221	0.5269	0.5315	0.5360	0.5404	0.5441	0.5487	0.5551	0.5623	0.5711	0.5813	0.5922	0.6027	0.6131	0.6231	0.6327	0.6411
Bangka Belitung	0.4915	0.4995	0.5075	0.5143	0.5210	0.5278	0.5348	0.5420	0.5475	0.5530	0.5569	0.5615	0.5667	0.5723	0.5780	0.5838	0.5894	0.5952	0.6016	0.6083	0.6152	0.6228	0.6307	0.6390	0.6474	0.6558	0.6632
Kepulauan Riau	0.5808	0.5934	0.6058	0.6168	0.6264	0.6351	0.6440	0.6528	0.6592	0.6646	0.6686	0.6719	0.6747	0.6775	0.6808	0.6841	0.6878	0.6924	0.6978	0.7042	0.7111	0.7183	0.7262	0.7343	0.7424	0.7507	0.7579
Kalimantan Utara	0.5884	0.5993	0.6104	0.6202	0.6299	0.6395	0.6491	0.6577	0.6661	0.6725	0.6788	0.6843	0.6888	0.6933	0.6980	0.7036	0.7087	0.7131	0.7201	0.7258	0.7314	0.7386	0.7459	0.7536	0.7614	0.7690	0.7754
Jakarta	0.6545	0.6626	0.6699	0.6800	0.6889	0.6973	0.7057	0.7142	0.7196	0.7246	0.7298	0.7350	0.7400	0.7450	0.7498	0.7543	0.7590	0.7639	0.7693	0.7753	0.7817	0.7885	0.7955	0.8028	0.8102	0.8177	0.8243
Jawa Barat	0.4959	0.5040	0.5121	0.5203	0.5290	0.5376	0.5462	0.5543	0.5596	0.5633	0.5663	0.5689	0.5715	0.5746	0.5777	0.5819	0.5873	0.5932	0.6001	0.6075	0.6153	0.6232	0.6311	0.6393	0.6475	0.6556	0.6627
Jawa Tengah	0.4574	0.4662	0.4757	0.4835	0.4915	0.4999	0.5081	0.5162	0.5211	0.5260	0.5292	0.5327	0.5363	0.5404	0.5446	0.5492	0.5549	0.5608	0.5675	0.5747	0.5824	0.5906	0.5990	0.6076	0.6162	0.6246	0.6320
Yogyakarta	0.4990	0.5084	0.5174	0.5304	0.5429	0.5537	0.5635	0.5719	0.5761	0.5804	0.5837	0.5873	0.5912	0.5949	0.5989	0.6026	0.6071	0.6122	0.6179	0.6243	0.6312	0.6388	0.6466	0.6549	0.6634	0.6717	0.6793
Jawa Timur	0.4818	0.4902	0.4982	0.5060	0.5138	0.5216	0.5298	0.5382	0.5446	0.5503	0.5561	0.5619	0.5675	0.5730	0.5786	0.5840	0.5899	0.5962	0.6026	0.6095	0.6170	0.6250	0.6334	0.6421	0.6508	0.6595	0.6671
Banten	0.4952	0.5041	0.5131	0.5210	0.5287	0.5364	0.5444	0.5532	0.5606	0.5678	0.5727	0.5769	0.5813	0.5855	0.5896	0.5934	0.5976	0.6021	0.6069	0.6124	0.6182	0.6264	0.6349	0.6436	0.6523	0.6609	0.6686
Bali	0.5059	0.5154	0.5243	0.5343	0.5437	0.5524	0.5611	0.5695	0.5747	0.5788	0.5819	0.5851	0.5888	0.5924	0.5963	0.6003	0.6043	0.6089	0.6143	0.6209	0.6280	0.6357	0.6442	0.6530	0.6616	0.6701	0.6776
Nusa Tenggara Barat	0.3744	0.3827	0.3922	0.4027	0.4133	0.4240	0.4343	0.4437	0.4517	0.4582	0.4660	0.4750	0.4832	0.4900	0.4979	0.5049	0.5112	0.5183	0.5239	0.5319	0.5389	0.5468	0.5540	0.5617	0.5694	0.5772	0.5841
Nusa Tenggara Timur	0.3805	0.3878	0.3952	0.4021	0.4087	0.4146	0.4201	0.4263	0.4268	0.4270	0.4289	0.4320	0.4356	0.4393	0.4434	0.4471	0.4512	0.4565	0.4628	0.4702	0.4786	0.4878	0.4972	0.5067	0.5161	0.5254	0.5336
Kalimantan Barat	0.4287	0.4393	0.4499	0.4611	0.4723	0.4832	0.4940	0.5051	0.5130	0.5199	0.5252	0.5298	0.5342	0.5382	0.5424	0.5462	0.5500	0.5545	0.5593	0.5649	0.5712	0.5781	0.5857	0.5938	0.6020	0.6102	0.6175
Kalimantan Tengah	0.5057	0.5145	0.5233	0.5342	0.5445	0.5541	0.5638	0.5728	0.5787	0.5832	0.5853	0.5861	0.5869	0.5877	0.5896	0.5915	0.5942	0.5982	0.6033	0.6096	0.6166	0.6246	0.6331	0.6418	0.6504	0.6590	0.6665
Kalimantan Selatan	0.4945	0.5025	0.5101	0.5186	0.5266	0.5343	0.5421	0.5490	0.5535	0.5580	0.5610	0.5635	0.5667	0.5702	0.5738	0.5771	0.5803	0.5842	0.5888	0.5944	0.6010	0.6086	0.6165	0.6249	0.6334	0.6418	0.6493
Kalimantan Timur	0.5977	0.6075	0.6168	0.6246	0.6324	0.6398	0.6471	0.6539	0.6609	0.6667	0.6733	0.6800	0.6856	0.6907	0.6959	0.7016	0.7067	0.7108	0.7169	0.7217	0.7263	0.7326	0.7394	0.7470	0.7549	0.7630	0.7700
Sulawesi Utara	0.5244	0.5318	0.5389	0.5477	0.5559	0.5639	0.5729	0.5811	0.5879	0.5908	0.5930	0.5946	0.5959	0.5972	0.5988	0.6009	0.6034	0.6071	0.6120	0.6187	0.6259	0.6337	0.6419	0.6505	0.6591	0.6675	0.6750
Sulawesi Tengah	0.4524	0.4601	0.4675	0.4761	0.4848	0.4931	0.5016	0.5090	0.5165	0.5227	0.5274	0.5326	0.5377	0.5424	0.5469	0.5510	0.5548	0.5594	0.5651	0.5717	0.5786	0.5876	0.5970	0.6064	0.6157	0.6249	0.6329
Sulawesi Selatan	0.4558	0.4648	0.4736	0.4815	0.4891	0.4965	0.5037	0.5104	0.5158	0.5204	0.5251	0.5302	0.5353	0.5403	0.5457	0.5498	0.5545	0.5598	0.5664	0.5744	0.5835	0.5932	0.6033	0.6133	0.6229	0.6322	0.6403
Sulawesi Tenggara	0.4324	0.4415	0.4501	0.4575	0.4649	0.4726	0.4804	0.4872	0.4938	0.4990	0.5033	0.5080	0.5133	0.5187	0.5245	0.5308	0.5373	0.5443	0.5523	0.5613	0.5702	0.5796	0.5891	0.5987	0.6081	0.6173	0.6253
Gorontalo	0.4068	0.4151	0.4236	0.4301	0.4367	0.4435	0.4504	0.4573	0.4617	0.4657	0.4672	0.4698	0.4734	0.4778	0.4825	0.4878	0.4934	0.4999	0.5075	0.5168	0.5263	0.5361	0.5458	0.5551	0.5642	0.5730	0.5808
Sulawesi Barat	0.4139	0.4221	0.4300	0.4361	0.4418	0.4472	0.4528	0.4583	0.4618	0.4656	0.4688	0.4728	0.4773	0.4814	0.4854	0.4891	0.4931	0.4985	0.5066	0.5156	0.5254	0.5362	0.5471	0.5579	0.5683	0.5785	0.5873
Maluku	0.5189	0.5256	0.5325	0.5389	0.5449	0.5506	0.5565	0.5624	0.5656	0.5668	0.5635	0.5587	0.5536	0.5481	0.5423	0.5362	0.5306	0.5273	0.5265	0.5291	0.5341	0.5413	0.5508	0.5610	0.5711	0.5809	0.5899
Maluku Utara	0.4577	0.4665	0.4758	0.4835	0.4909	0.4983	0.5060	0.5135	0.5184	0.5225	0.5241	0.5240	0.5228	0.5209	0.5183	0.5147	0.5108	0.5079	0.5072	0.5099	0.5151	0.5222	0.5313	0.5414	0.5516	0.5616	0.5708
Papua Barat	0.5246	0.5338	0.5428	0.5505	0.5579	0.5649	0.5716	0.5779	0.5817	0.5848	0.5869	0.5887	0.5902	0.5920	0.5940	0.5960	0.5981	0.6012	0.6070	0.6154	0.6261	0.6425	0.6574	0.6709	0.6833	0.6951	0.7057
Papua	0.5102	0.5162	0.5218	0.5286	0.5341	0.5401	0.5463	0.5517	0.5583	0.5623	0.5653	0.5688	0.5708	0.5729	0.5739	0.5775	0.5801	0.5826	0.5837	0.5854	0.5877	0.5891	0.5915	0.5952	0.5999	0.6053	0.6101
Oceania	0.3832	0.3882	0.3930	0.3978	0.4027	0.4073	0.4119	0.4161	0.4203	0.4245	0.4285	0.4325	0.4363	0.4399	0.4434	0.4469	0.4502	0.4537	0.4573	0.4609	0.4645	0.4682	0.4722	0.4762	0.4805	0.4851	0.4891
Fiji	0.5996	0.6065	0.6136	0.6203	0.6270	0.6336	0.6402	0.6462	0.6518	0.6579	0.6634	0.6687	0.6739	0.6788	0.6839	0.6887	0.6939	0.6988	0.7035	0.7078	0.7123	0.7165	0.7205	0.7248	0.7291	0.7328	0.7365
Kiribati	0.3074	0.3116	0.3155	0.3195	0.3239	0.3283	0.3332	0.3384	0.3445	0.3503	0.3569	0.3634	0.3698	0.3764	0.3827	0.3891	0.3947	0.4005	0.4061	0.4115	0.4165	0.4212	0.4261	0.4313	0.4366	0.4421	0.4462
Marshall Islands	0.4655	0.4700	0.4731	0.4744	0.4747	0.4759	0.4768	0.4786	0.4820	0.4867	0.4924	0.4985	0.5045	0.5097	0.5141	0.5181	0.5221	0.5267	0.5314	0.5363	0.5415	0.5469	0.5528	0.5588	0.5647	0.5707	0.5752
Federated States of Micronesia	0.4342	0.4458	0.4559	0.4656	0.4747	0.4848	0.4947	0.5034	0.5122	0.5207	0.5299	0.5388	0.5470	0.5548	0.5615	0.5676	0.5734	0.5782	0.5823	0.5862	0.5903	0.5948	0.5993	0.6032	0.6066	0.6100	0.6125
Papua New Guinea	0.2956	0.2999	0.3043	0.3098	0.3154	0.3202	0.3253	0.3297	0.3338	0.3380	0.3417	0.3452	0.3484	0.3517	0.3550	0.3586	0.3623	0.3666	0.3713	0.3763	0.3815	0.3875	0.3936	0.3997	0.4060	0.4126	0.4184
Samoa	0.4509	0.4584	0.4654	0.4720	0.4771	0.4819	0.4872	0.4925	0.4977	0.5015	0.5043	0.5074	0.5107	0.5141	0.5175	0.5213	0.5251	0.5291	0.5334	0.5368	0.5395	0.5424	0.5454	0.5483	0.5526	0.5582	0.5623
Solomon Islands	0.3180	0.3232	0.3291	0.3347	0.3401	0.3461	0.3520	0.3575	0.3628	0.3678	0.3707	0.3726	0.3738	0.3753	0.3770	0.3794	0.3826	0.3871	0.3931	0.3984	0.4043	0.4113	0.4185	0.4255	0.4321	0.4387	0.4437
Tonga	0.4544	0.4607	0.4662	0.4713	0.4760	0.4809	0.4860	0.4909	0.4958	0.5009	0.5059	0.5111	0.5164	0.5216	0.5266	0.5316	0.5366	0.5412	0.5459	0.5506	0.5555	0.5606	0.5658	0.5708	0.5761	0.5815	0.5857
Vanuatu	0.3350	0.3412	0.3473	0.3528	0.3586	0.3642	0.3698	0.3756	0.3816	0.3874	0.3935	0.3994	0.4046	0.4099	0.4151	0.4202	0.4256	0.4311	0.4370	0.4429	0.4486	0.4543	0.4599	0.4653	0.4708	0.4758	0.4791
American Samoa	0.6168	0.6218	0.6265	0.6308	0.6349	0.6390	0.6433	0.6476	0.6518	0.6562	0.6608	0.6656	0.6708	0.6760	0.6821	0.6884	0.6945	0.6998	0.7052	0.7113	0.7164	0.7209	0.7259	0.7305	0.7349	0.7392	0.7420
Guam	0.7554	0.7606	0.7653	0.7697	0.7740	0.7781	0.7817	0.7852	0.7884	0.7913	0.7941	0.7965	0.7988	0.8006	0.8024	0.8042	0.8058	0.8073	0.8087	0.8102	0.8118	0.8133	0.8151	0.8169	0.8189	0.8209	0.8225
Northern Mariana Islands	0.7051	0.7126	0.7200	0.7268	0.7330	0.7389	0.7445	0.7501	0.7554	0.7608	0.7662	0.7715	0.7769	0.7818	0.7865	0.7899	0.7923	0.7937	0.7949	0.7948	0.7946	0.7936	0.7930	0.7930	0.7938	0.7955	0.7972
Central Europe, Eastern Europe, and Central Asia	0.7393	0.7456	0.7510	0.7538	0.7553	0.7569	0.7577	0.7582	0.7580	0.7579	0.7593	0.7617	0.7649	0.7689	0.7734	0.7782	0.7824	0.7859	0.7900	0.7934	0.7975	0.8011	0.8038	0.8063	0.8090	0.8122	0.8156
Central Asia	0.6239	0.6298	0.6346	0.6384	0.6408	0.6421	0.6429	0.6434	0.6433	0.6435	0.6445	0.6467	0.6500	0.6545	0.6597	0.6656	0.6720	0.6788	0.6855	0.6910	0.6967	0.7026	0.7081	0.7136	0.7191	0.7242	0.7286
Armenia	0.6443	0.6483	0.6481	0.6468	0.6448	0.6431	0.6421	0.6413	0.6417	0.6431	0.6461	0.6514	0.6590	0.6676	0.6763	0.6855	0.6951	0.7049	0.7141	0.7195	0.7245	0.7296	0.7348	0.7398	0.7450	0.7502	0.7547
Azerbaijan	0.6678	0.6731	0.6763	0.6781	0.6783	0.6766	0.6738	0.6704	0.6667	0.6636	0.6621	0.6623	0.6642	0.6674	0.6711	0.6778	0.6881	0.7002	0.7119	0.7217	0.7310	0.7395	0.7467	0.7531	0.7585	0.7632	0.7668
Georgia	0.7197	0.7233	0.7223	0.7184	0.7126	0.7054	0.6977	0.6895	0.6819	0.6767	0.6745	0.6759	0.6793	0.6843	0.6895	0.6952	0.7012	0.7081	0.7141	0.7186	0.7227	0.7270	0.7314	0.7360	0.7410	0.7461	0.7513
Kazakhstan	0.6812	0.6866	0.6920	0.6962	0.6992	0.7011	0.7026	0.7038	0.7042	0.7042	0.7047	0.7062	0.7081	0.7101	0.7125	0.7150	0.7179	0.7211	0.7248	0.7278	0.7314	0.7356	0.7396	0.7438	0.7481	0.7526	0.7567
Kyrgyzstan	0.6036	0.6098	0.6144	0.6178	0.6190	0.6183	0.6169	0.6151	0.6123	0.6093	0.6069	0.6056	0.6047	0.6050	0.6061	0.6063	0.6062	0.6068	0.6082	0.6093	0.6099	0.6121	0.6140	0.6179	0.6224	0.6271	0.6315
Mongolia	0.5567	0.5728	0.5864	0.5966	0.6041	0.6106	0.6152	0.6193	0.6230	0.6268	0.6308	0.6350	0.6391	0.6430	0.6470	0.6503	0.6535	0.6564	0.6590	0.6604	0.6619	0.6643	0.6670	0.6702	0.6741	0.6783	0.6828
Tajikistan	0.5193	0.5299	0.5369	0.5419	0.5433	0.5418	0.5370	0.5316	0.5255	0.5193	0.5145	0.5128	0.5152	0.5202	0.5273	0.5345	0.5415	0.5475	0.5525	0.5562	0.5591	0.5612	0.5638	0.5672	0.5707	0.5742	0.5775
Turkmenistan	0.5769	0.5837	0.5898	0.5979	0.6051	0.6119	0.6185	0.6230	0.6267	0.6304	0.6358	0.6413	0.6471	0.6533	0.6598	0.6677	0.6760	0.6842	0.6922	0.7006	0.7088	0.7174	0.7259	0.7346	0.7433	0.7516	0.7586
Uzbekistan	0.5436	0.5512	0.5577	0.5650	0.5712	0.5769	0.5821	0.5868	0.5907	0.5944	0.5973	0.6002	0.6040	0.6092	0.6158	0.6227	0.6289	0.6356	0.6424	0.6492	0.6564	0.6636	0.6707	0.6780	0.6852	0.6921	0.6978
Central Europe	0.7414	0.7460	0.7497	0.7531	0.7579	0.7630	0.7664	0.7696	0.7731	0.7768	0.7809	0.7855	0.7900	0.7943	0.7989	0.8035	0.8084	0.8137	0.8190	0.8235	0.8277	0.8318	0.8356	0.8391	0.8424	0.8458	0.8491
Albania	0.5845	0.5849	0.5842	0.5850	0.5878	0.5926	0.6005	0.6067	0.6140	0.6232	0.6327	0.6424	0.6513	0.6595	0.6671	0.6739	0.6801	0.6861	0.6920	0.6974	0.7019	0.7064	0.7106	0.7143	0.7179	0.7215	0.7246
Bosnia and Herzegovina	0.5425	0.5424	0.5425	0.5431	0.5455	0.5519	0.5725	0.5968	0.6189	0.6349	0.6476	0.6581	0.6674	0.6749	0.6816	0.6877	0.6935	0.6993	0.7049	0.7096	0.7137	0.7175	0.7208	0.7241	0.7271	0.7302	0.7331
Bulgaria	0.7600	0.7668	0.7710	0.7711	0.7712	0.7715	0.7711	0.7705	0.7708	0.7713	0.7729	0.7756	0.7794	0.7838	0.7888	0.7942	0.8002	0.8069	0.8128	0.8179	0.8237	0.8283	0.8323	0.8360	0.8394	0.8428	0.8461
Croatia	0.7860	0.7885	0.7879	0.7853	0.7821	0.7789	0.7788	0.7847	0.7905	0.7933	0.7971	0.8014	0.8059	0.8106	0.8150	0.8193	0.8237	0.8284	0.8329	0.8360	0.8384	0.8409	0.8429	0.8447	0.8464	0.8481	0.8499
Czech Republic	0.8116	0.8160	0.8200	0.8262	0.8291	0.8303	0.8321	0.8337	0.8352	0.8369	0.8391	0.8416	0.8441	0.8467	0.8496	0.8527	0.8562	0.8601	0.8632	0.8659	0.8686	0.8710	0.8731	0.8749	0.8751	0.8778	0.8811
Hungary	0.7525	0.7561	0.7615	0.7658	0.7703	0.7766	0.7797	0.7826	0.7859	0.7895	0.7935	0.7980	0.8028	0.8073	0.8118	0.8162	0.8206	0.8246	0.8284	0.8311	0.8337	0.8362	0.8384	0.8408	0.8434	0.8462	0.8490
Macedonia	0.7002	0.6993	0.7012	0.7007	0.7006	0.7033	0.7075	0.7118	0.7157	0.7185	0.7228	0.7304	0.7370	0.7416	0.7455	0.7490	0.7530	0.7575	0.7622	0.7655	0.7704	0.7750	0.7785	0.7821	0.7858	0.7893	0.7926
Montenegro	0.7501	0.7512	0.7504	0.7468	0.7431	0.7404	0.7402	0.7411	0.7432	0.7450	0.7484	0.7532	0.7586	0.7641	0.7693	0.7734	0.7771	0.7810	0.7857	0.7903	0.7953	0.7999	0.8030	0.8061	0.8091	0.8123	0.8152
Poland	0.7347	0.7392	0.7454	0.7510	0.7586	0.7665	0.7725	0.7778	0.7834	0.7891	0.7950	0.8005	0.8055	0.8102	0.8150	0.8197	0.8245	0.8298	0.8350	0.8400	0.8451	0.8503	0.8552	0.8596	0.8639	0.8680	0.8720

Appendix Table 5B. SDI Values by location, 1990-2016																											
Location	1990	1991	1992	1993	1994	1995	1996	1997	1998	1999	2000	2001	2002	2003	2004	2005	2006	2007	2008	2009	2010	2011	2012	2013	2014	2015	2016
Romania	0.7307	0.7378	0.7384	0.7388	0.7398	0.7418	0.7444	0.7467	0.7488	0.7515	0.7546	0.7588	0.7637	0.7686	0.7743	0.7798	0.7864	0.7936	0.8015	0.8076	0.8127	0.8176	0.8222	0.8265	0.8301	0.8334	0.8379
Serbia	0.7005	0.7034	0.7037	0.7014	0.6988	0.6971	0.6985	0.7022	0.7067	0.7091	0.7115	0.7137	0.7173	0.7219	0.7278	0.7325	0.7374	0.7423	0.7475	0.7516	0.7551	0.7585	0.7615	0.7645	0.7668	0.7689	0.7709
Slovakia	0.7848	0.7882	0.7918	0.7974	0.8046	0.8100	0.8119	0.8142	0.8168	0.8192	0.8218	0.8247	0.8279	0.8312	0.8347	0.8385	0.8429	0.8483	0.8537	0.8576	0.8604	0.8651	0.8684	0.8714	0.8743	0.8771	0.8799
Slovenia	0.8133	0.8130	0.8124	0.8125	0.8133	0.8148	0.8170	0.8199	0.8231	0.8268	0.8309	0.8351	0.8393	0.8433	0.8474	0.8514	0.8556	0.8601	0.8629	0.8648	0.8668	0.8692	0.8715	0.8731	0.8742	0.8771	0.8807
Eastern Europe	0.7674	0.7750	0.7816	0.7820	0.7799	0.7775	0.7742	0.7712	0.7681	0.7662	0.7663	0.7677	0.7702	0.7744	0.7798	0.7858	0.7924	0.7995	0.8064	0.8097	0.8134	0.8166	0.8188	0.8207	0.8233	0.8261	0.8290
Belarus	0.7382	0.7435	0.7477	0.7508	0.7517	0.7480	0.7445	0.7420	0.7407	0.7404	0.7414	0.7437	0.7472	0.7520	0.7580	0.7649	0.7724	0.7800	0.7883	0.7948	0.8002	0.8053	0.8098	0.8138	0.8181	0.8219	0.8257
Estonia	0.7986	0.8071	0.8125	0.8144	0.8126	0.8120	0.8117	0.8132	0.8157	0.8184	0.8224	0.8270	0.8321	0.8375	0.8431	0.8471	0.8500	0.8537	0.8585	0.8614	0.8660	0.8718	0.8766	0.8803	0.8828	0.8851	0.8874
Latvia	0.7828	0.7892	0.7928	0.7939	0.7900	0.7862	0.7831	0.7814	0.7813	0.7826	0.7853	0.7896	0.7952	0.8013	0.8078	0.8148	0.8224	0.8306	0.8371	0.8406	0.8433	0.8466	0.8501	0.8509	0.8507	0.8514	0.8532
Lithuania	0.7916	0.7947	0.7998	0.8047	0.8051	0.8036	0.8013	0.8006	0.8013	0.8025	0.8051	0.8088	0.8136	0.8194	0.8255	0.8316	0.8378	0.8448	0.8512	0.8534	0.8559	0.8585	0.8618	0.8653	0.8685	0.8721	0.8762
Moldova	0.6465	0.6512	0.6525	0.6537	0.6523	0.6502	0.6469	0.6434	0.6373	0.6306	0.6257	0.6239	0.6254	0.6287	0.6339	0.6400	0.6464	0.6528	0.6599	0.6653	0.6712	0.6778	0.6832	0.6891	0.6947	0.6993	0.7034
Russia	0.7736	0.7823	0.7881	0.7874	0.7858	0.7837	0.7808	0.7781	0.7752	0.7735	0.7740	0.7754	0.7780	0.7820	0.7871	0.7929	0.7993	0.8061	0.8115	0.8145	0.8182	0.8211	0.8226	0.8238	0.8259	0.8286	0.8317
Ukraine	0.7481	0.7531	0.7572	0.7598	0.7579	0.7535	0.7481	0.7425	0.7369	0.7319	0.7287	0.7280	0.7293	0.7334	0.7400	0.7470	0.7546	0.7627	0.7702	0.7741	0.7779	0.7821	0.7854	0.7889	0.7911	0.7920	0.7927
High-income	0.8126	0.8169	0.8216	0.8260	0.8301	0.8336	0.8368	0.8398	0.8425	0.8451	0.8482	0.8519	0.8544	0.8563	0.8587	0.8603	0.8612	0.8629	0.8656	0.8682	0.8706	0.8728	0.8751	0.8772	0.8789	0.8807	0.8823
High-income Asia Pacific	0.8047	0.8095	0.8155	0.8210	0.8260	0.8309	0.8350	0.8386	0.8414	0.8439	0.8466	0.8490	0.8514	0.8538	0.8563	0.8588	0.8614	0.8642	0.8666	0.8684	0.8706	0.8727	0.8749	0.8771	0.8793	0.8815	0.8838
Brunei	0.7444	0.7505	0.7559	0.7610	0.7661	0.7716	0.7772	0.7831	0.7892	0.7958	0.8025	0.8086	0.8148	0.8210	0.8272	0.8333	0.8389	0.8440	0.8486	0.8525	0.8559	0.8585	0.8608	0.8632	0.8657	0.8686	0.8710
Japan	0.8222	0.8278	0.8326	0.8366	0.8402	0.8437	0.8469	0.8499	0.8522	0.8541	0.8560	0.8578	0.8594	0.8611	0.8630	0.8648	0.8668	0.8689	0.8706	0.8718	0.8734	0.8749	0.8765	0.8783	0.8800	0.8817	0.8836
South Korea	0.7384	0.7440	0.7534	0.7635	0.7720	0.7813	0.7911	0.7998	0.8047	0.8101	0.8156	0.8207	0.8261	0.8308	0.8355	0.8399	0.8446	0.8493	0.8534	0.8569	0.8608	0.8644	0.8679	0.8712	0.8745	0.8777	0.8810
Singapore	0.7409	0.7481	0.7535	0.7604	0.7684	0.7759	0.7839	0.7948	0.8021	0.8045	0.8122	0.8183	0.8234	0.8284	0.8339	0.8401	0.8463	0.8524	0.8579	0.8630	0.8667	0.8693	0.8727	0.8761	0.8795	0.8827	0.8858
Hokkaido	0.8134	0.8180	0.8223	0.8261	0.8297	0.8330	0.8362	0.8391	0.8412	0.8431	0.8449	0.8466	0.8481	0.8498	0.8515	0.8533	0.8551	0.8570	0.8586	0.8596	0.8611	0.8624	0.8640	0.8655	0.8671	0.8687	0.8704
Aomori	0.8055	0.8097	0.8142	0.8178	0.8219	0.8268	0.8311	0.8350	0.8377	0.8397	0.8416	0.8433	0.8449	0.8465	0.8483	0.8500	0.8518	0.8537	0.8554	0.8563	0.8578	0.8592	0.8607	0.8623	0.8639	0.8655	0.8672
Iwate	0.7966	0.8015	0.8069	0.8112	0.8159	0.8213	0.8257	0.8297	0.8329	0.8353	0.8376	0.8403	0.8422	0.8439	0.8457	0.8475	0.8494	0.8514	0.8532	0.8542	0.8558	0.8572	0.8588	0.8604	0.8620	0.8637	0.8655
Miyagi	0.8127	0.8184	0.8243	0.8284	0.8321	0.8356	0.8389	0.8419	0.8442	0.8461	0.8480	0.8498	0.8514	0.8531	0.8549	0.8567	0.8585	0.8605	0.8622	0.8633	0.8648	0.8662	0.8678	0.8694	0.8710	0.8727	0.8744
Akita	0.8019	0.8064	0.8110	0.8145	0.8186	0.8235	0.8279	0.8320	0.8343	0.8362	0.8382	0.8399	0.8416	0.8432	0.8451	0.8469	0.8488	0.8507	0.8524	0.8535	0.8550	0.8564	0.8579	0.8595	0.8611	0.8628	0.8645
Yamagata	0.7933	0.7983	0.8034	0.8073	0.8117	0.8166	0.8210	0.8251	0.8285	0.8313	0.8338	0.8369	0.8401	0.8429	0.8448	0.8467	0.8487	0.8507	0.8525	0.8536	0.8552	0.8567	0.8583	0.8600	0.8616	0.8633	0.8652
Fukushima	0.8008	0.8059	0.8112	0.8152	0.8196	0.8245	0.8287	0.8325	0.8358	0.8385	0.8410	0.8438	0.8469	0.8502	0.8533	0.8552	0.8567	0.8585	0.8603	0.8615	0.8627	0.8638	0.8647	0.8659	0.8678	0.8701	0.8725
Ibaraki	0.8165	0.8221	0.8279	0.8323	0.8371	0.8422	0.8460	0.8489	0.8511	0.8530	0.8549	0.8566	0.8582	0.8598	0.8616	0.8634	0.8652	0.8672	0.8689	0.8700	0.8715	0.8730	0.8745	0.8762	0.8778	0.8795	0.8813
Tochigi	0.8176	0.8234	0.8294	0.8340	0.8388	0.8440	0.8480	0.8509	0.8532	0.8552	0.8571	0.8589	0.8606	0.8623	0.8641	0.8660	0.8679	0.8699	0.8716	0.8727	0.8743	0.8757	0.8773	0.8790	0.8807	0.8824	0.8842
Gunma	0.8124	0.8178	0.8232	0.8273	0.8318	0.8368	0.8410	0.8449	0.8474	0.8494	0.8513	0.8531	0.8547	0.8564	0.8582	0.8601	0.8619	0.8639	0.8656	0.8667	0.8682	0.8697	0.8713	0.8730	0.8746	0.8763	0.8781
Saitama	0.8101	0.8152	0.8196	0.8235	0.8272	0.8307	0.8338	0.8366	0.8387	0.8404	0.8421	0.8437	0.8451	0.8466	0.8482	0.8498	0.8516	0.8535	0.8550	0.8560	0.8573	0.8586	0.8601	0.8616	0.8631	0.8646	0.8662
Chiba	0.8148	0.8196	0.8239	0.8279	0.8315	0.8350	0.8382	0.8409	0.8430	0.8448	0.8465	0.8481	0.8496	0.8511	0.8528	0.8545	0.8562	0.8581	0.8597	0.8606	0.8620	0.8634	0.8649	0.8664	0.8679	0.8695	0.8711
Tokyo	0.8622	0.8667	0.8708	0.8746	0.8780	0.8813	0.8846	0.8874	0.8897	0.8916	0.8935	0.8953	0.8970	0.8987	0.9006	0.9025	0.9045	0.9064	0.9079	0.9093	0.9108	0.9123	0.9138	0.9152	0.9167	0.9182	0.9198
Kanagawa	0.8214	0.8261	0.8304	0.8343	0.8379	0.8413	0.8444	0.8472	0.8493	0.8511	0.8529	0.8545	0.8560	0.8575	0.8592	0.8609	0.8627	0.8646	0.8662	0.8672	0.8686	0.8699	0.8714	0.8730	0.8745	0.8761	0.8778
Niigata	0.8053	0.8107	0.8163	0.8206	0.8252	0.8305	0.8350	0.8392	0.8429	0.8453	0.8473	0.8491	0.8508	0.8526	0.8544	0.8563	0.8583	0.8603	0.8621	0.8633	0.8649	0.8664	0.8680	0.8698	0.8714	0.8732	0.8750
Toyama	0.8171	0.8227	0.8282	0.8322	0.8364	0.8403	0.8437	0.8467	0.8491	0.8511	0.8531	0.8549	0.8566	0.8583	0.8602	0.8621	0.8640	0.8661	0.8678	0.8690	0.8706	0.8721	0.8738	0.8755	0.8772	0.8790	0.8808
Ishikawa	0.8146	0.8205	0.8263	0.8309	0.8356	0.8396	0.8430	0.8460	0.8483	0.8503	0.8523	0.8540	0.8557	0.8573	0.8592	0.8610	0.8629	0.8650	0.8667	0.8678	0.8694	0.8709	0.8725	0.8743	0.8759	0.8777	0.8795
Fukui	0.8099	0.8154	0.8208	0.8248	0.8291	0.8341	0.8383	0.8423	0.8458	0.8486	0.8512	0.8541	0.8570	0.8592	0.8611	0.8630	0.8640	0.8648	0.8659	0.8665	0.8674	0.8685	0.8694	0.8708	0.8730	0.8757	0.8784
Yamanashi	0.8076	0.8126	0.8178	0.8219	0.8266	0.8320	0.8366	0.8408	0.8443	0.8463	0.8482	0.8500	0.8517	0.8534	0.8552	0.8571	0.8590	0.8609	0.8627	0.8638	0.8653	0.8668	0.8684	0.8701	0.8718	0.8735	0.8754
Nagano	0.8050	0.8103	0.8159	0.8201	0.8247	0.8298	0.8341	0.8380	0.8414	0.8441	0.8466	0.8493	0.8519	0.8537	0.8555	0.8574	0.8593	0.8613	0.8631	0.8642	0.8658	0.8671	0.8681	0.8693	0.8713	0.8737	0.8759
Gifu	0.8112	0.8168	0.8223	0.8265	0.8308	0.8345	0.8378	0.8408	0.8431	0.8450	0.8469	0.8486	0.8503	0.8519	0.8537	0.8556	0.8574	0.8594	0.8611	0.8622	0.8637	0.8651	0.8667	0.8684	0.8700	0.8717	0.8734
Shizuoka	0.8229	0.8286	0.8343	0.8387	0.8432	0.8475	0.8507	0.8536	0.8559	0.8578	0.8596	0.8614	0.8630	0.8647	0.8665	0.8683	0.8701	0.8721	0.8738	0.8745	0.8753	0.8763	0.8772	0.8786	0.8807	0.8834	0.8861
Aichi	0.8334	0.8391	0.8448	0.8489	0.8525	0.8560	0.8592	0.8620	0.8642	0.8661	0.8679	0.8696	0.8713	0.8730	0.8747	0.8766	0.8785	0.8804	0.8821	0.8832	0.8846	0.8855	0.8865	0.8879	0.8901	0.8927	0.8945
Mie	0.8181	0.8239	0.8297	0.8340	0.8385	0.8425	0.8459	0.8489	0.8512	0.8532	0.8551	0.8570	0.8587	0.8604	0.8623	0.8642	0.8662	0.8682	0.8700	0.8711	0.8727	0.8742	0.8755	0.8768	0.8789	0.8810	0.8829
Shiga	0.8186	0.8246	0.8307	0.8352	0.8399	0.8452	0.8495	0.8534	0.8567	0.8593	0.8621	0.8636	0.8653	0.8670	0.8689	0.8708	0.8727	0.8747	0.8764	0.8773	0.8779	0.8788	0.8798	0.8811	0.8833	0.8861	0.8889
Kyoto	0.8206	0.8254	0.8299	0.8339	0.8377	0.8413	0.8446	0.8476	0.8499	0.8518	0.8537	0.8555	0.8571	0.8588	0.8606	0.8625	0.8644	0.8663	0.8681	0.8691	0.8707	0.8722	0.8738	0.8755	0.8772	0.8790	0.8808
Osaka	0.8317	0.8363	0.8406	0.8445	0.8481	0.8515	0.8547	0.8575	0.8596	0.8614	0.8632	0.8648	0.8663	0.8678	0.8694	0.8711	0.8729	0.8749	0.8765	0.8775	0.8790	0.8804	0.8819	0.8835	0.8851	0.8867	0.8884
Hyogo	0.8136	0.8185	0.8229	0.8270	0.8307	0.8343	0.8376	0.8405	0.8428	0.8447	0.8466	0.8483	0.8499	0.8515	0.8532	0.8550	0.8569	0.8589	0.8606	0.8616	0.8632	0.8646	0.8662	0.8678	0.8695	0.8711	0.8729
Nara	0.7972	0.8021	0.8067	0.8107	0.8145	0.8181	0.8213	0.8242	0.8264	0.8283	0.8301	0.8317	0.8332	0.8347	0.8364	0.8381	0.8398	0.8417	0.8433	0.8443	0.8457	0.8470	0.8485	0.8501	0.8517	0.8533	0.8550
Wakayama	0.8060	0.8117	0.8172	0.8214	0.8256	0.8293	0.8326	0.8356	0.8379	0.8398	0.8418	0.8435	0.8451	0.8468	0.8486	0.8504	0.8524	0.8544	0.8562	0.8573	0.8589	0.8604	0.8620	0.8637	0.8654	0.8671	0.8690
Tottori	0.7913	0.7968	0.8026	0.8071	0.8120	0.8174	0.8220	0.8261	0.8296	0.8322	0.8345	0.8372	0.8402	0.8433	0.8455	0.8475	0.8494	0.8515	0.8533	0.8544	0.8559	0.8569	0.8579	0.8592	0.8611	0.8636	0.8662
Shimane	0.7883	0.7935	0.7990	0.8032	0.8080	0.8134	0.8183	0.8228	0.8267	0.8299	0.8329	0.8361	0.8393	0.8425	0.8446	0.8466	0.8475	0.8485	0.8497	0.8502	0.8510	0.8519	0.8528	0.8542	0.8564	0.8592	0.8620
Okayama	0.8094	0.8152	0.8210	0.8254	0.8299	0.8348	0.8389	0.8426	0.8453	0.8474	0.8494	0.8513	0.8530	0.8548	0.8567	0.8587	0.8607	0.8628	0.8646	0.8658	0.8674	0.8690	0.8707	0.8724	0.8742	0.8761	0.8780
Hiroshima	0.8164	0.8222	0.8280	0.8326	0.8374	0.8416	0.8449	0.8479	0.8501	0.8521	0.8540	0.8558	0.8575	0.8592	0.8610	0.8628	0.8648	0.8668	0.8686	0.8685	0.8689	0.8697	0.8706	0.8721	0.8745	0.8775	0.8805
Yamaguchi	0.8131	0.8184	0.8238	0.8278	0.8322	0.8369	0.8402	0.8432	0.8456	0.8476	0.8496	0.8515	0.8532	0.8549	0.8568	0.8587	0.8607	0.8628	0.8646	0.8658	0.8667	0.8675	0.8684	0.8698	0.8720	0.8748	0.8776
Tokushima	0.8084	0.8140	0.8196	0.8238	0.8282	0.8319	0.8353	0.8384	0.8408	0.8429	0.8450	0.8468	0.8486	0.8504	0.8523	0.8542	0.8561	0.8581	0.8599	0.8609	0.8625	0.8640	0.8656	0.8673	0.8690	0.8707	0.8726
Kagawa	0.8107	0.8165	0.8223	0.8266	0.8310	0.8354	0.8388	0.8419	0.8443	0.8463	0.8484	0.8502	0.8519	0.8536	0.8555	0.8574	0.8593	0.8614	0.8627	0.8631	0.8638	0.8647	0.8656	0.8669	0.8691	0.8718	0.8746
Ehime	0.8040	0.8094	0.8148	0.8190	0.8235	0.8284	0.8320	0.8351	0.8374	0.8395	0.8415	0.8433	0.8450	0.8468	0.8486	0.8505	0.8525	0.8545	0.8563	0.8574	0.8590	0.8605	0.8622	0.8639	0.8656	0.8673	0.8691
Kochi	0.7916	0.7965	0.8013	0.8051	0.8095	0.8146	0.8192	0.8227	0.8251	0.8272	0.8293	0.8312	0.8329	0.8347	0.8367	0.8386	0.8405	0.8425	0.8442	0.8453	0.8469	0.8484	0.8500	0.8517	0.8534	0.8551	0.8570
Fukuoka	0.8161	0.8215	0.8261	0.8301	0.8338	0.8373	0.8407	0.8437	0.8461	0.8481	0.8500	0.8518	0.8535	0.8552	0.8571	0.8590	0.8609	0.8629	0.8646	0.8658	0.8673	0.8688	0.8705	0.8722	0.8739	0.8756	0.8775
Saga	0.7981	0.8032	0.8085	0.8123	0.8164	0.8210	0.8248	0.8284	0.8315	0.8339	0.8362	0.8392	0.8425	0.8459	0.8485	0.8505	0.8519	0.8531	0.8543	0.8549	0.8557	0.8566	0.8574	0.8587	0.8608	0.8634	0.8661
Nagasaki	0.7942	0.7990	0.8041	0.8081	0.8127	0.8178	0.8221	0.8261	0.8294	0.8319	0.8343	0.8371	0.8393	0.8410	0.8429	0.8447	0.8466	0.8477	0.8485	0.8486	0.8491	0.8498	0.8506	0.8518	0.8539	0.8567	0.8595
Kumamoto	0.7958	0.8009	0.8062	0.8101	0.8143	0.8190	0.8230	0.8267	0.8299	0.8323	0.8345	0.8370	0.8395	0.8413	0.8432	0.8451	0.8458	0.8464	0.8473	0.8477	0.8486	0.8496	0.8505	0.8519	0.8542	0.8569	0.8598

Appendix Table 5B. SDI Values by location, 1990-2016																											
**Location**	**1990**	**1991**	**1992**	**1993**	**1994**	**1995**	**1996**	**1997**	**1998**	**1999**	**2000**	**2001**	**2002**	**2003**	**2004**	**2005**	**2006**	**2007**	**2008**	**2009**	**2010**	**2011**	**2012**	**2013**	**2014**	**2015**	**2016**
Oita	0.8065	0.8116	0.8168	0.8207	0.8249	0.8297	0.8338	0.8376	0.8409	0.8430	0.8450	0.8468	0.8486	0.8504	0.8523	0.8543	0.8563	0.8584	0.8596	0.8602	0.8612	0.8623	0.8633	0.8647	0.8669	0.8696	0.8723
Miyazaki	0.7947	0.7995	0.8043	0.8078	0.8118	0.8165	0.8206	0.8246	0.8281	0.8307	0.8331	0.8358	0.8386	0.8413	0.8440	0.8451	0.8451	0.8457	0.8465	0.8468	0.8475	0.8483	0.8491	0.8504	0.8525	0.8553	0.8581
Kagoshima	0.7933	0.7985	0.8039	0.8080	0.8126	0.8177	0.8221	0.8262	0.8297	0.8322	0.8345	0.8370	0.8397	0.8419	0.8439	0.8459	0.8464	0.8472	0.8482	0.8487	0.8496	0.8506	0.8517	0.8531	0.8554	0.8582	0.8611
Okinawa	0.7821	0.7874	0.7931	0.7974	0.8023	0.8078	0.8121	0.8158	0.8187	0.8208	0.8227	0.8254	0.8284	0.8316	0.8347	0.8361	0.8360	0.8366	0.8372	0.8370	0.8373	0.8378	0.8385	0.8397	0.8420	0.8451	0.8481
Australasia	0.8154	0.8183	0.8208	0.8242	0.8279	0.8317	0.8355	0.8394	0.8427	0.8458	0.8493	0.8527	0.8556	0.8580	0.8602	0.8617	0.8612	0.8610	0.8637	0.8674	0.8703	0.8722	0.8751	0.8797	0.8838	0.8866	0.8886
Australia	0.8177	0.8203	0.8228	0.8263	0.8300	0.8340	0.8380	0.8419	0.8455	0.8490	0.8527	0.8559	0.8587	0.8615	0.8638	0.8652	0.8652	0.8653	0.8679	0.8717	0.8745	0.8760	0.8785	0.8831	0.8874	0.8904	0.8924
New Zealand	0.8031	0.8071	0.8096	0.8130	0.8163	0.8191	0.8221	0.8259	0.8278	0.8283	0.8309	0.8357	0.8390	0.8396	0.8414	0.8430	0.8402	0.8383	0.8415	0.8442	0.8471	0.8511	0.8556	0.8604	0.8632	0.8651	0.8668
Western Europe	0.8013	0.8067	0.8124	0.8172	0.8209	0.8245	0.8280	0.8316	0.8351	0.8381	0.8416	0.8453	0.8480	0.8499	0.8522	0.8542	0.8559	0.8575	0.8594	0.8613	0.8636	0.8664	0.8691	0.8714	0.8728	0.8743	0.8761
Andorra	0.8680	0.8700	0.8718	0.8731	0.8745	0.8760	0.8779	0.8807	0.8838	0.8869	0.8888	0.8906	0.8923	0.8941	0.8958	0.8975	0.8991	0.9008	0.9024	0.9040	0.9055	0.9071	0.9086	0.9101	0.9116	0.9131	0.9148
Austria	0.8298	0.8324	0.8355	0.8384	0.8410	0.8436	0.8462	0.8487	0.8514	0.8541	0.8570	0.8596	0.8622	0.8645	0.8668	0.8691	0.8714	0.8739	0.8764	0.8782	0.8802	0.8823	0.8845	0.8866	0.8886	0.8904	0.8922
Belgium	0.8432	0.8459	0.8503	0.8551	0.8589	0.8609	0.8625	0.8651	0.8674	0.8687	0.8705	0.8737	0.8765	0.8772	0.8773	0.8777	0.8785	0.8798	0.8815	0.8834	0.8862	0.8896	0.8930	0.8956	0.8969	0.8978	0.8988
Cyprus	0.7450	0.7476	0.7542	0.7651	0.7741	0.7827	0.7902	0.7981	0.8059	0.8124	0.8190	0.8260	0.8314	0.8349	0.8392	0.8432	0.8474	0.8517	0.8561	0.8596	0.8628	0.8655	0.8676	0.8688	0.8697	0.8707	0.8718
Denmark	0.8796	0.8796	0.8802	0.8808	0.8818	0.8854	0.8892	0.8925	0.8954	0.8970	0.8995	0.9031	0.9050	0.9052	0.9057	0.9059	0.9067	0.9076	0.9093	0.9108	0.9142	0.9190	0.9223	0.9246	0.9253	0.9252	0.9253
Finland	0.8408	0.8413	0.8421	0.8431	0.8449	0.8486	0.8523	0.8564	0.8600	0.8630	0.8672	0.8714	0.8742	0.8758	0.8780	0.8802	0.8827	0.8857	0.8881	0.8896	0.8924	0.8962	0.9001	0.9039	0.9062	0.9068	0.9071
France	0.7986	0.8046	0.8117	0.8174	0.8205	0.8229	0.8262	0.8295	0.8319	0.8333	0.8355	0.8396	0.8435	0.8459	0.8477	0.8487	0.8505	0.8531	0.8549	0.8562	0.8581	0.8607	0.8631	0.8653	0.8665	0.8675	0.8688
Germany	0.8288	0.8322	0.8354	0.8381	0.8406	0.8431	0.8453	0.8475	0.8497	0.8519	0.8542	0.8565	0.8586	0.8605	0.8623	0.8641	0.8662	0.8687	0.8711	0.8727	0.8748	0.8772	0.8796	0.8819	0.8842	0.8865	0.8887
Greece	0.7651	0.7699	0.7745	0.7788	0.7831	0.7874	0.7918	0.7964	0.8011	0.8057	0.8103	0.8150	0.8199	0.8246	0.8295	0.8337	0.8383	0.8428	0.8466	0.8499	0.8520	0.8526	0.8526	0.8524	0.8524	0.8525	0.8532
Iceland	0.8471	0.8517	0.8522	0.8554	0.8605	0.8622	0.8645	0.8686	0.8725	0.8748	0.8793	0.8864	0.8884	0.8879	0.8893	0.8916	0.8938	0.8953	0.8942	0.8944	0.9011	0.9064	0.9094	0.9144	0.9176	0.9199	0.9213
Ireland	0.7679	0.7753	0.7840	0.7917	0.7977	0.8023	0.8060	0.8110	0.8183	0.8262	0.8319	0.8365	0.8420	0.8481	0.8555	0.8605	0.8609	0.8610	0.8626	0.8642	0.8669	0.8705	0.8737	0.8768	0.8795	0.8826	0.8855
Israel	0.7362	0.7427	0.7484	0.7527	0.7570	0.7608	0.7637	0.7655	0.7668	0.7689	0.7723	0.7754	0.7765	0.7768	0.7786	0.7815	0.7843	0.7856	0.7859	0.7878	0.7910	0.7939	0.7957	0.7969	0.7980	0.7993	0.8010
Italy	0.7983	0.8027	0.8069	0.8105	0.8141	0.8176	0.8210	0.8243	0.8277	0.8309	0.8343	0.8375	0.8404	0.8431	0.8457	0.8480	0.8503	0.8527	0.8551	0.8567	0.8584	0.8601	0.8617	0.8630	0.8642	0.8655	0.8669
Luxembourg	0.8761	0.8797	0.8817	0.8845	0.8883	0.8905	0.8932	0.8979	0.9004	0.9005	0.9038	0.9091	0.9117	0.9124	0.9133	0.9148	0.9167	0.9192	0.9210	0.9215	0.9244	0.9276	0.9289	0.9318	0.9343	0.9355	0.9358
Malta	0.7519	0.7543	0.7588	0.7678	0.7755	0.7767	0.7776	0.7860	0.7953	0.8029	0.8140	0.8188	0.8220	0.8247	0.8269	0.8291	0.8313	0.8338	0.8363	0.8382	0.8404	0.8425	0.8450	0.8476	0.8498	0.8529	0.8563
Netherlands	0.8499	0.8542	0.8586	0.8623	0.8664	0.8706	0.8732	0.8744	0.8760	0.8777	0.8804	0.8840	0.8864	0.8893	0.8929	0.8956	0.8980	0.8995	0.9005	0.9020	0.9048	0.9088	0.9126	0.9153	0.9165	0.9173	0.9181
Norway	0.8473	0.8513	0.8556	0.8589	0.8616	0.8641	0.8680	0.8737	0.8775	0.8799	0.8851	0.8910	0.8937	0.8945	0.8962	0.8973	0.8986	0.9001	0.9008	0.9030	0.9075	0.9118	0.9163	0.9200	0.9218	0.9222	0.9224
Portugal	0.6868	0.6939	0.7013	0.7081	0.7138	0.7193	0.7247	0.7301	0.7355	0.7402	0.7460	0.7508	0.7553	0.7593	0.7630	0.7664	0.7698	0.7733	0.7765	0.7793	0.7821	0.7846	0.7867	0.7889	0.7912	0.7936	0.7962
Spain	0.7459	0.7524	0.7587	0.7643	0.7698	0.7752	0.7804	0.7857	0.7909	0.7962	0.8013	0.8063	0.8112	0.8159	0.8202	0.8244	0.8284	0.8325	0.8360	0.8385	0.8404	0.8419	0.8431	0.8440	0.8451	0.8465	0.8482
Sweden	0.8066	0.8105	0.8156	0.8217	0.8295	0.8382	0.8450	0.8494	0.8526	0.8550	0.8568	0.8574	0.8567	0.8569	0.8582	0.8581	0.8579	0.8592	0.8601	0.8593	0.8602	0.8639	0.8670	0.8687	0.8700	0.8711	0.8724
Switzerland	0.8689	0.8699	0.8721	0.8741	0.8741	0.8741	0.8741	0.8743	0.8749	0.8755	0.8764	0.8774	0.8784	0.8791	0.8800	0.8811	0.8826	0.8846	0.8867	0.8879	0.8893	0.8912	0.8932	0.8949	0.8965	0.8987	0.9013
United Kingdom	0.7635	0.7693	0.7754	0.7815	0.7874	0.7926	0.7972	0.8024	0.8083	0.8151	0.8216	0.8268	0.8298	0.8315	0.8343	0.8369	0.8383	0.8396	0.8416	0.8433	0.8460	0.8489	0.8528	0.8572	0.8600	0.8618	0.8634
Sweden except Stockholm	0.7841	0.7885	0.7941	0.8009	0.8093	0.8186	0.8259	0.8307	0.8342	0.8368	0.8389	0.8397	0.8393	0.8399	0.8415	0.8414	0.8414	0.8432	0.8448	0.8443	0.8458	0.8501	0.8538	0.8562	0.8580	0.8597	0.8612
Stockholm	0.8793	0.8817	0.8850	0.8890	0.8907	0.8920	0.8933	0.8948	0.8965	0.8986	0.9009	0.9029	0.9047	0.9061	0.9078	0.9094	0.9082	0.9071	0.9060	0.9042	0.9036	0.9051	0.9060	0.9059	0.9055	0.9050	0.9051
Northern Ireland	0.7817	0.7881	0.7950	0.8017	0.8073	0.8112	0.8148	0.8196	0.8256	0.8322	0.8380	0.8424	0.8458	0.8482	0.8503	0.8516	0.8515	0.8513	0.8528	0.8547	0.8557	0.8570	0.8595	0.8629	0.8664	0.8696	0.8724
Scotland	0.7553	0.7590	0.7642	0.7699	0.7751	0.7792	0.7824	0.7864	0.7918	0.7971	0.8014	0.8055	0.8093	0.8108	0.8127	0.8147	0.8156	0.8159	0.8177	0.8207	0.8232	0.8261	0.8296	0.8324	0.8338	0.8352	0.8369
Wales	0.7372	0.7422	0.7485	0.7555	0.7622	0.7671	0.7718	0.7779	0.7850	0.7924	0.7999	0.8065	0.8110	0.8131	0.8158	0.8189	0.8208	0.8224	0.8248	0.8267	0.8294	0.8330	0.8371	0.8420	0.8452	0.8470	0.8484
England	0.7647	0.7708	0.7769	0.7830	0.7890	0.7943	0.7991	0.8044	0.8103	0.8172	0.8239	0.8291	0.8319	0.8336	0.8366	0.8392	0.8407	0.8422	0.8441	0.8457	0.8484	0.8514	0.8553	0.8599	0.8628	0.8646	0.8662
Darlington	0.7463	0.7498	0.7560	0.7638	0.7723	0.7794	0.7854	0.7906	0.7959	0.8022	0.8081	0.8120	0.8130	0.8139	0.8172	0.8202	0.8226	0.8238	0.8241	0.8253	0.8306	0.8361	0.8414	0.8457	0.8478	0.8483	0.8486
Northumberland	0.7421	0.7456	0.7500	0.7556	0.7623	0.7687	0.7748	0.7812	0.7871	0.7920	0.7968	0.8001	0.8023	0.8036	0.8057	0.8080	0.8100	0.8122	0.8151	0.8171	0.8192	0.8214	0.8245	0.8282	0.8303	0.8314	0.8323
Stockton-on-Tees	0.7483	0.7529	0.7592	0.7663	0.7738	0.7800	0.7856	0.7917	0.7985	0.8058	0.8117	0.8154	0.8169	0.8181	0.8204	0.8224	0.8238	0.8253	0.8271	0.8287	0.8306	0.8326	0.8355	0.8398	0.8433	0.8459	0.8480
Newcastle upon Tyne	0.7630	0.7712	0.7797	0.7872	0.7924	0.7979	0.8019	0.8061	0.8115	0.8192	0.8268	0.8333	0.8400	0.8461	0.8522	0.8582	0.8614	0.8647	0.8684	0.8710	0.8734	0.8756	0.8785	0.8824	0.8849	0.8868	0.8886
North Tyneside	0.7366	0.7423	0.7488	0.7556	0.7622	0.7677	0.7725	0.7777	0.7838	0.7903	0.7967	0.8019	0.8051	0.8072	0.8114	0.8163	0.8204	0.8237	0.8267	0.8285	0.8312	0.8338	0.8368	0.8402	0.8425	0.8441	0.8458
Redcar and Cleveland	0.7143	0.7181	0.7225	0.7275	0.7334	0.7387	0.7437	0.7496	0.7569	0.7655	0.7737	0.7791	0.7815	0.7830	0.7860	0.7888	0.7909	0.7929	0.7945	0.7953	0.7961	0.7975	0.8015	0.8079	0.8127	0.8164	0.8192
County Durham	0.7284	0.7335	0.7396	0.7464	0.7533	0.7591	0.7644	0.7706	0.7774	0.7845	0.7914	0.7963	0.7986	0.8005	0.8032	0.8054	0.8074	0.8094	0.8118	0.8133	0.8157	0.8179	0.8218	0.8268	0.8297	0.8317	0.8334
Gateshead	0.7438	0.7495	0.7553	0.7613	0.7679	0.7737	0.7791	0.7848	0.7908	0.7969	0.8028	0.8077	0.8114	0.8142	0.8180	0.8214	0.8236	0.8258	0.8284	0.8304	0.8332	0.8367	0.8403	0.8442	0.8470	0.8490	0.8508
Middlesbrough	0.7272	0.7347	0.7414	0.7481	0.7551	0.7614	0.7668	0.7723	0.7784	0.7853	0.7911	0.7940	0.7951	0.7954	0.7997	0.8041	0.8083	0.8130	0.8165	0.8173	0.8184	0.8190	0.8209	0.8239	0.8258	0.8274	0.8289
South Tyneside	0.7069	0.7130	0.7190	0.7250	0.7309	0.7360	0.7407	0.7463	0.7530	0.7607	0.7684	0.7752	0.7802	0.7841	0.7887	0.7931	0.7963	0.7992	0.8020	0.8035	0.8053	0.8074	0.8103	0.8144	0.8167	0.8184	0.8201
Sunderland	0.7360	0.7415	0.7476	0.7541	0.7606	0.7666	0.7722	0.7784	0.7850	0.7914	0.7980	0.8044	0.8088	0.8120	0.8162	0.8203	0.8230	0.8261	0.8286	0.8301	0.8311	0.8321	0.8348	0.8385	0.8414	0.8435	0.8454
Hartlepool	0.7233	0.7285	0.7340	0.7397	0.7461	0.7526	0.7589	0.7648	0.7705	0.7768	0.7820	0.7854	0.7868	0.7877	0.7901	0.7928	0.7953	0.7985	0.8022	0.8049	0.8072	0.8100	0.8141	0.8185	0.8204	0.8210	0.8214
Cheshire East	0.7780	0.7826	0.7885	0.7952	0.8025	0.8091	0.8148	0.8207	0.8264	0.8323	0.8373	0.8421	0.8445	0.8455	0.8470	0.8481	0.8479	0.8473	0.8484	0.8506	0.8542	0.8580	0.8633	0.8689	0.8717	0.8730	0.8740
Stockport	0.7568	0.7626	0.7700	0.7776	0.7842	0.7893	0.7940	0.7997	0.8062	0.8134	0.8200	0.8250	0.8275	0.8287	0.8312	0.8334	0.8346	0.8362	0.8375	0.8379	0.8395	0.8412	0.8444	0.8491	0.8527	0.8553	0.8574
Trafford	0.7773	0.7837	0.7910	0.7987	0.8060	0.8116	0.8163	0.8214	0.8275	0.8346	0.8411	0.8462	0.8493	0.8512	0.8540	0.8552	0.8552	0.8560	0.8578	0.8601	0.8640	0.8679	0.8723	0.8775	0.8804	0.8819	0.8831
Cheshire West and Chester	0.7660	0.7730	0.7804	0.7873	0.7924	0.7950	0.7962	0.7985	0.8030	0.8116	0.8218	0.8312	0.8366	0.8389	0.8411	0.8434	0.8448	0.8460	0.8474	0.8488	0.8523	0.8566	0.8616	0.8657	0.8677	0.8686	0.8692
Sefton	0.7342	0.7399	0.7470	0.7543	0.7611	0.7666	0.7713	0.7767	0.7825	0.7888	0.7951	0.7996	0.8028	0.8051	0.8089	0.8114	0.8125	0.8129	0.8135	0.8139	0.8158	0.8181	0.8201	0.8227	0.8243	0.8253	0.8263
Lancashire	0.7488	0.7548	0.7615	0.7683	0.7749	0.7805	0.7858	0.7917	0.7979	0.8041	0.8104	0.8149	0.8171	0.8186	0.8216	0.8243	0.8257	0.8272	0.8289	0.8299	0.8319	0.8344	0.8380	0.8423	0.8450	0.8468	0.8482
Cumbria	0.7540	0.7574	0.7628	0.7696	0.7770	0.7832	0.7886	0.7943	0.7998	0.8044	0.8090	0.8124	0.8140	0.8153	0.8185	0.8201	0.8208	0.8213	0.8229	0.8247	0.8285	0.8323	0.8369	0.8418	0.8447	0.8464	0.8478
Bolton	0.7352	0.7409	0.7475	0.7544	0.7607	0.7656	0.7697	0.7744	0.7797	0.7855	0.7909	0.7952	0.7973	0.7983	0.7996	0.8002	0.7997	0.7999	0.8017	0.8036	0.8070	0.8105	0.8145	0.8189	0.8213	0.8225	0.8236
Wirral	0.7272	0.7330	0.7395	0.7462	0.7532	0.7595	0.7652	0.7712	0.7774	0.7827	0.7872	0.7897	0.7910	0.7923	0.7950	0.7973	0.7982	0.7984	0.7994	0.8002	0.8032	0.8075	0.8118	0.8166	0.8189	0.8197	0.8204
Bury	0.7325	0.7394	0.7469	0.7542	0.7608	0.7657	0.7697	0.7745	0.7805	0.7873	0.7933	0.7973	0.7990	0.8002	0.8029	0.8057	0.8066	0.8064	0.8056	0.8051	0.8080	0.8124	0.8181	0.8243	0.8278	0.8298	0.8312
St Helens	0.7219	0.7254	0.7310	0.7380	0.7450	0.7505	0.7550	0.7599	0.7658	0.7723	0.7799	0.7860	0.7897	0.7921	0.7957	0.7991	0.8014	0.8037	0.8061	0.8078	0.8098	0.8121	0.8161	0.8215	0.8256	0.8284	0.8305
Warrington	0.7738	0.7775	0.7828	0.7895	0.7965	0.8024	0.8073	0.8121	0.8173	0.8235	0.8303	0.8365	0.8407	0.8438	0.8472	0.8502	0.8515	0.8516	0.8523	0.8538	0.8579	0.8621	0.8675	0.8726	0.8753	0.8767	0.8778
Oldham	0.7200	0.7252	0.7313	0.7376	0.7432	0.7473	0.7506	0.7542	0.7583	0.7634	0.7686	0.7728	0.7746	0.7756	0.7779	0.7802	0.7816	0.7836	0.7865	0.7890	0.7925	0.7963	0.8004	0.8044	0.8064	0.8074	0.8081

Appendix Table 5B. SDI Values by location, 1990-2016

Location	1990	1991	1992	1993	1994	1995	1996	1997	1998	1999	2000	2001	2002	2003	2004	2005	2006	2007	2008	2009	2010	2011	2012	2013	2014	2015	2016
Rochdale	0.7201	0.7249	0.7308	0.7374	0.7442	0.7500	0.7549	0.7604	0.7665	0.7732	0.7789	0.7826	0.7841	0.7851	0.7868	0.7881	0.7876	0.7876	0.7890	0.7913	0.7954	0.7998	0.8047	0.8096	0.8125	0.8141	0.8153
Wigan	0.7274	0.7331	0.7393	0.7458	0.7523	0.7581	0.7633	0.7686	0.7739	0.7795	0.7846	0.7886	0.7910	0.7926	0.7950	0.7971	0.7981	0.7990	0.8001	0.8008	0.8036	0.8069	0.8114	0.8165	0.8200	0.8223	0.8241
Halton	0.7395	0.7450	0.7517	0.7592	0.7667	0.7730	0.7778	0.7816	0.7862	0.7924	0.7997	0.8063	0.8096	0.8106	0.8124	0.8143	0.8159	0.8175	0.8202	0.8224	0.8270	0.8317	0.8362	0.8419	0.8466	0.8503	0.8532
Liverpool	0.7381	0.7498	0.7596	0.7672	0.7730	0.7772	0.7814	0.7870	0.7945	0.8032	0.8121	0.8179	0.8241	0.8301	0.8358	0.8393	0.8419	0.8444	0.8477	0.8515	0.8551	0.8585	0.8620	0.8654	0.8671	0.8684	0.8695
Tameside	0.7239	0.7304	0.7373	0.7442	0.7507	0.7561	0.7607	0.7658	0.7715	0.7782	0.7856	0.7915	0.7950	0.7971	0.7999	0.8015	0.8011	0.8005	0.8004	0.8002	0.8020	0.8048	0.8088	0.8132	0.8161	0.8180	0.8196
Salford	0.7373	0.7442	0.7524	0.7611	0.7691	0.7752	0.7796	0.7843	0.7907	0.7990	0.8071	0.8136	0.8179	0.8209	0.8251	0.8284	0.8307	0.8325	0.8337	0.8352	0.8389	0.8430	0.8478	0.8529	0.8563	0.8586	0.8607
Blackburn with Darwen	0.7203	0.7263	0.7325	0.7390	0.7452	0.7505	0.7550	0.7599	0.7648	0.7698	0.7755	0.7794	0.7809	0.7818	0.7843	0.7857	0.7857	0.7875	0.7907	0.7935	0.7977	0.8021	0.8066	0.8121	0.8156	0.8179	0.8196
Knowsley	0.7201	0.7265	0.7327	0.7385	0.7442	0.7496	0.7553	0.7616	0.7676	0.7736	0.7807	0.7872	0.7906	0.7923	0.7954	0.7994	0.8030	0.8067	0.8109	0.8134	0.8162	0.8186	0.8220	0.8277	0.8316	0.8343	0.8365
Blackpool	0.7105	0.7218	0.7322	0.7401	0.7464	0.7508	0.7543	0.7593	0.7660	0.7726	0.7785	0.7816	0.7819	0.7800	0.7820	0.7860	0.7881	0.7877	0.7867	0.7878	0.7924	0.7962	0.7988	0.8013	0.8032	0.8060	0.8090
Manchester	0.7675	0.7746	0.7813	0.7887	0.7955	0.8014	0.8063	0.8117	0.8193	0.8292	0.8390	0.8474	0.8533	0.8575	0.8623	0.8660	0.8688	0.8719	0.8754	0.8784	0.8816	0.8843	0.8878	0.8922	0.8952	0.8974	0.8995
North Yorkshire	0.7620	0.7650	0.7693	0.7743	0.7802	0.7854	0.7902	0.7958	0.8019	0.8085	0.8146	0.8186	0.8207	0.8221	0.8255	0.8290	0.8312	0.8326	0.8342	0.8353	0.8377	0.8405	0.8441	0.8480	0.8493	0.8494	0.8498
East Riding of Yorkshire	0.7461	0.7521	0.7582	0.7647	0.7711	0.7766	0.7819	0.7877	0.7935	0.7990	0.8045	0.8084	0.8102	0.8113	0.8152	0.8178	0.8191	0.8207	0.8234	0.8254	0.8274	0.8283	0.8306	0.8337	0.8357	0.8371	0.8386
York	0.7808	0.7901	0.7985	0.8043	0.8098	0.8150	0.8192	0.8242	0.8306	0.8364	0.8422	0.8482	0.8540	0.8597	0.8654	0.8697	0.8735	0.8769	0.8795	0.8813	0.8829	0.8845	0.8862	0.8880	0.8898	0.8919	0.8940
North East Lincolnshire	0.7430	0.7484	0.7554	0.7623	0.7685	0.7731	0.7761	0.7795	0.7835	0.7888	0.7953	0.8006	0.8025	0.8021	0.8029	0.8055	0.8084	0.8132	0.8172	0.8216	0.8252	0.8281	0.8306	0.8333	0.8342	0.8340	0.8341
Calderdale	0.7456	0.7501	0.7553	0.7611	0.7673	0.7727	0.7774	0.7826	0.7884	0.7948	0.8008	0.8049	0.8062	0.8069	0.8100	0.8135	0.8158	0.8177	0.8195	0.8208	0.8241	0.8279	0.8328	0.8385	0.8424	0.8452	0.8473
North Lincolnshire	0.7411	0.7463	0.7514	0.7567	0.7632	0.7692	0.7751	0.7810	0.7870	0.7920	0.7964	0.8003	0.8020	0.8022	0.8033	0.8041	0.8046	0.8063	0.8095	0.8122	0.8163	0.8209	0.8260	0.8309	0.8334	0.8344	0.8350
Bradford	0.7257	0.7319	0.7380	0.7439	0.7499	0.7549	0.7592	0.7631	0.7674	0.7732	0.7789	0.7836	0.7861	0.7873	0.7898	0.7922	0.7948	0.7983	0.8023	0.8053	0.8078	0.8098	0.8121	0.8154	0.8171	0.8180	0.8189
Kirklees	0.7345	0.7409	0.7471	0.7531	0.7587	0.7632	0.7675	0.7725	0.7782	0.7847	0.7905	0.7946	0.7961	0.7973	0.8001	0.8028	0.8048	0.8071	0.8100	0.8117	0.8142	0.8168	0.8205	0.8249	0.8279	0.8300	0.8316
Leeds	0.7631	0.7706	0.7778	0.7846	0.7908	0.7962	0.8015	0.8074	0.8141	0.8225	0.8295	0.8357	0.8416	0.8471	0.8519	0.8571	0.8612	0.8652	0.8685	0.8693	0.8691	0.8684	0.8691	0.8717	0.8738	0.8756	0.8775
Sheffield	0.7533	0.7588	0.7644	0.7702	0.7760	0.7813	0.7864	0.7919	0.7983	0.8051	0.8119	0.8184	0.8231	0.8264	0.8308	0.8348	0.8384	0.8430	0.8475	0.8505	0.8530	0.8547	0.8572	0.8605	0.8629	0.8648	0.8665
Wakefield	0.7331	0.7383	0.7437	0.7495	0.7557	0.7610	0.7657	0.7709	0.7769	0.7843	0.7915	0.7971	0.7999	0.8015	0.8040	0.8058	0.8067	0.8076	0.8090	0.8100	0.8124	0.8153	0.8198	0.8250	0.8282	0.8306	0.8325
Rotherham	0.7200	0.7251	0.7307	0.7370	0.7434	0.7487	0.7532	0.7580	0.7634	0.7693	0.7757	0.7813	0.7850	0.7877	0.7912	0.7937	0.7949	0.7966	0.7987	0.8008	0.8040	0.8066	0.8094	0.8132	0.8162	0.8182	0.8200
Doncaster	0.7162	0.7205	0.7252	0.7303	0.7358	0.7409	0.7453	0.7505	0.7564	0.7626	0.7689	0.7739	0.7770	0.7790	0.7821	0.7849	0.7871	0.7899	0.7930	0.7959	0.8005	0.8046	0.8090	0.8135	0.8162	0.8177	0.8190
Kingston upon Hull, City of	0.7245	0.7315	0.7395	0.7470	0.7540	0.7594	0.7643	0.7697	0.7759	0.7827	0.7907	0.7966	0.8004	0.8035	0.8077	0.8122	0.8168	0.8215	0.8250	0.8261	0.8254	0.8245	0.8268	0.8312	0.8348	0.8376	0.8399
Barnsley	0.7121	0.7176	0.7238	0.7304	0.7367	0.7418	0.7468	0.7528	0.7596	0.7660	0.7718	0.7760	0.7782	0.7796	0.7822	0.7842	0.7860	0.7886	0.7907	0.7916	0.7939	0.7966	0.8009	0.8064	0.8099	0.8121	0.8139
Northamptonshire	0.7512	0.7572	0.7639	0.7706	0.7773	0.7832	0.7883	0.7934	0.7986	0.8048	0.8111	0.8156	0.8177	0.8189	0.8214	0.8235	0.8249	0.8265	0.8282	0.8296	0.8325	0.8353	0.8387	0.8429	0.8448	0.8454	0.8458
Leicestershire	0.7602	0.7649	0.7705	0.7762	0.7820	0.7873	0.7924	0.7981	0.8041	0.8100	0.8160	0.8208	0.8234	0.8256	0.8293	0.8328	0.8357	0.8390	0.8416	0.8431	0.8450	0.8468	0.8501	0.8542	0.8571	0.8592	0.8608
Lincolnshire	0.7411	0.7443	0.7488	0.7545	0.7613	0.7677	0.7732	0.7786	0.7842	0.7900	0.7960	0.8016	0.8051	0.8077	0.8100	0.8108	0.8108	0.8108	0.8119	0.8131	0.8160	0.8192	0.8233	0.8280	0.8306	0.8320	0.8333
Rutland	0.7707	0.7725	0.7765	0.7844	0.7901	0.7956	0.8007	0.8057	0.8106	0.8153	0.8201	0.8137	0.8016	0.7815	0.7759	0.7878	0.7972	0.8036	0.8087	0.8169	0.8306	0.8390	0.8392	0.8385	0.8384	0.8388	0.8397
Derby	0.7481	0.7555	0.7643	0.7735	0.7821	0.7887	0.7938	0.7984	0.8032	0.8098	0.8163	0.8219	0.8265	0.8294	0.8335	0.8366	0.8382	0.8408	0.8439	0.8451	0.8456	0.8461	0.8493	0.8546	0.8584	0.8615	0.8642
Derbyshire	0.7368	0.7415	0.7473	0.7536	0.7603	0.7663	0.7718	0.7775	0.7835	0.7903	0.7961	0.8006	0.8032	0.8047	0.8074	0.8098	0.8118	0.8137	0.8154	0.8161	0.8184	0.8217	0.8264	0.8320	0.8359	0.8386	0.8407
Nottinghamshire	0.7351	0.7399	0.7453	0.7510	0.7568	0.7616	0.7661	0.7712	0.7777	0.7859	0.7941	0.8004	0.8045	0.8073	0.8106	0.8137	0.8157	0.8173	0.8186	0.8189	0.8196	0.8211	0.8242	0.8285	0.8314	0.8335	0.8354
Nottingham	0.7601	0.7690	0.7774	0.7855	0.7933	0.8005	0.8072	0.8148	0.8224	0.8283	0.8341	0.8402	0.8462	0.8499	0.8554	0.8602	0.8633	0.8666	0.8698	0.8711	0.8710	0.8703	0.8713	0.8734	0.8751	0.8766	0.8780
Leicester	0.7330	0.7439	0.7532	0.7613	0.7684	0.7738	0.7777	0.7815	0.7861	0.7925	0.8005	0.8073	0.8114	0.8141	0.8184	0.8219	0.8249	0.8280	0.8314	0.8336	0.8357	0.8373	0.8397	0.8426	0.8449	0.8471	0.8491
Warwickshire	0.7659	0.7716	0.7781	0.7844	0.7906	0.7958	0.8007	0.8062	0.8119	0.8185	0.8246	0.8299	0.8335	0.8358	0.8383	0.8399	0.8407	0.8418	0.8437	0.8455	0.8488	0.8522	0.8563	0.8607	0.8630	0.8643	0.8654
Herefordshire, County of	0.7408	0.7455	0.7520	0.7590	0.7654	0.7703	0.7748	0.7803	0.7860	0.7935	0.8011	0.8067	0.8091	0.8110	0.8135	0.8149	0.8148	0.8144	0.8149	0.8160	0.8211	0.8281	0.8355	0.8420	0.8460	0.8483	0.8499
Solihull	0.7681	0.7727	0.7793	0.7867	0.7941	0.8007	0.8070	0.8136	0.8199	0.8256	0.8301	0.8338	0.8364	0.8388	0.8424	0.8461	0.8488	0.8511	0.8518	0.8513	0.8517	0.8533	0.8574	0.8611	0.8630	0.8636	0.8645
Shropshire	0.7461	0.7498	0.7551	0.7611	0.7677	0.7733	0.7782	0.7834	0.7896	0.7960	0.8016	0.8053	0.8076	0.8103	0.8145	0.8173	0.8177	0.8181	0.8203	0.8236	0.8289	0.8342	0.8388	0.8430	0.8451	0.8461	0.8471
Worcestershire	0.7419	0.7467	0.7526	0.7587	0.7649	0.7702	0.7750	0.7804	0.7871	0.7956	0.8042	0.8102	0.8129	0.8152	0.8188	0.8210	0.8224	0.8231	0.8242	0.8252	0.8281	0.8315	0.8359	0.8408	0.8439	0.8457	0.8471
Staffordshire	0.7438	0.7494	0.7556	0.7621	0.7681	0.7731	0.7777	0.7832	0.7896	0.7958	0.8017	0.8061	0.8087	0.8100	0.8130	0.8161	0.8183	0.8208	0.8237	0.8259	0.8289	0.8318	0.8347	0.8385	0.8408	0.8422	0.8433
Dudley	0.7310	0.7360	0.7414	0.7470	0.7525	0.7574	0.7620	0.7670	0.7725	0.7783	0.7840	0.7888	0.7923	0.7942	0.7972	0.8001	0.8020	0.8039	0.8055	0.8060	0.8071	0.8081	0.8101	0.8135	0.8157	0.8171	0.8183
Coventry	0.7496	0.7573	0.7643	0.7710	0.7769	0.7820	0.7869	0.7919	0.7974	0.8047	0.8122	0.8189	0.8232	0.8262	0.8300	0.8332	0.8347	0.8358	0.8371	0.8379	0.8399	0.8429	0.8483	0.8550	0.8598	0.8634	0.8660
Telford and Wrekin	0.7519	0.7573	0.7631	0.7689	0.7747	0.7801	0.7853	0.7918	0.7983	0.8044	0.8104	0.8150	0.8172	0.8171	0.8177	0.8179	0.8176	0.8185	0.8205	0.8217	0.8242	0.8276	0.8326	0.8383	0.8422	0.8446	0.8463
Stoke-on-Trent	0.7238	0.7334	0.7416	0.7488	0.7556	0.7610	0.7660	0.7716	0.7775	0.7828	0.7877	0.7898	0.7890	0.7879	0.7891	0.7909	0.7912	0.7904	0.7927	0.7963	0.8004	0.8039	0.8092	0.8150	0.8193	0.8233	0.8268
Walsall	0.7177	0.7223	0.7282	0.7351	0.7409	0.7443	0.7458	0.7475	0.7516	0.7586	0.7661	0.7712	0.7730	0.7732	0.7761	0.7790	0.7813	0.7837	0.7861	0.7881	0.7921	0.7959	0.8000	0.8045	0.8067	0.8079	0.8088
Wolverhampton	0.7279	0.7341	0.7405	0.7470	0.7527	0.7572	0.7609	0.7655	0.7720	0.7802	0.7884	0.7947	0.7985	0.8002	0.8033	0.8056	0.8068	0.8079	0.8093	0.8100	0.8127	0.8161	0.8205	0.8249	0.8273	0.8288	0.8301
Birmingham	0.7332	0.7416	0.7498	0.7570	0.7627	0.7670	0.7708	0.7752	0.7811	0.7882	0.7950	0.8012	0.8049	0.8080	0.8125	0.8166	0.8191	0.8224	0.8262	0.8291	0.8316	0.8335	0.8362	0.8405	0.8433	0.8452	0.8467
Sandwell	0.7195	0.7255	0.7319	0.7386	0.7446	0.7493	0.7532	0.7576	0.7627	0.7677	0.7734	0.7778	0.7806	0.7819	0.7847	0.7869	0.7879	0.7888	0.7898	0.7911	0.7942	0.7975	0.8027	0.8083	0.8116	0.8132	0.8146
Bedford	0.7707	0.7761	0.7807	0.7841	0.7877	0.7913	0.7955	0.8008	0.8071	0.8143	0.8220	0.8278	0.8312	0.8329	0.8364	0.8393	0.8407	0.8422	0.8430	0.8438	0.8451	0.8463	0.8478	0.8499	0.8505	0.8506	0.8511
Central Bedfordshire	0.7646	0.7699	0.7753	0.7810	0.7875	0.7942	0.8002	0.8057	0.8106	0.8146	0.8180	0.8204	0.8220	0.8233	0.8265	0.8292	0.8300	0.8306	0.8309	0.8306	0.8315	0.8338	0.8381	0.8438	0.8477	0.8504	0.8525
Suffolk	0.7570	0.7631	0.7682	0.7735	0.7795	0.7856	0.7910	0.7967	0.8021	0.8069	0.8113	0.8136	0.8140	0.8144	0.8174	0.8202	0.8220	0.8228	0.8239	0.8247	0.8270	0.8297	0.8331	0.8369	0.8384	0.8391	0.8396
Hertfordshire	0.7814	0.7884	0.7948	0.8005	0.8064	0.8117	0.8165	0.8219	0.8284	0.8359	0.8430	0.8482	0.8512	0.8526	0.8553	0.8576	0.8581	0.8589	0.8608	0.8621	0.8641	0.8661	0.8694	0.8737	0.8763	0.8779	0.8793
Essex	0.7504	0.7566	0.7626	0.7681	0.7739	0.7793	0.7844	0.7900	0.7959	0.8022	0.8080	0.8123	0.8143	0.8161	0.8194	0.8228	0.8253	0.8279	0.8297	0.8302	0.8314	0.8329	0.8358	0.8397	0.8423	0.8440	0.8454
Cambridgeshire	0.7828	0.7893	0.7955	0.8013	0.8074	0.8137	0.8203	0.8274	0.8331	0.8384	0.8438	0.8492	0.8540	0.8565	0.8596	0.8618	0.8623	0.8620	0.8623	0.8633	0.8665	0.8700	0.8739	0.8780	0.8802	0.8814	0.8825
Thurrock	0.7498	0.7586	0.7654	0.7705	0.7754	0.7795	0.7831	0.7871	0.7933	0.7995	0.8063	0.8111	0.8125	0.8122	0.8130	0.8130	0.8131	0.8139	0.8165	0.8189	0.8205	0.8220	0.8230	0.8254	0.8256	0.8247	0.8242
Norfolk	0.7541	0.7594	0.7645	0.7696	0.7752	0.7807	0.7860	0.7917	0.7976	0.8042	0.8100	0.8143	0.8166	0.8180	0.8211	0.8242	0.8265	0.8282	0.8299	0.8307	0.8318	0.8330	0.8361	0.8406	0.8439	0.8463	0.8484
Southend-on-Sea	0.7359	0.7422	0.7494	0.7569	0.7647	0.7718	0.7782	0.7848	0.7903	0.7943	0.7964	0.7965	0.7977	0.7994	0.8023	0.8035	0.8039	0.8057	0.8094	0.8133	0.8171	0.8192	0.8215	0.8248	0.8267	0.8275	0.8284
Peterborough	0.7629	0.7675	0.7727	0.7781	0.7848	0.7904	0.7943	0.7980	0.8026	0.8092	0.8151	0.8186	0.8185	0.8163	0.8162	0.8164	0.8164	0.8163	0.8159	0.8175	0.8231	0.8276	0.8308	0.8347	0.8369	0.8382	0.8393
Luton	0.7426	0.7520	0.7593	0.7652	0.7707	0.7752	0.7791	0.7832	0.7875	0.7927	0.7984	0.8027	0.8040	0.8041	0.8058	0.8085	0.8112	0.8159	0.8228	0.8288	0.8340	0.8371	0.8396	0.8428	0.8442	0.8444	0.8445
Richmond upon Thames	0.8129	0.8202	0.8268	0.8330	0.8389	0.8446	0.8498	0.8553	0.8611	0.8676	0.8731	0.8784	0.8822	0.8846	0.8872	0.8875	0.8867	0.8867	0.8882	0.8884	0.8893	0.8908	0.8946	0.9004	0.9048	0.9077	0.9102
Kensington and Chelsea	0.8552	0.8607	0.8660	0.8710	0.8749	0.8796	0.8841	0.8888	0.8936	0.8998	0.9059	0.9104	0.9141	0.9173	0.9205	0.9239	0.9267	0.9291	0.9317	0.9341	0.9364	0.9385	0.9396	0.9409	0.9421	0.9431	0.9445

Appendix Table 5B. SDI Values by location, 1990-2016																											
**Location**	**1990**	**1991**	**1992**	**1993**	**1994**	**1995**	**1996**	**1997**	**1998**	**1999**	**2000**	**2001**	**2002**	**2003**	**2004**	**2005**	**2006**	**2007**	**2008**	**2009**	**2010**	**2011**	**2012**	**2013**	**2014**	**2015**	**2016**
Barnet	0.7871	0.7923	0.7973	0.8029	0.8096	0.8163	0.8229	0.8294	0.8354	0.8410	0.8462	0.8511	0.8531	0.8541	0.8554	0.8551	0.8540	0.8534	0.8547	0.8567	0.8603	0.8646	0.8699	0.8749	0.8778	0.8791	0.8802
Westminster	0.8529	0.8603	0.8649	0.8692	0.8727	0.8772	0.8819	0.8864	0.8910	0.8963	0.9014	0.9059	0.9100	0.9136	0.9175	0.9213	0.9243	0.9267	0.9288	0.9306	0.9326	0.9346	0.9364	0.9379	0.9395	0.9411	0.9426
Bromley	0.7792	0.7841	0.7886	0.7933	0.7987	0.8037	0.8088	0.8147	0.8213	0.8289	0.8347	0.8390	0.8417	0.8435	0.8460	0.8473	0.8483	0.8498	0.8510	0.8510	0.8507	0.8505	0.8532	0.8570	0.8598	0.8616	0.8631
Bexley	0.7490	0.7556	0.7621	0.7684	0.7739	0.7780	0.7811	0.7843	0.7889	0.7946	0.8007	0.8060	0.8093	0.8112	0.8136	0.8148	0.8154	0.8167	0.8193	0.8211	0.8227	0.8240	0.8278	0.8329	0.8362	0.8382	0.8398
Redbridge	0.7568	0.7615	0.7666	0.7720	0.7774	0.7823	0.7869	0.7918	0.7972	0.8037	0.8098	0.8148	0.8182	0.8200	0.8221	0.8232	0.8245	0.8267	0.8297	0.8304	0.8307	0.8311	0.8336	0.8373	0.8398	0.8413	0.8428
Merton	0.7818	0.7919	0.7999	0.8069	0.8132	0.8182	0.8228	0.8285	0.8360	0.8447	0.8518	0.8567	0.8593	0.8608	0.8635	0.8652	0.8661	0.8672	0.8692	0.8696	0.8699	0.8706	0.8740	0.8785	0.8811	0.8823	0.8836
Brent	0.7838	0.7882	0.7924	0.7971	0.8024	0.8067	0.8105	0.8147	0.8198	0.8261	0.8340	0.8410	0.8446	0.8458	0.8472	0.8465	0.8425	0.8362	0.8320	0.8325	0.8402	0.8498	0.8582	0.8639	0.8656	0.8655	0.8655
Hillingdon	0.8074	0.8138	0.8205	0.8274	0.8341	0.8395	0.8442	0.8483	0.8534	0.8592	0.8636	0.8664	0.8680	0.8689	0.8706	0.8709	0.8702	0.8704	0.8723	0.8742	0.8759	0.8780	0.8822	0.8879	0.8919	0.8947	0.8968
Havering	0.7504	0.7561	0.7618	0.7677	0.7736	0.7785	0.7827	0.7870	0.7920	0.7981	0.8035	0.8076	0.8103	0.8120	0.8151	0.8181	0.8204	0.8221	0.8248	0.8267	0.8282	0.8287	0.8305	0.8330	0.8347	0.8359	0.8372
Kingston upon Thames	0.8039	0.8113	0.8179	0.8233	0.8277	0.8323	0.8368	0.8424	0.8496	0.8585	0.8639	0.8690	0.8738	0.8778	0.8811	0.8836	0.8859	0.8880	0.8908	0.8916	0.8920	0.8922	0.8949	0.8982	0.8999	0.9009	0.9017
Sutton	0.7623	0.7694	0.7762	0.7829	0.7898	0.7960	0.8016	0.8073	0.8137	0.8210	0.8277	0.8324	0.8341	0.8343	0.8361	0.8379	0.8401	0.8423	0.8452	0.8462	0.8468	0.8475	0.8502	0.8535	0.8556	0.8570	0.8585
Harrow	0.7719	0.7815	0.7894	0.7964	0.8020	0.8060	0.8096	0.8143	0.8210	0.8291	0.8356	0.8396	0.8410	0.8415	0.8443	0.8465	0.8473	0.8481	0.8492	0.8495	0.8511	0.8519	0.8536	0.8557	0.8561	0.8556	0.8556
Enfield	0.7669	0.7728	0.7783	0.7831	0.7867	0.7900	0.7932	0.7977	0.8037	0.8118	0.8199	0.8260	0.8285	0.8281	0.8279	0.8271	0.8262	0.8259	0.8280	0.8311	0.8367	0.8418	0.8468	0.8511	0.8530	0.8534	0.8537
Croydon	0.7718	0.7796	0.7874	0.7945	0.8009	0.8061	0.8103	0.8146	0.8198	0.8259	0.8317	0.8363	0.8394	0.8411	0.8430	0.8427	0.8412	0.8402	0.8409	0.8415	0.8435	0.8452	0.8476	0.8498	0.8502	0.8493	0.8488
Hammersmith and Fulham	0.8490	0.8564	0.8616	0.8668	0.8721	0.8772	0.8827	0.8871	0.8913	0.8970	0.9014	0.9057	0.9094	0.9124	0.9158	0.9191	0.9205	0.9216	0.9238	0.9274	0.9322	0.9357	0.9372	0.9385	0.9397	0.9408	0.9424
Ealing	0.7952	0.7998	0.8049	0.8104	0.8163	0.8219	0.8274	0.8329	0.8394	0.8472	0.8535	0.8580	0.8601	0.8602	0.8609	0.8604	0.8590	0.8575	0.8573	0.8570	0.8592	0.8630	0.8678	0.8726	0.8755	0.8773	0.8791
Greenwich	0.7527	0.7602	0.7681	0.7750	0.7804	0.7845	0.7882	0.7927	0.7983	0.8059	0.8140	0.8208	0.8249	0.8263	0.8264	0.8242	0.8214	0.8203	0.8220	0.8246	0.8296	0.8348	0.8407	0.8458	0.8476	0.8476	0.8477
Wandsworth	0.8158	0.8248	0.8328	0.8387	0.8440	0.8490	0.8544	0.8597	0.8653	0.8724	0.8789	0.8851	0.8903	0.8946	0.8985	0.9021	0.9057	0.9072	0.9095	0.9106	0.9122	0.9146	0.9189	0.9222	0.9243	0.9261	0.9284
Waltham Forest	0.7391	0.7462	0.7529	0.7598	0.7660	0.7705	0.7738	0.7775	0.7826	0.7896	0.7970	0.8034	0.8062	0.8065	0.8068	0.8052	0.8017	0.7982	0.7975	0.7997	0.8066	0.8143	0.8216	0.8282	0.8318	0.8337	0.8352
Camden	0.8574	0.8621	0.8669	0.8719	0.8759	0.8806	0.8825	0.8844	0.8884	0.8972	0.9043	0.9088	0.9122	0.9152	0.9186	0.9219	0.9244	0.9268	0.9289	0.9310	0.9330	0.9352	0.9372	0.9389	0.9404	0.9419	0.9434
Lambeth	0.7975	0.8113	0.8213	0.8283	0.8338	0.8380	0.8420	0.8468	0.8531	0.8605	0.8667	0.8710	0.8720	0.8727	0.8765	0.8809	0.8852	0.8908	0.8977	0.9033	0.9085	0.9137	0.9187	0.9206	0.9222	0.9237	0.9255
Lewisham	0.7552	0.7661	0.7749	0.7818	0.7869	0.7901	0.7928	0.7963	0.8019	0.8101	0.8188	0.8257	0.8278	0.8275	0.8285	0.8300	0.8315	0.8330	0.8348	0.8362	0.8396	0.8439	0.8496	0.8561	0.8603	0.8628	0.8646
Hounslow	0.7949	0.8046	0.8105	0.8147	0.8184	0.8219	0.8250	0.8294	0.8364	0.8465	0.8568	0.8638	0.8655	0.8638	0.8640	0.8652	0.8667	0.8688	0.8720	0.8735	0.8759	0.8783	0.8831	0.8886	0.8909	0.8914	0.8920
Southwark	0.8157	0.8254	0.8327	0.8378	0.8411	0.8439	0.8450	0.8476	0.8535	0.8639	0.8746	0.8826	0.8850	0.8851	0.8877	0.8919	0.8963	0.9004	0.9043	0.9075	0.9114	0.9163	0.9227	0.9293	0.9329	0.9347	0.9366
Newham	0.7397	0.7475	0.7540	0.7585	0.7603	0.7592	0.7582	0.7603	0.7652	0.7752	0.7902	0.8035	0.8085	0.8082	0.8096	0.8109	0.8090	0.8025	0.7946	0.7950	0.8132	0.8330	0.8439	0.8499	0.8522	0.8526	0.8527
Barking and Dagenham	0.7332	0.7392	0.7454	0.7518	0.7585	0.7627	0.7646	0.7684	0.7752	0.7839	0.7925	0.7991	0.8007	0.7975	0.7940	0.7898	0.7870	0.7873	0.7913	0.7944	0.7980	0.7997	0.8036	0.8102	0.8138	0.8146	0.8152
Haringey	0.7771	0.7902	0.7989	0.8050	0.8094	0.8129	0.8166	0.8209	0.8252	0.8310	0.8373	0.8435	0.8465	0.8471	0.8492	0.8509	0.8504	0.8486	0.8476	0.8480	0.8554	0.8647	0.8724	0.8775	0.8796	0.8801	0.8804
Hackney	0.8058	0.8136	0.8218	0.8282	0.8317	0.8315	0.8293	0.8291	0.8341	0.8441	0.8547	0.8629	0.8657	0.8660	0.8683	0.8712	0.8750	0.8807	0.8873	0.8937	0.9019	0.9095	0.9155	0.9203	0.9226	0.9233	0.9239
Islington	0.8369	0.8437	0.8507	0.8576	0.8638	0.8689	0.8733	0.8772	0.8818	0.8882	0.8936	0.8980	0.9016	0.9051	0.9086	0.9122	0.9154	0.9186	0.9217	0.9246	0.9272	0.9299	0.9323	0.9343	0.9363	0.9382	0.9401
Tower Hamlets	0.7938	0.7974	0.8010	0.8048	0.8091	0.8135	0.8187	0.8255	0.8342	0.8471	0.8594	0.8695	0.8756	0.8795	0.8854	0.8909	0.8962	0.9019	0.9075	0.9118	0.9155	0.9191	0.9238	0.9270	0.9289	0.9306	0.9328
Wokingham	0.8086	0.8104	0.8124	0.8145	0.8173	0.8203	0.8236	0.8286	0.8358	0.8458	0.8566	0.8649	0.8695	0.8719	0.8754	0.8791	0.8813	0.8826	0.8838	0.8833	0.8818	0.8803	0.8820	0.8869	0.8905	0.8932	0.8954
Buckinghamshire	0.7903	0.7955	0.8010	0.8062	0.8121	0.8179	0.8230	0.8282	0.8336	0.8393	0.8452	0.8491	0.8503	0.8509	0.8538	0.8558	0.8562	0.8559	0.8562	0.8565	0.8583	0.8608	0.8647	0.8690	0.8716	0.8731	0.8743
Surrey	0.7931	0.8001	0.8060	0.8111	0.8161	0.8209	0.8255	0.8309	0.8375	0.8460	0.8536	0.8589	0.8613	0.8628	0.8649	0.8664	0.8671	0.8680	0.8691	0.8697	0.8711	0.8724	0.8755	0.8798	0.8824	0.8842	0.8857
Windsor and Maidenhead	0.7988	0.8089	0.8171	0.8237	0.8303	0.8365	0.8414	0.8456	0.8510	0.8561	0.8614	0.8653	0.8685	0.8718	0.8762	0.8790	0.8799	0.8811	0.8827	0.8837	0.8854	0.8877	0.8912	0.8964	0.9001	0.9025	0.9046
West Berkshire	0.8086	0.8139	0.8202	0.8266	0.8329	0.8381	0.8422	0.8463	0.8513	0.8583	0.8646	0.8685	0.8694	0.8690	0.8691	0.8685	0.8661	0.8631	0.8619	0.8632	0.8677	0.8727	0.8781	0.8827	0.8849	0.8863	0.8873
Hampshire	0.7757	0.7811	0.7862	0.7913	0.7971	0.8027	0.8080	0.8133	0.8187	0.8245	0.8299	0.8340	0.8362	0.8369	0.8391	0.8408	0.8415	0.8424	0.8437	0.8448	0.8473	0.8504	0.8545	0.8594	0.8624	0.8643	0.8658
Bracknell Forest	0.7956	0.7993	0.8036	0.8084	0.8143	0.8196	0.8240	0.8280	0.8335	0.8416	0.8493	0.8548	0.8580	0.8599	0.8631	0.8664	0.8683	0.8699	0.8713	0.8717	0.8729	0.8746	0.8777	0.8816	0.8846	0.8867	0.8885
West Sussex	0.7685	0.7743	0.7800	0.7855	0.7916	0.7975	0.8028	0.8084	0.8139	0.8194	0.8239	0.8273	0.8284	0.8285	0.8305	0.8323	0.8338	0.8350	0.8365	0.8380	0.8409	0.8437	0.8474	0.8518	0.8539	0.8551	0.8561
Oxfordshire	0.7944	0.7995	0.8048	0.8101	0.8164	0.8222	0.8272	0.8324	0.8383	0.8450	0.8513	0.8559	0.8584	0.8604	0.8632	0.8653	0.8656	0.8658	0.8665	0.8675	0.8704	0.8743	0.8791	0.8840	0.8869	0.8886	0.8900
Reading	0.8061	0.8149	0.8219	0.8273	0.8323	0.8374	0.8420	0.8472	0.8531	0.8608	0.8690	0.8760	0.8800	0.8824	0.8848	0.8858	0.8837	0.8825	0.8837	0.8856	0.8895	0.8931	0.8970	0.9014	0.9039	0.9052	0.9065
Kent	0.7547	0.7601	0.7654	0.7705	0.7762	0.7815	0.7862	0.7913	0.7969	0.8029	0.8083	0.8125	0.8146	0.8160	0.8190	0.8215	0.8226	0.8239	0.8256	0.8267	0.8290	0.8315	0.8350	0.8392	0.8420	0.8437	0.8452
Brighton and Hove	0.7810	0.7904	0.7974	0.8026	0.8079	0.8132	0.8185	0.8240	0.8291	0.8339	0.8393	0.8452	0.8511	0.8569	0.8621	0.8671	0.8719	0.8743	0.8769	0.8799	0.8839	0.8870	0.8891	0.8913	0.8938	0.8964	0.8991
Medway	0.7383	0.7436	0.7485	0.7530	0.7582	0.7634	0.7686	0.7740	0.7797	0.7852	0.7911	0.7957	0.7988	0.8010	0.8044	0.8075	0.8097	0.8110	0.8125	0.8134	0.8148	0.8163	0.8198	0.8242	0.8270	0.8290	0.8307
East Sussex	0.7456	0.7500	0.7546	0.7592	0.7647	0.7703	0.7755	0.7808	0.7858	0.7911	0.7961	0.7994	0.8003	0.8006	0.8031	0.8055	0.8071	0.8080	0.8092	0.8102	0.8131	0.8167	0.8211	0.8262	0.8298	0.8323	0.8344
Portsmouth	0.7762	0.7844	0.7913	0.7969	0.8020	0.8064	0.8105	0.8150	0.8209	0.8277	0.8358	0.8434	0.8487	0.8526	0.8575	0.8614	0.8643	0.8669	0.8693	0.8700	0.8700	0.8682	0.8685	0.8710	0.8734	0.8758	0.8780
Isle of Wight	0.7438	0.7461	0.7483	0.7512	0.7560	0.7617	0.7678	0.7743	0.7803	0.7867	0.7955	0.8037	0.8086	0.8108	0.8123	0.8133	0.8144	0.8161	0.8183	0.8190	0.8206	0.8227	0.8257	0.8291	0.8313	0.8330	0.8348
Milton Keynes	0.7964	0.7990	0.8041	0.8107	0.8181	0.8247	0.8301	0.8349	0.8391	0.8435	0.8474	0.8496	0.8498	0.8504	0.8532	0.8554	0.8558	0.8554	0.8553	0.8564	0.8618	0.8680	0.8731	0.8779	0.8799	0.8799	0.8799
Southampton	0.7708	0.7816	0.7903	0.7975	0.8041	0.8096	0.8144	0.8192	0.8248	0.8316	0.8396	0.8447	0.8491	0.8537	0.8584	0.8628	0.8663	0.8681	0.8688	0.8681	0.8669	0.8659	0.8673	0.8708	0.8736	0.8761	0.8782
Slough	0.7952	0.8045	0.8119	0.8178	0.8243	0.8304	0.8358	0.8404	0.8439	0.8473	0.8517	0.8549	0.8542	0.8507	0.8484	0.8460	0.8433	0.8448	0.8500	0.8552	0.8609	0.8661	0.8706	0.8744	0.8748	0.8737	0.8731
South Gloucestershire	0.7829	0.7869	0.7910	0.7949	0.7991	0.8033	0.8086	0.8158	0.8239	0.8317	0.8389	0.8440	0.8464	0.8478	0.8510	0.8550	0.8581	0.8614	0.8646	0.8659	0.8673	0.8685	0.8707	0.8748	0.8777	0.8798	0.8815
Dorset	0.7540	0.7572	0.7618	0.7677	0.7748	0.7815	0.7875	0.7941	0.8007	0.8069	0.8115	0.8133	0.8136	0.8134	0.8149	0.8160	0.8154	0.8141	0.8140	0.8157	0.8212	0.8273	0.8325	0.8370	0.8394	0.8407	0.8416
Wiltshire	0.7656	0.7704	0.7755	0.7806	0.7868	0.7929	0.7982	0.8039	0.8096	0.8155	0.8209	0.8247	0.8265	0.8270	0.8289	0.8294	0.8278	0.8260	0.8253	0.8260	0.8304	0.8353	0.8402	0.8440	0.8456	0.8461	0.8467
North Somerset	0.7533	0.7564	0.7606	0.7658	0.7712	0.7764	0.7814	0.7866	0.7929	0.7990	0.8046	0.8086	0.8111	0.8133	0.8171	0.8211	0.8234	0.8253	0.8267	0.8264	0.8271	0.8287	0.8327	0.8381	0.8411	0.8427	0.8441
Devon	0.7527	0.7574	0.7623	0.7672	0.7723	0.7769	0.7816	0.7875	0.7943	0.8019	0.8090	0.8146	0.8182	0.8209	0.8252	0.8290	0.8315	0.8332	0.8344	0.8346	0.8356	0.8370	0.8404	0.8454	0.8489	0.8514	0.8534
Poole	0.7662	0.7710	0.7760	0.7817	0.7889	0.7959	0.8020	0.8082	0.8140	0.8200	0.8263	0.8299	0.8305	0.8304	0.8321	0.8343	0.8352	0.8354	0.8355	0.8361	0.8396	0.8451	0.8510	0.8561	0.8591	0.8610	0.8625
Bath and North East Somerset	0.7719	0.7766	0.7816	0.7869	0.7929	0.7983	0.8028	0.8084	0.8156	0.8240	0.8305	0.8368	0.8430	0.8493	0.8552	0.8605	0.8633	0.8666	0.8696	0.8705	0.8714	0.8719	0.8743	0.8785	0.8816	0.8840	0.8859
Gloucestershire	0.7697	0.7745	0.7795	0.7846	0.7903	0.7957	0.8008	0.8062	0.8116	0.8185	0.8260	0.8316	0.8353	0.8383	0.8420	0.8437	0.8438	0.8444	0.8453	0.8462	0.8486	0.8513	0.8548	0.8586	0.8607	0.8618	0.8629
Somerset	0.7523	0.7565	0.7617	0.7674	0.7743	0.7805	0.7855	0.7905	0.7953	0.8013	0.8070	0.8110	0.8124	0.8132	0.8160	0.8184	0.8195	0.8196	0.8199	0.8205	0.8232	0.8267	0.8304	0.8339	0.8351	0.8353	0.8356
Swindon	0.7896	0.7958	0.8012	0.8059	0.8104	0.8146	0.8188	0.8245	0.8312	0.8380	0.8442	0.8490	0.8510	0.8507	0.8515	0.8519	0.8515	0.8526	0.8549	0.8563	0.8576	0.8591	0.8614	0.8649	0.8661	0.8664	0.8669
Torbay	0.7382	0.7433	0.7485	0.7545	0.7612	0.7668	0.7716	0.7767	0.7819	0.7875	0.7927	0.7952	0.7938	0.7924	0.7943	0.7966	0.7984	0.8001	0.8022	0.8022	0.8017	0.8011	0.8026	0.8059	0.8086	0.8108	0.8129
Bristol, City of	0.7870	0.7961	0.8038	0.8106	0.8168	0.8221	0.8267	0.8323	0.8388	0.8467	0.8536	0.8588	0.8633	0.8653	0.8687	0.8722	0.8749	0.8784	0.8812	0.8818	0.8811	0.8798	0.8816	0.8865	0.8911	0.8950	0.8981

Appendix Table 5B. SDI Values by location, 1990-2016																											
**Location**	**1990**	**1991**	**1992**	**1993**	**1994**	**1995**	**1996**	**1997**	**1998**	**1999**	**2000**	**2001**	**2002**	**2003**	**2004**	**2005**	**2006**	**2007**	**2008**	**2009**	**2010**	**2011**	**2012**	**2013**	**2014**	**2015**	**2016**
Bournemouth	0.7587	0.7673	0.7751	0.7805	0.7859	0.7913	0.7968	0.8025	0.8084	0.8137	0.8202	0.8260	0.8313	0.8367	0.8415	0.8468	0.8464	0.8465	0.8474	0.8494	0.8532	0.8577	0.8619	0.8663	0.8689	0.8706	0.8722
Cornwall	0.7391	0.7434	0.7484	0.7541	0.7604	0.7659	0.7705	0.7760	0.7822	0.7888	0.7957	0.8009	0.8048	0.8084	0.8120	0.8149	0.8175	0.8194	0.8210	0.8215	0.8219	0.8227	0.8257	0.8297	0.8320	0.8335	0.8348
Plymouth	0.7567	0.7623	0.7695	0.7765	0.7826	0.7886	0.7933	0.7985	0.8040	0.8098	0.8151	0.8194	0.8216	0.8232	0.8268	0.8303	0.8329	0.8361	0.8391	0.8398	0.8401	0.8406	0.8430	0.8472	0.8498	0.8513	0.8529
North East England	0.7372	0.7426	0.7488	0.7554	0.7620	0.7678	0.7732	0.7789	0.7852	0.7919	0.7985	0.8037	0.8070	0.8094	0.8130	0.8164	0.8189	0.8215	0.8241	0.8259	0.8280	0.8303	0.8336	0.8378	0.8405	0.8424	0.8441
North West England	0.7457	0.7519	0.7588	0.7659	0.7725	0.7779	0.7827	0.7879	0.7939	0.8006	0.8073	0.8126	0.8157	0.8177	0.8208	0.8234	0.8247	0.8260	0.8278	0.8296	0.8327	0.8361	0.8403	0.8450	0.8479	0.8498	0.8514
Yorkshire and the Humber	0.7435	0.7492	0.7553	0.7614	0.7675	0.7727	0.7775	0.7828	0.7887	0.7954	0.8021	0.8075	0.8107	0.8128	0.8163	0.8196	0.8222	0.8251	0.8281	0.8300	0.8321	0.8339	0.8368	0.8406	0.8430	0.8447	0.8462
East Midlands	0.7459	0.7515	0.7577	0.7642	0.7708	0.7766	0.7818	0.7872	0.7931	0.7996	0.8061	0.8114	0.8145	0.8166	0.8198	0.8226	0.8245	0.8264	0.8284	0.8297	0.8316	0.8338	0.8371	0.8414	0.8441	0.8460	0.8476
West Midlands	0.7408	0.7470	0.7536	0.7602	0.7663	0.7712	0.7756	0.7807	0.7866	0.7932	0.7998	0.8049	0.8078	0.8097	0.8129	0.8156	0.8172	0.8189	0.8211	0.8230	0.8258	0.8288	0.8328	0.8374	0.8403	0.8422	0.8437
East of England	0.7623	0.7686	0.7745	0.7800	0.7859	0.7915	0.7967	0.8023	0.8083	0.8146	0.8203	0.8244	0.8265	0.8278	0.8307	0.8332	0.8347	0.8361	0.8377	0.8389	0.8410	0.8431	0.8462	0.8502	0.8526	0.8541	0.8554
Greater London	0.8067	0.8146	0.8214	0.8274	0.8327	0.8372	0.8413	0.8461	0.8521	0.8601	0.8681	0.8746	0.8781	0.8799	0.8828	0.8851	0.8856	0.8861	0.8877	0.8900	0.8942	0.8989	0.9042	0.9094	0.9123	0.9139	0.9149
South East England	0.7773	0.7832	0.7888	0.7941	0.7998	0.8054	0.8104	0.8157	0.8213	0.8278	0.8340	0.8386	0.8409	0.8423	0.8450	0.8471	0.8481	0.8490	0.8503	0.8513	0.8536	0.8561	0.8598	0.8642	0.8670	0.8687	0.8702
South West England	0.7628	0.7679	0.7732	0.7788	0.7847	0.7902	0.7951	0.8008	0.8070	0.8137	0.8202	0.8250	0.8276	0.8294	0.8325	0.8350	0.8363	0.8375	0.8388	0.8396	0.8415	0.8437	0.8470	0.8513	0.8540	0.8557	0.8572
Southern Latin America	0.6408	0.6473	0.6543	0.6619	0.6708	0.6780	0.6836	0.6883	0.6930	0.6976	0.7021	0.7062	0.7089	0.7120	0.7160	0.7202	0.7247	0.7293	0.7337	0.7379	0.7429	0.7487	0.7544	0.7597	0.7642	0.7681	0.7715
Argentina	0.6385	0.6452	0.6524	0.6602	0.6698	0.6766	0.6810	0.6840	0.6873	0.6910	0.6950	0.6985	0.7006	0.7029	0.7059	0.7098	0.7141	0.7186	0.7231	0.7272	0.7323	0.7385	0.7445	0.7500	0.7545	0.7582	0.7613
Chile	0.6446	0.6521	0.6601	0.6684	0.6766	0.6853	0.6939	0.7021	0.7096	0.7162	0.7220	0.7275	0.7319	0.7372	0.7436	0.7488	0.7538	0.7585	0.7629	0.7670	0.7717	0.7770	0.7822	0.7872	0.7918	0.7960	0.7998
Uruguay	0.6406	0.6425	0.6451	0.6479	0.6520	0.6561	0.6611	0.6669	0.6729	0.6780	0.6821	0.6854	0.6877	0.6900	0.6928	0.6963	0.7001	0.7043	0.7090	0.7138	0.7190	0.7244	0.7294	0.7343	0.7390	0.7433	0.7473
High-income North America	0.8521	0.8547	0.8575	0.8601	0.8626	0.8649	0.8670	0.8684	0.8696	0.8710	0.8733	0.8763	0.8776	0.8783	0.8798	0.8801	0.8797	0.8815	0.8854	0.8896	0.8929	0.8947	0.8962	0.8972	0.8983	0.8994	0.8999
Canada	0.8469	0.8494	0.8526	0.8554	0.8579	0.8622	0.8668	0.8703	0.8734	0.8775	0.8811	0.8839	0.8866	0.8886	0.8908	0.8919	0.8919	0.8923	0.8941	0.8967	0.8995	0.9014	0.9027	0.9040	0.9053	0.9066	0.9081
United States	0.8525	0.8551	0.8579	0.8604	0.8630	0.8650	0.8667	0.8679	0.8689	0.8700	0.8722	0.8752	0.8763	0.8769	0.8784	0.8785	0.8781	0.8801	0.8842	0.8886	0.8919	0.8938	0.8953	0.8963	0.8973	0.8985	0.8989
Greenland	0.7504	0.7498	0.7493	0.7491	0.7497	0.7502	0.7504	0.7530	0.7580	0.7617	0.7632	0.7638	0.7665	0.7706	0.7749	0.7795	0.7833	0.7858	0.7879	0.7916	0.7981	0.8042	0.8081	0.8105	0.8125	0.8144	0.8159
Alabama	0.8317	0.8355	0.8391	0.8424	0.8454	0.8475	0.8488	0.8497	0.8508	0.8526	0.8557	0.8600	0.8624	0.8638	0.8653	0.8650	0.8639	0.8653	0.8691	0.8738	0.8779	0.8801	0.8815	0.8821	0.8827	0.8835	0.8838
Alaska	0.8453	0.8461	0.8484	0.8522	0.8563	0.8594	0.8613	0.8625	0.8630	0.8629	0.8628	0.8631	0.8630	0.8644	0.8679	0.8706	0.8718	0.8740	0.8776	0.8813	0.8846	0.8871	0.8898	0.8914	0.8936	0.8958	0.8968
Arizona	0.8374	0.8395	0.8417	0.8438	0.8458	0.8470	0.8480	0.8484	0.8482	0.8478	0.8488	0.8515	0.8525	0.8529	0.8546	0.8551	0.8558	0.8606	0.8682	0.8749	0.8789	0.8803	0.8809	0.8810	0.8811	0.8813	0.8808
Arkansas	0.8221	0.8261	0.8300	0.8332	0.8356	0.8371	0.8386	0.8399	0.8416	0.8435	0.8460	0.8492	0.8505	0.8512	0.8527	0.8527	0.8522	0.8545	0.8588	0.8634	0.8667	0.8687	0.8708	0.8722	0.8736	0.8752	0.8760
California	0.8414	0.8430	0.8455	0.8481	0.8509	0.8538	0.8573	0.8605	0.8633	0.8656	0.8686	0.8719	0.8725	0.8721	0.8728	0.8726	0.8724	0.8750	0.8800	0.8853	0.8892	0.8911	0.8928	0.8942	0.8955	0.8972	0.8978
Colorado	0.8703	0.8725	0.8749	0.8777	0.8806	0.8826	0.8836	0.8833	0.8827	0.8822	0.8831	0.8853	0.8867	0.8881	0.8909	0.8922	0.8927	0.8951	0.8991	0.9030	0.9060	0.9080	0.9100	0.9116	0.9131	0.9144	0.9147
Connecticut	0.8892	0.8920	0.8946	0.8968	0.8988	0.9005	0.9018	0.9028	0.9038	0.9052	0.9075	0.9102	0.9110	0.9109	0.9118	0.9118	0.9115	0.9134	0.9170	0.9212	0.9243	0.9261	0.9277	0.9288	0.9296	0.9303	0.9301
Delaware	0.8671	0.8709	0.8745	0.8776	0.8801	0.8820	0.8829	0.8824	0.8818	0.8819	0.8838	0.8871	0.8883	0.8887	0.8899	0.8890	0.8873	0.8883	0.8921	0.8969	0.9007	0.9030	0.9053	0.9069	0.9081	0.9092	0.9094
District of Columbia	0.8739	0.8775	0.8833	0.8902	0.8978	0.9048	0.9113	0.9167	0.9210	0.9239	0.9266	0.9294	0.9296	0.9272	0.9260	0.9253	0.9248	0.9254	0.9278	0.9317	0.9352	0.9379	0.9409	0.9435	0.9457	0.9467	0.9475
Florida	0.8535	0.8572	0.8602	0.8629	0.8653	0.8674	0.8689	0.8697	0.8705	0.8711	0.8727	0.8753	0.8763	0.8766	0.8778	0.8779	0.8781	0.8812	0.8864	0.8915	0.8954	0.8971	0.8980	0.8981	0.8982	0.8985	0.8983
Georgia	0.8434	0.8477	0.8515	0.8546	0.8574	0.8594	0.8605	0.8605	0.8602	0.8601	0.8618	0.8655	0.8674	0.8683	0.8693	0.8683	0.8666	0.8689	0.8745	0.8802	0.8840	0.8859	0.8870	0.8874	0.8877	0.8882	0.8880
Hawaii	0.8540	0.8569	0.8597	0.8626	0.8660	0.8693	0.8723	0.8744	0.8753	0.8754	0.8755	0.8757	0.8741	0.8726	0.8738	0.8746	0.8746	0.8766	0.8805	0.8853	0.8892	0.8915	0.8938	0.8959	0.8980	0.9001	0.9009
Idaho	0.8330	0.8348	0.8370	0.8398	0.8425	0.8446	0.8462	0.8468	0.8476	0.8487	0.8507	0.8535	0.8542	0.8541	0.8551	0.8542	0.8527	0.8544	0.8587	0.8637	0.8675	0.8690	0.8702	0.8709	0.8715	0.8722	0.8721
Illinois	0.8557	0.8580	0.8605	0.8628	0.8655	0.8681	0.8706	0.8725	0.8740	0.8754	0.8778	0.8810	0.8827	0.8839	0.8863	0.8876	0.8883	0.8909	0.8948	0.8986	0.9016	0.9032	0.9048	0.9058	0.9067	0.9078	0.9081
Indiana	0.8477	0.8502	0.8529	0.8553	0.8575	0.8588	0.8596	0.8597	0.8599	0.8606	0.8625	0.8656	0.8668	0.8675	0.8690	0.8689	0.8681	0.8692	0.8722	0.8755	0.8779	0.8790	0.8801	0.8807	0.8815	0.8826	0.8831
Iowa	0.8542	0.8563	0.8585	0.8601	0.8622	0.8637	0.8652	0.8662	0.8670	0.8680	0.8702	0.8731	0.8744	0.8748	0.8762	0.8755	0.8743	0.8758	0.8797	0.8837	0.8867	0.8884	0.8897	0.8905	0.8914	0.8924	0.8929
Kansas	0.8565	0.8587	0.8606	0.8623	0.8639	0.8649	0.8656	0.8656	0.8658	0.8664	0.8683	0.8715	0.8729	0.8738	0.8754	0.8752	0.8741	0.8753	0.8789	0.8826	0.8856	0.8877	0.8899	0.8918	0.8933	0.8947	0.8952
Kentucky	0.8301	0.8330	0.8360	0.8388	0.8414	0.8433	0.8448	0.8459	0.8471	0.8488	0.8515	0.8548	0.8562	0.8567	0.8580	0.8580	0.8573	0.8587	0.8618	0.8651	0.8675	0.8686	0.8696	0.8703	0.8714	0.8730	0.8740
Louisiana	0.8238	0.8269	0.8300	0.8332	0.8368	0.8398	0.8422	0.8440	0.8457	0.8473	0.8498	0.8537	0.8559	0.8574	0.8596	0.8605	0.8612	0.8641	0.8686	0.8729	0.8764	0.8783	0.8802	0.8815	0.8830	0.8845	0.8853
Maine	0.8580	0.8620	0.8657	0.8690	0.8720	0.8740	0.8756	0.8766	0.8779	0.8797	0.8821	0.8849	0.8861	0.8870	0.8890	0.8900	0.8906	0.8927	0.8956	0.8986	0.9006	0.9015	0.9024	0.9031	0.9041	0.9053	0.9060
Maryland	0.8772	0.8804	0.8834	0.8862	0.8889	0.8908	0.8920	0.8924	0.8924	0.8929	0.8946	0.8974	0.8985	0.8992	0.9010	0.9015	0.9013	0.9030	0.9069	0.9115	0.9152	0.9173	0.9189	0.9196	0.9201	0.9207	0.9204
Massachusetts	0.8887	0.8918	0.8946	0.8972	0.8998	0.9019	0.9036	0.9048	0.9058	0.9073	0.9100	0.9132	0.9147	0.9159	0.9178	0.9186	0.9188	0.9205	0.9228	0.9256	0.9278	0.9292	0.9309	0.9322	0.9335	0.9346	0.9349
Michigan	0.8554	0.8582	0.8612	0.8643	0.8674	0.8697	0.8714	0.8724	0.8733	0.8747	0.8771	0.8802	0.8819	0.8832	0.8856	0.8866	0.8867	0.8880	0.8900	0.8915	0.8924	0.8926	0.8931	0.8937	0.8948	0.8963	0.8971
Minnesota	0.8699	0.8727	0.8752	0.8771	0.8790	0.8804	0.8815	0.8821	0.8830	0.8842	0.8863	0.8889	0.8897	0.8901	0.8915	0.8912	0.8903	0.8921	0.8958	0.8996	0.9025	0.9040	0.9054	0.9062	0.9072	0.9085	0.9091
Mississippi	0.8129	0.8162	0.8197	0.8236	0.8276	0.8308	0.8333	0.8348	0.8363	0.8381	0.8410	0.8452	0.8471	0.8478	0.8484	0.8472	0.8456	0.8477	0.8530	0.8592	0.8642	0.8672	0.8694	0.8709	0.8721	0.8732	0.8736
Missouri	0.8474	0.8503	0.8532	0.8560	0.8586	0.8604	0.8617	0.8627	0.8638	0.8653	0.8678	0.8709	0.8722	0.8730	0.8745	0.8746	0.8742	0.8759	0.8794	0.8830	0.8856	0.8869	0.8883	0.8895	0.8908	0.8922	0.8928
Montana	0.8451	0.8473	0.8495	0.8523	0.8551	0.8577	0.8600	0.8617	0.8633	0.8645	0.8661	0.8681	0.8687	0.8694	0.8715	0.8725	0.8730	0.8754	0.8790	0.8823	0.8848	0.8861	0.8878	0.8891	0.8906	0.8924	0.8933
Nebraska	0.8549	0.8577	0.8600	0.8617	0.8634	0.8648	0.8663	0.8669	0.8672	0.8675	0.8682	0.8699	0.8701	0.8708	0.8726	0.8725	0.8713	0.8727	0.8765	0.8808	0.8843	0.8863	0.8877	0.8883	0.8890	0.8898	0.8900
Nevada	0.8498	0.8526	0.8552	0.8569	0.8581	0.8592	0.8605	0.8615	0.8623	0.8633	0.8651	0.8677	0.8684	0.8683	0.8686	0.8669	0.8648	0.8676	0.8745	0.8819	0.8870	0.8888	0.8892	0.8885	0.8876	0.8870	0.8859
New Hampshire	0.8787	0.8824	0.8851	0.8872	0.8894	0.8913	0.8929	0.8943	0.8958	0.8974	0.9000	0.9030	0.9043	0.9054	0.9080	0.9097	0.9111	0.9135	0.9165	0.9191	0.9211	0.9225	0.9244	0.9261	0.9280	0.9300	0.9311
New Jersey	0.8749	0.8779	0.8808	0.8831	0.8853	0.8874	0.8892	0.8905	0.8915	0.8922	0.8936	0.8956	0.8962	0.8969	0.8989	0.8995	0.8992	0.9002	0.9037	0.9081	0.9116	0.9135	0.9153	0.9164	0.9173	0.9180	0.9179
New Mexico	0.8245	0.8265	0.8286	0.8315	0.8352	0.8385	0.8411	0.8428	0.8442	0.8454	0.8473	0.8504	0.8517	0.8525	0.8545	0.8554	0.8555	0.8578	0.8621	0.8667	0.8706	0.8731	0.8754	0.8771	0.8788	0.8804	0.8810
New York	0.8682	0.8712	0.8740	0.8767	0.8794	0.8822	0.8848	0.8869	0.8885	0.8901	0.8923	0.8952	0.8962	0.8968	0.8986	0.8992	0.8992	0.9011	0.9041	0.9075	0.9104	0.9124	0.9145	0.9163	0.9180	0.9192	0.9195
North Carolina	0.8437	0.8476	0.8515	0.8550	0.8579	0.8597	0.8605	0.8604	0.8599	0.8598	0.8614	0.8649	0.8668	0.8677	0.8686	0.8675	0.8659	0.8676	0.8725	0.8784	0.8829	0.8848	0.8862	0.8864	0.8866	0.8870	0.8869
North Dakota	0.8460	0.8484	0.8505	0.8521	0.8545	0.8566	0.8599	0.8625	0.8659	0.8685	0.8714	0.8737	0.8742	0.8745	0.8749	0.8740	0.8724	0.8743	0.8788	0.8831	0.8865	0.8883	0.8907	0.8917	0.8930	0.8946	0.8961
Ohio	0.8535	0.8558	0.8582	0.8605	0.8628	0.8645	0.8658	0.8665	0.8672	0.8683	0.8704	0.8736	0.8753	0.8765	0.8784	0.8789	0.8786	0.8799	0.8826	0.8852	0.8871	0.8880	0.8890	0.8898	0.8910	0.8924	0.8932
Oklahoma	0.8427	0.8442	0.8458	0.8472	0.8484	0.8490	0.8492	0.8492	0.8496	0.8504	0.8526	0.8559	0.8573	0.8582	0.8600	0.8607	0.8606	0.8623	0.8661	0.8694	0.8723	0.8743	0.8766	0.8785	0.8805	0.8823	0.8831
Oregon	0.8569	0.8601	0.8631	0.8656	0.8676	0.8689	0.8703	0.8714	0.8727	0.8747	0.8779	0.8818	0.8840	0.8854	0.8870	0.8866	0.8855	0.8867	0.8902	0.8940	0.8969	0.8983	0.8994	0.9002	0.9012	0.9026	0.9031
Pennsylvania	0.8579	0.8611	0.8641	0.8671	0.8702	0.8729	0.8752	0.8767	0.8778	0.8792	0.8812	0.8837	0.8847	0.8853	0.8867	0.8869	0.8866	0.8884	0.8918	0.8955	0.8985	0.9003	0.9021	0.9036	0.9051	0.9067	0.9076
Rhode Island	0.8670	0.8701	0.8732	0.8765	0.8799	0.8827	0.8847	0.8861	0.8874	0.8886	0.8903	0.8926	0.8939	0.8955	0.8981	0.8995	0.9002	0.9023	0.9055	0.9089	0.9118	0.9134	0.9150	0.9164	0.9180	0.9197	0.9206
South Carolina	0.8340	0.8389	0.8440	0.8488	0.8529	0.8554	0.8565	0.8566	0.8567	0.8577	0.8603	0.8643	0.8661	0.8664	0.8663	0.8642	0.8618	0.8632	0.8683	0.8744	0.8791	0.8815	0.8831	0.8838	0.8845	0.8853	0.8855

Appendix Table 5B. SDI Values by location, 1990-2016																											
Location	1990	1991	1992	1993	1994	1995	1996	1997	1998	1999	2000	2001	2002	2003	2004	2005	2006	2007	2008	2009	2010	2011	2012	2013	2014	2015	2016
South Dakota	0.8335	0.8361	0.8389	0.8423	0.8461	0.8489	0.8518	0.8534	0.8550	0.8567	0.8590	0.8614	0.8614	0.8617	0.8630	0.8625	0.8609	0.8633	0.8681	0.8725	0.8755	0.8771	0.8783	0.8792	0.8802	0.8812	0.8817
Tennessee	0.8372	0.8406	0.8442	0.8475	0.8507	0.8531	0.8547	0.8555	0.8566	0.8581	0.8605	0.8639	0.8656	0.8664	0.8674	0.8667	0.8654	0.8669	0.8709	0.8755	0.8788	0.8804	0.8816	0.8822	0.8830	0.8842	0.8847
Texas	0.8323	0.8338	0.8353	0.8371	0.8389	0.8404	0.8416	0.8425	0.8433	0.8439	0.8456	0.8486	0.8497	0.8505	0.8525	0.8531	0.8527	0.8550	0.8601	0.8656	0.8704	0.8732	0.8752	0.8761	0.8770	0.8780	0.8781
Utah	0.8223	0.8269	0.8306	0.8330	0.8345	0.8346	0.8344	0.8340	0.8342	0.8355	0.8387	0.8434	0.8456	0.8468	0.8489	0.8488	0.8476	0.8498	0.8546	0.8594	0.8628	0.8645	0.8660	0.8673	0.8685	0.8701	0.8704
Vermont	0.8689	0.8725	0.8758	0.8788	0.8820	0.8848	0.8870	0.8887	0.8905	0.8925	0.8951	0.8978	0.8989	0.9000	0.9024	0.9038	0.9045	0.9065	0.9093	0.9118	0.9135	0.9145	0.9157	0.9167	0.9180	0.9195	0.9204
Virginia	0.8684	0.8715	0.8743	0.8769	0.8796	0.8815	0.8828	0.8833	0.8835	0.8840	0.8855	0.8877	0.8881	0.8882	0.8897	0.8906	0.8912	0.8941	0.8986	0.9031	0.9066	0.9085	0.9102	0.9112	0.9121	0.9131	0.9134
Washington	0.8677	0.8704	0.8731	0.8758	0.8783	0.8801	0.8817	0.8832	0.8850	0.8875	0.8907	0.8941	0.8957	0.8964	0.8978	0.8972	0.8957	0.8965	0.8991	0.9021	0.9045	0.9058	0.9074	0.9085	0.9097	0.9111	0.9116
West Virginia	0.8318	0.8343	0.8372	0.8401	0.8431	0.8453	0.8472	0.8486	0.8496	0.8506	0.8520	0.8544	0.8558	0.8568	0.8586	0.8590	0.8586	0.8594	0.8620	0.8649	0.8672	0.8685	0.8700	0.8715	0.8733	0.8751	0.8763
Wisconsin	0.8586	0.8614	0.8641	0.8667	0.8694	0.8715	0.8731	0.8741	0.8750	0.8759	0.8776	0.8801	0.8812	0.8820	0.8837	0.8840	0.8837	0.8852	0.8882	0.8915	0.8941	0.8955	0.8970	0.8982	0.8995	0.9009	0.9016
Wyoming	0.8516	0.8539	0.8565	0.8597	0.8630	0.8650	0.8665	0.8684	0.8708	0.8735	0.8761	0.8783	0.8783	0.8780	0.8784	0.8774	0.8767	0.8795	0.8862	0.8928	0.8980	0.9008	0.9023	0.9028	0.9038	0.9049	0.9053
Latin America and Caribbean	0.5464	0.5536	0.5608	0.5678	0.5746	0.5813	0.5881	0.5950	0.6018	0.6082	0.6150	0.6212	0.6269	0.6323	0.6378	0.6434	0.6494	0.6554	0.6613	0.6665	0.6720	0.6779	0.6836	0.6892	0.6949	0.7005	0.7050
Caribbean	0.5883	0.5943	0.5991	0.6022	0.6047	0.6076	0.6104	0.6132	0.6171	0.6215	0.6260	0.6305	0.6351	0.6401	0.6452	0.6507	0.6562	0.6600	0.6631	0.6668	0.6707	0.6749	0.6794	0.6833	0.6876	0.6921	0.6958
Antigua and Barbuda	0.6844	0.6905	0.6956	0.7003	0.7044	0.7067	0.7097	0.7126	0.7156	0.7187	0.7215	0.7233	0.7253	0.7279	0.7310	0.7350	0.7402	0.7461	0.7518	0.7557	0.7587	0.7615	0.7645	0.7675	0.7708	0.7743	0.7776
The Bahamas	0.7188	0.7199	0.7206	0.7219	0.7244	0.7281	0.7331	0.7386	0.7446	0.7509	0.7565	0.7612	0.7651	0.7673	0.7686	0.7700	0.7714	0.7730	0.7740	0.7745	0.7753	0.7762	0.7773	0.7785	0.7799	0.7814	0.7829
Barbados	0.7262	0.7318	0.7367	0.7411	0.7444	0.7476	0.7514	0.7552	0.7589	0.7625	0.7661	0.7690	0.7705	0.7719	0.7735	0.7758	0.7789	0.7820	0.7845	0.7863	0.7876	0.7887	0.7899	0.7915	0.7930	0.7948	0.7966
Belize	0.4519	0.4691	0.4866	0.5022	0.5149	0.5247	0.5318	0.5372	0.5416	0.5462	0.5516	0.5570	0.5626	0.5686	0.5745	0.5799	0.5847	0.5890	0.5927	0.5961	0.5997	0.6038	0.6080	0.6121	0.6166	0.6211	0.6247
Cuba	0.6849	0.6934	0.6980	0.6970	0.6960	0.6952	0.6947	0.6923	0.6926	0.6943	0.6974	0.7008	0.7053	0.7120	0.7192	0.7254	0.7320	0.7386	0.7392	0.7430	0.7466	0.7513	0.7572	0.7613	0.7665	0.7723	0.7777
Dominica	0.5957	0.6000	0.6051	0.6119	0.6200	0.6288	0.6372	0.6448	0.6526	0.6594	0.6650	0.6698	0.6738	0.6778	0.6820	0.6858	0.6901	0.6951	0.7007	0.7055	0.7099	0.7140	0.7176	0.7212	0.7253	0.7294	0.7337
Dominican Republic	0.5269	0.5302	0.5346	0.5398	0.5453	0.5512	0.5579	0.5655	0.5737	0.5818	0.5893	0.5964	0.6033	0.6093	0.6152	0.6219	0.6294	0.6369	0.6434	0.6495	0.6560	0.6623	0.6684	0.6749	0.6822	0.6897	0.6961
Grenada	0.5001	0.5095	0.5189	0.5273	0.5356	0.5438	0.5522	0.5609	0.5707	0.5809	0.5907	0.5996	0.6086	0.6182	0.6273	0.6372	0.6457	0.6543	0.6621	0.6681	0.6738	0.6791	0.6837	0.6885	0.6936	0.6990	0.7038
Guyana	0.5170	0.5176	0.5202	0.5246	0.5303	0.5367	0.5445	0.5524	0.5588	0.5645	0.5690	0.5729	0.5761	0.5788	0.5815	0.5838	0.5871	0.5915	0.5962	0.6013	0.6071	0.6137	0.6209	0.6283	0.6357	0.6429	0.6486
Haiti	0.3033	0.3098	0.3154	0.3193	0.3214	0.3247	0.3291	0.3341	0.3394	0.3457	0.3520	0.3576	0.3628	0.3676	0.3718	0.3763	0.3810	0.3859	0.3910	0.3968	0.4016	0.4073	0.4128	0.4183	0.4235	0.4284	0.4319
Jamaica	0.5989	0.6038	0.6090	0.6145	0.6197	0.6247	0.6292	0.6330	0.6365	0.6398	0.6432	0.6467	0.6505	0.6548	0.6592	0.6639	0.6692	0.6743	0.6787	0.6821	0.6852	0.6884	0.6911	0.6937	0.6962	0.6990	0.7012
Saint Lucia	0.5548	0.5647	0.5745	0.5831	0.5907	0.5982	0.6062	0.6137	0.6215	0.6288	0.6354	0.6411	0.6471	0.6542	0.6617	0.6684	0.6738	0.6792	0.6844	0.6894	0.6925	0.6965	0.7001	0.7034	0.7066	0.7098	0.7131
Saint Vincent and the Grenadines	0.5159	0.5243	0.5329	0.5406	0.5475	0.5548	0.5620	0.5690	0.5758	0.5825	0.5890	0.5953	0.6016	0.6083	0.6149	0.6210	0.6272	0.6325	0.6377	0.6419	0.6452	0.6482	0.6513	0.6548	0.6588	0.6634	0.6675
Suriname	0.5658	0.5695	0.5723	0.5739	0.5750	0.5767	0.5796	0.5833	0.5867	0.5892	0.5925	0.5965	0.6014	0.6073	0.6141	0.6211	0.6283	0.6358	0.6434	0.6510	0.6587	0.6664	0.6735	0.6799	0.6858	0.6913	0.6957
Trinidad and Tobago	0.6546	0.6593	0.6631	0.6659	0.6676	0.6689	0.6713	0.6752	0.6805	0.6872	0.6944	0.7004	0.7063	0.7130	0.7193	0.7250	0.7314	0.7379	0.7452	0.7504	0.7551	0.7577	0.7600	0.7619	0.7635	0.7649	0.7662
Bermuda	0.7331	0.7372	0.7411	0.7449	0.7485	0.7521	0.7559	0.7597	0.7632	0.7666	0.7701	0.7739	0.7770	0.7806	0.7847	0.7886	0.7925	0.7963	0.7987	0.8008	0.8032	0.8057	0.8081	0.8105	0.8130	0.8155	0.8186
Puerto Rico	0.7669	0.7742	0.7812	0.7876	0.7934	0.7989	0.8046	0.8105	0.8166	0.8224	0.8280	0.8343	0.8399	0.8449	0.8496	0.8539	0.8576	0.8605	0.8628	0.8645	0.8658	0.8671	0.8683	0.8696	0.8712	0.8729	0.8747
Virgin Islands, U.S.	0.7766	0.7825	0.7889	0.7955	0.8021	0.8084	0.8144	0.8199	0.8250	0.8294	0.8331	0.8361	0.8388	0.8417	0.8454	0.8504	0.8554	0.8607	0.8644	0.8678	0.8710	0.8735	0.8752	0.8758	0.8764	0.8768	0.8775
Andean Latin America	0.5238	0.5298	0.5356	0.5415	0.5478	0.5545	0.5613	0.5683	0.5753	0.5819	0.5881	0.5939	0.5996	0.6047	0.6096	0.6146	0.6198	0.6251	0.6305	0.6351	0.6396	0.6443	0.6487	0.6529	0.6572	0.6618	0.6662
Bolivia	0.4249	0.4319	0.4389	0.4459	0.4532	0.4608	0.4688	0.4767	0.4852	0.4938	0.5022	0.5104	0.5183	0.5249	0.5314	0.5387	0.5460	0.5532	0.5603	0.5668	0.5732	0.5797	0.5862	0.5925	0.5987	0.6050	0.6103
Ecuador	0.5300	0.5367	0.5435	0.5499	0.5565	0.5630	0.5694	0.5760	0.5826	0.5883	0.5935	0.5984	0.6035	0.6083	0.6134	0.6187	0.6242	0.6294	0.6350	0.6401	0.6449	0.6502	0.6554	0.6604	0.6654	0.6702	0.6743
Peru	0.5480	0.5531	0.5580	0.5631	0.5690	0.5757	0.5823	0.5896	0.5966	0.6033	0.6097	0.6154	0.6208	0.6257	0.6302	0.6344	0.6389	0.6436	0.6484	0.6522	0.6561	0.6599	0.6633	0.6664	0.6697	0.6737	0.6780
Central Latin America	0.5435	0.5513	0.5599	0.5678	0.5748	0.5813	0.5882	0.5955	0.6023	0.6088	0.6154	0.6216	0.6273	0.6328	0.6389	0.6451	0.6519	0.6586	0.6651	0.6705	0.6763	0.6824	0.6887	0.6949	0.7009	0.7067	0.7113
Colombia	0.5503	0.5559	0.5617	0.5678	0.5744	0.5813	0.5882	0.5951	0.6011	0.6060	0.6109	0.6156	0.6200	0.6242	0.6287	0.6335	0.6391	0.6454	0.6516	0.6577	0.6643	0.6717	0.6790	0.6864	0.6937	0.7009	0.7068
Costa Rica	0.5700	0.5768	0.5841	0.5911	0.5976	0.6033	0.6083	0.6138	0.6202	0.6273	0.6340	0.6400	0.6456	0.6513	0.6567	0.6620	0.6677	0.6737	0.6794	0.6849	0.6910	0.6973	0.7037	0.7099	0.7159	0.7218	0.7260
El Salvador	0.4445	0.4508	0.4577	0.4650	0.4725	0.4803	0.4881	0.4961	0.5044	0.5128	0.5211	0.5292	0.5371	0.5447	0.5522	0.5598	0.5679	0.5762	0.5845	0.5922	0.6002	0.6088	0.6169	0.6246	0.6319	0.6390	0.6448
Guatemala	0.3648	0.3703	0.3763	0.3826	0.3893	0.3964	0.4040	0.4121	0.4203	0.4288	0.4376	0.4463	0.4550	0.4635	0.4718	0.4800	0.4887	0.4976	0.5063	0.5144	0.5221	0.5297	0.5367	0.5434	0.5498	0.5560	0.5608
Honduras	0.3809	0.3904	0.4000	0.4094	0.4180	0.4265	0.4349	0.4434	0.4515	0.4587	0.4663	0.4737	0.4809	0.4880	0.4951	0.5018	0.5084	0.5152	0.5216	0.5272	0.5329	0.5388	0.5448	0.5506	0.5565	0.5624	0.5673
Mexico	0.5552	0.5640	0.5729	0.5819	0.5907	0.5983	0.6062	0.6142	0.6219	0.6293	0.6368	0.6439	0.6506	0.6571	0.6639	0.6706	0.6776	0.6844	0.6905	0.6954	0.7007	0.7064	0.7122	0.7177	0.7233	0.7289	0.7335
Nicaragua	0.4342	0.4360	0.4379	0.4401	0.4431	0.4474	0.4527	0.4588	0.4658	0.4733	0.4810	0.4885	0.4956	0.5025	0.5095	0.5164	0.5232	0.5303	0.5370	0.5431	0.5491	0.5555	0.5620	0.5687	0.5756	0.5826	0.5883
Panama	0.6029	0.6066	0.6108	0.6155	0.6199	0.6238	0.6281	0.6335	0.6399	0.6465	0.6534	0.6599	0.6655	0.6703	0.6745	0.6787	0.6830	0.6878	0.6932	0.6983	0.7036	0.7101	0.7176	0.7260	0.7348	0.7439	0.7518
Venezuela	0.5905	0.6015	0.6185	0.6280	0.6295	0.6321	0.6366	0.6421	0.6467	0.6501	0.6537	0.6573	0.6596	0.6618	0.6668	0.6734	0.6808	0.6885	0.6962	0.7024	0.7083	0.7142	0.7208	0.7277	0.7336	0.7382	0.7417
Aguascalientes	0.5861	0.5967	0.6080	0.6169	0.6240	0.6308	0.6390	0.6476	0.6558	0.6630	0.6697	0.6754	0.6810	0.6869	0.6929	0.6985	0.7047	0.7106	0.7154	0.7187	0.7227	0.7278	0.7334	0.7390	0.7443	0.7494	0.7536
Baja California	0.6293	0.6352	0.6424	0.6497	0.6575	0.6657	0.6722	0.6761	0.6803	0.6866	0.6952	0.7031	0.7089	0.7128	0.7154	0.7176	0.7216	0.7277	0.7345	0.7397	0.7448	0.7510	0.7580	0.7638	0.7685	0.7727	0.7761
Baja California Sur	0.6435	0.6536	0.6625	0.6686	0.6739	0.6794	0.6859	0.6910	0.6925	0.6891	0.6887	0.6941	0.6980	0.7007	0.7071	0.7119	0.7106	0.7072	0.7062	0.7099	0.7184	0.7287	0.7377	0.7437	0.7482	0.7527	0.7566
Campeche	0.5300	0.5405	0.5514	0.5600	0.5670	0.5738	0.5825	0.5927	0.6034	0.6141	0.6248	0.6346	0.6433	0.6511	0.6582	0.6646	0.6711	0.6771	0.6817	0.6846	0.6882	0.6935	0.6999	0.7065	0.7134	0.7202	0.7258
Coahuila	0.6048	0.6172	0.6300	0.6403	0.6487	0.6558	0.6635	0.6712	0.6785	0.6851	0.6913	0.6968	0.7022	0.7078	0.7138	0.7197	0.7264	0.7332	0.7391	0.7432	0.7471	0.7509	0.7544	0.7577	0.7614	0.7659	0.7700
Colima	0.5912	0.6035	0.6155	0.6250	0.6323	0.6385	0.6452	0.6524	0.6592	0.6656	0.6720	0.6777	0.6832	0.6889	0.6948	0.7004	0.7063	0.7119	0.7164	0.7194	0.7228	0.7270	0.7319	0.7371	0.7425	0.7479	0.7525
Chiapas	0.3420	0.3549	0.3746	0.3927	0.4096	0.4267	0.4439	0.4594	0.4723	0.4827	0.4914	0.4992	0.5076	0.5173	0.5270	0.5361	0.5456	0.5548	0.5630	0.5698	0.5774	0.5854	0.5929	0.6003	0.6080	0.6158	0.6218
Chihuahua	0.5872	0.5969	0.6067	0.6142	0.6195	0.6235	0.6282	0.6336	0.6392	0.6450	0.6515	0.6582	0.6656	0.6735	0.6815	0.6892	0.6974	0.7054	0.7127	0.7190	0.7261	0.7343	0.7426	0.7504	0.7576	0.7640	0.7690
Distrito Federal	0.6712	0.6798	0.6859	0.6919	0.7015	0.7129	0.7226	0.7291	0.7333	0.7375	0.7431	0.7491	0.7554	0.7609	0.7661	0.7714	0.7758	0.7791	0.7819	0.7832	0.7851	0.7895	0.7956	0.8017	0.8076	0.8139	0.8191
Durango	0.5360	0.5442	0.5531	0.5589	0.5631	0.5682	0.5760	0.5856	0.5957	0.6055	0.6150	0.6238	0.6324	0.6412	0.6497	0.6574	0.6653	0.6724	0.6779	0.6812	0.6842	0.6877	0.6915	0.6955	0.7000	0.7049	0.7091
Guanajuato	0.4972	0.5105	0.5238	0.5343	0.5429	0.5515	0.5616	0.5726	0.5833	0.5934	0.6028	0.6111	0.6189	0.6266	0.6343	0.6418	0.6501	0.6584	0.6657	0.6714	0.6769	0.6826	0.6883	0.6939	0.6997	0.7057	0.7106
Guerrero	0.4410	0.4436	0.4450	0.4424	0.4385	0.4385	0.4445	0.4539	0.4639	0.4732	0.4831	0.4936	0.5048	0.5169	0.5291	0.5403	0.5513	0.5616	0.5701	0.5767	0.5847	0.5949	0.6060	0.6167	0.6263	0.6341	0.6395
Hidalgo	0.4905	0.5014	0.5126	0.5208	0.5271	0.5333	0.5412	0.5503	0.5596	0.5686	0.5772	0.5851	0.5927	0.6003	0.6081	0.6158	0.6243	0.6327	0.6402	0.6456	0.6507	0.6563	0.6624	0.6684	0.6744	0.6802	0.6848
Jalisco	0.5647	0.5766	0.5890	0.5988	0.6069	0.6146	0.6235	0.6329	0.6419	0.6501	0.6577	0.6643	0.6707	0.6771	0.6836	0.6898	0.6967	0.7033	0.7088	0.7125	0.7158	0.7194	0.7232	0.7272	0.7317	0.7365	0.7407
Mexico	0.5973	0.5983	0.5997	0.6160	0.6366	0.6460	0.6507	0.6552	0.6604	0.6663	0.6731	0.6794	0.6842	0.6874	0.6917	0.6968	0.7005	0.7032	0.7064	0.7106	0.7170	0.7228	0.7280	0.7333	0.7391	0.7453	0.7503
Michoacan de Ocampo	0.4755	0.4878	0.5005	0.5099	0.5174	0.5253	0.5353	0.5466	0.5578	0.5684	0.5786	0.5880	0.5972	0.6065	0.6158	0.6244	0.6333	0.6415	0.6481	0.6526	0.6566	0.6609	0.6655	0.6702	0.6753	0.6807	0.6853
Morelos	0.5872	0.5990	0.6108	0.6201	0.6274	0.6336	0.6403	0.6470	0.6533	0.6590	0.6645	0.6696	0.6748	0.6804	0.6862	0.6919	0.6982	0.7045	0.7100	0.7139	0.7178	0.7220	0.7261	0.7298	0.7335	0.7374	0.7409

Appendix Table 5B. SDI Values by location, 1990-2016

Location	1990	1991	1992	1993	1994	1995	1996	1997	1998	1999	2000	2001	2002	2003	2004	2005	2006	2007	2008	2009	2010	2011	2012	2013	2014	2015	2016
Nayarit	0.5334	0.5453	0.5574	0.5668	0.5745	0.5822	0.5916	0.6019	0.6120	0.6214	0.6304	0.6383	0.6454	0.6522	0.6585	0.6640	0.6697	0.6751	0.6794	0.6825	0.6867	0.6922	0.6983	0.7048	0.7114	0.7179	0.7233
Nuevo Leon	0.6596	0.6685	0.6779	0.6857	0.6917	0.6968	0.7027	0.7088	0.7147	0.7201	0.7251	0.7296	0.7340	0.7389	0.7440	0.7489	0.7545	0.7606	0.7646	0.7673	0.7697	0.7721	0.7746	0.7773	0.7803	0.7837	0.7869
Oaxaca	0.4464	0.4564	0.4656	0.4711	0.4738	0.4762	0.4809	0.4873	0.4941	0.5007	0.5076	0.5149	0.5234	0.5333	0.5441	0.5554	0.5679	0.5805	0.5921	0.6013	0.6093	0.6166	0.6231	0.6288	0.6341	0.6390	0.6428
Puebla	0.5046	0.5153	0.5256	0.5324	0.5368	0.5404	0.5455	0.5516	0.5581	0.5647	0.5716	0.5785	0.5860	0.5947	0.6041	0.6140	0.6252	0.6366	0.6469	0.6550	0.6622	0.6687	0.6742	0.6788	0.6833	0.6879	0.6917
Queretaro	0.5296	0.5401	0.5492	0.5585	0.5719	0.5893	0.6063	0.6189	0.6272	0.6362	0.6487	0.6606	0.6685	0.6730	0.6768	0.6817	0.6872	0.6927	0.6990	0.7045	0.7098	0.7154	0.7219	0.7281	0.7340	0.7397	0.7443
Quintana Roo	0.5756	0.5862	0.5982	0.6086	0.6174	0.6252	0.6336	0.6422	0.6504	0.6580	0.6652	0.6714	0.6771	0.6827	0.6879	0.6927	0.6980	0.7035	0.7086	0.7127	0.7179	0.7244	0.7313	0.7379	0.7441	0.7500	0.7548
San Luis Potosi	0.5002	0.5132	0.5260	0.5360	0.5439	0.5516	0.5612	0.5719	0.5827	0.5933	0.6037	0.6132	0.6222	0.6310	0.6396	0.6477	0.6563	0.6647	0.6721	0.6782	0.6852	0.6936	0.7025	0.7112	0.7196	0.7275	0.7337
Sinaloa	0.5807	0.5890	0.5973	0.6031	0.6075	0.6126	0.6202	0.6294	0.6392	0.6488	0.6579	0.6662	0.6739	0.6817	0.6893	0.6966	0.7044	0.7120	0.7183	0.7229	0.7271	0.7316	0.7360	0.7400	0.7439	0.7479	0.7513
Sonora	0.6140	0.6229	0.6324	0.6396	0.6450	0.6497	0.6558	0.6628	0.6699	0.6766	0.6833	0.6895	0.6958	0.7025	0.7093	0.7159	0.7233	0.7307	0.7371	0.7418	0.7463	0.7512	0.7563	0.7615	0.7668	0.7721	0.7767
Tabasco	0.5153	0.5254	0.5362	0.5445	0.5520	0.5606	0.5719	0.5844	0.5965	0.6072	0.6164	0.6239	0.6303	0.6362	0.6420	0.6476	0.6538	0.6600	0.6653	0.6693	0.6740	0.6796	0.6855	0.6910	0.6963	0.7015	0.7056
Tamaulipas	0.6047	0.6166	0.6287	0.6383	0.6459	0.6527	0.6601	0.6676	0.6744	0.6802	0.6853	0.6894	0.6932	0.6971	0.7014	0.7061	0.7122	0.7190	0.7257	0.7315	0.7378	0.7443	0.7505	0.7561	0.7613	0.7660	0.7699
Tlaxcala	0.5244	0.5329	0.5384	0.5454	0.5587	0.5728	0.5847	0.5929	0.5992	0.6051	0.6120	0.6202	0.6282	0.6354	0.6436	0.6512	0.6572	0.6641	0.6718	0.6769	0.6807	0.6854	0.6927	0.7011	0.7085	0.7147	0.7195
Veracruz de Ignacio de la Llave	0.5009	0.5098	0.5192	0.5261	0.5314	0.5370	0.5450	0.5545	0.5641	0.5733	0.5819	0.5897	0.5972	0.6050	0.6130	0.6208	0.6296	0.6385	0.6464	0.6526	0.6586	0.6647	0.6703	0.6754	0.6804	0.6855	0.6897
Yucatan	0.5447	0.5548	0.5657	0.5747	0.5824	0.5899	0.5986	0.6080	0.6171	0.6254	0.6334	0.6406	0.6474	0.6542	0.6608	0.6670	0.6736	0.6800	0.6854	0.6892	0.6930	0.6972	0.7014	0.7057	0.7101	0.7147	0.7187
Zacatecas	0.4980	0.5120	0.5259	0.5369	0.5458	0.5541	0.5635	0.5736	0.5833	0.5924	0.6009	0.6085	0.6155	0.6223	0.6289	0.6348	0.6410	0.6470	0.6520	0.6556	0.6602	0.6660	0.6722	0.6788	0.6857	0.6927	0.6986
Tropical Latin America	0.5390	0.5463	0.5531	0.5603	0.5677	0.5757	0.5835	0.5909	0.5984	0.6054	0.6129	0.6197	0.6259	0.6315	0.6370	0.6425	0.6482	0.6542	0.6604	0.6661	0.6723	0.6786	0.6845	0.6903	0.6965	0.7024	0.7071
Brazil	0.5402	0.5476	0.5544	0.5616	0.5691	0.5771	0.5849	0.5924	0.5998	0.6069	0.6143	0.6212	0.6273	0.6329	0.6384	0.6438	0.6495	0.6555	0.6616	0.6673	0.6734	0.6797	0.6855	0.6913	0.6974	0.7033	0.7079
Paraguay	0.4732	0.4783	0.4837	0.4898	0.4964	0.5036	0.5111	0.5192	0.5273	0.5353	0.5426	0.5498	0.5575	0.5655	0.5731	0.5802	0.5877	0.5958	0.6039	0.6110	0.6195	0.6280	0.6358	0.6446	0.6531	0.6613	0.6678
Acre	0.4490	0.4590	0.4665	0.4743	0.4831	0.4916	0.5007	0.5092	0.5166	0.5222	0.5282	0.5337	0.5386	0.5433	0.5485	0.5551	0.5626	0.5702	0.5782	0.5863	0.5948	0.6037	0.6123	0.6205	0.6286	0.6359	0.6416
Alagoas	0.4056	0.4140	0.4224	0.4314	0.4413	0.4515	0.4613	0.4695	0.4775	0.4852	0.4932	0.5007	0.5079	0.5149	0.5219	0.5289	0.5364	0.5444	0.5526	0.5603	0.5685	0.5768	0.5844	0.5917	0.5987	0.6049	0.6098
Amazonas	0.4862	0.4971	0.5056	0.5134	0.5226	0.5336	0.5438	0.5519	0.5601	0.5690	0.5784	0.5857	0.5917	0.5972	0.6030	0.6089	0.6152	0.6225	0.6301	0.6372	0.6448	0.6523	0.6594	0.6663	0.6735	0.6804	0.6858
Amapa	0.4774	0.4864	0.4941	0.5013	0.5088	0.5175	0.5279	0.5402	0.5543	0.5672	0.5773	0.5857	0.5939	0.6026	0.6109	0.6173	0.6233	0.6301	0.6377	0.6449	0.6523	0.6597	0.6655	0.6703	0.6748	0.6795	0.6838
Bahia	0.4469	0.4559	0.4638	0.4719	0.4806	0.4900	0.4985	0.5071	0.5166	0.5243	0.5318	0.5388	0.5452	0.5515	0.5580	0.5643	0.5709	0.5776	0.5845	0.5910	0.5982	0.6057	0.6128	0.6198	0.6268	0.6332	0.6383
Ceara	0.4391	0.4480	0.4551	0.4631	0.4717	0.4814	0.4916	0.5006	0.5088	0.5162	0.5233	0.5305	0.5376	0.5446	0.5515	0.5583	0.5655	0.5730	0.5808	0.5881	0.5961	0.6041	0.6115	0.6186	0.6257	0.6320	0.6371
Distrito Federal	0.6799	0.6859	0.6917	0.6984	0.7044	0.7101	0.7165	0.7230	0.7293	0.7353	0.7410	0.7472	0.7530	0.7582	0.7636	0.7691	0.7749	0.7811	0.7874	0.7933	0.7998	0.8063	0.8122	0.8179	0.8236	0.8288	0.8324
Espirito Santo	0.5527	0.5605	0.5684	0.5767	0.5842	0.5922	0.6000	0.6070	0.6141	0.6211	0.6281	0.6352	0.6421	0.6482	0.6541	0.6596	0.6652	0.6709	0.6766	0.6819	0.6877	0.6937	0.6993	0.7048	0.7109	0.7168	0.7214
Goias	0.5111	0.5199	0.5282	0.5367	0.5457	0.5554	0.5649	0.5736	0.5823	0.5901	0.5972	0.6041	0.6105	0.6165	0.6225	0.6285	0.6346	0.6408	0.6469	0.6525	0.6586	0.6647	0.6703	0.6760	0.6823	0.6885	0.6934
Maranhao	0.3798	0.3867	0.3933	0.4028	0.4129	0.4238	0.4353	0.4459	0.4571	0.4682	0.4789	0.4880	0.4958	0.5035	0.5105	0.5173	0.5246	0.5325	0.5408	0.5487	0.5571	0.5656	0.5735	0.5809	0.5884	0.5952	0.6005
Minas Gerais	0.5389	0.5457	0.5523	0.5590	0.5660	0.5738	0.5815	0.5891	0.5964	0.6030	0.6092	0.6155	0.6214	0.6269	0.6324	0.6378	0.6435	0.6495	0.6557	0.6614	0.6675	0.6738	0.6797	0.6855	0.6915	0.6971	0.7016
Mato Grosso do Sul	0.5141	0.5218	0.5299	0.5392	0.5481	0.5576	0.5672	0.5759	0.5841	0.5913	0.5979	0.6043	0.6101	0.6151	0.6204	0.6261	0.6320	0.6378	0.6435	0.6487	0.6541	0.6594	0.6644	0.6694	0.6753	0.6814	0.6863
Mato Grosso	0.5238	0.5318	0.5399	0.5492	0.5592	0.5693	0.5783	0.5867	0.5950	0.6022	0.6087	0.6154	0.6211	0.6259	0.6309	0.6361	0.6417	0.6474	0.6532	0.6585	0.6641	0.6697	0.6751	0.6803	0.6868	0.6934	0.6985
Para	0.4509	0.4582	0.4650	0.4733	0.4828	0.4932	0.5029	0.5107	0.5189	0.5262	0.5322	0.5371	0.5411	0.5448	0.5492	0.5541	0.5598	0.5665	0.5739	0.5813	0.5892	0.5974	0.6049	0.6119	0.6188	0.6251	0.6301
Paraiba	0.4359	0.4444	0.4509	0.4590	0.4672	0.4764	0.4860	0.4948	0.5035	0.5116	0.5192	0.5265	0.5334	0.5399	0.5463	0.5527	0.5595	0.5666	0.5741	0.5811	0.5886	0.5963	0.6033	0.6098	0.6164	0.6223	0.6270
Parana	0.5485	0.5563	0.5640	0.5721	0.5804	0.5890	0.5975	0.6058	0.6140	0.6216	0.6289	0.6361	0.6429	0.6488	0.6544	0.6597	0.6653	0.6710	0.6769	0.6822	0.6880	0.6942	0.6999	0.7057	0.7118	0.7176	0.7222
Pernambuco	0.4677	0.4770	0.4846	0.4924	0.5001	0.5078	0.5160	0.5231	0.5298	0.5361	0.5427	0.5491	0.5553	0.5612	0.5672	0.5730	0.5791	0.5854	0.5919	0.5980	0.6046	0.6112	0.6173	0.6234	0.6297	0.6357	0.6404
Piaui	0.4076	0.4152	0.4211	0.4284	0.4363	0.4449	0.4547	0.4644	0.4735	0.4819	0.4902	0.4980	0.5055	0.5125	0.5192	0.5257	0.5329	0.5405	0.5482	0.5553	0.5629	0.5706	0.5778	0.5846	0.5914	0.5976	0.6025
Rio de Janeiro	0.6204	0.6259	0.6311	0.6365	0.6420	0.6476	0.6533	0.6589	0.6645	0.6701	0.6763	0.6823	0.6876	0.6920	0.6965	0.7008	0.7051	0.7097	0.7144	0.7184	0.7229	0.7274	0.7315	0.7356	0.7403	0.7451	0.7491
Rio Grande do Norte	0.4610	0.4694	0.4770	0.4853	0.4939	0.5038	0.5151	0.5237	0.5309	0.5387	0.5465	0.5542	0.5615	0.5684	0.5753	0.5819	0.5887	0.5959	0.6033	0.6102	0.6177	0.6253	0.6322	0.6388	0.6454	0.6514	0.6562
Rondonia	0.4770	0.4860	0.4941	0.5021	0.5107	0.5195	0.5279	0.5362	0.5446	0.5524	0.5602	0.5680	0.5752	0.5821	0.5893	0.5966	0.6042	0.6118	0.6192	0.6258	0.6327	0.6398	0.6464	0.6528	0.6596	0.6659	0.6708
Roraima	0.5027	0.5121	0.5199	0.5274	0.5349	0.5419	0.5476	0.5516	0.5537	0.5583	0.5690	0.5810	0.5890	0.5952	0.6036	0.6123	0.6203	0.6276	0.6357	0.6439	0.6513	0.6568	0.6612	0.6662	0.6719	0.6778	0.6832
Rio Grande do Sul	0.5853	0.5922	0.5982	0.6038	0.6098	0.6165	0.6222	0.6283	0.6349	0.6407	0.6470	0.6538	0.6600	0.6654	0.6709	0.6763	0.6820	0.6877	0.6932	0.6980	0.7029	0.7078	0.7124	0.7171	0.7223	0.7274	0.7316
Santa Catarina	0.5690	0.5766	0.5839	0.5916	0.5998	0.6087	0.6167	0.6241	0.6317	0.6389	0.6465	0.6536	0.6598	0.6654	0.6708	0.6760	0.6814	0.6871	0.6930	0.6985	0.7045	0.7106	0.7165	0.7224	0.7287	0.7348	0.7396
Sergipe	0.4528	0.4632	0.4722	0.4810	0.4905	0.5010	0.5109	0.5197	0.5288	0.5373	0.5456	0.5538	0.5616	0.5689	0.5757	0.5822	0.5890	0.5964	0.6041	0.6115	0.6193	0.6272	0.6345	0.6415	0.6483	0.6545	0.6594
Sao Paulo	0.6153	0.6215	0.6275	0.6340	0.6403	0.6467	0.6529	0.6592	0.6655	0.6720	0.6794	0.6863	0.6922	0.6972	0.7018	0.7062	0.7110	0.7161	0.7211	0.7256	0.7305	0.7355	0.7402	0.7450	0.7504	0.7557	0.7600
Tocantins	0.4690	0.4774	0.4856	0.4938	0.5022	0.5114	0.5211	0.5304	0.5392	0.5475	0.5556	0.5634	0.5707	0.5775	0.5842	0.5906	0.5972	0.6043	0.6116	0.6185	0.6262	0.6342	0.6417	0.6491	0.6563	0.6627	0.6680
North Africa and Middle East	0.4752	0.4854	0.4954	0.5048	0.5135	0.5218	0.5306	0.5393	0.5476	0.5555	0.5636	0.5711	0.5785	0.5858	0.5933	0.6007	0.6081	0.6153	0.6220	0.6279	0.6339	0.6404	0.6470	0.6540	0.6612	0.6685	0.6741
North Africa and Middle East	0.4752	0.4854	0.4954	0.5048	0.5135	0.5218	0.5306	0.5393	0.5476	0.5555	0.5636	0.5711	0.5785	0.5858	0.5933	0.6007	0.6081	0.6153	0.6220	0.6279	0.6339	0.6404	0.6470	0.6540	0.6612	0.6685	0.6741
Algeria	0.4629	0.4719	0.4792	0.4849	0.4899	0.4947	0.4996	0.5046	0.5099	0.5157	0.5226	0.5298	0.5384	0.5486	0.5589	0.5697	0.5806	0.5913	0.6020	0.6119	0.6220	0.6320	0.6419	0.6513	0.6603	0.6692	0.6757
Bahrain	0.5861	0.5987	0.6053	0.6126	0.6227	0.6331	0.6405	0.6445	0.6463	0.6504	0.6629	0.6808	0.6939	0.6990	0.7006	0.7029	0.7070	0.7115	0.7158	0.7186	0.7195	0.7186	0.7181	0.7206	0.7257	0.7323	0.7382
Egypt	0.4577	0.4690	0.4797	0.4897	0.4994	0.5087	0.5180	0.5268	0.5350	0.5430	0.5508	0.5585	0.5658	0.5727	0.5791	0.5850	0.5906	0.5961	0.6015	0.6065	0.6117	0.6171	0.6226	0.6289	0.6365	0.6452	0.6525
Iran	0.5104	0.5363	0.5617	0.5855	0.6068	0.6252	0.6419	0.6558	0.6671	0.6759	0.6830	0.6887	0.6947	0.7010	0.7071	0.7133	0.7191	0.7254	0.7310	0.7363	0.7420	0.7480	0.7525	0.7570	0.7616	0.7662	0.7690
Iraq	0.3912	0.3984	0.4051	0.4117	0.4178	0.4230	0.4295	0.4369	0.4467	0.4592	0.4722	0.4845	0.4946	0.4981	0.4983	0.4925	0.4853	0.4752	0.4613	0.4472	0.4340	0.4287	0.4324	0.4401	0.4503	0.4625	0.4742
Jordan	0.4827	0.4972	0.5108	0.5237	0.5358	0.5476	0.5584	0.5683	0.5775	0.5863	0.5947	0.6027	0.6096	0.6153	0.6209	0.6264	0.6319	0.6373	0.6430	0.6492	0.6557	0.6626	0.6694	0.6761	0.6826	0.6890	0.6936
Kuwait	0.6519	0.6572	0.6604	0.6595	0.6534	0.6487	0.6549	0.6710	0.6885	0.7019	0.7110	0.7172	0.7213	0.7263	0.7317	0.7377	0.7472	0.7581	0.7697	0.7808	0.7908	0.7998	0.8081	0.8159	0.8230	0.8288	0.8325
Lebanon	0.6270	0.6361	0.6449	0.6531	0.6611	0.6691	0.6778	0.6870	0.6968	0.7062	0.7148	0.7228	0.7304	0.7375	0.7445	0.7511	0.7569	0.7631	0.7697	0.7774	0.7851	0.7918	0.7967	0.8008	0.8042	0.8071	0.8095
Libya	0.6054	0.6110	0.6169	0.6229	0.6296	0.6362	0.6434	0.6512	0.6589	0.6668	0.6751	0.6837	0.6923	0.7021	0.7122	0.7232	0.7344	0.7459	0.7575	0.7685	0.7806	0.7847	0.7928	0.7995	0.8036	0.8065	0.8064
Morocco	0.3812	0.3929	0.4044	0.4155	0.4271	0.4377	0.4494	0.4602	0.4709	0.4805	0.4896	0.4986	0.5072	0.5156	0.5237	0.5315	0.5399	0.5481	0.5565	0.5649	0.5731	0.5815	0.5894	0.5971	0.6044	0.6114	0.6173
Palestine	0.4107	0.4172	0.4241	0.4303	0.4373	0.4462	0.4565	0.4671	0.4776	0.4879	0.4971	0.5045	0.5096	0.5125	0.5101	0.5017	0.4898	0.4725	0.4520	0.4323	0.4183	0.4126	0.4122	0.4158	0.4249	0.4382	0.4497
Oman	0.2340	0.2555	0.2771	0.2980	0.3180	0.3370	0.3560	0.3758	0.3961	0.4166	0.4377	0.4581	0.4787	0.4993	0.5199	0.5398	0.5610	0.5815	0.6018	0.6208	0.6389	0.6582	0.6756	0.6910	0.7037	0.7145	0.7199
Qatar	0.6223	0.6310	0.6401	0.6498	0.6595	0.6687	0.6761	0.6837	0.6900	0.6961	0.7018	0.7081	0.7147	0.7216	0.7285	0.7350	0.7399	0.7451	0.7505	0.7557	0.7607	0.7649	0.7693	0.7740	0.7791	0.7845	0.7878
Saudi Arabia	0.3682	0.3829	0.3982	0.4137	0.4294	0.4453	0.4622	0.4794	0.4970	0.5146	0.5328	0.5509	0.5687	0.5867	0.6048	0.6231	0.6416	0.6598	0.6785	0.6960	0.7130	0.7298	0.7459	0.7609	0.7747	0.7877	0.7970

Appendix Table 5B. SDI Values by location, 1990-2016																											
**Location**	**1990**	**1991**	**1992**	**1993**	**1994**	**1995**	**1996**	**1997**	**1998**	**1999**	**2000**	**2001**	**2002**	**2003**	**2004**	**2005**	**2006**	**2007**	**2008**	**2009**	**2010**	**2011**	**2012**	**2013**	**2014**	**2015**	**2016**
Syria	0.3756	0.3857	0.3967	0.4077	0.4186	0.4295	0.4406	0.4513	0.4622	0.4725	0.4827	0.4929	0.5035	0.5138	0.5249	0.5372	0.5490	0.5604	0.5714	0.5823	0.5932	0.6030	0.6111	0.6181	0.6251	0.6320	0.6378
Tunisia	0.4755	0.4886	0.5017	0.5146	0.5270	0.5388	0.5510	0.5626	0.5731	0.5827	0.5912	0.5991	0.6058	0.6120	0.6179	0.6233	0.6289	0.6347	0.6404	0.6463	0.6527	0.6592	0.6667	0.6745	0.6823	0.6899	0.6962
Turkey	0.5760	0.5844	0.5921	0.5995	0.6054	0.6117	0.6189	0.6266	0.6342	0.6413	0.6491	0.6563	0.6634	0.6699	0.6767	0.6836	0.6907	0.6978	0.7050	0.7109	0.7173	0.7242	0.7310	0.7381	0.7458	0.7543	0.7617
United Arab Emirates	0.5568	0.5739	0.5902	0.6058	0.6200	0.6329	0.6445	0.6559	0.6669	0.6770	0.6861	0.6950	0.7031	0.7101	0.7160	0.7207	0.7251	0.7286	0.7319	0.7357	0.7401	0.7452	0.7507	0.7563	0.7620	0.7676	0.7716
Yemen	0.1731	0.1769	0.1847	0.1945	0.2055	0.2166	0.2286	0.2405	0.2525	0.2640	0.2757	0.2881	0.3015	0.3173	0.3345	0.3505	0.3616	0.3673	0.3711	0.3741	0.3790	0.3855	0.3979	0.4166	0.4341	0.4449	0.4504
Afghanistan	0.1578	0.1603	0.1634	0.1652	0.1652	0.1659	0.1664	0.1669	0.1676	0.1686	0.1700	0.1708	0.1751	0.1812	0.1878	0.1953	0.2029	0.2115	0.2196	0.2290	0.2383	0.2466	0.2548	0.2624	0.2693	0.2758	0.2810
Sudan	0.2948	0.2998	0.3045	0.3089	0.3127	0.3166	0.3204	0.3260	0.3322	0.3403	0.3507	0.3612	0.3718	0.3825	0.3933	0.4039	0.4150	0.4263	0.4373	0.4481	0.4588	0.4702	0.4802	0.4904	0.5005	0.5105	0.5185
Ha'il	0.3551	0.3693	0.3839	0.3988	0.4138	0.4292	0.4457	0.4625	0.4795	0.4965	0.5140	0.5317	0.5493	0.5670	0.5849	0.6027	0.6207	0.6386	0.6571	0.6743	0.6912	0.7078	0.7239	0.7389	0.7524	0.7649	0.7739
Qassim	0.3414	0.3572	0.3730	0.3888	0.4050	0.4216	0.4394	0.4576	0.4763	0.4953	0.5146	0.5342	0.5538	0.5729	0.5917	0.6101	0.6287	0.6471	0.6655	0.6838	0.7013	0.7173	0.7330	0.7482	0.7623	0.7757	0.7851
Riyadh	0.3692	0.3847	0.4005	0.4171	0.4340	0.4511	0.4685	0.4864	0.5048	0.5235	0.5428	0.5617	0.5804	0.5992	0.6181	0.6373	0.6562	0.6747	0.6934	0.7107	0.7273	0.7419	0.7558	0.7690	0.7826	0.7953	0.8044
Tabuk	0.3615	0.3758	0.3906	0.4059	0.4216	0.4376	0.4542	0.4713	0.4888	0.5066	0.5251	0.5434	0.5616	0.5799	0.5984	0.6197	0.6401	0.6594	0.6783	0.6954	0.7116	0.7286	0.7449	0.7600	0.7753	0.7897	0.7999
Madinah	0.3621	0.3770	0.3924	0.4080	0.4237	0.4397	0.4568	0.4744	0.4921	0.5100	0.5284	0.5466	0.5647	0.5830	0.6015	0.6197	0.6382	0.6564	0.6752	0.6927	0.7098	0.7272	0.7439	0.7595	0.7733	0.7862	0.7955
Makkah	0.3816	0.3962	0.4114	0.4268	0.4422	0.4578	0.4744	0.4913	0.5083	0.5253	0.5428	0.5601	0.5770	0.5941	0.6114	0.6281	0.6451	0.6617	0.6789	0.6948	0.7102	0.7261	0.7413	0.7553	0.7677	0.7792	0.7876
Bahah	0.3511	0.3642	0.3780	0.3919	0.4060	0.4203	0.4360	0.4519	0.4678	0.4837	0.5000	0.5164	0.5324	0.5488	0.5654	0.5822	0.5994	0.6164	0.6340	0.6503	0.6665	0.6829	0.6987	0.7135	0.7258	0.7372	0.7455
Northern Borders	0.4057	0.4197	0.4344	0.4496	0.4650	0.4804	0.4965	0.5130	0.5296	0.5462	0.5632	0.5798	0.5959	0.6122	0.6287	0.6470	0.6646	0.6812	0.6979	0.7130	0.7275	0.7415	0.7547	0.7665	0.7786	0.7900	0.7968
Jawf	0.4144	0.4309	0.4479	0.4649	0.4820	0.4991	0.5174	0.5359	0.5545	0.5730	0.5916	0.6099	0.6277	0.6455	0.6633	0.6812	0.6992	0.7169	0.7349	0.7515	0.7676	0.7837	0.7992	0.8136	0.8260	0.8376	0.8464
Jizan	0.3063	0.3171	0.3287	0.3405	0.3527	0.3651	0.3788	0.3927	0.4068	0.4209	0.4356	0.4504	0.4652	0.4804	0.4961	0.5125	0.5295	0.5464	0.5642	0.5805	0.5968	0.6136	0.6299	0.6452	0.6580	0.6701	0.6790
'Asir	0.3143	0.3275	0.3412	0.3551	0.3694	0.3842	0.4006	0.4172	0.4341	0.4510	0.4685	0.4865	0.5044	0.5225	0.5408	0.5593	0.5785	0.5975	0.6173	0.6359	0.6542	0.6723	0.6898	0.7065	0.7210	0.7346	0.7441
Najran	0.3088	0.3203	0.3324	0.3448	0.3575	0.3706	0.3851	0.4000	0.4152	0.4306	0.4466	0.4631	0.4794	0.4961	0.5131	0.5304	0.5483	0.5662	0.5849	0.6025	0.6198	0.6373	0.6544	0.6708	0.6855	0.6994	0.7093
Eastern Province	0.3775	0.3929	0.4089	0.4250	0.4412	0.4577	0.4751	0.4929	0.5110	0.5291	0.5478	0.5663	0.5844	0.6027	0.6211	0.6393	0.6576	0.6756	0.6941	0.7113	0.7280	0.7448	0.7610	0.7763	0.7901	0.8031	0.8126
South Asia	0.3490	0.3565	0.3639	0.3711	0.3785	0.3861	0.3940	0.4017	0.4095	0.4173	0.4248	0.4323	0.4398	0.4474	0.4554	0.4638	0.4726	0.4819	0.4909	0.5000	0.5095	0.5193	0.5290	0.5391	0.5495	0.5603	0.5693
South Asia	0.3490	0.3565	0.3639	0.3711	0.3785	0.3861	0.3940	0.4017	0.4095	0.4173	0.4248	0.4323	0.4398	0.4474	0.4554	0.4638	0.4726	0.4819	0.4909	0.5000	0.5095	0.5193	0.5290	0.5391	0.5495	0.5603	0.5693
Bangladesh	0.3146	0.3226	0.3310	0.3394	0.3474	0.3552	0.3628	0.3701	0.3771	0.3836	0.3898	0.3958	0.4016	0.4074	0.4135	0.4200	0.4269	0.4344	0.4423	0.4504	0.4586	0.4673	0.4759	0.4847	0.4936	0.5029	0.5109
Bhutan	0.3014	0.3096	0.3180	0.3270	0.3368	0.3476	0.3588	0.3704	0.3822	0.3939	0.4059	0.4184	0.4315	0.4445	0.4572	0.4697	0.4815	0.4940	0.5061	0.5178	0.5295	0.5412	0.5525	0.5626	0.5721	0.5814	0.5884
India	0.3570	0.3641	0.3712	0.3782	0.3854	0.3929	0.4007	0.4084	0.4161	0.4240	0.4317	0.4396	0.4473	0.4553	0.4636	0.4726	0.4821	0.4921	0.5019	0.5118	0.5222	0.5326	0.5430	0.5535	0.5642	0.5752	0.5844
Nepal	0.2423	0.2497	0.2578	0.2664	0.2756	0.2846	0.2932	0.3017	0.3102	0.3189	0.3278	0.3369	0.3455	0.3539	0.3621	0.3696	0.3769	0.3840	0.3911	0.3979	0.4043	0.4105	0.4171	0.4243	0.4332	0.4433	0.4522
Pakistan	0.2966	0.3089	0.3195	0.3296	0.3394	0.3493	0.3601	0.3715	0.3828	0.3924	0.4005	0.4083	0.4161	0.4236	0.4309	0.4375	0.4432	0.4484	0.4534	0.4585	0.4640	0.4705	0.4782	0.4877	0.4985	0.5100	0.5190
Andhra Pradesh	0.3294	0.3373	0.3448	0.3536	0.3628	0.3727	0.3832	0.3929	0.4035	0.4139	0.4245	0.4345	0.4437	0.4531	0.4625	0.4716	0.4814	0.4926	0.5035	0.5140	0.5251	0.5361	0.5462	0.5563	0.5664	0.5768	0.5853
Arunachal Pradesh	0.3296	0.3376	0.3456	0.3541	0.3619	0.3710	0.3796	0.3886	0.3972	0.4052	0.4132	0.4220	0.4302	0.4384	0.4475	0.4568	0.4663	0.4768	0.4867	0.4997	0.5128	0.5262	0.5392	0.5523	0.5652	0.5780	0.5889
Assam	0.3607	0.3671	0.3728	0.3780	0.3833	0.3889	0.3945	0.3999	0.4055	0.4131	0.4210	0.4289	0.4373	0.4456	0.4541	0.4622	0.4708	0.4794	0.4875	0.4955	0.5045	0.5136	0.5226	0.5320	0.5419	0.5524	0.5616
Bihar	0.3062	0.3116	0.3167	0.3183	0.3189	0.3178	0.3179	0.3189	0.3173	0.3169	0.3184	0.3206	0.3248	0.3286	0.3337	0.3382	0.3459	0.3539	0.3630	0.3708	0.3801	0.3899	0.4004	0.4108	0.4208	0.4309	0.4398
Chhattisgarh	0.3042	0.3102	0.3163	0.3226	0.3292	0.3363	0.3437	0.3516	0.3597	0.3685	0.3764	0.3856	0.3945	0.4045	0.4144	0.4248	0.4363	0.4487	0.4615	0.4733	0.4846	0.4967	0.5088	0.5211	0.5339	0.5470	0.5575
Delhi	0.5240	0.5287	0.5330	0.5379	0.5441	0.5504	0.5567	0.5651	0.5734	0.5823	0.5923	0.6022	0.6123	0.6225	0.6328	0.6431	0.6534	0.6638	0.6738	0.6844	0.6945	0.7047	0.7145	0.7244	0.7344	0.7445	0.7516
Goa	0.4730	0.4814	0.4900	0.5001	0.5108	0.5225	0.5351	0.5467	0.5605	0.5732	0.5856	0.5969	0.6076	0.6157	0.6228	0.6306	0.6381	0.6448	0.6537	0.6637	0.6733	0.6856	0.6964	0.7069	0.7180	0.7291	0.7384
Gujarat	0.3886	0.3952	0.4032	0.4109	0.4202	0.4293	0.4389	0.4478	0.4570	0.4655	0.4728	0.4800	0.4872	0.4953	0.5032	0.5124	0.5220	0.5322	0.5415	0.5511	0.5609	0.5705	0.5797	0.5886	0.5977	0.6072	0.6151
Haryana	0.3953	0.4049	0.4133	0.4220	0.4308	0.4394	0.4489	0.4580	0.4667	0.4754	0.4845	0.4939	0.5035	0.5134	0.5236	0.5342	0.5454	0.5570	0.5685	0.5799	0.5912	0.6024	0.6131	0.6238	0.6346	0.6457	0.6550
Himachal Pradesh	0.3743	0.3848	0.3958	0.4069	0.4186	0.4301	0.4432	0.4560	0.4688	0.4826	0.4959	0.5082	0.5197	0.5306	0.5415	0.5525	0.5641	0.5753	0.5867	0.5979	0.6099	0.6217	0.6330	0.6439	0.6542	0.6619	0.6694
Jammu and Kashmir	0.3427	0.3486	0.3531	0.3611	0.3688	0.3769	0.3844	0.3955	0.4052	0.4173	0.4292	0.4407	0.4524	0.4637	0.4753	0.4861	0.4972	0.5075	0.5181	0.5284	0.5386	0.5491	0.5592	0.5692	0.5792	0.5892	0.5977
Jharkhand	0.3003	0.3056	0.3109	0.3164	0.3223	0.3288	0.3345	0.3419	0.3497	0.3580	0.3653	0.3733	0.3813	0.3888	0.3973	0.4059	0.4145	0.4242	0.4329	0.4424	0.4533	0.4642	0.4753	0.4870	0.4993	0.5123	0.5230
Karnataka	0.3595	0.3680	0.3761	0.3850	0.3946	0.4047	0.4152	0.4252	0.4361	0.4467	0.4568	0.4655	0.4736	0.4813	0.4895	0.4992	0.5093	0.5206	0.5321	0.5427	0.5544	0.5654	0.5758	0.5858	0.5958	0.6059	0.6141
Kerala	0.4289	0.4365	0.4444	0.4538	0.4644	0.4762	0.4884	0.5002	0.5121	0.5242	0.5352	0.5446	0.5535	0.5618	0.5701	0.5796	0.5889	0.5985	0.6083	0.6185	0.6282	0.6382	0.6478	0.6573	0.6674	0.6762	0.6827
Madhya Pradesh	0.3319	0.3389	0.3449	0.3500	0.3548	0.3597	0.3650	0.3703	0.3749	0.3803	0.3851	0.3914	0.3972	0.4043	0.4113	0.4180	0.4254	0.4329	0.4406	0.4496	0.4590	0.4697	0.4811	0.4938	0.5069	0.5205	0.5322
Maharashtra	0.4177	0.4262	0.4350	0.4445	0.4539	0.4642	0.4747	0.4847	0.4938	0.5036	0.5115	0.5191	0.5265	0.5342	0.5427	0.5529	0.5642	0.5763	0.5877	0.5987	0.6099	0.6205	0.6300	0.6392	0.6484	0.6578	0.6657
Manipur	0.3637	0.3721	0.3797	0.3862	0.3907	0.3953	0.4003	0.4060	0.4127	0.4227	0.4327	0.4442	0.4553	0.4670	0.4802	0.4942	0.5068	0.5184	0.5289	0.5398	0.5494	0.5602	0.5705	0.5805	0.5906	0.6009	0.6100
Meghalaya	0.3422	0.3483	0.3533	0.3576	0.3600	0.3633	0.3669	0.3722	0.3791	0.3889	0.4019	0.4166	0.4307	0.4435	0.4554	0.4667	0.4774	0.4878	0.4978	0.5084	0.5189	0.5304	0.5411	0.5521	0.5634	0.5750	0.5847
Mizoram	0.4141	0.4219	0.4288	0.4359	0.4405	0.4456	0.4531	0.4593	0.4653	0.4729	0.4824	0.4920	0.5018	0.5111	0.5198	0.5284	0.5366	0.5450	0.5531	0.5623	0.5722	0.5815	0.5913	0.6011	0.6112	0.6215	0.6304
Nagaland	0.4008	0.4057	0.4093	0.4134	0.4160	0.4186	0.4220	0.4279	0.4344	0.4424	0.4564	0.4730	0.4901	0.5050	0.5190	0.5327	0.5452	0.5587	0.5720	0.5849	0.5969	0.6099	0.6221	0.6338	0.6453	0.6540	0.6613
Odisha	0.3050	0.3118	0.3179	0.3239	0.3302	0.3380	0.3442	0.3513	0.3592	0.3680	0.3762	0.3848	0.3928	0.4022	0.4127	0.4223	0.4332	0.4454	0.4569	0.4678	0.4797	0.4910	0.5026	0.5140	0.5256	0.5373	0.5469
Punjab	0.4094	0.4182	0.4270	0.4361	0.4454	0.4548	0.4646	0.4743	0.4838	0.4933	0.5026	0.5112	0.5190	0.5271	0.5353	0.5447	0.5547	0.5656	0.5764	0.5872	0.5976	0.6081	0.6179	0.6274	0.6365	0.6433	0.6499
Rajasthan	0.3147	0.3217	0.3288	0.3346	0.3414	0.3477	0.3548	0.3617	0.3678	0.3752	0.3819	0.3901	0.3970	0.4055	0.4137	0.4216	0.4305	0.4397	0.4490	0.4574	0.4678	0.4789	0.4898	0.5002	0.5105	0.5210	0.5295
Sikkim	0.3375	0.3457	0.3523	0.3609	0.3694	0.3782	0.3878	0.3979	0.4081	0.4183	0.4296	0.4413	0.4535	0.4658	0.4782	0.4903	0.5012	0.5115	0.5195	0.5360	0.5508	0.5653	0.5791	0.5929	0.6067	0.6204	0.6316
Tamil Nadu	0.3764	0.3851	0.3945	0.4054	0.4169	0.4285	0.4403	0.4518	0.4640	0.4756	0.4868	0.4963	0.5050	0.5131	0.5214	0.5310	0.5415	0.5524	0.5630	0.5752	0.5875	0.5994	0.6103	0.6212	0.6321	0.6404	0.6476
Telangana	0.3170	0.3251	0.3329	0.3421	0.3517	0.3621	0.3732	0.3835	0.3948	0.4060	0.4173	0.4281	0.4382	0.4486	0.4589	0.4700	0.4819	0.4948	0.5087	0.5211	0.5352	0.5485	0.5615	0.5742	0.5867	0.5993	0.6094
Tripura	0.3579	0.3622	0.3656	0.3720	0.3784	0.3863	0.3949	0.4040	0.4117	0.4248	0.4380	0.4511	0.4617	0.4704	0.4778	0.4847	0.4912	0.4969	0.5035	0.5108	0.5184	0.5272	0.5360	0.5450	0.5544	0.5642	0.5727
Uttar Pradesh	0.3196	0.3263	0.3319	0.3362	0.3400	0.3436	0.3477	0.3513	0.3546	0.3580	0.3621	0.3676	0.3742	0.3817	0.3897	0.3983	0.4067	0.4153	0.4233	0.4317	0.4408	0.4509	0.4616	0.4731	0.4852	0.4978	0.5083
Uttarakhand	0.3166	0.3242	0.3320	0.3399	0.3489	0.3584	0.3683	0.3786	0.3888	0.3997	0.4116	0.4237	0.4364	0.4492	0.4625	0.4766	0.4908	0.5053	0.5188	0.5354	0.5515	0.5679	0.5833	0.5983	0.6131	0.6276	0.6392
West Bengal	0.3660	0.3711	0.3762	0.3823	0.3888	0.3962	0.4039	0.4117	0.4204	0.4292	0.4380	0.4463	0.4540	0.4619	0.4696	0.4775	0.4863	0.4961	0.5062	0.5164	0.5264	0.5363	0.5459	0.5553	0.5650	0.5730	0.5803
The Six Minor Territories	0.4605	0.4680	0.4752	0.4823	0.4901	0.4981	0.5061	0.5156	0.5248	0.5339	0.5432	0.5524	0.5620	0.5719	0.5815	0.5918	0.6024	0.6129	0.6232	0.6339	0.6436	0.6535	0.6630	0.6724	0.6817	0.6912	0.6981
Andhra Pradesh, Urban	0.4337	0.4410	0.4472	0.4543	0.4616	0.4696	0.4787	0.4876	0.4983	0.5095	0.5213	0.5319	0.5412	0.5501	0.5587	0.5667	0.5755	0.5857	0.5951	0.6040	0.6133	0.6228	0.6317	0.6407	0.6503	0.6600	0.6679
Andhra Pradesh, Rural	0.2837	0.2911	0.2984	0.3072	0.3165	0.3267	0.3374	0.3473	0.3580	0.3684	0.3789	0.3889	0.3982	0.4074	0.4167	0.4255	0.4347	0.4452	0.4551	0.4647	0.4749	0.4854	0.4954	0.5057	0.5165	0.5276	0.5369
Arunachal Pradesh, Urban	0.4630	0.4688	0.4745	0.4809	0.4865	0.4933	0.4994	0.5056	0.5110	0.5157	0.5201	0.5249	0.5284	0.5316	0.5357	0.5407	0.5463	0.5522	0.5573	0.5653	0.5733	0.5818	0.5902	0.5993	0.6099	0.6210	0.6312

Appendix Table 5B. SDI Values by location, 1990-2016

Location	1990	1991	1992	1993	1994	1995	1996	1997	1998	1999	2000	2001	2002	2003	2004	2005	2006	2007	2008	2009	2010	2011	2012	2013	2014	2015	2016
Arunachal Pradesh, Rural	0.3092	0.3161	0.3232	0.3307	0.3376	0.3456	0.3533	0.3613	0.3691	0.3764	0.3838	0.3923	0.4006	0.4091	0.4187	0.4285	0.4384	0.4495	0.4602	0.4738	0.4876	0.5014	0.5147	0.5279	0.5410	0.5540	0.5647
Assam, Urban	0.4994	0.5036	0.5080	0.5132	0.5188	0.5247	0.5305	0.5360	0.5413	0.5488	0.5564	0.5637	0.5714	0.5786	0.5858	0.5924	0.5994	0.6060	0.6119	0.6175	0.6240	0.6305	0.6364	0.6426	0.6495	0.6567	0.6634
Assam, Rural	0.3415	0.3479	0.3534	0.3583	0.3632	0.3684	0.3736	0.3787	0.3840	0.3913	0.3989	0.4066	0.4149	0.4232	0.4317	0.4399	0.4486	0.4573	0.4656	0.4738	0.4829	0.4923	0.5015	0.5111	0.5215	0.5324	0.5419
Bihar, Urban	0.4412	0.4472	0.4530	0.4562	0.4591	0.4603	0.4629	0.4664	0.4667	0.4680	0.4712	0.4742	0.4788	0.4823	0.4868	0.4906	0.4978	0.5050	0.5134	0.5205	0.5288	0.5373	0.5459	0.5540	0.5614	0.5683	0.5746
Bihar, Rural	0.2813	0.2865	0.2916	0.2935	0.2944	0.2939	0.2945	0.2959	0.2950	0.2950	0.2969	0.2994	0.3038	0.3077	0.3128	0.3173	0.3249	0.3328	0.3417	0.3495	0.3587	0.3684	0.3789	0.3894	0.3996	0.4099	0.4190
Chhattisgarh, Urban	0.5000	0.5034	0.5067	0.5101	0.5135	0.5169	0.5214	0.5271	0.5326	0.5386	0.5433	0.5501	0.5571	0.5650	0.5723	0.5806	0.5902	0.6006	0.6112	0.6203	0.6285	0.6372	0.6452	0.6528	0.6611	0.6694	0.6767
Chhattisgarh, Rural	0.2509	0.2574	0.2639	0.2707	0.2779	0.2857	0.2936	0.3018	0.3102	0.3194	0.3277	0.3370	0.3458	0.3557	0.3654	0.3756	0.3869	0.3991	0.4117	0.4236	0.4352	0.4478	0.4606	0.4739	0.4878	0.5021	0.5135
Delhi, Urban	0.5305	0.5350	0.5388	0.5433	0.5490	0.5549	0.5610	0.5693	0.5775	0.5862	0.5961	0.6059	0.6157	0.6256	0.6356	0.6456	0.6557	0.6658	0.6757	0.6861	0.6959	0.7060	0.7159	0.7257	0.7357	0.7458	0.7530
Delhi, Rural	0.4570	0.4642	0.4706	0.4767	0.4832	0.4900	0.4967	0.5054	0.5147	0.5247	0.5356	0.5468	0.5579	0.5692	0.5803	0.5914	0.6025	0.6136	0.6243	0.6356	0.6465	0.6579	0.6692	0.6807	0.6920	0.7034	0.7130
Goa, Urban	0.5200	0.5265	0.5332	0.5414	0.5505	0.5608	0.5720	0.5823	0.5946	0.6057	0.6164	0.6262	0.6355	0.6421	0.6477	0.6541	0.6601	0.6651	0.6725	0.6812	0.6897	0.7011	0.7113	0.7213	0.7324	0.7436	0.7528
Goa, Rural	0.4409	0.4494	0.4581	0.4682	0.4789	0.4906	0.5031	0.5148	0.5287	0.5416	0.5544	0.5660	0.5767	0.5850	0.5923	0.6004	0.6083	0.6156	0.6252	0.6356	0.6456	0.6579	0.6685	0.6787	0.6898	0.7007	0.7101
Gujarat, Urban	0.4815	0.4870	0.4939	0.5010	0.5104	0.5194	0.5291	0.5381	0.5471	0.5553	0.5622	0.5686	0.5746	0.5810	0.5868	0.5942	0.6020	0.6106	0.6184	0.6266	0.6349	0.6429	0.6502	0.6569	0.6638	0.6708	0.6769
Gujarat, Rural	0.3342	0.3407	0.3486	0.3558	0.3644	0.3729	0.3819	0.3903	0.3991	0.4072	0.4141	0.4210	0.4277	0.4354	0.4430	0.4518	0.4611	0.4712	0.4804	0.4901	0.5001	0.5102	0.5202	0.5302	0.5410	0.5522	0.5614
Haryana, Urban	0.5062	0.5143	0.5210	0.5282	0.5360	0.5435	0.5518	0.5598	0.5672	0.5747	0.5823	0.5896	0.5964	0.6030	0.6097	0.6172	0.6256	0.6349	0.6446	0.6547	0.6647	0.6744	0.6831	0.6913	0.7003	0.7094	0.7171
Haryana, Rural	0.3544	0.3636	0.3719	0.3803	0.3889	0.3974	0.4068	0.4158	0.4243	0.4327	0.4413	0.4503	0.4593	0.4687	0.4783	0.4884	0.4994	0.5111	0.5230	0.5350	0.5469	0.5588	0.5700	0.5809	0.5925	0.6043	0.6142
Himachal Pradesh, Urban	0.5140	0.5221	0.5308	0.5397	0.5491	0.5580	0.5688	0.5793	0.5899	0.6017	0.6135	0.6244	0.6348	0.6449	0.6550	0.6649	0.6750	0.6847	0.6924	0.6994	0.7076	0.7154	0.7225	0.7293	0.7362	0.7430	0.7497
Himachal Pradesh, Rural	0.3599	0.3705	0.3814	0.3926	0.4043	0.4159	0.4291	0.4419	0.4549	0.4688	0.4822	0.4946	0.5061	0.5171	0.5281	0.5393	0.5511	0.5625	0.5741	0.5854	0.5976	0.6096	0.6210	0.6321	0.6433	0.6517	0.6593
Jammu and Kashmir, Urban	0.4515	0.4574	0.4615	0.4698	0.4779	0.4862	0.4941	0.5060	0.5159	0.5284	0.5402	0.5511	0.5619	0.5716	0.5813	0.5900	0.5989	0.6070	0.6155	0.6227	0.6293	0.6363	0.6430	0.6496	0.6566	0.6637	0.6707
Jammu and Kashmir, Rural	0.3068	0.3124	0.3168	0.3244	0.3316	0.3392	0.3464	0.3568	0.3661	0.3778	0.3892	0.4005	0.4120	0.4234	0.4352	0.4464	0.4577	0.4682	0.4791	0.4897	0.5002	0.5110	0.5216	0.5320	0.5426	0.5534	0.5624
Jharkhand, Urban	0.4920	0.4967	0.5020	0.5077	0.5140	0.5208	0.5272	0.5361	0.5448	0.5536	0.5608	0.5688	0.5766	0.5835	0.5914	0.5993	0.6069	0.6154	0.6223	0.6298	0.6382	0.6458	0.6526	0.6591	0.6659	0.6729	0.6794
Jharkhand, Rural	0.2688	0.2739	0.2794	0.2852	0.2916	0.2987	0.3051	0.3131	0.3214	0.3302	0.3380	0.3463	0.3544	0.3619	0.3703	0.3788	0.3872	0.3967	0.4052	0.4146	0.4255	0.4363	0.4475	0.4595	0.4722	0.4855	0.4964
Karnataka, Urban	0.4631	0.4707	0.4777	0.4860	0.4954	0.5050	0.5153	0.5251	0.5359	0.5464	0.5567	0.5655	0.5737	0.5813	0.5891	0.5980	0.6065	0.6155	0.6240	0.6311	0.6394	0.6474	0.6554	0.6635	0.6721	0.6809	0.6880
Karnataka, Rural	0.3064	0.3143	0.3219	0.3300	0.3386	0.3477	0.3573	0.3666	0.3770	0.3872	0.3972	0.4061	0.4148	0.4233	0.4322	0.4423	0.4526	0.4639	0.4750	0.4851	0.4961	0.5070	0.5179	0.5289	0.5403	0.5519	0.5614
Kerala, Urban	0.4597	0.4662	0.4738	0.4833	0.4940	0.5059	0.5182	0.5301	0.5420	0.5540	0.5649	0.5738	0.5820	0.5894	0.5966	0.6048	0.6127	0.6209	0.6291	0.6379	0.6462	0.6553	0.6642	0.6733	0.6833	0.6924	0.6989
Kerala, Rural	0.4177	0.4254	0.4331	0.4422	0.4524	0.4640	0.4761	0.4879	0.5001	0.5126	0.5241	0.5337	0.5428	0.5510	0.5591	0.5682	0.5771	0.5861	0.5952	0.6046	0.6134	0.6229	0.6324	0.6422	0.6530	0.6622	0.6692
Madhya Pradesh, Urban	0.4692	0.4760	0.4831	0.4903	0.4971	0.5040	0.5109	0.5172	0.5220	0.5271	0.5306	0.5348	0.5376	0.5414	0.5455	0.5503	0.5567	0.5639	0.5719	0.5814	0.5908	0.6003	0.6091	0.6176	0.6264	0.6348	0.6428
Madhya Pradesh, Rural	0.2797	0.2862	0.2917	0.2961	0.3004	0.3048	0.3094	0.3139	0.3177	0.3220	0.3255	0.3308	0.3357	0.3422	0.3487	0.3550	0.3621	0.3699	0.3782	0.3883	0.3990	0.4108	0.4231	0.4364	0.4507	0.4654	0.4776
Maharashtra, Urban	0.4932	0.4993	0.5058	0.5136	0.5216	0.5305	0.5401	0.5495	0.5582	0.5677	0.5761	0.5838	0.5910	0.5983	0.6065	0.6161	0.6266	0.6379	0.6484	0.6584	0.6687	0.6783	0.6868	0.6948	0.7032	0.7117	0.7189
Maharashtra, Rural	0.3641	0.3728	0.3819	0.3912	0.4003	0.4103	0.4206	0.4303	0.4394	0.4488	0.4567	0.4643	0.4718	0.4796	0.4881	0.4982	0.5096	0.5218	0.5333	0.5445	0.5558	0.5667	0.5767	0.5864	0.5965	0.6067	0.6153
Manipur, Urban	0.4268	0.4341	0.4407	0.4472	0.4527	0.4584	0.4647	0.4715	0.4787	0.4887	0.4981	0.5088	0.5190	0.5296	0.5414	0.5535	0.5640	0.5737	0.5823	0.5916	0.5998	0.6092	0.6182	0.6269	0.6361	0.6429	0.6497
Manipur, Rural	0.3408	0.3489	0.3563	0.3625	0.3666	0.3708	0.3754	0.3809	0.3875	0.3975	0.4077	0.4193	0.4303	0.4420	0.4552	0.4693	0.4820	0.4937	0.5042	0.5150	0.5246	0.5351	0.5452	0.5549	0.5650	0.5755	0.5847
Meghalaya, Urban	0.4730	0.4741	0.4740	0.4760	0.4794	0.4844	0.4901	0.4976	0.5057	0.5160	0.5293	0.5445	0.5588	0.5718	0.5837	0.5949	0.6046	0.6130	0.6209	0.6296	0.6381	0.6479	0.6570	0.6666	0.6769	0.6872	0.6956
Meghalaya, Rural	0.3066	0.3132	0.3187	0.3232	0.3253	0.3281	0.3312	0.3360	0.3424	0.3520	0.3647	0.3791	0.3929	0.4056	0.4174	0.4288	0.4398	0.4506	0.4611	0.4721	0.4829	0.4946	0.5054	0.5165	0.5280	0.5397	0.5496
Mizoram, Urban	0.4592	0.4661	0.4722	0.4795	0.4852	0.4916	0.5004	0.5076	0.5142	0.5218	0.5310	0.5396	0.5477	0.5549	0.5616	0.5684	0.5747	0.5808	0.5866	0.5934	0.6011	0.6083	0.6163	0.6245	0.6334	0.6428	0.6513
Mizoram, Rural	0.3701	0.3781	0.3852	0.3916	0.3946	0.3981	0.4038	0.4084	0.4135	0.4204	0.4297	0.4398	0.4507	0.4614	0.4719	0.4818	0.4914	0.5017	0.5118	0.5230	0.5348	0.5457	0.5572	0.5683	0.5795	0.5907	0.6002
Nagaland, Urban	0.5126	0.5174	0.5208	0.5249	0.5274	0.5296	0.5320	0.5361	0.5398	0.5443	0.5532	0.5641	0.5761	0.5860	0.5952	0.6042	0.6123	0.6219	0.6316	0.6411	0.6502	0.6604	0.6705	0.6804	0.6912	0.7023	0.7114
Nagaland, Rural	0.3788	0.3833	0.3866	0.3904	0.3928	0.3953	0.3988	0.4049	0.4118	0.4203	0.4348	0.4521	0.4693	0.4843	0.4983	0.5118	0.5241	0.5372	0.5503	0.5629	0.5747	0.5875	0.5996	0.6111	0.6218	0.6295	0.6370
Odisha, Urban	0.4380	0.4445	0.4499	0.4554	0.4613	0.4687	0.4744	0.4810	0.4882	0.4963	0.5038	0.5117	0.5189	0.5273	0.5363	0.5440	0.5522	0.5608	0.5686	0.5755	0.5835	0.5910	0.5993	0.6079	0.6174	0.6272	0.6354
Odisha, Rural	0.2811	0.2874	0.2930	0.2987	0.3045	0.3120	0.3179	0.3248	0.3326	0.3414	0.3497	0.3584	0.3665	0.3761	0.3868	0.3967	0.4079	0.4204	0.4322	0.4432	0.4553	0.4668	0.4786	0.4903	0.5023	0.5143	0.5242
Punjab, Urban	0.5126	0.5193	0.5259	0.5331	0.5407	0.5483	0.5561	0.5638	0.5708	0.5780	0.5853	0.5922	0.5984	0.6049	0.6112	0.6187	0.6267	0.6357	0.6446	0.6535	0.6622	0.6709	0.6793	0.6875	0.6959	0.7026	0.7090
Punjab, Rural	0.3639	0.3727	0.3814	0.3902	0.3990	0.4081	0.4177	0.4274	0.4371	0.4470	0.4566	0.4657	0.4742	0.4829	0.4919	0.5021	0.5126	0.5238	0.5347	0.5455	0.5559	0.5665	0.5766	0.5866	0.5964	0.6037	0.6108
Rajasthan, Urban	0.4330	0.4395	0.4462	0.4519	0.4589	0.4655	0.4733	0.4813	0.4889	0.4985	0.5068	0.5158	0.5228	0.5310	0.5385	0.5457	0.5535	0.5615	0.5693	0.5760	0.5846	0.5937	0.6022	0.6101	0.6180	0.6257	0.6324
Rajasthan, Rural	0.2728	0.2796	0.2864	0.2921	0.2987	0.3049	0.3119	0.3184	0.3240	0.3307	0.3369	0.3448	0.3516	0.3602	0.3685	0.3765	0.3854	0.3947	0.4040	0.4124	0.4229	0.4341	0.4453	0.4562	0.4673	0.4784	0.4874
Sikkim, Urban	0.4685	0.4773	0.4834	0.4911	0.4979	0.5042	0.5110	0.5181	0.5252	0.5327	0.5418	0.5510	0.5604	0.5699	0.5791	0.5880	0.5954	0.6014	0.6050	0.6175	0.6282	0.6389	0.6493	0.6603	0.6724	0.6850	0.6954
Sikkim, Rural	0.3209	0.3288	0.3352	0.3434	0.3517	0.3604	0.3698	0.3798	0.3899	0.3999	0.4108	0.4220	0.4337	0.4455	0.4573	0.4687	0.4789	0.4887	0.4964	0.5125	0.5271	0.5415	0.5554	0.5695	0.5838	0.5981	0.6098
Tamil Nadu, Urban	0.4528	0.4592	0.4666	0.4760	0.4856	0.4952	0.5052	0.5151	0.5260	0.5367	0.5475	0.5567	0.5652	0.5733	0.5813	0.5902	0.5995	0.6087	0.6170	0.6267	0.6365	0.6465	0.6563	0.6666	0.6775	0.6858	0.6933
Tamil Nadu, Rural	0.3327	0.3412	0.3497	0.3591	0.3688	0.3788	0.3891	0.3994	0.4108	0.4219	0.4331	0.4432	0.4528	0.4624	0.4721	0.4830	0.4943	0.5056	0.5161	0.5278	0.5395	0.5512	0.5624	0.5739	0.5858	0.5950	0.6032
Telangana, Urban	0.4402	0.4490	0.4567	0.4653	0.4741	0.4834	0.4935	0.5030	0.5144	0.5263	0.5387	0.5499	0.5596	0.5690	0.5776	0.5867	0.5964	0.6066	0.6175	0.6263	0.6372	0.6475	0.6578	0.6680	0.6787	0.6892	0.6981
Telangana, Rural	0.2653	0.2725	0.2797	0.2886	0.2980	0.3084	0.3196	0.3302	0.3417	0.3529	0.3643	0.3752	0.3854	0.3958	0.4061	0.4171	0.4286	0.4409	0.4543	0.4661	0.4797	0.4929	0.5061	0.5194	0.5331	0.5470	0.5582
Tripura, Urban	0.4674	0.4687	0.4692	0.4735	0.4785	0.4852	0.4926	0.5005	0.5068	0.5185	0.5303	0.5415	0.5493	0.5548	0.5587	0.5630	0.5670	0.5698	0.5739	0.5788	0.5844	0.5917	0.5994	0.6077	0.6172	0.6271	0.6354
Tripura, Rural	0.3360	0.3405	0.3441	0.3505	0.3569	0.3647	0.3733	0.3824	0.3900	0.4030	0.4160	0.4289	0.4393	0.4477	0.4549	0.4614	0.4672	0.4725	0.4787	0.4855	0.4927	0.5011	0.5095	0.5181	0.5276	0.5375	0.5463
Uttar Pradesh, Urban	0.4234	0.4296	0.4352	0.4404	0.4460	0.4513	0.4575	0.4631	0.4681	0.4729	0.4782	0.4838	0.4898	0.4960	0.5021	0.5089	0.5161	0.5236	0.5306	0.5380	0.5458	0.5539	0.5620	0.5699	0.5783	0.5867	0.5941
Uttar Pradesh, Rural	0.2888	0.2952	0.3005	0.3043	0.3075	0.3106	0.3142	0.3173	0.3203	0.3233	0.3272	0.3325	0.3391	0.3468	0.3550	0.3637	0.3723	0.3808	0.3889	0.3975	0.4069	0.4175	0.4288	0.4411	0.4542	0.4680	0.4793
Uttarakhand, Urban	0.4535	0.4588	0.4642	0.4694	0.4758	0.4823	0.4892	0.4963	0.5032	0.5106	0.5190	0.5276	0.5372	0.5465	0.5562	0.5667	0.5776	0.5894	0.6002	0.6145	0.6283	0.6425	0.6558	0.6687	0.6821	0.6952	0.7058
Uttarakhand, Rural	0.2767	0.2842	0.2918	0.2996	0.3086	0.3181	0.3281	0.3384	0.3488	0.3599	0.3722	0.3845	0.3974	0.4106	0.4243	0.4388	0.4534	0.4682	0.4819	0.4986	0.5149	0.5314	0.5469	0.5621	0.5774	0.5924	0.6043
West Bengal, Urban	0.4910	0.4956	0.5005	0.5066	0.5129	0.5199	0.5272	0.5348	0.5432	0.5517	0.5600	0.5676	0.5740	0.5802	0.5857	0.5912	0.5972	0.6044	0.6120	0.6193	0.6254	0.6318	0.6381	0.6444	0.6513	0.6583	0.6652
West Bengal, Rural	0.3124	0.3175	0.3226	0.3286	0.3352	0.3427	0.3505	0.3584	0.3672	0.3760	0.3844	0.3924	0.3997	0.4070	0.4141	0.4217	0.4305	0.4405	0.4511	0.4619	0.4725	0.4831	0.4934	0.5037	0.5142	0.5250	0.5330
The Six Minor Territories, Rural	0.3631	0.3709	0.3789	0.3873	0.3965	0.4063	0.4162	0.4276	0.4390	0.4506	0.4624	0.4743	0.4865	0.4991	0.5115	0.5248	0.5383	0.5519	0.5652	0.5788	0.5912	0.6034	0.6102	0.6167	0.6235	0.6304	0.6374
The Six Minor Territories, Urban	0.5234	0.5279	0.5328	0.5378	0.5438	0.5501	0.5566	0.5646	0.5719	0.5790	0.5862	0.5931	0.6002	0.6074	0.6142	0.6218	0.6297	0.6376	0.6453	0.6536	0.6612	0.6692	0.6769	0.6849	0.6933	0.7022	0.7099
Sub-Saharan Africa	0.3067	0.3113	0.3160	0.3206	0.3250	0.3293	0.3340	0.3385	0.3425	0.3462	0.3498	0.3537	0.3577	0.3617	0.3663	0.3715	0.3769	0.3828	0.3887	0.3945	0.4010	0.4082	0.4157	0.4229	0.4299	0.4367	0.4416
Central Sub-Saharan Africa	0.2984	0.3016	0.3045	0.3060	0.3071	0.3085	0.3103	0.3118	0.3136	0.3150	0.3156	0.3170	0.3195	0.3224	0.3267	0.3320	0.3381	0.3449	0.3521	0.3586	0.3662	0.3744	0.3824	0.3884	0.3940	0.4011	0.4063

Appendix Table 5B. SDI Values by location, 1990-2016

Location	1990	1991	1992	1993	1994	1995	1996	1997	1998	1999	2000	2001	2002	2003	2004	2005	2006	2007	2008	2009	2010	2011	2012	2013	2014	2015	2016
Angola	0.2522	0.2561	0.2597	0.2621	0.2647	0.2678	0.2719	0.2763	0.2814	0.2860	0.2894	0.2937	0.2991	0.3048	0.3115	0.3197	0.3294	0.3394	0.3487	0.3569	0.3660	0.3766	0.3883	0.3997	0.4109	0.4221	0.4309
Central African Republic	0.2466	0.2497	0.2522	0.2545	0.2576	0.2608	0.2630	0.2658	0.2692	0.2726	0.2757	0.2790	0.2826	0.2851	0.2881	0.2911	0.2947	0.2988	0.3028	0.3068	0.3113	0.3164	0.3217	0.3202	0.3186	0.3177	0.3161
Congo	0.3794	0.3864	0.3929	0.3989	0.4041	0.4095	0.4154	0.4208	0.4258	0.4300	0.4344	0.4389	0.4445	0.4499	0.4558	0.4618	0.4670	0.4709	0.4751	0.4799	0.4868	0.4945	0.5029	0.5109	0.5185	0.5258	0.5312
Democratic Republic of the Congo	0.2827	0.2837	0.2851	0.2843	0.2821	0.2799	0.2768	0.2729	0.2682	0.2635	0.2573	0.2513	0.2464	0.2434	0.2424	0.2430	0.2418	0.2414	0.2423	0.2438	0.2510	0.2602	0.2703	0.2822	0.2923	0.3036	0.3124
Equatorial Guinea	0.2904	0.2917	0.2970	0.3034	0.3106	0.3215	0.3425	0.3816	0.4091	0.4335	0.4590	0.4862	0.5094	0.5280	0.5476	0.5647	0.5786	0.5918	0.6048	0.6144	0.6236	0.6334	0.6428	0.6510	0.6584	0.6646	0.6693
Gabon	0.4576	0.4659	0.4738	0.4817	0.4897	0.4976	0.5058	0.5139	0.5214	0.5275	0.5310	0.5343	0.5395	0.5450	0.5510	0.5576	0.5636	0.5700	0.5761	0.5810	0.5863	0.5925	0.5990	0.6050	0.6109	0.6172	0.6224
Eastern Sub-Saharan Africa	0.2281	0.2340	0.2392	0.2441	0.2487	0.2536	0.2588	0.2637	0.2683	0.2728	0.2772	0.2818	0.2864	0.2911	0.2962	0.3017	0.3077	0.3144	0.3214	0.3288	0.3367	0.3451	0.3538	0.3625	0.3712	0.3797	0.3867
Burundi	0.1881	0.1929	0.1973	0.2004	0.2020	0.2022	0.2017	0.2012	0.2015	0.2025	0.2037	0.2055	0.2077	0.2103	0.2135	0.2169	0.2206	0.2244	0.2286	0.2336	0.2396	0.2458	0.2522	0.2590	0.2662	0.2723	0.2765
Comoros	0.2748	0.2819	0.2893	0.2967	0.3027	0.3094	0.3153	0.3207	0.3254	0.3300	0.3347	0.3401	0.3462	0.3525	0.3589	0.3656	0.3731	0.3805	0.3880	0.3955	0.4029	0.4104	0.4188	0.4275	0.4355	0.4433	0.4488
Djibouti	0.1989	0.2075	0.2155	0.2229	0.2296	0.2370	0.2444	0.2515	0.2583	0.2654	0.2724	0.2799	0.2875	0.2956	0.3039	0.3124	0.3215	0.3307	0.3403	0.3498	0.3594	0.3690	0.3789	0.3887	0.3986	0.4086	0.4157
Eritrea	0.1873	0.1931	0.1999	0.2081	0.2182	0.2292	0.2410	0.2538	0.2666	0.2789	0.2900	0.3000	0.3086	0.3152	0.3207	0.3257	0.3301	0.3346	0.3380	0.3418	0.3464	0.3524	0.3594	0.3667	0.3745	0.3827	0.3892
Ethiopia	0.1532	0.1558	0.1578	0.1605	0.1631	0.1665	0.1706	0.1751	0.1786	0.1824	0.1865	0.1915	0.1961	0.1999	0.2052	0.2120	0.2198	0.2291	0.2394	0.2496	0.2605	0.2721	0.2837	0.2953	0.3062	0.3168	0.3260
Kenya	0.3348	0.3463	0.3570	0.3668	0.3760	0.3845	0.3925	0.3996	0.4054	0.4104	0.4152	0.4200	0.4246	0.4293	0.4344	0.4397	0.4456	0.4522	0.4585	0.4652	0.4729	0.4810	0.4892	0.4975	0.5057	0.5139	0.5201
Madagascar	0.2548	0.2596	0.2636	0.2667	0.2695	0.2723	0.2753	0.2784	0.2818	0.2855	0.2895	0.2942	0.2973	0.3016	0.3062	0.3108	0.3146	0.3185	0.3231	0.3270	0.3311	0.3375	0.3440	0.3497	0.3554	0.3611	0.3654
Malawi	0.2046	0.2103	0.2146	0.2189	0.2225	0.2269	0.2320	0.2378	0.2431	0.2486	0.2539	0.2581	0.2628	0.2677	0.2732	0.2785	0.2835	0.2896	0.2960	0.3018	0.3077	0.3139	0.3196	0.3258	0.3335	0.3421	0.3484
Mozambique	0.1445	0.1485	0.1506	0.1533	0.1566	0.1584	0.1646	0.1720	0.1795	0.1864	0.1927	0.1997	0.2071	0.2139	0.2201	0.2257	0.2310	0.2362	0.2416	0.2473	0.2533	0.2599	0.2679	0.2772	0.2870	0.2971	0.3056
Rwanda	0.1814	0.1966	0.2083	0.2169	0.2196	0.2228	0.2259	0.2298	0.2335	0.2368	0.2403	0.2444	0.2500	0.2559	0.2632	0.2719	0.2823	0.2930	0.3051	0.3177	0.3302	0.3423	0.3539	0.3646	0.3743	0.3833	0.3904
Somalia	0.1868	0.1901	0.1921	0.1944	0.1953	0.1963	0.1975	0.1982	0.1989	0.1998	0.2011	0.2029	0.2054	0.2085	0.2122	0.2161	0.2204	0.2250	0.2300	0.2351	0.2403	0.2455	0.2507	0.2556	0.2603	0.2647	0.2679
Tanzania	0.2454	0.2512	0.2556	0.2593	0.2635	0.2705	0.2794	0.2876	0.2954	0.3024	0.3084	0.3142	0.3202	0.3263	0.3324	0.3389	0.3454	0.3523	0.3595	0.3668	0.3747	0.3835	0.3925	0.4016	0.4110	0.4205	0.4281
Uganda	0.1768	0.1836	0.1901	0.1963	0.2030	0.2091	0.2148	0.2203	0.2258	0.2317	0.2376	0.2438	0.2505	0.2574	0.2646	0.2722	0.2804	0.2892	0.2983	0.3078	0.3175	0.3277	0.3379	0.3484	0.3590	0.3696	0.3771
Zambia	0.2992	0.3074	0.3147	0.3213	0.3265	0.3312	0.3356	0.3396	0.3431	0.3464	0.3499	0.3535	0.3574	0.3616	0.3663	0.3714	0.3771	0.3847	0.3929	0.4013	0.4102	0.4193	0.4285	0.4372	0.4457	0.4539	0.4602
South Sudan	0.0824	0.0876	0.0921	0.0955	0.0983	0.1016	0.1053	0.1084	0.1113	0.1140	0.1167	0.1198	0.1231	0.1266	0.1303	0.1341	0.1383	0.1428	0.1476	0.1525	0.1579	0.1635	0.1696	0.1758	0.1817	0.1877	0.1916
Central	0.4130	0.4245	0.4350	0.4444	0.4533	0.4616	0.4695	0.4764	0.4823	0.4874	0.4920	0.4967	0.5011	0.5054	0.5100	0.5150	0.5206	0.5270	0.5332	0.5395	0.5460	0.5529	0.5597	0.5665	0.5730	0.5795	0.5847
Coast	0.2521	0.2639	0.2754	0.2860	0.2959	0.3049	0.3134	0.3210	0.3271	0.3321	0.3372	0.3426	0.3482	0.3542	0.3608	0.3680	0.3758	0.3846	0.3935	0.4031	0.4130	0.4233	0.4339	0.4445	0.4549	0.4653	0.4728
Eastern	0.3097	0.3218	0.3332	0.3436	0.3534	0.3624	0.3711	0.3787	0.3851	0.3906	0.3959	0.4014	0.4067	0.4122	0.4179	0.4240	0.4305	0.4378	0.4449	0.4522	0.4597	0.4674	0.4751	0.4828	0.4902	0.4976	0.5033
Nairobi	0.5261	0.5359	0.5447	0.5525	0.5600	0.5670	0.5738	0.5796	0.5847	0.5892	0.5934	0.5978	0.6021	0.6065	0.6114	0.6168	0.6231	0.6302	0.6372	0.6445	0.6521	0.6602	0.6685	0.6770	0.6855	0.6939	0.7008
North Eastern	0.0583	0.0594	0.0605	0.0616	0.0675	0.0752	0.0796	0.0815	0.0757	0.0682	0.0692	0.0703	0.0713	0.0723	0.0733	0.0744	0.0754	0.0765	0.0776	0.0786	0.0798	0.0812	0.0827	0.0844	0.0920	0.1117	0.1244
Nyanza	0.3182	0.3307	0.3426	0.3535	0.3638	0.3735	0.3827	0.3910	0.3982	0.4045	0.4106	0.4169	0.4230	0.4292	0.4358	0.4426	0.4501	0.4583	0.4663	0.4746	0.4829	0.4914	0.4999	0.5083	0.5164	0.5244	0.5305
Rift Valley	0.2921	0.3045	0.3164	0.3273	0.3374	0.3467	0.3555	0.3633	0.3697	0.3751	0.3806	0.3861	0.3916	0.3973	0.4034	0.4098	0.4167	0.4244	0.4320	0.4399	0.4481	0.4566	0.4653	0.4739	0.4824	0.4907	0.4968
Western	0.3323	0.3431	0.3531	0.3621	0.3705	0.3781	0.3852	0.3913	0.3962	0.4002	0.4042	0.4083	0.4123	0.4165	0.4209	0.4257	0.4311	0.4373	0.4432	0.4496	0.4561	0.4631	0.4702	0.4774	0.4843	0.4912	0.4964
Kiambu	0.4405	0.4516	0.4615	0.4703	0.4783	0.4857	0.4925	0.4983	0.5031	0.5071	0.5111	0.5152	0.5190	0.5227	0.5265	0.5308	0.5357	0.5415	0.5469	0.5529	0.5594	0.5665	0.5737	0.5811	0.5884	0.5955	0.6013
Kirinyaga	0.3887	0.3987	0.4080	0.4173	0.4273	0.4367	0.4458	0.4538	0.4607	0.4668	0.4725	0.4786	0.4843	0.4898	0.4953	0.5012	0.5076	0.5144	0.5205	0.5267	0.5331	0.5396	0.5460	0.5522	0.5582	0.5643	0.5691
Murang'a	0.3951	0.4057	0.4153	0.4237	0.4319	0.4397	0.4474	0.4543	0.4605	0.4662	0.4716	0.4769	0.4819	0.4869	0.4920	0.4972	0.5028	0.5091	0.5148	0.5203	0.5258	0.5314	0.5369	0.5422	0.5472	0.5522	0.5561
Nyandarua	0.3831	0.3948	0.4055	0.4148	0.4233	0.4314	0.4392	0.4461	0.4520	0.4574	0.4620	0.4662	0.4702	0.4744	0.4788	0.4836	0.4891	0.4958	0.5028	0.5102	0.5179	0.5260	0.5341	0.5422	0.5499	0.5577	0.5637
Nyeri	0.4194	0.4320	0.4433	0.4530	0.4617	0.4696	0.4770	0.4832	0.4883	0.4924	0.4960	0.4995	0.5030	0.5069	0.5117	0.5171	0.5236	0.5311	0.5385	0.5458	0.5527	0.5595	0.5659	0.5720	0.5774	0.5829	0.5873
Kilifi	0.1503	0.1664	0.1817	0.1969	0.2121	0.2258	0.2383	0.2492	0.2584	0.2665	0.2733	0.2798	0.2858	0.2909	0.2955	0.3006	0.3062	0.3134	0.3215	0.3304	0.3410	0.3523	0.3638	0.3755	0.3871	0.3984	0.4062
Kwale	0.2214	0.2338	0.2456	0.2546	0.2607	0.2659	0.2708	0.2750	0.2776	0.2796	0.2812	0.2829	0.2851	0.2888	0.2939	0.2996	0.3062	0.3144	0.3239	0.3343	0.3456	0.3573	0.3692	0.3812	0.3928	0.4042	0.4121
Lamu	0.2619	0.2687	0.2758	0.2833	0.2918	0.3004	0.3092	0.3174	0.3249	0.3319	0.3382	0.3441	0.3494	0.3548	0.3602	0.3654	0.3706	0.3760	0.3815	0.3881	0.3965	0.4056	0.4150	0.4247	0.4342	0.4437	0.4503
Mombasa	0.3598	0.3721	0.3837	0.3941	0.4037	0.4126	0.4212	0.4288	0.4349	0.4399	0.4436	0.4478	0.4522	0.4573	0.4632	0.4697	0.4768	0.4844	0.4914	0.4987	0.5083	0.5184	0.5287	0.5391	0.5495	0.5597	0.5675
TaitaTaveta	0.2763	0.2896	0.3025	0.3149	0.3271	0.3386	0.3494	0.3592	0.3671	0.3739	0.3795	0.3855	0.3920	0.3994	0.4078	0.4166	0.4260	0.4354	0.4442	0.4535	0.4638	0.4742	0.4844	0.4943	0.5037	0.5130	0.5197
TanaRiver	0.1074	0.1195	0.1320	0.1448	0.1571	0.1675	0.1764	0.1834	0.1878	0.1891	0.1875	0.1848	0.1820	0.1788	0.1753	0.1717	0.1686	0.1678	0.1708	0.1784	0.1908	0.2045	0.2191	0.2337	0.2477	0.2613	0.2707
Embu	0.3532	0.3646	0.3754	0.3858	0.3965	0.4068	0.4168	0.4259	0.4341	0.4415	0.4486	0.4560	0.4629	0.4689	0.4743	0.4798	0.4858	0.4925	0.4992	0.5063	0.5137	0.5213	0.5287	0.5359	0.5428	0.5497	0.5551
Isiolo	0.2702	0.2770	0.2835	0.2894	0.2948	0.3001	0.3055	0.3107	0.3154	0.3202	0.3254	0.3304	0.3344	0.3361	0.3358	0.3349	0.3338	0.3345	0.3362	0.3390	0.3431	0.3482	0.3539	0.3600	0.3662	0.3724	0.3767
Kitui	0.2552	0.2649	0.2742	0.2835	0.2932	0.3024	0.3113	0.3194	0.3267	0.3334	0.3394	0.3454	0.3513	0.3574	0.3639	0.3706	0.3778	0.3856	0.3937	0.4024	0.4111	0.4201	0.4291	0.4380	0.4466	0.4550	0.4614
Machakos	0.3297	0.3445	0.3584	0.3708	0.3824	0.3932	0.4033	0.4122	0.4196	0.4264	0.4322	0.4374	0.4422	0.4473	0.4530	0.4593	0.4663	0.4742	0.4824	0.4902	0.4975	0.5048	0.5120	0.5190	0.5258	0.5325	0.5379
Makueni	0.3008	0.3136	0.3254	0.3355	0.3440	0.3513	0.3581	0.3635	0.3671	0.3688	0.3691	0.3691	0.3698	0.3728	0.3782	0.3849	0.3930	0.4021	0.4114	0.4215	0.4318	0.4418	0.4513	0.4601	0.4678	0.4751	0.4803
Marsabit	0.2289	0.2337	0.2381	0.2420	0.2458	0.2495	0.2535	0.2573	0.2605	0.2636	0.2676	0.2723	0.2771	0.2824	0.2879	0.2934	0.2987	0.3042	0.3083	0.3117	0.3146	0.3180	0.3217	0.3258	0.3303	0.3350	0.3386
Meru	0.3185	0.3319	0.3446	0.3561	0.3668	0.3768	0.3863	0.3947	0.4018	0.4077	0.4133	0.4193	0.4249	0.4300	0.4347	0.4396	0.4448	0.4506	0.4553	0.4606	0.4674	0.4747	0.4824	0.4902	0.4978	0.5055	0.5115
TharakaNithi	0.3152	0.3272	0.3385	0.3488	0.3586	0.3679	0.3771	0.3855	0.3931	0.4002	0.4067	0.4135	0.4202	0.4276	0.4355	0.4437	0.4525	0.4619	0.4712	0.4802	0.4888	0.4973	0.5057	0.5139	0.5219	0.5298	0.5360
Nairobi	0.5261	0.5359	0.5447	0.5525	0.5600	0.5670	0.5738	0.5796	0.5847	0.5892	0.5934	0.5978	0.6021	0.6065	0.6114	0.6168	0.6231	0.6302	0.6372	0.6445	0.6521	0.6602	0.6685	0.6770	0.6855	0.6939	0.7008
Garissa	0.0624	0.0638	0.0651	0.0693	0.0746	0.0769	0.0769	0.0743	0.0728	0.0741	0.0753	0.0765	0.0777	0.0788	0.0800	0.0812	0.0825	0.0839	0.1063	0.1267	0.1411	0.1539	0.1659	0.1776	0.1899	0.2017	0.2100
Mandera	0.0555	0.0566	0.0645	0.0786	0.0883	0.0952	0.1001	0.1033	0.1020	0.0951	0.0862	0.0743	0.0704	0.0716	0.0726	0.0737	0.0747	0.0757	0.0766	0.0775	0.0785	0.0798	0.0814	0.0831	0.0850	0.0869	0.0886
Wajir	0.0557	0.0567	0.0577	0.0587	0.0597	0.0606	0.0616	0.0624	0.0632	0.0640	0.0646	0.0653	0.0660	0.0666	0.0673	0.0681	0.0689	0.0698	0.0707	0.0716	0.0726	0.0737	0.0750	0.0826	0.1013	0.1152	0.1242
HomaBay	0.2909	0.3013	0.3110	0.3203	0.3295	0.3380	0.3465	0.3542	0.3612	0.3679	0.3743	0.3796	0.3846	0.3896	0.3948	0.4007	0.4076	0.4161	0.4253	0.4351	0.4449	0.4548	0.4642	0.4727	0.4805	0.4879	0.4933
Kisii	0.3216	0.3357	0.3492	0.3622	0.3751	0.3872	0.3987	0.4087	0.4171	0.4246	0.4321	0.4394	0.4466	0.4541	0.4619	0.4699	0.4782	0.4868	0.4946	0.5022	0.5098	0.5177	0.5257	0.5338	0.5417	0.5496	0.5557
Kisumu	0.3407	0.3554	0.3689	0.3798	0.3883	0.3957	0.4027	0.4087	0.4134	0.4170	0.4210	0.4253	0.4298	0.4349	0.4411	0.4481	0.4561	0.4652	0.4750	0.4852	0.4945	0.5037	0.5123	0.5207	0.5286	0.5363	0.5421
Migori	0.2937	0.3043	0.3142	0.3239	0.3338	0.3434	0.3528	0.3616	0.3698	0.3778	0.3864	0.3953	0.4035	0.4106	0.4167	0.4225	0.4283	0.4346	0.4400	0.4455	0.4511	0.4575	0.4648	0.4726	0.4804	0.4884	0.4945
Nyamira	0.3855	0.3974	0.4086	0.4194	0.4300	0.4399	0.4494	0.4578	0.4645	0.4697	0.4758	0.4828	0.4896	0.4958	0.5014	0.5074	0.5139	0.5212	0.5283	0.5365	0.5454	0.5545	0.5634	0.5720	0.5801	0.5880	0.5940
Siaya	0.2638	0.2747	0.2851	0.2949	0.3048	0.3144	0.3240	0.3327	0.3404	0.3472	0.3546	0.3625	0.3707	0.3799	0.3901	0.4006	0.4113	0.4221	0.4320	0.4417	0.4509	0.4603	0.4698	0.4792	0.4885	0.4980	0.5054
Baringo	0.2801	0.2921	0.3035	0.3133	0.3216	0.3294	0.3371	0.3442	0.3509	0.3577	0.3656	0.3730	0.3792	0.3831	0.3854	0.3873	0.3897	0.3934	0.3983	0.4055	0.4136	0.4225	0.4316	0.4409	0.4498	0.4583	0.4646
Bomet	0.2675	0.2809	0.2933	0.3048	0.3161	0.3269	0.3376	0.3477	0.3566	0.3660	0.3769	0.3878	0.3984	0.4085	0.4182	0.4274	0.4364	0.4456	0.4536	0.4616	0.4698	0.4786	0.4877	0.4969	0.5057	0.5144	0.5209

Appendix Table 5B. SDI Values by location, 1990-2016

Location	1990	1991	1992	1993	1994	1995	1996	1997	1998	1999	2000	2001	2002	2003	2004	2005	2006	2007	2008	2009	2010	2011	2012	2013	2014	2015	2016
Elgeyo-Marakwet	0.2784	0.2908	0.3027	0.3130	0.3223	0.3310	0.3396	0.3475	0.3550	0.3623	0.3707	0.3795	0.3879	0.3951	0.4013	0.4078	0.4151	0.4240	0.4335	0.4437	0.4533	0.4631	0.4728	0.4825	0.4919	0.5010	0.5078
Kajiado	0.3250	0.3377	0.3498	0.3600	0.3683	0.3758	0.3828	0.3887	0.3926	0.3950	0.3986	0.4032	0.4087	0.4164	0.4263	0.4369	0.4480	0.4597	0.4706	0.4805	0.4889	0.4975	0.5064	0.5154	0.5244	0.5331	0.5395
Kericho	0.2422	0.2607	0.2783	0.2955	0.3125	0.3284	0.3431	0.3563	0.3678	0.3780	0.3881	0.3972	0.4057	0.4133	0.4204	0.4278	0.4360	0.4450	0.4550	0.4661	0.4764	0.4871	0.4979	0.5085	0.5187	0.5286	0.5360
Laikipia	0.3376	0.3490	0.3602	0.3725	0.3858	0.3983	0.4101	0.4202	0.4283	0.4348	0.4419	0.4491	0.4560	0.4629	0.4700	0.4779	0.4867	0.4965	0.5069	0.5178	0.5275	0.5372	0.5468	0.5563	0.5656	0.5747	0.5819
Nakuru	0.3207	0.3329	0.3445	0.3550	0.3647	0.3733	0.3815	0.3885	0.3941	0.3984	0.4030	0.4077	0.4131	0.4204	0.4299	0.4407	0.4526	0.4658	0.4787	0.4916	0.5034	0.5149	0.5260	0.5365	0.5464	0.5561	0.5634
Nandi	0.2704	0.2851	0.2993	0.3131	0.3265	0.3388	0.3501	0.3599	0.3675	0.3739	0.3816	0.3902	0.3990	0.4080	0.4172	0.4266	0.4362	0.4461	0.4552	0.4647	0.4735	0.4825	0.4914	0.5000	0.5083	0.5163	0.5220
Narok	0.2312	0.2441	0.2568	0.2692	0.2816	0.2934	0.3046	0.3145	0.3227	0.3281	0.3322	0.3358	0.3394	0.3451	0.3528	0.3607	0.3684	0.3753	0.3808	0.3875	0.3942	0.4018	0.4099	0.4182	0.4265	0.4348	0.4406
Samburu	0.2162	0.2202	0.2238	0.2268	0.2293	0.2314	0.2334	0.2348	0.2354	0.2348	0.2355	0.2377	0.2409	0.2454	0.2512	0.2574	0.2638	0.2701	0.2753	0.2812	0.2865	0.2917	0.2969	0.3023	0.3076	0.3128	0.3164
TransNzoia	0.2784	0.2962	0.3129	0.3278	0.3411	0.3533	0.3644	0.3741	0.3822	0.3896	0.3979	0.4054	0.4120	0.4176	0.4223	0.4270	0.4322	0.4384	0.4451	0.4532	0.4616	0.4706	0.4798	0.4889	0.4977	0.5064	0.5130
Turkana	0.2269	0.2311	0.2350	0.2385	0.2415	0.2441	0.2469	0.2492	0.2506	0.2501	0.2491	0.2484	0.2482	0.2488	0.2505	0.2530	0.2559	0.2600	0.2645	0.2685	0.2708	0.2733	0.2762	0.2794	0.2830	0.2867	0.2895
UasinGishu	0.3289	0.3421	0.3547	0.3659	0.3760	0.3857	0.3952	0.4042	0.4130	0.4220	0.4322	0.4428	0.4530	0.4619	0.4697	0.4775	0.4857	0.4948	0.5040	0.5133	0.5216	0.5302	0.5389	0.5475	0.5558	0.5639	0.5698
WestPokot	0.2300	0.2403	0.2500	0.2579	0.2642	0.2697	0.2747	0.2786	0.2805	0.2812	0.2827	0.2843	0.2862	0.2888	0.2923	0.2965	0.3013	0.3076	0.3142	0.3214	0.3281	0.3353	0.3429	0.3507	0.3588	0.3669	0.3729
Bungoma	0.3159	0.3265	0.3363	0.3451	0.3537	0.3620	0.3703	0.3778	0.3850	0.3918	0.3990	0.4065	0.4135	0.4199	0.4258	0.4316	0.4375	0.4440	0.4496	0.4555	0.4620	0.4691	0.4765	0.4841	0.4916	0.4990	0.5047
Busia	0.3307	0.3409	0.3505	0.3585	0.3653	0.3713	0.3769	0.3817	0.3849	0.3870	0.3900	0.3930	0.3960	0.3991	0.4024	0.4060	0.4102	0.4154	0.4207	0.4266	0.4329	0.4397	0.4467	0.4538	0.4608	0.4677	0.4728
Kakamega	0.3322	0.3438	0.3548	0.3647	0.3740	0.3825	0.3904	0.3969	0.4017	0.4051	0.4083	0.4112	0.4140	0.4173	0.4214	0.4261	0.4315	0.4375	0.4437	0.4501	0.4566	0.4634	0.4703	0.4772	0.4839	0.4906	0.4958
Vihiga	0.3611	0.3697	0.3775	0.3848	0.3913	0.3967	0.4015	0.4051	0.4076	0.4087	0.4102	0.4123	0.4148	0.4180	0.4224	0.4284	0.4362	0.4461	0.4563	0.4669	0.4765	0.4854	0.4933	0.5005	0.5066	0.5126	0.5167
Southern Sub-Saharan Africa	0.5581	0.5640	0.5700	0.5767	0.5832	0.5887	0.5949	0.6010	0.6060	0.6107	0.6153	0.6201	0.6249	0.6297	0.6350	0.6404	0.6459	0.6513	0.6564	0.6612	0.6664	0.6720	0.6772	0.6814	0.6850	0.6885	0.6914
Botswana	0.5080	0.5182	0.5275	0.5362	0.5441	0.5518	0.5603	0.5693	0.5776	0.5865	0.5949	0.6022	0.6093	0.6162	0.6234	0.6310	0.6385	0.6446	0.6502	0.6556	0.6619	0.6688	0.6754	0.6823	0.6892	0.6955	0.7009
Lesotho	0.3700	0.3763	0.3831	0.3901	0.3971	0.4034	0.4101	0.4167	0.4229	0.4287	0.4350	0.4417	0.4481	0.4546	0.4618	0.4689	0.4754	0.4822	0.4893	0.4963	0.5043	0.5128	0.5212	0.5293	0.5376	0.5455	0.5516
Namibia	0.4275	0.4334	0.4412	0.4501	0.4594	0.4687	0.4783	0.4878	0.4968	0.5053	0.5139	0.5223	0.5305	0.5385	0.5471	0.5552	0.5627	0.5693	0.5752	0.5803	0.5855	0.5912	0.5972	0.6037	0.6109	0.6182	0.6242
South Africa	0.5955	0.6009	0.6064	0.6127	0.6188	0.6240	0.6297	0.6355	0.6402	0.6446	0.6489	0.6533	0.6583	0.6634	0.6690	0.6752	0.6817	0.6883	0.6948	0.7010	0.7073	0.7138	0.7195	0.7237	0.7271	0.7304	0.7336
Swaziland	0.4541	0.4620	0.4698	0.4775	0.4845	0.4915	0.4982	0.5041	0.5092	0.5137	0.5181	0.5221	0.5250	0.5277	0.5307	0.5338	0.5367	0.5381	0.5383	0.5394	0.5443	0.5526	0.5618	0.5709	0.5784	0.5844	0.5890
Zimbabwe	0.4322	0.4428	0.4517	0.4607	0.4698	0.4773	0.4857	0.4926	0.4980	0.5020	0.5048	0.5071	0.5075	0.5056	0.5026	0.4981	0.4919	0.4854	0.4768	0.4719	0.4694	0.4700	0.4738	0.4793	0.4853	0.4915	0.4968
Eastern Cape	0.5429	0.5472	0.5515	0.5567	0.5621	0.5670	0.5727	0.5785	0.5829	0.5870	0.5913	0.5957	0.6008	0.6059	0.6118	0.6185	0.6257	0.6332	0.6404	0.6466	0.6530	0.6596	0.6654	0.6697	0.6730	0.6762	0.6792
Free State	0.5914	0.5966	0.6023	0.6093	0.6168	0.6235	0.6307	0.6371	0.6414	0.6451	0.6485	0.6522	0.6565	0.6610	0.6661	0.6718	0.6779	0.6842	0.6904	0.6962	0.7021	0.7083	0.7137	0.7177	0.7210	0.7241	0.7272
Gauteng	0.6917	0.6950	0.6982	0.7016	0.7046	0.7066	0.7090	0.7120	0.7148	0.7178	0.7209	0.7243	0.7277	0.7312	0.7353	0.7398	0.7445	0.7492	0.7539	0.7585	0.7633	0.7683	0.7726	0.7755	0.7775	0.7797	0.7819
KwaZulu-Natal	0.5709	0.5761	0.5814	0.5877	0.5939	0.5989	0.6047	0.6104	0.6150	0.6194	0.6240	0.6287	0.6337	0.6389	0.6447	0.6511	0.6580	0.6651	0.6721	0.6786	0.6852	0.6920	0.6980	0.7025	0.7060	0.7095	0.7128
Limpopo	0.5108	0.5175	0.5243	0.5322	0.5401	0.5468	0.5543	0.5619	0.5676	0.5723	0.5768	0.5818	0.5880	0.5945	0.6017	0.6098	0.6184	0.6272	0.6357	0.6432	0.6508	0.6586	0.6654	0.6706	0.6748	0.6787	0.6823
Mpumalanga	0.5567	0.5632	0.5697	0.5770	0.5838	0.5889	0.5947	0.6008	0.6056	0.6102	0.6147	0.6196	0.6252	0.6310	0.6374	0.6445	0.6519	0.6594	0.6669	0.6740	0.6811	0.6884	0.6948	0.6995	0.7033	0.7070	0.7105
North-West	0.5757	0.5805	0.5849	0.5896	0.5938	0.5968	0.6004	0.6043	0.6082	0.6127	0.6177	0.6228	0.6279	0.6330	0.6386	0.6448	0.6513	0.6580	0.6645	0.6705	0.6768	0.6832	0.6887	0.6927	0.6959	0.6990	0.7021
Northern Cape	0.5896	0.5938	0.5980	0.6026	0.6069	0.6101	0.6142	0.6189	0.6226	0.6260	0.6295	0.6331	0.6372	0.6414	0.6463	0.6517	0.6575	0.6636	0.6696	0.6754	0.6814	0.6877	0.6932	0.6972	0.7004	0.7036	0.7068
Western Cape	0.6813	0.6841	0.6871	0.6907	0.6946	0.6981	0.7026	0.7076	0.7111	0.7134	0.7153	0.7173	0.7195	0.7219	0.7247	0.7282	0.7319	0.7358	0.7397	0.7435	0.7476	0.7520	0.7557	0.7581	0.7598	0.7617	0.7636
Western Sub-Saharan Africa	0.2868	0.2898	0.2934	0.2973	0.3012	0.3053	0.3099	0.3143	0.3185	0.3222	0.3261	0.3304	0.3348	0.3394	0.3450	0.3511	0.3572	0.3637	0.3699	0.3760	0.3828	0.3906	0.3991	0.4073	0.4153	0.4225	0.4278
Benin	0.2168	0.2220	0.2273	0.2326	0.2373	0.2419	0.2469	0.2520	0.2571	0.2622	0.2674	0.2725	0.2776	0.2826	0.2877	0.2928	0.2981	0.3038	0.3096	0.3152	0.3205	0.3257	0.3304	0.3346	0.3398	0.3461	0.3514
Burkina Faso	0.1254	0.1301	0.1360	0.1414	0.1456	0.1501	0.1550	0.1598	0.1647	0.1693	0.1733	0.1775	0.1818	0.1862	0.1906	0.1954	0.2005	0.2058	0.2115	0.2174	0.2246	0.2337	0.2436	0.2533	0.2620	0.2691	0.2744
Cameroon	0.3409	0.3450	0.3491	0.3527	0.3562	0.3599	0.3644	0.3691	0.3739	0.3785	0.3831	0.3875	0.3920	0.3965	0.4003	0.4033	0.4062	0.4092	0.4122	0.4153	0.4187	0.4228	0.4276	0.4328	0.4387	0.4452	0.4505
Cape Verde	0.3172	0.3247	0.3325	0.3404	0.3490	0.3578	0.3672	0.3769	0.3874	0.3990	0.4103	0.4209	0.4314	0.4418	0.4521	0.4622	0.4726	0.4837	0.4946	0.5046	0.5142	0.5242	0.5336	0.5424	0.5510	0.5592	0.5658
Chad	0.1828	0.1867	0.1906	0.1937	0.1969	0.1998	0.2027	0.2058	0.2088	0.2116	0.2138	0.2166	0.2191	0.2222	0.2282	0.2350	0.2409	0.2465	0.2518	0.2566	0.2624	0.2678	0.2736	0.2796	0.2849	0.2900	0.2950
Cote d'Ivoire	0.2774	0.2873	0.2968	0.3058	0.3134	0.3201	0.3266	0.3324	0.3372	0.3412	0.3445	0.3477	0.3507	0.3531	0.3554	0.3577	0.3604	0.3630	0.3661	0.3694	0.3733	0.3769	0.3820	0.3879	0.3942	0.4010	0.4070
The Gambia	0.2543	0.2551	0.2552	0.2549	0.2542	0.2536	0.2551	0.2590	0.2636	0.2686	0.2741	0.2798	0.2847	0.2898	0.2953	0.3005	0.3053	0.3099	0.3147	0.3196	0.3248	0.3292	0.3341	0.3391	0.3437	0.3486	0.3525
Ghana	0.3562	0.3659	0.3752	0.3840	0.3925	0.4008	0.4088	0.4160	0.4215	0.4255	0.4286	0.4308	0.4333	0.4355	0.4378	0.4404	0.4431	0.4455	0.4476	0.4490	0.4511	0.4568	0.4661	0.4766	0.4860	0.4938	0.4994
Guinea	0.2134	0.2168	0.2200	0.2232	0.2264	0.2297	0.2335	0.2370	0.2403	0.2432	0.2459	0.2486	0.2515	0.2543	0.2572	0.2605	0.2643	0.2683	0.2730	0.2773	0.2822	0.2876	0.2933	0.2991	0.3052	0.3111	0.3159
Guinea-Bissau	0.2084	0.2129	0.2171	0.2216	0.2263	0.2311	0.2363	0.2422	0.2460	0.2502	0.2543	0.2584	0.2623	0.2670	0.2701	0.2743	0.2785	0.2830	0.2879	0.2927	0.2980	0.3045	0.3105	0.3163	0.3220	0.3281	0.3331
Liberia	0.2184	0.2179	0.2155	0.2110	0.2044	0.1960	0.1863	0.1829	0.1838	0.1899	0.2039	0.2185	0.2338	0.2406	0.2467	0.2525	0.2584	0.2641	0.2699	0.2758	0.2821	0.2893	0.2975	0.3065	0.3144	0.3213	0.3267
Mali	0.1380	0.1419	0.1457	0.1497	0.1534	0.1570	0.1607	0.1645	0.1684	0.1723	0.1759	0.1804	0.1851	0.1902	0.1950	0.1999	0.2053	0.2111	0.2172	0.2235	0.2300	0.2366	0.2431	0.2494	0.2561	0.2624	0.2674
Mauritania	0.3218	0.3271	0.3324	0.3382	0.3437	0.3494	0.3556	0.3611	0.3663	0.3716	0.3764	0.3812	0.3860	0.3910	0.3962	0.4018	0.4098	0.4173	0.4243	0.4307	0.4376	0.4451	0.4525	0.4599	0.4678	0.4754	0.4818
Niger	0.1377	0.1392	0.1401	0.1407	0.1412	0.1415	0.1422	0.1429	0.1445	0.1463	0.1476	0.1495	0.1516	0.1540	0.1560	0.1582	0.1607	0.1632	0.1661	0.1687	0.1718	0.1751	0.1792	0.1834	0.1877	0.1919	0.1955
Nigeria	0.3218	0.3223	0.3243	0.3269	0.3300	0.3333	0.3374	0.3413	0.3450	0.3485	0.3530	0.3582	0.3636	0.3695	0.3767	0.3846	0.3920	0.3997	0.4069	0.4143	0.4227	0.4325	0.4434	0.4538	0.4640	0.4730	0.4792
Sao Tome and Principe	0.2889	0.2939	0.2990	0.3043	0.3096	0.3148	0.3205	0.3258	0.3308	0.3355	0.3403	0.3457	0.3513	0.3571	0.3631	0.3692	0.3757	0.3818	0.3882	0.3933	0.3978	0.4026	0.4078	0.4129	0.4195	0.4275	0.4338
Senegal	0.2402	0.2453	0.2507	0.2568	0.2631	0.2697	0.2765	0.2832	0.2897	0.2951	0.2989	0.3019	0.3043	0.3067	0.3090	0.3116	0.3143	0.3173	0.3204	0.3237	0.3272	0.3313	0.3356	0.3401	0.3449	0.3499	0.3540
Sierra Leone	0.2175	0.2216	0.2247	0.2286	0.2331	0.2371	0.2406	0.2434	0.2458	0.2468	0.2488	0.2518	0.2569	0.2623	0.2678	0.2733	0.2792	0.2857	0.2924	0.2992	0.3060	0.3131	0.3218	0.3322	0.3415	0.3476	0.3521
Togo	0.2480	0.2545	0.2614	0.2670	0.2734	0.2805	0.2879	0.2955	0.3014	0.3069	0.3118	0.3166	0.3214	0.3263	0.3311	0.3356	0.3408	0.3460	0.3508	0.3559	0.3613	0.3671	0.3731	0.3788	0.3847	0.3916	0.3974

Appendix Table 6: GBD world population age standard		
**Age**	**Percent of Population**	**Rounded**
ENN	0.035667	0.04
LNN	0.106232	0.11
PNN	1.687834	1.69
0-1	1.829733	1.83
1-4	7.182279	7.18
5-9	8.692810	8.69
10-14	8.395204	8.40
15-19	8.098376	8.10
20-24	7.814477	7.81
25-29	7.559828	7.56
30-34	7.248519	7.25
35-39	6.855877	6.86
40-44	6.384231	6.38
45-49	5.849079	5.85
50-54	5.270976	5.27
55-59	4.678016	4.68
60-64	4.057540	4.06
65-69	3.359068	3.36
70-74	2.629266	2.63
75-79	1.896681	1.90
80-84	1.213480	1.21
85-89	0.644468	0.64
90-94	0.257603	0.26
95+	0.082490	0.08

Appendix Table 7. Number of all-cause mortality data sources by type and location, 1950–2016					
**Geography**	**SBH**	**CBH**	**HH**	**SIBS**	**VR/SRS/DSP**
Hong Kong Special Administrative Region of China	2	0	0	0	64
Macao Special Administrative Region of China	1	0	0	0	28
China (without Hong Kong and Macao)	4	0	55	0	16
Anhui	4	0	35	0	11
Beijing	4	0	34	0	17
Chongqing	3	0	34	0	0
Fujian	4	0	34	0	18
Gansu	4	0	34	0	18
Guangdong	4	0	34	0	11
Guangxi	4	0	34	0	18
Guizhou	4	0	34	0	0
Hainan	3	0	34	0	0
Hebei	4	0	34	0	11
Heilongjiang	4	0	33	0	11
Henan	4	0	41	0	11
Hubei	4	0	34	0	18
Hunan	4	0	34	0	11
Inner Mongolia	4	0	34	0	18
Jiangsu	3	0	34	0	18
Jiangxi	4	0	34	0	11
Jilin	4	0	35	0	0
Liaoning	4	0	34	0	18
Ningxia	4	0	34	0	0
Qinghai	4	0	35	0	0
Shaanxi	4	0	34	0	0
Shandong	4	0	34	0	18
Shanghai	4	0	35	0	14
Shanxi	4	0	34	0	0
Sichuan	4	0	33	0	0
Tianjin	4	0	34	0	17
Tibet	3	0	25	0	18
Xinjiang	4	0	31	0	11
Yunnan	4	0	34	0	11
Zhejiang	4	0	34	0	18
North Korea	0	0	1	0	0
Taiwan (Province of China)	0	0	0	0	60
Cambodia	10	5	0	4	0
Indonesia	43	15	0	5	0
Aceh	30	2	0	4	0
Bali	38	3	2	5	0
Bangka-Belitung Islands	24	3	2	4	0
Banten	32	3	2	4	0
Bengkulu	34	2	2	5	0
Gorontalo	22	2	0	5	0
Jakarta	38	3	4	5	0
Jambi	33	2	2	5	0
West Java	38	3	5	5	0
Central Java	38	3	5	5	0
East Java	38	3	5	5	0

Appendix Table 7. Number of all-cause mortality data sources by type and location, 1950–2016					
Geography	SBH	CBH	HH	SIBS	VR/SRS/DSP
West Kalimantan	34	2	2	5	0
South Kalimantan	38	3	2	5	0
Central Kalimantan	35	3	3	5	0
East Kalimantan	34	4	1	5	0
North Kalimantan	17	2	0	0	0
Riau Islands	28	3	0	5	0
Lampung	37	3	4	5	0
Maluku	32	3	0	4	0
North Maluku	24	3	0	3	0
West Nusa Tenggara	38	3	5	5	0
East Nusa Tenggara	34	3	5	5	0
Papua	28	3	0	4	0
West Papua	28	3	0	4	0
Riau	36	3	2	5	0
West Sulawesi	29	3	1	3	0
South Sulawesi	38	3	5	5	0
Central Sulawesi	34	2	5	5	0
Southeast Sulawesi	34	3	3	5	0
North Sulawesi	34	2	1	5	0
West Sumatra	38	3	5	5	0
South Sumatra	38	3	4	5	0
North Sumatra	38	3	5	5	0
Yogyakarta	38	3	1	5	0
Laos	3	2	0	1	0
Malaysia	3	1	0	0	35
Maldives	6	1	0	0	27
Mauritius	0	0	0	0	65
Myanmar	5	1	0	0	0
Philippines	11	6	0	2	61
Sri Lanka	5	5	0	0	58
Seychelles	2	0	1	0	45
Thailand	11	2	0	0	62
Timor-Leste	5	5	0	3	0
Vietnam	15	3	2	0	0
American Samoa	0	0	0	0	50
Federated States of Micronesia	4	0	0	0	0
Fiji	7	1	0	0	54
Guam	0	0	0	0	59
Kiribati	7	1	0	0	11
Marshall Islands	2	1	0	0	15
Northern Mariana Islands	0	0	0	0	16
Papua New Guinea	5	2	0	0	0
Samoa	5	1	2	0	18
Solomon Islands	4	1	0	0	0
Tonga	3	1	1	0	15
Vanuatu	4	1	0	0	0
Armenia	5	4	0	0	35
Azerbaijan	4	4	0	0	31
Georgia	3	3	0	0	31

Appendix Table 7. Number of all-cause mortality data sources by type and location, 1950–2016					
**Geography**	**SBH**	**CBH**	**HH**	**SIBS**	**VR/SRS/DSP**
Kazakhstan	8	2	0	0	33
Kyrgyzstan	7	4	0	0	35
Mongolia	8	3	0	0	28
Tajikistan	7	2	0	0	29
Turkmenistan	2	2	0	0	31
Uzbekistan	4	2	0	0	29
Albania	5	2	0	0	33
Bosnia and Herzegovina	0	0	0	0	19
Bulgaria	4	0	0	0	64
Croatia	0	0	0	0	30
Czech Republic	0	0	0	0	66
Hungary	1	0	0	0	66
Macedonia	2	0	0	0	25
Montenegro	1	0	4	0	19
Poland	1	0	0	0	65
Romania	4	2	0	0	60
Serbia	2	0	4	0	10
Slovakia	0	0	0	0	65
Slovenia	0	0	0	0	34
Belarus	2	0	0	0	56
Estonia	1	0	0	0	56
Latvia	1	0	0	0	56
Lithuania	0	0	0	0	57
Moldova	4	3	0	0	33
Russia	1	0	0	0	56
Ukraine	5	2	0	0	56
Brunei	1	0	0	0	45
Japan	0	0	0	0	65
Aichi	0	0	0	0	35
Akita	0	0	0	0	35
Aomori	0	0	0	0	35
Chiba	0	0	0	0	35
Ehime	0	0	0	0	35
Fukui	0	0	0	0	35
Fukuoka	0	0	0	0	35
Fukushima	0	0	0	0	35
Gifu	0	0	0	0	35
Gunma	0	0	0	0	35
Hiroshima	0	0	0	0	35
Hokkaido	0	0	0	0	35
Hyogo	0	0	0	0	35
Ibaraki	0	0	0	0	35
Ishikawa	0	0	0	0	35
Iwate	0	0	0	0	35
Kagawa	0	0	0	0	35
Kagoshima	0	0	0	0	35
Kanagawa	0	0	0	0	35
Kochi	0	0	0	0	35
Kumamoto	0	0	0	0	35

Appendix Table 7. Number of all-cause mortality data sources by type and location, 1950–2016					
Geography	SBH	CBH	HH	SIBS	VR/SRS/DSP
Kyoto	0	0	0	0	35
Mie	0	0	0	0	35
Miyagi	0	0	0	0	35
Miyazaki	0	0	0	0	35
Nagano	0	0	0	0	35
Nagasaki	0	0	0	0	35
Nara	0	0	0	0	35
Niigata	0	0	0	0	35
Oita	0	0	0	0	35
Okayama	0	0	0	0	35
Okinawa	0	0	0	0	35
Osaka	0	0	0	0	35
Saga	0	0	0	0	35
Saitama	0	0	0	0	35
Shiga	0	0	0	0	35
Shimane	0	0	0	0	35
Shizuoka	0	0	0	0	35
Tochigi	0	0	0	0	35
Tokushima	0	0	0	0	35
Tokyo	0	0	0	0	35
Tottori	0	0	0	0	35
Toyama	0	0	0	0	35
Wakayama	0	0	0	0	35
Yamagata	0	0	0	0	35
Yamaguchi	0	0	0	0	35
Yamanashi	0	0	0	0	35
South Korea	6	1	0	0	37
Singapore	0	0	0	0	64
Australia	0	0	0	0	65
New Zealand	0	0	0	0	65
Andorra	0	0	0	0	12
Austria	0	0	0	0	65
Belgium	2	0	0	0	65
Cyprus	3	0	0	0	41
Denmark	0	0	0	0	65
Finland	0	0	0	0	66
France	0	0	0	0	65
Germany	0	0	0	0	59
Greece	0	0	0	0	64
Iceland	0	0	0	0	66
Ireland	0	0	2	0	65
Israel	0	0	0	0	65
Italy	0	0	0	0	64
Luxembourg	0	0	0	0	64
Malta	0	0	0	0	65
Netherlands	0	0	0	0	66
Norway	0	0	0	0	65
Portugal	1	1	0	0	63
Spain	0	0	0	0	65

Appendix Table 7. Number of all-cause mortality data sources by type and location, 1950–2016					
**Geography**	**SBH**	**CBH**	**HH**	**SIBS**	**VR/SRS/DSP**
Sweden	0	0	0	0	65
Stockholm	0	0	0	0	29
Sweden except Stockholm	0	0	0	0	29
Switzerland	1	0	0	0	65
United Kingdom	0	0	0	0	63
England	0	0	0	0	35
East Midlands	0	0	0	0	35
Derby	0	0	0	0	35
Derbyshire	0	0	0	0	35
Leicester	0	0	0	0	35
Leicestershire	0	0	0	0	35
Lincolnshire	0	0	0	0	35
Northamptonshire	0	0	0	0	35
Nottingham	0	0	0	0	35
Nottinghamshire	0	0	0	0	35
Rutland	0	0	0	0	35
East of England	0	0	0	0	35
Bedford	0	0	0	0	35
Cambridgeshire	0	0	0	0	35
Central Bedfordshire	0	0	0	0	35
Essex	0	0	0	0	35
Hertfordshire	0	0	0	0	35
Luton	0	0	0	0	35
Norfolk	0	0	0	0	35
Peterborough	0	0	0	0	35
Southend-on-Sea	0	0	0	0	35
Suffolk	0	0	0	0	35
Thurrock	0	0	0	0	35
Greater London	0	0	0	0	35
Barking and Dagenham	0	0	0	0	35
Barnet	0	0	0	0	35
Bexley	0	0	0	0	35
Brent	0	0	0	0	35
Bromley	0	0	0	0	35
Camden	0	0	0	0	35
Croydon	0	0	0	0	35
Ealing	0	0	0	0	35
Enfield	0	0	0	0	35
Greenwich	0	0	0	0	35
Hackney	0	0	0	0	35
Hammersmith and Fulham	0	0	0	0	35
Haringey	0	0	0	0	35
Harrow	0	0	0	0	35
Havering	0	0	0	0	35
Hillingdon	0	0	0	0	35
Hounslow	0	0	0	0	35
Islington	0	0	0	0	35
Kensington and Chelsea	0	0	0	0	35
Kingston upon Thames	0	0	0	0	35

Appendix Table 7. Number of all-cause mortality data sources by type and location, 1950–2016					
Geography	SBH	CBH	HH	SIBS	VR/SRS/DSP
Lambeth	0	0	0	0	35
Lewisham	0	0	0	0	35
Merton	0	0	0	0	35
Newham	0	0	0	0	35
Redbridge	0	0	0	0	35
Richmond upon Thames	0	0	0	0	35
Southwark	0	0	0	0	35
Sutton	0	0	0	0	35
Tower Hamlets	0	0	0	0	35
Waltham Forest	0	0	0	0	35
Wandsworth	0	0	0	0	35
Westminster	0	0	0	0	35
North East England	0	0	0	0	35
County Durham	0	0	0	0	35
Darlington	0	0	0	0	35
Gateshead	0	0	0	0	35
Hartlepool	0	0	0	0	35
Middlesbrough	0	0	0	0	35
Newcastle upon Tyne	0	0	0	0	35
North Tyneside	0	0	0	0	35
Northumberland	0	0	0	0	35
Redcar and Cleveland	0	0	0	0	35
South Tyneside	0	0	0	0	35
Stockton-on-Tees	0	0	0	0	35
Sunderland	0	0	0	0	35
North West England	0	0	0	0	35
Blackburn with Darwen	0	0	0	0	35
Blackpool	0	0	0	0	35
Bolton	0	0	0	0	35
Bury	0	0	0	0	35
Cheshire East	0	0	0	0	35
Cheshire West and Chester	0	0	0	0	35
Cumbria	0	0	0	0	35
Halton	0	0	0	0	35
Knowsley	0	0	0	0	35
Lancashire	0	0	0	0	35
Liverpool	0	0	0	0	35
Manchester	0	0	0	0	35
Oldham	0	0	0	0	35
Rochdale	0	0	0	0	35
Salford	0	0	0	0	35
Sefton	0	0	0	0	35
St Helens	0	0	0	0	35
Stockport	0	0	0	0	35
Tameside	0	0	0	0	35
Trafford	0	0	0	0	35
Warrington	0	0	0	0	35
Wigan	0	0	0	0	35
Wirral	0	0	0	0	35

Appendix Table 7. Number of all-cause mortality data sources by type and location, 1950–2016					
Geography	SBH	CBH	HH	SIBS	VR/SRS/DSP
South East England	0	0	0	0	35
Bracknell Forest	0	0	0	0	35
Brighton and Hove	0	0	0	0	35
Buckinghamshire	0	0	0	0	35
East Sussex	0	0	0	0	35
Hampshire	0	0	0	0	35
Isle of Wight	0	0	0	0	35
Kent	0	0	0	0	35
Medway	0	0	0	0	35
Milton Keynes	0	0	0	0	35
Oxfordshire	0	0	0	0	35
Portsmouth	0	0	0	0	35
Reading	0	0	0	0	35
Slough	0	0	0	0	35
Southampton	0	0	0	0	35
Surrey	0	0	0	0	35
West Berkshire	0	0	0	0	35
West Sussex	0	0	0	0	35
Windsor and Maidenhead	0	0	0	0	35
Wokingham	0	0	0	0	35
South West England	0	0	0	0	35
Bath and North East Somerset	0	0	0	0	35
Bournemouth	0	0	0	0	35
Bristol, City of	0	0	0	0	35
Cornwall	0	0	0	0	35
Devon	0	0	0	0	35
Dorset	0	0	0	0	35
Gloucestershire	0	0	0	0	35
North Somerset	0	0	0	0	35
Plymouth	0	0	0	0	35
Poole	0	0	0	0	35
Somerset	0	0	0	0	35
South Gloucestershire	0	0	0	0	35
Swindon	0	0	0	0	35
Torbay	0	0	0	0	35
Wiltshire	0	0	0	0	35
West Midlands	0	0	0	0	35
Birmingham	0	0	0	0	35
Coventry	0	0	0	0	35
Dudley	0	0	0	0	35
Herefordshire, County of	0	0	0	0	35
Sandwell	0	0	0	0	35
Shropshire	0	0	0	0	35
Solihull	0	0	0	0	35
Staffordshire	0	0	0	0	35
Stoke-on-Trent	0	0	0	0	35
Telford and Wrekin	0	0	0	0	35
Walsall	0	0	0	0	35
Warwickshire	0	0	0	0	35

Appendix Table 7. Number of all-cause mortality data sources by type and location, 1950–2016					
Geography	SBH	CBH	HH	SIBS	VR/SRS/DSP
Wolverhampton	0	0	0	0	35
Worcestershire	0	0	0	0	35
Yorkshire and the Humber	0	0	0	0	35
Barnsley	0	0	0	0	35
Bradford	0	0	0	0	35
Calderdale	0	0	0	0	35
Doncaster	0	0	0	0	35
East Riding of Yorkshire	0	0	0	0	35
Kingston upon Hull, City of	0	0	0	0	35
Kirklees	0	0	0	0	35
Leeds	0	0	0	0	35
North East Lincolnshire	0	0	0	0	35
North Lincolnshire	0	0	0	0	35
North Yorkshire	0	0	0	0	35
Rotherham	0	0	0	0	35
Sheffield	0	0	0	0	35
Wakefield	0	0	0	0	35
York	0	0	0	0	35
Northern Ireland	0	0	0	0	64
Scotland	0	0	0	0	65
Wales	0	0	0	0	33
Argentina	3	0	0	0	59
Chile	4	0	0	0	65
Uruguay	5	0	0	0	64
Canada	0	0	0	0	67
Greenland	0	0	0	0	57
United States	0	0	0	0	66
Alabama	0	0	0	0	47
Alaska	0	0	0	0	47
Arizona	0	0	0	0	47
Arkansas	0	0	0	0	47
California	0	0	0	0	47
Colorado	0	0	0	0	47
Connecticut	0	0	0	0	47
Delaware	0	0	0	0	47
District of Columbia	0	0	0	0	47
Florida	0	0	0	0	47
Georgia	0	0	0	0	47
Hawaii	0	0	0	0	47
Idaho	0	0	0	0	47
Illinois	0	0	0	0	47
Indiana	0	0	0	0	47
Iowa	0	0	0	0	47
Kansas	0	0	0	0	47
Kentucky	0	0	0	0	47
Louisiana	0	0	0	0	47
Maine	0	0	0	0	47
Maryland	0	0	0	0	47
Massachusetts	0	0	0	0	47

Appendix Table 7. Number of all-cause mortality data sources by type and location, 1950–2016					
**Geography**	**SBH**	**CBH**	**HH**	**SIBS**	**VR/SRS/DSP**
Michigan	0	0	0	0	47
Minnesota	0	0	0	0	47
Mississippi	0	0	0	0	47
Missouri	0	0	0	0	47
Montana	0	0	0	0	47
Nebraska	0	0	0	0	47
Nevada	0	0	0	0	47
New Hampshire	0	0	0	0	47
New Jersey	0	0	0	0	47
New Mexico	0	0	0	0	47
New York	0	0	0	0	47
North Carolina	0	0	0	0	47
North Dakota	0	0	0	0	47
Ohio	0	0	0	0	47
Oklahoma	0	0	0	0	47
Oregon	0	0	0	0	47
Pennsylvania	0	0	0	0	47
Rhode Island	0	0	0	0	47
South Carolina	0	0	0	0	47
South Dakota	0	0	0	0	47
Tennessee	0	0	0	0	47
Texas	0	0	0	0	47
Utah	0	0	0	0	47
Vermont	0	0	0	0	47
Virginia	0	0	0	0	47
Washington	0	0	0	0	47
West Virginia	0	0	0	0	47
Wisconsin	0	0	0	0	47
Wyoming	0	0	0	0	47
Antigua and Barbuda	0	0	0	0	56
The Bahamas	0	0	0	0	47
Barbados	0	0	0	0	60
Belize	6	2	0	0	64
Bermuda	1	0	0	0	66
Cuba	1	0	0	0	56
Dominica	0	0	0	0	63
Dominican Republic	14	10	0	2	61
Grenada	0	0	0	0	54
Guyana	7	5	0	0	44
Haiti	8	7	0	2	0
Jamaica	5	2	1	0	45
Puerto Rico	1	1	0	0	65
Saint Lucia	0	0	0	0	56
Saint Vincent and the Grenadines	0	0	0	0	49
Suriname	2	0	0	0	52
Trinidad and Tobago	4	2	0	0	61
Virgin Islands, U.S.	0	0	0	0	49
Bolivia	12	5	0	3	16
Ecuador	17	6	0	2	61

Appendix Table 7. Number of all-cause mortality data sources by type and location, 1950–2016					
Geography	SBH	CBH	HH	SIBS	VR/SRS/DSP
Peru	19	13	0	5	62
Colombia	11	7	0	0	60
Costa Rica	7	3	0	0	64
El Salvador	10	6	2	3	64
Guatemala	8	6	0	1	63
Honduras	10	6	1	1	37
Mexico	15	3	0	0	66
Aguascalientes	14	1	0	0	37
Baja California	14	1	0	0	37
Baja California Sur	14	1	0	0	37
Campeche	14	1	0	0	37
Chiapas	14	1	0	0	36
Chihuahua	14	1	0	0	37
Coahuila	14	1	0	0	37
Colima	14	1	0	0	37
Distrito Federal	14	1	0	0	37
Durango	14	1	0	0	37
Guanajuato	14	1	0	0	37
Guerrero	14	1	0	0	37
Hidalgo	13	1	0	0	37
Jalisco	14	1	0	0	37
Mexico	14	1	0	0	31
Michoacan de Ocampo	14	1	0	0	37
Morelos	14	1	0	0	37
Nayarit	14	1	0	0	37
Nuevo Leon	14	1	0	0	37
Oaxaca	14	1	0	0	37
Puebla	14	1	0	0	37
Queretaro	14	1	0	0	33
Quintana Roo	14	1	0	0	37
San Luis Potosi	14	1	0	0	37
Sinaloa	14	1	0	0	37
Sonora	14	1	0	0	32
Tabasco	14	1	0	0	37
Tamaulipas	14	1	0	0	37
Tlaxcala	14	1	0	0	31
Veracruz de Ignacio de la Llave	14	1	0	0	37
Yucatan	14	1	0	0	37
Zacatecas	14	1	0	0	36
Nicaragua	12	5	0	1	50
Panama	8	1	0	0	64
Venezuela	4	1	0	0	63
Brazil	25	1	0	1	42
Acre	11	1	0	0	37
Alagoas	24	2	0	0	37
Amapa	11	1	0	0	37
Amazonas	12	1	0	0	37
Bahia	24	2	0	0	37
Ceara	24	2	0	0	30

Appendix Table 7. Number of all-cause mortality data sources by type and location, 1950–2016					
**Geography**	**SBH**	**CBH**	**HH**	**SIBS**	**VR/SRS/DSP**
Distrito Federal	24	1	0	0	37
Espirito Santo	23	1	0	0	37
Goias	24	1	0	0	31
Maranhao	23	2	0	0	31
Mato Grosso	24	1	0	0	37
Mato Grosso do Sul	22	1	0	0	36
Minas Gerais	24	1	0	0	37
Para	12	1	0	0	36
Paraiba	24	2	0	0	36
Parana	24	1	0	0	37
Pernambuco	24	2	0	0	37
Piaui	23	2	0	0	34
Rio de Janeiro	24	1	0	0	37
Rio Grande do Norte	24	2	0	0	36
Rio Grande do Sul	24	1	0	0	37
Rondonia	11	1	0	0	37
Roraima	11	1	0	0	37
Santa Catarina	23	1	0	0	37
Sao Paulo	23	1	0	0	37
Sergipe	24	2	0	0	37
Tocantins	9	1	0	0	25
Paraguay	10	5	0	1	62
Afghanistan	6	0	1	0	0
Algeria	5	4	0	0	18
Bahrain	4	2	0	0	32
Egypt	13	26	0	0	63
Iran	2	5	0	0	21
Iraq	7	6	2	2	15
Jordan	11	6	0	1	34
Kuwait	3	1	0	0	49
Lebanon	4	2	1	1	7
Libya	4	1	0	0	12
Morocco	9	7	0	2	0
Palestine	6	7	0	0	13
Oman	4	3	0	0	10
Qatar	1	2	0	0	32
Saudi Arabia	2	1	1	0	14
'Asir	0	0	0	0	14
Bahah	0	0	0	0	11
Eastern Province	0	0	0	0	11
Ha'il	0	0	0	0	14
Jawf	0	0	0	0	11
Jizan	0	0	0	0	14
Madinah	0	0	0	0	14
Makkah	0	0	0	0	11
Najran	0	0	0	0	14
Northern Borders	0	0	0	0	11
Qassim	0	0	0	0	13
Riyadh	0	0	0	0	10

Appendix Table 7. Number of all-cause mortality data sources by type and location, 1950–2016					
**Geography**	**SBH**	**CBH**	**HH**	**SIBS**	**VR/SRS/DSP**
Tabuk	0	0	0	0	13
Sudan	8	8	0	1	0
Syria	6	3	1	0	23
Tunisia	7	5	0	0	20
Turkey	14	6	0	0	26
United Arab Emirates	2	1	2	0	11
Yemen	6	7	0	0	0
Bangladesh	13	12	3	3	14
Bhutan	2	1	0	1	0
India	14	5	0	0	43
Andhra Pradesh	3	2	0	0	2
Andhra Pradesh, Rural	10	5	0	0	2
Andhra Pradesh, Urban	10	5	0	0	2
Arunachal Pradesh	9	4	0	0	0
Arunachal Pradesh, Rural	10	4	0	0	0
Arunachal Pradesh, Urban	11	4	0	0	29
Assam	14	5	0	0	21
Assam, Rural	14	5	0	0	20
Assam, Urban	14	5	0	0	32
Bihar	15	5	0	0	12
Bihar, Rural	15	5	0	0	11
Bihar, Urban	14	5	0	0	23
Chhattisgarh	11	3	0	0	8
Chhattisgarh, Rural	10	3	0	0	7
Chhattisgarh, Urban	10	3	0	0	20
Delhi	9	5	0	0	12
Delhi, Rural	10	3	0	0	11
Delhi, Urban	10	5	0	0	43
Goa	8	5	0	0	0
Goa, Rural	9	4	0	0	0
Goa, Urban	9	3	0	0	0
Gujarat	10	5	0	0	21
Gujarat, Rural	9	5	0	0	20
Gujarat, Urban	9	5	0	0	31
Haryana	11	5	0	0	21
Haryana, Rural	11	5	0	0	20
Haryana, Urban	11	5	0	0	49
Himachal Pradesh	11	5	0	0	21
Himachal Pradesh, Rural	11	5	0	0	20
Himachal Pradesh, Urban	11	4	0	0	32
Jammu and Kashmir	10	5	0	0	2
Jammu and Kashmir, Rural	10	5	0	0	2
Jammu and Kashmir, Urban	9	5	0	0	2
Jharkhand	11	3	0	0	12
Jharkhand, Rural	10	3	0	0	11
Jharkhand, Urban	10	3	0	0	11
Karnataka	10	5	0	0	21
Karnataka, Rural	10	5	0	0	20
Karnataka, Urban	10	5	0	0	20

Appendix Table 7. Number of all-cause mortality data sources by type and location, 1950–2016					
**Geography**	**SBH**	**CBH**	**HH**	**SIBS**	**VR/SRS/DSP**
Kerala	10	5	0	0	21
Kerala, Rural	10	5	0	0	20
Kerala, Urban	10	5	0	0	49
Madhya Pradesh	15	5	0	0	12
Madhya Pradesh, Rural	15	5	0	0	11
Madhya Pradesh, Urban	15	5	0	0	11
Maharashtra	10	5	0	0	21
Maharashtra, Rural	10	5	0	0	20
Maharashtra, Urban	10	5	0	0	49
Manipur	10	5	0	0	0
Manipur, Rural	10	5	0	0	0
Manipur, Urban	10	4	0	0	29
Meghalaya	10	5	0	0	0
Meghalaya, Rural	10	5	0	0	0
Meghalaya, Urban	10	5	0	0	12
Mizoram	11	5	0	0	0
Mizoram, Rural	11	4	0	0	0
Mizoram, Urban	11	5	0	0	0
Nagaland	8	4	0	0	0
Nagaland, Rural	10	4	0	0	0
Nagaland, Urban	10	4	0	0	29
Orissa	15	5	0	0	21
Orissa, Rural	15	5	0	0	20
Orissa, Urban	15	5	0	0	52
Punjab	10	5	0	0	21
Punjab, Rural	10	5	0	0	20
Punjab, Urban	10	5	0	0	20
Rajasthan	14	5	0	0	21
Rajasthan, Rural	14	5	0	0	20
Rajasthan, Urban	14	5	0	0	20
Sikkim	9	3	0	0	0
Sikkim, Rural	9	3	0	0	0
Sikkim, Urban	9	3	0	0	11
Tamil Nadu	10	5	0	0	21
Tamil Nadu, Rural	10	5	0	0	20
Tamil Nadu, Urban	10	5	0	0	38
Telangana	3	2	0	0	2
Telangana, Rural	2	2	0	0	2
Telangana, Urban	2	2	0	0	2
Tripura	10	5	0	0	0
Tripura, Rural	10	5	0	0	0
Tripura, Urban	9	5	0	0	0
Uttar Pradesh	15	5	0	0	16
Uttar Pradesh, Rural	15	5	0	0	15
Uttar Pradesh, Urban	15	5	0	0	44
Uttarakhand	10	3	0	0	2
Uttarakhand, Rural	9	3	0	0	2
Uttarakhand, Urban	9	3	0	0	14
West Bengal	11	5	0	0	21

Appendix Table 7. Number of all-cause mortality data sources by type and location, 1950–2016					
Geography	SBH	CBH	HH	SIBS	VR/SRS/DSP
West Bengal, Rural	10	5	0	0	20
West Bengal, Urban	11	5	0	0	20
The Six Minor Territories	4	1	0	0	0
The Six Minor Territories, Rural	3	0	0	0	0
The Six Minor Territories, Urban	3	1	0	0	29
Nepal	11	7	1	1	0
Pakistan	13	8	7	0	17
Angola	3	2	0	0	0
Central African Republic	5	1	1	1	0
Congo	4	3	0	2	0
Democratic Republic of the Congo	5	2	0	2	0
Equatorial Guinea	1	1	0	0	0
Gabon	2	2	0	2	0
Burundi	8	2	1	1	0
Comoros	4	2	1	1	0
Djibouti	3	1	1	0	0
Eritrea	3	2	0	1	0
Ethiopia	6	4	1	3	0
Kenya	17	8	0	4	0
Central	6	0	0	1	0
Kiambu	12	6	0	1	0
Kirinyaga	11	6	0	0	0
Murang'a	11	6	0	1	0
Nyandarua	11	6	0	1	0
Nyeri	11	6	0	1	0
Coast	6	0	0	1	0
Kilifi	12	6	0	1	0
Kwale	11	6	0	1	0
Lamu	9	4	0	0	0
Mombasa	11	7	0	0	0
TaitaTaveta	11	6	0	1	0
TanaRiver	9	3	0	1	0
Eastern	6	0	0	1	0
Embu	13	7	0	0	0
Isiolo	10	4	0	0	0
Kitui	14	7	0	1	0
Machakos	14	7	0	1	0
Makueni	8	4	0	1	0
Marsabit	8	4	0	0	0
Meru	11	7	0	1	0
TharakaNithi	7	4	0	1	0
Nairobi	5	0	0	1	0
Nairobi	11	6	0	1	0
North Eastern	3	0	0	1	0
Garissa	9	3	0	0	0
Mandera	9	3	0	1	0
Wajir	9	3	0	0	0
Nyanza	6	0	0	1	0
HomaBay	8	4	0	1	0

Appendix Table 7. Number of all-cause mortality data sources by type and location, 1950–2016					
Geography	SBH	CBH	HH	SIBS	VR/SRS/DSP
Kisii	11	7	0	1	0
Kisumu	11	7	0	1	0
Migori	6	4	0	1	0
Nyamira	7	4	0	1	0
Siaya	12	7	0	1	0
Rift Valley	6	0	0	1	0
Baringo	11	5	0	1	0
Bomet	6	3	0	1	0
Elgeyo-Marakwet	11	6	0	1	0
Kajiado	11	5	0	1	0
Kericho	12	6	0	0	0
Laikipia	11	5	0	0	0
Nakuru	11	6	0	1	0
Nandi	12	6	0	1	0
Narok	11	6	0	1	0
Samburu	7	3	0	0	0
TransNzoia	11	6	0	0	0
Turkana	8	3	0	1	0
UasinGishu	11	6	0	1	0
WestPokot	11	5	0	1	0
Western	6	0	0	1	0
Bungoma	12	6	0	1	0
Busia	11	6	0	0	0
Kakamega	11	6	0	0	0
Vihiga	6	3	0	1	0
Madagascar	9	4	1	4	9
Malawi	18	7	8	5	0
Mozambique	8	4	2	3	0
Rwanda	13	8	0	4	0
Somalia	2	1	0	0	0
South Sudan	2	1	0	0	0
Tanzania	13	7	2	4	0
Uganda	12	6	1	4	0
Zambia	11	5	0	5	0
Botswana	6	3	0	0	0
Lesotho	7	5	0	3	0
Namibia	6	4	2	4	0
South Africa	4	1	3	1	26
Eastern Cape	5	1	3	1	18
Free State	5	1	3	1	18
Gauteng	5	1	3	1	18
KwaZulu-Natal	6	1	3	1	18
Limpopo	5	1	3	1	18
Mpumalanga	5	1	3	1	18
North-West	5	1	3	1	18
Northern Cape	5	1	3	1	18
Western Cape	5	0	3	1	18
Swaziland	4	4	2	1	0
Zimbabwe	8	8	3	6	1

Appendix Table 7. Number of all-cause mortality data sources by type and location, 1950–2016					
**Geography**	**SBH**	**CBH**	**HH**	**SIBS**	**VR/SRS/DSP**
Benin	6	6	0	2	0
Burkina Faso	9	7	1	3	0
Cameroon	7	5	1	3	0
Cape Verde	3	2	0	0	8
Chad	4	4	0	3	0
Cote d'Ivoire	6	9	1	3	0
The Gambia	6	2	0	1	0
Ghana	16	10	1	1	0
Guinea	5	4	0	3	0
Guinea-Bissau	4	2	0	1	0
Liberia	6	4	0	2	0
Mali	9	5	4	4	0
Mauritania	7	4	1	2	0
Niger	6	4	0	3	0
Nigeria	13	5	0	2	0
Sao Tome and Principe	6	2	1	2	13
Senegal	11	10	0	2	0
Sierra Leone	6	2	1	2	0
Togo	7	3	0	2	0

Appendix Table 8. Number of all-cause mortality data sources by type and year, 1950-2016					
	SBH	CBH	HH	SIBS	VR/SRS/DSP
1950	0	0	0	0	67
1951	0	0	0	0	68
1952	0	0	0	0	73
1953	0	0	1	0	74
1954	1	0	0	0	75
1955	0	0	0	0	77
1956	2	0	0	0	78
1957	0	0	0	0	78
1958	1	0	1	0	79
1959	0	0	0	0	86
1960	23	0	2	0	88
1961	3	0	1	0	88
1962	0	0	0	0	85
1963	1	0	0	0	87
1964	1	0	0	0	90
1965	1	0	0	0	87
1966	4	0	0	0	84
1967	1	0	1	0	84
1968	1	0	0	0	138
1969	42	0	0	0	138
1970	39	0	1	0	135
1971	12	0	5	0	137
1972	2	0	0	0	136
1973	8	0	1	0	136
1974	6	4	1	0	140
1975	13	4	0	0	141
1976	14	7	1	0	143
1977	8	4	1	0	146
1978	10	8	0	0	143
1979	48	2	2	0	231
1980	112	4	1	0	254
1981	17	2	1	0	419
1982	37	1	6	0	424
1983	5	1	0	0	417
1984	7	1	1	0	426
1985	44	2	1	0	429
1986	32	7	2	0	437
1987	38	15	5	0	439
1988	47	35	3	0	426
1989	54	7	32	1	436
1990	148	7	1	0	441
1991	111	18	5	1	440
1992	162	120	3	7	438
1993	169	45	2	3	434

Appendix Table 8. Number of all-cause mortality data sources by type and year, 1950-2016

	SBH	CBH	HH	SIBS	VR/SRS/DSP
1994	121	12	3	36	438
1995	105	13	4	5	478
1996	110	45	36	7	495
1997	83	15	37	37	503
1998	121	135	47	16	506
1999	236	16	63	3	519
2000	277	21	100	11	524
2001	177	27	37	3	524
2002	69	12	76	29	530
2003	241	58	64	8	521
2004	77	46	88	7	553
2005	190	184	79	9	519
2006	229	22	80	8	556
2007	137	22	67	39	556
2008	201	74	68	6	557
2009	259	14	66	4	553
2010	202	17	97	9	552
2011	245	63	79	7	512
2012	159	27	63	40	532
2013	145	37	31	8	504
2014	110	78	30	50	449
2015	49	96	0	2	331
2016	0	4	0	0	1

Appendix Table 9. Life expectancy at birth in 2015, from World Population Prospects 2017, WHO, US Census Bureau, and GBD 2016								
Country	GBD 2016		WPP 2017		WHO		US Census Bureau	
	Female	Male	Female	Male	Female	Male	Female	Male
Afghanistan	58·9	56·9	64·6	62.0	61·9	59·3	52·3	49·5
Albania	80·5	74·6	80·3	76·1	80·7	75·1	81·0	75·5
Algeria	78·3	76·3	77·1	74·6	77·5	73·8	78·0	75·3
American Samoa	74·3	70·3					78·3	72·2
Andorra	85·8	79·3					85·0	80·6
Angola	64·7	63·3	63·8	58·2	54·0	50·9	56·8	54·5
Antigua and Barbuda	79·9	74·5	78·6	73·7	78·6	74·1	78·5	74·2
Argentina	79·9	73·2	80·2	72·6	79·9	72·7	80·2	73·8
Armenia	79·0	71·8	77·4	71·1	77·7	71·6	78·0	71·1
Australia	84·6	80·5	84·7	80·7	84·8	80·9	84·7	79·7
Austria	83·8	79·0	83·8	78·9	83·9	79·0	84·2	78·8
Azerbaijan	75·4	68·1	74·9	68·8	75·8	69·6	75·5	69·2
Bahrain	77·7	75·9	77·8	75·9	77·9	76·2	81·0	76·5
Bangladesh	74·7	70·1	73·8	70·6	73·1	70·6	75·1	70·8
Barbados	78·8	74·4	78·1	73·3	77·9	73·1	77·6	72·8
Belarus	78·6	68·0	78·1	67·0	78·0	66·5	78·4	66·9
Belgium	83·3	78·4	83·3	78·5	83·5	78·6	83·6	78·3
Belize	74·8	69·1	73·2	67·6	73·1	67·5	70·3	67·0
Benin	65·8	62·0	62·1	59·1	61·1	58·8	62·9	60·1
Bermuda	82·3	75·7					84·4	77·9
Bhutan	75·6	71·9	70·0	69·5	70·1	69·5	70·5	68·6
Bolivia	74·1	72·0	71·2	66·2	73·3	68·2	71·8	66·1
Bosnia and Herzegovina	79·9	74·8	79·2	74·2	79·7	75·0	79·8	73·5
Botswana	68·7	60·6	68·3	62·7	68·1	63·3	52·3	56·0
Brazil	78·9	71·5	78·9	71·6	78·7	71·4	77·3	70·0
Brunei	79·4	74·5	78·8	75·5	79·2	76·3	79·4	74·6
Bulgaria	78·5	71·6	78·1	71·2	78·0	71·1	77·9	71·1
Burkina Faso	61·6	59·0	60·4	59·1	60·5	59·1	57·2	53·1
Burundi	61·2	58·8	59·0	55·1	61·6	57·7	61·8	58·5
Cambodia	71·2	65·4	70·5	66·4	70·7	66·6	66·7	61·7
Cameroon	61·5	57·8	58·8	56·4	58·6	55·9	59·3	56·6
Canada	83·8	79·7	84·1	80·2	84·1	80·2	84·5	79·2
Cape Verde	78·4	68·4	74·5	70·6	75·0	71·3	74·2	69·6
Central African Republic	52·1	47·4	53·2	49·6	54·1	50·9	53·2	50·5
Chad	60·7	57·7	53·7	51·3	54·5	51·7	51·0	48·6
Chile	83·1	77·2	81·8	76·7	83·4	77·4	81·8	75·6
China	79·5	73·1	77·7	74·6	77·6	74·6	77·7	73·4
Colombia	80·9	75·2	77·8	70·7	78·4	71·2	78·8	72·3
Comoros	68·2	66·3	65·1	61·7	65·2	61·9	66·2	61·6
Congo	62·0	63·3	65·5	62·3	66·3	63·2	60·0	57·6
Costa Rica	83·5	78·4	82·1	77·3	82·2	77·1	81·2	75·8
Cote d'Ivoire	61·5	57·0	54·5	51·7	54·4	52·3	59·5	57·2
Croatia	80·5	74·2	80·7	74·1	81·2	74·7	79·0	72·5
Cuba	81·2	76·6	81·6	77·6	81·4	76·9	81·0	76·2
Cyprus	82·8	78·1	82·5	78·2	82·7	78·3	81·5	75·7
Czech Republic	81·8	76·1	81·5	75·6	81·7	75·9	81·6	75·5
Democratic Republic of the Congo	62·0	59·8	60·6	57·7	61·5	58·3	58·5	55·4

Appendix Table 9. Life expectancy at birth in 2015, from World Population Prospects 2017, WHO, US Census Bureau, and GBD 2016

Country	GBD 2016		WPP 2017		WHO		US Census Bureau	
	Female	Male	Female	Male	Female	Male	Female	Male
Denmark	82·7	78·7	82·5	78·6	82·5	78·6	81·8	76·8
Djibouti	68·2	64·0	63·8	60·5	65·3	61·8	65·4	60·3
Dominica	74·6	68·8					79·9	73·8
Dominican Republic	78·4	72·6	76·9	70·6	77·1	70·9	80·3	75·8
Ecuador	80·4	75·5	78·9	73·4	79·0	73·5	79·7	73·6
Egypt	74·7	69·2	73·6	69·1	73·2	68·8	73·9	71·1
El Salvador	78·9	71·2	77·7	68·5	77·9	68·8	77·9	71·1
Equatorial Guinea	66·2	64·2	58·9	56·2	60·0	56·6	65·0	62·8
Eritrea	64·1	62·6	66·8	62·5	67·0	62·4	67·1	62·1
Estonia	81·7	72·8	81·6	72·4	82·0	72·7	81·5	71·6
Ethiopia	66·0	64·3	66·7	63·0	66·8	62·8	63·9	59·1
Federated States of Micronesia	67·3	63·4	70·2	68·0	70·6	68·1	74·8	70·6
Fiji	67·6	63·3	73·3	67·3	73·1	67·0	75·2	69·8
Finland	84·4	78·6	84·0	78·2	83·8	78·3	83·9	77·8
France	85·3	79·1	85·3	79·3	85·4	79·4	85·0	78·7
Gabon	67·7	64·3	67·1	64·1	67·2	64·7	52·5	51·6
Georgia	78·6	68·8	77·4	68·9	78·3	70·3	80·4	71·9
Germany	83·3	78·4	83·2	78·5	83·4	78·7	83·0	78·3
Ghana	66·9	63·9	63·4	61·4	63·9	61·0	68·7	63·8
Greece	83·5	78·4	83·7	78·5	83·6	78·3	83·2	77·8
Greenland	73·0	67·8					74·9	69·4
Grenada	74·2	68·6	76·0	71·1	76·1	71·2	76·9	71·5
Guam	75·9	68·9	81·9	76·9			82·2	75·9
Guatemala	75·8	69·3	76·3	69·9	75·2	68·5	74·1	70·1
Guinea	61·0	59·1	60·0	58·9	59·8	58·2	61·7	58·6
Guinea-Bissau	60·7	55·7	58·7	55·3	60·5	57·2	52·3	48·2
Guyana	70·1	64·0	68·9	64·3	68·5	63·9	71·2	65·1
Haiti	64·2	63·5	65·2	60·8	65·5	61·5	65·9	60·8
Honduras	73·5	71·5	75·9	70·9	77·0	72·3	72·7	69·3
Hungary	79·1	72·2	79·2	72·1	79·1	72·3	79·6	72·0
Iceland	83·9	80·6	84·1	81·1	84·1	81·2	85·2	80·8
India	69·8	66·6	69·8	66·8	69·9	66·9	69·4	67·0
Indonesia	73·3	69·6	71·2	67·0	71·2	67·1	75·2	69·9
Iran	77·9	73·7	76·8	74·5	76·6	74·5	72·8	69·6
Iraq	70·4	64·9	72·0	67·4	71·8	66·2	77·2	72·6
Ireland	83·3	79·0	83·3	79·3	83·4	79·4	83·1	78·4
Israel	84·1	79·9	84·0	80·5	84·3	80·6	84·2	80·4
Italy	84·6	79·9	85·0	80·5	84·8	80·5	84·9	79·5
Jamaica	76·8	73·1	78·2	73·4	78·6	73·9	75·2	71·9
Japan	86·9	80·7	86·8	80·4	86·8	80·5	88·3	81·4
Jordan	78·2	75·3	75·9	72·5	75·9	72·5	75·8	73·0
Kazakhstan	76·1	67·1	74·4	64·7	74·7	65·7	75·5	65·3
Kenya	68·6	64·1	68·7	64·0	65·8	61·1	65·3	62·3
Kiribati	65·3	57·9	69·4	62·9	68·8	63·7	68·4	63·4
Kuwait	79·6	80·0	75·8	73·7	76·0	73·7	79·2	76·5
Kyrgyzstan	74·9	67·3	74·7	66·7	75·1	67·2	74·8	66·2
Laos	69·2	64·4	67·8	64·8	67·2	64·1	66·0	61·9

Appendix Table 9. Life expectancy at birth in 2015, from World Population Prospects 2017, WHO, US Census Bureau, and GBD 2016								
Country	GBD 2016		WPP 2017		WHO		US Census Bureau	
	Female	Male	Female	Male	Female	Male	Female	Male
Latvia	79·7	69·9	79·0	69·3	79·2	69·6	79·1	69·6
Lebanon	81·3	78·8	81·3	77·8	76·5	73·5	78·7	76·2
Lesotho	52·7	46·5	55·8	51·3	55·4	51·7	53·0	52·8
Liberia	64·2	63·5	62·9	61·0	62·9	59·8	60·3	56·9
Libya	77·0	71·4	74·8	69·1	75·6	70·1	78·1	74·5
Lithuania	80·2	69·5	79·7	68·9	79·1	68·1	80·5	69·2
Luxembourg	83·8	80·1	83·8	79·3	84·0	79·8	84·8	79·7
Macedonia	77·1	72·1	77·6	73·5	77·8	73·5	78·8	73·4
Madagascar	63·5	61·1	67·1	64·0	67·0	63·9	67·1	64·1
Malawi	61·8	57·1	64·7	59·7	59·9	56·7	62·7	58·7
Malaysia	77·8	72·9	77·5	73·0	77·3	72·7	77·7	72·0
Maldives	81·1	77·5	78·2	76·1	80·2	76·9	77·8	73·1
Mali	62·3	60·4	58·2	56·8	58·3	58·2	57·3	53·5
Malta	83·7	79·0	82·3	79·0	83·7	79·7	82·7	77·9
Marshall Islands	66·9	62·5					75·1	70·7
Mauritania	69·9	70·0	64·5	61·5	64·6	61·6	65·0	60·4
Mauritius	77·7	71·3	78·1	71·1	77·8	71·4	79·0	71·9
Mexico	78·9	73·5	79·4	74·5	79·5	73·9	78·6	72·9
Moldova	76·0	68·2	75·6	67·1	76·2	67·9	74·5	66·6
Mongolia	72·8	63·6	73·2	65·0	73·2	64·7	73·8	65·0
Montenegro	79·6	74·3	79·2	74·5	78·1	74·1	81·6	75·5
Morocco	76·2	73·3	76·7	74·3	75·4	73·3	79·9	73·6
Mozambique	61·9	56·0	59·7	55·5	59·4	55·7	53·7	52·2
Myanmar	72·9	66·2	68·7	64·0	68·5	64·6	68·8	63·9
Namibia	68·7	59·9	66·1	60·5	68·3	63·1	64·5	61·8
Nepal	70·9	68·4	71·4	68·3	70·8	67·7	70·5	69·0
Netherlands	83·6	79·7	83·5	79·8	83·6	80·0	83·5	79·1
New Zealand	83·4	79·6	83·4	80·0	83·3	80·0	83·2	79·0
Nicaragua	80·9	75·0	78·1	72·1	77·9	71·5	75·3	70·8
Niger	62·4	60·1	60·6	58·6	62·8	60·9	56·4	53·9
Nigeria	65·4	62·7	53·8	52·2	55·6	53·4	54·1	52·0
North Korea	73·5	67·8	74·7	67·7	74·0	67·0	74·2	66·3
Northern Mariana Islands	77·3	73·8					80·7	75·2
Norway	84·1	80·1	84·0	80·0	83·7	79·8	83·8	79·7
Oman	79·7	75·1	79·3	75·1	79·2	75·0	77·2	73·3
Pakistan	68·4	66·1	67·3	65·3	67·5	65·5	69·4	65·5
Palestine	73·4	70·2	75·3	71·4				
Panama	81·7	75·8	80·9	74·9	81·1	74·7	81·4	75·7
Papua New Guinea	61·8	59·2	67·9	63·0	65·4	60·6	69·4	64·8
Paraguay	76·9	71·9	75·2	70·9	76·0	72·2	79·8	74·3
Peru	81·5	77·5	77·4	72·1	78·0	73·1	75·6	71·5
Philippines	73·6	66·5	72·5	65·7	72·0	65·3	72·6	65·5
Poland	81·5	73·9	81·3	73·4	81·3	73·6	81·5	73·5
Portugal	83·9	77·7	83·9	77·9	83·9	78·2	82·6	75·9
Puerto Rico	82·2	74·8	83·5	75·8			82·9	75·6
Qatar	81·8	79·1	79·7	77·2	80·0	77·4	80·7	76·6
Romania	78·7	71·5	78·7	71·8	78·8	71·4	78·6	71·5

Appendix Table 9. Life expectancy at birth in 2015, from World Population Prospects 2017, WHO, US Census Bureau, and GBD 2016

Country	GBD 2016		WPP 2017		WHO		US Census Bureau	
	Female	Male	Female	Male	Female	Male	Female	Male
Russia	76·4	65·2	76·3	65·1	76·3	64·7	76·6	64·7
Rwanda	69·0	65·6	68·4	64·3	71·1	60·9	61·3	58·1
Saint Lucia	79·3	73·0	78·0	72·7	77·9	72·6	80·5	74·9
Saint Vincent and the Grenadines	74·7	68·6	75·3	70·9	75·2	71·3	77·1	73·1
Samoa	73·7	69·7	78·0	71·7	77·5	70·9	76·5	70·6
Sao Tome and Principe	71·8	68·8	68·6	64·3	69·4	65·6	65·9	63·3
Saudi Arabia	78·1	75·4	76·1	73·1	76·0	73·2	77·2	73·0
Senegal	67·5	64·3	68·5	64·6	68·6	64·6	63·4	59·3
Serbia	78·6	72·9	77·9	72·2	78·4	72·9	78·3	72·4
Seychelles	77·2	70·2	78·3	69·2	78·0	69·1	79·2	69·9
Sierra Leone	58·7	56·6	51·8	50·7	50·8	49·3	60·4	55·2
Singapore	86·0	81·2	84·9	80·7	86·1	80·0	87·5	82·1
Slovakia	80·3	73·3	80·1	73·1	80·2	72·9	80·7	73·3
Slovenia	83·7	77·7	83·6	77·8	83·7	77·9	81·9	74·4
Solomon Islands	63·9	61·7	71·9	68·9	70·8	67·9	77·9	72·5
Somalia	57·7	56·8	57·6	54·3	56·6	53·5	54·1	49·9
South Africa	64·8	58·7	65·1	58·2	66·2	59·3	63·9	60·8
South Korea	84·2	77·7	84·9	78·6	85·5	78·8	85·7	79·2
South Sudan	60·0	57·8	57·3	55·3	58·6	56·1	62·3	59·3
Spain	85·6	80·2	85·7	80·1	85·5	80·1	84·8	78·6
Sri Lanka	80·8	73·6	78·5	71·7	78·3	71·6	80·2	73·1
Sudan	69·9	66·1	65·8	62·6	65·9	62·4	65·9	61·6
Suriname	74·2	68·2	74·5	68·1	74·7	68·6	74·5	69·6
Swaziland	60·6	52·1	59·8	53·4	61·1	56·6	50·5	51·6
Sweden	83·9	80·2	84·1	80·5	84·0	80·7	84·0	80·1
Switzerland	85·2	81·0	85·1	81·1	85·3	81·3	84·9	80·2
Syria	73·5	63·4	77·0	65·1	69·9	59·9	77·2	72·3
Taiwan (Province of China)	82·8	76·7	82·7	76·9			83·3	76·9
Tajikistan	74·1	69·1	74·0	68·1	73·6	66·6	70·7	64·3
Tanzania	65·3	61·7	66·6	62·9	63·8	59·9	63·1	60·3
Thailand	80·7	74·5	78·9	71·4	78·0	71·9	77·8	71·2
The Bahamas	76·2	71·1	78·5	72·4	79·1	72·9	74·7	69·8
The Gambia	69·0	65·1	62·3	59·6	62·5	59·8	67·0	62·3
Timor-Leste	73·5	71·6	70·4	66·8	70·1	66·6	69·4	66·2
Togo	64·6	59·6	60·6	59·0	61·1	58·6	67·2	61·9
Tonga	73·2	67·4	75·9	69·9	76·4	70·6	77·6	74·5
Trinidad and Tobago	76·9	69·2	74·2	67·1	74·8	67·9	75·6	69·7
Tunisia	80·3	74·5	77·6	73·5	77·8	73·0	78·1	73·8
Turkey	82·1	75·7	78·7	72·2	78·9	72·6	77·0	72·3
Turkmenistan	73·6	66·2	71·1	64·2	70·5	62·2	72·9	66·8
Uganda	64·1	59·2	61·6	57·3	64·3	60·3	56·4	53·5
Ukraine	76·9	66·9	76·5	66·6	76·1	66·3	76·6	66·8
United Arab Emirates	78·5	74·4	78·6	76·4	78·6	76·4	80·0	74·7
United Kingdom	82·8	78·9	83·2	79·5	83·0	79·4	82·8	78·4
United States	81·2	76·5	81·6	76·9	81·6	76·9	82·0	77·3
Uruguay	81·0	73·3	80·8	73·7	80·4	73·3	80·3	73·9
Uzbekistan	73·2	66·9	73·9	68·4	72·7	66·1	76·8	70·5

Appendix Table 9. Life expectancy at birth in 2015, from World Population Prospects 2017, WHO, US Census Bureau, and GBD 2016

Country	GBD 2016		WPP 2017		WHO		US Census Bureau	
	Female	Male	Female	Male	Female	Male	Female	Male
Vanuatu	65·3	61·9	74·2	69·8	74·0	70·1	74·7	71·5
Venezuela	79·8	71·3	78·6	70·4	78·5	70·0	78·7	72·5
Vietnam	77·9	70·6	80·7	71·3	80·7	71·3	75·9	70·7
Virgin Islands, U.S.	78·6	70·4	81·9	77·2				
Yemen	68·2	66·2	66·2	63·3	67·2	64·3	67·4	63·1
Zambia	61·2	55·0	63·5	58·5	64·7	59·0	53·8	50·5
Zimbabwe	60·9	55·7	61·4	58·0	62·3	59·0	57·6	56·5

Appendix Table 10. Under-5 mortality reference sources by source date and location, 1950-2016			
**Location**	**Source**	**Source years**	**Method**
China (without Hong Kong and Macao)	1992-2013	Maternal and Child Health Surveillance System (MCHS)	HH
Hong Kong Special Administrative Region of China	1950-2013	Vital Registration	VR/SRS/DSP
Macao Special Administrative Region of China	1954-2010	Vital Registration	VR/SRS/DSP
Anhui	2000-2014	Maternal and Child Health Surveillance System (MCHS)	HH
Beijing	2000-2014	Maternal and Child Health Surveillance System (MCHS)	HH
Chongqing	2000-2014	Maternal and Child Health Surveillance System (MCHS)	HH
Fujian	1996-2012	Maternal and Child Mortality Data	HH
Gansu	1996-2002	Disease Surveillance Points (DSP)	VR/SRS/DSP
Guangdong	2000-2014	Maternal and Child Health Surveillance System (MCHS)	HH
Guangxi	2000-2014	Maternal and Child Health Surveillance System (MCHS)	HH
Guizhou	2000-2014	Maternal and Child Health Surveillance System (MCHS)	HH
Hainan	2000-2014	Maternal and Child Health Surveillance System (MCHS)	HH
Hebei	2000-2014	Maternal and Child Health Surveillance System (MCHS)	HH
Heilongjiang	2002-2014	Maternal and Child Health Surveillance System (MCHS)	HH
Henan	1991-2014	Maternal and Child Health Surveillance System (MCHS)	HH
Hubei	2000-2014	Maternal and Child Health Surveillance System (MCHS)	HH
Hunan	1996-2012	Maternal and Child Mortality Data	HH
Inner Mongolia	1996-2012	Maternal and Child Mortality Data	HH
Jiangsu	2000-2014	Maternal and Child Health Surveillance System (MCHS)	HH
Jiangxi	2000-2014	Maternal and Child Health Surveillance System (MCHS)	HH
Jilin	2000-2014	Maternal and Child Health Surveillance System (MCHS)	HH
Liaoning	1996-2012	Maternal and Child Mortality Data	HH
Ningxia	2000-2014	Maternal and Child Health Surveillance System (MCHS)	HH
Qinghai	2000-2014	Maternal and Child Health Surveillance System (MCHS)	HH
Shaanxi	2000-2014	Maternal and Child Health Surveillance System (MCHS)	HH
Shandong	2000-2014	Maternal and Child Health Surveillance System (MCHS)	HH
Shanghai	2000-2014	Maternal and Child Health Surveillance System (MCHS)	HH
Shanxi	2000-2014	Maternal and Child Health Surveillance System (MCHS)	HH
Sichuan	2002-2014	Maternal and Child Health Surveillance System (MCHS)	HH
Tianjin	2000-2014	Maternal and Child Health Surveillance System (MCHS)	HH
Tibet	2000	Census	SBH
Tibet	1996-2012	Maternal and Child Mortality Data	HH
Xinjiang	1990	Census	SBH
Xinjiang	2004-2014	Maternal and Child Health Surveillance System (MCHS)	HH
Yunnan	1996-2012	Maternal and Child Mortality Data	HH
Zhejiang	2000-2014	Maternal and Child Health Surveillance System (MCHS)	HH
Zhejiang	1996-2012	Maternal and Child Mortality Data	HH
Taiwan (Province of China)	1955-2014	Vital Registration	VR/SRS/DSP
Cambodia	2000	Demographic and Health Survey (DHS)	CBH
Cambodia	2005	Demographic and Health Survey (DHS)	CBH
Cambodia	2010	Demographic and Health Survey (DHS)	CBH
Cambodia	2014	Demographic and Health Survey (DHS)	CBH
Indonesia	1987	Demographic and Health Survey (DHS)	CBH
Indonesia	1991	Demographic and Health Survey (DHS)	CBH
Indonesia	1994	Demographic and Health Survey (DHS)	CBH
Indonesia	1997	Demographic and Health Survey (DHS)	CBH
Indonesia	2002	Demographic and Health Survey (DHS)	CBH
Indonesia	2007	Demographic and Health Survey (DHS)	CBH
Indonesia	2012	Demographic and Health Survey (DHS)	CBH
Aceh	1975	Census	CBH
Aceh	1977	Census	CBH
Aceh	1979	Census	CBH
Aceh	1980	Census	SBH
Aceh	1981	Census	CBH
Aceh	1983	Census	CBH
Aceh	1985	Census	CBH
Aceh	1987	Census	CBH
Aceh	1989	Census	CBH
Aceh	1990	Census	SBH
Aceh	1991	Census	CBH
Aceh	1993	Census	CBH
Aceh	1995	Census	CBH
Aceh	1997	Census	CBH
Aceh	1999	Census	CBH
Aceh	2000	Census	SBH
Aceh	2010	Census	SBH
Aceh	1987	Demographic and Health Survey (DHS)	CBH

Appendix Table 10. Under-5 mortality reference sources by source date and location, 1950-2016			
**Location**	**Source**	**Source years**	**Method**
Aceh	1987	Demographic and Health Survey (DHS)	SBH
Aceh	1989	Demographic and Health Survey (DHS)	CBH
Aceh	1991	Demographic and Health Survey (DHS)	CBH
Aceh	1991	Demographic and Health Survey (DHS)	SBH
Aceh	1993	Demographic and Health Survey (DHS)	CBH
Aceh	1994	Demographic and Health Survey (DHS)	SBH
Aceh	1995	Demographic and Health Survey (DHS)	CBH
Aceh	1997	Demographic and Health Survey (DHS)	SBH
Aceh	1997	Demographic and Health Survey (DHS)	CBH
Aceh	1999	Demographic and Health Survey (DHS)	CBH
Aceh	2001	Demographic and Health Survey (DHS)	CBH
Aceh	2003	Demographic and Health Survey (DHS)	CBH
Aceh	2005	Demographic and Health Survey (DHS)	CBH
Aceh	2007	Demographic and Health Survey (DHS)	SBH
Aceh	2007	Demographic and Health Survey (DHS)	CBH
Aceh	2009	Demographic and Health Survey (DHS)	CBH
Aceh	2011	Demographic and Health Survey (DHS)	CBH
Aceh	2012	Demographic and Health Survey (DHS)	SBH
Aceh	1985	Health Survey	SBH
Aceh	1992	Health Survey	SBH
Aceh	1993	Health Survey	SBH
Aceh	1994	Health Survey	SBH
Aceh	1995	Health Survey	SBH
Aceh	1996	Health Survey	SBH
Aceh	1998	Health Survey	SBH
Aceh	1999	Health Survey	SBH
Aceh	2002	Health Survey	SBH
Aceh	2003	Health Survey	SBH
Aceh	2004	Health Survey	SBH
Aceh	2006	Health Survey	SBH
Aceh	2007	Health Survey	SBH
Aceh	2008	Health Survey	SBH
Aceh	2009	Health Survey	SBH
Aceh	2010	Health Survey	SBH
Aceh	2011	Health Survey	SBH
Aceh	2012	Health Survey	SBH
Aceh	2013	Health Survey	SBH
Bali	1957	Census	CBH
Bali	1958	Census	CBH
Bali	1959	Census	CBH
Bali	1960	Census	CBH
Bali	1961	Census	CBH
Bali	1962	Census	CBH
Bali	1963	Census	CBH
Bali	1964	Census	CBH
Bali	1965	Census	CBH
Bali	1966	Census	CBH
Bali	1967	Census	CBH
Bali	1968	Census	CBH
Bali	1969	Census	CBH
Bali	1970	Census	CBH
Bali	1971	Census	CBH
Bali	1972	Census	CBH
Bali	1973	Census	CBH
Bali	1974	Census	CBH
Bali	1975	Census	CBH
Bali	1976	Census	CBH
Bali	1977	Census	CBH
Bali	1978	Census	CBH
Bali	1979	Census	CBH
Bali	1980	Census	SBH
Bali	1980	Census	CBH
Bali	1981	Census	CBH
Bali	1982	Census	CBH
Bali	1983	Census	CBH
Bali	1984	Census	CBH
Bali	1985	Census	CBH

Appendix Table 10. Under-5 mortality reference sources by source date and location, 1950-2016			
Location	Source	Source years	Method
Bali	1986	Census	CBH
Bali	1987	Census	CBH
Bali	1988	Census	CBH
Bali	1989	Census	CBH
Bali	1990	Census	CBH
Bali	1990	Census	SBH
Bali	1991	Census	CBH
Bali	1992	Census	CBH
Bali	1993	Census	CBH
Bali	1994	Census	CBH
Bali	1995	Census	CBH
Bali	1996	Census	CBH
Bali	1997	Census	CBH
Bali	1998	Census	CBH
Bali	1999	Census	CBH
Bali	2000	Census	SBH
Bali	2000	Census	CBH
Bali	2002	Census	CBH
Bali	2004	Census	CBH
Bali	2010	Census	SBH
Bali	1967	Demographic and Health Survey (DHS)	CBH
Bali	1969	Demographic and Health Survey (DHS)	CBH
Bali	1971	Demographic and Health Survey (DHS)	CBH
Bali	1973	Demographic and Health Survey (DHS)	CBH
Bali	1975	Demographic and Health Survey (DHS)	CBH
Bali	1977	Demographic and Health Survey (DHS)	CBH
Bali	1979	Demographic and Health Survey (DHS)	CBH
Bali	1981	Demographic and Health Survey (DHS)	CBH
Bali	1983	Demographic and Health Survey (DHS)	CBH
Bali	1985	Demographic and Health Survey (DHS)	CBH
Bali	1987	Demographic and Health Survey (DHS)	CBH
Bali	1987	Demographic and Health Survey (DHS)	SBH
Bali	1989	Demographic and Health Survey (DHS)	CBH
Bali	1991	Demographic and Health Survey (DHS)	SBH
Bali	1991	Demographic and Health Survey (DHS)	CBH
Bali	1993	Demographic and Health Survey (DHS)	CBH
Bali	1994	Demographic and Health Survey (DHS)	SBH
Bali	1995	Demographic and Health Survey (DHS)	CBH
Bali	1997	Demographic and Health Survey (DHS)	SBH
Bali	1997	Demographic and Health Survey (DHS)	CBH
Bali	1999	Demographic and Health Survey (DHS)	CBH
Bali	2001	Demographic and Health Survey (DHS)	CBH
Bali	2003	Demographic and Health Survey (DHS)	SBH
Bali	2003	Demographic and Health Survey (DHS)	CBH
Bali	2005	Demographic and Health Survey (DHS)	CBH
Bali	2007	Demographic and Health Survey (DHS)	SBH
Bali	2007	Demographic and Health Survey (DHS)	CBH
Bali	2009	Demographic and Health Survey (DHS)	CBH
Bali	2011	Demographic and Health Survey (DHS)	CBH
Bali	2012	Demographic and Health Survey (DHS)	SBH
Bali	1982	Health Survey	CBH
Bali	1984	Health Survey	CBH
Bali	1985	Health Survey	SBH
Bali	1986	Health Survey	CBH
Bali	1988	Health Survey	CBH
Bali	1990	Health Survey	CBH
Bali	1992	Health Survey	SBH
Bali	1992	Health Survey	CBH
Bali	1993	Health Survey	SBH
Bali	1994	Health Survey	SBH
Bali	1994	Health Survey	CBH
Bali	1995	Health Survey	SBH
Bali	1996	Health Survey	CBH
Bali	1996	Health Survey	SBH
Bali	1997	Health Survey	SBH
Bali	1998	Health Survey	CBH
Bali	1998	Health Survey	SBH

Appendix Table 10. Under-5 mortality reference sources by source date and location, 1950-2016			
**Location**	**Source**	**Source years**	**Method**
Bali	1999	Health Survey	SBH
Bali	2000	Health Survey	CBH
Bali	2000	Health Survey	SBH
Bali	2001	Health Survey	SBH
Bali	2002	Health Survey	SBH
Bali	2003	Health Survey	SBH
Bali	2004	Health Survey	SBH
Bali	2005	Health Survey	SBH
Bali	2006	Health Survey	SBH
Bali	2007	Health Survey	SBH
Bali	2008	Health Survey	SBH
Bali	2009	Health Survey	SBH
Bali	2010	Health Survey	SBH
Bali	2011	Health Survey	SBH
Bali	2012	Health Survey	SBH
Bali	2013	Health Survey	SBH
Bangka Belitung	1964	Census	CBH
Bangka Belitung	1966	Census	CBH
Bangka Belitung	1967	Census	CBH
Bangka Belitung	1968	Census	CBH
Bangka Belitung	1969	Census	CBH
Bangka Belitung	1970	Census	CBH
Bangka Belitung	1971	Census	CBH
Bangka Belitung	1972	Census	CBH
Bangka Belitung	1973	Census	CBH
Bangka Belitung	1974	Census	CBH
Bangka Belitung	1975	Census	CBH
Bangka Belitung	1976	Census	CBH
Bangka Belitung	1977	Census	CBH
Bangka Belitung	1978	Census	CBH
Bangka Belitung	1979	Census	CBH
Bangka Belitung	1980	Census	CBH
Bangka Belitung	1980	Census	SBH
Bangka Belitung	1981	Census	CBH
Bangka Belitung	1982	Census	CBH
Bangka Belitung	1983	Census	CBH
Bangka Belitung	1984	Census	CBH
Bangka Belitung	1985	Census	CBH
Bangka Belitung	1986	Census	CBH
Bangka Belitung	1987	Census	CBH
Bangka Belitung	1988	Census	CBH
Bangka Belitung	1989	Census	CBH
Bangka Belitung	1990	Census	CBH
Bangka Belitung	1990	Census	SBH
Bangka Belitung	1991	Census	CBH
Bangka Belitung	1992	Census	CBH
Bangka Belitung	1993	Census	CBH
Bangka Belitung	1994	Census	CBH
Bangka Belitung	1995	Census	CBH
Bangka Belitung	1996	Census	CBH
Bangka Belitung	1997	Census	CBH
Bangka Belitung	1998	Census	CBH
Bangka Belitung	1999	Census	CBH
Bangka Belitung	2000	Census	CBH
Bangka Belitung	2000	Census	SBH
Bangka Belitung	2002	Census	CBH
Bangka Belitung	2004	Census	CBH
Bangka Belitung	2010	Census	SBH
Bangka Belitung	1979	Demographic and Health Survey (DHS)	CBH
Bangka Belitung	1981	Demographic and Health Survey (DHS)	CBH
Bangka Belitung	1983	Demographic and Health Survey (DHS)	CBH
Bangka Belitung	1985	Demographic and Health Survey (DHS)	CBH
Bangka Belitung	1987	Demographic and Health Survey (DHS)	CBH
Bangka Belitung	1989	Demographic and Health Survey (DHS)	CBH
Bangka Belitung	1991	Demographic and Health Survey (DHS)	CBH
Bangka Belitung	1993	Demographic and Health Survey (DHS)	CBH
Bangka Belitung	1995	Demographic and Health Survey (DHS)	CBH

Appendix Table 10. Under-5 mortality reference sources by source date and location, 1950-2016			
**Location**	**Source**	**Source years**	**Method**
Bangka Belitung	1997	Demographic and Health Survey (DHS)	CBH
Bangka Belitung	1999	Demographic and Health Survey (DHS)	CBH
Bangka Belitung	2001	Demographic and Health Survey (DHS)	CBH
Bangka Belitung	2003	Demographic and Health Survey (DHS)	SBH
Bangka Belitung	2003	Demographic and Health Survey (DHS)	CBH
Bangka Belitung	2005	Demographic and Health Survey (DHS)	CBH
Bangka Belitung	2007	Demographic and Health Survey (DHS)	SBH
Bangka Belitung	2007	Demographic and Health Survey (DHS)	CBH
Bangka Belitung	2009	Demographic and Health Survey (DHS)	CBH
Bangka Belitung	2011	Demographic and Health Survey (DHS)	CBH
Bangka Belitung	2012	Demographic and Health Survey (DHS)	SBH
Bangka Belitung	1984	Health Survey	CBH
Bangka Belitung	1985	Health Survey	SBH
Bangka Belitung	1986	Health Survey	CBH
Bangka Belitung	1988	Health Survey	CBH
Bangka Belitung	1990	Health Survey	CBH
Bangka Belitung	1992	Health Survey	CBH
Bangka Belitung	1994	Health Survey	CBH
Bangka Belitung	1995	Health Survey	SBH
Bangka Belitung	1996	Health Survey	CBH
Bangka Belitung	1998	Health Survey	CBH
Bangka Belitung	2000	Health Survey	CBH
Bangka Belitung	2000	Health Survey	SBH
Bangka Belitung	2001	Health Survey	SBH
Bangka Belitung	2002	Health Survey	SBH
Bangka Belitung	2003	Health Survey	SBH
Bangka Belitung	2004	Health Survey	SBH
Bangka Belitung	2005	Health Survey	SBH
Bangka Belitung	2006	Health Survey	SBH
Bangka Belitung	2007	Health Survey	SBH
Bangka Belitung	2008	Health Survey	SBH
Bangka Belitung	2009	Health Survey	SBH
Bangka Belitung	2010	Health Survey	SBH
Bangka Belitung	2011	Health Survey	SBH
Bangka Belitung	2012	Health Survey	SBH
Bangka Belitung	2013	Health Survey	SBH
Banten	1964	Census	CBH
Banten	1965	Census	CBH
Banten	1966	Census	CBH
Banten	1967	Census	CBH
Banten	1968	Census	CBH
Banten	1969	Census	CBH
Banten	1970	Census	CBH
Banten	1971	Census	CBH
Banten	1972	Census	CBH
Banten	1973	Census	CBH
Banten	1974	Census	CBH
Banten	1975	Census	CBH
Banten	1976	Census	CBH
Banten	1977	Census	CBH
Banten	1978	Census	CBH
Banten	1979	Census	CBH
Banten	1980	Census	SBH
Banten	1980	Census	CBH
Banten	1981	Census	CBH
Banten	1982	Census	CBH
Banten	1983	Census	CBH
Banten	1984	Census	CBH
Banten	1985	Census	CBH
Banten	1986	Census	CBH
Banten	1987	Census	CBH
Banten	1988	Census	CBH
Banten	1989	Census	CBH
Banten	1990	Census	SBH
Banten	1990	Census	CBH
Banten	1991	Census	CBH
Banten	1992	Census	CBH

Appendix Table 10. Under-5 mortality reference sources by source date and location, 1950-2016			
**Location**	**Source**	**Source years**	**Method**
Banten	1993	Census	CBH
Banten	1994	Census	CBH
Banten	1995	Census	CBH
Banten	1996	Census	CBH
Banten	1997	Census	CBH
Banten	1998	Census	CBH
Banten	1999	Census	CBH
Banten	2000	Census	CBH
Banten	2000	Census	SBH
Banten	2002	Census	CBH
Banten	2004	Census	CBH
Banten	2010	Census	SBH
Banten	1973	Demographic and Health Survey (DHS)	CBH
Banten	1975	Demographic and Health Survey (DHS)	CBH
Banten	1977	Demographic and Health Survey (DHS)	CBH
Banten	1979	Demographic and Health Survey (DHS)	CBH
Banten	1981	Demographic and Health Survey (DHS)	CBH
Banten	1983	Demographic and Health Survey (DHS)	CBH
Banten	1985	Demographic and Health Survey (DHS)	CBH
Banten	1987	Demographic and Health Survey (DHS)	CBH
Banten	1989	Demographic and Health Survey (DHS)	CBH
Banten	1991	Demographic and Health Survey (DHS)	CBH
Banten	1993	Demographic and Health Survey (DHS)	CBH
Banten	1995	Demographic and Health Survey (DHS)	CBH
Banten	1997	Demographic and Health Survey (DHS)	CBH
Banten	1999	Demographic and Health Survey (DHS)	CBH
Banten	2001	Demographic and Health Survey (DHS)	CBH
Banten	2003	Demographic and Health Survey (DHS)	SBH
Banten	2003	Demographic and Health Survey (DHS)	CBH
Banten	2005	Demographic and Health Survey (DHS)	CBH
Banten	2007	Demographic and Health Survey (DHS)	CBH
Banten	2007	Demographic and Health Survey (DHS)	SBH
Banten	2009	Demographic and Health Survey (DHS)	CBH
Banten	2011	Demographic and Health Survey (DHS)	CBH
Banten	2012	Demographic and Health Survey (DHS)	SBH
Banten	1980	Health Survey	CBH
Banten	1982	Health Survey	CBH
Banten	1984	Health Survey	CBH
Banten	1985	Health Survey	SBH
Banten	1986	Health Survey	CBH
Banten	1988	Health Survey	CBH
Banten	1990	Health Survey	CBH
Banten	1992	Health Survey	CBH
Banten	1992	Health Survey	SBH
Banten	1993	Health Survey	SBH
Banten	1994	Health Survey	SBH
Banten	1994	Health Survey	CBH
Banten	1995	Health Survey	SBH
Banten	1996	Health Survey	CBH
Banten	1996	Health Survey	SBH
Banten	1998	Health Survey	CBH
Banten	1998	Health Survey	SBH
Banten	1999	Health Survey	SBH
Banten	2000	Health Survey	SBH
Banten	2000	Health Survey	CBH
Banten	2001	Health Survey	SBH
Banten	2002	Health Survey	SBH
Banten	2003	Health Survey	SBH
Banten	2004	Health Survey	SBH
Banten	2005	Health Survey	SBH
Banten	2006	Health Survey	SBH
Banten	2007	Health Survey	SBH
Banten	2008	Health Survey	SBH
Banten	2009	Health Survey	SBH
Banten	2010	Health Survey	SBH
Banten	2011	Health Survey	SBH
Banten	2012	Health Survey	SBH

Appendix Table 10. Under-5 mortality reference sources by source date and location, 1950-2016			
**Location**	**Source**	**Source years**	**Method**
Banten	2013	Health Survey	SBH
Bengkulu	1962	Census	CBH
Bengkulu	1964	Census	CBH
Bengkulu	1966	Census	CBH
Bengkulu	1967	Census	CBH
Bengkulu	1968	Census	CBH
Bengkulu	1969	Census	CBH
Bengkulu	1970	Census	CBH
Bengkulu	1971	Census	CBH
Bengkulu	1972	Census	CBH
Bengkulu	1973	Census	CBH
Bengkulu	1974	Census	CBH
Bengkulu	1975	Census	CBH
Bengkulu	1976	Census	CBH
Bengkulu	1977	Census	CBH
Bengkulu	1978	Census	CBH
Bengkulu	1979	Census	CBH
Bengkulu	1980	Census	CBH
Bengkulu	1980	Census	SBH
Bengkulu	1981	Census	CBH
Bengkulu	1982	Census	CBH
Bengkulu	1983	Census	CBH
Bengkulu	1984	Census	CBH
Bengkulu	1985	Census	CBH
Bengkulu	1986	Census	CBH
Bengkulu	1987	Census	CBH
Bengkulu	1988	Census	CBH
Bengkulu	1989	Census	CBH
Bengkulu	1990	Census	SBH
Bengkulu	1990	Census	CBH
Bengkulu	1991	Census	CBH
Bengkulu	1992	Census	CBH
Bengkulu	1993	Census	CBH
Bengkulu	1994	Census	CBH
Bengkulu	1995	Census	CBH
Bengkulu	1996	Census	CBH
Bengkulu	1997	Census	CBH
Bengkulu	1998	Census	CBH
Bengkulu	1999	Census	CBH
Bengkulu	2000	Census	CBH
Bengkulu	2000	Census	SBH
Bengkulu	2002	Census	CBH
Bengkulu	2004	Census	CBH
Bengkulu	2010	Census	SBH
Bengkulu	1971	Demographic and Health Survey (DHS)	CBH
Bengkulu	1973	Demographic and Health Survey (DHS)	CBH
Bengkulu	1975	Demographic and Health Survey (DHS)	CBH
Bengkulu	1977	Demographic and Health Survey (DHS)	CBH
Bengkulu	1979	Demographic and Health Survey (DHS)	CBH
Bengkulu	1981	Demographic and Health Survey (DHS)	CBH
Bengkulu	1983	Demographic and Health Survey (DHS)	CBH
Bengkulu	1985	Demographic and Health Survey (DHS)	CBH
Bengkulu	1987	Demographic and Health Survey (DHS)	SBH
Bengkulu	1987	Demographic and Health Survey (DHS)	CBH
Bengkulu	1989	Demographic and Health Survey (DHS)	CBH
Bengkulu	1991	Demographic and Health Survey (DHS)	CBH
Bengkulu	1991	Demographic and Health Survey (DHS)	SBH
Bengkulu	1993	Demographic and Health Survey (DHS)	CBH
Bengkulu	1994	Demographic and Health Survey (DHS)	SBH
Bengkulu	1995	Demographic and Health Survey (DHS)	CBH
Bengkulu	1997	Demographic and Health Survey (DHS)	SBH
Bengkulu	1997	Demographic and Health Survey (DHS)	CBH
Bengkulu	1999	Demographic and Health Survey (DHS)	CBH
Bengkulu	2001	Demographic and Health Survey (DHS)	CBH
Bengkulu	2003	Demographic and Health Survey (DHS)	SBH
Bengkulu	2003	Demographic and Health Survey (DHS)	CBH
Bengkulu	2005	Demographic and Health Survey (DHS)	CBH

Appendix Table 10. Under-5 mortality reference sources by source date and location, 1950-2016			
**Location**	**Source**	**Source years**	**Method**
Bengkulu	2007	Demographic and Health Survey (DHS)	CBH
Bengkulu	2007	Demographic and Health Survey (DHS)	SBH
Bengkulu	2009	Demographic and Health Survey (DHS)	CBH
Bengkulu	2011	Demographic and Health Survey (DHS)	CBH
Bengkulu	2012	Demographic and Health Survey (DHS)	SBH
Bengkulu	1985	Health Survey	SBH
Bengkulu	1992	Health Survey	SBH
Bengkulu	1993	Health Survey	SBH
Bengkulu	1994	Health Survey	SBH
Bengkulu	1995	Health Survey	SBH
Bengkulu	1996	Health Survey	SBH
Bengkulu	1998	Health Survey	SBH
Bengkulu	1999	Health Survey	SBH
Bengkulu	2000	Health Survey	SBH
Bengkulu	2001	Health Survey	SBH
Bengkulu	2002	Health Survey	SBH
Bengkulu	2003	Health Survey	SBH
Bengkulu	2004	Health Survey	SBH
Bengkulu	2005	Health Survey	SBH
Bengkulu	2006	Health Survey	SBH
Bengkulu	2007	Health Survey	SBH
Bengkulu	2008	Health Survey	SBH
Bengkulu	2009	Health Survey	SBH
Bengkulu	2010	Health Survey	SBH
Bengkulu	2011	Health Survey	SBH
Bengkulu	2012	Health Survey	SBH
Bengkulu	2013	Health Survey	SBH
Gorontalo	1966	Census	CBH
Gorontalo	1967	Census	CBH
Gorontalo	1968	Census	CBH
Gorontalo	1969	Census	CBH
Gorontalo	1970	Census	CBH
Gorontalo	1971	Census	CBH
Gorontalo	1972	Census	CBH
Gorontalo	1973	Census	CBH
Gorontalo	1974	Census	CBH
Gorontalo	1975	Census	CBH
Gorontalo	1976	Census	CBH
Gorontalo	1977	Census	CBH
Gorontalo	1978	Census	CBH
Gorontalo	1979	Census	CBH
Gorontalo	1980	Census	SBH
Gorontalo	1980	Census	CBH
Gorontalo	1981	Census	CBH
Gorontalo	1982	Census	CBH
Gorontalo	1983	Census	CBH
Gorontalo	1984	Census	CBH
Gorontalo	1985	Census	CBH
Gorontalo	1986	Census	CBH
Gorontalo	1987	Census	CBH
Gorontalo	1988	Census	CBH
Gorontalo	1989	Census	CBH
Gorontalo	1990	Census	CBH
Gorontalo	1990	Census	SBH
Gorontalo	1991	Census	CBH
Gorontalo	1992	Census	CBH
Gorontalo	1993	Census	CBH
Gorontalo	1994	Census	CBH
Gorontalo	1995	Census	CBH
Gorontalo	1996	Census	CBH
Gorontalo	1997	Census	CBH
Gorontalo	1998	Census	CBH
Gorontalo	1999	Census	CBH
Gorontalo	2000	Census	SBH
Gorontalo	2000	Census	CBH
Gorontalo	2002	Census	CBH
Gorontalo	2004	Census	CBH

Appendix Table 10. Under-5 mortality reference sources by source date and location, 1950-2016			
Location	Source	Source years	Method
Gorontalo	2010	Census	SBH
Gorontalo	1977	Demographic and Health Survey (DHS)	CBH
Gorontalo	1979	Demographic and Health Survey (DHS)	CBH
Gorontalo	1981	Demographic and Health Survey (DHS)	CBH
Gorontalo	1983	Demographic and Health Survey (DHS)	CBH
Gorontalo	1985	Demographic and Health Survey (DHS)	CBH
Gorontalo	1987	Demographic and Health Survey (DHS)	CBH
Gorontalo	1989	Demographic and Health Survey (DHS)	CBH
Gorontalo	1991	Demographic and Health Survey (DHS)	CBH
Gorontalo	1993	Demographic and Health Survey (DHS)	CBH
Gorontalo	1995	Demographic and Health Survey (DHS)	CBH
Gorontalo	1997	Demographic and Health Survey (DHS)	CBH
Gorontalo	1999	Demographic and Health Survey (DHS)	CBH
Gorontalo	2001	Demographic and Health Survey (DHS)	CBH
Gorontalo	2003	Demographic and Health Survey (DHS)	SBH
Gorontalo	2003	Demographic and Health Survey (DHS)	CBH
Gorontalo	2005	Demographic and Health Survey (DHS)	CBH
Gorontalo	2007	Demographic and Health Survey (DHS)	CBH
Gorontalo	2007	Demographic and Health Survey (DHS)	SBH
Gorontalo	2009	Demographic and Health Survey (DHS)	CBH
Gorontalo	2011	Demographic and Health Survey (DHS)	CBH
Gorontalo	2012	Demographic and Health Survey (DHS)	SBH
Gorontalo	1985	Health Survey	SBH
Gorontalo	1995	Health Survey	SBH
Gorontalo	2001	Health Survey	SBH
Gorontalo	2002	Health Survey	SBH
Gorontalo	2003	Health Survey	SBH
Gorontalo	2004	Health Survey	SBH
Gorontalo	2005	Health Survey	SBH
Gorontalo	2006	Health Survey	SBH
Gorontalo	2007	Health Survey	SBH
Gorontalo	2008	Health Survey	SBH
Gorontalo	2009	Health Survey	SBH
Gorontalo	2010	Health Survey	SBH
Gorontalo	2011	Health Survey	SBH
Gorontalo	2012	Health Survey	SBH
Gorontalo	2013	Health Survey	SBH
Jakarta	1958	Census	CBH
Jakarta	1960	Census	CBH
Jakarta	1962	Census	CBH
Jakarta	1964	Census	CBH
Jakarta	1965	Census	CBH
Jakarta	1966	Census	CBH
Jakarta	1967	Census	CBH
Jakarta	1968	Census	CBH
Jakarta	1969	Census	CBH
Jakarta	1970	Census	CBH
Jakarta	1971	Census	CBH
Jakarta	1972	Census	CBH
Jakarta	1973	Census	CBH
Jakarta	1974	Census	CBH
Jakarta	1975	Census	CBH
Jakarta	1976	Census	CBH
Jakarta	1977	Census	CBH
Jakarta	1978	Census	CBH
Jakarta	1979	Census	CBH
Jakarta	1980	Census	CBH
Jakarta	1980	Census	SBH
Jakarta	1981	Census	CBH
Jakarta	1982	Census	CBH
Jakarta	1983	Census	CBH
Jakarta	1984	Census	CBH
Jakarta	1985	Census	CBH
Jakarta	1986	Census	CBH
Jakarta	1987	Census	CBH
Jakarta	1988	Census	CBH
Jakarta	1989	Census	CBH

Appendix Table 10. Under-5 mortality reference sources by source date and location, 1950-2016			
**Location**	**Source**	**Source years**	**Method**
Jakarta	1990	Census	CBH
Jakarta	1990	Census	SBH
Jakarta	1991	Census	CBH
Jakarta	1992	Census	CBH
Jakarta	1993	Census	CBH
Jakarta	1994	Census	CBH
Jakarta	1995	Census	CBH
Jakarta	1996	Census	CBH
Jakarta	1997	Census	CBH
Jakarta	1998	Census	CBH
Jakarta	1999	Census	CBH
Jakarta	2000	Census	CBH
Jakarta	2000	Census	SBH
Jakarta	2002	Census	CBH
Jakarta	2004	Census	CBH
Jakarta	2010	Census	SBH
Jakarta	1963	Demographic and Health Survey (DHS)	CBH
Jakarta	1965	Demographic and Health Survey (DHS)	CBH
Jakarta	1967	Demographic and Health Survey (DHS)	CBH
Jakarta	1969	Demographic and Health Survey (DHS)	CBH
Jakarta	1971	Demographic and Health Survey (DHS)	CBH
Jakarta	1973	Demographic and Health Survey (DHS)	CBH
Jakarta	1975	Demographic and Health Survey (DHS)	CBH
Jakarta	1977	Demographic and Health Survey (DHS)	CBH
Jakarta	1979	Demographic and Health Survey (DHS)	CBH
Jakarta	1981	Demographic and Health Survey (DHS)	CBH
Jakarta	1983	Demographic and Health Survey (DHS)	CBH
Jakarta	1985	Demographic and Health Survey (DHS)	CBH
Jakarta	1987	Demographic and Health Survey (DHS)	SBH
Jakarta	1987	Demographic and Health Survey (DHS)	CBH
Jakarta	1989	Demographic and Health Survey (DHS)	CBH
Jakarta	1991	Demographic and Health Survey (DHS)	CBH
Jakarta	1991	Demographic and Health Survey (DHS)	SBH
Jakarta	1993	Demographic and Health Survey (DHS)	CBH
Jakarta	1994	Demographic and Health Survey (DHS)	SBH
Jakarta	1995	Demographic and Health Survey (DHS)	CBH
Jakarta	1997	Demographic and Health Survey (DHS)	CBH
Jakarta	1997	Demographic and Health Survey (DHS)	SBH
Jakarta	1999	Demographic and Health Survey (DHS)	CBH
Jakarta	2001	Demographic and Health Survey (DHS)	CBH
Jakarta	2003	Demographic and Health Survey (DHS)	SBH
Jakarta	2003	Demographic and Health Survey (DHS)	CBH
Jakarta	2005	Demographic and Health Survey (DHS)	CBH
Jakarta	2007	Demographic and Health Survey (DHS)	CBH
Jakarta	2007	Demographic and Health Survey (DHS)	SBH
Jakarta	2009	Demographic and Health Survey (DHS)	CBH
Jakarta	2011	Demographic and Health Survey (DHS)	CBH
Jakarta	2012	Demographic and Health Survey (DHS)	SBH
Jakarta	1980	Health Survey	CBH
Jakarta	1982	Health Survey	CBH
Jakarta	1984	Health Survey	CBH
Jakarta	1985	Health Survey	SBH
Jakarta	1986	Health Survey	CBH
Jakarta	1988	Health Survey	CBH
Jakarta	1990	Health Survey	CBH
Jakarta	1992	Health Survey	CBH
Jakarta	1992	Health Survey	SBH
Jakarta	1993	Health Survey	SBH
Jakarta	1994	Health Survey	CBH
Jakarta	1994	Health Survey	SBH
Jakarta	1995	Health Survey	SBH
Jakarta	1996	Health Survey	CBH
Jakarta	1996	Health Survey	SBH
Jakarta	1997	Health Survey	SBH
Jakarta	1998	Health Survey	SBH
Jakarta	1998	Health Survey	CBH
Jakarta	1999	Health Survey	SBH

Appendix Table 10. Under-5 mortality reference sources by source date and location, 1950-2016			
**Location**	**Source**	**Source years**	**Method**
Jakarta	2000	Health Survey	CBH
Jakarta	2000	Health Survey	SBH
Jakarta	2001	Health Survey	SBH
Jakarta	2002	Health Survey	SBH
Jakarta	2003	Health Survey	SBH
Jakarta	2004	Health Survey	SBH
Jakarta	2005	Health Survey	SBH
Jakarta	2006	Health Survey	SBH
Jakarta	2007	Health Survey	SBH
Jakarta	2008	Health Survey	SBH
Jakarta	2009	Health Survey	SBH
Jakarta	2010	Health Survey	SBH
Jakarta	2011	Health Survey	SBH
Jakarta	2012	Health Survey	SBH
Jakarta	2013	Health Survey	SBH
Jambi	1961	Census	CBH
Jambi	1963	Census	CBH
Jambi	1964	Census	CBH
Jambi	1965	Census	CBH
Jambi	1966	Census	CBH
Jambi	1967	Census	CBH
Jambi	1968	Census	CBH
Jambi	1969	Census	CBH
Jambi	1970	Census	CBH
Jambi	1971	Census	CBH
Jambi	1972	Census	CBH
Jambi	1973	Census	CBH
Jambi	1974	Census	CBH
Jambi	1975	Census	CBH
Jambi	1976	Census	CBH
Jambi	1977	Census	CBH
Jambi	1978	Census	CBH
Jambi	1979	Census	CBH
Jambi	1980	Census	CBH
Jambi	1980	Census	SBH
Jambi	1981	Census	CBH
Jambi	1982	Census	CBH
Jambi	1983	Census	CBH
Jambi	1984	Census	CBH
Jambi	1985	Census	CBH
Jambi	1986	Census	CBH
Jambi	1987	Census	CBH
Jambi	1988	Census	CBH
Jambi	1989	Census	CBH
Jambi	1990	Census	CBH
Jambi	1990	Census	SBH
Jambi	1991	Census	CBH
Jambi	1992	Census	CBH
Jambi	1993	Census	CBH
Jambi	1994	Census	CBH
Jambi	1995	Census	CBH
Jambi	1996	Census	CBH
Jambi	1997	Census	CBH
Jambi	1998	Census	CBH
Jambi	1999	Census	CBH
Jambi	2000	Census	CBH
Jambi	2000	Census	SBH
Jambi	2002	Census	CBH
Jambi	2004	Census	CBH
Jambi	2010	Census	SBH
Jambi	1973	Demographic and Health Survey (DHS)	CBH
Jambi	1975	Demographic and Health Survey (DHS)	CBH
Jambi	1977	Demographic and Health Survey (DHS)	CBH
Jambi	1979	Demographic and Health Survey (DHS)	CBH
Jambi	1981	Demographic and Health Survey (DHS)	CBH
Jambi	1983	Demographic and Health Survey (DHS)	CBH
Jambi	1985	Demographic and Health Survey (DHS)	CBH

Appendix Table 10. Under-5 mortality reference sources by source date and location, 1950-2016			
**Location**	**Source**	**Source years**	**Method**
Jambi	1987	Demographic and Health Survey (DHS)	CBH
Jambi	1989	Demographic and Health Survey (DHS)	CBH
Jambi	1991	Demographic and Health Survey (DHS)	SBH
Jambi	1991	Demographic and Health Survey (DHS)	CBH
Jambi	1993	Demographic and Health Survey (DHS)	CBH
Jambi	1994	Demographic and Health Survey (DHS)	SBH
Jambi	1995	Demographic and Health Survey (DHS)	CBH
Jambi	1997	Demographic and Health Survey (DHS)	CBH
Jambi	1997	Demographic and Health Survey (DHS)	SBH
Jambi	1999	Demographic and Health Survey (DHS)	CBH
Jambi	2001	Demographic and Health Survey (DHS)	CBH
Jambi	2003	Demographic and Health Survey (DHS)	CBH
Jambi	2003	Demographic and Health Survey (DHS)	SBH
Jambi	2005	Demographic and Health Survey (DHS)	CBH
Jambi	2007	Demographic and Health Survey (DHS)	SBH
Jambi	2007	Demographic and Health Survey (DHS)	CBH
Jambi	2009	Demographic and Health Survey (DHS)	CBH
Jambi	2011	Demographic and Health Survey (DHS)	CBH
Jambi	2012	Demographic and Health Survey (DHS)	SBH
Jambi	1985	Health Survey	SBH
Jambi	1992	Health Survey	SBH
Jambi	1993	Health Survey	SBH
Jambi	1994	Health Survey	SBH
Jambi	1995	Health Survey	SBH
Jambi	1996	Health Survey	SBH
Jambi	1998	Health Survey	SBH
Jambi	1999	Health Survey	SBH
Jambi	2000	Health Survey	SBH
Jambi	2001	Health Survey	SBH
Jambi	2002	Health Survey	SBH
Jambi	2003	Health Survey	SBH
Jambi	2004	Health Survey	SBH
Jambi	2005	Health Survey	SBH
Jambi	2006	Health Survey	SBH
Jambi	2007	Health Survey	SBH
Jambi	2008	Health Survey	SBH
Jambi	2009	Health Survey	SBH
Jambi	2010	Health Survey	SBH
Jambi	2011	Health Survey	SBH
Jambi	2012	Health Survey	SBH
Jambi	2013	Health Survey	SBH
Jawa Barat	1949	Census	CBH
Jawa Barat	1950	Census	CBH
Jawa Barat	1951	Census	CBH
Jawa Barat	1952	Census	CBH
Jawa Barat	1953	Census	CBH
Jawa Barat	1954	Census	CBH
Jawa Barat	1955	Census	CBH
Jawa Barat	1956	Census	CBH
Jawa Barat	1957	Census	CBH
Jawa Barat	1958	Census	CBH
Jawa Barat	1959	Census	CBH
Jawa Barat	1960	Census	CBH
Jawa Barat	1961	Census	CBH
Jawa Barat	1962	Census	CBH
Jawa Barat	1963	Census	CBH
Jawa Barat	1964	Census	CBH
Jawa Barat	1965	Census	CBH
Jawa Barat	1966	Census	CBH
Jawa Barat	1967	Census	CBH
Jawa Barat	1968	Census	CBH
Jawa Barat	1969	Census	CBH
Jawa Barat	1970	Census	CBH
Jawa Barat	1971	Census	CBH
Jawa Barat	1972	Census	CBH
Jawa Barat	1973	Census	CBH
Jawa Barat	1974	Census	CBH

Appendix Table 10. Under-5 mortality reference sources by source date and location, 1950-2016			
**Location**	**Source**	**Source years**	**Method**
Jawa Barat	1975	Census	CBH
Jawa Barat	1976	Census	CBH
Jawa Barat	1977	Census	CBH
Jawa Barat	1978	Census	CBH
Jawa Barat	1979	Census	CBH
Jawa Barat	1980	Census	CBH
Jawa Barat	1980	Census	SBH
Jawa Barat	1981	Census	CBH
Jawa Barat	1982	Census	CBH
Jawa Barat	1983	Census	CBH
Jawa Barat	1984	Census	CBH
Jawa Barat	1985	Census	CBH
Jawa Barat	1986	Census	CBH
Jawa Barat	1987	Census	CBH
Jawa Barat	1988	Census	CBH
Jawa Barat	1989	Census	CBH
Jawa Barat	1990	Census	CBH
Jawa Barat	1990	Census	SBH
Jawa Barat	1991	Census	CBH
Jawa Barat	1992	Census	CBH
Jawa Barat	1993	Census	CBH
Jawa Barat	1994	Census	CBH
Jawa Barat	1995	Census	CBH
Jawa Barat	1996	Census	CBH
Jawa Barat	1997	Census	CBH
Jawa Barat	1998	Census	CBH
Jawa Barat	1999	Census	CBH
Jawa Barat	2000	Census	SBH
Jawa Barat	2000	Census	CBH
Jawa Barat	2002	Census	CBH
Jawa Barat	2004	Census	CBH
Jawa Barat	2010	Census	SBH
Jawa Barat	1965	Demographic and Health Survey (DHS)	CBH
Jawa Barat	1967	Demographic and Health Survey (DHS)	CBH
Jawa Barat	1969	Demographic and Health Survey (DHS)	CBH
Jawa Barat	1971	Demographic and Health Survey (DHS)	CBH
Jawa Barat	1973	Demographic and Health Survey (DHS)	CBH
Jawa Barat	1975	Demographic and Health Survey (DHS)	CBH
Jawa Barat	1977	Demographic and Health Survey (DHS)	CBH
Jawa Barat	1979	Demographic and Health Survey (DHS)	CBH
Jawa Barat	1981	Demographic and Health Survey (DHS)	CBH
Jawa Barat	1983	Demographic and Health Survey (DHS)	CBH
Jawa Barat	1985	Demographic and Health Survey (DHS)	CBH
Jawa Barat	1987	Demographic and Health Survey (DHS)	CBH
Jawa Barat	1987	Demographic and Health Survey (DHS)	SBH
Jawa Barat	1989	Demographic and Health Survey (DHS)	CBH
Jawa Barat	1991	Demographic and Health Survey (DHS)	SBH
Jawa Barat	1991	Demographic and Health Survey (DHS)	CBH
Jawa Barat	1993	Demographic and Health Survey (DHS)	CBH
Jawa Barat	1994	Demographic and Health Survey (DHS)	SBH
Jawa Barat	1995	Demographic and Health Survey (DHS)	CBH
Jawa Barat	1997	Demographic and Health Survey (DHS)	CBH
Jawa Barat	1997	Demographic and Health Survey (DHS)	SBH
Jawa Barat	1999	Demographic and Health Survey (DHS)	CBH
Jawa Barat	2001	Demographic and Health Survey (DHS)	CBH
Jawa Barat	2003	Demographic and Health Survey (DHS)	SBH
Jawa Barat	2003	Demographic and Health Survey (DHS)	CBH
Jawa Barat	2005	Demographic and Health Survey (DHS)	CBH
Jawa Barat	2007	Demographic and Health Survey (DHS)	SBH
Jawa Barat	2007	Demographic and Health Survey (DHS)	CBH
Jawa Barat	2009	Demographic and Health Survey (DHS)	CBH
Jawa Barat	2011	Demographic and Health Survey (DHS)	CBH
Jawa Barat	2012	Demographic and Health Survey (DHS)	SBH
Jawa Barat	1978	Health Survey	CBH
Jawa Barat	1980	Health Survey	CBH
Jawa Barat	1982	Health Survey	CBH
Jawa Barat	1984	Health Survey	CBH

Appendix Table 10. Under-5 mortality reference sources by source date and location, 1950-2016			
**Location**	**Source**	**Source years**	**Method**
Jawa Barat	1985	Health Survey	SBH
Jawa Barat	1986	Health Survey	CBH
Jawa Barat	1988	Health Survey	CBH
Jawa Barat	1990	Health Survey	CBH
Jawa Barat	1992	Health Survey	CBH
Jawa Barat	1992	Health Survey	SBH
Jawa Barat	1993	Health Survey	SBH
Jawa Barat	1994	Health Survey	SBH
Jawa Barat	1994	Health Survey	CBH
Jawa Barat	1995	Health Survey	SBH
Jawa Barat	1996	Health Survey	CBH
Jawa Barat	1996	Health Survey	SBH
Jawa Barat	1997	Health Survey	SBH
Jawa Barat	1998	Health Survey	CBH
Jawa Barat	1998	Health Survey	SBH
Jawa Barat	1999	Health Survey	SBH
Jawa Barat	2000	Health Survey	SBH
Jawa Barat	2000	Health Survey	CBH
Jawa Barat	2001	Health Survey	SBH
Jawa Barat	2002	Health Survey	SBH
Jawa Barat	2003	Health Survey	SBH
Jawa Barat	2004	Health Survey	SBH
Jawa Barat	2005	Health Survey	SBH
Jawa Barat	2006	Health Survey	SBH
Jawa Barat	2007	Health Survey	SBH
Jawa Barat	2008	Health Survey	SBH
Jawa Barat	2009	Health Survey	SBH
Jawa Barat	2010	Health Survey	SBH
Jawa Barat	2011	Health Survey	SBH
Jawa Barat	2012	Health Survey	SBH
Jawa Barat	2013	Health Survey	SBH
Jawa Tengah	1949	Census	CBH
Jawa Tengah	1950	Census	CBH
Jawa Tengah	1951	Census	CBH
Jawa Tengah	1952	Census	CBH
Jawa Tengah	1953	Census	CBH
Jawa Tengah	1954	Census	CBH
Jawa Tengah	1955	Census	CBH
Jawa Tengah	1956	Census	CBH
Jawa Tengah	1957	Census	CBH
Jawa Tengah	1958	Census	CBH
Jawa Tengah	1959	Census	CBH
Jawa Tengah	1960	Census	CBH
Jawa Tengah	1961	Census	CBH
Jawa Tengah	1962	Census	CBH
Jawa Tengah	1963	Census	CBH
Jawa Tengah	1964	Census	CBH
Jawa Tengah	1965	Census	CBH
Jawa Tengah	1966	Census	CBH
Jawa Tengah	1967	Census	CBH
Jawa Tengah	1968	Census	CBH
Jawa Tengah	1969	Census	CBH
Jawa Tengah	1970	Census	CBH
Jawa Tengah	1971	Census	CBH
Jawa Tengah	1972	Census	CBH
Jawa Tengah	1973	Census	CBH
Jawa Tengah	1974	Census	CBH
Jawa Tengah	1975	Census	CBH
Jawa Tengah	1976	Census	CBH
Jawa Tengah	1977	Census	CBH
Jawa Tengah	1978	Census	CBH
Jawa Tengah	1979	Census	CBH
Jawa Tengah	1980	Census	SBH
Jawa Tengah	1980	Census	CBH
Jawa Tengah	1981	Census	CBH
Jawa Tengah	1982	Census	CBH
Jawa Tengah	1983	Census	CBH

Appendix Table 10. Under-5 mortality reference sources by source date and location, 1950-2016			
Location	Source	Source years	Method
Jawa Tengah	1984	Census	CBH
Jawa Tengah	1985	Census	CBH
Jawa Tengah	1986	Census	CBH
Jawa Tengah	1987	Census	CBH
Jawa Tengah	1988	Census	CBH
Jawa Tengah	1989	Census	CBH
Jawa Tengah	1990	Census	SBH
Jawa Tengah	1990	Census	CBH
Jawa Tengah	1991	Census	CBH
Jawa Tengah	1992	Census	CBH
Jawa Tengah	1993	Census	CBH
Jawa Tengah	1994	Census	CBH
Jawa Tengah	1995	Census	CBH
Jawa Tengah	1996	Census	CBH
Jawa Tengah	1997	Census	CBH
Jawa Tengah	1998	Census	CBH
Jawa Tengah	1999	Census	CBH
Jawa Tengah	2000	Census	SBH
Jawa Tengah	2000	Census	CBH
Jawa Tengah	2002	Census	CBH
Jawa Tengah	2004	Census	CBH
Jawa Tengah	2010	Census	SBH
Jawa Tengah	1965	Demographic and Health Survey (DHS)	CBH
Jawa Tengah	1967	Demographic and Health Survey (DHS)	CBH
Jawa Tengah	1969	Demographic and Health Survey (DHS)	CBH
Jawa Tengah	1971	Demographic and Health Survey (DHS)	CBH
Jawa Tengah	1973	Demographic and Health Survey (DHS)	CBH
Jawa Tengah	1975	Demographic and Health Survey (DHS)	CBH
Jawa Tengah	1977	Demographic and Health Survey (DHS)	CBH
Jawa Tengah	1979	Demographic and Health Survey (DHS)	CBH
Jawa Tengah	1981	Demographic and Health Survey (DHS)	CBH
Jawa Tengah	1983	Demographic and Health Survey (DHS)	CBH
Jawa Tengah	1985	Demographic and Health Survey (DHS)	CBH
Jawa Tengah	1987	Demographic and Health Survey (DHS)	SBH
Jawa Tengah	1987	Demographic and Health Survey (DHS)	CBH
Jawa Tengah	1989	Demographic and Health Survey (DHS)	CBH
Jawa Tengah	1991	Demographic and Health Survey (DHS)	CBH
Jawa Tengah	1991	Demographic and Health Survey (DHS)	SBH
Jawa Tengah	1993	Demographic and Health Survey (DHS)	CBH
Jawa Tengah	1994	Demographic and Health Survey (DHS)	SBH
Jawa Tengah	1995	Demographic and Health Survey (DHS)	CBH
Jawa Tengah	1997	Demographic and Health Survey (DHS)	CBH
Jawa Tengah	1997	Demographic and Health Survey (DHS)	SBH
Jawa Tengah	1999	Demographic and Health Survey (DHS)	CBH
Jawa Tengah	2001	Demographic and Health Survey (DHS)	CBH
Jawa Tengah	2003	Demographic and Health Survey (DHS)	CBH
Jawa Tengah	2003	Demographic and Health Survey (DHS)	SBH
Jawa Tengah	2005	Demographic and Health Survey (DHS)	CBH
Jawa Tengah	2007	Demographic and Health Survey (DHS)	CBH
Jawa Tengah	2007	Demographic and Health Survey (DHS)	SBH
Jawa Tengah	2009	Demographic and Health Survey (DHS)	CBH
Jawa Tengah	2011	Demographic and Health Survey (DHS)	CBH
Jawa Tengah	2012	Demographic and Health Survey (DHS)	SBH
Jawa Tengah	1980	Health Survey	CBH
Jawa Tengah	1982	Health Survey	CBH
Jawa Tengah	1984	Health Survey	CBH
Jawa Tengah	1985	Health Survey	SBH
Jawa Tengah	1986	Health Survey	CBH
Jawa Tengah	1988	Health Survey	CBH
Jawa Tengah	1990	Health Survey	CBH
Jawa Tengah	1992	Health Survey	CBH
Jawa Tengah	1992	Health Survey	SBH
Jawa Tengah	1993	Health Survey	SBH
Jawa Tengah	1994	Health Survey	CBH
Jawa Tengah	1994	Health Survey	SBH
Jawa Tengah	1995	Health Survey	SBH
Jawa Tengah	1996	Health Survey	CBH

Appendix Table 10. Under-5 mortality reference sources by source date and location, 1950-2016

Location	Source	Source years	Method
Jawa Tengah	1996	Health Survey	SBH
Jawa Tengah	1997	Health Survey	SBH
Jawa Tengah	1998	Health Survey	CBH
Jawa Tengah	1998	Health Survey	SBH
Jawa Tengah	1999	Health Survey	SBH
Jawa Tengah	2000	Health Survey	SBH
Jawa Tengah	2000	Health Survey	CBH
Jawa Tengah	2001	Health Survey	SBH
Jawa Tengah	2002	Health Survey	SBH
Jawa Tengah	2003	Health Survey	SBH
Jawa Tengah	2004	Health Survey	SBH
Jawa Tengah	2005	Health Survey	SBH
Jawa Tengah	2006	Health Survey	SBH
Jawa Tengah	2007	Health Survey	SBH
Jawa Tengah	2008	Health Survey	SBH
Jawa Tengah	2009	Health Survey	SBH
Jawa Tengah	2010	Health Survey	SBH
Jawa Tengah	2011	Health Survey	SBH
Jawa Tengah	2012	Health Survey	SBH
Jawa Tengah	2013	Health Survey	SBH
Jawa Timur	1949	Census	CBH
Jawa Timur	1950	Census	CBH
Jawa Timur	1951	Census	CBH
Jawa Timur	1952	Census	CBH
Jawa Timur	1953	Census	CBH
Jawa Timur	1954	Census	CBH
Jawa Timur	1955	Census	CBH
Jawa Timur	1956	Census	CBH
Jawa Timur	1957	Census	CBH
Jawa Timur	1958	Census	CBH
Jawa Timur	1959	Census	CBH
Jawa Timur	1960	Census	CBH
Jawa Timur	1961	Census	CBH
Jawa Timur	1962	Census	CBH
Jawa Timur	1963	Census	CBH
Jawa Timur	1964	Census	CBH
Jawa Timur	1965	Census	CBH
Jawa Timur	1966	Census	CBH
Jawa Timur	1967	Census	CBH
Jawa Timur	1968	Census	CBH
Jawa Timur	1969	Census	CBH
Jawa Timur	1970	Census	CBH
Jawa Timur	1971	Census	CBH
Jawa Timur	1972	Census	CBH
Jawa Timur	1973	Census	CBH
Jawa Timur	1974	Census	CBH
Jawa Timur	1975	Census	CBH
Jawa Timur	1976	Census	CBH
Jawa Timur	1977	Census	CBH
Jawa Timur	1978	Census	CBH
Jawa Timur	1979	Census	CBH
Jawa Timur	1980	Census	SBH
Jawa Timur	1980	Census	CBH
Jawa Timur	1981	Census	CBH
Jawa Timur	1982	Census	CBH
Jawa Timur	1983	Census	CBH
Jawa Timur	1984	Census	CBH
Jawa Timur	1985	Census	CBH
Jawa Timur	1986	Census	CBH
Jawa Timur	1987	Census	CBH
Jawa Timur	1988	Census	CBH
Jawa Timur	1989	Census	CBH
Jawa Timur	1990	Census	CBH
Jawa Timur	1990	Census	SBH
Jawa Timur	1991	Census	CBH
Jawa Timur	1992	Census	CBH
Jawa Timur	1993	Census	CBH

Appendix Table 10. Under-5 mortality reference sources by source date and location, 1950-2016			
**Location**	**Source**	**Source years**	**Method**
Jawa Timur	1994	Census	CBH
Jawa Timur	1995	Census	CBH
Jawa Timur	1996	Census	CBH
Jawa Timur	1997	Census	CBH
Jawa Timur	1998	Census	CBH
Jawa Timur	1999	Census	CBH
Jawa Timur	2000	Census	CBH
Jawa Timur	2000	Census	SBH
Jawa Timur	2002	Census	CBH
Jawa Timur	2004	Census	CBH
Jawa Timur	2010	Census	SBH
Jawa Timur	1963	Demographic and Health Survey (DHS)	CBH
Jawa Timur	1965	Demographic and Health Survey (DHS)	CBH
Jawa Timur	1967	Demographic and Health Survey (DHS)	CBH
Jawa Timur	1969	Demographic and Health Survey (DHS)	CBH
Jawa Timur	1971	Demographic and Health Survey (DHS)	CBH
Jawa Timur	1973	Demographic and Health Survey (DHS)	CBH
Jawa Timur	1975	Demographic and Health Survey (DHS)	CBH
Jawa Timur	1977	Demographic and Health Survey (DHS)	CBH
Jawa Timur	1979	Demographic and Health Survey (DHS)	CBH
Jawa Timur	1981	Demographic and Health Survey (DHS)	CBH
Jawa Timur	1983	Demographic and Health Survey (DHS)	CBH
Jawa Timur	1985	Demographic and Health Survey (DHS)	CBH
Jawa Timur	1987	Demographic and Health Survey (DHS)	CBH
Jawa Timur	1987	Demographic and Health Survey (DHS)	SBH
Jawa Timur	1989	Demographic and Health Survey (DHS)	CBH
Jawa Timur	1991	Demographic and Health Survey (DHS)	SBH
Jawa Timur	1991	Demographic and Health Survey (DHS)	CBH
Jawa Timur	1993	Demographic and Health Survey (DHS)	CBH
Jawa Timur	1994	Demographic and Health Survey (DHS)	SBH
Jawa Timur	1995	Demographic and Health Survey (DHS)	CBH
Jawa Timur	1997	Demographic and Health Survey (DHS)	CBH
Jawa Timur	1997	Demographic and Health Survey (DHS)	SBH
Jawa Timur	1999	Demographic and Health Survey (DHS)	CBH
Jawa Timur	2001	Demographic and Health Survey (DHS)	CBH
Jawa Timur	2003	Demographic and Health Survey (DHS)	CBH
Jawa Timur	2003	Demographic and Health Survey (DHS)	SBH
Jawa Timur	2005	Demographic and Health Survey (DHS)	CBH
Jawa Timur	2007	Demographic and Health Survey (DHS)	SBH
Jawa Timur	2007	Demographic and Health Survey (DHS)	CBH
Jawa Timur	2009	Demographic and Health Survey (DHS)	CBH
Jawa Timur	2011	Demographic and Health Survey (DHS)	CBH
Jawa Timur	2012	Demographic and Health Survey (DHS)	SBH
Jawa Timur	1972	Health Survey	CBH
Jawa Timur	1974	Health Survey	CBH
Jawa Timur	1976	Health Survey	CBH
Jawa Timur	1978	Health Survey	CBH
Jawa Timur	1980	Health Survey	CBH
Jawa Timur	1982	Health Survey	CBH
Jawa Timur	1984	Health Survey	CBH
Jawa Timur	1985	Health Survey	SBH
Jawa Timur	1986	Health Survey	CBH
Jawa Timur	1988	Health Survey	CBH
Jawa Timur	1990	Health Survey	CBH
Jawa Timur	1992	Health Survey	CBH
Jawa Timur	1992	Health Survey	SBH
Jawa Timur	1993	Health Survey	SBH
Jawa Timur	1994	Health Survey	CBH
Jawa Timur	1994	Health Survey	SBH
Jawa Timur	1995	Health Survey	SBH
Jawa Timur	1996	Health Survey	SBH
Jawa Timur	1996	Health Survey	CBH
Jawa Timur	1997	Health Survey	SBH
Jawa Timur	1998	Health Survey	SBH
Jawa Timur	1998	Health Survey	CBH
Jawa Timur	1999	Health Survey	SBH
Jawa Timur	2000	Health Survey	CBH

Appendix Table 10. Under-5 mortality reference sources by source date and location, 1950-2016			
**Location**	**Source**	**Source years**	**Method**
Jawa Timur	2000	Health Survey	SBH
Jawa Timur	2001	Health Survey	SBH
Jawa Timur	2002	Health Survey	SBH
Jawa Timur	2003	Health Survey	SBH
Jawa Timur	2004	Health Survey	SBH
Jawa Timur	2005	Health Survey	SBH
Jawa Timur	2006	Health Survey	SBH
Jawa Timur	2007	Health Survey	SBH
Jawa Timur	2008	Health Survey	SBH
Jawa Timur	2009	Health Survey	SBH
Jawa Timur	2010	Health Survey	SBH
Jawa Timur	2011	Health Survey	SBH
Jawa Timur	2012	Health Survey	SBH
Jawa Timur	2013	Health Survey	SBH
Kalimantan Barat	1962	Census	CBH
Kalimantan Barat	1963	Census	CBH
Kalimantan Barat	1964	Census	CBH
Kalimantan Barat	1965	Census	CBH
Kalimantan Barat	1966	Census	CBH
Kalimantan Barat	1967	Census	CBH
Kalimantan Barat	1968	Census	CBH
Kalimantan Barat	1969	Census	CBH
Kalimantan Barat	1970	Census	CBH
Kalimantan Barat	1971	Census	CBH
Kalimantan Barat	1972	Census	CBH
Kalimantan Barat	1973	Census	CBH
Kalimantan Barat	1974	Census	CBH
Kalimantan Barat	1975	Census	CBH
Kalimantan Barat	1976	Census	CBH
Kalimantan Barat	1977	Census	CBH
Kalimantan Barat	1978	Census	CBH
Kalimantan Barat	1979	Census	CBH
Kalimantan Barat	1980	Census	CBH
Kalimantan Barat	1980	Census	SBH
Kalimantan Barat	1981	Census	CBH
Kalimantan Barat	1982	Census	CBH
Kalimantan Barat	1983	Census	CBH
Kalimantan Barat	1984	Census	CBH
Kalimantan Barat	1985	Census	CBH
Kalimantan Barat	1986	Census	CBH
Kalimantan Barat	1987	Census	CBH
Kalimantan Barat	1988	Census	CBH
Kalimantan Barat	1989	Census	CBH
Kalimantan Barat	1990	Census	CBH
Kalimantan Barat	1990	Census	SBH
Kalimantan Barat	1991	Census	CBH
Kalimantan Barat	1992	Census	CBH
Kalimantan Barat	1993	Census	CBH
Kalimantan Barat	1994	Census	CBH
Kalimantan Barat	1995	Census	CBH
Kalimantan Barat	1996	Census	CBH
Kalimantan Barat	1997	Census	CBH
Kalimantan Barat	1998	Census	CBH
Kalimantan Barat	1999	Census	CBH
Kalimantan Barat	2000	Census	CBH
Kalimantan Barat	2000	Census	SBH
Kalimantan Barat	2002	Census	CBH
Kalimantan Barat	2004	Census	CBH
Kalimantan Barat	2010	Census	SBH
Kalimantan Barat	1969	Demographic and Health Survey (DHS)	CBH
Kalimantan Barat	1971	Demographic and Health Survey (DHS)	CBH
Kalimantan Barat	1973	Demographic and Health Survey (DHS)	CBH
Kalimantan Barat	1975	Demographic and Health Survey (DHS)	CBH
Kalimantan Barat	1977	Demographic and Health Survey (DHS)	CBH
Kalimantan Barat	1979	Demographic and Health Survey (DHS)	CBH
Kalimantan Barat	1981	Demographic and Health Survey (DHS)	CBH
Kalimantan Barat	1983	Demographic and Health Survey (DHS)	CBH

Appendix Table 10. Under-5 mortality reference sources by source date and location, 1950-2016

Location	Source	Source years	Method
Kalimantan Barat	1985	Demographic and Health Survey (DHS)	CBH
Kalimantan Barat	1987	Demographic and Health Survey (DHS)	SBH
Kalimantan Barat	1987	Demographic and Health Survey (DHS)	CBH
Kalimantan Barat	1989	Demographic and Health Survey (DHS)	CBH
Kalimantan Barat	1991	Demographic and Health Survey (DHS)	CBH
Kalimantan Barat	1991	Demographic and Health Survey (DHS)	SBH
Kalimantan Barat	1993	Demographic and Health Survey (DHS)	CBH
Kalimantan Barat	1994	Demographic and Health Survey (DHS)	SBH
Kalimantan Barat	1995	Demographic and Health Survey (DHS)	CBH
Kalimantan Barat	1997	Demographic and Health Survey (DHS)	CBH
Kalimantan Barat	1997	Demographic and Health Survey (DHS)	SBH
Kalimantan Barat	1999	Demographic and Health Survey (DHS)	CBH
Kalimantan Barat	2001	Demographic and Health Survey (DHS)	CBH
Kalimantan Barat	2003	Demographic and Health Survey (DHS)	SBH
Kalimantan Barat	2003	Demographic and Health Survey (DHS)	CBH
Kalimantan Barat	2005	Demographic and Health Survey (DHS)	CBH
Kalimantan Barat	2007	Demographic and Health Survey (DHS)	SBH
Kalimantan Barat	2007	Demographic and Health Survey (DHS)	CBH
Kalimantan Barat	2009	Demographic and Health Survey (DHS)	CBH
Kalimantan Barat	2011	Demographic and Health Survey (DHS)	CBH
Kalimantan Barat	2012	Demographic and Health Survey (DHS)	SBH
Kalimantan Barat	1985	Health Survey	SBH
Kalimantan Barat	1992	Health Survey	SBH
Kalimantan Barat	1993	Health Survey	SBH
Kalimantan Barat	1994	Health Survey	SBH
Kalimantan Barat	1995	Health Survey	SBH
Kalimantan Barat	1996	Health Survey	SBH
Kalimantan Barat	1998	Health Survey	SBH
Kalimantan Barat	1999	Health Survey	SBH
Kalimantan Barat	2000	Health Survey	SBH
Kalimantan Barat	2001	Health Survey	SBH
Kalimantan Barat	2002	Health Survey	SBH
Kalimantan Barat	2003	Health Survey	SBH
Kalimantan Barat	2004	Health Survey	SBH
Kalimantan Barat	2005	Health Survey	SBH
Kalimantan Barat	2006	Health Survey	SBH
Kalimantan Barat	2007	Health Survey	SBH
Kalimantan Barat	2008	Health Survey	SBH
Kalimantan Barat	2009	Health Survey	SBH
Kalimantan Barat	2010	Health Survey	SBH
Kalimantan Barat	2011	Health Survey	SBH
Kalimantan Barat	2012	Health Survey	SBH
Kalimantan Barat	2013	Health Survey	SBH
Kalimantan Selatan	1958	Census	CBH
Kalimantan Selatan	1960	Census	CBH
Kalimantan Selatan	1962	Census	CBH
Kalimantan Selatan	1963	Census	CBH
Kalimantan Selatan	1964	Census	CBH
Kalimantan Selatan	1965	Census	CBH
Kalimantan Selatan	1966	Census	CBH
Kalimantan Selatan	1967	Census	CBH
Kalimantan Selatan	1968	Census	CBH
Kalimantan Selatan	1969	Census	CBH
Kalimantan Selatan	1970	Census	CBH
Kalimantan Selatan	1971	Census	CBH
Kalimantan Selatan	1972	Census	CBH
Kalimantan Selatan	1973	Census	CBH
Kalimantan Selatan	1974	Census	CBH
Kalimantan Selatan	1975	Census	CBH
Kalimantan Selatan	1976	Census	CBH
Kalimantan Selatan	1977	Census	CBH
Kalimantan Selatan	1978	Census	CBH
Kalimantan Selatan	1979	Census	CBH
Kalimantan Selatan	1980	Census	SBH
Kalimantan Selatan	1980	Census	CBH
Kalimantan Selatan	1981	Census	CBH
Kalimantan Selatan	1982	Census	CBH

Appendix Table 10. Under-5 mortality reference sources by source date and location, 1950-2016

Location	Source	Source years	Method
Kalimantan Selatan	1983	Census	CBH
Kalimantan Selatan	1984	Census	CBH
Kalimantan Selatan	1985	Census	CBH
Kalimantan Selatan	1986	Census	CBH
Kalimantan Selatan	1987	Census	CBH
Kalimantan Selatan	1988	Census	CBH
Kalimantan Selatan	1989	Census	CBH
Kalimantan Selatan	1990	Census	SBH
Kalimantan Selatan	1990	Census	CBH
Kalimantan Selatan	1991	Census	CBH
Kalimantan Selatan	1992	Census	CBH
Kalimantan Selatan	1993	Census	CBH
Kalimantan Selatan	1994	Census	CBH
Kalimantan Selatan	1995	Census	CBH
Kalimantan Selatan	1996	Census	CBH
Kalimantan Selatan	1997	Census	CBH
Kalimantan Selatan	1998	Census	CBH
Kalimantan Selatan	1999	Census	CBH
Kalimantan Selatan	2000	Census	SBH
Kalimantan Selatan	2000	Census	CBH
Kalimantan Selatan	2002	Census	CBH
Kalimantan Selatan	2004	Census	CBH
Kalimantan Selatan	2010	Census	SBH
Kalimantan Selatan	1967	Demographic and Health Survey (DHS)	CBH
Kalimantan Selatan	1969	Demographic and Health Survey (DHS)	CBH
Kalimantan Selatan	1971	Demographic and Health Survey (DHS)	CBH
Kalimantan Selatan	1973	Demographic and Health Survey (DHS)	CBH
Kalimantan Selatan	1975	Demographic and Health Survey (DHS)	CBH
Kalimantan Selatan	1977	Demographic and Health Survey (DHS)	CBH
Kalimantan Selatan	1979	Demographic and Health Survey (DHS)	CBH
Kalimantan Selatan	1981	Demographic and Health Survey (DHS)	CBH
Kalimantan Selatan	1983	Demographic and Health Survey (DHS)	CBH
Kalimantan Selatan	1985	Demographic and Health Survey (DHS)	CBH
Kalimantan Selatan	1987	Demographic and Health Survey (DHS)	CBH
Kalimantan Selatan	1987	Demographic and Health Survey (DHS)	SBH
Kalimantan Selatan	1989	Demographic and Health Survey (DHS)	CBH
Kalimantan Selatan	1991	Demographic and Health Survey (DHS)	SBH
Kalimantan Selatan	1991	Demographic and Health Survey (DHS)	CBH
Kalimantan Selatan	1993	Demographic and Health Survey (DHS)	CBH
Kalimantan Selatan	1994	Demographic and Health Survey (DHS)	SBH
Kalimantan Selatan	1995	Demographic and Health Survey (DHS)	CBH
Kalimantan Selatan	1997	Demographic and Health Survey (DHS)	SBH
Kalimantan Selatan	1997	Demographic and Health Survey (DHS)	CBH
Kalimantan Selatan	1999	Demographic and Health Survey (DHS)	CBH
Kalimantan Selatan	2001	Demographic and Health Survey (DHS)	CBH
Kalimantan Selatan	2003	Demographic and Health Survey (DHS)	CBH
Kalimantan Selatan	2003	Demographic and Health Survey (DHS)	SBH
Kalimantan Selatan	2005	Demographic and Health Survey (DHS)	CBH
Kalimantan Selatan	2007	Demographic and Health Survey (DHS)	SBH
Kalimantan Selatan	2007	Demographic and Health Survey (DHS)	CBH
Kalimantan Selatan	2009	Demographic and Health Survey (DHS)	CBH
Kalimantan Selatan	2011	Demographic and Health Survey (DHS)	CBH
Kalimantan Selatan	2012	Demographic and Health Survey (DHS)	SBH
Kalimantan Selatan	1980	Health Survey	CBH
Kalimantan Selatan	1982	Health Survey	CBH
Kalimantan Selatan	1984	Health Survey	CBH
Kalimantan Selatan	1985	Health Survey	SBH
Kalimantan Selatan	1986	Health Survey	CBH
Kalimantan Selatan	1988	Health Survey	CBH
Kalimantan Selatan	1990	Health Survey	CBH
Kalimantan Selatan	1992	Health Survey	SBH
Kalimantan Selatan	1992	Health Survey	CBH
Kalimantan Selatan	1993	Health Survey	SBH
Kalimantan Selatan	1994	Health Survey	SBH
Kalimantan Selatan	1994	Health Survey	CBH
Kalimantan Selatan	1995	Health Survey	SBH
Kalimantan Selatan	1996	Health Survey	CBH

Appendix Table 10. Under-5 mortality reference sources by source date and location, 1950-2016			
Location	Source	Source years	Method
Kalimantan Selatan	1996	Health Survey	SBH
Kalimantan Selatan	1997	Health Survey	SBH
Kalimantan Selatan	1998	Health Survey	CBH
Kalimantan Selatan	1998	Health Survey	SBH
Kalimantan Selatan	1999	Health Survey	SBH
Kalimantan Selatan	2000	Health Survey	SBH
Kalimantan Selatan	2000	Health Survey	CBH
Kalimantan Selatan	2001	Health Survey	SBH
Kalimantan Selatan	2002	Health Survey	SBH
Kalimantan Selatan	2003	Health Survey	SBH
Kalimantan Selatan	2004	Health Survey	SBH
Kalimantan Selatan	2005	Health Survey	SBH
Kalimantan Selatan	2006	Health Survey	SBH
Kalimantan Selatan	2007	Health Survey	SBH
Kalimantan Selatan	2008	Health Survey	SBH
Kalimantan Selatan	2009	Health Survey	SBH
Kalimantan Selatan	2010	Health Survey	SBH
Kalimantan Selatan	2011	Health Survey	SBH
Kalimantan Selatan	2012	Health Survey	SBH
Kalimantan Selatan	2013	Health Survey	SBH
Kalimantan Tengah	1964	Census	CBH
Kalimantan Tengah	1966	Census	CBH
Kalimantan Tengah	1968	Census	CBH
Kalimantan Tengah	1969	Census	CBH
Kalimantan Tengah	1970	Census	CBH
Kalimantan Tengah	1971	Census	CBH
Kalimantan Tengah	1972	Census	CBH
Kalimantan Tengah	1973	Census	CBH
Kalimantan Tengah	1974	Census	CBH
Kalimantan Tengah	1975	Census	CBH
Kalimantan Tengah	1976	Census	CBH
Kalimantan Tengah	1977	Census	CBH
Kalimantan Tengah	1978	Census	CBH
Kalimantan Tengah	1979	Census	CBH
Kalimantan Tengah	1980	Census	CBH
Kalimantan Tengah	1980	Census	SBH
Kalimantan Tengah	1981	Census	CBH
Kalimantan Tengah	1982	Census	CBH
Kalimantan Tengah	1983	Census	CBH
Kalimantan Tengah	1984	Census	CBH
Kalimantan Tengah	1985	Census	CBH
Kalimantan Tengah	1986	Census	CBH
Kalimantan Tengah	1987	Census	CBH
Kalimantan Tengah	1988	Census	CBH
Kalimantan Tengah	1989	Census	CBH
Kalimantan Tengah	1990	Census	SBH
Kalimantan Tengah	1990	Census	CBH
Kalimantan Tengah	1991	Census	CBH
Kalimantan Tengah	1992	Census	CBH
Kalimantan Tengah	1993	Census	CBH
Kalimantan Tengah	1994	Census	CBH
Kalimantan Tengah	1995	Census	CBH
Kalimantan Tengah	1996	Census	CBH
Kalimantan Tengah	1997	Census	CBH
Kalimantan Tengah	1998	Census	CBH
Kalimantan Tengah	1999	Census	CBH
Kalimantan Tengah	2000	Census	CBH
Kalimantan Tengah	2000	Census	SBH
Kalimantan Tengah	2002	Census	CBH
Kalimantan Tengah	2004	Census	CBH
Kalimantan Tengah	2010	Census	SBH
Kalimantan Tengah	1973	Demographic and Health Survey (DHS)	CBH
Kalimantan Tengah	1975	Demographic and Health Survey (DHS)	CBH
Kalimantan Tengah	1977	Demographic and Health Survey (DHS)	CBH
Kalimantan Tengah	1979	Demographic and Health Survey (DHS)	CBH
Kalimantan Tengah	1981	Demographic and Health Survey (DHS)	CBH
Kalimantan Tengah	1983	Demographic and Health Survey (DHS)	CBH

Appendix Table 10. Under-5 mortality reference sources by source date and location, 1950-2016			
**Location**	**Source**	**Source years**	**Method**
Kalimantan Tengah	1985	Demographic and Health Survey (DHS)	CBH
Kalimantan Tengah	1987	Demographic and Health Survey (DHS)	CBH
Kalimantan Tengah	1989	Demographic and Health Survey (DHS)	CBH
Kalimantan Tengah	1991	Demographic and Health Survey (DHS)	SBH
Kalimantan Tengah	1991	Demographic and Health Survey (DHS)	CBH
Kalimantan Tengah	1993	Demographic and Health Survey (DHS)	CBH
Kalimantan Tengah	1994	Demographic and Health Survey (DHS)	SBH
Kalimantan Tengah	1995	Demographic and Health Survey (DHS)	CBH
Kalimantan Tengah	1997	Demographic and Health Survey (DHS)	SBH
Kalimantan Tengah	1997	Demographic and Health Survey (DHS)	CBH
Kalimantan Tengah	1999	Demographic and Health Survey (DHS)	CBH
Kalimantan Tengah	2001	Demographic and Health Survey (DHS)	CBH
Kalimantan Tengah	2003	Demographic and Health Survey (DHS)	SBH
Kalimantan Tengah	2003	Demographic and Health Survey (DHS)	CBH
Kalimantan Tengah	2005	Demographic and Health Survey (DHS)	CBH
Kalimantan Tengah	2007	Demographic and Health Survey (DHS)	SBH
Kalimantan Tengah	2007	Demographic and Health Survey (DHS)	CBH
Kalimantan Tengah	2009	Demographic and Health Survey (DHS)	CBH
Kalimantan Tengah	2011	Demographic and Health Survey (DHS)	CBH
Kalimantan Tengah	2012	Demographic and Health Survey (DHS)	SBH
Kalimantan Tengah	1985	Health Survey	SBH
Kalimantan Tengah	1987	Health Survey	CBH
Kalimantan Tengah	1992	Health Survey	SBH
Kalimantan Tengah	1993	Health Survey	SBH
Kalimantan Tengah	1994	Health Survey	SBH
Kalimantan Tengah	1995	Health Survey	SBH
Kalimantan Tengah	1996	Health Survey	SBH
Kalimantan Tengah	1998	Health Survey	SBH
Kalimantan Tengah	1999	Health Survey	SBH
Kalimantan Tengah	2000	Health Survey	SBH
Kalimantan Tengah	2001	Health Survey	SBH
Kalimantan Tengah	2002	Health Survey	SBH
Kalimantan Tengah	2003	Health Survey	SBH
Kalimantan Tengah	2004	Health Survey	SBH
Kalimantan Tengah	2005	Health Survey	SBH
Kalimantan Tengah	2006	Health Survey	SBH
Kalimantan Tengah	2007	Health Survey	SBH
Kalimantan Tengah	2008	Health Survey	SBH
Kalimantan Tengah	2009	Health Survey	SBH
Kalimantan Tengah	2010	Health Survey	SBH
Kalimantan Tengah	2011	Health Survey	SBH
Kalimantan Tengah	2012	Health Survey	SBH
Kalimantan Tengah	2013	Health Survey	SBH
Kalimantan Timur	1965	Census	CBH
Kalimantan Timur	1966	Census	CBH
Kalimantan Timur	1967	Census	CBH
Kalimantan Timur	1968	Census	CBH
Kalimantan Timur	1969	Census	CBH
Kalimantan Timur	1970	Census	CBH
Kalimantan Timur	1971	Census	CBH
Kalimantan Timur	1972	Census	CBH
Kalimantan Timur	1973	Census	CBH
Kalimantan Timur	1974	Census	CBH
Kalimantan Timur	1975	Census	CBH
Kalimantan Timur	1976	Census	CBH
Kalimantan Timur	1977	Census	CBH
Kalimantan Timur	1978	Census	CBH
Kalimantan Timur	1979	Census	CBH
Kalimantan Timur	1980	Census	CBH
Kalimantan Timur	1980	Census	SBH
Kalimantan Timur	1981	Census	CBH
Kalimantan Timur	1982	Census	CBH
Kalimantan Timur	1983	Census	CBH
Kalimantan Timur	1984	Census	CBH
Kalimantan Timur	1985	Census	CBH
Kalimantan Timur	1986	Census	CBH
Kalimantan Timur	1987	Census	CBH

Appendix Table 10. Under-5 mortality reference sources by source date and location, 1950-2016			
Location	Source	Source years	Method
Kalimantan Timur	1988	Census	CBH
Kalimantan Timur	1989	Census	CBH
Kalimantan Timur	1990	Census	CBH
Kalimantan Timur	1990	Census	SBH
Kalimantan Timur	1991	Census	CBH
Kalimantan Timur	1992	Census	CBH
Kalimantan Timur	1993	Census	CBH
Kalimantan Timur	1994	Census	CBH
Kalimantan Timur	1995	Census	CBH
Kalimantan Timur	1996	Census	CBH
Kalimantan Timur	1997	Census	CBH
Kalimantan Timur	1998	Census	CBH
Kalimantan Timur	1999	Census	CBH
Kalimantan Timur	2000	Census	SBH
Kalimantan Timur	2000	Census	CBH
Kalimantan Timur	2002	Census	CBH
Kalimantan Timur	2004	Census	CBH
Kalimantan Timur	2010	Census	SBH
Kalimantan Timur	1973	Demographic and Health Survey (DHS)	CBH
Kalimantan Timur	1975	Demographic and Health Survey (DHS)	CBH
Kalimantan Timur	1977	Demographic and Health Survey (DHS)	CBH
Kalimantan Timur	1979	Demographic and Health Survey (DHS)	CBH
Kalimantan Timur	1981	Demographic and Health Survey (DHS)	CBH
Kalimantan Timur	1983	Demographic and Health Survey (DHS)	CBH
Kalimantan Timur	1985	Demographic and Health Survey (DHS)	CBH
Kalimantan Timur	1987	Demographic and Health Survey (DHS)	CBH
Kalimantan Timur	1989	Demographic and Health Survey (DHS)	CBH
Kalimantan Timur	1991	Demographic and Health Survey (DHS)	SBH
Kalimantan Timur	1991	Demographic and Health Survey (DHS)	CBH
Kalimantan Timur	1993	Demographic and Health Survey (DHS)	CBH
Kalimantan Timur	1994	Demographic and Health Survey (DHS)	SBH
Kalimantan Timur	1995	Demographic and Health Survey (DHS)	CBH
Kalimantan Timur	1997	Demographic and Health Survey (DHS)	CBH
Kalimantan Timur	1997	Demographic and Health Survey (DHS)	SBH
Kalimantan Timur	1999	Demographic and Health Survey (DHS)	CBH
Kalimantan Timur	2001	Demographic and Health Survey (DHS)	CBH
Kalimantan Timur	2003	Demographic and Health Survey (DHS)	CBH
Kalimantan Timur	2003	Demographic and Health Survey (DHS)	SBH
Kalimantan Timur	2005	Demographic and Health Survey (DHS)	CBH
Kalimantan Timur	2007	Demographic and Health Survey (DHS)	CBH
Kalimantan Timur	2007	Demographic and Health Survey (DHS)	SBH
Kalimantan Timur	2009	Demographic and Health Survey (DHS)	CBH
Kalimantan Timur	2011	Demographic and Health Survey (DHS)	CBH
Kalimantan Timur	2012	Demographic and Health Survey (DHS)	SBH
Kalimantan Timur	1985	Health Survey	SBH
Kalimantan Timur	1992	Health Survey	SBH
Kalimantan Timur	1993	Health Survey	SBH
Kalimantan Timur	1994	Health Survey	SBH
Kalimantan Timur	1995	Health Survey	SBH
Kalimantan Timur	1995	Health Survey	CBH
Kalimantan Timur	1996	Health Survey	SBH
Kalimantan Timur	1997	Health Survey	CBH
Kalimantan Timur	1998	Health Survey	SBH
Kalimantan Timur	1999	Health Survey	SBH
Kalimantan Timur	1999	Health Survey	CBH
Kalimantan Timur	2000	Health Survey	SBH
Kalimantan Timur	2001	Health Survey	SBH
Kalimantan Timur	2001	Health Survey	CBH
Kalimantan Timur	2002	Health Survey	SBH
Kalimantan Timur	2003	Health Survey	CBH
Kalimantan Timur	2003	Health Survey	SBH
Kalimantan Timur	2004	Health Survey	SBH
Kalimantan Timur	2005	Health Survey	SBH
Kalimantan Timur	2005	Health Survey	CBH
Kalimantan Timur	2006	Health Survey	SBH
Kalimantan Timur	2007	Health Survey	CBH
Kalimantan Timur	2007	Health Survey	SBH

Appendix Table 10. Under-5 mortality reference sources by source date and location, 1950-2016			
Location	Source	Source years	Method
Kalimantan Timur	2008	Health Survey	SBH
Kalimantan Timur	2009	Health Survey	SBH
Kalimantan Timur	2009	Health Survey	CBH
Kalimantan Timur	2010	Health Survey	SBH
Kalimantan Timur	2011	Health Survey	SBH
Kalimantan Timur	2011	Health Survey	CBH
Kalimantan Timur	2012	Health Survey	SBH
Kalimantan Timur	2013	Health Survey	SBH
Kalimantan Utara	1972	Census	CBH
Kalimantan Utara	1974	Census	CBH
Kalimantan Utara	1975	Census	CBH
Kalimantan Utara	1976	Census	CBH
Kalimantan Utara	1977	Census	CBH
Kalimantan Utara	1978	Census	CBH
Kalimantan Utara	1979	Census	CBH
Kalimantan Utara	1980	Census	SBH
Kalimantan Utara	1980	Census	CBH
Kalimantan Utara	1981	Census	CBH
Kalimantan Utara	1982	Census	CBH
Kalimantan Utara	1983	Census	CBH
Kalimantan Utara	1984	Census	CBH
Kalimantan Utara	1985	Census	CBH
Kalimantan Utara	1986	Census	CBH
Kalimantan Utara	1987	Census	CBH
Kalimantan Utara	1988	Census	CBH
Kalimantan Utara	1989	Census	CBH
Kalimantan Utara	1990	Census	SBH
Kalimantan Utara	1990	Census	CBH
Kalimantan Utara	1991	Census	CBH
Kalimantan Utara	1992	Census	CBH
Kalimantan Utara	1993	Census	CBH
Kalimantan Utara	1994	Census	CBH
Kalimantan Utara	1995	Census	CBH
Kalimantan Utara	1996	Census	CBH
Kalimantan Utara	1997	Census	CBH
Kalimantan Utara	1998	Census	CBH
Kalimantan Utara	1999	Census	CBH
Kalimantan Utara	2000	Census	SBH
Kalimantan Utara	2000	Census	CBH
Kalimantan Utara	2002	Census	CBH
Kalimantan Utara	2004	Census	CBH
Kalimantan Utara	2010	Census	SBH
Kalimantan Utara	1989	Demographic and Health Survey (DHS)	CBH
Kalimantan Utara	1991	Demographic and Health Survey (DHS)	CBH
Kalimantan Utara	1993	Demographic and Health Survey (DHS)	CBH
Kalimantan Utara	1995	Demographic and Health Survey (DHS)	CBH
Kalimantan Utara	1997	Demographic and Health Survey (DHS)	CBH
Kalimantan Utara	1999	Demographic and Health Survey (DHS)	CBH
Kalimantan Utara	2001	Demographic and Health Survey (DHS)	CBH
Kalimantan Utara	2003	Demographic and Health Survey (DHS)	CBH
Kalimantan Utara	2005	Demographic and Health Survey (DHS)	CBH
Kalimantan Utara	2007	Demographic and Health Survey (DHS)	CBH
Kalimantan Utara	2009	Demographic and Health Survey (DHS)	CBH
Kalimantan Utara	2011	Demographic and Health Survey (DHS)	CBH
Kalimantan Utara	1985	Health Survey	SBH
Kalimantan Utara	1995	Health Survey	SBH
Kalimantan Utara	2001	Health Survey	SBH
Kalimantan Utara	2003	Health Survey	SBH
Kalimantan Utara	2004	Health Survey	SBH
Kalimantan Utara	2005	Health Survey	SBH
Kalimantan Utara	2006	Health Survey	SBH
Kalimantan Utara	2008	Health Survey	SBH
Kalimantan Utara	2009	Health Survey	SBH
Kalimantan Utara	2010	Health Survey	SBH
Kalimantan Utara	2011	Health Survey	SBH
Kalimantan Utara	2012	Health Survey	SBH
Kalimantan Utara	2013	Health Survey	SBH

Appendix Table 10. Under-5 mortality reference sources by source date and location, 1950-2016			
**Location**	**Source**	**Source years**	**Method**
Kepulauan Riau	1970	Census	CBH
Kepulauan Riau	1971	Census	CBH
Kepulauan Riau	1972	Census	CBH
Kepulauan Riau	1973	Census	CBH
Kepulauan Riau	1974	Census	CBH
Kepulauan Riau	1975	Census	CBH
Kepulauan Riau	1976	Census	CBH
Kepulauan Riau	1977	Census	CBH
Kepulauan Riau	1978	Census	CBH
Kepulauan Riau	1979	Census	CBH
Kepulauan Riau	1980	Census	SBH
Kepulauan Riau	1980	Census	CBH
Kepulauan Riau	1981	Census	CBH
Kepulauan Riau	1982	Census	CBH
Kepulauan Riau	1983	Census	CBH
Kepulauan Riau	1984	Census	CBH
Kepulauan Riau	1985	Census	CBH
Kepulauan Riau	1986	Census	CBH
Kepulauan Riau	1987	Census	CBH
Kepulauan Riau	1988	Census	CBH
Kepulauan Riau	1989	Census	CBH
Kepulauan Riau	1990	Census	CBH
Kepulauan Riau	1990	Census	SBH
Kepulauan Riau	1991	Census	CBH
Kepulauan Riau	1992	Census	CBH
Kepulauan Riau	1993	Census	CBH
Kepulauan Riau	1994	Census	CBH
Kepulauan Riau	1995	Census	CBH
Kepulauan Riau	1996	Census	CBH
Kepulauan Riau	1997	Census	CBH
Kepulauan Riau	1998	Census	CBH
Kepulauan Riau	1999	Census	CBH
Kepulauan Riau	2000	Census	CBH
Kepulauan Riau	2000	Census	SBH
Kepulauan Riau	2002	Census	CBH
Kepulauan Riau	2004	Census	CBH
Kepulauan Riau	2010	Census	SBH
Kepulauan Riau	1981	Demographic and Health Survey (DHS)	CBH
Kepulauan Riau	1983	Demographic and Health Survey (DHS)	CBH
Kepulauan Riau	1985	Demographic and Health Survey (DHS)	CBH
Kepulauan Riau	1987	Demographic and Health Survey (DHS)	CBH
Kepulauan Riau	1989	Demographic and Health Survey (DHS)	CBH
Kepulauan Riau	1991	Demographic and Health Survey (DHS)	CBH
Kepulauan Riau	1993	Demographic and Health Survey (DHS)	CBH
Kepulauan Riau	1995	Demographic and Health Survey (DHS)	CBH
Kepulauan Riau	1997	Demographic and Health Survey (DHS)	CBH
Kepulauan Riau	1999	Demographic and Health Survey (DHS)	CBH
Kepulauan Riau	2001	Demographic and Health Survey (DHS)	CBH
Kepulauan Riau	2003	Demographic and Health Survey (DHS)	CBH
Kepulauan Riau	2005	Demographic and Health Survey (DHS)	CBH
Kepulauan Riau	2007	Demographic and Health Survey (DHS)	SBH
Kepulauan Riau	2007	Demographic and Health Survey (DHS)	CBH
Kepulauan Riau	2009	Demographic and Health Survey (DHS)	CBH
Kepulauan Riau	2011	Demographic and Health Survey (DHS)	CBH
Kepulauan Riau	2012	Demographic and Health Survey (DHS)	SBH
Kepulauan Riau	1985	Health Survey	SBH
Kepulauan Riau	1992	Health Survey	SBH
Kepulauan Riau	1993	Health Survey	SBH
Kepulauan Riau	1994	Health Survey	SBH
Kepulauan Riau	1995	Health Survey	SBH
Kepulauan Riau	1996	Health Survey	SBH
Kepulauan Riau	1998	Health Survey	SBH
Kepulauan Riau	1999	Health Survey	SBH
Kepulauan Riau	2000	Health Survey	SBH
Kepulauan Riau	2001	Health Survey	SBH
Kepulauan Riau	2003	Health Survey	SBH
Kepulauan Riau	2004	Health Survey	SBH

Appendix Table 10. Under-5 mortality reference sources by source date and location, 1950-2016			
**Location**	**Source**	**Source years**	**Method**
Kepulauan Riau	2005	Health Survey	SBH
Kepulauan Riau	2006	Health Survey	SBH
Kepulauan Riau	2007	Health Survey	CBH
Kepulauan Riau	2007	Health Survey	SBH
Kepulauan Riau	2008	Health Survey	SBH
Kepulauan Riau	2009	Health Survey	SBH
Kepulauan Riau	2010	Health Survey	SBH
Kepulauan Riau	2011	Health Survey	SBH
Kepulauan Riau	2012	Health Survey	SBH
Kepulauan Riau	2013	Health Survey	SBH
Lampung	1960	Census	CBH
Lampung	1961	Census	CBH
Lampung	1962	Census	CBH
Lampung	1963	Census	CBH
Lampung	1964	Census	CBH
Lampung	1965	Census	CBH
Lampung	1966	Census	CBH
Lampung	1967	Census	CBH
Lampung	1968	Census	CBH
Lampung	1969	Census	CBH
Lampung	1970	Census	CBH
Lampung	1971	Census	CBH
Lampung	1972	Census	CBH
Lampung	1973	Census	CBH
Lampung	1974	Census	CBH
Lampung	1975	Census	CBH
Lampung	1976	Census	CBH
Lampung	1977	Census	CBH
Lampung	1978	Census	CBH
Lampung	1979	Census	CBH
Lampung	1980	Census	SBH
Lampung	1980	Census	CBH
Lampung	1981	Census	CBH
Lampung	1982	Census	CBH
Lampung	1983	Census	CBH
Lampung	1984	Census	CBH
Lampung	1985	Census	CBH
Lampung	1986	Census	CBH
Lampung	1987	Census	CBH
Lampung	1988	Census	CBH
Lampung	1989	Census	CBH
Lampung	1990	Census	CBH
Lampung	1990	Census	SBH
Lampung	1991	Census	CBH
Lampung	1992	Census	CBH
Lampung	1993	Census	CBH
Lampung	1994	Census	CBH
Lampung	1995	Census	CBH
Lampung	1996	Census	CBH
Lampung	1997	Census	CBH
Lampung	1998	Census	CBH
Lampung	1999	Census	CBH
Lampung	2000	Census	CBH
Lampung	2000	Census	SBH
Lampung	2002	Census	CBH
Lampung	2004	Census	CBH
Lampung	1969	Demographic and Health Survey (DHS)	CBH
Lampung	1971	Demographic and Health Survey (DHS)	CBH
Lampung	1973	Demographic and Health Survey (DHS)	CBH
Lampung	1975	Demographic and Health Survey (DHS)	CBH
Lampung	1977	Demographic and Health Survey (DHS)	CBH
Lampung	1979	Demographic and Health Survey (DHS)	CBH
Lampung	1981	Demographic and Health Survey (DHS)	CBH
Lampung	1983	Demographic and Health Survey (DHS)	CBH
Lampung	1985	Demographic and Health Survey (DHS)	CBH
Lampung	1987	Demographic and Health Survey (DHS)	SBH
Lampung	1987	Demographic and Health Survey (DHS)	CBH

Appendix Table 10. Under-5 mortality reference sources by source date and location, 1950-2016			
**Location**	**Source**	**Source years**	**Method**
Lampung	1989	Demographic and Health Survey (DHS)	CBH
Lampung	1991	Demographic and Health Survey (DHS)	SBH
Lampung	1991	Demographic and Health Survey (DHS)	CBH
Lampung	1993	Demographic and Health Survey (DHS)	CBH
Lampung	1994	Demographic and Health Survey (DHS)	SBH
Lampung	1995	Demographic and Health Survey (DHS)	CBH
Lampung	1997	Demographic and Health Survey (DHS)	SBH
Lampung	1997	Demographic and Health Survey (DHS)	CBH
Lampung	1999	Demographic and Health Survey (DHS)	CBH
Lampung	2001	Demographic and Health Survey (DHS)	CBH
Lampung	2003	Demographic and Health Survey (DHS)	CBH
Lampung	2003	Demographic and Health Survey (DHS)	SBH
Lampung	2005	Demographic and Health Survey (DHS)	CBH
Lampung	2007	Demographic and Health Survey (DHS)	SBH
Lampung	2007	Demographic and Health Survey (DHS)	CBH
Lampung	2009	Demographic and Health Survey (DHS)	CBH
Lampung	2011	Demographic and Health Survey (DHS)	CBH
Lampung	2012	Demographic and Health Survey (DHS)	SBH
Lampung	1980	Health Survey	CBH
Lampung	1982	Health Survey	CBH
Lampung	1984	Health Survey	CBH
Lampung	1985	Health Survey	SBH
Lampung	1986	Health Survey	CBH
Lampung	1988	Health Survey	CBH
Lampung	1990	Health Survey	CBH
Lampung	1992	Health Survey	SBH
Lampung	1992	Health Survey	CBH
Lampung	1993	Health Survey	SBH
Lampung	1994	Health Survey	CBH
Lampung	1994	Health Survey	SBH
Lampung	1995	Health Survey	SBH
Lampung	1996	Health Survey	CBH
Lampung	1996	Health Survey	SBH
Lampung	1997	Health Survey	SBH
Lampung	1998	Health Survey	SBH
Lampung	1998	Health Survey	CBH
Lampung	1999	Health Survey	SBH
Lampung	2000	Health Survey	CBH
Lampung	2000	Health Survey	SBH
Lampung	2001	Health Survey	SBH
Lampung	2002	Health Survey	SBH
Lampung	2003	Health Survey	SBH
Lampung	2004	Health Survey	SBH
Lampung	2005	Health Survey	SBH
Lampung	2006	Health Survey	SBH
Lampung	2007	Health Survey	SBH
Lampung	2008	Health Survey	SBH
Lampung	2009	Health Survey	SBH
Lampung	2010	Health Survey	SBH
Lampung	2011	Health Survey	SBH
Lampung	2012	Health Survey	SBH
Lampung	2013	Health Survey	SBH
Maluku	1970	Census	CBH
Maluku	1971	Census	CBH
Maluku	1972	Census	CBH
Maluku	1973	Census	CBH
Maluku	1974	Census	CBH
Maluku	1975	Census	CBH
Maluku	1976	Census	CBH
Maluku	1977	Census	CBH
Maluku	1978	Census	CBH
Maluku	1979	Census	CBH
Maluku	1980	Census	SBH
Maluku	1980	Census	CBH
Maluku	1981	Census	CBH
Maluku	1982	Census	CBH
Maluku	1983	Census	CBH

Appendix Table 10. Under-5 mortality reference sources by source date and location, 1950-2016			
Location	Source	Source years	Method
Maluku	1984	Census	CBH
Maluku	1985	Census	CBH
Maluku	1986	Census	CBH
Maluku	1987	Census	CBH
Maluku	1988	Census	CBH
Maluku	1989	Census	CBH
Maluku	1990	Census	SBH
Maluku	1990	Census	CBH
Maluku	1991	Census	CBH
Maluku	1992	Census	CBH
Maluku	1993	Census	CBH
Maluku	1994	Census	CBH
Maluku	1995	Census	CBH
Maluku	1996	Census	CBH
Maluku	1997	Census	CBH
Maluku	1998	Census	CBH
Maluku	1999	Census	CBH
Maluku	2000	Census	CBH
Maluku	2000	Census	SBH
Maluku	2002	Census	CBH
Maluku	2004	Census	CBH
Maluku	2010	Census	SBH
Maluku	1991	Demographic and Health Survey (DHS)	CBH
Maluku	1991	Demographic and Health Survey (DHS)	SBH
Maluku	1993	Demographic and Health Survey (DHS)	CBH
Maluku	1994	Demographic and Health Survey (DHS)	SBH
Maluku	1995	Demographic and Health Survey (DHS)	CBH
Maluku	1997	Demographic and Health Survey (DHS)	SBH
Maluku	1997	Demographic and Health Survey (DHS)	CBH
Maluku	1999	Demographic and Health Survey (DHS)	CBH
Maluku	2001	Demographic and Health Survey (DHS)	CBH
Maluku	2003	Demographic and Health Survey (DHS)	CBH
Maluku	2005	Demographic and Health Survey (DHS)	CBH
Maluku	2007	Demographic and Health Survey (DHS)	CBH
Maluku	2007	Demographic and Health Survey (DHS)	SBH
Maluku	2009	Demographic and Health Survey (DHS)	CBH
Maluku	2011	Demographic and Health Survey (DHS)	CBH
Maluku	2012	Demographic and Health Survey (DHS)	SBH
Maluku	1985	Health Survey	SBH
Maluku	1992	Health Survey	SBH
Maluku	1993	Health Survey	SBH
Maluku	1994	Health Survey	SBH
Maluku	1995	Health Survey	SBH
Maluku	1996	Health Survey	SBH
Maluku	1997	Health Survey	CBH
Maluku	1998	Health Survey	SBH
Maluku	1999	Health Survey	SBH
Maluku	1999	Health Survey	CBH
Maluku	2001	Health Survey	SBH
Maluku	2001	Health Survey	CBH
Maluku	2002	Health Survey	SBH
Maluku	2003	Health Survey	SBH
Maluku	2003	Health Survey	CBH
Maluku	2004	Health Survey	SBH
Maluku	2005	Health Survey	SBH
Maluku	2005	Health Survey	CBH
Maluku	2006	Health Survey	SBH
Maluku	2007	Health Survey	CBH
Maluku	2007	Health Survey	SBH
Maluku	2008	Health Survey	SBH
Maluku	2009	Health Survey	CBH
Maluku	2009	Health Survey	SBH
Maluku	2010	Health Survey	SBH
Maluku	2011	Health Survey	CBH
Maluku	2011	Health Survey	SBH
Maluku	2012	Health Survey	SBH
Maluku	2013	Health Survey	SBH

Appendix Table 10. Under-5 mortality reference sources by source date and location, 1950-2016

Location	Source	Source years	Method
Maluku Utara	1970	Census	CBH
Maluku Utara	1972	Census	CBH
Maluku Utara	1973	Census	CBH
Maluku Utara	1974	Census	CBH
Maluku Utara	1975	Census	CBH
Maluku Utara	1976	Census	CBH
Maluku Utara	1977	Census	CBH
Maluku Utara	1978	Census	CBH
Maluku Utara	1979	Census	CBH
Maluku Utara	1980	Census	CBH
Maluku Utara	1980	Census	SBH
Maluku Utara	1981	Census	CBH
Maluku Utara	1982	Census	CBH
Maluku Utara	1983	Census	CBH
Maluku Utara	1984	Census	CBH
Maluku Utara	1985	Census	CBH
Maluku Utara	1986	Census	CBH
Maluku Utara	1987	Census	CBH
Maluku Utara	1988	Census	CBH
Maluku Utara	1989	Census	CBH
Maluku Utara	1990	Census	CBH
Maluku Utara	1990	Census	SBH
Maluku Utara	1991	Census	CBH
Maluku Utara	1992	Census	CBH
Maluku Utara	1993	Census	CBH
Maluku Utara	1994	Census	CBH
Maluku Utara	1995	Census	CBH
Maluku Utara	1996	Census	CBH
Maluku Utara	1997	Census	CBH
Maluku Utara	1998	Census	CBH
Maluku Utara	1999	Census	CBH
Maluku Utara	2000	Census	CBH
Maluku Utara	2002	Census	CBH
Maluku Utara	2004	Census	CBH
Maluku Utara	2010	Census	SBH
Maluku Utara	1991	Demographic and Health Survey (DHS)	SBH
Maluku Utara	1991	Demographic and Health Survey (DHS)	CBH
Maluku Utara	1993	Demographic and Health Survey (DHS)	CBH
Maluku Utara	1994	Demographic and Health Survey (DHS)	SBH
Maluku Utara	1995	Demographic and Health Survey (DHS)	CBH
Maluku Utara	1997	Demographic and Health Survey (DHS)	CBH
Maluku Utara	1997	Demographic and Health Survey (DHS)	SBH
Maluku Utara	1999	Demographic and Health Survey (DHS)	CBH
Maluku Utara	2001	Demographic and Health Survey (DHS)	CBH
Maluku Utara	2003	Demographic and Health Survey (DHS)	CBH
Maluku Utara	2005	Demographic and Health Survey (DHS)	CBH
Maluku Utara	2007	Demographic and Health Survey (DHS)	SBH
Maluku Utara	2007	Demographic and Health Survey (DHS)	CBH
Maluku Utara	2009	Demographic and Health Survey (DHS)	CBH
Maluku Utara	2011	Demographic and Health Survey (DHS)	CBH
Maluku Utara	2012	Demographic and Health Survey (DHS)	SBH
Maluku Utara	1985	Health Survey	SBH
Maluku Utara	1995	Health Survey	SBH
Maluku Utara	1997	Health Survey	CBH
Maluku Utara	1999	Health Survey	CBH
Maluku Utara	2001	Health Survey	CBH
Maluku Utara	2001	Health Survey	SBH
Maluku Utara	2002	Health Survey	SBH
Maluku Utara	2003	Health Survey	CBH
Maluku Utara	2003	Health Survey	SBH
Maluku Utara	2004	Health Survey	SBH
Maluku Utara	2005	Health Survey	CBH
Maluku Utara	2005	Health Survey	SBH
Maluku Utara	2006	Health Survey	SBH
Maluku Utara	2007	Health Survey	CBH
Maluku Utara	2007	Health Survey	SBH
Maluku Utara	2008	Health Survey	SBH

Appendix Table 10. Under-5 mortality reference sources by source date and location, 1950-2016			
Location	Source	Source years	Method
Maluku Utara	2009	Health Survey	CBH
Maluku Utara	2009	Health Survey	SBH
Maluku Utara	2010	Health Survey	SBH
Maluku Utara	2011	Health Survey	SBH
Maluku Utara	2011	Health Survey	CBH
Maluku Utara	2012	Health Survey	SBH
Maluku Utara	2013	Health Survey	SBH
Nusa Tenggara Barat	1961	Census	CBH
Nusa Tenggara Barat	1962	Census	CBH
Nusa Tenggara Barat	1963	Census	CBH
Nusa Tenggara Barat	1964	Census	CBH
Nusa Tenggara Barat	1965	Census	CBH
Nusa Tenggara Barat	1966	Census	CBH
Nusa Tenggara Barat	1967	Census	CBH
Nusa Tenggara Barat	1968	Census	CBH
Nusa Tenggara Barat	1969	Census	CBH
Nusa Tenggara Barat	1970	Census	CBH
Nusa Tenggara Barat	1971	Census	CBH
Nusa Tenggara Barat	1972	Census	CBH
Nusa Tenggara Barat	1973	Census	CBH
Nusa Tenggara Barat	1974	Census	CBH
Nusa Tenggara Barat	1975	Census	CBH
Nusa Tenggara Barat	1976	Census	CBH
Nusa Tenggara Barat	1977	Census	CBH
Nusa Tenggara Barat	1978	Census	CBH
Nusa Tenggara Barat	1979	Census	CBH
Nusa Tenggara Barat	1980	Census	CBH
Nusa Tenggara Barat	1980	Census	SBH
Nusa Tenggara Barat	1981	Census	CBH
Nusa Tenggara Barat	1982	Census	CBH
Nusa Tenggara Barat	1983	Census	CBH
Nusa Tenggara Barat	1984	Census	CBH
Nusa Tenggara Barat	1985	Census	CBH
Nusa Tenggara Barat	1986	Census	CBH
Nusa Tenggara Barat	1987	Census	CBH
Nusa Tenggara Barat	1988	Census	CBH
Nusa Tenggara Barat	1989	Census	CBH
Nusa Tenggara Barat	1990	Census	CBH
Nusa Tenggara Barat	1990	Census	SBH
Nusa Tenggara Barat	1991	Census	CBH
Nusa Tenggara Barat	1992	Census	CBH
Nusa Tenggara Barat	1993	Census	CBH
Nusa Tenggara Barat	1994	Census	CBH
Nusa Tenggara Barat	1995	Census	CBH
Nusa Tenggara Barat	1996	Census	CBH
Nusa Tenggara Barat	1997	Census	CBH
Nusa Tenggara Barat	1998	Census	CBH
Nusa Tenggara Barat	1999	Census	CBH
Nusa Tenggara Barat	2000	Census	SBH
Nusa Tenggara Barat	2000	Census	CBH
Nusa Tenggara Barat	2002	Census	CBH
Nusa Tenggara Barat	2004	Census	CBH
Nusa Tenggara Barat	2010	Census	SBH
Nusa Tenggara Barat	1969	Demographic and Health Survey (DHS)	CBH
Nusa Tenggara Barat	1971	Demographic and Health Survey (DHS)	CBH
Nusa Tenggara Barat	1973	Demographic and Health Survey (DHS)	CBH
Nusa Tenggara Barat	1975	Demographic and Health Survey (DHS)	CBH
Nusa Tenggara Barat	1977	Demographic and Health Survey (DHS)	CBH
Nusa Tenggara Barat	1979	Demographic and Health Survey (DHS)	CBH
Nusa Tenggara Barat	1981	Demographic and Health Survey (DHS)	CBH
Nusa Tenggara Barat	1983	Demographic and Health Survey (DHS)	CBH
Nusa Tenggara Barat	1985	Demographic and Health Survey (DHS)	CBH
Nusa Tenggara Barat	1987	Demographic and Health Survey (DHS)	SBH
Nusa Tenggara Barat	1987	Demographic and Health Survey (DHS)	CBH
Nusa Tenggara Barat	1989	Demographic and Health Survey (DHS)	CBH
Nusa Tenggara Barat	1991	Demographic and Health Survey (DHS)	CBH
Nusa Tenggara Barat	1991	Demographic and Health Survey (DHS)	SBH

Appendix Table 10. Under-5 mortality reference sources by source date and location, 1950-2016			
Location	Source	Source years	Method
Nusa Tenggara Barat	1993	Demographic and Health Survey (DHS)	CBH
Nusa Tenggara Barat	1994	Demographic and Health Survey (DHS)	SBH
Nusa Tenggara Barat	1995	Demographic and Health Survey (DHS)	CBH
Nusa Tenggara Barat	1997	Demographic and Health Survey (DHS)	SBH
Nusa Tenggara Barat	1997	Demographic and Health Survey (DHS)	CBH
Nusa Tenggara Barat	1999	Demographic and Health Survey (DHS)	CBH
Nusa Tenggara Barat	2001	Demographic and Health Survey (DHS)	CBH
Nusa Tenggara Barat	2003	Demographic and Health Survey (DHS)	SBH
Nusa Tenggara Barat	2003	Demographic and Health Survey (DHS)	CBH
Nusa Tenggara Barat	2005	Demographic and Health Survey (DHS)	CBH
Nusa Tenggara Barat	2007	Demographic and Health Survey (DHS)	CBH
Nusa Tenggara Barat	2007	Demographic and Health Survey (DHS)	SBH
Nusa Tenggara Barat	2009	Demographic and Health Survey (DHS)	CBH
Nusa Tenggara Barat	2011	Demographic and Health Survey (DHS)	CBH
Nusa Tenggara Barat	2012	Demographic and Health Survey (DHS)	SBH
Nusa Tenggara Barat	1980	Health Survey	CBH
Nusa Tenggara Barat	1982	Health Survey	CBH
Nusa Tenggara Barat	1984	Health Survey	CBH
Nusa Tenggara Barat	1985	Health Survey	SBH
Nusa Tenggara Barat	1986	Health Survey	CBH
Nusa Tenggara Barat	1988	Health Survey	CBH
Nusa Tenggara Barat	1990	Health Survey	CBH
Nusa Tenggara Barat	1992	Health Survey	SBH
Nusa Tenggara Barat	1992	Health Survey	CBH
Nusa Tenggara Barat	1993	Health Survey	SBH
Nusa Tenggara Barat	1994	Health Survey	SBH
Nusa Tenggara Barat	1994	Health Survey	CBH
Nusa Tenggara Barat	1995	Health Survey	SBH
Nusa Tenggara Barat	1996	Health Survey	SBH
Nusa Tenggara Barat	1996	Health Survey	CBH
Nusa Tenggara Barat	1997	Health Survey	SBH
Nusa Tenggara Barat	1998	Health Survey	CBH
Nusa Tenggara Barat	1998	Health Survey	SBH
Nusa Tenggara Barat	1999	Health Survey	SBH
Nusa Tenggara Barat	2000	Health Survey	SBH
Nusa Tenggara Barat	2000	Health Survey	CBH
Nusa Tenggara Barat	2001	Health Survey	SBH
Nusa Tenggara Barat	2002	Health Survey	SBH
Nusa Tenggara Barat	2003	Health Survey	SBH
Nusa Tenggara Barat	2004	Health Survey	SBH
Nusa Tenggara Barat	2005	Health Survey	SBH
Nusa Tenggara Barat	2006	Health Survey	SBH
Nusa Tenggara Barat	2007	Health Survey	SBH
Nusa Tenggara Barat	2008	Health Survey	SBH
Nusa Tenggara Barat	2009	Health Survey	SBH
Nusa Tenggara Barat	2010	Health Survey	SBH
Nusa Tenggara Barat	2011	Health Survey	SBH
Nusa Tenggara Barat	2012	Health Survey	SBH
Nusa Tenggara Barat	2013	Health Survey	SBH
Nusa Tenggara Timur	1957	Census	CBH
Nusa Tenggara Timur	1958	Census	CBH
Nusa Tenggara Timur	1959	Census	CBH
Nusa Tenggara Timur	1960	Census	CBH
Nusa Tenggara Timur	1961	Census	CBH
Nusa Tenggara Timur	1962	Census	CBH
Nusa Tenggara Timur	1963	Census	CBH
Nusa Tenggara Timur	1964	Census	CBH
Nusa Tenggara Timur	1965	Census	CBH
Nusa Tenggara Timur	1966	Census	CBH
Nusa Tenggara Timur	1967	Census	CBH
Nusa Tenggara Timur	1968	Census	CBH
Nusa Tenggara Timur	1969	Census	CBH
Nusa Tenggara Timur	1970	Census	CBH
Nusa Tenggara Timur	1971	Census	CBH
Nusa Tenggara Timur	1972	Census	CBH
Nusa Tenggara Timur	1973	Census	CBH
Nusa Tenggara Timur	1974	Census	CBH

Appendix Table 10. Under-5 mortality reference sources by source date and location, 1950-2016			
Location	Source	Source years	Method
Nusa Tenggara Timur	1975	Census	CBH
Nusa Tenggara Timur	1976	Census	CBH
Nusa Tenggara Timur	1977	Census	CBH
Nusa Tenggara Timur	1978	Census	CBH
Nusa Tenggara Timur	1979	Census	CBH
Nusa Tenggara Timur	1980	Census	SBH
Nusa Tenggara Timur	1980	Census	CBH
Nusa Tenggara Timur	1981	Census	CBH
Nusa Tenggara Timur	1982	Census	CBH
Nusa Tenggara Timur	1983	Census	CBH
Nusa Tenggara Timur	1984	Census	CBH
Nusa Tenggara Timur	1985	Census	CBH
Nusa Tenggara Timur	1986	Census	CBH
Nusa Tenggara Timur	1987	Census	CBH
Nusa Tenggara Timur	1988	Census	CBH
Nusa Tenggara Timur	1989	Census	CBH
Nusa Tenggara Timur	1990	Census	SBH
Nusa Tenggara Timur	1990	Census	CBH
Nusa Tenggara Timur	1991	Census	CBH
Nusa Tenggara Timur	1992	Census	CBH
Nusa Tenggara Timur	1993	Census	CBH
Nusa Tenggara Timur	1994	Census	CBH
Nusa Tenggara Timur	1995	Census	CBH
Nusa Tenggara Timur	1996	Census	CBH
Nusa Tenggara Timur	1997	Census	CBH
Nusa Tenggara Timur	1998	Census	CBH
Nusa Tenggara Timur	1999	Census	CBH
Nusa Tenggara Timur	2000	Census	SBH
Nusa Tenggara Timur	2000	Census	CBH
Nusa Tenggara Timur	2002	Census	CBH
Nusa Tenggara Timur	2004	Census	CBH
Nusa Tenggara Timur	2010	Census	SBH
Nusa Tenggara Timur	1973	Demographic and Health Survey (DHS)	CBH
Nusa Tenggara Timur	1975	Demographic and Health Survey (DHS)	CBH
Nusa Tenggara Timur	1977	Demographic and Health Survey (DHS)	CBH
Nusa Tenggara Timur	1979	Demographic and Health Survey (DHS)	CBH
Nusa Tenggara Timur	1981	Demographic and Health Survey (DHS)	CBH
Nusa Tenggara Timur	1983	Demographic and Health Survey (DHS)	CBH
Nusa Tenggara Timur	1985	Demographic and Health Survey (DHS)	CBH
Nusa Tenggara Timur	1987	Demographic and Health Survey (DHS)	CBH
Nusa Tenggara Timur	1989	Demographic and Health Survey (DHS)	CBH
Nusa Tenggara Timur	1991	Demographic and Health Survey (DHS)	SBH
Nusa Tenggara Timur	1991	Demographic and Health Survey (DHS)	CBH
Nusa Tenggara Timur	1993	Demographic and Health Survey (DHS)	CBH
Nusa Tenggara Timur	1994	Demographic and Health Survey (DHS)	SBH
Nusa Tenggara Timur	1995	Demographic and Health Survey (DHS)	CBH
Nusa Tenggara Timur	1997	Demographic and Health Survey (DHS)	SBH
Nusa Tenggara Timur	1997	Demographic and Health Survey (DHS)	CBH
Nusa Tenggara Timur	1999	Demographic and Health Survey (DHS)	CBH
Nusa Tenggara Timur	2001	Demographic and Health Survey (DHS)	CBH
Nusa Tenggara Timur	2003	Demographic and Health Survey (DHS)	SBH
Nusa Tenggara Timur	2003	Demographic and Health Survey (DHS)	CBH
Nusa Tenggara Timur	2005	Demographic and Health Survey (DHS)	CBH
Nusa Tenggara Timur	2007	Demographic and Health Survey (DHS)	SBH
Nusa Tenggara Timur	2007	Demographic and Health Survey (DHS)	CBH
Nusa Tenggara Timur	2009	Demographic and Health Survey (DHS)	CBH
Nusa Tenggara Timur	2011	Demographic and Health Survey (DHS)	CBH
Nusa Tenggara Timur	2012	Demographic and Health Survey (DHS)	SBH
Nusa Tenggara Timur	1985	Health Survey	SBH
Nusa Tenggara Timur	1992	Health Survey	SBH
Nusa Tenggara Timur	1993	Health Survey	SBH
Nusa Tenggara Timur	1994	Health Survey	SBH
Nusa Tenggara Timur	1995	Health Survey	SBH
Nusa Tenggara Timur	1996	Health Survey	SBH
Nusa Tenggara Timur	1997	Health Survey	CBH
Nusa Tenggara Timur	1998	Health Survey	SBH
Nusa Tenggara Timur	1999	Health Survey	SBH

Appendix Table 10. Under-5 mortality reference sources by source date and location, 1950-2016			
**Location**	**Source**	**Source years**	**Method**
Nusa Tenggara Timur	1999	Health Survey	CBH
Nusa Tenggara Timur	2000	Health Survey	SBH
Nusa Tenggara Timur	2001	Health Survey	SBH
Nusa Tenggara Timur	2001	Health Survey	CBH
Nusa Tenggara Timur	2002	Health Survey	SBH
Nusa Tenggara Timur	2003	Health Survey	SBH
Nusa Tenggara Timur	2003	Health Survey	CBH
Nusa Tenggara Timur	2004	Health Survey	SBH
Nusa Tenggara Timur	2005	Health Survey	CBH
Nusa Tenggara Timur	2005	Health Survey	SBH
Nusa Tenggara Timur	2006	Health Survey	SBH
Nusa Tenggara Timur	2007	Health Survey	SBH
Nusa Tenggara Timur	2007	Health Survey	CBH
Nusa Tenggara Timur	2008	Health Survey	SBH
Nusa Tenggara Timur	2009	Health Survey	CBH
Nusa Tenggara Timur	2009	Health Survey	SBH
Nusa Tenggara Timur	2010	Health Survey	SBH
Nusa Tenggara Timur	2011	Health Survey	CBH
Nusa Tenggara Timur	2011	Health Survey	SBH
Nusa Tenggara Timur	2012	Health Survey	SBH
Nusa Tenggara Timur	2013	Health Survey	SBH
Papua	1970	Census	CBH
Papua	1972	Census	CBH
Papua	1974	Census	CBH
Papua	1975	Census	CBH
Papua	1976	Census	CBH
Papua	1977	Census	CBH
Papua	1978	Census	CBH
Papua	1979	Census	CBH
Papua	1980	Census	CBH
Papua	1980	Census	SBH
Papua	1981	Census	CBH
Papua	1982	Census	CBH
Papua	1983	Census	CBH
Papua	1984	Census	CBH
Papua	1985	Census	CBH
Papua	1986	Census	CBH
Papua	1987	Census	CBH
Papua	1988	Census	CBH
Papua	1989	Census	CBH
Papua	1990	Census	CBH
Papua	1991	Census	CBH
Papua	1992	Census	CBH
Papua	1993	Census	CBH
Papua	1994	Census	CBH
Papua	1995	Census	CBH
Papua	1996	Census	CBH
Papua	1997	Census	CBH
Papua	1998	Census	CBH
Papua	1999	Census	CBH
Papua	2000	Census	CBH
Papua	2000	Census	SBH
Papua	2002	Census	CBH
Papua	2004	Census	CBH
Papua	2010	Census	SBH
Papua	1993	Demographic and Health Survey (DHS)	CBH
Papua	1995	Demographic and Health Survey (DHS)	CBH
Papua	1997	Demographic and Health Survey (DHS)	CBH
Papua	1999	Demographic and Health Survey (DHS)	CBH
Papua	2001	Demographic and Health Survey (DHS)	CBH
Papua	2003	Demographic and Health Survey (DHS)	CBH
Papua	2005	Demographic and Health Survey (DHS)	CBH
Papua	2007	Demographic and Health Survey (DHS)	SBH
Papua	2007	Demographic and Health Survey (DHS)	CBH
Papua	2009	Demographic and Health Survey (DHS)	CBH
Papua	2011	Demographic and Health Survey (DHS)	CBH
Papua	2012	Demographic and Health Survey (DHS)	SBH

Appendix Table 10. Under-5 mortality reference sources by source date and location, 1950-2016			
Location	Source	Source years	Method
Papua	1985	Health Survey	SBH
Papua	1992	Health Survey	SBH
Papua	1993	Health Survey	SBH
Papua	1994	Health Survey	SBH
Papua	1995	Health Survey	SBH
Papua	1996	Health Survey	SBH
Papua	1997	Health Survey	CBH
Papua	1998	Health Survey	SBH
Papua	1999	Health Survey	SBH
Papua	1999	Health Survey	CBH
Papua	2000	Health Survey	SBH
Papua	2001	Health Survey	CBH
Papua	2001	Health Survey	SBH
Papua	2003	Health Survey	SBH
Papua	2003	Health Survey	CBH
Papua	2004	Health Survey	SBH
Papua	2005	Health Survey	CBH
Papua	2005	Health Survey	SBH
Papua	2006	Health Survey	SBH
Papua	2007	Health Survey	CBH
Papua	2007	Health Survey	SBH
Papua	2008	Health Survey	SBH
Papua	2009	Health Survey	SBH
Papua	2009	Health Survey	CBH
Papua	2010	Health Survey	SBH
Papua	2011	Health Survey	SBH
Papua	2011	Health Survey	CBH
Papua	2012	Health Survey	SBH
Papua	2013	Health Survey	SBH
Papua Barat	1973	Census	CBH
Papua Barat	1975	Census	CBH
Papua Barat	1976	Census	CBH
Papua Barat	1977	Census	CBH
Papua Barat	1978	Census	CBH
Papua Barat	1979	Census	CBH
Papua Barat	1980	Census	CBH
Papua Barat	1980	Census	SBH
Papua Barat	1981	Census	CBH
Papua Barat	1982	Census	CBH
Papua Barat	1983	Census	CBH
Papua Barat	1984	Census	CBH
Papua Barat	1985	Census	CBH
Papua Barat	1986	Census	CBH
Papua Barat	1987	Census	CBH
Papua Barat	1988	Census	CBH
Papua Barat	1989	Census	CBH
Papua Barat	1990	Census	CBH
Papua Barat	1991	Census	CBH
Papua Barat	1992	Census	CBH
Papua Barat	1993	Census	CBH
Papua Barat	1994	Census	CBH
Papua Barat	1995	Census	CBH
Papua Barat	1996	Census	CBH
Papua Barat	1997	Census	CBH
Papua Barat	1998	Census	CBH
Papua Barat	1999	Census	CBH
Papua Barat	2000	Census	SBH
Papua Barat	2000	Census	CBH
Papua Barat	2002	Census	CBH
Papua Barat	2004	Census	CBH
Papua Barat	2010	Census	SBH
Papua Barat	1991	Demographic and Health Survey (DHS)	CBH
Papua Barat	1993	Demographic and Health Survey (DHS)	CBH
Papua Barat	1995	Demographic and Health Survey (DHS)	CBH
Papua Barat	1997	Demographic and Health Survey (DHS)	CBH
Papua Barat	1999	Demographic and Health Survey (DHS)	CBH
Papua Barat	2001	Demographic and Health Survey (DHS)	CBH

Appendix Table 10. Under-5 mortality reference sources by source date and location, 1950-2016			
**Location**	**Source**	**Source years**	**Method**
Papua Barat	2003	Demographic and Health Survey (DHS)	CBH
Papua Barat	2005	Demographic and Health Survey (DHS)	CBH
Papua Barat	2007	Demographic and Health Survey (DHS)	CBH
Papua Barat	2007	Demographic and Health Survey (DHS)	SBH
Papua Barat	2009	Demographic and Health Survey (DHS)	CBH
Papua Barat	2011	Demographic and Health Survey (DHS)	CBH
Papua Barat	2012	Demographic and Health Survey (DHS)	SBH
Papua Barat	1985	Health Survey	SBH
Papua Barat	1992	Health Survey	SBH
Papua Barat	1993	Health Survey	SBH
Papua Barat	1994	Health Survey	SBH
Papua Barat	1995	Health Survey	SBH
Papua Barat	1996	Health Survey	SBH
Papua Barat	1997	Health Survey	CBH
Papua Barat	1998	Health Survey	SBH
Papua Barat	1999	Health Survey	SBH
Papua Barat	1999	Health Survey	CBH
Papua Barat	2000	Health Survey	SBH
Papua Barat	2001	Health Survey	CBH
Papua Barat	2001	Health Survey	SBH
Papua Barat	2003	Health Survey	CBH
Papua Barat	2003	Health Survey	SBH
Papua Barat	2004	Health Survey	SBH
Papua Barat	2005	Health Survey	CBH
Papua Barat	2005	Health Survey	SBH
Papua Barat	2006	Health Survey	SBH
Papua Barat	2007	Health Survey	CBH
Papua Barat	2007	Health Survey	SBH
Papua Barat	2008	Health Survey	SBH
Papua Barat	2009	Health Survey	SBH
Papua Barat	2009	Health Survey	CBH
Papua Barat	2010	Health Survey	SBH
Papua Barat	2011	Health Survey	CBH
Papua Barat	2011	Health Survey	SBH
Papua Barat	2012	Health Survey	SBH
Papua Barat	2013	Health Survey	SBH
Riau	1964	Census	CBH
Riau	1965	Census	CBH
Riau	1966	Census	CBH
Riau	1967	Census	CBH
Riau	1968	Census	CBH
Riau	1969	Census	CBH
Riau	1970	Census	CBH
Riau	1971	Census	CBH
Riau	1972	Census	CBH
Riau	1973	Census	CBH
Riau	1974	Census	CBH
Riau	1975	Census	CBH
Riau	1976	Census	CBH
Riau	1977	Census	CBH
Riau	1978	Census	CBH
Riau	1979	Census	CBH
Riau	1980	Census	CBH
Riau	1980	Census	SBH
Riau	1981	Census	CBH
Riau	1982	Census	CBH
Riau	1983	Census	CBH
Riau	1984	Census	CBH
Riau	1985	Census	CBH
Riau	1986	Census	CBH
Riau	1987	Census	CBH
Riau	1988	Census	CBH
Riau	1989	Census	CBH
Riau	1990	Census	CBH
Riau	1990	Census	SBH
Riau	1991	Census	CBH
Riau	1992	Census	CBH

Appendix Table 10. Under-5 mortality reference sources by source date and location, 1950-2016			
Location	Source	Source years	Method
Riau	1993	Census	CBH
Riau	1994	Census	CBH
Riau	1995	Census	CBH
Riau	1996	Census	CBH
Riau	1997	Census	CBH
Riau	1998	Census	CBH
Riau	1999	Census	CBH
Riau	2000	Census	CBH
Riau	2000	Census	SBH
Riau	2002	Census	CBH
Riau	2004	Census	CBH
Riau	2010	Census	SBH
Riau	1969	Demographic and Health Survey (DHS)	CBH
Riau	1971	Demographic and Health Survey (DHS)	CBH
Riau	1973	Demographic and Health Survey (DHS)	CBH
Riau	1975	Demographic and Health Survey (DHS)	CBH
Riau	1977	Demographic and Health Survey (DHS)	CBH
Riau	1979	Demographic and Health Survey (DHS)	CBH
Riau	1981	Demographic and Health Survey (DHS)	CBH
Riau	1983	Demographic and Health Survey (DHS)	CBH
Riau	1985	Demographic and Health Survey (DHS)	CBH
Riau	1987	Demographic and Health Survey (DHS)	CBH
Riau	1987	Demographic and Health Survey (DHS)	SBH
Riau	1989	Demographic and Health Survey (DHS)	CBH
Riau	1991	Demographic and Health Survey (DHS)	CBH
Riau	1991	Demographic and Health Survey (DHS)	SBH
Riau	1993	Demographic and Health Survey (DHS)	CBH
Riau	1994	Demographic and Health Survey (DHS)	SBH
Riau	1995	Demographic and Health Survey (DHS)	CBH
Riau	1997	Demographic and Health Survey (DHS)	SBH
Riau	1997	Demographic and Health Survey (DHS)	CBH
Riau	1999	Demographic and Health Survey (DHS)	CBH
Riau	2001	Demographic and Health Survey (DHS)	CBH
Riau	2003	Demographic and Health Survey (DHS)	SBH
Riau	2003	Demographic and Health Survey (DHS)	CBH
Riau	2005	Demographic and Health Survey (DHS)	CBH
Riau	2007	Demographic and Health Survey (DHS)	SBH
Riau	2007	Demographic and Health Survey (DHS)	CBH
Riau	2009	Demographic and Health Survey (DHS)	CBH
Riau	2011	Demographic and Health Survey (DHS)	CBH
Riau	2012	Demographic and Health Survey (DHS)	SBH
Riau	1985	Health Survey	SBH
Riau	1992	Health Survey	CBH
Riau	1992	Health Survey	SBH
Riau	1993	Health Survey	SBH
Riau	1994	Health Survey	CBH
Riau	1994	Health Survey	SBH
Riau	1995	Health Survey	SBH
Riau	1996	Health Survey	SBH
Riau	1996	Health Survey	CBH
Riau	1998	Health Survey	CBH
Riau	1998	Health Survey	SBH
Riau	1999	Health Survey	SBH
Riau	2000	Health Survey	CBH
Riau	2000	Health Survey	SBH
Riau	2001	Health Survey	SBH
Riau	2002	Health Survey	SBH
Riau	2003	Health Survey	SBH
Riau	2004	Health Survey	SBH
Riau	2005	Health Survey	SBH
Riau	2006	Health Survey	SBH
Riau	2007	Health Survey	SBH
Riau	2008	Health Survey	SBH
Riau	2009	Health Survey	SBH
Riau	2010	Health Survey	SBH
Riau	2011	Health Survey	SBH
Riau	2012	Health Survey	SBH

Appendix Table 10. Under-5 mortality reference sources by source date and location, 1950-2016			
**Location**	**Source**	**Source years**	**Method**
Riau	2013	Health Survey	SBH
Sulawesi Barat	1966	Census	CBH
Sulawesi Barat	1967	Census	CBH
Sulawesi Barat	1968	Census	CBH
Sulawesi Barat	1969	Census	CBH
Sulawesi Barat	1970	Census	CBH
Sulawesi Barat	1971	Census	CBH
Sulawesi Barat	1972	Census	CBH
Sulawesi Barat	1973	Census	CBH
Sulawesi Barat	1974	Census	CBH
Sulawesi Barat	1975	Census	CBH
Sulawesi Barat	1976	Census	CBH
Sulawesi Barat	1977	Census	CBH
Sulawesi Barat	1978	Census	CBH
Sulawesi Barat	1979	Census	CBH
Sulawesi Barat	1980	Census	SBH
Sulawesi Barat	1980	Census	CBH
Sulawesi Barat	1981	Census	CBH
Sulawesi Barat	1982	Census	CBH
Sulawesi Barat	1983	Census	CBH
Sulawesi Barat	1984	Census	CBH
Sulawesi Barat	1985	Census	CBH
Sulawesi Barat	1986	Census	CBH
Sulawesi Barat	1987	Census	CBH
Sulawesi Barat	1988	Census	CBH
Sulawesi Barat	1989	Census	CBH
Sulawesi Barat	1990	Census	CBH
Sulawesi Barat	1990	Census	SBH
Sulawesi Barat	1991	Census	CBH
Sulawesi Barat	1992	Census	CBH
Sulawesi Barat	1993	Census	CBH
Sulawesi Barat	1994	Census	CBH
Sulawesi Barat	1995	Census	CBH
Sulawesi Barat	1996	Census	CBH
Sulawesi Barat	1997	Census	CBH
Sulawesi Barat	1998	Census	CBH
Sulawesi Barat	1999	Census	CBH
Sulawesi Barat	2000	Census	CBH
Sulawesi Barat	2000	Census	SBH
Sulawesi Barat	2002	Census	CBH
Sulawesi Barat	2004	Census	CBH
Sulawesi Barat	2010	Census	SBH
Sulawesi Barat	1983	Demographic and Health Survey (DHS)	CBH
Sulawesi Barat	1985	Demographic and Health Survey (DHS)	CBH
Sulawesi Barat	1987	Demographic and Health Survey (DHS)	CBH
Sulawesi Barat	1989	Demographic and Health Survey (DHS)	CBH
Sulawesi Barat	1991	Demographic and Health Survey (DHS)	CBH
Sulawesi Barat	1993	Demographic and Health Survey (DHS)	CBH
Sulawesi Barat	1995	Demographic and Health Survey (DHS)	CBH
Sulawesi Barat	1997	Demographic and Health Survey (DHS)	CBH
Sulawesi Barat	1999	Demographic and Health Survey (DHS)	CBH
Sulawesi Barat	2001	Demographic and Health Survey (DHS)	CBH
Sulawesi Barat	2003	Demographic and Health Survey (DHS)	CBH
Sulawesi Barat	2005	Demographic and Health Survey (DHS)	CBH
Sulawesi Barat	2007	Demographic and Health Survey (DHS)	SBH
Sulawesi Barat	2007	Demographic and Health Survey (DHS)	CBH
Sulawesi Barat	2009	Demographic and Health Survey (DHS)	CBH
Sulawesi Barat	2011	Demographic and Health Survey (DHS)	CBH
Sulawesi Barat	2012	Demographic and Health Survey (DHS)	SBH
Sulawesi Barat	1984	Health Survey	CBH
Sulawesi Barat	1985	Health Survey	SBH
Sulawesi Barat	1986	Health Survey	CBH
Sulawesi Barat	1988	Health Survey	CBH
Sulawesi Barat	1990	Health Survey	CBH
Sulawesi Barat	1992	Health Survey	SBH
Sulawesi Barat	1992	Health Survey	CBH
Sulawesi Barat	1993	Health Survey	SBH

Appendix Table 10. Under-5 mortality reference sources by source date and location, 1950-2016			
**Location**	**Source**	**Source years**	**Method**
Sulawesi Barat	1994	Health Survey	CBH
Sulawesi Barat	1994	Health Survey	SBH
Sulawesi Barat	1995	Health Survey	SBH
Sulawesi Barat	1996	Health Survey	SBH
Sulawesi Barat	1996	Health Survey	CBH
Sulawesi Barat	1998	Health Survey	SBH
Sulawesi Barat	1998	Health Survey	CBH
Sulawesi Barat	1999	Health Survey	SBH
Sulawesi Barat	2000	Health Survey	SBH
Sulawesi Barat	2001	Health Survey	SBH
Sulawesi Barat	2003	Health Survey	SBH
Sulawesi Barat	2004	Health Survey	SBH
Sulawesi Barat	2005	Health Survey	SBH
Sulawesi Barat	2006	Health Survey	SBH
Sulawesi Barat	2007	Health Survey	SBH
Sulawesi Barat	2008	Health Survey	SBH
Sulawesi Barat	2009	Health Survey	SBH
Sulawesi Barat	2010	Health Survey	SBH
Sulawesi Barat	2011	Health Survey	SBH
Sulawesi Barat	2012	Health Survey	SBH
Sulawesi Barat	2013	Health Survey	SBH
Sulawesi Selatan	1949	Census	CBH
Sulawesi Selatan	1951	Census	CBH
Sulawesi Selatan	1952	Census	CBH
Sulawesi Selatan	1953	Census	CBH
Sulawesi Selatan	1954	Census	CBH
Sulawesi Selatan	1955	Census	CBH
Sulawesi Selatan	1956	Census	CBH
Sulawesi Selatan	1957	Census	CBH
Sulawesi Selatan	1958	Census	CBH
Sulawesi Selatan	1959	Census	CBH
Sulawesi Selatan	1960	Census	CBH
Sulawesi Selatan	1961	Census	CBH
Sulawesi Selatan	1962	Census	CBH
Sulawesi Selatan	1963	Census	CBH
Sulawesi Selatan	1964	Census	CBH
Sulawesi Selatan	1965	Census	CBH
Sulawesi Selatan	1966	Census	CBH
Sulawesi Selatan	1967	Census	CBH
Sulawesi Selatan	1968	Census	CBH
Sulawesi Selatan	1969	Census	CBH
Sulawesi Selatan	1970	Census	CBH
Sulawesi Selatan	1971	Census	CBH
Sulawesi Selatan	1972	Census	CBH
Sulawesi Selatan	1973	Census	CBH
Sulawesi Selatan	1974	Census	CBH
Sulawesi Selatan	1975	Census	CBH
Sulawesi Selatan	1976	Census	CBH
Sulawesi Selatan	1977	Census	CBH
Sulawesi Selatan	1978	Census	CBH
Sulawesi Selatan	1979	Census	CBH
Sulawesi Selatan	1980	Census	CBH
Sulawesi Selatan	1980	Census	SBH
Sulawesi Selatan	1981	Census	CBH
Sulawesi Selatan	1982	Census	CBH
Sulawesi Selatan	1983	Census	CBH
Sulawesi Selatan	1984	Census	CBH
Sulawesi Selatan	1985	Census	CBH
Sulawesi Selatan	1986	Census	CBH
Sulawesi Selatan	1987	Census	CBH
Sulawesi Selatan	1988	Census	CBH
Sulawesi Selatan	1989	Census	CBH
Sulawesi Selatan	1990	Census	SBH
Sulawesi Selatan	1990	Census	CBH
Sulawesi Selatan	1991	Census	CBH
Sulawesi Selatan	1992	Census	CBH
Sulawesi Selatan	1993	Census	CBH

Appendix Table 10. Under-5 mortality reference sources by source date and location, 1950-2016			
**Location**	**Source**	**Source years**	**Method**
Sulawesi Selatan	1994	Census	CBH
Sulawesi Selatan	1995	Census	CBH
Sulawesi Selatan	1996	Census	CBH
Sulawesi Selatan	1997	Census	CBH
Sulawesi Selatan	1998	Census	CBH
Sulawesi Selatan	1999	Census	CBH
Sulawesi Selatan	2000	Census	SBH
Sulawesi Selatan	2000	Census	CBH
Sulawesi Selatan	2002	Census	CBH
Sulawesi Selatan	2004	Census	CBH
Sulawesi Selatan	2010	Census	SBH
Sulawesi Selatan	1967	Demographic and Health Survey (DHS)	CBH
Sulawesi Selatan	1969	Demographic and Health Survey (DHS)	CBH
Sulawesi Selatan	1971	Demographic and Health Survey (DHS)	CBH
Sulawesi Selatan	1973	Demographic and Health Survey (DHS)	CBH
Sulawesi Selatan	1975	Demographic and Health Survey (DHS)	CBH
Sulawesi Selatan	1977	Demographic and Health Survey (DHS)	CBH
Sulawesi Selatan	1979	Demographic and Health Survey (DHS)	CBH
Sulawesi Selatan	1981	Demographic and Health Survey (DHS)	CBH
Sulawesi Selatan	1983	Demographic and Health Survey (DHS)	CBH
Sulawesi Selatan	1985	Demographic and Health Survey (DHS)	CBH
Sulawesi Selatan	1987	Demographic and Health Survey (DHS)	SBH
Sulawesi Selatan	1987	Demographic and Health Survey (DHS)	CBH
Sulawesi Selatan	1989	Demographic and Health Survey (DHS)	CBH
Sulawesi Selatan	1991	Demographic and Health Survey (DHS)	CBH
Sulawesi Selatan	1991	Demographic and Health Survey (DHS)	SBH
Sulawesi Selatan	1993	Demographic and Health Survey (DHS)	CBH
Sulawesi Selatan	1994	Demographic and Health Survey (DHS)	SBH
Sulawesi Selatan	1995	Demographic and Health Survey (DHS)	CBH
Sulawesi Selatan	1997	Demographic and Health Survey (DHS)	CBH
Sulawesi Selatan	1997	Demographic and Health Survey (DHS)	SBH
Sulawesi Selatan	1999	Demographic and Health Survey (DHS)	CBH
Sulawesi Selatan	2001	Demographic and Health Survey (DHS)	CBH
Sulawesi Selatan	2003	Demographic and Health Survey (DHS)	CBH
Sulawesi Selatan	2003	Demographic and Health Survey (DHS)	SBH
Sulawesi Selatan	2005	Demographic and Health Survey (DHS)	CBH
Sulawesi Selatan	2007	Demographic and Health Survey (DHS)	SBH
Sulawesi Selatan	2007	Demographic and Health Survey (DHS)	CBH
Sulawesi Selatan	2009	Demographic and Health Survey (DHS)	CBH
Sulawesi Selatan	2011	Demographic and Health Survey (DHS)	CBH
Sulawesi Selatan	2012	Demographic and Health Survey (DHS)	SBH
Sulawesi Selatan	1978	Health Survey	CBH
Sulawesi Selatan	1980	Health Survey	CBH
Sulawesi Selatan	1982	Health Survey	CBH
Sulawesi Selatan	1984	Health Survey	CBH
Sulawesi Selatan	1985	Health Survey	SBH
Sulawesi Selatan	1986	Health Survey	CBH
Sulawesi Selatan	1988	Health Survey	CBH
Sulawesi Selatan	1990	Health Survey	CBH
Sulawesi Selatan	1992	Health Survey	SBH
Sulawesi Selatan	1992	Health Survey	CBH
Sulawesi Selatan	1993	Health Survey	SBH
Sulawesi Selatan	1994	Health Survey	CBH
Sulawesi Selatan	1994	Health Survey	SBH
Sulawesi Selatan	1995	Health Survey	SBH
Sulawesi Selatan	1996	Health Survey	SBH
Sulawesi Selatan	1996	Health Survey	CBH
Sulawesi Selatan	1997	Health Survey	SBH
Sulawesi Selatan	1998	Health Survey	CBH
Sulawesi Selatan	1998	Health Survey	SBH
Sulawesi Selatan	1999	Health Survey	SBH
Sulawesi Selatan	2000	Health Survey	SBH
Sulawesi Selatan	2000	Health Survey	CBH
Sulawesi Selatan	2001	Health Survey	SBH
Sulawesi Selatan	2002	Health Survey	SBH
Sulawesi Selatan	2003	Health Survey	SBH
Sulawesi Selatan	2004	Health Survey	SBH

Appendix Table 10. Under-5 mortality reference sources by source date and location, 1950-2016			
**Location**	**Source**	**Source years**	**Method**
Sulawesi Selatan	2005	Health Survey	SBH
Sulawesi Selatan	2006	Health Survey	SBH
Sulawesi Selatan	2007	Health Survey	SBH
Sulawesi Selatan	2008	Health Survey	SBH
Sulawesi Selatan	2009	Health Survey	SBH
Sulawesi Selatan	2010	Health Survey	SBH
Sulawesi Selatan	2011	Health Survey	SBH
Sulawesi Selatan	2012	Health Survey	SBH
Sulawesi Selatan	2013	Health Survey	SBH
Sulawesi Tengah	1963	Census	CBH
Sulawesi Tengah	1964	Census	CBH
Sulawesi Tengah	1965	Census	CBH
Sulawesi Tengah	1966	Census	CBH
Sulawesi Tengah	1967	Census	CBH
Sulawesi Tengah	1968	Census	CBH
Sulawesi Tengah	1969	Census	CBH
Sulawesi Tengah	1970	Census	CBH
Sulawesi Tengah	1971	Census	CBH
Sulawesi Tengah	1972	Census	CBH
Sulawesi Tengah	1973	Census	CBH
Sulawesi Tengah	1974	Census	CBH
Sulawesi Tengah	1975	Census	CBH
Sulawesi Tengah	1976	Census	CBH
Sulawesi Tengah	1977	Census	CBH
Sulawesi Tengah	1978	Census	CBH
Sulawesi Tengah	1979	Census	CBH
Sulawesi Tengah	1980	Census	CBH
Sulawesi Tengah	1980	Census	SBH
Sulawesi Tengah	1981	Census	CBH
Sulawesi Tengah	1982	Census	CBH
Sulawesi Tengah	1983	Census	CBH
Sulawesi Tengah	1984	Census	CBH
Sulawesi Tengah	1985	Census	CBH
Sulawesi Tengah	1986	Census	CBH
Sulawesi Tengah	1987	Census	CBH
Sulawesi Tengah	1988	Census	CBH
Sulawesi Tengah	1989	Census	CBH
Sulawesi Tengah	1990	Census	SBH
Sulawesi Tengah	1990	Census	CBH
Sulawesi Tengah	1991	Census	CBH
Sulawesi Tengah	1992	Census	CBH
Sulawesi Tengah	1993	Census	CBH
Sulawesi Tengah	1994	Census	CBH
Sulawesi Tengah	1995	Census	CBH
Sulawesi Tengah	1996	Census	CBH
Sulawesi Tengah	1997	Census	CBH
Sulawesi Tengah	1998	Census	CBH
Sulawesi Tengah	1999	Census	CBH
Sulawesi Tengah	2000	Census	SBH
Sulawesi Tengah	2000	Census	CBH
Sulawesi Tengah	2002	Census	CBH
Sulawesi Tengah	2004	Census	CBH
Sulawesi Tengah	2010	Census	SBH
Sulawesi Tengah	1971	Demographic and Health Survey (DHS)	CBH
Sulawesi Tengah	1973	Demographic and Health Survey (DHS)	CBH
Sulawesi Tengah	1975	Demographic and Health Survey (DHS)	CBH
Sulawesi Tengah	1977	Demographic and Health Survey (DHS)	CBH
Sulawesi Tengah	1979	Demographic and Health Survey (DHS)	CBH
Sulawesi Tengah	1981	Demographic and Health Survey (DHS)	CBH
Sulawesi Tengah	1983	Demographic and Health Survey (DHS)	CBH
Sulawesi Tengah	1985	Demographic and Health Survey (DHS)	CBH
Sulawesi Tengah	1987	Demographic and Health Survey (DHS)	CBH
Sulawesi Tengah	1987	Demographic and Health Survey (DHS)	SBH
Sulawesi Tengah	1989	Demographic and Health Survey (DHS)	CBH
Sulawesi Tengah	1991	Demographic and Health Survey (DHS)	CBH
Sulawesi Tengah	1991	Demographic and Health Survey (DHS)	SBH
Sulawesi Tengah	1993	Demographic and Health Survey (DHS)	CBH

Appendix Table 10. Under-5 mortality reference sources by source date and location, 1950-2016			
**Location**	**Source**	**Source years**	**Method**
Sulawesi Tengah	1994	Demographic and Health Survey (DHS)	SBH
Sulawesi Tengah	1995	Demographic and Health Survey (DHS)	CBH
Sulawesi Tengah	1997	Demographic and Health Survey (DHS)	SBH
Sulawesi Tengah	1997	Demographic and Health Survey (DHS)	CBH
Sulawesi Tengah	1999	Demographic and Health Survey (DHS)	CBH
Sulawesi Tengah	2001	Demographic and Health Survey (DHS)	CBH
Sulawesi Tengah	2003	Demographic and Health Survey (DHS)	SBH
Sulawesi Tengah	2003	Demographic and Health Survey (DHS)	CBH
Sulawesi Tengah	2005	Demographic and Health Survey (DHS)	CBH
Sulawesi Tengah	2007	Demographic and Health Survey (DHS)	SBH
Sulawesi Tengah	2007	Demographic and Health Survey (DHS)	CBH
Sulawesi Tengah	2009	Demographic and Health Survey (DHS)	CBH
Sulawesi Tengah	2011	Demographic and Health Survey (DHS)	CBH
Sulawesi Tengah	2012	Demographic and Health Survey (DHS)	SBH
Sulawesi Tengah	1985	Health Survey	SBH
Sulawesi Tengah	1992	Health Survey	SBH
Sulawesi Tengah	1993	Health Survey	SBH
Sulawesi Tengah	1994	Health Survey	SBH
Sulawesi Tengah	1995	Health Survey	SBH
Sulawesi Tengah	1996	Health Survey	SBH
Sulawesi Tengah	1998	Health Survey	SBH
Sulawesi Tengah	1999	Health Survey	SBH
Sulawesi Tengah	2000	Health Survey	SBH
Sulawesi Tengah	2001	Health Survey	SBH
Sulawesi Tengah	2002	Health Survey	SBH
Sulawesi Tengah	2003	Health Survey	SBH
Sulawesi Tengah	2004	Health Survey	SBH
Sulawesi Tengah	2005	Health Survey	SBH
Sulawesi Tengah	2006	Health Survey	SBH
Sulawesi Tengah	2007	Health Survey	SBH
Sulawesi Tengah	2008	Health Survey	SBH
Sulawesi Tengah	2009	Health Survey	SBH
Sulawesi Tengah	2010	Health Survey	SBH
Sulawesi Tengah	2011	Health Survey	SBH
Sulawesi Tengah	2012	Health Survey	SBH
Sulawesi Tengah	2013	Health Survey	SBH
Sulawesi Tenggara	1962	Census	CBH
Sulawesi Tenggara	1964	Census	CBH
Sulawesi Tenggara	1966	Census	CBH
Sulawesi Tenggara	1967	Census	CBH
Sulawesi Tenggara	1968	Census	CBH
Sulawesi Tenggara	1969	Census	CBH
Sulawesi Tenggara	1970	Census	CBH
Sulawesi Tenggara	1971	Census	CBH
Sulawesi Tenggara	1972	Census	CBH
Sulawesi Tenggara	1973	Census	CBH
Sulawesi Tenggara	1974	Census	CBH
Sulawesi Tenggara	1975	Census	CBH
Sulawesi Tenggara	1976	Census	CBH
Sulawesi Tenggara	1977	Census	CBH
Sulawesi Tenggara	1978	Census	CBH
Sulawesi Tenggara	1979	Census	CBH
Sulawesi Tenggara	1980	Census	SBH
Sulawesi Tenggara	1980	Census	CBH
Sulawesi Tenggara	1981	Census	CBH
Sulawesi Tenggara	1982	Census	CBH
Sulawesi Tenggara	1983	Census	CBH
Sulawesi Tenggara	1984	Census	CBH
Sulawesi Tenggara	1985	Census	CBH
Sulawesi Tenggara	1986	Census	CBH
Sulawesi Tenggara	1987	Census	CBH
Sulawesi Tenggara	1988	Census	CBH
Sulawesi Tenggara	1989	Census	CBH
Sulawesi Tenggara	1990	Census	SBH
Sulawesi Tenggara	1990	Census	CBH
Sulawesi Tenggara	1991	Census	CBH
Sulawesi Tenggara	1992	Census	CBH

Appendix Table 10. Under-5 mortality reference sources by source date and location, 1950-2016			
**Location**	**Source**	**Source years**	**Method**
Sulawesi Tenggara	1993	Census	CBH
Sulawesi Tenggara	1994	Census	CBH
Sulawesi Tenggara	1995	Census	CBH
Sulawesi Tenggara	1996	Census	CBH
Sulawesi Tenggara	1997	Census	CBH
Sulawesi Tenggara	1998	Census	CBH
Sulawesi Tenggara	1999	Census	CBH
Sulawesi Tenggara	2000	Census	CBH
Sulawesi Tenggara	2000	Census	SBH
Sulawesi Tenggara	2002	Census	CBH
Sulawesi Tenggara	2004	Census	CBH
Sulawesi Tenggara	2010	Census	SBH
Sulawesi Tenggara	1973	Demographic and Health Survey (DHS)	CBH
Sulawesi Tenggara	1975	Demographic and Health Survey (DHS)	CBH
Sulawesi Tenggara	1977	Demographic and Health Survey (DHS)	CBH
Sulawesi Tenggara	1979	Demographic and Health Survey (DHS)	CBH
Sulawesi Tenggara	1981	Demographic and Health Survey (DHS)	CBH
Sulawesi Tenggara	1983	Demographic and Health Survey (DHS)	CBH
Sulawesi Tenggara	1985	Demographic and Health Survey (DHS)	CBH
Sulawesi Tenggara	1987	Demographic and Health Survey (DHS)	SBH
Sulawesi Tenggara	1987	Demographic and Health Survey (DHS)	CBH
Sulawesi Tenggara	1989	Demographic and Health Survey (DHS)	CBH
Sulawesi Tenggara	1991	Demographic and Health Survey (DHS)	CBH
Sulawesi Tenggara	1991	Demographic and Health Survey (DHS)	SBH
Sulawesi Tenggara	1993	Demographic and Health Survey (DHS)	CBH
Sulawesi Tenggara	1994	Demographic and Health Survey (DHS)	SBH
Sulawesi Tenggara	1995	Demographic and Health Survey (DHS)	CBH
Sulawesi Tenggara	1997	Demographic and Health Survey (DHS)	SBH
Sulawesi Tenggara	1997	Demographic and Health Survey (DHS)	CBH
Sulawesi Tenggara	1999	Demographic and Health Survey (DHS)	CBH
Sulawesi Tenggara	2001	Demographic and Health Survey (DHS)	CBH
Sulawesi Tenggara	2003	Demographic and Health Survey (DHS)	SBH
Sulawesi Tenggara	2003	Demographic and Health Survey (DHS)	CBH
Sulawesi Tenggara	2005	Demographic and Health Survey (DHS)	CBH
Sulawesi Tenggara	2007	Demographic and Health Survey (DHS)	CBH
Sulawesi Tenggara	2007	Demographic and Health Survey (DHS)	SBH
Sulawesi Tenggara	2009	Demographic and Health Survey (DHS)	CBH
Sulawesi Tenggara	2011	Demographic and Health Survey (DHS)	CBH
Sulawesi Tenggara	2012	Demographic and Health Survey (DHS)	SBH
Sulawesi Tenggara	1985	Health Survey	SBH
Sulawesi Tenggara	1992	Health Survey	SBH
Sulawesi Tenggara	1993	Health Survey	SBH
Sulawesi Tenggara	1994	Health Survey	SBH
Sulawesi Tenggara	1995	Health Survey	CBH
Sulawesi Tenggara	1995	Health Survey	SBH
Sulawesi Tenggara	1996	Health Survey	SBH
Sulawesi Tenggara	1997	Health Survey	CBH
Sulawesi Tenggara	1998	Health Survey	SBH
Sulawesi Tenggara	1999	Health Survey	CBH
Sulawesi Tenggara	1999	Health Survey	SBH
Sulawesi Tenggara	2000	Health Survey	SBH
Sulawesi Tenggara	2001	Health Survey	SBH
Sulawesi Tenggara	2001	Health Survey	CBH
Sulawesi Tenggara	2002	Health Survey	SBH
Sulawesi Tenggara	2003	Health Survey	CBH
Sulawesi Tenggara	2003	Health Survey	SBH
Sulawesi Tenggara	2004	Health Survey	SBH
Sulawesi Tenggara	2005	Health Survey	SBH
Sulawesi Tenggara	2005	Health Survey	CBH
Sulawesi Tenggara	2006	Health Survey	SBH
Sulawesi Tenggara	2007	Health Survey	CBH
Sulawesi Tenggara	2007	Health Survey	SBH
Sulawesi Tenggara	2008	Health Survey	SBH
Sulawesi Tenggara	2009	Health Survey	CBH
Sulawesi Tenggara	2009	Health Survey	SBH
Sulawesi Tenggara	2010	Health Survey	SBH
Sulawesi Tenggara	2011	Health Survey	CBH

Appendix Table 10. Under-5 mortality reference sources by source date and location, 1950-2016			
Location	Source	Source years	Method
Sulawesi Tenggara	2011	Health Survey	SBH
Sulawesi Tenggara	2012	Health Survey	SBH
Sulawesi Tenggara	2013	Health Survey	SBH
Sulawesi Utara	1960	Census	CBH
Sulawesi Utara	1962	Census	CBH
Sulawesi Utara	1964	Census	CBH
Sulawesi Utara	1965	Census	CBH
Sulawesi Utara	1966	Census	CBH
Sulawesi Utara	1967	Census	CBH
Sulawesi Utara	1968	Census	CBH
Sulawesi Utara	1969	Census	CBH
Sulawesi Utara	1970	Census	CBH
Sulawesi Utara	1971	Census	CBH
Sulawesi Utara	1972	Census	CBH
Sulawesi Utara	1973	Census	CBH
Sulawesi Utara	1974	Census	CBH
Sulawesi Utara	1975	Census	CBH
Sulawesi Utara	1976	Census	CBH
Sulawesi Utara	1977	Census	CBH
Sulawesi Utara	1978	Census	CBH
Sulawesi Utara	1979	Census	CBH
Sulawesi Utara	1980	Census	SBH
Sulawesi Utara	1980	Census	CBH
Sulawesi Utara	1981	Census	CBH
Sulawesi Utara	1982	Census	CBH
Sulawesi Utara	1983	Census	CBH
Sulawesi Utara	1984	Census	CBH
Sulawesi Utara	1985	Census	CBH
Sulawesi Utara	1986	Census	CBH
Sulawesi Utara	1987	Census	CBH
Sulawesi Utara	1988	Census	CBH
Sulawesi Utara	1989	Census	CBH
Sulawesi Utara	1990	Census	SBH
Sulawesi Utara	1990	Census	CBH
Sulawesi Utara	1991	Census	CBH
Sulawesi Utara	1992	Census	CBH
Sulawesi Utara	1993	Census	CBH
Sulawesi Utara	1994	Census	CBH
Sulawesi Utara	1995	Census	CBH
Sulawesi Utara	1996	Census	CBH
Sulawesi Utara	1997	Census	CBH
Sulawesi Utara	1998	Census	CBH
Sulawesi Utara	1999	Census	CBH
Sulawesi Utara	2000	Census	SBH
Sulawesi Utara	2000	Census	CBH
Sulawesi Utara	2002	Census	CBH
Sulawesi Utara	2004	Census	CBH
Sulawesi Utara	2010	Census	SBH
Sulawesi Utara	1973	Demographic and Health Survey (DHS)	CBH
Sulawesi Utara	1975	Demographic and Health Survey (DHS)	CBH
Sulawesi Utara	1977	Demographic and Health Survey (DHS)	CBH
Sulawesi Utara	1979	Demographic and Health Survey (DHS)	CBH
Sulawesi Utara	1981	Demographic and Health Survey (DHS)	CBH
Sulawesi Utara	1983	Demographic and Health Survey (DHS)	CBH
Sulawesi Utara	1985	Demographic and Health Survey (DHS)	CBH
Sulawesi Utara	1987	Demographic and Health Survey (DHS)	CBH
Sulawesi Utara	1987	Demographic and Health Survey (DHS)	SBH
Sulawesi Utara	1989	Demographic and Health Survey (DHS)	CBH
Sulawesi Utara	1991	Demographic and Health Survey (DHS)	CBH
Sulawesi Utara	1991	Demographic and Health Survey (DHS)	SBH
Sulawesi Utara	1993	Demographic and Health Survey (DHS)	CBH
Sulawesi Utara	1994	Demographic and Health Survey (DHS)	SBH
Sulawesi Utara	1995	Demographic and Health Survey (DHS)	CBH
Sulawesi Utara	1997	Demographic and Health Survey (DHS)	CBH
Sulawesi Utara	1997	Demographic and Health Survey (DHS)	SBH
Sulawesi Utara	1999	Demographic and Health Survey (DHS)	CBH
Sulawesi Utara	2001	Demographic and Health Survey (DHS)	CBH

Appendix Table 10. Under-5 mortality reference sources by source date and location, 1950-2016			
**Location**	**Source**	**Source years**	**Method**
Sulawesi Utara	2003	Demographic and Health Survey (DHS)	CBH
Sulawesi Utara	2003	Demographic and Health Survey (DHS)	SBH
Sulawesi Utara	2005	Demographic and Health Survey (DHS)	CBH
Sulawesi Utara	2007	Demographic and Health Survey (DHS)	CBH
Sulawesi Utara	2007	Demographic and Health Survey (DHS)	SBH
Sulawesi Utara	2009	Demographic and Health Survey (DHS)	CBH
Sulawesi Utara	2011	Demographic and Health Survey (DHS)	CBH
Sulawesi Utara	2012	Demographic and Health Survey (DHS)	SBH
Sulawesi Utara	1985	Health Survey	SBH
Sulawesi Utara	1992	Health Survey	SBH
Sulawesi Utara	1993	Health Survey	SBH
Sulawesi Utara	1994	Health Survey	SBH
Sulawesi Utara	1995	Health Survey	SBH
Sulawesi Utara	1996	Health Survey	SBH
Sulawesi Utara	1998	Health Survey	SBH
Sulawesi Utara	1999	Health Survey	SBH
Sulawesi Utara	2000	Health Survey	SBH
Sulawesi Utara	2001	Health Survey	SBH
Sulawesi Utara	2002	Health Survey	SBH
Sulawesi Utara	2003	Health Survey	SBH
Sulawesi Utara	2004	Health Survey	SBH
Sulawesi Utara	2005	Health Survey	SBH
Sulawesi Utara	2006	Health Survey	SBH
Sulawesi Utara	2007	Health Survey	SBH
Sulawesi Utara	2008	Health Survey	SBH
Sulawesi Utara	2009	Health Survey	SBH
Sulawesi Utara	2010	Health Survey	SBH
Sulawesi Utara	2011	Health Survey	SBH
Sulawesi Utara	2012	Health Survey	SBH
Sulawesi Utara	2013	Health Survey	SBH
Sumatera Barat	1952	Census	CBH
Sumatera Barat	1953	Census	CBH
Sumatera Barat	1954	Census	CBH
Sumatera Barat	1955	Census	CBH
Sumatera Barat	1956	Census	CBH
Sumatera Barat	1957	Census	CBH
Sumatera Barat	1958	Census	CBH
Sumatera Barat	1959	Census	CBH
Sumatera Barat	1960	Census	CBH
Sumatera Barat	1961	Census	CBH
Sumatera Barat	1962	Census	CBH
Sumatera Barat	1963	Census	CBH
Sumatera Barat	1964	Census	CBH
Sumatera Barat	1965	Census	CBH
Sumatera Barat	1966	Census	CBH
Sumatera Barat	1967	Census	CBH
Sumatera Barat	1968	Census	CBH
Sumatera Barat	1969	Census	CBH
Sumatera Barat	1970	Census	CBH
Sumatera Barat	1971	Census	CBH
Sumatera Barat	1972	Census	CBH
Sumatera Barat	1973	Census	CBH
Sumatera Barat	1974	Census	CBH
Sumatera Barat	1975	Census	CBH
Sumatera Barat	1976	Census	CBH
Sumatera Barat	1977	Census	CBH
Sumatera Barat	1978	Census	CBH
Sumatera Barat	1979	Census	CBH
Sumatera Barat	1980	Census	CBH
Sumatera Barat	1980	Census	SBH
Sumatera Barat	1981	Census	CBH
Sumatera Barat	1982	Census	CBH
Sumatera Barat	1983	Census	CBH
Sumatera Barat	1984	Census	CBH
Sumatera Barat	1985	Census	CBH
Sumatera Barat	1986	Census	CBH
Sumatera Barat	1987	Census	CBH

Appendix Table 10. Under-5 mortality reference sources by source date and location, 1950-2016

Location	Source	Source years	Method
Sumatera Barat	1988	Census	CBH
Sumatera Barat	1989	Census	CBH
Sumatera Barat	1990	Census	CBH
Sumatera Barat	1990	Census	SBH
Sumatera Barat	1991	Census	CBH
Sumatera Barat	1992	Census	CBH
Sumatera Barat	1993	Census	CBH
Sumatera Barat	1994	Census	CBH
Sumatera Barat	1995	Census	CBH
Sumatera Barat	1996	Census	CBH
Sumatera Barat	1997	Census	CBH
Sumatera Barat	1998	Census	CBH
Sumatera Barat	1999	Census	CBH
Sumatera Barat	2000	Census	SBH
Sumatera Barat	2000	Census	CBH
Sumatera Barat	2002	Census	CBH
Sumatera Barat	2004	Census	CBH
Sumatera Barat	2010	Census	SBH
Sumatera Barat	1969	Demographic and Health Survey (DHS)	CBH
Sumatera Barat	1971	Demographic and Health Survey (DHS)	CBH
Sumatera Barat	1973	Demographic and Health Survey (DHS)	CBH
Sumatera Barat	1975	Demographic and Health Survey (DHS)	CBH
Sumatera Barat	1977	Demographic and Health Survey (DHS)	CBH
Sumatera Barat	1979	Demographic and Health Survey (DHS)	CBH
Sumatera Barat	1981	Demographic and Health Survey (DHS)	CBH
Sumatera Barat	1983	Demographic and Health Survey (DHS)	CBH
Sumatera Barat	1985	Demographic and Health Survey (DHS)	CBH
Sumatera Barat	1987	Demographic and Health Survey (DHS)	SBH
Sumatera Barat	1987	Demographic and Health Survey (DHS)	CBH
Sumatera Barat	1989	Demographic and Health Survey (DHS)	CBH
Sumatera Barat	1991	Demographic and Health Survey (DHS)	SBH
Sumatera Barat	1991	Demographic and Health Survey (DHS)	CBH
Sumatera Barat	1993	Demographic and Health Survey (DHS)	CBH
Sumatera Barat	1994	Demographic and Health Survey (DHS)	SBH
Sumatera Barat	1995	Demographic and Health Survey (DHS)	CBH
Sumatera Barat	1997	Demographic and Health Survey (DHS)	CBH
Sumatera Barat	1997	Demographic and Health Survey (DHS)	SBH
Sumatera Barat	1999	Demographic and Health Survey (DHS)	CBH
Sumatera Barat	2001	Demographic and Health Survey (DHS)	CBH
Sumatera Barat	2003	Demographic and Health Survey (DHS)	SBH
Sumatera Barat	2003	Demographic and Health Survey (DHS)	CBH
Sumatera Barat	2005	Demographic and Health Survey (DHS)	CBH
Sumatera Barat	2007	Demographic and Health Survey (DHS)	CBH
Sumatera Barat	2007	Demographic and Health Survey (DHS)	SBH
Sumatera Barat	2009	Demographic and Health Survey (DHS)	CBH
Sumatera Barat	2011	Demographic and Health Survey (DHS)	CBH
Sumatera Barat	2012	Demographic and Health Survey (DHS)	SBH
Sumatera Barat	1982	Health Survey	CBH
Sumatera Barat	1984	Health Survey	CBH
Sumatera Barat	1985	Health Survey	SBH
Sumatera Barat	1986	Health Survey	CBH
Sumatera Barat	1988	Health Survey	CBH
Sumatera Barat	1990	Health Survey	CBH
Sumatera Barat	1992	Health Survey	CBH
Sumatera Barat	1992	Health Survey	SBH
Sumatera Barat	1993	Health Survey	SBH
Sumatera Barat	1994	Health Survey	SBH
Sumatera Barat	1994	Health Survey	CBH
Sumatera Barat	1995	Health Survey	SBH
Sumatera Barat	1996	Health Survey	SBH
Sumatera Barat	1996	Health Survey	CBH
Sumatera Barat	1997	Health Survey	SBH
Sumatera Barat	1998	Health Survey	SBH
Sumatera Barat	1998	Health Survey	CBH
Sumatera Barat	1999	Health Survey	SBH
Sumatera Barat	2000	Health Survey	CBH
Sumatera Barat	2000	Health Survey	SBH

Appendix Table 10. Under-5 mortality reference sources by source date and location, 1950-2016			
Location	Source	Source years	Method
Sumatera Barat	2001	Health Survey	SBH
Sumatera Barat	2002	Health Survey	SBH
Sumatera Barat	2003	Health Survey	SBH
Sumatera Barat	2004	Health Survey	SBH
Sumatera Barat	2005	Health Survey	SBH
Sumatera Barat	2006	Health Survey	SBH
Sumatera Barat	2007	Health Survey	SBH
Sumatera Barat	2008	Health Survey	SBH
Sumatera Barat	2009	Health Survey	SBH
Sumatera Barat	2010	Health Survey	SBH
Sumatera Barat	2011	Health Survey	SBH
Sumatera Barat	2012	Health Survey	SBH
Sumatera Barat	2013	Health Survey	SBH
Sumatera Selatan	1960	Census	CBH
Sumatera Selatan	1961	Census	CBH
Sumatera Selatan	1962	Census	CBH
Sumatera Selatan	1963	Census	CBH
Sumatera Selatan	1964	Census	CBH
Sumatera Selatan	1965	Census	CBH
Sumatera Selatan	1966	Census	CBH
Sumatera Selatan	1967	Census	CBH
Sumatera Selatan	1968	Census	CBH
Sumatera Selatan	1969	Census	CBH
Sumatera Selatan	1970	Census	CBH
Sumatera Selatan	1971	Census	CBH
Sumatera Selatan	1972	Census	CBH
Sumatera Selatan	1973	Census	CBH
Sumatera Selatan	1974	Census	CBH
Sumatera Selatan	1975	Census	CBH
Sumatera Selatan	1976	Census	CBH
Sumatera Selatan	1977	Census	CBH
Sumatera Selatan	1978	Census	CBH
Sumatera Selatan	1979	Census	CBH
Sumatera Selatan	1980	Census	CBH
Sumatera Selatan	1980	Census	SBH
Sumatera Selatan	1981	Census	CBH
Sumatera Selatan	1982	Census	CBH
Sumatera Selatan	1983	Census	CBH
Sumatera Selatan	1984	Census	CBH
Sumatera Selatan	1985	Census	CBH
Sumatera Selatan	1986	Census	CBH
Sumatera Selatan	1987	Census	CBH
Sumatera Selatan	1988	Census	CBH
Sumatera Selatan	1989	Census	CBH
Sumatera Selatan	1990	Census	SBH
Sumatera Selatan	1990	Census	CBH
Sumatera Selatan	1991	Census	CBH
Sumatera Selatan	1992	Census	CBH
Sumatera Selatan	1993	Census	CBH
Sumatera Selatan	1994	Census	CBH
Sumatera Selatan	1995	Census	CBH
Sumatera Selatan	1996	Census	CBH
Sumatera Selatan	1997	Census	CBH
Sumatera Selatan	1998	Census	CBH
Sumatera Selatan	1999	Census	CBH
Sumatera Selatan	2000	Census	CBH
Sumatera Selatan	2000	Census	SBH
Sumatera Selatan	2002	Census	CBH
Sumatera Selatan	2004	Census	CBH
Sumatera Selatan	2010	Census	SBH
Sumatera Selatan	1967	Demographic and Health Survey (DHS)	CBH
Sumatera Selatan	1969	Demographic and Health Survey (DHS)	CBH
Sumatera Selatan	1971	Demographic and Health Survey (DHS)	CBH
Sumatera Selatan	1973	Demographic and Health Survey (DHS)	CBH
Sumatera Selatan	1975	Demographic and Health Survey (DHS)	CBH
Sumatera Selatan	1977	Demographic and Health Survey (DHS)	CBH
Sumatera Selatan	1979	Demographic and Health Survey (DHS)	CBH

Appendix Table 10. Under-5 mortality reference sources by source date and location, 1950-2016

Location	Source	Source years	Method
Sumatera Selatan	1981	Demographic and Health Survey (DHS)	CBH
Sumatera Selatan	1983	Demographic and Health Survey (DHS)	CBH
Sumatera Selatan	1985	Demographic and Health Survey (DHS)	CBH
Sumatera Selatan	1987	Demographic and Health Survey (DHS)	CBH
Sumatera Selatan	1987	Demographic and Health Survey (DHS)	SBH
Sumatera Selatan	1989	Demographic and Health Survey (DHS)	CBH
Sumatera Selatan	1991	Demographic and Health Survey (DHS)	CBH
Sumatera Selatan	1991	Demographic and Health Survey (DHS)	SBH
Sumatera Selatan	1993	Demographic and Health Survey (DHS)	CBH
Sumatera Selatan	1994	Demographic and Health Survey (DHS)	SBH
Sumatera Selatan	1995	Demographic and Health Survey (DHS)	CBH
Sumatera Selatan	1997	Demographic and Health Survey (DHS)	CBH
Sumatera Selatan	1997	Demographic and Health Survey (DHS)	SBH
Sumatera Selatan	1999	Demographic and Health Survey (DHS)	CBH
Sumatera Selatan	2001	Demographic and Health Survey (DHS)	CBH
Sumatera Selatan	2003	Demographic and Health Survey (DHS)	CBH
Sumatera Selatan	2003	Demographic and Health Survey (DHS)	SBH
Sumatera Selatan	2005	Demographic and Health Survey (DHS)	CBH
Sumatera Selatan	2007	Demographic and Health Survey (DHS)	CBH
Sumatera Selatan	2007	Demographic and Health Survey (DHS)	SBH
Sumatera Selatan	2009	Demographic and Health Survey (DHS)	CBH
Sumatera Selatan	2011	Demographic and Health Survey (DHS)	CBH
Sumatera Selatan	2012	Demographic and Health Survey (DHS)	SBH
Sumatera Selatan	1982	Health Survey	CBH
Sumatera Selatan	1984	Health Survey	CBH
Sumatera Selatan	1985	Health Survey	SBH
Sumatera Selatan	1986	Health Survey	CBH
Sumatera Selatan	1988	Health Survey	CBH
Sumatera Selatan	1990	Health Survey	CBH
Sumatera Selatan	1992	Health Survey	SBH
Sumatera Selatan	1992	Health Survey	CBH
Sumatera Selatan	1993	Health Survey	SBH
Sumatera Selatan	1994	Health Survey	SBH
Sumatera Selatan	1994	Health Survey	CBH
Sumatera Selatan	1995	Health Survey	SBH
Sumatera Selatan	1996	Health Survey	CBH
Sumatera Selatan	1996	Health Survey	SBH
Sumatera Selatan	1997	Health Survey	SBH
Sumatera Selatan	1998	Health Survey	CBH
Sumatera Selatan	1998	Health Survey	SBH
Sumatera Selatan	1999	Health Survey	SBH
Sumatera Selatan	2000	Health Survey	CBH
Sumatera Selatan	2000	Health Survey	SBH
Sumatera Selatan	2001	Health Survey	SBH
Sumatera Selatan	2002	Health Survey	SBH
Sumatera Selatan	2003	Health Survey	SBH
Sumatera Selatan	2004	Health Survey	SBH
Sumatera Selatan	2005	Health Survey	SBH
Sumatera Selatan	2006	Health Survey	SBH
Sumatera Selatan	2007	Health Survey	SBH
Sumatera Selatan	2008	Health Survey	SBH
Sumatera Selatan	2009	Health Survey	SBH
Sumatera Selatan	2010	Health Survey	SBH
Sumatera Selatan	2011	Health Survey	SBH
Sumatera Selatan	2012	Health Survey	SBH
Sumatera Selatan	2013	Health Survey	SBH
Sumatera Utara	1951	Census	CBH
Sumatera Utara	1952	Census	CBH
Sumatera Utara	1953	Census	CBH
Sumatera Utara	1954	Census	CBH
Sumatera Utara	1955	Census	CBH
Sumatera Utara	1956	Census	CBH
Sumatera Utara	1957	Census	CBH
Sumatera Utara	1958	Census	CBH
Sumatera Utara	1959	Census	CBH
Sumatera Utara	1960	Census	CBH
Sumatera Utara	1961	Census	CBH

Appendix Table 10. Under-5 mortality reference sources by source date and location, 1950-2016			
Location	Source	Source years	Method
Sumatera Utara	1962	Census	CBH
Sumatera Utara	1963	Census	CBH
Sumatera Utara	1964	Census	CBH
Sumatera Utara	1965	Census	CBH
Sumatera Utara	1966	Census	CBH
Sumatera Utara	1967	Census	CBH
Sumatera Utara	1968	Census	CBH
Sumatera Utara	1969	Census	CBH
Sumatera Utara	1970	Census	CBH
Sumatera Utara	1971	Census	CBH
Sumatera Utara	1972	Census	CBH
Sumatera Utara	1973	Census	CBH
Sumatera Utara	1974	Census	CBH
Sumatera Utara	1975	Census	CBH
Sumatera Utara	1976	Census	CBH
Sumatera Utara	1977	Census	CBH
Sumatera Utara	1978	Census	CBH
Sumatera Utara	1979	Census	CBH
Sumatera Utara	1980	Census	CBH
Sumatera Utara	1980	Census	SBH
Sumatera Utara	1981	Census	CBH
Sumatera Utara	1982	Census	CBH
Sumatera Utara	1983	Census	CBH
Sumatera Utara	1984	Census	CBH
Sumatera Utara	1985	Census	CBH
Sumatera Utara	1986	Census	CBH
Sumatera Utara	1987	Census	CBH
Sumatera Utara	1988	Census	CBH
Sumatera Utara	1989	Census	CBH
Sumatera Utara	1990	Census	SBH
Sumatera Utara	1990	Census	CBH
Sumatera Utara	1991	Census	CBH
Sumatera Utara	1992	Census	CBH
Sumatera Utara	1993	Census	CBH
Sumatera Utara	1994	Census	CBH
Sumatera Utara	1995	Census	CBH
Sumatera Utara	1996	Census	CBH
Sumatera Utara	1997	Census	CBH
Sumatera Utara	1998	Census	CBH
Sumatera Utara	1999	Census	CBH
Sumatera Utara	2000	Census	SBH
Sumatera Utara	2000	Census	CBH
Sumatera Utara	2002	Census	CBH
Sumatera Utara	2004	Census	CBH
Sumatera Utara	2010	Census	SBH
Sumatera Utara	1967	Demographic and Health Survey (DHS)	CBH
Sumatera Utara	1969	Demographic and Health Survey (DHS)	CBH
Sumatera Utara	1971	Demographic and Health Survey (DHS)	CBH
Sumatera Utara	1973	Demographic and Health Survey (DHS)	CBH
Sumatera Utara	1975	Demographic and Health Survey (DHS)	CBH
Sumatera Utara	1977	Demographic and Health Survey (DHS)	CBH
Sumatera Utara	1979	Demographic and Health Survey (DHS)	CBH
Sumatera Utara	1981	Demographic and Health Survey (DHS)	CBH
Sumatera Utara	1983	Demographic and Health Survey (DHS)	CBH
Sumatera Utara	1985	Demographic and Health Survey (DHS)	CBH
Sumatera Utara	1987	Demographic and Health Survey (DHS)	SBH
Sumatera Utara	1987	Demographic and Health Survey (DHS)	CBH
Sumatera Utara	1989	Demographic and Health Survey (DHS)	CBH
Sumatera Utara	1991	Demographic and Health Survey (DHS)	SBH
Sumatera Utara	1991	Demographic and Health Survey (DHS)	CBH
Sumatera Utara	1993	Demographic and Health Survey (DHS)	CBH
Sumatera Utara	1994	Demographic and Health Survey (DHS)	SBH
Sumatera Utara	1995	Demographic and Health Survey (DHS)	CBH
Sumatera Utara	1997	Demographic and Health Survey (DHS)	CBH
Sumatera Utara	1997	Demographic and Health Survey (DHS)	SBH
Sumatera Utara	1999	Demographic and Health Survey (DHS)	CBH
Sumatera Utara	2001	Demographic and Health Survey (DHS)	CBH

Appendix Table 10. Under-5 mortality reference sources by source date and location, 1950-2016			
**Location**	**Source**	**Source years**	**Method**
Sumatera Utara	2003	Demographic and Health Survey (DHS)	CBH
Sumatera Utara	2003	Demographic and Health Survey (DHS)	SBH
Sumatera Utara	2005	Demographic and Health Survey (DHS)	CBH
Sumatera Utara	2007	Demographic and Health Survey (DHS)	CBH
Sumatera Utara	2007	Demographic and Health Survey (DHS)	SBH
Sumatera Utara	2009	Demographic and Health Survey (DHS)	CBH
Sumatera Utara	2011	Demographic and Health Survey (DHS)	CBH
Sumatera Utara	2012	Demographic and Health Survey (DHS)	SBH
Sumatera Utara	1982	Health Survey	CBH
Sumatera Utara	1984	Health Survey	CBH
Sumatera Utara	1985	Health Survey	SBH
Sumatera Utara	1986	Health Survey	CBH
Sumatera Utara	1988	Health Survey	CBH
Sumatera Utara	1990	Health Survey	CBH
Sumatera Utara	1992	Health Survey	SBH
Sumatera Utara	1992	Health Survey	CBH
Sumatera Utara	1993	Health Survey	SBH
Sumatera Utara	1994	Health Survey	SBH
Sumatera Utara	1994	Health Survey	CBH
Sumatera Utara	1995	Health Survey	SBH
Sumatera Utara	1996	Health Survey	SBH
Sumatera Utara	1996	Health Survey	CBH
Sumatera Utara	1997	Health Survey	SBH
Sumatera Utara	1998	Health Survey	SBH
Sumatera Utara	1998	Health Survey	CBH
Sumatera Utara	1999	Health Survey	SBH
Sumatera Utara	2000	Health Survey	CBH
Sumatera Utara	2000	Health Survey	SBH
Sumatera Utara	2001	Health Survey	SBH
Sumatera Utara	2002	Health Survey	SBH
Sumatera Utara	2003	Health Survey	SBH
Sumatera Utara	2004	Health Survey	SBH
Sumatera Utara	2005	Health Survey	SBH
Sumatera Utara	2006	Health Survey	SBH
Sumatera Utara	2007	Health Survey	SBH
Sumatera Utara	2008	Health Survey	SBH
Sumatera Utara	2009	Health Survey	SBH
Sumatera Utara	2010	Health Survey	SBH
Sumatera Utara	2011	Health Survey	SBH
Sumatera Utara	2012	Health Survey	SBH
Sumatera Utara	2013	Health Survey	SBH
Yogyakarta	1955	Census	CBH
Yogyakarta	1957	Census	CBH
Yogyakarta	1958	Census	CBH
Yogyakarta	1959	Census	CBH
Yogyakarta	1960	Census	CBH
Yogyakarta	1961	Census	CBH
Yogyakarta	1962	Census	CBH
Yogyakarta	1963	Census	CBH
Yogyakarta	1964	Census	CBH
Yogyakarta	1965	Census	CBH
Yogyakarta	1966	Census	CBH
Yogyakarta	1967	Census	CBH
Yogyakarta	1968	Census	CBH
Yogyakarta	1969	Census	CBH
Yogyakarta	1970	Census	CBH
Yogyakarta	1971	Census	CBH
Yogyakarta	1972	Census	CBH
Yogyakarta	1973	Census	CBH
Yogyakarta	1974	Census	CBH
Yogyakarta	1975	Census	CBH
Yogyakarta	1976	Census	CBH
Yogyakarta	1977	Census	CBH
Yogyakarta	1978	Census	CBH
Yogyakarta	1979	Census	CBH
Yogyakarta	1980	Census	SBH
Yogyakarta	1980	Census	CBH

Appendix Table 10. Under-5 mortality reference sources by source date and location, 1950-2016			
**Location**	**Source**	**Source years**	**Method**
Yogyakarta	1981	Census	CBH
Yogyakarta	1982	Census	CBH
Yogyakarta	1983	Census	CBH
Yogyakarta	1984	Census	CBH
Yogyakarta	1985	Census	CBH
Yogyakarta	1986	Census	CBH
Yogyakarta	1987	Census	CBH
Yogyakarta	1988	Census	CBH
Yogyakarta	1989	Census	CBH
Yogyakarta	1990	Census	CBH
Yogyakarta	1990	Census	SBH
Yogyakarta	1991	Census	CBH
Yogyakarta	1992	Census	CBH
Yogyakarta	1993	Census	CBH
Yogyakarta	1994	Census	CBH
Yogyakarta	1995	Census	CBH
Yogyakarta	1996	Census	CBH
Yogyakarta	1997	Census	CBH
Yogyakarta	1998	Census	CBH
Yogyakarta	1999	Census	CBH
Yogyakarta	2000	Census	CBH
Yogyakarta	2000	Census	SBH
Yogyakarta	2002	Census	CBH
Yogyakarta	2004	Census	CBH
Yogyakarta	2010	Census	SBH
Yogyakarta	1965	Demographic and Health Survey (DHS)	CBH
Yogyakarta	1967	Demographic and Health Survey (DHS)	CBH
Yogyakarta	1969	Demographic and Health Survey (DHS)	CBH
Yogyakarta	1971	Demographic and Health Survey (DHS)	CBH
Yogyakarta	1973	Demographic and Health Survey (DHS)	CBH
Yogyakarta	1975	Demographic and Health Survey (DHS)	CBH
Yogyakarta	1977	Demographic and Health Survey (DHS)	CBH
Yogyakarta	1979	Demographic and Health Survey (DHS)	CBH
Yogyakarta	1981	Demographic and Health Survey (DHS)	CBH
Yogyakarta	1983	Demographic and Health Survey (DHS)	CBH
Yogyakarta	1985	Demographic and Health Survey (DHS)	CBH
Yogyakarta	1987	Demographic and Health Survey (DHS)	SBH
Yogyakarta	1987	Demographic and Health Survey (DHS)	CBH
Yogyakarta	1989	Demographic and Health Survey (DHS)	CBH
Yogyakarta	1991	Demographic and Health Survey (DHS)	CBH
Yogyakarta	1991	Demographic and Health Survey (DHS)	SBH
Yogyakarta	1993	Demographic and Health Survey (DHS)	CBH
Yogyakarta	1994	Demographic and Health Survey (DHS)	SBH
Yogyakarta	1995	Demographic and Health Survey (DHS)	CBH
Yogyakarta	1997	Demographic and Health Survey (DHS)	SBH
Yogyakarta	1997	Demographic and Health Survey (DHS)	CBH
Yogyakarta	1999	Demographic and Health Survey (DHS)	CBH
Yogyakarta	2001	Demographic and Health Survey (DHS)	CBH
Yogyakarta	2003	Demographic and Health Survey (DHS)	SBH
Yogyakarta	2003	Demographic and Health Survey (DHS)	CBH
Yogyakarta	2005	Demographic and Health Survey (DHS)	CBH
Yogyakarta	2007	Demographic and Health Survey (DHS)	CBH
Yogyakarta	2007	Demographic and Health Survey (DHS)	SBH
Yogyakarta	2009	Demographic and Health Survey (DHS)	CBH
Yogyakarta	2011	Demographic and Health Survey (DHS)	CBH
Yogyakarta	2012	Demographic and Health Survey (DHS)	SBH
Yogyakarta	1982	Health Survey	CBH
Yogyakarta	1984	Health Survey	CBH
Yogyakarta	1985	Health Survey	SBH
Yogyakarta	1986	Health Survey	CBH
Yogyakarta	1988	Health Survey	CBH
Yogyakarta	1990	Health Survey	CBH
Yogyakarta	1992	Health Survey	SBH
Yogyakarta	1992	Health Survey	CBH
Yogyakarta	1993	Health Survey	SBH
Yogyakarta	1994	Health Survey	CBH
Yogyakarta	1994	Health Survey	SBH

Appendix Table 10. Under-5 mortality reference sources by source date and location, 1950-2016			
Location	Source	Source years	Method
Yogyakarta	1995	Health Survey	SBH
Yogyakarta	1996	Health Survey	SBH
Yogyakarta	1996	Health Survey	CBH
Yogyakarta	1997	Health Survey	SBH
Yogyakarta	1998	Health Survey	CBH
Yogyakarta	1998	Health Survey	SBH
Yogyakarta	1999	Health Survey	SBH
Yogyakarta	2000	Health Survey	SBH
Yogyakarta	2000	Health Survey	CBH
Yogyakarta	2001	Health Survey	SBH
Yogyakarta	2002	Health Survey	SBH
Yogyakarta	2003	Health Survey	SBH
Yogyakarta	2004	Health Survey	SBH
Yogyakarta	2005	Health Survey	SBH
Yogyakarta	2006	Health Survey	SBH
Yogyakarta	2007	Health Survey	SBH
Yogyakarta	2008	Health Survey	SBH
Yogyakarta	2009	Health Survey	SBH
Yogyakarta	2010	Health Survey	SBH
Yogyakarta	2011	Health Survey	SBH
Yogyakarta	2012	Health Survey	SBH
Yogyakarta	2013	Health Survey	SBH
Laos	2011	Multiple Indicator Cluster Survey (MICS)	CBH
Malaysia	1952-2012	Vital Registration	VR/SRS/DSP
Maldives	1974-2013	Vital Registration	VR/SRS/DSP
Mauritius	1950-2014	Vital Registration	VR/SRS/DSP
Myanmar	2014	Census	SBH
Myanmar	1997	Health Survey	SBH
Myanmar	2001	Health Survey	SBH
Myanmar	2007	Health Survey	SBH
Myanmar	2010	Multiple Indicator Cluster Survey (MICS)	CBH
Philippines	1993	Demographic and Health Survey (DHS)	CBH
Philippines	1998	Demographic and Health Survey (DHS)	CBH
Philippines	2003	Demographic and Health Survey (DHS)	CBH
Philippines	2008	Demographic and Health Survey (DHS)	CBH
Philippines	2013	Demographic and Health Survey (DHS)	CBH
Sri Lanka	1950-2010	Vital Registration	VR/SRS/DSP
Seychelles	1952-2014	Vital Registration	VR/SRS/DSP
Thailand	1987	Demographic and Health Survey (DHS)	CBH
Timor-Leste	2009	Demographic and Health Survey (DHS)	CBH
Vietnam	1997	Demographic and Health Survey (DHS)	CBH
Vietnam	2002	Demographic and Health Survey (DHS)	CBH
American Samoa	1952-2012	Vital Registration	VR/SRS/DSP
Federated States of Micronesia	2000	Census	SBH
Federated States of Micronesia	2003	Vital Registration	VR/SRS/DSP
Fiji	2007	Census	SBH
Fiji	1986	Census (IPUMS)	SBH
Fiji	1996	Census (IPUMS)	SBH
Guam	1950-2012	Vital Registration	VR/SRS/DSP
Kiribati	2005	Census	SBH
Kiribati	2010	Census	SBH
Kiribati	2009	Demographic Survey	CBH
Kiribati	2009	Demographic Survey	SBH
Marshall Islands	1999	Census	SBH
Marshall Islands	2011	Census	SBH
Marshall Islands	2006	Demographic and Health Survey (DHS)	CBH
Northern Mariana Islands	1998-2012	Vital Registration	VR/SRS/DSP
Papua New Guinea	2000	Census	SBH
Papua New Guinea	1991	Demographic and Health Survey (DHS)	SBH
Papua New Guinea	1996	Demographic and Health Survey (DHS)	CBH
Papua New Guinea	1997	Demographic and Health Survey (DHS)	SBH
Papua New Guinea	2006	Demographic and Health Survey (DHS)	CBH
Samoa	2009	Demographic Survey	CBH
Solomon Islands	1999	Census	SBH
Solomon Islands	2009	Census	SBH
Solomon Islands	2006	Demographic and Health Survey (DHS)	CBH
Tonga	2012	National Demographic and Health Survey	CBH

Appendix Table 10. Under-5 mortality reference sources by source date and location, 1950-2016			
**Location**	**Source**	**Source years**	**Method**
Vanuatu	2013	National Demographic and Health Survey	CBH
Armenia	2000	Demographic and Health Survey (DHS)	CBH
Armenia	2005	Demographic and Health Survey (DHS)	CBH
Armenia	2010	Demographic and Health Survey (DHS)	CBH
Armenia	2016	Demographic and Health Survey (DHS)	CBH
Azerbaijan	2006	Demographic and Health Survey (DHS)	CBH
Azerbaijan	2011	National Demographic and Health Survey	CBH
Georgia	2005	Multiple Indicator Cluster Survey (MICS)	SBH
Georgia	1999	Reproductive Health Survey (RHS)	SBH
Georgia	1999	Reproductive Health Survey (RHS)	CBH
Georgia	2005	Reproductive Health Survey (RHS)	CBH
Georgia	2005	Reproductive Health Survey (RHS)	SBH
Georgia	2010	Reproductive Health Survey (RHS)	CBH
Kazakhstan	2006	Multiple Indicator Cluster Survey (MICS)	SBH
Kazakhstan	2010	Multiple Indicator Cluster Survey (MICS)	SBH
Kazakhstan	2008-2015	Vital Registration	VR/SRS/DSP
Kyrgyzstan	1997	Demographic and Health Survey (DHS)	CBH
Kyrgyzstan	2012	Demographic and Health Survey (DHS)	CBH
Mongolia	1989	Census (IPUMS)	SBH
Mongolia	2000	Multiple Indicator Cluster Survey (MICS)	SBH
Mongolia	2005	Multiple Indicator Cluster Survey (MICS)	SBH
Mongolia	2010	Multiple Indicator Cluster Survey (MICS)	SBH
Mongolia	2013	Multiple Indicator Cluster Survey (MICS)	SBH
Mongolia	2013	Multiple Indicator Cluster Survey (MICS)	CBH
Mongolia	1998	Reproductive Health Survey (RHS)	CBH
Mongolia	1998	Reproductive Health Survey (RHS)	SBH
Mongolia	2003	Reproductive Health Survey (RHS)	SBH
Mongolia	2008	Reproductive Health Survey (RHS)	SBH
Tajikistan	1989	Census	SBH
Tajikistan	2012	Demographic and Health Survey (DHS)	SBH
Tajikistan	2012	Demographic and Health Survey (DHS)	CBH
Tajikistan	2010	Health Survey	CBH
Tajikistan	1999	Living Standards Measurement Study (LSMS)	SBH
Tajikistan	2003	Living Standards Measurement Study (LSMS)	SBH
Tajikistan	2007	Living Standards Measurement Study (LSMS)	SBH
Tajikistan	2000	Multiple Indicator Cluster Survey (MICS)	SBH
Tajikistan	2005	Multiple Indicator Cluster Survey (MICS)	SBH
Turkmenistan	2000	Demographic and Health Survey (DHS)	CBH
Uzbekistan	1996	Demographic and Health Survey (DHS)	CBH
Albania	2008	Demographic and Health Survey (DHS)	CBH
Bosnia and Herzegovina	1985-2014	Vital Registration	VR/SRS/DSP
Bulgaria	1950-2013	Vital Registration	VR/SRS/DSP
Croatia	1985-2013	Vital Registration	VR/SRS/DSP
Czech Republic	1950-2015	Vital Registration	VR/SRS/DSP
Hungary	1950-2015	Vital Registration	VR/SRS/DSP
Macedonia	1982-2013	Vital Registration	VR/SRS/DSP
Montenegro	1995-2013	Vital Registration	VR/SRS/DSP
Poland	1950-2014	Vital Registration	VR/SRS/DSP
Romania	1956-2015	Vital Registration	VR/SRS/DSP
Serbia	1995-2014	Vital Registration	VR/SRS/DSP
Slovakia	1950-2014	Vital Registration	VR/SRS/DSP
Slovenia	1982-2015	Vital Registration	VR/SRS/DSP
Belarus	1989	Census	SBH
Belarus	2005	Multiple Indicator Cluster Survey (MICS)	SBH
Estonia	1959-2014	Vital Registration	VR/SRS/DSP
Latvia	1959-2014	Vital Registration	VR/SRS/DSP
Lithuania	1959-2015	Vital Registration	VR/SRS/DSP
Moldova	2005	Demographic and Health Survey (DHS)	CBH
Russia	1959-2014	Vital Registration	VR/SRS/DSP
Ukraine	2007	Demographic and Health Survey (DHS)	CBH
Brunei	1950-2014	Vital Registration	VR/SRS/DSP
Japan	1950-2014	Vital Registration	VR/SRS/DSP
Aichi	1979-2013	Vital Registration	VR/SRS/DSP
Akita	1979-2013	Vital Registration	VR/SRS/DSP
Aomori	1979-2013	Vital Registration	VR/SRS/DSP
Chiba	1979-2013	Vital Registration	VR/SRS/DSP
Ehime	1979-2013	Vital Registration	VR/SRS/DSP

Appendix Table 10. Under-5 mortality reference sources by source date and location, 1950-2016			
**Location**	**Source**	**Source years**	**Method**
Fukui	1979-2013	Vital Registration	VR/SRS/DSP
Fukuoka	1979-2013	Vital Registration	VR/SRS/DSP
Fukushima	1979-2013	Vital Registration	VR/SRS/DSP
Gifu	1979-2013	Vital Registration	VR/SRS/DSP
Gunma	1979-2013	Vital Registration	VR/SRS/DSP
Hiroshima	1979-2013	Vital Registration	VR/SRS/DSP
Hokkaido	1979-2013	Vital Registration	VR/SRS/DSP
Hyogo	1979-2013	Vital Registration	VR/SRS/DSP
Ibaraki	1979-2013	Vital Registration	VR/SRS/DSP
Ishikawa	1979-2013	Vital Registration	VR/SRS/DSP
Iwate	1979-2013	Vital Registration	VR/SRS/DSP
Kagawa	1979-2013	Vital Registration	VR/SRS/DSP
Kagoshima	1979-2013	Vital Registration	VR/SRS/DSP
Kanagawa	1979-2013	Vital Registration	VR/SRS/DSP
Kochi	1979-2013	Vital Registration	VR/SRS/DSP
Kumamoto	1979-2013	Vital Registration	VR/SRS/DSP
Kyoto	1979-2013	Vital Registration	VR/SRS/DSP
Mie	1979-2013	Vital Registration	VR/SRS/DSP
Miyagi	1979-2013	Vital Registration	VR/SRS/DSP
Miyazaki	1979-2013	Vital Registration	VR/SRS/DSP
Nagano	1979-2013	Vital Registration	VR/SRS/DSP
Nagasaki	1979-2013	Vital Registration	VR/SRS/DSP
Nara	1979-2013	Vital Registration	VR/SRS/DSP
Niigata	1979-2013	Vital Registration	VR/SRS/DSP
Oita	1979-2013	Vital Registration	VR/SRS/DSP
Okayama	1979-2013	Vital Registration	VR/SRS/DSP
Okinawa	1979-2013	Vital Registration	VR/SRS/DSP
Osaka	1979-2013	Vital Registration	VR/SRS/DSP
Saga	1979-2013	Vital Registration	VR/SRS/DSP
Saitama	1979-2013	Vital Registration	VR/SRS/DSP
Shiga	1979-2013	Vital Registration	VR/SRS/DSP
Shimane	1979-2013	Vital Registration	VR/SRS/DSP
Shizuoka	1979-2013	Vital Registration	VR/SRS/DSP
Tochigi	1979-2013	Vital Registration	VR/SRS/DSP
Tokushima	1979-2013	Vital Registration	VR/SRS/DSP
Tokyo	1979-2013	Vital Registration	VR/SRS/DSP
Tottori	1979-2013	Vital Registration	VR/SRS/DSP
Toyama	1979-2013	Vital Registration	VR/SRS/DSP
Wakayama	1979-2013	Vital Registration	VR/SRS/DSP
Yamagata	1979-2013	Vital Registration	VR/SRS/DSP
Yamaguchi	1979-2013	Vital Registration	VR/SRS/DSP
Yamanashi	1979-2013	Vital Registration	VR/SRS/DSP
South Korea	1999-2013	Vital Registration	VR/SRS/DSP
Singapore	1950-2015	Vital Registration	VR/SRS/DSP
Australia	1950-2014	Vital Registration	VR/SRS/DSP
New Zealand	1950-2014	Vital Registration	VR/SRS/DSP
Andorra	1950-2012	Vital Registration	VR/SRS/DSP
Austria	1950-2014	Vital Registration	VR/SRS/DSP
Belgium	1950-2014	Vital Registration	VR/SRS/DSP
Cyprus	1974-2014	Vital Registration	VR/SRS/DSP
Denmark	1950-2014	Vital Registration	VR/SRS/DSP
Finland	1950-2015	Vital Registration	VR/SRS/DSP
France	1950-2014	Vital Registration	VR/SRS/DSP
Germany	1956-2014	Vital Registration	VR/SRS/DSP
Greece	1951-2014	Vital Registration	VR/SRS/DSP
Iceland	1950-2015	Vital Registration	VR/SRS/DSP
Ireland	1950-2014	Vital Registration	VR/SRS/DSP
Israel	1950-2014	Vital Registration	VR/SRS/DSP
Italy	1950-2013	Vital Registration	VR/SRS/DSP
Luxembourg	1950-2014	Vital Registration	VR/SRS/DSP
Malta	1950-2014	Vital Registration	VR/SRS/DSP
Netherlands	1950-2015	Vital Registration	VR/SRS/DSP
Norway	1950-2014	Vital Registration	VR/SRS/DSP
Portugal	1950-2014	Vital Registration	VR/SRS/DSP
Spain	1950-2014	Vital Registration	VR/SRS/DSP
Sweden	1950-2014	Vital Registration	VR/SRS/DSP
Stockholm	1980-2010	Vital Registration	VR/SRS/DSP

Appendix Table 10. Under-5 mortality reference sources by source date and location, 1950-2016			
Location	Source	Source years	Method
Sweden except Stockholm	1980-2010	Vital Registration	VR/SRS/DSP
Switzerland	1950-2014	Vital Registration	VR/SRS/DSP
United Kingdom	1950-2013	Vital Registration	VR/SRS/DSP
England	1981-2015	Vital Registration	VR/SRS/DSP
East Midlands	1981-2015	Vital Registration	VR/SRS/DSP
Derby	1981-2015	Vital Registration	VR/SRS/DSP
Derbyshire	1981-2015	Vital Registration	VR/SRS/DSP
Leicester	1981-2015	Vital Registration	VR/SRS/DSP
Leicestershire	1981-2015	Vital Registration	VR/SRS/DSP
Lincolnshire	1981-2015	Vital Registration	VR/SRS/DSP
Northamptonshire	1981-2015	Vital Registration	VR/SRS/DSP
Nottingham	1981-2015	Vital Registration	VR/SRS/DSP
Nottinghamshire	1981-2015	Vital Registration	VR/SRS/DSP
Rutland	1981-2015	Vital Registration	VR/SRS/DSP
East of England	1981-2015	Vital Registration	VR/SRS/DSP
Bedford	1981-2015	Vital Registration	VR/SRS/DSP
Cambridgeshire	1981-2015	Vital Registration	VR/SRS/DSP
Central Bedfordshire	1981-2015	Vital Registration	VR/SRS/DSP
Essex	1981-2015	Vital Registration	VR/SRS/DSP
Hertfordshire	1981-2015	Vital Registration	VR/SRS/DSP
Luton	1981-2015	Vital Registration	VR/SRS/DSP
Norfolk	1981-2015	Vital Registration	VR/SRS/DSP
Peterborough	1981-2015	Vital Registration	VR/SRS/DSP
Southend-on-Sea	1981-2015	Vital Registration	VR/SRS/DSP
Suffolk	1981-2015	Vital Registration	VR/SRS/DSP
Thurrock	1981-2015	Vital Registration	VR/SRS/DSP
Greater London	1981-2015	Vital Registration	VR/SRS/DSP
Barking and Dagenham	1981-2015	Vital Registration	VR/SRS/DSP
Barnet	1981-2015	Vital Registration	VR/SRS/DSP
Bexley	1981-2015	Vital Registration	VR/SRS/DSP
Brent	1981-2015	Vital Registration	VR/SRS/DSP
Bromley	1981-2015	Vital Registration	VR/SRS/DSP
Camden	1981-2015	Vital Registration	VR/SRS/DSP
Croydon	1981-2015	Vital Registration	VR/SRS/DSP
Ealing	1981-2015	Vital Registration	VR/SRS/DSP
Enfield	1981-2015	Vital Registration	VR/SRS/DSP
Greenwich	1981-2015	Vital Registration	VR/SRS/DSP
Hackney	1981-2015	Vital Registration	VR/SRS/DSP
Hammersmith and Fulham	1981-2015	Vital Registration	VR/SRS/DSP
Haringey	1981-2015	Vital Registration	VR/SRS/DSP
Harrow	1981-2015	Vital Registration	VR/SRS/DSP
Havering	1981-2015	Vital Registration	VR/SRS/DSP
Hillingdon	1981-2015	Vital Registration	VR/SRS/DSP
Hounslow	1981-2015	Vital Registration	VR/SRS/DSP
Islington	1981-2015	Vital Registration	VR/SRS/DSP
Kensington and Chelsea	1981-2015	Vital Registration	VR/SRS/DSP
Kingston upon Thames	1981-2015	Vital Registration	VR/SRS/DSP
Lambeth	1981-2015	Vital Registration	VR/SRS/DSP
Lewisham	1981-2015	Vital Registration	VR/SRS/DSP
Merton	1981-2015	Vital Registration	VR/SRS/DSP
Newham	1981-2015	Vital Registration	VR/SRS/DSP
Redbridge	1981-2015	Vital Registration	VR/SRS/DSP
Richmond upon Thames	1981-2015	Vital Registration	VR/SRS/DSP
Southwark	1981-2015	Vital Registration	VR/SRS/DSP
Sutton	1981-2015	Vital Registration	VR/SRS/DSP
Tower Hamlets	1981-2015	Vital Registration	VR/SRS/DSP
Waltham Forest	1981-2015	Vital Registration	VR/SRS/DSP
Wandsworth	1981-2015	Vital Registration	VR/SRS/DSP
Westminster	1981-2015	Vital Registration	VR/SRS/DSP
North East England	1981-2015	Vital Registration	VR/SRS/DSP
County Durham	1981-2015	Vital Registration	VR/SRS/DSP
Darlington	1981-2015	Vital Registration	VR/SRS/DSP
Gateshead	1981-2015	Vital Registration	VR/SRS/DSP
Hartlepool	1981-2015	Vital Registration	VR/SRS/DSP
Middlesbrough	1981-2015	Vital Registration	VR/SRS/DSP
Newcastle upon Tyne	1981-2015	Vital Registration	VR/SRS/DSP
North Tyneside	1981-2015	Vital Registration	VR/SRS/DSP

Appendix Table 10. Under-5 mortality reference sources by source date and location, 1950-2016			
Location	Source	Source years	Method
Northumberland	1981-2015	Vital Registration	VR/SRS/DSP
Redcar and Cleveland	1981-2015	Vital Registration	VR/SRS/DSP
South Tyneside	1981-2015	Vital Registration	VR/SRS/DSP
Stockton-on-Tees	1981-2015	Vital Registration	VR/SRS/DSP
Sunderland	1981-2015	Vital Registration	VR/SRS/DSP
North West England	1981-2015	Vital Registration	VR/SRS/DSP
Blackburn with Darwen	1981-2015	Vital Registration	VR/SRS/DSP
Blackpool	1981-2015	Vital Registration	VR/SRS/DSP
Bolton	1981-2015	Vital Registration	VR/SRS/DSP
Bury	1981-2015	Vital Registration	VR/SRS/DSP
Cheshire East	1981-2015	Vital Registration	VR/SRS/DSP
Cheshire West and Chester	1981-2015	Vital Registration	VR/SRS/DSP
Cumbria	1981-2015	Vital Registration	VR/SRS/DSP
Halton	1981-2015	Vital Registration	VR/SRS/DSP
Knowsley	1981-2015	Vital Registration	VR/SRS/DSP
Lancashire	1981-2015	Vital Registration	VR/SRS/DSP
Liverpool	1981-2015	Vital Registration	VR/SRS/DSP
Manchester	1981-2015	Vital Registration	VR/SRS/DSP
Oldham	1981-2015	Vital Registration	VR/SRS/DSP
Rochdale	1981-2015	Vital Registration	VR/SRS/DSP
Salford	1981-2015	Vital Registration	VR/SRS/DSP
Sefton	1981-2015	Vital Registration	VR/SRS/DSP
St Helens	1981-2015	Vital Registration	VR/SRS/DSP
Stockport	1981-2015	Vital Registration	VR/SRS/DSP
Tameside	1981-2015	Vital Registration	VR/SRS/DSP
Trafford	1981-2015	Vital Registration	VR/SRS/DSP
Warrington	1981-2015	Vital Registration	VR/SRS/DSP
Wigan	1981-2015	Vital Registration	VR/SRS/DSP
Wirral	1981-2015	Vital Registration	VR/SRS/DSP
South East England	1981-2015	Vital Registration	VR/SRS/DSP
Bracknell Forest	1981-2015	Vital Registration	VR/SRS/DSP
Brighton and Hove	1981-2015	Vital Registration	VR/SRS/DSP
Buckinghamshire	1981-2015	Vital Registration	VR/SRS/DSP
East Sussex	1981-2015	Vital Registration	VR/SRS/DSP
Hampshire	1981-2015	Vital Registration	VR/SRS/DSP
Isle of Wight	1981-2015	Vital Registration	VR/SRS/DSP
Kent	1981-2015	Vital Registration	VR/SRS/DSP
Medway	1981-2015	Vital Registration	VR/SRS/DSP
Milton Keynes	1981-2015	Vital Registration	VR/SRS/DSP
Oxfordshire	1981-2015	Vital Registration	VR/SRS/DSP
Portsmouth	1981-2015	Vital Registration	VR/SRS/DSP
Reading	1981-2015	Vital Registration	VR/SRS/DSP
Slough	1981-2015	Vital Registration	VR/SRS/DSP
Southampton	1981-2015	Vital Registration	VR/SRS/DSP
Surrey	1981-2015	Vital Registration	VR/SRS/DSP
West Berkshire	1981-2015	Vital Registration	VR/SRS/DSP
West Sussex	1981-2015	Vital Registration	VR/SRS/DSP
Windsor and Maidenhead	1981-2015	Vital Registration	VR/SRS/DSP
Wokingham	1981-2015	Vital Registration	VR/SRS/DSP
South West England	1981-2015	Vital Registration	VR/SRS/DSP
Bath and North East Somerset	1981-2015	Vital Registration	VR/SRS/DSP
Bournemouth	1981-2015	Vital Registration	VR/SRS/DSP
Bristol, City of	1981-2015	Vital Registration	VR/SRS/DSP
Cornwall	1981-2015	Vital Registration	VR/SRS/DSP
Devon	1981-2015	Vital Registration	VR/SRS/DSP
Dorset	1981-2015	Vital Registration	VR/SRS/DSP
Gloucestershire	1981-2015	Vital Registration	VR/SRS/DSP
North Somerset	1981-2015	Vital Registration	VR/SRS/DSP
Plymouth	1981-2015	Vital Registration	VR/SRS/DSP
Poole	1981-2015	Vital Registration	VR/SRS/DSP
Somerset	1981-2015	Vital Registration	VR/SRS/DSP
South Gloucestershire	1981-2015	Vital Registration	VR/SRS/DSP
Swindon	1981-2015	Vital Registration	VR/SRS/DSP
Torbay	1981-2015	Vital Registration	VR/SRS/DSP
Wiltshire	1981-2015	Vital Registration	VR/SRS/DSP
West Midlands	1981-2015	Vital Registration	VR/SRS/DSP
Birmingham	1981-2015	Vital Registration	VR/SRS/DSP

Appendix Table 10. Under-5 mortality reference sources by source date and location, 1950-2016			
**Location**	**Source**	**Source years**	**Method**
Coventry	1981-2015	Vital Registration	VR/SRS/DSP
Dudley	1981-2015	Vital Registration	VR/SRS/DSP
Herefordshire, County of	1981-2015	Vital Registration	VR/SRS/DSP
Sandwell	1981-2015	Vital Registration	VR/SRS/DSP
Shropshire	1981-2015	Vital Registration	VR/SRS/DSP
Solihull	1981-2015	Vital Registration	VR/SRS/DSP
Staffordshire	1981-2015	Vital Registration	VR/SRS/DSP
Stoke-on-Trent	1981-2015	Vital Registration	VR/SRS/DSP
Telford and Wrekin	1981-2015	Vital Registration	VR/SRS/DSP
Walsall	1981-2015	Vital Registration	VR/SRS/DSP
Warwickshire	1981-2015	Vital Registration	VR/SRS/DSP
Wolverhampton	1981-2015	Vital Registration	VR/SRS/DSP
Worcestershire	1981-2015	Vital Registration	VR/SRS/DSP
Yorkshire and the Humber	1981-2015	Vital Registration	VR/SRS/DSP
Barnsley	1981-2015	Vital Registration	VR/SRS/DSP
Bradford	1981-2015	Vital Registration	VR/SRS/DSP
Calderdale	1981-2015	Vital Registration	VR/SRS/DSP
Doncaster	1981-2015	Vital Registration	VR/SRS/DSP
East Riding of Yorkshire	1981-2015	Vital Registration	VR/SRS/DSP
Kingston upon Hull, City of	1981-2015	Vital Registration	VR/SRS/DSP
Kirklees	1981-2015	Vital Registration	VR/SRS/DSP
Leeds	1981-2015	Vital Registration	VR/SRS/DSP
North East Lincolnshire	1981-2015	Vital Registration	VR/SRS/DSP
North Lincolnshire	1981-2015	Vital Registration	VR/SRS/DSP
North Yorkshire	1981-2015	Vital Registration	VR/SRS/DSP
Rotherham	1981-2015	Vital Registration	VR/SRS/DSP
Sheffield	1981-2015	Vital Registration	VR/SRS/DSP
Wakefield	1981-2015	Vital Registration	VR/SRS/DSP
York	1981-2015	Vital Registration	VR/SRS/DSP
Northern Ireland	1950-2013	Vital Registration	VR/SRS/DSP
Scotland	1950-2014	Vital Registration	VR/SRS/DSP
Wales	1981-2013	Vital Registration	VR/SRS/DSP
Argentina	1950-2014	Vital Registration	VR/SRS/DSP
Chile	1950-2014	Vital Registration	VR/SRS/DSP
Uruguay	1950-2014	Vital Registration	VR/SRS/DSP
Canada	1950-2016	Vital Registration	VR/SRS/DSP
Greenland	1952-2014	Vital Registration	VR/SRS/DSP
United States	1950-2015	Vital Registration	VR/SRS/DSP
Alabama	1968-2014	Vital Registration	VR/SRS/DSP
Alaska	1968-2014	Vital Registration	VR/SRS/DSP
Arizona	1968-2014	Vital Registration	VR/SRS/DSP
Arkansas	1968-2014	Vital Registration	VR/SRS/DSP
California	1968-2014	Vital Registration	VR/SRS/DSP
Colorado	1968-2014	Vital Registration	VR/SRS/DSP
Connecticut	1968-2014	Vital Registration	VR/SRS/DSP
Delaware	1968-2014	Vital Registration	VR/SRS/DSP
District of Columbia	1968-2014	Vital Registration	VR/SRS/DSP
Florida	1968-2014	Vital Registration	VR/SRS/DSP
Georgia	1968-2014	Vital Registration	VR/SRS/DSP
Hawaii	1968-2014	Vital Registration	VR/SRS/DSP
Idaho	1968-2014	Vital Registration	VR/SRS/DSP
Illinois	1968-2014	Vital Registration	VR/SRS/DSP
Indiana	1968-2014	Vital Registration	VR/SRS/DSP
Iowa	1968-2014	Vital Registration	VR/SRS/DSP
Kansas	1968-2014	Vital Registration	VR/SRS/DSP
Kentucky	1968-2014	Vital Registration	VR/SRS/DSP
Louisiana	1968-2014	Vital Registration	VR/SRS/DSP
Maine	1968-2014	Vital Registration	VR/SRS/DSP
Maryland	1968-2014	Vital Registration	VR/SRS/DSP
Massachusetts	1968-2014	Vital Registration	VR/SRS/DSP
Michigan	1968-2014	Vital Registration	VR/SRS/DSP
Minnesota	1968-2014	Vital Registration	VR/SRS/DSP
Mississippi	1968-2014	Vital Registration	VR/SRS/DSP
Missouri	1968-2014	Vital Registration	VR/SRS/DSP
Montana	1968-2014	Vital Registration	VR/SRS/DSP
Nebraska	1968-2014	Vital Registration	VR/SRS/DSP
Nevada	1968-2014	Vital Registration	VR/SRS/DSP

Appendix Table 10. Under-5 mortality reference sources by source date and location, 1950-2016			
**Location**	**Source**	**Source years**	**Method**
New Hampshire	1968-2014	Vital Registration	VR/SRS/DSP
New Jersey	1968-2014	Vital Registration	VR/SRS/DSP
New Mexico	1968-2014	Vital Registration	VR/SRS/DSP
New York	1968-2014	Vital Registration	VR/SRS/DSP
North Carolina	1968-2014	Vital Registration	VR/SRS/DSP
North Dakota	1968-2014	Vital Registration	VR/SRS/DSP
Ohio	1968-2014	Vital Registration	VR/SRS/DSP
Oklahoma	1968-2014	Vital Registration	VR/SRS/DSP
Oregon	1968-2014	Vital Registration	VR/SRS/DSP
Pennsylvania	1968-2014	Vital Registration	VR/SRS/DSP
Rhode Island	1968-2014	Vital Registration	VR/SRS/DSP
South Carolina	1968-2014	Vital Registration	VR/SRS/DSP
South Dakota	1968-2014	Vital Registration	VR/SRS/DSP
Tennessee	1968-2014	Vital Registration	VR/SRS/DSP
Texas	1968-2014	Vital Registration	VR/SRS/DSP
Utah	1968-2014	Vital Registration	VR/SRS/DSP
Vermont	1968-2014	Vital Registration	VR/SRS/DSP
Virginia	1968-2014	Vital Registration	VR/SRS/DSP
Washington	1968-2014	Vital Registration	VR/SRS/DSP
West Virginia	1968-2014	Vital Registration	VR/SRS/DSP
Wisconsin	1968-2014	Vital Registration	VR/SRS/DSP
Wyoming	1968-2014	Vital Registration	VR/SRS/DSP
Antigua and Barbuda	1950-2014	Vital Registration	VR/SRS/DSP
The Bahamas	1965-2013	Vital Registration	VR/SRS/DSP
Barbados	1950-2013	Vital Registration	VR/SRS/DSP
Belize	1991	Census	SBH
Belize	2000	Census	SBH
Belize	2006	Multiple Indicator Cluster Survey (MICS)	SBH
Belize	2011	Multiple Indicator Cluster Survey (MICS)	SBH
Belize	1991	Reproductive Health Survey (RHS)	CBH
Belize	1991	Reproductive Health Survey (RHS)	SBH
Belize	1999	Reproductive Health Survey (RHS)	CBH
Belize	1999	Reproductive Health Survey (RHS)	SBH
Bermuda	1950-2015	Vital Registration	VR/SRS/DSP
Cuba	1959-2014	Vital Registration	VR/SRS/DSP
Dominica	1950-2014	Vital Registration	VR/SRS/DSP
Dominican Republic	1986	Demographic and Health Survey (DHS)	CBH
Dominican Republic	1991	Demographic and Health Survey (DHS)	CBH
Dominican Republic	1996	Demographic and Health Survey (DHS)	CBH
Dominican Republic	2002	Demographic and Health Survey (DHS)	CBH
Dominican Republic	2007	Demographic and Health Survey (DHS)	CBH
Dominican Republic	2013	Demographic and Health Survey (DHS)	CBH
Grenada	1950-2015	Vital Registration	VR/SRS/DSP
Guyana	2009	Demographic and Health Survey (DHS)	CBH
Haiti	1994	Demographic and Health Survey (DHS)	CBH
Haiti	2000	Demographic and Health Survey (DHS)	CBH
Haiti	2005	Demographic and Health Survey (DHS)	CBH
Haiti	2012	Demographic and Health Survey (DHS)	CBH
Jamaica	1982	Census (IPUMS)	SBH
Jamaica	2001	Census (IPUMS)	SBH
Jamaica	1999	Multiple Indicator Cluster Survey (MICS)	SBH
Jamaica	2005	Multiple Indicator Cluster Survey (MICS)	SBH
Jamaica	2008	Reproductive Health Survey (RHS)	CBH
Puerto Rico	1950-2014	Vital Registration	VR/SRS/DSP
Saint Lucia	1950-2014	Vital Registration	VR/SRS/DSP
Saint Vincent and the Grenadines	1950-2015	Vital Registration	VR/SRS/DSP
Suriname	1999	Multiple Indicator Cluster Survey (MICS)	SBH
Suriname	2006	Multiple Indicator Cluster Survey (MICS)	SBH
Trinidad and Tobago	1987	Demographic and Health Survey (DHS)	CBH
Virgin Islands, U.S.	1950-2012	Vital Registration	VR/SRS/DSP
Bolivia	1989	Demographic and Health Survey (DHS)	CBH
Bolivia	1993	Demographic and Health Survey (DHS)	CBH
Bolivia	1998	Demographic and Health Survey (DHS)	CBH
Bolivia	2003	Demographic and Health Survey (DHS)	CBH
Bolivia	2008	Demographic and Health Survey (DHS)	CBH
Ecuador	1987	Demographic and Health Survey (DHS)	CBH
Ecuador	1989	Reproductive Health Survey (RHS)	CBH

Appendix Table 10. Under-5 mortality reference sources by source date and location, 1950-2016			
**Location**	**Source**	**Source years**	**Method**
Ecuador	1994	Reproductive Health Survey (RHS)	CBH
Ecuador	1999	Reproductive Health Survey (RHS)	CBH
Ecuador	2004	Reproductive Health Survey (RHS)	CBH
Peru	1986	Demographic and Health Survey (DHS)	CBH
Peru	1991	Demographic and Health Survey (DHS)	CBH
Peru	1996	Demographic and Health Survey (DHS)	CBH
Peru	2000	Demographic and Health Survey (DHS)	CBH
Peru	2003	Demographic and Health Survey (DHS)	CBH
Peru	2004	Demographic and Health Survey (DHS)	CBH
Peru	2009	Demographic and Health Survey (DHS)	CBH
Peru	2010	Demographic and Health Survey (DHS)	CBH
Peru	2011	Demographic and Health Survey (DHS)	CBH
Peru	2012	Demographic and Health Survey (DHS)	CBH
Peru	2013	Demographic and Health Survey (DHS)	CBH
Peru	2014	Demographic and Health Survey (DHS)	CBH
Colombia	1986	Demographic and Health Survey (DHS)	CBH
Colombia	1990	Demographic and Health Survey (DHS)	CBH
Colombia	1995	Demographic and Health Survey (DHS)	CBH
Colombia	2000	Demographic and Health Survey (DHS)	CBH
Colombia	2004	Demographic and Health Survey (DHS)	CBH
Colombia	2009	Demographic and Health Survey (DHS)	CBH
Costa Rica	1973	Census (IPUMS)	SBH
Costa Rica	1984	Census (IPUMS)	SBH
Costa Rica	2000	Census (IPUMS)	SBH
Costa Rica	1986	Reproductive Health Survey (RHS)	SBH
Costa Rica	1986	Reproductive Health Survey (RHS)	CBH
Costa Rica	1992	Reproductive Health Survey (RHS)	CBH
Costa Rica	1992	Reproductive Health Survey (RHS)	SBH
El Salvador	1985	Demographic and Health Survey (DHS)	CBH
Guatemala	1987	Demographic and Health Survey (DHS)	CBH
Guatemala	1995	Demographic and Health Survey (DHS)	CBH
Guatemala	1998	Demographic and Health Survey (DHS)	CBH
Guatemala	2014	Demographic and Health Survey (DHS)	CBH
Guatemala	2002	Reproductive Health Survey (RHS)	CBH
Guatemala	2008	Reproductive Health Survey (RHS)	CBH
Honduras	2005	Demographic and Health Survey (DHS)	CBH
Honduras	2011	Demographic and Health Survey (DHS)	CBH
Mexico	1987	Demographic and Health Survey (DHS)	CBH
Aguascalientes	1990	Census	SBH
Aguascalientes	2005	Census	SBH
Aguascalientes	2010	Census	SBH
Aguascalientes	2015	Census	SBH
Aguascalientes	1990	Census (IPUMS)	SBH
Aguascalientes	2000	Census (IPUMS)	SBH
Aguascalientes	2005	Census (IPUMS)	SBH
Aguascalientes	2009	Mexico Household Income and Expenditure Survey (ENIGH)	SBH
Aguascalientes	1991	National Survey of Demographic Dynamics (ENADID)	SBH
Aguascalientes	1992	National Survey of Demographic Dynamics (ENADID)	CBH
Aguascalientes	2006	National Survey of Demographic Dynamics (ENADID)	SBH
Aguascalientes	2009	National Survey of Demographic Dynamics (ENADID)	SBH
Aguascalientes	2014	National Survey of Demographic Dynamics (ENADID)	SBH
Baja California	1979-2015	Vital Registration	VR/SRS/DSP
Baja California Sur	1979-2015	Vital Registration	VR/SRS/DSP
Campeche	1990	Census	SBH
Campeche	2005	Census	SBH
Campeche	2010	Census	SBH
Campeche	2015	Census	SBH
Campeche	1990	Census (IPUMS)	SBH
Campeche	2000	Census (IPUMS)	SBH
Campeche	2005	Census (IPUMS)	SBH
Campeche	2009	Mexico Household Income and Expenditure Survey (ENIGH)	SBH
Campeche	1991	National Survey of Demographic Dynamics (ENADID)	SBH
Campeche	1992	National Survey of Demographic Dynamics (ENADID)	CBH
Campeche	2006	National Survey of Demographic Dynamics (ENADID)	SBH
Campeche	2009	National Survey of Demographic Dynamics (ENADID)	SBH
Campeche	2014	National Survey of Demographic Dynamics (ENADID)	SBH
Chiapas	1990	Census	SBH

Appendix Table 10. Under-5 mortality reference sources by source date and location, 1950-2016			
**Location**	**Source**	**Source years**	**Method**
Chiapas	2005	Census	SBH
Chiapas	2010	Census	SBH
Chiapas	2015	Census	SBH
Chiapas	1990	Census (IPUMS)	SBH
Chiapas	2000	Census (IPUMS)	SBH
Chiapas	2005	Census (IPUMS)	SBH
Chiapas	2009	Mexico Household Income and Expenditure Survey (ENIGH)	SBH
Chiapas	1991	National Survey of Demographic Dynamics (ENADID)	SBH
Chiapas	1992	National Survey of Demographic Dynamics (ENADID)	CBH
Chiapas	2006	National Survey of Demographic Dynamics (ENADID)	SBH
Chiapas	2009	National Survey of Demographic Dynamics (ENADID)	SBH
Chiapas	2014	National Survey of Demographic Dynamics (ENADID)	SBH
Chihuahua	1990	Census	SBH
Chihuahua	2005	Census	SBH
Chihuahua	2010	Census	SBH
Chihuahua	2015	Census	SBH
Chihuahua	1990	Census (IPUMS)	SBH
Chihuahua	2000	Census (IPUMS)	SBH
Chihuahua	2005	Census (IPUMS)	SBH
Chihuahua	2009	Mexico Household Income and Expenditure Survey (ENIGH)	SBH
Chihuahua	1991	National Survey of Demographic Dynamics (ENADID)	SBH
Chihuahua	1992	National Survey of Demographic Dynamics (ENADID)	CBH
Chihuahua	2006	National Survey of Demographic Dynamics (ENADID)	SBH
Chihuahua	2009	National Survey of Demographic Dynamics (ENADID)	SBH
Chihuahua	2014	National Survey of Demographic Dynamics (ENADID)	SBH
Coahuila	1990	Census	SBH
Coahuila	2005	Census	SBH
Coahuila	2010	Census	SBH
Coahuila	2015	Census	SBH
Coahuila	1990	Census (IPUMS)	SBH
Coahuila	2000	Census (IPUMS)	SBH
Coahuila	2005	Census (IPUMS)	SBH
Coahuila	2009	Mexico Household Income and Expenditure Survey (ENIGH)	SBH
Coahuila	1991	National Survey of Demographic Dynamics (ENADID)	SBH
Coahuila	1992	National Survey of Demographic Dynamics (ENADID)	CBH
Coahuila	2006	National Survey of Demographic Dynamics (ENADID)	SBH
Coahuila	2009	National Survey of Demographic Dynamics (ENADID)	SBH
Coahuila	2014	National Survey of Demographic Dynamics (ENADID)	SBH
Colima	1990	Census	SBH
Colima	2005	Census	SBH
Colima	2010	Census	SBH
Colima	2015	Census	SBH
Colima	1990	Census (IPUMS)	SBH
Colima	2000	Census (IPUMS)	SBH
Colima	2005	Census (IPUMS)	SBH
Colima	2009	Mexico Household Income and Expenditure Survey (ENIGH)	SBH
Colima	1991	National Survey of Demographic Dynamics (ENADID)	SBH
Colima	1992	National Survey of Demographic Dynamics (ENADID)	CBH
Colima	2006	National Survey of Demographic Dynamics (ENADID)	SBH
Colima	2009	National Survey of Demographic Dynamics (ENADID)	SBH
Colima	2014	National Survey of Demographic Dynamics (ENADID)	SBH
Distrito Federal	1979-2015	Vital Registration	VR/SRS/DSP
Durango	1990	Census	SBH
Durango	2005	Census	SBH
Durango	2010	Census	SBH
Durango	2015	Census	SBH
Durango	1990	Census (IPUMS)	SBH
Durango	2000	Census (IPUMS)	SBH
Durango	2005	Census (IPUMS)	SBH
Durango	2009	Mexico Household Income and Expenditure Survey (ENIGH)	SBH
Durango	1991	National Survey of Demographic Dynamics (ENADID)	SBH
Durango	1992	National Survey of Demographic Dynamics (ENADID)	CBH
Durango	2006	National Survey of Demographic Dynamics (ENADID)	SBH
Durango	2009	National Survey of Demographic Dynamics (ENADID)	SBH
Durango	2014	National Survey of Demographic Dynamics (ENADID)	SBH
Guanajuato	1990	Census	SBH
Guanajuato	2005	Census	SBH

Appendix Table 10. Under-5 mortality reference sources by source date and location, 1950-2016			
**Location**	**Source**	**Source years**	**Method**
Guanajuato	2010	Census	SBH
Guanajuato	2015	Census	SBH
Guanajuato	1990	Census (IPUMS)	SBH
Guanajuato	2000	Census (IPUMS)	SBH
Guanajuato	2005	Census (IPUMS)	SBH
Guanajuato	2009	Mexico Household Income and Expenditure Survey (ENIGH)	SBH
Guanajuato	1991	National Survey of Demographic Dynamics (ENADID)	SBH
Guanajuato	1992	National Survey of Demographic Dynamics (ENADID)	CBH
Guanajuato	2006	National Survey of Demographic Dynamics (ENADID)	SBH
Guanajuato	2009	National Survey of Demographic Dynamics (ENADID)	SBH
Guanajuato	2014	National Survey of Demographic Dynamics (ENADID)	SBH
Guerrero	1990	Census	SBH
Guerrero	2005	Census	SBH
Guerrero	2010	Census	SBH
Guerrero	2015	Census	SBH
Guerrero	1990	Census (IPUMS)	SBH
Guerrero	2000	Census (IPUMS)	SBH
Guerrero	2005	Census (IPUMS)	SBH
Guerrero	2009	Mexico Household Income and Expenditure Survey (ENIGH)	SBH
Guerrero	1991	National Survey of Demographic Dynamics (ENADID)	SBH
Guerrero	1992	National Survey of Demographic Dynamics (ENADID)	CBH
Guerrero	2006	National Survey of Demographic Dynamics (ENADID)	SBH
Guerrero	2009	National Survey of Demographic Dynamics (ENADID)	SBH
Guerrero	2014	National Survey of Demographic Dynamics (ENADID)	SBH
Hidalgo	1990	Census	SBH
Hidalgo	2005	Census	SBH
Hidalgo	2010	Census	SBH
Hidalgo	1990	Census (IPUMS)	SBH
Hidalgo	2000	Census (IPUMS)	SBH
Hidalgo	2005	Census (IPUMS)	SBH
Hidalgo	2009	Mexico Household Income and Expenditure Survey (ENIGH)	SBH
Hidalgo	1991	National Survey of Demographic Dynamics (ENADID)	SBH
Hidalgo	1992	National Survey of Demographic Dynamics (ENADID)	CBH
Hidalgo	2006	National Survey of Demographic Dynamics (ENADID)	SBH
Hidalgo	2009	National Survey of Demographic Dynamics (ENADID)	SBH
Hidalgo	2014	National Survey of Demographic Dynamics (ENADID)	SBH
Jalisco	1990	Census	SBH
Jalisco	2005	Census	SBH
Jalisco	2010	Census	SBH
Jalisco	2015	Census	SBH
Jalisco	1990	Census (IPUMS)	SBH
Jalisco	2000	Census (IPUMS)	SBH
Jalisco	2005	Census (IPUMS)	SBH
Jalisco	2009	Mexico Household Income and Expenditure Survey (ENIGH)	SBH
Jalisco	1991	National Survey of Demographic Dynamics (ENADID)	SBH
Jalisco	1992	National Survey of Demographic Dynamics (ENADID)	CBH
Jalisco	2006	National Survey of Demographic Dynamics (ENADID)	SBH
Jalisco	2009	National Survey of Demographic Dynamics (ENADID)	SBH
Jalisco	2014	National Survey of Demographic Dynamics (ENADID)	SBH
Mexico	1979-2015	Vital Registration	VR/SRS/DSP
Michoacan de Ocampo	1990	Census	SBH
Michoacan de Ocampo	2005	Census	SBH
Michoacan de Ocampo	2010	Census	SBH
Michoacan de Ocampo	2015	Census	SBH
Michoacan de Ocampo	1990	Census (IPUMS)	SBH
Michoacan de Ocampo	2000	Census (IPUMS)	SBH
Michoacan de Ocampo	2005	Census (IPUMS)	SBH
Michoacan de Ocampo	2009	Mexico Household Income and Expenditure Survey (ENIGH)	SBH
Michoacan de Ocampo	1991	National Survey of Demographic Dynamics (ENADID)	SBH
Michoacan de Ocampo	1992	National Survey of Demographic Dynamics (ENADID)	CBH
Michoacan de Ocampo	2006	National Survey of Demographic Dynamics (ENADID)	SBH
Michoacan de Ocampo	2009	National Survey of Demographic Dynamics (ENADID)	SBH
Michoacan de Ocampo	2014	National Survey of Demographic Dynamics (ENADID)	SBH
Morelos	1990	Census	SBH
Morelos	2005	Census	SBH
Morelos	2010	Census	SBH
Morelos	2015	Census	SBH

Appendix Table 10. Under-5 mortality reference sources by source date and location, 1950-2016			
**Location**	**Source**	**Source years**	**Method**
Morelos	1990	Census (IPUMS)	SBH
Morelos	2000	Census (IPUMS)	SBH
Morelos	2005	Census (IPUMS)	SBH
Morelos	2009	Mexico Household Income and Expenditure Survey (ENIGH)	SBH
Morelos	1991	National Survey of Demographic Dynamics (ENADID)	SBH
Morelos	1992	National Survey of Demographic Dynamics (ENADID)	CBH
Morelos	2006	National Survey of Demographic Dynamics (ENADID)	SBH
Morelos	2009	National Survey of Demographic Dynamics (ENADID)	SBH
Morelos	2014	National Survey of Demographic Dynamics (ENADID)	SBH
Nayarit	1990	Census	SBH
Nayarit	2005	Census	SBH
Nayarit	2010	Census	SBH
Nayarit	2015	Census	SBH
Nayarit	1990	Census (IPUMS)	SBH
Nayarit	2000	Census (IPUMS)	SBH
Nayarit	2005	Census (IPUMS)	SBH
Nayarit	2009	Mexico Household Income and Expenditure Survey (ENIGH)	SBH
Nayarit	1991	National Survey of Demographic Dynamics (ENADID)	SBH
Nayarit	1992	National Survey of Demographic Dynamics (ENADID)	CBH
Nayarit	2006	National Survey of Demographic Dynamics (ENADID)	SBH
Nayarit	2009	National Survey of Demographic Dynamics (ENADID)	SBH
Nayarit	2014	National Survey of Demographic Dynamics (ENADID)	SBH
Nuevo Leon	1990	Census	SBH
Nuevo Leon	2005	Census	SBH
Nuevo Leon	2010	Census	SBH
Nuevo Leon	2015	Census	SBH
Nuevo Leon	1990	Census (IPUMS)	SBH
Nuevo Leon	2000	Census (IPUMS)	SBH
Nuevo Leon	2005	Census (IPUMS)	SBH
Nuevo Leon	2009	Mexico Household Income and Expenditure Survey (ENIGH)	SBH
Nuevo Leon	1991	National Survey of Demographic Dynamics (ENADID)	SBH
Nuevo Leon	1992	National Survey of Demographic Dynamics (ENADID)	CBH
Nuevo Leon	2006	National Survey of Demographic Dynamics (ENADID)	SBH
Nuevo Leon	2009	National Survey of Demographic Dynamics (ENADID)	SBH
Nuevo Leon	2014	National Survey of Demographic Dynamics (ENADID)	SBH
Oaxaca	1990	Census	SBH
Oaxaca	2005	Census	SBH
Oaxaca	2010	Census	SBH
Oaxaca	2015	Census	SBH
Oaxaca	1990	Census (IPUMS)	SBH
Oaxaca	2000	Census (IPUMS)	SBH
Oaxaca	2005	Census (IPUMS)	SBH
Oaxaca	2009	Mexico Household Income and Expenditure Survey (ENIGH)	SBH
Oaxaca	1991	National Survey of Demographic Dynamics (ENADID)	SBH
Oaxaca	1992	National Survey of Demographic Dynamics (ENADID)	CBH
Oaxaca	2006	National Survey of Demographic Dynamics (ENADID)	SBH
Oaxaca	2009	National Survey of Demographic Dynamics (ENADID)	SBH
Oaxaca	2014	National Survey of Demographic Dynamics (ENADID)	SBH
Puebla	1990	Census	SBH
Puebla	2005	Census	SBH
Puebla	2010	Census	SBH
Puebla	2015	Census	SBH
Puebla	1990	Census (IPUMS)	SBH
Puebla	2000	Census (IPUMS)	SBH
Puebla	2005	Census (IPUMS)	SBH
Puebla	2009	Mexico Household Income and Expenditure Survey (ENIGH)	SBH
Puebla	1991	National Survey of Demographic Dynamics (ENADID)	SBH
Puebla	1992	National Survey of Demographic Dynamics (ENADID)	CBH
Puebla	2006	National Survey of Demographic Dynamics (ENADID)	SBH
Puebla	2009	National Survey of Demographic Dynamics (ENADID)	SBH
Puebla	2014	National Survey of Demographic Dynamics (ENADID)	SBH
Queretaro	1979-2015	Vital Registration	VR/SRS/DSP
Quintana Roo	1990	Census	SBH
Quintana Roo	2005	Census	SBH
Quintana Roo	2010	Census	SBH
Quintana Roo	2015	Census	SBH
Quintana Roo	1990	Census (IPUMS)	SBH

Appendix Table 10. Under-5 mortality reference sources by source date and location, 1950-2016			
**Location**	**Source**	**Source years**	**Method**
Quintana Roo	2000	Census (IPUMS)	SBH
Quintana Roo	2005	Census (IPUMS)	SBH
Quintana Roo	2009	Mexico Household Income and Expenditure Survey (ENIGH)	SBH
Quintana Roo	1991	National Survey of Demographic Dynamics (ENADID)	SBH
Quintana Roo	1992	National Survey of Demographic Dynamics (ENADID)	CBH
Quintana Roo	2006	National Survey of Demographic Dynamics (ENADID)	SBH
Quintana Roo	2009	National Survey of Demographic Dynamics (ENADID)	SBH
Quintana Roo	2014	National Survey of Demographic Dynamics (ENADID)	SBH
San Luis Potosi	1990	Census	SBH
San Luis Potosi	2005	Census	SBH
San Luis Potosi	2010	Census	SBH
San Luis Potosi	2015	Census	SBH
San Luis Potosi	1990	Census (IPUMS)	SBH
San Luis Potosi	2000	Census (IPUMS)	SBH
San Luis Potosi	2005	Census (IPUMS)	SBH
San Luis Potosi	2009	Mexico Household Income and Expenditure Survey (ENIGH)	SBH
San Luis Potosi	1991	National Survey of Demographic Dynamics (ENADID)	SBH
San Luis Potosi	1992	National Survey of Demographic Dynamics (ENADID)	CBH
San Luis Potosi	2006	National Survey of Demographic Dynamics (ENADID)	SBH
San Luis Potosi	2009	National Survey of Demographic Dynamics (ENADID)	SBH
San Luis Potosi	2014	National Survey of Demographic Dynamics (ENADID)	SBH
Sinaloa	1990	Census	SBH
Sinaloa	2005	Census	SBH
Sinaloa	2010	Census	SBH
Sinaloa	2015	Census	SBH
Sinaloa	1990	Census (IPUMS)	SBH
Sinaloa	2000	Census (IPUMS)	SBH
Sinaloa	2005	Census (IPUMS)	SBH
Sinaloa	2009	Mexico Household Income and Expenditure Survey (ENIGH)	SBH
Sinaloa	1991	National Survey of Demographic Dynamics (ENADID)	SBH
Sinaloa	1992	National Survey of Demographic Dynamics (ENADID)	CBH
Sinaloa	2006	National Survey of Demographic Dynamics (ENADID)	SBH
Sinaloa	2009	National Survey of Demographic Dynamics (ENADID)	SBH
Sinaloa	2014	National Survey of Demographic Dynamics (ENADID)	SBH
Sonora	1990	Census	SBH
Sonora	2005	Census	SBH
Sonora	2010	Census	SBH
Sonora	2015	Census	SBH
Sonora	1990	Census (IPUMS)	SBH
Sonora	2000	Census (IPUMS)	SBH
Sonora	2005	Census (IPUMS)	SBH
Sonora	1991	National Survey of Demographic Dynamics (ENADID)	SBH
Sonora	1992	National Survey of Demographic Dynamics (ENADID)	CBH
Sonora	2006	National Survey of Demographic Dynamics (ENADID)	SBH
Sonora	2009	National Survey of Demographic Dynamics (ENADID)	SBH
Sonora	2014	National Survey of Demographic Dynamics (ENADID)	SBH
Tabasco	1990	Census	SBH
Tabasco	2005	Census	SBH
Tabasco	2010	Census	SBH
Tabasco	2015	Census	SBH
Tabasco	1990	Census (IPUMS)	SBH
Tabasco	2000	Census (IPUMS)	SBH
Tabasco	2005	Census (IPUMS)	SBH
Tabasco	2009	Mexico Household Income and Expenditure Survey (ENIGH)	SBH
Tabasco	1991	National Survey of Demographic Dynamics (ENADID)	SBH
Tabasco	1992	National Survey of Demographic Dynamics (ENADID)	CBH
Tabasco	2006	National Survey of Demographic Dynamics (ENADID)	SBH
Tabasco	2009	National Survey of Demographic Dynamics (ENADID)	SBH
Tabasco	2014	National Survey of Demographic Dynamics (ENADID)	SBH
Tamaulipas	1990	Census	SBH
Tamaulipas	2005	Census	SBH
Tamaulipas	2010	Census	SBH
Tamaulipas	2015	Census	SBH
Tamaulipas	1990	Census (IPUMS)	SBH
Tamaulipas	2000	Census (IPUMS)	SBH
Tamaulipas	2005	Census (IPUMS)	SBH
Tamaulipas	2009	Mexico Household Income and Expenditure Survey (ENIGH)	SBH

Appendix Table 10. Under-5 mortality reference sources by source date and location, 1950-2016			
Location	Source	Source years	Method
Tamaulipas	1991	National Survey of Demographic Dynamics (ENADID)	SBH
Tamaulipas	1992	National Survey of Demographic Dynamics (ENADID)	CBH
Tamaulipas	2006	National Survey of Demographic Dynamics (ENADID)	SBH
Tamaulipas	2009	National Survey of Demographic Dynamics (ENADID)	SBH
Tamaulipas	2014	National Survey of Demographic Dynamics (ENADID)	SBH
Tlaxcala	1979-2015	Vital Registration	VR/SRS/DSP
Veracruz de Ignacio de la Llave	1990	Census	SBH
Veracruz de Ignacio de la Llave	2005	Census	SBH
Veracruz de Ignacio de la Llave	2010	Census	SBH
Veracruz de Ignacio de la Llave	2015	Census	SBH
Veracruz de Ignacio de la Llave	1990	Census (IPUMS)	SBH
Veracruz de Ignacio de la Llave	2000	Census (IPUMS)	SBH
Veracruz de Ignacio de la Llave	2005	Census (IPUMS)	SBH
Veracruz de Ignacio de la Llave	2009	Mexico Household Income and Expenditure Survey (ENIGH)	SBH
Veracruz de Ignacio de la Llave	1991	National Survey of Demographic Dynamics (ENADID)	SBH
Veracruz de Ignacio de la Llave	1992	National Survey of Demographic Dynamics (ENADID)	CBH
Veracruz de Ignacio de la Llave	2006	National Survey of Demographic Dynamics (ENADID)	SBH
Veracruz de Ignacio de la Llave	2009	National Survey of Demographic Dynamics (ENADID)	SBH
Veracruz de Ignacio de la Llave	2014	National Survey of Demographic Dynamics (ENADID)	SBH
Yucatan	1990	Census	SBH
Yucatan	2005	Census	SBH
Yucatan	2010	Census	SBH
Yucatan	2015	Census	SBH
Yucatan	1990	Census (IPUMS)	SBH
Yucatan	2000	Census (IPUMS)	SBH
Yucatan	2005	Census (IPUMS)	SBH
Yucatan	2009	Mexico Household Income and Expenditure Survey (ENIGH)	SBH
Yucatan	1991	National Survey of Demographic Dynamics (ENADID)	SBH
Yucatan	1992	National Survey of Demographic Dynamics (ENADID)	CBH
Yucatan	2006	National Survey of Demographic Dynamics (ENADID)	SBH
Yucatan	2009	National Survey of Demographic Dynamics (ENADID)	SBH
Yucatan	2014	National Survey of Demographic Dynamics (ENADID)	SBH
Zacatecas	1990	Census	SBH
Zacatecas	2005	Census	SBH
Zacatecas	2010	Census	SBH
Zacatecas	2015	Census	SBH
Zacatecas	1990	Census (IPUMS)	SBH
Zacatecas	2000	Census (IPUMS)	SBH
Zacatecas	2005	Census (IPUMS)	SBH
Zacatecas	2009	Mexico Household Income and Expenditure Survey (ENIGH)	SBH
Zacatecas	1991	National Survey of Demographic Dynamics (ENADID)	SBH
Zacatecas	1992	National Survey of Demographic Dynamics (ENADID)	CBH
Zacatecas	2006	National Survey of Demographic Dynamics (ENADID)	SBH
Zacatecas	2009	National Survey of Demographic Dynamics (ENADID)	SBH
Zacatecas	2014	National Survey of Demographic Dynamics (ENADID)	SBH
Nicaragua	1997	Demographic and Health Survey (DHS)	CBH
Nicaragua	2001	Demographic and Health Survey (DHS)	CBH
Panama	1990	Census (IPUMS)	SBH
Panama	2000	Census (IPUMS)	SBH
Panama	2010	Census (IPUMS)	SBH
Panama	1997	Living Standards Measurement Study (LSMS)	SBH
Panama	2003	Living Standards Measurement Study (LSMS)	SBH
Venezuela	1950-2013	Vital Registration	VR/SRS/DSP
Brazil	1996	Demographic and Health Survey (DHS)	CBH
Acre	2005	Brazil National Household Sample Survey (PNAD)	SBH
Acre	2006	Brazil National Household Sample Survey (PNAD)	SBH
Acre	2007	Brazil National Household Sample Survey (PNAD)	SBH
Acre	2008	Brazil National Household Sample Survey (PNAD)	SBH
Acre	2009	Brazil National Household Sample Survey (PNAD)	SBH
Alagoas	2005	Brazil National Household Sample Survey (PNAD)	SBH
Amapa	2008	Brazil National Household Sample Survey (PNAD)	SBH
Amazonas	1996	Demographic and Health Survey (DHS)	CBH
Bahia	1992	Brazil National Household Sample Survey (PNAD)	SBH
Bahia	1993	Brazil National Household Sample Survey (PNAD)	SBH
Bahia	1995	Brazil National Household Sample Survey (PNAD)	SBH
Bahia	1996	Brazil National Household Sample Survey (PNAD)	SBH
Bahia	1997	Brazil National Household Sample Survey (PNAD)	SBH

Appendix Table 10. Under-5 mortality reference sources by source date and location, 1950-2016			
**Location**	**Source**	**Source years**	**Method**
Bahia	1998	Brazil National Household Sample Survey (PNAD)	SBH
Bahia	1999	Brazil National Household Sample Survey (PNAD)	SBH
Bahia	2001	Brazil National Household Sample Survey (PNAD)	SBH
Bahia	2002	Brazil National Household Sample Survey (PNAD)	SBH
Bahia	2003	Brazil National Household Sample Survey (PNAD)	SBH
Bahia	2004	Brazil National Household Sample Survey (PNAD)	SBH
Bahia	2005	Brazil National Household Sample Survey (PNAD)	SBH
Bahia	2006	Brazil National Household Sample Survey (PNAD)	SBH
Bahia	2007	Brazil National Household Sample Survey (PNAD)	SBH
Bahia	2008	Brazil National Household Sample Survey (PNAD)	SBH
Bahia	2009	Brazil National Household Sample Survey (PNAD)	SBH
Ceara	1991	Demographic and Health Survey (DHS)	CBH
Ceara	1996	Demographic and Health Survey (DHS)	CBH
Espirito Santo	1992	Brazil National Household Sample Survey (PNAD)	SBH
Espirito Santo	1993	Brazil National Household Sample Survey (PNAD)	SBH
Espirito Santo	1995	Brazil National Household Sample Survey (PNAD)	SBH
Espirito Santo	1996	Brazil National Household Sample Survey (PNAD)	SBH
Espirito Santo	1997	Brazil National Household Sample Survey (PNAD)	SBH
Espirito Santo	1998	Brazil National Household Sample Survey (PNAD)	SBH
Espirito Santo	1999	Brazil National Household Sample Survey (PNAD)	SBH
Espirito Santo	2001	Brazil National Household Sample Survey (PNAD)	SBH
Espirito Santo	2002	Brazil National Household Sample Survey (PNAD)	SBH
Espirito Santo	2003	Brazil National Household Sample Survey (PNAD)	SBH
Espirito Santo	2004	Brazil National Household Sample Survey (PNAD)	SBH
Espirito Santo	2005	Brazil National Household Sample Survey (PNAD)	SBH
Espirito Santo	2006	Brazil National Household Sample Survey (PNAD)	SBH
Espirito Santo	2007	Brazil National Household Sample Survey (PNAD)	SBH
Espirito Santo	2008	Brazil National Household Sample Survey (PNAD)	SBH
Espirito Santo	2009	Brazil National Household Sample Survey (PNAD)	SBH
Goias	1992	Brazil National Household Sample Survey (PNAD)	SBH
Goias	1993	Brazil National Household Sample Survey (PNAD)	SBH
Goias	1995	Brazil National Household Sample Survey (PNAD)	SBH
Goias	1996	Brazil National Household Sample Survey (PNAD)	SBH
Goias	1997	Brazil National Household Sample Survey (PNAD)	SBH
Goias	1998	Brazil National Household Sample Survey (PNAD)	SBH
Goias	1999	Brazil National Household Sample Survey (PNAD)	SBH
Goias	2001	Brazil National Household Sample Survey (PNAD)	SBH
Goias	2002	Brazil National Household Sample Survey (PNAD)	SBH
Goias	2003	Brazil National Household Sample Survey (PNAD)	SBH
Goias	2004	Brazil National Household Sample Survey (PNAD)	SBH
Goias	2005	Brazil National Household Sample Survey (PNAD)	SBH
Goias	2006	Brazil National Household Sample Survey (PNAD)	SBH
Goias	2007	Brazil National Household Sample Survey (PNAD)	SBH
Goias	2008	Brazil National Household Sample Survey (PNAD)	SBH
Goias	2009	Brazil National Household Sample Survey (PNAD)	SBH
Maranhao	1992	Brazil National Household Sample Survey (PNAD)	SBH
Maranhao	1993	Brazil National Household Sample Survey (PNAD)	SBH
Maranhao	1995	Brazil National Household Sample Survey (PNAD)	SBH
Maranhao	1996	Brazil National Household Sample Survey (PNAD)	SBH
Maranhao	1997	Brazil National Household Sample Survey (PNAD)	SBH
Maranhao	1998	Brazil National Household Sample Survey (PNAD)	SBH
Maranhao	1999	Brazil National Household Sample Survey (PNAD)	SBH
Maranhao	2001	Brazil National Household Sample Survey (PNAD)	SBH
Maranhao	2002	Brazil National Household Sample Survey (PNAD)	SBH
Maranhao	2003	Brazil National Household Sample Survey (PNAD)	SBH
Maranhao	2004	Brazil National Household Sample Survey (PNAD)	SBH
Maranhao	2005	Brazil National Household Sample Survey (PNAD)	SBH
Maranhao	2006	Brazil National Household Sample Survey (PNAD)	SBH
Maranhao	2007	Brazil National Household Sample Survey (PNAD)	SBH
Maranhao	2008	Brazil National Household Sample Survey (PNAD)	SBH
Maranhao	2009	Brazil National Household Sample Survey (PNAD)	SBH
Mato Grosso	1992	Brazil National Household Sample Survey (PNAD)	SBH
Mato Grosso	1993	Brazil National Household Sample Survey (PNAD)	SBH
Mato Grosso	1995	Brazil National Household Sample Survey (PNAD)	SBH
Mato Grosso	1996	Brazil National Household Sample Survey (PNAD)	SBH
Mato Grosso	1997	Brazil National Household Sample Survey (PNAD)	SBH
Mato Grosso	1998	Brazil National Household Sample Survey (PNAD)	SBH

Appendix Table 10. Under-5 mortality reference sources by source date and location, 1950-2016			
Location	Source	Source years	Method
Mato Grosso	1999	Brazil National Household Sample Survey (PNAD)	SBH
Mato Grosso	2001	Brazil National Household Sample Survey (PNAD)	SBH
Mato Grosso	2002	Brazil National Household Sample Survey (PNAD)	SBH
Mato Grosso	2003	Brazil National Household Sample Survey (PNAD)	SBH
Mato Grosso	2004	Brazil National Household Sample Survey (PNAD)	SBH
Mato Grosso	2005	Brazil National Household Sample Survey (PNAD)	SBH
Mato Grosso	2006	Brazil National Household Sample Survey (PNAD)	SBH
Mato Grosso	2007	Brazil National Household Sample Survey (PNAD)	SBH
Mato Grosso	2008	Brazil National Household Sample Survey (PNAD)	SBH
Mato Grosso	2009	Brazil National Household Sample Survey (PNAD)	SBH
Mato Grosso do Sul	1992	Brazil National Household Sample Survey (PNAD)	SBH
Mato Grosso do Sul	1993	Brazil National Household Sample Survey (PNAD)	SBH
Mato Grosso do Sul	1995	Brazil National Household Sample Survey (PNAD)	SBH
Mato Grosso do Sul	1996	Brazil National Household Sample Survey (PNAD)	SBH
Mato Grosso do Sul	1997	Brazil National Household Sample Survey (PNAD)	SBH
Mato Grosso do Sul	1998	Brazil National Household Sample Survey (PNAD)	SBH
Mato Grosso do Sul	1999	Brazil National Household Sample Survey (PNAD)	SBH
Mato Grosso do Sul	2001	Brazil National Household Sample Survey (PNAD)	SBH
Mato Grosso do Sul	2002	Brazil National Household Sample Survey (PNAD)	SBH
Mato Grosso do Sul	2003	Brazil National Household Sample Survey (PNAD)	SBH
Mato Grosso do Sul	2004	Brazil National Household Sample Survey (PNAD)	SBH
Mato Grosso do Sul	2005	Brazil National Household Sample Survey (PNAD)	SBH
Mato Grosso do Sul	2006	Brazil National Household Sample Survey (PNAD)	SBH
Mato Grosso do Sul	2007	Brazil National Household Sample Survey (PNAD)	SBH
Mato Grosso do Sul	2008	Brazil National Household Sample Survey (PNAD)	SBH
Mato Grosso do Sul	2009	Brazil National Household Sample Survey (PNAD)	SBH
Minas Gerais	1992	Brazil National Household Sample Survey (PNAD)	SBH
Minas Gerais	1993	Brazil National Household Sample Survey (PNAD)	SBH
Minas Gerais	1995	Brazil National Household Sample Survey (PNAD)	SBH
Minas Gerais	1996	Brazil National Household Sample Survey (PNAD)	SBH
Minas Gerais	1997	Brazil National Household Sample Survey (PNAD)	SBH
Minas Gerais	1998	Brazil National Household Sample Survey (PNAD)	SBH
Minas Gerais	1999	Brazil National Household Sample Survey (PNAD)	SBH
Minas Gerais	2001	Brazil National Household Sample Survey (PNAD)	SBH
Minas Gerais	2002	Brazil National Household Sample Survey (PNAD)	SBH
Minas Gerais	2003	Brazil National Household Sample Survey (PNAD)	SBH
Minas Gerais	2004	Brazil National Household Sample Survey (PNAD)	SBH
Minas Gerais	2005	Brazil National Household Sample Survey (PNAD)	SBH
Minas Gerais	2006	Brazil National Household Sample Survey (PNAD)	SBH
Minas Gerais	2007	Brazil National Household Sample Survey (PNAD)	SBH
Minas Gerais	2008	Brazil National Household Sample Survey (PNAD)	SBH
Minas Gerais	2009	Brazil National Household Sample Survey (PNAD)	SBH
Para	2005	Brazil National Household Sample Survey (PNAD)	SBH
Para	2006	Brazil National Household Sample Survey (PNAD)	SBH
Para	2007	Brazil National Household Sample Survey (PNAD)	SBH
Para	2008	Brazil National Household Sample Survey (PNAD)	SBH
Para	2009	Brazil National Household Sample Survey (PNAD)	SBH
Paraiba	1991	Demographic and Health Survey (DHS)	CBH
Paraiba	1996	Demographic and Health Survey (DHS)	CBH
Parana	1996	Demographic and Health Survey (DHS)	CBH
Pernambuco	1992	Brazil National Household Sample Survey (PNAD)	SBH
Pernambuco	1993	Brazil National Household Sample Survey (PNAD)	SBH
Pernambuco	1995	Brazil National Household Sample Survey (PNAD)	SBH
Pernambuco	1996	Brazil National Household Sample Survey (PNAD)	SBH
Pernambuco	1997	Brazil National Household Sample Survey (PNAD)	SBH
Pernambuco	1998	Brazil National Household Sample Survey (PNAD)	SBH
Pernambuco	1999	Brazil National Household Sample Survey (PNAD)	SBH
Pernambuco	2001	Brazil National Household Sample Survey (PNAD)	SBH
Pernambuco	2002	Brazil National Household Sample Survey (PNAD)	SBH
Pernambuco	2003	Brazil National Household Sample Survey (PNAD)	SBH
Pernambuco	2004	Brazil National Household Sample Survey (PNAD)	SBH
Pernambuco	2005	Brazil National Household Sample Survey (PNAD)	SBH
Pernambuco	2006	Brazil National Household Sample Survey (PNAD)	SBH
Pernambuco	2007	Brazil National Household Sample Survey (PNAD)	SBH
Pernambuco	2008	Brazil National Household Sample Survey (PNAD)	SBH
Pernambuco	2009	Brazil National Household Sample Survey (PNAD)	SBH
Piaui	2000	Census	SBH

Appendix Table 10. Under-5 mortality reference sources by source date and location, 1950-2016			
**Location**	**Source**	**Source years**	**Method**
Piaui	1970	Census (IPUMS)	SBH
Piaui	1980	Census (IPUMS)	SBH
Piaui	1991	Census (IPUMS)	SBH
Rio de Janeiro	1979-2015	Vital Registration	VR/SRS/DSP
Rio Grande do Norte	2001	Brazil National Household Sample Survey (PNAD)	SBH
Rio Grande do Norte	2002	Brazil National Household Sample Survey (PNAD)	SBH
Rio Grande do Sul	1979-2015	Vital Registration	VR/SRS/DSP
Rondonia	2000	Census	SBH
Rondonia	1970	Census (IPUMS)	SBH
Rondonia	1980	Census (IPUMS)	SBH
Rondonia	1991	Census (IPUMS)	SBH
Roraima	2006	Brazil National Household Sample Survey (PNAD)	SBH
Santa Catarina	1992	Brazil National Household Sample Survey (PNAD)	SBH
Santa Catarina	1993	Brazil National Household Sample Survey (PNAD)	SBH
Santa Catarina	1995	Brazil National Household Sample Survey (PNAD)	SBH
Santa Catarina	1996	Brazil National Household Sample Survey (PNAD)	SBH
Santa Catarina	1997	Brazil National Household Sample Survey (PNAD)	SBH
Santa Catarina	1998	Brazil National Household Sample Survey (PNAD)	SBH
Santa Catarina	1999	Brazil National Household Sample Survey (PNAD)	SBH
Santa Catarina	2001	Brazil National Household Sample Survey (PNAD)	SBH
Santa Catarina	2002	Brazil National Household Sample Survey (PNAD)	SBH
Santa Catarina	2003	Brazil National Household Sample Survey (PNAD)	SBH
Santa Catarina	2004	Brazil National Household Sample Survey (PNAD)	SBH
Santa Catarina	2005	Brazil National Household Sample Survey (PNAD)	SBH
Santa Catarina	2006	Brazil National Household Sample Survey (PNAD)	SBH
Santa Catarina	2007	Brazil National Household Sample Survey (PNAD)	SBH
Santa Catarina	2008	Brazil National Household Sample Survey (PNAD)	SBH
Santa Catarina	2009	Brazil National Household Sample Survey (PNAD)	SBH
Sao Paulo	1979-2015	Vital Registration	VR/SRS/DSP
Sergipe	1992	Brazil National Household Sample Survey (PNAD)	SBH
Sergipe	1993	Brazil National Household Sample Survey (PNAD)	SBH
Sergipe	1995	Brazil National Household Sample Survey (PNAD)	SBH
Sergipe	1996	Brazil National Household Sample Survey (PNAD)	SBH
Sergipe	1997	Brazil National Household Sample Survey (PNAD)	SBH
Sergipe	1998	Brazil National Household Sample Survey (PNAD)	SBH
Sergipe	1999	Brazil National Household Sample Survey (PNAD)	SBH
Sergipe	2001	Brazil National Household Sample Survey (PNAD)	SBH
Sergipe	2002	Brazil National Household Sample Survey (PNAD)	SBH
Sergipe	2003	Brazil National Household Sample Survey (PNAD)	SBH
Sergipe	2004	Brazil National Household Sample Survey (PNAD)	SBH
Sergipe	2005	Brazil National Household Sample Survey (PNAD)	SBH
Sergipe	2006	Brazil National Household Sample Survey (PNAD)	SBH
Sergipe	2007	Brazil National Household Sample Survey (PNAD)	SBH
Sergipe	2008	Brazil National Household Sample Survey (PNAD)	SBH
Sergipe	2009	Brazil National Household Sample Survey (PNAD)	SBH
Tocantins	2005	Brazil National Household Sample Survey (PNAD)	SBH
Tocantins	2006	Brazil National Household Sample Survey (PNAD)	SBH
Tocantins	2007	Brazil National Household Sample Survey (PNAD)	SBH
Tocantins	2008	Brazil National Household Sample Survey (PNAD)	SBH
Tocantins	2009	Brazil National Household Sample Survey (PNAD)	SBH
Paraguay	1990	Demographic and Health Survey (DHS)	CBH
Paraguay	1995	Reproductive Health Survey (RHS)	CBH
Paraguay	1998	Reproductive Health Survey (RHS)	CBH
Paraguay	2004	Reproductive Health Survey (RHS)	CBH
Paraguay	2008	Reproductive Health Survey (RHS)	CBH
Afghanistan	2008	Afghanistan Living Conditions Survey	SBH
Afghanistan	2012	Afghanistan Living Conditions Survey	SBH
Afghanistan	2006	Health Survey	SBH
Afghanistan	2000	Multiple Indicator Cluster Survey (MICS)	SBH
Afghanistan	2003	Multiple Indicator Cluster Survey (MICS)	SBH
Afghanistan	2010	Multiple Indicator Cluster Survey (MICS)	SBH
Algeria	2000	Multiple Indicator Cluster Survey (MICS)	SBH
Algeria	2012	Multiple Indicator Cluster Survey (MICS)	SBH
Algeria	2012	Multiple Indicator Cluster Survey (MICS)	CBH
Algeria	1992	Pan Arab Project for Child Development (PAPCHILD)	SBH
Algeria	1992	Pan Arab Project for Child Development (PAPCHILD)	CBH
Algeria	2002	Pan Arab Project for Family Health (PAPFAM)	SBH

Appendix Table 10. Under-5 mortality reference sources by source date and location, 1950-2016			
**Location**	**Source**	**Source years**	**Method**
Algeria	2002	Pan Arab Project for Family Health (PAPFAM)	CBH
Bahrain	1980-2012	Vital Registration	VR/SRS/DSP
Egypt	1988	Demographic and Health Survey (DHS)	CBH
Egypt	1992	Demographic and Health Survey (DHS)	CBH
Egypt	1995	Demographic and Health Survey (DHS)	CBH
Egypt	1997	Demographic and Health Survey (DHS)	CBH
Egypt	2000	Demographic and Health Survey (DHS)	CBH
Egypt	2003	Demographic and Health Survey (DHS)	CBH
Egypt	2005	Demographic and Health Survey (DHS)	CBH
Egypt	2008	Demographic and Health Survey (DHS)	CBH
Egypt	2014	Demographic and Health Survey (DHS)	CBH
Egypt	1998	Interim Demographic and Health Survey (DHS)	CBH
Iran	2006	Census (IPUMS)	SBH
Iran	2010	Demographic and Health Survey (DHS)	CBH
Iran	1967	Health Survey	CBH
Iran	1969	Health Survey	CBH
Iran	1971	Health Survey	CBH
Iran	1973	Health Survey	CBH
Iran	1974	Health Survey	CBH
Iran	1975	Health Survey	CBH
Iran	1977	Health Survey	CBH
Iran	1979	Health Survey	CBH
Iran	1981	Health Survey	CBH
Iran	1983	Health Survey	CBH
Iran	1984	Health Survey	CBH
Iran	1985	Health Survey	CBH
Iran	1987	Health Survey	CBH
Iran	1989	Health Survey	CBH
Iran	1991	Health Survey	CBH
Iran	1993	Health Survey	CBH
Iran	1994	Health Survey	CBH
Iran	1995	Health Survey	CBH
Iran	1997	Health Survey	CBH
Iran	1999	Health Survey	SBH
Iran	1999	Health Survey	CBH
Iraq	2006	Multiple Indicator Cluster Survey (MICS)	SBH
Iraq	2006	Multiple Indicator Cluster Survey (MICS)	CBH
Iraq	2011	Multiple Indicator Cluster Survey (MICS)	SBH
Iraq	2011	Multiple Indicator Cluster Survey (MICS)	CBH
Jordan	1990	Demographic and Health Survey (DHS)	CBH
Jordan	1997	Demographic and Health Survey (DHS)	CBH
Jordan	2002	Demographic and Health Survey (DHS)	CBH
Jordan	2007	Demographic and Health Survey (DHS)	CBH
Jordan	2009	Demographic and Health Survey (DHS)	CBH
Jordan	2012	Demographic and Health Survey (DHS)	CBH
Kuwait	1962-2014	Vital Registration	VR/SRS/DSP
Lebanon	2000	Multiple Indicator Cluster Survey (MICS)	SBH
Lebanon	2009	Multiple Indicator Cluster Survey (MICS)	SBH
Lebanon	1996	Pan Arab Project for Child Development (PAPCHILD)	CBH
Lebanon	1996	Pan Arab Project for Child Development (PAPCHILD)	SBH
Lebanon	2004	Pan Arab Project for Family Health (PAPFAM)	SBH
Lebanon	2004	Pan Arab Project for Family Health (PAPFAM)	CBH
Lebanon	2011-2015	Vital Registration	VR/SRS/DSP
Libya	2003	Multiple Indicator Cluster Survey (MICS)	SBH
Libya	1995	Pan Arab Project for Child Development (PAPCHILD)	CBH
Libya	1995	Pan Arab Project for Child Development (PAPCHILD)	SBH
Libya	2007	Pan Arab Project for Family Health (PAPFAM)	SBH
Morocco	1987	Demographic and Health Survey (DHS)	CBH
Morocco	1992	Demographic and Health Survey (DHS)	CBH
Morocco	2003	Demographic and Health Survey (DHS)	CBH
Palestine	1997	Census (IPUMS)	SBH
Palestine	2007	Census (IPUMS)	SBH
Palestine	2004	Demographic and Health Survey (DHS)	SBH
Palestine	2000	Health Survey	SBH
Palestine	2000	Health Survey	CBH
Palestine	2001	Health Survey	CBH
Palestine	2010	Multiple Indicator Cluster Survey (MICS)	CBH

Appendix Table 10. Under-5 mortality reference sources by source date and location, 1950-2016			
**Location**	**Source**	**Source years**	**Method**
Palestine	2010	Multiple Indicator Cluster Survey (MICS)	SBH
Palestine	2014	Multiple Indicator Cluster Survey (MICS)	SBH
Palestine	2014	Multiple Indicator Cluster Survey (MICS)	CBH
Palestine	1992	National Demographic and Health Survey	CBH
Palestine	2004	National Demographic and Health Survey	CBH
Palestine	2004	Pan Arab Project for Family Health (PAPFAM)	CBH
Oman	2004-2013	Vital Registration	VR/SRS/DSP
Qatar	1987	Gulf Child Health Survey	CBH
Qatar	1987	Gulf Child Health Survey	SBH
Qatar	1998	Gulf Family Health Survey	CBH
Saudi Arabia	2004	Census	HH
Saudi Arabia	2006	Demographic Research Bulletin 2007	HH
Saudi Arabia	1988	Gulf Child Health Survey	SBH
Saudi Arabia	1996	Gulf Family Health Survey	CBH
Saudi Arabia	1990	Levels, Trends and Differentials of Infant and Child Mortality	SBH
'Asir	1999-2012	Vital Registration	VR/SRS/DSP
Bahah	1999-2012	Vital Registration	VR/SRS/DSP
Eastern Province	1999-2012	Vital Registration	VR/SRS/DSP
Ha'il	1999-2012	Vital Registration	VR/SRS/DSP
Jawf	1999-2012	Vital Registration	VR/SRS/DSP
Jizan	1999-2012	Vital Registration	VR/SRS/DSP
Madinah	1999-2012	Vital Registration	VR/SRS/DSP
Makkah	1999-2012	Vital Registration	VR/SRS/DSP
Najran	1999-2012	Vital Registration	VR/SRS/DSP
Northern Borders	1999-2012	Vital Registration	VR/SRS/DSP
Qassim	1999-2012	Vital Registration	VR/SRS/DSP
Riyadh	2010-2012	Vital Registration	VR/SRS/DSP
Tabuk	1999-2012	Vital Registration	VR/SRS/DSP
Sudan	2010	Multiple Indicator Cluster Survey (MICS)	CBH
Sudan	2014	Multiple Indicator Cluster Survey (MICS)	CBH
Syria	2000-2010	Vital Registration	VR/SRS/DSP
Tunisia	1988	Demographic and Health Survey (DHS)	CBH
Turkey	1993	Demographic and Health Survey (DHS)	CBH
Turkey	1998	Demographic and Health Survey (DHS)	CBH
Turkey	2003	Demographic and Health Survey (DHS)	CBH
Turkey	2008	Demographic and Health Survey (DHS)	CBH
Turkey	2013	Demographic and Health Survey (DHS)	CBH
United Arab Emirates	2004-2007	Vital Registration	VR/SRS/DSP
Yemen	1991	Demographic and Health Survey (DHS)	CBH
Yemen	1997	Demographic and Health Survey (DHS)	CBH
Yemen	2013	Demographic and Health Survey (DHS)	CBH
Yemen	2003	Pan Arab Project for Family Health (PAPFAM)	CBH
Bangladesh	1993	Demographic and Health Survey (DHS)	CBH
Bangladesh	1996	Demographic and Health Survey (DHS)	CBH
Bangladesh	1999	Demographic and Health Survey (DHS)	CBH
Bangladesh	2004	Demographic and Health Survey (DHS)	CBH
Bangladesh	2007	Demographic and Health Survey (DHS)	CBH
Bangladesh	2011	Demographic and Health Survey (DHS)	CBH
Bangladesh	2014	Demographic and Health Survey (DHS)	CBH
Bhutan	2005	Census	SBH
Bhutan	1994	Health Survey	CBH
Bhutan	2010	Multiple Indicator Cluster Survey (MICS)	SBH
India	1992	Demographic and Health Survey (DHS)	CBH
India	1998	Demographic and Health Survey (DHS)	CBH
India	2005	Demographic and Health Survey (DHS)	CBH
India	2015	Demographic and Health Survey (DHS)	CBH
Andhra Pradesh	2015	Demographic and Health Survey (DHS)	CBH
Andhra Pradesh	2005	Human Development Survey	CBH
Andhra Pradesh	2005	Human Development Survey	SBH
Andhra Pradesh	2003	India District Level Household Survey (DLHS)	SBH
Andhra Pradesh	2008	India District Level Household Survey (DLHS)	SBH
Andhra Pradesh, Rural	1992	Demographic and Health Survey (DHS)	SBH
Andhra Pradesh, Rural	1999	Demographic and Health Survey (DHS)	SBH
Andhra Pradesh, Rural	2006	Demographic and Health Survey (DHS)	SBH
Andhra Pradesh, Rural	2015	Demographic and Health Survey (DHS)	CBH
Andhra Pradesh, Urban	1992	Demographic and Health Survey (DHS)	SBH
Andhra Pradesh, Urban	1999	Demographic and Health Survey (DHS)	SBH

Appendix Table 10. Under-5 mortality reference sources by source date and location, 1950-2016			
**Location**	**Source**	**Source years**	**Method**
Andhra Pradesh, Urban	2006	Demographic and Health Survey (DHS)	SBH
Andhra Pradesh, Urban	2015	Demographic and Health Survey (DHS)	CBH
Arunachal Pradesh	1992	Demographic and Health Survey (DHS)	SBH
Arunachal Pradesh	1999	Demographic and Health Survey (DHS)	SBH
Arunachal Pradesh	2006	Demographic and Health Survey (DHS)	SBH
Arunachal Pradesh	2015	Demographic and Health Survey (DHS)	CBH
Arunachal Pradesh, Rural	1992	Demographic and Health Survey (DHS)	SBH
Arunachal Pradesh, Rural	1993	Demographic and Health Survey (DHS)	SBH
Arunachal Pradesh, Rural	1999	Demographic and Health Survey (DHS)	SBH
Arunachal Pradesh, Rural	2006	Demographic and Health Survey (DHS)	SBH
Arunachal Pradesh, Rural	2015	Demographic and Health Survey (DHS)	CBH
Arunachal Pradesh, Urban	1992	Demographic and Health Survey (DHS)	SBH
Arunachal Pradesh, Urban	1993	Demographic and Health Survey (DHS)	SBH
Arunachal Pradesh, Urban	1999	Demographic and Health Survey (DHS)	SBH
Arunachal Pradesh, Urban	2006	Demographic and Health Survey (DHS)	SBH
Arunachal Pradesh, Urban	2015	Demographic and Health Survey (DHS)	CBH
Assam	1992	Demographic and Health Survey (DHS)	SBH
Assam	1999	Demographic and Health Survey (DHS)	SBH
Assam	2006	Demographic and Health Survey (DHS)	SBH
Assam	2015	Demographic and Health Survey (DHS)	CBH
Assam, Rural	1992	Demographic and Health Survey (DHS)	SBH
Assam, Rural	1993	Demographic and Health Survey (DHS)	SBH
Assam, Rural	1999	Demographic and Health Survey (DHS)	SBH
Assam, Rural	2006	Demographic and Health Survey (DHS)	SBH
Assam, Rural	2015	Demographic and Health Survey (DHS)	CBH
Assam, Urban	1992	Demographic and Health Survey (DHS)	SBH
Assam, Urban	1999	Demographic and Health Survey (DHS)	SBH
Assam, Urban	2006	Demographic and Health Survey (DHS)	SBH
Assam, Urban	2015	Demographic and Health Survey (DHS)	CBH
Bihar	1992	Demographic and Health Survey (DHS)	SBH
Bihar	1999	Demographic and Health Survey (DHS)	SBH
Bihar	2006	Demographic and Health Survey (DHS)	SBH
Bihar	2015	Demographic and Health Survey (DHS)	CBH
Bihar, Rural	1992	Demographic and Health Survey (DHS)	SBH
Bihar, Rural	1993	Demographic and Health Survey (DHS)	SBH
Bihar, Rural	1999	Demographic and Health Survey (DHS)	SBH
Bihar, Rural	2006	Demographic and Health Survey (DHS)	SBH
Bihar, Rural	2015	Demographic and Health Survey (DHS)	CBH
Bihar, Urban	1992	Demographic and Health Survey (DHS)	SBH
Bihar, Urban	1993	Demographic and Health Survey (DHS)	SBH
Bihar, Urban	1999	Demographic and Health Survey (DHS)	SBH
Bihar, Urban	2006	Demographic and Health Survey (DHS)	SBH
Bihar, Urban	2015	Demographic and Health Survey (DHS)	CBH
Bihar, Urban	2004-2015	Health Survey	VR/SRS/DSP
Chhattisgarh	2006	Demographic and Health Survey (DHS)	SBH
Chhattisgarh	2015	Demographic and Health Survey (DHS)	CBH
Chhattisgarh, Rural	2006	Demographic and Health Survey (DHS)	SBH
Chhattisgarh, Rural	2015	Demographic and Health Survey (DHS)	CBH
Chhattisgarh, Urban	2006	Demographic and Health Survey (DHS)	SBH
Chhattisgarh, Urban	2015	Demographic and Health Survey (DHS)	CBH
Delhi	1992	Demographic and Health Survey (DHS)	SBH
Delhi	1999	Demographic and Health Survey (DHS)	SBH
Delhi	2006	Demographic and Health Survey (DHS)	SBH
Delhi	2015	Demographic and Health Survey (DHS)	CBH
Delhi, Rural	1992	Demographic and Health Survey (DHS)	SBH
Delhi, Rural	1993	Demographic and Health Survey (DHS)	SBH
Delhi, Rural	1999	Demographic and Health Survey (DHS)	SBH
Delhi, Rural	2006	Demographic and Health Survey (DHS)	SBH
Delhi, Urban	1992	Demographic and Health Survey (DHS)	SBH
Delhi, Urban	1993	Demographic and Health Survey (DHS)	SBH
Delhi, Urban	1999	Demographic and Health Survey (DHS)	SBH
Delhi, Urban	2006	Demographic and Health Survey (DHS)	SBH
Delhi, Urban	2015	Demographic and Health Survey (DHS)	CBH
Goa	1992	Demographic and Health Survey (DHS)	SBH
Goa	1999	Demographic and Health Survey (DHS)	SBH
Goa	2006	Demographic and Health Survey (DHS)	SBH
Goa	2015	Demographic and Health Survey (DHS)	CBH

Appendix Table 10. Under-5 mortality reference sources by source date and location, 1950-2016			
**Location**	**Source**	**Source years**	**Method**
Goa, Rural	1992	Demographic and Health Survey (DHS)	SBH
Goa, Rural	1999	Demographic and Health Survey (DHS)	SBH
Goa, Rural	2006	Demographic and Health Survey (DHS)	SBH
Goa, Urban	1992	Demographic and Health Survey (DHS)	SBH
Goa, Urban	1993	Demographic and Health Survey (DHS)	SBH
Goa, Urban	1999	Demographic and Health Survey (DHS)	SBH
Goa, Urban	2006	Demographic and Health Survey (DHS)	SBH
Gujarat	1992	Demographic and Health Survey (DHS)	SBH
Gujarat	1999	Demographic and Health Survey (DHS)	SBH
Gujarat	2006	Demographic and Health Survey (DHS)	SBH
Gujarat	2015	Demographic and Health Survey (DHS)	CBH
Gujarat, Rural	1992	Demographic and Health Survey (DHS)	SBH
Gujarat, Rural	1993	Demographic and Health Survey (DHS)	SBH
Gujarat, Rural	1999	Demographic and Health Survey (DHS)	SBH
Gujarat, Rural	2006	Demographic and Health Survey (DHS)	SBH
Gujarat, Rural	2015	Demographic and Health Survey (DHS)	CBH
Gujarat, Urban	1992	Demographic and Health Survey (DHS)	SBH
Gujarat, Urban	1993	Demographic and Health Survey (DHS)	SBH
Gujarat, Urban	1999	Demographic and Health Survey (DHS)	SBH
Gujarat, Urban	2006	Demographic and Health Survey (DHS)	SBH
Gujarat, Urban	2015	Demographic and Health Survey (DHS)	CBH
Haryana	1992	Demographic and Health Survey (DHS)	SBH
Haryana	1999	Demographic and Health Survey (DHS)	SBH
Haryana	2006	Demographic and Health Survey (DHS)	SBH
Haryana	2015	Demographic and Health Survey (DHS)	CBH
Haryana, Rural	1992	Demographic and Health Survey (DHS)	SBH
Haryana, Rural	1993	Demographic and Health Survey (DHS)	SBH
Haryana, Rural	1999	Demographic and Health Survey (DHS)	SBH
Haryana, Rural	2006	Demographic and Health Survey (DHS)	SBH
Haryana, Rural	2015	Demographic and Health Survey (DHS)	CBH
Haryana, Urban	1992	Demographic and Health Survey (DHS)	SBH
Haryana, Urban	1993	Demographic and Health Survey (DHS)	SBH
Haryana, Urban	1999	Demographic and Health Survey (DHS)	SBH
Haryana, Urban	2006	Demographic and Health Survey (DHS)	SBH
Haryana, Urban	2015	Demographic and Health Survey (DHS)	CBH
Himachal Pradesh	1992	Demographic and Health Survey (DHS)	SBH
Himachal Pradesh	1999	Demographic and Health Survey (DHS)	SBH
Himachal Pradesh	2006	Demographic and Health Survey (DHS)	SBH
Himachal Pradesh	2015	Demographic and Health Survey (DHS)	CBH
Himachal Pradesh, Rural	1992	Demographic and Health Survey (DHS)	SBH
Himachal Pradesh, Rural	1999	Demographic and Health Survey (DHS)	SBH
Himachal Pradesh, Rural	2006	Demographic and Health Survey (DHS)	SBH
Himachal Pradesh, Rural	2015	Demographic and Health Survey (DHS)	CBH
Himachal Pradesh, Urban	1992	Demographic and Health Survey (DHS)	SBH
Himachal Pradesh, Urban	1999	Demographic and Health Survey (DHS)	SBH
Himachal Pradesh, Urban	2006	Demographic and Health Survey (DHS)	SBH
Jammu and Kashmir	1992	Demographic and Health Survey (DHS)	SBH
Jammu and Kashmir	1999	Demographic and Health Survey (DHS)	SBH
Jammu and Kashmir	2006	Demographic and Health Survey (DHS)	SBH
Jammu and Kashmir	2015	Demographic and Health Survey (DHS)	CBH
Jammu and Kashmir, Rural	1992	Demographic and Health Survey (DHS)	SBH
Jammu and Kashmir, Rural	1993	Demographic and Health Survey (DHS)	SBH
Jammu and Kashmir, Rural	1999	Demographic and Health Survey (DHS)	SBH
Jammu and Kashmir, Rural	2006	Demographic and Health Survey (DHS)	SBH
Jammu and Kashmir, Rural	2015	Demographic and Health Survey (DHS)	CBH
Jammu and Kashmir, Urban	1992	Demographic and Health Survey (DHS)	SBH
Jammu and Kashmir, Urban	1993	Demographic and Health Survey (DHS)	SBH
Jammu and Kashmir, Urban	1999	Demographic and Health Survey (DHS)	SBH
Jammu and Kashmir, Urban	2006	Demographic and Health Survey (DHS)	SBH
Jammu and Kashmir, Urban	2015	Demographic and Health Survey (DHS)	CBH
Jharkhand	2006	Demographic and Health Survey (DHS)	SBH
Jharkhand	2015	Demographic and Health Survey (DHS)	CBH
Jharkhand, Rural	2006	Demographic and Health Survey (DHS)	SBH
Jharkhand, Rural	2015	Demographic and Health Survey (DHS)	CBH
Jharkhand, Urban	2006	Demographic and Health Survey (DHS)	SBH
Jharkhand, Urban	2015	Demographic and Health Survey (DHS)	CBH
Karnataka	1992	Demographic and Health Survey (DHS)	SBH

Appendix Table 10. Under-5 mortality reference sources by source date and location, 1950-2016			
Location	Source	Source years	Method
Karnataka	1999	Demographic and Health Survey (DHS)	SBH
Karnataka	2006	Demographic and Health Survey (DHS)	SBH
Karnataka	2015	Demographic and Health Survey (DHS)	CBH
Karnataka, Rural	1992	Demographic and Health Survey (DHS)	SBH
Karnataka, Rural	1999	Demographic and Health Survey (DHS)	SBH
Karnataka, Rural	2006	Demographic and Health Survey (DHS)	SBH
Karnataka, Rural	2015	Demographic and Health Survey (DHS)	CBH
Karnataka, Urban	1992	Demographic and Health Survey (DHS)	SBH
Karnataka, Urban	1993	Demographic and Health Survey (DHS)	SBH
Karnataka, Urban	1999	Demographic and Health Survey (DHS)	SBH
Karnataka, Urban	2006	Demographic and Health Survey (DHS)	SBH
Karnataka, Urban	2015	Demographic and Health Survey (DHS)	CBH
Kerala	1992	Demographic and Health Survey (DHS)	SBH
Kerala	1999	Demographic and Health Survey (DHS)	SBH
Kerala	2006	Demographic and Health Survey (DHS)	SBH
Kerala	2015	Demographic and Health Survey (DHS)	CBH
Kerala, Rural	1992	Demographic and Health Survey (DHS)	SBH
Kerala, Rural	1999	Demographic and Health Survey (DHS)	SBH
Kerala, Rural	2006	Demographic and Health Survey (DHS)	SBH
Kerala, Rural	2015	Demographic and Health Survey (DHS)	CBH
Kerala, Urban	1992	Demographic and Health Survey (DHS)	SBH
Kerala, Urban	1999	Demographic and Health Survey (DHS)	SBH
Kerala, Urban	2006	Demographic and Health Survey (DHS)	SBH
Kerala, Urban	2015	Demographic and Health Survey (DHS)	CBH
Madhya Pradesh	1992	Demographic and Health Survey (DHS)	SBH
Madhya Pradesh	1999	Demographic and Health Survey (DHS)	SBH
Madhya Pradesh	2006	Demographic and Health Survey (DHS)	SBH
Madhya Pradesh	2015	Demographic and Health Survey (DHS)	CBH
Madhya Pradesh, Rural	1992	Demographic and Health Survey (DHS)	SBH
Madhya Pradesh, Rural	1999	Demographic and Health Survey (DHS)	SBH
Madhya Pradesh, Rural	2006	Demographic and Health Survey (DHS)	SBH
Madhya Pradesh, Rural	2015	Demographic and Health Survey (DHS)	CBH
Madhya Pradesh, Urban	1992	Demographic and Health Survey (DHS)	SBH
Madhya Pradesh, Urban	1999	Demographic and Health Survey (DHS)	SBH
Madhya Pradesh, Urban	2006	Demographic and Health Survey (DHS)	SBH
Madhya Pradesh, Urban	2015	Demographic and Health Survey (DHS)	CBH
Maharashtra	1992	Demographic and Health Survey (DHS)	SBH
Maharashtra	1999	Demographic and Health Survey (DHS)	SBH
Maharashtra	2006	Demographic and Health Survey (DHS)	SBH
Maharashtra	2015	Demographic and Health Survey (DHS)	CBH
Maharashtra, Rural	1992	Demographic and Health Survey (DHS)	SBH
Maharashtra, Rural	1999	Demographic and Health Survey (DHS)	SBH
Maharashtra, Rural	2006	Demographic and Health Survey (DHS)	SBH
Maharashtra, Rural	2015	Demographic and Health Survey (DHS)	CBH
Maharashtra, Urban	1992	Demographic and Health Survey (DHS)	SBH
Maharashtra, Urban	1993	Demographic and Health Survey (DHS)	SBH
Maharashtra, Urban	1999	Demographic and Health Survey (DHS)	SBH
Maharashtra, Urban	2006	Demographic and Health Survey (DHS)	SBH
Maharashtra, Urban	2015	Demographic and Health Survey (DHS)	CBH
Manipur	1992	Demographic and Health Survey (DHS)	SBH
Manipur	1999	Demographic and Health Survey (DHS)	SBH
Manipur	2006	Demographic and Health Survey (DHS)	SBH
Manipur	2015	Demographic and Health Survey (DHS)	CBH
Manipur, Rural	1992	Demographic and Health Survey (DHS)	SBH
Manipur, Rural	1993	Demographic and Health Survey (DHS)	SBH
Manipur, Rural	1999	Demographic and Health Survey (DHS)	SBH
Manipur, Rural	2006	Demographic and Health Survey (DHS)	SBH
Manipur, Rural	2015	Demographic and Health Survey (DHS)	CBH
Manipur, Urban	1992	Demographic and Health Survey (DHS)	SBH
Manipur, Urban	1993	Demographic and Health Survey (DHS)	SBH
Manipur, Urban	1999	Demographic and Health Survey (DHS)	SBH
Manipur, Urban	2006	Demographic and Health Survey (DHS)	SBH
Manipur, Urban	2015	Demographic and Health Survey (DHS)	CBH
Meghalaya	1992	Demographic and Health Survey (DHS)	SBH
Meghalaya	1999	Demographic and Health Survey (DHS)	SBH
Meghalaya	2006	Demographic and Health Survey (DHS)	SBH
Meghalaya	2015	Demographic and Health Survey (DHS)	CBH

Appendix Table 10. Under-5 mortality reference sources by source date and location, 1950-2016			
**Location**	**Source**	**Source years**	**Method**
Meghalaya, Rural	1992	Demographic and Health Survey (DHS)	SBH
Meghalaya, Rural	1993	Demographic and Health Survey (DHS)	SBH
Meghalaya, Rural	1999	Demographic and Health Survey (DHS)	SBH
Meghalaya, Rural	2006	Demographic and Health Survey (DHS)	SBH
Meghalaya, Rural	2015	Demographic and Health Survey (DHS)	CBH
Meghalaya, Urban	1992	Demographic and Health Survey (DHS)	SBH
Meghalaya, Urban	1993	Demographic and Health Survey (DHS)	SBH
Meghalaya, Urban	1999	Demographic and Health Survey (DHS)	SBH
Meghalaya, Urban	2006	Demographic and Health Survey (DHS)	SBH
Meghalaya, Urban	2015	Demographic and Health Survey (DHS)	CBH
Mizoram	1992	Demographic and Health Survey (DHS)	SBH
Mizoram	1999	Demographic and Health Survey (DHS)	SBH
Mizoram	2006	Demographic and Health Survey (DHS)	SBH
Mizoram	2015	Demographic and Health Survey (DHS)	CBH
Mizoram, Rural	1992	Demographic and Health Survey (DHS)	SBH
Mizoram, Rural	1993	Demographic and Health Survey (DHS)	SBH
Mizoram, Rural	1999	Demographic and Health Survey (DHS)	SBH
Mizoram, Rural	2006	Demographic and Health Survey (DHS)	SBH
Mizoram, Rural	2015	Demographic and Health Survey (DHS)	CBH
Mizoram, Urban	1992	Demographic and Health Survey (DHS)	SBH
Mizoram, Urban	1993	Demographic and Health Survey (DHS)	SBH
Mizoram, Urban	1999	Demographic and Health Survey (DHS)	SBH
Mizoram, Urban	2006	Demographic and Health Survey (DHS)	SBH
Mizoram, Urban	2015	Demographic and Health Survey (DHS)	CBH
Nagaland	1999	Demographic and Health Survey (DHS)	SBH
Nagaland	2006	Demographic and Health Survey (DHS)	SBH
Nagaland	2015	Demographic and Health Survey (DHS)	CBH
Nagaland, Rural	1999	Demographic and Health Survey (DHS)	SBH
Nagaland, Rural	2006	Demographic and Health Survey (DHS)	SBH
Nagaland, Rural	2015	Demographic and Health Survey (DHS)	CBH
Nagaland, Urban	1992	Demographic and Health Survey (DHS)	SBH
Nagaland, Urban	1993	Demographic and Health Survey (DHS)	SBH
Nagaland, Urban	1999	Demographic and Health Survey (DHS)	SBH
Nagaland, Urban	2006	Demographic and Health Survey (DHS)	SBH
Nagaland, Urban	2015	Demographic and Health Survey (DHS)	CBH
Odisha	1992	Demographic and Health Survey (DHS)	SBH
Odisha	1999	Demographic and Health Survey (DHS)	SBH
Odisha	2006	Demographic and Health Survey (DHS)	SBH
Odisha	2015	Demographic and Health Survey (DHS)	CBH
Odisha, Rural	1992	Demographic and Health Survey (DHS)	SBH
Odisha, Rural	1993	Demographic and Health Survey (DHS)	SBH
Odisha, Rural	1999	Demographic and Health Survey (DHS)	SBH
Odisha, Rural	2006	Demographic and Health Survey (DHS)	SBH
Odisha, Rural	2015	Demographic and Health Survey (DHS)	CBH
Odisha, Urban	1992	Demographic and Health Survey (DHS)	SBH
Odisha, Urban	1993	Demographic and Health Survey (DHS)	SBH
Odisha, Urban	1999	Demographic and Health Survey (DHS)	SBH
Odisha, Urban	2006	Demographic and Health Survey (DHS)	SBH
Odisha, Urban	2015	Demographic and Health Survey (DHS)	CBH
Punjab	1992	Demographic and Health Survey (DHS)	SBH
Punjab	1999	Demographic and Health Survey (DHS)	SBH
Punjab	2006	Demographic and Health Survey (DHS)	SBH
Punjab	2015	Demographic and Health Survey (DHS)	CBH
Punjab, Rural	1992	Demographic and Health Survey (DHS)	SBH
Punjab, Rural	1993	Demographic and Health Survey (DHS)	SBH
Punjab, Rural	1999	Demographic and Health Survey (DHS)	SBH
Punjab, Rural	2006	Demographic and Health Survey (DHS)	SBH
Punjab, Rural	2015	Demographic and Health Survey (DHS)	CBH
Punjab, Urban	1992	Demographic and Health Survey (DHS)	SBH
Punjab, Urban	1993	Demographic and Health Survey (DHS)	SBH
Punjab, Urban	1999	Demographic and Health Survey (DHS)	SBH
Punjab, Urban	2006	Demographic and Health Survey (DHS)	SBH
Punjab, Urban	2015	Demographic and Health Survey (DHS)	CBH
Rajasthan	1999	Demographic and Health Survey (DHS)	SBH
Rajasthan	2006	Demographic and Health Survey (DHS)	SBH
Rajasthan	2015	Demographic and Health Survey (DHS)	CBH
Rajasthan, Rural	1992	Demographic and Health Survey (DHS)	SBH

Appendix Table 10. Under-5 mortality reference sources by source date and location, 1950-2016			
**Location**	**Source**	**Source years**	**Method**
Rajasthan, Rural	1993	Demographic and Health Survey (DHS)	SBH
Rajasthan, Rural	1999	Demographic and Health Survey (DHS)	SBH
Rajasthan, Rural	2006	Demographic and Health Survey (DHS)	SBH
Rajasthan, Rural	2015	Demographic and Health Survey (DHS)	CBH
Rajasthan, Urban	1992	Demographic and Health Survey (DHS)	SBH
Rajasthan, Urban	1993	Demographic and Health Survey (DHS)	SBH
Rajasthan, Urban	1999	Demographic and Health Survey (DHS)	SBH
Rajasthan, Urban	2006	Demographic and Health Survey (DHS)	SBH
Rajasthan, Urban	2015	Demographic and Health Survey (DHS)	CBH
Sikkim	1999	Demographic and Health Survey (DHS)	SBH
Sikkim	2006	Demographic and Health Survey (DHS)	SBH
Sikkim	2015	Demographic and Health Survey (DHS)	CBH
Sikkim, Rural	1999	Demographic and Health Survey (DHS)	SBH
Sikkim, Rural	2006	Demographic and Health Survey (DHS)	SBH
Sikkim, Rural	2015	Demographic and Health Survey (DHS)	CBH
Sikkim, Urban	1999	Demographic and Health Survey (DHS)	SBH
Sikkim, Urban	2006	Demographic and Health Survey (DHS)	SBH
Sikkim, Urban	2015	Demographic and Health Survey (DHS)	CBH
Tamil Nadu	1992	Demographic and Health Survey (DHS)	SBH
Tamil Nadu	1999	Demographic and Health Survey (DHS)	SBH
Tamil Nadu	2006	Demographic and Health Survey (DHS)	SBH
Tamil Nadu	2015	Demographic and Health Survey (DHS)	CBH
Tamil Nadu, Rural	1992	Demographic and Health Survey (DHS)	SBH
Tamil Nadu, Rural	1999	Demographic and Health Survey (DHS)	SBH
Tamil Nadu, Rural	2006	Demographic and Health Survey (DHS)	SBH
Tamil Nadu, Rural	2015	Demographic and Health Survey (DHS)	CBH
Tamil Nadu, Urban	1992	Demographic and Health Survey (DHS)	SBH
Tamil Nadu, Urban	1999	Demographic and Health Survey (DHS)	SBH
Tamil Nadu, Urban	2006	Demographic and Health Survey (DHS)	SBH
Tamil Nadu, Urban	2015	Demographic and Health Survey (DHS)	CBH
Telangana	2015	Demographic and Health Survey (DHS)	CBH
Telangana	2005	Human Development Survey	CBH
Telangana	2005	Human Development Survey	SBH
Telangana	2003	India District Level Household Survey (DLHS)	SBH
Telangana	2008	India District Level Household Survey (DLHS)	SBH
Telangana, Rural	2015	Demographic and Health Survey (DHS)	CBH
Telangana, Rural	2003	India District Level Household Survey (DLHS)	SBH
Telangana, Rural	2008	India District Level Household Survey (DLHS)	SBH
Telangana, Urban	2015	Demographic and Health Survey (DHS)	CBH
Telangana, Urban	2003	India District Level Household Survey (DLHS)	SBH
Telangana, Urban	2008	India District Level Household Survey (DLHS)	SBH
Tripura	1992	Demographic and Health Survey (DHS)	SBH
Tripura	1999	Demographic and Health Survey (DHS)	SBH
Tripura	2006	Demographic and Health Survey (DHS)	SBH
Tripura	2015	Demographic and Health Survey (DHS)	CBH
Tripura, Rural	1992	Demographic and Health Survey (DHS)	SBH
Tripura, Rural	1993	Demographic and Health Survey (DHS)	SBH
Tripura, Rural	1999	Demographic and Health Survey (DHS)	SBH
Tripura, Rural	2006	Demographic and Health Survey (DHS)	SBH
Tripura, Rural	2015	Demographic and Health Survey (DHS)	CBH
Tripura, Urban	1992	Demographic and Health Survey (DHS)	SBH
Tripura, Urban	1993	Demographic and Health Survey (DHS)	SBH
Tripura, Urban	1999	Demographic and Health Survey (DHS)	SBH
Tripura, Urban	2006	Demographic and Health Survey (DHS)	SBH
Tripura, Urban	2015	Demographic and Health Survey (DHS)	CBH
Uttar Pradesh	1992	Demographic and Health Survey (DHS)	SBH
Uttar Pradesh	1999	Demographic and Health Survey (DHS)	SBH
Uttar Pradesh	2006	Demographic and Health Survey (DHS)	SBH
Uttar Pradesh	2015	Demographic and Health Survey (DHS)	CBH
Uttar Pradesh, Rural	1992	Demographic and Health Survey (DHS)	SBH
Uttar Pradesh, Rural	1999	Demographic and Health Survey (DHS)	SBH
Uttar Pradesh, Rural	2006	Demographic and Health Survey (DHS)	SBH
Uttar Pradesh, Rural	2015	Demographic and Health Survey (DHS)	CBH
Uttar Pradesh, Urban	1992	Demographic and Health Survey (DHS)	SBH
Uttar Pradesh, Urban	1999	Demographic and Health Survey (DHS)	SBH
Uttar Pradesh, Urban	2006	Demographic and Health Survey (DHS)	SBH
Uttar Pradesh, Urban	2015	Demographic and Health Survey (DHS)	CBH

Appendix Table 10. Under-5 mortality reference sources by source date and location, 1950-2016			
**Location**	**Source**	**Source years**	**Method**
Uttarakhand	2006	Demographic and Health Survey (DHS)	SBH
Uttarakhand	2015	Demographic and Health Survey (DHS)	CBH
Uttarakhand, Rural	2006	Demographic and Health Survey (DHS)	SBH
Uttarakhand, Rural	2015	Demographic and Health Survey (DHS)	CBH
Uttarakhand, Urban	2006	Demographic and Health Survey (DHS)	SBH
Uttarakhand, Urban	2015	Demographic and Health Survey (DHS)	CBH
West Bengal	1992	Demographic and Health Survey (DHS)	SBH
West Bengal	1999	Demographic and Health Survey (DHS)	SBH
West Bengal	2006	Demographic and Health Survey (DHS)	SBH
West Bengal	2015	Demographic and Health Survey (DHS)	CBH
West Bengal, Rural	1992	Demographic and Health Survey (DHS)	SBH
West Bengal, Rural	1999	Demographic and Health Survey (DHS)	SBH
West Bengal, Rural	2006	Demographic and Health Survey (DHS)	SBH
West Bengal, Rural	2015	Demographic and Health Survey (DHS)	CBH
West Bengal, Urban	1992	Demographic and Health Survey (DHS)	SBH
West Bengal, Urban	1999	Demographic and Health Survey (DHS)	SBH
West Bengal, Urban	2006	Demographic and Health Survey (DHS)	SBH
West Bengal, Urban	2015	Demographic and Health Survey (DHS)	CBH
The Six Minor Territories	2001	Census	SBH
The Six Minor Territories	2005	Human Development Survey	CBH
The Six Minor Territories	2005	Human Development Survey	SBH
The Six Minor Territories	2008	India District Level Household Survey (DLHS)	SBH
The Six Minor Territories	2013	India District Level Household Survey (DLHS)	SBH
The Six Minor Territories, Rural	2001	Census	SBH
The Six Minor Territories, Rural	2008	India District Level Household Survey (DLHS)	SBH
The Six Minor Territories, Rural	2013	India District Level Household Survey (DLHS)	SBH
The Six Minor Territories, Urban	2001	Census	SBH
The Six Minor Territories, Urban	2005	Human Development Survey	CBH
The Six Minor Territories, Urban	2008	India District Level Household Survey (DLHS)	SBH
The Six Minor Territories, Urban	2013	India District Level Household Survey (DLHS)	SBH
Nepal	1996	Demographic and Health Survey (DHS)	CBH
Nepal	2001	Demographic and Health Survey (DHS)	CBH
Nepal	2006	Demographic and Health Survey (DHS)	CBH
Nepal	2011	Demographic and Health Survey (DHS)	CBH
Pakistan	1990	Demographic and Health Survey (DHS)	CBH
Pakistan	2006	Demographic and Health Survey (DHS)	CBH
Pakistan	2012	Demographic and Health Survey (DHS)	CBH
Angola	2016	Demographic and Health Survey (DHS)	CBH
Central African Republic	1994	Demographic and Health Survey (DHS)	CBH
Central African Republic	1994	Demographic and Health Survey (DHS)	SBH
Central African Republic	2000	Multiple Indicator Cluster Survey (MICS)	SBH
Central African Republic	2006	Multiple Indicator Cluster Survey (MICS)	SBH
Central African Republic	2010	Multiple Indicator Cluster Survey (MICS)	SBH
Congo	2005	Demographic and Health Survey (DHS)	CBH
Congo	2011	Demographic and Health Survey (DHS)	CBH
Democratic Republic of the Congo	2007	Demographic and Health Survey (DHS)	CBH
Democratic Republic of the Congo	2013	Demographic and Health Survey (DHS)	CBH
Equatorial Guinea	2011	Demographic and Health Survey (DHS)	CBH
Equatorial Guinea	2000	Multiple Indicator Cluster Survey (MICS)	SBH
Gabon	2000	Demographic and Health Survey (DHS)	CBH
Gabon	2012	Demographic and Health Survey (DHS)	CBH
Burundi	1987	Demographic and Health Survey (DHS)	CBH
Burundi	2010	Demographic and Health Survey (DHS)	CBH
Comoros	1996	Demographic and Health Survey (DHS)	CBH
Djibouti	1991	Demographic Survey	SBH
Djibouti	2006	Multiple Indicator Cluster Survey (MICS)	SBH
Djibouti	2002	Pan Arab Project for Family Health (PAPFAM)	SBH
Djibouti	2002	Pan Arab Project for Family Health (PAPFAM)	CBH
Eritrea	1995	Demographic and Health Survey (DHS)	CBH
Eritrea	2002	Demographic and Health Survey (DHS)	CBH
Ethiopia	2000	Demographic and Health Survey (DHS)	CBH
Ethiopia	2005	Demographic and Health Survey (DHS)	CBH
Ethiopia	2010	Demographic and Health Survey (DHS)	CBH
Ethiopia	2016	Demographic and Health Survey (DHS)	CBH
Kenya	1988	Demographic and Health Survey (DHS)	CBH
Kenya	1993	Demographic and Health Survey (DHS)	CBH
Kenya	1998	Demographic and Health Survey (DHS)	CBH

Appendix Table 10. Under-5 mortality reference sources by source date and location, 1950-2016			
**Location**	**Source**	**Source years**	**Method**
Kenya	2003	Demographic and Health Survey (DHS)	CBH
Kenya	2008	Demographic and Health Survey (DHS)	CBH
Kenya	2014	Demographic and Health Survey (DHS)	CBH
Central	1989	Demographic and Health Survey (DHS)	SBH
Central	1993	Demographic and Health Survey (DHS)	SBH
Central	1998	Demographic and Health Survey (DHS)	SBH
Central	2003	Demographic and Health Survey (DHS)	SBH
Central	2009	Demographic and Health Survey (DHS)	SBH
Central	2014	Demographic and Health Survey (DHS)	SBH
Kiambu	1988	Demographic and Health Survey (DHS)	CBH
Kiambu	1993	Demographic and Health Survey (DHS)	CBH
Kiambu	1998	Demographic and Health Survey (DHS)	CBH
Kiambu	2003	Demographic and Health Survey (DHS)	CBH
Kiambu	2014	Demographic and Health Survey (DHS)	CBH
Kirinyaga	1988	Demographic and Health Survey (DHS)	CBH
Kirinyaga	1998	Demographic and Health Survey (DHS)	CBH
Kirinyaga	2003	Demographic and Health Survey (DHS)	CBH
Kirinyaga	2008	Demographic and Health Survey (DHS)	CBH
Kirinyaga	2014	Demographic and Health Survey (DHS)	CBH
Murang'a	1988	Demographic and Health Survey (DHS)	CBH
Murang'a	1993	Demographic and Health Survey (DHS)	CBH
Murang'a	1998	Demographic and Health Survey (DHS)	CBH
Murang'a	2003	Demographic and Health Survey (DHS)	CBH
Murang'a	2014	Demographic and Health Survey (DHS)	CBH
Nyandarua	1988	Demographic and Health Survey (DHS)	CBH
Nyandarua	1993	Demographic and Health Survey (DHS)	CBH
Nyandarua	1998	Demographic and Health Survey (DHS)	CBH
Nyandarua	2003	Demographic and Health Survey (DHS)	CBH
Nyandarua	2008	Demographic and Health Survey (DHS)	CBH
Nyandarua	2014	Demographic and Health Survey (DHS)	CBH
Nyeri	1988	Demographic and Health Survey (DHS)	CBH
Nyeri	1993	Demographic and Health Survey (DHS)	CBH
Nyeri	1998	Demographic and Health Survey (DHS)	CBH
Nyeri	2003	Demographic and Health Survey (DHS)	CBH
Nyeri	2008	Demographic and Health Survey (DHS)	CBH
Nyeri	2014	Demographic and Health Survey (DHS)	CBH
Coast	1989	Demographic and Health Survey (DHS)	SBH
Coast	1993	Demographic and Health Survey (DHS)	SBH
Coast	1998	Demographic and Health Survey (DHS)	SBH
Coast	2003	Demographic and Health Survey (DHS)	SBH
Coast	2009	Demographic and Health Survey (DHS)	SBH
Coast	2014	Demographic and Health Survey (DHS)	SBH
Kilifi	1988	Demographic and Health Survey (DHS)	CBH
Kilifi	1993	Demographic and Health Survey (DHS)	CBH
Kilifi	1998	Demographic and Health Survey (DHS)	CBH
Kilifi	2003	Demographic and Health Survey (DHS)	CBH
Kilifi	2008	Demographic and Health Survey (DHS)	CBH
Kilifi	2014	Demographic and Health Survey (DHS)	CBH
Kwale	1988	Demographic and Health Survey (DHS)	CBH
Kwale	1993	Demographic and Health Survey (DHS)	CBH
Kwale	1998	Demographic and Health Survey (DHS)	CBH
Kwale	2003	Demographic and Health Survey (DHS)	CBH
Kwale	2008	Demographic and Health Survey (DHS)	CBH
Kwale	2014	Demographic and Health Survey (DHS)	CBH
Lamu	1993	Demographic and Health Survey (DHS)	CBH
Lamu	2003	Demographic and Health Survey (DHS)	CBH
Lamu	2008	Demographic and Health Survey (DHS)	CBH
Lamu	2014	Demographic and Health Survey (DHS)	CBH
Mombasa	1988	Demographic and Health Survey (DHS)	CBH
Mombasa	1993	Demographic and Health Survey (DHS)	CBH
Mombasa	1998	Demographic and Health Survey (DHS)	CBH
Mombasa	2003	Demographic and Health Survey (DHS)	CBH
Mombasa	2008	Demographic and Health Survey (DHS)	CBH
Mombasa	2014	Demographic and Health Survey (DHS)	CBH
TaitaTaveta	1988	Demographic and Health Survey (DHS)	CBH
TaitaTaveta	1993	Demographic and Health Survey (DHS)	CBH
TaitaTaveta	2003	Demographic and Health Survey (DHS)	CBH

Appendix Table 10. Under-5 mortality reference sources by source date and location, 1950-2016			
Location	Source	Source years	Method
TaitaTaveta	2008	Demographic and Health Survey (DHS)	CBH
TaitaTaveta	2014	Demographic and Health Survey (DHS)	CBH
TanaRiver	2008	Demographic and Health Survey (DHS)	CBH
TanaRiver	2014	Demographic and Health Survey (DHS)	CBH
Eastern	1989	Demographic and Health Survey (DHS)	SBH
Eastern	1993	Demographic and Health Survey (DHS)	SBH
Eastern	1998	Demographic and Health Survey (DHS)	SBH
Eastern	2003	Demographic and Health Survey (DHS)	SBH
Eastern	2009	Demographic and Health Survey (DHS)	SBH
Eastern	2014	Demographic and Health Survey (DHS)	SBH
Embu	1988	Demographic and Health Survey (DHS)	CBH
Embu	1993	Demographic and Health Survey (DHS)	CBH
Embu	2008	Demographic and Health Survey (DHS)	CBH
Embu	2014	Demographic and Health Survey (DHS)	CBH
Isiolo	2011	Multiple Indicator Cluster Survey (MICS)	SBH
Kitui	1988	Demographic and Health Survey (DHS)	CBH
Kitui	1993	Demographic and Health Survey (DHS)	CBH
Kitui	1998	Demographic and Health Survey (DHS)	CBH
Kitui	2003	Demographic and Health Survey (DHS)	CBH
Kitui	2008	Demographic and Health Survey (DHS)	CBH
Kitui	2014	Demographic and Health Survey (DHS)	CBH
Machakos	1988	Demographic and Health Survey (DHS)	CBH
Machakos	1993	Demographic and Health Survey (DHS)	CBH
Machakos	1998	Demographic and Health Survey (DHS)	CBH
Machakos	2003	Demographic and Health Survey (DHS)	CBH
Machakos	2008	Demographic and Health Survey (DHS)	CBH
Machakos	2014	Demographic and Health Survey (DHS)	CBH
Makueni	2003	Demographic and Health Survey (DHS)	CBH
Makueni	2008	Demographic and Health Survey (DHS)	CBH
Makueni	2014	Demographic and Health Survey (DHS)	CBH
Marsabit	2003	Demographic and Health Survey (DHS)	SBH
Meru	1988	Demographic and Health Survey (DHS)	CBH
Meru	1993	Demographic and Health Survey (DHS)	CBH
Meru	1998	Demographic and Health Survey (DHS)	CBH
Meru	2003	Demographic and Health Survey (DHS)	CBH
Meru	2008	Demographic and Health Survey (DHS)	CBH
Meru	2014	Demographic and Health Survey (DHS)	CBH
TharakaNithi	2008	Demographic and Health Survey (DHS)	CBH
TharakaNithi	2014	Demographic and Health Survey (DHS)	CBH
Nairobi	1989	Demographic and Health Survey (DHS)	SBH
Nairobi	1993	Demographic and Health Survey (DHS)	SBH
Nairobi	1998	Demographic and Health Survey (DHS)	SBH
Nairobi	2003	Demographic and Health Survey (DHS)	SBH
Nairobi	2009	Demographic and Health Survey (DHS)	SBH
Nairobi	1988	Demographic and Health Survey (DHS)	CBH
Nairobi	1993	Demographic and Health Survey (DHS)	CBH
Nairobi	1998	Demographic and Health Survey (DHS)	CBH
Nairobi	2003	Demographic and Health Survey (DHS)	CBH
Nairobi	2008	Demographic and Health Survey (DHS)	CBH
Nairobi	2014	Demographic and Health Survey (DHS)	CBH
North Eastern	2003	Demographic and Health Survey (DHS)	SBH
North Eastern	2009	Demographic and Health Survey (DHS)	SBH
North Eastern	2014	Demographic and Health Survey (DHS)	SBH
Garissa	2003	Demographic and Health Survey (DHS)	CBH
Garissa	2008	Demographic and Health Survey (DHS)	CBH
Garissa	2014	Demographic and Health Survey (DHS)	CBH
Mandera	2007	Multiple Indicator Cluster Survey (MICS)	SBH
Wajir	2003	Demographic and Health Survey (DHS)	SBH
Nyanza	1989	Demographic and Health Survey (DHS)	SBH
Nyanza	1993	Demographic and Health Survey (DHS)	SBH
Nyanza	1998	Demographic and Health Survey (DHS)	SBH
Nyanza	2003	Demographic and Health Survey (DHS)	SBH
Nyanza	2009	Demographic and Health Survey (DHS)	SBH
Nyanza	2014	Demographic and Health Survey (DHS)	SBH
HomaBay	2003	Demographic and Health Survey (DHS)	CBH
HomaBay	2008	Demographic and Health Survey (DHS)	CBH
HomaBay	2014	Demographic and Health Survey (DHS)	CBH

Appendix Table 10. Under-5 mortality reference sources by source date and location, 1950-2016			
Location	Source	Source years	Method
Kisii	1988	Demographic and Health Survey (DHS)	CBH
Kisii	1993	Demographic and Health Survey (DHS)	CBH
Kisii	1998	Demographic and Health Survey (DHS)	CBH
Kisii	2008	Demographic and Health Survey (DHS)	CBH
Kisii	2014	Demographic and Health Survey (DHS)	CBH
Kisumu	1988	Demographic and Health Survey (DHS)	CBH
Kisumu	1993	Demographic and Health Survey (DHS)	CBH
Kisumu	1998	Demographic and Health Survey (DHS)	CBH
Kisumu	2008	Demographic and Health Survey (DHS)	CBH
Kisumu	2014	Demographic and Health Survey (DHS)	CBH
Migori	2003	Demographic and Health Survey (DHS)	CBH
Migori	2008	Demographic and Health Survey (DHS)	CBH
Migori	2014	Demographic and Health Survey (DHS)	CBH
Nyamira	1998	Demographic and Health Survey (DHS)	CBH
Nyamira	2008	Demographic and Health Survey (DHS)	CBH
Nyamira	2014	Demographic and Health Survey (DHS)	CBH
Siaya	1988	Demographic and Health Survey (DHS)	CBH
Siaya	1993	Demographic and Health Survey (DHS)	CBH
Siaya	1998	Demographic and Health Survey (DHS)	CBH
Siaya	2003	Demographic and Health Survey (DHS)	CBH
Siaya	2008	Demographic and Health Survey (DHS)	CBH
Siaya	2014	Demographic and Health Survey (DHS)	CBH
Rift Valley	1989	Demographic and Health Survey (DHS)	SBH
Rift Valley	1993	Demographic and Health Survey (DHS)	SBH
Rift Valley	1998	Demographic and Health Survey (DHS)	SBH
Rift Valley	2003	Demographic and Health Survey (DHS)	SBH
Rift Valley	2009	Demographic and Health Survey (DHS)	SBH
Rift Valley	2014	Demographic and Health Survey (DHS)	SBH
Baringo	2009	Demographic and Health Survey (DHS)	SBH
Bomet	2008	Demographic and Health Survey (DHS)	CBH
Bomet	2014	Demographic and Health Survey (DHS)	CBH
Elgeyo-Marakwet	1993	Demographic and Health Survey (DHS)	CBH
Elgeyo-Marakwet	1998	Demographic and Health Survey (DHS)	CBH
Elgeyo-Marakwet	2003	Demographic and Health Survey (DHS)	CBH
Elgeyo-Marakwet	2008	Demographic and Health Survey (DHS)	CBH
Elgeyo-Marakwet	2014	Demographic and Health Survey (DHS)	CBH
Kajiado	1988	Demographic and Health Survey (DHS)	CBH
Kajiado	1998	Demographic and Health Survey (DHS)	CBH
Kajiado	2008	Demographic and Health Survey (DHS)	CBH
Kajiado	2014	Demographic and Health Survey (DHS)	CBH
Kericho	1988	Demographic and Health Survey (DHS)	CBH
Kericho	1993	Demographic and Health Survey (DHS)	CBH
Kericho	1998	Demographic and Health Survey (DHS)	CBH
Kericho	2008	Demographic and Health Survey (DHS)	CBH
Kericho	2014	Demographic and Health Survey (DHS)	CBH
Laikipia	1988	Demographic and Health Survey (DHS)	CBH
Laikipia	2003	Demographic and Health Survey (DHS)	CBH
Laikipia	2008	Demographic and Health Survey (DHS)	CBH
Laikipia	2014	Demographic and Health Survey (DHS)	CBH
Nakuru	1988	Demographic and Health Survey (DHS)	CBH
Nakuru	1993	Demographic and Health Survey (DHS)	CBH
Nakuru	1998	Demographic and Health Survey (DHS)	CBH
Nakuru	2003	Demographic and Health Survey (DHS)	CBH
Nakuru	2008	Demographic and Health Survey (DHS)	CBH
Nakuru	2014	Demographic and Health Survey (DHS)	CBH
Nandi	1988	Demographic and Health Survey (DHS)	CBH
Nandi	1993	Demographic and Health Survey (DHS)	CBH
Nandi	1998	Demographic and Health Survey (DHS)	CBH
Nandi	2014	Demographic and Health Survey (DHS)	CBH
Narok	1988	Demographic and Health Survey (DHS)	CBH
Narok	1993	Demographic and Health Survey (DHS)	CBH
Narok	1998	Demographic and Health Survey (DHS)	CBH
Narok	2003	Demographic and Health Survey (DHS)	CBH
Narok	2008	Demographic and Health Survey (DHS)	CBH
Narok	2014	Demographic and Health Survey (DHS)	CBH
Samburu	2009	Demographic and Health Survey (DHS)	SBH
TransNzoia	1988	Demographic and Health Survey (DHS)	CBH

Appendix Table 10. Under-5 mortality reference sources by source date and location, 1950-2016			
Location	Source	Source years	Method
TransNzoia	1993	Demographic and Health Survey (DHS)	CBH
TransNzoia	1998	Demographic and Health Survey (DHS)	CBH
TransNzoia	2003	Demographic and Health Survey (DHS)	CBH
TransNzoia	2014	Demographic and Health Survey (DHS)	CBH
Turkana	2003	Demographic and Health Survey (DHS)	CBH
Turkana	2008	Demographic and Health Survey (DHS)	CBH
Turkana	2014	Demographic and Health Survey (DHS)	CBH
UasinGishu	1988	Demographic and Health Survey (DHS)	CBH
UasinGishu	1993	Demographic and Health Survey (DHS)	CBH
UasinGishu	1998	Demographic and Health Survey (DHS)	CBH
UasinGishu	2003	Demographic and Health Survey (DHS)	CBH
UasinGishu	2014	Demographic and Health Survey (DHS)	CBH
WestPokot	2009	Demographic and Health Survey (DHS)	SBH
Western	1989	Demographic and Health Survey (DHS)	SBH
Western	1993	Demographic and Health Survey (DHS)	SBH
Western	1998	Demographic and Health Survey (DHS)	SBH
Western	2003	Demographic and Health Survey (DHS)	SBH
Western	2009	Demographic and Health Survey (DHS)	SBH
Western	2014	Demographic and Health Survey (DHS)	SBH
Bungoma	1988	Demographic and Health Survey (DHS)	CBH
Bungoma	1993	Demographic and Health Survey (DHS)	CBH
Bungoma	1998	Demographic and Health Survey (DHS)	CBH
Bungoma	2003	Demographic and Health Survey (DHS)	CBH
Bungoma	2014	Demographic and Health Survey (DHS)	CBH
Busia	1988	Demographic and Health Survey (DHS)	CBH
Busia	1993	Demographic and Health Survey (DHS)	CBH
Busia	1998	Demographic and Health Survey (DHS)	CBH
Busia	2003	Demographic and Health Survey (DHS)	CBH
Busia	2008	Demographic and Health Survey (DHS)	CBH
Busia	2014	Demographic and Health Survey (DHS)	CBH
Kakamega	1988	Demographic and Health Survey (DHS)	CBH
Kakamega	1993	Demographic and Health Survey (DHS)	CBH
Kakamega	1998	Demographic and Health Survey (DHS)	CBH
Kakamega	2003	Demographic and Health Survey (DHS)	CBH
Kakamega	2008	Demographic and Health Survey (DHS)	CBH
Kakamega	2014	Demographic and Health Survey (DHS)	CBH
Vihiga	2008	Demographic and Health Survey (DHS)	CBH
Vihiga	2014	Demographic and Health Survey (DHS)	CBH
Madagascar	1992	Demographic and Health Survey (DHS)	CBH
Madagascar	1997	Demographic and Health Survey (DHS)	CBH
Madagascar	2003	Demographic and Health Survey (DHS)	CBH
Malawi	1992	Demographic and Health Survey (DHS)	CBH
Malawi	2000	Demographic and Health Survey (DHS)	CBH
Malawi	2004	Demographic and Health Survey (DHS)	CBH
Malawi	2010	Demographic and Health Survey (DHS)	CBH
Mozambique	1997	Demographic and Health Survey (DHS)	CBH
Mozambique	2003	Demographic and Health Survey (DHS)	CBH
Mozambique	2011	Demographic and Health Survey (DHS)	CBH
Rwanda	1992	Demographic and Health Survey (DHS)	CBH
Rwanda	2000	Demographic and Health Survey (DHS)	CBH
Rwanda	2005	Demographic and Health Survey (DHS)	CBH
Rwanda	2007	Demographic and Health Survey (DHS)	CBH
Rwanda	2010	Demographic and Health Survey (DHS)	CBH
Rwanda	2014	Demographic and Health Survey (DHS)	CBH
Somalia	1999	Multiple Indicator Cluster Survey (MICS)	SBH
Somalia	2006	Multiple Indicator Cluster Survey (MICS)	CBH
Somalia	2006	Multiple Indicator Cluster Survey (MICS)	SBH
South Sudan	2008	Census (IPUMS)	SBH
Tanzania	1991	Demographic and Health Survey (DHS)	CBH
Tanzania	1996	Demographic and Health Survey (DHS)	CBH
Tanzania	1999	Demographic and Health Survey (DHS)	CBH
Tanzania	2004	Demographic and Health Survey (DHS)	CBH
Tanzania	2009	Demographic and Health Survey (DHS)	CBH
Tanzania	2015	Demographic and Health Survey (DHS)	CBH
Uganda	1988	Demographic and Health Survey (DHS)	CBH
Uganda	1995	Demographic and Health Survey (DHS)	CBH
Uganda	2000	Demographic and Health Survey (DHS)	CBH

Appendix Table 10. Under-5 mortality reference sources by source date and location, 1950-2016			
**Location**	**Source**	**Source years**	**Method**
Uganda	2006	Demographic and Health Survey (DHS)	CBH
Uganda	2011	Demographic and Health Survey (DHS)	CBH
Zambia	1992	Demographic and Health Survey (DHS)	CBH
Zambia	1996	Demographic and Health Survey (DHS)	CBH
Zambia	2001	Demographic and Health Survey (DHS)	CBH
Zambia	2007	Demographic and Health Survey (DHS)	CBH
Zambia	2013	Demographic and Health Survey (DHS)	CBH
Botswana	1988	Demographic and Health Survey (DHS)	CBH
Lesotho	1986	Census	SBH
Lesotho	2001	Demographic Survey	SBH
Lesotho	2011	Demographic Survey	SBH
Lesotho	2004	Demographic and Health Survey (DHS)	CBH
Lesotho	2004	Demographic and Health Survey (DHS)	SBH
Lesotho	2009	Demographic and Health Survey (DHS)	SBH
Lesotho	2009	Demographic and Health Survey (DHS)	CBH
Lesotho	2014	Demographic and Health Survey (DHS)	CBH
Lesotho	2000	Multiple Indicator Cluster Survey (MICS)	SBH
Namibia	1992	Demographic and Health Survey (DHS)	CBH
Namibia	2000	Demographic and Health Survey (DHS)	CBH
Namibia	2006	Demographic and Health Survey (DHS)	CBH
Namibia	2013	Demographic and Health Survey (DHS)	CBH
South Africa	1998	Demographic and Health Survey (DHS)	CBH
South Africa	2006	Survey (IPUMS)	HH
South Africa	2012	Vital Registration	VR/SRS/DSP
Eastern Cape	1996	Census (IPUMS)	SBH
Free State	2000	Census	HH
Free State	2006	Census	HH
Free State	2011	Census	HH
Free State	1996	Census (IPUMS)	SBH
Gauteng	2000	Census	HH
Gauteng	2006	Census	HH
Gauteng	2011	Census	HH
Gauteng	1996	Census (IPUMS)	SBH
KwaZulu-Natal	1996	Census (IPUMS)	SBH
Limpopo	1996	Census (IPUMS)	SBH
Mpumalanga	1996	Census (IPUMS)	SBH
North-West	2000	Census	HH
North-West	2006	Census	HH
North-West	2011	Census	HH
North-West	1996	Census (IPUMS)	SBH
Northern Cape	1996	Census (IPUMS)	SBH
Western Cape	1996	Census (IPUMS)	SBH
Swaziland	2006	Demographic and Health Survey (DHS)	SBH
Swaziland	2006	Demographic and Health Survey (DHS)	CBH
Swaziland	2000	Multiple Indicator Cluster Survey (MICS)	SBH
Swaziland	2010	Multiple Indicator Cluster Survey (MICS)	SBH
Swaziland	2010	Multiple Indicator Cluster Survey (MICS)	CBH
Swaziland	2014	Multiple Indicator Cluster Survey (MICS)	SBH
Swaziland	2014	Multiple Indicator Cluster Survey (MICS)	CBH
Zimbabwe	1988	Demographic and Health Survey (DHS)	CBH
Zimbabwe	1994	Demographic and Health Survey (DHS)	CBH
Zimbabwe	1999	Demographic and Health Survey (DHS)	CBH
Zimbabwe	2005	Demographic and Health Survey (DHS)	CBH
Zimbabwe	2010	Demographic and Health Survey (DHS)	CBH
Zimbabwe	2015	Demographic and Health Survey (DHS)	CBH
Benin	1996	Demographic and Health Survey (DHS)	CBH
Benin	2001	Demographic and Health Survey (DHS)	CBH
Benin	2006	Demographic and Health Survey (DHS)	CBH
Burkina Faso	1992	Demographic and Health Survey (DHS)	CBH
Burkina Faso	1998	Demographic and Health Survey (DHS)	CBH
Burkina Faso	2003	Demographic and Health Survey (DHS)	CBH
Burkina Faso	2010	Demographic and Health Survey (DHS)	CBH
Cameroon	1991	Demographic and Health Survey (DHS)	CBH
Cameroon	1998	Demographic and Health Survey (DHS)	CBH
Cameroon	2004	Demographic and Health Survey (DHS)	CBH
Cameroon	2011	Demographic and Health Survey (DHS)	CBH
Cape Verde	1955-2012	Vital Registration	VR/SRS/DSP

Appendix Table 10. Under-5 mortality reference sources by source date and location, 1950-2016			
**Location**	**Source**	**Source years**	**Method**
Chad	1996	Demographic and Health Survey (DHS)	CBH
Chad	2004	Demographic and Health Survey (DHS)	CBH
Chad	2014	Demographic and Health Survey (DHS)	CBH
Cote d'Ivoire	1994	Demographic and Health Survey (DHS)	CBH
Cote d'Ivoire	1998	Demographic and Health Survey (DHS)	CBH
Cote d'Ivoire	2011	Demographic and Health Survey (DHS)	CBH
The Gambia	2013	Demographic and Health Survey (DHS)	CBH
Ghana	1988	Demographic and Health Survey (DHS)	CBH
Ghana	1993	Demographic and Health Survey (DHS)	CBH
Ghana	1998	Demographic and Health Survey (DHS)	CBH
Ghana	2003	Demographic and Health Survey (DHS)	CBH
Ghana	2008	Demographic and Health Survey (DHS)	CBH
Ghana	2014	Demographic and Health Survey (DHS)	CBH
Ghana	2007	Special Demographic and Health Survey	CBH
Guinea	1992	Demographic and Health Survey (DHS)	CBH
Guinea	1999	Demographic and Health Survey (DHS)	CBH
Guinea	2005	Demographic and Health Survey (DHS)	CBH
Guinea	2012	Demographic and Health Survey (DHS)	CBH
Guinea-Bissau	2000	Multiple Indicator Cluster Survey (MICS)	SBH
Guinea-Bissau	2006	Multiple Indicator Cluster Survey (MICS)	SBH
Guinea-Bissau	2010	Multiple Indicator Cluster Survey (MICS)	SBH
Guinea-Bissau	2014	Multiple Indicator Cluster Survey (MICS)	CBH
Guinea-Bissau	2014	Multiple Indicator Cluster Survey (MICS)	SBH
Liberia	1986	Demographic and Health Survey (DHS)	CBH
Liberia	2006	Demographic and Health Survey (DHS)	CBH
Liberia	2013	Demographic and Health Survey (DHS)	CBH
Mali	1987	Demographic and Health Survey (DHS)	CBH
Mali	1995	Demographic and Health Survey (DHS)	CBH
Mali	2001	Demographic and Health Survey (DHS)	CBH
Mali	2006	Demographic and Health Survey (DHS)	CBH
Mali	2012	Demographic and Health Survey (DHS)	CBH
Mauritania	2000	Demographic and Health Survey (DHS)	CBH
Niger	1992	Demographic and Health Survey (DHS)	CBH
Niger	1998	Demographic and Health Survey (DHS)	CBH
Niger	2006	Demographic and Health Survey (DHS)	CBH
Niger	2012	Demographic and Health Survey (DHS)	CBH
Nigeria	1990	Demographic and Health Survey (DHS)	CBH
Nigeria	2003	Demographic and Health Survey (DHS)	CBH
Nigeria	2008	Demographic and Health Survey (DHS)	CBH
Nigeria	2013	Demographic and Health Survey (DHS)	CBH
Sao Tome and Principe	1991	Census	SBH
Sao Tome and Principe	2008	Demographic and Health Survey (DHS)	CBH
Sao Tome and Principe	2008	Demographic and Health Survey (DHS)	SBH
Sao Tome and Principe	2000	Multiple Indicator Cluster Survey (MICS)	SBH
Sao Tome and Principe	2006	Multiple Indicator Cluster Survey (MICS)	SBH
Sao Tome and Principe	2014	Multiple Indicator Cluster Survey (MICS)	SBH
Sao Tome and Principe	2014	Multiple Indicator Cluster Survey (MICS)	CBH
Sao Tome and Principe	1955-1987	Vital Registration	VR/SRS/DSP
Senegal	1986	Demographic and Health Survey (DHS)	CBH
Senegal	1992	Demographic and Health Survey (DHS)	CBH
Senegal	1997	Demographic and Health Survey (DHS)	CBH
Senegal	2005	Demographic and Health Survey (DHS)	CBH
Senegal	2010	Demographic and Health Survey (DHS)	CBH
Senegal	2012	Demographic and Health Survey (DHS)	CBH
Senegal	2014	Demographic and Health Survey (DHS)	CBH
Senegal	2015	Demographic and Health Survey (DHS)	CBH
Sierra Leone	2008	Demographic and Health Survey (DHS)	CBH
Sierra Leone	2013	Demographic and Health Survey (DHS)	CBH
Togo	1988	Demographic and Health Survey (DHS)	CBH
Togo	1998	Demographic and Health Survey (DHS)	CBH
Togo	2013	Demographic and Health Survey (DHS)	CBH

Appendix Table 11A. Under-5 mortality rates by location, both sexes combined, 1970, 1975, 1980, 1985, 1990, 1995, 2000, 2005, 2010, 2016										
**Location**	**1970**	**1975**	**1980**	**1985**	**1990**	**1995**	**2000**	**2005**	**2010**	**2016**
Global	144·60 (137·92 to 151·66)	127·15 (122·28 to 132·23)	111·19 (107·16 to 115·19)	97·34 (93·96 to 100·55)	85·08 (82·15 to 87·96)	77·56 (74·99 to 80·11)	69·39 (67·16 to 71·80)	59·84 (57·31 to 62·43)	49·82 (46·43 to 53·51)	38·42 (34·48 to 43·05)
High SDI	88·08 (82·31 to 94·51)	69·40 (65·89 to 73·12)	56·63 (53·23 to 60·04)	45·18 (42·80 to 47·69)	37·49 (35·57 to 39·71)	31·39 (29·67 to 33·16)	24·73 (23·11 to 26·44)	18·85 (17·41 to 20·47)	14·46 (13·06 to 16·00)	11·33 (9·80 to 13·16)
High-middle SDI	26·16 (25·58 to 26·83)	20·55 (20·00 to 21·17)	15·61 (15·17 to 16·15)	12·30 (12·03 to 12·61)	10·26 (10·08 to 10·50)	8·21 (8·07 to 8·38)	6·89 (6·84 to 6·95)	6·23 (6·18 to 6·28)	5·42 (5·37 to 5·47)	4·93 (4·58 to 5·33)
Middle SDI	207·59 (197·60 to 218·10)	182·67 (174·52 to 191·11)	159·20 (152·84 to 165·09)	139·23 (134·26 to 144·14)	120·16 (115·86 to 124·43)	107·35 (103·45 to 111·13)	94·08 (90·82 to 97·41)	80·47 (77·13 to 84·01)	66·20 (61·66 to 70·94)	49·56 (44·81 to 55·19)
Low-middle SDI	238·21 (220·73 to 256·90)	223·15 (212·32 to 233·78)	205·89 (198·49 to 213·76)	190·14 (183·90 to 196·71)	173·50 (167·83 to 179·42)	159·64 (154·34 to 165·05)	140·71 (135·75 to 145·70)	116·92 (111·67 to 122·35)	96·16 (88·94 to 104·10)	70·18 (60·87 to 80·70)
Low SDI	135·90 (127·88 to 144·27)	111·88 (106·01 to 117·74)	91·34 (86·64 to 96·34)	74·26 (70·49 to 78·14)	60·05 (56·90 to 63·46)	49·21 (46·90 to 51·54)	40·22 (38·34 to 42·22)	32·56 (30·73 to 34·29)	25·45 (23·49 to 27·59)	19·25 (16·69 to 22·17)
High-income	29·66 (29·10 to 30·30)	23·64 (23·08 to 24·25)	17·08 (16·65 to 17·62)	13·22 (12·96 to 13·53)	11·23 (11·06 to 11·47)	8·94 (8·81 to 9·10)	7·56 (7·52 to 7·61)	6·77 (6·73 to 6·83)	5·94 (5·89 to 5·99)	5·40 (5·08 to 5·77)
High-income North America	23·92 (23·76 to 24·07)	19·04 (18·90 to 19·16)	15·05 (14·94 to 15·17)	12·51 (12·40 to 12·61)	10·90 (10·81 to 10·99)	8·97 (8·89 to 9·05)	8·05 (7·98 to 8·13)	7·88 (7·81 to 7·96)	7·07 (7·00 to 7·13)	6·69 (6·46 to 6·95)
Canada	22·86 (22·43 to 23·25)	17·29 (16·97 to 17·62)	12·54 (12·27 to 12·82)	9·68 (9·46 to 9·89)	8·29 (8·09 to 8·51)	7·27 (7·08 to 7·46)	6·18 (6·00 to 6·35)	6·09 (5·91 to 6·27)	5·72 (5·56 to 5·88)	5·37 (5·18 to 5·56)
Greenland	46·81 (43·14 to 50·05)	39·46 (36·31 to 42·82)	39·08 (35·83 to 42·68)	38·04 (34·51 to 41·91)	38·73 (34·94 to 42·99)	27·04 (24·25 to 30·14)	19·41 (17·20 to 21·99)	18·06 (15·89 to 20·71)	16·32 (14·16 to 18·72)	14·14 (11·85 to 16·88)
United States	24·08 (23·78 to 24·40)	19·24 (18·99 to 19·50)	15·30 (15·10 to 15·52)	12·76 (12·57 to 12·96)	11·10 (10·94 to 11·27)	9·09 (8·97 to 9·23)	8·20 (8·11 to 8·30)	8·05 (7·93 to 8·17)	7·21 (7·09 to 7·33)	6·85 (6·66 to 7·04)
Australasia	21·50 (21·15 to 21·84)	17·88 (17·57 to 18·20)	13·58 (13·30 to 13·86)	11·96 (11·70 to 12·23)	9·67 (9·43 to 9·90)	7·35 (7·17 to 7·53)	6·59 (6·42 to 6·75)	6·00 (5·83 to 6·17)	5·16 (5·01 to 5·31)	4·16 (3·84 to 4·52)
Australia	21·63 (21·20 to 22·05)	17·63 (17·27 to 18·02)	13·11 (12·78 to 13·42)	11·59 (11·30 to 11·88)	9·40 (9·12 to 9·66)	7·05 (6·85 to 7·26)	6·37 (6·18 to 6·56)	5·85 (5·68 to 6·03)	4·90 (4·75 to 5·08)	3·89 (3·59 to 4·24)
New Zealand	20·93 (20·38 to 21·50)	18·92 (18·38 to 19·41)	15·68 (15·19 to 16·16)	13·69 (13·29 to 14·13)	10·89 (10·53 to 11·26)	8·66 (8·37 to 8·97)	7·54 (7·26 to 7·84)	6·65 (6·38 to 6·95)	6·35 (6·10 to 6·59)	5·57 (5·18 to 6·00)
High-income Asia Pacific	30·77 (28·25 to 33·74)	21·82 (19·42 to 24·42)	15·29 (13·22 to 17·81)	10·63 (9·27 to 12·23)	8·30 (7·31 to 9·72)	6·77 (6·10 to 7·55)	5·34 (5·26 to 5·43)	4·29 (4·21 to 4·36)	3·45 (3·38 to 3·53)	2·92 (2·64 to 3·23)
Brunei	69·59 (66·95 to 72·45)	43·49 (41·43 to 45·66)	23·82 (22·44 to 25·32)	15·26 (14·31 to 16·25)	11·56 (10·81 to 12·43)	9·73 (9·06 to 10·52)	8·83 (8·23 to 9·48)	8·75 (8·17 to 9·37)	8·64 (7·98 to 9·34)	9·17 (8·04 to 10·49)
Japan	17·37 (17·18 to 17·57)	13·68 (13·51 to 13·85)	10·10 (9·97 to 10·25)	7·44 (7·31 to 7·58)	6·35 (6·23 to 6·47)	5·93 (5·76 to 6·11)	4·52 (4·43 to 4·62)	3·77 (3·69 to 3·85)	3·16 (3·08 to 3·25)	2·64 (2·41 to 2·89)
Singapore	27·58 (26·77 to 28·41)	18·95 (18·27 to 19·65)	14·94 (14·39 to 15·51)	10·71 (10·25 to 11·22)	7·54 (7·19 to 7·86)	5·11 (4·85 to 5·39)	3·84 (3·62 to 4·08)	3·03 (2·84 to 3·26)	2·71 (2·53 to 2·91)	2·27 (2·04 to 2·53)
South Korea	57·67 (50·20 to 66·34)	39·54 (32·07 to 47·63)	25·13 (19·13 to 32·48)	17·64 (13·17 to 22·74)	11·99 (9·02 to 16·23)	8·27 (6·51 to 10·38)	7·05 (6·87 to 7·24)	5·58 (5·40 to 5·75)	4·11 (3·97 to 4·25)	3·52 (2·78 to 4·39)
Western Europe	26·32 (26·14 to 26·53)	20·77 (20·59 to 20·94)	14·90 (14·80 to 15·02)	11·31 (11·19 to 11·42)	9·32 (9·23 to 9·41)	6·91 (6·82 to 6·99)	5·73 (5·66 to 5·80)	4·83 (4·77 to 4·89)	4·26 (4·20 to 4·32)	3·70 (3·26 to 4·19)
Andorra	22·33 (17·39 to 28·62)	13·11 (10·18 to 16·67)	7·61 (5·91 to 9·79)	6·79 (5·30 to 8·54)	6·09 (4·80 to 7·71)	4·34 (3·44 to 5·40)	3·17 (2·50 to 3·97)	3·31 (2·64 to 4·17)	3·17 (2·52 to 3·97)	3·31 (2·60 to 4·27)
Austria	29·67 (28·80 to 30·49)	24·09 (23·29 to 24·85)	16·90 (16·27 to 17·58)	13·26 (12·68 to 13·80)	9·68 (9·24 to 10·16)	6·79 (6·45 to 7·17)	5·72 (5·39 to 6·06)	4·92 (4·63 to 5·24)	4·55 (4·27 to 4·83)	3·37 (2·81 to 4·05)
Belgium	24·63 (24·27 to 25·02)	19·31 (18·97 to 19·65)	14·82 (14·55 to 15·12)	11·77 (11·52 to 12·04)	9·99 (9·76 to 10·21)	7·63 (7·42 to 7·82)	5·85 (5·67 to 6·02)	4·94 (4·79 to 5·10)	4·49 (4·35 to 4·66)	3·95 (3·61 to 4·33)
Cyprus	40·31 (36·31 to 44·32)	30·90 (29·49 to 32·28)	20·47 (19·65 to 21·33)	15·25 (14·61 to 16·02)	12·85 (12·26 to 13·48)	10·46 (9·90 to 11·03)	8·11 (7·60 to 8·64)	6·16 (5·70 to 6·63)	4·35 (3·95 to 4·77)	3·28 (2·82 to 3·80)
Denmark	17·12 (16·49 to 17·77)	12·62 (12·11 to 13·15)	10·31 (9·85 to 10·79)	9·58 (9·08 to 10·10)	9·02 (8·60 to 9·51)	6·67 (6·33 to 7·03)	5·60 (5·30 to 5·93)	4·88 (4·60 to 5·17)	4·11 (3·85 to 4·38)	4·07 (3·50 to 4·74)
Finland	16·47 (15·68 to 17·34)	12·72 (12·00 to 13·50)	8·88 (8·34 to 9·48)	7·34 (6·85 to 7·83)	6·82 (6·32 to 7·34)	5·00 (4·62 to 5·39)	4·24 (3·89 to 4·63)	3·95 (3·60 to 4·32)	3·01 (2·73 to 3·32)	2·19 (1·77 to 2·70)
France	18·40 (18·12 to 18·68)	16·51 (16·25 to 16·76)	12·48 (12·26 to 12·74)	10·02 (9·81 to 10·22)	8·87 (8·67 to 9·07)	6·11 (5·94 to 6·28)	5·39 (5·24 to 5·56)	4·39 (4·26 to 4·53)	4·19 (4·06 to 4·31)	3·82 (3·18 to 4·59)
Germany	25·86 (25·58 to 26·14)	21·72 (21·42 to 22·01)	15·27 (15·03 to 15·54)	11·04 (10·84 to 11·27)	9·11 (8·91 to 9·31)	6·52 (6·36 to 6·70)	5·38 (5·23 to 5·54)	4·75 (4·61 to 4·90)	4·22 (4·08 to 4·36)	3·61 (3·00 to 4·44)
Greece	34·05 (33·38 to 34·74)	26·83 (26·24 to 27·40)	20·29 (19·80 to 20·78)	15·44 (15·01 to 15·86)	10·69 (10·35 to 11·05)	8·61 (8·33 to 8·91)	6·34 (6·10 to 6·58)	4·55 (4·36 to 4·74)	4·04 (3·87 to 4·22)	3·91 (3·56 to 4·31)
Iceland	15·99 (14·77 to 17·28)	13·33 (12·30 to 14·54)	9·72 (8·85 to 10·66)	7·88 (7·12 to 8·71)	6·53 (5·92 to 7·26)	5·85 (5·20 to 6·58)	4·01 (3·55 to 4·57)	3·17 (2·79 to 3·62)	2·61 (2·26 to 3·00)	2·20 (1·85 to 2·62)
Ireland	22·64 (21·84 to 23·47)	20·34 (19·60 to 21·11)	14·17 (13·58 to 14·75)	10·73 (10·25 to 11·22)	9·34 (8·88 to 9·81)	7·38 (6·96 to 7·80)	7·25 (6·85 to 7·68)	4·98 (4·68 to 5·29)	4·19 (3·94 to 4·44)	3·91 (3·37 to 4·54)
Israel	30·47 (28·10 to 33·15)	29·16 (28·27 to 30·10)	17·18 (16·53 to 17·84)	13·76 (13·20 to 14·33)	11·86 (11·40 to 12·32)	8·88 (8·53 to 9·27)	7·08 (6·79 to 7·39)	5·63 (5·39 to 5·88)	4·62 (4·41 to 4·84)	3·58 (3·13 to 4·11)
Italy	32·75 (32·40 to 33·11)	23·32 (23·00 to 23·62)	16·46 (16·14 to 16·80)	11·91 (11·65 to 12·16)	9·29 (9·07 to 9·52)	7·38 (7·17 to 7·59)	5·34 (5·18 to 5·51)	4·28 (4·14 to 4·44)	3·97 (3·84 to 4·11)	3·21 (2·53 to 4·05)
Luxembourg	24·07 (22·49 to 25·86)	17·00 (15·77 to 18·35)	13·53 (12·41 to 14·72)	11·29 (10·36 to 12·29)	8·99 (8·15 to 9·85)	5·92 (5·38 to 6·53)	4·68 (4·22 to 5·22)	3·40 (3·05 to 3·85)	2·93 (2·56 to 3·34)	2·15 (1·82 to 2·56)
Malta	27·95 (26·39 to 29·81)	20·57 (19·21 to 21·99)	16·40 (15·31 to 17·50)	13·82 (12·79 to 14·84)	10·45 (9·63 to 11·26)	9·57 (8·74 to 10·38)	7·76 (7·01 to 8·54)	7·26 (6·55 to 8·10)	7·30 (6·54 to 8·12)	6·28 (5·38 to 7·34)
Netherlands	16·23 (15·83 to 16·66)	13·77 (13·37 to 14·18)	10·85 (10·50 to 11·20)	9·81 (9·47 to 10·18)	8·67 (8·37 to 8·97)	7·00 (6·74 to 7·29)	6·33 (6·09 to 6·58)	5·55 (5·33 to 5·77)	4·42 (4·23 to 4·62)	3·80 (3·45 to 4·20)
Norway	16·71 (16·04 to 17·40)	13·73 (13·11 to 14·35)	10·26 (9·72 to 10·89)	10·28 (9·75 to 10·84)	8·61 (8·15 to 9·09)	5·55 (5·23 to 5·87)	4·94 (4·63 to 5·23)	4·01 (3·75 to 4·30)	3·24 (3·01 to 3·47)	2·71 (2·31 to 3·19)
Portugal	64·48 (63·98 to 64·98)	43·44 (42·95 to 43·95)	29·10 (28·78 to 29·44)	20·64 (20·36 to 20·92)	14·16 (13·92 to 14·38)	9·72 (9·53 to 9·92)	7·02 (6·85 to 7·20)	4·98 (4·84 to 5·15)	3·77 (3·64 to 3·90)	3·12 (2·91 to 3·35)
Spain	31·70 (31·30 to 32·12)	21·82 (21·48 to 22·15)	15·16 (14·89 to 15·45)	11·11 (10·85 to 11·37)	9·39 (9·13 to 9·66)	6·91 (6·70 to 7·12)	5·30 (5·12 to 5·49)	4·60 (4·44 to 4·77)	3·85 (3·71 to 3·99)	3·26 (2·83 to 3·77)
Sweden	13·35 (12·74 to 13·92)	10·44 (9·93 to 11·00)	8·08 (7·66 to 8·57)	7·08 (6·67 to 7·51)	6·91 (6·54 to 7·30)	4·75 (4·47 to 5·04)	3·95 (3·66 to 4·25)	3·36 (3·12 to 3·61)	3·13 (2·88 to 3·41)	2·64 (2·16 to 3·22)
Stockholm	13·29 (9·98 to 17·56)	10·70 (7·87 to 14·10)	8·71 (7·73 to 9·82)	7·04 (6·26 to 7·87)	6·82 (6·19 to 7·48)	4·90 (4·36 to 5·52)	3·92 (3·44 to 4·41)	3·28 (2·89 to 3·71)	2·57 (2·24 to 2·91)	2·28 (1·85 to 2·80)
Sweden except Stockholm	13·40 (12·56 to 14·23)	10·40 (9·65 to 11·12)	7·97 (7·52 to 8·45)	7·09 (6·67 to 7·51)	6·91 (6·51 to 7·32)	4·71 (4·42 to 5·00)	3·95 (3·66 to 4·23)	3·39 (3·14 to 3·65)	3·28 (3·02 to 3·54)	2·74 (2·21 to 3·38)
Switzerland	18·41 (17·89 to 18·95)	13·73 (13·27 to 14·18)	10·44 (10·03 to 10·86)	8·85 (8·49 to 9·22)	8·47 (8·10 to 8·82)	6·28 (5·99 to 6·61)	5·81 (5·53 to 6·11)	5·02 (4·75 to 5·30)	4·46 (4·22 to 4·72)	3·95 (3·31 to 4·72)
United Kingdom	21·66 (21·32 to 22·00)	18·43 (18·11 to 18·74)	14·51 (14·24 to 14·78)	11·37 (11·13 to 11·60)	9·52 (9·33 to 9·73)	7·33 (7·17 to 7·50)	6·63 (6·44 to 6·82)	5·92 (5·82 to 6·03)	5·12 (4·99 to 5·25)	4·57 (4·19 to 4·99)
England	20·92 (20·35 to 21·44)	17·77 (17·34 to 18·18)	14·21 (13·90 to 14·54)	11·19 (10·99 to 11·40)	9·53 (9·35 to 9·71)	7·28 (7·13 to 7·43)	6·64 (6·43 to 6·86)	5·91 (5·79 to 6·05)	5·14 (5·02 to 5·27)	4·59 (4·26 to 4·95)
East Midlands	20·43 (19·02 to 21·84)	17·33 (16·34 to 18·25)	13·84 (12·90 to 14·72)	10·61 (10·06 to 11·19)	9·57 (9·09 to 10·06)	7·09 (6·72 to 7·47)	6·53 (6·13 to 6·97)	5·90 (5·58 to 6·24)	5·13 (4·83 to 5·42)	5·06 (4·56 to 5·63)
East of England	18·90 (16·12 to 21·90)	15·63 (13·79 to 17·53)	12·35 (11·22 to 13·54)	9·77 (9·31 to 10·24)	7·48 (7·06 to 7·91)	6·25 (5·90 to 6·66)	5·35 (5·03 to 5·68)	4·94 (4·64 to 5·25)	4·46 (4·17 to 4·79)	3·89 (3·25 to 4·57)
Greater London	22·14 (20·30 to 23·85)	19·01 (18·13 to 19·90)	15·36 (14·86 to 15·83)	11·88 (11·53 to 12·25)	10·24 (9·93 to 10·57)	8·12 (7·84 to 8·43)	7·32 (7·04 to 7·64)	6·09 (5·86 to 6·33)	5·37 (5·14 to 5·63)	4·09 (3·61 to 4·59)
North East England	20·34 (18·05 to 22·72)	17·30 (16·11 to 18·48)	13·86 (12·83 to 14·96)	10·87 (10·31 to 11·48)	9·78 (9·23 to 10·36)	7·60 (7·15 to 8·10)	6·84 (6·31 to 7·39)	5·60 (5·13 to 6·06)	4·62 (4·28 to 5·00)	4·35 (3·86 to 4·91)
North West England	21·69 (17·92 to 26·01)	18·62 (14·85 to 23·17)	14·92 (13·60 to 16·33)	11·66 (11·23 to 12·10)	9·84 (9·46 to 10·26)	7·56 (7·20 to 7·93)	7·22 (6·84 to 7·61)	6·53 (6·21 to 6·85)	5·64 (5·34 to 5·94)	5·02 (4·51 to 5·56)
South East England	18·86 (17·25 to 20·52)	15·93 (15·12 to 16·74)	12·74 (12·24 to 13·22)	10·34 (10·00 to 10·69)	8·65 (8·31 to 9·00)	6·05 (5·80 to 6·33)	5·28 (5·01 to 5·57)	4·76 (4·54 to 4·98)	4·26 (4·02 to 4·49)	3·74 (3·36 to 4·14)
South West England	19·78 (18·13 to 21·47)	16·61 (15·58 to 17·64)	13·21 (12·32 to 14·17)	10·36 (9·80 to 10·96)	8·39 (7·94 to 8·81)	6·48 (6·13 to 6·85)	5·66 (5·27 to 6·07)	5·10 (4·82 to 5·43)	4·30 (4·02 to 4·57)	4·26 (3·82 to 4·76)
West Midlands	22·30 (20·64 to 24·07)	19·14 (18·12 to 20·20)	15·44 (14·87 to 15·96)	12·45 (11·89 to 13·04)	11·45 (10·98 to 11·89)	8·13 (7·74 to 8·50)	7·84 (7·41 to 8·27)	7·46 (7·14 to 7·79)	6·47 (6·15 to 6·80)	6·60 (5·98 to 7·25)
Yorkshire and the Humber	22·30 (20·63 to 23·93)	19·09 (18·16 to 20·00)	15·36 (14·49 to 16·31)	12·23 (11·84 to 12·62)	10·23 (9·78 to 10·68)	8·23 (7·87 to 8·62)	7·74 (7·37 to 8·14)	6·91 (6·48 to 7·37)	5·88 (5·59 to 6·18)	5·20 (4·70 to 5·73)

Appendix Table 11A. Under-5 mortality rates by location, both sexes combined, 1970, 1975, 1980, 1985, 1990, 1995, 2000, 2005, 2010, 2016

Location	1970	1975	1980	1985	1990	1995	2000	2005	2010	2016
Northern Ireland	27·46 (25·89 to 29·09)	21·80 (20·49 to 23·09)	16·06 (14·95 to 17·19)	12·17 (11·27 to 13·21)	9·52 (8·73 to 10·41)	8·25 (7·50 to 9·02)	6·54 (5·86 to 7·27)	6·58 (5·98 to 7·26)	5·94 (5·34 to 6·61)	4·81 (3·28 to 6·82)
Scotland	22·91 (22·19 to 23·58)	20·12 (19·41 to 20·84)	14·35 (13·77 to 14·96)	11·59 (11·02 to 12·13)	9·51 (9·03 to 10·02)	7·45 (7·01 to 7·88)	6·48 (6·07 to 6·86)	5·91 (5·52 to 6·29)	4·52 (4·23 to 4·81)	4·08 (3·20 to 5·15)
Wales	22·79 (17·69 to 29·39)	19·64 (15·08 to 25·05)	15·77 (13·95 to 17·77)	11·72 (11·01 to 12·47)	8·78 (8·23 to 9·34)	7·18 (6·70 to 7·67)	6·35 (5·90 to 6·85)	5·24 (4·84 to 5·67)	4·69 (4·35 to 5·06)	4·35 (3·55 to 5·30)
Southern Latin America	84·98 (84·56 to 85·37)	66·93 (66·43 to 67·51)	43·24 (43·01 to 43·48)	32·38 (32·19 to 32·56)	28·52 (28·34 to 28·70)	23·44 (23·29 to 23·60)	17·78 (17·65 to 17·92)	14·56 (14·44 to 14·67)	12·68 (12·54 to 12·83)	11·00 (10·46 to 11·61)
Argentina	82·51 (81·89 to 83·14)	66·09 (65·33 to 66·92)	44·25 (43·97 to 44·55)	35·03 (34·80 to 35·28)	32·59 (32·35 to 32·81)	27·98 (27·77 to 28·20)	20·46 (20·30 to 20·64)	16·32 (16·18 to 16·47)	14·00 (13·87 to 14·14)	12·20 (11·73 to 12·72)
Chile	96·64 (96·04 to 97·24)	71·00 (70·48 to 71·53)	39·81 (39·42 to 40·20)	24·99 (24·70 to 25·29)	20·28 (20·02 to 20·54)	14·09 (13·90 to 14·28)	11·04 (10·87 to 11·21)	9·42 (9·26 to 9·58)	9·07 (8·87 to 9·31)	7·85 (7·41 to 8·32)
Uruguay	55·02 (53·42 to 56·63)	58·87 (57·25 to 60·60)	46·66 (45·09 to 48·26)	36·83 (35·42 to 38·26)	25·79 (24·74 to 26·87)	21·91 (20·95 to 22·80)	16·01 (15·24 to 16·76)	14·18 (13·49 to 14·94)	10·81 (9·62 to 12·31)	8·76 (6·75 to 11·31)
Central Europe, Eastern Europe, and Central Asi	52·81 (47·36 to 58·98)	44·97 (42·30 to 47·98)	40·32 (38·43 to 42·41)	36·37 (34·98 to 37·80)	32·35 (31·06 to 33·69)	33·26 (31·57 to 35·13)	28·00 (26·20 to 29·97)	21·86 (19·84 to 24·11)	16·76 (14·71 to 19·16)	13·55 (10·91 to 17·05)
Eastern Europe	32·66 (28·95 to 37·63)	31·87 (29·00 to 35·53)	29·55 (27·78 to 31·61)	25·79 (24·68 to 27·07)	21·34 (20·37 to 22·40)	22·52 (21·37 to 23·81)	19·83 (18·64 to 21·24)	13·90 (13·07 to 14·90)	9·91 (9·25 to 10·70)	8·35 (7·61 to 9·38)
Belarus	36·13 (29·02 to 44·28)	30·14 (24·67 to 36·55)	26·30 (21·70 to 31·36)	23·89 (19·97 to 28·57)	20·58 (17·15 to 24·61)	18·72 (15·48 to 22·38)	16·07 (13·17 to 19·31)	11·65 (9·39 to 14·20)	7·95 (6·21 to 10·17)	5·53 (4·19 to 7·43)
Estonia	21·84 (20·54 to 23·20)	22·17 (20·87 to 23·53)	22·67 (21·33 to 24·07)	20·25 (19·00 to 21·61)	16·90 (15·85 to 17·98)	18·50 (17·15 to 19·75)	11·28 (10·26 to 12·34)	7·28 (6·56 to 8·18)	4·80 (4·22 to 5·44)	3·15 (2·40 to 4·07)
Latvia	22·08 (20·84 to 23·32)	25·93 (24·58 to 27·31)	21·57 (20·41 to 22·75)	17·39 (16·34 to 18·45)	17·60 (16·63 to 18·63)	20·77 (19·55 to 22·01)	13·14 (12·12 to 14·32)	9·90 (9·00 to 10·83)	7·85 (7·10 to 8·69)	4·82 (3·32 to 6·80)
Lithuania	23·13 (22·27 to 24·05)	23·91 (23·05 to 24·93)	19·25 (18·42 to 20·07)	18·91 (18·09 to 19·72)	13·73 (13·14 to 14·38)	15·81 (15·09 to 16·51)	11·24 (10·62 to 11·87)	8·76 (8·18 to 9·33)	5·92 (5·53 to 6·39)	4·66 (4·09 to 5·30)
Moldova	71·19 (47·98 to 102·04)	61·83 (42·52 to 86·87)	55·90 (44·36 to 69·31)	43·26 (35·44 to 51·99)	32·28 (26·30 to 38·81)	35·07 (28·89 to 42·29)	31·40 (25·25 to 38·27)	16·81 (12·87 to 21·44)	16·09 (12·07 to 20·75)	11·63 (7·71 to 16·80)
Russia	28·15 (28·03 to 28·29)	29·74 (29·60 to 29·86)	28·28 (28·05 to 28·51)	26·16 (25·87 to 26·42)	21·53 (21·43 to 21·63)	23·08 (22·84 to 23·31)	19·83 (19·46 to 20·22)	13·72 (13·61 to 13·83)	9·61 (9·54 to 9·68)	8·37 (8·09 to 8·70)
Ukraine	41·16 (26·60 to 59·55)	35·98 (24·92 to 49·77)	32·17 (24·58 to 41·17)	24·14 (19·56 to 29·97)	20·61 (16·68 to 24·96)	21·11 (17·00 to 26·02)	20·91 (16·18 to 26·51)	15·42 (11·65 to 20·11)	11·33 (8·33 to 14·92)	9·18 (5·68 to 14·10)
Central Europe	44·49 (41·41 to 48·23)	34·19 (32·09 to 36·62)	28·56 (27·26 to 30·00)	24·51 (23·69 to 25·41)	21·80 (21·27 to 22·40)	18·64 (18·20 to 19·12)	13·73 (13·27 to 14·21)	10·58 (10·26 to 10·92)	7·90 (7·67 to 8·14)	6·20 (5·12 to 7·61)
Albania	108·99 (73·32 to 158·98)	79·04 (54·18 to 110·32)	62·35 (47·10 to 80·39)	54·43 (46·30 to 64·19)	40·70 (34·65 to 47·59)	36·90 (31·43 to 43·17)	27·47 (22·65 to 33·11)	21·94 (17·01 to 27·62)	17·34 (12·24 to 23·89)	13·72 (9·12 to 20·19)
Bosnia and Herzegovina	54·29 (39·33 to 74·75)	42·98 (32·84 to 55·76)	33·08 (27·08 to 39·84)	24·85 (23·84 to 25·86)	16·62 (15·97 to 17·25)	21·65 (19·14 to 24·38)	10·94 (10·39 to 11·53)	8·64 (8·11 to 9·18)	6·79 (6·39 to 7·20)	5·18 (4·46 to 5·99)
Bulgaria	32·12 (31·21 to 33·06)	27·39 (26·56 to 28·23)	24·27 (23·54 to 25·07)	18·85 (18·15 to 19·57)	18·37 (17·68 to 19·10)	18·25 (17·47 to 19·06)	16·49 (15·67 to 17·36)	12·90 (12·17 to 13·63)	11·27 (10·64 to 11·94)	8·34 (5·69 to 12·02)
Croatia	36·05 (25·64 to 50·11)	28·57 (21·29 to 36·91)	22·80 (18·57 to 27·63)	18·23 (17·40 to 19·12)	12·62 (12·07 to 13·20)	11·89 (10·83 to 13·01)	9·13 (8·64 to 9·63)	6·91 (6·52 to 7·34)	5·54 (5·20 to 5·90)	4·52 (3·83 to 5·31)
Czech Republic	24·00 (23·23 to 24·77)	21·86 (21·23 to 22·49)	18·52 (17·96 to 19·11)	14·35 (13·81 to 14·91)	11·87 (11·36 to 12·43)	9·07 (8·62 to 9·56)	5·37 (5·00 to 5·76)	4·28 (3·99 to 4·59)	3·49 (3·25 to 3·75)	2·82 (2·38 to 3·34)
Hungary	40·01 (39·13 to 40·95)	36·90 (36·11 to 37·70)	25·04 (24·39 to 25·75)	22·48 (21·77 to 23·23)	17·03 (16·42 to 17·67)	12·63 (12·11 to 13·18)	10·57 (10·08 to 11·13)	7·48 (7·07 to 7·94)	5·99 (5·60 to 6·39)	5·58 (4·49 to 6·94)
Macedonia	69·48 (39·03 to 112·77)	56·68 (32·00 to 91·81)	53·17 (37·35 to 74·46)	42·48 (30·56 to 57·26)	35·57 (33·99 to 37·17)	23·80 (22·48 to 25·12)	14·42 (13·44 to 15·45)	13·83 (12·82 to 14·97)	9·74 (8·90 to 10·71)	10·06 (6·39 to 15·28)
Montenegro	33·48 (21·88 to 50·01)	28·19 (18·89 to 43·15)	23·36 (15·62 to 34·41)	19·23 (12·85 to 27·39)	15·39 (11·41 to 20·18)	15·50 (13·80 to 17·30)	16·95 (15·41 to 18·54)	10·82 (9·87 to 11·83)	6·86 (6·12 to 7·67)	5·10 (3·90 to 6·56)
Poland	38·56 (38·05 to 39·08)	28·64 (28·24 to 29·07)	24·63 (24·26 to 25·00)	21·02 (20·70 to 21·35)	18·57 (18·24 to 18·91)	15·98 (15·64 to 16·34)	9·57 (9·28 to 9·87)	7·46 (7·21 to 7·72)	5·86 (5·65 to 6·09)	4·50 (3·04 to 6·54)
Romania	56·07 (55·40 to 56·79)	40·88 (40·30 to 41·46)	34·60 (34·06 to 35·09)	31·41 (30·88 to 31·94)	33·99 (33·45 to 34·58)	25·87 (25·28 to 26·47)	23·33 (22·72 to 23·94)	18·25 (17·74 to 18·81)	13·33 (12·89 to 13·80)	9·50 (8·15 to 11·02)
Serbia	43·95 (29·08 to 63·09)	36·05 (24·75 to 51·53)	31·17 (21·53 to 43·19)	27·05 (19·37 to 36·21)	20·24 (15·59 to 25·96)	21·46 (20·69 to 22·26)	15·13 (12·54 to 18·20)	12·35 (10·22 to 14·87)	10·24 (9·75 to 10·76)	7·89 (6·58 to 9·30)
Slovakia	30·64 (29·56 to 31·66)	27·81 (26·86 to 28·89)	23·73 (22·84 to 24·62)	18·57 (17·80 to 19·36)	14·20 (13·52 to 14·92)	12·49 (11·79 to 13·22)	9·91 (9·26 to 10·61)	8·65 (8·07 to 9·30)	6·58 (6·09 to 7·11)	6·05 (4·56 to 7·99)
Slovenia	28·81 (20·97 to 38·77)	22·66 (17·75 to 28·73)	18·19 (15·93 to 20·65)	15·13 (14·33 to 15·97)	10·18 (9·51 to 10·82)	7·15 (6·66 to 7·68)	5·43 (4·96 to 5·89)	4·37 (4·00 to 4·76)	3·22 (2·93 to 3·54)	2·37 (2·02 to 2·76)
Central Asia	105·82 (89·78 to 124·27)	88·50 (80·29 to 96·85)	77·25 (72·15 to 82·90)	68·91 (64·97 to 73·00)	59·10 (55·21 to 63·32)	58·99 (54·60 to 64·15)	50·13 (45·43 to 55·40)	40·74 (34·95 to 47·35)	31·68 (25·74 to 38·45)	24·50 (17·92 to 32·91)
Armenia	107·70 (78·74 to 143·76)	82·94 (65·10 to 103·83)	68·41 (57·55 to 80·79)	56·18 (48·85 to 64·43)	45·53 (39·22 to 52·33)	43·19 (37·26 to 50·00)	30·71 (26·14 to 36·37)	23·72 (18·99 to 29·25)	16·98 (13·21 to 21·34)	12·11 (8·94 to 16·07)
Azerbaijan	126·62 (91·87 to 167·36)	96·44 (71·18 to 127·73)	89·49 (72·01 to 110·14)	85·70 (71·37 to 99·66)	77·56 (65·00 to 90·73)	76·06 (64·67 to 89·09)	62·90 (53·18 to 74·78)	51·14 (41·87 to 61·62)	38·58 (27·79 to 51·05)	30·01 (21·36 to 41·08)
Georgia	101·68 (78·25 to 129·66)	77·22 (63·05 to 93·14)	58·61 (51·16 to 67·47)	47·94 (42·63 to 53·34)	42·66 (38·24 to 47·61)	42·75 (38·31 to 48·00)	37·90 (33·31 to 42·98)	27·73 (22·95 to 32·99)	20·66 (16·17 to 26·05)	15·42 (11·42 to 20·44)
Kazakhstan	61·55 (47·36 to 77·64)	54·90 (46·96 to 63·74)	48·57 (42·00 to 55·25)	41·82 (37·04 to 47·18)	37·20 (32·78 to 41·41)	40·51 (35·10 to 46·31)	36·92 (30·78 to 43·58)	29·31 (23·50 to 36·05)	20·24 (16·12 to 24·84)	13·49 (9·89 to 18·01)
Kyrgyzstan	135·91 (116·74 to 157·15)	111·86 (100·58 to 123·93)	94·84 (86·74 to 102·90)	81·09 (75·35 to 86·83)	66·18 (61·80 to 70·66)	56·35 (52·56 to 60·38)	49·83 (45·98 to 53·63)	41·89 (38·41 to 45·56)	35·45 (31·84 to 39·22)	28·94 (24·23 to 34·35)
Mongolia	151·94 (128·47 to 176·99)	136·39 (118·33 to 156·91)	124·66 (110·64 to 139·32)	109·91 (100·61 to 120·05)	91·89 (84·21 to 99·44)	75·12 (68·59 to 81·83)	57·58 (52·17 to 63·19)	41·03 (36·27 to 45·88)	29·48 (25·44 to 34·10)	21·21 (16·71 to 26·59)
Tajikistan	190·29 (145·73 to 241·74)	169·62 (140·46 to 202·71)	135·92 (116·35 to 154·60)	117·25 (101·35 to 134·13)	98·58 (85·50 to 111·90)	92·73 (80·63 to 105·07)	74·67 (64·40 to 86·46)	54·23 (45·29 to 64·32)	43·87 (35·42 to 54·16)	37·57 (27·10 to 50·82)
Turkmenistan	180·44 (126·11 to 244·80)	149·62 (108·17 to 198·49)	124·39 (99·50 to 155·34)	106·06 (89·14 to 124·44)	92·38 (77·33 to 108·07)	88·26 (73·89 to 105·97)	80·85 (64·36 to 100·76)	65·81 (48·61 to 84·48)	49·99 (35·67 to 67·31)	37·02 (25·16 to 52·60)
Uzbekistan	92·55 (69·88 to 122·24)	75·41 (61·90 to 90·08)	67·89 (57·81 to 78·53)	61·05 (52·79 to 70·47)	48·89 (42·28 to 56·09)	50·92 (44·47 to 58·71)	42·57 (35·76 to 50·65)	37·66 (28·15 to 49·18)	30·64 (22·26 to 41·06)	23·49 (16·75 to 32·12)
Latin America and Caribbean	118·46 (111·12 to 125·77)	99·30 (94·48 to 104·84)	79·87 (75·89 to 84·27)	65·34 (62·28 to 68·72)	51·95 (49·71 to 54·41)	41·83 (39·48 to 44·46)	33·07 (30·64 to 35·52)	27·70 (25·68 to 29·97)	25·73 (22·22 to 29·97)	18·69 (14·73 to 23·77)
Central Latin America	105·84 (101·86 to 109·85)	87·86 (84·32 to 91·63)	68·31 (65·00 to 71·66)	54·21 (51·47 to 56·93)	43·09 (40·69 to 45·53)	35·59 (33·37 to 38·00)	28·73 (26·42 to 30·96)	23·93 (21·75 to 26·11)	20·21 (17·82 to 22·77)	16·70 (13·96 to 20·06)
Colombia	94·12 (88·02 to 100·46)	75·29 (70·21 to 80·54)	53·87 (49·84 to 58·30)	43·96 (40·40 to 47·96)	33·92 (31·06 to 36·92)	30·65 (27·97 to 33·65)	26·65 (23·83 to 29·53)	23·78 (20·99 to 26·95)	19·97 (17·03 to 23·55)	15·48 (12·60 to 19·00)
Costa Rica	69·82 (65·44 to 74·48)	48·49 (44·57 to 52·38)	32·12 (28·88 to 35·58)	24·08 (21·33 to 27·07)	19·95 (17·34 to 22·82)	17·29 (14·67 to 20·36)	15·41 (12·88 to 18·56)	13·91 (11·55 to 16·72)	12·50 (10·19 to 15·13)	10·58 (8·15 to 13·67)
El Salvador	148·81 (132·14 to 165·68)	118·41 (106·45 to 130·71)	95·35 (86·64 to 105·13)	75·64 (68·64 to 82·96)	53·34 (48·31 to 59·45)	39·48 (34·97 to 44·06)	29·33 (25·72 to 33·62)	20·72 (17·54 to 24·32)	15·78 (12·93 to 18·95)	11·88 (9·53 to 14·79)
Guatemala	178·90 (168·48 to 190·20)	150·01 (141·93 to 157·71)	124·14 (118·10 to 130·56)	100·42 (95·49 to 105·48)	80·39 (76·22 to 84·82)	63·90 (59·96 to 67·61)	51·09 (47·42 to 54·86)	42·19 (38·43 to 46·42)	34·36 (30·55 to 38·88)	26·72 (22·88 to 31·24)
Honduras	134·49 (123·48 to 146·40)	111·78 (104·54 to 119·51)	87·13 (82·42 to 92·28)	68·90 (65·29 to 72·76)	55·01 (52·00 to 58·32)	45·06 (42·33 to 47·95)	36·77 (34·01 to 39·58)	30·67 (27·78 to 33·70)	26·29 (23·19 to 30·04)	21·38 (18·27 to 25·41)
Mexico	104·22 (98·06 to 110·65)	87·78 (82·18 to 93·62)	70·60 (65·34 to 76·02)	55·36 (50·78 to 59·90)	43·92 (39·72 to 47·93)	34·68 (31·18 to 38·40)	27·65 (24·21 to 31·12)	22·62 (19·37 to 26·34)	18·98 (15·59 to 22·74)	15·49 (12·14 to 19·69)
Nicaragua	172·45 (161·06 to 185·00)	137·32 (129·54 to 145·12)	106·05 (100·67 to 111·96)	90·90 (83·38 to 98·28)	64·14 (60·75 to 67·48)	50·51 (47·42 to 53·63)	36·11 (33·60 to 38·69)	26·73 (24·25 to 29·42)	20·54 (17·99 to 23·60)	15·06 (12·66 to 18·21)
Panama	59·37 (54·84 to 63·82)	45·40 (41·51 to 49·56)	37·11 (33·30 to 41·04)	30·83 (27·24 to 34·72)	27·88 (24·50 to 31·69)	25·51 (22·30 to 29·43)	23·15 (19·98 to 27·00)	21·26 (18·14 to 24·86)	19·10 (15·91 to 22·90)	16·61 (13·14 to 20·95)
Venezuela	68·84 (68·43 to 69·27)	60·66 (60·28 to 61·05)	41·69 (41·40 to 41·97)	32·84 (32·59 to 33·08)	28·74 (28·52 to 28·97)	27·82 (27·59 to 28·07)	21·43 (21·24 to 21·61)	18·29 (18·13 to 18·45)	16·96 (16·80 to 17·12)	16·83 (16·01 to 17·73)

Appendix Table 11A. Under-5 mortality rates by location, both sexes combined, 1970, 1975, 1980, 1985, 1990, 1995, 2000, 2005, 2010, 2016

Location	1970	1975	1980	1985	1990	1995	2000	2005	2010	2016
Andean Latin America	191·60 (179·05 to 204·99)	149·95 (144·14 to 156·08)	122·97 (116·36 to 129·74)	99·73 (95·65 to 103·94)	78·03 (75·10 to 81·02)	59·66 (57·47 to 61·92)	44·30 (42·29 to 46·38)	34·88 (32·70 to 37·14)	26·97 (23·98 to 30·22)	20·36 (16·25 to 25·40)
Bolivia	229·97 (215·71 to 244·20)	192·59 (182·32 to 202·94)	159·82 (151·36 to 168·57)	135·80 (129·02 to 142·54)	114·36 (108·29 to 120·76)	92·38 (86·61 to 98·30)	72·60 (66·67 to 78·73)	58·32 (51·62 to 65·37)	45·76 (38·30 to 54·09)	33·79 (26·33 to 43·16)
Ecuador	127·01 (120·30 to 133·79)	101·69 (96·86 to 106·89)	77·37 (73·79 to 81·47)	60·24 (57·09 to 63·46)	48·18 (45·65 to 51·01)	39·45 (36·94 to 41·97)	32·82 (30·14 to 35·70)	27·07 (24·28 to 30·14)	21·70 (18·87 to 24·78)	17·27 (14·50 to 20·71)
Peru	205·93 (184·36 to 230·31)	155·86 (146·52 to 165·24)	131·05 (122·84 to 139·61)	105·75 (99·84 to 111·46)	79·32 (74·68 to 84·05)	57·21 (53·84 to 60·87)	38·18 (35·78 to 40·65)	28·60 (26·67 to 30·54)	21·85 (19·82 to 23·85)	16·61 (13·23 to 20·81)
Caribbean	112·24 (104·71 to 120·52)	100·57 (94·20 to 107·37)	93·28 (85·86 to 101·58)	80·29 (74·48 to 87·42)	69·05 (63·05 to 75·07)	60·45 (55·64 to 66·63)	50·06 (45·13 to 55·58)	48·16 (42·86 to 53·86)	74·52 (47·84 to 109·03)	33·48 (23·62 to 46·84)
Antigua and Barbuda	33·44 (24·74 to 43·44)	27·37 (19·78 to 36·94)	20·95 (14·82 to 28·52)	14·76 (10·54 to 20·16)	14·62 (10·49 to 19·62)	16·84 (12·41 to 22·10)	16·39 (12·23 to 21·96)	14·45 (10·80 to 19·55)	12·46 (9·06 to 17·66)	11·16 (7·40 to 16·47)
The Bahamas	48·70 (32·48 to 69·73)	40·01 (27·02 to 56·40)	33·16 (21·76 to 48·93)	27·66 (17·26 to 41·12)	22·52 (13·64 to 34·58)	17·90 (11·78 to 26·33)	14·01 (9·16 to 19·93)	14·14 (9·67 to 19·54)	12·30 (8·17 to 18·25)	10·98 (5·86 to 20·34)
Barbados	46·01 (31·93 to 64·10)	33·91 (23·05 to 48·03)	25·43 (16·70 to 37·72)	21·37 (13·31 to 32·50)	18·77 (11·22 to 29·28)	17·79 (10·68 to 28·10)	17·81 (10·64 to 27·61)	16·90 (11·21 to 23·84)	15·50 (10·35 to 22·44)	14·73 (7·91 to 25·69)
Belize	98·87 (77·98 to 122·60)	77·05 (63·08 to 93·40)	62·27 (50·57 to 74·12)	47·39 (39·13 to 56·30)	39·60 (32·86 to 47·52)	29·72 (23·50 to 37·33)	28·06 (21·84 to 35·28)	22·14 (16·40 to 30·07)	17·52 (12·40 to 24·05)	15·06 (8·79 to 24·64)
Bermuda	27·47 (22·85 to 32·76)	22·45 (18·17 to 27·37)	21·19 (16·95 to 25·97)	14·35 (11·10 to 18·52)	12·13 (9·42 to 15·53)	9·49 (7·15 to 12·69)	4·37 (2·95 to 6·43)	6·20 (4·56 to 8·26)	4·76 (3·38 to 6·51)	3·89 (2·53 to 5·82)
Cuba	40·60 (40·29 to 40·94)	30·12 (29·82 to 30·41)	23·87 (23·42 to 24·37)	18·97 (18·37 to 19·65)	13·98 (13·61 to 14·39)	11·28 (11·07 to 11·48)	8·66 (8·50 to 8·83)	7·18 (7·03 to 7·34)	6·08 (5·92 to 6·23)	5·31 (5·01 to 5·67)
Dominica	56·76 (41·66 to 74·51)	34·93 (24·92 to 46·96)	22·50 (15·61 to 30·24)	18·31 (12·52 to 25·27)	17·01 (11·84 to 23·62)	17·53 (12·43 to 23·61)	18·66 (13·36 to 25·23)	20·35 (15·01 to 27·72)	23·67 (16·94 to 33·83)	24·95 (15·65 to 38·47)
Dominican Republic	127·06 (114·83 to 139·62)	107·00 (96·56 to 117·58)	86·85 (78·95 to 95·43)	72·52 (66·22 to 79·54)	60·39 (54·93 to 66·10)	46·72 (42·33 to 51·55)	37·43 (33·76 to 41·53)	36·93 (33·00 to 41·20)	32·68 (28·50 to 37·50)	27·42 (21·69 to 34·46)
Grenada	53·61 (32·88 to 81·07)	45·33 (26·14 to 70·22)	35·46 (18·03 to 63·58)	26·72 (13·33 to 49·41)	19·76 (11·15 to 33·59)	16·23 (9·89 to 24·19)	15·48 (9·88 to 22·44)	14·33 (8·98 to 21·61)	14·55 (9·59 to 21·45)	15·42 (8·42 to 27·94)
Guyana	89·36 (82·84 to 96·46)	77·96 (70·80 to 85·19)	64·84 (58·60 to 71·45)	57·76 (52·83 to 62·98)	52·17 (48·30 to 56·11)	44·97 (41·45 to 48·45)	39·18 (35·98 to 42·80)	37·31 (33·86 to 41·05)	33·01 (28·72 to 37·78)	24·87 (19·75 to 31·25)
Haiti	268·20 (240·78 to 298·47)	230·20 (208·57 to 253·05)	202·38 (179·17 to 227·06)	172·84 (155·03 to 193·97)	148·85 (132·14 to 166·33)	126·52 (112·11 to 143·62)	100·03 (86·08 to 114·66)	88·78 (75·52 to 103·17)	158·04 (89·63 to 244·22)	55·41 (37·42 to 79·25)
Jamaica	58·07 (48·96 to 67·89)	48·17 (40·26 to 58·48)	40·87 (31·44 to 51·46)	34·74 (26·54 to 44·19)	30·87 (22·99 to 40·39)	27·18 (19·60 to 36·73)	24·75 (17·53 to 33·45)	22·15 (14·57 to 33·68)	21·86 (13·64 to 34·52)	20·06 (12·21 to 31·69)
Puerto Rico	30·15 (29·57 to 30·71)	22·61 (22·13 to 23·07)	20·05 (19·60 to 20·52)	17·61 (16·91 to 18·54)	15·79 (15·36 to 16·21)	15·07 (14·67 to 15·50)	12·54 (12·13 to 12·93)	10·53 (10·14 to 10·92)	8·89 (8·53 to 9·27)	6·59 (5·80 to 7·51)
Saint Lucia	62·20 (35·88 to 103·91)	43·25 (27·55 to 65·03)	30·29 (18·66 to 46·14)	24·31 (13·75 to 39·25)	21·00 (12·00 to 35·09)	19·05 (11·87 to 29·90)	18·07 (11·40 to 26·98)	19·91 (12·44 to 30·03)	20·14 (13·29 to 29·80)	17·47 (9·11 to 31·17)
Saint Vincent and the Grenadines	85·91 (57·61 to 119·29)	63·14 (41·16 to 89·38)	44·62 (29·16 to 62·96)	31·69 (20·27 to 46·58)	26·62 (17·45 to 38·85)	24·33 (16·42 to 33·80)	23·28 (16·52 to 31·52)	21·62 (15·55 to 29·19)	19·39 (13·66 to 27·21)	16·63 (10·39 to 26·83)
Suriname	69·12 (57·58 to 83·80)	64·42 (54·71 to 75·31)	59·03 (50·73 to 68·14)	53·97 (46·93 to 61·55)	50·05 (43·66 to 57·45)	47·42 (40·99 to 54·56)	45·41 (38·14 to 53·29)	42·32 (34·49 to 51·21)	38·92 (30·88 to 47·95)	34·76 (26·83 to 44·78)
Trinidad and Tobago	52·99 (48·37 to 57·82)	47·78 (43·18 to 52·87)	37·92 (33·39 to 42·85)	30·44 (25·91 to 35·35)	28·07 (23·20 to 33·49)	27·30 (22·30 to 32·95)	27·48 (22·30 to 33·83)	24·39 (18·89 to 31·64)	22·17 (15·29 to 30·68)	20·95 (12·26 to 34·16)
Virgin Islands, U.S.	31·63 (28·90 to 34·33)	28·16 (25·64 to 30·85)	25·17 (23·04 to 27·41)	20·88 (19·28 to 22·55)	19·09 (17·53 to 20·79)	17·17 (15·64 to 18·82)	14·00 (12·67 to 15·50)	12·10 (10·72 to 13·62)	10·82 (9·26 to 12·50)	9·10 (7·39 to 11·19)
Tropical Latin America	115·52 (99·26 to 131·74)	98·63 (87·12 to 111·51)	78·37 (69·56 to 88·19)	65·66 (59·39 to 73·11)	51·67 (46·48 to 56·92)	40·50 (35·90 to 45·86)	31·53 (26·86 to 36·52)	25·73 (21·35 to 30·88)	20·93 (16·54 to 26·80)	17·08 (12·93 to 22·42)
Brazil	114·65 (98·03 to 131·18)	98·43 (86·87 to 111·27)	78·75 (69·78 to 88·68)	65·51 (58·98 to 73·00)	52·18 (46·80 to 57·68)	41·07 (36·30 to 46·65)	31·70 (26·85 to 36·96)	25·62 (21·12 to 31·14)	20·82 (16·47 to 26·66)	16·91 (12·82 to 22·07)
Paraguay	70·46 (63·28 to 78·42)	62·06 (56·38 to 68·16)	52·88 (47·84 to 57·99)	43·96 (40·26 to 48·28)	33·44 (30·33 to 36·65)	28·92 (25·94 to 32·12)	28·86 (25·22 to 32·64)	26·67 (22·63 to 30·92)	23·03 (17·98 to 28·90)	18·86 (14·35 to 24·65)
Southeast Asia, East Asia, and Oceania	123·48 (113·34 to 134·02)	101·57 (94·32 to 108·99)	83·98 (78·38 to 90·19)	69·97 (65·06 to 75·31)	58·45 (54·16 to 63·11)	49·95 (46·25 to 53·99)	41·28 (38·56 to 44·22)	31·02 (29·06 to 33·00)	23·59 (21·76 to 25·59)	17·46 (15·34 to 19·89)
East Asia	116·28 (103·90 to 129·67)	92·98 (83·35 to 102·93)	76·54 (68·26 to 85·49)	63·34 (56·58 to 70·86)	52·80 (47·15 to 59·44)	45·74 (40·45 to 51·53)	36·94 (33·05 to 41·23)	24·14 (21·71 to 26·60)	17·08 (15·38 to 18·81)	12·37 (10·64 to 14·62)
China	118·77 (106·13 to 132·38)	95·21 (85·08 to 105·50)	78·62 (69·98 to 87·93)	64·97 (57·85 to 72·80)	54·13 (48·29 to 60·96)	44·57 (39·69 to 49·83)	34·94 (31·41 to 38·87)	23·94 (21·50 to 26·54)	16·61 (14·93 to 18·37)	11·78 (10·24 to 13·74)
North Korea	57·96 (45·18 to 72·87)	41·71 (32·14 to 51·71)	33·10 (25·47 to 42·00)	30·43 (23·77 to 38·18)	27·98 (21·44 to 35·73)	91·84 (38·48 to 166·69)	87·70 (42·23 to 146·41)	34·02 (26·80 to 43·02)	30·91 (24·26 to 39·22)	26·20 (20·37 to 34·30)
Taiwan (Province of China)	27·68 (27·23 to 28·13)	19·73 (19·34 to 20·14)	15·72 (15·37 to 16·07)	11·08 (10·81 to 11·37)	8·74 (8·48 to 9·00)	8·94 (8·67 to 9·22)	8·64 (8·38 to 8·89)	6·92 (6·69 to 7·18)	5·43 (5·21 to 5·66)	4·71 (4·18 to 5·38)
Southeast Asia	144·22 (137·73 to 150·97)	122·92 (116·71 to 129·88)	99·42 (95·47 to 103·62)	83·32 (79·86 to 86·79)	69·50 (66·25 to 72·90)	56·67 (54·06 to 59·49)	46·75 (44·30 to 49·49)	38·62 (36·09 to 41·37)	30·46 (27·82 to 33·34)	22·28 (19·25 to 25·78)
Cambodia	196·47 (167·10 to 227·90)	283·35 (192·84 to 382·31)	158·44 (142·98 to 174·25)	138·47 (129·26 to 148·43)	119·31 (112·15 to 127·10)	111·91 (104·82 to 118·74)	94·78 (88·06 to 101·52)	66·93 (60·76 to 73·65)	46·15 (40·62 to 51·97)	31·88 (26·10 to 38·62)
Indonesia	177·53 (170·08 to 185·04)	149·72 (142·87 to 156·58)	125·69 (120·80 to 131·57)	105·43 (100·79 to 110·25)	86·67 (83·18 to 90·52)	68·19 (64·97 to 71·54)	54·09 (50·70 to 57·56)	43·59 (40·29 to 47·08)	34·60 (30·41 to 39·03)	25·24 (20·54 to 30·96)
Laos	295·17 (208·34 to 402·64)	263·49 (196·74 to 338·87)	236·84 (189·95 to 285·55)	204·55 (178·05 to 232·77)	185·47 (164·64 to 207·28)	164·95 (147·00 to 185·71)	140·88 (125·25 to 159·15)	112·51 (97·74 to 128·39)	82·56 (68·76 to 98·06)	58·21 (40·87 to 80·42)
Malaysia	56·26 (55·58 to 56·99)	43·40 (41·58 to 45·13)	31·25 (29·25 to 33·34)	22·55 (22·17 to 22·90)	16·84 (16·53 to 17·20)	13·19 (12·95 to 13·44)	9·34 (9·12 to 9·55)	8·06 (7·87 to 8·25)	7·93 (7·58 to 8·30)	6·00 (5·22 to 6·91)
Maldives	398·86 (379·83 to 420·65)	327·93 (319·85 to 335·53)	275·94 (270·40 to 281·19)	187·94 (183·90 to 191·94)	110·00 (107·09 to 112·95)	60·10 (58·08 to 62·03)	31·30 (29·90 to 32·72)	18·11 (17·13 to 19·08)	11·74 (10·94 to 12·61)	7·49 (6·63 to 8·58)
Mauritius	87·36 (85·74 to 89·00)	61·64 (60·35 to 62·99)	40·11 (39·20 to 41·13)	29·66 (28·80 to 30·54)	22·78 (22·06 to 23·49)	20·33 (19·67 to 21·01)	18·05 (17·37 to 18·70)	16·42 (15·77 to 17·11)	15·71 (15·02 to 16·51)	13·92 (12·46 to 15·59)
Myanmar	205·88 (165·32 to 246·59)	163·03 (133·07 to 197·12)	135·12 (109·96 to 161·75)	117·63 (96·74 to 140·38)	105·83 (88·59 to 125·56)	95·67 (79·38 to 116·42)	83·36 (67·80 to 100·92)	65·97 (53·31 to 81·22)	44·52 (34·75 to 56·43)	27·70 (20·83 to 37·50)
Philippines	101·10 (95·71 to 107·04)	90·25 (85·50 to 95·21)	80·69 (76·24 to 85·25)	69·33 (65·39 to 73·38)	56·22 (53·19 to 59·42)	45·36 (42·50 to 48·23)	38·73 (35·86 to 41·52)	34·54 (31·42 to 37·92)	30·27 (26·82 to 34·06)	24·12 (20·37 to 28·57)
Sri Lanka	71·07 (70·27 to 71·80)	69·70 (68·90 to 70·51)	44·77 (44·18 to 45·37)	32·60 (31·97 to 33·19)	32·73 (31·55 to 34·02)	20·86 (20·39 to 21·33)	16·92 (16·40 to 17·43)	17·56 (17·19 to 17·93)	13·39 (12·39 to 14·45)	6·79 (5·08 to 9·01)
Seychelles	71·04 (67·38 to 75·07)	49·29 (46·43 to 52·38)	31·43 (29·08 to 33·77)	23·09 (21·10 to 25·35)	18·71 (16·82 to 20·69)	14·47 (12·89 to 16·17)	13·38 (11·93 to 15·01)	13·31 (11·82 to 14·88)	12·98 (11·46 to 14·76)	11·38 (9·31 to 13·90)
Thailand	89·21 (84·30 to 94·16)	68·96 (64·27 to 73·96)	52·14 (47·84 to 57·01)	39·28 (35·11 to 43·71)	28·99 (24·95 to 33·57)	20·73 (17·54 to 24·54)	15·62 (13·14 to 18·71)	11·91 (10·05 to 14·00)	9·02 (7·47 to 10·76)	6·41 (5·11 to 8·02)
Timor-Leste	305·77 (247·19 to 372·87)	276·95 (237·12 to 323·00)	256·26 (222·09 to 291·48)	185·84 (164·77 to 208·19)	152·41 (134·60 to 171·39)	119·52 (103·34 to 137·18)	99·81 (83·61 to 117·48)	69·97 (54·81 to 86·68)	48·96 (33·85 to 68·09)	35·26 (21·10 to 55·41)
Vietnam	112·44 (92·09 to 136·88)	84·71 (76·39 to 93·90)	68·83 (63·80 to 74·06)	57·04 (53·30 to 60·99)	46·60 (43·66 to 49·63)	36·37 (33·91 to 38·78)	27·74 (25·47 to 30·01)	21·94 (19·67 to 24·37)	17·62 (15·27 to 20·38)	13·15 (10·93 to 16·10)
Oceania	108·62 (87·90 to 131·25)	96·35 (80·19 to 114·82)	86·56 (73·01 to 101·41)	80·22 (67·20 to 94·51)	74·94 (61·18 to 90·33)	68·65 (53·66 to 86·50)	63·74 (46·83 to 86·50)	61·64 (40·30 to 89·03)	55·61 (33·84 to 86·26)	44·95 (25·98 to 73·77)
American Samoa	34·45 (31·33 to 37·67)	26·72 (23·68 to 30·05)	20·69 (18·16 to 23·49)	17·12 (15·12 to 19·39)	16·47 (14·64 to 18·27)	15·18 (13·50 to 16·98)	13·89 (12·49 to 15·38)	12·49 (11·17 to 13·95)	10·50 (9·09 to 12·21)	8·50 (6·72 to 10·73)
Federated States of Micronesia	85·91 (61·46 to 114·03)	73·29 (51·02 to 99·01)	62·69 (46·50 to 84·48)	53·79 (41·95 to 68·66)	45·69 (34·29 to 59·25)	37·99 (27·16 to 50·82)	35·19 (23·13 to 52·43)	22·77 (15·10 to 33·25)	20·40 (13·31 to 29·71)	17·32 (11·21 to 26·05)
Fiji	51·88 (45·77 to 58·17)	49·12 (39·71 to 60·07)	38·40 (29·29 to 49·98)	30·76 (22·19 to 40·76)	27·28 (18·47 to 39·93)	28·61 (20·51 to 38·63)	30·69 (24·25 to 38·38)	34·65 (27·01 to 43·30)	37·30 (27·52 to 49·45)	37·81 (22·36 to 60·99)
Guam	33·01 (30·40 to 35·72)	25·63 (23·51 to 28·01)	17·14 (15·42 to 18·92)	13·63 (12·12 to 15·30)	12·61 (11·33 to 13·99)	12·08 (10·88 to 13·39)	11·29 (10·16 to 12·41)	12·49 (11·37 to 13·76)	13·97 (12·43 to 15·65)	13·66 (10·21 to 18·14)

Appendix Table 11A. Under-5 mortality rates by location, both sexes combined, 1970, 1975, 1980, 1985, 1990, 1995, 2000, 2005, 2010, 2016

Location	1970	1975	1980	1985	1990	1995	2000	2005	2010	2016
Kiribati	134·93 (101·16 to 173·20)	106·82 (70·97 to 153·82)	92·96 (56·21 to 150·17)	91·08 (57·48 to 137·73)	82·15 (60·10 to 106·21)	71·97 (57·12 to 89·43)	65·45 (51·53 to 82·09)	62·77 (46·08 to 82·80)	58·58 (36·58 to 88·67)	48·37 (25·30 to 85·40)
Marshall Islands	79·14 (43·21 to 130·56)	64·81 (34·81 to 110·86)	53·53 (31·22 to 88·26)	50·36 (33·33 to 74·15)	42·66 (30·99 to 59·32)	35·38 (24·51 to 49·33)	40·28 (24·50 to 60·38)	33·01 (21·18 to 48·41)	27·41 (15·46 to 43·65)	22·15 (11·84 to 37·95)
Northern Mariana Islands	18·95 (10·38 to 32·15)	14·28 (7·51 to 26·20)	11·05 (6·09 to 19·32)	8·72 (4·48 to 14·80)	6·84 (3·59 to 11·43)	5·84 (3·23 to 9·23)	4·53 (3·38 to 5·90)	3·45 (2·55 to 4·53)	2·15 (1·51 to 3·05)	2·46 (1·35 to 4·12)
Papua New Guinea	126·64 (99·58 to 155·41)	112·37 (90·27 to 136·05)	104·73 (86·73 to 124·82)	98·46 (81·12 to 117·78)	91·02 (73·30 to 111·43)	81·70 (62·74 to 103·98)	74·06 (52·28 to 101·76)	70·72 (43·94 to 105·17)	63·09 (36·61 to 99·55)	49·91 (28·27 to 83·00)
Samoa	58·32 (45·42 to 72·72)	46·07 (32·94 to 62·74)	31·68 (20·08 to 49·58)	26·99 (15·05 to 46·02)	22·46 (11·67 to 40·88)	18·66 (9·19 to 35·43)	15·82 (7·60 to 28·91)	14·22 (6·83 to 26·04)	12·61 (5·99 to 23·17)	10·39 (4·84 to 20·16)
Solomon Islands	116·69 (88·63 to 146·46)	93·35 (67·31 to 125·52)	61·21 (43·30 to 87·50)	49·84 (34·66 to 68·09)	44·75 (32·46 to 59·73)	38·81 (28·53 to 51·05)	35·93 (26·37 to 47·74)	35·83 (25·17 to 48·72)	31·75 (21·77 to 45·02)	25·50 (16·74 to 37·87)
Tonga	39·77 (28·29 to 53·94)	36·15 (25·25 to 49·60)	33·76 (22·24 to 48·50)	28·75 (17·82 to 43·72)	25·52 (15·15 to 39·44)	24·68 (14·54 to 38·74)	23·63 (13·89 to 38·14)	22·32 (13·21 to 36·30)	21·44 (12·27 to 36·28)	18·58 (9·77 to 32·90)
Vanuatu	97·14 (67·47 to 137·44)	75·41 (49·57 to 109·32)	57·34 (37·83 to 82·80)	46·74 (33·43 to 62·69)	41·55 (31·97 to 52·64)	38·38 (29·73 to 48·80)	39·64 (30·68 to 50·54)	41·41 (31·31 to 53·70)	36·89 (25·86 to 50·54)	31·56 (20·71 to 48·59)
North Africa and Middle East	204·06 (192·14 to 215·63)	160·59 (153·01 to 168·49)	126·60 (120·36 to 132·80)	100·52 (94·77 to 106·92)	74·57 (71·24 to 78·30)	61·94 (59·04 to 64·82)	50·73 (47·91 to 53·67)	41·53 (38·17 to 44·95)	33·85 (30·27 to 37·53)	27·90 (23·44 to 33·63)
North Africa and Middle East	204·06 (192·14 to 215·63)	160·59 (153·01 to 168·49)	126·60 (120·36 to 132·80)	100·52 (94·77 to 106·92)	74·57 (71·24 to 78·30)	61·94 (59·04 to 64·82)	50·73 (47·91 to 53·67)	41·53 (38·17 to 44·95)	33·85 (30·27 to 37·53)	27·90 (23·44 to 33·63)
Afghanistan	241·97 (188·11 to 300·76)	230·76 (187·25 to 278·45)	224·01 (186·28 to 263·21)	204·89 (173·75 to 238·24)	154·79 (139·39 to 170·71)	148·08 (134·95 to 161·79)	135·86 (122·90 to 149·04)	112·02 (98·97 to 125·66)	86·90 (73·51 to 103·42)	66·61 (52·93 to 85·68)
Algeria	175·97 (155·06 to 196·38)	141·52 (126·30 to 157·69)	104·22 (94·31 to 114·66)	69·36 (63·28 to 75·81)	49·31 (44·56 to 54·30)	40·51 (36·38 to 45·09)	33·13 (29·42 to 36·85)	27·61 (24·34 to 31·20)	23·63 (20·28 to 27·27)	18·21 (14·15 to 23·86)
Bahrain	88·87 (69·44 to 110·71)	53·84 (43·95 to 65·78)	28·82 (26·48 to 31·44)	21·34 (19·84 to 22·91)	21·70 (20·25 to 23·25)	17·76 (16·35 to 19·27)	12·29 (11·27 to 13·39)	12·78 (11·60 to 13·94)	8·18 (7·43 to 9·00)	6·86 (5·35 to 8·68)
Egypt	244·98 (226·19 to 263·82)	206·86 (189·54 to 223·24)	159·77 (144·51 to 175·00)	114·71 (104·58 to 125·74)	83·38 (75·66 to 91·71)	61·74 (55·36 to 68·65)	44·05 (38·90 to 49·80)	35·11 (30·15 to 40·24)	28·03 (23·79 to 32·92)	19·34 (13·25 to 27·75)
Iran	246·73 (203·63 to 292·00)	142·38 (119·25 to 168·20)	107·03 (90·60 to 123·09)	83·79 (70·89 to 98·22)	67·38 (57·66 to 78·97)	50·48 (42·13 to 59·63)	40·79 (32·95 to 50·10)	29·47 (22·08 to 38·69)	22·00 (16·06 to 29·67)	17·80 (12·59 to 24·53)
Iraq	124·52 (105·68 to 143·29)	102·94 (92·24 to 113·88)	81·74 (73·12 to 93·66)	72·19 (62·62 to 87·55)	52·29 (48·91 to 55·66)	50·83 (47·60 to 54·22)	42·18 (39·34 to 45·32)	40·67 (36·23 to 45·52)	33·41 (28·84 to 38·59)	28·47 (22·51 to 36·53)
Jordan	103·25 (92·75 to 113·90)	81·80 (75·23 to 88·38)	57·73 (53·99 to 61·93)	41·66 (38·98 to 44·33)	34·21 (32·13 to 36·36)	30·29 (28·42 to 32·27)	26·90 (25·02 to 28·87)	23·31 (21·39 to 25·27)	19·46 (17·19 to 21·91)	17·16 (14·25 to 21·30)
Kuwait	48·98 (46·82 to 51·11)	44·99 (43·28 to 46·66)	31·77 (30·30 to 33·26)	21·00 (20·00 to 22·11)	15·17 (13·14 to 17·38)	12·72 (11·90 to 13·54)	12·78 (11·92 to 13·67)	11·29 (10·56 to 12·12)	10·78 (10·08 to 11·50)	9·45 (7·21 to 12·19)
Lebanon	66·26 (53·26 to 82·77)	66·72 (51·20 to 84·57)	70·80 (46·87 to 102·85)	43·53 (33·57 to 55·88)	34·22 (28·32 to 41·21)	24·88 (20·19 to 30·33)	18·36 (14·14 to 23·26)	13·74 (9·83 to 18·57)	11·19 (8·24 to 14·84)	9·46 (6·98 to 12·52)
Libya	117·88 (104·52 to 132·60)	86·67 (78·01 to 96·45)	62·33 (55·61 to 69·20)	48·11 (42·99 to 53·84)	40·18 (35·58 to 45·08)	32·19 (27·70 to 37·09)	26·69 (21·88 to 32·33)	21·32 (16·32 to 27·02)	15·71 (11·57 to 20·89)	13·88 (9·74 to 19·71)
Morocco	188·43 (179·86 to 197·15)	157·93 (151·19 to 165·48)	122·82 (116·95 to 128·82)	93·12 (88·07 to 98·33)	70·78 (66·06 to 75·35)	56·14 (51·66 to 60·93)	45·53 (40·00 to 51·46)	36·97 (30·51 to 44·06)	29·33 (23·00 to 36·86)	21·72 (16·40 to 28·57)
Palestine	81·48 (60·09 to 108·03)	73·54 (59·53 to 89·62)	61·18 (53·04 to 71·61)	43·77 (38·14 to 50·82)	40·26 (35·01 to 45·52)	29·57 (25·40 to 34·31)	27·09 (23·34 to 31·80)	25·15 (21·57 to 29·46)	21·63 (18·22 to 25·46)	16·72 (11·95 to 22·81)
Oman	274·00 (241·28 to 309·70)	201·54 (174·99 to 227·79)	133·65 (113·87 to 154·24)	79·66 (66·11 to 95·09)	48·12 (38·75 to 59·45)	28·28 (22·90 to 34·90)	15·80 (13·52 to 18·44)	7·72 (7·35 to 8·09)	8·56 (8·21 to 8·92)	8·04 (7·03 to 9·15)
Qatar	73·19 (50·53 to 99·87)	63·07 (47·70 to 82·03)	35·12 (24·97 to 47·24)	26·99 (20·10 to 35·10)	21·45 (15·30 to 29·47)	18·81 (13·42 to 25·76)	16·49 (11·68 to 22·45)	13·59 (9·65 to 18·83)	11·42 (8·06 to 15·52)	9·42 (6·17 to 13·84)
Saudi Arabia	148·78 (117·81 to 184·52)	108·69 (89·08 to 132·70)	80·47 (66·22 to 96·45)	59·87 (48·39 to 73·30)	46·77 (36·47 to 59·25)	33·88 (25·33 to 45·16)	23·47 (16·99 to 32·08)	16·47 (11·51 to 22·83)	11·32 (7·86 to 16·35)	6·56 (4·43 to 9·84)
Sudan	196·64 (181·20 to 215·18)	189·71 (174·01 to 205·92)	176·95 (160·36 to 194·59)	231·15 (176·96 to 288·77)	135·93 (123·64 to 149·26)	115·02 (103·55 to 127·01)	94·37 (85·17 to 104·52)	78·01 (70·07 to 87·11)	75·02 (65·52 to 85·39)	61·56 (48·27 to 79·77)
Syria	104·73 (97·45 to 112·03)	81·13 (75·18 to 86·99)	62·73 (57·56 to 68·30)	50·96 (46·30 to 56·09)	42·63 (38·74 to 46·73)	28·86 (26·84 to 30·96)	18·11 (17·82 to 18·39)	15·51 (15·33 to 15·69)	15·27 (15·00 to 15·55)	27·05 (13·67 to 46·95)
Tunisia	159·48 (151·32 to 167·82)	117·93 (111·69 to 124·33)	86·01 (80·51 to 91·20)	63·09 (58·89 to 67·78)	47·89 (44·44 to 51·71)	35·95 (32·75 to 39·28)	26·60 (23·75 to 29·55)	20·67 (17·84 to 23·67)	16·79 (13·99 to 19·97)	12·54 (9·99 to 15·73)
Turkey	192·26 (175·44 to 208·82)	160·98 (146·95 to 177·73)	124·62 (111·95 to 138·65)	92·37 (82·16 to 103·17)	70·61 (63·00 to 79·00)	55·55 (49·01 to 63·12)	40·19 (34·51 to 46·70)	28·95 (23·91 to 34·99)	22·00 (16·50 to 29·23)	15·70 (10·98 to 22·06)
United Arab Emirates	112·70 (77·87 to 155·38)	80·72 (53·03 to 120·58)	53·18 (33·95 to 77·26)	32·42 (18·24 to 53·07)	22·29 (11·93 to 36·61)	15·50 (8·02 to 27·39)	10·58 (5·67 to 17·86)	6·46 (5·10 to 8·15)	6·63 (3·81 to 10·64)	5·75 (2·99 to 10·29)
Yemen	316·70 (300·83 to 332·74)	249·88 (237·23 to 262·04)	193·23 (182·81 to 203·63)	154·62 (146·09 to 163·91)	129·54 (121·92 to 137·49)	108·71 (102·02 to 115·71)	88·43 (82·18 to 94·98)	68·68 (62·96 to 75·44)	52·07 (46·11 to 59·24)	43·61 (36·07 to 53·84)
South Asia	210·83 (198·61 to 224·35)	183·59 (173·92 to 193·22)	158·74 (151·64 to 165·65)	136·16 (130·69 to 141·76)	115·65 (110·51 to 120·85)	99·33 (94·55 to 104·27)	84·81 (80·62 to 89·41)	71·72 (66·95 to 76·77)	57·44 (52·30 to 63·26)	40·65 (35·89 to 46·62)
South Asia	210·83 (198·61 to 224·35)	183·59 (173·92 to 193·22)	158·74 (151·64 to 165·65)	136·16 (130·69 to 141·76)	115·65 (110·51 to 120·85)	99·33 (94·55 to 104·27)	84·81 (80·62 to 89·41)	71·72 (66·95 to 76·77)	57·44 (52·30 to 63·26)	40·65 (35·89 to 46·62)
Bangladesh	240·57 (226·62 to 254·54)	211·46 (202·36 to 220·73)	192·25 (184·53 to 200·34)	166·73 (160·34 to 173·91)	136·81 (131·54 to 142·28)	108·98 (104·16 to 113·53)	86·07 (81·73 to 90·51)	67·62 (63·61 to 71·64)	50·85 (46·96 to 55·07)	33·97 (30·27 to 38·20)
Bhutan	261·29 (225·41 to 300·19)	231·87 (202·77 to 263·15)	205·60 (182·87 to 231·44)	170·45 (152·30 to 189·33)	134·33 (121·16 to 147·95)	107·12 (96·45 to 118·04)	85·36 (75·99 to 96·05)	64·96 (56·86 to 74·56)	48·32 (41·40 to 56·47)	32·91 (27·61 to 39·82)
India	209·46 (195·20 to 225·77)	181·43 (171·26 to 192·03)	154·88 (147·14 to 162·60)	132·08 (125·52 to 138·69)	112·32 (106·84 to 117·85)	96·87 (91·78 to 102·15)	82·95 (78·02 to 88·06)	70·30 (64·63 to 76·31)	55·76 (49·09 to 63·35)	39·17 (33·02 to 47·30)
Nepal	246·60 (235·95 to 258·26)	225·69 (215·04 to 235·93)	201·08 (191·83 to 210·66)	169·88 (161·80 to 178·15)	136·57 (130·23 to 142·96)	105·56 (100·15 to 111·21)	81·04 (75·98 to 86·15)	62·67 (57·84 to 67·56)	45·99 (41·74 to 50·59)	29·35 (25·78 to 33·49)
Pakistan	175·90 (168·09 to 183·84)	158·50 (151·27 to 165·76)	142·88 (136·56 to 149·18)	126·52 (120·91 to 131·96)	111·49 (106·58 to 116·15)	99·87 (95·31 to 104·65)	90·75 (86·00 to 95·70)	84·46 (78·02 to 90·84)	71·01 (64·03 to 78·26)	53·45 (46·87 to 61·07)
Sub-Saharan Africa	231·07 (215·99 to 246·54)	214·47 (204·48 to 224·64)	196·99 (190·13 to 204·38)	183·19 (177·78 to 188·62)	171·82 (167·08 to 176·62)	161·21 (156·67 to 165·79)	146·32 (142·13 to 150·55)	124·62 (120·28 to 129·23)	102·62 (96·69 to 109·11)	76·72 (68·94 to 85·61)
Southern Sub-Saharan Africa	126·42 (111·64 to 143·02)	113·44 (105·78 to 121·36)	103·15 (97·44 to 109·13)	88·68 (84·32 to 93·32)	74·21 (70·61 to 77·72)	69·72 (65·52 to 74·30)	75·74 (70·48 to 81·61)	77·97 (71·04 to 85·06)	65·66 (59·34 to 71·73)	46·45 (39·18 to 55·52)
Botswana	100·29 (87·80 to 113·40)	81·05 (72·50 to 90·09)	68·27 (61·37 to 75·42)	58·04 (52·21 to 64·02)	49·58 (44·33 to 54·86)	57·86 (51·54 to 64·77)	63·55 (55·91 to 72·35)	47·12 (39·67 to 55·23)	26·78 (21·27 to 33·51)	14·92 (11·10 to 20·42)
Lesotho	115·69 (107·71 to 123·93)	108·45 (100·08 to 117·74)	99·05 (90·92 to 107·55)	92·63 (84·62 to 100·66)	84·97 (78·43 to 91·67)	85·04 (78·33 to 92·04)	99·58 (91·47 to 108·08)	110·52 (100·18 to 121·98)	89·36 (76·69 to 102·68)	66·60 (53·22 to 83·03)
Namibia	103·15 (83·36 to 128·11)	97·80 (83·74 to 112·88)	97·32 (85·12 to 109·84)	82·26 (73·07 to 93·10)	71·76 (64·47 to 80·44)	59·68 (52·48 to 67·36)	63·79 (55·45 to 73·42)	65·63 (56·52 to 76·56)	55·20 (44·92 to 66·10)	36·49 (23·99 to 53·27)

Appendix Table 11A. Under-5 mortality rates by location, both sexes combined, 1970, 1975, 1980, 1985, 1990, 1995, 2000, 2005, 2010, 2016

Location	1970	1975	1980	1985	1990	1995	2000	2005	2010	2016
South Africa	133·37 (116·33 to 152·92)	119·96 (110·37 to 130·11)	109·18 (101·11 to 117·26)	95·52 (89·45 to 101·72)	78·57 (73·17 to 83·77)	69·83 (63·73 to 76·61)	75·65 (69·05 to 83·07)	76·84 (67·24 to 86·88)	62·03 (54·04 to 69·48)	43·40 (33·17 to 55·91)
Swaziland	188·24 (138·63 to 250·28)	149·65 (119·70 to 183·54)	118·41 (103·74 to 135·89)	90·96 (82·22 to 100·27)	67·67 (61·68 to 73·57)	70·14 (64·84 to 76·02)	93·69 (86·44 to 101·15)	104·53 (96·04 to 113·60)	74·24 (65·71 to 83·63)	49·23 (39·22 to 61·08)
Zimbabwe	107·99 (97·11 to 119·39)	97·87 (90·06 to 105·94)	90·19 (83·98 to 96·61)	74·09 (69·76 to 78·75)	66·53 (62·92 to 70·43)	72·62 (68·50 to 77·56)	77·38 (72·45 to 82·32)	82·75 (77·51 to 88·36)	78·02 (70·86 to 85·79)	56·51 (47·29 to 68·59)
Western Sub-Saharan Africa	265·53 (249·62 to 281·50)	243·75 (230·68 to 257·83)	221·95 (211·42 to 232·59)	205·64 (197·13 to 213·89)	194·59 (188·03 to 201·38)	184·08 (178·20 to 190·35)	169·14 (163·44 to 175·16)	148·47 (141·83 to 155·06)	124·94 (116·84 to 133·73)	97·35 (87·82 to 108·66)
Benin	279·55 (265·60 to 293·03)	248·35 (236·20 to 259·86)	221·41 (210·59 to 232·28)	197·94 (187·47 to 208·49)	180·22 (170·79 to 189·87)	161·37 (152·05 to 171·05)	139·47 (130·00 to 148·83)	115·60 (105·85 to 127·12)	94·94 (84·18 to 107·84)	72·17 (60·47 to 87·45)
Burkina Faso	307·66 (284·57 to 332·78)	285·09 (268·05 to 302·84)	256·30 (243·73 to 269·12)	232·18 (221·10 to 243·40)	211·06 (201·58 to 220·86)	197·51 (187·69 to 206·51)	178·14 (168·49 to 188·14)	155·50 (144·24 to 167·04)	134·26 (121·53 to 148·92)	108·80 (94·70 to 126·67)
Cameroon	206·23 (195·08 to 217·99)	185·42 (175·14 to 195·10)	166·86 (157·71 to 175·93)	146·49 (137·65 to 155·34)	136·35 (127·87 to 145·05)	139·78 (131·04 to 148·69)	136·24 (126·49 to 147·85)	124·91 (112·32 to 138·66)	109·31 (93·60 to 127·48)	88·08 (70·15 to 110·07)
Cape Verde	182·33 (177·24 to 187·49)	136·17 (131·39 to 141·13)	91·86 (88·05 to 95·86)	90·66 (87·15 to 94·52)	64·68 (61·98 to 67·68)	71·85 (57·81 to 87·03)	45·03 (39·12 to 51·44)	33·07 (28·83 to 37·74)	23·16 (21·24 to 25·33)	17·05 (14·91 to 19·46)
Chad	257·31 (231·52 to 285·50)	238·44 (219·82 to 258·41)	228·38 (213·45 to 243·71)	218·68 (205·95 to 231·96)	197·58 (186·35 to 209·04)	185·97 (174·52 to 197·26)	182·74 (170·72 to 194·91)	164·03 (150·28 to 177·86)	140·28 (125·47 to 156·96)	113·05 (97·92 to 132·28)
Cote d'Ivoire	220·74 (210·67 to 230·67)	188·76 (180·18 to 197·19)	161·79 (154·61 to 169·67)	152·36 (144·76 to 160·35)	148·78 (141·18 to 156·70)	147·66 (139·04 to 155·99)	136·95 (128·20 to 146·49)	125·17 (114·01 to 137·02)	108·17 (94·78 to 121·90)	85·34 (72·54 to 100·36)
The Gambia	187·69 (162·95 to 214·29)	166·79 (147·12 to 186·92)	146·16 (131·69 to 161·77)	127·01 (115·88 to 138·61)	112·95 (103·57 to 122·19)	98·99 (91·17 to 106·92)	85·97 (78·95 to 93·17)	72·72 (65·82 to 80·16)	60·36 (53·19 to 67·95)	48·67 (41·52 to 57·93)
Ghana	174·49 (165·31 to 183·77)	158·73 (151·19 to 167·03)	148·98 (141·91 to 156·50)	140·17 (133·78 to 146·75)	123·12 (117·75 to 128·73)	110·59 (105·16 to 116·07)	101·11 (95·96 to 106·85)	90·07 (84·63 to 95·86)	74·41 (67·39 to 81·54)	52·30 (43·97 to 63·19)
Guinea	311·24 (272·56 to 350·55)	298·30 (272·47 to 326·52)	275·07 (256·09 to 294·85)	252·96 (237·42 to 269·12)	226·09 (213·53 to 239·37)	201·10 (188·04 to 214·47)	172·77 (160·14 to 186·01)	149·77 (135·84 to 163·97)	126·21 (110·98 to 143·72)	97·49 (80·65 to 119·63)
Guinea-Bissau	307·81 (268·91 to 351·87)	275·45 (244·23 to 310·20)	251·89 (226·89 to 277·55)	235·52 (216·36 to 254·97)	209·66 (195·21 to 226·12)	183·21 (169·88 to 196·35)	157·85 (145·88 to 169·98)	137·85 (124·88 to 150·96)	101·87 (91·32 to 112·74)	71·82 (62·02 to 82·69)
Liberia	293·14 (267·18 to 319·34)	264·94 (243·96 to 286·57)	242·88 (226·40 to 260·24)	228·70 (215·34 to 242·21)	218·41 (207·09 to 230·24)	214·18 (203·19 to 226·21)	171·50 (162·63 to 180·42)	122·12 (114·19 to 129·67)	93·56 (85·18 to 102·71)	65·29 (56·34 to 76·73)
Mali	354·22 (331·75 to 377·76)	326·58 (309·28 to 344·51)	288·93 (274·50 to 303·47)	262·66 (250·23 to 274·93)	236·32 (224·85 to 249·02)	214·82 (203·24 to 227·37)	192·36 (181·47 to 205·50)	166·02 (153·95 to 178·71)	140·79 (126·42 to 157·24)	117·10 (97·65 to 142·37)
Mauritania	171·84 (163·59 to 180·98)	161·14 (153·25 to 169·14)	142·18 (135·23 to 149·53)	124·81 (117·66 to 131·89)	110·27 (103·72 to 117·21)	100·60 (93·69 to 107·45)	92·50 (85·59 to 100·04)	83·52 (75·71 to 91·27)	66·76 (58·99 to 74·86)	50·33 (43·14 to 58·79)
Niger	329·61 (303·37 to 358·29)	331·08 (310·53 to 352·83)	320·10 (303·38 to 337·46)	310·13 (294·36 to 325·96)	293·37 (278·98 to 308·46)	267·10 (252·03 to 282·17)	224·81 (210·29 to 239·75)	178·70 (163·04 to 194·91)	140·74 (124·02 to 160·04)	108·22 (90·93 to 130·61)
Nigeria	267·13 (240·49 to 294·42)	244·57 (224·39 to 265·94)	223·51 (208·96 to 238·22)	209·50 (199·09 to 220·60)	206·73 (196·67 to 216·92)	197·42 (187·80 to 207·59)	183·78 (174·44 to 193·34)	163·88 (153·52 to 173·95)	139·22 (127·67 to 151·56)	108·69 (93·04 to 126·20)
Sao Tome and Principe	148·48 (142·07 to 154·76)	117·84 (110·33 to 125·38)	112·82 (106·37 to 119·82)	123·68 (118·30 to 129·57)	105·66 (98·07 to 113·08)	91·28 (83·97 to 99·21)	73·85 (66·92 to 80·65)	60·29 (52·64 to 68·65)	41·78 (36·63 to 47·66)	30·39 (25·59 to 36·11)
Senegal	269·32 (257·97 to 280·92)	241·93 (232·50 to 251·83)	203·98 (195·66 to 212·24)	172·04 (165·04 to 179·27)	145·25 (139·48 to 150·96)	132·69 (127·07 to 138·05)	114·93 (109·55 to 120·58)	91·99 (86·97 to 97·00)	68·94 (63·94 to 74·37)	49·40 (43·93 to 56·26)
Sierra Leone	304·18 (268·02 to 346·76)	284·59 (254·53 to 316·83)	273·67 (250·92 to 296·26)	261·40 (244·80 to 279·08)	244·50 (231·07 to 259·24)	230·38 (217·56 to 242·51)	215·15 (202·89 to 226·59)	186·71 (174·20 to 198·90)	154·13 (140·44 to 167·53)	115·58 (101·48 to 131·17)
Togo	220·16 (202·31 to 238·53)	194·77 (181·23 to 208·13)	171·83 (160·90 to 183·25)	157·82 (147·94 to 168·11)	145·11 (136·24 to 154·60)	134·60 (125·57 to 143·59)	122·43 (113·73 to 131·27)	109·58 (100·02 to 119·09)	92·37 (82·84 to 103·43)	68·99 (59·71 to 80·81)
Eastern Sub-Saharan Africa	219·05 (205·28 to 231·74)	207·38 (196·64 to 217·55)	192·58 (184·33 to 200·76)	183·06 (176·38 to 189·57)	168·46 (162·93 to 173·83)	154·33 (149·32 to 159·36)	133·31 (128·20 to 138·52)	106·25 (101·14 to 111·79)	83·43 (77·12 to 90·11)	59·57 (51·61 to 69·70)
Burundi	240·15 (207·16 to 271·39)	233·23 (207·48 to 262·10)	213·13 (189·44 to 236·38)	184·31 (165·88 to 203·08)	176·69 (159·31 to 195·67)	174·14 (156·98 to 192·41)	164·29 (145·58 to 182·81)	126·72 (111·65 to 143·58)	97·15 (80·27 to 115·06)	80·79 (56·50 to 116·66)
Comoros	200·94 (165·98 to 237·29)	182·89 (160·23 to 205·77)	164·86 (146·64 to 183·28)	139·78 (125·49 to 155·83)	119·75 (107·33 to 131·99)	105·59 (94·89 to 117·27)	97·73 (86·50 to 110·18)	80·19 (69·91 to 91·97)	68·33 (57·05 to 80·84)	52·60 (39·85 to 70·56)
Djibouti	138·12 (116·78 to 161·51)	128·24 (112·09 to 145·56)	122·12 (109·33 to 135·61)	120·78 (109·96 to 132·25)	115·85 (106·22 to 125·61)	111·37 (101·41 to 121·80)	102·40 (91·38 to 113·64)	84·73 (73·16 to 97·93)	68·95 (57·83 to 83·11)	48·03 (39·19 to 59·77)
Eritrea	225·88 (197·02 to 257·24)	203·69 (183·28 to 224·45)	186·71 (169·66 to 202·60)	169·96 (156·44 to 185·45)	148·71 (135·80 to 161·87)	114·93 (103·85 to 127·08)	92·83 (80·84 to 105·52)	77·82 (65·10 to 91·87)	67·60 (54·70 to 82·83)	51·19 (40·51 to 65·79)
Ethiopia	235·76 (214·67 to 257·86)	235·36 (218·56 to 252·64)	220·04 (206·41 to 233·18)	203·01 (192·04 to 214·93)	188·38 (178·49 to 198·69)	164·96 (156·21 to 174·05)	140·09 (131·61 to 148·90)	106·14 (97·49 to 114·74)	74·33 (66·19 to 83·93)	43·37 (36·98 to 51·66)
Kenya	143·02 (135·31 to 151·22)	126·24 (119·21 to 133·90)	110·18 (103·08 to 117·37)	100·46 (94·84 to 107·17)	95·42 (90·12 to 101·34)	97·74 (91·73 to 103·65)	90·26 (84·24 to 96·63)	74·12 (68·33 to 80·87)	56·63 (50·55 to 62·98)	43·44 (36·88 to 51·17)
Madagascar	174·21 (153·37 to 197·76)	166·89 (148·58 to 187·91)	165·86 (149·22 to 182·45)	170·98 (153·74 to 188·20)	149·28 (136·43 to 162·55)	135·48 (122·82 to 148·72)	117·27 (104·80 to 129·19)	101·39 (88·78 to 113·98)	96·37 (82·07 to 110·99)	79·73 (63·38 to 101·85)
Malawi	329·90 (298·65 to 364·77)	293·79 (267·30 to 320·48)	257·61 (238·20 to 278·36)	240·83 (224·03 to 259·24)	223·37 (207·69 to 239·51)	193·77 (179·96 to 209·19)	165·15 (151·73 to 178·82)	123·92 (112·76 to 134·50)	100·30 (89·16 to 112·49)	68·99 (55·33 to 87·40)
Mozambique	254·02 (224·00 to 283·15)	237·68 (216·47 to 258·31)	228·83 (214·63 to 243·43)	254·76 (237·15 to 271·60)	213·40 (203·75 to 223·29)	193·64 (184·86 to 202·56)	161·49 (153·40 to 170·19)	129·92 (121·26 to 138·36)	103·72 (93·77 to 114·26)	76·15 (65·91 to 89·23)
Rwanda	247·89 (226·93 to 269·12)	242·85 (223·77 to 263·08)	210·75 (194·43 to 227·73)	172·85 (159·33 to 187·55)	168·96 (156·47 to 182·09)	185·05 (168·32 to 202·25)	156·24 (142·40 to 170·22)	105·94 (95·19 to 116·38)	66·73 (58·31 to 75·58)	49·22 (39·57 to 62·28)
Somalia	197·01 (163·70 to 233·74)	226·06 (189·56 to 265·45)	189·13 (164·66 to 215·59)	194·29 (174·02 to 215·19)	178·33 (163·13 to 194·56)	168·54 (154·11 to 184·17)	155·57 (141·21 to 170·50)	133·05 (117·00 to 149·88)	146·29 (123·35 to 173·44)	97·20 (81·27 to 117·78)
South Sudan	256·10 (195·18 to 332·17)	244·37 (192·56 to 306·39)	233·45 (192·98 to 276·63)	221·89 (194·36 to 251·54)	200·08 (180·77 to 221·76)	170·82 (154·88 to 186·21)	143·59 (131·25 to 155·96)	123·62 (110·72 to 137·01)	107·62 (91·54 to 125·11)	93·50 (73·44 to 117·42)
Tanzania	209·89 (194·41 to 224·63)	191·33 (181·10 to 201·72)	176·29 (168·39 to 185·19)	165·79 (158·83 to 172·74)	154·88 (148·76 to 161·48)	143·43 (137·01 to 149·82)	123·81 (117·83 to 130·04)	100·03 (93·84 to 106·57)	76·95 (69·94 to 84·23)	55·72 (48·23 to 65·29)
Uganda	199·96 (184·24 to 215·35)	191·00 (179·56 to 202·70)	192·22 (181·62 to 202·68)	182·85 (174·40 to 191·81)	170·31 (163·26 to 177·21)	156·74 (150·35 to 163·59)	134·51 (128·33 to 140·70)	112·53 (106·40 to 118·70)	90·24 (83·18 to 97·53)	62·43 (55·00 to 71·80)

Appendix Table 11A. Under-5 mortality rates by location, both sexes combined, 1970, 1975, 1980, 1985, 1990, 1995, 2000, 2005, 2010, 2016

Location	1970	1975	1980	1985	1990	1995	2000	2005	2010	2016
Zambia	184·85 (163·58 to 208·94)	163·93 (147·19 to 180·88)	161·38 (146·64 to 177·87)	167·49 (153·73 to 182·53)	169·97 (156·76 to 183·69)	161·06 (147·58 to 174·90)	142·33 (128·60 to 155·19)	113·28 (101·44 to 125·31)	84·20 (72·60 to 97·04)	59·75 (47·74 to 73·91)
Central Sub-Saharan Africa	210·50 (169·61 to 254·65)	197·44 (164·18 to 232·97)	183·57 (161·29 to 207·56)	165·13 (149·96 to 182·35)	166·75 (153·31 to 180·56)	160·49 (146·89 to 175·44)	150·29 (135·55 to 166·22)	126·84 (114·58 to 139·63)	105·05 (90·53 to 119·79)	74·90 (56·49 to 97·42)
Angola	205·00 (157·46 to 260·68)	197·68 (154·66 to 243·56)	192·70 (157·45 to 230·47)	185·04 (160·26 to 210·72)	188·17 (166·20 to 212·70)	163·99 (144·24 to 184·46)	151·22 (132·34 to 172·10)	121·98 (106·93 to 139·82)	92·93 (77·84 to 111·23)	54·45 (40·40 to 71·78)
Central African Republic	187·66 (155·59 to 222·99)	189·69 (159·53 to 222·22)	179·65 (154·26 to 210·81)	176·11 (151·84 to 202·81)	177·19 (153·84 to 203·75)	176·48 (150·43 to 205·99)	179·30 (152·17 to 210·10)	170·18 (140·83 to 203·84)	155·44 (123·68 to 191·23)	130·55 (97·21 to 176·90)
Congo	174·64 (133·79 to 224·15)	151·01 (114·00 to 194·69)	126·30 (105·08 to 149·85)	101·95 (89·64 to 114·27)	95·44 (86·38 to 106·08)	99·54 (88·88 to 111·46)	103·42 (91·96 to 115·72)	87·26 (77·27 to 97·94)	68·13 (56·79 to 81·87)	52·85 (37·78 to 74·94)
Democratic Republic of the Congo	215·95 (165·97 to 270·08)	201·41 (162·02 to 247·20)	185·55 (155·01 to 217·25)	162·46 (142·38 to 185·01)	164·11 (144·81 to 184·05)	163·12 (143·25 to 185·65)	152·43 (131·94 to 176·34)	129·44 (112·15 to 149·05)	109·46 (90·77 to 130·40)	80·48 (58·50 to 107·73)
Equatorial Guinea	238·70 (178·67 to 309·35)	217·01 (173·65 to 264·27)	188·57 (155·96 to 222·76)	182·15 (154·49 to 211·71)	194·29 (166·46 to 226·91)	203·30 (172·30 to 238·15)	146·81 (118·81 to 177·06)	112·19 (85·44 to 143·94)	87·48 (62·63 to 120·07)	57·03 (38·65 to 84·95)
Gabon	171·16 (143·60 to 201·79)	140·12 (121·59 to 159·66)	113·82 (102·04 to 126·49)	95·65 (87·24 to 104·07)	85·07 (77·89 to 92·57)	75·38 (69·03 to 81·97)	69·88 (63·50 to 76·46)	65·43 (58·20 to 73·06)	55·85 (47·90 to 65·50)	43·66 (35·51 to 54·61)

Appendix Table 11B. Under-5 mortality rates by location, males, 1970, 1975, 1980, 1985, 1990, 1995, 2000, 2005, 2010, 2016

Location	1970	1975	1980	1985	1990	1995	2000	2005	2010	2016
Global	149·38 (140·47 to 158·96)	131·24 (124·73 to 137·95)	114·77 (109·59 to 120·15)	100·48 (96·16 to 104·87)	87·93 (84·19 to 91·64)	80·18 (76·93 to 83·47)	71·71 (68·56 to 75·09)	61·94 (58·53 to 65·43)	51·80 (47·59 to 56·35)	40·37 (35·58 to 46·09)
High SDI	92·53 (86·31 to 99·65)	73·35 (69·41 to 77·49)	60·13 (56·43 to 64·01)	48·24 (45·69 to 51·00)	40·31 (38·14 to 42·74)	33·94 (31·92 to 35·94)	26·90 (25·00 to 28·82)	20·64 (18·90 to 22·54)	15·87 (14·22 to 17·67)	12·46 (10·66 to 14·61)
High-middle SDI	28·87 (28·16 to 29·67)	22·81 (22·14 to 23·54)	17·37 (16·84 to 17·99)	13·67 (13·33 to 14·06)	11·40 (11·18 to 11·67)	9·06 (8·87 to 9·26)	7·58 (7·49 to 7·67)	6·85 (6·76 to 6·93)	5·94 (5·84 to 6·03)	5·39 (4·98 to 5·87)
Middle SDI	213·50 (199·23 to 229·17)	186·97 (175·96 to 198·41)	162·68 (154·75 to 171·06)	142·13 (135·64 to 148·28)	122·76 (117·61 to 128·34)	109·58 (104·75 to 114·53)	95·95 (91·37 to 100·64)	82·11 (77·69 to 86·55)	67·85 (62·37 to 73·72)	51·39 (45·66 to 58·36)
Low-middle SDI	247·89 (225·71 to 271·67)	231·91 (217·70 to 246·41)	213·81 (203·92 to 224·17)	197·45 (189·05 to 206·20)	180·60 (173·09 to 188·44)	166·38 (159·34 to 173·49)	146·65 (139·92 to 153·73)	122·27 (115·29 to 129·67)	101·00 (91·96 to 111·12)	74·42 (63·50 to 87·25)
Low SDI	139·56 (130·59 to 149·06)	115·31 (108·62 to 121·89)	94·54 (89·58 to 100·09)	77·10 (72·70 to 81·58)	62·62 (58·84 to 66·69)	51·68 (48·91 to 54·54)	42·56 (40·37 to 45·07)	34·61 (32·30 to 36·83)	27·24 (24·88 to 29·85)	20·77 (17·79 to 24·22)
High-income	32·51 (31·81 to 33·26)	26·01 (25·37 to 26·74)	18·89 (18·38 to 19·51)	14·64 (14·30 to 15·02)	12·44 (12·22 to 12·71)	9·85 (9·68 to 10·05)	8·31 (8·23 to 8·40)	7·44 (7·35 to 7·52)	6·50 (6·40 to 6·59)	5·91 (5·50 to 6·36)
High-income North America	26·71 (26·44 to 26·98)	21·27 (21·08 to 21·47)	16·79 (16·63 to 16·97)	13·91 (13·76 to 14·05)	12·10 (11·97 to 12·23)	9·92 (9·81 to 10·02)	8·89 (8·77 to 9·00)	8·69 (8·58 to 8·80)	7·77 (7·65 to 7·87)	7·34 (7·05 to 7·64)
Canada	25·52 (24·95 to 26·09)	19·21 (18·76 to 19·66)	13·98 (13·63 to 14·35)	10·69 (10·41 to 10·96)	9·19 (8·93 to 9·46)	7·97 (7·73 to 8·19)	6·73 (6·47 to 6·99)	6·59 (6·38 to 6·81)	6·15 (5·95 to 6·35)	5·77 (5·41 to 6·16)
Greenland	52·43 (48·13 to 56·70)	44·63 (40·64 to 48·59)	43·78 (39·55 to 48·32)	41·91 (37·76 to 46·58)	42·23 (37·25 to 47·28)	29·46 (26·08 to 33·25)	21·54 (18·92 to 24·38)	19·61 (17·14 to 22·79)	17·19 (14·62 to 19·99)	14·84 (11·64 to 18·80)
United States	26·93 (26·56 to 27·30)	21·41 (21·12 to 21·72)	16·98 (16·72 to 17·23)	14·21 (13·92 to 14·46)	12·30 (12·10 to 12·50)	10·01 (9·84 to 10·17)	9·02 (8·87 to 9·17)	8·86 (8·71 to 9·01)	7·89 (7·74 to 8·04)	7·47 (7·17 to 7·77)
Australasia	24·08 (23·64 to 24·50)	20·10 (19·71 to 20·50)	15·27 (14·92 to 15·61)	13·44 (13·17 to 13·75)	10·86 (10·58 to 11·13)	8·19 (7·96 to 8·42)	7·30 (7·11 to 7·50)	6·63 (6·43 to 6·84)	5·68 (5·50 to 5·87)	4·55 (4·15 to 5·01)
Australia	24·18 (23·66 to 24·66)	19·78 (19·33 to 20·24)	14·70 (14·31 to 15·08)	13·01 (12·69 to 13·34)	10·53 (10·21 to 10·83)	7·85 (7·61 to 8·10)	7·05 (6·83 to 7·29)	6·47 (6·25 to 6·69)	5·40 (5·22 to 5·59)	4·26 (3·88 to 4·68)
New Zealand	23·66 (22·92 to 24·40)	21·47 (20·82 to 22·08)	17·78 (17·18 to 18·36)	15·48 (14·94 to 16·05)	12·32 (11·87 to 12·75)	9·68 (9·32 to 10·07)	8·41 (7·99 to 8·84)	7·36 (7·02 to 7·74)	6·99 (6·69 to 7·29)	6·10 (5·56 to 6·71)
High-income Asia Pacific	32·92 (30·10 to 36·34)	23·51 (20·87 to 26·50)	16·52 (14·21 to 19·35)	11·51 (9·97 to 13·30)	9·00 (7·89 to 10·53)	7·31 (6·57 to 8·21)	5·76 (5·65 to 5·88)	4·61 (4·49 to 4·73)	3·71 (3·60 to 3·82)	3·13 (2·83 to 3·47)
Brunei	74·94 (70·80 to 79·24)	47·41 (44·48 to 50·40)	26·33 (24·65 to 28·11)	16·92 (15·79 to 18·03)	12·86 (11·98 to 13·83)	10·85 (10·07 to 11·76)	9·82 (9·04 to 10·63)	9·71 (8·97 to 10·44)	9·63 (8·83 to 10·50)	10·25 (8·78 to 11·95)
Japan	19·53 (19·25 to 19·82)	15·34 (15·10 to 15·59)	11·28 (11·08 to 11·48)	8·18 (8·02 to 8·35)	6·93 (6·78 to 7·09)	6·46 (6·25 to 6·64)	4·88 (4·76 to 5·01)	4·04 (3·94 to 4·15)	3·37 (3·27 to 3·49)	2·80 (2·50 to 3·14)
Singapore	30·58 (29·67 to 31·56)	21·18 (20·39 to 21·99)	16·67 (16·03 to 17·31)	11·94 (11·41 to 12·52)	8·37 (7·97 to 8·75)	5·65 (5·35 to 5·97)	4·25 (4·02 to 4·51)	3·37 (3·15 to 3·63)	3·02 (2·81 to 3·25)	2·55 (2·27 to 2·86)
South Korea	60·04 (51·57 to 70·21)	41·34 (33·14 to 50·41)	26·35 (19·87 to 34·45)	18·49 (13·76 to 23·96)	12·65 (9·49 to 17·13)	8·78 (6·80 to 11·23)	7·49 (7·27 to 7·72)	5·91 (5·70 to 6·14)	4·36 (4·17 to 4·55)	3·74 (2·94 to 4·69)
Western Europe	29·24 (28·98 to 29·52)	23·18 (22·96 to 23·41)	16·68 (16·54 to 16·84)	12·65 (12·51 to 12·80)	10·44 (10·32 to 10·57)	7·66 (7·55 to 7·77)	6·31 (6·21 to 6·41)	5·31 (5·23 to 5·40)	4·66 (4·58 to 4·75)	4·05 (3·54 to 4·64)
Andorra	25·04 (18·95 to 32·77)	14·80 (11·15 to 19·26)	8·59 (6·50 to 11·25)	7·59 (5·82 to 9·69)	6·69 (5·11 to 8·65)	4·73 (3·58 to 6·06)	3·42 (2·61 to 4·37)	3·56 (2·73 to 4·64)	3·40 (2·61 to 4·38)	3·54 (2·66 to 4·75)
Austria	33·37 (32·27 to 34·44)	27·21 (26·23 to 28·22)	19·13 (18·37 to 19·93)	14·99 (14·27 to 15·70)	10·89 (10·39 to 11·46)	7·58 (7·19 to 8·03)	6·40 (6·00 to 6·78)	5·49 (5·12 to 5·89)	5·08 (4·75 to 5·43)	3·77 (3·08 to 4·59)
Belgium	27·81 (27·38 to 28·27)	21·89 (21·48 to 22·30)	16·83 (16·51 to 17·17)	13·41 (13·12 to 13·71)	11·40 (11·10 to 11·68)	8·68 (8·44 to 8·90)	6·61 (6·39 to 6·83)	5·57 (5·40 to 5·76)	5·06 (4·90 to 5·25)	4·44 (4·04 to 4·89)
Cyprus	45·39 (40·24 to 50·63)	34·90 (32·58 to 36·88)	23·33 (22·12 to 24·51)	17·19 (15·99 to 18·31)	14·30 (13·00 to 15·50)	11·45 (10·58 to 12·27)	8·72 (7·93 to 9·48)	6·67 (6·06 to 7·32)	4·81 (4·29 to 5·36)	3·63 (3·05 to 4·24)
Denmark	19·97 (19·19 to 20·76)	14·66 (14·01 to 15·31)	11·80 (11·20 to 12·41)	10·81 (10·21 to 11·43)	10·18 (9·62 to 10·77)	7·39 (6·88 to 7·89)	6·16 (5·75 to 6·58)	5·41 (5·00 to 5·81)	4·39 (4·02 to 4·78)	4·36 (3·55 to 5·32)
Finland	18·63 (17·74 to 19·65)	14·40 (13·57 to 15·29)	10·02 (9·37 to 10·71)	8·24 (7·67 to 8·81)	7·62 (7·08 to 8·23)	5·55 (5·12 to 6·01)	4·72 (4·31 to 5·16)	4·39 (3·99 to 4·83)	3·32 (3·01 to 3·67)	2·41 (1·92 to 3·02)
France	20·71 (20·35 to 21·06)	18·62 (18·30 to 18·94)	14·14 (13·85 to 14·45)	11·34 (11·10 to 11·59)	10·08 (9·85 to 10·32)	6·86 (6·68 to 7·06)	6·02 (5·84 to 6·21)	4·88 (4·72 to 5·04)	4·62 (4·45 to 4·78)	4·21 (3·46 to 5·12)
Germany	29·13 (28·71 to 29·53)	24·49 (24·07 to 24·92)	17·16 (16·85 to 17·50)	12·36 (12·10 to 12·64)	10·24 (10·00 to 10·49)	7·24 (6·99 to 7·52)	5·92 (5·75 to 6·10)	5·20 (5·04 to 5·38)	4·62 (4·46 to 4·77)	3·94 (3·19 to 4·94)
Greece	36·22 (35·49 to 37·01)	28·88 (28·21 to 29·51)	22·07 (21·51 to 22·64)	16·83 (16·37 to 17·31)	11·66 (11·29 to 12·05)	9·35 (9·03 to 9·69)	6·84 (6·57 to 7·10)	4·87 (4·66 to 5·07)	4·32 (4·14 to 4·52)	4·18 (3·78 to 4·63)
Iceland	18·22 (16·80 to 19·82)	15·19 (13·79 to 16·80)	11·02 (9·99 to 12·11)	8·83 (7·93 to 9·80)	7·30 (6·59 to 8·13)	6·47 (5·75 to 7·26)	4·45 (3·93 to 5·05)	3·50 (3·08 to 4·01)	2·87 (2·47 to 3·31)	2·41 (1·97 to 2·93)
Ireland	25·00 (24·05 to 25·98)	22·48 (21·61 to 23·36)	15·64 (14·94 to 16·35)	11·88 (11·21 to 12·52)	10·37 (9·85 to 10·92)	8·14 (7·66 to 8·63)	7·97 (7·51 to 8·46)	5·41 (5·05 to 5·77)	4·55 (4·27 to 4·83)	4·25 (3·59 to 5·03)
Israel	31·55 (29·57 to 33·81)	30·76 (29·85 to 31·74)	18·36 (17·69 to 19·04)	14·73 (14·13 to 15·31)	12·77 (12·27 to 13·26)	9·57 (9·17 to 9·98)	7·59 (7·27 to 7·92)	5·98 (5·72 to 6·24)	4·92 (4·69 to 5·15)	3·82 (3·33 to 4·39)
Italy	35·86 (35·24 to 36·46)	25·77 (25·33 to 26·21)	18·16 (17·76 to 18·57)	13·11 (12·79 to 13·42)	10·17 (9·90 to 10·47)	7·95 (7·70 to 8·22)	5·71 (5·46 to 5·98)	4·61 (4·44 to 4·80)	4·27 (4·12 to 4·44)	3·48 (2·68 to 4·48)
Luxembourg	27·82 (25·60 to 30·28)	19·77 (18·05 to 21·62)	15·60 (13·97 to 17·27)	12·95 (11·74 to 14·14)	10·25 (9·19 to 11·30)	6·63 (5·88 to 7·40)	5·19 (4·51 to 5·94)	3·76 (3·30 to 4·29)	3·17 (2·70 to 3·65)	2·32 (1·82 to 2·91)
Malta	31·30 (29·23 to 33·72)	23·25 (21·10 to 25·35)	18·75 (17·41 to 20·04)	15·77 (14·52 to 17·10)	11·90 (10·89 to 12·84)	10·81 (9·83 to 11·77)	8·65 (7·79 to 9·53)	8·00 (7·16 to 8·99)	7·85 (6·91 to 8·93)	6·67 (5·46 to 8·11)
Netherlands	18·31 (17·81 to 18·83)	15·49 (15·03 to 15·99)	12·21 (11·78 to 12·62)	11·01 (10·61 to 11·42)	9·74 (9·37 to 10·09)	7·82 (7·53 to 8·17)	7·00 (6·71 to 7·32)	6·12 (5·85 to 6·40)	4·86 (4·63 to 5·08)	4·18 (3·73 to 4·68)
Norway	19·10 (18·35 to 19·92)	15·70 (14·97 to 16·40)	11·72 (11·08 to 12·43)	11·72 (11·11 to 12·37)	9·78 (9·23 to 10·36)	6·25 (5·89 to 6·63)	5·55 (5·17 to 5·89)	4·50 (4·18 to 4·83)	3·63 (3·37 to 3·89)	3·02 (2·54 to 3·60)
Portugal	69·65 (68·52 to 70·82)	47·61 (46·65 to 48·54)	32·24 (31·61 to 32·94)	22·99 (22·56 to 23·39)	15·79 (15·48 to 16·08)	10·85 (10·60 to 11·12)	7·77 (7·53 to 8·02)	5·48 (5·29 to 5·70)	4·14 (3·99 to 4·29)	3·44 (3·16 to 3·74)
Spain	35·11 (34·43 to 35·78)	24·32 (23·84 to 24·79)	16·96 (16·58 to 17·32)	12·39 (12·05 to 12·74)	10·37 (10·05 to 10·70)	7·59 (7·33 to 7·84)	5·73 (5·50 to 5·97)	5·02 (4·83 to 5·22)	4·10 (3·94 to 4·28)	3·50 (2·95 to 4·14)
Sweden	15·06 (14·36 to 15·76)	11·74 (11·19 to 12·38)	9·03 (8·56 to 9·57)	7·88 (7·41 to 8·38)	7·71 (7·29 to 8·17)	5·27 (4·95 to 5·60)	4·39 (4·06 to 4·73)	3·72 (3·46 to 3·99)	3·47 (3·18 to 3·79)	2·94 (2·38 to 3·61)
Stockholm	15·11 (11·31 to 19·98)	12·16 (8·92 to 16·06)	9·87 (8·74 to 11·13)	7·97 (7·07 to 8·93)	7·69 (6·97 to 8·44)	5·48 (4·86 to 6·19)	4·38 (3·85 to 4·94)	3·65 (3·23 to 4·12)	2·85 (2·47 to 3·26)	2·52 (2·06 to 3·10)
Sweden except Stockholm	14·86 (13·93 to 15·76)	11·57 (10·74 to 12·39)	8·85 (8·32 to 9·40)	7·87 (7·38 to 8·35)	7·72 (7·29 to 8·17)	5·24 (4·91 to 5·55)	4·40 (4·05 to 4·74)	3·78 (3·51 to 4·08)	3·67 (3·36 to 3·99)	3·07 (2·49 to 3·80)
Switzerland	20·93 (20·24 to 21·61)	15·67 (15·09 to 16·30)	11·86 (11·36 to 12·42)	9·95 (9·50 to 10·41)	9·48 (9·04 to 9·93)	6·99 (6·65 to 7·38)	6·46 (6·09 to 6·87)	5·50 (5·18 to 5·82)	4·80 (4·50 to 5·15)	4·31 (3·50 to 5·29)
United Kingdom	24·28 (23·85 to 24·72)	20·68 (20·31 to 21·07)	16·21 (15·89 to 16·56)	12·74 (12·48 to 13·00)	10·69 (10·45 to 10·92)	8·14 (7·95 to 8·35)	7·32 (7·10 to 7·55)	6·50 (6·36 to 6·65)	5·62 (5·47 to 5·77)	5·03 (4·54 to 5·57)
England	23·38 (22·63 to 24·09)	19·91 (19·37 to 20·46)	15·94 (15·57 to 16·34)	12·55 (12·31 to 12·80)	10·66 (10·44 to 10·88)	8·07 (7·90 to 8·25)	7·32 (7·07 to 7·56)	6·48 (6·33 to 6·64)	5·63 (5·48 to 5·78)	5·04 (4·66 to 5·44)
East Midlands	23·20 (21·56 to 24·83)	19·71 (18·62 to 20·78)	15·75 (14·66 to 16·77)	12·05 (11·42 to 12·70)	10·84 (10·29 to 11·43)	7·99 (7·55 to 8·43)	7·35 (6·86 to 7·88)	6·63 (6·26 to 7·01)	5·77 (5·43 to 6·11)	5·71 (5·12 to 6·34)
East of England	21·32 (18·06 to 24·86)	17·67 (15·49 to 19·88)	13·99 (12·69 to 15·40)	11·07 (10·53 to 11·62)	8·42 (7·95 to 8·90)	7·00 (6·60 to 7·47)	5·96 (5·59 to 6·35)	5·48 (5·16 to 5·83)	4·93 (4·60 to 5·30)	4·30 (3·59 to 5·07)
Greater London	24·73 (22·79 to 26·62)	21·29 (20·37 to 22·19)	17·21 (16·64 to 17·79)	13·30 (12·87 to 13·75)	11·43 (11·05 to 11·82)	9·01 (8·68 to 9·36)	8·07 (7·70 to 8·47)	6·66 (6·40 to 6·94)	5·85 (5·56 to 6·14)	4·43 (3·91 to 4·97)
North East England	22·65 (20·19 to 25·24)	19·32 (18·09 to 20·55)	15·52 (14·36 to 16·72)	12·19 (11·56 to 12·91)	10·97 (10·34 to 11·63)	8·51 (7·95 to 9·10)	7·63 (7·04 to 8·26)	6·25 (5·72 to 6·76)	5·13 (4·75 to 5·56)	4·84 (4·29 to 5·46)
North West England	24·17 (19·89 to 28·97)	20·80 (16·60 to 25·78)	16·68 (15·19 to 18·28)	13·07 (12·55 to 13·59)	11·03 (10·59 to 11·51)	8·43 (8·02 to 8·84)	8·04 (7·60 to 8·51)	7·24 (6·89 to 7·63)	6·24 (5·89 to 6·59)	5·55 (4·97 to 6·15)
South East England	21·39 (19·48 to 23·36)	18·08 (17·13 to 19·08)	14·47 (13·85 to 15·03)	11·72 (11·32 to 12·16)	9·75 (9·37 to 10·16)	6·76 (6·48 to 7·06)	5·87 (5·55 to 6·21)	5·27 (5·03 to 5·52)	4·70 (4·45 to 4·96)	4·11 (3·69 to 4·55)
South West England	22·45 (20·57 to 24·38)	18·88 (17·71 to 20·13)	15·02 (13·99 to 16·11)	11·75 (11·08 to 12·47)	9·48 (8·98 to 9·98)	7·29 (6·88 to 7·68)	6·34 (5·89 to 6·82)	5·71 (5·39 to 6·09)	4·81 (4·50 to 5·13)	4·77 (4·27 to 5·33)
West Midlands	24·83 (22·90 to 26·81)	21·38 (20·22 to 22·60)	17·28 (16·58 to 17·92)	13·96 (13·31 to 14·62)	12·85 (12·34 to 13·35)	9·12 (8·68 to 9·53)	8·80 (8·31 to 9·32)	8·37 (8·00 to 8·74)	7·24 (6·86 to 7·62)	7·38 (6·69 to 8·10)
Yorkshire and the Humber	24·87 (23·24 to 26·55)	21·36 (20·38 to 22·30)	17·21 (16·22 to 18·26)	13·67 (13·22 to 14·12)	11·40 (10·87 to 11·91)	9·10 (8·68 to 9·53)	8·52 (8·09 to 9·00)	7·56 (7·09 to 8·06)	6·38 (6·04 to 6·72)	5·63 (5·08 to 6·20)

Appendix Table 11B. Under-5 mortality rates by location, males, 1970, 1975, 1980, 1985, 1990, 1995, 2000, 2005, 2010, 2016										
**Location**	**1970**	**1975**	**1980**	**1985**	**1990**	**1995**	**2000**	**2005**	**2010**	**2016**
Northern Ireland	30·32 (28·64 to 32·19)	24·20 (22·74 to 25·71)	17·91 (16·65 to 19·16)	13·63 (12·61 to 14·84)	10·69 (9·79 to 11·69)	9·22 (8·36 to 10·11)	7·28 (6·47 to 8·13)	7·33 (6·67 to 8·11)	6·60 (5·93 to 7·37)	5·32 (3·57 to 7·64)
Scotland	25·83 (24·95 to 26·65)	22·71 (21·86 to 23·58)	16·24 (15·55 to 16·94)	13·15 (12·48 to 13·80)	10·79 (10·25 to 11·38)	8·40 (7·90 to 8·91)	7·27 (6·78 to 7·73)	6·61 (6·14 to 7·06)	5·04 (4·69 to 5·38)	4·54 (3·51 to 5·80)
Wales	25·56 (20·06 to 32·58)	22·11 (17·09 to 27·93)	17·83 (15·78 to 20·09)	13·30 (12·46 to 14·16)	9·96 (9·32 to 10·62)	8·12 (7·55 to 8·69)	7·15 (6·62 to 7·73)	5·88 (5·44 to 6·36)	5·26 (4·88 to 5·69)	4·88 (3·96 to 5·94)
Southern Latin America	90·83 (89·94 to 91·69)	72·18 (71·20 to 73·13)	47·27 (46·82 to 47·70)	35·68 (35·38 to 36·00)	31·47 (31·17 to 31·75)	25·91 (25·70 to 26·12)	19·63 (19·41 to 19·83)	16·03 (15·86 to 16·20)	13·92 (13·74 to 14·10)	12·10 (11·37 to 12·91)
Argentina	88·14 (87·02 to 89·27)	71·09 (69·67 to 72·60)	48·39 (47·95 to 48·82)	38·64 (38·29 to 39·01)	35·95 (35·60 to 36·26)	30·96 (30·66 to 31·27)	22·60 (22·35 to 22·84)	17·96 (17·77 to 18·16)	15·40 (15·22 to 15·57)	13·45 (12·80 to 14·15)
Chile	103·12 (101·28 to 104·80)	76·84 (75·55 to 78·04)	43·45 (42·69 to 44·21)	27·43 (26·91 to 27·93)	22·33 (21·91 to 22·76)	15·42 (15·09 to 15·74)	12·06 (11·82 to 12·31)	10·33 (10·08 to 10·58)	9·84 (9·57 to 10·15)	8·49 (7·84 to 9·20)
Uruguay	60·04 (58·32 to 61·83)	64·02 (62·23 to 65·86)	51·24 (49·57 to 52·99)	40·69 (39·07 to 42·31)	28·79 (27·62 to 30·04)	24·57 (23·50 to 25·56)	18·03 (17·15 to 18·89)	15·99 (15·20 to 16·86)	12·23 (10·87 to 13·93)	9·91 (7·61 to 12·84)
Central Europe, Eastern Europe, and Central Asi	57·25 (51·00 to 64·53)	49·09 (45·89 to 52·73)	44·19 (41·99 to 46·72)	40·12 (38·44 to 41·87)	35·84 (34·30 to 37·47)	36·75 (34·82 to 38·89)	31·06 (28·96 to 33·32)	24·45 (22·09 to 27·09)	18·73 (16·44 to 21·50)	15·19 (12·17 to 19·30)
Eastern Europe	36·84 (32·60 to 42·42)	35·96 (32·74 to 40·16)	33·43 (31·43 to 35·82)	29·29 (27·99 to 30·76)	24·30 (23·17 to 25·55)	25·45 (24·13 to 26·87)	22·37 (21·01 to 23·96)	15·70 (14·75 to 16·88)	11·13 (10·37 to 12·03)	9·36 (8·44 to 10·62)
Belarus	40·93 (32·86 to 50·19)	34·29 (28·05 to 41·50)	30·03 (24·75 to 35·84)	27·30 (22·78 to 32·70)	23·57 (19·60 to 28·21)	21·45 (17·73 to 25·62)	18·43 (15·11 to 22·14)	13·38 (10·77 to 16·32)	9·10 (7·11 to 11·67)	6·30 (4·76 to 8·50)
Estonia	25·16 (23·60 to 26·76)	25·76 (24·20 to 27·40)	26·32 (24·69 to 27·93)	23·42 (21·85 to 25·07)	19·45 (18·18 to 20·84)	21·13 (19·53 to 22·60)	12·88 (11·72 to 14·10)	8·23 (7·29 to 9·31)	5·32 (4·67 to 6·13)	3·42 (2·59 to 4·46)
Latvia	25·61 (24·10 to 27·10)	29·87 (28·10 to 31·49)	24·78 (23·28 to 26·30)	19·81 (18·57 to 21·10)	19·94 (18·69 to 21·31)	22·88 (21·13 to 24·74)	14·35 (12·87 to 16·02)	10·87 (9·85 to 11·98)	8·57 (7·63 to 9·58)	5·23 (3·38 to 7·70)
Lithuania	26·05 (24·95 to 27·16)	26·92 (25·80 to 28·12)	21·68 (20·68 to 22·70)	21·26 (20·28 to 22·18)	15·35 (14·64 to 16·13)	17·61 (16·78 to 18·47)	12·58 (11·88 to 13·29)	9·78 (9·07 to 10·47)	6·53 (6·07 to 7·08)	5·09 (4·36 to 5·91)
Moldova	78·41 (52·66 to 112·50)	68·27 (46·77 to 94·74)	61·86 (49·11 to 76·88)	48·28 (39·48 to 58·17)	36·15 (29·39 to 43·51)	38·97 (32·13 to 47·25)	34·94 (28·04 to 42·79)	18·94 (14·45 to 24·11)	18·16 (13·56 to 23·53)	13·18 (8·68 to 18·93)
Russia	31·78 (31·52 to 32·02)	33·52 (33·24 to 33·77)	32·01 (31·67 to 32·33)	29·69 (29·31 to 30·03)	24·54 (24·35 to 24·73)	26·08 (25·77 to 26·38)	22·34 (21·92 to 22·82)	15·49 (15·33 to 15·63)	10·76 (10·64 to 10·87)	9·36 (9·03 to 9·74)
Ukraine	46·41 (29·83 to 67·19)	40·71 (27·90 to 56·67)	36·36 (27·53 to 46·67)	27·49 (22·27 to 34·01)	23·44 (18·91 to 28·52)	23·90 (19·25 to 29·58)	23·67 (18·28 to 30·12)	17·47 (13·11 to 22·92)	12·79 (9·40 to 16·92)	10·32 (6·38 to 16·02)
Central Europe	48·55 (45·06 to 52·70)	37·70 (35·39 to 40·40)	31·65 (30·15 to 33·29)	27·10 (26·16 to 28·14)	24·13 (23·46 to 24·85)	20·39 (19·88 to 20·93)	15·16 (14·64 to 15·72)	11·73 (11·36 to 12·14)	8·73 (8·46 to 9·01)	6·85 (5·58 to 8·53)
Albania	106·99 (69·86 to 161·46)	78·76 (52·18 to 112·44)	63·04 (47·33 to 82·51)	55·69 (46·83 to 66·03)	42·62 (36·14 to 49·90)	38·58 (32·61 to 45·28)	29·22 (24·22 to 35·36)	23·86 (18·50 to 30·15)	19·06 (13·49 to 26·47)	15·22 (9·72 to 23·01)
Bosnia and Herzegovina	59·00 (42·50 to 81·79)	47·19 (35·76 to 61·75)	36·72 (29·96 to 44·60)	27·94 (26·70 to 29·13)	18·85 (18·13 to 19·58)	22·65 (20·08 to 25·30)	12·45 (11·80 to 13·11)	9·81 (9·21 to 10·41)	7·67 (7·21 to 8·13)	5·82 (5·01 to 6·74)
Bulgaria	35·48 (34·47 to 36·59)	30·36 (29·44 to 31·31)	27·00 (26·22 to 27·92)	21·05 (20·28 to 21·87)	20·49 (19·73 to 21·33)	20·33 (19·45 to 21·27)	18·37 (17·44 to 19·34)	14·40 (13·57 to 15·21)	12·62 (11·86 to 13·43)	9·32 (6·32 to 13·50)
Croatia	40·31 (27·98 to 57·04)	32·08 (23·43 to 42·15)	25·72 (20·52 to 31·65)	20·57 (19·54 to 21·70)	14·21 (13·55 to 14·90)	12·98 (11·99 to 14·07)	10·15 (9·54 to 10·76)	7·62 (7·14 to 8·15)	6·08 (5·69 to 6·52)	4·96 (4·17 to 5·85)
Czech Republic	27·29 (26·35 to 28·24)	24·95 (24·19 to 25·70)	21·18 (20·52 to 21·87)	16·38 (15·73 to 17·01)	13·54 (12·93 to 14·19)	10·33 (9·81 to 10·87)	6·06 (5·65 to 6·50)	4·83 (4·50 to 5·19)	3·94 (3·65 to 4·23)	3·18 (2·66 to 3·81)
Hungary	44·16 (42·77 to 45·50)	40·85 (39·66 to 42·10)	27·94 (27·02 to 28·81)	24·91 (23·93 to 25·90)	18·78 (17·99 to 19·61)	13·91 (13·28 to 14·59)	11·60 (11·03 to 12·26)	8·21 (7·71 to 8·74)	6·34 (5·91 to 6·75)	6·19 (4·84 to 7·85)
Macedonia	71·41 (40·13 to 115·79)	58·76 (33·13 to 95·24)	55·21 (38·69 to 77·39)	44·69 (32·14 to 60·14)	37·79 (36·10 to 39·47)	25·74 (24·32 to 27·18)	15·73 (14·66 to 16·85)	15·10 (13·97 to 16·36)	10·65 (9·76 to 11·73)	10·98 (6·98 to 16·65)
Montenegro	35·89 (23·04 to 54·14)	30·47 (19·78 to 48·06)	25·37 (16·70 to 37·69)	21·09 (14·17 to 30·34)	17·05 (12·67 to 22·45)	17·24 (15·35 to 19·24)	18·91 (17·22 to 20·73)	12·18 (11·11 to 13·35)	7·71 (6·81 to 8·62)	5·72 (4·38 to 7·38)
Poland	43·49 (42·63 to 44·32)	32·56 (31·90 to 33·17)	28·06 (27·54 to 28·61)	23·60 (23·08 to 24·07)	20·81 (20·31 to 21·28)	17·50 (16·98 to 18·01)	10·49 (10·09 to 10·88)	8·18 (7·86 to 8·49)	6·36 (6·10 to 6·64)	4·86 (3·14 to 7·30)
Romania	60·38 (59·49 to 61·34)	44·42 (43·66 to 45·17)	37·79 (37·11 to 38·41)	34·51 (33·82 to 35·16)	37·35 (36·70 to 38·07)	28·54 (27·86 to 29·23)	25·79 (25·08 to 26·47)	20·27 (19·70 to 20·93)	14·83 (14·34 to 15·37)	10·58 (9·07 to 12·34)
Serbia	48·20 (31·77 to 69·01)	39·86 (27·35 to 56·98)	34·65 (23·93 to 48·11)	30·31 (21·80 to 40·63)	22·83 (17·59 to 29·31)	23·60 (22·72 to 24·49)	17·02 (14·09 to 20·45)	13·98 (11·54 to 16·86)	11·60 (11·04 to 12·19)	8·90 (7·42 to 10·50)
Slovakia	34·43 (33·02 to 35·72)	31·46 (30·13 to 32·87)	26·98 (25·90 to 28·11)	21·05 (20·12 to 22·03)	15·96 (15·18 to 16·77)	13·96 (13·13 to 14·83)	11·02 (10·29 to 11·81)	9·59 (8·89 to 10·35)	7·27 (6·63 to 7·90)	6·62 (4·84 to 8·94)
Slovenia	32·47 (23·30 to 44·19)	25·71 (20·01 to 32·69)	20·74 (18·19 to 23·52)	17·30 (16·38 to 18·29)	11·65 (10·86 to 12·40)	8·11 (7·54 to 8·72)	6·10 (5·57 to 6·63)	4·87 (4·45 to 5·32)	3·57 (3·25 to 3·94)	2·62 (2·23 to 3·05)
Central Asia	111·48 (93·02 to 132·63)	93·67 (84·13 to 104·22)	82·14 (76·46 to 88·98)	74·37 (69·61 to 78·99)	64·48 (59·88 to 69·44)	64·59 (59·69 to 70·36)	55·14 (49·75 to 61·02)	45·36 (38·72 to 52·98)	35·33 (28·56 to 43·07)	27·48 (19·94 to 37·38)
Armenia	112·44 (81·93 to 151·13)	87·48 (68·85 to 109·58)	73·01 (61·14 to 86·63)	60·74 (52·77 to 69·97)	49·83 (42·82 to 57·59)	47·35 (40·75 to 54·48)	33·95 (28·90 to 40·15)	26·36 (21·08 to 32·37)	18·92 (14·78 to 23·82)	13·51 (10·00 to 17·94)
Azerbaijan	130·23 (93·91 to 174·01)	99·77 (72·40 to 134·50)	92·74 (74·21 to 114·86)	89·89 (75·12 to 104·94)	83·08 (69·77 to 97·66)	81·82 (69·18 to 96·25)	67·73 (56·90 to 80·71)	54·92 (44·77 to 66·12)	41·63 (29·32 to 56·61)	32·61 (22·41 to 45·80)
Georgia	109·15 (82·76 to 140·41)	83·58 (67·59 to 102·21)	64·22 (55·19 to 74·86)	53·13 (47·14 to 59·22)	47·66 (42·63 to 53·22)	47·51 (42·30 to 53·52)	42·11 (36·72 to 48·13)	30·94 (25·43 to 37·08)	23·11 (17·99 to 29·36)	17·27 (12·62 to 23·11)
Kazakhstan	66·80 (51·04 to 85·19)	60·07 (51·29 to 70·09)	53·59 (46·39 to 61·06)	46·53 (41·36 to 52·57)	41·69 (36·75 to 46·26)	45·28 (39·25 to 51·79)	41·30 (34·44 to 48·71)	32·90 (26·36 to 40·40)	22·84 (18·16 to 28·21)	15·26 (11·08 to 20·56)
Kyrgyzstan	146·11 (124·67 to 170·17)	120·52 (107·75 to 133·73)	102·54 (93·43 to 112·05)	88·15 (81·33 to 95·09)	72·53 (67·70 to 77·56)	61·95 (57·76 to 66·35)	54·88 (50·48 to 59·33)	46·28 (42·07 to 50·63)	39·22 (35·11 to 43·47)	32·24 (26·81 to 38·51)
Mongolia	161·31 (134·47 to 190·56)	145·32 (123·82 to 170·19)	133·38 (116·86 to 151·01)	118·29 (108·19 to 129·67)	99·45 (91·27 to 108·04)	82·05 (74·52 to 90·17)	63·49 (56·99 to 70·21)	45·97 (40·34 to 51·46)	33·56 (28·69 to 39·04)	24·32 (18·88 to 30·85)
Tajikistan	188·12 (140·80 to 244·24)	167·68 (136·87 to 204·53)	134·23 (113·84 to 155·53)	122·98 (102·22 to 144·93)	104·93 (90·73 to 120·80)	99·48 (85·39 to 113·80)	79·63 (67·32 to 92·52)	60·89 (50·63 to 72·35)	48·79 (38·58 to 60·44)	42·14 (30·18 to 57·30)
Turkmenistan	193·83 (130·62 to 272·28)	160·66 (115·46 to 214·54)	133·66 (106·67 to 166·31)	114·78 (96·23 to 135·46)	101·42 (83·81 to 119·94)	98·09 (81·44 to 119·30)	91·44 (71·59 to 115·36)	75·22 (55·47 to 96·79)	56·78 (40·49 to 76·51)	42·15 (28·66 to 60·45)
Uzbekistan	98·92 (74·62 to 130·29)	81·18 (66·64 to 97·08)	73·45 (62·27 to 84·85)	66·50 (57·27 to 76·73)	53·72 (46·57 to 61·69)	55·92 (48·84 to 64·60)	47·07 (39·38 to 56·34)	41·87 (31·17 to 54·81)	34·31 (24·92 to 45·92)	26·48 (18·87 to 36·31)
Latin America and Caribbean	126·66 (118·03 to 134·95)	106·64 (101·04 to 113·27)	85·99 (81·18 to 91·29)	70·38 (66·79 to 74·22)	56·08 (53·41 to 59·14)	45·33 (42·47 to 48·50)	36·04 (33·18 to 38·88)	30·31 (27·90 to 32·98)	28·05 (24·11 to 32·80)	20·60 (16·17 to 26·29)
Central Latin America	111·44 (106·90 to 115·94)	93·11 (89·20 to 97·39)	72·95 (69·21 to 76·68)	58·13 (55·02 to 61·13)	46·59 (43·86 to 49·38)	38·71 (36·17 to 41·53)	31·43 (28·79 to 33·99)	26·28 (23·84 to 28·72)	22·26 (19·58 to 25·10)	18·46 (15·38 to 22·24)
Colombia	98·15 (91·98 to 104·60)	79·22 (73·69 to 84·73)	57·60 (53·34 to 62·28)	47·29 (43·46 to 51·47)	36·96 (33·76 to 40·22)	33·49 (30·54 to 36·75)	29·21 (26·10 to 32·41)	26·13 (23·11 to 29·56)	22·06 (18·81 to 26·03)	17·15 (13·89 to 21·14)
Costa Rica	74·60 (69·90 to 79·54)	52·88 (48·35 to 57·35)	35·64 (31·82 to 39·59)	26·78 (23·71 to 30·09)	22·19 (19·20 to 25·32)	19·16 (16·24 to 22·63)	17·08 (14·27 to 20·64)	15·34 (12·73 to 18·45)	13·77 (11·19 to 16·70)	11·62 (8·82 to 15·22)
El Salvador	158·20 (140·26 to 176·16)	126·70 (113·54 to 140·54)	102·43 (93·09 to 112·22)	80·40 (73·20 to 88·13)	57·63 (51·98 to 64·35)	43·14 (38·33 to 48·21)	32·17 (27·94 to 37·05)	22·80 (19·25 to 26·78)	17·45 (14·09 to 21·15)	13·17 (10·40 to 16·60)
Guatemala	182·73 (171·89 to 195·16)	153·99 (145·04 to 162·82)	127·77 (121·24 to 134·52)	103·81 (98·35 to 109·17)	83·61 (78·65 to 88·77)	67·07 (62·91 to 71·01)	54·18 (50·16 to 58·26)	45·08 (40·67 to 49·77)	37·01 (32·81 to 41·98)	29·07 (24·69 to 34·26)
Honduras	139·54 (128·14 to 151·67)	116·32 (108·71 to 124·40)	91·30 (86·30 to 96·96)	72·84 (68·90 to 76·93)	58·64 (55·35 to 62·30)	48·40 (45·44 to 51·57)	39·76 (36·70 to 42·84)	33·39 (30·14 to 36·83)	28·75 (25·30 to 32·85)	23·50 (20·09 to 27·94)
Mexico	110·72 (104·56 to 117·35)	94·03 (88·59 to 100·15)	76·26 (70·48 to 82·35)	60·22 (54·88 to 65·38)	48·09 (43·17 to 52·87)	38·21 (34·19 to 42·53)	30·67 (26·75 to 34·68)	25·17 (21·56 to 29·36)	21·11 (17·44 to 25·30)	17·27 (13·49 to 22·02)
Nicaragua	186·31 (173·46 to 200·16)	148·82 (140·16 to 157·68)	115·42 (109·36 to 121·63)	96·79 (89·81 to 103·85)	70·42 (66·52 to 74·18)	55·77 (52·35 to 59·25)	40·22 (37·40 to 43·16)	30·06 (27·19 to 33·10)	23·19 (20·30 to 26·66)	17·03 (14·32 to 20·61)
Panama	62·11 (57·48 to 66·88)	48·16 (43·96 to 52·70)	39·55 (35·31 to 44·06)	33·10 (29·07 to 37·42)	29·99 (26·29 to 34·10)	27·45 (23·89 to 31·68)	24·98 (21·55 to 29·11)	23·04 (19·63 to 26·96)	20·68 (17·17 to 24·95)	18·02 (14·10 to 22·93)

Appendix Table 11B. Under-5 mortality rates by location, males, 1970, 1975, 1980, 1985, 1990, 1995, 2000, 2005, 2010, 2016										
Location	1970	1975	1980	1985	1990	1995	2000	2005	2010	2016
Venezuela	72·35 (71·71 to 73·00)	64·72 (64·18 to 65·29)	45·25 (44·84 to 45·68)	35·92 (35·58 to 36·24)	31·65 (31·36 to 31·94)	30·70 (30·37 to 31·03)	23·74 (23·49 to 23·98)	20·28 (20·07 to 20·49)	18·81 (18·56 to 19·05)	18·65 (17·62 to 19·78)
Andean Latin America	196·74 (183·53 to 211·57)	155·49 (148·98 to 162·24)	127·79 (120·14 to 135·65)	104·05 (98·79 to 109·14)	81·68 (78·35 to 85·39)	63·02 (60·24 to 65·92)	47·49 (45·24 to 49·84)	37·95 (35·22 to 40·67)	29·69 (26·15 to 33·60)	22·62 (17·75 to 28·67)
Bolivia	237·42 (220·93 to 254·08)	199·59 (187·83 to 210·78)	166·10 (155·09 to 177·46)	140·99 (131·26 to 150·46)	119·18 (112·04 to 126·41)	96·72 (90·18 to 103·73)	76·69 (69·88 to 84·06)	62·46 (54·05 to 71·34)	49·63 (40·70 to 59·97)	37·14 (28·24 to 48·40)
Ecuador	129·08 (122·05 to 136·15)	104·04 (98·86 to 109·52)	80·01 (75·84 to 84·80)	63·20 (59·25 to 67·14)	51·10 (47·98 to 54·57)	42·16 (39·42 to 44·93)	35·36 (32·60 to 38·35)	29·44 (26·26 to 32·75)	23·72 (20·68 to 27·14)	18·94 (15·81 to 22·70)
Peru	211·61 (189·76 to 236·08)	162·34 (152·75 to 172·78)	136·36 (126·93 to 145·47)	110·39 (103·68 to 117·22)	82·87 (78·14 to 88·04)	60·50 (56·78 to 64·67)	41·32 (38·59 to 44·00)	31·59 (29·38 to 33·86)	24·45 (22·15 to 26·73)	18·74 (14·65 to 23·82)
Caribbean	122·23 (113·72 to 132·26)	109·52 (102·16 to 116·94)	100·87 (92·78 to 109·74)	85·94 (79·15 to 93·82)	73·63 (67·13 to 80·57)	64·42 (58·95 to 71·02)	53·39 (47·87 to 59·17)	51·41 (44·58 to 58·50)	77·21 (49·95 to 111·94)	36·10 (25·40 to 50·63)
Antigua and Barbuda	36·56 (25·27 to 49·57)	30·38 (21·37 to 41·42)	23·68 (16·60 to 32·93)	16·88 (12·05 to 23·07)	16·73 (11·92 to 22·63)	19·38 (14·14 to 25·85)	19·15 (14·00 to 26·01)	16·98 (12·48 to 23·23)	14·66 (10·49 to 21·00)	13·17 (8·50 to 19·62)
The Bahamas	53·07 (34·81 to 76·64)	42·90 (28·53 to 61·85)	35·53 (23·00 to 52·29)	29·92 (18·61 to 44·32)	24·11 (14·74 to 36·85)	19·26 (12·53 to 27·95)	15·10 (9·81 to 21·81)	14·99 (9·82 to 21·58)	12·92 (8·50 to 19·32)	11·66 (6·18 to 21·64)
Barbados	50·73 (35·48 to 71·49)	38·29 (26·11 to 54·03)	28·54 (18·68 to 42·80)	23·90 (14·75 to 36·47)	20·61 (11·75 to 32·45)	18·52 (10·97 to 29·21)	17·76 (10·69 to 27·41)	17·28 (11·39 to 24·80)	16·68 (11·09 to 24·92)	15·17 (7·46 to 28·42)
Belize	108·00 (84·80 to 132·66)	84·04 (68·44 to 102·36)	67·72 (54·96 to 80·42)	52·42 (43·10 to 62·83)	43·59 (35·88 to 52·38)	32·86 (25·72 to 41·35)	30·67 (23·57 to 38·55)	24·24 (17·85 to 32·95)	19·16 (13·53 to 26·26)	16·62 (9·28 to 27·98)
Bermuda	29·80 (24·69 to 36·16)	23·96 (18·89 to 29·70)	22·23 (17·58 to 27·87)	15·06 (11·60 to 19·58)	12·96 (9·94 to 16·58)	10·29 (7·64 to 13·86)	4·80 (3·18 to 7·21)	6·97 (5·10 to 9·29)	5·38 (3·81 to 7·38)	4·43 (2·75 to 6·82)
Cuba	44·19 (43·55 to 44·89)	33·22 (32·81 to 33·62)	26·59 (26·03 to 27·21)	21·28 (20·61 to 22·05)	15·74 (15·31 to 16·20)	12·73 (12·48 to 12·97)	9·76 (9·55 to 9·96)	8·05 (7·87 to 8·23)	6·80 (6·63 to 6·97)	5·94 (5·56 to 6·39)
Dominica	58·79 (42·66 to 77·66)	37·00 (26·41 to 49·97)	24·31 (16·85 to 32·98)	19·41 (13·04 to 27·31)	18·03 (12·60 to 25·08)	18·22 (12·87 to 24·75)	19·15 (13·64 to 26·12)	20·77 (15·25 to 28·35)	23·53 (16·63 to 33·70)	24·87 (14·79 to 40·32)
Dominican Republic	135·66 (122·55 to 149·06)	114·46 (103·27 to 125·74)	93·33 (84·83 to 102·46)	78·18 (71·45 to 85·79)	65·44 (59·50 to 71·66)	51·14 (46·32 to 56·39)	41·33 (37·27 to 45·84)	40·80 (36·46 to 45·52)	36·35 (31·70 to 41·75)	30·73 (24·27 to 38·71)
Grenada	55·57 (34·00 to 84·26)	47·30 (27·08 to 74·01)	37·33 (18·68 to 67·73)	28·18 (13·94 to 52·54)	20·93 (11·81 to 35·50)	17·25 (10·48 to 25·64)	16·52 (10·50 to 23·86)	15·38 (9·67 to 23·27)	15·70 (10·32 to 23·19)	16·77 (9·02 to 30·79)
Guyana	96·94 (89·75 to 104·74)	85·06 (77·26 to 92·96)	71·32 (64·25 to 78·93)	63·75 (57·96 to 69·70)	57·68 (53·37 to 62·08)	49·91 (45·91 to 53·71)	43·60 (40·06 to 47·70)	41·51 (37·65 to 45·67)	36·86 (31·98 to 42·27)	27·98 (22·10 to 35·31)
Haiti	293·27 (260·77 to 328·43)	250·94 (226·62 to 275·66)	217·93 (194·36 to 245·30)	183·29 (164·10 to 206·17)	156·93 (138·06 to 176·06)	133·06 (117·73 to 151·90)	105·14 (90·31 to 120·50)	93·56 (77·10 to 111·42)	161·71 (93·26 to 248·88)	59·01 (39·66 to 85·04)
Jamaica	62·15 (52·57 to 72·66)	51·86 (43·08 to 62·99)	43·86 (33·65 to 55·37)	37·11 (28·22 to 47·16)	32·93 (24·35 to 43·05)	29·00 (20·72 to 39·16)	26·37 (18·52 to 35·87)	23·61 (15·57 to 35·97)	23·25 (14·45 to 37·11)	21·44 (12·67 to 34·68)
Puerto Rico	33·20 (32·42 to 33·96)	25·10 (24·50 to 25·70)	22·27 (21·77 to 22·87)	19·44 (18·59 to 20·40)	17·47 (16·83 to 18·06)	16·67 (16·14 to 17·23)	13·90 (13·44 to 14·35)	11·69 (11·20 to 12·17)	9·87 (9·47 to 10·34)	7·27 (6·31 to 8·38)
Saint Lucia	67·27 (38·20 to 113·35)	47·84 (30·36 to 72·34)	34·01 (20·73 to 51·88)	27·32 (15·41 to 43·99)	23·47 (13·53 to 39·29)	21·09 (13·05 to 32·90)	19·78 (12·43 to 29·82)	21·54 (13·29 to 32·51)	21·72 (14·27 to 32·00)	19·01 (9·54 to 34·78)
Saint Vincent and the Grenadines	89·31 (59·55 to 124·75)	67·12 (43·87 to 96·71)	48·44 (31·86 to 68·34)	34·77 (22·12 to 51·06)	29·62 (19·40 to 43·48)	27·37 (18·53 to 38·07)	26·27 (18·61 to 35·68)	24·43 (17·47 to 33·32)	22·22 (15·66 to 31·30)	19·21 (11·99 to 31·04)
Suriname	74·61 (61·33 to 90·89)	69·87 (58·94 to 82·29)	64·68 (55·07 to 75·29)	59·25 (50·90 to 68·19)	54·65 (47·78 to 62·80)	51·36 (44·38 to 59·10)	49·15 (41·34 to 57·76)	45·84 (37·05 to 55·21)	42·01 (33·30 to 52·01)	37·57 (28·44 to 49·24)
Trinidad and Tobago	58·42 (53·13 to 63·68)	52·99 (48·01 to 58·57)	42·39 (36·97 to 48·25)	34·19 (28·90 to 39·88)	31·49 (25·85 to 37·75)	30·59 (24·71 to 37·25)	30·63 (24·77 to 37·77)	27·15 (21·01 to 35·43)	24·64 (17·08 to 34·13)	23·30 (13·37 to 38·52)
Virgin Islands, U.S.	36·15 (33·07 to 39·16)	32·28 (29·41 to 35·34)	28·89 (26·27 to 31·63)	23·96 (21·92 to 26·13)	21·85 (19·98 to 23·84)	19·60 (17·85 to 21·49)	16·02 (14·53 to 17·66)	13·86 (12·24 to 15·59)	12·39 (10·60 to 14·43)	10·43 (8·37 to 12·95)
Tropical Latin America	126·85 (108·75 to 145·14)	108·32 (95·87 to 122·70)	86·16 (76·41 to 97·17)	72·12 (65·08 to 80·25)	56·67 (51·02 to 63·09)	44·48 (39·33 to 50·62)	34·73 (29·77 to 40·47)	28·40 (23·44 to 34·11)	23·13 (18·04 to 29·97)	18·87 (14·27 to 24·88)
Brazil	126·52 (107·94 to 145·03)	108·26 (95·56 to 122·72)	86·46 (76·32 to 97·84)	71·79 (64·60 to 80·07)	57·59 (51·64 to 63·64)	45·48 (39·68 to 52·42)	35·25 (29·82 to 41·13)	28·61 (23·67 to 34·59)	23·29 (18·11 to 30·14)	18·96 (14·40 to 24·84)
Paraguay	76·23 (68·38 to 85·15)	67·89 (61·22 to 74·94)	58·53 (52·93 to 64·26)	48·76 (44·59 to 53·79)	37·26 (33·58 to 40·93)	32·32 (28·81 to 36·08)	32·08 (27·81 to 36·55)	29·60 (25·09 to 34·32)	25·60 (20·02 to 32·19)	21·00 (15·74 to 27·78)
Southeast Asia, East Asia, and Oceania	126·70 (115·68 to 138·49)	104·79 (96·91 to 112·82)	87·35 (81·28 to 94·06)	72·98 (67·52 to 78·50)	61·28 (56·29 to 66·43)	52·80 (48·64 to 57·40)	44·00 (40·93 to 47·35)	33·51 (31·07 to 36·02)	25·70 (23·52 to 28·10)	19·20 (16·69 to 22·10)
East Asia	117·11 (104·35 to 130·53)	93·95 (83·78 to 104·20)	77·60 (69·27 to 86·71)	64·53 (57·50 to 72·27)	54·17 (48·18 to 61·29)	47·37 (41·81 to 53·54)	38·62 (34·67 to 42·92)	25·72 (23·10 to 28·41)	18·45 (16·61 to 20·34)	13·48 (11·57 to 15·92)
China	119·58 (106·41 to 133·40)	96·16 (85·71 to 106·79)	79·66 (71·01 to 89·03)	66·13 (58·84 to 74·18)	55·47 (49·17 to 62·74)	46·10 (40·97 to 51·60)	36·54 (32·85 to 40·61)	25·50 (22·83 to 28·25)	17·97 (16·08 to 19·89)	12·87 (11·16 to 15·03)
North Korea	60·21 (46·55 to 76·14)	43·99 (33·82 to 54·75)	35·26 (26·82 to 45·17)	32·58 (25·18 to 41·25)	30·15 (23·06 to 38·66)	96·59 (41·09 to 174·34)	92·33 (45·02 to 152·80)	36·59 (28·68 to 46·59)	33·42 (26·06 to 42·71)	28·53 (21·95 to 37·73)
Taiwan (Province of China)	29·00 (28·41 to 29·61)	21·10 (20·63 to 21·66)	16·93 (16·50 to 17·38)	11·98 (11·67 to 12·34)	9·41 (9·12 to 9·73)	9·66 (9·34 to 9·98)	9·31 (9·02 to 9·60)	7·45 (7·16 to 7·76)	5·86 (5·56 to 6·16)	5·07 (4·42 to 5·89)
Southeast Asia	154·19 (143·54 to 164·07)	131·51 (122·81 to 140·15)	107·59 (101·99 to 113·21)	90·18 (85·99 to 94·53)	75·50 (71·41 to 79·71)	61·80 (58·28 to 65·58)	51·19 (47·72 to 55·02)	42·56 (39·05 to 46·33)	33·75 (30·45 to 37·25)	24·89 (21·33 to 29·02)
Cambodia	205·14 (173·97 to 238·77)	274·41 (200·68 to 358·99)	169·82 (153·40 to 187·29)	148·25 (138·99 to 158·43)	128·83 (121·13 to 137·23)	120·75 (113·17 to 128·09)	102·56 (95·23 to 109·96)	72·99 (66·34 to 80·29)	51·04 (44·90 to 57·49)	35·64 (29·17 to 43·23)
Indonesia	188·64 (175·46 to 201·33)	159·40 (148·97 to 169·85)	133·40 (124·80 to 142·22)	112·14 (104·57 to 119·56)	94·12 (88·38 to 100·26)	75·12 (70·36 to 80·07)	60·15 (54·47 to 65·97)	49·49 (45·13 to 54·10)	39·59 (34·15 to 45·16)	29·18 (23·34 to 36·20)
Laos	313·42 (220·75 to 426·80)	280·76 (209·86 to 360·28)	252·76 (202·93 to 306·31)	219·28 (191·22 to 249·68)	199·55 (176·89 to 222·57)	178·12 (158·21 to 200·57)	152·52 (134·94 to 171·76)	122·10 (106·13 to 139·23)	90·11 (75·06 to 107·37)	64·18 (45·02 to 89·16)
Malaysia	61·62 (60·45 to 62·83)	47·71 (45·17 to 50·14)	34·28 (31·55 to 37·13)	24·78 (24·18 to 25·37)	18·45 (18·03 to 18·92)	14·42 (14·11 to 14·76)	10·20 (9·92 to 10·49)	8·81 (8·58 to 9·06)	8·67 (8·25 to 9·10)	6·51 (5·47 to 7·72)
Maldives	413·27 (392·94 to 435·73)	339·36 (330·71 to 347·94)	285·13 (278·89 to 291·09)	193·47 (188·41 to 198·34)	113·23 (110·16 to 116·39)	63·07 (61·00 to 65·18)	33·86 (32·31 to 35·42)	19·93 (18·84 to 21·00)	12·99 (12·08 to 13·94)	8·28 (7·32 to 9·46)
Mauritius	92·62 (90·29 to 94·95)	66·51 (64·61 to 68·36)	44·28 (43·04 to 45·48)	33·30 (32·26 to 34·39)	25·78 (24·96 to 26·66)	23·00 (22·12 to 23·86)	20·37 (19·51 to 21·17)	18·43 (17·71 to 19·21)	17·56 (16·74 to 18·50)	15·53 (13·71 to 17·63)
Myanmar	227·09 (182·20 to 272·42)	180·12 (146·81 to 217·75)	149·66 (121·80 to 179·25)	130·30 (107·17 to 155·46)	117·38 (98·30 to 139·35)	106·19 (88·08 to 129·28)	92·77 (75·44 to 112·40)	73·87 (59·65 to 91·01)	50·44 (39·37 to 63·99)	31·75 (23·86 to 43·02)
Philippines	109·75 (103·50 to 116·72)	98·21 (93·09 to 103·83)	88·05 (82·99 to 93·33)	75·92 (71·65 to 80·39)	62·00 (58·50 to 65·58)	50·39 (47·16 to 53·71)	43·17 (39·94 to 46·36)	38·63 (35·14 to 42·41)	33·98 (30·11 to 38·26)	27·20 (22·90 to 32·32)
Sri Lanka	74·87 (73·37 to 76·42)	73·56 (72·04 to 75·04)	48·57 (47·61 to 49·56)	35·46 (34·60 to 36·26)	35·20 (33·82 to 36·71)	22·41 (21·77 to 22·98)	18·27 (17·69 to 18·87)	19·05 (18·56 to 19·55)	14·05 (13·13 to 15·03)	7·32 (5·37 to 9·89)
Seychelles	75·13 (71·08 to 79·52)	53·05 (49·52 to 56·68)	34·55 (31·94 to 37·45)	25·49 (23·22 to 28·08)	20·60 (18·37 to 22·92)	15·79 (13·85 to 17·87)	14·51 (12·93 to 16·30)	14·44 (12·81 to 16·18)	14·10 (12·38 to 16·06)	12·36 (9·84 to 15·46)
Thailand	94·78 (88·46 to 100·65)	75·26 (69·68 to 80·94)	57·57 (52·65 to 62·94)	43·57 (38·71 to 48·83)	32·29 (27·58 to 37·60)	22·73 (19·07 to 27·04)	17·00 (14·17 to 20·39)	13·08 (11·00 to 15·46)	9·91 (8·13 to 11·97)	6·97 (5·39 to 8·95)
Timor-Leste	313·41 (251·29 to 382·16)	283·02 (242·60 to 331·68)	263·66 (227·75 to 299·05)	192·63 (170·94 to 217·01)	158·56 (140·19 to 178·98)	124·31 (107·35 to 142·11)	104·50 (87·70 to 123·49)	74·20 (58·36 to 91·96)	52·79 (36·37 to 73·80)	38·50 (22·99 to 61·24)
Vietnam	121·26 (101·52 to 144·08)	95·27 (85·85 to 105·74)	78·61 (72·61 to 84·82)	65·29 (60·95 to 69·71)	53·49 (50·11 to 57·07)	41·92 (39·00 to 44·82)	32·11 (29·42 to 34·85)	25·40 (22·66 to 28·27)	20·41 (17·61 to 23·73)	15·27 (12·64 to 18·77)
Oceania	115·47 (93·52 to 139·84)	102·25 (84·76 to 121·81)	91·74 (77·36 to 107·65)	84·96 (71·59 to 100·39)	79·39 (64·60 to 95·91)	72·96 (56·95 to 92·62)	67·86 (49·70 to 92·16)	65·75 (42·78 to 95·22)	59·63 (36·04 to 92·98)	48·61 (27·87 to 80·46)
American Samoa	38·94 (35·18 to 42·71)	30·45 (26·71 to 34·53)	23·71 (20·60 to 27·17)	19·57 (17·12 to 22·26)	18·76 (16·52 to 20·95)	17·29 (15·18 to 19·55)	15·73 (13·97 to 17·58)	14·16 (12·59 to 15·87)	11·90 (10·19 to 13·98)	9·62 (7·49 to 12·27)
Federated States of Micronesia	93·01 (65·32 to 125·66)	79·29 (54·82 to 107·41)	67·62 (50·07 to 91·52)	58·08 (45·08 to 74·73)	49·56 (36·81 to 64·68)	41·53 (29·04 to 56·35)	38·70 (24·89 to 58·32)	25·28 (16·69 to 36·97)	22·69 (14·71 to 33·09)	19·33 (12·46 to 29·09)

Appendix Table 11B. Under-5 mortality rates by location, males, 1970, 1975, 1980, 1985, 1990, 1995, 2000, 2005, 2010, 2016

Location	1970	1975	1980	1985	1990	1995	2000	2005	2010	2016
Fiji	54·85 (48·21 to 61·92)	52·04 (42·22 to 63·80)	41·04 (31·27 to 53·35)	33·17 (23·87 to 44·05)	29·58 (19·86 to 43·53)	30·94 (22·10 to 41·96)	33·10 (26·02 to 41·64)	37·22 (28·93 to 46·67)	40·01 (29·41 to 53·12)	40·67 (23·83 to 66·06)
Guam	37·16 (34·08 to 40·55)	28·24 (24·78 to 31·80)	18·52 (15·89 to 21·34)	14·85 (12·38 to 17·48)	13·81 (11·90 to 15·78)	13·23 (11·30 to 15·24)	12·43 (10·81 to 13·94)	13·76 (12·14 to 15·51)	15·48 (13·00 to 17·97)	15·21 (10·63 to 21·04)
Kiribati	142·65 (105·01 to 185·60)	112·83 (74·38 to 164·32)	97·93 (58·87 to 158·74)	94·99 (60·31 to 142·90)	85·31 (63·02 to 111·52)	74·69 (59·28 to 92·97)	68·07 (53·35 to 85·39)	65·34 (47·35 to 87·64)	61·14 (37·79 to 92·95)	50·85 (26·43 to 89·80)
Marshall Islands	89·58 (49·08 to 148·79)	73·34 (39·51 to 125·34)	60·43 (35·28 to 100·11)	56·17 (37·24 to 81·37)	47·46 (34·13 to 66·41)	39·39 (27·08 to 55·10)	44·59 (27·32 to 67·45)	36·80 (23·45 to 53·87)	30·73 (17·12 to 49·70)	24·95 (13·27 to 43·80)
Northern Mariana Islands	22·41 (12·20 to 38·32)	16·79 (8·50 to 31·27)	12·84 (6·99 to 22·69)	9·95 (5·10 to 17·09)	7·70 (4·02 to 13·00)	6·53 (3·59 to 10·33)	5·01 (3·78 to 6·57)	3·80 (2·82 to 5·03)	2·37 (1·65 to 3·42)	2·70 (1·49 to 4·43)
Papua New Guinea	133·98 (105·58 to 164·35)	118·74 (95·34 to 144·80)	110·47 (91·16 to 131·86)	103·73 (85·41 to 124·54)	95·94 (77·39 to 117·70)	86·43 (66·18 to 111·01)	78·53 (55·02 to 108·33)	75·16 (46·14 to 112·57)	67·43 (38·81 to 107·48)	53·82 (30·19 to 90·18)
Samoa	63·28 (48·37 to 80·67)	50·00 (35·57 to 69·55)	34·53 (21·53 to 54·91)	29·56 (15·98 to 51·59)	24·75 (12·31 to 46·05)	20·64 (9·84 to 40·33)	17·51 (8·23 to 32·69)	15·75 (7·37 to 29·13)	13·99 (6·44 to 26·08)	11·54 (5·16 to 22·92)
Solomon Islands	125·60 (95·12 to 158·58)	100·07 (71·86 to 135·35)	66·19 (46·68 to 94·25)	53·98 (37·32 to 73·74)	48·58 (34·97 to 65·42)	42·35 (30·83 to 56·48)	39·26 (28·64 to 52·02)	39·16 (27·44 to 53·34)	34·88 (23·64 to 50·19)	28·21 (18·43 to 41·90)
Tonga	46·13 (32·13 to 63·65)	42·12 (28·58 to 58·56)	39·58 (25·60 to 57·23)	33·86 (20·90 to 51·74)	30·14 (17·91 to 46·52)	29·19 (17·19 to 46·10)	27·99 (16·30 to 45·27)	26·44 (15·57 to 43·10)	25·41 (14·51 to 43·11)	22·02 (11·53 to 39·05)
Vanuatu	104·83 (72·36 to 148·68)	81·55 (52·93 to 119·33)	62·14 (40·55 to 90·26)	50·73 (36·31 to 67·74)	45·19 (34·53 to 57·49)	41·81 (32·16 to 53·04)	43·11 (33·43 to 54·74)	44·98 (34·05 to 58·76)	40·30 (27·91 to 55·54)	34·73 (22·23 to 54·55)
North Africa and Middle East	207·51 (194·45 to 220·85)	162·92 (154·35 to 172·05)	128·62 (121·44 to 135·84)	102·73 (96·28 to 109·67)	77·03 (73·25 to 81·56)	64·46 (60·83 to 68·07)	53·29 (49·68 to 57·13)	43·81 (39·66 to 48·13)	35·97 (31·88 to 40·63)	29·55 (24·16 to 36·31)
North Africa and Middle East	207·51 (194·45 to 220·85)	162·92 (154·35 to 172·05)	128·62 (121·44 to 135·84)	102·73 (96·28 to 109·67)	77·03 (73·25 to 81·56)	64·46 (60·83 to 68·07)	53·29 (49·68 to 57·13)	43·81 (39·66 to 48·13)	35·97 (31·88 to 40·63)	29·55 (24·16 to 36·31)
Afghanistan	223·83 (169·42 to 291·44)	213·59 (169·90 to 264·86)	207·13 (168·25 to 249·35)	190·65 (158·31 to 226·91)	149·56 (130·00 to 172·50)	145·67 (128·54 to 164·72)	135·35 (117·83 to 155·18)	112·76 (94·01 to 134·83)	88·10 (69·77 to 113·10)	67·97 (50·10 to 94·41)
Algeria	181·29 (159·27 to 203·19)	145·73 (130·17 to 162·89)	107·80 (97·22 to 119·02)	72·84 (66·35 to 79·86)	52·47 (47·13 to 58·20)	43·19 (38·81 to 48·09)	35·52 (31·55 to 39·54)	29·70 (26·19 to 33·57)	25·47 (21·75 to 29·45)	19·70 (15·30 to 25·83)
Bahrain	87·55 (67·98 to 109·89)	53·88 (43·67 to 65·87)	29·61 (27·10 to 32·35)	22·04 (20·40 to 23·72)	22·33 (20·78 to 23·95)	18·37 (16·83 to 19·92)	12·83 (11·72 to 14·00)	13·32 (12·08 to 14·57)	8·52 (7·71 to 9·37)	7·12 (5·47 to 9·14)
Egypt	234·85 (217·26 to 253·47)	199·19 (182·97 to 215·19)	155·17 (140·51 to 170·17)	112·18 (102·35 to 123·04)	81·35 (73·71 to 89·36)	61·14 (54·93 to 68·02)	45·02 (39·94 to 50·98)	36·28 (30·99 to 41·53)	29·42 (24·97 to 34·67)	20·64 (13·84 to 30·12)
Iran	256·69 (211·53 to 303·63)	148·21 (124·19 to 175·64)	111·22 (94·14 to 127·70)	87·32 (74·06 to 102·42)	71·21 (61·06 to 83·41)	54·04 (45·10 to 63·70)	44·05 (35·57 to 54·12)	32·23 (24·14 to 42·33)	24·26 (17·71 to 32·69)	19·70 (13·92 to 27·15)
Iraq	135·20 (111·88 to 158·69)	111·18 (97·39 to 124·99)	87·34 (78·43 to 98·88)	75·99 (67·38 to 89·21)	57·17 (53·20 to 61·33)	55·17 (51·44 to 59·25)	45·66 (42·24 to 49·31)	42·74 (38·25 to 47·48)	35·49 (30·47 to 41·33)	29·84 (23·78 to 38·18)
Jordan	98·92 (88·37 to 109·75)	79·76 (72·52 to 86·71)	57·58 (52·94 to 62·89)	42·36 (39·38 to 45·46)	34·83 (32·30 to 37·55)	30·96 (28·70 to 33·36)	27·43 (25·48 to 29·57)	23·59 (21·48 to 25·74)	19·97 (17·58 to 22·64)	17·45 (14·29 to 21·65)
Kuwait	50·60 (48·24 to 53·02)	46·90 (44·90 to 48·79)	33·81 (32·07 to 35·44)	22·55 (21·43 to 23·75)	15·81 (13·82 to 17·87)	13·47 (12·60 to 14·41)	13·56 (12·67 to 14·51)	12·02 (11·23 to 12·92)	11·53 (10·79 to 12·28)	10·10 (7·57 to 13·23)
Lebanon	64·16 (51·58 to 80·56)	62·92 (49·00 to 79·24)	64·63 (44·77 to 90·79)	41·29 (32·28 to 52·61)	33·05 (27·39 to 39·75)	24·37 (19·67 to 29·70)	18·01 (13·73 to 23·04)	13·47 (9·52 to 18·41)	10·94 (8·08 to 14·81)	9·09 (6·70 to 12·08)
Libya	119·52 (105·65 to 135·05)	88·53 (79·41 to 98·49)	64·56 (57·69 to 71·77)	50·45 (45·11 to 56·41)	42·39 (37·40 to 47·76)	34·21 (29·40 to 39·64)	28·50 (23·29 to 34·62)	22·91 (17·53 to 29·07)	16·95 (12·46 to 22·59)	14·48 (10·18 to 20·37)
Morocco	204·47 (193·37 to 216·31)	171·03 (162·12 to 180·59)	132·86 (125·24 to 140·36)	100·48 (94·37 to 107·05)	75·36 (69·87 to 80·91)	59·99 (54·55 to 65·23)	49·12 (42·73 to 55·46)	40·06 (32·32 to 48·70)	31·84 (24·54 to 40·38)	23·72 (17·23 to 32·15)
Palestine	77·27 (57·00 to 102·93)	69·80 (56·20 to 85·33)	57·85 (49·78 to 67·71)	42·10 (36·45 to 49·01)	39·46 (34·17 to 44·84)	29·83 (25·85 to 34·63)	27·88 (23·97 to 32·83)	26·78 (22·83 to 31·75)	23·35 (19·50 to 27·60)	18·13 (13·03 to 24·73)
Oman	267·55 (234·96 to 303·74)	196·76 (169·85 to 223·75)	130·70 (110·60 to 151·73)	79·10 (65·15 to 95·50)	49·02 (39·25 to 61·39)	29·59 (23·74 to 36·85)	16·82 (14·26 to 19·71)	8·22 (7·81 to 8·64)	9·16 (8·78 to 9·58)	8·61 (7·52 to 9·75)
Qatar	72·71 (50·26 to 99·35)	63·23 (47·87 to 82·21)	36·20 (25·83 to 48·62)	28·28 (20·94 to 37·19)	22·83 (16·06 to 31·93)	20·15 (14·38 to 27·94)	17·79 (12·51 to 24·42)	14·76 (10·52 to 20·52)	12·47 (8·86 to 16·97)	10·33 (6·82 to 15·04)
Saudi Arabia	161·24 (126·67 to 200·41)	117·85 (95·56 to 144·01)	87·71 (71·82 to 106·12)	65·37 (52·44 to 80·33)	51·24 (39·55 to 65·14)	37·43 (27·79 to 50·36)	26·23 (18·91 to 36·17)	18·48 (12·64 to 25·82)	12·67 (8·73 to 18·46)	7·27 (4·89 to 10·83)
Sudan	201·65 (183·12 to 223·83)	195·82 (179·77 to 214·66)	183·56 (165·86 to 203·02)	240·65 (181·82 to 300·56)	143·22 (128·87 to 158·17)	122·75 (110·26 to 135·20)	101·91 (91·33 to 113·73)	85·02 (74·97 to 95·36)	82·16 (70·60 to 94·19)	67·93 (52·90 to 88·21)
Syria	105·54 (98·03 to 113·82)	81·33 (74·91 to 87·78)	64·72 (58·74 to 71·06)	53·92 (48·48 to 59·74)	46·17 (41·68 to 50·93)	31·66 (28·92 to 34·57)	20·01 (19·16 to 20·77)	17·22 (16·64 to 17·69)	17·00 (16·35 to 17·59)	24·99 (14·13 to 40·62)
Tunisia	165·76 (155·99 to 176·01)	122·29 (114·87 to 130·10)	89·53 (84·04 to 94·92)	66·98 (62·45 to 72·01)	51·68 (47·79 to 55·87)	39·40 (35·86 to 43·15)	29·55 (26·33 to 32·99)	23·12 (19·83 to 26·74)	18·86 (15·71 to 22·42)	14·14 (11·14 to 17·90)
Turkey	202·25 (184·30 to 220·81)	168·97 (153·89 to 187·01)	131·09 (117·31 to 146·57)	97·24 (86·55 to 108·74)	74·53 (66·25 to 84·17)	59·10 (52·08 to 67·72)	43·36 (37·12 to 50·24)	31·59 (25·82 to 38·43)	24·09 (18·02 to 32·05)	17·25 (11·87 to 24·56)
United Arab Emirates	116·71 (80·67 to 161·03)	84·23 (55·35 to 125·79)	56·47 (36·04 to 82·06)	35·15 (19·78 to 57·55)	24·46 (13·09 to 40·17)	17·13 (8·86 to 30·26)	11·71 (6·27 to 19·76)	7·10 (5·61 to 8·95)	7·30 (4·20 to 11·71)	6·32 (3·29 to 11·31)
Yemen	326·23 (307·43 to 347·66)	257·78 (245·03 to 271·64)	198·70 (186·90 to 210·53)	157·99 (147·94 to 168·55)	131·51 (122·26 to 141·00)	110·04 (102·39 to 118·09)	89·69 (82·36 to 97·13)	70·08 (63·43 to 77·47)	53·72 (47·25 to 61·33)	44·62 (36·86 to 55·29)
South Asia	213·90 (195·88 to 233·11)	184·39 (172·61 to 196·37)	158·29 (149·49 to 167·21)	135·50 (128·55 to 142·62)	114·92 (108·96 to 120·99)	98·21 (92·68 to 104·52)	83·35 (78·05 to 89·05)	70·24 (64·88 to 76·04)	56·42 (50·70 to 63·06)	40·41 (34·93 to 47·48)
South Asia	213·90 (195·88 to 233·11)	184·39 (172·61 to 196·37)	158·29 (149·49 to 167·21)	135·50 (128·55 to 142·62)	114·92 (108·96 to 120·99)	98·21 (92·68 to 104·52)	83·35 (78·05 to 89·05)	70·24 (64·88 to 76·04)	56·42 (50·70 to 63·06)	40·41 (34·93 to 47·48)
Bangladesh	254·94 (238·22 to 270·70)	216·73 (204·62 to 228·79)	191·98 (181·78 to 202·83)	167·66 (159·58 to 176·78)	139·33 (132·11 to 146·43)	110·90 (104·35 to 117·40)	87·93 (81·81 to 93·89)	69·46 (64·64 to 74·33)	52·63 (48·15 to 57·26)	35·67 (31·60 to 40·27)
Bhutan	263·59 (222·24 to 307·50)	232·90 (199·69 to 269·50)	205·23 (177·69 to 235·21)	169·54 (149·26 to 190·59)	133·11 (118·94 to 147·78)	106·15 (95·24 to 117·83)	84·91 (75·11 to 95·53)	65·13 (56·90 to 75·38)	49·11 (41·41 to 58·57)	34·01 (27·83 to 42·21)
India	215·70 (195·89 to 236·99)	186·72 (172·97 to 200·74)	155·45 (143·93 to 167·24)	131·21 (122·37 to 140·03)	110·60 (103·86 to 117·58)	94·27 (88·08 to 100·51)	80·15 (74·70 to 85·73)	67·44 (61·57 to 73·98)	53·30 (46·47 to 61·42)	37·86 (31·51 to 46·04)
Nepal	249·31 (237·50 to 261·26)	227·41 (214·09 to 240·70)	201·21 (188·86 to 214·04)	168·86 (158·37 to 179·56)	135·72 (127·68 to 144·24)	105·03 (98·37 to 111·70)	80·96 (75·65 to 86·39)	63·06 (57·77 to 68·17)	47·00 (42·12 to 52·43)	30·61 (26·37 to 35·58)
Pakistan	171·43 (161·51 to 181·50)	153·15 (144·86 to 161·43)	137·48 (130·52 to 145·03)	122·53 (116·18 to 129·07)	108·61 (103·25 to 113·81)	97·28 (92·25 to 102·71)	88·95 (83·48 to 94·77)	83·31 (76·67 to 89·78)	70·59 (63·22 to 78·35)	53·95 (46·25 to 63·08)
Sub-Saharan Africa	244·14 (225·19 to 263·78)	227·49 (214·76 to 240·16)	209·44 (199·99 to 219·40)	194·35 (187·09 to 201·63)	181·91 (175·46 to 188·37)	170·54 (164·62 to 176·72)	154·73 (148·86 to 160·98)	132·12 (126·28 to 138·16)	109·16 (101·51 to 117·47)	82·09 (72·70 to 93·23)
Southern Sub-Saharan Africa	135·87 (119·06 to 154·19)	122·90 (114·09 to 131·78)	112·57 (105·92 to 119·58)	97·13 (92·11 to 102·42)	81·28 (76·76 to 85·57)	76·07 (71·16 to 81·43)	81·87 (75·42 to 88·66)	83·75 (75·74 to 91·99)	70·77 (63·13 to 78·37)	50·49 (42·04 to 61·31)
Botswana	105·59 (91·46 to 120·10)	86·24 (76·31 to 96·64)	73·66 (65·86 to 82·05)	63·68 (57·41 to 70·21)	54·89 (48·70 to 61·29)	63·61 (55·52 to 72·43)	69·53 (60·29 to 79·86)	52·13 (43·03 to 61·86)	30·13 (23·54 to 38·32)	16·91 (12·31 to 23·54)
Lesotho	119·06 (109·05 to 129·29)	110·88 (98·89 to 123·09)	101·65 (89·93 to 113·61)	97·48 (86·23 to 109·56)	92·13 (82·73 to 101·85)	93·50 (84·25 to 103·07)	109·95 (99·00 to 121·08)	122·35 (109·93 to 135·90)	99·34 (85·52 to 114·13)	74·42 (59·15 to 92·78)

Appendix Table 11B. Under-5 mortality rates by location, males, 1970, 1975, 1980, 1985, 1990, 1995, 2000, 2005, 2010, 2016

Location	1970	1975	1980	1985	1990	1995	2000	2005	2010	2016
Namibia	100·64 (80·93 to 123·32)	97·90 (83·56 to 114·35)	100·30 (86·62 to 115·73)	87·30 (76·54 to 99·39)	77·24 (68·02 to 87·41)	64·79 (56·07 to 73·95)	69·69 (59·43 to 80·62)	72·52 (60·52 to 85·94)	61·31 (49·41 to 74·35)	40·99 (26·81 to 60·75)
South Africa	140·11 (121·70 to 161·70)	126·85 (116·31 to 137·89)	116·37 (107·61 to 125·25)	102·43 (95·67 to 109·60)	84·20 (78·44 to 90·00)	74·39 (67·74 to 81·68)	79·44 (72·11 to 87·20)	80·17 (69·75 to 90·81)	65·21 (56·44 to 73·65)	46·27 (34·86 to 60·45)
Swaziland	195·23 (142·19 to 263·55)	157·04 (122·65 to 195·07)	126·49 (107·54 to 147·52)	99·50 (87·37 to 111·90)	75·20 (68·26 to 82·41)	77·94 (70·98 to 85·20)	103·33 (93·48 to 113·46)	114·66 (104·13 to 126·13)	81·78 (71·60 to 93·30)	54·75 (43·37 to 68·63)
Zimbabwe	116·36 (101·63 to 132·15)	107·59 (98·08 to 117·23)	100·55 (93·16 to 108·15)	83·23 (77·86 to 88·80)	75·02 (70·39 to 79·93)	80·93 (75·00 to 87·44)	85·34 (77·43 to 93·28)	90·36 (83·51 to 97·44)	85·10 (76·22 to 94·45)	62·13 (49·84 to 78·17)
Western Sub-Saharan Africa	277·36 (258·34 to 297·01)	256·08 (239·50 to 273·25)	234·01 (220·00 to 247·92)	216·60 (206·10 to 227·45)	204·70 (196·61 to 213·24)	193·51 (185·71 to 201·89)	178·00 (170·27 to 186·31)	156·65 (148·76 to 165·25)	132·25 (122·63 to 142·88)	103·40 (92·45 to 116·63)
Benin	289·77 (272·72 to 305·10)	261·16 (247·01 to 275·27)	236·34 (221·82 to 251·08)	211·71 (195·62 to 226·37)	191·59 (177·58 to 205·62)	170·41 (158·89 to 182·39)	146·76 (135·41 to 158·03)	121·66 (110·41 to 133·80)	100·28 (88·20 to 114·16)	76·78 (64·06 to 93·08)
Burkina Faso	317·58 (287·60 to 350·24)	294·90 (272·35 to 319·17)	265·71 (248·07 to 284·11)	240·83 (225·48 to 256·79)	219·39 (208·02 to 231·84)	206·15 (193·67 to 218·48)	187·11 (173·07 to 201·52)	164·68 (150·36 to 179·72)	143·46 (126·93 to 162·20)	117·25 (99·63 to 139·75)
Cameroon	218·44 (206·02 to 231·94)	195·88 (184·92 to 206·24)	175·93 (165·20 to 186·72)	154·25 (144·62 to 163·88)	143·46 (134·46 to 152·83)	147·04 (136·77 to 157·22)	143·36 (132·24 to 156·61)	131·49 (117·43 to 146·78)	115·26 (97·78 to 135·32)	93·27 (73·48 to 117·72)
Cape Verde	191·00 (184·56 to 197·57)	142·62 (136·88 to 148·39)	96·55 (92·04 to 101·34)	95·54 (91·39 to 100·06)	69·26 (65·59 to 73·14)	77·51 (62·42 to 93·88)	49·55 (43·02 to 56·80)	37·02 (32·19 to 42·47)	26·30 (24·04 to 28·83)	19·50 (17·03 to 22·33)
Chad	270·88 (238·82 to 305·83)	249·84 (225·68 to 274·82)	237·26 (219·33 to 255·55)	226·22 (211·40 to 241·67)	203·92 (190·27 to 218·80)	192·22 (177·69 to 206·34)	189·18 (173·63 to 205·63)	170·24 (153·39 to 187·56)	146·00 (128·59 to 166·61)	118·10 (100·08 to 141·38)
Cote d'Ivoire	240·97 (228·45 to 252·49)	207·31 (197·03 to 218·33)	178·75 (169·30 to 188·18)	168·31 (158·62 to 178·54)	164·25 (153·96 to 174·70)	163·08 (152·72 to 173·47)	151·61 (141·13 to 162·39)	139·19 (125·35 to 153·33)	120·98 (104·30 to 137·99)	96·14 (80·14 to 114·90)
The Gambia	209·41 (181·30 to 240·42)	186·01 (162·61 to 209·77)	162·88 (144·85 to 182·60)	140·15 (124·59 to 155·92)	123·25 (109·13 to 137·16)	106·98 (96·68 to 118·02)	92·19 (83·32 to 101·80)	77·66 (69·39 to 86·66)	64·57 (56·28 to 73·47)	52·39 (44·51 to 62·42)
Ghana	187·89 (177·53 to 198·29)	170·53 (161·78 to 180·11)	159·23 (151·26 to 167·58)	149·38 (141·69 to 156·73)	131·75 (124·50 to 139·00)	118·90 (112·97 to 125·00)	109·25 (103·71 to 115·28)	98·11 (92·09 to 104·54)	81·96 (74·50 to 89·84)	58·48 (49·17 to 70·65)
Guinea	316·07 (270·85 to 363·81)	303·56 (271·77 to 337·41)	281·49 (257·44 to 306·19)	261·01 (241·71 to 281·55)	234·86 (219·73 to 250·49)	210·22 (195·07 to 225·88)	181·48 (165·69 to 198·78)	157·91 (140·46 to 176·13)	133·44 (114·68 to 155·41)	103·63 (83·54 to 130·43)
Guinea-Bissau	340·52 (296·05 to 390·64)	306·19 (270·40 to 346·32)	279·56 (250·75 to 308·57)	259·79 (235·93 to 283·60)	230·30 (210·64 to 251·94)	200·71 (184·43 to 216·84)	172·63 (158·56 to 186·78)	150·39 (136·05 to 165·73)	111·45 (99·07 to 123·65)	79·07 (68·16 to 91·62)
Liberia	312·36 (280·11 to 345·06)	280·28 (255·81 to 305·99)	255·88 (236·19 to 275·48)	241·17 (224·29 to 258·43)	228·42 (214·06 to 243·52)	224·00 (212·04 to 237·17)	179·63 (169·60 to 189·81)	127·63 (117·50 to 137·58)	97·93 (87·54 to 109·68)	69·04 (58·24 to 83·05)
Mali	363·15 (334·82 to 392·02)	335·52 (314·07 to 358·12)	297·37 (280·34 to 315·40)	271·19 (255·77 to 285·76)	244·84 (231·63 to 259·03)	223·41 (210·01 to 237·45)	201·08 (187·04 to 217·04)	175·25 (159·46 to 191·72)	149·80 (131·90 to 170·09)	125·20 (102·15 to 155·53)
Mauritania	180·62 (171·55 to 190·20)	168·24 (158·69 to 178·36)	148·70 (139·83 to 157·65)	131·21 (123·14 to 139·40)	116·50 (108·86 to 124·79)	107·11 (98·76 to 115·70)	99·05 (90·75 to 107·85)	89·93 (80·52 to 99·40)	72·59 (63·39 to 82·25)	55·43 (46·80 to 65·62)
Niger	338·65 (308·70 to 371·91)	338·09 (314·06 to 362·91)	325·19 (305·78 to 345·65)	314·47 (296·01 to 333·67)	297·69 (281·95 to 314·47)	271·86 (255·27 to 288·34)	229·27 (212·00 to 246·74)	182·74 (164·98 to 201·09)	144·34 (125·48 to 166·04)	111·41 (92·35 to 136·39)
Nigeria	279·10 (249·77 to 308·48)	258·47 (235·44 to 282·42)	237·61 (218·78 to 256·43)	221·79 (209·59 to 235·12)	217·88 (206·70 to 229·64)	207·39 (196·37 to 218·43)	192·83 (182·34 to 203·22)	172·04 (161·41 to 183·01)	146·30 (133·68 to 160·14)	114·33 (97·72 to 133·46)
Sao Tome and Principe	153·39 (144·14 to 163·46)	123·17 (113·51 to 132·85)	119·49 (108·78 to 130·56)	131·09 (122·46 to 140·18)	112·92 (102·77 to 123·00)	97·95 (87·69 to 108·32)	80·43 (70·96 to 90·49)	67·03 (57·00 to 77·41)	47·44 (40·84 to 54·51)	34·96 (28·59 to 42·34)
Senegal	273·72 (257·14 to 291·82)	248·00 (234·12 to 262·47)	212·50 (199·60 to 225·55)	183·23 (173·12 to 193·68)	154·11 (145·80 to 162·53)	140·32 (131·02 to 150·05)	123·61 (114·28 to 133·35)	99·88 (93·75 to 106·18)	75·90 (69·98 to 82·56)	55·07 (48·74 to 62·66)
Sierra Leone	313·72 (275·18 to 356·41)	293·62 (262·83 to 327·93)	282·71 (257·69 to 307·84)	271·05 (251·88 to 291·26)	254·49 (238·68 to 271·02)	239·74 (225·28 to 253·39)	225·01 (211·35 to 237·83)	195·85 (181·94 to 208·70)	161·98 (147·26 to 176·45)	121·78 (106·73 to 138·88)
Togo	236·26 (213·38 to 259·01)	207·33 (190·35 to 224·35)	181·43 (168·95 to 194·32)	166·89 (155·50 to 178·56)	154·18 (143·00 to 166·22)	143·62 (131·94 to 155·25)	131·13 (119·67 to 142·59)	117·68 (105·81 to 130·28)	99·78 (88·13 to 113·49)	75·22 (63·96 to 89·64)
Eastern Sub-Saharan Africa	235·97 (218·30 to 252·45)	223·48 (209·72 to 236·30)	207·37 (197·03 to 217·63)	195·92 (187·53 to 204·30)	179·61 (172·31 to 186·59)	164·40 (158·07 to 170·83)	141·70 (135·43 to 148·13)	113·32 (106·59 to 119·97)	89·33 (81·25 to 97·86)	64·33 (54·85 to 76·46)
Burundi	245·59 (212·67 to 278·54)	239·37 (211·79 to 268·86)	219·98 (196·17 to 244·09)	191·41 (172·15 to 211·80)	184·39 (165·72 to 205·44)	182·14 (163·59 to 201·59)	172·28 (152·38 to 192·76)	133·44 (117·59 to 150·94)	102·84 (84·03 to 123·15)	86·17 (58·94 to 126·96)
Comoros	223·19 (176·57 to 271·04)	200·46 (169·90 to 229·59)	178·08 (156·41 to 199·75)	149·01 (132·25 to 168·69)	126·42 (111·42 to 141·92)	110·49 (97·60 to 124·57)	101·53 (88·06 to 115·72)	82·85 (70·63 to 95·79)	70·80 (56·43 to 87·89)	55·18 (39·09 to 78·68)
Djibouti	150·24 (125·82 to 177·80)	139·44 (119·55 to 160·64)	132·62 (117·45 to 148·75)	130·55 (117·77 to 143·82)	124·57 (112·93 to 136·38)	119·40 (106·48 to 132·65)	109·61 (95·77 to 123·67)	90·85 (77·00 to 106·41)	74·27 (61·15 to 90·99)	52·34 (41·92 to 66·31)
Eritrea	246·08 (213·32 to 281·41)	221·81 (200·07 to 245·48)	203·01 (184·78 to 220·57)	184·26 (169·27 to 200·99)	160·87 (146·01 to 175·50)	124·24 (112·03 to 137·95)	99·99 (86·80 to 114·20)	84·57 (70·17 to 100·42)	73·76 (59·24 to 91·08)	56·38 (44·28 to 73·01)
Ethiopia	265·77 (234·72 to 297·00)	264·48 (241·68 to 287·43)	247·28 (230·22 to 264·86)	225·01 (209·74 to 241·15)	204·75 (191·43 to 219·26)	178·70 (167·49 to 190·22)	149·98 (139·80 to 161·10)	114·11 (103·45 to 125·49)	80·27 (69·59 to 93·59)	47·60 (38·93 to 58·75)
Kenya	152·01 (143·24 to 160·93)	134·47 (127·14 to 142·67)	117·71 (109·90 to 125·52)	107·63 (101·60 to 114·78)	102·40 (96·45 to 108·72)	104·74 (98·21 to 111·10)	96·72 (90·19 to 103·55)	79·63 (73·46 to 86·70)	61·24 (54·75 to 67·92)	47·28 (40·04 to 55·85)
Madagascar	186·98 (163·77 to 213·39)	178·62 (158·76 to 200·45)	176·67 (158·60 to 195·70)	181·18 (162·93 to 200·86)	157·60 (144·11 to 171·74)	142·66 (129·04 to 157·19)	123·32 (109·96 to 136·11)	106·54 (93·04 to 119·86)	101·17 (85·23 to 117·43)	83·90 (66·63 to 107·05)
Malawi	348·92 (316·09 to 385·84)	310·83 (282·82 to 339·36)	272·30 (251·38 to 294·80)	253·61 (235·65 to 272·70)	234·52 (217·76 to 252·11)	203·08 (188·34 to 219·61)	172·94 (158·59 to 187·47)	129·75 (118·20 to 141·08)	105·25 (93·50 to 118·52)	73·12 (58·15 to 93·42)
Mozambique	266·65 (235·09 to 297·89)	249·63 (227·00 to 271·97)	240·25 (224·86 to 256·09)	265·53 (247·36 to 283·36)	224·49 (213·71 to 235·44)	204·02 (194·59 to 213·46)	170·24 (161·60 to 179·47)	137·07 (127·80 to 146·17)	109·72 (98·93 to 121·23)	81·15 (70·00 to 95·43)
Rwanda	263·06 (240·23 to 287·84)	257·25 (234·72 to 279·86)	222·39 (203·95 to 241·24)	182·30 (167·33 to 199·47)	177·02 (163·21 to 192·04)	193·70 (175·10 to 212·26)	164·51 (149·19 to 180·51)	111·68 (100·11 to 123·88)	71·04 (61·68 to 81·07)	53·12 (41·31 to 69·28)
Somalia	225·98 (179·91 to 274·97)	254·60 (204·95 to 304·91)	215·34 (178·63 to 252·99)	217·63 (183·87 to 249·54)	196·61 (169·66 to 221·87)	184·27 (159·92 to 209·70)	168·79 (146·94 to 192·01)	143·84 (120·95 to 168·95)	156·38 (124·74 to 192·46)	104·65 (81·38 to 134·67)
South Sudan	274·01 (208·36 to 358·12)	261·54 (205·84 to 328·67)	249·81 (205·68 to 297·30)	236·62 (207·10 to 267·85)	212·45 (191·21 to 235·01)	181·41 (164·30 to 198·30)	152·28 (138·95 to 165·63)	131·44 (117·57 to 145·70)	114·34 (97·19 to 133·10)	99·52 (78·09 to 125·48)
Tanzania	215·62 (198·78 to 231·85)	196·90 (185·88 to 207·83)	181·73 (173·43 to 190·87)	170·94 (163·60 to 178·29)	159·68 (152·69 to 167·25)	147·88 (141·11 to 154·66)	127·67 (121·42 to 134·23)	103·47 (96·82 to 110·20)	80·19 (72·65 to 88·20)	58·79 (50·57 to 69·36)

Appendix Table 11B. Under-5 mortality rates by location, males, 1970, 1975, 1980, 1985, 1990, 1995, 2000, 2005, 2010, 2016

Location	1970	1975	1980	1985	1990	1995	2000	2005	2010	2016
Uganda	221·58 (203·09 to 240·04)	211·48 (198·19 to 225·09)	211·06 (199·56 to 222·86)	200·35 (190·87 to 210·06)	187·31 (179·23 to 194·79)	172·24 (164·99 to 179·71)	147·62 (140·55 to 154·64)	123·49 (116·47 to 130·51)	99·29 (91·19 to 108·12)	69·27 (60·58 to 80·28)
Zambia	190·44 (167·19 to 216·64)	167·66 (149·37 to 184·97)	163·61 (148·24 to 181·39)	171·55 (156·58 to 188·18)	177·09 (160·23 to 194·64)	169·57 (153·70 to 185·53)	152·53 (137·68 to 167·62)	123·91 (109·74 to 137·34)	93·90 (80·06 to 109·56)	67·30 (53·63 to 83·83)
Central Sub-Saharan Africa	216·10 (170·66 to 265·91)	203·30 (166·63 to 240·45)	189·93 (165·74 to 214·55)	171·69 (155·59 to 190·16)	173·96 (159·30 to 188·68)	167·96 (152·75 to 184·26)	157·73 (142·09 to 174·81)	133·50 (120·69 to 147·57)	111·08 (95·36 to 127·04)	79·97 (60·07 to 104·95)
Angola	215·35 (164·96 to 274·24)	207·64 (161·88 to 257·51)	202·44 (165·39 to 242·42)	194·22 (168·45 to 221·02)	197·10 (174·22 to 222·41)	171·96 (151·22 to 193·52)	158·93 (139·36 to 180·76)	128·68 (113·04 to 147·54)	98·72 (82·94 to 118·43)	58·97 (43·93 to 77·91)
Central African Republic	180·31 (147·56 to 217·68)	185·22 (153·52 to 218·81)	179·37 (151·83 to 209·78)	180·59 (152·64 to 208·60)	185·09 (158·83 to 215·26)	185·11 (155·26 to 217·34)	188·60 (158·30 to 225·13)	179·18 (146·24 to 217·89)	163·90 (129·47 to 204·20)	137·97 (101·61 to 186·69)
Congo	167·46 (121·89 to 230·84)	145·78 (106·63 to 199·01)	123·67 (99·49 to 150·55)	102·73 (88·88 to 117·12)	99·09 (87·53 to 112·89)	104·43 (89·83 to 121·66)	108·83 (92·84 to 125·37)	92·56 (79·94 to 105·96)	73·22 (57·87 to 92·14)	57·73 (37·59 to 87·11)
Democratic Republic of the Congo	221·48 (167·54 to 282·88)	207·26 (164·83 to 255·79)	191·71 (160·02 to 225·06)	168·51 (147·18 to 193·20)	170·77 (149·91 to 192·86)	170·43 (148·50 to 194·01)	159·76 (138·40 to 185·71)	135·97 (117·44 to 157·45)	115·44 (94·83 to 138·18)	85·59 (62·20 to 114·53)
Equatorial Guinea	246·31 (179·00 to 326·15)	224·91 (175·04 to 278·60)	196·57 (159·36 to 235·82)	191·34 (159·67 to 225·39)	205·59 (175·13 to 241·55)	216·07 (182·59 to 254·44)	156·55 (126·50 to 189·56)	120·17 (90·33 to 156·07)	94·32 (64·90 to 132·67)	62·44 (40·87 to 95·65)
Gabon	186·70 (153·03 to 223·36)	153·05 (130·31 to 177·08)	124·55 (109·62 to 140·57)	105·08 (94·44 to 115·95)	94·04 (85·24 to 103·15)	83·98 (76·77 to 91·84)	78·38 (70·76 to 86·32)	73·82 (65·13 to 83·52)	63·53 (53·89 to 75·68)	50·10 (40·11 to 63·50)

Appendix Table 11C. Under-5 mortality rates by location, females, 1970, 1975, 1980, 1985, 1990, 1995, 2000, 2005, 2010, 2016

Location	1970	1975	1980	1985	1990	1995	2000	2005	2010	2016
Global	139·58 (134·84 to 144·50)	122·85 (119·08 to 126·69)	107·42 (104·03 to 110·86)	94·01 (91·34 to 96·67)	82·05 (79·70 to 84·56)	74·77 (72·76 to 76·85)	66·89 (65·48 to 68·36)	57·60 (55·80 to 59·34)	47·71 (45·24 to 50·40)	36·35 (33·32 to 39·86)
High SDI	83·37 (78·14 to 88·98)	65·22 (62·10 to 68·44)	52·93 (49·71 to 56·12)	41·93 (39·62 to 44·28)	34·48 (32·72 to 36·45)	28·65 (27·20 to 30·19)	22·35 (20·99 to 23·80)	16·91 (15·77 to 18·23)	12·95 (11·82 to 14·22)	10·12 (8·85 to 11·63)
High-middle SDI	23·28 (22·77 to 23·86)	18·17 (17·66 to 18·73)	13·76 (13·39 to 14·20)	10·85 (10·64 to 11·10)	9·05 (8·89 to 9·26)	7·32 (7·20 to 7·46)	6·17 (6·11 to 6·22)	5·58 (5·54 to 5·63)	4·88 (4·85 to 4·91)	4·44 (4·17 to 4·76)
Middle SDI	201·40 (195·28 to 207·77)	178·17 (171·75 to 184·24)	155·55 (150·58 to 160·45)	136·18 (131·95 to 140·71)	117·42 (113·24 to 121·60)	104·99 (101·87 to 108·15)	92·08 (89·79 to 94·35)	78·71 (75·95 to 81·45)	64·42 (61·02 to 68·10)	47·60 (43·92 to 51·84)
Low-middle SDI	228·30 (215·13 to 241·85)	214·19 (206·33 to 221·92)	197·81 (192·44 to 203·18)	182·64 (177·83 to 187·56)	166·19 (161·51 to 170·80)	152·66 (147·85 to 157·69)	134·53 (130·93 to 138·23)	111·37 (107·59 to 115·35)	91·12 (85·68 to 96·91)	65·77 (58·19 to 74·16)
Low SDI	132·06 (124·61 to 139·68)	108·26 (102·95 to 113·84)	87·95 (83·26 to 93·10)	71·23 (67·76 to 74·87)	57·26 (54·59 to 60·20)	46·52 (44·44 to 48·66)	37·65 (36·01 to 39·25)	30·32 (29·05 to 31·58)	23·49 (21·98 to 25·17)	17·60 (15·47 to 20·06)
High-income	26·65 (26·12 to 27·23)	21·13 (20·64 to 21·68)	15·18 (14·82 to 15·61)	11·73 (11·52 to 11·97)	9·96 (9·81 to 10·17)	7·97 (7·86 to 8·10)	6·77 (6·73 to 6·82)	6·07 (6·03 to 6·12)	5·35 (5·32 to 5·37)	4·86 (4·61 to 5·15)
High-income North America	20·97 (20·73 to 21·23)	16·68 (16·50 to 16·86)	13·21 (13·07 to 13·36)	11·02 (10·90 to 11·16)	9·63 (9·52 to 9·75)	7·97 (7·87 to 8·07)	7·18 (7·10 to 7·27)	7·04 (6·95 to 7·12)	6·34 (6·26 to 6·41)	6·02 (5·81 to 6·24)
Canada	20·03 (19·52 to 20·51)	15·26 (14·85 to 15·66)	11·02 (10·73 to 11·34)	8·63 (8·38 to 8·87)	7·35 (7·13 to 7·58)	6·52 (6·31 to 6·73)	5·59 (5·46 to 5·71)	5·55 (5·37 to 5·75)	5·26 (5·08 to 5·44)	4·94 (4·93 to 4·94)
Greenland	40·94 (37·14 to 45·03)	34·05 (31·11 to 37·35)	34·46 (31·25 to 38·38)	33·83 (30·25 to 38·01)	35·20 (31·24 to 39·79)	24·63 (21·67 to 28·19)	17·36 (15·20 to 19·88)	16·45 (14·34 to 18·90)	15·35 (13·24 to 17·81)	13·42 (12·07 to 14·92)
United States	21·08 (20·71 to 21·44)	16·97 (16·61 to 17·29)	13·53 (13·30 to 13·78)	11·25 (11·07 to 11·42)	9·85 (9·65 to 10·04)	8·14 (7·98 to 8·29)	7·35 (7·25 to 7·46)	7·20 (7·07 to 7·34)	6·50 (6·37 to 6·65)	6·20 (6·12 to 6·28)
Australasia	18·77 (18·42 to 19·15)	15·54 (15·21 to 15·87)	11·80 (11·53 to 12·08)	10·40 (10·15 to 10·65)	8·42 (8·20 to 8·64)	6·46 (6·31 to 6·60)	5·83 (5·68 to 5·99)	5·34 (5·18 to 5·49)	4·61 (4·47 to 4·74)	3·75 (3·52 to 4·01)
Australia	18·95 (18·51 to 19·38)	15·37 (15·01 to 15·75)	11·43 (11·13 to 11·74)	10·09 (9·81 to 10·39)	8·20 (7·95 to 8·45)	6·20 (6·02 to 6·40)	5·65 (5·47 to 5·83)	5·21 (5·05 to 5·36)	4·38 (4·23 to 4·53)	3·51 (3·28 to 3·77)
New Zealand	18·05 (17·46 to 18·64)	16·23 (15·69 to 16·77)	13·46 (12·96 to 13·95)	11·81 (11·43 to 12·21)	9·37 (9·05 to 9·71)	7·57 (7·26 to 7·89)	6·64 (6·44 to 6·83)	5·91 (5·64 to 6·18)	5·67 (5·43 to 5·91)	5·01 (4·79 to 5·26)
High-income Asia Pacific	28·49 (26·17 to 31·06)	20·03 (17·86 to 22·47)	13·99 (12·21 to 16·18)	9·70 (8·52 to 11·10)	7·55 (6·67 to 8·81)	6·18 (5·59 to 6·83)	4·90 (4·78 to 5·01)	3·94 (3·84 to 4·04)	3·19 (3·11 to 3·26)	2·70 (2·44 to 3·01)
Brunei	63·95 (61·65 to 66·37)	39·36 (37·69 to 41·05)	21·18 (19·81 to 22·64)	13·52 (12·64 to 14·46)	10·19 (9·46 to 11·01)	8·54 (7·93 to 9·29)	7·80 (7·32 to 8·31)	7·73 (7·22 to 8·29)	7·60 (7·06 to 8·16)	8·03 (7·25 to 8·96)
Japan	15·08 (14·85 to 15·34)	11·92 (11·70 to 12·13)	8·87 (8·70 to 9·03)	6·67 (6·52 to 6·81)	5·74 (5·61 to 5·87)	5·38 (5·19 to 5·58)	4·14 (4·04 to 4·24)	3·48 (3·40 to 3·57)	2·94 (2·86 to 3·03)	2·47 (2·31 to 2·64)
Singapore	24·41 (23·60 to 25·19)	16·56 (15·96 to 17·23)	13·09 (12·60 to 13·60)	9·39 (8·98 to 9·83)	6·64 (6·34 to 6·92)	4·53 (4·32 to 4·77)	3·40 (3·19 to 3·60)	2·67 (2·51 to 2·86)	2·38 (2·21 to 2·55)	1·98 (1·79 to 2·18)
South Korea	55·12 (48·27 to 62·75)	37·61 (30·69 to 45·34)	23·82 (18·33 to 30·46)	16·66 (12·56 to 21·58)	11·23 (8·46 to 15·04)	7·71 (6·15 to 9·53)	6·57 (6·33 to 6·82)	5·22 (5·02 to 5·44)	3·84 (3·69 to 4·01)	3·29 (2·59 to 4·09)
Western Europe	23·23 (23·00 to 23·46)	18·21 (18·02 to 18·39)	13·02 (12·90 to 13·15)	9·89 (9·77 to 10·01)	8·15 (8·06 to 8·23)	6·11 (6·04 to 6·18)	5·11 (5·06 to 5·17)	4·32 (4·27 to 4·38)	3·84 (3·80 to 3·88)	3·32 (2·97 to 3·72)
Andorra	19·45 (15·88 to 24·03)	11·31 (9·11 to 13·95)	6·57 (5·23 to 8·24)	5·93 (4·74 to 7·35)	5·41 (4·45 to 6·61)	3·93 (3·28 to 4·70)	2·89 (2·39 to 3·52)	3·05 (2·51 to 3·74)	2·93 (2·43 to 3·54)	3·07 (2·55 to 3·76)
Austria	25·77 (24·87 to 26·69)	20·79 (20·04 to 21·54)	14·55 (13·96 to 15·24)	11·43 (10·92 to 11·90)	8·40 (7·98 to 8·84)	5·96 (5·63 to 6·31)	5·01 (4·70 to 5·32)	4·31 (4·08 to 4·56)	3·98 (3·75 to 4·24)	2·95 (2·52 to 3·47)
Belgium	21·27 (20·95 to 21·62)	16·57 (16·29 to 16·85)	12·70 (12·44 to 12·96)	10·04 (9·82 to 10·28)	8·52 (8·35 to 8·68)	6·53 (6·37 to 6·70)	5·04 (4·92 to 5·17)	4·28 (4·14 to 4·42)	3·89 (3·76 to 4·03)	3·43 (3·15 to 3·73)
Cyprus	34·89 (31·26 to 38·96)	26·66 (24·93 to 28·83)	17·44 (16·46 to 18·66)	13·20 (12·45 to 14·13)	11·33 (11·00 to 11·70)	9·42 (8·70 to 10·28)	7·43 (6·75 to 8·23)	5·64 (5·09 to 6·26)	3·88 (3·49 to 4·32)	2·92 (2·47 to 3·41)
Denmark	14·12 (13·54 to 14·71)	10·45 (9·95 to 10·97)	8·75 (8·28 to 9·29)	8·27 (7·75 to 8·83)	7·79 (7·33 to 8·33)	5·91 (5·59 to 6·27)	5·02 (4·67 to 5·39)	4·32 (4·06 to 4·59)	3·81 (3·56 to 4·05)	3·75 (3·44 to 4·12)
Finland	14·19 (13·46 to 14·98)	10·96 (10·31 to 11·63)	7·70 (7·21 to 8·25)	6·40 (5·95 to 6·84)	5·98 (5·53 to 6·45)	4·42 (4·09 to 4·79)	3·73 (3·43 to 4·07)	3·48 (3·18 to 3·79)	2·69 (2·42 to 2·96)	1·96 (1·62 to 2·37)
France	15·98 (15·70 to 16·27)	14·29 (14·04 to 14·55)	10·73 (10·51 to 10·97)	8·63 (8·43 to 8·82)	7·59 (7·41 to 7·77)	5·32 (5·16 to 5·47)	4·74 (4·60 to 4·90)	3·88 (3·75 to 4·03)	3·74 (3·63 to 3·83)	3·41 (2·88 to 4·04)
Germany	22·40 (22·03 to 22·81)	18·80 (18·43 to 19·16)	13·28 (12·99 to 13·56)	9·64 (9·42 to 9·89)	7·91 (7·71 to 8·11)	5·76 (5·68 to 5·83)	4·81 (4·66 to 4·96)	4·27 (4·14 to 4·43)	3·79 (3·66 to 3·94)	3·27 (2·79 to 3·90)
Greece	31·72 (31·08 to 32·38)	24·63 (24·05 to 25·16)	18·38 (17·91 to 18·87)	13·96 (13·54 to 14·34)	9·65 (9·33 to 9·98)	7·81 (7·56 to 8·10)	5·81 (5·59 to 6·04)	4·21 (4·04 to 4·37)	3·74 (3·58 to 3·91)	3·62 (3·32 to 3·96)
Iceland	13·61 (12·57 to 14·75)	11·38 (10·72 to 12·19)	8·35 (7·65 to 9·17)	6·88 (6·24 to 7·59)	5·73 (5·15 to 6·38)	5·21 (4·62 to 5·93)	3·55 (3·12 to 4·05)	2·82 (2·48 to 3·25)	2·34 (2·02 to 2·68)	1·98 (1·73 to 2·29)
Ireland	20·12 (19·28 to 21·00)	18·04 (17·31 to 18·85)	12·58 (12·01 to 13·18)	9·49 (9·18 to 9·84)	8·23 (7·82 to 8·68)	6·57 (6·18 to 6·99)	6·47 (6·11 to 6·90)	4·53 (4·23 to 4·82)	3·80 (3·57 to 4·05)	3·54 (3·14 to 4·02)
Israel	29·34 (26·58 to 32·47)	27·48 (26·62 to 28·38)	15·94 (15·29 to 16·58)	12·75 (12·19 to 13·30)	10·91 (10·49 to 11·34)	8·16 (7·83 to 8·52)	6·55 (6·29 to 6·83)	5·27 (5·04 to 5·50)	4·30 (4·11 to 4·51)	3·33 (2·92 to 3·81)
Italy	29·45 (28·84 to 30·00)	20·70 (20·30 to 21·13)	14·65 (14·26 to 15·05)	10·63 (10·34 to 10·92)	8·36 (8·11 to 8·62)	6·77 (6·55 to 6·98)	4·95 (4·87 to 5·03)	3·93 (3·79 to 4·10)	3·65 (3·52 to 3·80)	2·92 (2·36 to 3·60)
Luxembourg	20·18 (18·92 to 21·55)	14·10 (13·12 to 15·11)	11·37 (10·68 to 12·08)	9·55 (8·65 to 10·54)	7·68 (6·90 to 8·47)	5·17 (4·67 to 5·75)	4·13 (3·79 to 4·54)	3·02 (2·66 to 3·45)	2·69 (2·33 to 3·12)	1·98 (1·81 to 2·18)
Malta	24·40 (22·76 to 26·23)	17·74 (17·09 to 18·41)	13·90 (12·92 to 15·01)	11·75 (10·85 to 12·64)	8·91 (8·17 to 9·73)	8·26 (7·53 to 9·00)	6·82 (6·09 to 7·56)	6·48 (5·78 to 7·28)	6·72 (6·03 to 7·45)	5·87 (5·30 to 6·52)
Netherlands	14·04 (13·63 to 14·46)	11·94 (11·57 to 12·33)	9·41 (9·09 to 9·76)	8·54 (8·22 to 8·88)	7·54 (7·26 to 7·85)	6·14 (5·89 to 6·41)	5·62 (5·41 to 5·85)	4·94 (4·74 to 5·14)	3·97 (3·78 to 4·15)	3·41 (3·15 to 3·70)
Norway	14·18 (13·56 to 14·79)	11·65 (11·12 to 12·21)	8·72 (8·24 to 9·27)	8·76 (8·31 to 9·24)	7·37 (6·99 to 7·76)	4·81 (4·54 to 5·11)	4·30 (4·03 to 4·57)	3·50 (3·29 to 3·73)	2·84 (2·63 to 3·04)	2·37 (2·06 to 2·75)
Portugal	58·99 (57·81 to 60·21)	39·01 (38·30 to 39·74)	25·76 (25·34 to 26·24)	18·16 (17·79 to 18·53)	12·42 (12·14 to 12·73)	8·52 (8·31 to 8·73)	6·22 (6·07 to 6·38)	4·46 (4·32 to 4·61)	3·38 (3·25 to 3·51)	2·79 (2·65 to 2·94)
Spain	28·08 (27·46 to 28·70)	19·15 (18·71 to 19·57)	13·24 (12·93 to 13·60)	9·74 (9·47 to 10·04)	8·35 (8·08 to 8·64)	6·19 (5·95 to 6·42)	4·84 (4·66 to 5·03)	4·14 (3·98 to 4·32)	3·58 (3·43 to 3·73)	3·01 (2·70 to 3·37)
Sweden	11·54 (11·00 to 12·07)	9·06 (8·59 to 9·55)	7·07 (6·68 to 7·51)	6·24 (5·85 to 6·62)	6·06 (5·72 to 6·41)	4·20 (3·92 to 4·47)	3·48 (3·23 to 3·74)	2·98 (2·76 to 3·20)	2·77 (2·55 to 3·00)	2·33 (1·93 to 2·80)
Stockholm	11·39 (8·60 to 15·04)	9·16 (6·76 to 12·06)	7·47 (6·59 to 8·43)	6·09 (5·41 to 6·80)	5·91 (5·35 to 6·47)	4·28 (3·78 to 4·81)	3·44 (3·03 to 3·86)	2·89 (2·55 to 3·27)	2·26 (2·00 to 2·54)	2·02 (1·63 to 2·48)
Sweden except Stockholm	11·84 (11·02 to 12·65)	9·16 (8·53 to 9·80)	7·05 (6·62 to 7·50)	6·25 (5·89 to 6·64)	6·06 (5·69 to 6·43)	4·15 (3·89 to 4·41)	3·47 (3·23 to 3·70)	2·97 (2·75 to 3·19)	2·87 (2·67 to 3·06)	2·39 (1·93 to 2·94)
Switzerland	15·77 (15·21 to 16·33)	11·68 (11·22 to 12·14)	8·93 (8·56 to 9·34)	7·70 (7·34 to 8·08)	7·40 (7·04 to 7·73)	5·53 (5·24 to 5·82)	5·13 (4·89 to 5·36)	4·52 (4·24 to 4·79)	4·10 (3·88 to 4·35)	3·57 (3·11 to 4·12)
United Kingdom	18·91 (18·56 to 19·28)	16·07 (15·74 to 16·38)	12·71 (12·45 to 12·99)	9·92 (9·68 to 10·16)	8·30 (8·11 to 8·49)	6·48 (6·32 to 6·64)	5·90 (5·74 to 6·07)	5·31 (5·20 to 5·43)	4·61 (4·47 to 4·73)	4·08 (3·83 to 4·38)
England	18·33 (17·80 to 18·81)	15·51 (15·10 to 15·91)	12·38 (12·10 to 12·69)	9·76 (9·57 to 9·95)	8·34 (8·17 to 8·52)	6·44 (6·29 to 6·59)	5·93 (5·76 to 6·13)	5·32 (5·19 to 5·44)	4·63 (4·51 to 4·76)	4·12 (3·82 to 4·44)
East Midlands	17·52 (16·28 to 18·80)	14·83 (13·98 to 15·68)	11·83 (11·00 to 12·58)	9·07 (8·59 to 9·59)	8·22 (7·80 to 8·64)	6·13 (5·79 to 6·47)	5·68 (5·31 to 6·07)	5·13 (4·83 to 5·44)	4·47 (4·20 to 4·73)	4·40 (3·95 to 4·88)
East of England	16·36 (14·04 to 18·85)	13·49 (11·97 to 15·06)	10·63 (9·66 to 11·63)	8·41 (8·00 to 8·83)	6·49 (6·12 to 6·86)	5·46 (5·14 to 5·82)	4·71 (4·45 to 4·99)	4·37 (4·09 to 4·66)	3·97 (3·69 to 4·25)	3·49 (2·93 to 4·10)
Greater London	19·43 (17·69 to 21·07)	16·64 (15·75 to 17·54)	13·42 (12·94 to 13·89)	10·39 (10·04 to 10·76)	9·00 (8·69 to 9·32)	7·19 (6·92 to 7·48)	6·54 (6·28 to 6·82)	5·49 (5·26 to 5·74)	4·88 (4·67 to 5·10)	3·72 (3·30 to 4·17)
North East England	17·93 (15·76 to 20·18)	15·18 (13·98 to 16·37)	12·12 (11·22 to 13·14)	9·47 (8·96 to 10·02)	8·52 (8·02 to 9·03)	6·65 (6·23 to 7·09)	5·99 (5·56 to 6·45)	4·94 (4·52 to 5·34)	4·07 (3·77 to 4·39)	3·83 (3·39 to 4·31)
North West England	19·07 (15·83 to 22·75)	16·31 (13·01 to 20·17)	13·05 (11·85 to 14·35)	10·18 (9·77 to 10·60)	8·60 (8·24 to 8·98)	6·64 (6·32 to 6·99)	6·36 (6·03 to 6·70)	5·78 (5·48 to 6·09)	5·01 (4·74 to 5·28)	4·46 (4·00 to 4·96)
South East England	16·17 (14·81 to 17·56)	13·65 (12·99 to 14·29)	10·91 (10·48 to 11·33)	8·89 (8·59 to 9·19)	7·49 (7·18 to 7·82)	5·31 (5·09 to 5·56)	4·67 (4·43 to 4·93)	4·23 (4·02 to 4·44)	3·80 (3·59 to 4·00)	3·34 (2·99 to 3·70)
South West England	16·97 (15·62 to 18·39)	14·23 (13·37 to 15·09)	11·31 (10·52 to 12·15)	8·87 (8·35 to 9·42)	7·24 (6·83 to 7·65)	5·64 (5·32 to 5·98)	4·95 (4·61 to 5·29)	4·46 (4·19 to 4·76)	3·76 (3·52 to 4·00)	3·72 (3·33 to 4·14)
West Midlands	19·65 (18·02 to 21·32)	16·79 (15·82 to 17·78)	13·52 (13·01 to 14·02)	10·87 (10·34 to 11·40)	9·96 (9·54 to 10·36)	7·09 (6·73 to 7·45)	6·83 (6·44 to 7·19)	6·50 (6·19 to 6·80)	5·65 (5·37 to 5·93)	5·74 (5·20 to 6·33)
Yorkshire and the Humber	19·61 (17·98 to 21·25)	16·73 (15·77 to 17·69)	13·44 (12·65 to 14·29)	10·71 (10·33 to 11·10)	9·01 (8·59 to 9·43)	7·31 (6·96 to 7·69)	6·93 (6·61 to 7·31)	6·23 (5·83 to 6·66)	5·35 (5·10 to 5·64)	4·76 (4·31 to 5·25)

Appendix Table 11C. Under-5 mortality rates by location, females, 1970, 1975, 1980, 1985, 1990, 1995, 2000, 2005, 2010, 2016										
Location	1970	1975	1980	1985	1990	1995	2000	2005	2010	2016
Northern Ireland	24·43 (22·96 to 25·93)	19·25 (18·08 to 20·45)	14·11 (13·08 to 15·09)	10·64 (9·81 to 11·60)	8·29 (7·58 to 9·04)	7·21 (6·56 to 7·87)	5·75 (5·18 to 6·37)	5·79 (5·24 to 6·39)	5·25 (4·74 to 5·81)	4·23 (2·96 to 5·89)
Scotland	19·82 (19·18 to 20·50)	17·37 (16·71 to 18·03)	12·36 (11·83 to 12·90)	9·95 (9·47 to 10·40)	8·16 (7·73 to 8·60)	6·45 (6·07 to 6·84)	5·64 (5·29 to 5·98)	5·18 (4·86 to 5·50)	3·99 (3·72 to 4·24)	3·60 (2·88 to 4·47)
Wales	19·91 (15·17 to 26·00)	17·06 (12·98 to 22·04)	13·63 (12·03 to 15·49)	10·06 (9·42 to 10·73)	7·54 (7·05 to 8·03)	6·19 (5·78 to 6·62)	5·50 (5·10 to 5·91)	4·55 (4·20 to 4·92)	4·09 (3·77 to 4·42)	3·81 (3·10 to 4·61)
Southern Latin America	78·91 (78·17 to 79·61)	61·49 (60·89 to 62·03)	39·04 (38·76 to 39·34)	28·94 (28·72 to 29·16)	25·46 (25·25 to 25·67)	20·87 (20·67 to 21·08)	15·86 (15·73 to 16·00)	13·03 (12·91 to 13·15)	11·38 (11·23 to 11·54)	9·86 (9·52 to 10·24)
Argentina	76·66 (75·77 to 77·57)	60·89 (60·22 to 61·55)	39·95 (39·52 to 40·42)	31·27 (30·96 to 31·59)	29·09 (28·74 to 29·43)	24·87 (24·60 to 25·16)	18·24 (18·03 to 18·45)	14·62 (14·45 to 14·80)	12·55 (12·39 to 12·72)	10·90 (10·61 to 11·22)
Chile	89·91 (88·30 to 91·49)	64·93 (63·71 to 66·17)	36·03 (35·30 to 36·77)	22·45 (21·96 to 22·91)	18·14 (17·76 to 18·54)	12·70 (12·46 to 12·96)	9·97 (9·74 to 10·21)	8·48 (8·29 to 8·67)	8·27 (8·05 to 8·53)	7·18 (6·97 to 7·41)
Uruguay	49·74 (48·33 to 51·27)	53·46 (51·98 to 55·02)	41·85 (40·36 to 43·27)	32·78 (31·44 to 34·04)	22·63 (21·71 to 23·64)	19·12 (18·28 to 19·92)	13·88 (13·22 to 14·54)	12·28 (11·69 to 12·95)	9·33 (8·28 to 10·62)	7·55 (5·85 to 9·71)
Central Europe, Eastern Europe, and Central Asi	48·13 (43·44 to 53·40)	40·64 (38·46 to 42·90)	36·24 (34·70 to 37·98)	32·43 (31·20 to 33·69)	28·66 (27·60 to 29·74)	29·57 (28·06 to 31·13)	24·75 (23·27 to 26·39)	19·10 (17·46 to 20·94)	14·66 (12·92 to 16·65)	11·81 (9·58 to 14·76)
Eastern Europe	28·26 (25·03 to 32·46)	27·57 (25·21 to 30·65)	25·48 (23·97 to 27·17)	22·11 (21·15 to 23·23)	18·22 (17·41 to 19·11)	19·42 (18·40 to 20·54)	17·14 (16·11 to 18·37)	11·98 (11·27 to 12·84)	8·62 (8·04 to 9·32)	7·29 (6·73 to 8·07)
Belarus	31·05 (24·95 to 38·07)	25·77 (21·06 to 31·33)	22·37 (18·48 to 26·68)	20·28 (16·97 to 24·29)	17·40 (14·53 to 20·79)	15·81 (13·09 to 18·96)	13·56 (11·13 to 16·33)	9·82 (7·92 to 11·97)	6·73 (5·26 to 8·60)	4·71 (3·59 to 6·29)
Estonia	18·32 (17·08 to 19·71)	18·37 (17·21 to 19·59)	18·82 (17·62 to 20·07)	16·90 (15·78 to 18·09)	14·19 (13·21 to 15·23)	15·69 (14·45 to 17·02)	9·59 (8·62 to 10·51)	6·28 (5·63 to 6·95)	4·26 (3·71 to 4·85)	2·87 (2·17 to 3·70)
Latvia	18·36 (17·28 to 19·64)	21·75 (20·53 to 23·22)	18·17 (17·02 to 19·35)	14·84 (13·85 to 15·95)	15·15 (14·16 to 16·15)	18·52 (17·35 to 19·83)	11·87 (11·07 to 12·78)	8·88 (7·95 to 9·85)	7·10 (6·36 to 7·95)	4·38 (3·25 to 5·84)
Lithuania	20·09 (19·20 to 21·04)	20·75 (19·85 to 21·68)	16·68 (15·90 to 17·48)	16·42 (15·63 to 17·24)	12·02 (11·43 to 12·63)	13·89 (13·20 to 14·64)	9·83 (9·22 to 10·39)	7·70 (7·18 to 8·21)	5·28 (4·89 to 5·74)	4·21 (3·81 to 4·67)
Moldova	63·52 (42·64 to 90·90)	55·00 (38·02 to 77·36)	49·58 (38·98 to 61·94)	37·94 (30·90 to 45·58)	28·18 (22·86 to 33·89)	30·97 (25·60 to 37·22)	27·63 (22·31 to 33·59)	14·57 (11·15 to 18·58)	13·89 (10·52 to 17·90)	10·00 (6·58 to 14·53)
Russia	24·35 (24·12 to 24·59)	25·76 (25·51 to 26·03)	24·37 (24·07 to 24·68)	22·44 (22·12 to 22·72)	18·35 (18·17 to 18·53)	19·91 (19·64 to 20·19)	17·16 (16·79 to 17·53)	11·85 (11·72 to 12·00)	8·39 (8·30 to 8·50)	7·33 (7·08 to 7·63)
Ukraine	35·63 (23·18 to 51·40)	31·00 (21·51 to 42·72)	27·75 (21·49 to 35·32)	20·59 (16·60 to 25·53)	17·61 (14·23 to 21·40)	18·14 (14·54 to 22·40)	17·98 (13·97 to 22·85)	13·23 (10·16 to 17·15)	9·77 (7·15 to 12·82)	7·95 (4·93 to 12·20)
Central Europe	40·19 (37·51 to 43·44)	30·47 (28·61 to 32·65)	25·28 (24·12 to 26·54)	21·76 (21·04 to 22·51)	19·34 (18·88 to 19·86)	16·79 (16·36 to 17·25)	12·22 (11·80 to 12·70)	9·37 (9·09 to 9·65)	7·03 (6·83 to 7·24)	5·51 (4·64 to 6·65)
Albania	111·13 (77·57 to 156·98)	79·34 (55·83 to 109·05)	61·61 (46·97 to 78·94)	53·09 (44·65 to 63·08)	38·63 (32·81 to 45·29)	35·09 (29·55 to 41·37)	25·58 (20·83 to 30·87)	19·88 (15·45 to 24·97)	15·49 (10·93 to 21·16)	12·11 (8·48 to 17·15)
Bosnia and Herzegovina	49·28 (35·91 to 67·47)	38·49 (29·74 to 49·67)	29·21 (23·99 to 34·93)	21·55 (20·74 to 22·42)	14·25 (13·67 to 14·79)	20·60 (18·07 to 23·51)	9·34 (8·87 to 9·86)	7·40 (6·96 to 7·86)	5·86 (5·49 to 6·23)	4·50 (3·87 to 5·20)
Bulgaria	28·57 (27·72 to 29·44)	24·23 (23·46 to 25·07)	21·37 (20·69 to 22·07)	16·52 (15·90 to 17·13)	16·14 (15·48 to 16·80)	16·06 (15·33 to 16·79)	14·50 (13·76 to 15·26)	11·32 (10·68 to 11·96)	9·84 (9·31 to 10·41)	7·31 (5·03 to 10·45)
Croatia	31·55 (23·24 to 42·66)	24·84 (19·07 to 31·77)	19·71 (16·45 to 23·33)	15·76 (14·98 to 16·60)	10·92 (10·38 to 11·50)	10·73 (9·52 to 12·05)	8·05 (7·60 to 8·52)	6·17 (5·74 to 6·61)	4·97 (4·62 to 5·34)	4·05 (3·42 to 4·74)
Czech Republic	20·53 (19·88 to 21·24)	18·60 (18·05 to 19·16)	15·71 (15·22 to 16·25)	12·21 (11·73 to 12·68)	10·10 (9·70 to 10·54)	7·75 (7·35 to 8·17)	4·65 (4·33 to 4·98)	3·70 (3·45 to 3·97)	3·02 (2·81 to 3·24)	2·43 (2·08 to 2·86)
Hungary	35·61 (34·37 to 36·92)	32·71 (31·61 to 33·83)	21·97 (21·21 to 22·78)	19·91 (19·16 to 20·73)	15·18 (14·54 to 15·93)	11·27 (10·68 to 11·87)	9·48 (8·94 to 10·02)	6·72 (6·29 to 7·14)	5·61 (5·21 to 6·02)	4·94 (4·12 to 5·96)
Macedonia	67·44 (37·86 to 109·57)	54·49 (30·81 to 88·34)	51·02 (35·92 to 71·32)	40·15 (28·85 to 54·30)	33·24 (31·72 to 34·75)	21·75 (20·51 to 22·97)	13·04 (12·14 to 13·97)	12·50 (11·56 to 13·51)	8·77 (8·00 to 9·63)	9·10 (5·77 to 13·83)
Montenegro	30·87 (20·68 to 44·93)	25·73 (17·76 to 38·09)	21·19 (14·40 to 30·70)	17·22 (11·51 to 24·26)	13·60 (10·09 to 17·81)	13·63 (12·06 to 15·30)	14·82 (13·36 to 16·35)	9·35 (8·51 to 10·28)	5·96 (5·29 to 6·69)	4·44 (3·40 to 5·68)
Poland	33·35 (32·56 to 34·16)	24·51 (24·00 to 25·06)	21·01 (20·51 to 21·49)	18·30 (17·83 to 18·80)	16·21 (15·78 to 16·66)	14·38 (13·92 to 14·86)	8·60 (8·27 to 8·95)	6·71 (6·45 to 7·00)	5·33 (5·09 to 5·57)	4·13 (2·94 to 5·74)
Romania	51·52 (50·66 to 52·36)	37·13 (36·45 to 37·80)	31·22 (30·66 to 31·81)	28·13 (27·55 to 28·69)	30·44 (29·84 to 31·07)	23·05 (22·49 to 23·66)	20·73 (20·14 to 21·32)	16·12 (15·62 to 16·65)	11·74 (11·34 to 12·17)	8·35 (7·16 to 9·70)
Serbia	39·49 (26·25 to 56·70)	32·05 (21·98 to 45·83)	27·51 (19·02 to 38·09)	23·63 (16·93 to 31·72)	17·51 (13·54 to 22·41)	19·21 (18·52 to 19·93)	13·15 (10·89 to 15·85)	10·63 (8·82 to 12·76)	8·81 (8·40 to 9·26)	6·82 (5·70 to 8·04)
Slovakia	26·66 (25·55 to 27·77)	23·98 (23·25 to 24·82)	20·32 (19·52 to 21·19)	15·96 (15·19 to 16·74)	12·35 (11·69 to 13·04)	10·95 (10·32 to 11·63)	8·75 (8·14 to 9·46)	7·66 (7·11 to 8·27)	5·86 (5·45 to 6·30)	5·46 (4·26 to 6·99)
Slovenia	24·97 (18·49 to 33·09)	19·44 (15·43 to 24·40)	15·49 (13·52 to 17·64)	12·84 (12·12 to 13·55)	8·64 (8·10 to 9·19)	6·13 (5·69 to 6·63)	4·71 (4·29 to 5·13)	3·83 (3·50 to 4·16)	2·85 (2·59 to 3·13)	2·11 (1·79 to 2·45)
Central Asia	99·88 (86·37 to 115·39)	83·07 (76·31 to 89·72)	72·11 (67·70 to 76·87)	63·17 (59·71 to 66·83)	53·43 (50·03 to 57·09)	53·06 (49·04 to 57·59)	44·80 (40·93 to 49·29)	35·80 (30·91 to 41·33)	27·77 (22·74 to 33·74)	21·33 (15·69 to 28·47)
Armenia	102·69 (75·19 to 137·65)	78·17 (61·15 to 98·31)	63·58 (53·77 to 74·55)	51·35 (44·52 to 58·91)	40·90 (35·33 to 47·01)	38·41 (33·16 to 44·25)	26·92 (22·82 to 31·68)	20·68 (16·61 to 25·36)	14·78 (11·42 to 18·67)	10·52 (7·78 to 13·93)
Azerbaijan	122·78 (90·44 to 159·64)	92·90 (69·53 to 120·93)	86·04 (69·34 to 106·40)	81·25 (67·35 to 94·94)	71·64 (59·65 to 84·30)	69·55 (58·94 to 82·31)	57·25 (48·35 to 68·59)	46·73 (38·01 to 56·52)	35·05 (26·16 to 45·28)	27·09 (20·17 to 35·77)
Georgia	93·64 (73·10 to 117·61)	70·39 (58·12 to 83·93)	52·56 (46·60 to 59·57)	42·35 (37·64 to 47·08)	37·29 (33·58 to 41·67)	37·46 (33·62 to 41·85)	33·23 (29·47 to 37·35)	24·17 (20·26 to 28·41)	17·95 (14·20 to 22·41)	13·39 (10·09 to 17·50)
Kazakhstan	55·95 (43·41 to 70·17)	49·40 (42·40 to 57·14)	43·22 (37·22 to 49·20)	36·83 (32·51 to 41·52)	32·44 (28·62 to 36·21)	35·43 (30·75 to 40·64)	32·28 (26·85 to 38·06)	25·50 (20·46 to 31·37)	17·47 (13·88 to 21·46)	11·60 (8·64 to 15·31)
Kyrgyzstan	125·15 (108·35 to 143·59)	102·71 (92·61 to 113·34)	86·70 (79·93 to 93·68)	73·67 (68·91 to 78·31)	59·48 (55·57 to 63·43)	50·39 (47·02 to 53·99)	44·53 (41·15 to 47·84)	37·24 (34·43 to 40·15)	31·46 (28·38 to 34·66)	25·44 (21·51 to 29·95)
Mongolia	142·30 (121·15 to 164·82)	127·19 (112·18 to 143·24)	115·68 (103·48 to 128·14)	101·27 (91·86 to 110·87)	84·10 (76·27 to 91·64)	67·99 (62·30 to 73·74)	51·49 (46·94 to 56·34)	35·94 (31·97 to 40·01)	25·29 (21·99 to 29·08)	17·99 (14·48 to 22·21)
Tajikistan	192·57 (144·73 to 247·08)	171·66 (139·72 to 207·59)	137·69 (115·83 to 159·83)	111·24 (96·79 to 126·76)	91·91 (78·34 to 106·33)	85·63 (72·67 to 98·66)	69·46 (59·22 to 81·73)	47·23 (39·04 to 56·59)	38·70 (30·51 to 48·20)	32·77 (23·19 to 45·12)
Turkmenistan	166·43 (121·59 to 217·37)	138·08 (100·45 to 182·47)	114·69 (91·58 to 141·35)	96·95 (80·74 to 114·75)	82·92 (70·52 to 95·62)	77·97 (65·81 to 92·52)	69·78 (56·58 to 85·37)	55·97 (41·39 to 72·02)	42·89 (30·56 to 57·85)	31·65 (21·37 to 45·16)
Uzbekistan	85·86 (64·95 to 113·35)	69·36 (56·85 to 82·59)	62·06 (52·89 to 71·75)	55·33 (47·84 to 63·59)	43·72 (37·74 to 50·14)	45·61 (39·87 to 52·50)	37·80 (31·83 to 44·76)	33·20 (25·03 to 43·04)	26·76 (19·53 to 36·00)	20·32 (14·51 to 27·80)
Latin America and Caribbean	109·84 (103·64 to 116·15)	91·59 (87·32 to 96·60)	73·45 (69·90 to 77·24)	60·04 (57·45 to 63·02)	47·62 (45·71 to 49·57)	38·15 (36·24 to 40·27)	29·95 (27·89 to 32·03)	24·96 (23·25 to 26·85)	23·29 (20·23 to 26·95)	16·69 (13·23 to 20·91)
Central Latin America	99·98 (96·43 to 103·50)	82·36 (79·19 to 85·78)	63·45 (60·48 to 66·55)	50·09 (47·62 to 52·59)	39·42 (37·33 to 41·47)	32·31 (30·47 to 34·30)	25·90 (23·94 to 27·77)	21·45 (19·58 to 23·37)	18·05 (15·97 to 20·31)	14·85 (12·48 to 17·74)
Colombia	89·90 (84·00 to 96·16)	71·16 (66·43 to 76·04)	49·96 (46·22 to 54·07)	40·47 (37·04 to 44·26)	30·74 (28·14 to 33·45)	27·66 (25·23 to 30·34)	23·96 (21·48 to 26·62)	21·31 (18·82 to 24·19)	17·78 (15·11 to 21·02)	13·72 (11·24 to 16·75)
Costa Rica	64·81 (60·64 to 69·11)	43·88 (40·60 to 47·17)	28·43 (25·73 to 31·23)	21·24 (18·81 to 23·95)	17·60 (15·32 to 20·18)	15·32 (13·04 to 18·07)	13·66 (11·46 to 16·39)	12·40 (10·30 to 14·96)	11·18 (9·12 to 13·51)	9·48 (7·45 to 12·05)
El Salvador	138·94 (123·40 to 154·51)	109·72 (98·20 to 121·45)	87·92 (79·59 to 97·59)	70·65 (63·88 to 77·74)	48·85 (44·04 to 54·45)	35·63 (31·51 to 39·84)	26·36 (23·38 to 29·89)	18·55 (15·74 to 21·66)	14·02 (11·70 to 16·67)	10·53 (8·62 to 12·88)
Guatemala	174·87 (165·50 to 185·56)	145·82 (138·92 to 152·67)	120·34 (114·55 to 126·55)	96·87 (92·30 to 101·68)	77·00 (73·55 to 80·57)	60·57 (56·89 to 64·10)	47·85 (44·48 to 51·25)	39·16 (35·92 to 42·73)	31·57 (28·17 to 35·57)	24·25 (20·99 to 28·07)
Honduras	129·19 (118·56 to 140·61)	107·01 (99·97 to 114·61)	82·74 (78·22 to 87·59)	64·76 (61·38 to 68·34)	51·20 (48·38 to 54·21)	41·55 (39·01 to 44·25)	33·63 (31·18 to 36·15)	27·81 (25·27 to 30·48)	23·72 (20·97 to 27·08)	19·15 (16·38 to 22·71)
Mexico	97·40 (91·16 to 104·54)	81·23 (75·10 to 87·30)	64·67 (59·76 to 69·64)	50·26 (46·30 to 54·12)	39·54 (36·07 to 42·79)	30·97 (28·08 to 34·01)	24·48 (21·50 to 27·50)	19·94 (17·05 to 23·19)	16·73 (13·68 to 20·11)	13·61 (10·72 to 17·17)
Nicaragua	157·90 (147·76 to 169·00)	125·24 (118·47 to 132·47)	96·20 (91·20 to 101·68)	84·71 (76·25 to 92·94)	57·54 (54·49 to 60·69)	44·99 (42·11 to 47·79)	31·79 (29·50 to 34·13)	23·24 (21·11 to 25·51)	17·74 (15·55 to 20·32)	12·98 (10·90 to 15·66)
Panama	56·50 (52·36 to 60·82)	42·51 (38·83 to 46·36)	34·54 (31·18 to 37·89)	28·45 (25·18 to 31·97)	25·67 (22·59 to 29·19)	23·47 (20·50 to 27·15)	21·23 (18·39 to 24·78)	19·38 (16·58 to 22·66)	17·45 (14·53 to 20·81)	15·13 (12·12 to 18·86)

Appendix Table 11C. Under-5 mortality rates by location, females, 1970, 1975, 1980, 1985, 1990, 1995, 2000, 2005, 2010, 2016										
**Location**	**1970**	**1975**	**1980**	**1985**	**1990**	**1995**	**2000**	**2005**	**2010**	**2016**
Venezuela	65·15 (64·54 to 65·77)	56·39 (55·81 to 56·94)	37·95 (37·56 to 38·36)	29·61 (29·29 to 29·92)	25·69 (25·43 to 25·97)	24·80 (24·52 to 25·09)	19·00 (18·78 to 19·21)	16·20 (16·02 to 16·37)	15·02 (14·91 to 15·14)	14·91 (14·31 to 15·57)
Andean Latin America	186·23 (174·28 to 199·33)	144·15 (138·65 to 150·37)	117·93 (111·83 to 124·07)	95·21 (91·62 to 98·87)	74·21 (71·60 to 77·06)	56·14 (54·21 to 58·11)	40·95 (39·01 to 42·97)	31·65 (29·91 to 33·46)	24·12 (21·65 to 26·77)	17·98 (14·68 to 21·96)
Bolivia	222·15 (208·19 to 236·12)	185·24 (173·94 to 196·47)	153·22 (146·48 to 160·36)	130·35 (126·27 to 134·75)	109·31 (102·57 to 116·02)	87·83 (81·99 to 93·44)	68·30 (63·18 to 73·64)	53·97 (48·92 to 59·10)	41·70 (35·62 to 48·31)	30·27 (24·32 to 37·67)
Ecuador	124·83 (117·78 to 131·67)	99·22 (93·93 to 104·12)	74·59 (71·17 to 78·16)	57·13 (54·64 to 59·90)	45·11 (42·91 to 47·47)	36·60 (34·18 to 39·15)	30·15 (27·61 to 32·96)	24·57 (22·04 to 27·35)	19·57 (17·02 to 22·37)	15·51 (12·98 to 18·59)
Peru	200·01 (177·42 to 224·20)	149·06 (139·27 to 158·91)	125·48 (117·00 to 134·54)	100·89 (94·78 to 106·83)	75·60 (70·92 to 80·69)	53·76 (50·62 to 57·20)	34·90 (32·58 to 37·40)	25·47 (23·70 to 27·36)	19·12 (17·40 to 20·96)	14·37 (11·73 to 17·65)
Caribbean	101·72 (94·99 to 109·06)	91·17 (84·90 to 97·79)	85·30 (78·19 to 93·60)	74·37 (68·48 to 81·25)	64·25 (58·67 to 70·36)	56·28 (51·57 to 62·15)	46·58 (41·83 to 51·82)	44·75 (40·60 to 49·19)	71·77 (44·50 to 106·99)	30·73 (21·87 to 42·58)
Antigua and Barbuda	30·22 (23·87 to 37·65)	24·27 (17·54 to 32·16)	18·14 (12·74 to 24·75)	12·57 (8·89 to 17·21)	12·45 (8·88 to 16·72)	14·23 (10·51 to 18·48)	13·55 (10·38 to 17·72)	11·85 (9·01 to 15·78)	10·19 (7·50 to 14·30)	9·10 (6·26 to 13·22)
The Bahamas	44·08 (29·14 to 63·29)	36·95 (25·43 to 51·33)	30·65 (20·06 to 45·80)	25·28 (15·71 to 37·88)	20·84 (12·72 to 32·07)	16·46 (10·60 to 24·24)	12·86 (8·63 to 18·40)	13·25 (9·32 to 17·93)	11·65 (7·65 to 17·17)	10·26 (5·41 to 19·19)
Barbados	41·13 (28·27 to 58·43)	29·37 (20·17 to 41·80)	22·20 (14·53 to 32·75)	18·75 (11·40 to 28·83)	16·86 (10·23 to 26·62)	17·03 (10·16 to 26·92)	17·86 (10·55 to 28·03)	16·50 (10·90 to 23·69)	14·28 (9·54 to 20·56)	14·27 (8·38 to 22·86)
Belize	89·46 (70·74 to 110·19)	69·85 (56·99 to 85·43)	56·66 (45·86 to 67·63)	42·21 (34·72 to 50·40)	35·49 (29·22 to 42·70)	26·49 (20·93 to 33·44)	25·37 (19·50 to 31·62)	19·98 (14·77 to 27·07)	15·84 (11·19 to 21·75)	13·46 (8·29 to 21·20)
Bermuda	25·12 (20·66 to 30·40)	20·91 (16·75 to 25·47)	20·14 (15·91 to 24·77)	13·62 (10·40 to 17·81)	11·30 (8·62 to 14·63)	8·61 (6·46 to 11·59)	3·93 (2·72 to 5·79)	5·42 (3·94 to 7·31)	4·14 (2·91 to 5·69)	3·33 (2·31 to 4·80)
Cuba	36·80 (36·49 to 37·15)	26·84 (26·49 to 27·23)	21·00 (20·55 to 21·52)	16·53 (15·94 to 17·16)	12·12 (11·78 to 12·50)	9·75 (9·57 to 9·93)	7·51 (7·37 to 7·66)	6·27 (6·13 to 6·42)	5·31 (5·17 to 5·45)	4·65 (4·43 to 4·91)
Dominica	54·62 (39·85 to 71·27)	32·76 (23·25 to 44·25)	20·61 (14·22 to 28·10)	17·16 (11·81 to 23·72)	15·95 (11·11 to 22·18)	16·80 (11·94 to 22·60)	18·14 (13·01 to 24·48)	19·90 (14·68 to 27·14)	23·82 (17·11 to 33·67)	25·05 (16·56 to 36·52)
Dominican Republic	118·03 (106·78 to 129·78)	99·16 (89·59 to 109·07)	80·04 (72·74 to 88·04)	66·59 (60·74 to 72·96)	55·09 (50·15 to 60·30)	42·07 (38·14 to 46·44)	33·35 (30·07 to 36·97)	32·86 (29·31 to 36·66)	28·83 (25·12 to 33·03)	23·94 (18·99 to 30·03)
Grenada	51·56 (31·57 to 78·66)	43·26 (25·35 to 66·34)	33·49 (17·39 to 59·22)	25·19 (12·83 to 46·08)	18·54 (10·51 to 31·09)	15·15 (9·28 to 22·57)	14·39 (9·20 to 21·06)	13·23 (8·18 to 19·99)	13·33 (8·84 to 19·55)	13·99 (7·80 to 24·95)
Guyana	81·39 (75·50 to 87·67)	70·50 (64·05 to 77·01)	58·04 (52·80 to 63·75)	51·47 (47·39 to 55·79)	46·40 (42·85 to 49·95)	39·79 (36·59 to 42·99)	34·54 (31·67 to 37·73)	32·89 (29·88 to 36·14)	28·96 (25·30 to 33·04)	21·61 (17·28 to 26·98)
Haiti	241·88 (215·52 to 269·44)	208·41 (187·54 to 232·52)	186·04 (162·49 to 210·79)	161·87 (143·34 to 183·05)	140·36 (124·17 to 158·40)	119·66 (105·02 to 135·98)	94·66 (81·37 to 108·79)	83·77 (73·42 to 95·14)	154·40 (86·16 to 245·05)	51·64 (35·07 to 73·95)
Jamaica	53·78 (45·19 to 63·13)	44·30 (36·87 to 53·36)	37·72 (29·19 to 47·42)	32·26 (24·75 to 40·80)	28·71 (21·43 to 37·46)	25·27 (18·35 to 34·15)	23·04 (16·51 to 31·04)	20·62 (13·51 to 31·35)	20·39 (12·69 to 31·87)	18·60 (11·74 to 28·56)
Puerto Rico	26·94 (26·27 to 27·64)	19·99 (19·44 to 20·52)	17·72 (17·24 to 18·19)	15·69 (14·94 to 16·59)	14·03 (13·71 to 14·33)	13·39 (13·00 to 13·81)	11·11 (10·70 to 11·51)	9·31 (9·00 to 9·62)	7·86 (7·53 to 8·20)	5·88 (5·26 to 6·59)
Saint Lucia	56·99 (33·17 to 94·45)	38·52 (25·03 to 56·83)	26·46 (16·61 to 39·68)	21·22 (12·04 to 34·23)	18·46 (10·61 to 30·85)	16·95 (10·54 to 26·90)	16·32 (10·27 to 24·76)	18·23 (11·47 to 27·23)	18·52 (11·99 to 27·41)	15·89 (8·66 to 27·45)
Saint Vincent and the Grenadines	82·42 (54·82 to 115·62)	59·04 (38·08 to 84·01)	40·68 (26·62 to 58·20)	28·51 (18·32 to 42·06)	23·54 (15·39 to 34·38)	21·20 (14·23 to 29·42)	20·19 (14·32 to 27·54)	18·73 (13·46 to 25·37)	16·47 (11·64 to 23·05)	13·97 (8·69 to 22·35)
Suriname	63·18 (52·77 to 75·44)	58·54 (49·89 to 68·21)	52·93 (45·85 to 60·33)	48·26 (42·65 to 54·53)	45·09 (39·23 to 51·99)	43·17 (37·19 to 49·78)	41·37 (34·74 to 48·70)	38·52 (31·36 to 46·50)	35·60 (28·33 to 43·69)	31·74 (25·11 to 40·01)
Trinidad and Tobago	47·34 (43·08 to 51·74)	42·37 (38·20 to 47·00)	33·27 (29·50 to 37·17)	26·55 (22·74 to 30·60)	24·52 (20·60 to 29·08)	23·89 (19·75 to 28·40)	24·21 (19·65 to 29·82)	21·52 (16·59 to 27·96)	19·59 (13·59 to 27·07)	18·51 (11·11 to 29·62)
Virgin Islands, U.S.	26·83 (24·41 to 29·14)	23·79 (21·64 to 26·08)	21·22 (19·52 to 22·97)	17·62 (16·42 to 18·83)	16·17 (14·84 to 17·63)	14·61 (13·29 to 16·01)	11·87 (10·75 to 13·18)	10·24 (9·07 to 11·54)	9·15 (7·91 to 10·50)	7·68 (6·34 to 9·32)
Tropical Latin America	103·51 (88·75 to 118·57)	88·38 (78·02 to 99·92)	70·16 (62·23 to 78·82)	58·86 (52·94 to 65·70)	46·42 (41·53 to 51·17)	36·31 (32·10 to 40·96)	28·17 (23·93 to 32·82)	22·94 (18·99 to 27·69)	18·62 (14·98 to 23·49)	15·21 (11·59 to 19·75)
Brazil	102·24 (87·18 to 118·05)	88·13 (77·11 to 100·20)	70·67 (61·96 to 79·76)	58·93 (52·85 to 65·85)	46·50 (41·44 to 51·83)	36·45 (32·62 to 40·68)	27·98 (23·53 to 32·64)	22·48 (18·34 to 27·51)	18·23 (14·59 to 23·17)	14·75 (11·14 to 19·29)
Paraguay	64·40 (57·51 to 71·96)	55·94 (50·82 to 60·76)	46·96 (42·48 to 51·50)	38·92 (35·40 to 42·69)	29·42 (26·73 to 32·14)	25·34 (22·89 to 28·06)	25·48 (22·45 to 28·75)	23·58 (20·20 to 27·48)	20·32 (15·90 to 25·38)	16·62 (12·89 to 21·36)
Southeast Asia, East Asia, and Oceania	120·07 (110·51 to 129·86)	98·15 (91·18 to 105·10)	80·42 (74·87 to 86·52)	66·77 (61·77 to 71·99)	55·38 (51·43 to 59·72)	46·84 (43·49 to 50·52)	38·27 (35·99 to 40·82)	28·26 (26·76 to 29·85)	21·25 (19·79 to 22·81)	15·53 (13·82 to 17·53)
East Asia	115·40 (103·20 to 128·23)	91·94 (82·48 to 101·56)	75·40 (67·31 to 84·21)	62·07 (55·14 to 69·67)	51·29 (45·78 to 57·42)	43·90 (38·71 to 49·46)	35·03 (31·18 to 39·15)	22·30 (20·13 to 24·62)	15·49 (13·98 to 17·07)	11·09 (9·54 to 13·12)
China	117·97 (105·33 to 131·18)	94·23 (84·49 to 104·20)	77·53 (69·03 to 86·74)	63·73 (56·58 to 71·62)	52·64 (46·93 to 58·90)	42·84 (38·29 to 47·91)	33·08 (29·52 to 36·72)	22·10 (19·84 to 24·56)	15·03 (13·52 to 16·64)	10·52 (9·15 to 12·28)
North Korea	55·60 (43·78 to 69·17)	39·32 (30·41 to 48·49)	30·83 (23·94 to 38·72)	28·17 (22·22 to 35·03)	25·70 (19·80 to 32·66)	86·91 (35·71 to 159·09)	82·90 (39·34 to 138·87)	31·32 (24·95 to 39·23)	28·27 (22·39 to 35·60)	23·76 (18·71 to 30·70)
Taiwan (Province of China)	26·26 (25·64 to 26·84)	18·27 (17·80 to 18·72)	14·43 (14·05 to 14·83)	10·11 (9·83 to 10·41)	7·99 (7·72 to 8·26)	8·16 (7·88 to 8·44)	7·91 (7·66 to 8·18)	6·35 (6·14 to 6·59)	4·97 (4·80 to 5·15)	4·32 (3·91 to 4·83)
Southeast Asia	133·78 (129·75 to 138·34)	113·94 (108·22 to 120·08)	90·86 (87·09 to 94·74)	76·13 (72·35 to 79·72)	63·21 (59·93 to 66·73)	51·29 (49·20 to 53·44)	42·09 (40·59 to 43·67)	34·46 (32·61 to 36·59)	26·97 (24·64 to 29·33)	19·52 (16·89 to 22·56)
Cambodia	187·48 (160·40 to 216·72)	292·18 (183·38 to 405·07)	146·51 (131·99 to 161·12)	128·36 (119·20 to 138·26)	109·44 (102·84 to 116·60)	102·74 (96·40 to 109·16)	86·74 (80·58 to 92·90)	60·49 (55·00 to 66·54)	41·03 (36·18 to 46·27)	27·94 (22·89 to 33·84)
Indonesia	165·89 (160·28 to 171·26)	139·58 (132·05 to 147·10)	117·61 (112·01 to 123·27)	98·39 (94·01 to 103·30)	78·84 (75·68 to 82·20)	60·91 (58·01 to 64·20)	47·72 (46·16 to 49·38)	37·40 (34·96 to 40·04)	29·37 (26·41 to 32·65)	21·09 (17·62 to 25·31)
Laos	276·02 (195·66 to 373·48)	245·37 (182·97 to 314·77)	220·12 (177·62 to 265·21)	189·09 (164·56 to 215·50)	170·68 (152·07 to 191·04)	151·11 (134·16 to 170·56)	128·65 (114·12 to 145·47)	102·45 (89·26 to 117·32)	74·63 (62·03 to 88·79)	51·95 (36·54 to 71·67)
Malaysia	50·58 (49·53 to 51·67)	38·83 (37·55 to 40·02)	28·04 (26·65 to 29·43)	20·18 (19·62 to 20·76)	15·14 (14·71 to 15·61)	11·88 (11·58 to 12·20)	8·42 (8·17 to 8·68)	7·27 (7·04 to 7·49)	7·15 (6·80 to 7·53)	5·46 (4·95 to 6·04)
Maldives	383·62 (364·78 to 404·74)	315·79 (307·94 to 323·49)	266·23 (260·59 to 271·90)	182·12 (178·78 to 185·44)	106·49 (103·67 to 109·51)	56·94 (54·91 to 58·80)	28·60 (27·32 to 29·92)	16·17 (15·29 to 17·04)	10·37 (9·65 to 11·16)	6·63 (5·87 to 7·62)
Mauritius	81·88 (79·66 to 84·27)	56·57 (54·91 to 58·18)	35·77 (34·79 to 36·88)	25·88 (25·00 to 26·76)	19·66 (19·01 to 20·39)	17·55 (16·99 to 18·12)	15·63 (15·12 to 16·18)	14·32 (13·69 to 14·96)	13·78 (13·15 to 14·48)	12·23 (11·16 to 13·47)
Myanmar	184·05 (148·01 to 220·41)	145·43 (118·63 to 175·71)	120·13 (97·78 to 143·73)	104·59 (86·01 to 124·85)	93·94 (78·60 to 111·41)	84·84 (70·46 to 103·19)	73·67 (59·93 to 89·10)	57·83 (46·78 to 71·13)	38·42 (29·99 to 48·63)	23·53 (17·72 to 31·81)
Philippines	91·94 (87·39 to 96·81)	81·81 (77·59 to 86·20)	72·89 (69·06 to 76·71)	62·36 (58·81 to 65·92)	50·10 (47·46 to 52·83)	40·03 (37·63 to 42·49)	34·02 (31·51 to 36·49)	30·20 (27·52 to 33·13)	26·34 (23·34 to 29·66)	20·86 (17·69 to 24·61)
Sri Lanka	67·12 (65·62 to 68·65)	65·68 (64·23 to 67·15)	40·79 (39·81 to 41·83)	29·60 (28·80 to 30·46)	30·18 (28·87 to 31·54)	19·24 (18·70 to 19·82)	15·51 (14·89 to 16·13)	16·01 (15·52 to 16·48)	12·71 (11·50 to 14·05)	6·24 (4·79 to 8·10)
Seychelles	66·70 (62·44 to 71·50)	45·30 (42·40 to 48·61)	28·12 (25·90 to 30·25)	20·55 (18·65 to 22·60)	16·71 (14·97 to 18·52)	13·06 (11·75 to 14·45)	12·18 (10·80 to 13·65)	12·12 (10·71 to 13·62)	11·80 (10·39 to 13·39)	10·33 (8·75 to 12·24)
Thailand	83·33 (78·55 to 88·41)	62·33 (57·82 to 67·14)	46·42 (42·59 to 51·09)	34·79 (30·90 to 38·69)	25·51 (22·12 to 29·42)	18·61 (15·71 to 22·20)	14·15 (11·95 to 17·00)	10·67 (9·03 to 12·53)	8·07 (6·77 to 9·54)	5·81 (4·81 to 7·04)
Timor-Leste	297·74 (241·36 to 363·32)	270·72 (231·45 to 315·29)	248·56 (214·93 to 284·89)	178·72 (158·27 to 201·85)	145·97 (128·60 to 164·86)	114·49 (98·76 to 132·07)	94·90 (79·26 to 112·42)	65·51 (51·33 to 81·42)	44·94 (31·56 to 62·49)	31·86 (19·09 to 49·95)
Vietnam	103·23 (81·50 to 129·34)	73·63 (66·32 to 81·72)	58·56 (54·59 to 62·77)	48·37 (45·32 to 51·86)	39·29 (36·81 to 41·89)	30·47 (28·41 to 32·39)	23·08 (21·34 to 24·86)	18·12 (16·33 to 20·04)	14·50 (12·66 to 16·65)	10·82 (9·05 to 13·16)
Oceania	101·27 (81·88 to 121·95)	90·06 (75·14 to 107·14)	81·00 (68·31 to 94·26)	75·16 (63·16 to 88·53)	70·15 (57·62 to 84·46)	64·02 (50·30 to 80·17)	59·31 (43·83 to 79·60)	57·23 (37·65 to 82·40)	51·29 (31·49 to 79·02)	41·02 (24·01 to 66·70)
American Samoa	29·62 (27·05 to 32·23)	22·70 (20·37 to 25·19)	17·44 (15·53 to 19·54)	14·49 (12·96 to 16·24)	14·01 (12·59 to 15·43)	12·99 (11·78 to 14·31)	11·85 (10·79 to 12·96)	10·69 (9·61 to 11·87)	8·99 (7·89 to 10·34)	7·30 (5·89 to 9·06)
Federated States of Micronesia	78·30 (57·26 to 102·31)	66·87 (47·01 to 89·96)	57·40 (42·98 to 76·47)	49·21 (38·22 to 62·82)	41·55 (31·37 to 53·61)	34·20 (25·22 to 44·80)	31·45 (21·13 to 45·71)	20·08 (13·33 to 29·17)	17·94 (11·77 to 26·24)	15·19 (9·90 to 22·61)
Fiji	48·74 (43·21 to 54·36)	46·01 (37·24 to 56·17)	35·60 (27·23 to 46·14)	28·21 (20·46 to 37·31)	24·85 (17·01 to 36·08)	26·14 (18·82 to 35·20)	28·13 (22·41 to 34·93)	31·93 (25·09 to 39·72)	34·42 (25·48 to 45·55)	34·78 (20·80 to 55·60)

Appendix Table 11C. Under-5 mortality rates by location, females, 1970, 1975, 1980, 1985, 1990, 1995, 2000, 2005, 2010, 2016

Location	1970	1975	1980	1985	1990	1995	2000	2005	2010	2016
Guam	28·61 (25·99 to 31·76)	22·86 (21·40 to 24·49)	15·68 (14·53 to 16·82)	12·34 (11·84 to 13·00)	11·35 (10·31 to 12·42)	10·87 (10·19 to 11·57)	10·08 (9·10 to 11·18)	11·15 (10·13 to 12·39)	12·37 (11·56 to 13·27)	12·02 (9·77 to 15·06)
Kiribati	126·82 (95·75 to 161·14)	100·52 (66·73 to 143·53)	87·65 (53·12 to 142·96)	86·90 (55·44 to 130·41)	78·76 (58·02 to 101·96)	69·07 (54·32 to 85·69)	62·66 (49·66 to 78·28)	60·01 (44·52 to 77·55)	55·83 (35·02 to 83·70)	45·75 (24·16 to 79·53)
Marshall Islands	68·18 (36·66 to 114·46)	55·85 (29·80 to 95·97)	46·28 (27·65 to 76·47)	44·27 (29·09 to 64·59)	37·61 (27·26 to 51·99)	31·17 (21·58 to 42·75)	35·77 (21·94 to 54·80)	29·03 (18·18 to 42·45)	23·92 (13·43 to 37·87)	19·21 (10·22 to 32·79)
Northern Mariana Islands	15·64 (8·65 to 26·15)	11·69 (6·55 to 21·00)	9·08 (4·95 to 15·71)	7·37 (3·88 to 12·55)	5·90 (3·06 to 9·82)	5·11 (2·85 to 8·17)	4·01 (2·94 to 5·30)	3·08 (2·20 to 4·09)	1·92 (1·31 to 2·72)	2·21 (1·19 to 3·74)
Papua New Guinea	118·71 (92·94 to 146·09)	105·49 (84·69 to 127·82)	98·54 (81·43 to 117·18)	92·77 (76·42 to 110·72)	85·71 (69·17 to 104·87)	76·59 (58·99 to 97·16)	69·24 (49·48 to 94·63)	65·92 (41·21 to 97·56)	58·40 (34·24 to 91·22)	45·68 (26·21 to 75·23)
Samoa	52·96 (41·53 to 65·36)	41·84 (30·02 to 56·48)	28·61 (18·49 to 43·87)	24·21 (14·28 to 40·06)	19·98 (10·89 to 35·29)	16·53 (8·44 to 30·31)	13·99 (6·92 to 24·72)	12·56 (6·25 to 22·36)	11·12 (5·47 to 19·74)	9·15 (4·49 to 17·17)
Solomon Islands	107·16 (82·09 to 134·01)	86·16 (62·79 to 115·55)	55·88 (39·73 to 80·15)	45·40 (31·92 to 62·31)	40·65 (29·65 to 53·47)	35·03 (26·10 to 45·69)	32·36 (23·72 to 42·89)	32·27 (22·49 to 44·09)	28·42 (19·54 to 40·38)	22·62 (14·96 to 33·22)
Tonga	33·09 (24·36 to 44·09)	29·89 (21·47 to 40·33)	27·66 (18·48 to 39·30)	23·40 (14·62 to 35·60)	20·68 (12·40 to 31·81)	19·94 (11·87 to 31·17)	19·06 (11·29 to 30·55)	17·98 (10·70 to 29·16)	17·28 (9·88 to 29·15)	14·96 (7·90 to 26·33)
Vanuatu	88·92 (61·72 to 124·84)	68·84 (46·20 to 98·82)	52·20 (34·73 to 75·10)	42·48 (30·60 to 57·04)	37·66 (28·83 to 48·22)	34·70 (26·89 to 43·86)	35·94 (27·88 to 46·02)	37·58 (28·99 to 48·52)	33·24 (23·53 to 44·37)	28·20 (19·09 to 42·24)
North Africa and Middle East	200·49 (189·81 to 210·97)	158·19 (151·73 to 164·85)	124·51 (118·99 to 129·86)	98·22 (92·69 to 104·02)	71·99 (69·03 to 75·04)	59·31 (56·95 to 61·70)	48·05 (45·84 to 50·42)	39·12 (36·70 to 41·70)	31·62 (28·72 to 34·61)	26·17 (22·62 to 30·78)
North Africa and Middle East	200·49 (189·81 to 210·97)	158·19 (151·73 to 164·85)	124·51 (118·99 to 129·86)	98·22 (92·69 to 104·02)	71·99 (69·03 to 75·04)	59·31 (56·95 to 61·70)	48·05 (45·84 to 50·42)	39·12 (36·70 to 41·70)	31·62 (28·72 to 34·61)	26·17 (22·62 to 30·78)
Afghanistan	261·19 (207·74 to 312·10)	248·96 (205·75 to 292·71)	241·84 (205·66 to 279·61)	219·88 (188·44 to 255·18)	160·33 (147·78 to 174·16)	150·64 (137·51 to 164·71)	136·41 (125·93 to 147·91)	111·24 (103·74 to 119·22)	85·63 (76·91 to 96·07)	65·17 (55·79 to 76·01)
Algeria	170·38 (150·18 to 190·71)	137·09 (122·32 to 152·83)	100·45 (90·89 to 110·18)	65·71 (59·91 to 71·80)	45·98 (41·97 to 50·25)	37·70 (33·83 to 41·85)	30·63 (27·25 to 34·23)	25·41 (22·38 to 28·82)	21·69 (18·61 to 24·92)	16·64 (12·91 to 21·72)
Bahrain	90·25 (71·06 to 111·59)	53·81 (44·03 to 65·58)	28·00 (25·76 to 30·61)	20·60 (19·14 to 22·17)	21·06 (19·61 to 22·60)	17·13 (15·78 to 18·62)	11·72 (10·74 to 12·81)	12·23 (11·12 to 13·40)	7·84 (7·14 to 8·62)	6·59 (5·22 to 8·21)
Egypt	255·72 (234·80 to 276·24)	214·99 (197·07 to 232·22)	164·66 (148·71 to 180·61)	117·40 (106·98 to 128·92)	85·54 (77·44 to 94·18)	62·38 (55·85 to 69·92)	43·03 (37·86 to 48·67)	33·86 (29·07 to 38·87)	26·54 (22·51 to 31·09)	17·97 (12·63 to 25·23)
Iran	236·27 (195·29 to 279·61)	136·26 (114·06 to 160·68)	102·62 (86·83 to 117·98)	80·08 (67·91 to 93·72)	63·35 (54·19 to 74·30)	46·79 (39·05 to 55·36)	37·39 (30·23 to 45·91)	26·56 (19·89 to 34·92)	19·63 (14·32 to 26·47)	15·81 (11·17 to 21·77)
Iraq	113·16 (98·71 to 127·55)	94·16 (85·54 to 102·69)	75·78 (66·68 to 88·13)	68·16 (56·82 to 85·99)	47·10 (43·91 to 50·32)	46·22 (42·93 to 49·75)	38·47 (35·57 to 41·83)	38·47 (33·37 to 44·15)	31·20 (26·66 to 36·23)	27·04 (21·03 to 35·07)
Jordan	107·81 (97·16 to 118·88)	83·95 (77·07 to 90·94)	57·89 (53·99 to 61·86)	40·92 (37·91 to 44·01)	33·56 (31·17 to 35·87)	29·60 (27·35 to 31·92)	26·35 (24·20 to 28·50)	23·02 (20·92 to 25·13)	18·92 (16·67 to 21·37)	16·85 (13·72 to 20·87)
Kuwait	47·29 (45·12 to 49·43)	43·00 (41·15 to 44·85)	29·67 (28·19 to 31·17)	19·39 (18·44 to 20·48)	14·50 (12·30 to 16·86)	11·94 (11·14 to 12·74)	11·96 (11·08 to 12·83)	10·53 (9·81 to 11·34)	9·99 (9·30 to 10·71)	8·77 (6·84 to 11·10)
Lebanon	68·47 (54·68 to 85·16)	70·78 (53·43 to 91·46)	77·41 (48·85 to 114·86)	45·92 (34·75 to 60·19)	35·46 (29·28 to 42·99)	25·41 (20·59 to 30·89)	18·73 (14·41 to 23·77)	14·03 (10·12 to 18·75)	11·46 (8·42 to 15·17)	9·86 (7·23 to 13·15)
Libya	116·16 (103·28 to 129·74)	84·72 (76·04 to 94·43)	59·96 (53·41 to 66·83)	45·64 (40·80 to 50·89)	37·84 (33·59 to 42·31)	30·05 (25·95 to 34·48)	24·78 (20·31 to 29·91)	19·64 (15·05 to 24·95)	14·40 (10·63 to 19·13)	13·24 (9·14 to 18·97)
Morocco	171·59 (162·16 to 181·90)	144·17 (136·66 to 152·29)	112·28 (105·79 to 119·17)	85·37 (79·63 to 90·74)	65·93 (61·36 to 70·50)	52·06 (47·57 to 56·94)	41·72 (36·68 to 47·25)	33·70 (28·68 to 39·00)	26·68 (21·28 to 33·07)	19·60 (15·52 to 24·78)
Palestine	85·90 (63·23 to 115·94)	77·47 (62·88 to 94·38)	64·69 (55·40 to 75·73)	45·52 (39·47 to 52·67)	41·11 (35·85 to 46·75)	29·29 (24·69 to 33·87)	26·25 (22·24 to 31·15)	23·43 (19·96 to 27·73)	19·82 (16·50 to 23·50)	15·24 (10·80 to 20·84)
Oman	280·77 (248·19 to 316·45)	206·56 (180·05 to 232·73)	136·76 (117·82 to 157·14)	80·26 (67·04 to 94·55)	47·18 (38·59 to 57·94)	26·91 (21·99 to 32·89)	14·73 (12·66 to 17·17)	7·20 (6·82 to 7·55)	7·92 (7·54 to 8·30)	7·45 (6·50 to 8·51)
Qatar	73·68 (50·99 to 101·19)	62·91 (47·68 to 81·76)	34·00 (24·54 to 45·82)	25·63 (19·24 to 32·96)	19·98 (14·44 to 26·98)	17·41 (12·29 to 23·78)	15·13 (10·81 to 20·60)	12·37 (8·80 to 17·17)	10·32 (7·22 to 14·10)	8·46 (5·47 to 12·53)
Saudi Arabia	135·99 (107·58 to 169·08)	99·27 (81·98 to 120·46)	73·03 (60·88 to 87·68)	54·21 (43·67 to 66·05)	42·16 (32·74 to 53·20)	30·23 (22·74 to 39·99)	20·63 (15·15 to 27·96)	14·40 (10·37 to 19·69)	9·93 (6·96 to 14·20)	5·84 (3·94 to 8·86)
Sudan	191·44 (174·76 to 209·42)	183·35 (164·49 to 200·38)	170·08 (152·16 to 188·15)	221·37 (171·60 to 276·90)	128·34 (115·94 to 142·86)	106·99 (94·89 to 119·89)	86·54 (77·50 to 97·15)	70·73 (62·02 to 79·42)	67·59 (58·21 to 77·74)	54·94 (42·73 to 71·16)
Syria	103·88 (95·57 to 112·08)	80·91 (74·40 to 87·80)	60·64 (55·01 to 66·41)	47·84 (43·39 to 52·66)	38·91 (34·73 to 43·32)	25·92 (24·09 to 27·91)	16·12 (15·57 to 16·74)	13·71 (13·23 to 14·33)	13·46 (12·91 to 14·13)	29·24 (12·88 to 53·07)
Tunisia	152·88 (145·76 to 160·42)	113·36 (108·00 to 118·41)	82·32 (76·96 to 87·68)	59·02 (54·81 to 63·16)	43·92 (40·62 to 47·39)	32·32 (29·43 to 35·52)	23·50 (21·11 to 26·20)	18·09 (15·82 to 20·44)	14·63 (12·19 to 17·41)	10·86 (8·78 to 13·46)
Turkey	181·77 (165·56 to 198·22)	152·59 (139·22 to 167·92)	117·82 (105·62 to 130·71)	87·26 (77·50 to 97·35)	66·50 (59·56 to 74·28)	51·83 (45·47 to 58·91)	36·87 (31·40 to 42·81)	26·18 (21·77 to 31·38)	19·80 (14·89 to 26·16)	14·08 (10·02 to 19·43)
United Arab Emirates	108·51 (75·02 to 149·41)	77·05 (50·62 to 115·14)	49·74 (31·76 to 72·23)	29·57 (16·63 to 48·39)	20·02 (10·72 to 32·89)	13·81 (7·15 to 24·40)	9·40 (5·04 to 15·88)	5·79 (4·57 to 7·30)	5·93 (3·41 to 9·52)	5·15 (2·68 to 9·23)
Yemen	306·69 (291·83 to 320·91)	241·58 (227·54 to 255·03)	187·48 (176·42 to 198·29)	151·09 (142·83 to 159·41)	127·49 (120·92 to 134·76)	107·32 (100·75 to 113·79)	87·11 (81·56 to 93·02)	67·21 (61·82 to 73·36)	50·34 (44·55 to 57·45)	42·54 (34·93 to 52·56)
South Asia	207·70 (197·68 to 217·51)	182·84 (173·39 to 192·36)	159·28 (152·02 to 167·17)	136·90 (130·76 to 143·15)	116·43 (110·89 to 122·15)	100·55 (95·72 to 105·33)	86·40 (82·60 to 90·23)	73·34 (68·96 to 77·94)	58·56 (54·00 to 63·40)	40·91 (36·87 to 45·54)
South Asia	207·70 (197·68 to 217·51)	182·84 (173·39 to 192·36)	159·28 (152·02 to 167·17)	136·90 (130·76 to 143·15)	116·43 (110·89 to 122·15)	100·55 (95·72 to 105·33)	86·40 (82·60 to 90·23)	73·34 (68·96 to 77·94)	58·56 (54·00 to 63·40)	40·91 (36·87 to 45·54)
Bangladesh	225·54 (209·75 to 242·26)	205·93 (194·62 to 217·84)	192·54 (183·85 to 201·78)	165·76 (157·44 to 174·35)	134·16 (127·17 to 141·12)	106·97 (101·38 to 112·77)	84·13 (79·44 to 88·78)	65·70 (61·07 to 70·32)	49·00 (44·76 to 53·74)	32·18 (28·47 to 36·65)
Bhutan	258·90 (226·35 to 293·10)	230·80 (205·00 to 258·09)	205·98 (185·89 to 227·96)	171·41 (155·53 to 188·56)	135·60 (123·49 to 148·99)	108·13 (97·02 to 119·32)	85·84 (76·31 to 97·11)	64·78 (57·10 to 74·06)	47·51 (41·28 to 54·41)	31·77 (27·38 to 37·33)
India	202·90 (186·87 to 217·39)	175·88 (165·25 to 187·31)	154·28 (147·80 to 161·03)	133·02 (125·95 to 140·03)	114·18 (107·22 to 120·96)	99·70 (93·32 to 105·82)	86·03 (80·67 to 91·81)	73·47 (67·44 to 79·57)	58·49 (52·31 to 65·68)	40·61 (33·89 to 48·63)
Nepal	243·76 (232·35 to 255·57)	223·90 (215·10 to 232·35)	200·95 (194·40 to 207·49)	170·95 (165·13 to 176·86)	137·46 (133·00 to 141·87)	106·12 (101·53 to 110·77)	81·13 (76·20 to 86·17)	62·25 (57·61 to 66·95)	44·91 (41·26 to 48·99)	28·02 (25·15 to 31·26)
Pakistan	180·67 (173·82 to 187·72)	164·19 (157·52 to 171·55)	148·63 (142·37 to 155·07)	130·77 (125·14 to 136·09)	114·55 (109·38 to 119·88)	102·66 (97·92 to 107·63)	92·72 (88·23 to 97·60)	85·72 (79·26 to 92·46)	71·46 (64·65 to 78·57)	52·90 (47·54 to 58·88)
Sub-Saharan Africa	217·46 (206·43 to 228·91)	200·92 (193·28 to 208·69)	184·03 (179·30 to 189·22)	171·59 (167·43 to 176·06)	161·33 (157·70 to 164·89)	151·52 (147·27 to 155·81)	137·60 (133·99 to 140·99)	116·86 (113·66 to 120·35)	95·84 (91·28 to 100·56)	71·16 (64·96 to 78·39)
Southern Sub-Saharan Africa	116·74 (104·48 to 131·01)	103·76 (96·98 to 111·09)	93·51 (88·04 to 98·80)	80·03 (75·98 to 84·30)	66·97 (63·82 to 70·23)	63·19 (59·58 to 67·26)	69·45 (65·22 to 74·33)	72·03 (65·98 to 78·66)	60·42 (55·37 to 65·53)	42·31 (36·12 to 50·19)
Botswana	94·83 (83·76 to 106·59)	75·71 (67·77 to 83·86)	62·72 (56·07 to 69·33)	52·23 (46·54 to 58·20)	44·11 (39·57 to 48·68)	51·95 (46·98 to 57·32)	57·38 (51·08 to 64·46)	41·96 (35·53 to 48·65)	23·33 (18·86 to 28·67)	12·86 (9·85 to 17·20)
Lesotho	112·22 (102·69 to 122·69)	105·95 (98·29 to 114·53)	96·36 (89·68 to 103·12)	87·63 (81·96 to 93·42)	77·60 (72·48 to 83·42)	76·33 (70·98 to 81·96)	88·90 (82·23 to 96·00)	98·34 (88·53 to 108·66)	79·08 (67·89 to 91·66)	58·54 (46·73 to 73·10)
Namibia	105·74 (83·85 to 132·66)	97·69 (81·73 to 115·57)	94·25 (81·18 to 107·77)	77·07 (67·13 to 89·11)	66·13 (57·90 to 75·55)	54·43 (47·08 to 62·46)	57·71 (49·74 to 67·57)	58·54 (49·83 to 68·30)	48·90 (39·72 to 59·08)	31·85 (20·85 to 46·21)

Appendix Table 11C. Under-5 mortality rates by location, females, 1970, 1975, 1980, 1985, 1990, 1995, 2000, 2005, 2010, 2016										
Location	1970	1975	1980	1985	1990	1995	2000	2005	2010	2016
South Africa	126·53 (110·94 to 145·36)	112·94 (104·19 to 122·49)	101·82 (94·02 to 109·58)	88·41 (82·58 to 94·58)	72·78 (67·63 to 77·81)	65·14 (59·30 to 71·45)	71·76 (65·62 to 78·63)	73·41 (64·71 to 83·32)	58·76 (51·59 to 65·53)	40·43 (31·37 to 51·42)
Swaziland	181·04 (135·11 to 237·87)	142·04 (114·50 to 172·59)	110·10 (95·95 to 125·11)	82·18 (73·85 to 91·52)	59·91 (54·21 to 66·22)	62·10 (56·77 to 68·00)	83·76 (77·00 to 90·95)	94·08 (84·73 to 103·80)	66·47 (57·93 to 75·83)	43·54 (34·43 to 53·88)
Zimbabwe	99·36 (91·14 to 107·26)	87·95 (80·58 to 95·55)	79·62 (73·35 to 85·94)	64·77 (60·47 to 69·57)	57·86 (54·20 to 61·81)	64·14 (60·11 to 68·26)	69·27 (67·00 to 71·62)	74·98 (69·39 to 80·92)	70·79 (64·18 to 77·68)	50·76 (44·68 to 58·83)
Western Sub-Saharan Africa	253·13 (240·22 to 266·02)	230·82 (220·57 to 241·47)	209·30 (202·25 to 216·70)	194·15 (187·83 to 200·54)	184·01 (178·82 to 189·32)	174·21 (168·67 to 180·04)	159·88 (155·08 to 164·64)	139·91 (134·44 to 145·20)	117·31 (110·71 to 124·43)	91·02 (82·97 to 100·13)
Benin	268·92 (251·82 to 285·06)	235·03 (221·02 to 248·37)	205·87 (194·76 to 216·65)	183·61 (175·92 to 191·63)	168·39 (160·61 to 176·43)	151·95 (142·45 to 161·87)	131·89 (122·06 to 142·01)	109·30 (99·15 to 120·10)	89·38 (78·57 to 102·43)	67·37 (56·09 to 81·53)
Burkina Faso	297·27 (281·10 to 314·72)	274·83 (262·93 to 287·19)	246·45 (238·39 to 254·64)	223·14 (214·68 to 231·55)	202·34 (192·10 to 212·27)	188·47 (179·54 to 197·12)	168·75 (162·94 to 174·68)	145·90 (137·55 to 154·23)	124·64 (114·64 to 135·31)	99·97 (89·54 to 112·98)
Cameroon	193·66 (183·45 to 204·49)	174·64 (165·10 to 183·74)	157·51 (149·68 to 165·23)	138·49 (130·43 to 146·48)	129·03 (120·91 to 137·40)	132·31 (124·65 to 140·39)	128·91 (120·54 to 138·81)	118·15 (106·86 to 130·33)	103·17 (89·13 to 119·14)	82·72 (66·71 to 102·19)
Cape Verde	173·40 (167·58 to 179·56)	129·52 (124·51 to 134·80)	87·03 (83·30 to 91·50)	85·65 (82·03 to 89·50)	59·98 (57·26 to 62·73)	66·02 (52·85 to 79·93)	40·36 (35·07 to 46·27)	29·00 (25·45 to 33·22)	19·91 (18·28 to 21·75)	14·52 (12·68 to 16·55)
Chad	243·34 (222·97 to 264·03)	226·70 (211·45 to 242·49)	219·23 (205·33 to 233·43)	210·91 (198·90 to 223·35)	191·05 (181·42 to 200·15)	179·53 (170·62 to 188·25)	176·09 (167·00 to 185·57)	157·63 (147·10 to 168·05)	134·39 (122·71 to 147·82)	107·85 (95·70 to 122·91)
Cote d'Ivoire	199·91 (189·15 to 210·37)	169·66 (161·15 to 178·07)	144·31 (136·84 to 151·94)	135·93 (129·25 to 142·84)	132·86 (126·50 to 139·66)	131·78 (123·98 to 139·99)	121·86 (113·72 to 130·52)	110·73 (101·61 to 120·55)	94·98 (84·92 to 105·41)	74·21 (64·71 to 85·39)
The Gambia	165·32 (144·17 to 187·66)	146·98 (129·46 to 165·31)	128·94 (115·84 to 142·73)	113·47 (104·45 to 122·09)	102·33 (96·63 to 107·95)	90·76 (84·60 to 97·12)	79·57 (73·19 to 86·23)	67·63 (61·17 to 74·98)	56·02 (49·67 to 63·14)	44·84 (38·18 to 53·56)
Ghana	160·38 (152·12 to 169·31)	146·32 (139·26 to 154·07)	138·20 (131·24 to 145·67)	130·47 (124·84 to 136·79)	114·03 (110·35 to 118·20)	101·84 (96·46 to 107·20)	92·53 (87·56 to 97·95)	81·60 (76·36 to 86·84)	66·47 (60·19 to 72·92)	45·79 (38·45 to 55·15)
Guinea	306·30 (274·88 to 337·01)	292·92 (271·79 to 314·82)	268·51 (254·19 to 282·94)	244·71 (232·09 to 257·60)	217·11 (204·15 to 229·50)	191·77 (179·89 to 203·16)	163·85 (154·30 to 173·44)	141·45 (130·67 to 152·01)	118·81 (106·87 to 132·97)	91·21 (77·69 to 108·58)
Guinea-Bissau	274·16 (240·66 to 311·46)	243·78 (216·56 to 273·68)	223·39 (201·58 to 246·24)	210·52 (194·46 to 226·99)	188·40 (177·57 to 200·60)	165·19 (154·61 to 175·66)	142·63 (132·23 to 153·28)	124·93 (113·17 to 137·37)	92·00 (82·27 to 102·04)	64·36 (55·46 to 74·11)
Liberia	272·89 (253·70 to 292·11)	248·77 (231·26 to 267·44)	229·17 (213·94 to 244·65)	215·55 (205·56 to 226·02)	207·88 (198·71 to 217·23)	203·83 (193·36 to 214·91)	162·92 (154·34 to 172·07)	116·32 (110·75 to 121·62)	88·94 (82·77 to 95·62)	61·33 (54·35 to 70·07)
Mali	344·85 (327·58 to 363·46)	317·20 (303·64 to 331·44)	280·07 (267·24 to 293·10)	253·69 (242·30 to 265·05)	227·37 (216·98 to 238·78)	205·80 (194·34 to 218·38)	183·20 (173·44 to 193·27)	156·33 (147·38 to 165·18)	131·34 (120·37 to 143·90)	108·59 (92·92 to 128·55)
Mauritania	162·62 (154·62 to 171·47)	153·68 (146·84 to 160·29)	135·34 (128·45 to 141·80)	118·09 (111·42 to 125·00)	103·74 (97·48 to 110·08)	93·77 (88·23 to 99·34)	85·63 (79·89 to 91·68)	76·79 (70·29 to 83·19)	60·64 (54·46 to 67·07)	44·98 (39·29 to 51·61)
Niger	320·11 (298·56 to 344·18)	323·72 (306·39 to 342·35)	314·76 (301·11 to 328·77)	305·58 (292·13 to 319·33)	288·83 (274·80 to 302·74)	262·12 (248·16 to 276·22)	220·12 (208·14 to 232·55)	174·46 (160·89 to 188·37)	136·97 (121·75 to 153·61)	104·87 (89·44 to 124·54)
Nigeria	254·45 (228·67 to 280·41)	229·85 (212·23 to 248·66)	208·58 (198·01 to 219·16)	196·48 (186·34 to 206·61)	194·92 (184·47 to 205·03)	186·87 (176·87 to 196·67)	174·20 (165·13 to 184·44)	155·24 (144·83 to 165·37)	131·72 (120·18 to 143·62)	102·71 (87·79 to 119·24)
Sao Tome and Principe	143·43 (134·41 to 152·75)	112·35 (103·52 to 122·47)	105·96 (100·73 to 111·44)	116·04 (109·08 to 123·55)	98·19 (90·12 to 106·97)	84·41 (77·24 to 92·58)	67·06 (62·01 to 72·61)	53·36 (47·38 to 59·89)	35·95 (32·00 to 40·49)	25·68 (22·50 to 29·68)
Senegal	264·76 (251·52 to 278·35)	235·64 (222·40 to 250·10)	195·16 (185·04 to 206·25)	160·45 (151·31 to 169·95)	136·07 (128·41 to 144·01)	124·78 (119·11 to 130·31)	105·95 (101·99 to 110·10)	83·82 (78·37 to 90·25)	61·72 (56·88 to 67·07)	43·52 (38·19 to 49·83)
Sierra Leone	294·46 (259·58 to 334·95)	275·40 (247·01 to 305·54)	264·46 (242·68 to 286·38)	251·57 (237·36 to 266·99)	234·33 (222·11 to 247·39)	220·85 (209·04 to 232·44)	205·12 (194·01 to 216·32)	177·41 (165·11 to 188·94)	146·14 (133·28 to 158·77)	109·28 (95·85 to 124·01)
Togo	203·77 (190·33 to 217·67)	181·98 (171·41 to 192·76)	162·06 (152·05 to 172·20)	148·58 (139·55 to 157·88)	135·88 (128·38 to 143·19)	125·41 (118·76 to 132·17)	113·56 (107·36 to 119·97)	101·33 (94·36 to 108·58)	84·83 (77·44 to 93·30)	62·65 (55·38 to 71·82)
Eastern Sub-Saharan Africa	201·41 (191·77 to 210·60)	190·61 (182·69 to 198·42)	177·22 (170·58 to 184·22)	169·74 (164·76 to 175·05)	156·93 (152·47 to 161·32)	143·93 (139·33 to 148·59)	124·67 (119·89 to 129·68)	98·98 (94·68 to 103·62)	77·36 (72·57 to 82·31)	54·67 (48·28 to 62·80)
Burundi	234·55 (201·24 to 267·03)	226·91 (201·37 to 256·40)	206·07 (182·61 to 230·28)	176·99 (159·36 to 195·55)	168·76 (152·00 to 186·67)	165·91 (149·21 to 184·22)	156·06 (138·87 to 174·05)	119·79 (105·37 to 135·76)	91·28 (76·53 to 107·06)	75·25 (53·99 to 106·04)
Comoros	177·57 (154·22 to 202·70)	164·44 (145·06 to 184·23)	150·97 (132·47 to 169·95)	130·10 (113·70 to 146·94)	112·75 (100·66 to 125·25)	100·45 (89·22 to 113·35)	93·77 (81·54 to 107·39)	77·40 (66·64 to 89·78)	65·74 (56·36 to 75·73)	49·90 (40·65 to 62·03)
Djibouti	125·53 (107·63 to 145·11)	116·60 (103·88 to 129·99)	111·21 (100·80 to 122·24)	110·63 (101·43 to 120·08)	106·78 (98·54 to 114·86)	103·02 (95·82 to 111·00)	94·90 (86·65 to 103·59)	78·38 (69·21 to 89·17)	63·43 (54·21 to 74·72)	43·55 (36·36 to 52·98)
Eritrea	204·61 (180·04 to 231·18)	184·63 (166·94 to 202·35)	169·57 (154·02 to 184·12)	154·91 (141·93 to 169·28)	135·93 (124·35 to 148·21)	105·13 (95·37 to 115·89)	85·29 (75·03 to 96·24)	70·72 (59·60 to 82·67)	61·11 (49·77 to 74·25)	45·72 (36·54 to 58·20)
Ethiopia	204·56 (191·81 to 217·52)	205·07 (191·30 to 218·47)	191·72 (179·46 to 204·36)	180·13 (170·63 to 189·91)	171·36 (161·40 to 181·62)	150·68 (140·06 to 161·19)	129·82 (120·83 to 140·01)	97·86 (90·42 to 106·06)	68·16 (62·25 to 74·67)	38·97 (34·95 to 44·29)
Kenya	133·94 (126·65 to 141·67)	117·88 (111·50 to 125·48)	102·49 (95·84 to 109·20)	93·12 (87·82 to 99·38)	88·27 (83·29 to 93·71)	90·53 (85·14 to 95·95)	83·60 (77·98 to 89·46)	68·45 (62·87 to 74·94)	51·88 (46·09 to 57·88)	39·48 (33·62 to 46·27)
Madagascar	161·23 (142·71 to 182·51)	154·98 (137·74 to 174·39)	154·87 (139·00 to 171·29)	160·62 (144·19 to 176·65)	140·82 (128·40 to 153·64)	128·19 (116·29 to 141·29)	111·11 (99·43 to 122·84)	96·12 (84·23 to 108·69)	91·43 (78·64 to 105·22)	75·44 (59·85 to 96·09)
Malawi	310·50 (280·97 to 344·47)	276·42 (251·46 to 301·07)	242·63 (224·23 to 262·80)	227·79 (211·99 to 245·47)	212·01 (197·65 to 227·61)	184·26 (171·37 to 198·72)	157·21 (144·86 to 170·52)	117·98 (107·26 to 128·44)	95·19 (84·65 to 106·02)	64·73 (52·42 to 81·20)
Mozambique	241·01 (213·00 to 268·54)	225·37 (206·09 to 244·62)	217·07 (203·59 to 230·30)	243·68 (226·85 to 260·34)	201·97 (193·35 to 210·78)	182·95 (174·74 to 191·16)	152·48 (144·57 to 160·55)	122·56 (114·49 to 130·47)	97·54 (88·46 to 107·15)	71·00 (61·70 to 82·83)
Rwanda	232·57 (211·65 to 253·14)	228·31 (208·78 to 248·72)	198·99 (181·77 to 216·96)	163·30 (149·44 to 178·24)	160·82 (148·34 to 174·96)	176·32 (159·03 to 193·25)	147·89 (134·39 to 162·01)	100·14 (89·17 to 110·93)	62·35 (54·25 to 70·21)	45·24 (37·79 to 55·12)
Somalia	167·17 (146·70 to 191·59)	196·58 (172·72 to 223·10)	162·13 (149·62 to 177·18)	170·24 (159·92 to 180·47)	159·52 (151·47 to 168·06)	152·33 (143·90 to 161·08)	141·96 (131·35 to 153·93)	121·94 (110·87 to 133·98)	135·95 (120·30 to 155·03)	89·53 (81·00 to 100·36)
South Sudan	237·48 (181·12 to 305·17)	226·51 (178·98 to 283·29)	216·43 (178·73 to 256·27)	206·57 (181·01 to 233·72)	187·23 (169·35 to 207·53)	159·80 (145·33 to 173·92)	134·56 (123·15 to 146·34)	115·48 (103·24 to 127·53)	100·64 (85·78 to 116·41)	87·25 (68·50 to 109·41)
Tanzania	203·99 (190·01 to 217·45)	185·59 (176·09 to 195·59)	170·69 (162·92 to 179·35)	160·49 (153·81 to 167·40)	149·92 (144·24 to 155·67)	138·86 (132·70 to 144·92)	119·82 (113·94 to 125·96)	96·50 (90·52 to 102·86)	73·62 (66·97 to 80·39)	52·55 (45·83 to 61·10)
Uganda	177·69 (164·89 to 190·02)	169·91 (160·19 to 179·95)	172·83 (162·88 to 182·78)	164·84 (157·09 to 173·51)	152·81 (146·22 to 159·13)	140·79 (134·54 to 147·42)	121·01 (115·67 to 126·49)	101·25 (95·84 to 106·68)	80·92 (74·95 to 87·09)	55·38 (49·27 to 63·06)

Appendix Table 11C. Under-5 mortality rates by location, females, 1970, 1975, 1980, 1985, 1990, 1995, 2000, 2005, 2010, 2016										
**Location**	**1970**	**1975**	**1980**	**1985**	**1990**	**1995**	**2000**	**2005**	**2010**	**2016**
Zambia	179·10<br>(157·73 to 204·27)	160·08<br>(141·71 to 179·55)	159·09<br>(143·06 to 177·55)	163·32<br>(148·41 to 180·00)	162·63<br>(150·08 to 175·72)	152·30<br>(137·25 to 167·47)	131·82<br>(117·76 to 145·57)	102·33<br>(90·60 to 114·83)	74·22<br>(64·15 to 85·21)	51·96<br>(41·35 to 64·13)
Central Sub-Saharan Africa	204·71<br>(168·62 to 244·67)	191·37<br>(160·10 to 224·31)	177·00<br>(155·32 to 200·34)	158·34<br>(142·52 to 175·43)	159·29<br>(145·57 to 172·89)	152·78<br>(139·39 to 167·63)	142·60<br>(128·62 to 157·34)	120·00<br>(108·05 to 132·84)	98·85<br>(85·64 to 112·95)	69·69<br>(52·79 to 90·59)
Angola	194·34<br>(149·31 to 246·70)	187·43<br>(146·91 to 230·92)	182·68<br>(148·97 to 218·35)	175·59<br>(151·67 to 200·11)	178·98<br>(157·70 to 201·92)	155·78<br>(137·06 to 175·16)	143·28<br>(125·30 to 163·30)	115·08<br>(101·04 to 132·00)	86·97<br>(72·72 to 104·19)	49·80<br>(37·02 to 65·74)
Central African Republic	195·23<br>(161·07 to 233·17)	194·29<br>(162·36 to 230·35)	179·93<br>(152·72 to 213·32)	171·51<br>(144·42 to 199·96)	169·05<br>(145·04 to 195·26)	167·60<br>(142·14 to 198·44)	169·73<br>(141·36 to 202·51)	160·91<br>(130·56 to 196·69)	146·73<br>(112·81 to 184·40)	122·91<br>(88·99 to 167·58)
Congo	182·03<br>(144·92 to 219·71)	156·41<br>(121·86 to 193·15)	129·00<br>(106·71 to 153·35)	101·14<br>(87·48 to 115·63)	91·69<br>(80·98 to 104·28)	94·50<br>(84·39 to 105·87)	97·85<br>(86·38 to 110·08)	81·79<br>(71·03 to 93·57)	62·89<br>(53·47 to 73·21)	47·82<br>(37·98 to 62·39)
Democratic Republic of the Congo	210·26<br>(165·32 to 259·81)	195·39<br>(156·83 to 238·72)	179·19<br>(149·45 to 211·60)	156·23<br>(136·21 to 179·56)	157·24<br>(138·09 to 177·71)	155·59<br>(137·01 to 177·80)	144·89<br>(124·71 to 167·47)	122·71<br>(106·03 to 141·44)	103·29<br>(85·24 to 123·08)	75·23<br>(54·36 to 101·14)
Equatorial Guinea	230·87<br>(176·10 to 290·75)	208·88<br>(171·09 to 251·95)	180·33<br>(151·48 to 210·69)	172·68<br>(148·57 to 200·10)	182·65<br>(156·37 to 212·29)	190·15<br>(161·07 to 223·00)	136·78<br>(111·15 to 164·74)	103·97<br>(81·26 to 130·74)	80·42<br>(60·16 to 106·84)	51·46<br>(36·35 to 73·93)
Gabon	155·15<br>(132·16 to 179·13)	126·80<br>(112·64 to 141·22)	102·77<br>(93·67 to 112·63)	85·94<br>(79·02 to 92·80)	75·84<br>(69·50 to 82·42)	66·51<br>(60·88 to 72·49)	61·13<br>(55·73 to 66·83)	56·79<br>(51·02 to 62·86)	47·94<br>(41·85 to 55·01)	37·02<br>(30·78 to 45·45)

Appendix Table 12A. Under-5 death numbers by location, both sexes combined, 1970, 1975, 1980, 1985, 1990, 1995, 2000, 2005, 2010, 2016										
**Location**	**1970**	**1975**	**1980**	**1985**	**1990**	**1995**	**2000**	**2005**	**2010**	**2016**
Global	16,370·68 (16,056·12 to 16,698·02)	14,625·48 (14,367·04 to 14,913·05)	13,262·11 (13,011·64 to 13,507·42)	12,263·01 (12,039·25 to 12,485·18)	11,051·04 (10,832·93 to 11,257·59)	9,874·87 (9,659·99 to 10,084·05)	8,747·57 (8,524·34 to 8,950·23)	7,674·69 (7,477·90 to 7,866·89)	6,630·59 (6,422·02 to 6,842·72)	4,999·79 (4,776·15 to 5,236·95)
High SDI	1,414·05 (1,318·40 to 1,517·92)	1,134·65 (1,065·11 to 1,201·40)	949·40 (887·01 to 1,012·26)	785·33 (738·38 to 834·98)	618·13 (583·77 to 655·79)	454·55 (428·67 to 483·80)	342·01 (320·91 to 368·32)	264·64 (239·54 to 300·54)	216·02 (191·83 to 250·78)	163·17 (142·99 to 196·03)
High-middle SDI	352·84 (344·30 to 362·93)	250·71 (243·97 to 258·47)	191·80 (186·44 to 198·16)	146·37 (143·39 to 149·79)	123·91 (121·84 to 126·84)	93·65 (92·28 to 95·20)	76·72 (76·34 to 77·12)	68·53 (68·18 to 68·89)	60·00 (59·69 to 60·34)	53·34 (51·71 to 55·18)
Middle SDI	6,483·59 (6,274·02 to 6,704·36)	6,110·86 (5,920·80 to 6,298·41)	5,790·72 (5,619·04 to 5,950·07)	5,557·71 (5,392·74 to 5,711·79)	5,106·99 (4,962·34 to 5,244·88)	4,697·15 (4,538·53 to 4,858·07)	4,220·56 (4,064·83 to 4,367·81)	3,715·53 (3,573·25 to 3,864·34)	3,174·88 (3,016·58 to 3,340·48)	2,275·93 (2,116·11 to 2,443·54)
Low-middle SDI	2,432·00 (2,290·70 to 2,574·74)	2,524·32 (2,389·66 to 2,649·43)	2,580·35 (2,446·81 to 2,705·03)	2,614·88 (2,484·23 to 2,752·85)	2,608·49 (2,479·03 to 2,730·48)	2,656·20 (2,500·39 to 2,787·50)	2,625·74 (2,478·97 to 2,751·11)	2,470·87 (2,345·41 to 2,591·81)	2,294·54 (2,151·84 to 2,431·89)	1,904·74 (1,767·18 to 2,048·42)
Low SDI	5,679·60 (5,509·67 to 5,850·01)	4,598·48 (4,478·62 to 4,728·93)	3,744·08 (3,632·65 to 3,858·41)	3,153·42 (3,067·88 to 3,234·87)	2,588·45 (2,523·52 to 2,655·65)	1,968·92 (1,922·10 to 2,016·55)	1,478·69 (1,444·79 to 1,511·80)	1,151·53 (1,120·85 to 1,180·85)	882·20 (853·21 to 910·28)	600·32 (567·87 to 636·34)
High-income	408·91 (400·29 to 418·62)	294·25 (287·56 to 301·89)	211·98 (206·60 to 218·40)	158·77 (155·73 to 162·21)	138·51 (136·43 to 141·51)	105·58 (104·20 to 107·15)	89·17 (88·20 to 90·17)	79·96 (79·17 to 80·79)	70·71 (70·01 to 71·40)	62·78 (60·73 to 64·76)
High-income North America	94·67 (93·53 to 95·85)	65·81 (65·11 to 66·49)	58·92 (58·58 to 59·26)	51·32 (51·04 to 51·62)	49·06 (48·79 to 49·37)	38·86 (38·61 to 39·08)	35·19 (34·98 to 35·40)	35·51 (35·29 to 35·74)	30·98 (30·82 to 31·17)	29·64 (28·86 to 30·37)
Canada	8·20 (8·02 to 8·36)	6·03 (5·90 to 6·17)	4·59 (4·48 to 4·70)	3·64 (3·54 to 3·73)	3·31 (3·22 to 3·41)	2·72 (2·65 to 2·80)	2·06 (2·00 to 2·12)	2·09 (2·01 to 2·17)	2·14 (2·07 to 2·20)	2·15 (1·78 to 2·58)
Greenland	0·07 (0·07 to 0·08)	0·04 (0·04 to 0·05)	0·04 (0·04 to 0·04)	0·04 (0·04 to 0·04)	0·04 (0·04 to 0·05)	0·03 (0·02 to 0·03)	0·02 (0·01 to 0·02)	0·01 (0·01 to 0·02)	0·01 (0·01 to 0·01)	0·01 (0·01 to 0·01)
United States	86·34 (85·21 to 87·53)	59·70 (59·02 to 60·40)	54·26 (53·95 to 54·58)	47·61 (47·35 to 47·89)	45·69 (45·43 to 45·97)	36·09 (35·88 to 36·30)	33·09 (32·90 to 33·31)	33·39 (33·18 to 33·61)	28·82 (28·66 to 29·00)	27·47 (26·81 to 28·08)
Australasia	6·89 (6·76 to 7·01)	5·17 (5·07 to 5·27)	3·76 (3·68 to 3·84)	3·50 (3·42 to 3·58)	3·04 (2·96 to 3·11)	2·29 (2·24 to 2·35)	2·00 (1·95 to 2·05)	1·89 (1·83 to 1·94)	1·82 (1·77 to 1·88)	1·49 (1·32 to 1·67)
Australia	5·58 (5·46 to 5·70)	4·11 (4·01 to 4·21)	2·96 (2·89 to 3·04)	2·79 (2·72 to 2·87)	2·40 (2·33 to 2·47)	1·80 (1·74 to 1·85)	1·57 (1·52 to 1·62)	1·50 (1·45 to 1·55)	1·43 (1·38 to 1·48)	1·17 (1·02 to 1·35)
New Zealand	1·30 (1·27 to 1·34)	1·06 (1·03 to 1·09)	0·80 (0·77 to 0·82)	0·70 (0·68 to 0·73)	0·64 (0·62 to 0·66)	0·50 (0·48 to 0·52)	0·43 (0·41 to 0·45)	0·39 (0·37 to 0·40)	0·39 (0·38 to 0·41)	0·32 (0·27 to 0·38)
High-income Asia Pacific	89·50 (81·04 to 99·50)	61·19 (54·54 to 68·71)	38·53 (33·37 to 44·96)	23·16 (20·19 to 26·59)	16·47 (14·47 to 19·28)	13·04 (11·76 to 14·47)	9·63 (9·49 to 9·78)	6·77 (6·66 to 6·88)	5·47 (5·38 to 5·56)	4·13 (3·75 to 4·55)
Brunei	0·25 (0·24 to 0·26)	0·19 (0·18 to 0·20)	0·13 (0·12 to 0·14)	0·09 (0·09 to 0·10)	0·08 (0·07 to 0·09)	0·07 (0·07 to 0·08)	0·07 (0·07 to 0·08)	0·06 (0·06 to 0·07)	0·06 (0·05 to 0·06)	0·06 (0·05 to 0·07)
Japan	33·32 (32·41 to 34·17)	25·64 (25·00 to 26·25)	16·26 (16·09 to 16·42)	10·89 (10·77 to 11·01)	7·99 (7·90 to 8·09)	7·13 (6·95 to 7·33)	5·35 (5·28 to 5·43)	4·16 (4·09 to 4·22)	3·44 (3·38 to 3·49)	2·55 (2·45 to 2·67)
Singapore	1·26 (1·22 to 1·30)	0·77 (0·74 to 0·80)	0·60 (0·58 to 0·63)	0·45 (0·43 to 0·47)	0·36 (0·35 to 0·38)	0·25 (0·23 to 0·26)	0·17 (0·16 to 0·18)	0·11 (0·10 to 0·12)	0·10 (0·09 to 0·10)	0·08 (0·06 to 0·10)
South Korea	54·67 (46·43 to 64·39)	34·60 (27·94 to 41·81)	21·54 (16·42 to 28·03)	11·72 (8·78 to 15·10)	8·03 (6·05 to 10·87)	5·59 (4·38 to 7·02)	4·04 (3·92 to 4·16)	2·44 (2·36 to 2·53)	1·87 (1·80 to 1·95)	1·43 (1·08 to 1·84)
Western Europe	152·52 (151·59 to 153·48)	104·42 (103·77 to 105·10)	71·56 (71·02 to 72·12)	51·24 (50·88 to 51·63)	42·75 (42·38 to 43·11)	29·85 (29·55 to 30·13)	24·77 (24·52 to 25·01)	21·34 (21·12 to 21·56)	19·46 (19·27 to 19·66)	16·35 (15·12 to 17·67)
Andorra	<0·01 (<0·01 to <0·01)	<0·01 (<0·01 to <0·01)	<0·01 (<0·01 to <0·01)	<0·01 (<0·01 to <0·01)	<0·01 (<0·01 to <0·01)	<0·01 (<0·01 to <0·01)	<0·01 (<0·01 to <0·01)	<0·01 (<0·01 to <0·01)	<0·01 (<0·01 to <0·01)	<0·01 (<0·01 to <0·01)
Austria	3·35 (3·25 to 3·45)	2·23 (2·15 to 2·30)	1·52 (1·46 to 1·59)	1·15 (1·10 to 1·20)	0·87 (0·83 to 0·92)	0·61 (0·58 to 0·65)	0·45 (0·42 to 0·48)	0·39 (0·36 to 0·41)	0·36 (0·33 to 0·38)	0·28 (0·21 to 0·36)
Belgium	3·47 (3·41 to 3·53)	2·30 (2·26 to 2·35)	1·81 (1·76 to 1·85)	1·36 (1·32 to 1·40)	1·23 (1·20 to 1·26)	0·89 (0·87 to 0·92)	0·68 (0·66 to 0·70)	0·59 (0·57 to 0·61)	0·57 (0·55 to 0·59)	0·51 (0·43 to 0·61)
Cyprus	0·55 (0·49 to 0·63)	0·33 (0·31 to 0·34)	0·25 (0·24 to 0·26)	0·19 (0·18 to 0·19)	0·15 (0·15 to 0·16)	0·11 (0·10 to 0·11)	0·07 (0·06 to 0·07)	0·05 (0·05 to 0·06)	0·04 (0·04 to 0·05)	0·03 (0·03 to 0·04)
Denmark	1·24 (1·19 to 1·29)	0·88 (0·84 to 0·92)	0·59 (0·56 to 0·62)	0·51 (0·49 to 0·54)	0·56 (0·54 to 0·59)	0·46 (0·43 to 0·48)	0·37 (0·35 to 0·40)	0·32 (0·30 to 0·34)	0·26 (0·24 to 0·27)	0·24 (0·19 to 0·30)
Finland	1·05 (1·00 to 1·11)	0·82 (0·77 to 0·87)	0·56 (0·53 to 0·60)	0·46 (0·43 to 0·49)	0·44 (0·41 to 0·48)	0·32 (0·29 to 0·34)	0·24 (0·22 to 0·27)	0·23 (0·21 to 0·25)	0·18 (0·16 to 0·20)	0·13 (0·10 to 0·17)
France	15·61 (15·35 to 15·88)	12·33 (12·11 to 12·55)	9·78 (9·58 to 10·01)	7·70 (7·53 to 7·86)	6·76 (6·60 to 6·92)	4·45 (4·32 to 4·58)	4·13 (4·00 to 4·26)	3·44 (3·33 to 3·55)	3·34 (3·24 to 3·45)	3·03 (2·36 to 3·88)
Germany	27·61 (27·20 to 28·02)	17·43 (17·08 to 17·78)	12·84 (12·57 to 13·12)	9·07 (8·87 to 9·29)	7·83 (7·63 to 8·03)	5·09 (4·95 to 5·23)	4·10 (3·98 to 4·22)	3·29 (3·19 to 3·40)	2·81 (2·72 to 2·91)	2·53 (2·00 to 3·24)
Greece	4·87 (4·76 to 4·99)	3·80 (3·71 to 3·89)	2·90 (2·83 to 2·98)	1·79 (1·73 to 1·84)	1·10 (1·06 to 1·15)	0·89 (0·86 to 0·93)	0·65 (0·62 to 0·68)	0·50 (0·48 to 0·52)	0·46 (0·44 to 0·48)	0·36 (0·28 to 0·46)
Iceland	0·07 (0·06 to 0·07)	0·06 (0·05 to 0·06)	0·04 (0·04 to 0·05)	0·03 (0·03 to 0·03)	0·03 (0·03 to 0·03)	0·03 (0·02 to 0·03)	0·02 (0·01 to 0·02)	0·01 (0·01 to 0·02)	0·01 (0·01 to 0·01)	0·01 (0·01 to 0·01)
Ireland	1·47 (1·42 to 1·53)	1·36 (1·31 to 1·42)	1·02 (0·98 to 1·07)	0·67 (0·64 to 0·70)	0·50 (0·48 to 0·53)	0·36 (0·34 to 0·39)	0·40 (0·38 to 0·42)	0·31 (0·29 to 0·33)	0·32 (0·30 to 0·34)	0·25 (0·18 to 0·35)
Israel	2·25 (2·08 to 2·44)	2·54 (2·45 to 2·63)	1·73 (1·67 to 1·80)	1·45 (1·38 to 1·50)	1·22 (1·17 to 1·27)	1·00 (0·96 to 1·05)	0·92 (0·89 to 0·97)	0·82 (0·78 to 0·85)	0·75 (0·71 to 0·78)	0·61 (0·53 to 0·70)
Italy	30·09 (29·67 to 30·56)	19·40 (19·06 to 19·73)	10·76 (10·51 to 11·01)	6·92 (6·74 to 7·10)	5·31 (5·15 to 5·47)	3·90 (3·78 to 4·03)	2·87 (2·77 to 2·98)	2·37 (2·28 to 2·47)	2·20 (2·12 to 2·29)	1·60 (1·12 to 2·26)
Luxembourg	0·11 (0·10 to 0·11)	0·07 (0·06 to 0·07)	0·06 (0·05 to 0·06)	0·05 (0·04 to 0·05)	0·04 (0·04 to 0·05)	0·03 (0·03 to 0·04)	0·03 (0·02 to 0·03)	0·02 (0·02 to 0·02)	0·02 (0·01 to 0·02)	0·01 (0·01 to 0·02)
Malta	0·15 (0·14 to 0·16)	0·12 (0·11 to 0·13)	0·09 (0·09 to 0·10)	0·08 (0·07 to 0·09)	0·06 (0·06 to 0·07)	0·05 (0·04 to 0·05)	0·03 (0·03 to 0·04)	0·03 (0·02 to 0·03)	0·03 (0·03 to 0·03)	0·02 (0·02 to 0·03)
Netherlands	3·78 (3·66 to 3·91)	2·47 (2·39 to 2·55)	1·93 (1·87 to 2·00)	1·75 (1·68 to 1·82)	1·69 (1·62 to 1·75)	1·33 (1·28 to 1·39)	1·29 (1·24 to 1·34)	1·06 (1·01 to 1·11)	0·82 (0·78 to 0·85)	0·67 (0·54 to 0·84)
Norway	1·07 (1·02 to 1·11)	0·76 (0·73 to 0·80)	0·52 (0·49 to 0·55)	0·53 (0·50 to 0·56)	0·52 (0·49 to 0·54)	0·34 (0·32 to 0·36)	0·29 (0·27 to 0·31)	0·23 (0·22 to 0·25)	0·20 (0·18 to 0·21)	0·17 (0·13 to 0·21)
Portugal	11·95 (11·82 to 12·08)	7·64 (7·46 to 7·84)	4·54 (4·47 to 4·61)	2·73 (2·68 to 2·77)	1·66 (1·63 to 1·70)	1·06 (1·03 to 1·09)	0·81 (0·79 to 0·83)	0·54 (0·52 to 0·56)	0·38 (0·37 to 0·39)	0·26 (0·22 to 0·30)
Spain	21·12 (20·79 to 21·48)	14·67 (14·40 to 14·94)	8·56 (8·38 to 8·74)	5·04 (4·90 to 5·18)	3·74 (3·63 to 3·85)	2·53 (2·44 to 2·62)	2·09 (2·01 to 2·18)	2·11 (2·04 to 2·20)	1·87 (1·80 to 1·95)	1·40 (1·03 to 1·87)
Sweden	1·48 (1·38 to 1·59)	1·08 (1·00 to 1·15)	0·78 (0·74 to 0·82)	0·70 (0·66 to 0·74)	0·83 (0·79 to 0·88)	0·49 (0·47 to 0·52)	0·36 (0·33 to 0·38)	0·35 (0·32 to 0·37)	0·36 (0·33 to 0·38)	0·31 (0·24 to 0·40)
Stockholm	0·21 (0·16 to 0·28)	0·16 (0·12 to 0·21)	0·13 (0·11 to 0·14)	0·11 (0·10 to 0·13)	0·14 (0·13 to 0·16)	0·09 (0·08 to 0·10)	0·07 (0·06 to 0·08)	0·07 (0·06 to 0·08)	0·06 (0·06 to 0·07)	0·06 (0·05 to 0·08)
Sweden except Stockholm	1·27 (1·19 to 1·35)	0·91 (0·85 to 0·98)	0·65 (0·61 to 0·69)	0·58 (0·55 to 0·62)	0·69 (0·65 to 0·73)	0·40 (0·38 to 0·42)	0·29 (0·27 to 0·31)	0·28 (0·26 to 0·30)	0·29 (0·27 to 0·32)	0·25 (0·18 to 0·33)
Switzerland	1·81 (1·76 to 1·87)	1·09 (1·05 to 1·13)	0·76 (0·73 to 0·80)	0·66 (0·63 to 0·69)	0·70 (0·67 to 0·73)	0·52 (0·49 to 0·54)	0·45 (0·42 to 0·47)	0·37 (0·35 to 0·39)	0·35 (0·34 to 0·38)	0·34 (0·26 to 0·44)
United Kingdom	19·30 (18·87 to 19·74)	12·97 (12·68 to 13·26)	10·44 (10·29 to 10·59)	8·37 (8·29 to 8·46)	7·44 (7·36 to 7·52)	5·36 (5·30 to 5·43)	4·49 (4·44 to 4·55)	4·30 (4·25 to 4·35)	4·12 (4·07 to 4·17)	3·57 (3·46 to 3·68)
England	15·33 (15·03 to 15·62)	10·28 (10·07 to 10·50)	8·42 (8·31 to 8·54)	6·85 (6·79 to 6·92)	6·26 (6·19 to 6·32)	4·47 (4·42 to 4·52)	3·80 (3·75 to 3·84)	3·66 (3·61 to 3·70)	3·53 (3·49 to 3·57)	3·07 (3·01 to 3·13)
East Midlands	1·18 (1·14 to 1·23)	0·82 (0·79 to 0·86)	0·68 (0·65 to 0·70)	0·52 (0·50 to 0·54)	0·51 (0·49 to 0·52)	0·35 (0·34 to 0·37)	0·30 (0·29 to 0·31)	0·29 (0·28 to 0·31)	0·28 (0·27 to 0·29)	0·27 (0·26 to 0·29)
East of England	1·29 (1·19 to 1·39)	0·88 (0·80 to 0·96)	0·73 (0·70 to 0·77)	0·61 (0·59 to 0·63)	0·50 (0·48 to 0·52)	0·40 (0·38 to 0·41)	0·32 (0·31 to 0·33)	0·32 (0·31 to 0·34)	0·32 (0·31 to 0·34)	0·28 (0·26 to 0·30)
Greater London	2·58 (2·49 to 2·67)	1·75 (1·68 to 1·81)	1·43 (1·39 to 1·47)	1·18 (1·15 to 1·20)	1·13 (1·11 to 1·15)	0·86 (0·84 to 0·88)	0·77 (0·75 to 0·79)	0·70 (0·68 to 0·72)	0·72 (0·71 to 0·74)	0·53 (0·52 to 0·55)
North East England	0·90 (0·84 to 0·96)	0·57 (0·53 to 0·62)	0·46 (0·44 to 0·49)	0·36 (0·34 to 0·37)	0·32 (0·31 to 0·34)	0·23 (0·22 to 0·24)	0·18 (0·17 to 0·19)	0·15 (0·15 to 0·16)	0·13 (0·13 to 0·14)	0·12 (0·11 to 0·13)
North West England	2·62 (2·46 to 2·77)	1·69 (1·58 to 1·80)	1·35 (1·30 to 1·40)	1·07 (1·05 to 1·10)	0·94 (0·92 to 0·97)	0·65 (0·63 to 0·66)	0·56 (0·54 to 0·57)	0·53 (0·52 to 0·55)	0·50 (0·49 to 0·52)	0·42 (0·41 to 0·44)
South East England	1·88 (1·79 to 1·98)	1·31 (1·24 to 1·37)	1·10 (1·06 to 1·15)	0·95 (0·92 to 0·98)	0·86 (0·84 to 0·89)	0·57 (0·55 to 0·58)	0·46 (0·44 to 0·48)	0·45 (0·44 to 0·47)	0·45 (0·44 to 0·47)	0·38 (0·37 to 0·40)
South West England	1·13 (1·08 to 1·19)	0·79 (0·75 to 0·83)	0·66 (0·63 to 0·68)	0·54 (0·52 to 0·55)	0·47 (0·45 to 0·49)	0·35 (0·34 to 0·36)	0·28 (0·27 to 0·29)	0·27 (0·26 to 0·28)	0·26 (0·25 to 0·27)	0·25 (0·24 to 0·26)

Appendix Table 12A. Under-5 death numbers by location, both sexes combined, 1970, 1975, 1980, 1985, 1990, 1995, 2000, 2005, 2010, 2016										
**Location**	**1970**	**1975**	**1980**	**1985**	**1990**	**1995**	**2000**	**2005**	**2010**	**2016**
West Midlands	1·99 (1·86 to 2·12)	1·30 (1·20 to 1·42)	1·05 (1·00 to 1·10)	0·86 (0·83 to 0·89)	0·84 (0·81 to 0·87)	0·55 (0·53 to 0·57)	0·49 (0·47 to 0·51)	0·50 (0·48 to 0·52)	0·47 (0·45 to 0·49)	0·47 (0·43 to 0·51)
Yorkshire and the Humber	1·76 (1·67 to 1·84)	1·17 (1·10 to 1·23)	0·95 (0·92 to 0·99)	0·77 (0·75 to 0·79)	0·68 (0·66 to 0·71)	0·52 (0·50 to 0·54)	0·44 (0·43 to 0·46)	0·43 (0·41 to 0·44)	0·38 (0·37 to 0·40)	0·34 (0·32 to 0·35)
Northern Ireland	0·84 (0·77 to 0·91)	0·62 (0·58 to 0·66)	0·47 (0·44 to 0·50)	0·34 (0·32 to 0·37)	0·25 (0·23 to 0·27)	0·20 (0·18 to 0·22)	0·14 (0·13 to 0·16)	0·15 (0·14 to 0·16)	0·15 (0·14 to 0·17)	0·11 (0·08 to 0·16)
Scotland	2·04 (1·97 to 2·10)	1·36 (1·31 to 1·41)	0·98 (0·94 to 1·02)	0·76 (0·72 to 0·79)	0·62 (0·59 to 0·65)	0·45 (0·42 to 0·47)	0·35 (0·33 to 0·37)	0·32 (0·30 to 0·34)	0·27 (0·25 to 0·29)	0·24 (0·17 to 0·32)
Wales	1·09 (0·83 to 1·43)	0·72 (0·55 to 0·93)	0·57 (0·50 to 0·65)	0·42 (0·39 to 0·44)	0·31 (0·29 to 0·33)	0·24 (0·23 to 0·26)	0·20 (0·19 to 0·22)	0·17 (0·16 to 0·19)	0·17 (0·16 to 0·18)	0·15 (0·12 to 0·19)
Southern Latin America	65·34 (64·69 to 65·98)	57·66 (56·52 to 58·89)	39·22 (38·89 to 39·55)	29·56 (29·27 to 29·85)	27·20 (26·92 to 27·48)	21·55 (21·29 to 21·80)	17·59 (16·67 to 18·50)	14·46 (13·72 to 15·20)	12·97 (12·34 to 13·61)	11·18 (9·87 to 12·62)
Argentina	38·59 (37·99 to 39·19)	37·56 (36·43 to 38·79)	27·78 (27·47 to 28·08)	21·56 (21·32 to 21·81)	20·10 (19·85 to 20·34)	16·43 (16·21 to 16·66)	13·92 (13·00 to 14·83)	11·52 (10·80 to 12·25)	10·24 (9·63 to 10·87)	8·85 (7·65 to 10·23)
Chile	23·67 (23·44 to 23·89)	16·93 (16·74 to 17·14)	9·23 (9·11 to 9·36)	6·24 (6·15 to 6·34)	5·80 (5·71 to 5·89)	3·88 (3·81 to 3·95)	2·80 (2·76 to 2·85)	2·25 (2·19 to 2·30)	2·22 (2·04 to 2·39)	1·89 (1·55 to 2·30)
Uruguay	3·08 (2·98 to 3·17)	3·17 (3·08 to 3·27)	2·21 (2·14 to 2·30)	1·75 (1·68 to 1·83)	1·29 (1·23 to 1·36)	1·23 (1·13 to 1·34)	0·87 (0·80 to 0·93)	0·69 (0·63 to 0·76)	0·51 (0·44 to 0·59)	0·43 (0·28 to 0·62)
Central Europe, Eastern Europe, and Central Asi	331·20 (310·30 to 353·87)	302·38 (287·52 to 317·29)	281·62 (270·66 to 294·07)	270·62 (261·68 to 280·42)	218·86 (211·32 to 226·58)	172·51 (165·91 to 180·10)	126·45 (120·24 to 133·21)	105·52 (97·42 to 114·92)	92·92 (82·94 to 105·15)	77·55 (67·27 to 91·46)
Eastern Europe	96·57 (86·13 to 110·34)	100·50 (91·89 to 111·24)	96·34 (90·20 to 103·63)	91·77 (88·06 to 96·51)	64·58 (61·72 to 67·78)	47·79 (45·49 to 50·26)	37·11 (35·07 to 39·58)	29·03 (27·23 to 31·23)	24·58 (23·01 to 26·56)	21·75 (19·33 to 24·79)
Belarus	5·36 (4·28 to 6·61)	4·44 (3·59 to 5·34)	4·06 (3·32 to 4·89)	4·00 (3·34 to 4·77)	3·00 (2·50 to 3·60)	2·01 (1·66 to 2·41)	1·51 (1·23 to 1·82)	1·09 (0·88 to 1·35)	0·86 (0·67 to 1·09)	0·64 (0·48 to 0·86)
Estonia	0·47 (0·44 to 0·49)	0·47 (0·44 to 0·50)	0·50 (0·47 to 0·53)	0·48 (0·45 to 0·51)	0·37 (0·35 to 0·40)	0·26 (0·24 to 0·28)	0·15 (0·13 to 0·16)	0·10 (0·09 to 0·12)	0·07 (0·06 to 0·08)	0·04 (0·03 to 0·06)
Latvia	0·76 (0·71 to 0·80)	0·89 (0·84 to 0·94)	0·76 (0·72 to 0·80)	0·71 (0·66 to 0·75)	0·66 (0·63 to 0·70)	0·47 (0·44 to 0·50)	0·26 (0·24 to 0·28)	0·21 (0·19 to 0·23)	0·16 (0·14 to 0·18)	0·11 (0·07 to 0·16)
Lithuania	1·28 (1·23 to 1·34)	1·25 (1·19 to 1·31)	0·98 (0·94 to 1·02)	1·09 (1·04 to 1·14)	0·78 (0·75 to 0·82)	0·66 (0·63 to 0·69)	0·39 (0·36 to 0·41)	0·26 (0·24 to 0·28)	0·19 (0·18 to 0·20)	0·14 (0·11 to 0·17)
Moldova	5·40 (3·55 to 7·89)	5·00 (3·35 to 7·02)	4·80 (3·78 to 5·96)	3·99 (3·25 to 4·80)	2·59 (2·11 to 3·12)	2·04 (1·69 to 2·47)	1·37 (1·10 to 1·67)	0·76 (0·58 to 0·97)	0·74 (0·55 to 0·95)	0·52 (0·35 to 0·76)
Russia	53·51 (52·92 to 54·17)	61·84 (61·14 to 62·54)	61·25 (60·54 to 62·03)	62·76 (61·98 to 63·59)	43·44 (42·99 to 43·92)	31·75 (31·28 to 32·26)	25·22 (24·64 to 25·85)	19·91 (19·63 to 20·21)	16·87 (16·66 to 17·10)	15·82 (14·49 to 17·27)
Ukraine	29·80 (19·16 to 43·24)	26·62 (18·34 to 37·04)	23·98 (18·06 to 31·06)	18·76 (15·12 to 23·25)	13·72 (11·10 to 16·81)	10·60 (8·47 to 13·04)	8·22 (6·37 to 10·45)	6·68 (5·01 to 8·80)	5·69 (4·18 to 7·58)	4·48 (2·51 to 7·46)
Central Europe	85·08 (80·79 to 90·35)	72·44 (68·96 to 76·42)	59·14 (56·67 to 61·93)	48·12 (46·44 to 49·85)	37·52 (36·56 to 38·50)	25·55 (24·97 to 26·12)	16·33 (15·88 to 16·81)	12·22 (11·86 to 12·61)	9·30 (9·03 to 9·60)	6·86 (5·91 to 8·02)
Albania	6·21 (3·57 to 10·34)	4·91 (2·90 to 7·78)	4·35 (3·01 to 6·11)	4·30 (3·49 to 5·22)	3·46 (2·89 to 4·07)	2·64 (2·21 to 3·12)	1·45 (1·18 to 1·77)	0·86 (0·66 to 1·11)	0·61 (0·42 to 0·88)	0·54 (0·32 to 0·84)
Bosnia and Herzegovina	4·60 (3·32 to 6·35)	3·33 (2·48 to 4·40)	2·39 (1·91 to 3·00)	1·96 (1·86 to 2·06)	1·20 (1·15 to 1·25)	1·17 (1·04 to 1·33)	0·38 (0·36 to 0·40)	0·29 (0·27 to 0·31)	0·24 (0·22 to 0·25)	0·17 (0·13 to 0·20)
Bulgaria	4·39 (4·25 to 4·53)	3·96 (3·83 to 4·09)	3·15 (3·05 to 3·26)	2·30 (2·21 to 2·39)	1·94 (1·87 to 2·03)	1·37 (1·30 to 1·43)	1·17 (1·11 to 1·24)	0·90 (0·85 to 0·96)	0·82 (0·77 to 0·87)	0·55 (0·33 to 0·88)
Croatia	2·35 (1·64 to 3·29)	1·87 (1·37 to 2·44)	1·51 (1·22 to 1·86)	1·17 (1·11 to 1·23)	0·70 (0·67 to 0·74)	0·60 (0·54 to 0·65)	0·38 (0·36 to 0·40)	0·28 (0·26 to 0·30)	0·23 (0·21 to 0·24)	0·17 (0·14 to 0·20)
Czech Republic	3·49 (3·36 to 3·61)	4·08 (3·96 to 4·21)	2·90 (2·79 to 3·02)	1·96 (1·87 to 2·03)	1·56 (1·48 to 1·63)	0·88 (0·83 to 0·93)	0·48 (0·44 to 0·51)	0·43 (0·40 to 0·46)	0·40 (0·37 to 0·43)	0·30 (0·23 to 0·41)
Hungary	6·03 (5·88 to 6·19)	6·87 (6·70 to 7·05)	3·77 (3·67 to 3·89)	2·93 (2·83 to 3·03)	2·16 (2·07 to 2·24)	1·39 (1·33 to 1·46)	1·02 (0·97 to 1·07)	0·73 (0·68 to 0·78)	0·55 (0·51 to 0·58)	0·50 (0·37 to 0·68)
Macedonia	2·91 (1·61 to 4·82)	2·32 (1·24 to 3·88)	2·16 (1·50 to 3·04)	1·63 (1·17 to 2·21)	1·24 (1·18 to 1·30)	0·77 (0·73 to 0·82)	0·40 (0·37 to 0·44)	0·32 (0·30 to 0·35)	0·23 (0·21 to 0·25)	0·22 (0·14 to 0·33)
Montenegro	0·36 (0·24 to 0·54)	0·30 (0·20 to 0·46)	0·25 (0·16 to 0·38)	0·21 (0·13 to 0·31)	0·15 (0·11 to 0·20)	0·14 (0·12 to 0·16)	0·14 (0·13 to 0·15)	0·08 (0·08 to 0·09)	0·05 (0·05 to 0·06)	0·04 (0·03 to 0·05)
Poland	20·42 (20·08 to 20·76)	18·22 (17·88 to 18·59)	16·77 (16·47 to 17·11)	14·07 (13·69 to 14·45)	10·02 (9·80 to 10·25)	6·70 (6·52 to 6·89)	3·57 (3·44 to 3·71)	2·74 (2·64 to 2·85)	2·37 (2·26 to 2·48)	1·68 (0·99 to 2·72)
Romania	24·61 (24·22 to 24·99)	17·59 (17·27 to 17·90)	14·26 (14·02 to 14·53)	11·47 (11·22 to 11·71)	10·89 (10·66 to 11·13)	6·18 (5·99 to 6·37)	5·14 (4·97 to 5·29)	3·88 (3·72 to 4·05)	2·44 (2·35 to 2·54)	1·66 (1·35 to 2·07)
Serbia	6·44 (4·26 to 9·24)	5·66 (3·83 to 8·10)	4·81 (3·32 to 6·65)	4·07 (2·92 to 5·41)	2·82 (2·18 to 3·61)	2·80 (2·70 to 2·90)	1·56 (1·28 to 1·87)	1·17 (0·97 to 1·41)	0·88 (0·83 to 0·93)	0·64 (0·53 to 0·76)
Slovakia	2·45 (2·36 to 2·54)	2·67 (2·58 to 2·78)	2·25 (2·17 to 2·34)	1·66 (1·59 to 1·74)	1·15 (1·09 to 1·21)	0·78 (0·74 to 0·83)	0·53 (0·50 to 0·57)	0·46 (0·43 to 0·50)	0·41 (0·38 to 0·44)	0·34 (0·23 to 0·49)
Slovenia	0·81 (0·58 to 1·12)	0·67 (0·51 to 0·88)	0·55 (0·47 to 0·63)	0·39 (0·37 to 0·41)	0·23 (0·22 to 0·25)	0·14 (0·13 to 0·15)	0·10 (0·09 to 0·11)	0·08 (0·07 to 0·09)	0·07 (0·06 to 0·08)	0·05 (0·04 to 0·06)
Central Asia	149·56 (133·03 to 167·45)	129·44 (118·97 to 140·67)	126·13 (117·33 to 136·11)	130·73 (122·53 to 139·39)	116·76 (109·81 to 124·38)	99·17 (92·95 to 106·08)	73·02 (67·23 to 79·48)	64·27 (56·55 to 73·08)	59·04 (49·26 to 71·16)	48·95 (39·27 to 61·62)
Armenia	6·69 (4·60 to 9·47)	5·64 (4·36 to 7·30)	5·68 (4·77 to 6·79)	5·01 (4·33 to 5·82)	3·65 (3·15 to 4·23)	2·16 (1·84 to 2·52)	1·16 (0·97 to 1·38)	0·91 (0·70 to 1·16)	0·70 (0·54 to 0·90)	0·55 (0·39 to 0·74)
Azerbaijan	18·75 (12·95 to 25·60)	13·91 (10·21 to 18·65)	14·34 (11·46 to 17·82)	15·62 (13·07 to 18·33)	14·64 (12·24 to 17·31)	12·20 (10·32 to 14·41)	8·00 (6·76 to 9·48)	7·81 (6·24 to 9·61)	7·60 (5·49 to 10·05)	5·73 (3·76 to 8·37)
Georgia	9·17 (6·94 to 11·94)	7·25 (5·78 to 9·00)	5·56 (4·50 to 6·78)	4·69 (3·98 to 5·47)	3·89 (3·38 to 4·45)	2·65 (2·31 to 3·05)	1·96 (1·69 to 2·26)	1·61 (1·29 to 1·99)	1·39 (1·04 to 1·81)	1·01 (0·73 to 1·39)
Kazakhstan	24·12 (17·46 to 31·67)	21·26 (17·59 to 25·60)	19·20 (16·32 to 22·15)	17·37 (15·26 to 19·64)	14·30 (12·54 to 16·05)	11·94 (10·13 to 13·98)	8·92 (7·31 to 10·73)	8·61 (6·77 to 10·75)	7·52 (5·93 to 9·33)	5·33 (3·88 to 7·15)
Kyrgyzstan	13·28 (10·64 to 16·65)	10·90 (9·15 to 12·87)	10·12 (8·77 to 11·55)	10·19 (9·16 to 11·22)	8·55 (7·88 to 9·26)	6·37 (5·86 to 6·93)	5·02 (4·58 to 5·50)	4·52 (4·07 to 5·02)	4·93 (4·35 to 5·55)	4·44 (3·56 to 5·38)
Mongolia	7·92 (6·19 to 9·83)	7·42 (5·87 to 9·08)	7·61 (6·20 to 9·12)	7·61 (6·56 to 8·68)	6·42 (5·70 to 7·16)	4·16 (3·67 to 4·70)	2·81 (2·46 to 3·17)	2·08 (1·78 to 2·41)	1·94 (1·62 to 2·29)	1·65 (1·23 to 2·13)
Tajikistan	21·07 (16·05 to 27·30)	19·99 (16·35 to 24·32)	18·61 (15·83 to 21·67)	20·25 (17·51 to 23·38)	19·42 (16·70 to 22·38)	16·39 (14·19 to 18·80)	11·92 (9·87 to 14·47)	9·01 (7·50 to 10·90)	8·79 (6·86 to 11·07)	9·41 (5·86 to 14·34)
Turkmenistan	12·35 (8·40 to 17·22)	11·10 (7·48 to 15·54)	10·69 (8·36 to 13·60)	11·30 (9·34 to 13·41)	11·40 (9·61 to 13·32)	10·50 (8·77 to 12·64)	8·28 (6·11 to 11·00)	6·73 (4·67 to 9·10)	5·62 (3·69 to 7·91)	4·47 (2·94 to 6·54)
Uzbekistan	36·21 (25·53 to 49·03)	31·96 (25·07 to 39·87)	34·32 (28·01 to 41·33)	38·70 (32·77 to 45·40)	34·49 (29·15 to 40·13)	32·80 (28·11 to 38·45)	24·95 (20·38 to 30·26)	22·97 (16·47 to 30·96)	20·55 (12·04 to 31·61)	16·37 (8·64 to 27·70)
Latin America and Caribbean	1,042·38 (1,014·49 to 1,070·47)	948·91 (927·56 to 970·02)	830·07 (810·87 to 851·00)	700·91 (685·98 to 716·06)	573·03 (559·62 to 586·71)	461·77 (451·04 to 473·57)	358·67 (347·13 to 370·54)	291·18 (281·69 to 301·35)	267·10 (243·22 to 293·75)	185·21 (175·07 to 197·95)
Central Latin America	390·40 (380·45 to 400·83)	356·70 (347·76 to 366·17)	304·44 (296·66 to 312·88)	257·48 (251·11 to 264·56)	217·96 (211·77 to 224·12)	184·61 (179·52 to 189·58)	147·85 (143·01 to 152·64)	119·61 (115·39 to 124·35)	98·17 (93·97 to 102·51)	75·72 (71·77 to 80·32)
Colombia	63·13 (57·42 to 69·60)	54·55 (49·55 to 59·64)	42·27 (37·85 to 46·60)	37·48 (33·57 to 41·53)	30·36 (26·41 to 34·59)	27·28 (24·38 to 30·23)	22·60 (20·22 to 25·10)	19·75 (17·40 to 22·65)	15·98 (13·42 to 18·92)	10·65 (8·61 to 13·36)
Costa Rica	4·14 (3·85 to 4·46)	2·83 (2·59 to 3·09)	2·21 (1·96 to 2·47)	1·92 (1·68 to 2·18)	1·60 (1·38 to 1·85)	1·32 (1·09 to 1·60)	1·16 (0·95 to 1·41)	0·99 (0·80 to 1·20)	0·85 (0·65 to 1·10)	0·63 (0·41 to 0·94)
El Salvador	21·27 (18·54 to 24·18)	18·79 (16·82 to 20·86)	15·41 (13·85 to 17·06)	11·44 (10·28 to 12·64)	8·20 (7·24 to 9·28)	6·12 (5·24 to 7·15)	4·31 (3·67 to 5·01)	2·77 (2·18 to 3·43)	1·89 (1·47 to 2·39)	1·25 (0·86 to 1·77)
Guatemala	42·62 (38·09 to 47·49)	39·24 (35·10 to 43·68)	34·85 (31·35 to 38·45)	30·91 (27·96 to 33·91)	27·68 (25·34 to 30·22)	24·32 (22·32 to 26·42)	20·76 (18·69 to 22·92)	17·44 (15·45 to 19·44)	13·88 (12·29 to 15·74)	10·95 (9·09 to 13·08)
Honduras	16·47 (14·85 to 18·17)	15·23 (13·87 to 16·70)	13·16 (11·84 to 14·42)	11·54 (10·35 to 12·78)	9·84 (8·64 to 11·03)	8·06 (6·79 to 9·29)	6·47 (5·46 to 7·52)	5·41 (4·62 to 6·26)	4·92 (4·17 to 5·81)	4·17 (3·28 to 5·17)
Mexico	199·95 (194·01 to 206·12)	185·96 (180·04 to 192·03)	164·13 (159·40 to 169·30)	135·98 (132·05 to 139·74)	114·29 (111·15 to 117·73)	93·66 (90·65 to 96·95)	73·58 (70·77 to 76·35)	57·15 (54·40 to 59·89)	46·31 (43·82 to 48·99)	35·57 (33·24 to 38·12)
Nicaragua	12·84 (11·94 to 13·83)	11·50 (10·83 to 12·17)	10·20 (9·67 to 10·80)	9·97 (9·15 to 10·74)	7·91 (7·46 to 8·36)	7·00 (6·53 to 7·47)	5·23 (4·83 to 5·64)	3·80 (3·44 to 4·18)	2·75 (2·40 to 3·15)	1·79 (1·50 to 2·17)
Panama	3·18 (2·89 to 3·49)	2·48 (2·24 to 2·73)	2·07 (1·82 to 2·32)	1·76 (1·53 to 1·99)	1·68 (1·45 to 1·93)	1·67 (1·41 to 2·00)	1·56 (1·33 to 1·86)	1·43 (1·16 to 1·72)	1·37 (1·12 to 1·65)	1·14 (0·80 to 1·61)
Venezuela	26·80 (26·61 to 26·99)	26·12 (25·91 to 26·34)	20·14 (19·95 to 20·33)	16·50 (16·32 to 16·68)	16·39 (16·18 to 16·60)	15·16 (14·58 to 15·79)	12·19 (11·03 to 13·43)	10·88 (10·47 to 11·30)	10·22 (10·08 to 10·36)	9·58 (8·12 to 11·27)

Appendix Table 12A. Under-5 death numbers by location, both sexes combined, 1970, 1975, 1980, 1985, 1990, 1995, 2000, 2005, 2010, 2016										
Location	1970	1975	1980	1985	1990	1995	2000	2005	2010	2016
Andean Latin America	162·89 (151·63 to 174·35)	143·16 (135·93 to 150·28)	131·41 (124·23 to 138·90)	114·63 (108·94 to 120·74)	92·98 (88·77 to 97·46)	72·00 (68·71 to 75·57)	52·82 (49·52 to 55·93)	41·53 (38·34 to 44·75)	34·25 (31·16 to 37·26)	28·19 (24·29 to 32·41)
Bolivia	42·91 (39·35 to 46·60)	39·72 (37·07 to 42·33)	36·27 (33·88 to 38·94)	32·45 (30·22 to 34·86)	27·88 (26·00 to 29·72)	23·54 (21·69 to 25·40)	19·25 (17·19 to 21·66)	15·65 (13·64 to 17·81)	12·49 (10·25 to 15·03)	9·74 (7·35 to 12·63)
Ecuador	26·61 (24·05 to 28·97)	24·69 (22·23 to 26·95)	21·82 (19·88 to 23·64)	18·65 (17·13 to 20·28)	15·62 (13·97 to 17·47)	13·26 (11·77 to 14·76)	10·97 (9·58 to 12·49)	9·15 (7·77 to 10·60)	7·66 (6·41 to 9·04)	6·34 (5·13 to 7·73)
Peru	93·37 (82·77 to 104·31)	78·75 (72·36 to 84·72)	73·32 (66·95 to 79·81)	63·53 (58·60 to 68·58)	49·48 (46·01 to 53·25)	35·20 (32·26 to 38·24)	22·60 (20·67 to 24·50)	16·72 (14·89 to 18·70)	14·10 (12·66 to 15·58)	12·11 (9·61 to 15·23)
Caribbean	101·68 (88·51 to 115·18)	88·82 (75·84 to 100·79)	79·08 (67·36 to 91·95)	69·99 (60·51 to 80·01)	61·83 (52·80 to 70·43)	52·47 (45·01 to 60·31)	43·46 (35·12 to 53·06)	40·89 (34·16 to 48·09)	63·79 (40·11 to 88·42)	27·23 (19·75 to 37·76)
Antigua and Barbuda	0·06 (0·05 to 0·08)	0·05 (0·03 to 0·06)	0·03 (0·02 to 0·04)	0·02 (0·01 to 0·03)	0·02 (0·01 to 0·02)	0·02 (0·01 to 0·03)	0·02 (0·01 to 0·03)	0·02 (0·01 to 0·03)	0·02 (0·01 to 0·02)	0·01 (0·01 to 0·02)
The Bahamas	0·23 (0·15 to 0·34)	0·20 (0·14 to 0·29)	0·18 (0·12 to 0·27)	0·16 (0·10 to 0·24)	0·13 (0·08 to 0·22)	0·11 (0·07 to 0·16)	0·08 (0·05 to 0·11)	0·08 (0·06 to 0·11)	0·08 (0·05 to 0·12)	0·08 (0·03 to 0·16)
Barbados	0·24 (0·17 to 0·34)	0·17 (0·11 to 0·23)	0·12 (0·08 to 0·17)	0·10 (0·06 to 0·15)	0·07 (0·04 to 0·11)	0·06 (0·03 to 0·10)	0·06 (0·03 to 0·10)	0·06 (0·04 to 0·08)	0·05 (0·03 to 0·08)	0·04 (0·02 to 0·09)
Belize	0·54 (0·42 to 0·66)	0·44 (0·36 to 0·54)	0·38 (0·31 to 0·46)	0·35 (0·29 to 0·42)	0·30 (0·24 to 0·36)	0·19 (0·15 to 0·25)	0·21 (0·15 to 0·27)	0·19 (0·13 to 0·26)	0·16 (0·10 to 0·23)	0·15 (0·07 to 0·29)
Bermuda	0·03 (0·02 to 0·03)	0·02 (0·01 to 0·02)	0·01 (0·01 to 0·02)	0·01 (0·01 to 0·01)	0·01 (0·01 to 0·01)	0·01 (0·01 to 0·01)	<0·01 (<0·01 to 0·01)	0·01 (<0·01 to 0·01)	<0·01 (<0·01 to 0·01)	<0·01 (<0·01 to 0·01)
Cuba	10·60 (10·48 to 10·73)	5·95 (5·86 to 6·04)	3·34 (3·25 to 3·42)	3·25 (3·13 to 3·36)	2·57 (2·49 to 2·64)	1·66 (1·62 to 1·71)	1·28 (1·25 to 1·31)	0·87 (0·85 to 0·90)	0·78 (0·75 to 0·80)	0·60 (0·48 to 0·74)
Dominica	0·17 (0·12 to 0·22)	0·08 (0·05 to 0·11)	0·04 (0·03 to 0·06)	0·03 (0·02 to 0·05)	0·03 (0·02 to 0·04)	0·03 (0·02 to 0·04)	0·02 (0·02 to 0·03)	0·02 (0·02 to 0·03)	0·03 (0·02 to 0·04)	0·03 (0·02 to 0·04)
Dominican Republic	20·53 (17·61 to 23·50)	19·97 (17·35 to 22·65)	17·40 (15·34 to 19·61)	13·55 (11·83 to 15·37)	11·90 (9·88 to 13·96)	10·02 (8·04 to 12·01)	7·98 (6·75 to 9·33)	7·79 (6·22 to 9·64)	6·66 (5·16 to 8·50)	5·11 (3·63 to 6·89)
Grenada	0·16 (0·09 to 0·24)	0·13 (0·07 to 0·20)	0·09 (0·05 to 0·17)	0·09 (0·04 to 0·16)	0·05 (0·03 to 0·09)	0·04 (0·02 to 0·06)	0·03 (0·02 to 0·05)	0·03 (0·02 to 0·04)	0·03 (0·02 to 0·04)	0·03 (0·01 to 0·05)
Guyana	1·96 (1·71 to 2·23)	1·83 (1·63 to 2·05)	1·58 (1·40 to 1·79)	1·28 (1·07 to 1·51)	1·17 (0·93 to 1·43)	0·98 (0·78 to 1·17)	0·76 (0·62 to 0·92)	0·61 (0·50 to 0·73)	0·45 (0·36 to 0·56)	0·34 (0·25 to 0·45)
Haiti	54·14 (41·55 to 67·08)	49·80 (37·90 to 61·20)	47·39 (36·20 to 59·62)	44·03 (34·78 to 53·51)	39·28 (31·21 to 47·11)	33·87 (27·24 to 41·27)	28·40 (20·17 to 37·63)	27·20 (20·75 to 34·14)	51·93 (28·24 to 76·44)	17·95 (11·07 to 27·81)
Jamaica	4·12 (3·42 to 4·88)	3·18 (2·60 to 3·88)	2·73 (1·98 to 3·66)	2·36 (1·72 to 3·14)	2·15 (1·46 to 3·15)	1·85 (1·19 to 2·75)	1·59 (1·04 to 2·36)	1·31 (0·78 to 2·09)	1·26 (0·63 to 2·28)	1·13 (0·61 to 1·88)
Puerto Rico	2·16 (2·12 to 2·21)	1·75 (1·71 to 1·79)	1·46 (1·43 to 1·50)	1·12 (1·07 to 1·17)	0·98 (0·96 to 1·01)	0·85 (0·80 to 0·90)	0·64 (0·59 to 0·70)	0·48 (0·43 to 0·54)	0·38 (0·34 to 0·43)	0·28 (0·22 to 0·34)
Saint Lucia	0·30 (0·17 to 0·50)	0·20 (0·13 to 0·32)	0·14 (0·09 to 0·21)	0·11 (0·06 to 0·18)	0·08 (0·05 to 0·14)	0·07 (0·04 to 0·11)	0·05 (0·03 to 0·08)	0·05 (0·03 to 0·08)	0·04 (0·03 to 0·07)	0·03 (0·02 to 0·06)
Saint Vincent and the Grenadines	0·33 (0·22 to 0·47)	0·24 (0·16 to 0·34)	0·16 (0·11 to 0·24)	0·11 (0·07 to 0·16)	0·08 (0·05 to 0·13)	0·07 (0·04 to 0·10)	0·05 (0·03 to 0·07)	0·04 (0·03 to 0·06)	0·04 (0·03 to 0·05)	0·03 (0·02 to 0·05)
Suriname	0·92 (0·74 to 1·15)	0·73 (0·61 to 0·88)	0·65 (0·56 to 0·77)	0·64 (0·53 to 0·75)	0·59 (0·47 to 0·74)	0·58 (0·44 to 0·75)	0·55 (0·40 to 0·71)	0·49 (0·32 to 0·66)	0·41 (0·26 to 0·60)	0·33 (0·19 to 0·54)
Trinidad and Tobago	1·50 (1·30 to 1·70)	1·34 (1·15 to 1·56)	1·25 (1·04 to 1·50)	1·06 (0·86 to 1·29)	0·70 (0·55 to 0·87)	0·57 (0·41 to 0·76)	0·53 (0·38 to 0·71)	0·46 (0·33 to 0·62)	0·38 (0·20 to 0·66)	0·28 (0·10 to 0·65)
Virgin Islands, U.S.	0·08 (0·07 to 0·09)	0·07 (0·06 to 0·08)	0·06 (0·06 to 0·07)	0·05 (0·04 to 0·05)	0·04 (0·04 to 0·05)	0·03 (0·03 to 0·04)	0·02 (0·02 to 0·02)	0·02 (0·02 to 0·02)	0·01 (0·01 to 0·02)	0·01 (0·01 to 0·01)
Tropical Latin America	387·40 (370·54 to 405·48)	360·23 (345·43 to 374·83)	315·13 (303·32 to 327·17)	258·80 (250·15 to 266·66)	200·25 (193·69 to 206·68)	152·70 (147·62 to 157·82)	114·54 (109·77 to 119·57)	89·16 (84·74 to 93·59)	70·89 (66·06 to 75·87)	54·08 (50·14 to 57·89)
Brazil	381·56 (364·76 to 400·08)	354·61 (339·89 to 369·19)	309·91 (298·01 to 321·75)	253·62 (245·07 to 261·63)	195·65 (189·13 to 202·07)	148·45 (143·43 to 153·59)	110·33 (105·67 to 115·43)	85·44 (81·01 to 89·88)	67·81 (63·16 to 72·69)	51·69 (47·75 to 55·42)
Paraguay	5·85 (5·18 to 6·59)	5·61 (5·01 to 6·23)	5·23 (4·52 to 5·91)	5·18 (4·66 to 5·72)	4·60 (4·15 to 5·09)	4·25 (3·66 to 4·92)	4·21 (3·61 to 4·91)	3·72 (3·09 to 4·44)	3·08 (2·33 to 4·01)	2·39 (1·76 to 3·20)
Southeast Asia, East Asia, and Oceania	4,680·63 (4,513·53 to 4,853·10)	3,585·26 (3,466·93 to 3,709·45)	2,822·90 (2,724·44 to 2,917·13)	2,435·14 (2,357·40 to 2,509·11)	2,047·19 (1,985·54 to 2,113·07)	1,595·70 (1,531·67 to 1,664·88)	1,190·57 (1,138·10 to 1,249·71)	834·76 (801·60 to 872·18)	628·63 (600·11 to 657·86)	422·43 (395·54 to 450·63)
East Asia	3,228·78 (3,076·56 to 3,393·65)	2,293·55 (2,192·21 to 2,399·57)	1,717·13 (1,638·80 to 1,797·43)	1,467·79 (1,401·89 to 1,533·43)	1,218·52 (1,167·87 to 1,275·60)	896·63 (845·74 to 954·87)	605·37 (562·94 to 653·52)	351·00 (335·82 to 366·91)	243·15 (230·82 to 256·31)	154·53 (140·90 to 170·05)
China	3,183·91 (3,035·01 to 3,344·13)	2,263·60 (2,164·70 to 2,368·33)	1,694·78 (1,618·63 to 1,777·04)	1,446·29 (1,381·46 to 1,510·94)	1,198·00 (1,146·90 to 1,255·53)	825·37 (794·59 to 858·67)	534·34 (514·66 to 555·90)	327·06 (314·22 to 339·79)	223·01 (213·60 to 233·18)	137·53 (125·38 to 150·62)
North Korea	34·30 (22·94 to 48·28)	22·76 (14·79 to 31·96)	16·11 (10·40 to 23·36)	17·71 (11·60 to 25·61)	17·69 (11·18 to 26·29)	68·36 (29·58 to 111·61)	68·52 (32·65 to 108·18)	22·47 (14·92 to 32·82)	19·13 (12·47 to 28·20)	16·08 (10·40 to 24·93)
Taiwan (Province of China)	10·57 (10·39 to 10·74)	7·19 (7·03 to 7·34)	6·25 (6·10 to 6·39)	3·79 (3·68 to 3·90)	2·83 (2·75 to 2·92)	2·91 (2·82 to 3·00)	2·51 (2·43 to 2·58)	1·48 (1·43 to 1·54)	1·01 (0·96 to 1·06)	0·92 (0·80 to 1·08)
Southeast Asia	1,434·28 (1,368·14 to 1,507·80)	1,273·98 (1,212·37 to 1,341·93)	1,088·97 (1,037·31 to 1,139·35)	950·63 (913·40 to 990·37)	811·75 (774·83 to 853·12)	682·08 (645·10 to 728·72)	568·30 (536·47 to 605·54)	466·90 (437·56 to 501·46)	369·88 (345·80 to 397·36)	254·38 (231·69 to 277·91)
Cambodia	50·45 (41·52 to 60·33)	81·66 (51·43 to 113·35)	40·81 (35·11 to 47·06)	36·57 (31·63 to 41·75)	33·63 (30·06 to 37·27)	34·39 (31·12 to 38·16)	30·55 (27·63 to 33·69)	23·97 (21·29 to 26·92)	17·83 (15·23 to 20·53)	12·45 (9·87 to 15·32)
Indonesia	751·42 (728·26 to 774·03)	637·80 (611·66 to 663·67)	556·13 (535·14 to 576·52)	472·82 (457·44 to 489·61)	389·09 (377·24 to 401·64)	321·50 (312·52 to 330·86)	265·40 (256·90 to 273·94)	215·27 (207·04 to 223·78)	172·48 (163·74 to 182·31)	112·70 (102·57 to 123·67)
Laos	36·85 (22·46 to 55·60)	36·28 (23·62 to 51·26)	31·55 (22·45 to 41·44)	29·23 (21·81 to 36·24)	30·02 (23·31 to 37·16)	30·06 (23·02 to 37·70)	26·33 (20·73 to 32·31)	21·70 (16·48 to 27·59)	18·16 (11·66 to 26·60)	14·87 (7·49 to 25·62)
Malaysia	19·67 (19·42 to 19·94)	16·24 (15·53 to 16·90)	12·76 (11·80 to 13·73)	10·79 (10·60 to 10·99)	8·51 (8·34 to 8·69)	7·09 (6·94 to 7·24)	5·00 (4·88 to 5·13)	3·94 (3·82 to 4·05)	4·15 (3·88 to 4·41)	3·06 (2·58 to 3·58)
Maldives	1·19 (1·03 to 1·36)	1·01 (0·88 to 1·15)	0·93 (0·81 to 1·06)	0·74 (0·64 to 0·84)	0·49 (0·43 to 0·56)	0·29 (0·25 to 0·33)	0·16 (0·14 to 0·17)	0·10 (0·10 to 0·11)	0·08 (0·07 to 0·09)	0·04 (0·04 to 0·05)
Mauritius	1·91 (1·87 to 1·95)	1·40 (1·37 to 1·43)	0·96 (0·94 to 0·99)	0·54 (0·52 to 0·56)	0·50 (0·48 to 0·52)	0·44 (0·43 to 0·46)	0·36 (0·34 to 0·37)	0·28 (0·27 to 0·30)	0·22 (0·21 to 0·23)	0·19 (0·15 to 0·23)
Myanmar	154·73 (106·39 to 211·51)	138·39 (99·13 to 185·24)	127·40 (91·31 to 166·31)	120·82 (91·72 to 153·29)	114·15 (83·77 to 150·14)	104·11 (73·47 to 146·59)	91·70 (63·18 to 127·52)	72·83 (47·93 to 105·00)	46·65 (30·23 to 67·75)	26·23 (15·54 to 44·11)
Philippines	127·47 (118·85 to 136·39)	127·24 (117·26 to 137·54)	128·95 (118·39 to 139·81)	121·81 (112·78 to 131·05)	107·13 (98·91 to 116·35)	92·66 (85·80 to 99·67)	83·51 (76·56 to 90·92)	77·55 (70·12 to 85·67)	69·63 (60·41 to 80·07)	58·02 (47·89 to 69·70)
Sri Lanka	25·50 (25·19 to 25·79)	25·81 (25·23 to 26·40)	18·74 (18·47 to 19·01)	12·74 (12·50 to 13·00)	11·96 (11·45 to 12·51)	7·37 (7·21 to 7·55)	5·84 (5·66 to 6·02)	6·47 (6·31 to 6·63)	4·81 (4·34 to 5·25)	1·92 (1·24 to 2·87)
Seychelles	0·14 (0·13 to 0·15)	0·09 (0·09 to 0·10)	0·06 (0·05 to 0·06)	0·04 (0·04 to 0·05)	0·03 (0·03 to 0·03)	0·02 (0·02 to 0·03)	0·02 (0·02 to 0·02)	0·02 (0·02 to 0·02)	0·02 (0·02 to 0·03)	0·02 (0·01 to 0·02)
Thailand	123·10 (107·98 to 138·25)	99·86 (86·94 to 113·76)	72·58 (58·27 to 87·54)	48·76 (39·89 to 58·94)	34·63 (27·18 to 42·82)	24·15 (18·19 to 31·13)	16·43 (12·16 to 21·43)	10·46 (8·02 to 13·69)	6·59 (5·28 to 8·23)	3·69 (2·63 to 4·95)
Timor-Leste	6·71 (4·74 to 9·09)	6·79 (5·02 to 8·58)	6·02 (4·55 to 7·63)	4·58 (3·43 to 5·91)	3·88 (3·00 to 4·84)	3·17 (2·47 to 3·87)	2·02 (1·51 to 2·63)	1·72 (1·20 to 2·32)	1·69 (1·11 to 2·47)	1·18 (0·65 to 1·90)
Vietnam	132·87 (106·06 to 162·52)	99·96 (84·97 to 117·11)	90·87 (77·80 to 105·13)	90·05 (75·77 to 105·54)	76·71 (63·14 to 91·62)	56·03 (46·73 to 66·47)	40·29 (35·31 to 45·59)	31·96 (26·70 to 38·07)	27·14 (21·46 to 34·20)	19·72 (14·91 to 26·17)
Oceania	17·57 (14·54 to 20·88)	17·73 (14·79 to 20·91)	16·79 (14·20 to 19·60)	16·73 (14·07 to 19·62)	16·92 (13·92 to 20·23)	16·99 (13·42 to 21·15)	16·91 (12·35 to 22·51)	16·85 (11·05 to 24·24)	15·60 (9·65 to 23·77)	13·53 (8·26 to 21·60)
American Samoa	0·04 (0·03 to 0·04)	0·03 (0·03 to 0·04)	0·03 (0·02 to 0·03)	0·02 (0·02 to 0·03)	0·03 (0·02 to 0·03)	0·03 (0·02 to 0·03)	0·02 (0·02 to 0·03)	0·02 (0·02 to 0·02)	0·02 (0·01 to 0·02)	0·01 (0·01 to 0·02)
Federated States of Micronesia	0·26 (0·18 to 0·34)	0·21 (0·15 to 0·29)	0·21 (0·16 to 0·29)	0·20 (0·15 to 0·26)	0·16 (0·12 to 0·22)	0·13 (0·09 to 0·17)	0·10 (0·07 to 0·14)	0·05 (0·03 to 0·08)	0·04 (0·02 to 0·06)	0·03 (0·02 to 0·05)
Fiji	0·93 (0·82 to 1·04)	0·99 (0·79 to 1·20)	0·80 (0·61 to 1·03)	0·63 (0·45 to 0·83)	0·48 (0·32 to 0·70)	0·45 (0·32 to 0·60)	0·43 (0·33 to 0·53)	0·44 (0·34 to 0·55)	0·43 (0·32 to 0·57)	0·38 (0·22 to 0·62)
Guam	0·07 (0·07 to 0·08)	0·07 (0·06 to 0·07)	0·05 (0·04 to 0·05)	0·04 (0·04 to 0·05)	0·04 (0·04 to 0·05)	0·04 (0·04 to 0·05)	0·04 (0·03 to 0·05)	0·04 (0·04 to 0·05)	0·05 (0·04 to 0·05)	0·05 (0·03 to 0·06)
Kiribati	0·30 (0·22 to 0·38)	0·26 (0·17 to 0·39)	0·26 (0·15 to 0·44)	0·26 (0·16 to 0·41)	0·25 (0·18 to 0·33)	0·22 (0·17 to 0·27)	0·19 (0·15 to 0·24)	0·18 (0·13 to 0·25)	0·17 (0·11 to 0·26)	0·14 (0·07 to 0·24)

Appendix Table 12A. Under-5 death numbers by location, both sexes combined, 1970, 1975, 1980, 1985, 1990, 1995, 2000, 2005, 2010, 2016										
**Location**	**1970**	**1975**	**1980**	**1985**	**1990**	**1995**	**2000**	**2005**	**2010**	**2016**
Marshall Islands	0·07 (0·04 to 0·12)	0·07 (0·04 to 0·12)	0·06 (0·04 to 0·10)	0·07 (0·05 to 0·10)	0·07 (0·05 to 0·09)	0·06 (0·04 to 0·09)	0·08 (0·05 to 0·11)	0·06 (0·04 to 0·09)	0·06 (0·03 to 0·10)	0·05 (0·02 to 0·08)
Northern Mariana Islands	0·01 (<0·01 to 0·01)	0·01 (<0·01 to 0·01)	0·01 (<0·01 to 0·01)	0·01 (<0·01 to 0·02)	0·01 (0·01 to 0·02)	0·01 (0·01 to 0·02)	0·01 (0·01 to 0·02)	0·01 (0·01 to 0·01)	0·01 (<0·01 to 0·01)	0·01 (<0·01 to 0·01)
Papua New Guinea	12·94 (10·17 to 15·97)	13·45 (10·78 to 16·41)	13·15 (10·81 to 15·69)	13·34 (10·93 to 16·08)	13·82 (11·09 to 16·93)	14·19 (10·88 to 18·20)	14·27 (10·03 to 19·67)	14·35 (8·79 to 21·51)	13·43 (7·64 to 21·30)	11·59 (6·53 to 19·40)
Samoa	0·33 (0·25 to 0·42)	0·26 (0·18 to 0·37)	0·18 (0·11 to 0·31)	0·16 (0·08 to 0·29)	0·13 (0·06 to 0·24)	0·10 (0·05 to 0·20)	0·09 (0·04 to 0·17)	0·08 (0·04 to 0·15)	0·07 (0·03 to 0·13)	0·06 (0·03 to 0·11)
Solomon Islands	0·76 (0·55 to 0·98)	0·71 (0·48 to 1·01)	0·55 (0·36 to 0·83)	0·50 (0·34 to 0·71)	0·50 (0·34 to 0·70)	0·50 (0·36 to 0·69)	0·53 (0·38 to 0·73)	0·58 (0·39 to 0·80)	0·54 (0·35 to 0·81)	0·43 (0·27 to 0·66)
Tonga	0·12 (0·08 to 0·17)	0·11 (0·07 to 0·15)	0·11 (0·07 to 0·15)	0·09 (0·06 to 0·14)	0·09 (0·05 to 0·14)	0·08 (0·04 to 0·13)	0·07 (0·04 to 0·12)	0·07 (0·04 to 0·12)	0·07 (0·04 to 0·11)	0·05 (0·03 to 0·09)
Vanuatu	0·37 (0·25 to 0·52)	0·33 (0·22 to 0·48)	0·29 (0·19 to 0·42)	0·27 (0·19 to 0·36)	0·26 (0·20 to 0·33)	0·26 (0·19 to 0·33)	0·28 (0·21 to 0·35)	0·31 (0·23 to 0·40)	0·30 (0·21 to 0·41)	0·27 (0·17 to 0·41)
North Africa and Middle East	1,612·18 (1,500·92 to 1,736·49)	1,405·97 (1,304·24 to 1,495·41)	1,225·13 (1,139·14 to 1,313·87)	1,055·52 (981·44 to 1,128·22)	805·96 (756·02 to 857·73)	672·74 (623·47 to 728·99)	569·22 (515·91 to 621·10)	494·48 (444·82 to 550·52)	441·29 (389·87 to 496·34)	357·07 (298·18 to 421·63)
North Africa and Middle East	1,612·18 (1,500·92 to 1,736·49)	1,405·97 (1,304·24 to 1,495·41)	1,225·13 (1,139·14 to 1,313·87)	1,055·52 (981·44 to 1,128·22)	805·96 (756·02 to 857·73)	672·74 (623·47 to 728·99)	569·22 (515·91 to 621·10)	494·48 (444·82 to 550·52)	441·29 (389·87 to 496·34)	357·07 (298·18 to 421·63)
Afghanistan	126·11 (87·97 to 164·00)	133·30 (96·15 to 173·72)	136·13 (102·60 to 172·45)	110·88 (83·62 to 138·31)	79·71 (59·95 to 98·66)	95·03 (69·94 to 118·24)	100·10 (76·88 to 122·45)	98·23 (76·04 to 120·75)	81·04 (59·64 to 103·31)	74·79 (49·40 to 103·93)
Algeria	103·23 (85·23 to 121·58)	91·06 (74·32 to 107·59)	74·05 (60·71 to 87·25)	54·52 (43·50 to 65·63)	41·37 (32·87 to 50·52)	37·29 (28·21 to 48·30)	34·01 (25·91 to 43·14)	29·37 (22·85 to 36·33)	23·98 (17·16 to 31·90)	16·03 (10·49 to 23·80)
Bahrain	0·65 (0·47 to 0·84)	0·46 (0·36 to 0·56)	0·31 (0·28 to 0·34)	0·28 (0·26 to 0·30)	0·31 (0·29 to 0·33)	0·24 (0·22 to 0·26)	0·17 (0·15 to 0·18)	0·16 (0·14 to 0·17)	0·15 (0·13 to 0·16)	0·13 (0·10 to 0·17)
Egypt	351·26 (315·15 to 389·36)	317·26 (284·04 to 352·38)	258·52 (225·82 to 291·52)	194·87 (174·08 to 217·67)	142·63 (128·26 to 158·14)	101·06 (90·08 to 113·01)	75·77 (65·61 to 86·72)	66·39 (56·29 to 77·36)	60·68 (50·04 to 73·34)	41·67 (28·52 to 60·81)
Iran	320·82 (244·46 to 402·30)	208·43 (160·75 to 262·54)	182·46 (137·96 to 230·52)	164·11 (129·32 to 200·56)	133·04 (107·65 to 161·32)	73·63 (54·87 to 96·22)	52·06 (34·10 to 74·91)	44·45 (21·39 to 78·73)	37·57 (15·43 to 70·78)	27·87 (11·24 to 59·62)
Iraq	54·79 (29·48 to 72·93)	51·12 (27·17 to 66·99)	45·58 (22·97 to 60·24)	44·37 (22·49 to 59·83)	34·62 (14·56 to 50·06)	37·79 (16·00 to 57·74)	35·53 (18·68 to 52·15)	39·18 (26·79 to 52·40)	45·68 (26·29 to 62·83)	46·39 (22·89 to 69·72)
Jordan	9·05 (8·13 to 9·98)	8·04 (7·40 to 8·70)	5·38 (4·96 to 5·82)	4·32 (3·83 to 4·84)	3·75 (3·31 to 4·18)	4·27 (3·70 to 4·91)	3·85 (3·39 to 4·31)	3·59 (3·19 to 4·03)	3·58 (3·08 to 4·11)	3·38 (2·69 to 4·28)
Kuwait	1·76 (1·68 to 1·84)	2·04 (1·96 to 2·12)	1·66 (1·58 to 1·74)	1·16 (1·11 to 1·22)	0·68 (0·58 to 0·78)	0·57 (0·53 to 0·60)	0·54 (0·50 to 0·57)	0·53 (0·50 to 0·57)	0·62 (0·58 to 0·67)	0·52 (0·36 to 0·73)
Lebanon	6·19 (4·92 to 7·81)	6·55 (4·99 to 8·19)	6·54 (4·31 to 8·93)	3·56 (2·68 to 4·56)	2·41 (1·96 to 2·96)	1·64 (1·24 to 2·12)	1·06 (0·77 to 1·40)	0·74 (0·49 to 1·07)	0·62 (0·40 to 0·90)	0·64 (0·44 to 0·89)
Libya	8·77 (7·17 to 10·39)	7·09 (6·00 to 8·21)	5·62 (4·68 to 6·69)	4·83 (3·77 to 5·98)	4·42 (3·42 to 5·52)	3·95 (3·14 to 4·82)	3·60 (2·70 to 4·58)	2·94 (2·08 to 3·91)	1·97 (1·34 to 2·71)	1·15 (0·78 to 1·62)
Morocco	103·12 (95·84 to 111·05)	91·87 (85·65 to 97·81)	79·55 (74·16 to 85·40)	66·55 (61·90 to 71·15)	52·90 (48·64 to 57·15)	39·92 (35·70 to 44·19)	29·12 (25·19 to 33·24)	21·16 (17·46 to 25·51)	15·16 (11·81 to 19·31)	9·72 (7·18 to 12·90)
Palestine	4·56 (2·63 to 6·45)	4·34 (2·23 to 6·11)	3·83 (1·63 to 5·45)	3·07 (1·23 to 4·49)	3·35 (1·42 to 4·90)	2·97 (1·45 to 4·22)	3·09 (1·40 to 4·69)	3·15 (1·67 to 4·72)	3·98 (2·48 to 5·45)	3·85 (1·87 to 6·03)
Oman	10·28 (8·94 to 11·71)	8·94 (7·49 to 10·36)	7·17 (5·78 to 8·59)	5·16 (4·18 to 6·27)	3·49 (2·79 to 4·35)	2·26 (1·80 to 2·87)	1·44 (1·18 to 1·75)	0·71 (0·61 to 0·82)	0·75 (0·63 to 0·87)	0·73 (0·53 to 0·97)
Qatar	0·28 (0·19 to 0·39)	0·30 (0·23 to 0·39)	0·25 (0·17 to 0·35)	0·25 (0·19 to 0·33)	0·24 (0·17 to 0·33)	0·21 (0·15 to 0·28)	0·18 (0·12 to 0·24)	0·19 (0·13 to 0·26)	0·24 (0·16 to 0·33)	0·24 (0·16 to 0·36)
Saudi Arabia	42·04 (36·89 to 47·44)	37·83 (34·37 to 41·60)	34·27 (30·39 to 38·38)	33·89 (29·47 to 39·11)	29·53 (25·36 to 34·40)	21·70 (18·11 to 26·39)	16·29 (13·49 to 20·11)	11·11 (9·25 to 13·61)	6·88 (5·68 to 8·38)	3·10 (2·47 to 3·92)
Sudan	73·27 (59·93 to 87·31)	79·40 (61·83 to 95·61)	83·56 (66·24 to 101·50)	125·85 (93·14 to 158·75)	79·79 (68·35 to 92·79)	84·60 (70·05 to 98·99)	83·00 (67·41 to 100·69)	72·44 (54·03 to 90·88)	69·65 (50·73 to 91·23)	54·99 (30·70 to 93·08)
Syria	27·94 (25·10 to 30·71)	25·42 (22·13 to 28·53)	22·45 (19·25 to 25·66)	20·57 (16·92 to 24·72)	18·84 (15·24 to 22·40)	13·78 (11·59 to 16·00)	9·09 (7·72 to 10·43)	7·60 (6·40 to 8·96)	7·81 (6·39 to 9·44)	8·87 (4·45 to 13·37)
Tunisia	28·37 (25·85 to 30·98)	22·77 (21·20 to 24·44)	18·50 (17·04 to 20·03)	14·45 (12·82 to 16·23)	10·59 (9·49 to 11·72)	7·00 (6·18 to 7·89)	4·49 (3·90 to 5·14)	3·51 (3·01 to 4·07)	3·11 (2·54 to 3·72)	1·96 (1·45 to 2·55)
Turkey	243·01 (206·87 to 278·79)	227·23 (189·85 to 267·37)	186·72 (150·06 to 227·83)	134·76 (106·21 to 164·36)	95·06 (76·78 to 115·63)	74·09 (61·23 to 88·10)	52·89 (43·78 to 63·14)	36·74 (29·85 to 45·09)	28·19 (20·97 to 37·53)	18·90 (13·02 to 26·76)
United Arab Emirates	1·20 (0·82 to 1·67)	1·47 (0·97 to 2·21)	1·92 (1·22 to 2·82)	1·72 (0·96 to 2·82)	1·26 (0·67 to 2·10)	1·04 (0·53 to 1·87)	0·79 (0·42 to 1·35)	0·64 (0·51 to 0·81)	1·08 (0·61 to 1·74)	0·90 (0·47 to 1·60)
Yemen	94·82 (45·89 to 122·20)	80·57 (39·53 to 102·96)	69·95 (35·92 to 88·74)	65·74 (36·38 to 82·52)	67·46 (47·64 to 81·62)	69·24 (42·26 to 89·86)	61·82 (33·04 to 84·32)	51·25 (28·19 to 72·09)	48·20 (26·90 to 68·84)	40·98 (18·76 to 65·89)
South Asia	5,366·38 (5,209·66 to 5,534·73)	5,067·42 (4,928·83 to 5,197·21)	4,794·04 (4,650·25 to 4,937·27)	4,437·44 (4,299·02 to 4,575·73)	3,915·49 (3,792·26 to 4,032·64)	3,380·94 (3,284·53 to 3,479·37)	2,892·66 (2,826·07 to 2,960·31)	2,472·16 (2,408·66 to 2,538·15)	1,960·35 (1,887·41 to 2,033·09)	1,240·45 (1,164·16 to 1,319·65)
South Asia	5,366·38 (5,209·66 to 5,534·73)	5,067·42 (4,928·83 to 5,197·21)	4,794·04 (4,650·25 to 4,937·27)	4,437·44 (4,299·02 to 4,575·73)	3,915·49 (3,792·26 to 4,032·64)	3,380·94 (3,284·53 to 3,479·37)	2,892·66 (2,826·07 to 2,960·31)	2,472·16 (2,408·66 to 2,538·15)	1,960·35 (1,887·41 to 2,033·09)	1,240·45 (1,164·16 to 1,319·65)
Bangladesh	631·13 (580·00 to 683·80)	552·88 (513·72 to 593·17)	533·92 (500·32 to 570·03)	521·72 (489·36 to 554·57)	462·90 (437·05 to 487·89)	361·54 (339·40 to 384·53)	278·32 (258·98 to 298·02)	221·16 (204·62 to 239·51)	161·53 (146·65 to 178·16)	96·48 (83·39 to 111·49)
Bhutan	3·53 (2·98 to 4·09)	3·55 (3·05 to 4·11)	3·44 (2·91 to 4·00)	3·06 (2·56 to 3·61)	2·70 (2·32 to 3·08)	2·04 (1·75 to 2·31)	1·68 (1·43 to 1·98)	1·30 (1·10 to 1·54)	0·89 (0·72 to 1·09)	0·51 (0·40 to 0·64)
India	4,164·68 (4,029·85 to 4,316·67)	3,939·89 (3,818·38 to 4,059·02)	3,662·56 (3,530·39 to 3,792·57)	3,294·63 (3,166·00 to 3,418·50)	2,880·69 (2,780·09 to 2,982·31)	2,523·01 (2,444·22 to 2,604·31)	2,191·29 (2,141·40 to 2,246·55)	1,862·70 (1,811·61 to 1,916·17)	1,425·38 (1,372·17 to 1,477·86)	865·58 (817·51 to 919·13)
Nepal	120·83 (91·07 to 148·93)	121·83 (92·56 to 151·32)	119·47 (89·92 to 147·46)	110·90 (85·95 to 134·55)	97·84 (76·31 to 120·09)	78·84 (63·81 to 94·28)	62·77 (50·01 to 76·22)	45·21 (35·45 to 55·30)	34·63 (27·77 to 41·99)	24·30 (17·91 to 31·92)
Pakistan	446·23 (393·91 to 494·09)	449·27 (404·72 to 493·20)	474·64 (436·20 to 514·47)	507·12 (467·88 to 548·00)	471·35 (421·80 to 521·93)	415·52 (367·54 to 461·60)	358·60 (326·25 to 393·15)	341·80 (307·25 to 378·59)	337·92 (289·98 to 392·14)	253·58 (196·10 to 318·21)
Sub-Saharan Africa	2,928·99 (2,750·59 to 3,100·66)	3,021·29 (2,838·87 to 3,195·05)	3,096·38 (2,930·95 to 3,239·41)	3,204·60 (3,051·82 to 3,347·91)	3,352·00 (3,205·75 to 3,481·76)	3,485·63 (3,302·04 to 3,661·63)	3,520·81 (3,332·03 to 3,699·38)	3,396·64 (3,224·25 to 3,563·92)	3,169·59 (2,981·77 to 3,368·22)	2,654·30 (2,464·11 to 2,878·44)
Southern Sub-Saharan Africa	116·35 (103·97 to 131·00)	117·15 (105·31 to 130·08)	119·30 (107·96 to 131·87)	115·64 (104·58 to 128·48)	106·79 (99·05 to 115·01)	104·46 (98·84 to 110·22)	118·28 (110·35 to 126·98)	129·23 (117·72 to 141·80)	115·16 (102·56 to 129·19)	86·51 (73·63 to 101·50)
Botswana	2·55 (1·81 to 3·36)	2·34 (1·60 to 3·08)	2·29 (1·61 to 2·94)	2·19 (1·50 to 2·90)	1·99 (1·41 to 2·63)	2·50 (1·62 to 3·44)	2·91 (1·77 to 4·40)	2·28 (1·45 to 3·30)	1·41 (0·94 to 2·01)	0·81 (0·47 to 1·33)
Lesotho	4·39 (3·58 to 5·20)	4·42 (3·59 to 5·33)	4·34 (3·42 to 5·42)	4·32 (3·30 to 5·40)	4·02 (3·07 to 5·06)	4·26 (3·33 to 5·26)	5·20 (4·14 to 6·37)	5·84 (4·94 to 6·90)	4·89 (3·99 to 5·94)	3·70 (2·78 to 4·90)
Namibia	3·11 (2·42 to 3·94)	3·29 (2·76 to 3·93)	3·52 (3·02 to 4·10)	3·29 (2·83 to 3·81)	3·67 (3·22 to 4·23)	3·46 (2·99 to 3·96)	3·89 (3·32 to 4·61)	3·92 (3·29 to 4·63)	3·50 (2·75 to 4·39)	2·60 (1·65 to 3·85)
South Africa	82·40 (70·16 to 97·01)	82·70 (71·20 to 95·62)	83·84 (73·05 to 96·27)	81·15 (70·51 to 93·26)	71·53 (64·07 to 79·34)	65·12 (60·24 to 69·98)	73·20 (66·03 to 80·45)	78·86 (68·49 to 90·62)	63·87 (52·52 to 77·50)	46·30 (35·52 to 60·49)
Swaziland	3·27 (2·26 to 4·52)	2·89 (2·11 to 3·84)	2·56 (2·01 to 3·23)	2·24 (1·85 to 2·64)	1·99 (1·64 to 2·35)	2·15 (1·79 to 2·51)	3·11 (2·60 to 3·62)	3·74 (3·17 to 4·34)	3·16 (2·74 to 3·65)	2·17 (1·65 to 2·77)
Zimbabwe	20·64 (18·09 to 23·38)	21·51 (19·35 to 23·74)	22·74 (20·53 to 24·89)	22·45 (20·17 to 24·59)	23·57 (21·57 to 25·58)	26·97 (24·60 to 29·54)	29·97 (26·28 to 33·79)	34·59 (31·36 to 38·29)	38·32 (34·02 to 42·93)	30·93 (25·18 to 37·94)
Western Sub-Saharan Africa	1,419·32 (1,280·49 to 1,560·11)	1,418·66 (1,288·55 to 1,542·34)	1,418·90 (1,307·66 to 1,532·12)	1,447·05 (1,342·16 to 1,548·44)	1,529·89 (1,448·02 to 1,616·54)	1,622·88 (1,510·18 to 1,736·30)	1,681·32 (1,563·64 to 1,792·93)	1,689·54 (1,567·58 to 1,825·53)	1,626·05 (1,484·40 to 1,772·26)	1,403·81 (1,260·69 to 1,553·70)
Benin	36·91 (33·78 to 40·19)	35·79 (32·74 to 38·72)	35·60 (33·01 to 38·17)	36·01 (33·49 to 38·68)	36·70 (33·46 to 39·98)	37·80 (33·80 to 41·86)	37·28 (33·10 to 41·49)	36·33 (32·62 to 40·79)	34·39 (29·90 to 39·72)	30·75 (25·04 to 37·67)
Burkina Faso	83·72 (59·94 to 102·65)	83·08 (60·09 to 102·25)	80·62 (60·16 to 98·27)	81·30 (61·71 to 97·77)	83·77 (68·41 to 96·41)	86·88 (73·20 to 99·46)	88·83 (71·46 to 104·14)	90·23 (68·88 to 109·89)	88·58 (66·70 to 109·03)	77·80 (54·45 to 100·77)

Appendix Table 12A. Under-5 death numbers by location, both sexes combined, 1970, 1975, 1980, 1985, 1990, 1995, 2000, 2005, 2010, 2016										
**Location**	**1970**	**1975**	**1980**	**1985**	**1990**	**1995**	**2000**	**2005**	**2010**	**2016**
Cameroon	62·91 (58·37 to 67·57)	60·88 (56·19 to 65·39)	60·08 (55·41 to 64·92)	59·60 (55·24 to 64·34)	63·26 (58·39 to 68·19)	72·37 (65·94 to 78·92)	78·31 (69·44 to 88·32)	81·68 (71·51 to 92·82)	82·65 (70·24 to 96·43)	75·66 (60·31 to 95·30)
Cape Verde	1·62 (1·51 to 1·74)	1·19 (1·12 to 1·27)	0·87 (0·83 to 0·92)	1·04 (0·97 to 1·11)	0·80 (0·73 to 0·87)	0·95 (0·74 to 1·16)	0·61 (0·52 to 0·71)	0·49 (0·41 to 0·58)	0·36 (0·30 to 0·42)	0·26 (0·22 to 0·31)
Chad	43·45 (39·05 to 48·08)	45·08 (41·54 to 48·93)	47·27 (44·22 to 50·41)	49·43 (46·48 to 52·51)	50·71 (47·64 to 53·97)	54·89 (51·05 to 58·67)	62·91 (58·38 to 67·53)	67·59 (61·95 to 73·29)	69·32 (61·84 to 77·92)	69·12 (59·74 to 80·70)
Cote d'Ivoire	49·58 (42·35 to 56·22)	50·56 (43·85 to 56·74)	52·82 (47·53 to 58·97)	62·25 (55·79 to 68·30)	69·04 (59·89 to 77·99)	74·37 (64·98 to 84·01)	78·02 (67·94 to 88·28)	78·88 (62·74 to 95·10)	75·21 (59·05 to 91·91)	68·57 (50·87 to 87·78)
The Gambia	4·59 (3·86 to 5·44)	4·55 (3·84 to 5·33)	4·57 (3·93 to 5·24)	4·44 (3·80 to 5·09)	4·80 (4·09 to 5·51)	4·95 (4·21 to 5·71)	4·93 (4·23 to 5·61)	4·65 (3·90 to 5·40)	4·25 (3·54 to 5·09)	3·85 (3·07 to 4·73)
Ghana	65·96 (57·25 to 74·60)	64·39 (54·91 to 73·51)	64·22 (55·55 to 72·66)	69·20 (60·29 to 77·89)	67·74 (60·71 to 74·65)	63·26 (55·55 to 71·12)	60·51 (51·97 to 69·62)	63·09 (55·04 to 72·57)	63·27 (55·14 to 72·15)	48·35 (38·13 to 60·17)
Guinea	55·33 (48·59 to 62·21)	55·32 (50·68 to 60·46)	52·04 (48·48 to 55·79)	51·77 (48·62 to 55·01)	52·60 (49·67 to 55·71)	58·72 (54·96 to 62·66)	57·11 (52·91 to 61·56)	54·08 (49·06 to 59·30)	50·61 (44·45 to 57·69)	44·46 (36·76 to 54·58)
Guinea-Bissau	9·06 (7·93 to 10·36)	9·02 (7·97 to 10·18)	8·99 (8·06 to 9·98)	8·99 (8·22 to 9·81)	8·56 (7·92 to 9·29)	8·14 (7·51 to 8·77)	7·76 (7·13 to 8·40)	7·60 (6·85 to 8·40)	6·23 (5·55 to 6·92)	4·88 (4·21 to 5·63)
Liberia	20·25 (18·10 to 22·63)	20·26 (18·27 to 22·31)	20·87 (18·86 to 23·01)	22·29 (20·17 to 24·37)	20·81 (19·08 to 22·69)	19·41 (17·76 to 21·10)	19·94 (18·28 to 21·68)	15·67 (14·23 to 17·16)	13·24 (11·71 to 14·92)	10·15 (8·42 to 12·12)
Mali	101·65 (94·62 to 109·07)	100·93 (94·89 to 106·90)	96·33 (91·07 to 101·83)	95·42 (90·13 to 100·66)	90·64 (85·96 to 95·39)	92·32 (86·95 to 98·18)	95·06 (88·78 to 101·94)	93·99 (86·51 to 102·26)	89·83 (80·35 to 100·41)	84·21 (69·73 to 102·12)
Mauritania	7·24 (6·88 to 7·64)	7·58 (7·18 to 8·00)	7·51 (7·07 to 7·96)	7·45 (6·91 to 8·00)	7·34 (6·79 to 7·90)	7·45 (6·92 to 7·98)	7·69 (7·10 to 8·29)	7·90 (7·15 to 8·70)	6·89 (6·03 to 7·80)	5·55 (4·74 to 6·51)
Niger	78·24 (71·67 to 85·25)	87·60 (80·57 to 94·56)	94·87 (87·36 to 102·59)	104·01 (96·31 to 112·44)	111·49 (104·40 to 118·76)	118·03 (110·19 to 125·69)	115·67 (106·83 to 124·71)	106·00 (96·42 to 116·16)	97·14 (85·16 to 111·33)	91·69 (76·38 to 110·92)
Nigeria	695·85 (561·82 to 835·53)	688·90 (557·58 to 810·96)	691·59 (577·68 to 805·64)	693·63 (599·92 to 794·84)	761·28 (681·70 to 839·20)	824·58 (711·60 to 932·68)	872·73 (756·93 to 986·41)	887·37 (767·36 to 1,020·05)	857·94 (719·12 to 1,003·82)	717·18 (580·77 to 862·55)
Sao Tome and Principe	0·44 (0·42 to 0·46)	0·38 (0·35 to 0·41)	0·42 (0·39 to 0·45)	0·50 (0·48 to 0·53)	0·46 (0·43 to 0·50)	0·44 (0·40 to 0·48)	0·39 (0·35 to 0·44)	0·35 (0·30 to 0·40)	0·26 (0·23 to 0·30)	0·21 (0·18 to 0·25)
Senegal	48·49 (44·24 to 53·33)	49·20 (45·50 to 52·86)	45·70 (42·20 to 49·18)	43·07 (39·85 to 46·30)	41·40 (38·54 to 44·28)	42·52 (40·00 to 45·11)	39·78 (37·02 to 42·52)	37·82 (34·63 to 41·13)	33·26 (30·00 to 36·70)	27·14 (23·55 to 31·19)
Sierra Leone	34·34 (30·27 to 39·18)	34·45 (30·71 to 38·41)	35·68 (32·59 to 38·82)	36·51 (34·11 to 39·01)	37·17 (35·11 to 39·41)	34·40 (32·59 to 36·15)	32·44 (30·54 to 34·29)	34·28 (31·84 to 36·64)	32·70 (29·66 to 35·70)	27·47 (24·10 to 31·23)
Togo	19·62 (16·41 to 22·94)	19·47 (16·28 to 22·55)	18·82 (15·31 to 22·29)	20·12 (17·15 to 23·22)	21·29 (18·61 to 24·16)	21·38 (18·36 to 24·70)	21·35 (17·60 to 25·28)	21·54 (16·31 to 27·25)	19·91 (15·50 to 24·43)	16·50 (12·83 to 20·49)
Eastern Sub-Saharan Africa	1,091·97 (1,048·33 to 1,135·33)	1,175·08 (1,131·91 to 1,219·09)	1,237·72 (1,196·03 to 1,278·22)	1,320·19 (1,280·23 to 1,358·07)	1,350·67 (1,313·05 to 1,389·45)	1,353·05 (1,312·54 to 1,395·23)	1,288·20 (1,251·16 to 1,326·56)	1,153·76 (1,114·92 to 1,192·29)	1,017·04 (975·31 to 1,058·16)	813·26 (764·61 to 864·97)
Burundi	40·18 (34·52 to 45·75)	41·04 (36·38 to 46·25)	42·56 (37·73 to 47·65)	41·98 (37·75 to 46·50)	44·32 (39·57 to 49·53)	44·97 (40·29 to 49·96)	42·86 (37·76 to 47·95)	39·21 (34·34 to 44·50)	38·14 (31·41 to 45·58)	38·83 (27·05 to 56·24)
Comoros	1·86 (1·54 to 2·20)	1·87 (1·64 to 2·11)	1·99 (1·77 to 2·23)	1·90 (1·69 to 2·12)	1·76 (1·56 to 1·94)	1·66 (1·48 to 1·85)	1·73 (1·52 to 1·97)	1·59 (1·34 to 1·87)	1·40 (1·15 to 1·66)	1·01 (0·77 to 1·35)
Djibouti	1·11 (0·91 to 1·32)	1·39 (1·17 to 1·63)	2·00 (1·69 to 2·31)	2·43 (2·08 to 2·78)	3·09 (2·59 to 3·56)	3·40 (2·81 to 3·99)	3·35 (2·77 to 3·92)	2·92 (2·31 to 3·63)	2·48 (1·80 to 3·33)	1·75 (1·15 to 2·62)
Eritrea	18·80 (16·02 to 21·97)	19·23 (16·60 to 21·73)	19·82 (17·61 to 22·10)	20·18 (17·73 to 22·84)	19·33 (17·00 to 21·75)	13·85 (11·97 to 15·83)	11·60 (9·85 to 13·57)	11·80 (9·48 to 14·31)	11·40 (8·92 to 14·37)	8·38 (6·21 to 11·18)
Ethiopia	286·99 (258·50 to 316·44)	324·61 (294·16 to 357·39)	324·83 (298·56 to 352·75)	331·28 (307·66 to 354·51)	354·84 (330·25 to 378·52)	356·05 (329·11 to 384·79)	334·31 (308·81 to 361·36)	280·60 (255·37 to 308·00)	215·63 (189·30 to 246·47)	144·77 (121·89 to 173·21)
Kenya	76·60 (74·76 to 78·43)	80·42 (78·60 to 82·31)	81·71 (79·46 to 83·75)	84·72 (82·68 to 86·71)	89·71 (87·23 to 92·08)	101·00 (98·37 to 103·88)	101·75 (99·06 to 104·74)	91·24 (88·73 to 94·01)	74·71 (71·74 to 77·69)	59·02 (56·17 to 62·40)
Madagascar	54·93 (48·21 to 63·36)	59·99 (52·88 to 68·03)	67·61 (60·42 to 75·50)	78·47 (69·48 to 87·63)	76·07 (68·68 to 83·73)	78·03 (69·76 to 86·70)	76·27 (67·22 to 85·79)	72·63 (62·69 to 82·34)	75·17 (62·77 to 87·88)	69·16 (53·90 to 88·77)
Malawi	84·67 (75·76 to 93·96)	82·52 (74·82 to 90·46)	80·04 (73·40 to 87·30)	82·71 (76·10 to 90·18)	97·79 (90·02 to 105·71)	87·07 (80·17 to 94·91)	79·57 (72·65 to 86·88)	65·54 (59·01 to 71·94)	61·04 (52·98 to 69·86)	48·69 (39·20 to 61·92)
Mozambique	106·54 (90·40 to 124·53)	111·66 (96·09 to 127·87)	123·81 (107·14 to 140·61)	148·57 (130·94 to 166·29)	116·02 (98·23 to 132·77)	123·76 (107·25 to 140·35)	120·00 (108·04 to 132·36)	111·66 (101·16 to 122·32)	103·16 (90·45 to 115·67)	82·92 (68·27 to 99·34)
Rwanda	45·04 (40·98 to 49·13)	52·69 (47·98 to 57·64)	54·62 (50·03 to 59·60)	52·06 (47·69 to 57·02)	55·69 (50·92 to 60·78)	51·71 (46·01 to 57·60)	48·59 (42·65 to 54·63)	38·76 (34·38 to 43·64)	26·00 (22·65 to 29·64)	19·49 (15·24 to 24·85)
Somalia	25·00 (17·75 to 33·34)	31·94 (24·68 to 40·34)	39·42 (30·91 to 49·14)	43·35 (33·73 to 53·10)	39·33 (29·93 to 49·79)	35·77 (26·56 to 45·44)	34·64 (26·92 to 42·99)	32·15 (25·60 to 39·47)	39·07 (31·40 to 47·72)	28·45 (20·37 to 37·13)
South Sudan	54·98 (41·35 to 72·30)	56·89 (43·66 to 72·18)	60·18 (48·91 to 73·23)	65·68 (55·92 to 75·85)	63·13 (53·47 to 72·88)	50·58 (43·27 to 58·03)	48·54 (42·37 to 54·25)	50·68 (43·92 to 58·31)	54·84 (45·17 to 66·05)	61·48 (46·95 to 79·96)
Tanzania	152·63 (138·07 to 167·05)	158·65 (144·51 to 172·36)	165·48 (149·54 to 181·50)	174·05 (158·50 to 189·98)	180·25 (162·88 to 197·21)	189·71 (172·32 to 206·57)	174·77 (161·51 to 188·78)	156·83 (142·22 to 171·91)	136·54 (116·80 to 157·29)	108·48 (86·93 to 132·08)
Uganda	101·43 (93·00 to 109·40)	110·29 (103·56 to 117·59)	126·69 (119·24 to 134·13)	136·68 (129·91 to 143·80)	145·70 (138·38 to 153·00)	151·36 (143·74 to 158·95)	147·75 (140·94 to 154·90)	142·73 (135·00 to 150·64)	130·97 (120·50 to 141·95)	103·11 (90·12 to 118·30)
Zambia	40·82 (36·00 to 46·09)	41·43 (36·98 to 45·92)	46·43 (42·10 to 51·28)	55·52 (50·74 to 61·19)	62·93 (57·48 to 68·56)	63·46 (57·72 to 69·50)	61·70 (54·99 to 68·09)	54·71 (48·52 to 61·02)	45·85 (39·48 to 53·07)	37·29 (29·72 to 46·29)
Central Sub-Saharan Africa	301·36 (196·50 to 405·05)	310·41 (202·96 to 416·93)	320·46 (219·89 to 409·90)	321·72 (222·99 to 406·43)	364·66 (249·86 to 462·49)	405·24 (278·86 to 518·83)	433·02 (296·86 to 554·55)	424·11 (313·96 to 528·82)	411·34 (286·86 to 534·49)	350·71 (236·86 to 481·88)
Angola	60·60 (42·35 to 82·20)	65·42 (49·42 to 83·39)	73·64 (59·19 to 90·78)	83·37 (70·33 to 97·72)	96·62 (81·97 to 111·92)	97·01 (82·18 to 113·41)	102·67 (87·05 to 119·61)	96·26 (80·92 to 114·04)	85·53 (68·93 to 104·78)	59·18 (39·12 to 85·06)
Central African Republic	13·22 (10·91 to 15·80)	14·38 (12·02 to 16·86)	14·99 (12·85 to 17·70)	16·90 (14·49 to 19·51)	18·58 (16·12 to 21·45)	20·78 (17·66 to 24·38)	23·37 (19·71 to 27·39)	23·66 (19·44 to 28·41)	23·72 (18·76 to 29·26)	22·78 (16·80 to 31·40)
Congo	9·75 (7·22 to 12·99)	9·45 (7·16 to 12·31)	8·87 (7·18 to 10·79)	8·10 (6·94 to 9·37)	8·64 (7·36 to 10·07)	10·23 (8·73 to 11·88)	12·08 (10·24 to 14·17)	11·25 (9·79 to 12·80)	10·08 (8·08 to 12·36)	8·42 (5·89 to 11·95)
Democratic Republic of the Congo	210·66 (107·13 to 317·45)	215·83 (111·23 to 319·40)	218·71 (118·45 to 307·44)	208·46 (112·18 to 292·24)	235·11 (123·44 to 331·80)	271·23 (146·18 to 382·00)	289·80 (160·11 to 407·57)	288·17 (178·80 to 391·17)	287·66 (163·85 to 407·51)	256·79 (148·94 to 383·25)
Equatorial Guinea	2·94 (2·15 to 3·83)	1·91 (1·51 to 2·36)	1·38 (1·12 to 1·68)	2·29 (1·82 to 2·84)	3·10 (2·49 to 3·87)	3·42 (2·78 to 4·13)	2·40 (1·88 to 3·01)	1·92 (1·39 to 2·56)	1·67 (1·14 to 2·33)	1·26 (0·83 to 1·88)
Gabon	4·18 (2·98 to 5·49)	3·43 (2·58 to 4·39)	2·87 (2·11 to 3·68)	2·61 (1·90 to 3·34)	2·60 (1·88 to 3·32)	2·57 (1·93 to 3·24)	2·68 (2·14 to 3·23)	2·85 (2·20 to 3·55)	2·68 (2·05 to 3·46)	2·29 (1·63 to 3·08)

Appendix Table 12B. Under-5 death numbers by location, males, 1970, 1975, 1980, 1985, 1990, 1995, 2000, 2005, 2010, 2016

Location	1970	1975	1980	1985	1990	1995	2000	2005	2010	2016
Global	8,731·04 (8,550·95 to 8,913·11)	7,808·33 (7,657·37 to 7,956·20)	7,018·65 (6,882·84 to 7,153·77)	6,482·72 (6,359·81 to 6,602·51)	5,862·67 (5,744·94 to 5,974·74)	5,246·08 (5,127·65 to 5,362·99)	4,662·01 (4,542·47 to 4,771·18)	4,103·16 (3,997·69 to 4,207·25)	3,556·82 (3,444·93 to 3,671·75)	2,708·08 (2,584·39 to 2,837·97)
High SDI	760·34 (708·19 to 816·17)	613·99 (576·19 to 649·73)	515·85 (481·93 to 550·04)	429·69 (404·59 to 455·54)	341·60 (322·34 to 361·96)	253·62 (239·35 to 270·04)	193·37 (181·43 to 208·26)	150·63 (136·29 to 170·88)	122·99 (109·39 to 143·05)	92·90 (81·46 to 111·71)
High-middle SDI	200·35 (195·53 to 206·02)	142·76 (138·88 to 147·14)	109·30 (106·30 to 112·78)	83·51 (81·84 to 85·47)	70·65 (69·48 to 72·33)	52·88 (52·09 to 53·82)	43·18 (42·93 to 43·42)	38·51 (38·30 to 38·73)	33·56 (33·37 to 33·76)	29·80 (28·81 to 30·89)
Middle SDI	3,463·17 (3,343·74 to 3,588·98)	3,258·38 (3,152·49 to 3,363·98)	3,036·98 (2,945·55 to 3,126·75)	2,904·57 (2,813·68 to 2,989·01)	2,670·16 (2,593·48 to 2,743·53)	2,455·99 (2,370·56 to 2,548·10)	2,215·24 (2,129·83 to 2,294·80)	1,956·41 (1,878·70 to 2,038·17)	1,678·18 (1,592·13 to 1,767·70)	1,217·59 (1,128·40 to 1,311·10)
Low-middle SDI	1,297·88 (1,219·10 to 1,377·08)	1,344·41 (1,271·45 to 1,413·55)	1,365·50 (1,293·19 to 1,431·93)	1,381·73 (1,315·03 to 1,451·61)	1,381·29 (1,313·17 to 1,447·26)	1,409·13 (1,325·30 to 1,479·94)	1,393·93 (1,314·65 to 1,464·42)	1,316·52 (1,246·37 to 1,379·30)	1,227·89 (1,151·18 to 1,300·37)	1,029·27 (952·97 to 1,107·11)
Low SDI	3,004·56 (2,916·60 to 3,098·33)	2,445·26 (2,379·46 to 2,515·14)	1,987·87 (1,929·58 to 2,049·29)	1,680·33 (1,634·75 to 1,725·82)	1,396·19 (1,362·35 to 1,434·32)	1,072·06 (1,046·91 to 1,098·30)	814·19 (794·48 to 832·50)	639·11 (622·25 to 655·54)	492·57 (476·07 to 508·51)	337·24 (319·06 to 357·80)
High-income	230·48 (225·38 to 236·34)	166·11 (162·32 to 170·43)	120·03 (117·00 to 123·72)	90·20 (88·52 to 92·15)	78·66 (77·50 to 80·37)	59·58 (58·79 to 60·52)	50·13 (49·57 to 50·71)	44·89 (44·44 to 45·38)	39·52 (39·09 to 39·92)	35·08 (33·85 to 36·28)
High-income North America	54·26 (53·58 to 54·97)	37·51 (37·12 to 37·90)	33·49 (33·28 to 33·70)	29·25 (29·08 to 29·42)	27·81 (27·63 to 28·01)	21·85 (21·71 to 22·00)	19·76 (19·63 to 19·91)	19·96 (19·83 to 20·10)	17·32 (17·21 to 17·43)	16·53 (16·05 to 16·99)
Canada	4·71 (4·59 to 4·82)	3·44 (3·35 to 3·54)	2·62 (2·55 to 2·70)	2·06 (2·00 to 2·12)	1·88 (1·82 to 1·94)	1·53 (1·48 to 1·58)	1·15 (1·11 to 1·20)	1·16 (1·11 to 1·21)	1·18 (1·14 to 1·22)	1·19 (0·96 to 1·46)
Greenland	0·04 (0·04 to 0·05)	0·02 (0·02 to 0·03)	0·02 (0·02 to 0·02)	0·02 (0·02 to 0·02)	0·02 (0·02 to 0·03)	0·02 (0·01 to 0·02)	0·01 (0·01 to 0·01)	0·01 (0·01 to 0·01)	0·01 (0·01 to 0·01)	0·01 (<0·01 to 0·01)
United States	49·48 (48·80 to 50·20)	34·02 (33·64 to 34·42)	30·83 (30·63 to 31·03)	27·15 (26·99 to 27·32)	25·89 (25·71 to 26·07)	20·30 (20·17 to 20·43)	18·59 (18·47 to 18·72)	18·78 (18·66 to 18·92)	16·12 (16·03 to 16·24)	15·33 (14·92 to 15·70)
Australasia	3·96 (3·88 to 4·03)	2·98 (2·92 to 3·04)	2·17 (2·12 to 2·22)	2·02 (1·97 to 2·07)	1·75 (1·70 to 1·80)	1·31 (1·28 to 1·35)	1·14 (1·10 to 1·17)	1·07 (1·04 to 1·10)	1·03 (1·00 to 1·06)	0·84 (0·74 to 0·95)
Australia	3·20 (3·13 to 3·28)	2·36 (2·31 to 2·43)	1·71 (1·66 to 1·75)	1·61 (1·57 to 1·65)	1·38 (1·34 to 1·42)	1·03 (0·99 to 1·06)	0·89 (0·86 to 0·92)	0·85 (0·82 to 0·88)	0·81 (0·78 to 0·84)	0·66 (0·56 to 0·76)
New Zealand	0·75 (0·73 to 0·78)	0·62 (0·60 to 0·64)	0·46 (0·45 to 0·48)	0·41 (0·39 to 0·42)	0·37 (0·36 to 0·38)	0·29 (0·27 to 0·30)	0·24 (0·23 to 0·26)	0·22 (0·21 to 0·23)	0·22 (0·21 to 0·23)	0·18 (0·15 to 0·22)
High-income Asia Pacific	49·52 (44·69 to 55·33)	33·99 (30·29 to 38·17)	21·39 (18·52 to 24·99)	12·91 (11·26 to 14·86)	9·24 (8·12 to 10·84)	7·29 (6·55 to 8·15)	5·35 (5·26 to 5·44)	3·73 (3·66 to 3·80)	2·99 (2·94 to 3·05)	2·25 (2·04 to 2·49)
Brunei	0·14 (0·13 to 0·14)	0·11 (0·10 to 0·11)	0·08 (0·07 to 0·08)	0·05 (0·05 to 0·06)	0·05 (0·04 to 0·05)	0·04 (0·04 to 0·05)	0·04 (0·04 to 0·04)	0·04 (0·03 to 0·04)	0·03 (0·03 to 0·04)	0·03 (0·03 to 0·04)
Japan	19·24 (18·71 to 19·76)	14·75 (14·37 to 15·11)	9·30 (9·20 to 9·39)	6·12 (6·05 to 6·19)	4·47 (4·41 to 4·53)	3·98 (3·89 to 4·09)	2·96 (2·92 to 3·00)	2·28 (2·24 to 2·32)	1·88 (1·85 to 1·91)	1·39 (1·32 to 1·45)
Singapore	0·72 (0·69 to 0·74)	0·44 (0·43 to 0·46)	0·35 (0·33 to 0·36)	0·26 (0·25 to 0·27)	0·21 (0·20 to 0·22)	0·14 (0·13 to 0·15)	0·10 (0·09 to 0·10)	0·06 (0·06 to 0·07)	0·06 (0·05 to 0·06)	0·05 (0·04 to 0·06)
South Korea	29·43 (24·77 to 35·09)	18·69 (14·89 to 22·87)	11·67 (8·82 to 15·27)	6·47 (4·82 to 8·37)	4·51 (3·38 to 6·13)	3·13 (2·41 to 3·99)	2·25 (2·17 to 2·32)	1·35 (1·29 to 1·41)	1·03 (0·98 to 1·07)	0·79 (0·59 to 1·01)
Western Europe	87·10 (86·48 to 87·71)	59·91 (59·49 to 60·37)	41·11 (40·77 to 41·44)	29·42 (29·19 to 29·66)	24·56 (24·34 to 24·80)	16·97 (16·79 to 17·16)	13·99 (13·84 to 14·14)	12·02 (11·90 to 12·16)	10·91 (10·79 to 11·04)	9·20 (8·45 to 9·99)
Andorra	<0·01 (<0·01 to <0·01)	<0·01 (<0·01 to <0·01)	<0·01 (<0·01 to <0·01)	<0·01 (<0·01 to <0·01)	<0·01 (<0·01 to <0·01)	<0·01 (<0·01 to <0·01)	<0·01 (<0·01 to <0·01)	<0·01 (<0·01 to <0·01)	<0·01 (<0·01 to <0·01)	<0·01 (<0·01 to <0·01)
Austria	1·93 (1·87 to 2·00)	1·29 (1·24 to 1·34)	0·89 (0·85 to 0·92)	0·67 (0·64 to 0·70)	0·50 (0·48 to 0·53)	0·35 (0·33 to 0·37)	0·26 (0·24 to 0·27)	0·22 (0·21 to 0·24)	0·20 (0·19 to 0·22)	0·16 (0·12 to 0·21)
Belgium	2·01 (1·98 to 2·05)	1·34 (1·31 to 1·37)	1·05 (1·03 to 1·08)	0·80 (0·77 to 0·82)	0·72 (0·70 to 0·74)	0·52 (0·50 to 0·53)	0·39 (0·38 to 0·41)	0·34 (0·33 to 0·35)	0·33 (0·32 to 0·34)	0·29 (0·25 to 0·35)
Cyprus	0·32 (0·28 to 0·37)	0·19 (0·18 to 0·20)	0·15 (0·14 to 0·15)	0·11 (0·10 to 0·11)	0·09 (0·08 to 0·10)	0·06 (0·06 to 0·07)	0·04 (0·03 to 0·04)	0·03 (0·03 to 0·03)	0·02 (0·02 to 0·03)	0·02 (0·01 to 0·03)
Denmark	0·74 (0·71 to 0·77)	0·52 (0·50 to 0·55)	0·35 (0·33 to 0·36)	0·30 (0·28 to 0·32)	0·33 (0·31 to 0·35)	0·26 (0·24 to 0·28)	0·21 (0·20 to 0·23)	0·18 (0·17 to 0·19)	0·14 (0·13 to 0·15)	0·13 (0·10 to 0·17)
Finland	0·61 (0·58 to 0·64)	0·48 (0·45 to 0·51)	0·33 (0·30 to 0·35)	0·26 (0·25 to 0·28)	0·25 (0·23 to 0·27)	0·18 (0·17 to 0·19)	0·14 (0·13 to 0·15)	0·13 (0·12 to 0·14)	0·10 (0·09 to 0·11)	0·07 (0·05 to 0·10)
France	9·01 (8·83 to 9·18)	7·13 (6·98 to 7·27)	5·68 (5·55 to 5·81)	4·47 (4·36 to 4·57)	3·94 (3·84 to 4·04)	2·56 (2·49 to 2·64)	2·36 (2·29 to 2·44)	1·96 (1·89 to 2·02)	1·89 (1·82 to 1·96)	1·71 (1·32 to 2·22)
Germany	15·98 (15·70 to 16·26)	10·10 (9·88 to 10·33)	7·41 (7·24 to 7·58)	5·22 (5·09 to 5·36)	4·52 (4·40 to 4·66)	2·91 (2·80 to 3·01)	2·32 (2·24 to 2·39)	1·85 (1·79 to 1·91)	1·58 (1·53 to 1·64)	1·42 (1·10 to 1·85)
Greece	2·68 (2·61 to 2·74)	2·11 (2·06 to 2·17)	1·63 (1·59 to 1·68)	1·01 (0·97 to 1·04)	0·62 (0·60 to 0·65)	0·50 (0·48 to 0·52)	0·36 (0·35 to 0·38)	0·27 (0·26 to 0·29)	0·25 (0·24 to 0·27)	0·20 (0·15 to 0·26)
Iceland	0·04 (0·04 to 0·04)	0·03 (0·03 to 0·04)	0·02 (0·02 to 0·03)	0·02 (0·02 to 0·02)	0·02 (0·02 to 0·02)	0·01 (0·01 to 0·02)	0·01 (0·01 to 0·01)	0·01 (0·01 to 0·01)	0·01 (0·01 to 0·01)	0·01 (<0·01 to 0·01)
Ireland	0·84 (0·81 to 0·88)	0·78 (0·75 to 0·81)	0·58 (0·56 to 0·61)	0·39 (0·36 to 0·41)	0·29 (0·27 to 0·30)	0·21 (0·19 to 0·22)	0·23 (0·21 to 0·24)	0·17 (0·16 to 0·19)	0·18 (0·17 to 0·19)	0·14 (0·10 to 0·20)
Israel	1·20 (1·12 to 1·28)	1·37 (1·33 to 1·42)	0·95 (0·92 to 0·99)	0·79 (0·76 to 0·82)	0·67 (0·65 to 0·70)	0·55 (0·53 to 0·58)	0·51 (0·49 to 0·53)	0·44 (0·43 to 0·46)	0·41 (0·39 to 0·43)	0·33 (0·29 to 0·39)
Italy	16·97 (16·66 to 17·32)	11·04 (10·81 to 11·26)	6·11 (5·96 to 6·26)	3·92 (3·81 to 4·04)	2·99 (2·90 to 3·10)	2·17 (2·09 to 2·25)	1·58 (1·51 to 1·66)	1·32 (1·27 to 1·37)	1·22 (1·17 to 1·27)	0·90 (0·61 to 1·28)
Luxembourg	0·06 (0·06 to 0·07)	0·04 (0·04 to 0·04)	0·03 (0·03 to 0·04)	0·03 (0·02 to 0·03)	0·03 (0·02 to 0·03)	0·02 (0·02 to 0·02)	0·01 (0·01 to 0·02)	0·01 (0·01 to 0·01)	0·01 (0·01 to 0·01)	0·01 (0·01 to 0·01)
Malta	0·09 (0·08 to 0·09)	0·07 (0·06 to 0·08)	0·06 (0·05 to 0·06)	0·05 (0·04 to 0·05)	0·04 (0·03 to 0·04)	0·03 (0·03 to 0·03)	0·02 (0·02 to 0·02)	0·02 (0·01 to 0·02)	0·02 (0·01 to 0·02)	0·01 (0·01 to 0·02)
Netherlands	2·19 (2·12 to 2·27)	1·43 (1·38 to 1·48)	1·12 (1·08 to 1·16)	1·01 (0·97 to 1·05)	0·97 (0·94 to 1·01)	0·77 (0·73 to 0·80)	0·73 (0·70 to 0·76)	0·60 (0·57 to 0·63)	0·46 (0·44 to 0·48)	0·38 (0·30 to 0·48)
Norway	0·63 (0·60 to 0·65)	0·45 (0·43 to 0·47)	0·31 (0·29 to 0·32)	0·31 (0·29 to 0·33)	0·30 (0·28 to 0·32)	0·19 (0·18 to 0·21)	0·17 (0·16 to 0·18)	0·13 (0·12 to 0·14)	0·11 (0·10 to 0·12)	0·10 (0·08 to 0·12)
Portugal	6·64 (6·52 to 6·76)	4·31 (4·18 to 4·45)	2·59 (2·53 to 2·65)	1·56 (1·53 to 1·59)	0·96 (0·93 to 0·98)	0·61 (0·59 to 0·63)	0·46 (0·44 to 0·48)	0·30 (0·29 to 0·32)	0·21 (0·21 to 0·22)	0·15 (0·12 to 0·17)
Spain	12·05 (11·79 to 12·30)	8·43 (8·24 to 8·61)	4·93 (4·82 to 5·05)	2·90 (2·81 to 2·99)	2·13 (2·06 to 2·20)	1·43 (1·38 to 1·49)	1·17 (1·11 to 1·23)	1·19 (1·14 to 1·24)	1·03 (0·98 to 1·08)	0·77 (0·55 to 1·06)
Sweden	0·87 (0·81 to 0·93)	0·62 (0·58 to 0·67)	0·45 (0·42 to 0·47)	0·40 (0·38 to 0·42)	0·48 (0·45 to 0·50)	0·28 (0·26 to 0·30)	0·20 (0·19 to 0·22)	0·20 (0·18 to 0·21)	0·20 (0·19 to 0·22)	0·18 (0·14 to 0·23)
Stockholm	0·13 (0·09 to 0·16)	0·10 (0·07 to 0·13)	0·08 (0·07 to 0·09)	0·07 (0·06 to 0·07)	0·08 (0·07 to 0·09)	0·05 (0·05 to 0·06)	0·04 (0·04 to 0·05)	0·04 (0·03 to 0·04)	0·04 (0·03 to 0·04)	0·04 (0·03 to 0·05)
Sweden except Stockholm	0·74 (0·69 to 0·79)	0·53 (0·49 to 0·57)	0·37 (0·35 to 0·40)	0·33 (0·31 to 0·36)	0·40 (0·37 to 0·42)	0·23 (0·21 to 0·24)	0·16 (0·15 to 0·18)	0·16 (0·15 to 0·17)	0·17 (0·15 to 0·18)	0·14 (0·10 to 0·19)
Switzerland	1·06 (1·02 to 1·09)	0·64 (0·61 to 0·67)	0·45 (0·43 to 0·47)	0·38 (0·36 to 0·40)	0·40 (0·38 to 0·42)	0·29 (0·28 to 0·31)	0·25 (0·24 to 0·27)	0·21 (0·19 to 0·22)	0·20 (0·18 to 0·21)	0·19 (0·14 to 0·25)
United Kingdom	11·11 (10·87 to 11·36)	7·48 (7·31 to 7·65)	5·98 (5·90 to 6·07)	4·81 (4·76 to 4·86)	4·28 (4·24 to 4·33)	3·05 (3·02 to 3·09)	2·54 (2·51 to 2·58)	2·42 (2·39 to 2·45)	2·31 (2·29 to 2·34)	2·01 (1·95 to 2·09)
England	8·82 (8·65 to 8·99)	5·92 (5·80 to 6·05)	4·83 (4·76 to 4·90)	3·93 (3·90 to 3·97)	3·60 (3·56 to 3·63)	2·54 (2·51 to 2·57)	2·14 (2·12 to 2·17)	2·05 (2·03 to 2·08)	1·98 (1·96 to 2·00)	1·73 (1·70 to 1·77)
East Midlands	0·69 (0·66 to 0·72)	0·48 (0·46 to 0·50)	0·39 (0·38 to 0·41)	0·30 (0·29 to 0·31)	0·29 (0·28 to 0·31)	0·20 (0·20 to 0·21)	0·17 (0·17 to 0·18)	0·17 (0·16 to 0·18)	0·16 (0·15 to 0·17)	0·16 (0·15 to 0·16)
East of England	0·75 (0·69 to 0·80)	0·51 (0·46 to 0·56)	0·42 (0·40 to 0·45)	0·35 (0·34 to 0·36)	0·29 (0·28 to 0·30)	0·23 (0·22 to 0·24)	0·18 (0·17 to 0·19)	0·18 (0·17 to 0·19)	0·18 (0·17 to 0·19)	0·15 (0·14 to 0·17)
Greater London	1·47 (1·42 to 1·53)	1·00 (0·97 to 1·04)	0·82 (0·79 to 0·84)	0·67 (0·66 to 0·68)	0·64 (0·63 to 0·66)	0·48 (0·47 to 0·50)	0·43 (0·42 to 0·44)	0·39 (0·38 to 0·40)	0·40 (0·39 to 0·41)	0·30 (0·29 to 0·31)
North East England	0·52 (0·48 to 0·55)	0·33 (0·31 to 0·36)	0·26 (0·25 to 0·28)	0·21 (0·20 to 0·21)	0·19 (0·18 to 0·19)	0·13 (0·13 to 0·14)	0·10 (0·10 to 0·11)	0·09 (0·08 to 0·09)	0·08 (0·07 to 0·08)	0·07 (0·06 to 0·08)
North West England	1·50 (1·41 to 1·59)	0·97 (0·91 to 1·03)	0·77 (0·74 to 0·80)	0·61 (0·60 to 0·63)	0·54 (0·53 to 0·56)	0·37 (0·36 to 0·38)	0·31 (0·30 to 0·32)	0·30 (0·29 to 0·31)	0·28 (0·27 to 0·29)	0·24 (0·23 to 0·25)
South East England	1·10 (1·04 to 1·16)	0·76 (0·73 to 0·80)	0·64 (0·62 to 0·67)	0·55 (0·53 to 0·56)	0·50 (0·48 to 0·51)	0·32 (0·31 to 0·33)	0·26 (0·25 to 0·27)	0·25 (0·24 to 0·26)	0·25 (0·25 to 0·26)	0·22 (0·21 to 0·23)
South West England	0·66 (0·63 to 0·69)	0·46 (0·44 to 0·48)	0·38 (0·36 to 0·40)	0·31 (0·30 to 0·32)	0·27 (0·26 to 0·28)	0·20 (0·19 to 0·21)	0·16 (0·15 to 0·17)	0·15 (0·15 to 0·16)	0·15 (0·14 to 0·15)	0·14 (0·14 to 0·15)
West Midlands	1·13 (1·06 to 1·20)	0·74 (0·68 to 0·81)	0·60 (0·57 to 0·62)	0·49 (0·48 to 0·51)	0·48 (0·47 to 0·50)	0·31 (0·30 to 0·32)	0·28 (0·27 to 0·29)	0·29 (0·27 to 0·30)	0·27 (0·26 to 0·28)	0·27 (0·25 to 0·30)

Appendix Table 12B. Under-5 death numbers by location, males, 1970, 1975, 1980, 1985, 1990, 1995, 2000, 2005, 2010, 2016

Location	1970	1975	1980	1985	1990	1995	2000	2005	2010	2016
Yorkshire and the Humber	1·00 (0·95 to 1·05)	0·66 (0·63 to 0·70)	0·54 (0·52 to 0·56)	0·44 (0·43 to 0·45)	0·39 (0·38 to 0·40)	0·29 (0·28 to 0·30)	0·25 (0·24 to 0·26)	0·24 (0·23 to 0·24)	0·21 (0·20 to 0·22)	0·18 (0·17 to 0·19)
Northern Ireland	0·48 (0·44 to 0·52)	0·36 (0·33 to 0·38)	0·27 (0·25 to 0·29)	0·20 (0·18 to 0·22)	0·14 (0·13 to 0·16)	0·11 (0·10 to 0·13)	0·08 (0·07 to 0·09)	0·08 (0·08 to 0·09)	0·09 (0·08 to 0·09)	0·07 (0·04 to 0·10)
Scotland	1·19 (1·14 to 1·23)	0·79 (0·75 to 0·82)	0·56 (0·54 to 0·59)	0·44 (0·41 to 0·46)	0·36 (0·34 to 0·38)	0·26 (0·24 to 0·27)	0·20 (0·19 to 0·21)	0·19 (0·17 to 0·20)	0·15 (0·14 to 0·16)	0·13 (0·10 to 0·18)
Wales	0·63 (0·48 to 0·81)	0·41 (0·32 to 0·53)	0·33 (0·29 to 0·37)	0·24 (0·23 to 0·26)	0·18 (0·17 to 0·19)	0·14 (0·13 to 0·15)	0·12 (0·11 to 0·13)	0·10 (0·09 to 0·11)	0·10 (0·09 to 0·11)	0·08 (0·07 to 0·11)
Southern Latin America	35·64 (35·17 to 36·09)	31·71 (31·04 to 32·50)	21·87 (21·65 to 22·08)	16·61 (16·43 to 16·79)	15·30 (15·13 to 15·48)	12·14 (11·99 to 12·31)	9·90 (9·38 to 10·42)	8·12 (7·70 to 8·55)	7·27 (6·90 to 7·64)	6·27 (5·48 to 7·14)
Argentina	21·04 (20·67 to 21·43)	20·61 (19·95 to 21·40)	15·48 (15·29 to 15·67)	12·12 (11·97 to 12·28)	11·31 (11·15 to 11·47)	9·27 (9·13 to 9·41)	7·84 (7·32 to 8·36)	6·46 (6·06 to 6·88)	5·74 (5·39 to 6·10)	4·98 (4·26 to 5·81)
Chile	12·87 (12·62 to 13·10)	9·34 (9·16 to 9·51)	5·14 (5·04 to 5·24)	3·49 (3·43 to 3·56)	3·25 (3·19 to 3·32)	2·17 (2·11 to 2·22)	1·56 (1·53 to 1·60)	1·26 (1·21 to 1·30)	1·23 (1·12 to 1·33)	1·05 (0·84 to 1·30)
Uruguay	1·72 (1·67 to 1·77)	1·76 (1·71 to 1·82)	1·24 (1·20 to 1·29)	0·99 (0·95 to 1·03)	0·74 (0·71 to 0·78)	0·71 (0·65 to 0·77)	0·50 (0·46 to 0·54)	0·40 (0·36 to 0·44)	0·29 (0·25 to 0·34)	0·25 (0·16 to 0·36)
Central Europe, Eastern Europe, and Central Asi	184·56 (173·04 to 197·44)	169·64 (161·39 to 178·09)	158·48 (152·14 to 165·52)	153·28 (147·88 to 158·76)	124·60 (120·36 to 129·13)	98·15 (94·23 to 102·45)	72·31 (68·64 to 76·29)	60·84 (56·06 to 66·29)	53·52 (47·75 to 60·59)	44·73 (38·67 to 52·84)
Eastern Europe	55·83 (49·68 to 63·78)	58·11 (52·96 to 64·41)	55·84 (52·24 to 60·08)	53·45 (51·25 to 56·21)	37·77 (36·07 to 39·59)	27·79 (26·45 to 29·22)	21·56 (20·37 to 22·94)	16·88 (15·83 to 18·16)	14·20 (13·29 to 15·36)	12·52 (11·06 to 14·34)
Belarus	3·12 (2·49 to 3·85)	2·59 (2·10 to 3·12)	2·38 (1·95 to 2·86)	2·35 (1·96 to 2·81)	1·77 (1·47 to 2·13)	1·19 (0·98 to 1·42)	0·89 (0·73 to 1·08)	0·65 (0·52 to 0·80)	0·51 (0·40 to 0·64)	0·38 (0·28 to 0·51)
Estonia	0·28 (0·26 to 0·29)	0·28 (0·26 to 0·30)	0·30 (0·28 to 0·32)	0·29 (0·27 to 0·31)	0·22 (0·21 to 0·24)	0·15 (0·14 to 0·17)	0·09 (0·08 to 0·09)	0·06 (0·05 to 0·07)	0·04 (0·04 to 0·05)	0·02 (0·02 to 0·03)
Latvia	0·45 (0·42 to 0·48)	0·53 (0·50 to 0·56)	0·45 (0·42 to 0·48)	0·41 (0·39 to 0·44)	0·39 (0·36 to 0·41)	0·27 (0·25 to 0·29)	0·15 (0·13 to 0·16)	0·12 (0·11 to 0·13)	0·09 (0·08 to 0·10)	0·06 (0·04 to 0·09)
Lithuania	0·74 (0·71 to 0·77)	0·72 (0·68 to 0·76)	0·57 (0·54 to 0·59)	0·63 (0·60 to 0·66)	0·45 (0·43 to 0·47)	0·38 (0·36 to 0·40)	0·22 (0·21 to 0·24)	0·15 (0·14 to 0·16)	0·11 (0·10 to 0·12)	0·08 (0·06 to 0·10)
Moldova	3·06 (2·00 to 4·48)	2·84 (1·89 to 4·01)	2·73 (2·15 to 3·39)	2·29 (1·87 to 2·77)	1·49 (1·22 to 1·80)	1·16 (0·96 to 1·41)	0·78 (0·63 to 0·96)	0·44 (0·33 to 0·56)	0·43 (0·32 to 0·55)	0·31 (0·20 to 0·44)
Russia	30·93 (30·54 to 31·37)	35·71 (35·26 to 36·18)	35·50 (35·03 to 36·00)	36·51 (36·00 to 37·05)	25·43 (25·11 to 25·75)	18·46 (18·16 to 18·77)	14·63 (14·30 to 14·99)	11·56 (11·38 to 11·74)	9·71 (9·57 to 9·85)	9·08 (8·25 to 10·00)
Ukraine	17·25 (11·07 to 25·07)	15·44 (10·53 to 21·48)	13·91 (10·39 to 18·11)	10·97 (8·83 to 13·66)	8·02 (6·47 to 9·81)	6·18 (4·93 to 7·61)	4·80 (3·70 to 6·10)	3·91 (2·91 to 5·18)	3·31 (2·43 to 4·42)	2·60 (1·45 to 4·34)
Central Europe	47·71 (45·31 to 50·63)	41·07 (39·07 to 43·31)	33·69 (32·31 to 35·23)	27·35 (26·40 to 28·37)	21·34 (20·78 to 21·90)	14·36 (14·04 to 14·70)	9·27 (9·01 to 9·55)	6·96 (6·75 to 7·20)	5·28 (5·12 to 5·46)	3·90 (3·33 to 4·59)
Albania	3·16 (1·75 to 5·53)	2·53 (1·44 to 4·08)	2·28 (1·53 to 3·26)	2·27 (1·83 to 2·77)	1·87 (1·57 to 2·21)	1·43 (1·20 to 1·69)	0·81 (0·66 to 0·99)	0·49 (0·37 to 0·64)	0·35 (0·24 to 0·50)	0·31 (0·18 to 0·49)
Bosnia and Herzegovina	2·58 (1·85 to 3·56)	1·88 (1·39 to 2·51)	1·37 (1·09 to 1·72)	1·14 (1·08 to 1·20)	0·70 (0·67 to 0·73)	0·63 (0·56 to 0·72)	0·22 (0·21 to 0·24)	0·17 (0·16 to 0·18)	0·14 (0·13 to 0·15)	0·10 (0·08 to 0·12)
Bulgaria	2·50 (2·42 to 2·58)	2·26 (2·19 to 2·33)	1·80 (1·74 to 1·87)	1·32 (1·27 to 1·37)	1·11 (1·07 to 1·16)	0·78 (0·74 to 0·82)	0·67 (0·63 to 0·71)	0·52 (0·49 to 0·55)	0·47 (0·44 to 0·50)	0·31 (0·19 to 0·51)
Croatia	1·35 (0·92 to 1·93)	1·08 (0·77 to 1·43)	0·88 (0·69 to 1·10)	0·68 (0·64 to 0·72)	0·41 (0·39 to 0·43)	0·33 (0·31 to 0·36)	0·22 (0·20 to 0·23)	0·16 (0·15 to 0·17)	0·13 (0·12 to 0·14)	0·10 (0·08 to 0·12)
Czech Republic	2·04 (1·97 to 2·11)	2·40 (2·32 to 2·47)	1·71 (1·64 to 1·78)	1·15 (1·10 to 1·19)	0·91 (0·87 to 0·96)	0·51 (0·49 to 0·54)	0·28 (0·26 to 0·30)	0·25 (0·23 to 0·27)	0·23 (0·21 to 0·25)	0·18 (0·13 to 0·24)
Hungary	3·43 (3·31 to 3·54)	3·92 (3·79 to 4·05)	2·17 (2·09 to 2·24)	1·67 (1·60 to 1·74)	1·22 (1·17 to 1·28)	0·79 (0·75 to 0·83)	0·58 (0·54 to 0·61)	0·41 (0·38 to 0·44)	0·30 (0·28 to 0·32)	0·29 (0·20 to 0·40)
Macedonia	1·53 (0·85 to 2·54)	1·23 (0·66 to 2·06)	1·15 (0·80 to 1·63)	0·88 (0·63 to 1·19)	0·67 (0·64 to 0·71)	0·43 (0·40 to 0·45)	0·23 (0·21 to 0·25)	0·18 (0·17 to 0·19)	0·13 (0·12 to 0·14)	0·12 (0·08 to 0·19)
Montenegro	0·20 (0·13 to 0·31)	0·17 (0·11 to 0·27)	0·14 (0·09 to 0·21)	0·12 (0·08 to 0·18)	0·09 (0·06 to 0·12)	0·08 (0·07 to 0·09)	0·08 (0·07 to 0·09)	0·05 (0·04 to 0·05)	0·03 (0·03 to 0·03)	0·02 (0·02 to 0·03)
Poland	11·83 (11·56 to 12·08)	10·64 (10·40 to 10·87)	9·82 (9·59 to 10·05)	8·11 (7·85 to 8·36)	5·77 (5·61 to 5·92)	3·77 (3·64 to 3·89)	2·01 (1·93 to 2·10)	1·54 (1·48 to 1·61)	1·32 (1·25 to 1·39)	0·93 (0·53 to 1·56)
Romania	13·60 (13·36 to 13·84)	9·82 (9·61 to 10·02)	8·01 (7·85 to 8·17)	6·48 (6·33 to 6·63)	6·15 (6·02 to 6·29)	3·50 (3·39 to 3·61)	2·92 (2·82 to 3·01)	2·21 (2·12 to 2·32)	1·40 (1·34 to 1·45)	0·95 (0·77 to 1·19)
Serbia	3·62 (2·39 to 5·19)	3·21 (2·16 to 4·61)	2·74 (1·89 to 3·79)	2·34 (1·68 to 3·11)	1·63 (1·26 to 2·09)	1·58 (1·52 to 1·63)	0·90 (0·74 to 1·08)	0·68 (0·56 to 0·82)	0·51 (0·48 to 0·54)	0·37 (0·31 to 0·44)
Slovakia	1·41 (1·35 to 1·47)	1·55 (1·48 to 1·62)	1·31 (1·26 to 1·37)	0·97 (0·92 to 1·01)	0·66 (0·63 to 0·69)	0·45 (0·42 to 0·48)	0·30 (0·28 to 0·33)	0·26 (0·24 to 0·28)	0·23 (0·21 to 0·25)	0·19 (0·13 to 0·28)
Slovenia	0·47 (0·33 to 0·66)	0·39 (0·30 to 0·51)	0·32 (0·27 to 0·37)	0·23 (0·21 to 0·24)	0·14 (0·13 to 0·14)	0·08 (0·07 to 0·09)	0·06 (0·05 to 0·06)	0·05 (0·04 to 0·05)	0·04 (0·04 to 0·04)	0·03 (0·02 to 0·03)
Central Asia	81·02 (71·56 to 91·10)	70·46 (64·39 to 77·22)	68·95 (64·08 to 74·34)	72·47 (67·57 to 77·32)	65·50 (61·56 to 69·87)	56·00 (52·34 to 60·18)	41·49 (38·02 to 45·28)	37·00 (32·47 to 42·10)	34·05 (28·37 to 41·13)	28·32 (22·61 to 35·87)
Armenia	3·59 (2·45 to 5·12)	3·06 (2·37 to 3·92)	3·11 (2·60 to 3·72)	2·78 (2·40 to 3·23)	2·06 (1·77 to 2·40)	1·26 (1·07 to 1·47)	0·69 (0·58 to 0·82)	0·54 (0·42 to 0·69)	0·42 (0·32 to 0·53)	0·32 (0·23 to 0·44)
Azerbaijan	9·94 (6·88 to 13·72)	7·41 (5·34 to 10·09)	7·66 (6·09 to 9·57)	8·44 (7·05 to 9·93)	8·11 (6·79 to 9·63)	6·89 (5·76 to 8·21)	4·62 (3·88 to 5·50)	4·52 (3·62 to 5·58)	4·41 (3·11 to 5·94)	3·30 (2·09 to 4·94)
Georgia	5·10 (3·79 to 6·74)	4·07 (3·20 to 5·10)	3·16 (2·52 to 3·89)	2·69 (2·29 to 3·15)	2·25 (1·95 to 2·57)	1·54 (1·34 to 1·78)	1·15 (0·99 to 1·33)	0·95 (0·75 to 1·18)	0·82 (0·61 to 1·07)	0·59 (0·42 to 0·82)
Kazakhstan	13·50 (9·60 to 17·89)	12·00 (9·89 to 14·54)	10·92 (9·32 to 12·63)	9·95 (8·72 to 11·29)	8·24 (7·20 to 9·26)	6·87 (5·82 to 8·00)	5·13 (4·21 to 6·16)	4·97 (3·92 to 6·22)	4·37 (3·44 to 5·43)	3·10 (2·23 to 4·20)
Kyrgyzstan	7·33 (5·83 to 9·25)	6·03 (5·04 to 7·16)	5·63 (4·84 to 6·46)	5·69 (5·09 to 6·29)	4·81 (4·41 to 5·22)	3·61 (3·31 to 3·93)	2·84 (2·57 to 3·12)	2·57 (2·30 to 2·87)	2·81 (2·46 to 3·17)	2·54 (2·03 to 3·10)
Mongolia	4·27 (3·26 to 5·35)	4·01 (3·09 to 5·01)	4·13 (3·33 to 5·00)	4·16 (3·55 to 4·80)	3·53 (3·13 to 3·95)	2·30 (2·02 to 2·61)	1·57 (1·37 to 1·79)	1·19 (1·00 to 1·38)	1·12 (0·93 to 1·34)	0·96 (0·71 to 1·25)
Tajikistan	10·68 (7·86 to 14·33)	10·14 (7·94 to 12·91)	9·43 (7·84 to 11·27)	10·89 (9·04 to 12·95)	10·59 (9·11 to 12·29)	9·00 (7·73 to 10·46)	6·51 (5·28 to 7·85)	5·19 (4·32 to 6·27)	5·01 (3·89 to 6·31)	5·40 (3·36 to 8·24)
Turkmenistan	6·78 (4·43 to 9·74)	6·10 (4·08 to 8·63)	5·88 (4·52 to 7·50)	6·26 (5·20 to 7·45)	6·40 (5·31 to 7·58)	5·96 (4·91 to 7·26)	4·79 (3·49 to 6·44)	3·93 (2·72 to 5·32)	3·26 (2·14 to 4·61)	2·60 (1·70 to 3·83)
Uzbekistan	19·82 (13·90 to 26·90)	17·63 (13·85 to 22·00)	19·04 (15·61 to 22·93)	21·61 (18·36 to 25·28)	19·51 (16·42 to 22·77)	18·57 (15·88 to 21·76)	14·20 (11·56 to 17·23)	13·14 (9·32 to 17·80)	11·84 (6·94 to 18·29)	9·49 (4·99 to 16·18)
Latin America and Caribbean	571·97 (556·38 to 587·54)	521·72 (509·51 to 533·32)	456·70 (446·24 to 468·52)	384·99 (376·28 to 393·70)	315·98 (308·67 to 323·57)	255·84 (249·56 to 262·72)	199·82 (193·33 to 206·21)	162·83 (157·59 to 168·58)	148·30 (135·96 to 162·37)	104·27 (98·37 to 111·04)
Central Latin America	209·53 (204·13 to 215·15)	193·05 (188·20 to 198·21)	166·20 (161·94 to 170·88)	141·27 (137·76 to 145·00)	120·66 (117·23 to 124·14)	102·80 (99·94 to 105·64)	82·75 (80·00 to 85·49)	67·16 (64·74 to 69·84)	55·26 (52·86 to 57·73)	42·76 (40·46 to 45·42)
Colombia	33·74 (30·66 to 37·13)	29·41 (26·72 to 32·15)	23·15 (20·72 to 25·55)	20·65 (18·52 to 22·90)	16·94 (14·73 to 19·31)	15·27 (13·63 to 16·93)	12·69 (11·33 to 14·09)	11·11 (9·78 to 12·75)	9·04 (7·58 to 10·69)	6·04 (4·86 to 7·61)
Costa Rica	2·26 (2·10 to 2·44)	1·58 (1·44 to 1·73)	1·26 (1·11 to 1·41)	1·10 (0·96 to 1·24)	0·91 (0·78 to 1·05)	0·75 (0·62 to 0·90)	0·66 (0·54 to 0·80)	0·56 (0·45 to 0·68)	0·48 (0·36 to 0·62)	0·35 (0·22 to 0·54)
El Salvador	11·59 (10·06 to 13·20)	10·31 (9·19 to 11·48)	8·48 (7·60 to 9·42)	6·22 (5·58 to 6·89)	4·54 (4·00 to 5·13)	3·43 (2·93 to 4·00)	2·42 (2·04 to 2·84)	1·56 (1·23 to 1·94)	1·07 (0·82 to 1·37)	0·71 (0·48 to 1·02)
Guatemala	22·31 (19·92 to 24·90)	20·65 (18·49 to 23·08)	18·38 (16·56 to 20·33)	16·38 (14·79 to 17·99)	14·76 (13·41 to 16·18)	13·08 (11·99 to 14·26)	11·28 (10·11 to 12·49)	9·54 (8·42 to 10·69)	7·66 (6·76 to 8·70)	6·10 (5·03 to 7·34)
Honduras	8·77 (7·91 to 9·67)	8·13 (7·40 to 8·95)	7·07 (6·37 to 7·77)	6·26 (5·58 to 6·96)	5·37 (4·70 to 6·04)	4·43 (3·74 to 5·13)	3·58 (3·03 to 4·16)	3·02 (2·57 to 3·49)	2·76 (2·33 to 3·26)	2·35 (1·85 to 2·91)
Mexico	107·60 (104·40 to 111·11)	100·94 (97·70 to 104·28)	89·84 (87·15 to 92·78)	75·02 (72·82 to 77·15)	63·50 (61·66 to 65·52)	52·37 (50·65 to 54·26)	41·37 (39·76 to 42·96)	32·21 (30·68 to 33·76)	26·10 (24·67 to 27·61)	20·10 (18·75 to 21·57)
Nicaragua	7·11 (6·59 to 7·67)	6·39 (6·01 to 6·76)	5·69 (5·38 to 6·03)	5·44 (5·06 to 5·81)	4·45 (4·19 to 4·71)	3·96 (3·70 to 4·23)	2·98 (2·76 to 3·22)	2·19 (1·98 to 2·41)	1·59 (1·39 to 1·83)	1·04 (0·87 to 1·26)
Panama	1·70 (1·55 to 1·87)	1·35 (1·22 to 1·49)	1·13 (0·99 to 1·28)	0·97 (0·84 to 1·10)	0·93 (0·80 to 1·07)	0·92 (0·78 to 1·11)	0·86 (0·73 to 1·03)	0·79 (0·65 to 0·96)	0·76 (0·62 to 0·92)	0·63 (0·44 to 0·90)
Venezuela	14·43 (14·29 to 14·57)	14·29 (14·14 to 14·45)	11·20 (11·09 to 11·33)	9·24 (9·13 to 9·35)	9·25 (9·13 to 9·39)	8·57 (8·21 to 8·97)	6·92 (6·26 to 7·64)	6·18 (5·94 to 6·43)	5·80 (5·71 to 5·90)	5·44 (4·58 to 6·44)
Andean Latin America	85·74 (79·83 to 91·75)	76·12 (72·31 to 79·88)	69·98 (65·93 to 74·22)	61·26 (58·04 to 64·53)	49·84 (47·53 to 52·36)	38·95 (37·03 to 40·87)	29·00 (27·13 to 30·79)	23·15 (21·24 to 25·11)	19·31 (17·38 to 21·08)	16·04 (13·68 to 18·63)
Bolivia	22·71 (20·63 to 24·78)	21·10 (19·63 to 22·58)	19·32 (17·85 to 21·03)	17·26 (15·85 to 18·90)	14·88 (13·85 to 15·96)	12·63 (11·61 to 13·76)	10·42 (9·22 to 11·83)	8·59 (7·31 to 9·96)	6·94 (5·59 to 8·49)	5·48 (4·07 to 7·24)
Ecuador	13·86 (12·54 to 15·12)	12·95 (11·69 to 14·20)	11·57 (10·52 to 12·61)	10·03 (9·12 to 10·98)	8·48 (7·54 to 9·52)	7·26 (6·45 to 8·09)	6·05 (5·27 to 6·89)	5·10 (4·32 to 5·92)	4·29 (3·58 to 5·09)	3·56 (2·87 to 4·37)

Appendix Table 12B. Under-5 death numbers by location, males, 1970, 1975, 1980, 1985, 1990, 1995, 2000, 2005, 2010, 2016

Location	1970	1975	1980	1985	1990	1995	2000	2005	2010	2016
Peru	49·17 (43·48 to 55·09)	42·07 (38·75 to 45·23)	39·10 (35·55 to 42·63)	33·98 (31·15 to 36·67)	26·47 (24·63 to 28·52)	19·06 (17·44 to 20·81)	12·53 (11·41 to 13·61)	9·46 (8·40 to 10·60)	8·08 (7·20 to 8·98)	7·00 (5·46 to 8·93)
Caribbean	56·63 (48·99 to 64·41)	49·41 (42·05 to 56·10)	43·74 (37·06 to 50·95)	38·37 (33·23 to 43·83)	33·80 (28·88 to 38·57)	28·66 (24·66 to 33·04)	23·76 (19·23 to 28·81)	22·38 (18·53 to 26·60)	33·89 (21·69 to 46·83)	15·06 (10·96 to 20·90)
Antigua and Barbuda	0·04 (0·02 to 0·05)	0·03 (0·02 to 0·03)	0·02 (0·01 to 0·02)	0·01 (0·01 to 0·01)	0·01 (0·01 to 0·01)	0·01 (0·01 to 0·02)	0·01 (0·01 to 0·02)	0·01 (0·01 to 0·02)	0·01 (0·01 to 0·01)	0·01 (<0·01 to 0·01)
The Bahamas	0·13 (0·09 to 0·19)	0·11 (0·07 to 0·16)	0·10 (0·06 to 0·15)	0·09 (0·05 to 0·13)	0·07 (0·04 to 0·12)	0·06 (0·04 to 0·09)	0·04 (0·03 to 0·06)	0·05 (0·03 to 0·06)	0·04 (0·03 to 0·07)	0·04 (0·02 to 0·09)
Barbados	0·13 (0·09 to 0·19)	0·09 (0·06 to 0·13)	0·07 (0·04 to 0·10)	0·05 (0·03 to 0·08)	0·04 (0·02 to 0·07)	0·03 (0·02 to 0·05)	0·03 (0·01 to 0·05)	0·03 (0·02 to 0·04)	0·03 (0·02 to 0·04)	0·02 (0·01 to 0·05)
Belize	0·30 (0·23 to 0·37)	0·24 (0·20 to 0·30)	0·21 (0·17 to 0·25)	0·20 (0·16 to 0·24)	0·17 (0·14 to 0·20)	0·11 (0·08 to 0·14)	0·12 (0·09 to 0·15)	0·10 (0·07 to 0·15)	0·09 (0·06 to 0·13)	0·09 (0·04 to 0·17)
Bermuda	0·02 (0·01 to 0·02)	0·01 (0·01 to 0·01)	0·01 (0·01 to 0·01)	0·01 (<0·01 to 0·01)	0·01 (<0·01 to 0·01)	<0·01 (<0·01 to 0·01)	<0·01 (<0·01 to <0·01)	<0·01 (<0·01 to <0·01)	<0·01 (<0·01 to <0·01)	<0·01 (<0·01 to <0·01)
Cuba	5·93 (5·84 to 6·04)	3·37 (3·30 to 3·43)	1·91 (1·86 to 1·96)	1·87 (1·81 to 1·94)	1·48 (1·44 to 1·53)	0·96 (0·94 to 0·99)	0·74 (0·72 to 0·76)	0·50 (0·49 to 0·52)	0·45 (0·43 to 0·46)	0·34 (0·27 to 0·43)
Dominica	0·09 (0·06 to 0·12)	0·04 (0·03 to 0·06)	0·02 (0·02 to 0·03)	0·02 (0·01 to 0·03)	0·02 (0·01 to 0·02)	0·01 (0·01 to 0·02)	0·01 (0·01 to 0·02)	0·01 (0·01 to 0·02)	0·01 (0·01 to 0·02)	0·01 (0·01 to 0·02)
Dominican Republic	11·25 (9·65 to 12·86)	10·95 (9·51 to 12·43)	9·58 (8·46 to 10·79)	7·48 (6·54 to 8·49)	6·61 (5·48 to 7·75)	5·62 (4·50 to 6·73)	4·51 (3·82 to 5·27)	4·41 (3·52 to 5·45)	3·79 (2·94 to 4·84)	2·94 (2·08 to 3·96)
Grenada	0·08 (0·05 to 0·13)	0·07 (0·04 to 0·11)	0·05 (0·03 to 0·09)	0·05 (0·02 to 0·09)	0·03 (0·02 to 0·05)	0·02 (0·01 to 0·03)	0·02 (0·01 to 0·03)	0·02 (0·01 to 0·02)	0·01 (0·01 to 0·02)	0·01 (0·01 to 0·03)
Guyana	1·09 (0·95 to 1·24)	1·02 (0·91 to 1·15)	0·89 (0·78 to 1·01)	0·72 (0·60 to 0·85)	0·67 (0·52 to 0·81)	0·56 (0·44 to 0·67)	0·43 (0·35 to 0·53)	0·35 (0·28 to 0·42)	0·26 (0·21 to 0·32)	0·19 (0·14 to 0·26)
Haiti	30·33 (22·89 to 37·81)	27·80 (21·02 to 34·37)	26·13 (19·70 to 32·91)	23·91 (18·77 to 29·03)	21·20 (16·69 to 25·43)	18·24 (14·70 to 22·42)	15·28 (10·81 to 20·08)	14·68 (10·97 to 18·81)	27·20 (15·08 to 39·52)	9·79 (5·95 to 15·19)
Jamaica	2·26 (1·87 to 2·69)	1·75 (1·43 to 2·15)	1·50 (1·08 to 2·02)	1·29 (0·94 to 1·71)	1·18 (0·80 to 1·72)	1·01 (0·65 to 1·50)	0·87 (0·56 to 1·29)	0·71 (0·42 to 1·14)	0·69 (0·34 to 1·27)	0·62 (0·33 to 1·05)
Puerto Rico	1·22 (1·19 to 1·25)	1·00 (0·97 to 1·02)	0·83 (0·81 to 0·85)	0·63 (0·61 to 0·66)	0·56 (0·54 to 0·58)	0·48 (0·45 to 0·52)	0·36 (0·33 to 0·40)	0·27 (0·24 to 0·31)	0·22 (0·19 to 0·25)	0·16 (0·12 to 0·20)
Saint Lucia	0·16 (0·09 to 0·28)	0·11 (0·07 to 0·18)	0·08 (0·05 to 0·12)	0·06 (0·03 to 0·10)	0·05 (0·03 to 0·08)	0·04 (0·02 to 0·06)	0·03 (0·02 to 0·05)	0·03 (0·02 to 0·04)	0·02 (0·01 to 0·04)	0·02 (0·01 to 0·03)
Saint Vincent and the Grenadines	0·18 (0·12 to 0·25)	0·13 (0·08 to 0·19)	0·09 (0·06 to 0·13)	0·06 (0·04 to 0·09)	0·05 (0·03 to 0·07)	0·04 (0·02 to 0·06)	0·03 (0·02 to 0·04)	0·02 (0·02 to 0·03)	0·02 (0·01 to 0·03)	0·02 (0·01 to 0·03)
Suriname	0·51 (0·41 to 0·65)	0·41 (0·34 to 0·50)	0·37 (0·31 to 0·44)	0·36 (0·30 to 0·43)	0·33 (0·26 to 0·42)	0·32 (0·24 to 0·42)	0·31 (0·22 to 0·40)	0·27 (0·18 to 0·37)	0·23 (0·14 to 0·34)	0·18 (0·10 to 0·31)
Trinidad and Tobago	0·84 (0·73 to 0·96)	0·76 (0·65 to 0·88)	0·71 (0·59 to 0·86)	0·60 (0·49 to 0·74)	0·40 (0·31 to 0·50)	0·33 (0·23 to 0·44)	0·30 (0·22 to 0·40)	0·26 (0·19 to 0·36)	0·22 (0·11 to 0·38)	0·16 (0·06 to 0·37)
Virgin Islands, U.S.	0·05 (0·04 to 0·05)	0·04 (0·04 to 0·05)	0·04 (0·03 to 0·04)	0·03 (0·03 to 0·03)	0·02 (0·02 to 0·03)	0·02 (0·02 to 0·02)	0·01 (0·01 to 0·01)	0·01 (0·01 to 0·01)	0·01 (0·01 to 0·01)	0·01 (<0·01 to 0·01)
Tropical Latin America	220·07 (210·39 to 230·77)	203·14 (194·86 to 211·83)	176·77 (170·02 to 184·08)	144·10 (139·30 to 148·69)	111·68 (107·98 to 115·34)	85·43 (82·21 to 88·56)	64·31 (61·63 to 67·24)	50·14 (47·68 to 52·70)	39·84 (37·15 to 42·75)	30·42 (28·16 to 32·53)
Brazil	216·83 (207·11 to 227·45)	200·00 (191·91 to 208·58)	173·81 (167·13 to 180·90)	141·15 (136·37 to 145·77)	109·06 (105·52 to 112·73)	83·00 (79·80 to 86·14)	61·91 (59·25 to 64·87)	48·03 (45·52 to 50·50)	38·09 (35·50 to 40·88)	29·05 (26·88 to 31·13)
Paraguay	3·24 (2·87 to 3·66)	3·15 (2·80 to 3·52)	2·96 (2·57 to 3·37)	2·95 (2·64 to 3·29)	2·63 (2·37 to 2·92)	2·43 (2·08 to 2·83)	2·40 (2·04 to 2·81)	2·11 (1·75 to 2·52)	1·75 (1·33 to 2·28)	1·36 (0·99 to 1·84)
Southeast Asia, East Asia, and Oceania	2,448·48 (2,362·06 to 2,540·33)	1,883·69 (1,821·50 to 1,948·98)	1,495·23 (1,444·27 to 1,545·79)	1,299·68 (1,258·49 to 1,341·07)	1,113·26 (1,079·39 to 1,150·11)	882·32 (847·50 to 919·38)	668·50 (638·86 to 700·61)	479·86 (461·10 to 501·17)	364·67 (348·41 to 381·90)	246·32 (230·46 to 262·79)
East Asia	1,643·88 (1,567·01 to 1,729·45)	1,169·96 (1,118·68 to 1,224·49)	881·18 (840·92 to 922·65)	761·79 (727·77 to 795·36)	645·51 (618·05 to 676·72)	485·29 (458·92 to 516·86)	335·10 (312·05 to 360·69)	200·16 (191·41 to 209·24)	140·45 (133·45 to 147·74)	89·53 (81·62 to 98·57)
China	1,619·91 (1,542·48 to 1,705·21)	1,153·71 (1,101·34 to 1,207·46)	868·92 (830·28 to 911·27)	749·95 (716·28 to 784·01)	634·14 (606·19 to 664·65)	446·75 (429·71 to 464·71)	296·70 (285·76 to 309·05)	186·96 (179·75 to 194·24)	129·28 (123·88 to 135·11)	80·04 (73·00 to 87·68)
North Korea	18·25 (12·08 to 25·92)	12·29 (7·93 to 17·37)	8·79 (5·60 to 12·85)	9·72 (6·32 to 14·22)	9·77 (6·15 to 14·54)	36·90 (16·16 to 60·03)	36·99 (17·82 to 58·26)	12·38 (8·18 to 18·21)	10·60 (6·84 to 15·75)	8·97 (5·74 to 14·05)
Taiwan (Province of China)	5·71 (5·59 to 5·84)	3·96 (3·87 to 4·07)	3·47 (3·38 to 3·57)	2·12 (2·05 to 2·19)	1·59 (1·54 to 1·65)	1·63 (1·58 to 1·69)	1·41 (1·36 to 1·46)	0·83 (0·80 to 0·87)	0·57 (0·53 to 0·60)	0·52 (0·44 to 0·61)
Southeast Asia	794·92 (758·36 to 836·96)	703·99 (671·21 to 740·05)	604·82 (577·09 to 633·75)	528·72 (506·94 to 551·84)	458·46 (437·39 to 482·12)	387·67 (366·80 to 414·30)	324·07 (305·42 to 344·86)	270·37 (253·75 to 290·03)	215·54 (201·23 to 231·38)	149·20 (135·88 to 163·19)
Cambodia	26·83 (21·91 to 32·20)	39·67 (27·06 to 52·85)	22·33 (19·26 to 25·83)	19·97 (17·31 to 22·79)	18·48 (16·46 to 20·50)	18·89 (17·06 to 21·01)	16·82 (15·17 to 18·59)	13·41 (11·92 to 15·07)	10·12 (8·65 to 11·63)	7·13 (5·66 to 8·76)
Indonesia	418·41 (403·57 to 432·85)	353·24 (338·09 to 368·42)	304·46 (293·79 to 315·96)	258·66 (249·26 to 268·58)	218·77 (211·32 to 226·59)	183·06 (177·99 to 188·63)	151·87 (146·38 to 157·49)	126·31 (121·31 to 131·48)	101·67 (96·10 to 107·70)	66·95 (60·69 to 73·73)
Laos	20·05 (12·19 to 30·34)	19·81 (12·87 to 28·05)	17·24 (12·24 to 22·75)	16·07 (11·94 to 20·06)	16·56 (12·74 to 20·51)	16·64 (12·72 to 20·94)	14·60 (11·51 to 18·00)	12·07 (9·11 to 15·35)	10·16 (6·50 to 14·92)	8·40 (4·22 to 14·47)
Malaysia	11·08 (10·86 to 11·30)	9·19 (8·67 to 9·66)	7·20 (6·56 to 7·84)	6·11 (5·95 to 6·26)	4·79 (4·67 to 4·92)	3·99 (3·89 to 4·09)	2·81 (2·73 to 2·90)	2·21 (2·14 to 2·29)	2·33 (2·18 to 2·49)	1·71 (1·40 to 2·05)
Maldives	0·63 (0·55 to 0·73)	0·54 (0·47 to 0·61)	0·49 (0·43 to 0·56)	0·39 (0·34 to 0·45)	0·26 (0·23 to 0·30)	0·16 (0·14 to 0·18)	0·09 (0·08 to 0·10)	0·06 (0·05 to 0·06)	0·05 (0·04 to 0·05)	0·03 (0·02 to 0·03)
Mauritius	1·03 (1·00 to 1·06)	0·77 (0·75 to 0·80)	0·54 (0·52 to 0·56)	0·31 (0·30 to 0·32)	0·29 (0·28 to 0·30)	0·26 (0·25 to 0·27)	0·21 (0·20 to 0·22)	0·16 (0·16 to 0·17)	0·12 (0·12 to 0·13)	0·11 (0·08 to 0·13)
Myanmar	86·61 (59·24 to 118·76)	77·63 (55·60 to 103·95)	71·63 (51·28 to 93·53)	67·93 (51·54 to 86·29)	64·24 (47·11 to 84·58)	58·63 (41·27 to 82·64)	51·78 (35·64 to 72·10)	41·38 (27·18 to 59·71)	26·81 (17·35 to 38·99)	15·25 (9·03 to 25·67)
Philippines	71·24 (66·17 to 76·47)	71·31 (65·49 to 77·18)	72·46 (66·32 to 78·67)	68·66 (63·51 to 73·84)	60·82 (56·12 to 66·17)	52·98 (48·89 to 57·12)	47·90 (43·87 to 52·18)	44·64 (40·30 to 49·27)	40·22 (34·89 to 46·22)	33·67 (27·72 to 40·57)
Sri Lanka	13·68 (13·38 to 13·98)	13·89 (13·49 to 14·30)	10·39 (10·18 to 10·61)	7·07 (6·90 to 7·24)	6·56 (6·26 to 6·88)	4·04 (3·93 to 4·15)	3·22 (3·12 to 3·33)	3·59 (3·49 to 3·69)	2·57 (2·35 to 2·80)	1·06 (0·67 to 1·61)
Seychelles	0·07 (0·07 to 0·08)	0·05 (0·05 to 0·05)	0·03 (0·03 to 0·04)	0·02 (0·02 to 0·03)	0·02 (0·02 to 0·02)	0·01 (0·01 to 0·01)	0·01 (0·01 to 0·01)	0·01 (0·01 to 0·01)	0·01 (0·01 to 0·01)	0·01 (0·01 to 0·01)
Thailand	67·24 (58·21 to 76·13)	55·97 (48·28 to 64·00)	41·05 (32·67 to 49·40)	27·68 (22·71 to 33·62)	19·79 (15·42 to 24·66)	13·61 (10·32 to 17·50)	9·21 (6·81 to 12·00)	5·92 (4·53 to 7·75)	3·73 (2·98 to 4·70)	2·07 (1·44 to 2·84)
Timor-Leste	3·53 (2·52 to 4·76)	3·56 (2·61 to 4·55)	3·17 (2·36 to 4·05)	2·44 (1·78 to 3·19)	2·07 (1·58 to 2·61)	1·69 (1·31 to 2·08)	1·08 (0·80 to 1·41)	0·93 (0·65 to 1·27)	0·94 (0·60 to 1·37)	0·66 (0·36 to 1·07)
Vietnam	73·25 (59·72 to 88·39)	57·56 (48·75 to 67·50)	53·14 (45·31 to 61·58)	52·78 (44·38 to 61·87)	45·22 (37·32 to 54·15)	33·27 (27·72 to 39·63)	24·07 (21·00 to 27·31)	19·32 (16·10 to 23·03)	16·57 (13·02 to 20·94)	12·00 (9·06 to 15·98)
Oceania	9·69 (8·03 to 11·52)	9·74 (8·13 to 11·48)	9·23 (7·79 to 10·74)	9·18 (7·73 to 10·77)	9·30 (7·64 to 11·18)	9·36 (7·37 to 11·69)	9·33 (6·79 to 12·48)	9·32 (6·09 to 13·48)	8·68 (5·33 to 13·32)	7·58 (4·59 to 12·20)
American Samoa	0·02 (0·02 to 0·02)	0·02 (0·02 to 0·02)	0·02 (0·01 to 0·02)	0·01 (0·01 to 0·02)	0·02 (0·01 to 0·02)	0·02 (0·01 to 0·02)	0·01 (0·01 to 0·02)	0·01 (0·01 to 0·01)	0·01 (0·01 to 0·01)	0·01 (0·01 to 0·01)
Federated States of Micronesia	0·14 (0·10 to 0·20)	0·12 (0·08 to 0·16)	0·12 (0·09 to 0·16)	0·11 (0·08 to 0·14)	0·09 (0·07 to 0·12)	0·07 (0·05 to 0·10)	0·06 (0·04 to 0·08)	0·03 (0·02 to 0·04)	0·02 (0·01 to 0·04)	0·02 (0·01 to 0·03)
Fiji	0·50 (0·44 to 0·57)	0·54 (0·43 to 0·66)	0·44 (0·33 to 0·57)	0·35 (0·25 to 0·46)	0·27 (0·18 to 0·40)	0·25 (0·18 to 0·34)	0·24 (0·18 to 0·30)	0·24 (0·19 to 0·30)	0·24 (0·18 to 0·32)	0·21 (0·12 to 0·35)
Guam	0·04 (0·04 to 0·05)	0·04 (0·03 to 0·04)	0·03 (0·02 to 0·03)	0·02 (0·02 to 0·03)	0·03 (0·02 to 0·03)	0·02 (0·02 to 0·03)	0·02 (0·02 to 0·03)	0·02 (0·02 to 0·03)	0·03 (0·02 to 0·03)	0·03 (0·02 to 0·04)
Kiribati	0·16 (0·12 to 0·21)	0·14 (0·09 to 0·21)	0·14 (0·08 to 0·24)	0·14 (0·09 to 0·22)	0·14 (0·10 to 0·18)	0·12 (0·09 to 0·15)	0·10 (0·08 to 0·13)	0·10 (0·07 to 0·14)	0·09 (0·06 to 0·14)	0·07 (0·04 to 0·13)
Marshall Islands	0·04 (0·02 to 0·07)	0·04 (0·02 to 0·07)	0·04 (0·02 to 0·06)	0·04 (0·03 to 0·06)	0·04 (0·03 to 0·05)	0·04 (0·02 to 0·05)	0·04 (0·03 to 0·06)	0·04 (0·02 to 0·05)	0·03 (0·02 to 0·06)	0·03 (0·01 to 0·05)
Northern Mariana Islands	<0·01 (<0·01 to 0·01)	<0·01 (<0·01 to 0·01)	<0·01 (<0·01 to 0·01)	0·01 (<0·01 to 0·01)	0·01 (<0·01 to 0·01)	0·01 (<0·01 to 0·01)	0·01 (<0·01 to 0·01)	0·01 (<0·01 to 0·01)	<0·01 (<0·01 to 0·01)	<0·01 (<0·01 to 0·01)
Papua New Guinea	7·12 (5·59 to 8·79)	7·38 (5·88 to 9·00)	7·20 (5·92 to 8·62)	7·30 (5·99 to 8·81)	7·57 (6·02 to 9·30)	7·80 (5·96 to 10·04)	7·87 (5·48 to 10·89)	7·93 (4·82 to 11·95)	7·46 (4·20 to 11·92)	6·49 (3·62 to 10·95)
Samoa	0·19 (0·14 to 0·24)	0·15 (0·10 to 0·21)	0·10 (0·06 to 0·17)	0·09 (0·05 to 0·17)	0·07 (0·03 to 0·14)	0·06 (0·03 to 0·12)	0·05 (0·02 to 0·10)	0·05 (0·02 to 0·09)	0·04 (0·02 to 0·08)	0·03 (0·01 to 0·06)
Solomon Islands	0·42 (0·31 to 0·55)	0·39 (0·26 to 0·57)	0·31 (0·20 to 0·46)	0·28 (0·19 to 0·40)	0·28 (0·19 to 0·39)	0·28 (0·20 to 0·39)	0·30 (0·21 to 0·41)	0·33 (0·22 to 0·45)	0·31 (0·20 to 0·46)	0·25 (0·15 to 0·38)

Appendix Table 12B. Under-5 death numbers by location, males, 1970, 1975, 1980, 1985, 1990, 1995, 2000, 2005, 2010, 2016

Location	1970	1975	1980	1985	1990	1995	2000	2005	2010	2016
Tonga	0·07 (0·05 to 0·10)	0·07 (0·04 to 0·09)	0·06 (0·04 to 0·09)	0·06 (0·03 to 0·08)	0·05 (0·03 to 0·08)	0·05 (0·03 to 0·08)	0·04 (0·02 to 0·07)	0·04 (0·02 to 0·07)	0·04 (0·02 to 0·07)	0·03 (0·02 to 0·05)
Vanuatu	0·21 (0·14 to 0·29)	0·19 (0·12 to 0·27)	0·16 (0·11 to 0·24)	0·15 (0·11 to 0·20)	0·14 (0·11 to 0·18)	0·14 (0·11 to 0·19)	0·16 (0·12 to 0·20)	0·17 (0·13 to 0·23)	0·17 (0·12 to 0·23)	0·15 (0·09 to 0·24)
North Africa and Middle East	841·36 (783·56 to 905·55)	732·58 (678·07 to 779·47)	639·33 (595·05 to 688·35)	553·96 (514·49 to 593·24)	427·26 (400·15 to 455·86)	359·09 (332·03 to 389·48)	306·82 (278·90 to 334·90)	267·79 (238·39 to 299·24)	240·81 (212·35 to 273·10)	193·97 (160·49 to 231·11)
North Africa and Middle East	841·36 (783·56 to 905·55)	732·58 (678·07 to 779·47)	639·33 (595·05 to 688·35)	553·96 (514·49 to 593·24)	427·26 (400·15 to 455·86)	359·09 (332·03 to 389·48)	306·82 (278·90 to 334·90)	267·79 (238·39 to 299·24)	240·81 (212·35 to 273·10)	193·97 (160·49 to 231·11)
Afghanistan	60·09 (40·92 to 81·22)	63·54 (45·42 to 84·35)	64·73 (47·81 to 84·39)	52·96 (39·29 to 66·74)	39·71 (28·74 to 50·41)	48·24 (34·72 to 61·11)	51·37 (38·63 to 64·56)	50·96 (37·82 to 65·26)	42·34 (29·30 to 57·54)	39·32 (24·54 to 58·61)
Algeria	54·54 (44·48 to 64·80)	48·10 (39·17 to 57·08)	39·29 (32·12 to 46·50)	29·36 (23·15 to 35·42)	22·57 (17·72 to 27·67)	20·39 (15·36 to 26·51)	18·69 (14·20 to 23·63)	16·19 (12·45 to 20·08)	13·24 (9·41 to 17·66)	8·88 (5·83 to 13·23)
Bahrain	0·33 (0·24 to 0·43)	0·23 (0·18 to 0·29)	0·16 (0·14 to 0·18)	0·15 (0·14 to 0·16)	0·16 (0·15 to 0·17)	0·13 (0·11 to 0·14)	0·09 (0·08 to 0·10)	0·08 (0·08 to 0·09)	0·08 (0·07 to 0·09)	0·07 (0·05 to 0·09)
Egypt	173·46 (155·36 to 192·14)	157·37 (140·41 to 174·88)	129·32 (112·88 to 145·96)	98·13 (87·51 to 109·93)	71·57 (64·18 to 79·38)	51·49 (45·99 to 57·79)	39·95 (34·49 to 45·88)	35·42 (30·01 to 41·32)	32·89 (27·07 to 39·93)	22·88 (15·31 to 33·87)
Iran	171·10 (130·27 to 214·87)	111·28 (85·83 to 140·62)	97·27 (73·37 to 123·03)	87·71 (69·00 to 107·39)	71·95 (58·23 to 87·41)	40·13 (29·93 to 52·50)	28·70 (18·76 to 41·26)	24·92 (11·91 to 44·19)	21·24 (8·71 to 40·01)	15·80 (6·35 to 33·93)
Iraq	30·70 (16·37 to 41·17)	28·50 (15·02 to 37·51)	25·17 (12·62 to 33·46)	24·18 (11·74 to 32·83)	19·54 (8·12 to 28·45)	21·17 (8·83 to 32·41)	19·84 (10·57 to 29·15)	21·26 (14·54 to 28·34)	25·02 (14·27 to 34·63)	25·12 (12·21 to 37·90)
Jordan	4·46 (3·97 to 4·95)	4·03 (3·68 to 4·37)	2·76 (2·51 to 3·03)	2·26 (1·99 to 2·52)	1·96 (1·72 to 2·21)	2·24 (1·93 to 2·61)	2·02 (1·78 to 2·26)	1·87 (1·65 to 2·11)	1·89 (1·62 to 2·18)	1·76 (1·40 to 2·25)
Kuwait	0·92 (0·88 to 0·97)	1·08 (1·04 to 1·13)	0·90 (0·85 to 0·94)	0·63 (0·60 to 0·67)	0·36 (0·31 to 0·41)	0·31 (0·29 to 0·33)	0·29 (0·27 to 0·31)	0·29 (0·27 to 0·31)	0·34 (0·32 to 0·36)	0·28 (0·19 to 0·40)
Lebanon	3·07 (2·43 to 3·88)	3·16 (2·44 to 3·92)	3·05 (2·10 to 4·07)	1·73 (1·32 to 2·20)	1·19 (0·95 to 1·49)	0·82 (0·62 to 1·07)	0·53 (0·39 to 0·71)	0·37 (0·24 to 0·55)	0·31 (0·20 to 0·45)	0·32 (0·22 to 0·44)
Libya	4·56 (3·71 to 5·43)	3·72 (3·13 to 4·32)	2·99 (2·48 to 3·56)	2·60 (2·02 to 3·23)	2·40 (1·84 to 2·99)	2·16 (1·70 to 2·65)	1·98 (1·48 to 2·53)	1·62 (1·16 to 2·17)	1·09 (0·74 to 1·50)	0·62 (0·41 to 0·88)
Morocco	57·29 (52·55 to 62·08)	50·94 (47·24 to 54·65)	44·07 (40·87 to 47·40)	36·84 (33·79 to 39·95)	28·94 (26·24 to 31·60)	21·93 (19·43 to 24·25)	16·16 (13·84 to 18·53)	11·78 (9·48 to 14·55)	8·46 (6·47 to 10·92)	5·46 (3·90 to 7·46)
Palestine	2·22 (1·26 to 3·20)	2·11 (1·06 to 2·98)	1·85 (0·79 to 2·62)	1·51 (0·61 to 2·22)	1·68 (0·71 to 2·46)	1·54 (0·74 to 2·22)	1·64 (0·70 to 2·56)	1·72 (0·91 to 2·60)	2·21 (1·37 to 3·03)	2·14 (1·05 to 3·37)
Oman	5·15 (4·46 to 5·90)	4·48 (3·70 to 5·25)	3·60 (2·88 to 4·34)	2·63 (2·11 to 3·24)	1·82 (1·44 to 2·31)	1·21 (0·96 to 1·54)	0·78 (0·63 to 0·96)	0·39 (0·33 to 0·45)	0·41 (0·34 to 0·48)	0·40 (0·29 to 0·53)
Qatar	0·14 (0·10 to 0·20)	0·15 (0·11 to 0·20)	0·13 (0·09 to 0·18)	0·14 (0·10 to 0·18)	0·13 (0·09 to 0·18)	0·11 (0·08 to 0·16)	0·10 (0·07 to 0·13)	0·10 (0·07 to 0·15)	0·13 (0·09 to 0·18)	0·14 (0·09 to 0·20)
Saudi Arabia	23·30 (20·47 to 26·34)	21·00 (19·04 to 23·13)	19·14 (16·97 to 21·50)	18·93 (16·50 to 21·73)	16·54 (14·21 to 19·37)	12·25 (10·25 to 14·82)	9·28 (7·68 to 11·46)	6·34 (5·27 to 7·80)	3·91 (3·22 to 4·76)	1·74 (1·39 to 2·20)
Sudan	38·35 (30·97 to 46·14)	41·83 (32·43 to 50·96)	44·25 (34·76 to 54·40)	66·84 (49·77 to 84·93)	42·93 (36·38 to 49·84)	46·13 (38·18 to 54·02)	45·74 (36·75 to 55·83)	40·24 (30·17 to 50·39)	38·89 (28·15 to 51·02)	30·94 (17·62 to 52·59)
Syria	14·43 (13·02 to 15·91)	13·06 (11·32 to 14·64)	11·86 (10·14 to 13·59)	11·15 (9·03 to 13·36)	10·46 (8·43 to 12·53)	7·75 (6·44 to 9·09)	5·14 (4·34 to 5·92)	4·32 (3·60 to 5·10)	4·45 (3·61 to 5·39)	4·20 (2·36 to 6·06)
Tunisia	15·10 (13·70 to 16·60)	12·10 (11·15 to 13·09)	9·87 (9·09 to 10·71)	7·86 (6·92 to 8·85)	5·85 (5·22 to 6·51)	3·93 (3·47 to 4·44)	2·55 (2·22 to 2·93)	2·01 (1·71 to 2·35)	1·79 (1·47 to 2·15)	1·13 (0·83 to 1·49)
Turkey	131·07 (111·31 to 150·35)	122·26 (102·36 to 144·11)	100·63 (80·69 to 122·95)	72·60 (56·90 to 88·29)	51·36 (41·31 to 63·05)	40·38 (33·30 to 48·25)	29·22 (24·08 to 34·90)	20·53 (16·47 to 25·30)	15·81 (11·68 to 21·13)	10·63 (7·22 to 15·23)
United Arab Emirates	0·64 (0·44 to 0·88)	0·79 (0·52 to 1·18)	1·04 (0·66 to 1·53)	0·95 (0·53 to 1·56)	0·71 (0·37 to 1·18)	0·59 (0·30 to 1·05)	0·45 (0·24 to 0·76)	0·36 (0·29 to 0·45)	0·61 (0·34 to 0·98)	0·50 (0·26 to 0·90)
Yemen	50·07 (24·00 to 65·14)	42·60 (20·41 to 54·50)	36·90 (18·92 to 47·02)	34·49 (18·82 to 43·75)	35·17 (24·76 to 43·08)	35·95 (21·95 to 46·85)	32·14 (17·26 to 43·91)	26·80 (14·71 to 37·78)	25·53 (14·32 to 36·59)	21·50 (9·62 to 34·62)
South Asia	2,876·67 (2,774·89 to 2,972·52)	2,701·53 (2,620·05 to 2,785·38)	2,471·88 (2,392·33 to 2,549·78)	2,268·79 (2,193·22 to 2,342·31)	1,995·19 (1,930·74 to 2,057·69)	1,712·50 (1,663·75 to 1,764·36)	1,467·41 (1,429·86 to 1,508·28)	1,252·13 (1,216·51 to 1,291·68)	991·97 (953·31 to 1,030·29)	636·49 (595·84 to 678·36)
South Asia	2,876·67 (2,774·89 to 2,972·52)	2,701·53 (2,620·05 to 2,785·38)	2,471·88 (2,392·33 to 2,549·78)	2,268·79 (2,193·22 to 2,342·31)	1,995·19 (1,930·74 to 2,057·69)	1,712·50 (1,663·75 to 1,764·36)	1,467·41 (1,429·86 to 1,508·28)	1,252·13 (1,216·51 to 1,291·68)	991·97 (953·31 to 1,030·29)	636·49 (595·84 to 678·36)
Bangladesh	342·24 (313·78 to 371·76)	289·76 (266·54 to 313·42)	273·35 (253·74 to 293·32)	269·25 (250·79 to 288·79)	241·63 (225·26 to 259·39)	188·24 (174·39 to 202·64)	145·55 (133·63 to 158·03)	116·31 (106·19 to 127·36)	85·56 (76·74 to 94·73)	51·84 (44·60 to 59·83)
Bhutan	1·82 (1·51 to 2·14)	1·82 (1·54 to 2·14)	1·75 (1·46 to 2·08)	1·56 (1·28 to 1·87)	1·37 (1·15 to 1·58)	1·03 (0·88 to 1·18)	0·85 (0·73 to 1·01)	0·66 (0·55 to 0·80)	0·46 (0·37 to 0·57)	0·27 (0·20 to 0·34)
India	2,245·47 (2,154·27 to 2,339·82)	2,122·95 (2,049·23 to 2,195·42)	1,899·56 (1,828·67 to 1,972·08)	1,687·86 (1,621·28 to 1,753·92)	1,465·50 (1,417·22 to 1,517·41)	1,273·21 (1,232·86 to 1,320·00)	1,105·30 (1,076·86 to 1,135·54)	935·83 (908·49 to 964·97)	712·56 (686·53 to 739·34)	438·02 (413·32 to 465·25)
Nepal	62·49 (47·27 to 77·14)	62·79 (47·63 to 78·15)	61·15 (45·60 to 75·84)	56·41 (43·41 to 68·60)	49·89 (38·30 to 62·28)	40·46 (32·32 to 48·82)	32·44 (25·85 to 39·40)	23·39 (18·27 to 28·75)	18·23 (14·57 to 22·22)	13·07 (9·53 to 17·45)
Pakistan	224·66 (195·22 to 252·75)	224·20 (201·19 to 247·42)	236·06 (214·15 to 259·06)	253·71 (232·61 to 277·59)	236·81 (212·32 to 263·21)	209·56 (184·98 to 234·14)	183·26 (165·18 to 201·82)	175·93 (157·55 to 196·71)	175·16 (149·31 to 204·75)	133·30 (101·11 to 170·03)
Sub-Saharan Africa	1,577·53 (1,478·55 to 1,673·38)	1,633·07 (1,530·71 to 1,724·78)	1,677·00 (1,584·59 to 1,759·51)	1,731·81 (1,648·70 to 1,809·69)	1,807·72 (1,727·50 to 1,880·84)	1,878·60 (1,777·12 to 1,977·09)	1,897·01 (1,794·51 to 1,995·54)	1,834·82 (1,741·68 to 1,925·62)	1,718·03 (1,613·91 to 1,828·88)	1,447·21 (1,341·46 to 1,568·31)
Southern Sub-Saharan Africa	63·48 (56·50 to 71·76)	64·39 (57·68 to 71·52)	66·00 (59·72 to 73·14)	64·21 (57·75 to 71·36)	59·28 (54·89 to 63·87)	57·76 (54·55 to 61·05)	64·83 (60·24 to 69·86)	70·39 (64·11 to 77·14)	62·90 (56·07 to 70·75)	47·64 (40·33 to 56·34)
Botswana	1·37 (0·96 to 1·81)	1·26 (0·86 to 1·67)	1·25 (0·89 to 1·61)	1·22 (0·85 to 1·63)	1·12 (0·78 to 1·49)	1·39 (0·90 to 1·93)	1·62 (0·97 to 2·43)	1·28 (0·81 to 1·87)	0·80 (0·53 to 1·16)	0·47 (0·27 to 0·77)
Lesotho	2·29 (1·82 to 2·80)	2·29 (1·84 to 2·82)	2·26 (1·77 to 2·85)	2·31 (1·73 to 2·99)	2·22 (1·64 to 2·88)	2·38 (1·80 to 3·03)	2·92 (2·29 to 3·64)	3·28 (2·76 to 3·91)	2·76 (2·24 to 3·38)	2·10 (1·57 to 2·75)
Namibia	1·54 (1·20 to 1·94)	1·67 (1·40 to 2·00)	1·84 (1·55 to 2·17)	1·77 (1·50 to 2·08)	2·01 (1·71 to 2·34)	1·91 (1·61 to 2·21)	2·16 (1·80 to 2·57)	2·20 (1·80 to 2·65)	1·97 (1·52 to 2·49)	1·48 (0·92 to 2·22)
South Africa	45·25 (38·47 to 53·14)	45·64 (39·19 to 52·75)	46·43 (40·47 to 53·43)	44·91 (38·96 to 51·70)	39·39 (35·16 to 43·83)	35·68 (32·99 to 38·33)	39·69 (35·79 to 43·61)	42·46 (36·81 to 48·81)	34·48 (28·38 to 41·78)	25·19 (19·31 to 32·98)
Swaziland	1·72 (1·16 to 2·45)	1·54 (1·10 to 2·08)	1·39 (1·07 to 1·77)	1·24 (1·01 to 1·49)	1·12 (0·90 to 1·36)	1·21 (0·97 to 1·45)	1·74 (1·42 to 2·06)	2·08 (1·75 to 2·43)	1·77 (1·49 to 2·06)	1·23 (0·93 to 1·59)
Zimbabwe	11·30 (9·67 to 13·07)	11·98 (10·70 to 13·38)	12·82 (11·55 to 14·14)	12·75 (11·44 to 13·99)	13·43 (12·27 to 14·62)	15·18 (13·71 to 16·83)	16·70 (14·15 to 19·33)	19·09 (17·15 to 21·21)	21·12 (18·57 to 23·84)	17·18 (13·46 to 21·84)
Western Sub-Saharan Africa	759·23 (682·48 to 837·93)	763·10 (691·46 to 831·00)	765·96 (702·63 to 831·56)	780·29 (722·65 to 837·77)	824·06 (778·30 to 873·66)	873·60 (808·14 to 934·72)	906·20 (840·00 to 968·08)	912·99 (843·31 to 987·39)	881·29 (803·59 to 962·92)	763·29 (686·58 to 845·33)
Benin	19·53 (17·75 to 21·38)	19·21 (17·57 to 20·80)	19·40 (17·84 to 20·99)	19·65 (17·86 to 21·46)	19·91 (17·84 to 22·04)	20·37 (18·18 to 22·59)	20·02 (17·74 to 22·35)	19·53 (17·31 to 22·09)	18·55 (15·92 to 21·75)	16·70 (13·67 to 20·43)
Burkina Faso	44·20 (31·81 to 54·72)	43·96 (31·64 to 54·48)	42·77 (31·70 to 52·83)	43·15 (32·38 to 52·42)	44·57 (36·06 to 51·73)	46·40 (38·93 to 53·71)	47·74 (38·14 to 56·29)	48·88 (37·29 to 59·69)	48·41 (35·88 to 60·75)	42·88 (29·61 to 55·98)
Cameroon	33·81 (31·26 to 36·45)	32·65 (30·01 to 35·27)	32·17 (29·52 to 35·01)	31·87 (29·43 to 34·55)	33·80 (31·21 to 36·57)	38·66 (34·94 to 42·39)	41·84 (36·83 to 47·50)	43·65 (37·99 to 49·91)	44·24 (37·34 to 52·07)	40·66 (32·02 to 51·69)
Cape Verde	0·86 (0·80 to 0·93)	0·63 (0·59 to 0·67)	0·47 (0·44 to 0·49)	0·56 (0·52 to 0·60)	0·43 (0·39 to 0·48)	0·52 (0·40 to 0·63)	0·34 (0·29 to 0·40)	0·28 (0·23 to 0·33)	0·21 (0·17 to 0·24)	0·15 (0·13 to 0·18)
Chad	23·20 (20·51 to 26·18)	23·96 (21·75 to 26·40)	24·91 (23·10 to 26·83)	25·95 (24·19 to 27·76)	26·57 (24·61 to 28·68)	28·81 (26·40 to 31·18)	33·08 (30·17 to 36·09)	35·63 (32·04 to 39·38)	36·64 (32·13 to 41·77)	36·67 (31·02 to 43·79)
Cote d'Ivoire	27·46 (23·25 to 31·26)	28·18 (24·16 to 31·84)	29·62 (26·29 to 33·34)	34·90 (31·16 to 38·60)	38·68 (33·35 to 43·99)	41·70 (36·25 to 47·53)	43·87 (37·73 to 50·12)	44·55 (35·13 to 54·12)	42·72 (32·93 to 52·94)	39·23 (28·90 to 50·89)
The Gambia	2·61 (2·12 to 3·14)	2·58 (2·14 to 3·09)	2·59 (2·21 to 3·01)	2·49 (2·11 to 2·88)	2·66 (2·23 to 3·11)	2·72 (2·30 to 3·19)	2·68 (2·29 to 3·10)	2·52 (2·06 to 2·98)	2·31 (1·88 to 2·84)	2·11 (1·65 to 2·60)
Ghana	36·43 (31·50 to 41·16)	35·48 (30·18 to 40·60)	35·21 (30·44 to 39·90)	37·84 (32·95 to 42·63)	37·17 (33·08 to 41·27)	34·88 (30·53 to 39·45)	33·54 (28·61 to 38·63)	35·25 (30·68 to 40·41)	35·72 (31·05 to 40·70)	27·72 (21·97 to 34·50)
Guinea	28·44 (24·43 to 32·69)	28·49 (25·58 to 31·57)	26·95 (24·67 to 29·32)	27·05 (25·09 to 29·16)	27·68 (25·91 to 29·52)	31·09 (28·88 to 33·46)	30·36 (27·69 to 33·28)	28·86 (25·65 to 32·20)	27·09 (23·26 to 31·59)	23·92 (19·27 to 30·12)
Guinea-Bissau	5·08 (4·43 to 5·85)	5·09 (4·48 to 5·78)	5·06 (4·52 to 5·65)	5·03 (4·55 to 5·52)	4·77 (4·32 to 5·25)	4·53 (4·14 to 4·91)	4·31 (3·92 to 4·69)	4·21 (3·78 to 4·67)	3·46 (3·05 to 3·86)	2·72 (2·34 to 3·17)

Appendix Table 12B. Under-5 death numbers by location, males, 1970, 1975, 1980, 1985, 1990, 1995, 2000, 2005, 2010, 2016

Location	1970	1975	1980	1985	1990	1995	2000	2005	2010	2016
Liberia	11·07 (9·80 to 12·51)	11·00 (9·82 to 12·21)	11·29 (10·03 to 12·63)	12·07 (10·74 to 13·40)	11·15 (10·13 to 12·30)	10·42 (9·46 to 11·36)	10·72 (9·82 to 11·69)	8·40 (7·54 to 9·29)	7·12 (6·17 to 8·15)	5·51 (4·48 to 6·70)
Mali	53·40 (49·14 to 57·93)	53·13 (49·47 to 57·06)	50·80 (47·56 to 54·20)	50·48 (47·50 to 53·58)	48·11 (45·23 to 51·06)	49·21 (46·14 to 52·50)	50·93 (47·25 to 55·25)	50·85 (45·97 to 56·28)	48·97 (43·04 to 55·64)	46·14 (37·35 to 57·07)
Mauritania	3·90 (3·69 to 4·14)	4·06 (3·80 to 4·32)	4·03 (3·77 to 4·31)	4·01 (3·71 to 4·33)	3·98 (3·67 to 4·29)	4·07 (3·73 to 4·40)	4·22 (3·86 to 4·59)	4·36 (3·89 to 4·84)	3·84 (3·32 to 4·39)	3·13 (2·63 to 3·72)
Niger	41·18 (37·41 to 45·34)	45·83 (41·79 to 49·77)	49·38 (45·04 to 53·83)	54·04 (49·63 to 58·98)	57·98 (53·86 to 62·29)	61·60 (57·20 to 65·97)	60·47 (55·42 to 65·77)	55·56 (50·07 to 61·61)	51·08 (44·23 to 59·23)	48·41 (39·81 to 59·32)
Nigeria	374·19 (300·95 to 452·89)	374·55 (302·66 to 442·54)	378·20 (314·04 to 443·32)	377·70 (324·77 to 433·68)	413·03 (369·81 to 458·95)	445·93 (383·29 to 506·32)	471·38 (407·94 to 533·48)	479·51 (414·33 to 549·83)	463·93 (388·18 to 542·96)	388·12 (314·15 to 469·09)
Sao Tome and Principe	0·23 (0·22 to 0·25)	0·20 (0·19 to 0·22)	0·22 (0·20 to 0·25)	0·27 (0·25 to 0·29)	0·25 (0·23 to 0·27)	0·24 (0·21 to 0·27)	0·22 (0·19 to 0·25)	0·20 (0·17 to 0·23)	0·15 (0·13 to 0·18)	0·12 (0·10 to 0·15)
Senegal	25·09 (22·69 to 27·78)	25·68 (23·49 to 27·96)	24·23 (22·08 to 26·49)	23·35 (21·40 to 25·25)	22·35 (20·57 to 24·16)	22·89 (21·09 to 24·84)	21·79 (19·77 to 23·80)	20·93 (19·04 to 22·83)	18·66 (16·76 to 20·71)	15·41 (13·33 to 17·68)
Sierra Leone	17·87 (15·69 to 20·37)	17·93 (15·97 to 20·07)	18·60 (16·91 to 20·33)	19·10 (17·73 to 20·58)	19·52 (18·27 to 20·81)	18·05 (17·05 to 19·08)	17·12 (16·03 to 18·16)	18·14 (16·81 to 19·42)	17·33 (15·69 to 18·92)	14·60 (12·79 to 16·67)
Togo	10·63 (8·86 to 12·47)	10·45 (8·69 to 12·16)	10·03 (8·17 to 11·92)	10·75 (9·14 to 12·42)	11·42 (9·89 to 13·00)	11·51 (9·78 to 13·37)	11·55 (9·31 to 13·90)	11·68 (8·76 to 14·94)	10·85 (8·34 to 13·46)	9·08 (6·96 to 11·44)
Eastern Sub-Saharan Africa	597·89 (570·79 to 626·13)	643·41 (617·10 to 669·44)	676·84 (651·62 to 700·16)	717·58 (693·01 to 740·78)	731·27 (707·85 to 754·51)	731·98 (707·49 to 755·55)	695·34 (673·71 to 716·72)	625·01 (602·16 to 647·81)	553·23 (527·40 to 579·84)	446·35 (416·75 to 477·30)
Burundi	20·86 (18·01 to 23·84)	21·39 (18·89 to 24·11)	22·32 (19·80 to 24·89)	22·14 (19·86 to 24·67)	23·48 (20·99 to 26·30)	23·87 (21·34 to 26·68)	22·81 (20·08 to 25·57)	20·97 (18·35 to 23·85)	20·50 (16·60 to 24·77)	21·02 (14·31 to 31·00)
Comoros	1·06 (0·84 to 1·28)	1·05 (0·90 to 1·21)	1·10 (0·97 to 1·25)	1·04 (0·91 to 1·18)	0·95 (0·83 to 1·07)	0·89 (0·78 to 1·00)	0·92 (0·79 to 1·06)	0·84 (0·70 to 1·00)	0·74 (0·59 to 0·92)	0·55 (0·38 to 0·78)
Djibouti	0·62 (0·50 to 0·74)	0·77 (0·64 to 0·92)	1·11 (0·92 to 1·30)	1·34 (1·13 to 1·55)	1·69 (1·40 to 1·98)	1·86 (1·51 to 2·21)	1·83 (1·50 to 2·18)	1·60 (1·25 to 2·00)	1·36 (0·97 to 1·85)	0·97 (0·63 to 1·48)
Eritrea	10·51 (8·90 to 12·33)	10·74 (9·26 to 12·18)	11·05 (9·77 to 12·38)	11·22 (9·82 to 12·74)	10·72 (9·40 to 12·09)	7·67 (6·61 to 8·78)	6·41 (5·42 to 7·54)	6·58 (5·24 to 8·03)	6·38 (4·96 to 8·07)	4·73 (3·49 to 6·36)
Ethiopia	164·98 (144·68 to 185·42)	186·03 (166·04 to 207·21)	186·07 (168·41 to 203·31)	187·15 (171·58 to 202·89)	196·58 (180·66 to 213·43)	196·68 (180·55 to 214·40)	182·45 (167·18 to 197·95)	153·85 (137·68 to 171·07)	118·75 (100·98 to 140·10)	81·04 (65·51 to 100·47)
Kenya	41·52 (40·55 to 42·54)	43·60 (42·60 to 44·63)	44·37 (43·15 to 45·49)	46·12 (45·04 to 47·24)	48·86 (47·53 to 50·18)	54·93 (53·50 to 56·53)	55·36 (53·90 to 56·99)	49·85 (48·50 to 51·38)	41·18 (39·51 to 42·83)	32·81 (31·19 to 34·73)
Madagascar	29·72 (25·88 to 34·32)	32·37 (28·49 to 36·73)	36·31 (32·09 to 40·85)	41·92 (37·09 to 47·21)	40·48 (36·45 to 44·58)	41·43 (37·06 to 46·08)	40·48 (35·60 to 45·54)	38·60 (33·46 to 43·78)	40·01 (33·25 to 46·96)	36·95 (29·06 to 47·55)
Malawi	45·20 (40·46 to 50·30)	44·06 (39·94 to 48·32)	42·69 (39·09 to 46·68)	43·97 (40·36 to 47·90)	51·82 (47·63 to 56·13)	46·08 (42·38 to 50·46)	42·08 (38·32 to 46·11)	34·65 (31·18 to 38·02)	32·43 (28·00 to 37·22)	26·19 (20·93 to 33·55)
Mozambique	56·74 (48·21 to 66·28)	59·51 (51·08 to 68·28)	65·96 (57·02 to 75·23)	78·55 (69·03 to 88·28)	61·94 (52·32 to 71·12)	66·17 (57·28 to 75·25)	64·19 (57·88 to 70·90)	59·79 (54·14 to 65·49)	55·38 (48·52 to 62·24)	44·83 (36·83 to 53·94)
Rwanda	24·04 (21·69 to 26·38)	28·04 (25·32 to 30·88)	28·95 (26·37 to 31·58)	27·58 (25·17 to 30·42)	29·30 (26·77 to 32·19)	27·21 (24·16 to 30·24)	25·71 (22·54 to 29·06)	20·53 (18·18 to 23·23)	13·93 (11·98 to 16·02)	10·63 (8·06 to 13·98)
Somalia	14·56 (10·07 to 19·84)	18·28 (13·93 to 23·62)	22·78 (17·45 to 29·00)	24·64 (18·60 to 30·90)	22·02 (15·93 to 28·79)	19·89 (13·92 to 26·27)	19·11 (13·99 to 24·69)	17·66 (13·38 to 22·53)	21·19 (16·54 to 26·74)	15·54 (10·60 to 21·32)
South Sudan	30·01 (22·48 to 39·50)	31·06 (23·62 to 39·52)	32·86 (26·57 to 40·06)	35·73 (30·29 to 41·50)	34·16 (28·75 to 39·63)	27·37 (23·35 to 31·46)	26·27 (22·81 to 29·42)	27·50 (23·69 to 31·81)	29·74 (24·37 to 36·01)	33·39 (25·30 to 43·62)
Tanzania	79·54 (71·63 to 87·56)	82·84 (75·26 to 90·17)	86·55 (77·98 to 95·07)	91·06 (82·59 to 99·58)	94·32 (85·07 to 103·51)	99·27 (89·90 to 108·23)	91·49 (84·61 to 98·92)	82·38 (74·60 to 90·52)	72·25 (61·39 to 83·60)	58·11 (46·29 to 71·18)
Uganda	56·98 (51·95 to 61·76)	61·92 (57·93 to 66·05)	70·52 (66·33 to 74·75)	75·92 (72·07 to 79·90)	81·28 (77·14 to 85·39)	84·37 (79·96 to 88·51)	82·25 (78·37 to 86·44)	79·45 (75·00 to 84·02)	73·11 (66·87 to 79·83)	58·04 (50·37 to 67·06)
Zambia	21·35 (18·68 to 24·31)	21·50 (19·14 to 23·89)	23·89 (21·51 to 26·65)	28·88 (26·19 to 32·07)	33·30 (29·61 to 37·21)	33·92 (30·68 to 37·59)	33·57 (29·99 to 37·21)	30·37 (26·67 to 33·89)	25·94 (21·97 to 30·41)	21·31 (16·84 to 26·74)
Central Sub-Saharan Africa	156·93 (101·91 to 211·82)	162·16 (105·92 to 218·96)	168·21 (115·59 to 214·82)	169·74 (116·71 to 215·21)	193·11 (132·29 to 245·56)	215·26 (146·54 to 276·48)	230·64 (157·08 to 297·86)	226·44 (167·76 to 281·42)	220·61 (154·00 to 285·06)	189·93 (129·71 to 261·84)
Angola	32·27 (22·46 to 43·93)	34·82 (26·35 to 44·51)	39·20 (31·42 to 48·34)	44·33 (37·46 to 52·09)	51·30 (43·49 to 59·46)	51·55 (43·54 to 60·56)	54·71 (46·19 to 63·90)	51·48 (43·14 to 61·09)	46·06 (37·10 to 56·46)	32·49 (21·59 to 46·73)
Central African Republic	6·45 (5·24 to 7·82)	7·13 (5·90 to 8·48)	7·61 (6·42 to 8·92)	8·81 (7·42 to 10·23)	9·86 (8·47 to 11·49)	11·07 (9·21 to 13·03)	12·48 (10·46 to 14·92)	12·64 (10·25 to 15·39)	12·70 (9·98 to 15·76)	12·22 (8·93 to 16·74)
Congo	4·74 (3·34 to 6·66)	4·63 (3·36 to 6·34)	4·41 (3·49 to 5·53)	4·14 (3·48 to 4·85)	4·55 (3·81 to 5·45)	5·44 (4·49 to 6·55)	6·46 (5·21 to 7·94)	6·06 (5·07 to 7·12)	5·50 (4·21 to 7·13)	4·67 (3·00 to 7·06)
Democratic Republic of the Congo	109·60 (54·85 to 167·59)	112·68 (57·82 to 166·74)	114·67 (62·01 to 160·40)	109·78 (58·88 to 155·24)	124·27 (64·76 to 175·98)	143·90 (75·13 to 205·39)	154·16 (85·49 to 217·67)	153·59 (95·08 to 208·27)	153·89 (87·97 to 217·46)	138·52 (80·43 to 206·68)
Equatorial Guinea	1·54 (1·09 to 2·05)	1·00 (0·76 to 1·28)	0·73 (0·58 to 0·91)	1·23 (0·96 to 1·53)	1·67 (1·31 to 2·10)	1·84 (1·46 to 2·26)	1·30 (1·01 to 1·65)	1·04 (0·74 to 1·40)	0·91 (0·61 to 1·30)	0·70 (0·45 to 1·07)
Gabon	2·31 (1·62 to 3·09)	1·90 (1·42 to 2·45)	1·59 (1·17 to 2·05)	1·46 (1·06 to 1·88)	1·46 (1·04 to 1·88)	1·46 (1·08 to 1·86)	1·53 (1·21 to 1·85)	1·63 (1·24 to 2·05)	1·54 (1·17 to 2·01)	1·33 (0·94 to 1·81)

Appendix Table 12C. Under-5 death numbers by location, females, 1970, 1975, 1980, 1985, 1990, 1995, 2000, 2005, 2010, 2016

Location	1970	1975	1980	1985	1990	1995	2000	2005	2010	2016
Global	7,639·63 (7,490·57 to 7,794·95)	6,817·15 (6,691·58 to 6,958·54)	6,243·46 (6,123·84 to 6,366·19)	5,780·29 (5,666·79 to 5,890·19)	5,188·36 (5,082·69 to 5,286·98)	4,628·79 (4,528·45 to 4,728·72)	4,085·55 (3,984·29 to 4,180·50)	3,571·53 (3,480·37 to 3,661·37)	3,073·77 (2,975·73 to 3,171·71)	2,291·72 (2,190·04 to 2,398·66)
High SDI	653·72 (609·51 to 702·00)	520·66 (488·55 to 551·75)	433·56 (404·68 to 462·53)	355·64 (333·71 to 378·24)	276·52 (260·77 to 293·33)	200·93 (189·40 to 214·15)	148·64 (139·39 to 160·30)	114·01 (103·05 to 129·70)	93·03 (82·46 to 108·31)	70·27 (61·60 to 84·32)
High-middle SDI	152·49 (148·82 to 156·69)	107·94 (104·95 to 111·34)	82·50 (80·11 to 85·33)	62·86 (61·53 to 64·47)	53·26 (52·38 to 54·47)	40·77 (40·19 to 41·38)	33·55 (33·36 to 33·74)	30·02 (29·84 to 30·20)	26·44 (26·28 to 26·60)	23·54 (22·90 to 24·29)
Middle SDI	3,020·42 (2,926·42 to 3,118·74)	2,852·49 (2,759·21 to 2,950·19)	2,753·74 (2,666·62 to 2,833·56)	2,653·13 (2,567·99 to 2,729·47)	2,436·83 (2,365·89 to 2,507·58)	2,241·16 (2,161·80 to 2,315·69)	2,005·32 (1,932·31 to 2,074·42)	1,759·12 (1,694·78 to 1,828·58)	1,496·70 (1,424·41 to 1,573·17)	1,058·34 (987·46 to 1,134·00)
Low-middle SDI	1,134·12 (1,066·83 to 1,196·44)	1,179·91 (1,113·80 to 1,241·66)	1,214·84 (1,149·84 to 1,278·31)	1,233·15 (1,169·97 to 1,301·70)	1,227·20 (1,163·08 to 1,287·04)	1,247·07 (1,174·60 to 1,310·84)	1,231·80 (1,162·79 to 1,293·93)	1,154·34 (1,099·17 to 1,209·66)	1,066·65 (1,002·27 to 1,130·48)	875·47 (814·28 to 942·02)
Low SDI	2,675·04 (2,592·34 to 2,759·81)	2,153·22 (2,091·56 to 2,215·79)	1,756·21 (1,701·98 to 1,811·38)	1,473·09 (1,432·25 to 1,511·51)	1,192·26 (1,159·66 to 1,225·16)	896·86 (874·21 to 919·42)	664·50 (648·03 to 680·60)	512·42 (498·18 to 526·51)	389·63 (376·29 to 402·70)	263·08 (248·88 to 278·76)
High-income	178·44 (174·69 to 182·70)	128·15 (125·08 to 131·57)	91·95 (89·58 to 94·83)	68·57 (67·21 to 70·12)	59·85 (58·91 to 61·12)	46·00 (45·41 to 46·61)	39·04 (38·60 to 39·47)	35·07 (34·69 to 35·44)	31·19 (30·88 to 31·51)	27·70 (26·89 to 28·50)
High-income North America	40·41 (39·90 to 40·94)	28·30 (27·98 to 28·62)	25·43 (25·26 to 25·59)	22·07 (21·94 to 22·22)	21·26 (21·11 to 21·42)	17·00 (16·88 to 17·12)	15·42 (15·32 to 15·53)	15·55 (15·43 to 15·67)	13·66 (13·57 to 13·76)	13·11 (12·82 to 13·40)
Canada	3·49 (3·39 to 3·58)	2·59 (2·51 to 2·66)	1·96 (1·90 to 2·02)	1·58 (1·53 to 1·63)	1·43 (1·38 to 1·48)	1·19 (1·15 to 1·23)	0·91 (0·88 to 0·93)	0·93 (0·89 to 0·97)	0·96 (0·92 to 0·99)	0·96 (0·83 to 1·11)
Greenland	0·03 (0·03 to 0·03)	0·02 (0·02 to 0·02)	0·02 (0·02 to 0·02)	0·02 (0·01 to 0·02)	0·02 (0·02 to 0·02)	0·01 (0·01 to 0·01)	0·01 (0·01 to 0·01)	0·01 (0·01 to 0·01)	0·01 (<0·01 to 0·01)	<0·01 (<0·01 to 0·01)
United States	36·87 (36·36 to 37·39)	25·68 (25·36 to 25·99)	23·44 (23·28 to 23·59)	20·47 (20·34 to 20·59)	19·80 (19·66 to 19·96)	15·79 (15·68 to 15·91)	14·50 (14·40 to 14·61)	14·61 (14·50 to 14·72)	12·70 (12·61 to 12·79)	12·14 (11·88 to 12·39)
Australasia	2·93 (2·87 to 2·99)	2·18 (2·14 to 2·23)	1·59 (1·55 to 1·62)	1·48 (1·44 to 1·52)	1·29 (1·25 to 1·32)	0·98 (0·96 to 1·01)	0·86 (0·84 to 0·89)	0·82 (0·79 to 0·84)	0·79 (0·77 to 0·82)	0·65 (0·59 to 0·72)
Australia	2·38 (2·32 to 2·44)	1·74 (1·70 to 1·79)	1·26 (1·22 to 1·29)	1·18 (1·15 to 1·22)	1·02 (0·99 to 1·05)	0·77 (0·75 to 0·79)	0·68 (0·66 to 0·70)	0·65 (0·63 to 0·67)	0·62 (0·60 to 0·64)	0·51 (0·45 to 0·58)
New Zealand	0·55 (0·53 to 0·57)	0·44 (0·43 to 0·46)	0·33 (0·32 to 0·35)	0·30 (0·29 to 0·31)	0·27 (0·26 to 0·28)	0·21 (0·20 to 0·22)	0·18 (0·18 to 0·19)	0·17 (0·16 to 0·17)	0·17 (0·16 to 0·18)	0·14 (0·12 to 0·16)
High-income Asia Pacific	39·97 (36·30 to 44·15)	27·20 (24·21 to 30·51)	17·13 (14·88 to 19·91)	10·25 (8·95 to 11·80)	7·23 (6·36 to 8·43)	5·75 (5·22 to 6·32)	4·29 (4·20 to 4·37)	3·04 (2·99 to 3·10)	2·48 (2·43 to 2·53)	1·87 (1·70 to 2·06)
Brunei	0·11 (0·11 to 0·12)	0·08 (0·08 to 0·09)	0·06 (0·05 to 0·06)	0·04 (0·04 to 0·04)	0·03 (0·03 to 0·04)	0·03 (0·03 to 0·03)	0·03 (0·03 to 0·03)	0·03 (0·03 to 0·03)	0·02 (0·02 to 0·03)	0·03 (0·02 to 0·03)
Japan	14·08 (13·70 to 14·45)	10·89 (10·62 to 11·15)	6·96 (6·89 to 7·04)	4·77 (4·71 to 4·83)	3·52 (3·48 to 3·57)	3·15 (3·06 to 3·25)	2·39 (2·36 to 2·43)	1·87 (1·84 to 1·91)	1·56 (1·54 to 1·59)	1·17 (1·12 to 1·21)
Singapore	0·54 (0·52 to 0·56)	0·33 (0·31 to 0·34)	0·25 (0·24 to 0·26)	0·19 (0·18 to 0·20)	0·15 (0·15 to 0·16)	0·11 (0·10 to 0·11)	0·07 (0·07 to 0·08)	0·05 (0·04 to 0·05)	0·04 (0·04 to 0·04)	0·03 (0·03 to 0·04)
South Korea	25·24 (21·61 to 29·50)	15·91 (12·95 to 19·18)	9·86 (7·60 to 12·65)	5·25 (3·96 to 6·80)	3·52 (2·65 to 4·73)	2·46 (1·95 to 3·03)	1·79 (1·72 to 1·86)	1·10 (1·05 to 1·15)	0·85 (0·81 to 0·89)	0·65 (0·49 to 0·83)
Western Europe	65·43 (64·88 to 65·97)	44·51 (44·15 to 44·86)	30·45 (30·18 to 30·72)	21·82 (21·64 to 22·02)	18·18 (18·01 to 18·35)	12·87 (12·75 to 13·00)	10·78 (10·68 to 10·89)	9·32 (9·21 to 9·42)	8·55 (8·47 to 8·65)	7·16 (6·66 to 7·68)
Andorra	<0·01 (<0·01 to <0·01)	<0·01 (<0·01 to <0·01)	<0·01 (<0·01 to <0·01)	<0·01 (<0·01 to <0·01)	<0·01 (<0·01 to <0·01)	<0·01 (<0·01 to <0·01)	<0·01 (<0·01 to <0·01)	<0·01 (<0·01 to <0·01)	<0·01 (<0·01 to <0·01)	<0·01 (<0·01 to <0·01)
Austria	1·42 (1·36 to 1·46)	0·94 (0·90 to 0·97)	0·64 (0·61 to 0·67)	0·48 (0·46 to 0·50)	0·37 (0·35 to 0·39)	0·26 (0·25 to 0·28)	0·19 (0·18 to 0·20)	0·16 (0·16 to 0·17)	0·15 (0·14 to 0·16)	0·12 (0·09 to 0·15)
Belgium	1·45 (1·43 to 1·48)	0·96 (0·94 to 0·98)	0·75 (0·73 to 0·77)	0·56 (0·55 to 0·58)	0·51 (0·50 to 0·52)	0·37 (0·36 to 0·38)	0·29 (0·28 to 0·29)	0·25 (0·24 to 0·26)	0·24 (0·23 to 0·25)	0·22 (0·18 to 0·26)
Cyprus	0·23 (0·21 to 0·27)	0·14 (0·13 to 0·15)	0·10 (0·10 to 0·11)	0·08 (0·07 to 0·08)	0·07 (0·06 to 0·07)	0·05 (0·04 to 0·05)	0·03 (0·03 to 0·03)	0·02 (0·02 to 0·03)	0·02 (0·02 to 0·02)	0·01 (0·01 to 0·02)
Denmark	0·50 (0·48 to 0·52)	0·35 (0·34 to 0·37)	0·24 (0·23 to 0·26)	0·22 (0·20 to 0·23)	0·24 (0·22 to 0·25)	0·20 (0·19 to 0·21)	0·16 (0·15 to 0·18)	0·14 (0·13 to 0·15)	0·12 (0·11 to 0·12)	0·11 (0·09 to 0·13)
Finland	0·44 (0·42 to 0·47)	0·35 (0·32 to 0·37)	0·24 (0·22 to 0·26)	0·20 (0·18 to 0·21)	0·19 (0·18 to 0·21)	0·14 (0·13 to 0·15)	0·10 (0·10 to 0·11)	0·10 (0·09 to 0·11)	0·08 (0·07 to 0·09)	0·06 (0·04 to 0·07)
France	6·60 (6·48 to 6·74)	5·20 (5·10 to 5·31)	4·10 (4·01 to 4·20)	3·23 (3·15 to 3·31)	2·82 (2·75 to 2·88)	1·89 (1·83 to 1·95)	1·77 (1·71 to 1·83)	1·48 (1·43 to 1·54)	1·45 (1·41 to 1·49)	1·32 (1·04 to 1·66)
Germany	11·63 (11·41 to 11·85)	7·33 (7·15 to 7·51)	5·42 (5·28 to 5·57)	3·85 (3·74 to 3·96)	3·30 (3·21 to 3·40)	2·18 (2·15 to 2·22)	1·78 (1·72 to 1·84)	1·44 (1·39 to 1·49)	1·23 (1·18 to 1·28)	1·11 (0·91 to 1·38)
Greece	2·19 (2·14 to 2·25)	1·68 (1·64 to 1·72)	1·27 (1·23 to 1·30)	0·78 (0·75 to 0·81)	0·48 (0·46 to 0·50)	0·39 (0·38 to 0·41)	0·29 (0·28 to 0·30)	0·22 (0·21 to 0·23)	0·21 (0·20 to 0·22)	0·16 (0·13 to 0·20)
Iceland	0·03 (0·03 to 0·03)	0·02 (0·02 to 0·03)	0·02 (0·02 to 0·02)	0·01 (0·01 to 0·01)	0·01 (0·01 to 0·01)	0·01 (0·01 to 0·01)	0·01 (0·01 to 0·01)	0·01 (0·01 to 0·01)	0·01 (<0·01 to 0·01)	<0·01 (<0·01 to 0·01)
Ireland	0·63 (0·61 to 0·66)	0·58 (0·56 to 0·61)	0·44 (0·42 to 0·46)	0·29 (0·28 to 0·30)	0·21 (0·20 to 0·23)	0·16 (0·15 to 0·17)	0·17 (0·16 to 0·18)	0·14 (0·13 to 0·14)	0·14 (0·13 to 0·15)	0·11 (0·08 to 0·15)
Israel	1·05 (0·95 to 1·16)	1·16 (1·13 to 1·20)	0·78 (0·75 to 0·81)	0·65 (0·62 to 0·68)	0·55 (0·53 to 0·57)	0·45 (0·43 to 0·47)	0·42 (0·40 to 0·43)	0·37 (0·36 to 0·39)	0·34 (0·32 to 0·35)	0·28 (0·24 to 0·32)
Italy	13·12 (12·84 to 13·41)	8·36 (8·17 to 8·55)	4·65 (4·52 to 4·78)	3·00 (2·91 to 3·09)	2·31 (2·24 to 2·39)	1·74 (1·67 to 1·80)	1·29 (1·26 to 1·32)	1·06 (1·01 to 1·10)	0·98 (0·94 to 1·02)	0·70 (0·51 to 0·97)
Luxembourg	0·04 (0·04 to 0·05)	0·03 (0·02 to 0·03)	0·02 (0·02 to 0·02)	0·02 (0·02 to 0·02)	0·02 (0·02 to 0·02)	0·01 (0·01 to 0·02)	0·01 (0·01 to 0·01)	0·01 (0·01 to 0·01)	0·01 (0·01 to 0·01)	0·01 (0·01 to 0·01)
Malta	0·06 (0·06 to 0·07)	0·05 (0·05 to 0·05)	0·04 (0·04 to 0·04)	0·03 (0·03 to 0·04)	0·03 (0·02 to 0·03)	0·02 (0·02 to 0·02)	0·01 (0·01 to 0·02)	0·01 (0·01 to 0·01)	0·01 (0·01 to 0·01)	0·01 (0·01 to 0·01)
Netherlands	1·59 (1·54 to 1·65)	1·04 (1·01 to 1·08)	0·81 (0·79 to 0·85)	0·74 (0·71 to 0·77)	0·71 (0·68 to 0·74)	0·57 (0·54 to 0·59)	0·56 (0·53 to 0·58)	0·46 (0·44 to 0·48)	0·36 (0·34 to 0·37)	0·29 (0·24 to 0·36)
Norway	0·44 (0·42 to 0·46)	0·31 (0·30 to 0·33)	0·21 (0·20 to 0·23)	0·22 (0·21 to 0·23)	0·21 (0·20 to 0·23)	0·14 (0·13 to 0·15)	0·12 (0·12 to 0·13)	0·10 (0·09 to 0·10)	0·08 (0·08 to 0·09)	0·07 (0·06 to 0·09)
Portugal	5·31 (5·20 to 5·43)	3·33 (3·24 to 3·42)	1·95 (1·92 to 1·99)	1·17 (1·14 to 1·19)	0·71 (0·69 to 0·73)	0·45 (0·44 to 0·47)	0·35 (0·34 to 0·36)	0·23 (0·23 to 0·24)	0·16 (0·16 to 0·17)	0·11 (0·10 to 0·13)
Spain	9·06 (8·85 to 9·29)	6·24 (6·08 to 6·39)	3·62 (3·53 to 3·73)	2·14 (2·07 to 2·21)	1·61 (1·55 to 1·67)	1·10 (1·05 to 1·14)	0·92 (0·89 to 0·96)	0·92 (0·89 to 0·97)	0·84 (0·81 to 0·88)	0·62 (0·47 to 0·81)
Sweden	0·62 (0·57 to 0·66)	0·45 (0·42 to 0·48)	0·33 (0·31 to 0·35)	0·30 (0·28 to 0·32)	0·36 (0·34 to 0·38)	0·21 (0·20 to 0·22)	0·15 (0·14 to 0·16)	0·15 (0·14 to 0·16)	0·15 (0·14 to 0·16)	0·13 (0·11 to 0·17)
Stockholm	0·09 (0·07 to 0·12)	0·07 (0·05 to 0·09)	0·05 (0·05 to 0·06)	0·05 (0·04 to 0·05)	0·06 (0·05 to 0·07)	0·04 (0·03 to 0·04)	0·03 (0·03 to 0·03)	0·03 (0·03 to 0·03)	0·03 (0·02 to 0·03)	0·03 (0·02 to 0·04)
Sweden except Stockholm	0·53 (0·49 to 0·56)	0·38 (0·36 to 0·41)	0·28 (0·26 to 0·30)	0·25 (0·24 to 0·27)	0·30 (0·28 to 0·31)	0·17 (0·16 to 0·18)	0·12 (0·11 to 0·13)	0·12 (0·11 to 0·13)	0·13 (0·12 to 0·14)	0·11 (0·08 to 0·14)
Switzerland	0·76 (0·73 to 0·79)	0·45 (0·43 to 0·47)	0·32 (0·31 to 0·34)	0·28 (0·27 to 0·29)	0·30 (0·28 to 0·31)	0·22 (0·21 to 0·23)	0·19 (0·18 to 0·20)	0·16 (0·15 to 0·17)	0·16 (0·15 to 0·17)	0·15 (0·12 to 0·19)
United Kingdom	8·19 (8·00 to 8·38)	5·50 (5·37 to 5·62)	4·45 (4·39 to 4·51)	3·56 (3·52 to 3·60)	3·16 (3·12 to 3·19)	2·31 (2·28 to 2·34)	1·95 (1·92 to 1·97)	1·88 (1·86 to 1·90)	1·80 (1·78 to 1·83)	1·55 (1·51 to 1·60)
England	6·51 (6·38 to 6·63)	4·36 (4·27 to 4·45)	3·59 (3·54 to 3·65)	2·92 (2·89 to 2·95)	2·66 (2·63 to 2·69)	1·93 (1·91 to 1·95)	1·65 (1·63 to 1·67)	1·60 (1·58 to 1·62)	1·55 (1·53 to 1·57)	1·34 (1·32 to 1·37)
East Midlands	0·49 (0·47 to 0·52)	0·34 (0·33 to 0·36)	0·29 (0·27 to 0·30)	0·22 (0·21 to 0·23)	0·21 (0·20 to 0·22)	0·15 (0·14 to 0·16)	0·13 (0·12 to 0·13)	0·13 (0·12 to 0·13)	0·12 (0·12 to 0·13)	0·12 (0·11 to 0·12)
East of England	0·54 (0·50 to 0·58)	0·37 (0·34 to 0·41)	0·31 (0·29 to 0·33)	0·26 (0·25 to 0·27)	0·21 (0·20 to 0·22)	0·17 (0·16 to 0·18)	0·14 (0·13 to 0·14)	0·14 (0·14 to 0·15)	0·14 (0·14 to 0·15)	0·13 (0·12 to 0·14)
Greater London	1·10 (1·07 to 1·14)	0·75 (0·72 to 0·78)	0·62 (0·60 to 0·64)	0·50 (0·49 to 0·51)	0·49 (0·48 to 0·50)	0·37 (0·36 to 0·38)	0·34 (0·33 to 0·35)	0·31 (0·31 to 0·32)	0·32 (0·32 to 0·33)	0·24 (0·23 to 0·24)
North East England	0·38 (0·36 to 0·41)	0·24 (0·23 to 0·26)	0·20 (0·19 to 0·21)	0·15 (0·15 to 0·16)	0·14 (0·13 to 0·14)	0·10 (0·09 to 0·10)	0·08 (0·07 to 0·08)	0·07 (0·06 to 0·07)	0·06 (0·05 to 0·06)	0·05 (0·05 to 0·06)
North West England	1·11 (1·05 to 1·18)	0·72 (0·67 to 0·77)	0·58 (0·55 to 0·60)	0·46 (0·45 to 0·47)	0·40 (0·39 to 0·41)	0·28 (0·27 to 0·29)	0·24 (0·23 to 0·25)	0·23 (0·23 to 0·24)	0·22 (0·21 to 0·23)	0·19 (0·18 to 0·19)
South East England	0·78 (0·74 to 0·82)	0·54 (0·52 to 0·57)	0·46 (0·44 to 0·48)	0·40 (0·39 to 0·41)	0·36 (0·36 to 0·38)	0·25 (0·24 to 0·25)	0·20 (0·19 to 0·21)	0·20 (0·19 to 0·20)	0·20 (0·19 to 0·21)	0·16 (0·16 to 0·17)
South West England	0·47 (0·45 to 0·50)	0·33 (0·31 to 0·35)	0·28 (0·26 to 0·29)	0·23 (0·22 to 0·23)	0·20 (0·19 to 0·20)	0·15 (0·15 to 0·16)	0·12 (0·11 to 0·13)	0·12 (0·11 to 0·12)	0·11 (0·11 to 0·12)	0·11 (0·10 to 0·11)
West Midlands	0·86 (0·80 to 0·92)	0·56 (0·51 to 0·61)	0·45 (0·43 to 0·48)	0·37 (0·36 to 0·38)	0·35 (0·34 to 0·37)	0·23 (0·23 to 0·24)	0·21 (0·20 to 0·22)	0·22 (0·21 to 0·22)	0·20 (0·19 to 0·21)	0·19 (0·18 to 0·21)

Appendix Table 12C. Under-5 death numbers by location, females, 1970, 1975, 1980, 1985, 1990, 1995, 2000, 2005, 2010, 2016

Location	1970	1975	1980	1985	1990	1995	2000	2005	2010	2016
Yorkshire and the Humber	0·76 (0·72 to 0·79)	0·50 (0·48 to 0·53)	0·41 (0·40 to 0·43)	0·33 (0·32 to 0·34)	0·30 (0·29 to 0·30)	0·23 (0·22 to 0·23)	0·20 (0·19 to 0·20)	0·19 (0·19 to 0·20)	0·17 (0·17 to 0·18)	0·15 (0·15 to 0·16)
Northern Ireland	0·36 (0·33 to 0·39)	0·27 (0·25 to 0·28)	0·20 (0·19 to 0·22)	0·15 (0·14 to 0·16)	0·11 (0·10 to 0·12)	0·08 (0·08 to 0·09)	0·06 (0·06 to 0·07)	0·06 (0·06 to 0·07)	0·07 (0·06 to 0·07)	0·05 (0·03 to 0·06)
Scotland	0·85 (0·82 to 0·88)	0·57 (0·55 to 0·59)	0·41 (0·39 to 0·43)	0·32 (0·30 to 0·34)	0·26 (0·24 to 0·27)	0·19 (0·18 to 0·20)	0·15 (0·14 to 0·16)	0·14 (0·13 to 0·15)	0·12 (0·11 to 0·12)	0·10 (0·08 to 0·14)
Wales	0·46 (0·35 to 0·61)	0·30 (0·23 to 0·40)	0·24 (0·21 to 0·28)	0·18 (0·16 to 0·19)	0·13 (0·12 to 0·14)	0·10 (0·10 to 0·11)	0·08 (0·08 to 0·09)	0·07 (0·07 to 0·08)	0·07 (0·07 to 0·08)	0·06 (0·05 to 0·08)
Southern Latin America	29·70 (29·29 to 30·08)	25·95 (25·44 to 26·53)	17·36 (17·16 to 17·56)	12·95 (12·80 to 13·10)	11·89 (11·76 to 12·04)	9·40 (9·28 to 9·53)	7·69 (7·27 to 8·11)	6·34 (6·02 to 6·67)	5·71 (5·43 to 5·99)	4·90 (4·38 to 5·48)
Argentina	17·55 (17·23 to 17·88)	16·95 (16·43 to 17·51)	12·29 (12·12 to 12·46)	9·43 (9·30 to 9·57)	8·80 (8·67 to 8·93)	7·16 (7·05 to 7·28)	6·08 (5·68 to 6·49)	5·06 (4·75 to 5·39)	4·50 (4·23 to 4·77)	3·88 (3·39 to 4·42)
Chile	10·79 (10·57 to 11·00)	7·60 (7·44 to 7·77)	4·10 (4·00 to 4·19)	2·75 (2·68 to 2·82)	2·54 (2·49 to 2·60)	1·72 (1·68 to 1·75)	1·24 (1·21 to 1·27)	0·99 (0·96 to 1·02)	0·99 (0·91 to 1·07)	0·85 (0·72 to 1·01)
Uruguay	1·36 (1·31 to 1·40)	1·40 (1·36 to 1·45)	0·97 (0·94 to 1·01)	0·76 (0·73 to 0·79)	0·55 (0·53 to 0·58)	0·52 (0·48 to 0·57)	0·37 (0·34 to 0·39)	0·29 (0·27 to 0·32)	0·21 (0·18 to 0·25)	0·18 (0·12 to 0·26)
Central Europe, Eastern Europe, and Central Asi	146·64 (137·30 to 156·64)	132·74 (126·31 to 139·21)	123·14 (118·30 to 128·49)	117·34 (113·27 to 121·60)	94·26 (90·93 to 97·86)	74·36 (71·36 to 77·70)	54·14 (51·43 to 56·97)	44·67 (41·26 to 48·54)	39·40 (35·22 to 44·56)	32·82 (28·48 to 38·54)
Eastern Europe	40·74 (36·39 to 46·35)	42·39 (38·84 to 46·86)	40·51 (38·04 to 43·53)	38·32 (36·78 to 40·26)	26·81 (25·66 to 28·09)	20·01 (19·04 to 21·05)	15·55 (14·72 to 16·59)	12·15 (11·42 to 13·00)	10·39 (9·71 to 11·22)	9·23 (8·26 to 10·45)
Belarus	2·24 (1·80 to 2·76)	1·85 (1·50 to 2·22)	1·68 (1·38 to 2·03)	1·65 (1·38 to 1·97)	1·23 (1·02 to 1·48)	0·82 (0·68 to 0·98)	0·62 (0·50 to 0·75)	0·45 (0·36 to 0·55)	0·36 (0·28 to 0·45)	0·26 (0·20 to 0·35)
Estonia	0·19 (0·18 to 0·20)	0·19 (0·18 to 0·20)	0·20 (0·19 to 0·22)	0·20 (0·18 to 0·21)	0·15 (0·14 to 0·16)	0·11 (0·10 to 0·12)	0·06 (0·05 to 0·07)	0·04 (0·04 to 0·05)	0·03 (0·03 to 0·04)	0·02 (0·01 to 0·03)
Latvia	0·31 (0·29 to 0·33)	0·36 (0·34 to 0·39)	0·31 (0·29 to 0·33)	0·29 (0·27 to 0·32)	0·28 (0·26 to 0·30)	0·20 (0·19 to 0·22)	0·11 (0·11 to 0·12)	0·09 (0·08 to 0·10)	0·07 (0·06 to 0·08)	0·05 (0·03 to 0·07)
Lithuania	0·55 (0·52 to 0·57)	0·53 (0·50 to 0·56)	0·41 (0·39 to 0·43)	0·46 (0·44 to 0·48)	0·33 (0·31 to 0·35)	0·28 (0·27 to 0·30)	0·16 (0·15 to 0·17)	0·11 (0·10 to 0·12)	0·08 (0·08 to 0·09)	0·06 (0·05 to 0·07)
Moldova	2·34 (1·53 to 3·39)	2·16 (1·45 to 3·05)	2·06 (1·62 to 2·56)	1·70 (1·39 to 2·04)	1·10 (0·90 to 1·32)	0·87 (0·73 to 1·05)	0·58 (0·47 to 0·71)	0·32 (0·24 to 0·41)	0·31 (0·23 to 0·40)	0·22 (0·15 to 0·32)
Russia	22·58 (22·28 to 22·91)	26·13 (25·76 to 26·49)	25·75 (25·36 to 26·15)	26·24 (25·82 to 26·64)	18·02 (17·78 to 18·27)	13·30 (13·06 to 13·54)	10·59 (10·33 to 10·86)	8·35 (8·21 to 8·50)	7·16 (7·05 to 7·29)	6·74 (6·26 to 7·28)
Ukraine	12·55 (8·08 to 18·25)	11·17 (7·76 to 15·57)	10·08 (7·66 to 12·98)	7·78 (6·28 to 9·66)	5·70 (4·63 to 6·97)	4·42 (3·52 to 5·46)	3·43 (2·65 to 4·38)	2·78 (2·10 to 3·62)	2·38 (1·75 to 3·16)	1·88 (1·06 to 3·12)
Central Europe	37·36 (35·43 to 39·73)	31·37 (29·82 to 33·22)	25·45 (24·40 to 26·74)	20·76 (20·03 to 21·54)	16·19 (15·74 to 16·64)	11·19 (10·92 to 11·45)	7·06 (6·87 to 7·27)	5·26 (5·10 to 5·42)	4·02 (3·90 to 4·15)	2·96 (2·57 to 3·42)
Albania	3·05 (1·81 to 4·84)	2·38 (1·45 to 3·65)	2·07 (1·47 to 2·83)	2·02 (1·66 to 2·45)	1·59 (1·32 to 1·89)	1·21 (1·01 to 1·42)	0·64 (0·52 to 0·78)	0·37 (0·29 to 0·48)	0·26 (0·18 to 0·37)	0·23 (0·14 to 0·35)
Bosnia and Herzegovina	2·02 (1·47 to 2·78)	1·44 (1·08 to 1·89)	1·02 (0·82 to 1·28)	0·82 (0·78 to 0·87)	0·50 (0·48 to 0·52)	0·54 (0·48 to 0·61)	0·16 (0·15 to 0·17)	0·12 (0·11 to 0·13)	0·10 (0·09 to 0·11)	0·07 (0·06 to 0·08)
Bulgaria	1·89 (1·83 to 1·95)	1·70 (1·64 to 1·76)	1·35 (1·30 to 1·39)	0·98 (0·94 to 1·02)	0·83 (0·80 to 0·87)	0·59 (0·56 to 0·61)	0·50 (0·47 to 0·53)	0·38 (0·36 to 0·41)	0·35 (0·33 to 0·37)	0·23 (0·14 to 0·37)
Croatia	1·00 (0·71 to 1·35)	0·79 (0·59 to 1·02)	0·64 (0·53 to 0·77)	0·49 (0·46 to 0·52)	0·30 (0·28 to 0·31)	0·26 (0·23 to 0·29)	0·16 (0·15 to 0·17)	0·12 (0·11 to 0·13)	0·10 (0·09 to 0·11)	0·07 (0·06 to 0·09)
Czech Republic	1·45 (1·40 to 1·50)	1·69 (1·64 to 1·74)	1·20 (1·15 to 1·25)	0·81 (0·77 to 0·84)	0·64 (0·61 to 0·68)	0·36 (0·35 to 0·39)	0·20 (0·19 to 0·22)	0·18 (0·17 to 0·19)	0·17 (0·15 to 0·18)	0·13 (0·10 to 0·17)
Hungary	2·61 (2·52 to 2·71)	2·96 (2·85 to 3·07)	1·61 (1·55 to 1·67)	1·26 (1·21 to 1·31)	0·93 (0·89 to 0·98)	0·60 (0·57 to 0·64)	0·44 (0·42 to 0·47)	0·32 (0·29 to 0·34)	0·25 (0·23 to 0·27)	0·22 (0·16 to 0·28)
Macedonia	1·37 (0·76 to 2·28)	1·08 (0·58 to 1·81)	1·01 (0·70 to 1·42)	0·75 (0·54 to 1·02)	0·56 (0·54 to 0·59)	0·34 (0·32 to 0·36)	0·18 (0·16 to 0·19)	0·14 (0·13 to 0·15)	0·10 (0·09 to 0·11)	0·10 (0·06 to 0·15)
Montenegro	0·16 (0·11 to 0·23)	0·13 (0·09 to 0·19)	0·11 (0·07 to 0·16)	0·09 (0·06 to 0·13)	0·06 (0·05 to 0·08)	0·06 (0·05 to 0·07)	0·06 (0·05 to 0·06)	0·03 (0·03 to 0·04)	0·02 (0·02 to 0·02)	0·02 (0·01 to 0·02)
Poland	8·59 (8·38 to 8·81)	7·58 (7·40 to 7·79)	6·96 (6·79 to 7·15)	5·96 (5·76 to 6·17)	4·26 (4·13 to 4·39)	2·93 (2·83 to 3·05)	1·56 (1·50 to 1·63)	1·20 (1·15 to 1·26)	1·05 (0·99 to 1·10)	0·75 (0·47 to 1·16)
Romania	11·01 (10·79 to 11·23)	7·77 (7·61 to 7·92)	6·25 (6·12 to 6·39)	4·99 (4·87 to 5·11)	4·74 (4·63 to 4·85)	2·67 (2·59 to 2·76)	2·22 (2·15 to 2·29)	1·66 (1·59 to 1·74)	1·05 (1·00 to 1·09)	0·71 (0·58 to 0·88)
Serbia	2·82 (1·87 to 4·06)	2·45 (1·67 to 3·52)	2·07 (1·43 to 2·86)	1·73 (1·25 to 2·30)	1·19 (0·92 to 1·52)	1·22 (1·18 to 1·26)	0·66 (0·54 to 0·79)	0·49 (0·41 to 0·59)	0·37 (0·35 to 0·39)	0·27 (0·22 to 0·32)
Slovakia	1·04 (0·99 to 1·08)	1·12 (1·09 to 1·17)	0·94 (0·90 to 0·98)	0·70 (0·67 to 0·73)	0·49 (0·46 to 0·51)	0·34 (0·31 to 0·36)	0·23 (0·21 to 0·25)	0·20 (0·18 to 0·21)	0·18 (0·17 to 0·19)	0·15 (0·11 to 0·21)
Slovenia	0·34 (0·25 to 0·46)	0·28 (0·22 to 0·36)	0·23 (0·19 to 0·26)	0·16 (0·15 to 0·17)	0·10 (0·09 to 0·10)	0·06 (0·05 to 0·06)	0·04 (0·04 to 0·04)	0·03 (0·03 to 0·04)	0·03 (0·03 to 0·03)	0·02 (0·02 to 0·03)
Central Asia	68·54 (61·05 to 76·44)	58·98 (54·29 to 64·04)	57·18 (53·34 to 61·64)	58·26 (54·57 to 62·04)	51·26 (48·25 to 54·54)	43·17 (40·40 to 46·34)	31·53 (29·04 to 34·32)	27·27 (23·99 to 31·01)	25·00 (20·88 to 30·11)	20·63 (16·58 to 26·06)
Armenia	3·10 (2·15 to 4·36)	2·59 (1·98 to 3·35)	2·57 (2·17 to 3·06)	2·23 (1·91 to 2·59)	1·58 (1·37 to 1·83)	0·90 (0·77 to 1·05)	0·47 (0·39 to 0·56)	0·37 (0·28 to 0·47)	0·29 (0·22 to 0·36)	0·22 (0·16 to 0·30)
Azerbaijan	8·82 (6·28 to 11·90)	6·50 (4·82 to 8·48)	6·68 (5·34 to 8·28)	7·18 (6·01 to 8·41)	6·53 (5·46 to 7·75)	5·31 (4·50 to 6·27)	3·38 (2·84 to 4·02)	3·28 (2·60 to 4·07)	3·19 (2·38 to 4·12)	2·43 (1·67 to 3·42)
Georgia	4·07 (3·12 to 5·24)	3·18 (2·56 to 3·89)	2·40 (1·97 to 2·89)	1·99 (1·69 to 2·33)	1·64 (1·42 to 1·87)	1·11 (0·97 to 1·27)	0·81 (0·71 to 0·93)	0·66 (0·54 to 0·81)	0·57 (0·43 to 0·73)	0·42 (0·31 to 0·56)
Kazakhstan	10·62 (7·79 to 13·79)	9·26 (7·69 to 11·11)	8·27 (7·02 to 9·58)	7·41 (6·51 to 8·41)	6·06 (5·30 to 6·79)	5·07 (4·28 to 5·93)	3·79 (3·11 to 4·59)	3·64 (2·87 to 4·52)	3·15 (2·50 to 3·90)	2·23 (1·64 to 2·95)
Kyrgyzstan	5·95 (4·81 to 7·37)	4·87 (4·11 to 5·72)	4·49 (3·93 to 5·08)	4·50 (4·08 to 4·93)	3·75 (3·45 to 4·04)	2·77 (2·53 to 3·01)	2·18 (2·00 to 2·38)	1·95 (1·77 to 2·15)	2·12 (1·89 to 2·38)	1·90 (1·54 to 2·28)
Mongolia	3·65 (2·88 to 4·48)	3·40 (2·75 to 4·07)	3·47 (2·87 to 4·12)	3·45 (2·99 to 3·94)	2·90 (2·56 to 3·23)	1·86 (1·64 to 2·08)	1·24 (1·09 to 1·39)	0·90 (0·78 to 1·03)	0·82 (0·69 to 0·96)	0·69 (0·53 to 0·88)
Tajikistan	10·39 (7·90 to 13·57)	9·85 (8·08 to 11·82)	9·18 (7·65 to 10·87)	9·35 (8·05 to 10·65)	8·83 (7·51 to 10·30)	7·38 (6·28 to 8·60)	5·41 (4·44 to 6·65)	3·83 (3·15 to 4·64)	3·78 (2·94 to 4·80)	4·00 (2·42 to 6·17)
Turkmenistan	5·57 (3·90 to 7·58)	5·00 (3·43 to 6·99)	4·82 (3·76 to 6·12)	5·04 (4·16 to 6·00)	5·00 (4·28 to 5·77)	4·54 (3·83 to 5·38)	3·49 (2·63 to 4·56)	2·80 (1·94 to 3·78)	2·36 (1·55 to 3·30)	1·87 (1·24 to 2·76)
Uzbekistan	16·38 (11·56 to 22·07)	14·32 (11·22 to 17·93)	15·28 (12·46 to 18·36)	17·10 (14·49 to 20·10)	14·98 (12·64 to 17·44)	14·23 (12·18 to 16·70)	10·75 (8·82 to 13·00)	9·84 (7·10 to 13·18)	8·72 (5·08 to 13·42)	6·88 (3·65 to 11·61)
Latin America and Caribbean	470·41 (458·59 to 483·42)	427·19 (417·36 to 437·06)	373·37 (364·46 to 382·60)	315·92 (309·08 to 322·90)	257·05 (251·06 to 263·71)	205·93 (201·06 to 210·94)	158·85 (153·69 to 164·35)	128·35 (124·24 to 132·86)	118·80 (107·14 to 131·62)	80·94 (76·60 to 86·70)
Central Latin America	180·88 (176·20 to 185·75)	163·65 (159·52 to 167·98)	138·24 (134·69 to 142·07)	116·22 (113·28 to 119·40)	97·30 (94·56 to 100·03)	81·81 (79·56 to 83·94)	65·10 (63·05 to 67·14)	52·45 (50·62 to 54·50)	42·91 (41·12 to 44·79)	32·96 (31·31 to 34·87)
Colombia	29·39 (26·68 to 32·50)	25·14 (22·84 to 27·42)	19·12 (17·13 to 21·11)	16·83 (15·12 to 18·72)	13·42 (11·67 to 15·28)	12·01 (10·74 to 13·29)	9·91 (8·88 to 11·01)	8·64 (7·61 to 9·91)	6·94 (5·83 to 8·22)	4·61 (3·75 to 5·75)
Costa Rica	1·87 (1·74 to 2·02)	1·25 (1·15 to 1·36)	0·96 (0·85 to 1·06)	0·83 (0·73 to 0·94)	0·69 (0·60 to 0·79)	0·57 (0·47 to 0·69)	0·50 (0·41 to 0·61)	0·43 (0·35 to 0·52)	0·37 (0·28 to 0·48)	0·27 (0·18 to 0·40)
El Salvador	9·68 (8·40 to 10·97)	8·49 (7·60 to 9·44)	6·94 (6·26 to 7·67)	5·21 (4·66 to 5·79)	3·66 (3·23 to 4·15)	2·70 (2·30 to 3·16)	1·89 (1·63 to 2·18)	1·21 (0·96 to 1·50)	0·82 (0·64 to 1·03)	0·54 (0·38 to 0·75)
Guatemala	20·31 (18·31 to 22·55)	18·59 (16·70 to 20·61)	16·47 (14·83 to 18·21)	14·53 (13·15 to 15·94)	12·93 (11·86 to 14·08)	11·24 (10·31 to 12·17)	9·48 (8·52 to 10·45)	7·90 (7·05 to 8·74)	6·22 (5·51 to 7·06)	4·85 (4·06 to 5·74)
Honduras	7·70 (6·95 to 8·48)	7·09 (6·47 to 7·74)	6·08 (5·47 to 6·68)	5·28 (4·76 to 5·81)	4·46 (3·93 to 4·98)	3·63 (3·06 to 4·17)	2·89 (2·43 to 3·35)	2·39 (2·05 to 2·76)	2·16 (1·84 to 2·55)	1·82 (1·44 to 2·26)
Mexico	92·34 (89·60 to 95·16)	85·03 (82·22 to 87·81)	74·28 (72·16 to 76·66)	60·96 (59·24 to 62·64)	50·79 (49·46 to 52·29)	41·29 (39·97 to 42·68)	32·21 (31·01 to 33·40)	24·94 (23·72 to 26·16)	20·21 (19·14 to 21·37)	15·47 (14·49 to 16·55)
Nicaragua	5·73 (5·34 to 6·16)	5·11 (4·82 to 5·42)	4·51 (4·27 to 4·78)	4·53 (4·10 to 4·96)	3·46 (3·26 to 3·66)	3·04 (2·83 to 3·24)	2·25 (2·07 to 2·43)	1·61 (1·46 to 1·77)	1·16 (1·02 to 1·32)	0·75 (0·63 to 0·91)
Panama	1·48 (1·34 to 1·62)	1·13 (1·02 to 1·25)	0·94 (0·83 to 1·05)	0·79 (0·69 to 0·89)	0·75 (0·65 to 0·86)	0·75 (0·63 to 0·90)	0·70 (0·59 to 0·83)	0·63 (0·52 to 0·77)	0·61 (0·50 to 0·74)	0·51 (0·36 to 0·71)
Venezuela	12·37 (12·23 to 12·50)	11·83 (11·69 to 11·96)	8·94 (8·83 to 9·05)	7·26 (7·16 to 7·35)	7·14 (7·03 to 7·24)	6·59 (6·36 to 6·83)	5·27 (4·78 to 5·80)	4·70 (4·52 to 4·88)	4·41 (4·36 to 4·47)	4·14 (3·54 to 4·83)
Andean Latin America	77·15 (71·52 to 83·01)	67·04 (63·49 to 70·50)	61·43 (58·20 to 64·95)	53·37 (50·79 to 56·17)	43·14 (41·06 to 45·42)	33·05 (31·52 to 34·73)	23·82 (22·40 to 25·29)	18·38 (17·10 to 19·74)	14·94 (13·63 to 16·18)	12·15 (10·62 to 13·80)
Bolivia	20·20 (18·49 to 21·95)	18·62 (17·33 to 19·86)	16·96 (15·86 to 18·07)	15·19 (14·37 to 16·03)	13·00 (12·07 to 13·92)	10·91 (10·05 to 11·84)	8·83 (7·90 to 9·88)	7·06 (6·32 to 7·90)	5·55 (4·67 to 6·54)	4·26 (3·29 to 5·39)
Ecuador	12·75 (11·58 to 13·92)	11·74 (10·54 to 12·86)	10·25 (9·35 to 11·08)	8·63 (7·99 to 9·31)	7·13 (6·44 to 7·95)	6·00 (5·33 to 6·69)	4·92 (4·30 to 5·59)	4·05 (3·46 to 4·66)	3·37 (2·83 to 3·97)	2·78 (2·27 to 3·36)

Appendix Table 12C. Under-5 death numbers by location, females, 1970, 1975, 1980, 1985, 1990, 1995, 2000, 2005, 2010, 2016										
Location	1970	1975	1980	1985	1990	1995	2000	2005	2010	2016
Peru	44·20 (39·05 to 49·84)	36·68 (33·66 to 39·81)	34·22 (31·24 to 37·44)	29·55 (27·23 to 32·06)	23·01 (21·24 to 24·78)	16·14 (14·76 to 17·53)	10·07 (9·19 to 10·99)	7·27 (6·48 to 8·16)	6·02 (5·42 to 6·63)	5·11 (4·15 to 6·31)
Caribbean	45·04 (39·26 to 50·95)	39·41 (33·61 to 44·82)	35·34 (29·96 to 41·15)	31·62 (27·42 to 36·21)	28·04 (24·04 to 32·31)	23·80 (20·27 to 27·37)	19·70 (15·82 to 24·22)	18·51 (15·58 to 21·66)	29·90 (18·35 to 41·87)	12·17 (8·77 to 17·02)
Antigua and Barbuda	0·03 (0·02 to 0·03)	0·02 (0·01 to 0·03)	0·01 (0·01 to 0·02)	0·01 (0·01 to 0·01)	0·01 (<0·01 to 0·01)	0·01 (0·01 to 0·01)	0·01 (0·01 to 0·01)	0·01 (0·01 to 0·01)	0·01 (<0·01 to 0·01)	0·01 (<0·01 to 0·01)
The Bahamas	0·10 (0·07 to 0·15)	0·09 (0·06 to 0·12)	0·08 (0·05 to 0·12)	0·07 (0·04 to 0·11)	0·06 (0·03 to 0·10)	0·05 (0·03 to 0·07)	0·03 (0·02 to 0·05)	0·04 (0·03 to 0·05)	0·04 (0·02 to 0·06)	0·03 (0·02 to 0·08)
Barbados	0·11 (0·07 to 0·15)	0·07 (0·05 to 0·10)	0·05 (0·03 to 0·07)	0·04 (0·02 to 0·06)	0·03 (0·02 to 0·05)	0·03 (0·02 to 0·05)	0·03 (0·01 to 0·05)	0·03 (0·02 to 0·04)	0·02 (0·01 to 0·03)	0·02 (0·01 to 0·04)
Belize	0·24 (0·19 to 0·30)	0·20 (0·16 to 0·24)	0·17 (0·14 to 0·21)	0·15 (0·13 to 0·18)	0·13 (0·11 to 0·16)	0·08 (0·07 to 0·11)	0·09 (0·07 to 0·12)	0·08 (0·06 to 0·12)	0·07 (0·05 to 0·10)	0·07 (0·03 to 0·12)
Bermuda	0·01 (0·01 to 0·02)	0·01 (0·01 to 0·01)	0·01 (0·01 to 0·01)	0·01 (<0·01 to 0·01)	<0·01 (<0·01 to 0·01)	<0·01 (<0·01 to 0·01)	<0·01 (<0·01 to <0·01)	<0·01 (<0·01 to <0·01)	<0·01 (<0·01 to <0·01)	<0·01 (<0·01 to <0·01)
Cuba	4·67 (4·62 to 4·73)	2·58 (2·54 to 2·63)	1·43 (1·39 to 1·47)	1·37 (1·33 to 1·43)	1·08 (1·05 to 1·12)	0·70 (0·68 to 0·72)	0·54 (0·52 to 0·55)	0·37 (0·36 to 0·38)	0·33 (0·32 to 0·34)	0·25 (0·20 to 0·31)
Dominica	0·08 (0·05 to 0·11)	0·04 (0·02 to 0·05)	0·02 (0·01 to 0·03)	0·02 (0·01 to 0·02)	0·01 (0·01 to 0·02)	0·01 (0·01 to 0·02)	0·01 (0·01 to 0·02)	0·01 (0·01 to 0·02)	0·01 (0·01 to 0·02)	0·01 (0·01 to 0·02)
Dominican Republic	9·28 (7·94 to 10·63)	9·02 (7·85 to 10·22)	7·82 (6·90 to 8·80)	6·07 (5·30 to 6·90)	5·29 (4·39 to 6·20)	4·40 (3·55 to 5·28)	3·47 (2·94 to 4·06)	3·38 (2·70 to 4·18)	2·86 (2·22 to 3·65)	2·18 (1·55 to 2·93)
Grenada	0·07 (0·04 to 0·11)	0·06 (0·04 to 0·09)	0·04 (0·02 to 0·08)	0·04 (0·02 to 0·07)	0·02 (0·01 to 0·04)	0·02 (0·01 to 0·03)	0·01 (0·01 to 0·02)	0·01 (0·01 to 0·02)	0·01 (0·01 to 0·02)	0·01 (0·01 to 0·02)
Guyana	0·87 (0·76 to 0·99)	0·81 (0·72 to 0·90)	0·69 (0·61 to 0·78)	0·56 (0·47 to 0·65)	0·51 (0·40 to 0·62)	0·42 (0·34 to 0·51)	0·33 (0·27 to 0·40)	0·26 (0·22 to 0·31)	0·19 (0·15 to 0·24)	0·14 (0·11 to 0·19)
Haiti	23·81 (18·15 to 29·45)	22·00 (16·75 to 27·11)	21·26 (16·18 to 27·12)	20·12 (16·09 to 24·38)	18·08 (14·32 to 21·91)	15·63 (12·52 to 18·98)	13·11 (9·36 to 17·41)	12·52 (9·76 to 15·54)	24·74 (13·24 to 36·84)	8·16 (5·02 to 12·79)
Jamaica	1·86 (1·53 to 2·20)	1·43 (1·17 to 1·74)	1·23 (0·89 to 1·64)	1·07 (0·79 to 1·43)	0·98 (0·66 to 1·44)	0·84 (0·54 to 1·25)	0·72 (0·47 to 1·07)	0·59 (0·35 to 0·96)	0·57 (0·29 to 1·03)	0·51 (0·28 to 0·83)
Puerto Rico	0·94 (0·92 to 0·97)	0·75 (0·73 to 0·77)	0·63 (0·61 to 0·65)	0·48 (0·46 to 0·51)	0·43 (0·42 to 0·44)	0·37 (0·35 to 0·39)	0·28 (0·26 to 0·30)	0·21 (0·19 to 0·23)	0·17 (0·15 to 0·19)	0·12 (0·10 to 0·15)
Saint Lucia	0·13 (0·08 to 0·23)	0·09 (0·06 to 0·14)	0·06 (0·04 to 0·09)	0·05 (0·03 to 0·08)	0·04 (0·02 to 0·06)	0·03 (0·02 to 0·05)	0·02 (0·01 to 0·04)	0·02 (0·01 to 0·03)	0·02 (0·01 to 0·03)	0·01 (0·01 to 0·03)
Saint Vincent and the Grenadines	0·16 (0·10 to 0·22)	0·11 (0·07 to 0·16)	0·07 (0·05 to 0·11)	0·05 (0·03 to 0·07)	0·04 (0·02 to 0·05)	0·03 (0·02 to 0·04)	0·02 (0·01 to 0·03)	0·02 (0·01 to 0·03)	0·02 (0·01 to 0·02)	0·01 (0·01 to 0·02)
Suriname	0·40 (0·33 to 0·50)	0·32 (0·27 to 0·38)	0·28 (0·24 to 0·33)	0·27 (0·23 to 0·32)	0·26 (0·20 to 0·32)	0·25 (0·19 to 0·33)	0·24 (0·18 to 0·31)	0·21 (0·14 to 0·29)	0·18 (0·11 to 0·27)	0·15 (0·09 to 0·23)
Trinidad and Tobago	0·66 (0·57 to 0·74)	0·59 (0·50 to 0·68)	0·54 (0·45 to 0·64)	0·45 (0·37 to 0·55)	0·30 (0·24 to 0·37)	0·25 (0·18 to 0·32)	0·23 (0·17 to 0·30)	0·20 (0·14 to 0·27)	0·17 (0·09 to 0·29)	0·12 (0·05 to 0·27)
Virgin Islands, U.S.	0·03 (0·03 to 0·04)	0·03 (0·03 to 0·03)	0·03 (0·02 to 0·03)	0·02 (0·02 to 0·02)	0·02 (0·02 to 0·02)	0·01 (0·01 to 0·02)	0·01 (0·01 to 0·01)	0·01 (0·01 to 0·01)	0·01 (<0·01 to 0·01)	<0·01 (<0·01 to <0·01)
Tropical Latin America	167·34 (159·59 to 175·22)	157·08 (150·47 to 163·88)	138·36 (133·07 to 143·48)	114·70 (110·97 to 118·32)	88·57 (85·69 to 91·66)	67·27 (65·29 to 69·34)	50·23 (48·10 to 52·48)	39·01 (37·04 to 41·08)	31·04 (28·93 to 33·21)	23·67 (21·99 to 25·36)
Brazil	164·73 (157·03 to 172·63)	154·62 (148·02 to 161·42)	136·10 (130·91 to 141·25)	112·47 (108·75 to 116·14)	86·60 (83·68 to 89·68)	65·45 (63·50 to 67·52)	48·41 (46·35 to 50·63)	37·41 (35·48 to 39·46)	29·72 (27·65 to 31·86)	22·64 (20·96 to 24·31)
Paraguay	2·61 (2·31 to 2·94)	2·47 (2·21 to 2·73)	2·26 (1·96 to 2·55)	2·23 (2·01 to 2·48)	1·97 (1·78 to 2·17)	1·82 (1·56 to 2·09)	1·82 (1·58 to 2·09)	1·61 (1·34 to 1·90)	1·32 (1·01 to 1·73)	1·03 (0·77 to 1·36)
Southeast Asia, East Asia, and Oceania	2,232·15 (2,154·12 to 2,317·32)	1,701·57 (1,644·71 to 1,762·24)	1,327·66 (1,282·07 to 1,372·93)	1,135·46 (1,099·75 to 1,169·64)	933·94 (905·89 to 965·12)	713·37 (684·84 to 744·43)	522·07 (498·06 to 548·50)	354·90 (340·57 to 370·45)	263·96 (251·86 to 276·47)	176·11 (164·81 to 187·76)
East Asia	1,584·90 (1,509·67 to 1,666·01)	1,123·59 (1,073·74 to 1,175·33)	835·95 (798·32 to 876·18)	706·00 (673·30 to 736·94)	573·02 (549·57 to 599·60)	411·34 (387·98 to 438·36)	270·27 (250·42 to 292·56)	150·84 (144·18 to 157·73)	102·70 (97·34 to 108·24)	64·99 (59·25 to 71·56)
China	1,564·00 (1,488·73 to 1,643·28)	1,109·89 (1,059·34 to 1,161·22)	825·86 (788·97 to 866·33)	696·34 (665·20 to 726·76)	563·86 (540·03 to 591·11)	378·61 (364·34 to 393·69)	237·64 (228·81 to 247·32)	140·10 (134·42 to 145·55)	93·73 (89·77 to 98·03)	57·48 (52·36 to 63·00)
North Korea	16·05 (10·87 to 22·38)	10·47 (6·84 to 14·76)	7·31 (4·77 to 10·49)	7·99 (5·28 to 11·40)	7·92 (5·03 to 11·71)	31·45 (13·44 to 51·61)	31·53 (14·85 to 50·01)	10·09 (6·74 to 14·58)	8·53 (5·61 to 12·45)	7·11 (4·66 to 10·88)
Taiwan (Province of China)	4·85 (4·74 to 4·96)	3·22 (3·14 to 3·31)	2·77 (2·70 to 2·85)	1·67 (1·62 to 1·72)	1·24 (1·20 to 1·28)	1·27 (1·23 to 1·32)	1·10 (1·06 to 1·13)	0·65 (0·62 to 0·67)	0·44 (0·42 to 0·46)	0·40 (0·35 to 0·46)
Southeast Asia	639·36 (608·90 to 672·20)	569·99 (539·40 to 602·85)	484·15 (461·68 to 507·20)	421·91 (405·16 to 439·04)	353·29 (337·53 to 371·40)	294·41 (278·26 to 314·09)	244·23 (230·78 to 260·43)	196·53 (183·97 to 211·21)	154·33 (144·37 to 165·64)	105·18 (95·91 to 114·98)
Cambodia	23·62 (19·54 to 28·06)	41·99 (24·19 to 61·35)	18·48 (15·88 to 21·25)	16·60 (14·34 to 18·93)	15·15 (13·58 to 16·75)	15·50 (14·06 to 17·15)	13·73 (12·46 to 15·11)	10·56 (9·39 to 11·85)	7·71 (6·59 to 8·88)	5·33 (4·22 to 6·57)
Indonesia	333·01 (322·44 to 343·31)	284·56 (272·60 to 296·39)	251·66 (241·57 to 261·29)	214·16 (206·88 to 222·02)	170·32 (165·37 to 175·75)	138·43 (134·31 to 142·56)	113·53 (109·84 to 117·05)	88·96 (85·55 to 92·39)	70·81 (67·38 to 74·67)	45·75 (41·91 to 50·00)
Laos	16·80 (10·28 to 25·22)	16·47 (10·76 to 23·22)	14·31 (10·19 to 18·84)	13·17 (9·91 to 16·29)	13·45 (10·57 to 16·64)	13·42 (10·30 to 16·77)	11·73 (9·26 to 14·35)	9·64 (7·38 to 12·16)	8·00 (5·16 to 11·71)	6·47 (3·26 to 11·23)
Malaysia	8·59 (8·41 to 8·77)	7·05 (6·78 to 7·30)	5·55 (5·23 to 5·90)	4·69 (4·56 to 4·82)	3·71 (3·60 to 3·83)	3·10 (3·02 to 3·19)	2·19 (2·12 to 2·26)	1·72 (1·66 to 1·78)	1·82 (1·69 to 1·94)	1·35 (1·18 to 1·54)
Maldives	0·56 (0·49 to 0·63)	0·47 (0·41 to 0·54)	0·43 (0·38 to 0·49)	0·35 (0·30 to 0·40)	0·23 (0·20 to 0·26)	0·13 (0·11 to 0·15)	0·07 (0·06 to 0·08)	0·05 (0·04 to 0·05)	0·03 (0·03 to 0·04)	0·02 (0·02 to 0·02)
Mauritius	0·88 (0·85 to 0·90)	0·63 (0·61 to 0·65)	0·42 (0·41 to 0·43)	0·23 (0·22 to 0·24)	0·21 (0·20 to 0·22)	0·19 (0·18 to 0·19)	0·15 (0·15 to 0·16)	0·12 (0·12 to 0·13)	0·09 (0·09 to 0·10)	0·08 (0·07 to 0·10)
Myanmar	68·12 (47·14 to 92·95)	60·76 (43·51 to 81·14)	55·77 (40·02 to 72·79)	52·89 (40·18 to 67·00)	49·91 (36·66 to 65·56)	45·49 (32·13 to 63·94)	39·92 (27·53 to 55·42)	31·45 (20·76 to 45·28)	19·84 (12·88 to 28·76)	10·98 (6·51 to 18·44)
Philippines	56·23 (52·71 to 59·93)	55·93 (51·72 to 60·35)	56·49 (52·07 to 61·00)	53·15 (49·16 to 57·13)	46·31 (42·81 to 50·17)	39·68 (36·89 to 42·65)	35·60 (32·72 to 38·70)	32·91 (29·73 to 36·40)	29·42 (25·50 to 33·80)	24·35 (20·18 to 29·14)
Sri Lanka	11·83 (11·56 to 12·11)	11·92 (11·57 to 12·29)	8·35 (8·14 to 8·56)	5·67 (5·50 to 5·83)	5·40 (5·15 to 5·67)	3·33 (3·24 to 3·43)	2·61 (2·51 to 2·72)	2·88 (2·79 to 2·97)	2·24 (1·99 to 2·47)	0·86 (0·57 to 1·26)
Seychelles	0·06 (0·06 to 0·07)	0·04 (0·04 to 0·04)	0·03 (0·02 to 0·03)	0·02 (0·02 to 0·02)	0·01 (0·01 to 0·01)	0·01 (0·01 to 0·01)	0·01 (0·01 to 0·01)	0·01 (0·01 to 0·01)	0·01 (0·01 to 0·01)	0·01 (0·01 to 0·01)
Thailand	55·86 (49·12 to 63·03)	43·89 (38·09 to 50·05)	31·53 (25·13 to 37·96)	21·08 (17·19 to 25·39)	14·84 (11·79 to 18·26)	10·54 (7·96 to 13·45)	7·22 (5·37 to 9·44)	4·54 (3·51 to 5·92)	2·85 (2·29 to 3·53)	1·62 (1·19 to 2·14)
Timor-Leste	3·18 (2·24 to 4·28)	3·23 (2·40 to 4·08)	2·85 (2·17 to 3·55)	2·14 (1·65 to 2·71)	1·81 (1·42 to 2·23)	1·48 (1·15 to 1·82)	0·94 (0·70 to 1·22)	0·78 (0·55 to 1·04)	0·76 (0·50 to 1·10)	0·52 (0·29 to 0·84)
Vietnam	59·63 (45·62 to 74·58)	42·39 (36·15 to 49·50)	37·72 (32·31 to 43·55)	37·27 (31·44 to 43·66)	31·48 (25·91 to 37·68)	22·76 (19·02 to 26·93)	16·22 (14·28 to 18·30)	12·64 (10·61 to 15·00)	10·57 (8·39 to 13·27)	7·72 (5·87 to 10·19)
Oceania	7·88 (6·52 to 9·36)	7·99 (6·67 to 9·42)	7·57 (6·40 to 8·86)	7·55 (6·34 to 8·86)	7·62 (6·26 to 9·10)	7·62 (6·06 to 9·48)	7·57 (5·56 to 9·97)	7·53 (4·97 to 10·76)	6·92 (4·32 to 10·47)	5·95 (3·67 to 9·40)
American Samoa	0·02 (0·01 to 0·02)	0·01 (0·01 to 0·01)	0·01 (0·01 to 0·01)	0·01 (0·01 to 0·01)	0·01 (0·01 to 0·01)	0·01 (0·01 to 0·01)	0·01 (0·01 to 0·01)	0·01 (0·01 to 0·01)	0·01 (0·01 to 0·01)	0·01 (<0·01 to 0·01)
Federated States of Micronesia	0·11 (0·08 to 0·15)	0·09 (0·07 to 0·12)	0·09 (0·07 to 0·13)	0·09 (0·07 to 0·11)	0·07 (0·05 to 0·10)	0·05 (0·04 to 0·07)	0·04 (0·03 to 0·06)	0·02 (0·01 to 0·03)	0·02 (0·01 to 0·03)	0·01 (0·01 to 0·02)
Fiji	0·42 (0·37 to 0·47)	0·45 (0·36 to 0·55)	0·36 (0·27 to 0·46)	0·28 (0·20 to 0·37)	0·21 (0·14 to 0·31)	0·20 (0·14 to 0·27)	0·19 (0·15 to 0·24)	0·20 (0·15 to 0·24)	0·19 (0·14 to 0·26)	0·17 (0·10 to 0·27)
Guam	0·03 (0·03 to 0·04)	0·03 (0·03 to 0·03)	0·02 (0·02 to 0·02)	0·02 (0·02 to 0·02)	0·02 (0·02 to 0·02)	0·02 (0·02 to 0·02)	0·02 (0·02 to 0·02)	0·02 (0·02 to 0·02)	0·02 (0·02 to 0·02)	0·02 (0·02 to 0·03)
Kiribati	0·14 (0·10 to 0·17)	0·12 (0·08 to 0·18)	0·12 (0·07 to 0·20)	0·12 (0·07 to 0·19)	0·12 (0·08 to 0·15)	0·10 (0·08 to 0·13)	0·09 (0·07 to 0·11)	0·08 (0·06 to 0·11)	0·08 (0·05 to 0·12)	0·06 (0·03 to 0·11)
Marshall Islands	0·03 (0·02 to 0·05)	0·03 (0·02 to 0·05)	0·03 (0·02 to 0·04)	0·03 (0·02 to 0·04)	0·03 (0·02 to 0·04)	0·03 (0·02 to 0·04)	0·03 (0·02 to 0·05)	0·03 (0·02 to 0·04)	0·02 (0·01 to 0·04)	0·02 (0·01 to 0·03)
Northern Mariana Islands	<0·01 (<0·01 to 0·01)	<0·01 (<0·01 to 0·01)	<0·01 (<0·01 to <0·01)	<0·01 (<0·01 to 0·01)	<0·01 (<0·01 to 0·01)	0·01 (<0·01 to 0·01)	0·01 (<0·01 to 0·01)	<0·01 (<0·01 to 0·01)	<0·01 (<0·01 to <0·01)	<0·01 (<0·01 to 0·01)
Papua New Guinea	5·82 (4·56 to 7·17)	6·06 (4·86 to 7·40)	5·94 (4·89 to 7·09)	6·03 (4·93 to 7·27)	6·25 (5·01 to 7·61)	6·38 (4·91 to 8·16)	6·41 (4·55 to 8·71)	6·43 (3·98 to 9·53)	5·97 (3·44 to 9·37)	5·09 (2·91 to 8·44)
Samoa	0·14 (0·11 to 0·18)	0·11 (0·08 to 0·16)	0·08 (0·05 to 0·13)	0·07 (0·04 to 0·12)	0·05 (0·03 to 0·10)	0·04 (0·02 to 0·08)	0·04 (0·02 to 0·07)	0·03 (0·02 to 0·06)	0·03 (0·02 to 0·06)	0·02 (0·01 to 0·04)
Solomon Islands	0·33 (0·25 to 0·43)	0·31 (0·21 to 0·45)	0·24 (0·16 to 0·37)	0·22 (0·15 to 0·32)	0·22 (0·15 to 0·30)	0·22 (0·16 to 0·30)	0·23 (0·16 to 0·32)	0·25 (0·17 to 0·35)	0·23 (0·15 to 0·35)	0·19 (0·12 to 0·29)

Appendix Table 12C. Under-5 death numbers by location, females, 1970, 1975, 1980, 1985, 1990, 1995, 2000, 2005, 2010, 2016

Location	1970	1975	1980	1985	1990	1995	2000	2005	2010	2016
Tonga	0·05 (0·03 to 0·07)	0·04 (0·03 to 0·06)	0·04 (0·03 to 0·06)	0·04 (0·02 to 0·05)	0·03 (0·02 to 0·05)	0·03 (0·02 to 0·05)	0·03 (0·02 to 0·05)	0·03 (0·02 to 0·05)	0·03 (0·01 to 0·04)	0·02 (0·01 to 0·03)
Vanuatu	0·16 (0·11 to 0·23)	0·15 (0·10 to 0·21)	0·13 (0·09 to 0·19)	0·12 (0·08 to 0·16)	0·11 (0·09 to 0·14)	0·11 (0·08 to 0·14)	0·12 (0·09 to 0·15)	0·13 (0·10 to 0·17)	0·13 (0·09 to 0·17)	0·12 (0·08 to 0·17)
North Africa and Middle East	770·82 (717·22 to 828·90)	673·39 (626·01 to 715·56)	585·79 (546·17 to 628·16)	501·56 (466·45 to 535·96)	378·70 (355·25 to 402·49)	313·65 (290·67 to 340·32)	262·40 (237·59 to 285·77)	226·69 (204·48 to 251·19)	200·48 (178·26 to 223·61)	163·09 (137·46 to 190·44)
North Africa and Middle East	770·82 (717·22 to 828·90)	673·39 (626·01 to 715·56)	585·79 (546·17 to 628·16)	501·56 (466·45 to 535·96)	378·70 (355·25 to 402·49)	313·65 (290·67 to 340·32)	262·40 (237·59 to 285·77)	226·69 (204·48 to 251·19)	200·48 (178·26 to 223·61)	163·09 (137·46 to 190·44)
Afghanistan	66·02 (46·55 to 84·87)	69·76 (51·06 to 89·24)	71·40 (54·11 to 89·25)	57·92 (43·96 to 72·28)	39·99 (30·60 to 48·97)	46·80 (34·70 to 57·45)	48·73 (37·04 to 58·79)	47·27 (37·93 to 56·55)	38·70 (30·18 to 47·50)	35·47 (25·07 to 46·33)
Algeria	48·69 (40·44 to 56·76)	42·96 (35·17 to 50·50)	34·77 (28·68 to 40·82)	25·16 (20·15 to 30·12)	18·81 (15·11 to 22·90)	16·91 (12·87 to 21·73)	15·32 (11·67 to 19·40)	13·18 (10·40 to 16·20)	10·74 (7·75 to 14·18)	7·15 (4·67 to 10·62)
Bahrain	0·32 (0·24 to 0·41)	0·22 (0·17 to 0·27)	0·15 (0·13 to 0·16)	0·13 (0·12 to 0·14)	0·15 (0·14 to 0·16)	0·11 (0·10 to 0·12)	0·08 (0·07 to 0·09)	0·07 (0·07 to 0·08)	0·07 (0·06 to 0·08)	0·06 (0·05 to 0·08)
Egypt	177·80 (158·93 to 197·23)	159·88 (143·35 to 177·45)	129·20 (113·13 to 145·42)	96·74 (86·34 to 107·82)	71·05 (63·76 to 78·91)	49·56 (44·21 to 55·60)	35·83 (31·03 to 41·29)	30·97 (26·18 to 36·17)	27·79 (23·05 to 33·37)	18·79 (13·21 to 26·87)
Iran	149·71 (114·05 to 187·66)	97·15 (74·96 to 122·57)	85·19 (64·55 to 107·49)	76·39 (60·33 to 93·30)	61·09 (49·48 to 73·98)	33·50 (24·94 to 43·75)	23·36 (15·38 to 33·60)	19·53 (9·47 to 34·53)	16·32 (6·70 to 30·77)	12·07 (4·89 to 25·82)
Iraq	24·09 (13·15 to 31·63)	22·62 (12·04 to 29·22)	20·40 (10·33 to 27·07)	20·19 (10·55 to 27·25)	15·08 (6·41 to 21·75)	16·63 (7·04 to 25·33)	15·69 (8·37 to 22·86)	17·92 (12·14 to 24·17)	20·66 (11·86 to 28·81)	21·27 (10·63 to 31·96)
Jordan	4·59 (4·15 to 5·07)	4·02 (3·69 to 4·37)	2·63 (2·42 to 2·84)	2·06 (1·81 to 2·31)	1·79 (1·58 to 2·01)	2·03 (1·74 to 2·34)	1·84 (1·60 to 2·08)	1·72 (1·52 to 1·94)	1·69 (1·45 to 1·95)	1·61 (1·28 to 2·05)
Kuwait	0·83 (0·79 to 0·87)	0·96 (0·92 to 1·00)	0·76 (0·72 to 0·80)	0·53 (0·50 to 0·56)	0·32 (0·27 to 0·37)	0·26 (0·24 to 0·28)	0·25 (0·23 to 0·26)	0·24 (0·22 to 0·26)	0·28 (0·26 to 0·30)	0·24 (0·17 to 0·32)
Lebanon	3·12 (2·48 to 3·93)	3·39 (2·53 to 4·28)	3·49 (2·22 to 4·88)	1·83 (1·35 to 2·36)	1·22 (1·00 to 1·48)	0·82 (0·62 to 1·05)	0·52 (0·38 to 0·70)	0·37 (0·24 to 0·53)	0·31 (0·20 to 0·44)	0·33 (0·23 to 0·45)
Libya	4·20 (3·45 to 4·96)	3·37 (2·87 to 3·89)	2·63 (2·20 to 3·13)	2·23 (1·74 to 2·75)	2·03 (1·58 to 2·51)	1·79 (1·43 to 2·18)	1·62 (1·22 to 2·05)	1·31 (0·93 to 1·74)	0·88 (0·60 to 1·20)	0·53 (0·36 to 0·74)
Morocco	45·82 (42·05 to 49·70)	40·93 (37·99 to 44·02)	35·48 (32·60 to 38·39)	29·71 (27·57 to 31·60)	23·96 (22·07 to 25·87)	17·99 (16·08 to 20·08)	12·96 (11·28 to 14·92)	9·37 (7·94 to 11·04)	6·70 (5·27 to 8·46)	4·26 (3·28 to 5·45)
Palestine	2·34 (1·38 to 3·28)	2·23 (1·16 to 3·12)	1·97 (0·84 to 2·80)	1·56 (0·64 to 2·30)	1·67 (0·72 to 2·45)	1·43 (0·71 to 2·07)	1·45 (0·70 to 2·12)	1·43 (0·78 to 2·14)	1·78 (1·09 to 2·47)	1·71 (0·83 to 2·66)
Oman	5·13 (4·47 to 5·82)	4·46 (3·77 to 5·13)	3·57 (2·90 to 4·26)	2·53 (2·06 to 3·05)	1·67 (1·35 to 2·04)	1·05 (0·84 to 1·32)	0·65 (0·54 to 0·79)	0·32 (0·28 to 0·37)	0·34 (0·29 to 0·39)	0·33 (0·24 to 0·44)
Qatar	0·14 (0·09 to 0·19)	0·15 (0·11 to 0·19)	0·12 (0·08 to 0·16)	0·12 (0·09 to 0·15)	0·11 (0·08 to 0·14)	0·09 (0·07 to 0·13)	0·08 (0·06 to 0·11)	0·08 (0·06 to 0·11)	0·10 (0·07 to 0·15)	0·11 (0·07 to 0·16)
Saudi Arabia	18·74 (16·39 to 21·31)	16·83 (15·28 to 18·55)	15·14 (13·38 to 17·02)	14·96 (12·98 to 17·27)	12·99 (11·09 to 15·24)	9·44 (7·85 to 11·50)	7·02 (5·78 to 8·71)	4·78 (3·98 to 5·79)	2·97 (2·45 to 3·62)	1·36 (1·08 to 1·71)
Sudan	34·92 (28·51 to 41·67)	37·57 (29·68 to 45·68)	39·32 (31·12 to 47·38)	59·02 (43·72 to 74·70)	36·86 (31·60 to 42·97)	38·47 (31·37 to 45·79)	37·26 (30·09 to 45·34)	32·20 (23·78 to 40·61)	30·77 (22·17 to 40·40)	24·06 (13·50 to 40·49)
Syria	13·51 (12·01 to 14·97)	12·37 (10·85 to 13·93)	10·59 (9·04 to 12·21)	9·42 (7·75 to 11·34)	8·39 (6·78 to 10·07)	6·04 (5·05 to 6·99)	3·94 (3·35 to 4·58)	3·28 (2·74 to 3·86)	3·36 (2·74 to 4·07)	4·67 (2·12 to 7·22)
Tunisia	13·27 (12·20 to 14·44)	10·67 (9·97 to 11·40)	8·63 (7·98 to 9·34)	6·59 (5·83 to 7·40)	4·74 (4·27 to 5·25)	3·07 (2·70 to 3·47)	1·93 (1·68 to 2·22)	1·50 (1·30 to 1·72)	1·32 (1·09 to 1·57)	0·82 (0·62 to 1·07)
Turkey	111·94 (95·75 to 128·53)	104·96 (87·62 to 123·52)	86·10 (69·71 to 105·44)	62·15 (49·09 to 75·80)	43·71 (35·41 to 52·85)	33·71 (27·87 to 40·26)	23·67 (19·62 to 28·30)	16·21 (13·28 to 19·78)	12·38 (9·21 to 16·40)	8·26 (5·79 to 11·51)
United Arab Emirates	0·56 (0·39 to 0·78)	0·69 (0·45 to 1·03)	0·88 (0·56 to 1·29)	0·77 (0·43 to 1·26)	0·56 (0·29 to 0·92)	0·45 (0·23 to 0·81)	0·35 (0·18 to 0·59)	0·28 (0·22 to 0·35)	0·47 (0·27 to 0·76)	0·39 (0·21 to 0·70)
Yemen	44·74 (21·88 to 57·25)	37·98 (18·58 to 48·84)	33·05 (17·18 to 41·77)	31·25 (17·48 to 38·87)	32·29 (22·70 to 38·73)	33·29 (20·28 to 43·05)	29·68 (15·75 to 40·43)	24·45 (13·48 to 34·48)	22·68 (12·59 to 32·24)	19·47 (9·09 to 30·86)
South Asia	2,489·71 (2,413·70 to 2,569·59)	2,365·89 (2,295·21 to 2,440·33)	2,322·16 (2,239·44 to 2,399·01)	2,168·65 (2,092·00 to 2,247·14)	1,920·29 (1,853·94 to 1,984·41)	1,668·45 (1,613·22 to 1,720·03)	1,425·25 (1,389·01 to 1,461·37)	1,220·03 (1,187·49 to 1,252·77)	968·38 (930·31 to 1,007·83)	603·96 (565·22 to 643·79)
South Asia	2,489·71 (2,413·70 to 2,569·59)	2,365·89 (2,295·21 to 2,440·33)	2,322·16 (2,239·44 to 2,399·01)	2,168·65 (2,092·00 to 2,247·14)	1,920·29 (1,853·94 to 1,984·41)	1,668·45 (1,613·22 to 1,720·03)	1,425·25 (1,389·01 to 1,461·37)	1,220·03 (1,187·49 to 1,252·77)	968·38 (930·31 to 1,007·83)	603·96 (565·22 to 643·79)
Bangladesh	288·89 (263·50 to 314·89)	263·12 (242·10 to 284·45)	260·57 (242·75 to 280·21)	252·47 (234·45 to 269·13)	221·27 (207·73 to 234·39)	173·30 (161·75 to 185·73)	132·76 (122·73 to 142·34)	104·85 (96·60 to 113·61)	75·97 (68·19 to 84·15)	44·64 (38·43 to 52·45)
Bhutan	1·71 (1·48 to 1·95)	1·73 (1·51 to 1·98)	1·69 (1·45 to 1·94)	1·51 (1·28 to 1·75)	1·33 (1·16 to 1·50)	1·01 (0·88 to 1·14)	0·83 (0·71 to 0·97)	0·64 (0·54 to 0·74)	0·43 (0·36 to 0·51)	0·24 (0·19 to 0·30)
India	1,919·21 (1,851·93 to 1,988·44)	1,816·93 (1,752·03 to 1,883·68)	1,763·00 (1,689·61 to 1,836·95)	1,606·78 (1,535·25 to 1,678·09)	1,415·19 (1,360·00 to 1,472·23)	1,249·80 (1,205·26 to 1,295·49)	1,085·99 (1,056·67 to 1,117·60)	926·87 (900·22 to 953·40)	712·82 (684·67 to 742·17)	427·56 (401·81 to 455·07)
Nepal	58·33 (43·80 to 71·67)	59·04 (45·20 to 73·54)	58·32 (43·90 to 71·39)	54·49 (41·93 to 66·11)	47·95 (37·64 to 58·00)	38·37 (31·38 to 45·28)	30·34 (24·34 to 36·71)	21·81 (17·25 to 26·58)	16·39 (13·27 to 19·79)	11·23 (8·36 to 14·58)
Pakistan	221·57 (198·37 to 243·00)	225·07 (202·71 to 246·82)	238·58 (220·98 to 256·48)	253·41 (235·28 to 272·74)	234·55 (209·74 to 258·84)	205·96 (183·54 to 228·69)	175·33 (160·36 to 192·27)	165·86 (149·60 to 183·36)	162·76 (139·86 to 188·53)	120·28 (95·10 to 149·33)
Sub-Saharan Africa	1,351·46 (1,267·34 to 1,429·31)	1,388·23 (1,305·22 to 1,465·27)	1,419·38 (1,344·59 to 1,485·68)	1,472·79 (1,403·93 to 1,538·28)	1,544·28 (1,477·05 to 1,603·71)	1,607·02 (1,521·38 to 1,689·16)	1,623·80 (1,536·26 to 1,704·87)	1,561·82 (1,483·43 to 1,640·87)	1,451·55 (1,364·85 to 1,540·94)	1,207·09 (1,118·95 to 1,310·64)
Southern Sub-Saharan Africa	52·88 (47·17 to 59·52)	52·75 (47·47 to 58·44)	53·30 (48·22 to 58·80)	51·43 (46·59 to 57·03)	47·50 (44·05 to 50·99)	46·70 (44·22 to 49·16)	53·45 (49·87 to 57·08)	58·84 (53·65 to 64·42)	52·25 (46·51 to 58·55)	38·88 (33·31 to 45·79)
Botswana	1·19 (0·85 to 1·54)	1·07 (0·73 to 1·42)	1·03 (0·73 to 1·34)	0·97 (0·66 to 1·28)	0·87 (0·62 to 1·17)	1·10 (0·72 to 1·52)	1·30 (0·78 to 1·95)	1·00 (0·64 to 1·44)	0·60 (0·41 to 0·85)	0·34 (0·20 to 0·56)
Lesotho	2·09 (1·74 to 2·42)	2·12 (1·75 to 2·54)	2·08 (1·65 to 2·57)	2·01 (1·57 to 2·47)	1·81 (1·45 to 2·18)	1·88 (1·52 to 2·25)	2·29 (1·83 to 2·74)	2·56 (2·16 to 3·00)	2·13 (1·73 to 2·59)	1·60 (1·20 to 2·15)
Namibia	1·57 (1·21 to 2·01)	1·62 (1·32 to 1·96)	1·68 (1·42 to 1·97)	1·52 (1·29 to 1·79)	1·67 (1·44 to 1·93)	1·55 (1·33 to 1·79)	1·73 (1·46 to 2·09)	1·72 (1·45 to 2·04)	1·52 (1·18 to 1·90)	1·12 (0·70 to 1·63)
South Africa	37·15 (31·60 to 43·75)	37·06 (31·93 to 42·75)	37·41 (32·61 to 42·86)	36·24 (31·58 to 41·75)	32·15 (28·88 to 35·53)	29·44 (27·25 to 31·61)	33·51 (30·25 to 36·90)	36·40 (31·69 to 41·81)	29·39 (24·17 to 35·80)	21·11 (16·19 to 27·52)
Swaziland	1·55 (1·10 to 2·12)	1·35 (1·01 to 1·78)	1·17 (0·93 to 1·47)	1·00 (0·83 to 1·18)	0·87 (0·73 to 1·01)	0·94 (0·81 to 1·06)	1·37 (1·17 to 1·56)	1·66 (1·43 to 1·92)	1·40 (1·21 to 1·60)	0·95 (0·72 to 1·21)
Zimbabwe	9·34 (8·34 to 10·41)	9·52 (8·48 to 10·59)	9·93 (8·93 to 10·96)	9·70 (8·71 to 10·69)	10·14 (9·21 to 11·14)	11·79 (10·68 to 12·94)	13·27 (12·07 to 14·47)	15·50 (14·05 to 17·11)	17·20 (15·14 to 19·27)	13·76 (11·78 to 16·15)
Western Sub-Saharan Africa	660·08 (597·66 to 725·61)	655·56 (596·99 to 712·60)	652·95 (603·58 to 701·87)	666·76 (619·00 to 713·24)	705·83 (668·61 to 744·59)	749·28 (699·36 to 800·57)	775·12 (721·46 to 830·14)	776·56 (719·81 to 838·77)	744·75 (680·86 to 813·23)	640·52 (574·72 to 708·23)
Benin	17·38 (15·75 to 19·04)	16·58 (15·05 to 18·07)	16·20 (15·01 to 17·34)	16·35 (15·46 to 17·27)	16·79 (15·40 to 18·22)	17·43 (15·58 to 19·37)	17·26 (15·31 to 19·40)	16·81 (15·20 to 18·80)	15·85 (13·77 to 18·19)	14·05 (11·47 to 17·09)
Burkina Faso	39·52 (28·37 to 47·97)	39·12 (28·46 to 47·83)	37·85 (28·24 to 45·50)	38·15 (29·03 to 45·55)	39·21 (32·45 to 44·70)	40·49 (34·36 to 46·10)	41·09 (33·00 to 47·96)	41·35 (31·51 to 50·01)	40·16 (30·99 to 49·03)	34·92 (24·71 to 44·78)
Cameroon	29·09 (27·04 to 31·15)	28·23 (26·04 to 30·44)	27·91 (25·85 to 29·99)	27·73 (25·79 to 29·80)	29·46 (27·24 to 31·64)	33·71 (31·01 to 36·61)	36·47 (32·64 to 40·82)	38·03 (33·42 to 42·80)	38·41 (32·91 to 44·50)	35·00 (28·25 to 43·61)
Cape Verde	0·76 (0·71 to 0·81)	0·56 (0·52 to 0·60)	0·41 (0·39 to 0·43)	0·49 (0·45 to 0·52)	0·36 (0·33 to 0·40)	0·43 (0·34 to 0·53)	0·27 (0·23 to 0·31)	0·21 (0·18 to 0·25)	0·15 (0·13 to 0·17)	0·11 (0·09 to 0·13)
Chad	20·25 (18·64 to 21·91)	21·12 (19·68 to 22·56)	22·36 (20·93 to 23·80)	23·48 (22·13 to 24·86)	24·14 (22·89 to 25·36)	26·08 (24·62 to 27·53)	29·83 (28·12 to 31·56)	31·95 (29·79 to 34·10)	32·67 (29·77 to 35·96)	32·44 (28·74 to 36·94)
Cote d'Ivoire	22·12 (18·94 to 25·06)	22·38 (19·59 to 24·93)	23·20 (21·04 to 25·64)	27·35 (24·61 to 29·94)	30·36 (26·38 to 34·07)	32·67 (28·46 to 36·83)	34·15 (29·97 to 38·50)	34·32 (28·00 to 40·82)	32·49 (26·17 to 39·13)	29·34 (22·22 to 36·92)
The Gambia	1·99 (1·71 to 2·30)	1·97 (1·71 to 2·25)	1·98 (1·70 to 2·27)	1·95 (1·70 to 2·21)	2·14 (1·85 to 2·40)	2·23 (1·91 to 2·58)	2·24 (1·95 to 2·55)	2·12 (1·83 to 2·43)	1·94 (1·65 to 2·27)	1·75 (1·40 to 2·15)
Ghana	29·53 (25·59 to 33·16)	28·91 (24·72 to 32·88)	29·00 (25·10 to 32·78)	31·36 (27·30 to 35·34)	30·56 (27·50 to 33·58)	28·38 (24·89 to 31·86)	26·97 (23·18 to 31·08)	27·85 (24·34 to 32·06)	27·55 (24·10 to 31·43)	20·63 (16·21 to 25·43)
Guinea	26·89 (24·21 to 29·66)	26·83 (24·97 to 28·80)	25·08 (23·74 to 26·43)	24·72 (23·46 to 25·99)	24·92 (23·47 to 26·34)	27·62 (25·94 to 29·26)	26·75 (25·18 to 28·32)	25·22 (23·30 to 27·11)	23·52 (21·15 to 26·36)	20·54 (17·48 to 24·45)
Guinea-Bissau	3·98 (3·50 to 4·51)	3·93 (3·49 to 4·42)	3·93 (3·52 to 4·36)	3·96 (3·63 to 4·29)	3·79 (3·56 to 4·05)	3·62 (3·37 to 3·86)	3·45 (3·20 to 3·71)	3·39 (3·06 to 3·73)	2·77 (2·47 to 3·09)	2·15 (1·85 to 2·48)

Appendix Table 12C. Under-5 death numbers by location, females, 1970, 1975, 1980, 1985, 1990, 1995, 2000, 2005, 2010, 2016										
Location	1970	1975	1980	1985	1990	1995	2000	2005	2010	2016
Liberia	9·18 (8·28 to 10·13)	9·26 (8·38 to 10·12)	9·58 (8·79 to 10·38)	10·22 (9·43 to 11·01)	9·66 (8·96 to 10·37)	8·99 (8·22 to 9·75)	9·21 (8·45 to 10·03)	7·27 (6·66 to 7·87)	6·12 (5·51 to 6·76)	4·64 (3·95 to 5·39)
Mali	48·25 (45·23 to 51·12)	47·80 (45·27 to 50·26)	45·53 (43·17 to 47·94)	44·93 (42·68 to 47·18)	42·53 (40·61 to 44·70)	43·11 (40·70 to 45·86)	44·13 (41·60 to 47·01)	43·15 (40·36 to 46·14)	40·86 (37·18 to 44·83)	38·07 (32·37 to 45·06)
Mauritania	3·34 (3·17 to 3·53)	3·52 (3·36 to 3·69)	3·48 (3·28 to 3·68)	3·43 (3·18 to 3·69)	3·37 (3·12 to 3·62)	3·38 (3·17 to 3·60)	3·47 (3·23 to 3·71)	3·54 (3·22 to 3·86)	3·05 (2·71 to 3·42)	2·42 (2·11 to 2·79)
Niger	37·06 (34·14 to 40·02)	41·77 (38·70 to 44·72)	45·49 (42·21 to 48·80)	49·96 (46·67 to 53·39)	53·51 (50·51 to 56·80)	56·44 (52·99 to 59·78)	55·20 (51·41 to 59·00)	50·44 (46·36 to 54·69)	46·06 (40·92 to 52·09)	43·28 (36·68 to 51·51)
Nigeria	321·66 (260·40 to 384·16)	314·35 (256·54 to 371·36)	313·39 (263·23 to 362·79)	315·93 (271·94 to 362·19)	348·25 (312·36 to 384·57)	378·65 (329·42 to 427·20)	401·35 (348·63 to 456·25)	407·86 (352·02 to 469·61)	394·01 (330·90 to 461·25)	329·06 (266·96 to 392·27)
Sao Tome and Principe	0·21 (0·20 to 0·22)	0·18 (0·16 to 0·20)	0·19 (0·18 to 0·20)	0·23 (0·22 to 0·25)	0·21 (0·20 to 0·23)	0·20 (0·18 to 0·22)	0·18 (0·16 to 0·19)	0·15 (0·13 to 0·17)	0·11 (0·10 to 0·13)	0·09 (0·08 to 0·10)
Senegal	23·40 (21·24 to 25·75)	23·52 (21·59 to 25·53)	21·46 (19·72 to 23·32)	19·72 (18·00 to 21·42)	19·04 (17·57 to 20·62)	19·63 (18·41 to 20·91)	17·99 (16·81 to 19·24)	16·89 (15·43 to 18·53)	14·60 (13·04 to 16·09)	11·73 (10·04 to 13·65)
Sierra Leone	16·47 (14·48 to 18·75)	16·52 (14·75 to 18·41)	17·08 (15·64 to 18·58)	17·41 (16·38 to 18·51)	17·65 (16·75 to 18·65)	16·35 (15·50 to 17·20)	15·32 (14·49 to 16·18)	16·14 (14·96 to 17·29)	15·37 (13·99 to 16·78)	12·87 (11·30 to 14·62)
Togo	8·99 (7·58 to 10·41)	9·01 (7·55 to 10·40)	8·79 (7·19 to 10·42)	9·38 (7·94 to 10·83)	9·87 (8·69 to 11·12)	9·87 (8·53 to 11·31)	9·80 (8·22 to 11·44)	9·86 (7·55 to 12·32)	9·05 (7·09 to 11·01)	7·42 (5·85 to 9·06)
Eastern Sub-Saharan Africa	494·08 (476·86 to 510·69)	531·66 (512·96 to 549·67)	560·89 (543·30 to 578·52)	602·61 (586·32 to 619·39)	619·39 (602·37 to 636·08)	621·07 (600·98 to 641·70)	592·86 (575·60 to 612·03)	528·76 (510·93 to 546·71)	463·81 (445·93 to 481·51)	366·91 (346·46 to 388·75)
Burundi	19·32 (16·46 to 22·11)	19·65 (17·40 to 22·17)	20·24 (17·85 to 22·79)	19·85 (17·84 to 22·02)	20·85 (18·63 to 23·24)	21·10 (18·87 to 23·63)	20·06 (17·60 to 22·42)	18·24 (15·96 to 20·71)	17·64 (14·73 to 20·83)	17·81 (12·74 to 25·24)
Comoros	0·80 (0·70 to 0·91)	0·82 (0·72 to 0·92)	0·89 (0·78 to 1·00)	0·86 (0·75 to 0·97)	0·81 (0·73 to 0·89)	0·77 (0·69 to 0·86)	0·81 (0·70 to 0·93)	0·75 (0·62 to 0·89)	0·66 (0·56 to 0·76)	0·47 (0·38 to 0·58)
Djibouti	0·49 (0·41 to 0·58)	0·62 (0·53 to 0·71)	0·89 (0·77 to 1·02)	1·09 (0·95 to 1·23)	1·39 (1·19 to 1·59)	1·54 (1·29 to 1·79)	1·52 (1·27 to 1·77)	1·33 (1·06 to 1·61)	1·12 (0·83 to 1·47)	0·78 (0·52 to 1·14)
Eritrea	8·30 (7·13 to 9·64)	8·49 (7·37 to 9·57)	8·77 (7·82 to 9·72)	8·96 (7·91 to 10·10)	8·61 (7·61 to 9·65)	6·18 (5·36 to 7·04)	5·19 (4·44 to 6·02)	5·22 (4·22 to 6·29)	5·02 (3·96 to 6·29)	3·65 (2·72 to 4·82)
Ethiopia	122·01 (112·52 to 131·68)	138·59 (127·10 to 150·22)	138·75 (127·69 to 149·67)	144·13 (134·73 to 152·75)	158·26 (148·28 to 168·57)	159·37 (145·97 to 173·52)	151·85 (139·56 to 166·85)	126·75 (115·55 to 139·29)	96·87 (87·31 to 107·43)	63·73 (56·28 to 72·81)
Kenya	35·09 (34·23 to 35·94)	36·82 (36·01 to 37·68)	37·34 (36·32 to 38·27)	38·60 (37·65 to 39·52)	40·85 (39·72 to 41·93)	46·06 (44·85 to 47·37)	46·38 (45·14 to 47·74)	41·39 (40·22 to 42·60)	33·54 (32·23 to 34·90)	26·21 (24·96 to 27·62)
Madagascar	25·20 (22·07 to 28·78)	27·62 (24·33 to 31·37)	31·30 (28·05 to 34·78)	36·55 (32·38 to 40·48)	35·59 (32·13 to 39·22)	36·60 (32·68 to 40·80)	35·79 (31·63 to 40·22)	34·03 (29·42 to 38·64)	35·16 (29·54 to 40·93)	32·20 (25·07 to 41·54)
Malawi	39·47 (35·39 to 43·74)	38·46 (34·94 to 42·13)	37·35 (34·20 to 40·74)	38·74 (35·63 to 42·21)	45·97 (42·39 to 49·68)	40·99 (37·83 to 44·55)	37·49 (34·22 to 40·95)	30·88 (27·86 to 33·78)	28·60 (24·88 to 32·66)	22·51 (18·27 to 28·36)
Mozambique	49·80 (42·31 to 58·26)	52·15 (44·93 to 59·65)	57·85 (50·13 to 65·50)	70·02 (61·71 to 78·21)	54·09 (46·01 to 61·68)	57·59 (49·89 to 65·24)	55·81 (50·23 to 61·58)	51·88 (47·01 to 56·93)	47·78 (41·94 to 53·40)	38·08 (31·45 to 45·44)
Rwanda	21·00 (19·04 to 22·95)	24·65 (22·49 to 27·10)	25·67 (23·30 to 28·35)	24·48 (22·31 to 26·95)	26·39 (23·97 to 29·05)	24·50 (21·62 to 27·45)	22·88 (20·01 to 25·93)	18·23 (15·83 to 20·62)	12·07 (10·48 to 13·72)	8·86 (7·21 to 10·88)
Somalia	10·44 (7·74 to 13·45)	13·65 (10·81 to 16·83)	16·64 (13·51 to 20·10)	18·71 (14·85 to 22·30)	17·31 (13·73 to 20·98)	15·88 (12·63 to 19·12)	15·53 (12·76 to 18·53)	14·49 (12·07 to 16·93)	17·88 (14·58 to 21·40)	12·91 (9·68 to 16·19)
South Sudan	24·97 (18·87 to 32·65)	25·83 (19·90 to 32·69)	27·32 (22·26 to 33·05)	29·95 (25·64 to 34·30)	28·97 (24·72 to 33·26)	23·21 (19·95 to 26·55)	22·27 (19·55 to 24·79)	23·18 (20·17 to 26·53)	25·10 (20·91 to 29·99)	28·09 (21·50 to 36·32)
Tanzania	73·08 (66·33 to 79·65)	75·82 (69·13 to 82·37)	78·93 (71·39 to 86·58)	82·99 (75·81 to 90·48)	85·93 (77·80 to 93·71)	90·43 (82·45 to 98·24)	83·28 (76·94 to 89·88)	74·45 (67·67 to 81·27)	64·30 (55·28 to 73·71)	50·37 (40·59 to 60·87)
Uganda	44·45 (41·10 to 47·59)	48·37 (45·46 to 51·48)	56·17 (52·76 to 59·79)	60·76 (57·60 to 64·12)	64·42 (61·14 to 67·72)	66·98 (63·51 to 70·64)	65·50 (62·57 to 68·58)	63·28 (59·90 to 66·84)	57·86 (53·48 to 62·46)	45·07 (39·79 to 51·24)
Zambia	19·47 (17·12 to 22·37)	19·92 (17·58 to 22·39)	22·54 (20·31 to 25·11)	26·64 (24·11 to 29·60)	29·64 (27·39 to 31·85)	29·54 (26·85 to 32·47)	28·13 (24·71 to 31·56)	24·34 (21·47 to 27·59)	19·91 (17·30 to 22·85)	15·98 (12·62 to 19·78)
Central Sub-Saharan Africa	144·43 (94·74 to 192·02)	148·25 (97·96 to 199·30)	152·25 (104·94 to 194·65)	151·98 (106·38 to 190·50)	171·55 (118·57 to 215·73)	189·98 (130·87 to 242·53)	202·38 (140·32 to 257·06)	197·66 (147·51 to 246·81)	190·73 (133·32 to 249·70)	160·78 (109·36 to 220·52)
Angola	28·33 (20·03 to 38·21)	30·59 (23·13 to 38·83)	34·44 (27·73 to 42·43)	39·03 (32·88 to 45·69)	45·32 (38·49 to 52·50)	45·46 (38·59 to 53·04)	47·96 (40·82 to 55·83)	44·78 (37·78 to 52·90)	39·47 (31·83 to 48·33)	26·69 (17·67 to 38·33)
Central African Republic	6·77 (5·56 to 8·13)	7·24 (6·03 to 8·60)	7·38 (6·25 to 8·76)	8·09 (6·79 to 9·45)	8·72 (7·49 to 10·08)	9·71 (8·21 to 11·56)	10·89 (9·05 to 13·01)	11·02 (8·88 to 13·46)	11·03 (8·52 to 13·95)	10·56 (7·57 to 14·53)
Congo	5·01 (3·87 to 6·25)	4·82 (3·77 to 5·98)	4·47 (3·60 to 5·41)	3·96 (3·33 to 4·63)	4·09 (3·47 to 4·79)	4·79 (4·15 to 5·49)	5·62 (4·91 to 6·39)	5·19 (4·51 to 5·91)	4·58 (3·79 to 5·54)	3·75 (2·91 to 4·89)
Democratic Republic of the Congo	101·05 (51·87 to 149·91)	103·15 (52·59 to 153·13)	104·03 (56·79 to 146·72)	98·68 (53·64 to 137·42)	110·84 (59·14 to 156·12)	127·34 (68·94 to 178·07)	135·64 (75·05 to 190·44)	134·59 (84·03 to 183·36)	133·77 (76·50 to 191·15)	118·26 (68·23 to 176·14)
Equatorial Guinea	1·40 (1·06 to 1·78)	0·91 (0·74 to 1·09)	0·65 (0·54 to 0·78)	1·07 (0·85 to 1·31)	1·44 (1·17 to 1·78)	1·57 (1·30 to 1·88)	1·10 (0·87 to 1·37)	0·88 (0·65 to 1·16)	0·75 (0·54 to 1·04)	0·56 (0·38 to 0·81)
Gabon	1·87 (1·35 to 2·42)	1·53 (1·17 to 1·93)	1·28 (0·95 to 1·62)	1·15 (0·85 to 1·47)	1·14 (0·85 to 1·44)	1·12 (0·85 to 1·39)	1·16 (0·92 to 1·39)	1·22 (0·96 to 1·49)	1·13 (0·88 to 1·45)	0·96 (0·69 to 1·27)

Appendix Table 13A. Estimates of life expectancy at birth by location, both sexes combined, 1970, 1975, 1980, 1985, 1990, 1995, 2000, 2005, 2010, 2016

Location	1970	1975	1980	1985	1990	1995	2000	2005	2010	2016
Global	58·4 (57·9-58·9)	60·4 (60·0-60·8)	62·1 (61·8-62·4)	63·7 (63·5-64·0)	65·1 (64·9-65·3)	65·7 (65·5-65·9)	66·8 (66·6-67·0)	68·4 (68·1-68·6)	70·3 (70·0-70·6)	72·5 (72·1-72·8)
High SDI	70·9 (70·7-71·1)	72·2 (72·1-72·4)	73·6 (73·5-73·7)	74·7 (74·7-74·8)	75·8 (75·7-75·9)	76·7 (76·6-76·7)	77·9 (77·9-78·0)	79·0 (79·0-79·1)	80·1 (80·1-80·2)	80·7 (80·6-80·9)
High-middle SDI	65·0 (64·5-65·6)	66·7 (66·3-67·1)	67·6 (67·1-68·0)	69·0 (68·7-69·4)	70·0 (69·6-70·3)	69·3 (69·0-69·7)	70·9 (70·5-71·2)	72·2 (71·9-72·6)	74·7 (74·4-74·9)	76·4 (75·5-77·2)
Middle SDI	58·8 (58·1-59·5)	61·5 (61·0-61·9)	63·2 (62·8-63·6)	65·1 (64·8-65·5)	66·6 (66·4-66·9)	67·9 (67·7-68·1)	69·1 (69·0-69·3)	70·6 (70·4-70·8)	72·3 (72·1-72·5)	74·2 (73·9-74·4)
Low-middle SDI	49·8 (49·0-50·6)	52·3 (51·6-53·0)	55·2 (54·7-55·7)	57·2 (56·7-57·6)	59·0 (58·6-59·4)	60·1 (59·7-60·5)	61·3 (61·0-61·7)	63·1 (62·7-63·5)	65·4 (65·0-65·8)	67·8 (67·3-68·3)
Low SDI	47·4 (46·3-48·6)	48·9 (48·1-49·6)	50·2 (49·6-50·8)	51·2 (50·5-51·8)	52·5 (52·0-52·9)	53·0 (52·5-53·4)	53·9 (53·5-54·4)	56·5 (56·0-57·0)	58·8 (57·9-59·6)	63·0 (62·3-63·7)
High-income	70·7 (70·5-70·8)	72·1 (72·0-72·2)	73·8 (73·7-73·8)	75·0 (74·9-75·1)	76·0 (76·0-76·1)	77·0 (76·9-77·0)	78·2 (78·1-78·2)	79·3 (79·2-79·3)	80·3 (80·3-80·4)	80·9 (80·8-81·1)
High-income North America	70·9 (70·8-71·0)	72·5 (72·4-72·6)	74·0 (74·0-74·1)	74·9 (74·8-75·0)	75·6 (75·6-75·7)	76·2 (76·1-76·2)	77·2 (77·1-77·3)	77·9 (77·9-78·0)	79·0 (79·0-79·1)	79·2 (79·0-79·3)
Canada	72·6 (72·3-72·8)	73·5 (73·3-73·8)	75·2 (74·9-75·4)	76·5 (76·2-76·7)	77·4 (77·2-77·7)	78·1 (77·9-78·3)	79·3 (79·0-79·5)	80·2 (80·0-80·4)	81·3 (81·1-81·6)	81·9 (81·5-82·2)
Greenland	63·3 (62·4-64·1)	62·0 (61·1-62·9)	61·4 (60·6-62·3)	60·8 (60·0-61·6)	61·4 (60·3-62·4)	64·0 (63·1-64·9)	65·0 (64·2-65·9)	66·0 (65·2-66·8)	68·3 (67·4-69·3)	70·0 (67·9-72·0)
United States	70·8 (70·7-70·9)	72·4 (72·3-72·5)	73·9 (73·8-74·0)	74·8 (74·7-74·8)	75·5 (75·4-75·5)	76·0 (75·9-76·0)	77·0 (76·9-77·0)	77·7 (77·6-77·8)	78·8 (78·7-78·8)	78·9 (78·7-79·0)
Australasia	71·1 (71·0-71·3)	72·5 (72·3-72·7)	74·2 (74·1-74·4)	75·4 (75·2-75·5)	76·8 (76·6-77·0)	78·1 (77·9-78·2)	79·5 (79·4-79·7)	80·9 (80·7-81·0)	81·8 (81·7-82·0)	82·4 (81·9-82·8)
Australia	71·1 (70·8-71·3)	72·6 (72·4-72·8)	74·5 (74·3-74·7)	75·6 (75·4-75·8)	77·0 (76·8-77·2)	78·3 (78·1-78·5)	79·7 (79·5-79·9)	81·1 (80·9-81·3)	82·0 (81·8-82·2)	82·5 (82·0-83·0)
New Zealand	71·4 (71·2-71·7)	72·1 (71·8-72·4)	73·1 (72·9-73·4)	74·1 (73·8-74·3)	75·6 (75·4-75·9)	77·0 (76·7-77·3)	78·6 (78·3-78·8)	79·9 (79·7-80·1)	80·9 (80·7-81·2)	81·5 (80·6-82·3)
High-income Asia Pacific	69·7 (68·7-70·5)	72·0 (71·3-72·6)	74·2 (73·8-74·5)	76·0 (75·7-76·3)	77·4 (77·2-77·7)	78·6 (78·3-78·9)	80·0 (79·8-80·2)	81·3 (81·1-81·5)	82·4 (82·2-82·6)	83·3 (82·6-83·9)
Brunei	65·6 (64·4-66·9)	69·2 (68·0-70·4)	69·8 (68·2-71·8)	72·0 (71·1-72·9)	73·3 (72·5-74·2)	74·2 (73·3-75·1)	75·8 (75·1-76·5)	76·6 (76·1-77·2)	77·0 (76·3-77·6)	76·9 (75·5-78·6)
Japan	72·3 (72·0-72·6)	74·3 (74·1-74·5)	76·2 (76·2-76·3)	77·8 (77·7-77·8)	78·9 (78·9-79·0)	79·8 (79·6-79·9)	81·0 (81·0-81·1)	82·0 (82·0-82·1)	82·9 (82·9-82·9)	83·9 (83·8-84·1)
Singapore	68·4 (67·2-69·6)	70·2 (69·1-71·2)	71·4 (70·4-72·5)	73·1 (72·0-74·4)	75·4 (74·3-76·5)	77·4 (76·4-78·4)	79·4 (78·3-80·4)	81·0 (80·0-81·9)	82·8 (81·7-83·7)	83·7 (81·9-85·4)
South Korea	61·8 (58·2-65·8)	64·1 (61·6-66·7)	67·3 (66·1-68·6)	69·7 (68·5-71·0)	72·0 (70·9-73·1)	74·2 (73·1-75·3)	76·0 (75·2-77·0)	78·4 (77·6-79·3)	80·2 (79·4-81·2)	81·0 (78·5-83·6)
Western Europe	71·6 (71·5-71·7)	72·6 (72·5-72·8)	74·0 (73·9-74·1)	75·2 (75·1-75·3)	76·3 (76·2-76·4)	77·3 (77·2-77·4)	78·6 (78·5-78·7)	79·8 (79·7-79·9)	80·9 (80·8-81·0)	81·7 (81·5-81·9)
Andorra	76·0 (73·3-78·7)	77·6 (75·2-80·0)	78·9 (76·8-80·9)	79·2 (77·3-81·2)	79·6 (78·1-81·5)	80·5 (78·7-82·2)	81·9 (80·4-83·4)	82·5 (81·2-83·7)	82·7 (81·4-84·0)	82·5 (80·9-84·1)
Austria	70·2 (70·0-70·4)	71·4 (71·2-71·6)	72·7 (72·5-72·9)	74·1 (73·8-74·3)	75·7 (75·5-75·9)	76·8 (76·7-77·0)	78·3 (78·1-78·5)	79·6 (79·4-79·8)	80·6 (80·4-80·8)	81·5 (81·0-82·0)
Belgium	70·9 (70·6-71·2)	72·0 (71·7-72·4)	73·2 (72·9-73·5)	74·5 (74·2-74·8)	76·0 (75·7-76·3)	76·9 (76·6-77·2)	77·8 (77·5-78·2)	79·2 (78·9-79·5)	80·0 (79·7-80·3)	80·9 (80·1-81·8)
Cyprus	70·3 (69·7-70·8)	71·1 (70·7-71·5)	72·8 (72·5-73·2)	74·0 (73·7-74·4)	74·9 (74·6-75·3)	75·7 (75·3-76·0)	76·8 (76·5-77·2)	78·0 (77·7-78·4)	79·9 (79·5-80·2)	80·5 (80·0-80·9)
Denmark	73·3 (72·8-73·9)	74·1 (73·6-74·6)	74·3 (73·7-74·9)	74·7 (74·2-75·3)	75·2 (74·6-75·8)	75·6 (75·1-76·2)	77·0 (76·4-77·5)	78·1 (77·5-78·7)	79·5 (79·0-80·0)	80·8 (79·8-81·8)
Finland	70·1 (69·8-70·4)	71·7 (71·4-72·0)	73·6 (73·4-73·9)	74·6 (74·3-74·9)	75·1 (74·8-75·3)	76·7 (76·5-77·0)	77·8 (77·6-78·0)	79·0 (78·7-79·2)	80·0 (79·8-80·3)	81·7 (81·1-82·4)
France	72·5 (72·2-72·7)	73·2 (72·9-73·5)	74·6 (74·3-74·8)	75·8 (75·6-76·1)	77·2 (76·9-77·4)	78·1 (77·9-78·4)	79·1 (78·9-79·4)	80·4 (80·2-80·6)	81·5 (81·3-81·7)	82·3 (81·9-82·8)
Germany	70·8 (70·3-71·2)	71·7 (71·3-72·1)	73·2 (72·7-73·6)	74·5 (74·0-74·9)	75·5 (75·0-75·9)	76·6 (76·2-77·1)	78·0 (77·6-78·4)	79·2 (78·9-79·7)	80·1 (79·8-80·6)	80·9 (80·2-81·6)
Greece	73·5 (73·3-73·7)	74·6 (74·4-74·8)	77·1 (76·9-77·4)	76·2 (76·0-76·5)	77·0 (76·7-77·2)	77·8 (77·5-78·0)	78·6 (78·4-78·9)	79·6 (79·4-79·8)	80·4 (80·2-80·6)	81·0 (80·3-81·6)
Iceland	73·8 (73·4-74·2)	75·1 (74·7-75·5)	76·6 (76·2-77·1)	77·3 (76·9-77·7)	78·0 (77·6-78·5)	78·5 (78·0-79·0)	79·9 (79·5-80·3)	81·2 (80·8-81·6)	82·0 (81·7-82·4)	82·3 (81·6-83·0)
Ireland	71·0 (70·6-71·5)	71·6 (71·1-72·0)	72·5 (72·0-73·0)	73·7 (73·3-74·2)	74·8 (74·4-75·3)	75·8 (75·3-76·2)	76·6 (76·1-77·1)	78·9 (78·5-79·4)	80·4 (80·0-80·8)	81·1 (80·0-82·2)
Israel	69·5 (68·0-71·0)	71·5 (70·7-72·4)	73·6 (72·7-74·6)	74·5 (73·6-75·4)	75·8 (75·0-76·6)	77·0 (76·2-78·0)	78·5 (77·6-79·5)	79·7 (78·9-80·5)	81·5 (80·7-82·2)	82·1 (80·6-83·6)
Italy	71·6 (71·4-71·8)	72·8 (72·7-73·1)	74·1 (73·9-74·3)	75·7 (75·5-75·9)	77·0 (76·8-77·2)	78·1 (77·9-78·3)	79·6 (79·4-79·8)	80·9 (80·7-81·1)	81·8 (81·6-81·9)	82·3 (81·8-82·9)
Luxembourg	69·9 (69·6-70·3)	71·2 (70·8-71·5)	72·6 (72·2-73·0)	73·9 (73·5-74·2)	75·2 (74·8-75·5)	76·8 (76·5-77·2)	78·5 (78·2-78·9)	80·0 (79·7-80·4)	81·4 (81·1-81·8)	82·1 (81·3-83·0)
Malta	69·5 (68·7-70·3)	70·7 (70·0-71·5)	71·6 (70·8-72·3)	73·6 (72·9-74·3)	75·8 (75·0-76·5)	77·1 (76·4-77·9)	77·5 (76·8-78·2)	79·3 (78·6-80·0)	80·3 (79·6-81·0)	81·4 (80·0-82·9)
Netherlands	73·7 (73·3-74·1)	74·6 (74·3-75·0)	75·8 (75·4-76·2)	76·4 (76·0-76·7)	77·0 (76·7-77·4)	77·5 (77·2-77·9)	78·1 (77·8-78·5)	79·5 (79·2-79·8)	80·8 (80·5-81·1)	81·6 (80·9-82·3)
Norway	74·0 (73·6-74·3)	74·8 (74·4-75·1)	75·7 (75·3-76·1)	76·1 (75·7-76·5)	76·8 (76·4-77·2)	77·9 (77·6-78·3)	78·7 (78·4-79·1)	80·2 (79·8-80·5)	81·1 (80·7-81·4)	82·1 (81·3-82·9)
Portugal	67·5 (67·2-67·7)	69·8 (69·5-70·1)	71·7 (71·4-71·9)	73·2 (73·0-73·5)	74·3 (74·0-74·6)	75·3 (75·0-75·6)	76·7 (76·5-77·0)	78·4 (78·1-78·7)	79·9 (79·6-80·2)	81·0 (80·4-81·6)
Spain	72·0 (71·8-72·1)	73·5 (73·3-73·7)	75·4 (75·3-75·6)	76·5 (76·3-76·6)	77·1 (76·9-77·3)	78·1 (78·0-78·3)	79·4 (79·2-79·6)	80·5 (80·3-80·6)	82·0 (81·8-82·1)	83·0 (82·6-83·3)
Sweden	74·5 (74·0-75·1)	75·0 (74·5-75·4)	75·8 (75·5-76·1)	76·9 (76·6-77·2)	77·6 (77·4-77·9)	78·9 (78·6-79·1)	79·7 (79·5-80·0)	80·5 (80·3-80·8)	81·5 (81·3-81·8)	82·0 (81·1-83·0)
Stockholm	74·1 (72·2-76·1)	74·6 (73·1-76·0)	75·5 (75·0-76·1)	76·8 (76·3-77·3)	77·3 (76·8-77·8)	78·7 (78·3-79·2)	79·7 (79·3-80·1)	80·8 (80·4-81·2)	81·9 (81·5-82·3)	82·3 (80·6-83·9)
Sweden except Stockholm	74·6 (74·1-75·1)	75·1 (74·6-75·5)	75·8 (75·5-76·2)	76·9 (76·5-77·2)	77·7 (77·4-78·0)	78·9 (78·6-79·2)	79·7 (79·4-80·0)	80·5 (80·2-80·8)	81·5 (81·2-81·7)	82·0 (80·9-83·1)
Switzerland	73·0 (72·5-73·6)	74·7 (74·2-75·3)	75·7 (75·1-76·2)	76·9 (76·4-77·5)	77·5 (76·9-78·0)	78·5 (78·0-79·0)	80·0 (79·4-80·5)	81·3 (80·8-81·7)	82·3 (81·8-82·8)	83·2 (81·3-84·9)
United Kingdom	71·9 (71·7-72·0)	72·5 (72·4-72·7)	73·6 (73·5-73·7)	74·6 (74·5-74·7)	75·7 (75·6-75·8)	76·7 (76·6-76·8)	77·8 (77·7-77·9)	79·1 (79·0-79·2)	80·4 (80·3-80·5)	80·9 (80·7-81·0)
England	72·2 (72·0-72·3)	72·8 (72·7-72·9)	73·8 (73·7-73·9)	74·9 (74·8-74·9)	76·0 (75·9-76·0)	76·9 (76·9-77·0)	78·1 (78·0-78·2)	79·4 (79·3-79·4)	80·7 (80·6-80·7)	81·2 (81·1-81·3)
East Midlands	72·0 (71·4-72·5)	72·7 (72·2-73·2)	73·8 (73·4-74·2)	74·9 (74·6-75·1)	75·9 (75·7-76·1)	76·9 (76·7-77·2)	78·1 (77·8-78·3)	79·3 (79·0-79·5)	80·5 (80·3-80·7)	81·0 (80·6-81·3)
East of England	73·1 (72·6-73·7)	73·9 (73·4-74·3)	74·9 (74·6-75·3)	75·8 (75·6-76·1)	77·0 (76·8-77·3)	78·0 (77·7-78·2)	79·0 (78·8-79·2)	80·3 (80·0-80·5)	81·5 (81·2-81·7)	81·9 (81·6-82·3)
Greater London	72·6 (72·3-72·8)	73·1 (72·8-73·4)	74·0 (73·8-74·2)	75·0 (74·8-75·2)	76·0 (75·8-76·1)	76·8 (76·6-76·9)	78·0 (77·8-78·1)	79·5 (79·3-79·6)	81·1 (81·0-81·3)	81·8 (81·6-82·0)
North East England	70·6 (70·1-71·1)	71·4 (71·0-71·8)	72·4 (72·1-72·7)	73·4 (73·1-73·6)	74·4 (74·2-74·7)	75·4 (75·2-75·6)	76·7 (76·5-76·9)	77·9 (77·7-78·1)	79·3 (79·0-79·5)	79·8 (79·4-80·1)
North West England	70·9 (70·5-71·4)	71·6 (71·1-72·0)	72·6 (72·4-72·9)	73·7 (73·5-73·9)	74·7 (74·5-74·9)	75·7 (75·5-75·8)	76·8 (76·6-77·0)	78·0 (77·9-78·2)	79·3 (79·2-79·5)	79·9 (79·6-80·2)
South East England	73·5 (73·0-73·9)	74·1 (73·7-74·5)	75·1 (74·8-75·3)	76·0 (75·7-76·2)	77·1 (76·9-77·3)	78·0 (77·8-78·2)	79·2 (79·0-79·4)	80·4 (80·2-80·6)	81·7 (81·5-81·8)	82·1 (81·8-82·4)
South West England	73·3 (72·8-73·7)	73·9 (73·6-74·3)	74·9 (74·7-75·2)	76·0 (75·8-76·2)	77·2 (77·0-77·3)	78·1 (77·9-78·3)	79·2 (79·0-79·4)	80·4 (80·2-80·6)	81·5 (81·4-81·7)	81·9 (81·6-82·2)
West Midlands	71·6 (71·1-72·1)	72·3 (71·9-72·8)	73·3 (73·0-73·6)	74·3 (74·0-74·5)	75·4 (75·2-75·7)	76·6 (76·3-76·8)	77·6 (77·3-77·8)	78·8 (78·6-79·0)	80·2 (80·0-80·4)	80·7 (80·3-81·0)
Yorkshire and the Humber	71·4 (70·9-71·8)	72·0 (71·7-72·4)	73·1 (72·8-73·4)	74·1 (73·9-74·3)	75·4 (75·2-75·6)	76·4 (76·2-76·6)	77·5 (77·3-77·7)	78·8 (78·6-78·9)	79·9 (79·7-80·1)	80·4 (80·1-80·7)
Northern Ireland	70·8 (70·2-71·3)	71·0 (70·4-71·5)	72·1 (71·6-72·7)	73·5 (72·8-74·2)	74·9 (74·2-75·7)	76·1 (75·5-76·9)	77·4 (76·7-78·1)	78·6 (77·9-79·2)	79·6 (79·0-80·2)	80·1 (79·1-81·4)
Scotland	70·5 (70·0-70·9)	71·0 (70·5-71·5)	72·1 (71·7-72·6)	72·9 (72·3-73·6)	73·9 (73·3-74·6)	74·8 (74·2-75·5)	76·0 (75·3-76·7)	77·1 (76·4-77·7)	78·4 (77·8-79·0)	79·1 (78·0-80·0)

Appendix Table 13A. Estimates of life expectancy at birth by location, both sexes combined, 1970, 1975, 1980, 1985, 1990, 1995, 2000, 2005, 2010, 2016

Location	1970	1975	1980	1985	1990	1995	2000	2005	2010	2016
Wales	71·2 (69·6–72·8)	71·9 (70·6–73·3)	73·1 (72·2–74·1)	74·4 (73·7–75·0)	75·5 (74·9–76·1)	76·3 (75·8–77·0)	77·4 (76·9–78·1)	78·8 (78·2–79·4)	79·7 (79·1–80·3)	80·1 (79·0–81·1)
Southern Latin America	64·9 (64·5–65·2)	67·2 (66·8–67·7)	69·9 (69·5–70·2)	71·5 (71·2–71·8)	72·5 (72·2–72·8)	73·7 (73·4–74·0)	75·0 (74·7–75·2)	76·0 (75·7–76·3)	76·7 (76·5–77·0)	77·7 (76·9–78·4)
Argentina	65·1 (64·7–65·6)	67·2 (66·5–67·8)	69·7 (69·3–70·1)	71·2 (70·9–71·6)	72·1 (71·7–72·5)	73·1 (72·7–73·5)	74·2 (73·8–74·5)	75·2 (74·8–75·6)	75·9 (75·5–76·2)	76·7 (76·0–77·5)
Chile	63·4 (62·8–64·0)	67·2 (66·5–67·8)	70·2 (69·6–70·9)	72·1 (71·5–72·7)	73·5 (72·9–74·1)	75·4 (74·8–76·0)	77·3 (76·7–77·8)	78·4 (77·9–79·0)	79·2 (78·7–79·8)	80·3 (78·1–82·5)
Uruguay	68·4 (68·1–68·7)	68·6 (68·3–69·0)	70·1 (69·7–70·4)	71·6 (71·3–71·9)	72·7 (72·4–73·1)	73·5 (73·2–73·8)	74·8 (74·5–75·1)	75·6 (75·4–75·9)	76·4 (76·1–76·8)	77·3 (76·7–78·0)
Central Europe, Eastern Europe, and Central Asi	67·7 (67·0–68·2)	68·2 (67·7–68·6)	68·0 (67·5–68·4)	68·6 (68·2–69·0)	69·2 (68·8–69·6)	66·3 (65·8–66·9)	67·6 (67·1–68·1)	68·2 (67·7–68·8)	71·0 (70·4–71·5)	72·7 (71·1–74·2)
Eastern Europe	69·1 (68·4–69·8)	68·7 (67·9–69·4)	68·0 (67·2–68·7)	68·7 (68·0–69·3)	69·5 (68·7–70·2)	65·1 (64·2–66·0)	66·1 (65·2–67·0)	66·3 (65·4–67·2)	69·7 (68·9–70·5)	71·4 (68·7–74·0)
Belarus	71·3 (70·6–72·0)	71·6 (70·9–72·2)	70·8 (70·2–71·3)	70·7 (70·2–71·3)	70·7 (70·2–71·3)	68·2 (67·7–68·8)	68·3 (67·7–68·9)	68·7 (68·1–69·3)	70·3 (69·8–70·9)	73·6 (71·9–75·5)
Estonia	70·6 (70·1–71·0)	70·2 (69·7–70·6)	69·5 (69·1–70·0)	69·7 (69·3–70·2)	70·2 (69·7–70·6)	67·7 (67·2–68·2)	70·8 (70·4–71·3)	72·9 (72·5–73·4)	76·2 (75·8–76·7)	77·6 (76·5–78·8)
Latvia	70·4 (69·9–70·9)	69·3 (68·7–69·8)	69·1 (68·6–69·7)	69·7 (69·2–70·2)	69·6 (69·1–70·2)	66·4 (65·9–67·1)	70·5 (69·9–71·1)	70·8 (70·3–71·4)	73·2 (72·7–73·7)	75·1 (73·8–76·4)
Lithuania	71·3 (70·9–71·7)	71·2 (70·8–71·6)	70·8 (70·4–71·2)	70·9 (70·5–71·3)	71·5 (71·1–71·9)	68·9 (68·5–69·4)	72·1 (71·7–72·5)	71·3 (70·9–71·8)	73·2 (72·8–73·7)	75·2 (74·3–76·0)
Moldova	62·7 (59·7–65·7)	64·0 (61·5–66·5)	65·0 (63·4–66·5)	65·5 (64·4–66·6)	67·3 (66·3–68·2)	64·6 (63·6–65·5)	67·6 (66·7–68·5)	68·6 (67·7–69·5)	69·7 (68·7–70·6)	72·2 (71·0–73·5)
Russia	68·9 (68·0–69·9)	68·3 (67·3–69·2)	67·4 (66·3–68·5)	68·2 (67·2–69·1)	69·2 (68·1–70·3)	64·3 (63·0–65·7)	65·3 (64·1–66·5)	65·6 (64·4–66·9)	69·2 (68·1–70·4)	70·9 (67·1–74·5)
Ukraine	69·5 (68·1–70·6)	69·5 (68·4–70·5)	69·0 (68·1–69·8)	69·7 (69·1–70·3)	70·1 (69·5–70·7)	66·5 (65·8–67·3)	67·4 (66·6–68·1)	67·1 (66·4–67·8)	70·5 (69·8–71·1)	72·1 (69·4–74·7)
Central Europe	69·1 (68·7–69·4)	70·2 (69·9–70·4)	70·2 (70·0–70·4)	70·5 (70·3–70·7)	70·9 (70·8–71·1)	71·1 (70·9–71·3)	72·9 (72·7–73·0)	74·1 (73·9–74·3)	75·4 (75·3–75·6)	76·9 (76·5–77·2)
Albania	67·4 (63·7–70·2)	70·3 (67·7–72·3)	72·2 (70·6–73·7)	72·9 (72·1–73·7)	73·7 (73·2–74·3)	74·1 (73·5–74·5)	74·3 (73·8–74·7)	74·8 (74·3–75·2)	76·2 (75·5–76·8)	77·6 (76·5–78·8)
Bosnia and Herzegovina	66·5 (63·9–68·8)	68·1 (66·0–70·1)	69·6 (68·0–71·3)	71·0 (69·7–72·3)	72·1 (71·0–73·3)	68·7 (67·3–70·0)	74·9 (73·7–76·0)	75·8 (74·7–76·8)	76·8 (75·7–77·8)	77·6 (76·2–79·0)
Bulgaria	71·0 (70·7–71·3)	71·2 (70·9–71·5)	71·2 (70·9–71·5)	71·2 (70·9–71·5)	71·4 (71·1–71·7)	70·9 (70·5–71·2)	71·7 (71·3–72·0)	72·6 (72·3–72·9)	73·8 (73·5–74·1)	75·0 (73·5–76·6)
Croatia	68·0 (65·4–70·4)	69·4 (67·0–71·7)	70·5 (68·4–72·5)	71·1 (70·1–72·0)	72·2 (71·5–73·0)	72·3 (71·3–73·2)	73·9 (73·3–74·6)	75·1 (74·5–75·8)	76·4 (75·7–76·9)	77·4 (76·2–78·6)
Czech Republic	69·6 (69·4–69·9)	70·4 (70·2–70·6)	70·6 (70·4–70·8)	71·1 (70·9–71·3)	71·8 (71·6–72·0)	73·4 (73·1–73·6)	75·0 (74·8–75·3)	76·2 (76·0–76·4)	77·5 (77·3–77·7)	79·1 (78·6–79·6)
Hungary	69·2 (68·7–69·6)	69·4 (68·9–70·0)	69·3 (68·8–69·8)	69·1 (68·5–69·6)	69·5 (69·0–70·1)	70·1 (69·5–70·6)	71·8 (71·2–72·3)	73·1 (72·6–73·7)	74·6 (74·1–75·1)	75·7 (74·7–76·8)
Macedonia	67·4 (64·1–69·7)	68·8 (66·2–70·7)	69·3 (67·7–70·7)	70·1 (68·9–71·2)	70·5 (69·9–71·1)	71·1 (70·6–71·6)	72·2 (71·7–72·7)	72·9 (72·4–73·4)	74·0 (73·5–74·4)	74·8 (74·2–75·5)
Montenegro	72·7 (71·2–74·0)	73·4 (72·0–74·7)	74·2 (73·0–75·3)	74·4 (73·3–75·3)	75·0 (74·2–75·9)	74·0 (73·3–74·6)	73·8 (73·3–74·4)	75·0 (74·5–75·5)	76·4 (75·7–77·1)	77·0 (76·2–78·0)
Poland	69·9 (69·6–70·2)	70·8 (70·5–71·2)	70·4 (70·0–70·7)	70·7 (70·3–71·0)	70·9 (70·6–71·3)	71·8 (71·5–72·2)	73·8 (73·4–74·1)	75·2 (74·8–75·6)	76·3 (76·0–76·6)	77·9 (77·1–78·8)
Romania	68·4 (68·0–68·8)	70·1 (69·7–70·5)	69·7 (69·2–70·1)	69·8 (69·4–70·3)	69·8 (69·3–70·3)	69·3 (68·8–69·7)	71·1 (70·6–71·6)	72·4 (71·9–72·8)	73·6 (73·1–74·1)	75·2 (74·2–76·2)
Serbia	70·0 (68·4–71·4)	71·1 (69·6–72·4)	71·7 (70·4–72·9)	72·1 (70·9–73·2)	72·6 (71·7–73·5)	70·7 (70·1–71·3)	72·1 (71·6–72·8)	73·2 (72·7–73·8)	74·6 (74·2–75·1)	75·9 (75·4–76·5)
Slovakia	69·8 (69·3–70·2)	70·3 (69·9–70·7)	70·5 (70·0–70·9)	70·7 (70·3–71·2)	71·0 (70·5–71·4)	72·4 (72·0–72·8)	73·3 (72·8–73·7)	74·2 (73·8–74·6)	75·6 (75·2–75·9)	77·0 (75·9–78·0)
Slovenia	68·0 (65·1–71·0)	69·4 (66·8–71·8)	70·5 (69·0–72·1)	71·7 (71·0–72·6)	73·6 (72·9–74·4)	74·6 (73·8–75·3)	76·0 (75·3–76·8)	77·6 (76·9–78·4)	79·5 (78·9–80·1)	80·8 (79·8–82·1)
Central Asia	62·1 (60·6–63·8)	64·2 (62·9–65·3)	65·7 (64·9–66·4)	66·8 (66·3–67·4)	67·3 (66·8–67·7)	65·2 (64·7–65·8)	66·1 (65·6–66·6)	66·8 (66·1–67·4)	69·0 (68·4–69·6)	71·3 (70·4–72·2)
Armenia	65·6 (62·5–68·5)	68·1 (65·9–70·5)	69·8 (68·4–71·1)	70·8 (70·0–71·6)	70·3 (69·6–71·0)	69·3 (68·5–70·1)	72·0 (71·3–72·7)	72·5 (71·8–73·2)	73·3 (72·6–73·9)	75·9 (74·8–76·9)
Azerbaijan	61·4 (58·0–64·6)	64·6 (61·9–67·2)	65·7 (63·9–67·5)	66·1 (64·9–67·3)	66·3 (65·1–67·5)	64·9 (63·6–66·0)	66·5 (65·4–67·5)	67·6 (66·5–68·8)	69·1 (67·9–70·3)	72·0 (70·1–74·0)
Georgia	64·4 (61·5–67·5)	67·1 (64·3–69·6)	69·2 (67·8–70·8)	70·0 (69·0–70·9)	69·7 (68·8–70·6)	70·6 (69·7–71·4)	71·8 (70·9–72·7)	72·1 (71·3–72·9)	72·8 (71·9–73·6)	73·9 (72·0–75·7)
Kazakhstan	64·0 (60·6–67·6)	65·1 (62·0–68·5)	66·2 (64·3–68·2)	67·6 (66·4–68·9)	67·9 (66·9–69·1)	64·2 (63·0–65·5)	64·4 (63·2–65·6)	64·8 (63·5–66·0)	68·3 (67·2–69·5)	71·7 (69·7–73·7)
Kyrgyzstan	59·7 (57·5–62·3)	62·1 (59·9–64·2)	64·1 (62·9–65·2)	65·4 (64·8–66·0)	66·0 (65·5–66·6)	64·0 (63·5–64·6)	66·3 (65·7–66·9)	66·5 (66·0–67·0)	68·4 (67·8–68·9)	71·2 (70·2–72·3)
Mongolia	54·5 (52·5–56·6)	58·0 (56·3–59·8)	58·9 (57·4–60·4)	60·8 (59·6–62·2)	62·4 (61·3–63·6)	61·6 (60·6–62·7)	62·7 (61·8–63·7)	63·7 (62·7–64·7)	66·0 (64·9–67·2)	68·1 (66·5–69·8)
Tajikistan	56·4 (52·6–59·9)	58·6 (56·1–60·9)	61·8 (60·1–63·5)	63·8 (62·2–65·4)	65·0 (63·6–66·3)	63·8 (62·5–65·2)	65·2 (63·8–66·4)	67·3 (66·0–68·5)	69·5 (68·2–70·7)	71·8 (70·1–73·3)
Turkmenistan	55·9 (51·4–59·7)	58·7 (55·4–61·7)	61·3 (59·2–63·0)	63·0 (61·7–64·3)	63·7 (62·6–64·9)	63·1 (61·8–64·1)	63·6 (62·2–64·8)	65·3 (64·0–66·5)	67·9 (66·6–69·1)	70·2 (69·0–71·3)
Uzbekistan	63·5 (61·2–65·6)	65·3 (63·6–67·1)	66·5 (65·2–67·8)	67·8 (66·8–68·7)	68·4 (67·6–69·2)	66·2 (65·4–66·9)	66·3 (65·5–67·1)	66·7 (65·6–67·7)	68·8 (67·9–69·6)	70·3 (69·0–71·6)
Latin America and Caribbean	61·0 (60·2–61·8)	63·9 (63·5–64·3)	66·1 (65·8–66·5)	67·5 (67·2–67·8)	69·5 (69·3–69·7)	70·7 (70·5–70·9)	72·4 (72·2–72·6)	73·7 (73·5–73·9)	73·4 (72·2–74·4)	75·8 (75·4–76·2)
Central Latin America	62·5 (62·1–62·9)	64·8 (64·5–65·2)	67·1 (66·8–67·5)	68·5 (68·2–68·9)	71·1 (70·9–71·4)	72·3 (72·0–72·5)	73·8 (73·6–74·1)	74·8 (74·6–75·1)	75·5 (75·2–75·7)	76·5 (76·0–76·9)
Colombia	66·0 (65·4–66·5)	66·4 (65·9–66·9)	69·0 (68·4–69·5)	67·9 (67·0–68·8)	71·3 (70·9–71·7)	71·9 (71·5–72·3)	73·2 (72·8–73·6)	75·0 (74·6–75·3)	76·4 (76·1–76·8)	78·3 (77·3–79·2)
Costa Rica	68·9 (68·4–69·4)	71·8 (71·3–72·2)	74·5 (74·1–75·0)	75·9 (75·5–76·3)	76·5 (76·1–77·0)	77·0 (76·5–77·4)	78·0 (77·5–78·4)	79·3 (78·9–79·7)	80·4 (79·9–80·8)	81·0 (80·2–81·7)
El Salvador	58·8 (57·5–60·1)	61·8 (60·8–62·8)	64·8 (63·8–65·8)	64·5 (63·4–65·6)	68·1 (67·5–68·8)	70·3 (69·6–70·9)	72·2 (71·6–72·9)	73·2 (72·6–73·8)	74·3 (73·6–74·8)	75·5 (74·2–76·8)
Guatemala	50·8 (49·1–52·6)	55·8 (54·0–57·4)	58·8 (57·0–60·7)	62·0 (60·6–63·5)	64·6 (63·3–66·1)	66·6 (65·1–68·3)	68·6 (67·0–70·3)	69·8 (67·9–71·6)	71·3 (69·6–73·0)	72·8 (70·0–75·6)
Honduras	57·3 (56·0–58·8)	60·8 (59·3–62·3)	64·1 (62·5–65·7)	65·4 (63·9–66·9)	67·2 (65·3–68·8)	68·5 (66·2–70·4)	69·5 (67·1–72·1)	70·6 (67·9–73·6)	71·6 (68·8–74·7)	72·7 (69·8–75·4)
Mexico	62·1 (61·5–62·7)	64·6 (64·1–65·1)	66·6 (66·2–67·0)	69·0 (68·6–69·4)	71·5 (71·2–71·8)	72·8 (72·5–73·2)	74·6 (74·3–74·9)	75·2 (74·9–75·5)	75·4 (75·1–75·8)	76·4 (76·0–76·8)
Nicaragua	58·4 (57·3–59·4)	64·5 (63·7–65·2)	67·5 (66·5–68·6)	65·2 (62·3–68·4)	73·6 (73·1–74·1)	73·8 (73·2–74·3)	75·7 (75·2–76·1)	76·6 (76·2–77·1)	77·4 (77·0–77·9)	78·3 (76·7–79·7)
Panama	68·3 (67·5–69·0)	71·4 (70·7–72·2)	73·6 (72·9–74·3)	74·4 (73·6–75·0)	74·9 (74·1–75·7)	75·4 (74·6–76·1)	76·8 (76·1–77·4)	77·4 (76·7–78·2)	77·7 (76·9–78·5)	78·9 (77·5–80·2)
Venezuela	66·8 (66·2–67·5)	67·8 (67·2–68·4)	69·9 (69·2–70·5)	71·7 (71·1–72·4)	72·6 (72·0–73·3)	72·9 (72·1–73·7)	74·2 (73·5–74·9)	75·0 (74·3–75·8)	75·3 (74·5–76·0)	75·4 (73·3–77·1)
Andean Latin America	51·4 (46·7–56·2)	61·0 (60·4–61·7)	63·4 (62·7–64·0)	65·4 (64·9–66·0)	68·2 (67·7–68·8)	70·2 (69·7–70·7)	73·2 (72·7–73·6)	75·2 (74·7–75·7)	76·3 (75·7–76·9)	77·9 (76·8–78·9)
Bolivia	50·2 (48·8–51·5)	53·4 (52·3–54·6)	56·3 (55·3–57·4)	58·7 (57·6–59·8)	61·3 (60·3–62·4)	64·3 (63·3–65·3)	67·6 (66·9–68·4)	70·1 (69·0–71·0)	71·6 (70·0–73·1)	73·2 (71·1–75·3)
Ecuador	63·1 (62·4–63·8)	65·3 (64·6–65·9)	67·7 (67·1–68·3)	69·8 (69·3–70·4)	71·4 (70·9–71·8)	72·8 (72·3–73·2)	74·2 (73·7–74·7)	75·5 (75·0–76·1)	76·7 (76·2–77·2)	77·8 (77·1–78·6)
Peru	47·4 (40·3–55·8)	62·2 (61·1–63·2)	64·1 (63·1–65·0)	66·0 (65·1–66·8)	69·4 (68·6–70·2)	71·2 (70·4–72·0)	74·8 (74·1–75·6)	77·1 (76·3–77·9)	77·9 (77·1–78·8)	79·7 (77·9–81·4)
Caribbean	63·2 (62·4–64·0)	64·6 (63·9–65·4)	65·7 (64·9–66·5)	66·8 (66·1–67·4)	67·7 (67·1–68·2)	68·3 (67·7–68·8)	69·7 (69·1–70·3)	70·3 (69·6–71·1)	58·1 (48·7–69·2)	73·2 (72·0–74·3)
Antigua and Barbuda	67·0 (65·9–68·0)	68·6 (67·6–69·7)	70·8 (69·7–71·9)	72·7 (71·7–73·7)	73·8 (72·9–74·7)	74·4 (73·5–75·4)	74·8 (73·9–75·7)	76·0 (75·1–76·8)	76·9 (76·0–77·8)	77·4 (76·1–78·5)
The Bahamas	64·8 (63·1–66·1)	66·8 (65·6–67·8)	68·0 (66·9–69·0)	69·1 (68·1–70·1)	70·5 (69·5–71·3)	70·6 (69·8–71·3)	71·3 (70·7–71·9)	73·0 (72·4–73·7)	73·9 (73·2–74·5)	73·9 (72·5–75·1)

Appendix Table 13A. Estimates of life expectancy at birth by location, both sexes combined, 1970, 1975, 1980, 1985, 1990, 1995, 2000, 2005, 2010, 2016

Location	1970	1975	1980	1985	1990	1995	2000	2005	2010	2016
Barbados	69·5 (68·0-70·6)	71·1 (70·0-72·1)	72·8 (71·8-73·7)	73·4 (72·4-74·2)	73·7 (72·7-74·5)	73·8 (72·9-74·7)	75·0 (74·0-75·9)	76·0 (75·2-76·8)	76·8 (75·9-77·5)	76·6 (75·4-77·7)
Belize	66·4 (64·6-68·2)	68·2 (66·9-69·4)	69·2 (68·1-70·4)	70·9 (70·0-71·8)	71·6 (70·5-72·5)	70·4 (69·5-71·5)	68·6 (67·7-69·5)	69·8 (69·0-70·7)	71·1 (70·3-72·0)	71·8 (70·3-73·4)
Bermuda	66·9 (66·1-67·6)	68·2 (67·4-69·0)	69·0 (68·2-69·8)	69·3 (68·5-70·0)	69·7 (68·9-70·5)	71·2 (70·4-72·0)	72·5 (71·8-73·2)	75·2 (74·5-75·9)	77·0 (76·3-77·7)	78·9 (77·7-80·1)
Cuba	71·9 (71·5-72·2)	73·4 (73·0-73·7)	74·1 (73·6-74·4)	74·4 (74·0-74·9)	74·8 (74·4-75·2)	75·2 (74·8-75·6)	76·5 (76·2-76·9)	77·6 (77·2-78·0)	78·3 (78·0-78·7)	78·9 (78·2-79·7)
Dominica	67·3 (66·0-68·5)	70·3 (69·3-71·2)	73·1 (72·2-73·9)	73·2 (72·4-74·0)	72·0 (71·2-72·8)	71·6 (70·9-72·4)	72·9 (72·1-73·7)	73·3 (72·4-74·2)	73·0 (71·9-73·9)	72·9 (71·3-74·5)
Dominican Republic	64·9 (63·9-66·0)	67·1 (66·0-68·2)	69·1 (68·1-70·0)	69·9 (69·1-70·7)	70·7 (70·0-71·5)	72·0 (71·2-72·7)	73·1 (72·2-74·1)	73·3 (72·3-74·6)	74·5 (73·4-75·7)	75·6 (74·3-77·3)
Grenada	67·6 (65·2-69·6)	69·6 (67·4-71·5)	70·3 (68·1-72·2)	70·2 (68·3-71·8)	70·9 (69·5-72·1)	71·5 (70·4-72·5)	71·3 (70·2-72·3)	71·1 (70·0-72·0)	71·3 (70·3-72·5)	71·6 (70·0-73·3)
Guyana	60·7 (59·7-61·7)	61·7 (60·8-62·6)	63·1 (62·2-64·1)	64·1 (63·2-65·0)	64·2 (63·4-65·0)	64·9 (64·3-65·7)	65·4 (64·7-66·3)	64·6 (63·8-65·5)	65·4 (64·5-66·3)	67·7 (66·4-69·2)
Haiti	45·5 (43·1-47·9)	48·2 (46·1-50·5)	50·3 (48·2-52·6)	52·4 (50·7-54·1)	54·0 (52·5-55·5)	55·1 (53·5-56·6)	56·8 (55·0-58·6)	58·0 (56·1-60·0)	31·6 (21·0-53·5)	64·0 (61·4-66·7)
Jamaica	68·8 (67·8-69·8)	70·9 (69·8-72·1)	71·6 (70·5-72·7)	72·7 (71·8-73·6)	74·4 (73·3-75·4)	74·2 (72·9-75·3)	73·8 (72·8-74·8)	74·7 (73·6-75·7)	74·6 (73·4-75·7)	74·9 (73·1-76·7)
Puerto Rico	71·8 (71·3-72·4)	72·5 (72·0-73·0)	74·0 (73·4-74·5)	73·9 (73·3-74·6)	74·0 (73·4-74·5)	73·8 (73·2-74·3)	76·1 (75·6-76·6)	77·1 (76·6-77·6)	78·0 (77·5-78·5)	78·8 (77·8-79·7)
Saint Lucia	65·8 (62·9-67·8)	67·2 (65·6-68·4)	69·0 (67·8-70·0)	70·4 (69·2-71·3)	71·9 (70·9-72·7)	73·1 (72·2-73·9)	74·2 (73·4-74·8)	74·1 (73·3-74·9)	75·4 (74·6-76·2)	76·1 (74·9-77·1)
Saint Vincent and the Grenadines	65·0 (62·7-67·2)	66·8 (64·8-68·4)	69·1 (67·5-70·4)	70·4 (69·2-71·5)	70·7 (69·6-71·6)	70·4 (69·5-71·3)	70·1 (69·3-70·9)	71·1 (70·4-71·7)	71·5 (70·8-72·2)	71·6 (70·6-72·6)
Suriname	64·6 (63·4-65·6)	64·9 (64·0-65·8)	66·0 (65·3-66·7)	68·7 (68·1-69·3)	70·1 (69·5-70·6)	70·2 (69·6-70·8)	69·6 (69·0-70·3)	69·4 (68·7-70·1)	70·2 (69·5-70·9)	71·3 (70·3-72·3)
Trinidad and Tobago	65·4 (64·9-66·0)	66·5 (65·9-67·0)	68·1 (67·6-68·6)	68·9 (68·3-69·5)	69·0 (68·4-69·5)	69·2 (68·7-69·8)	69·4 (68·8-70·0)	71·3 (70·6-71·9)	72·4 (71·6-73·2)	73·0 (71·7-74·2)
Virgin Islands, U.S.	67·2 (66·0-68·1)	68·7 (67·6-69·6)	70·1 (69·3-71·0)	72·0 (71·3-73·0)	72·6 (71·7-73·7)	72·6 (71·7-73·4)	73·3 (72·5-74·0)	73·8 (73·1-74·4)	73·9 (73·0-74·8)	74·6 (73·4-76·2)
Tropical Latin America	61·4 (60·2-62·5)	63·6 (62·7-64·5)	65·9 (65·1-66·5)	67·0 (66·4-67·5)	68·6 (68·1-69·0)	69·8 (69·4-70·2)	71·4 (71·0-71·8)	73·0 (72·5-73·3)	74·1 (73·7-74·6)	75·2 (74·7-75·7)
Brazil	61·3 (60·1-62·5)	63·5 (62·6-64·4)	65·7 (65·0-66·4)	66·9 (66·3-67·4)	68·4 (68·0-68·9)	69·7 (69·2-70·1)	71·4 (70·9-71·8)	72·9 (72·5-73·3)	74·2 (73·7-74·6)	75·2 (74·7-75·7)
Paraguay	69·6 (68·8-70·3)	70·6 (70·0-71·2)	72·2 (71·5-72·8)	73·3 (72·7-73·8)	74·6 (74·1-75·1)	74·5 (73·8-75·0)	74·4 (73·9-74·9)	74·1 (73·4-74·6)	74·0 (73·3-74·7)	74·5 (73·3-75·6)
Southeast Asia, East Asia, and Oceania	59·6 (58·8-60·4)	61·8 (61·1-62·4)	63·6 (63·1-64·0)	65·4 (65·0-65·8)	66·5 (66·2-66·9)	67·7 (67·3-68·0)	69·1 (68·9-69·4)	71·2 (71·0-71·4)	73·2 (73·0-73·4)	75·1 (74·8-75·3)
East Asia	61·2 (60·2-62·0)	63·5 (62·7-64·2)	64·5 (63·8-65·1)	66·2 (65·7-66·8)	67·2 (66·6-67·6)	68·3 (67·8-68·7)	69·8 (69·5-70·2)	72·3 (72·0-72·5)	74·4 (74·2-74·6)	76·3 (76·0-76·5)
China	60·9 (60·0-61·8)	63·2 (62·5-63·9)	64·2 (63·5-64·9)	66·0 (65·4-66·6)	67·0 (66·4-67·4)	68·3 (67·9-68·7)	69·9 (69·6-70·2)	72·3 (72·0-72·5)	74·4 (74·2-74·6)	76·4 (76·1-76·6)
North Korea	67·6 (66·1-69·0)	70·1 (68·9-71·4)	71·2 (70·0-72·4)	71·6 (70·4-72·8)	71·9 (70·8-73·3)	64·3 (57·9-70·7)	63·8 (58·2-69·3)	70·0 (69·0-71·0)	70·3 (69·4-71·2)	70·9 (70·0-71·7)
Taiwan (Province of China)	68·7 (68·3-69·2)	70·7 (70·3-71·2)	71·6 (71·1-72·0)	73·0 (72·6-73·4)	73·9 (73·5-74·4)	74·7 (74·2-75·1)	76·1 (75·7-76·5)	77·5 (77·0-77·9)	79·0 (78·6-79·4)	79·7 (78·5-81·0)
Southeast Asia	55·5 (54·4-56·4)	57·4 (55·9-58·9)	61·4 (61·1-61·9)	63·6 (63·2-63·9)	65·3 (64·9-65·7)	66·7 (66·4-67·0)	67·9 (67·5-68·2)	69·3 (68·9-69·7)	70·9 (70·5-71·3)	72·7 (72·2-73·1)
Cambodia	44·3 (41·7-46·9)	22·9 (14·4-44·6)	52·6 (50·7-54·5)	54·2 (52·5-55·9)	57·4 (56·5-58·3)	58·2 (57·5-59·0)	59·5 (58·6-60·4)	63·0 (62·1-63·8)	66·2 (65·4-67·1)	68·7 (67·8-69·7)
Indonesia	53·7 (53·0-54·4)	56·5 (55·8-57·1)	59·3 (58·7-59·8)	61·8 (61·3-62·2)	63·6 (63·2-64·0)	65·7 (65·4-66·1)	67·2 (66·9-67·6)	68·5 (68·2-68·9)	69·9 (69·4-70·3)	71·7 (71·0-72·2)
Laos	42·1 (36·0-47·1)	45·6 (41·1-49·7)	47·8 (44·8-50·6)	50·7 (48·9-52·4)	52·4 (51·0-53·8)	54·5 (53·1-55·7)	57·0 (55·8-58·2)	60·3 (59·1-61·4)	63·8 (62·6-64·9)	67·2 (65·6-68·6)
Malaysia	64·4 (64·1-64·8)	66·8 (66·4-67·1)	68·8 (68·4-69·2)	70·4 (70·2-70·7)	71·6 (71·3-71·8)	72·3 (72·1-72·5)	73·0 (72·8-73·2)	73·8 (73·6-74·0)	74·4 (74·2-74·7)	75·6 (75·1-76·0)
Maldives	39·2 (36·4-41·9)	44·3 (41·9-46·9)	48·3 (46·4-50·5)	55·4 (54·6-56·3)	62·5 (61·7-63·4)	67·8 (66·8-68·9)	72·1 (71·3-72·8)	75·7 (75·0-76·5)	77·8 (77·1-78·5)	79·3 (77·5-81·1)
Mauritius	60·8 (60·4-61·1)	62·7 (62·3-63·1)	65·8 (65·4-66·2)	67·5 (67·2-68·0)	69·3 (68·9-69·7)	70·3 (69·9-70·7)	71·4 (71·0-71·8)	72·3 (71·9-72·7)	73·3 (72·9-73·8)	74·6 (73·2-75·9)
Myanmar	48·4 (45·7-50·8)	52·5 (50·2-54·7)	55·4 (53·4-57·3)	57·3 (55·5-59·0)	58·6 (57·0-60·1)	59·5 (57·8-61·0)	61·1 (59·3-62·7)	63·6 (61·6-65·0)	66·8 (65·0-68·1)	70·0 (68·8-71·1)
Philippines	62·9 (62·2-63·8)	63·7 (63·0-64·4)	64·7 (64·0-65·4)	65·4 (64·8-66·0)	67·0 (66·4-67·7)	68·0 (67·4-68·6)	68·1 (67·5-68·7)	68·2 (67·5-68·8)	68·9 (68·2-69·5)	70·1 (68·7-71·5)
Sri Lanka	64·7 (64·1-65·4)	64·7 (64·0-65·3)	68·7 (68·0-69·3)	70·1 (69·4-70·8)	71·3 (70·5-72·1)	72·0 (71·1-72·9)	72·5 (71·8-73·3)	73·9 (73·1-74·7)	74·4 (73·3-75·5)	77·4 (75·2-79·7)
Seychelles	65·4 (64·2-66·4)	67·9 (67·0-68·8)	70·2 (69·4-71·1)	69·7 (69·1-70·5)	69·0 (68·3-69·8)	69·9 (69·1-70·7)	70·9 (70·2-71·5)	72·0 (71·4-72·6)	73·2 (72·6-73·9)	73·7 (72·3-74·9)
Thailand	62·3 (61·7-63·0)	64·9 (64·3-65·6)	66·2 (65·7-66·9)	68·7 (68·2-69·3)	70·6 (70·0-71·1)	70·8 (70·1-71·4)	71·9 (71·2-72·5)	74·1 (73·6-74·6)	76·5 (76·0-77·0)	77·7 (76·7-78·8)
Timor-Leste	43·7 (39·3-47·9)	42·9 (39·6-46·4)	46·8 (44·0-49·8)	54·3 (52·2-56·8)	57·9 (55·8-60·0)	60·8 (59·0-62·6)	63·6 (62·0-65·3)	67·2 (65·6-68·9)	71·1 (69·0-73·1)	72·7 (70·4-75·0)
Vietnam	53·6 (47·5-59·9)	61·2 (59·1-63·3)	64·5 (63·0-65·9)	66·6 (65·1-68·1)	68·2 (66·8-69·7)	69·5 (68·1-71·2)	70·8 (69·5-72·2)	71·8 (70·5-73·2)	73·1 (71·9-74·6)	74·6 (73·4-75·6)
Oceania	56·3 (54·5-58·2)	57·2 (55·5-59·0)	57·8 (56·0-59·4)	58·1 (56·4-59·8)	58·5 (56·6-60·1)	59·1 (57·3-61·0)	59·6 (57·5-61·5)	59·6 (57·3-61·7)	60·5 (58·0-62·7)	62·2 (59·8-64·4)
American Samoa	66·9 (65·7-68·1)	68·9 (67·4-70·3)	69·7 (68·2-71·0)	70·2 (68·9-71·3)	70·5 (69·4-71·6)	70·7 (69·4-72·1)	71·5 (70·4-72·7)	71·6 (70·4-72·7)	72·4 (71·2-73·5)	72·4 (70·5-74·3)
Federated States of Micronesia	59·7 (57·2-62·7)	60·6 (58·2-63·2)	61·6 (59·5-64·0)	62·4 (60·3-64·6)	63·2 (60·9-65·3)	64·2 (62·0-66·4)	64·7 (62·4-67·0)	65·6 (63·2-67·9)	65·3 (62·4-67·9)	65·5 (62·5-68·3)
Fiji	64·2 (62·5-66·0)	64·0 (62·5-65·8)	64·6 (63·0-66·2)	65·3 (63·7-66·7)	65·2 (62·5-67·9)	64·5 (62·3-66·6)	64·2 (62·5-65·7)	64·8 (63·3-66·3)	65·2 (63·3-67·1)	65·4 (62·4-68·6)
Guam	67·5 (66·5-68·7)	68·4 (67·3-69·5)	71·3 (70·2-72·3)	72·9 (71·8-74·0)	73·1 (72·1-74·0)	73·3 (72·2-74·4)	73·9 (72·9-74·7)	73·5 (72·6-74·3)	72·1 (71·2-73·2)	72·3 (70·7-73·9)
Kiribati	54·8 (52·0-57·6)	57·7 (54·6-60·6)	58·7 (55·0-61·6)	57·9 (54·8-60·4)	58·1 (56·4-59·8)	58·5 (57·2-59·7)	59·4 (58·1-60·6)	60·1 (58·4-61·7)	60·7 (58·5-62·7)	61·8 (59·1-64·1)
Marshall Islands	60·4 (56·8-63·6)	62·4 (58·9-65·3)	63·9 (61·2-66·4)	64·3 (62·4-65·9)	64·5 (63·2-65·6)	64·4 (63·2-65·4)	63·2 (61·7-64·7)	63·1 (61·5-64·5)	63·6 (61·9-65·5)	64·9 (62·9-67·0)
Northern Mariana Islands	69·2 (66·9-71·6)	70·7 (68·3-72·9)	71·9 (69·6-74·1)	72·5 (70·3-74·7)	73·6 (71·6-75·9)	75·2 (73·4-77·0)	76·1 (74·6-77·8)	76·1 (74·6-77·7)	75·9 (74·2-77·4)	75·5 (73·8-77·1)
Papua New Guinea	53·9 (51·5-56·5)	55·0 (52·7-57·5)	55·2 (52·9-57·5)	55·4 (53·3-57·7)	55·9 (53·6-58·2)	56·9 (54·5-59·4)	57·6 (54·9-60·2)	57·6 (54·7-60·3)	58·7 (55·6-61·4)	60·8 (57·9-63·6)
Samoa	65·0 (63·6-66·4)	65·5 (64·1-66·7)	67·4 (65·9-68·6)	68·0 (66·2-69·5)	68·9 (67·2-70·5)	69·6 (67·9-71·2)	70·3 (68·6-72·0)	71·1 (69·6-72·7)	71·4 (70·0-73·1)	71·9 (70·3-73·6)
Solomon Islands	54·4 (51·9-57·2)	54·4 (51·9-57·1)	58·7 (56·3-61·2)	59·7 (57·4-62·2)	60·1 (57·9-62·5)	60·9 (58·7-63·1)	61·4 (59·3-63·7)	61·0 (58·8-63·3)	61·7 (59·3-64·0)	63·0 (60·4-65·5)
Tonga	65·4 (63·5-67·5)	65·9 (64·0-67·9)	65·9 (63·9-67·9)	66·8 (64·9-68·8)	67·9 (66·0-69·7)	68·4 (66·4-70·1)	68·7 (66·9-70·3)	69·4 (67·8-70·9)	69·8 (68·0-71·5)	70·4 (68·5-72·3)
Vanuatu	56·7 (53·7-59·4)	58·5 (55·7-61·1)	60·1 (57·8-62·4)	61·1 (59·1-63·2)	61·7 (59·7-63·7)	62·1 (60·1-64·2)	62·3 (60·3-64·2)	62·2 (59·9-64·4)	63·0 (60·4-65·2)	63·9 (61·2-66·3)
North Africa and Middle East	54·9 (54·0-55·8)	58·5 (57·9-59·1)	61·1 (60·4-61·8)	63·5 (62·6-64·4)	66·9 (66·5-67·3)	68·1 (67·8-68·4)	69·7 (69·4-70·1)	70·9 (70·4-71·3)	72·3 (71·9-72·8)	73·2 (72·6-73·8)
North Africa and Middle East	54·9 (54·0-55·8)	58·5 (57·9-59·1)	61·1 (60·4-61·8)	63·5 (62·6-64·4)	66·9 (66·5-67·3)	68·1 (67·8-68·4)	69·7 (69·4-70·1)	70·9 (70·4-71·3)	72·3 (71·9-72·8)	73·2 (72·6-73·8)
Afghanistan	45·8 (42·2-49·2)	46·3 (43·4-49·0)	43·3 (39·6-47·1)	41·4 (35·6-48·1)	51·6 (50·3-52·9)	51·0 (49·8-52·4)	51·6 (50·4-52·9)	53·8 (52·5-55·1)	56·2 (54·8-57·4)	57·9 (56·3-59·5)
Algeria	57·5 (55·8-59·2)	59·5 (58·1-60·9)	62·1 (61·1-63·2)	68·0 (67·3-68·9)	71·0 (70·2-71·9)	72·3 (71·3-73·2)	73·8 (72·9-74·8)	75·4 (74·4-76·2)	76·5 (76·0-77·1)	77·4 (76·5-78·2)
Bahrain	64·5 (62·0-66·9)	67·4 (65·3-69·5)	69·7 (68·4-71·3)	70·4 (69·3-71·6)	69·6 (68·4-70·7)	70·2 (69·0-71·3)	71·4 (70·2-72·5)	73·5 (72·3-74·6)	75·9 (74·8-77·0)	76·8 (75·0-78·5)

Appendix Table 13A. Estimates of life expectancy at birth by location, both sexes combined, 1970, 1975, 1980, 1985, 1990, 1995, 2000, 2005, 2010, 2016

Location	1970	1975	1980	1985	1990	1995	2000	2005	2010	2016
Egypt	51·5 (50·1-52·8)	54·7 (53·5-56·0)	58·8 (57·7-60·0)	61·1 (60·1-61·9)	65·3 (64·5-66·1)	67·7 (66·9-68·4)	69·9 (69·2-70·5)	70·2 (69·6-70·9)	70·5 (69·8-71·1)	72·1 (70·7-73·5)
Iran	55·4 (51·9-58·9)	64·4 (62·1-66·7)	66·3 (64·1-68·4)	68·1 (65·8-70·4)	67·9 (65·7-70·2)	70·7 (69·3-72·2)	71·4 (70·1-72·8)	72·4 (70·5-74·1)	74·4 (72·5-76·2)	75·9 (74·2-77·6)
Iraq	60·5 (59·0-62·1)	61·0 (59·7-62·3)	61·1 (57·5-64·7)	60·1 (55·5-65·1)	66·1 (64·4-67·7)	65·2 (63·8-66·6)	66·0 (64·6-67·3)	64·7 (62·7-66·9)	67·2 (65·2-69·3)	67·6 (65·1-70·3)
Jordan	62·0 (60·1-63·7)	64·6 (62·8-66·1)	66·7 (64·9-68·3)	69·2 (67·9-70·6)	71·8 (70·6-72·9)	72·6 (71·1-74·1)	73·1 (71·3-74·9)	73·1 (71·2-74·9)	75·7 (73·8-77·5)	76·3 (74·1-78·5)
Kuwait	69·2 (67·8-70·6)	69·7 (68·5-70·8)	71·6 (70·3-72·9)	73·7 (72·5-74·8)	75·6 (74·0-77·3)	76·1 (74·9-77·3)	76·4 (75·4-77·6)	77·1 (75·8-78·2)	78·2 (77·0-79·3)	79·7 (77·4-82·1)
Lebanon	66·6 (63·9-69·1)	59·3 (52·6-67·0)	52·5 (43·4-64·9)	64·2 (59·3-69·0)	69·1 (67·6-70·9)	73·5 (72·5-74·7)	76·5 (75·2-77·5)	78·3 (77·3-79·3)	79·8 (78·9-80·7)	80·1 (78·9-81·1)
Libya	66·0 (64·6-67·4)	69·0 (67·7-70·1)	71·3 (70·3-72·4)	72·5 (71·5-73·5)	73·0 (72·1-74·0)	74·0 (73·0-75·0)	74·3 (73·3-75·3)	75·2 (74·1-76·2)	76·1 (74·9-77·1)	75·1 (73·7-76·4)
Morocco	55·3 (54·3-56·1)	57·8 (57·0-58·6)	61·0 (60·2-61·8)	64·6 (63·9-65·3)	67·2 (66·5-67·9)	69·0 (68·2-69·7)	70·9 (70·1-71·6)	72·2 (71·4-73·0)	73·6 (72·7-74·5)	75·0 (74·1-75·9)
Palestine	62·3 (58·7-66·0)	67·1 (64·9-69·2)	69·0 (67·1-70·9)	70·7 (69·0-72·3)	71·0 (69·6-72·4)	72·0 (71·1-73·0)	71·4 (70·8-72·0)	71·8 (71·4-72·1)	72·2 (71·9-72·5)	71·8 (71·2-72·4)
Oman	50·7 (48·1-53·1)	56·3 (54·3-58·3)	62·6 (61·0-64·3)	67·6 (66·4-69·0)	70·6 (69·6-71·7)	72·7 (71·8-73·6)	74·4 (73·6-75·2)	75·8 (75·0-76·5)	75·7 (75·0-76·6)	77·0 (76·3-77·7)
Qatar	66·6 (62·7-70·4)	67·7 (64·0-71·3)	69·5 (66·6-72·6)	72·7 (70·6-74·5)	75·0 (73·0-76·8)	74·6 (72·8-76·4)	75·0 (73·1-76·9)	76·4 (74·6-78·3)	79·0 (77·3-80·7)	79·9 (77·1-82·5)
Saudi Arabia	64·5 (61·8-67·1)	68·6 (66·7-70·4)	71·0 (69·6-72·5)	71·9 (70·7-73·2)	71·9 (70·7-73·1)	72·7 (71·6-73·7)	72·8 (72·1-73·6)	73·7 (73·2-74·3)	75·0 (74·4-75·5)	77·1 (76·4-77·8)
Sudan	52·5 (51·2-53·8)	53·5 (52·4-54·6)	54·9 (53·6-56·1)	50·5 (46·8-54·5)	58·5 (57·6-59·5)	60·4 (59·5-61·3)	62·6 (61·7-63·4)	64·9 (64·1-65·6)	66·3 (65·4-67·1)	68·3 (66·9-69·4)
Syria	64·5 (63·6-65·4)	67·0 (66·2-67·7)	68·7 (68·0-69·4)	70·0 (69·3-70·7)	70·9 (70·2-71·6)	72·1 (71·1-72·8)	73·2 (72·6-73·9)	74·8 (74·2-75·3)	76·0 (75·5-76·6)	68·0 (62·2-74·2)
Tunisia	57·5 (56·6-58·5)	62·1 (61·2-62·9)	65·5 (64·6-66·3)	69·8 (68·8-70·6)	71·6 (70·8-72·4)	72·8 (71·9-73·6)	74·9 (73·8-75·9)	76·0 (74·9-77·2)	76·8 (75·4-78·3)	77·5 (75·9-79·1)
Turkey	56·9 (54·6-59·5)	60·0 (58·2-61·9)	64·0 (62·5-65·4)	67·9 (66·6-69·1)	70·1 (69·2-71·0)	70·8 (70·1-71·6)	74·1 (73·4-74·8)	76·7 (76·0-77·4)	78·3 (77·3-79·2)	79·2 (77·6-80·8)
United Arab Emirates	65·1 (61·6-68·7)	68·2 (64·6-71·4)	70·6 (67·8-73·6)	72·2 (69·6-74·7)	72·5 (70·1-74·6)	73·1 (71·2-75·0)	74·3 (72·9-75·7)	76·0 (75·0-76·7)	75·6 (74·3-77·1)	75·5 (73·5-77·4)
Yemen	42·0 (40·2-43·6)	48·6 (47·3-50·0)	53·2 (51·9-54·6)	56·6 (55·4-57·9)	58·7 (57·5-59·9)	60·5 (59·3-61·7)	62·6 (61·5-63·8)	65·0 (63·9-66·1)	67·5 (66·2-68·7)	66·7 (65·2-68·0)
South Asia	49·0 (48·1-49·9)	52·0 (51·4-52·7)	54·7 (54·1-55·3)	56·9 (56·5-57·4)	59·1 (58·7-59·5)	60·9 (60·5-61·3)	62·5 (62·1-62·9)	64·3 (63·8-64·7)	66·4 (65·9-66·8)	68·8 (68·2-69·2)
South Asia	49·0 (48·1-49·9)	52·0 (51·4-52·7)	54·7 (54·1-55·3)	56·9 (56·5-57·4)	59·1 (58·7-59·5)	60·9 (60·5-61·3)	62·5 (62·1-62·9)	64·3 (63·8-64·7)	66·4 (65·9-66·8)	68·8 (68·2-69·2)
Bangladesh	43·8 (40·2-48·1)	52·4 (51·1-53·9)	53·8 (52·8-54·9)	55·3 (54·4-56·1)	58·1 (57·3-59·0)	61·7 (60·8-62·5)	65·4 (64·7-66·2)	67·9 (67·3-68·6)	70·3 (69·5-71·0)	72·6 (71·4-73·7)
Bhutan	48·8 (45·9-51·5)	51·2 (48·7-53·6)	53·3 (51·1-55·4)	56·4 (54·5-58·2)	59·9 (58·3-61·5)	62·9 (61·5-64·3)	65·6 (64·2-66·9)	69·5 (68·2-70·6)	71·9 (70·5-73·2)	73·8 (72·3-75·3)
India	49·0 (48·0-50·0)	51·5 (50·8-52·2)	54·4 (53·8-55·0)	56·8 (56·3-57·3)	59·0 (58·5-59·4)	60·7 (60·3-61·2)	62·3 (61·8-62·7)	64·2 (63·6-64·7)	66·1 (65·5-66·7)	68·5 (67·9-69·1)
Nepal	49·0 (48·0-50·0)	50·6 (49·6-51·6)	52·4 (51·6-53·4)	54·9 (54·0-55·8)	57·7 (56·9-58·5)	60·6 (59·9-61·3)	63·7 (63·0-64·4)	66·0 (65·3-66·7)	68·3 (67·5-69·1)	70·9 (70·1-71·7)
Pakistan	56·9 (55·4-58·4)	58·6 (57·3-60·0)	60·3 (59·1-61·4)	61·7 (60·9-62·5)	62·4 (61·7-63·2)	62·0 (61·2-62·8)	62·6 (61·8-63·5)	62·5 (61·3-63·7)	65·3 (64·0-66·5)	67·6 (66·1-69·1)
Sub-Saharan Africa	49·4 (48·4-50·4)	51·0 (50·2-51·7)	52·4 (51·9-53·0)	53·4 (52·9-53·9)	53·7 (53·3-54·1)	53·4 (52·9-53·8)	52·8 (52·4-53·3)	54·7 (54·2-55·1)	58·2 (57·7-58·8)	62·9 (62·1-63·5)
Southern Sub-Saharan Africa	57·1 (55·7-58·5)	58·7 (57·7-59·8)	59·9 (59·1-60·7)	62·1 (61·5-62·7)	63·8 (63·0-64·5)	61·6 (60·4-62·8)	53·2 (52·0-54·4)	49·7 (48·7-50·7)	53·3 (52·5-54·1)	61·6 (60·7-62·6)
Botswana	56·6 (55·3-58·0)	59·6 (58·5-60·9)	61·7 (60·6-63·0)	63·9 (62·7-65·3)	64·4 (61·9-67·1)	56·2 (50·4-63·8)	46·6 (41·1-53·7)	47·7 (43·6-52·5)	56·8 (52·9-61·0)	65·2 (61·7-71·4)
Lesotho	56·0 (53·8-58·1)	57·2 (55·0-59·5)	58·9 (56·7-61·1)	59·8 (57·9-61·8)	60·4 (59·0-62·0)	57·7 (55·5-59·8)	48·8 (45·8-51·9)	43·3 (40·3-46·3)	46·5 (44·1-48·7)	50·3 (47·6-52·7)
Namibia	58·3 (56·4-60·1)	59·1 (57·7-60·6)	59·4 (58·1-60·5)	60·9 (59·9-61·9)	61·5 (60·5-62·5)	59·9 (57·9-61·8)	53·6 (50·5-57·1)	51·6 (48·6-54·8)	58·6 (56·1-61·4)	64·8 (62·1-68·1)
South Africa	57·2 (55·3-59·0)	58·7 (57·4-60·1)	59·8 (58·8-60·9)	61·9 (61·1-62·7)	64·3 (63·7-65·0)	64·2 (63·4-64·9)	55·4 (54·1-56·4)	51·1 (50·0-52·3)	54·3 (53·4-55·3)	62·4 (61·2-63·5)
Swaziland	51·4 (47·6-55·2)	54·9 (52·2-57·8)	57·8 (55·4-60·1)	60·2 (58·0-62·3)	62·4 (60·5-64·2)	59·3 (56·1-62·5)	47·4 (43·2-52·0)	42·3 (39·2-45·9)	46·9 (44·0-49·9)	57·4 (53·9-61·6)
Zimbabwe	57·6 (56·0-59·2)	59·0 (57·7-60·4)	60·4 (59·2-61·6)	63·1 (62·0-64·1)	61·6 (58·7-65·2)	53·5 (49·3-58·4)	46·6 (43·3-50·5)	45·3 (42·8-48·1)	49·5 (47·5-51·3)	59·2 (57·1-61·4)
Western Sub-Saharan Africa	48·9 (47·6-50·1)	50·9 (49·8-52·0)	52·9 (52·0-53·8)	54·2 (53·4-54·9)	54·4 (53·6-55·1)	54·4 (53·5-55·2)	54·8 (53·8-55·7)	56·6 (55·7-57·4)	59·6 (58·7-60·5)	63·3 (62·3-64·2)
Benin	47·2 (46·1-48·3)	49·6 (48·5-50·7)	51·6 (50·6-52·5)	53·8 (52·8-54·7)	55·5 (54·6-56·5)	56·5 (55·5-57·5)	57·6 (56·5-58·7)	59·5 (58·4-60·6)	61·8 (60·6-63·0)	64·4 (63·1-65·7)
Burkina Faso	44·4 (42·8-45·8)	46·1 (45·0-47·2)	48·2 (47·4-49·1)	50·1 (49·3-50·9)	50·7 (49·6-51·8)	50·7 (49·4-52·2)	52·2 (51·1-53·3)	55·1 (54·1-56·1)	57·9 (56·9-58·9)	60·8 (59·5-61·9)
Cameroon	51·7 (50·5-53·0)	53·6 (52·4-54·8)	55·6 (54·4-56·7)	57·8 (56·8-58·9)	58·4 (57·3-59·6)	56·5 (54·9-58·1)	54·4 (52·9-56·1)	54·9 (53·2-56·6)	57·2 (55·4-59·1)	60·1 (57·8-62·4)
Cape Verde	57·6 (55·7-59·6)	61·7 (60·1-63·4)	65·4 (64·3-66·4)	65·8 (65·0-66·7)	68·0 (67·1-69·0)	67·3 (65·6-69·2)	69·8 (68·0-71·8)	71·0 (69·4-72·9)	72·3 (71·2-73·5)	73·6 (72·3-75·1)
Chad	49·2 (47·3-51·0)	51·2 (49·8-52·7)	51·2 (49·9-52·5)	52·4 (51·4-53·4)	53·3 (52·2-54·3)	53·5 (52·4-54·6)	52·1 (50·9-53·5)	53·8 (52·5-55·3)	56·5 (55·0-57·9)	59·8 (58·2-61·4)
Cote d'Ivoire	50·9 (49·9-51·9)	53·5 (52·6-54·4)	56·0 (55·1-56·8)	56·3 (55·4-57·2)	54·8 (53·7-55·7)	52·5 (51·2-53·8)	52·4 (51·1-53·7)	53·5 (52·1-54·9)	56·2 (54·7-57·7)	59·9 (58·4-61·3)
The Gambia	55·0 (53·0-56·9)	56·8 (55·1-58·6)	58·7 (57·1-60·3)	60·4 (59·0-61·8)	61·6 (60·3-62·9)	62·7 (61·5-63·9)	63·3 (62·2-64·5)	64·3 (63·1-65·4)	65·5 (64·4-66·7)	67·3 (66·1-68·7)
Ghana	55·2 (53·9-56·5)	56·7 (55·5-58·0)	57·5 (56·3-58·9)	57·9 (56·7-59·2)	58·8 (57·6-60·0)	59·5 (58·2-60·8)	59·3 (58·0-60·7)	60·1 (58·9-61·4)	62·2 (61·0-63·4)	66·0 (64·7-67·5)
Guinea	45·1 (42·3-47·7)	46·1 (44·0-48·2)	48·1 (46·5-49·7)	49·8 (48·5-51·1)	51·9 (50·7-53·1)	53·3 (52·2-54·5)	54·7 (53·5-55·8)	56·2 (54·8-57·5)	58·0 (56·4-59·6)	60·7 (58·8-62·5)
Guinea-Bissau	40·1 (36·9-43·3)	44·9 (42·7-47·0)	46·6 (44·7-48·5)	47·5 (46·0-49·3)	49·2 (47·7-50·6)	50·8 (49·5-52·2)	52·2 (50·9-53·6)	53·1 (51·6-54·7)	55·6 (54·1-57·1)	58·8 (57·2-60·1)
Liberia	47·5 (45·7-49·4)	49·8 (48·3-51·4)	51·5 (50·2-52·8)	52·3 (51·1-53·5)	49·8 (48·7-51·0)	50·3 (49·3-51·3)	54·4 (53·4-55·6)	58·1 (57·1-59·3)	60·8 (59·8-61·7)	64·4 (63·4-65·3)
Mali	39·8 (38·0-41·9)	41·6 (40·0-43·3)	44·9 (43·5-46·1)	47·2 (46·1-48·4)	49·3 (48·3-50·4)	51·2 (50·1-52·4)	53·3 (52·1-54·6)	56·5 (55·3-57·8)	59·4 (57·8-61·1)	61·8 (59·6-64·2)
Mauritania	54·2 (52·0-56·4)	54·7 (52·7-56·9)	57·1 (55·5-58·9)	58·6 (57·4-59·9)	59·9 (58·9-61·1)	61·5 (60·5-62·6)	63·6 (62·5-64·7)	65·6 (64·2-67·0)	68·1 (66·5-69·9)	70·2 (68·3-72·4)
Niger	43·6 (41·6-45·7)	43·3 (41·6-44·8)	43·9 (42·4-45·2)	44·7 (43·4-46·0)	47·1 (45·8-48·3)	49·0 (47·7-50·3)	52·2 (51·0-53·4)	55·6 (54·1-57·0)	58·8 (57·2-60·5)	61·7 (59·7-63·7)
Nigeria	50·1 (47·8-52·2)	52·5 (50·7-54·4)	54·6 (53·0-56·2)	55·6 (54·1-57·0)	54·9 (53·3-56·3)	54·7 (52·9-56·5)	54·9 (53·1-56·9)	56·8 (55·2-58·6)	60·5 (58·8-62·3)	65·0 (63·2-67·0)
Sao Tome and Principe	58·4 (57·6-59·3)	61·6 (60·6-62·5)	63·0 (62·2-63·8)	62·4 (61·5-63·3)	63·3 (62·1-64·5)	63·8 (62·6-65·0)	64·5 (63·5-65·5)	66·1 (65·1-67·1)	68·9 (67·9-70·0)	70·6 (69·1-72·1)
Senegal	47·5 (46·6-48·5)	49·3 (48·3-50·2)	52·3 (51·4-53·2)	55·5 (54·5-56·3)	57·5 (56·8-58·3)	58·4 (57·6-59·2)	59·7 (58·9-60·5)	61·7 (60·9-62·6)	64·0 (63·3-64·8)	66·2 (65·3-67·1)
Sierra Leone	45·6 (42·7-48·0)	47·2 (44·8-49·4)	48·2 (46·3-49·9)	49·2 (47·8-50·5)	50·5 (49·3-51·7)	49·8 (48·6-51·1)	50·9 (49·8-52·0)	52·4 (51·3-53·6)	55·0 (53·8-56·3)	59·1 (57·7-60·6)
Togo	51·9 (50·3-53·3)	54·0 (52·7-55·3)	55·9 (54·8-57·0)	56·7 (55·6-57·7)	57·4 (56·4-58·4)	57·2 (56·1-58·3)	56·3 (54·8-58·0)	56·4 (54·9-58·1)	58·7 (57·4-60·1)	62·6 (61·3-63·9)
Eastern Sub-Saharan Africa	48·3 (47·3-49·3)	49·4 (48·6-50·3)	50·6 (49·8-51·4)	50·9 (50·3-51·6)	51·2 (50·7-51·7)	51·0 (50·4-51·6)	51·5 (50·9-52·1)	54·8 (54·3-55·4)	59·0 (58·2-59·6)	63·4 (62·5-64·2)
Burundi	44·7 (42·6-46·9)	45·2 (43·4-47·2)	46·3 (44·5-48·2)	47·6 (45·9-49·5)	48·0 (46·1-49·8)	47·0 (45·2-48·7)	47·9 (46·2-49·5)	53·1 (51·6-54·5)	57·7 (56·0-59·5)	60·3 (57·9-62·7)
Comoros	51·1 (48·4-53·7)	52·9 (50·8-54·9)	54·5 (52·6-56·3)	56·6 (55·0-58·2)	58·6 (57·4-60·2)	60·0 (58·8-61·2)	61·5 (60·3-62·8)	64·3 (63·1-65·5)	65·9 (64·6-67·4)	67·5 (65·9-69·2)

Appendix Table 13A. Estimates of life expectancy at birth by location, both sexes combined, 1970, 1975, 1980, 1985, 1990, 1995, 2000, 2005, 2010, 2016

Location	1970	1975	1980	1985	1990	1995	2000	2005	2010	2016
Djibouti	59·7 (58·0-61·5)	60·8 (59·3-62·3)	61·1 (59·7-62·4)	61·0 (59·8-62·2)	61·2 (60·2-62·1)	61·3 (60·1-62·4)	61·3 (59·7-63·3)	62·0 (59·6-64·1)	63·6 (61·5-65·8)	66·7 (64·7-68·9)
Eritrea	45·3 (43·2-47·2)	47·0 (45·6-48·5)	48·5 (47·2-49·7)	49·8 (48·6-51·0)	52·3 (51·1-53·6)	56·5 (55·3-57·8)	58·1 (56·8-59·4)	60·4 (59·0-61·9)	61·6 (60·1-63·2)	63·7 (62·1-65·3)
Ethiopia	43·7 (41·9-45·5)	43·6 (41·9-45·3)	44·6 (43·0-46·2)	45·8 (44·6-47·2)	46·6 (45·7-47·6)	49·9 (48·8-50·9)	51·1 (50·1-52·2)	56·1 (54·8-57·3)	61·3 (59·8-62·8)	65·6 (63·9-67·5)
Kenya	57·9 (57·3-58·6)	59·8 (59·2-60·5)	61·7 (61·0-62·3)	62·6 (62·0-63·2)	61·4 (60·8-62·0)	57·2 (56·5-58·0)	55·2 (54·5-55·9)	57·4 (56·8-58·0)	62·8 (62·1-63·3)	66·8 (66·1-67·6)
Madagascar	50·7 (49·0-52·3)	53·1 (51·6-54·6)	54·8 (53·3-56·2)	54·2 (52·9-55·5)	55·6 (54·5-56·7)	56·9 (55·7-57·9)	58·5 (57·2-59·6)	60·1 (58·7-61·4)	60·8 (59·0-62·9)	62·7 (60·4-65·2)
Malawi	43·5 (41·1-45·6)	46·4 (44·6-48·2)	49·4 (47·8-50·9)	50·0 (48·3-51·5)	48·7 (46·3-51·0)	46·0 (43·3-49·0)	44·7 (42·1-47·8)	47·6 (45·3-50·5)	53·0 (51·0-55·3)	60·2 (58·0-62·6)
Mozambique	47·6 (45·5-49·6)	49·5 (48·0-51·1)	50·3 (49·1-51·4)	45·1 (42·3-48·2)	50·7 (49·8-51·7)	51·3 (49·7-52·7)	51·4 (49·2-53·6)	52·4 (49·8-55·0)	54·5 (52·2-56·9)	60·0 (57·9-61·9)
Rwanda	46·6 (44·6-48·5)	47·3 (45·5-49·0)	49·9 (48·3-51·6)	52·3 (50·9-53·7)	49·1 (47·7-50·6)	44·6 (42·8-46·3)	49·4 (47·9-51·0)	57·8 (56·5-59·2)	64·7 (63·4-66·1)	67·8 (66·1-69·4)
Somalia	49·7 (47·0-52·2)	47·9 (45·1-50·5)	50·7 (48·5-52·8)	50·2 (48·2-52·0)	50·6 (48·7-52·3)	51·3 (49·7-53·2)	52·0 (50·2-53·8)	53·8 (52·0-55·7)	53·1 (50·7-55·3)	57·1 (54·9-59·2)
South Sudan	49·1 (44·1-53·5)	50·2 (46·0-54·0)	51·2 (48·1-54·3)	51·4 (48·9-54·0)	51·5 (49·3-53·9)	53·3 (51·0-55·8)	54·5 (52·1-57·2)	56·6 (53·9-59·5)	58·1 (55·4-61·2)	59·7 (57·0-62·6)
Tanzania	52·3 (50·9-53·6)	54·2 (53·1-55·4)	55·8 (54·7-56·9)	56·4 (55·3-57·3)	54·9 (53·6-56·1)	52·9 (51·1-54·8)	52·9 (51·2-55·0)	55·3 (53·7-57·3)	59·3 (57·8-61·0)	64·3 (62·8-65·9)
Uganda	50·3 (48·9-51·7)	51·3 (50·2-52·5)	49·4 (47·4-51·3)	49·9 (47·9-51·6)	49·1 (47·0-51·0)	46·9 (44·6-49·3)	48·7 (47·2-50·3)	52·8 (51·5-54·0)	56·8 (55·4-58·1)	62·2 (60·8-63·8)
Zambia	55·6 (52·3-59·0)	57·6 (54·9-60·6)	57·7 (55·5-60·0)	56·2 (54·2-58·4)	52·1 (50·0-54·1)	46·4 (43·7-48·9)	44·1 (41·7-46·7)	46·0 (43·8-48·2)	52·0 (49·9-53·9)	58·6 (56·0-61·3)
Central Sub-Saharan Africa	49·6 (46·7-52·3)	50·8 (48·5-53·2)	51·8 (50·2-53·4)	53·0 (51·8-54·3)	52·6 (51·6-53·7)	52·4 (51·3-53·4)	52·8 (51·6-53·9)	55·0 (54·0-56·1)	57·6 (56·5-58·8)	61·7 (60·1-63·2)
Angola	48·6 (44·6-52·9)	49·5 (45·8-53·3)	49·9 (46·8-53·1)	50·2 (47·5-53·3)	50·2 (47·6-53·0)	51·9 (49·3-55·0)	53·3 (50·6-56·5)	56·4 (53·5-59·4)	60·0 (57·0-63·1)	64·6 (61·0-67·8)
Central African Republic	48·2 (45·6-50·6)	48·4 (46·1-50·7)	49·6 (47·5-51·5)	49·9 (48·0-51·6)	48·1 (46·2-49·9)	46·1 (43·8-48·5)	44·4 (41·7-47·0)	44·9 (42·5-47·6)	47·4 (44·8-50·2)	50·2 (47·3-53·3)
Congo	48·8 (45·4-51·9)	51·0 (47·8-53·9)	53·0 (50·6-55·5)	55·0 (53·0-57·0)	54·0 (52·5-55·6)	52·6 (51·2-54·2)	52·8 (51·3-54·3)	56·5 (55·0-58·0)	60·3 (58·5-62·2)	62·9 (60·5-65·5)
Democratic Republic of the Congo	50·3 (46·8-53·6)	51·6 (48·6-54·3)	52·6 (50·4-54·8)	54·2 (52·7-55·7)	53·8 (52·3-55·3)	53·1 (51·6-54·5)	53·4 (51·8-54·8)	55·3 (54·0-56·6)	57·5 (56·0-58·9)	61·6 (59·6-63·3)
Equatorial Guinea	44·2 (40·0-48·4)	46·0 (42·7-49·5)	48·6 (45·8-51·7)	49·3 (46·6-52·2)	48·1 (45·4-51·1)	47·8 (44·8-51·0)	54·3 (50·8-58·1)	58·1 (53·9-62·8)	60·9 (56·9-65·5)	65·5 (61·2-70·0)
Gabon	52·0 (49·3-54·8)	55·1 (52·7-57·6)	57·7 (55·9-59·7)	59·1 (57·6-60·7)	59·4 (58·1-60·7)	59·6 (58·3-60·9)	59·1 (57·6-60·7)	59·9 (58·2-61·5)	62·8 (61·0-64·6)	66·5 (64·4-68·7)

Appendix Table 13B. Estimates of life expectancy at birth by location, males, 1970, 1975, 1980, 1985, 1990, 1995, 2000, 2005, 2010, 2016

Location	1970	1975	1980	1985	1990	1995	2000	2005	2010	2016
Global	56·3 (55·6-57·0)	58·2 (57·7-58·7)	59·9 (59·5-60·3)	61·4 (61·1-61·7)	62·7 (62·4-63·0)	63·2 (62·9-63·5)	64·4 (64·1-64·6)	65·9 (65·6-66·1)	67·8 (67·4-68·1)	69·8 (69·3-70·2)
High SDI	67·6 (67·4-67·9)	68·8 (68·6-69·0)	70·1 (70·0-70·2)	71·3 (71·2-71·4)	72·3 (72·2-72·4)	73·3 (73·2-73·4)	74·8 (74·7-74·9)	76·1 (76·0-76·1)	77·3 (77·2-77·4)	78·1 (77·8-78·3)
High-middle SDI	61·8 (61·1-62·4)	63·3 (62·8-63·9)	63·9 (63·4-64·5)	65·6 (65·1-66·0)	66·4 (65·9-66·9)	65·4 (64·9-66·0)	67·0 (66·5-67·6)	68·5 (68·0-69·0)	71·2 (70·8-71·7)	73·1 (72·0-74·1)
Middle SDI	57·3 (56·4-58·1)	59·9 (59·4-60·4)	61·4 (61·0-61·8)	63·1 (62·7-63·5)	64·4 (64·1-64·8)	65·5 (65·2-65·8)	66·6 (66·4-66·9)	68·0 (67·8-68·3)	69·6 (69·3-69·8)	71·2 (70·9-71·6)
Low-middle SDI	49·0 (47·9-50·0)	51·5 (50·6-52·4)	54·4 (53·8-55·1)	56·4 (55·8-56·9)	58·1 (57·6-58·6)	59·1 (58·6-59·5)	60·1 (59·7-60·6)	61·7 (61·2-62·1)	63·7 (63·2-64·2)	65·9 (65·3-66·5)
Low SDI	46·5 (45·0-48·0)	47·9 (47·0-49·0)	49·1 (48·3-50·0)	50·0 (49·1-50·9)	51·2 (50·6-51·8)	51·9 (51·3-52·5)	53·0 (52·4-53·5)	55·7 (55·1-56·3)	57·7 (56·7-58·7)	61·8 (60·9-62·7)
High-income	67·4 (67·2-67·7)	68·7 (68·5-68·9)	70·3 (70·2-70·4)	71·6 (71·5-71·7)	72·6 (72·5-72·8)	73·6 (73·5-73·7)	75·1 (75·0-75·2)	76·3 (76·2-76·4)	77·6 (77·5-77·7)	78·3 (78·0-78·5)
High-income North America	67·3 (67·1-67·4)	68·7 (68·6-68·8)	70·3 (70·2-70·4)	71·4 (71·3-71·5)	72·1 (72·0-72·3)	72·9 (72·8-73·0)	74·5 (74·4-74·6)	75·4 (75·3-75·5)	76·6 (76·5-76·7)	76·8 (76·5-77·0)
Canada	69·3 (68·9-69·7)	70·0 (69·7-70·4)	71·7 (71·4-72·1)	73·2 (72·8-73·5)	74·2 (73·9-74·5)	75·1 (74·8-75·4)	76·6 (76·3-76·9)	77·8 (77·6-78·1)	79·1 (78·8-79·4)	79·8 (79·3-80·2)
Greenland	60·4 (59·1-61·7)	59·8 (58·6-60·9)	58·1 (56·9-59·3)	57·8 (56·6-58·9)	57·7 (56·3-59·1)	61·3 (60·0-62·5)	63·0 (61·8-64·1)	64·2 (63·1-65·4)	66·6 (65·3-67·8)	67·8 (64·7-70·6)
United States	67·1 (66·9-67·2)	68·6 (68·5-68·7)	70·2 (70·0-70·3)	71·2 (71·1-71·3)	71·9 (71·8-72·1)	72·7 (72·5-72·8)	74·2 (74·1-74·3)	75·1 (75·0-75·2)	76·3 (76·2-76·4)	76·5 (76·2-76·7)
Australasia	67·9 (67·7-68·2)	69·2 (68·9-69·4)	70·9 (70·6-71·1)	72·2 (71·9-72·4)	73·8 (73·5-74·0)	75·2 (75·0-75·4)	76·9 (76·6-77·1)	78·5 (78·3-78·8)	79·7 (79·4-79·9)	80·3 (79·6-81·0)
Australia	67·8 (67·5-68·1)	69·2 (68·9-69·5)	71·1 (70·8-71·4)	72·4 (72·1-72·7)	74·0 (73·7-74·2)	75·4 (75·2-75·7)	77·0 (76·8-77·3)	78·7 (78·5-79·0)	79·8 (79·5-80·0)	80·5 (79·7-81·2)
New Zealand	68·4 (68·1-68·8)	69·0 (68·6-69·3)	70·0 (69·7-70·4)	71·0 (70·7-71·3)	72·7 (72·4-73·1)	74·2 (73·9-74·6)	76·0 (75·7-76·3)	77·8 (77·5-78·1)	79·0 (78·7-79·3)	79·5 (78·3-80·8)
High-income Asia Pacific	66·7 (65·3-68·0)	69·0 (68·1-69·9)	71·1 (70·6-71·6)	72·7 (72·3-73·1)	74·1 (73·7-74·5)	75·1 (74·7-75·5)	76·4 (76·1-76·7)	77·8 (77·5-78·1)	78·9 (78·6-79·2)	80·1 (79·1-80·9)
Brunei	65·0 (63·4-66·8)	68·2 (66·5-69·6)	68·7 (66·9-71·9)	70·5 (69·3-71·7)	71·7 (70·7-72·8)	72·5 (71·3-73·6)	73·8 (72·8-74·6)	74·7 (73·9-75·7)	74·9 (74·1-75·8)	74·6 (72·5-77·3)
Japan	69·6 (69·2-70·0)	71·7 (71·4-72·0)	73·5 (73·4-73·5)	74·8 (74·8-74·9)	75·9 (75·9-76·0)	76·5 (76·3-76·6)	77·5 (77·5-77·6)	78·5 (78·5-78·6)	79·5 (79·4-79·5)	80·8 (80·6-81·1)
Singapore	65·5 (63·9-67·0)	67·2 (65·7-68·7)	68·8 (67·3-70·4)	70·5 (68·7-72·1)	72·9 (71·3-74·4)	75·0 (73·5-76·3)	77·0 (75·4-78·6)	78·6 (77·2-80·0)	80·2 (78·7-81·6)	81·3 (78·8-83·7)
South Korea	58·0 (53·2-63·6)	60·3 (57·2-63·8)	63·0 (61·4-64·9)	65·3 (63·6-66·8)	67·7 (66·2-69·3)	70·2 (68·6-71·7)	72·3 (71·0-73·7)	75·0 (73·7-76·4)	76·8 (75·6-78·2)	77·7 (74·3-81·5)
Western Europe	68·5 (68·3-68·6)	69·4 (69·2-69·5)	70·6 (70·5-70·8)	71·9 (71·7-72·0)	72·9 (72·8-73·1)	74·0 (73·9-74·2)	75·5 (75·3-75·6)	76·9 (76·8-77·1)	78·3 (78·1-78·4)	79·2 (78·9-79·5)
Andorra	71·8 (68·1-75·5)	73·7 (70·8-77·4)	75·4 (72·9-78·3)	75·8 (73·5-78·8)	76·2 (74·0-78·8)	77·2 (74·9-79·4)	78·5 (76·4-80·4)	79·0 (77·4-80·8)	79·4 (77·6-81·3)	79·3 (77·4-81·9)
Austria	66·6 (66·3-66·9)	67·7 (67·4-68·0)	69·0 (68·7-69·3)	70·4 (70·1-70·7)	72·1 (71·9-72·4)	73·4 (73·1-73·7)	75·1 (74·8-75·3)	76·7 (76·4-76·9)	77·8 (77·6-78·1)	79·1 (78·5-79·9)
Belgium	67·8 (67·3-68·2)	68·8 (68·4-69·3)	69·8 (69·4-70·3)	71·1 (70·7-71·6)	72·6 (72·1-73·1)	73·6 (73·1-74·0)	74·6 (74·2-75·1)	76·2 (75·8-76·7)	77·4 (76·9-77·8)	78·4 (77·2-79·5)
Cyprus	68·4 (67·6-69·1)	69·4 (68·8-70·0)	71·2 (70·7-71·7)	72·2 (71·7-72·7)	72·9 (72·4-73·4)	73·5 (73·0-74·0)	74·4 (73·9-74·9)	75·5 (75·0-76·1)	77·4 (76·9-78·0)	78·1 (77·5-78·8)
Denmark	70·8 (70·0-71·6)	71·3 (70·5-72·1)	71·4 (70·5-72·2)	71·8 (71·0-72·6)	72·4 (71·7-73·3)	73·0 (72·3-73·8)	74·6 (73·8-75·3)	75·8 (75·1-76·6)	77·4 (76·7-78·2)	78·8 (77·3-80·1)
Finland	65·9 (65·6-66·3)	67·3 (67·0-67·7)	69·2 (68·8-69·6)	70·3 (70·0-70·7)	71·0 (70·6-71·3)	72·9 (72·5-73·3)	74·3 (73·9-74·6)	75·5 (75·1-75·9)	76·8 (76·4-77·1)	78·9 (78·0-79·8)
France	68·7 (68·4-69·2)	69·3 (68·9-69·7)	70·5 (70·1-70·9)	71·8 (71·4-72·2)	73·1 (72·7-73·5)	74·1 (73·8-74·4)	75·3 (75·0-75·7)	76·8 (76·5-77·2)	78·1 (77·8-78·5)	79·2 (78·6-79·8)
Germany	67·6 (66·9-68·3)	68·4 (67·7-69·0)	69·7 (69·1-70·3)	71·0 (70·4-71·7)	72·0 (71·4-72·7)	73·2 (72·6-73·9)	74·8 (74·3-75·4)	76·4 (75·8-77·0)	77·5 (76·9-78·1)	78·5 (77·4-79·5)
Greece	71·5 (71·1-71·9)	72·4 (72·0-72·7)	74·9 (74·5-75·3)	73·7 (73·4-74·0)	74·5 (74·1-74·9)	75·0 (74·7-75·4)	75·8 (75·5-76·2)	76·9 (76·5-77·2)	77·7 (77·4-78·0)	78·4 (77·5-79·5)
Iceland	71·1 (70·6-71·7)	72·2 (71·7-72·7)	73·7 (73·2-74·3)	74·5 (74·0-75·1)	75·7 (75·1-76·2)	76·4 (75·8-77·0)	77·9 (77·4-78·4)	79·4 (78·9-79·9)	80·2 (79·7-80·7)	80·6 (79·7-81·4)
Ireland	68·8 (68·1-69·5)	69·1 (68·4-69·7)	69·8 (69·1-70·4)	71·0 (70·3-71·6)	72·2 (71·6-72·9)	73·1 (72·5-73·7)	74·0 (73·4-74·6)	76·6 (76·0-77·2)	78·2 (77·6-78·8)	79·0 (77·5-80·4)
Israel	67·3 (65·4-69·4)	69·7 (68·4-71·0)	71·8 (70·5-73·1)	72·6 (71·4-73·9)	74·1 (73·0-75·3)	75·0 (73·7-76·2)	76·3 (75·0-77·7)	77·6 (76·4-78·7)	79·4 (78·2-80·5)	80·0 (77·8-82·3)
Italy	68·6 (68·3-68·9)	69·7 (69·4-70·0)	70·7 (70·4-71·0)	72·4 (72·1-72·7)	73·7 (73·4-74·0)	74·8 (74·5-75·1)	76·5 (76·3-76·8)	78·0 (77·8-78·3)	79·1 (78·9-79·4)	79·9 (79·1-80·7)
Luxembourg	66·4 (65·9-66·9)	67·7 (67·2-68·2)	69·1 (68·6-69·6)	70·3 (69·8-70·8)	71·8 (71·3-72·2)	73·5 (73·0-73·9)	75·2 (74·8-75·8)	77·3 (76·8-77·7)	79·2 (78·7-79·6)	80·3 (79·1-81·4)
Malta	67·3 (66·3-68·4)	68·6 (67·6-69·6)	69·5 (68·5-70·5)	71·6 (70·6-72·6)	73·7 (72·7-74·7)	74·8 (73·8-75·8)	75·1 (74·2-76·1)	77·0 (76·1-78·0)	78·0 (77·0-79·0)	79·0 (77·1-81·0)
Netherlands	70·9 (70·4-71·4)	71·5 (71·0-72·1)	72·5 (71·9-73·0)	73·1 (72·5-73·6)	73·9 (73·4-74·3)	74·7 (74·2-75·1)	75·5 (75·1-76·1)	77·2 (76·7-77·7)	78·8 (78·3-79·2)	79·6 (78·6-80·6)
Norway	71·0 (70·4-71·6)	71·7 (71·2-72·2)	72·4 (71·8-72·9)	72·7 (72·2-73·3)	73·6 (73·1-74·2)	75·1 (74·6-75·6)	76·0 (75·5-76·5)	77·8 (77·3-78·3)	78·9 (78·4-79·3)	80·1 (79·0-81·3)
Portugal	64·2 (63·8-64·6)	66·4 (66·0-66·9)	68·1 (67·7-68·5)	69·7 (69·3-70·0)	70·7 (70·3-71·1)	71·6 (71·2-72·0)	73·1 (72·7-73·5)	74·9 (74·5-75·4)	76·6 (76·3-77·0)	77·8 (76·9-78·7)
Spain	69·1 (68·9-69·4)	70·6 (70·3-70·8)	72·3 (72·1-72·5)	73·2 (72·9-73·4)	73·6 (73·3-73·9)	74·5 (74·2-74·8)	75·9 (75·7-76·2)	77·2 (77·0-77·4)	79·0 (78·8-79·2)	80·3 (79·7-80·8)
Sweden	72·1 (71·3-72·9)	72·2 (71·5-72·8)	72·8 (72·4-73·2)	73·9 (73·5-74·3)	74·9 (74·5-75·3)	76·3 (75·9-76·6)	77·4 (77·0-77·7)	78·4 (78·0-78·7)	79·5 (79·2-79·9)	80·1 (78·8-81·4)
Stockholm	71·8 (68·9-74·6)	71·6 (69·4-73·7)	72·1 (71·2-72·8)	73·4 (72·7-74·0)	74·1 (73·5-74·8)	75·9 (75·3-76·5)	77·3 (76·8-77·9)	78·6 (78·0-79·1)	79·8 (79·2-80·3)	80·3 (78·0-82·6)
Sweden except Stockholm	72·1 (71·4-72·9)	72·3 (71·7-72·9)	73·0 (72·5-73·5)	74·0 (73·5-74·5)	75·0 (74·6-75·5)	76·4 (75·9-76·8)	77·4 (76·9-77·8)	78·3 (77·9-78·8)	79·4 (79·0-79·9)	80·1 (78·5-81·5)
Switzerland	70·0 (69·2-70·8)	71·4 (70·6-72·3)	72·4 (71·6-73·2)	73·6 (72·8-74·4)	74·0 (73·2-74·7)	75·3 (74·5-76·1)	77·1 (76·4-77·9)	78·7 (77·9-79·4)	80·0 (79·3-80·7)	81·0 (78·1-83·6)
United Kingdom	68·7 (68·5-68·9)	69·4 (69·2-69·6)	70·5 (70·4-70·7)	71·6 (71·5-71·8)	72·9 (72·7-73·0)	74·0 (73·8-74·1)	75·3 (75·2-75·5)	76·8 (76·7-77·0)	78·3 (78·2-78·5)	78·9 (78·7-79·1)
England	69·0 (68·8-69·2)	69·7 (69·5-69·9)	70·8 (70·7-70·9)	71·9 (71·8-72·0)	73·1 (73·0-73·2)	74·3 (74·2-74·4)	75·6 (75·5-75·7)	77·1 (77·1-77·2)	78·6 (78·6-78·7)	79·2 (79·1-79·4)
East Midlands	68·9 (68·1-69·6)	69·6 (69·0-70·3)	70·8 (70·3-71·3)	72·0 (71·7-72·3)	73·2 (72·9-73·5)	74·3 (74·0-74·7)	75·7 (75·4-76·1)	77·1 (76·8-77·4)	78·5 (78·2-78·8)	79·1 (78·6-79·6)
East of England	70·2 (69·4-71·0)	71·0 (70·3-71·6)	72·1 (71·6-72·6)	73·1 (72·8-73·4)	74·4 (74·1-74·8)	75·5 (75·2-75·8)	76·7 (76·4-77·1)	78·2 (77·9-78·5)	79·6 (79·3-80·0)	80·1 (79·6-80·6)
Greater London	69·2 (68·8-69·6)	69·8 (69·5-70·2)	70·7 (70·5-71·0)	71·8 (71·6-72·0)	72·9 (72·7-73·1)	73·8 (73·6-74·0)	75·3 (75·1-75·5)	77·0 (76·8-77·2)	79·0 (78·8-79·1)	79·7 (79·4-79·9)
North East England	67·5 (66·9-68·2)	68·3 (67·7-68·8)	69·4 (69·0-69·8)	70·5 (70·2-70·8)	71·5 (71·3-71·8)	72·7 (72·5-73·0)	74·1 (73·8-74·4)	75·6 (75·3-75·9)	77·2 (76·9-77·5)	77·8 (77·3-78·3)
North West England	67·7 (67·1-68·3)	68·4 (67·8-68·9)	69·5 (69·1-69·8)	70·6 (70·4-70·9)	71·8 (71·5-72·0)	72·9 (72·7-73·1)	74·2 (74·0-74·5)	75·7 (75·5-75·9)	77·2 (77·0-77·5)	77·9 (77·6-78·3)
South East England	70·3 (69·7-70·9)	71·0 (70·4-71·5)	72·1 (71·7-72·5)	73·1 (72·8-73·4)	74·4 (74·1-74·6)	75·5 (75·2-75·8)	76·8 (76·5-77·1)	78·3 (78·1-78·6)	79·7 (79·5-80·0)	80·2 (79·9-80·6)
South West England	70·2 (69·6-70·8)	70·9 (70·4-71·4)	72·0 (71·6-72·3)	73·1 (72·8-73·4)	74·3 (74·1-74·6)	75·5 (75·2-75·7)	76·8 (76·5-77·0)	78·2 (78·0-78·5)	79·5 (79·2-79·7)	80·0 (79·6-80·3)
West Midlands	68·5 (67·9-69·2)	69·2 (68·6-69·9)	70·2 (69·8-70·6)	71·3 (71·0-71·7)	72·6 (72·3-72·8)	73·9 (73·6-74·2)	75·0 (74·7-75·3)	76·5 (76·2-76·7)	78·1 (77·8-78·4)	78·6 (78·1-79·0)
Yorkshire and the Humber	68·1 (67·5-68·8)	68·9 (68·3-69·4)	70·0 (69·6-70·4)	71·1 (70·8-71·4)	72·5 (72·2-72·7)	73·7 (73·4-73·9)	74·9 (74·6-75·1)	76·5 (76·3-76·8)	77·8 (77·6-78·1)	78·4 (78·0-78·8)
Northern Ireland	67·8 (67·1-68·6)	67·7 (66·9-68·4)	68·8 (68·1-69·7)	70·3 (69·2-71·5)	71·9 (70·9-72·9)	73·3 (72·4-74·2)	74·7 (73·7-75·7)	76·1 (75·1-77·0)	77·3 (76·3-78·3)	77·9 (76·1-79·7)
Scotland	67·2 (66·6-67·9)	67·8 (67·1-68·4)	69·0 (68·4-69·6)	69·8 (68·9-70·7)	70·9 (70·0-71·8)	71·9 (71·0-72·8)	73·2 (72·2-74·1)	74·5 (73·5-75·3)	76·1 (75·2-77·0)	76·9 (75·4-78·4)

Appendix Table 13B. Estimates of life expectancy at birth by location, males, 1970, 1975, 1980, 1985, 1990, 1995, 2000, 2005, 2010, 2016										
**Location**	**1970**	**1975**	**1980**	**1985**	**1990**	**1995**	**2000**	**2005**	**2010**	**2016**
Wales	68·0 (66·0-70·0)	68·8 (67·1-70·6)	70·0 (68·8-71·3)	71·3 (70·4-72·2)	72·6 (71·7-73·3)	73·6 (72·8-74·4)	74·9 (74·0-75·9)	76·5 (75·6-77·3)	77·6 (76·6-78·6)	77·9 (76·4-79·4)
Southern Latin America	61·6 (61·1-62·2)	63·9 (63·2-64·7)	66·5 (66·1-66·9)	68·1 (67·6-68·5)	69·0 (68·5-69·4)	70·0 (69·6-70·5)	71·4 (71·0-71·8)	72·6 (72·2-73·0)	73·4 (73·0-73·8)	74·4 (73·3-75·4)
Argentina	61·9 (61·1-62·5)	63·8 (62·8-64·9)	66·3 (65·8-66·9)	67·8 (67·3-68·4)	68·5 (68·0-69·1)	69·3 (68·7-69·8)	70·5 (70·0-71·0)	71·7 (71·1-72·2)	72·5 (72·1-73·0)	73·3 (72·3-74·4)
Chile	60·3 (59·4-61·3)	64·1 (63·3-65·1)	67·0 (66·2-68·1)	68·8 (67·9-69·7)	70·2 (69·3-71·1)	72·2 (71·4-73·1)	74·3 (73·3-75·1)	75·5 (74·7-76·3)	76·2 (75·4-76·9)	77·3 (74·0-80·5)
Uruguay	65·1 (64·6-65·5)	65·3 (64·9-65·8)	66·7 (66·2-67·1)	68·1 (67·7-68·5)	69·1 (68·7-69·5)	69·5 (69·1-69·9)	70·9 (70·5-71·3)	71·8 (71·4-72·2)	72·5 (72·0-73·0)	73·4 (72·6-74·4)
Central Europe, Eastern Europe, and Central Asi	63·4 (62·7-64·2)	63·7 (63·1-64·4)	63·2 (62·5-63·9)	64·1 (63·5-64·6)	64·6 (63·9-65·2)	61·1 (60·3-62·0)	62·5 (61·7-63·3)	63·0 (62·3-63·9)	66·2 (65·4-66·9)	68·2 (66·1-70·2)
Eastern Europe	63·8 (62·7-64·9)	63·2 (62·1-64·3)	62·2 (61·1-63·3)	63·4 (62·5-64·4)	64·2 (63·1-65·3)	59·0 (57·7-60·5)	60·0 (58·8-61·4)	60·2 (59·0-61·5)	64·0 (62·8-65·3)	66·1 (62·6-69·7)
Belarus	66·7 (65·8-67·7)	66·5 (65·6-67·4)	65·5 (64·6-66·3)	65·8 (65·1-66·6)	65·7 (64·9-66·5)	62·6 (61·8-63·5)	62·6 (61·8-63·5)	62·8 (62·0-63·6)	64·5 (63·7-65·2)	68·2 (65·6-70·8)
Estonia	65·8 (65·1-66·4)	65·0 (64·3-65·7)	64·2 (63·6-64·9)	64·7 (64·0-65·4)	64·8 (64·2-65·5)	61·6 (60·9-62·3)	65·5 (64·8-66·2)	67·4 (66·8-68·1)	71·1 (70·6-71·8)	73·0 (71·3-74·7)
Latvia	65·9 (65·1-66·7)	63·8 (63·0-64·7)	63·7 (62·9-64·5)	64·7 (64·0-65·6)	64·3 (63·4-65·2)	60·3 (59·4-61·2)	64·9 (64·0-65·7)	65·1 (64·3-66·0)	68·0 (67·2-68·7)	70·0 (68·1-72·0)
Lithuania	67·0 (66·3-67·6)	66·6 (66·0-67·2)	65·7 (65·1-66·3)	66·0 (65·4-66·6)	66·4 (65·8-67·0)	63·1 (62·5-63·8)	66·6 (65·9-67·2)	65·4 (64·8-66·1)	67·5 (66·8-68·2)	69·7 (68·6-70·9)
Moldova	59·3 (55·5-63·2)	60·5 (57·3-63·9)	61·4 (59·2-63·5)	62·1 (60·5-63·7)	63·6 (62·3-65·0)	60·7 (59·4-62·0)	63·8 (62·5-65·1)	64·5 (63·1-65·9)	65·6 (64·3-66·9)	68·3 (66·6-70·3)
Russia	63·2 (61·7-64·7)	62·4 (60·9-63·9)	61·3 (59·7-62·9)	62·7 (61·3-64·0)	63·7 (62·1-65·3)	58·0 (56·1-59·9)	59·0 (57·3-60·8)	59·3 (57·7-61·1)	63·4 (61·7-65·0)	65·4 (60·8-70·7)
Ukraine	64·9 (63·4-66·3)	64·7 (63·3-65·9)	63·8 (62·7-64·9)	65·0 (64·1-65·8)	65·2 (64·2-66·0)	61·0 (60·0-62·0)	61·7 (60·7-62·7)	61·3 (60·3-62·4)	65·4 (64·4-66·4)	67·0 (63·3-71·4)
Central Europe	66·2 (65·8-66·5)	67·1 (66·8-67·5)	66·8 (66·5-67·1)	67·0 (66·7-67·2)	67·2 (66·9-67·5)	67·1 (66·9-67·4)	69·2 (69·0-69·5)	70·5 (70·2-70·7)	71·8 (71·6-72·0)	73·5 (72·9-74·0)
Albania	66·0 (61·9-69·1)	68·6 (65·8-71·1)	70·3 (68·5-72·0)	70·7 (69·6-71·7)	71·0 (70·3-71·6)	70·8 (70·2-71·4)	71·4 (70·8-71·9)	72·3 (71·7-72·8)	73·6 (72·8-74·4)	74·7 (73·1-76·4)
Bosnia and Herzegovina	63·5 (60·2-66·6)	65·1 (62·3-67·9)	66·6 (64·2-68·9)	67·9 (66·2-69·7)	69·0 (67·5-70·6)	64·8 (63·0-66·5)	72·0 (70·5-73·5)	73·1 (71·7-74·5)	74·2 (72·7-75·8)	74·9 (73·0-76·9)
Bulgaria	68·9 (68·5-69·3)	68·8 (68·4-69·3)	68·6 (68·2-69·0)	68·2 (67·8-68·7)	68·2 (67·8-68·6)	67·3 (66·8-67·8)	68·4 (67·9-68·9)	69·1 (68·6-69·6)	70·4 (69·9-70·9)	71·7 (69·6-73·9)
Croatia	64·4 (60·8-67·8)	65·7 (62·6-68·7)	66·7 (64·0-69·4)	67·2 (65·8-68·6)	68·3 (67·3-69·5)	68·3 (67·1-69·6)	70·2 (69·3-71·1)	71·5 (70·6-72·5)	72·9 (72·0-73·8)	74·2 (72·6-76·0)
Czech Republic	66·1 (65·8-66·5)	66·9 (66·6-67·2)	67·1 (66·8-67·4)	67·5 (67·2-67·8)	68·1 (67·8-68·4)	69·8 (69·4-70·1)	71·6 (71·3-72·0)	73·0 (72·7-73·3)	74·4 (74·1-74·7)	76·2 (75·5-76·9)
Hungary	66·3 (65·7-66·9)	66·4 (65·7-67·1)	65·8 (65·1-66·5)	65·1 (64·3-65·9)	65·3 (64·5-66·1)	65·6 (64·8-66·4)	67·5 (66·8-68·3)	68·9 (68·1-69·7)	70·7 (69·9-71·4)	72·2 (70·7-73·9)
Macedonia	65·3 (62·1-67·8)	66·6 (64·0-68·7)	67·3 (65·7-68·8)	68·0 (66·6-69·2)	68·2 (67·5-69·1)	68·8 (68·1-69·5)	69·8 (69·1-70·5)	70·5 (69·9-71·2)	71·6 (70·9-72·3)	72·4 (71·5-73·3)
Montenegro	70·0 (68·3-71·6)	70·9 (69·1-72·4)	71·1 (69·6-72·6)	71·2 (69·8-72·6)	72·2 (71·0-73·3)	70·9 (70·0-71·8)	70·8 (69·9-71·6)	72·1 (71·3-72·8)	73·6 (72·7-74·6)	74·4 (73·1-75·5)
Poland	66·4 (66·0-66·9)	67·1 (66·6-67·6)	66·2 (65·7-66·7)	66·6 (66·1-67·1)	66·5 (66·0-67·1)	67·5 (66·9-68·0)	69·6 (69·0-70·1)	71·0 (70·4-71·6)	72·1 (71·7-72·6)	74·1 (72·8-75·4)
Romania	66·2 (65·6-66·9)	67·8 (67·2-68·4)	67·0 (66·4-67·7)	66·8 (66·2-67·5)	66·6 (65·9-67·3)	65·5 (64·7-66·2)	67·6 (66·9-68·4)	68·8 (68·1-69·5)	69·8 (69·1-70·5)	71·7 (70·2-73·1)
Serbia	67·8 (66·0-69·5)	68·7 (66·8-70·2)	68·9 (67·1-70·6)	69·0 (67·4-70·7)	69·6 (68·5-70·9)	67·5 (66·7-68·4)	69·4 (68·6-70·3)	70·6 (69·8-71·4)	71·9 (71·3-72·6)	73·0 (72·2-73·9)
Slovakia	66·7 (66·0-67·3)	66·8 (66·2-67·5)	66·8 (66·1-67·4)	66·9 (66·3-67·5)	66·7 (66·1-67·4)	68·3 (67·8-69·0)	69·1 (68·6-69·7)	70·3 (69·6-70·8)	71·8 (71·1-72·4)	73·4 (71·9-75·0)
Slovenia	64·0 (59·9-68·3)	65·2 (61·7-68·7)	66·3 (64·2-68·7)	67·6 (66·3-68·9)	69·5 (68·5-70·8)	70·6 (69·5-71·7)	72·1 (71·1-73·2)	73·9 (72·8-75·1)	76·2 (75·2-77·2)	77·8 (76·2-79·5)
Central Asia	58·7 (56·6-60·7)	60·5 (58·7-62·1)	61·8 (60·7-62·9)	63·1 (62·4-63·8)	63·4 (62·8-64·0)	61·3 (60·6-62·0)	62·2 (61·6-62·9)	62·8 (62·0-63·6)	65·2 (64·5-66·0)	67·5 (66·4-68·7)
Armenia	62·4 (58·5-66·2)	64·7 (61·7-67·9)	66·4 (64·6-68·2)	67·8 (66·7-68·9)	66·8 (65·9-67·8)	65·0 (64·0-66·0)	68·4 (67·4-69·3)	69·1 (68·1-70·1)	70·2 (69·3-71·2)	72·0 (70·5-73·5)
Azerbaijan	57·9 (54·0-61·7)	60·9 (57·4-64·1)	61·8 (59·6-64·1)	62·4 (60·9-64·0)	62·3 (60·8-63·7)	60·9 (59·2-62·3)	63·3 (61·9-64·6)	64·5 (63·0-66·1)	66·1 (64·5-67·7)	68·4 (65·7-71·2)
Georgia	60·1 (56·1-64·0)	62·6 (58·9-66·6)	64·7 (62·4-66·9)	65·8 (64·5-67·2)	65·4 (64·1-66·8)	66·4 (65·0-67·8)	67·8 (66·5-69·1)	67·7 (66·6-69·0)	68·0 (66·7-69·2)	69·1 (66·2-72·2)
Kazakhstan	59·5 (54·8-64·2)	60·6 (56·1-65·0)	61·3 (58·7-64·2)	62·8 (60·9-64·8)	63·0 (61·4-64·8)	59·1 (57·5-60·9)	59·0 (57·3-60·7)	59·2 (57·4-61·1)	63·4 (61·7-65·0)	67·1 (64·1-70·0)
Kyrgyzstan	55·9 (52·7-59·0)	57·9 (55·0-60·8)	59·5 (58·0-61·2)	61·4 (60·6-62·2)	61·8 (61·1-62·7)	59·8 (59·0-60·5)	62·2 (61·5-62·9)	62·6 (61·8-63·4)	64·3 (63·5-65·0)	67·5 (66·1-69·0)
Mongolia	53·4 (50·9-55·8)	56·4 (54·1-58·6)	56·9 (54·9-58·8)	58·7 (56·9-60·4)	59·8 (58·2-61·2)	58·9 (57·6-60·3)	59·9 (58·8-61·3)	59·8 (58·3-61·3)	62·0 (60·5-63·7)	63·6 (61·4-65·8)
Tajikistan	55·3 (51·2-58·9)	57·3 (54·5-60·2)	60·4 (58·3-62·7)	61·5 (59·4-63·6)	62·4 (60·7-64·2)	61·2 (59·4-63·1)	63·0 (61·3-64·7)	65·0 (63·5-66·7)	67·0 (65·3-68·7)	69·4 (67·1-71·5)
Turkmenistan	52·5 (47·5-56·7)	55·2 (51·5-58·4)	57·7 (55·5-59·6)	59·6 (58·2-60·9)	60·3 (59·0-61·6)	59·5 (58·1-60·6)	59·7 (58·1-61·0)	61·2 (59·8-62·7)	64·2 (62·7-65·4)	66·5 (65·1-67·8)
Uzbekistan	60·7 (57·9-63·4)	62·4 (60·2-64·4)	63·2 (61·5-64·8)	64·7 (63·5-65·9)	65·3 (64·2-66·3)	63·2 (62·1-64·3)	63·4 (62·3-64·5)	63·7 (62·2-65·0)	65·7 (64·5-66·8)	67·1 (65·3-68·8)
Latin America and Caribbean	58·6 (57·7-59·5)	61·4 (60·9-61·9)	63·3 (62·9-63·7)	64·6 (64·2-64·9)	66·4 (66·1-66·7)	67·6 (67·3-67·9)	69·3 (69·0-69·5)	70·6 (70·4-70·9)	70·1 (68·8-71·3)	72·8 (72·2-73·2)
Central Latin America	60·5 (60·0-61·0)	62·4 (62·0-62·9)	64·2 (63·8-64·6)	65·5 (65·1-65·9)	68·1 (67·8-68·4)	69·2 (68·8-69·5)	70·8 (70·4-71·1)	71·9 (71·6-72·2)	72·4 (72·1-72·8)	73·6 (72·9-74·2)
Colombia	63·9 (63·2-64·5)	64·2 (63·5-64·8)	66·5 (65·8-67·2)	64·5 (63·5-65·6)	67·6 (67·0-68·2)	68·2 (67·6-68·7)	69·4 (68·9-69·9)	71·8 (71·3-72·4)	73·5 (73·0-74·0)	75·4 (74·2-76·7)
Costa Rica	67·6 (66·9-68·1)	70·1 (69·4-70·7)	72·5 (72·0-73·1)	73·8 (73·3-74·3)	74·2 (73·7-74·8)	74·5 (74·0-75·0)	75·5 (74·9-76·0)	76·8 (76·2-77·4)	77·9 (77·3-78·4)	78·5 (77·4-79·6)
El Salvador	56·2 (54·7-57·7)	58·6 (57·5-59·9)	61·4 (60·1-62·8)	60·1 (58·6-61·5)	63·9 (63·0-64·9)	66·2 (65·3-67·1)	67·9 (66·9-68·9)	69·0 (68·1-69·9)	70·0 (69·0-71·0)	71·5 (69·3-73·4)
Guatemala	49·6 (47·4-51·9)	54·3 (52·1-56·4)	56·5 (54·1-59·0)	60·3 (58·1-62·4)	62·8 (61·0-65·1)	63·7 (61·3-66·0)	65·3 (62·6-68·0)	66·2 (63·3-68·8)	67·9 (65·3-70·8)	69·4 (65·4-73·9)
Honduras	56·4 (54·9-58·3)	59·9 (58·2-61·8)	63·7 (61·4-66·1)	65·8 (63·3-68·5)	67·4 (63·8-70·2)	68·2 (64·2-71·6)	68·9 (65·2-72·9)	69·7 (65·6-74·0)	70·6 (66·4-74·8)	71·6 (67·3-75·8)
Mexico	59·9 (59·1-60·6)	61·9 (61·2-62·5)	63·1 (62·5-63·6)	65·7 (65·2-66·2)	68·3 (67·9-68·7)	69·8 (69·5-70·2)	71·8 (71·4-72·1)	72·6 (72·2-72·9)	72·6 (72·2-72·9)	73·7 (73·2-74·2)
Nicaragua	56·0 (54·7-57·4)	61·5 (60·6-62·4)	65·1 (63·7-66·8)	60·7 (57·1-64·5)	70·7 (70·0-71·4)	70·8 (70·0-71·6)	72·8 (72·2-73·5)	73·8 (73·1-74·4)	74·7 (74·1-75·3)	75·2 (72·9-77·7)
Panama	67·2 (66·2-68·4)	69·8 (68·7-70·8)	71·5 (70·5-72·5)	72·2 (71·2-73·1)	72·5 (71·4-73·6)	72·5 (71·4-73·5)	74·1 (73·1-75·2)	74·8 (73·8-75·9)	74·8 (73·6-75·9)	76·0 (74·0-78·1)
Venezuela	65·3 (64·3-66·3)	65·9 (65·0-66·8)	67·4 (66·4-68·4)	69·4 (68·3-70·5)	70·0 (68·9-70·9)	69·9 (68·7-71·1)	70·7 (69·7-71·7)	71·4 (70·2-72·4)	71·3 (70·1-72·5)	71·3 (68·2-73·8)
Andean Latin America	49·0 (43·9-54·6)	59·6 (58·8-60·5)	62·0 (61·2-62·9)	63·9 (63·2-64·7)	66·5 (65·7-67·1)	68·5 (67·8-69·2)	71·1 (70·5-71·7)	73·2 (72·5-73·8)	74·3 (73·5-75·1)	76·0 (74·6-77·5)
Bolivia	48·7 (47·0-50·4)	51·9 (50·6-53·4)	55·0 (53·7-56·4)	57·6 (56·2-59·2)	60·5 (59·2-61·8)	63·6 (62·4-64·8)	66·2 (65·3-67·2)	68·7 (67·4-70·1)	70·5 (68·6-72·9)	72·2 (69·7-75·0)
Ecuador	61·7 (60·8-62·5)	63·7 (62·9-64·4)	66·0 (65·2-66·7)	67·7 (67·0-68·4)	69·0 (68·3-69·6)	70·3 (69·6-71·0)	71·6 (70·9-72·3)	72·8 (72·1-73·5)	74·1 (73·4-74·8)	75·4 (74·1-76·5)
Peru	44·6 (36·9-53·9)	60·9 (59·4-62·3)	62·8 (61·6-64·0)	64·4 (63·3-65·6)	67·4 (66·3-68·5)	69·4 (68·3-70·6)	72·7 (71·7-73·8)	75·1 (74·0-76·1)	75·9 (74·6-77·1)	77·8 (75·3-80·3)
Caribbean	61·5 (60·6-62·4)	62·9 (62·1-63·8)	63·9 (63·0-64·7)	65·1 (64·3-65·7)	65·9 (65·3-66·6)	66·3 (65·7-66·9)	67·7 (67·1-68·4)	68·5 (67·7-69·3)	54·4 (44·3-66·8)	71·0 (69·7-72·3)
Antigua and Barbuda	64·9 (63·4-66·2)	65·8 (64·5-67·2)	67·7 (66·4-69·3)	69·8 (68·7-71·2)	70·8 (69·6-72·0)	71·4 (70·2-72·6)	71·7 (70·5-72·8)	73·3 (72·2-74·6)	74·1 (72·7-75·4)	74·7 (72·7-76·4)
The Bahamas	62·3 (60·6-63·9)	64·1 (62·8-65·4)	65·3 (64·1-66·4)	65·7 (64·5-66·8)	67·1 (66·0-68·1)	67·0 (66·1-67·9)	68·0 (67·2-68·8)	70·1 (69·3-70·9)	71·1 (70·3-71·9)	71·3 (69·7-72·9)

Appendix Table 13B. Estimates of life expectancy at birth by location, males, 1970, 1975, 1980, 1985, 1990, 1995, 2000, 2005, 2010, 2016

Location	1970	1975	1980	1985	1990	1995	2000	2005	2010	2016
Barbados	67·2 (65·5–68·7)	68·8 (67·3–70·0)	70·4 (69·1–71·5)	70·7 (69·5–71·7)	70·9 (69·7–72·0)	71·0 (69·8–72·2)	72·2 (71·0–73·5)	73·5 (72·4–74·5)	74·2 (73·2–75·2)	74·4 (72·8–75·9)
Belize	64·7 (62·7–66·7)	66·2 (64·8–67·5)	67·5 (66·1–68·8)	69·0 (67·8–70·2)	69·4 (67·8–70·5)	67·5 (66·2–68·9)	65·6 (64·4–66·7)	67·0 (65·8–68·1)	68·6 (67·4–69·7)	69·1 (67·1–71·4)
Bermuda	64·3 (63·2–65·3)	65·2 (64·2–66·4)	65·9 (64·8–67·2)	66·1 (65·0–67·2)	66·8 (65·7–67·9)	68·8 (67·7–69·8)	70·2 (69·2–71·2)	73·0 (72·0–73·9)	74·2 (73·3–75·3)	75·7 (74·1–77·2)
Cuba	70·4 (69·8–70·9)	71·7 (71·2–72·3)	72·2 (71·6–72·7)	72·4 (71·8–73·0)	72·7 (72·1–73·3)	73·0 (72·4–73·6)	74·4 (73·8–75·0)	75·5 (74·9–76·1)	76·3 (75·7–76·9)	76·7 (75·6–77·7)
Dominica	66·9 (65·5–68·2)	69·6 (68·4–70·7)	72·3 (71·2–73·3)	72·1 (71·0–73·2)	70·4 (69·5–71·5)	69·6 (68·5–70·7)	70·5 (69·5–71·7)	70·8 (69·7–71·9)	70·3 (69·1–71·5)	70·2 (67·9–72·4)
Dominican Republic	63·5 (62·4–64·7)	65·6 (64·3–66·8)	67·5 (66·4–68·6)	68·3 (67·4–69·4)	68·9 (67·9–70·0)	69·8 (68·8–70·7)	70·4 (69·4–71·7)	70·5 (69·3–72·1)	71·6 (70·5–73·4)	72·9 (71·2–74·9)
Grenada	65·3 (62·5–67·9)	66·8 (64·4–69·3)	67·7 (64·9–70·2)	67·7 (65·2–70·1)	68·7 (66·9–70·4)	68·9 (67·4–70·3)	68·3 (67·0–69·9)	68·2 (66·9–69·6)	68·6 (67·2–70·1)	68·8 (66·7–71·1)
Guyana	58·4 (57·1–59·6)	59·3 (57·9–60·5)	60·4 (59·1–61·7)	61·1 (59·9–62·4)	60·9 (59·8–62·1)	62·0 (60·9–63·1)	62·3 (61·1–63·5)	61·4 (60·4–62·6)	62·5 (61·3–63·8)	64·7 (63·0–66·4)
Haiti	44·1 (41·1–47·0)	47·1 (44·5–49·9)	49·5 (47·0–52·1)	51·9 (49·8–54·1)	53·9 (52·0–55·8)	54·8 (52·9–56·6)	56·6 (54·6–58·7)	58·2 (55·8–60·7)	28·1 (18·4–51·9)	63·6 (60·2–66·9)
Jamaica	66·7 (65·4–67·9)	68·9 (67·5–70·4)	70·0 (68·7–71·4)	71·4 (70·3–72·5)	73·4 (72·1–74·7)	73·0 (71·4–74·5)	72·3 (70·8–73·6)	73·3 (71·9–74·7)	72·9 (71·4–74·3)	73·1 (70·4–75·5)
Puerto Rico	68·7 (68·0–69·5)	69·0 (68·3–69·8)	70·4 (69·6–71·2)	70·4 (69·5–71·2)	69·7 (68·8–70·5)	69·1 (68·2–69·9)	72·0 (71·3–72·9)	73·2 (72·4–74·0)	74·1 (73·3–74·9)	75·0 (73·8–76·4)
Saint Lucia	63·7 (60·6–65·9)	65·0 (63·2–66·4)	66·8 (65·4–68·0)	68·1 (66·8–69·2)	69·5 (68·3–70·6)	70·6 (69·6–71·6)	71·6 (70·6–72·5)	71·1 (70·0–72·0)	72·2 (71·2–73·2)	73·0 (71·6–74·3)
Saint Vincent and the Grenadines	62·6 (60·1–65·0)	64·4 (62·2–66·2)	67·0 (65·2–68·4)	68·5 (67·0–70·1)	68·5 (67·2–69·9)	68·4 (67·3–69·5)	67·7 (66·6–68·6)	68·6 (67·7–69·5)	68·9 (68·0–69·8)	68·8 (67·4–70·0)
Suriname	62·8 (61·6–63·9)	62·7 (61·7–63·6)	63·6 (62·8–64·4)	66·5 (65·8–67·2)	67·7 (67·0–68·4)	67·9 (67·1–68·5)	67·2 (66·4–67·9)	66·6 (65·9–67·3)	67·2 (66·4–68·0)	68·4 (67·1–69·7)
Trinidad and Tobago	63·5 (62·9–64·2)	64·3 (63·6–65·0)	65·6 (64·9–66·4)	66·3 (65·6–67·0)	66·3 (65·6–67·0)	66·3 (65·5–67·1)	66·4 (65·6–67·1)	67·9 (67·2–68·7)	68·8 (67·9–69·7)	69·3 (67·8–70·8)
Virgin Islands, U.S.	64·0 (62·5–65·3)	65·3 (63·8–66·7)	66·6 (65·4–67·9)	68·7 (67·7–70·1)	69·2 (68·0–70·5)	69·0 (67·7–70·1)	69·6 (68·5–70·4)	70·0 (68·9–71·0)	70·0 (68·5–71·3)	70·6 (68·8–72·9)
Tropical Latin America	58·3 (56·9–59·6)	60·4 (59·3–61·3)	62·5 (61·7–63·2)	63·5 (62·9–64·1)	64·8 (64·3–65·4)	66·0 (65·6–66·5)	67·7 (67·2–68·2)	69·3 (68·8–69·7)	70·5 (70·0–71·0)	71·6 (71·0–72·1)
Brazil	58·2 (56·8–59·6)	60·3 (59·2–61·3)	62·3 (61·5–63·1)	63·4 (62·8–64·0)	64·6 (64·1–65·2)	65·8 (65·3–66·3)	67·6 (67·1–68·1)	69·2 (68·7–69·7)	70·5 (69·9–70·9)	71·6 (70·9–72·1)
Paraguay	68·2 (67·3–69·1)	68·9 (68·0–69·8)	70·6 (69·9–71·4)	71·7 (71·0–72·5)	72·9 (72·2–73·6)	72·6 (71·9–73·3)	72·3 (71·6–72·9)	72·1 (71·4–72·7)	71·8 (70·8–72·7)	72·1 (70·3–73·5)
Southeast Asia, East Asia, and Oceania	58·3 (57·4–59·1)	60·2 (59·4–61·0)	61·9 (61·4–62·4)	63·6 (63·1–64·0)	64·5 (64·0–64·9)	65·5 (65·1–65·8)	66·7 (66·4–67·1)	68·7 (68·4–69·0)	70·5 (70·2–70·7)	72·1 (71·8–72·4)
East Asia	60·1 (59·1–61·0)	62·2 (61·4–63·0)	62·9 (62·3–63·6)	64·5 (63·9–65·1)	65·2 (64·6–65·7)	66·1 (65·6–66·6)	67·5 (67·1–67·8)	69·7 (69·4–70·0)	71·7 (71·4–71·9)	73·3 (73·0–73·6)
China	59·9 (58·9–60·8)	62·0 (61·2–62·8)	62·7 (62·0–63·4)	64·3 (63·7–64·9)	65·0 (64·4–65·5)	66·2 (65·7–66·6)	67·6 (67·2–68·0)	69·7 (69·4–70·0)	71·7 (71·4–72·0)	73·4 (73·0–73·7)
North Korea	65·5 (63·9–67·0)	67·7 (66·2–69·1)	68·6 (67·1–70·3)	68·9 (67·3–70·7)	69·2 (67·6–71·1)	61·4 (54·9–67·8)	60·9 (55·2–66·7)	67·1 (66·0–68·3)	67·4 (66·2–68·5)	67·9 (66·7–69·1)
Taiwan (Province of China)	66·6 (66·0–67·3)	68·6 (67·9–69·2)	69·4 (68·7–70·0)	70·8 (70·1–71·3)	71·5 (70·9–72·1)	72·1 (71·4–72·7)	73·4 (72·8–74·1)	74·6 (73·9–75·3)	76·1 (75·4–76·7)	76·7 (75·0–78·5)
Southeast Asia	53·4 (51·9–54·7)	55·1 (53·0–57·0)	59·5 (58·9–60·0)	61·5 (61·1–62·0)	63·1 (62·7–63·6)	64·4 (63·9–64·8)	65·5 (65·1–66·0)	66·9 (66·3–67·4)	68·3 (67·8–68·8)	70·0 (69·3–70·6)
Cambodia	40·0 (37·3–42·9)	19·1 (12·9–40·6)	49·9 (47·4–52·5)	51·6 (49·3–53·8)	55·5 (54·3–56·7)	56·2 (55·1–57·3)	57·1 (55·6–58·2)	60·2 (59·0–61·4)	63·2 (62·0–64·5)	65·7 (64·5–67·0)
Indonesia	52·9 (51·8–53·9)	55·5 (54·6–56·5)	58·4 (57·6–59·2)	60·8 (60·1–61·5)	62·4 (61·8–62·9)	64·5 (64·0–64·9)	66·1 (65·6–66·6)	67·2 (66·6–67·7)	68·2 (67·5–68·9)	69·8 (68·8–70·7)
Laos	40·1 (33·8–45·4)	44·1 (39·4–48·6)	46·3 (43·1–49·4)	49·1 (47·2–51·0)	50·7 (49·2–52·2)	52·7 (51·3–54·1)	55·1 (53·7–56·4)	58·2 (56·9–59·4)	61·5 (60·3–62·7)	64·8 (62·9–66·4)
Malaysia	62·7 (62·3–63·2)	64·8 (64·3–65·3)	66·8 (66·3–67·3)	68·4 (68·0–68·8)	69·6 (69·3–69·9)	70·2 (69·9–70·5)	71·0 (70·6–71·3)	72·0 (71·7–72·3)	72·5 (72·1–72·8)	73·2 (72·4–73·9)
Maldives	38·8 (35·4–42·5)	44·3 (40·9–47·6)	48·7 (46·3–51·5)	56·1 (55·0–57·3)	62·8 (61·7–64·0)	67·6 (66·0–69·1)	71·6 (70·5–72·5)	74·7 (73·8–75·7)	76·3 (75·3–77·3)	77·6 (75·0–80·3)
Mauritius	58·1 (57·6–58·6)	58·9 (58·4–59·4)	62·0 (61·4–62·5)	63·9 (63·4–64·4)	65·4 (64·9–65·9)	66·4 (65·9–67·0)	68·1 (67·6–68·7)	69·1 (68·5–69·7)	69·9 (69·3–70·5)	71·4 (69·6–73·4)
Myanmar	45·6 (42·8–48·3)	49·8 (47·4–52·2)	52·9 (50·9–55·0)	54·9 (53·1–56·8)	56·3 (54·5–58·0)	57·1 (55·2–58·7)	58·4 (56·0–60·2)	60·6 (57·8–62·5)	63·7 (61·0–65·3)	66·7 (65·2–67·9)
Philippines	59·7 (58·8–60·8)	60·6 (59·6–61·5)	61·3 (60·3–62·2)	61·9 (61·0–62·7)	63·5 (62·6–64·4)	64·3 (63·5–65·2)	64·3 (63·5–65·2)	64·5 (63·7–65·4)	65·5 (64·6–66·4)	66·6 (64·6–68·7)
Sri Lanka	63·2 (62·4–64·1)	62·8 (61·9–63·7)	66·6 (65·7–67·5)	67·2 (66·1–68·3)	67·8 (66·7–68·9)	68·3 (67·0–69·7)	68·6 (67·5–69·8)	70·1 (68·8–71·4)	70·6 (68·9–72·2)	73·7 (70·5–76·9)
Seychelles	62·8 (61·4–63·9)	64·8 (63·6–65·9)	66·7 (65·7–67·7)	66·5 (65·5–67·6)	64·5 (63·4–65·6)	64·9 (63·7–65·9)	66·7 (65·7–67·6)	68·2 (67·3–69·1)	69·6 (68·7–70·6)	70·3 (68·2–72·0)
Thailand	59·9 (59·1–60·8)	62·1 (61·2–63·0)	63·0 (62·2–63·8)	65·6 (64·8–66·4)	67·2 (66·4–68·0)	66·9 (65·9–67·8)	68·3 (67·2–69·3)	70·9 (70·2–71·7)	73·5 (72·8–74·3)	74·6 (72·9–76·2)
Timor-Leste	44·6 (40·0–49·5)	42·0 (38·0–46·2)	46·5 (43·1–50·2)	55·0 (52·5–57·4)	58·4 (56·1–60·8)	60·8 (58·8–63·0)	63·6 (61·7–65·6)	66·6 (64·6–68·8)	70·3 (67·7–72·8)	71·7 (68·7–74·7)
Vietnam	49·1 (41·7–57·1)	58·2 (55·4–60·9)	61·9 (59·7–63·6)	63·8 (61·9–65·5)	65·3 (63·2–67·1)	66·3 (64·3–68·3)	67·3 (65·7–69·5)	68·3 (66·5–70·6)	69·5 (67·5–71·7)	70·9 (69·1–72·7)
Oceania	54·7 (52·7–56·6)	55·3 (53·6–57·1)	55·9 (54·2–57·5)	56·3 (54·6–57·9)	56·8 (55·1–58·5)	57·6 (55·7–59·4)	58·2 (56·1–60·1)	58·3 (56·0–60·4)	59·1 (56·5–61·3)	60·7 (58·2–62·9)
American Samoa	64·2 (62·4–66·0)	65·7 (63·6–67·6)	66·7 (64·7–68·5)	67·5 (65·8–69·2)	66·9 (65·4–68·5)	67·8 (65·7–69·7)	69·0 (67·5–70·5)	69·0 (67·5–70·5)	70·1 (68·4–71·6)	70·4 (67·8–72·7)
Federated States of Micronesia	57·9 (55·3–61·1)	58·5 (56·1–61·4)	59·4 (57·3–61·9)	60·2 (58·0–62·5)	61·0 (58·8–63·2)	62·1 (59·9–64·3)	62·7 (60·5–64·9)	64·0 (61·9–66·2)	63·5 (60·4–66·0)	63·6 (60·7–66·3)
Fiji	61·9 (59·5–64·4)	61·4 (59·5–63·8)	62·0 (60·0–64·2)	62·4 (60·3–64·4)	62·7 (59·2–66·2)	62·1 (59·0–65·0)	61·8 (59·6–63·8)	62·6 (60·6–64·9)	63·2 (61·0–65·8)	63·3 (59·2–68·0)
Guam	64·9 (63·5–66·4)	65·5 (63·8–67·0)	68·1 (66·4–69·7)	69·9 (68·1–71·4)	70·3 (68·9–71·6)	70·7 (69·1–72·1)	71·1 (69·5–72·3)	70·4 (69·1–71·5)	69·0 (67·5–70·5)	69·0 (66·9–71·3)
Kiribati	52·4 (49·2–55·7)	55·1 (51·7–58·5)	56·1 (51·9–59·4)	55·4 (52·2–58·3)	55·5 (53·8–57·4)	55·5 (54·0–56·9)	55·9 (54·5–57·3)	56·6 (54·6–58·4)	57·1 (54·7–59·6)	58·2 (55·4–61·1)
Marshall Islands	57·1 (53·0–61·1)	58·9 (54·9–62·6)	60·3 (57·3–63·5)	60·8 (58·6–62·8)	61·5 (59·9–62·8)	61·9 (60·5–63·1)	61·0 (59·0–63·0)	61·1 (59·3–63·0)	61·6 (59·4–63·9)	62·7 (60·1–65·4)
Northern Mariana Islands	68·1 (65·1–71·5)	69·6 (66·8–72·5)	70·7 (68·0–73·6)	71·5 (68·8–74·2)	72·7 (70·2–75·4)	74·1 (71·8–76·3)	75·0 (73·0–77·0)	74·8 (72·8–76·8)	74·4 (72·3–76·5)	73·9 (71·5–76·2)
Papua New Guinea	52·4 (50·0–55·0)	53·3 (51·1–55·8)	53·5 (51·3–55·8)	53·8 (51·7–56·0)	54·5 (52·2–56·6)	55·6 (53·2–58·1)	56·4 (53·7–59·0)	56·5 (53·6–59·2)	57·5 (54·3–60·2)	59·5 (56·5–62·3)
Samoa	62·6 (60·8–64·4)	62·6 (60·9–64·3)	64·3 (62·4–65·9)	64·9 (62·7–66·6)	65·8 (63·5–67·8)	66·8 (64·5–68·8)	67·8 (65·8–69·8)	68·8 (66·8–70·7)	69·4 (67·5–71·5)	69·9 (67·9–72·2)
Solomon Islands	52·9 (50·3–55·9)	52·5 (49·8–55·2)	57·0 (54·6–59·5)	58·1 (55·8–60·4)	58·6 (56·4–61·0)	59·6 (57·4–61·8)	60·2 (58·2–62·4)	60·0 (57·8–62·3)	60·6 (58·4–62·9)	61·9 (59·3–64·3)
Tonga	63·5 (60·9–66·4)	64·0 (61·2–66·9)	64·0 (61·3–66·8)	64·8 (62·2–67·5)	65·7 (63·1–68·3)	66·1 (63·7–68·4)	66·4 (64·2–68·6)	66·7 (64·5–68·9)	67·0 (64·7–69·2)	67·5 (65·2–70·1)
Vanuatu	55·1 (51·9–57·9)	56·7 (53·8–59·4)	58·2 (55·9–60·5)	59·2 (57·3–61·2)	59·9 (57·9–61·9)	60·4 (58·5–62·4)	60·7 (58·7–62·5)	60·6 (58·5–62·8)	61·4 (58·9–63·7)	62·2 (59·7–64·7)
North Africa and Middle East	52·9 (51·8–54·0)	56·5 (55·7–57·3)	58·9 (58·0–59·8)	61·2 (60·2–62·4)	65·0 (64·4–65·6)	66·2 (65·7–66·7)	67·8 (67·3–68·2)	69·0 (68·3–69·6)	70·4 (69·8–71·0)	70·9 (70·1–71·7)
North Africa and Middle East	52·9 (51·8–54·0)	56·5 (55·7–57·3)	58·9 (58·0–59·8)	61·2 (60·2–62·4)	65·0 (64·4–65·6)	66·2 (65·7–66·7)	67·8 (67·3–68·2)	69·0 (68·3–69·6)	70·4 (69·8–71·0)	70·9 (70·1–71·7)
Afghanistan	47·1 (43·0–50·7)	47·6 (44·4–50·4)	42·9 (38·2–47·9)	39·5 (32·5–48·3)	51·9 (50·1–53·9)	51·0 (49·3–52·7)	51·4 (49·7–53·4)	53·6 (51·8–55·6)	55·6 (53·7–57·5)	56·9 (54·5–59·2)
Algeria	55·9 (54·0–57·9)	58·1 (56·4–59·8)	60·9 (59·5–62·2)	66·3 (65·3–67·4)	69·4 (68·2–70·7)	70·6 (69·3–72·0)	72·4 (71·0–73·6)	74·1 (72·9–75·3)	75·5 (74·6–76·5)	76·4 (75·1–77·7)
Bahrain	63·0 (60·0–65·8)	65·9 (63·0–68·5)	67·8 (65·9–69·9)	68·6 (67·1–70·2)	68·5 (66·9–70·2)	69·4 (67·6–71·1)	70·2 (68·7–71·8)	72·7 (71·1–74·3)	75·4 (73·8–77·1)	76·0 (73·4–78·5)

Appendix Table 13B. Estimates of life expectancy at birth by location, males, 1970, 1975, 1980, 1985, 1990, 1995, 2000, 2005, 2010, 2016

Location	1970	1975	1980	1985	1990	1995	2000	2005	2010	2016
Egypt	49·7 (48·3-50·9)	52·5 (51·3-53·8)	56·6 (55·4-58·0)	59·0 (58·0-60·0)	63·3 (62·4-64·3)	65·4 (64·5-66·3)	67·3 (66·5-68·2)	67·7 (66·9-68·7)	68·0 (67·1-68·9)	69·4 (67·6-71·5)
Iran	53·1 (49·3-56·8)	62·1 (59·3-64·8)	63·4 (60·7-66·1)	65·1 (62·2-68·1)	65·2 (62·6-68·1)	68·2 (66·1-70·6)	69·0 (67·0-70·9)	70·2 (67·4-72·7)	72·4 (69·6-74·9)	73·8 (71·2-76·2)
Iraq	58·0 (56·0-60·1)	58·7 (57·0-60·3)	58·2 (54·1-63·0)	57·0 (51·3-63·7)	64·7 (62·1-67·4)	63·7 (61·2-65·9)	64·2 (62·0-66·4)	62·2 (59·5-65·4)	65·1 (62·1-68·1)	64·9 (61·4-69·1)
Jordan	60·2 (57·5-62·8)	62·4 (59·9-64·7)	64·8 (62·2-66·9)	68·3 (66·3-70·0)	71·0 (69·2-72·6)	71·9 (69·8-73·7)	72·6 (69·7-74·9)	72·8 (70·3-75·1)	74·6 (71·7-77·3)	74·7 (71·4-78·2)
Kuwait	67·1 (65·2-69·0)	67·7 (65·9-69·5)	70·3 (68·6-72·1)	72·9 (71·2-74·6)	74·5 (72·2-76·9)	75·7 (73·9-77·2)	76·3 (74·9-78·0)	76·9 (75·1-78·6)	78·6 (76·9-80·3)	80·0 (76·4-83·6)
Lebanon	65·2 (61·9-68·5)	55·0 (47·0-64·4)	46·4 (36·4-61·7)	60·0 (53·9-66·3)	66·4 (64·6-68·8)	72·8 (71·8-74·0)	75·9 (74·3-77·3)	77·8 (76·2-79·5)	79·0 (77·7-80·3)	78·8 (77·1-80·4)
Libya	64·7 (63·0-66·4)	67·6 (65·9-69·0)	69·7 (68·2-71·1)	70·9 (69·5-72·3)	71·6 (70·3-73·0)	72·8 (71·4-74·0)	73·1 (71·9-74·3)	73·7 (72·5-75·1)	74·3 (72·7-75·9)	72·6 (71·0-74·6)
Morocco	53·0 (51·8-54·2)	55·5 (54·5-56·7)	59·0 (58·1-59·9)	62·7 (61·9-63·6)	65·7 (64·9-66·6)	67·7 (66·8-68·5)	69·4 (68·5-70·3)	70·9 (69·9-71·8)	72·2 (71·2-73·2)	73·5 (72·5-74·5)
Palestine	60·4 (56·1-64·9)	66·3 (63·4-69·1)	68·2 (65·7-70·9)	69·9 (67·8-72·4)	70·1 (68·3-72·0)	71·2 (70·0-72·5)	69·7 (69·0-70·5)	69·6 (69·1-69·9)	70·5 (70·1-70·9)	70·2 (69·5-71·0)
Oman	50·6 (48·1-53·2)	55·8 (53·5-58·1)	62·1 (60·1-64·1)	66·7 (65·1-68·4)	69·5 (68·1-70·8)	71·8 (70·7-72·8)	72·9 (71·5-74·2)	73·9 (72·8-75·0)	74·1 (73·0-75·1)	75·3 (74·5-76·3)
Qatar	64·8 (60·1-69·9)	65·8 (61·4-70·5)	67·2 (63·4-71·2)	72·0 (69·0-74·2)	74·8 (72·3-77·1)	74·3 (71·9-76·7)	74·3 (71·7-76·8)	75·3 (73·0-78·2)	78·2 (75·9-80·6)	79·0 (75·4-82·6)
Saudi Arabia	62·1 (59·0-65·1)	66·5 (63·9-68·7)	69·3 (67·3-71·2)	70·5 (68·8-72·2)	70·6 (69·0-72·1)	72·0 (70·7-73·3)	72·6 (71·6-73·6)	73·2 (72·4-73·9)	74·2 (73·5-75·0)	76·0 (75·0-77·0)
Sudan	51·3 (49·7-52·7)	52·6 (51·2-53·8)	53·8 (52·4-55·1)	49·2 (45·1-53·4)	57·6 (56·5-58·7)	59·4 (58·3-60·5)	61·3 (60·3-62·3)	63·4 (62·5-64·5)	64·6 (63·5-65·7)	66·4 (64·9-67·7)
Syria	62·3 (61·1-63·5)	64·6 (63·7-65·5)	66·4 (65·4-67·4)	68·1 (67·1-69·0)	69·1 (68·1-70·1)	70·5 (69·0-71·5)	71·3 (70·4-72·4)	73·0 (72·2-73·8)	74·0 (73·1-75·0)	63·2 (56·3-70·7)
Tunisia	56·1 (54·8-57·4)	60·5 (59·4-61·6)	63·5 (62·4-64·8)	67·7 (66·4-69·0)	69·4 (68·2-70·6)	70·4 (69·3-71·6)	72·4 (71·1-73·9)	73·5 (72·0-75·3)	74·3 (71·8-76·4)	74·6 (72·1-77·2)
Turkey	53·2 (50·1-56·7)	56·3 (54·0-58·7)	60·3 (58·5-62·3)	64·6 (62·8-66·2)	66·8 (65·7-68·0)	67·4 (66·4-68·4)	70·6 (69·7-71·4)	73·4 (72·5-74·2)	75·0 (73·8-76·3)	75·8 (73·7-78·1)
United Arab Emirates	64·5 (60·4-68·8)	67·7 (63·5-71·7)	70·0 (66·8-73·8)	71·6 (68·1-75·0)	71·7 (68·5-74·8)	72·1 (69·6-74·7)	73·2 (71·5-74·9)	74·9 (73·8-76·0)	74·6 (73·0-76·3)	74·5 (72·1-76·8)
Yemen	40·0 (37·8-41·9)	47·2 (45·9-48·6)	52·1 (50·9-53·4)	56·0 (54·9-57·3)	58·6 (57·4-59·8)	60·5 (59·3-61·7)	62·6 (61·5-63·8)	64·8 (63·7-66·0)	67·3 (65·9-68·5)	65·5 (63·7-67·1)
South Asia	48·5 (47·2-49·7)	51·7 (50·9-52·5)	54·4 (53·7-55·1)	56·5 (55·9-57·0)	58·5 (57·9-59·0)	60·1 (59·5-60·6)	61·5 (61·0-62·0)	63·0 (62·5-63·6)	64·8 (64·3-65·4)	67·1 (66·5-67·7)
South Asia	48·5 (47·2-49·7)	51·7 (50·9-52·5)	54·4 (53·7-55·1)	56·5 (55·9-57·0)	58·5 (57·9-59·0)	60·1 (59·5-60·6)	61·5 (61·0-62·0)	63·0 (62·5-63·6)	64·8 (64·3-65·4)	67·1 (66·5-67·7)
Bangladesh	41·5 (37·3-46·5)	52·1 (50·4-54·0)	54·0 (52·6-55·5)	55·0 (53·8-56·2)	57·6 (56·6-58·7)	61·0 (59·9-62·2)	64·2 (63·2-65·3)	66·4 (65·5-67·3)	68·4 (67·4-69·5)	70·5 (68·8-72·0)
Bhutan	49·2 (46·2-52·6)	51·7 (48·8-54·8)	54·0 (51·4-56·7)	57·1 (54·7-59·6)	60·7 (58·5-62·9)	63·5 (61·6-65·4)	65·3 (63·5-67·1)	68·7 (67·0-70·4)	70·5 (68·6-72·3)	72·2 (70·1-74·4)
India	48·5 (47·2-49·8)	50·9 (49·9-51·8)	54·0 (53·1-54·8)	56·3 (55·6-57·0)	58·3 (57·7-58·9)	59·9 (59·3-60·5)	61·2 (60·7-61·7)	62·9 (62·3-63·5)	64·6 (63·9-65·3)	66·9 (66·2-67·6)
Nepal	48·6 (47·4-49·7)	50·3 (49·1-51·5)	52·3 (51·1-53·6)	54·9 (53·8-56·1)	57·7 (56·7-58·7)	60·4 (59·5-61·3)	62·8 (61·9-63·6)	65·3 (64·3-66·2)	67·4 (66·2-68·5)	69·7 (68·5-71·0)
Pakistan	56·6 (54·6-58·9)	58·4 (56·4-60·3)	60·0 (58·4-61·6)	61·6 (60·5-62·7)	62·4 (61·4-63·4)	62·1 (61·0-63·2)	62·4 (61·3-63·5)	61·7 (60·3-63·2)	64·4 (62·8-66·1)	66·4 (64·3-68·4)
Sub-Saharan Africa	48·2 (46·9-49·4)	49·7 (48·7-50·6)	51·1 (50·3-51·8)	52·0 (51·4-52·6)	52·0 (51·5-52·7)	51·8 (51·2-52·4)	51·5 (50·9-52·0)	53·6 (53·0-54·1)	57·0 (56·3-57·6)	61·2 (60·3-62·0)
Southern Sub-Saharan Africa	55·0 (53·3-56·8)	56·4 (55·0-57·7)	57·4 (56·3-58·6)	59·2 (58·5-60·1)	60·2 (59·3-61·2)	57·5 (56·3-58·7)	50·4 (49·3-51·5)	48·1 (47·2-49·1)	51·5 (50·7-52·2)	58·4 (57·4-59·3)
Botswana	54·8 (53·3-56·2)	57·5 (56·2-58·9)	59·3 (58·1-60·8)	61·4 (60·0-63·0)	61·2 (58·4-64·2)	52·6 (47·2-59·6)	44·7 (39·9-50·8)	46·3 (42·8-50·6)	53·9 (50·7-57·7)	61·7 (58·4-67·0)
Lesotho	53·2 (50·2-56·2)	54·3 (51·4-57·5)	55·8 (53·0-58·8)	56·4 (54·1-59·3)	56·6 (54·6-58·8)	53·5 (51·3-55·6)	45·8 (43·1-48·4)	41·1 (38·8-43·7)	43·9 (41·7-46·0)	47·1 (44·6-49·7)
Namibia	57·1 (55·0-59·4)	57·7 (55·9-59·6)	57·2 (55·7-58·9)	58·1 (56·9-59·5)	58·3 (57·0-59·6)	56·7 (54·7-58·5)	51·1 (48·4-53·7)	49·3 (46·8-52·0)	55·8 (53·5-58·4)	60·4 (57·8-63·3)
South Africa	55·2 (53·0-57·5)	56·5 (54·8-58·4)	57·5 (56·0-58·8)	58·9 (57·9-60·0)	60·7 (59·9-61·6)	59·8 (58·9-60·6)	52·5 (51·4-53·5)	49·7 (48·6-50·7)	52·7 (51·7-53·6)	59·2 (57·9-60·3)
Swaziland	49·1 (45·0-53·1)	52·4 (49·4-55·5)	55·1 (52·3-57·6)	57·2 (54·7-59·7)	59·1 (56·9-61·4)	55·6 (52·6-58·6)	44·9 (41·0-49·1)	40·9 (38·1-44·1)	44·7 (41·9-47·7)	53·3 (50·2-57·1)
Zimbabwe	55·8 (53·6-58·1)	56·9 (55·0-58·9)	58·4 (56·7-60·0)	61·2 (59·8-62·6)	59·0 (55·5-63·7)	50·6 (46·5-55·4)	44·7 (41·8-48·3)	44·0 (41·8-46·4)	47·9 (46·0-49·6)	56·7 (54·4-59·3)
Western Sub-Saharan Africa	47·8 (46·2-49·3)	49·9 (48·5-51·2)	51·9 (50·7-53·0)	53·2 (52·2-54·2)	53·3 (52·3-54·3)	53·3 (52·2-54·3)	53·6 (52·6-54·6)	55·5 (54·5-56·5)	58·4 (57·3-59·4)	61·9 (60·6-63·1)
Benin	45·6 (44·4-47·0)	47·8 (46·4-49·1)	49·6 (48·3-50·9)	51·8 (50·4-53·1)	53·3 (52·0-54·6)	54·3 (53·1-55·6)	55·4 (54·2-56·7)	57·4 (56·1-58·7)	59·8 (58·5-61·3)	62·5 (60·9-64·0)
Burkina Faso	43·0 (41·0-45·0)	44·7 (43·2-46·1)	46·8 (45·6-48·1)	48·9 (47·7-50·0)	49·1 (47·9-50·5)	49·0 (47·6-50·5)	50·6 (49·4-51·8)	53·8 (52·7-54·9)	56·7 (55·4-57·9)	59·5 (58·0-60·8)
Cameroon	51·3 (49·8-53·1)	53·4 (52·0-55·0)	55·4 (54·0-56·8)	57·3 (56·1-58·7)	57·3 (55·8-58·8)	55·0 (53·1-56·7)	53·1 (51·5-54·8)	53·6 (51·9-55·3)	55·9 (54·0-58·0)	58·3 (56·0-60·6)
Cape Verde	55·3 (52·8-58·2)	59·4 (57·1-61·6)	62·9 (61·3-64·6)	62·7 (61·4-64·1)	64·1 (62·8-65·5)	63·0 (60·9-65·3)	65·2 (62·4-67·9)	66·0 (63·7-69·2)	67·2 (65·7-69·1)	68·8 (66·6-71·1)
Chad	46·9 (44·5-49·3)	49·5 (47·6-51·3)	49·5 (48·0-50·9)	51·2 (50·0-52·5)	52·1 (50·7-53·3)	52·2 (50·8-53·5)	50·6 (49·2-52·0)	52·5 (50·9-54·1)	55·1 (53·3-56·7)	58·3 (56·4-60·3)
Cote d'Ivoire	48·9 (47·6-50·1)	51·6 (50·4-52·7)	54·4 (53·4-55·4)	54·6 (53·4-55·6)	52·4 (51·0-53·6)	49·9 (48·4-51·3)	50·2 (48·8-51·7)	51·6 (50·1-53·2)	54·3 (52·6-56·0)	57·7 (55·8-59·5)
The Gambia	53·5 (50·9-56·0)	55·5 (53·4-57·6)	57·5 (55·6-59·5)	59·4 (57·7-61·3)	60·9 (59·2-62·6)	62·1 (60·6-63·7)	62·3 (60·8-63·8)	62·6 (61·2-64·1)	63·8 (62·3-65·3)	65·5 (64·0-66·9)
Ghana	54·3 (52·7-55·8)	55·8 (54·3-57·5)	56·7 (55·2-58·3)	57·1 (55·6-58·7)	57·8 (56·2-59·4)	58·4 (56·7-60·1)	58·0 (56·6-59·5)	58·7 (57·5-60·0)	60·9 (59·5-62·2)	64·5 (62·8-66·1)
Guinea	45·3 (42·0-48·6)	46·5 (43·9-48·9)	48·5 (46·3-50·6)	50·4 (48·6-52·1)	52·1 (50·7-53·6)	53·0 (51·6-54·4)	53·9 (52·3-55·3)	55·3 (53·6-57·0)	57·0 (55·0-58·9)	59·8 (57·3-62·1)
Guinea-Bissau	36·2 (32·6-40·0)	41·7 (39·2-44·2)	43·6 (41·5-45·8)	44·7 (42·8-46·8)	46·6 (44·7-48·6)	48·3 (46·6-50·1)	49·9 (48·3-51·6)	50·8 (49·3-52·6)	53·5 (52·0-55·3)	56·3 (54·5-58·2)
Liberia	46·1 (43·7-48·4)	48·7 (46·8-50·6)	50·6 (49·0-52·3)	51·6 (50·2-53·1)	48·0 (46·6-49·5)	49·1 (47·9-50·3)	54·1 (52·9-55·3)	58·1 (56·8-59·3)	60·6 (59·4-61·8)	64·0 (62·8-65·2)
Mali	39·4 (37·2-41·9)	41·2 (39·3-43·3)	44·6 (42·9-46·3)	46·9 (45·5-48·4)	49·4 (48·1-50·7)	51·3 (50·0-52·7)	53·3 (51·8-54·9)	55·9 (54·4-57·6)	58·5 (56·6-60·5)	61·0 (58·1-63·8)
Mauritania	53·5 (50·8-56·4)	53·7 (51·1-56·4)	56·4 (54·3-58·7)	57·9 (56·3-59·6)	59·5 (58·1-61·1)	61·5 (60·0-63·0)	64·1 (62·6-65·5)	66·0 (64·3-67·8)	68·5 (66·4-70·7)	70·3 (67·8-73·2)
Niger	41·9 (39·4-44·4)	41·7 (39·7-43·7)	42·7 (41·1-44·3)	43·7 (42·1-45·4)	46·2 (44·6-47·7)	48·6 (47·2-50·3)	51·7 (50·2-53·4)	54·8 (53·2-56·6)	57·8 (55·9-59·8)	60·6 (58·2-62·9)
Nigeria	49·2 (46·6-52·0)	51·6 (49·2-54·0)	53·6 (51·4-55·8)	54·7 (52·9-56·6)	54·0 (52·0-55·9)	54·0 (51·8-56·3)	54·1 (52·1-56·3)	56·1 (54·3-58·1)	59·6 (57·6-61·6)	63·7 (61·2-66·2)
Sao Tome and Principe	56·3 (55·1-57·4)	59·5 (58·2-60·7)	61·0 (59·7-62·2)	60·5 (59·2-61·8)	61·9 (60·5-63·5)	63·2 (61·8-64·8)	64·0 (62·7-65·4)	65·0 (63·6-66·4)	67·9 (66·4-69·4)	69·0 (66·7-71·5)
Senegal	46·1 (44·7-47·5)	47·8 (46·6-49·1)	50·9 (49·6-52·1)	53·8 (52·7-54·9)	56·0 (55·0-56·9)	56·9 (55·9-57·9)	58·1 (57·0-59·3)	60·1 (59·1-61·3)	62·4 (61·3-63·5)	64·6 (63·3-66·0)
Sierra Leone	43·7 (40·6-46·4)	45·4 (42·9-47·7)	46·3 (44·3-48·2)	47·2 (45·4-48·8)	48·4 (46·8-50·0)	47·3 (45·9-48·8)	49·1 (47·8-50·4)	51·1 (49·9-52·4)	54·1 (52·7-55·5)	58·1 (56·4-59·8)
Togo	50·1 (48·1-51·9)	52·5 (51·0-54·1)	54·6 (53·3-55·9)	55·4 (54·2-56·7)	56·1 (54·9-57·4)	55·3 (54·0-56·7)	54·3 (52·7-55·9)	54·3 (52·7-55·9)	56·5 (55·0-58·1)	60·1 (58·2-61·7)
Eastern Sub-Saharan Africa	47·0 (45·7-48·5)	48·1 (47·0-49·3)	49·1 (48·1-50·2)	49·4 (48·5-50·4)	49·5 (48·8-50·2)	49·6 (48·9-50·3)	50·3 (49·7-51·0)	53·8 (53·2-54·5)	57·7 (56·9-58·5)	61·7 (60·6-62·8)
Burundi	43·7 (41·5-46·1)	44·1 (42·0-46·6)	45·2 (43·0-47·6)	46·5 (44·3-48·9)	46·7 (44·4-49·2)	45·4 (43·4-47·4)	46·9 (45·0-48·8)	52·3 (50·4-54·2)	56·8 (54·5-59·1)	59·2 (56·0-62·3)
Comoros	49·4 (46·0-52·6)	51·4 (48·8-53·9)	53·2 (50·7-55·4)	55·5 (53·2-57·7)	57·8 (55·9-59·7)	59·3 (57·5-61·0)	61·0 (59·4-62·7)	63·7 (62·2-65·2)	65·1 (63·4-66·9)	66·6 (64·4-68·8)

Appendix Table 13B. Estimates of life expectancy at birth by location, males, 1970, 1975, 1980, 1985, 1990, 1995, 2000, 2005, 2010, 2016

Location	1970	1975	1980	1985	1990	1995	2000	2005	2010	2016
Djibouti	57·1 (55·0-59·3)	58·2 (56·3-60·1)	58·5 (56·9-60·2)	58·5 (57·1-59·9)	58·4 (57·2-59·7)	58·7 (57·3-60·2)	59·0 (57·1-61·0)	59·8 (57·7-61·8)	61·6 (59·5-63·6)	64·7 (62·5-66·7)
Eritrea	43·2 (41·0-45·7)	45·0 (43·4-46·8)	46·4 (45·0-48·0)	47·9 (46·5-49·4)	51·0 (49·5-52·5)	55·7 (54·0-57·3)	56·9 (55·1-58·6)	59·7 (57·7-61·7)	60·9 (58·8-63·1)	62·9 (60·6-65·1)
Ethiopia	41·4 (39·0-44·1)	41·2 (39·1-43·5)	42·2 (40·2-44·2)	43·7 (42·2-45·4)	44·4 (43·2-45·7)	49·0 (47·6-50·4)	50·4 (49·0-51·8)	56·0 (54·3-57·4)	60·7 (58·5-62·7)	64·7 (62·1-67·6)
Kenya	57·7 (56·9-58·4)	59·5 (58·8-60·3)	61·3 (60·6-62·1)	62·1 (61·4-62·8)	60·2 (59·4-61·1)	55·6 (54·8-56·6)	53·7 (53·0-54·6)	56·1 (55·4-56·7)	61·2 (60·6-61·9)	64·7 (63·9-65·6)
Madagascar	50·4 (48·3-52·3)	52·4 (50·4-54·4)	53·8 (52·0-55·7)	53·2 (51·7-54·9)	54·4 (53·1-55·7)	55·9 (54·5-57·3)	57·6 (56·1-59·0)	59·1 (57·3-60·9)	59·8 (57·3-62·5)	61·5 (58·4-64·8)
Malawi	42·7 (40·2-45·2)	45·4 (43·4-47·5)	48·3 (46·5-50·1)	49·1 (46·9-51·1)	47·6 (44·2-51·2)	44·7 (41·8-48·1)	43·6 (41·1-46·3)	46·5 (44·3-49·0)	51·2 (49·2-53·5)	57·9 (55·3-60·5)
Mozambique	46·2 (44·0-48·4)	48·3 (46·6-50·0)	49·1 (47·8-50·5)	42·8 (39·3-46·7)	48·5 (47·3-49·7)	49·1 (47·4-50·7)	49·1 (47·0-51·1)	50·1 (47·9-52·4)	52·2 (50·0-54·3)	57·0 (54·9-59·2)
Rwanda	45·7 (43·3-48·3)	46·5 (44·1-48·7)	49·3 (47·3-51·4)	51·7 (49·9-53·4)	47·5 (45·5-49·5)	41·8 (39·8-43·8)	47·5 (46·0-49·3)	56·2 (54·9-57·9)	63·0 (61·5-64·5)	66·0 (63·9-67·9)
Somalia	48·2 (45·0-51·3)	46·3 (42·8-49·7)	49·4 (46·7-52·2)	49·2 (46·8-51·7)	49·6 (47·5-51·7)	50·7 (48·6-52·9)	51·6 (49·4-53·7)	53·5 (51·2-55·7)	52·4 (49·6-55·2)	56·6 (54·1-59·1)
South Sudan	47·6 (42·5-52·2)	48·8 (44·2-52·7)	49·7 (46·5-53·0)	50·0 (47·5-52·6)	49·9 (47·4-52·6)	52·0 (49·5-54·6)	53·2 (50·7-55·9)	55·6 (53·0-58·4)	57·2 (54·6-60·1)	58·7 (56·1-61·5)
Tanzania	51·6 (50·0-53·1)	53·4 (52·0-54·8)	54·8 (53·6-56·1)	55·5 (54·3-56·7)	53·7 (52·1-55·3)	51·8 (49·7-53·9)	52·1 (50·3-54·1)	54·6 (53·1-56·4)	58·3 (56·7-59·9)	62·6 (60·5-64·5)
Uganda	48·8 (47·2-50·5)	49·9 (48·4-51·5)	47·1 (44·6-49·9)	47·4 (45·0-49·9)	46·3 (43·4-48·9)	44·0 (41·6-46·7)	46·3 (44·7-48·0)	50·9 (49·5-52·3)	55·0 (53·5-56·4)	59·8 (58·0-61·4)
Zambia	55·8 (51·7-60·2)	57·8 (54·4-61·8)	58·0 (55·2-61·4)	56·8 (54·1-59·7)	52·2 (49·4-54·8)	45·8 (42·8-48·8)	43·3 (40·9-45·9)	44·6 (42·4-46·7)	50·0 (47·8-52·3)	55·6 (52·5-59·3)
Central Sub-Saharan Africa	48·3 (45·2-51·3)	49·5 (47·0-51·9)	50·4 (48·7-52·1)	51·4 (50·0-52·7)	50·8 (49·7-52·0)	50·8 (49·7-51·9)	51·4 (50·2-52·7)	54·1 (53·0-55·2)	56·9 (55·7-58·2)	60·6 (58·9-62·3)
Angola	45·8 (41·7-50·0)	46·6 (42·9-50·6)	47·1 (43·9-50·5)	47·6 (44·9-50·7)	48·3 (45·6-51·1)	50·8 (48·0-53·9)	52·7 (49·9-55·9)	56·2 (53·3-59·1)	59·8 (56·8-62·8)	63·9 (60·3-67·1)
Central African Republic	46·6 (43·7-49·6)	46·6 (44·0-49·2)	47·5 (45·2-49·9)	47·3 (45·2-49·3)	45·4 (43·2-47·7)	43·4 (40·8-46·2)	42·1 (39·1-45·1)	42·9 (40·0-46·0)	45·3 (42·2-48·5)	47·9 (44·5-51·6)
Congo	49·1 (44·7-53·2)	51·1 (47·3-54·9)	52·9 (49·9-56·1)	54·1 (51·5-56·7)	52·2 (50·0-54·6)	51·2 (49·2-53·3)	52·2 (50·3-54·3)	57·3 (55·4-58·9)	61·6 (59·3-64·3)	63·6 (60·1-67·3)
Democratic Republic of the Congo	49·7 (45·8-53·4)	51·0 (47·8-53·8)	51·9 (49·5-54·1)	53·1 (51·3-54·8)	52·3 (50·7-53·9)	51·7 (50·0-53·1)	51·9 (50·2-53·5)	54·3 (52·9-55·7)	56·7 (55·0-58·3)	60·4 (58·3-62·4)
Equatorial Guinea	42·7 (38·0-47·3)	44·4 (40·7-48·1)	46·8 (43·8-50·0)	47·5 (44·7-50·5)	46·5 (43·7-49·7)	46·6 (43·5-50·0)	53·5 (49·8-57·3)	57·5 (53·6-62·1)	60·7 (56·6-65·3)	64·5 (60·2-69·1)
Gabon	48·8 (45·2-52·5)	51·7 (48·6-55·1)	54·2 (51·6-57·1)	55·5 (53·5-57·7)	56·0 (54·3-57·8)	56·4 (54·9-58·3)	57·5 (55·5-59·7)	59·1 (56·7-61·1)	61·6 (59·1-64·0)	64·9 (61·4-68·4)

Appendix Table 13C: Estimates of life expectancy at birth by location, females, 1970, 1975, 1980, 1985, 1990, 1995, 2000, 2005, 2010, 2016

Location	1970	1975	1980	1985	1990	1995	2000	2005	2010	2016
Global	60·5 (60·2–60·9)	62·6 (62·3–62·9)	64·4 (64·1–64·7)	66·0 (65·8–66·3)	67·6 (67·3–67·8)	68·3 (68·1–68·5)	69·4 (69·2–69·6)	71·0 (70·8–71·2)	73·0 (72·8–73·3)	75·3 (75·0–75·6)
High SDI	74·2 (73·9–74·4)	75·6 (75·5–75·8)	77·1 (77·0–77·2)	78·1 (78·0–78·2)	79·1 (79·0–79·2)	80·0 (79·9–80·1)	80·9 (80·9–81·0)	81·9 (81·8–82·0)	82·9 (82·8–83·0)	83·4 (83·2–83·6)
High-middle SDI	68·1 (67·6–68·6)	69·9 (69·5–70·4)	71·1 (70·6–71·5)	72·4 (72·0–72·8)	73·6 (73·2–73·9)	73·4 (73·1–73·8)	74·9 (74·5–75·3)	76·2 (75·7–76·5)	78·2 (77·8–78·5)	79·8 (78·6–80·8)
Middle SDI	60·4 (59·8–61·0)	63·2 (62·7–63·6)	65·2 (64·7–65·6)	67·3 (66·9–67·6)	69·1 (68·8–69·4)	70·6 (70·3–70·8)	71·9 (71·7–72·1)	73·4 (73·2–73·6)	75·3 (75·1–75·5)	77·4 (77·1–77·7)
Low-middle SDI	50·7 (50·1–51·3)	53·2 (52·6–53·9)	56·0 (55·5–56·5)	58·0 (57·6–58·4)	60·0 (59·5–60·4)	61·2 (60·8–61·6)	62·6 (62·3–63·0)	64·6 (64·2–65·0)	67·2 (66·8–67·6)	69·9 (69·4–70·4)
Low SDI	48·4 (47·5–49·4)	49·8 (49·1–50·5)	51·2 (50·7–51·8)	52·4 (51·8–53·0)	53·7 (53·3–54·2)	54·1 (53·6–54·6)	54·9 (54·5–55·4)	57·3 (56·8–57·8)	59·9 (59·1–60·6)	64·3 (63·5–65·0)
High-income	74·0 (73·7–74·2)	75·4 (75·3–75·6)	77·1 (77·0–77·2)	78·3 (78·2–78·4)	79·3 (79·2–79·4)	80·2 (80·1–80·3)	81·1 (81·0–81·2)	82·1 (82·0–82·2)	83·0 (82·9–83·1)	83·5 (83·3–83·7)
High-income North America	74·8 (74·7–74·9)	76·4 (76·3–76·5)	77·8 (77·7–77·9)	78·4 (78·3–78·5)	79·0 (78·9–79·1)	79·3 (79·2–79·4)	79·8 (79·7–79·9)	80·4 (80·3–80·5)	81·4 (81·3–81·5)	81·5 (81·3–81·7)
Canada	76·2 (75·9–76·5)	77·3 (77·0–77·6)	78·9 (78·5–79·2)	79·8 (79·5–80·2)	80·6 (80·3–80·9)	81·0 (80·7–81·3)	81·8 (81·5–82·1)	82·5 (82·3–82·8)	83·4 (83·2–83·7)	83·9 (83·5–84·3)
Greenland	66·6 (65·4–67·8)	64·5 (63·4–65·7)	65·6 (64·4–66·8)	64·5 (63·3–65·6)	66·1 (64·7–67·5)	67·2 (65·9–68·5)	67·5 (66·4–68·7)	68·2 (67·1–69·5)	70·3 (69·2–71·6)	72·8 (70·4–75·6)
United States	74·7 (74·6–74·8)	76·3 (76·2–76·4)	77·7 (77·6–77·8)	78·2 (78·1–78·3)	78·9 (78·8–79·0)	79·2 (79·1–79·3)	79·6 (79·5–79·7)	80·2 (80·1–80·3)	81·2 (81·1–81·3)	81·2 (81·0–81·5)
Australasia	74·6 (74·4–74·8)	76·1 (75·8–76·3)	77·8 (77·6–78·0)	78·6 (78·4–78·8)	79·8 (79·6–80·1)	80·9 (80·7–81·2)	82·1 (81·9–82·3)	83·2 (83·0–83·4)	83·9 (83·7–84·1)	84·4 (83·8–85·0)
Australia	74·6 (74·3–74·9)	76·2 (75·9–76·5)	78·1 (77·8–78·4)	78·9 (78·7–79·2)	80·1 (79·9–80·4)	81·2 (81·0–81·5)	82·4 (82·1–82·6)	83·4 (83·2–83·7)	84·2 (83·9–84·4)	84·6 (83·8–85·3)
New Zealand	74·7 (74·3–75·0)	75·4 (75·0–75·7)	76·3 (76·0–76·7)	77·2 (76·9–77·6)	78·5 (78·2–78·9)	79·7 (79·4–80·0)	81·0 (80·7–81·3)	81·9 (81·6–82·2)	82·8 (82·5–83·1)	83·4 (82·3–84·6)
High-income Asia Pacific	72·6 (71·3–73·7)	74·9 (74·1–75·6)	77·2 (76·9–77·5)	79·2 (78·8–79·5)	80·7 (80·4–81·0)	82·0 (81·7–82·2)	83·5 (83·2–83·7)	84·7 (84·5–85·0)	85·7 (85·5–85·9)	86·4 (85·6–87·1)
Brunei	66·2 (64·6–68·3)	70·3 (68·9–72·2)	71·1 (68·7–73·1)	74·0 (72·5–75·1)	75·4 (73·9–76·7)	76·3 (74·6–77·6)	78·2 (77·2–79·3)	78·9 (77·9–79·7)	79·3 (78·3–80·0)	79·5 (77·8–81·7)
Japan	75·0 (74·6–75·4)	76·9 (76·6–77·1)	78·8 (78·8–78·9)	80·5 (80·5–80·6)	81·8 (81·8–81·9)	82·9 (82·8–83·0)	84·4 (84·3–84·4)	85·4 (85·3–85·5)	86·2 (86·1–86·3)	86·9 (86·7–87·2)
Singapore	71·8 (70·4–73·4)	73·6 (72·2–75·1)	74·4 (73·0–75·7)	75·9 (74·5–77·4)	78·1 (76·5–79·7)	79·8 (78·3–81·4)	81·7 (80·3–83·1)	83·3 (81·9–84·6)	85·2 (84·0–86·4)	86·1 (83·9–88·4)
South Korea	66·0 (60·5–71·9)	68·3 (64·9–71·9)	72·0 (70·5–73·4)	74·3 (72·8–75·8)	76·3 (75·0–77·7)	78·1 (76·9–79·3)	79·6 (78·6–80·7)	81·7 (80·7–82·8)	83·5 (82·4–84·4)	84·2 (81·2–87·1)
Western Europe	74·6 (74·4–74·8)	75·8 (75·6–76·0)	77·3 (77·1–77·4)	78·4 (78·2–78·6)	79·5 (79·3–79·6)	80·5 (80·4–80·7)	81·5 (81·4–81·7)	82·6 (82·4–82·7)	83·5 (83·3–83·6)	84·1 (83·8–84·4)
Andorra	80·0 (76·5–83·7)	81·1 (78·3–84·6)	82·1 (79·5–85·1)	82·3 (79·9–85·3)	82·8 (80·3–85·3)	83·4 (80·9–85·7)	85·0 (82·5–87·0)	85·9 (83·7–87·5)	85·9 (83·5–87·6)	85·8 (83·0–87·7)
Austria	73·5 (73·2–73·7)	74·8 (74·5–75·0)	76·1 (75·8–76·3)	77·3 (77·1–77·6)	78·9 (78·6–79·1)	80·0 (79·7–80·2)	81·2 (80·9–81·4)	82·3 (82·0–82·5)	83·2 (82·9–83·4)	83·9 (83·2–84·5)
Belgium	74·1 (73·7–74·5)	75·3 (74·8–75·7)	76·6 (76·2–77·0)	77·8 (77·4–78·2)	79·3 (78·8–79·7)	80·2 (79·8–80·5)	80·9 (80·5–81·3)	82·0 (81·6–82·4)	82·6 (82·2–83·0)	83·4 (82·3–84·5)
Cyprus	72·2 (71·6–72·7)	72·9 (72·3–73·5)	74·4 (74·0–74·9)	75·9 (75·5–76·4)	77·0 (76·5–77·4)	78·0 (77·5–78·4)	79·3 (78·8–79·7)	80·5 (80·1–81·0)	82·3 (81·8–82·8)	82·8 (82·3–83·4)
Denmark	75·9 (75·2–76·6)	76·9 (76·2–77·7)	77·4 (76·7–78·1)	77·6 (76·8–78·4)	78·0 (77·1–78·8)	78·2 (77·5–79·0)	79·2 (78·5–80·0)	80·3 (79·6–81·1)	81·5 (80·8–82·2)	82·8 (81·5–84·2)
Finland	74·1 (73·8–74·5)	76·0 (75·6–76·3)	77·9 (77·5–78·2)	78·6 (78·3–79·0)	79·0 (78·7–79·3)	80·3 (80·0–80·7)	81·2 (80·9–81·5)	82·3 (82·0–82·7)	83·2 (82·9–83·5)	84·6 (83·9–85·4)
France	76·2 (75·9–76·5)	77·2 (76·9–77·5)	78·7 (78·4–79·0)	79·9 (79·6–80·2)	81·2 (80·9–81·5)	82·1 (81·9–82·4)	82·8 (82·5–83·1)	83·9 (83·6–84·1)	84·7 (84·4–85·0)	85·4 (84·9–86·0)
Germany	73·6 (73·1–74·2)	74·7 (74·1–75·2)	76·2 (75·6–76·7)	77·4 (76·9–78·0)	78·5 (78·0–79·1)	79·7 (79·2–80·2)	80·9 (80·4–81·5)	81·9 (81·5–82·4)	82·6 (82·2–83·2)	83·3 (82·5–84·3)
Greece	75·5 (75·1–75·8)	76·8 (76·4–77·1)	79·3 (79·0–79·7)	78·7 (78·4–79·0)	79·5 (79·2–79·9)	80·6 (80·3–80·9)	81·5 (81·2–81·7)	82·4 (82·1–82·7)	83·1 (82·9–83·4)	83·5 (82·8–84·3)
Iceland	76·8 (76·2–77·4)	78·2 (77·6–78·8)	79·7 (79·1–80·3)	80·3 (79·7–80·8)	80·4 (79·8–81·0)	80·6 (80·0–81·2)	81·9 (81·3–82·4)	83·0 (82·4–83·6)	83·9 (83·4–84·4)	84·0 (82·9–85·0)
Ireland	73·5 (72·9–74·2)	74·3 (73·6–74·9)	75·5 (74·9–76·2)	76·7 (76·1–77·4)	77·6 (77·0–78·3)	78·5 (77·9–79·2)	79·3 (78·7–79·9)	81·2 (80·7–81·8)	82·6 (82·0–83·1)	83·3 (82·0–84·7)
Israel	71·9 (70·2–73·5)	73·4 (72·2–74·5)	75·5 (74·3–76·7)	76·4 (75·3–77·5)	77·6 (76·4–78·6)	78·9 (77·9–80·0)	80·6 (79·5–81·7)	81·8 (80·7–82·9)	83·5 (82·5–84·5)	84·1 (82·2–85·9)
Italy	74·5 (74·2–74·8)	76·0 (75·7–76·3)	77·5 (77·2–77·7)	78·9 (78·6–79·1)	80·3 (80·0–80·5)	81·3 (81·1–81·5)	82·5 (82·3–82·8)	83·6 (83·4–83·8)	84·2 (84·0–84·5)	84·6 (84·0–85·3)
Luxembourg	73·6 (73·2–74·2)	74·8 (74·3–75·3)	76·1 (75·6–76·6)	77·3 (76·8–77·8)	78·5 (78·0–79·0)	80·1 (79·6–80·6)	81·6 (81·1–82·1)	82·6 (82·1–83·1)	83·5 (83·1–84·0)	83·9 (82·8–85·3)
Malta	71·7 (70·6–72·7)	72·8 (71·8–73·8)	73·6 (72·6–74·6)	75·6 (74·5–76·6)	77·8 (76·7–78·9)	79·4 (78·4–80·5)	79·8 (78·8–80·8)	81·3 (80·2–82·2)	82·5 (81·5–83·5)	83·8 (82·1–85·8)
Netherlands	76·5 (76·0–77·0)	77·8 (77·3–78·2)	79·1 (78·6–79·6)	79·7 (79·2–80·1)	80·1 (79·6–80·6)	80·3 (79·8–80·7)	80·6 (80·2–81·0)	81·6 (81·2–82·0)	82·7 (82·2–83·2)	83·6 (82·6–84·5)
Norway	77·1 (76·6–77·5)	78·0 (77·5–78·5)	79·1 (78·6–79·6)	79·5 (79·1–80·0)	80·1 (79·6–80·5)	80·8 (80·3–81·2)	81·4 (80·9–81·8)	82·4 (81·9–82·9)	83·2 (82·7–83·7)	84·1 (82·9–85·3)
Portugal	70·6 (70·3–70·9)	73·0 (72·7–73·4)	75·1 (74·8–75·4)	76·7 (76·4–77·1)	77·9 (77·6–78·2)	79·0 (78·7–79·4)	80·2 (79·9–80·6)	81·7 (81·4–82·1)	83·1 (82·7–83·3)	84·0 (83·4–84·8)
Spain	74·7 (74·5–74·9)	76·4 (76·1–76·6)	78·5 (78·3–78·7)	79·7 (79·5–79·9)	80·6 (80·4–80·8)	81·8 (81·6–82·0)	82·8 (82·7–83·0)	83·7 (83·5–83·9)	84·9 (84·7–85·1)	85·6 (85·1–86·0)
Sweden	77·0 (76·3–77·8)	78·0 (77·4–78·6)	78·9 (78·5–79·3)	79·9 (79·5–80·3)	80·4 (80·1–80·8)	81·4 (81·0–81·8)	82·0 (81·6–82·3)	82·6 (82·3–82·9)	83·5 (83·2–83·9)	84·0 (82·6–85·2)
Stockholm	76·3 (74·0–78·9)	77·4 (75·6–79·4)	78·9 (78·1–79·7)	80·1 (79·5–80·7)	80·2 (79·6–80·8)	81·3 (80·8–81·9)	81·9 (81·3–82·4)	82·8 (82·3–83·4)	83·8 (83·2–84·4)	84·2 (82·2–86·5)
Sweden except Stockholm	77·2 (76·5–77·8)	78·1 (77·5–78·7)	78·9 (78·5–79·4)	79·9 (79·5–80·3)	80·5 (80·1–80·9)	81·4 (81·0–81·8)	82·0 (81·6–82·4)	82·6 (82·2–83·0)	83·5 (83·1–83·9)	83·9 (82·3–85·3)
Switzerland	76·0 (75·2–76·7)	78·0 (77·2–78·7)	78·9 (78·2–79·6)	80·2 (79·5–80·9)	80·9 (80·2–81·5)	81·6 (80·9–82·2)	82·6 (81·9–83·3)	83·7 (83·1–84·2)	84·4 (83·8–84·9)	85·2 (82·6–87·6)
United Kingdom	75·0 (74·7–75·2)	75·6 (75·4–75·8)	76·6 (76·4–76·7)	77·5 (77·4–77·6)	78·5 (78·3–78·6)	79·3 (79·2–79·4)	80·2 (80·1–80·4)	81·2 (81·1–81·4)	82·3 (82·2–82·5)	82·9 (82·6–83·1)
England	75·2 (75·0–75·4)	75·9 (75·7–76·0)	76·8 (76·7–76·9)	77·7 (77·6–77·8)	78·7 (78·6–78·8)	79·5 (79·4–79·6)	80·4 (80·4–80·5)	81·5 (81·4–81·6)	82·6 (82·5–82·7)	83·1 (83·0–83·3)
East Midlands	75·1 (74·3–75·9)	75·8 (75·2–76·4)	76·8 (76·4–77·3)	77·8 (77·4–78·1)	78·6 (78·3–78·9)	79·5 (79·1–79·8)	80·4 (80·1–80·7)	81·3 (81·0–81·6)	82·4 (82·1–82·8)	82·9 (82·4–83·4)
East of England	76·0 (75·3–76·7)	76·7 (76·1–77·4)	77·7 (77·2–78·1)	78·5 (78·2–78·8)	79·5 (79·2–79·9)	80·3 (80·0–80·7)	81·2 (80·9–81·5)	82·2 (81·9–82·5)	83·2 (82·9–83·5)	83·7 (83·2–84·2)
Greater London	75·7 (75·3–76·1)	76·2 (75·8–76·5)	77·1 (76·9–77·4)	78·0 (77·8–78·2)	78·9 (78·7–79·1)	79·6 (79·4–79·8)	80·5 (80·3–80·7)	81·8 (81·6–81·9)	83·2 (83·0–83·4)	83·9 (83·6–84·2)
North East England	73·8 (73·1–74·4)	74·5 (73·9–75·1)	75·5 (75·1–75·9)	76·2 (75·9–76·5)	77·2 (76·9–77·5)	77·9 (77·6–78·3)	79·2 (78·9–79·5)	80·1 (79·8–80·4)	81·3 (80·9–81·6)	81·7 (81·2–82·2)
North West England	74·0 (73·5–74·6)	74·7 (74·2–75·2)	75·6 (75·3–75·9)	76·6 (76·3–76·8)	77·5 (77·3–77·7)	78·3 (78·1–78·5)	79·3 (79·0–79·5)	80·2 (80·0–80·5)	81·3 (81·1–81·6)	81·9 (81·5–82·2)
South East England	76·4 (75·8–77·0)	77·0 (76·5–77·5)	77·9 (77·5–78·3)	78·7 (78·4–78·9)	79·6 (79·4–79·9)	80·4 (80·2–80·7)	81·3 (81·1–81·6)	82·3 (82·1–82·6)	83·5 (83·2–83·7)	83·9 (83·5–84·3)
South West England	76·2 (75·5–76·7)	76·8 (76·3–77·3)	77·7 (77·3–78·1)	78·7 (78·4–79·0)	79·8 (79·6–80·1)	80·6 (80·4–80·9)	81·5 (81·2–81·7)	82·4 (82·2–82·7)	83·5 (83·2–83·7)	83·8 (83·4–84·2)
West Midlands	74·7 (74·0–75·4)	75·4 (74·8–76·1)	76·4 (75·9–76·8)	77·2 (77·0–77·6)	78·2 (78·0–78·5)	79·2 (78·9–79·5)	80·0 (79·7–80·4)	81·1 (80·8–81·4)	82·3 (82·0–82·6)	82·8 (82·3–83·2)
Yorkshire and the Humber	74·5 (73·9–75·2)	75·2 (74·7–75·7)	76·2 (75·8–76·5)	77·1 (76·8–77·3)	78·2 (77·9–78·4)	79·0 (78·7–79·2)	80·0 (79·7–80·3)	80·9 (80·7–81·1)	81·9 (81·7–82·2)	82·4 (82·0–82·8)
Northern Ireland	73·7 (73·0–74·4)	74·3 (73·6–75·0)	75·5 (74·7–76·2)	76·5 (75·5–77·4)	77·9 (77·0–78·8)	78·8 (77·9–79·9)	80·0 (79·0–80·9)	80·9 (80·0–81·8)	81·9 (81·1–82·8)	82·4 (80·8–84·0)
Scotland	73·6 (73·0–74·2)	74·2 (73·5–74·8)	75·1 (74·5–75·8)	75·9 (75·0–76·8)	76·8 (75·9–77·7)	77·6 (76·8–78·5)	78·6 (77·8–79·6)	79·5 (78·5–80·4)	80·5 (79·7–81·3)	81·2 (79·7–82·6)

Appendix Table 13C: Estimates of life expectancy at birth by location, females, 1970, 1975, 1980, 1985, 1990, 1995, 2000, 2005, 2010, 2016

Location	1970	1975	1980	1985	1990	1995	2000	2005	2010	2016
Wales	74·4 (72·3-76·4)	75·1 (73·2-76·8)	76·2 (74·9-77·4)	77·4 (76·5-78·3)	78·4 (77·5-79·2)	79·0 (78·2-79·8)	79·9 (79·0-80·7)	80·9 (80·2-81·7)	81·7 (80·9-82·6)	82·2 (80·8-83·5)
Southern Latin America	68·5 (68·0-69·0)	70·8 (70·1-71·5)	73·4 (73·1-73·8)	75·1 (74·7-75·4)	76·1 (75·8-76·5)	77·5 (77·1-77·8)	78·5 (78·2-78·9)	79·3 (79·0-79·7)	79·9 (79·5-80·2)	80·9 (79·9-81·8)
Argentina	68·9 (68·2-69·5)	70·9 (70·0-71·7)	73·4 (72·9-73·9)	74·8 (74·4-75·3)	75·8 (75·3-76·3)	76·9 (76·5-77·5)	77·9 (77·5-78·4)	78·6 (78·2-79·1)	79·1 (78·6-79·6)	80·0 (79·1-80·9)
Chile	66·6 (65·7-67·4)	70·2 (69·3-71·1)	73·4 (72·6-74·2)	75·5 (74·7-76·3)	76·8 (76·1-77·7)	78·6 (77·9-79·4)	80·2 (79·6-80·9)	81·2 (80·5-81·9)	82·1 (81·4-82·7)	83·2 (80·2-86·1)
Uruguay	72·0 (71·6-72·5)	72·3 (71·9-72·7)	73·8 (73·4-74·1)	75·2 (74·8-75·6)	76·5 (76·1-76·9)	77·5 (77·1-77·9)	78·7 (78·4-79·1)	79·4 (79·0-79·7)	80·2 (79·8-80·6)	81·1 (80·2-81·9)
Central Europe, Eastern Europe, and Central Asia	71·4 (70·8-72·0)	72·2 (71·7-72·7)	72·4 (71·9-72·9)	72·8 (72·3-73·2)	73·6 (73·1-74·1)	71·9 (71·3-72·4)	73·0 (72·5-73·5)	73·7 (73·1-74·2)	75·8 (75·3-76·3)	77·2 (75·0-78·9)
Eastern Europe	73·4 (72·6-74·2)	73·3 (72·5-74·0)	73·1 (72·3-73·9)	73·4 (72·6-74·1)	74·4 (73·6-75·2)	71·7 (70·9-72·6)	72·6 (71·9-73·4)	72·9 (72·0-73·8)	75·3 (74·5-76·2)	76·6 (73·2-79·7)
Belarus	75·0 (74·2-75·8)	75·8 (75·1-76·5)	75·4 (74·8-76·1)	75·1 (74·5-75·8)	75·4 (74·8-76·0)	74·0 (73·4-74·6)	74·3 (73·8-74·9)	74·9 (74·3-75·5)	76·4 (75·8-76·9)	78·8 (76·6-81·0)
Estonia	74·7 (74·2-75·2)	74·8 (74·2-75·3)	74·4 (73·9-74·9)	74·4 (73·8-74·9)	75·3 (74·8-75·8)	74·2 (73·7-74·8)	76·1 (75·6-76·7)	78·2 (77·7-78·7)	80·9 (80·4-81·4)	81·8 (80·7-83·5)
Latvia	74·3 (73·7-74·9)	74·3 (73·7-75·0)	74·1 (73·5-74·7)	74·3 (73·6-74·8)	74·6 (74·0-75·2)	72·9 (72·1-73·5)	75·9 (75·4-76·6)	76·4 (75·8-77·1)	78·2 (77·6-78·7)	79·8 (78·2-81·3)
Lithuania	75·2 (74·7-75·7)	75·6 (75·1-76·1)	75·8 (75·3-76·2)	75·5 (75·0-75·9)	76·4 (75·9-76·8)	74·9 (74·4-75·4)	77·5 (77·0-77·9)	77·4 (77·0-77·9)	78·8 (78·4-79·3)	80·3 (79·4-81·3)
Moldova	65·8 (62·4-69·4)	67·3 (64·6-70·2)	68·3 (66·4-70·2)	68·6 (67·2-70·0)	70·8 (69·7-71·9)	68·6 (67·5-69·7)	71·4 (70·3-72·4)	72·6 (71·6-73·6)	73·9 (72·9-74·9)	76·1 (74·6-77·8)
Russia	73·5 (72·6-74·7)	73·2 (72·0-74·2)	72·9 (71·7-74·1)	73·2 (72·1-74·2)	74·3 (73·2-75·5)	71·4 (70·1-72·6)	72·2 (71·0-73·3)	72·5 (71·2-73·8)	75·1 (73·9-76·3)	76·2 (71·4-80·7)
Ukraine	73·1 (71·8-74·3)	73·5 (72·5-74·5)	73·4 (72·5-74·3)	73·9 (73·1-74·6)	74·6 (73·9-75·3)	72·2 (71·5-73·0)	73·2 (72·4-73·9)	73·2 (72·4-73·9)	75·4 (74·7-76·2)	77·0 (73·5-80·5)
Central Europe	71·9 (71·5-72·2)	73·3 (72·9-73·6)	73·6 (73·4-73·9)	74·1 (73·9-74·3)	74·8 (74·6-75·0)	75·2 (75·0-75·4)	76·6 (76·4-76·8)	77·8 (77·6-78·0)	79·1 (78·9-79·3)	80·3 (79·8-80·7)
Albania	68·8 (65·4-71·6)	72·1 (69·4-74·3)	74·2 (72·5-75·9)	75·3 (74·3-76·2)	76·8 (76·2-77·4)	77·5 (76·8-78·0)	77·5 (76·9-78·0)	77·6 (77·0-78·1)	79·0 (78·2-79·8)	80·7 (79·4-82·1)
Bosnia and Herzegovina	69·4 (66·0-72·5)	71·1 (68·4-73·9)	72·6 (70·5-75·0)	73·8 (72·2-75·7)	75·1 (73·8-76·7)	73·0 (71·5-74·7)	77·6 (76·1-79·2)	78·3 (77·0-79·7)	79·3 (78·0-80·6)	80·2 (78·4-82·1)
Bulgaria	73·2 (72·8-73·6)	73·7 (73·3-74·1)	73·9 (73·6-74·3)	74·4 (74·1-74·8)	74·7 (74·4-75·1)	74·8 (74·4-75·2)	75·1 (74·8-75·5)	76·2 (75·9-76·6)	77·4 (77·0-77·7)	78·5 (76·5-80·4)
Croatia	71·5 (68·4-74·8)	73·0 (69·6-76·1)	74·2 (71·1-76·8)	74·9 (73·7-76·1)	76·1 (75·2-76·9)	76·2 (75·3-77·2)	77·6 (76·8-78·4)	78·7 (77·9-79·5)	79·7 (79·0-80·4)	80·5 (79·0-82·1)
Czech Republic	73·2 (72·9-73·5)	74·0 (73·7-74·2)	74·1 (73·9-74·4)	74·7 (74·5-75·0)	75·6 (75·3-75·8)	76·9 (76·6-77·2)	78·4 (78·1-78·6)	79·4 (79·1-79·7)	80·6 (80·3-80·8)	81·9 (81·3-82·5)
Hungary	72·0 (71·4-72·7)	72·4 (71·9-73·0)	72·9 (72·2-73·5)	73·2 (72·5-73·8)	73·9 (73·2-74·6)	74·7 (74·1-75·4)	76·1 (75·5-76·7)	77·3 (76·7-78·0)	78·4 (77·8-78·9)	79·1 (77·7-80·3)
Macedonia	69·6 (66·5-72·0)	71·1 (68·4-73·1)	71·5 (69·8-73·0)	72·5 (71·2-73·7)	72·9 (72·1-73·7)	73·6 (72·9-74·2)	74·6 (74·0-75·2)	75·3 (74·6-76·0)	76·4 (75·8-77·0)	77·4 (76·6-78·1)
Montenegro	75·2 (73·6-76·7)	75·6 (74·0-77·4)	76·9 (75·4-78·2)	77·3 (75·6-78·4)	77·8 (76·6-78·8)	77·0 (76·1-77·9)	76·9 (76·1-77·7)	77·9 (77·3-78·6)	79·2 (78·3-80·0)	79·7 (78·7-81·2)
Poland	73·2 (72·8-73·7)	74·5 (74·0-74·9)	74·7 (74·3-75·0)	74·8 (74·4-75·3)	75·4 (75·0-75·9)	76·3 (75·9-76·7)	77·9 (77·5-78·4)	79·4 (78·9-79·8)	80·5 (80·1-80·9)	81·7 (80·6-82·8)
Romania	70·5 (70·0-71·1)	72·3 (71·7-72·9)	72·3 (71·7-72·8)	72·9 (72·3-73·4)	73·2 (72·6-73·8)	73·4 (72·8-74·0)	74·8 (74·2-75·4)	76·0 (75·5-76·6)	77·5 (77·0-78·2)	78·9 (77·7-80·1)
Serbia	72·2 (70·6-73·8)	73·6 (72·1-75·2)	74·5 (73·1-76·0)	75·1 (73·9-76·4)	75·6 (74·6-76·7)	73·9 (73·3-74·8)	74·9 (74·2-75·6)	75·9 (75·2-76·6)	77·3 (76·8-77·9)	78·8 (78·1-79·4)
Slovakia	73·0 (72·5-73·6)	74·0 (73·5-74·4)	74·4 (73·9-74·9)	74·8 (74·3-75·3)	75·4 (74·9-75·9)	76·4 (75·9-76·9)	77·4 (76·8-77·9)	78·1 (77·7-78·6)	79·3 (78·8-79·7)	80·4 (79·1-81·8)
Slovenia	72·0 (68·2-76·1)	73·4 (70·6-76·5)	74·6 (72·8-76·6)	75·8 (74·8-76·8)	77·5 (76·6-78·5)	78·4 (77·4-79·3)	79·8 (79·0-80·6)	81·1 (80·2-82·1)	82·7 (81·9-83·6)	83·8 (82·5-85·3)
Central Asia	65·3 (63·4-67·1)	67·5 (65·9-69·0)	69·2 (68·3-70·0)	70·3 (69·6-70·9)	71·0 (70·5-71·4)	69·3 (68·7-69·9)	70·1 (69·5-70·6)	70·8 (70·2-71·5)	72·9 (72·3-73·4)	75·1 (74·1-76·0)
Armenia	68·5 (65·1-71·6)	71·2 (68·6-74·0)	72·9 (71·4-74·4)	73·6 (72·7-74·5)	73·6 (72·9-74·5)	73·5 (72·6-74·3)	75·3 (74·6-76·1)	75·9 (75·0-76·7)	76·5 (75·6-77·2)	79·3 (78·0-80·7)
Azerbaijan	64·5 (60·7-68·1)	67·8 (64·8-70·9)	69·1 (67·0-71·2)	69·4 (68·0-70·8)	70·1 (68·9-71·4)	68·9 (67·6-70·2)	69·9 (68·6-71·0)	70·8 (69·5-72·3)	72·2 (70·9-73·6)	75·8 (73·5-77·9)
Georgia	68·5 (65·0-72·2)	71·1 (68·0-74·5)	73·3 (71·4-75·2)	73·7 (72·7-74·8)	73·7 (72·6-74·8)	74·5 (73·6-75·5)	75·6 (74·7-76·7)	76·4 (75·5-77·2)	77·7 (76·8-78·4)	78·8 (76·6-81·4)
Kazakhstan	68·0 (63·5-73·0)	69·3 (65·0-73·9)	70·8 (68·5-73·3)	72·0 (70·5-73·6)	72·6 (71·3-73·8)	69·6 (68·1-71·1)	70·2 (68·6-71·7)	70·7 (69·2-72·3)	73·4 (72·2-74·6)	76·2 (73·9-78·5)
Kyrgyzstan	63·3 (60·3-66·4)	65·9 (62·9-68·7)	68·3 (67·0-69·6)	69·1 (68·4-69·8)	70·1 (69·5-70·7)	68·5 (67·9-69·2)	70·6 (69·9-71·3)	70·7 (70·1-71·3)	72·6 (72·0-73·2)	74·9 (73·8-76·1)
Mongolia	55·7 (53·6-57·7)	59·7 (57·7-62·0)	61·0 (59·0-63·1)	63·1 (61·4-65·0)	65·2 (63·8-66·8)	64·6 (63·4-65·8)	65·7 (64·5-66·8)	68·2 (67·1-69·3)	70·4 (69·0-71·7)	73·0 (71·1-75·1)
Tajikistan	57·3 (53·3-61·1)	59·7 (56·8-62·4)	63·1 (60·9-65·3)	66·0 (64·2-67·8)	67·4 (65·8-69·1)	66·5 (64·7-68·2)	67·4 (66·0-69·1)	69·7 (68·2-71·0)	72·2 (70·5-73·8)	74·3 (72·4-76·1)
Turkmenistan	59·3 (55·5-62·7)	62·1 (59·0-65·1)	64·7 (62·6-66·5)	66·3 (64·9-67·6)	67·1 (66·1-68·1)	66·8 (65·7-67·7)	67·7 (66·5-68·8)	69·5 (68·2-70·6)	71·7 (70·5-72·8)	74·0 (72·7-75·1)
Uzbekistan	66·1 (63·4-68·7)	68·1 (65·7-70·4)	69·4 (67·7-71·1)	70·6 (69·4-71·7)	71·3 (70·3-72·2)	69·1 (68·1-70·0)	69·4 (68·4-70·4)	69·7 (68·6-70·8)	71·9 (70·9-72·8)	73·5 (71·9-75·1)
Latin America and Caribbean	63·5 (62·8-64·2)	66·6 (66·2-67·0)	69·1 (68·8-69·5)	70·5 (70·2-70·8)	72·7 (72·5-72·9)	73·9 (73·7-74·1)	75·6 (75·4-75·9)	76·8 (76·6-77·0)	76·7 (75·7-77·6)	78·9 (78·4-79·3)
Central Latin America	64·6 (64·2-65·0)	67·3 (66·9-67·7)	70·3 (69·9-70·6)	71·7 (71·4-72·0)	74·3 (74·0-74·5)	75·4 (75·1-75·7)	77·0 (76·7-77·2)	77·7 (77·5-78·0)	78·5 (78·2-78·8)	79·4 (79·0-79·9)
Colombia	68·1 (67·4-68·7)	68·7 (68·1-69·3)	71·5 (70·9-72·2)	71·4 (70·6-72·3)	75·1 (74·7-75·6)	75·8 (75·4-76·2)	77·1 (76·7-77·5)	78·1 (77·7-78·5)	79·4 (79·0-79·8)	81·0 (80·0-82·1)
Costa Rica	70·3 (69·7-70·9)	73·6 (73·0-74·1)	76·7 (76·1-77·2)	78·0 (77·5-78·6)	78·9 (78·3-79·4)	79·5 (79·0-80·1)	80·5 (80·1-81·0)	81·9 (81·4-82·3)	82·9 (82·4-83·4)	83·6 (82·6-84·5)
El Salvador	61·5 (60·2-62·8)	65·1 (64·1-66·2)	68·2 (67·1-69·3)	69·2 (68·2-70·1)	72·5 (71·8-73·2)	74·3 (73·6-75·0)	76·5 (75·8-77·1)	77·1 (76·5-77·8)	78·2 (77·5-78·8)	79·1 (77·6-80·5)
Guatemala	52·0 (49·6-54·4)	57·3 (55·1-59·7)	61·3 (59·1-63·5)	63·7 (61·9-65·7)	66·5 (64·8-68·4)	69·6 (67·7-71·4)	72·0 (69·9-74·2)	73·4 (71·5-75·3)	74·6 (72·5-76·8)	76·0 (72·4-79·8)
Honduras	58·2 (56·3-60·2)	61·6 (59·4-63·6)	64·5 (62·6-66·4)	65·2 (63·6-66·9)	67·2 (65·7-68·9)	68·9 (67·0-70·7)	70·2 (67·4-72·9)	71·4 (68·2-75·0)	72·6 (69·1-76·6)	73·7 (70·4-77·7)
Mexico	64·4 (63·7-65·2)	67·5 (66·8-68·1)	70·4 (69·9-70·8)	72·4 (72·0-72·8)	74·8 (74·5-75·1)	75·9 (75·6-76·2)	77·4 (77·1-77·7)	77·9 (77·5-78·2)	78·4 (78·0-78·7)	79·1 (78·7-79·6)
Nicaragua	60·8 (59·5-62·0)	67·5 (66·7-68·3)	69·9 (68·5-71·3)	69·8 (67·6-72·3)	76·4 (75·9-77·0)	76·7 (76·0-77·4)	78·4 (77·8-79·0)	79·4 (78·8-80·0)	80·0 (79·5-80·6)	81·2 (79·2-83·2)
Panama	69·4 (68·3-70·5)	73·2 (72·3-74·2)	75·9 (75·0-76·9)	76·8 (75·8-77·7)	77·5 (76·7-78·5)	78·5 (77·5-79·3)	79·6 (78·7-80·4)	80·2 (79·3-81·1)	80·9 (80·1-81·8)	82·0 (80·4-83·6)
Venezuela	68·5 (67·6-69·3)	69·9 (69·0-70·7)	72·5 (71·7-73·3)	74·2 (73·4-75·0)	75·4 (74·6-76·3)	76·2 (75·1-77·3)	77·9 (77·1-78·8)	78·9 (78·1-79·7)	79·5 (78·6-80·3)	79·8 (77·5-81·8)
Andean Latin America	53·9 (49·9-57·9)	62·4 (61·6-63·2)	64·8 (64·0-65·6)	67·0 (66·3-67·7)	70·1 (69·4-70·8)	71·9 (71·2-72·5)	75·3 (74·7-75·9)	77·3 (76·6-77·9)	78·3 (77·5-79·1)	79·8 (78·3-81·2)
Bolivia	51·7 (50·0-53·3)	54·9 (53·4-56·3)	57·7 (56·4-59·1)	59·7 (58·3-61·1)	62·2 (60·7-63·6)	65·0 (63·6-66·4)	69·1 (68·1-70·0)	71·4 (70·0-72·6)	72·8 (70·4-74·9)	74·3 (71·2-77·0)
Ecuador	64·5 (63·6-65·4)	66·8 (66·1-67·6)	69·5 (68·8-70·3)	72·0 (71·4-72·7)	73·9 (73·3-74·5)	75·3 (74·8-75·9)	76·9 (76·3-77·5)	78·4 (77·8-79·0)	79·3 (78·7-79·9)	80·4 (79·4-81·3)
Peru	50·6 (44·3-57·5)	63·5 (62·1-64·8)	65·3 (64·2-66·5)	67·5 (66·5-68·5)	71·4 (70·4-72·5)	73·0 (71·8-73·9)	76·9 (75·9-77·9)	79·1 (78·0-80·2)	80·0 (78·9-81·0)	81·6 (79·2-84·0)
Caribbean	65·0 (63·9-66·1)	66·4 (65·4-67·5)	67·5 (66·5-68·5)	68·5 (67·7-69·3)	69·5 (68·8-70·1)	70·3 (69·6-70·9)	71·6 (70·9-72·4)	72·2 (71·4-73·1)	62·2 (53·8-71·5)	75·4 (74·0-76·6)
Antigua and Barbuda	68·8 (67·5-70·1)	71·0 (69·7-72·3)	73·4 (72·0-74·9)	75·1 (73·8-76·5)	76·7 (75·5-77·8)	77·3 (76·2-78·5)	77·8 (76·6-78·9)	78·5 (77·4-79·7)	79·6 (78·3-80·9)	79·9 (78·3-81·6)
The Bahamas	67·3 (65·7-68·7)	69·4 (68·2-70·4)	70·8 (69·6-71·8)	72·7 (71·6-73·7)	74·0 (73·0-74·9)	74·1 (73·3-74·9)	74·6 (73·8-75·2)	75·8 (75·1-76·6)	76·5 (75·6-77·4)	76·3 (74·6-78·0)

Appendix Table 13C: Estimates of life expectancy at birth by location, females, 1970, 1975, 1980, 1985, 1990, 1995, 2000, 2005, 2010, 2016

Location	1970	1975	1980	1985	1990	1995	2000	2005	2010	2016
Barbados	71·3 (69·9-72·6)	73·1 (72·0-74·2)	74·9 (73·9-75·9)	75·8 (74·7-76·7)	76·2 (75·1-77·1)	76·5 (75·3-77·6)	77·5 (76·4-78·6)	78·3 (77·4-79·2)	79·2 (78·2-80·1)	78·7 (77·3-80·1)
Belize	68·2 (66·4-69·9)	70·2 (68·9-71·5)	71·1 (69·6-72·7)	73·0 (71·5-74·2)	73·9 (72·7-75·2)	73·9 (72·9-75·0)	72·1 (71·0-73·0)	73·1 (72·1-73·9)	74·2 (73·2-75·3)	74·9 (73·1-76·8)
Bermuda	69·8 (68·7-70·8)	71·6 (70·5-72·6)	72·5 (71·4-73·5)	72·9 (71·8-73·9)	72·9 (71·9-74·0)	73·7 (72·7-74·8)	75·0 (74·1-76·0)	77·5 (76·4-78·4)	79·8 (78·9-81·0)	82·4 (80·7-84·5)
Cuba	73·6 (73·1-74·1)	75·2 (74·7-75·7)	76·2 (75·6-76·7)	76·7 (76·1-77·3)	77·0 (76·4-77·6)	77·5 (76·8-78·0)	78·8 (78·2-79·3)	79·7 (79·2-80·2)	80·5 (80·0-81·0)	81·3 (80·3-82·4)
Dominica	67·8 (66·4-69·2)	71·1 (70·0-72·3)	73·9 (72·8-75·0)	74·3 (73·3-75·3)	73·7 (72·7-74·7)	73·8 (72·9-74·7)	75·3 (74·2-76·6)	76·0 (74·8-77·1)	75·8 (74·6-76·8)	75·8 (73·9-77·4)
Dominican Republic	66·4 (65·1-67·8)	68·7 (67·4-70·1)	70·8 (69·5-72·1)	71·6 (70·6-73·0)	72·7 (72·0-73·6)	74·4 (73·5-75·4)	76·0 (75·1-77·4)	76·4 (75·1-78·0)	77·6 (76·1-79·4)	78·6 (76·9-80·6)
Grenada	69·4 (66·7-71·7)	71·8 (69·5-74·3)	72·7 (70·4-75·1)	72·4 (70·3-74·5)	73·1 (71·5-74·3)	73·9 (72·5-75·0)	74·1 (72·8-75·4)	73·9 (72·5-75·2)	74·1 (72·7-75·4)	74·4 (72·3-76·7)
Guyana	63·2 (62·0-64·4)	64·5 (63·4-65·8)	66·3 (65·1-67·5)	67·4 (66·3-68·6)	67·8 (66·8-68·9)	68·3 (67·4-69·2)	69·0 (67·9-70·0)	68·1 (67·0-69·2)	68·5 (67·4-69·6)	71·2 (69·6-72·9)
Haiti	47·1 (44·2-50·4)	49·4 (46·7-52·7)	51·2 (48·7-54·0)	53·0 (51·0-55·1)	54·3 (52·6-56·1)	55·5 (53·7-57·2)	57·0 (54·9-59·0)	57·9 (55·7-60·3)	36·1 (25·0-54·7)	64·4 (61·4-67·5)
Jamaica	70·9 (69·7-72·1)	72·9 (71·4-74·3)	73·2 (71·9-74·6)	74·0 (72·9-75·2)	75·4 (74·0-76·6)	75·4 (73·6-77·2)	75·4 (74·1-76·7)	76·0 (74·8-77·3)	76·4 (74·9-77·9)	76·8 (74·5-79·1)
Puerto Rico	75·2 (74·6-75·8)	76·2 (75·6-76·8)	77·7 (77·1-78·3)	77·7 (76·9-78·4)	78·4 (77·9-79·1)	78·6 (78·1-79·3)	80·1 (79·4-80·8)	80·9 (80·3-81·5)	81·8 (81·2-82·3)	82·4 (81·3-83·5)
Saint Lucia	67·7 (64·9-69·6)	69·2 (67·8-70·4)	71·1 (70·0-72·1)	72·5 (71·4-73·5)	74·3 (73·2-75·2)	75·6 (74·8-76·5)	76·8 (75·9-77·6)	77·3 (76·3-78·1)	78·7 (77·9-79·6)	79·3 (78·1-80·4)
Saint Vincent and the Grenadines	66·9 (64·4-69·2)	68·9 (66·8-70·6)	71·0 (69·4-72·4)	72·3 (71·1-73·4)	72·7 (71·7-73·7)	72·5 (71·5-73·3)	72·8 (71·9-73·6)	73·7 (73·1-74·5)	74·4 (73·6-75·3)	74·8 (73·7-76·1)
Suriname	66·4 (65·1-67·7)	67·4 (66·4-68·4)	68·6 (67·9-69·4)	71·0 (70·4-71·7)	72·5 (71·9-73·2)	72·7 (72·0-73·4)	72·2 (71·4-72·9)	72·3 (71·5-73·1)	73·4 (72·6-74·2)	74·4 (73·1-75·4)
Trinidad and Tobago	67·5 (66·9-68·2)	68·7 (68·1-69·4)	70·6 (70·0-71·2)	71·6 (71·0-72·3)	71·9 (71·3-72·6)	72·3 (71·6-73·0)	72·6 (71·9-73·3)	74·7 (74·0-75·4)	76·3 (75·3-77·2)	77·0 (75·6-78·2)
Virgin Islands, U.S.	70·7 (69·2-71·9)	72·2 (70·8-73·2)	73·6 (72·5-74·6)	75·2 (74·2-76·3)	76·1 (75·2-77·5)	76·4 (75·3-77·5)	77·2 (76·2-78·3)	77·7 (76·8-78·5)	78·1 (77·3-79·0)	78·8 (77·5-80·1)
Tropical Latin America	64·6 (63·4-65·8)	67·1 (66·2-67·9)	69·5 (68·8-70·2)	70·6 (70·1-71·2)	72·5 (72·0-72·9)	73·7 (73·3-74·1)	75·3 (74·9-75·7)	76·7 (76·3-77·1)	77·8 (77·4-78·2)	78·9 (78·4-79·4)
Brazil	64·6 (63·4-65·9)	67·0 (66·1-67·9)	69·4 (68·7-70·1)	70·6 (70·0-71·1)	72·4 (71·9-72·8)	73·6 (73·2-74·1)	75·3 (74·8-75·7)	76·8 (76·3-77·1)	77·9 (77·5-78·3)	79·0 (78·5-79·5)
Paraguay	70·9 (70·1-71·6)	72·2 (71·6-72·9)	73·7 (72·9-74·4)	74·8 (74·1-75·4)	76·3 (75·5-77·0)	76·4 (75·4-77·1)	76·6 (75·8-77·2)	76·2 (75·3-77·1)	76·3 (75·5-77·3)	77·1 (75·7-78·5)
Southeast Asia, East Asia, and Oceania	61·0 (60·3-61·8)	63·4 (62·8-64·0)	65·3 (64·8-65·8)	67·4 (66·9-67·8)	68·8 (68·4-69·2)	70·2 (69·8-70·5)	71·8 (71·5-72·1)	74·0 (73·8-74·2)	76·2 (76·0-76·4)	78·4 (78·1-78·6)
East Asia	62·3 (61·4-63·2)	64·8 (64·0-65·5)	66·1 (65·3-66·8)	68·1 (67·4-68·7)	69·4 (68·8-70·0)	70·7 (70·2-71·2)	72·5 (72·1-72·9)	75·2 (74·9-75·5)	77·6 (77·3-77·8)	79·8 (79·5-80·1)
China	62·0 (61·1-62·9)	64·5 (63·7-65·3)	65·8 (65·0-66·5)	67·9 (67·2-68·5)	69·2 (68·6-69·8)	70·7 (70·3-71·2)	72·7 (72·3-73·0)	75·2 (74·9-75·5)	77·6 (77·3-77·9)	79·9 (79·6-80·2)
North Korea	69·2 (67·3-71·0)	71·9 (70·1-73·5)	73·1 (71·6-74·7)	73·6 (72·2-75·2)	74·1 (72·8-75·7)	66·7 (60·2-72·5)	66·3 (60·7-71·7)	72·4 (71·2-73·8)	72·8 (71·8-74·1)	73·6 (72·5-74·7)
Taiwan (Province of China)	71·3 (70·7-71·9)	73·4 (72·8-73·9)	74·3 (73·7-74·9)	75·7 (75·2-76·2)	76·9 (76·4-77·4)	77·8 (77·3-78·3)	79·2 (78·7-79·8)	80·8 (80·3-81·2)	82·3 (81·8-82·8)	82·8 (81·4-84·4)
Southeast Asia	57·7 (56·9-58·5)	59·9 (58·8-61·0)	63·4 (62·9-64·0)	65·7 (65·2-66·2)	67·5 (67·1-68·0)	69·1 (68·7-69·5)	70·3 (70·0-70·7)	71·8 (71·5-72·2)	73·5 (73·2-73·9)	75·5 (75·0-76·0)
Cambodia	49·4 (46·9-51·8)	30·5 (19·3-49·6)	55·2 (53·5-57·0)	56·7 (55·2-58·3)	59·2 (58·3-60·4)	60·1 (59·3-61·0)	61·9 (61·0-62·8)	65·5 (64·6-66·4)	69·0 (68·1-70·0)	71·6 (70·5-72·7)
Indonesia	54·7 (53·9-55·4)	57·5 (56·6-58·3)	60·2 (59·6-60·9)	62·8 (62·2-63·4)	64·9 (64·3-65·4)	67·0 (66·6-67·5)	68·4 (68·1-68·8)	70·0 (69·5-70·4)	71·6 (71·0-72·1)	73·6 (73·0-74·1)
Laos	44·2 (38·5-49·1)	47·2 (43·0-51·0)	49·4 (46·5-52·1)	52·3 (50·5-53·9)	54·2 (52·7-55·5)	56·3 (54·9-57·5)	59·0 (57·7-60·2)	62·4 (61·2-63·5)	66·1 (64·9-67·2)	69·7 (68·0-71·1)
Malaysia	66·5 (66·0-66·9)	69·0 (68·6-69·5)	71·1 (70·6-71·6)	72·7 (72·3-73·1)	73·7 (73·4-74·0)	74·5 (74·2-74·8)	75·3 (75·0-75·5)	75·9 (75·6-76·1)	76·5 (76·1-76·8)	78·0 (77·5-78·6)
Maldives	39·4 (35·8-43·2)	44·2 (40·8-47·8)	47·7 (45·0-50·9)	54·6 (53·2-55·8)	62·0 (60·9-63·2)	68·1 (66·6-69·6)	72·7 (71·7-73·8)	76·7 (75·7-77·7)	79·5 (78·5-80·5)	81·3 (78·6-83·9)
Mauritius	63·5 (63·0-64·1)	66·9 (66·3-67·4)	70·1 (69·5-70·7)	71·5 (70·9-72·0)	73·4 (72·9-73·9)	74·4 (73·8-74·9)	74·8 (74·3-75·3)	75·7 (75·1-76·2)	76·9 (76·4-77·4)	77·8 (76·0-79·6)
Myanmar	51·3 (48·8-53·5)	55·2 (53·2-57·3)	57·9 (56·1-59·7)	59·7 (58·1-61·3)	60·9 (59·5-62·3)	62·1 (60·6-63·4)	63·8 (62·3-65·2)	66·6 (65·2-67·8)	70·0 (68·7-71·1)	73·4 (72·4-74·3)
Philippines	66·3 (65·4-67·2)	67·1 (66·3-68·0)	68·5 (67·7-69·3)	69·4 (68·6-70·1)	70·9 (70·2-71·6)	72·0 (71·3-72·7)	72·2 (71·4-72·9)	72·1 (71·3-72·9)	72·6 (71·8-73·4)	73·9 (72·1-75·8)
Sri Lanka	66·5 (65·7-67·4)	67·0 (66·2-67·9)	71·2 (70·3-72·0)	73·5 (72·7-74·3)	75·4 (74·6-76·3)	76·1 (75·1-77·1)	76·9 (76·1-77·9)	78·0 (76·9-78·9)	78·3 (77·0-79·5)	81·1 (78·7-83·8)
Seychelles	68·0 (66·2-69·6)	71·3 (69·8-72·5)	74·0 (72·7-75·4)	73·2 (72·5-74·1)	74·1 (73·5-74·8)	75·9 (75·1-77·1)	75·6 (75·0-76·4)	75·9 (75·3-76·9)	77·1 (76·2-77·9)	77·4 (76·0-78·9)
Thailand	64·8 (64·0-65·6)	67·9 (67·2-68·7)	69·7 (69·0-70·5)	72·0 (71·3-72·7)	74·1 (73·4-74·7)	74·9 (74·3-75·6)	75·6 (74·9-76·4)	77·4 (76·8-77·9)	79·6 (79·0-80·2)	80·9 (79·6-82·0)
Timor-Leste	43·1 (38·6-47·5)	44·0 (40·4-47·9)	47·4 (44·2-51·0)	53·9 (51·2-57·1)	57·6 (55·0-60·6)	61·0 (58·7-63·4)	63·8 (61·7-66·1)	67·7 (65·6-70·0)	72·0 (69·5-74·2)	73·7 (70·6-76·6)
Vietnam	58·6 (54·0-63·4)	64·2 (62·2-66·7)	67·0 (65·3-69·3)	69·3 (67·5-71·5)	71·1 (69·5-73·0)	72·7 (70·9-74·9)	74·1 (72·6-76·4)	75·2 (73·7-77·5)	76·6 (75·0-78·3)	78·1 (76·6-79·1)
Oceania	58·2 (56·4-60·1)	59·3 (57·6-61·1)	59·9 (58·1-61·7)	60·1 (58·4-61·9)	60·3 (58·4-62·0)	60·8 (58·9-62·7)	61·1 (59·0-63·1)	61·1 (58·8-63·1)	61·9 (59·5-64·1)	63·8 (61·4-66·0)
American Samoa	70·0 (68·7-71·5)	72·6 (70·9-74·2)	73·2 (71·1-75·0)	73·4 (71·7-74·9)	75·0 (73·8-76·4)	74·3 (72·5-75·7)	74·4 (72·8-75·7)	74·5 (72·6-75·9)	74·7 (73·1-76·4)	74·4 (72·0-77·0)
Federated States of Micronesia	61·7 (59·5-64·4)	62·9 (60·6-65·3)	64·1 (61·9-66·3)	64·9 (62·7-67·0)	65·6 (63·2-67·7)	66·4 (64·2-68·6)	66·8 (64·5-69·3)	67·2 (64·4-69·8)	67·3 (64·2-70·0)	67·6 (64·5-70·5)
Fiji	67·0 (65·3-68·9)	67·2 (65·4-69·0)	67·6 (65·8-69·6)	68·7 (66·8-70·5)	68·0 (63·9-72·2)	67·2 (64·0-70·6)	66·8 (64·6-69·1)	67·2 (65·2-69·2)	67·4 (64·9-69·6)	67·7 (63·5-71·9)
Guam	71·0 (69·7-72·3)	72·4 (71·1-73·8)	75·2 (74·1-76·4)	76·6 (75·2-77·9)	76·4 (75·2-77·7)	76·4 (75·4-77·8)	77·1 (76·1-78·3)	76·9 (75·7-77·9)	75·7 (74·4-77·0)	76·1 (74·3-78·1)
Kiribati	57·4 (54·1-60·7)	60·4 (57·0-63·7)	61·4 (57·3-64·5)	60·5 (57·3-63·1)	60·9 (59·0-62·7)	61·8 (60·5-63·1)	63·0 (61·5-64·4)	63·8 (61·9-65·7)	64·4 (62·1-66·8)	65·6 (62·4-68·3)
Marshall Islands	64·4 (60·2-68·0)	66·5 (63·0-69·6)	68·1 (65·4-70·8)	68·6 (66·5-70·7)	68·0 (66·5-69·4)	67·1 (65·6-68·5)	65·6 (63·8-67·2)	65·2 (63·4-67·0)	65·8 (63·5-68·3)	67·3 (64·4-70·1)
Northern Mariana Islands	70·3 (67·1-73·5)	71·9 (68·4-75·2)	73·1 (69·8-76·2)	73·7 (70·6-76·5)	74·7 (72·0-77·5)	76·4 (73·8-78·8)	77·3 (75·0-79·7)	77·6 (75·4-79·8)	77·6 (75·3-79·7)	77·4 (74·9-79·9)
Papua New Guinea	55·6 (53·2-58·2)	56·8 (54·5-59·3)	57·1 (54·7-59·5)	57·2 (54·9-59·4)	57·5 (55·2-59·8)	58·4 (56·0-60·8)	58·9 (56·1-61·4)	58·8 (56·0-61·4)	59·9 (56·9-62·6)	62·2 (59·4-64·9)
Samoa	67·5 (66·2-69·1)	68·6 (67·2-70·1)	70·8 (69·3-72·3)	71·6 (69·8-73·3)	72·3 (70·3-74·3)	72·7 (70·5-74·7)	73·1 (71·1-75·2)	73·5 (71·7-75·5)	73·5 (71·8-75·4)	74·0 (72·0-76·1)
Solomon Islands	56·3 (53·8-58·9)	56·8 (54·4-59·6)	60·9 (58·3-63·4)	61·6 (59·1-64·1)	61·8 (59·5-64·3)	62·4 (60·1-64·7)	62·7 (60·5-65·0)	62·1 (59·7-64·6)	62·8 (60·2-65·4)	64·2 (61·5-66·9)
Tonga	67·5 (65·1-70·0)	68·1 (65·6-70·6)	68·0 (65·4-70·6)	68·9 (66·2-71·6)	70·1 (67·6-72·6)	70·7 (68·2-73·0)	71·0 (69·0-73·2)	72·2 (70·2-74·2)	72·7 (70·5-74·9)	73·3 (70·9-75·6)
Vanuatu	58·9 (56·0-61·5)	60·9 (58·1-63·5)	62·7 (60·4-65·0)	63·5 (61·3-65·6)	63·9 (61·8-66·0)	64·1 (61·9-66·2)	64·1 (62·0-66·1)	63·9 (61·3-66·1)	64·7 (61·8-67·0)	65·7 (62·8-68·2)
North Africa and Middle East	57·0 (56·1-57·9)	60·7 (60·0-61·3)	63·5 (62·8-64·1)	65·9 (65·2-66·6)	68·9 (68·4-69·4)	70·1 (69·7-70·5)	71·8 (71·4-72·2)	72·9 (72·5-73·4)	74·3 (73·8-74·9)	75·6 (74·9-76·3)
North Africa and Middle East	57·0 (56·1-57·9)	60·7 (60·0-61·3)	63·5 (62·8-64·1)	65·9 (65·2-66·6)	68·9 (68·4-69·4)	70·1 (69·7-70·5)	71·8 (71·4-72·2)	72·9 (72·5-73·4)	74·3 (73·8-74·9)	75·6 (74·9-76·3)
Afghanistan	44·4 (41·2-47·7)	44·9 (42·2-47·7)	43·6 (40·6-46·5)	43·7 (39·5-48·3)	51·4 (49·8-52·9)	51·2 (49·7-52·7)	51·8 (50·5-53·2)	54·1 (52·6-55·6)	56·8 (55·2-58·3)	59·2 (57·5-60·8)
Algeria	59·1 (57·2-60·9)	60·9 (59·2-62·6)	63·4 (62·1-64·6)	69·7 (68·7-70·8)	72·6 (71·3-73·8)	74·0 (72·6-75·1)	75·4 (74·1-76·5)	76·6 (75·2-77·5)	77·6 (76·9-78·2)	78·4 (77·5-79·3)
Bahrain	66·5 (63·1-69·5)	69·7 (66·8-72·7)	72·9 (70·7-75·0)	73·1 (71·4-74·8)	71·0 (69·3-72·6)	71·3 (69·6-73·0)	73·1 (71·5-74·6)	74·6 (73·1-76·2)	76·7 (75·3-78·2)	77·8 (75·7-80·1)

Appendix Table 13C: Estimates of life expectancy at birth by location, females, 1970, 1975, 1980, 1985, 1990, 1995, 2000, 2005, 2010, 2016

Location	1970	1975	1980	1985	1990	1995	2000	2005	2010	2016
Egypt	53·4 (51·8-55·0)	57·1 (55·7-58·4)	61·0 (59·6-62·4)	63·2 (62·0-64·2)	67·3 (66·3-68·2)	70·0 (69·1-70·8)	72·5 (71·5-73·3)	72·8 (72·0-73·6)	73·0 (72·2-73·8)	75·0 (73·2-77·0)
Iran	58·0 (54·5-61·7)	66·9 (64·5-69·6)	69·6 (67·5-71·9)	71·5 (69·4-73·8)	71·1 (68·9-73·4)	73·7 (71·9-75·5)	74·2 (72·5-75·8)	74·8 (72·5-76·9)	76·5 (74·3-79·0)	78·4 (76·6-80·6)
Iraq	63·2 (61·5-64·6)	63·5 (61·8-65·2)	64·2 (61·1-67·7)	63·5 (60·0-67·4)	67·6 (65·6-69·7)	66·9 (65·4-68·3)	67·8 (66·3-69·1)	67·4 (65·2-69·5)	69·4 (66·8-71·9)	70·5 (67·4-73·5)
Jordan	64·2 (61·8-66·4)	67·1 (64·8-69·0)	68·8 (66·5-71·1)	70·3 (68·7-72·3)	72·6 (70·9-74·8)	73·5 (71·5-75·7)	73·6 (71·3-76·0)	73·5 (71·1-76·0)	76·9 (74·3-79·6)	78·0 (74·9-81·0)
Kuwait	72·0 (70·4-73·7)	72·4 (70·8-74·1)	73·5 (72·1-75·2)	74·9 (73·3-76·4)	76·7 (74·8-78·6)	76·7 (75·3-78·1)	76·6 (75·2-78·2)	77·2 (75·7-78·8)	77·8 (76·4-79·1)	79·5 (76·6-82·7)
Lebanon	68·0 (64·4-71·5)	64·3 (59·2-69·8)	59·9 (52·5-68·4)	68·7 (64·9-72·5)	71·9 (70·1-74·0)	74·2 (72·7-76·3)	77·0 (75·3-78·3)	78·8 (78·0-80·0)	80·7 (79·5-81·9)	81·4 (79·9-83·0)
Libya	67·4 (65·9-69·1)	70·6 (69·2-72·0)	73·2 (71·9-74·6)	74·3 (73·3-75·7)	74·7 (73·4-75·8)	75·5 (74·0-76·6)	75·7 (74·1-77·0)	76·7 (75·0-77·9)	77·9 (76·6-78·8)	77·6 (76·1-78·7)
Morocco	57·6 (56·4-58·8)	60·1 (59·0-61·1)	63·0 (61·9-64·1)	66·5 (65·5-67·4)	68·7 (67·6-69·7)	70·3 (69·1-71·4)	72·3 (71·2-73·3)	73·5 (72·2-74·7)	74·9 (73·6-76·3)	76·4 (75·1-77·7)
Palestine	64·5 (60·6-68·0)	67·9 (64·6-70·9)	69·9 (67·1-72·5)	71·5 (68·9-73·8)	71·8 (69·7-73·7)	72·9 (71·7-74·2)	73·2 (72·5-73·7)	74·2 (73·8-74·6)	74·0 (73·7-74·4)	73·5 (72·8-74·2)
Oman	50·7 (48·0-53·1)	56·9 (54·6-59·0)	63·2 (61·4-64·9)	68·6 (67·2-70·0)	71·9 (70·5-73·3)	74·0 (72·7-75·6)	76·2 (75·2-77·5)	78·1 (77·3-79·2)	78·1 (77·0-79·2)	79·9 (79·2-80·8)
Qatar	69·9 (65·1-74·5)	71·6 (67·6-75·7)	74·4 (70·8-77·5)	74·2 (71·8-76·8)	75·3 (73·1-77·8)	75·3 (73·4-77·6)	76·3 (74·4-78·5)	78·4 (76·8-80·1)	80·8 (79·0-83·0)	81·8 (78·9-85·2)
Saudi Arabia	67·0 (64·2-69·8)	70·8 (68·9-72·7)	72·9 (71·2-74·5)	73·5 (72·0-75·0)	73·5 (72·1-74·9)	73·7 (72·6-75·0)	73·4 (72·6-74·2)	74·6 (74·0-75·2)	76·0 (75·4-76·6)	78·7 (77·8-79·6)
Sudan	53·8 (52·4-55·2)	54·5 (53·1-55·9)	56·1 (54·6-57·4)	51·9 (48·3-55·9)	59·5 (58·3-60·6)	61·4 (60·3-62·6)	63·9 (62·8-64·9)	66·3 (65·4-67·3)	68·0 (67·0-69·0)	70·3 (69·0-71·5)
Syria	67·2 (66·1-68·3)	69·8 (69·0-70·6)	71·4 (70·6-72·1)	72·0 (71·1-73·0)	72·8 (71·7-73·9)	73·7 (72·9-74·8)	75·3 (74·8-75·8)	76·8 (76·1-77·6)	78·3 (77·6-79·1)	73·6 (69·1-77·7)
Tunisia	59·0 (57·7-60·2)	63·8 (62·7-64·8)	67·7 (66·6-68·8)	72·0 (70·8-73·1)	74·1 (73·1-75·2)	75·6 (74·4-76·6)	77·6 (76·5-78·8)	78·6 (77·4-80·3)	79·5 (78·2-81·6)	80·5 (78·9-82·7)
Turkey	60·9 (58·1-63·8)	64·0 (62·0-66·2)	68·0 (66·3-69·6)	71·4 (69·9-72·9)	73·5 (72·4-74·5)	74·4 (73·5-75·3)	77·6 (76·7-78·5)	80·0 (79·0-80·9)	81·4 (80·2-82·5)	82·3 (80·4-84·3)
United Arab Emirates	66·0 (61·7-70·2)	69·0 (64·4-72·8)	71·7 (67·8-75·1)	73·3 (70·0-76·5)	73·8 (71·1-76·7)	75·0 (72·8-77·2)	76·5 (74·8-78·0)	78·3 (77·1-79·2)	78·4 (76·9-79·9)	78·6 (76·2-80·6)
Yemen	44·0 (42·4-45·7)	49·9 (48·4-51·4)	54·2 (52·9-55·7)	57·1 (55·9-58·4)	58·8 (57·6-60·1)	60·4 (59·2-61·7)	62·6 (61·5-63·9)	65·1 (64·0-66·3)	67·7 (66·6-68·9)	67·9 (66·6-69·2)
South Asia	49·5 (48·7-50·3)	52·4 (51·6-53·1)	55·1 (54·4-55·7)	57·4 (56·9-58·0)	59·8 (59·2-60·3)	61·7 (61·2-62·2)	63·7 (63·3-64·1)	65·7 (65·3-66·2)	68·0 (67·6-68·5)	70·6 (70·1-71·1)
South Asia	49·5 (48·7-50·3)	52·4 (51·6-53·1)	55·1 (54·4-55·7)	57·4 (56·9-58·0)	59·8 (59·2-60·3)	61·7 (61·2-62·2)	63·7 (63·3-64·1)	65·7 (65·3-66·2)	68·0 (67·6-68·5)	70·6 (70·1-71·1)
Bangladesh	46·5 (43·1-50·2)	52·7 (50·6-54·7)	53·7 (52·2-55·3)	55·5 (54·3-56·7)	58·6 (57·3-59·9)	62·5 (61·2-63·6)	66·7 (65·7-67·9)	69·7 (68·7-70·6)	72·3 (71·3-73·2)	75·1 (73·5-76·4)
Bhutan	48·3 (45·2-51·4)	50·6 (47·8-53·4)	52·6 (49·9-55·1)	55·6 (53·2-57·9)	59·1 (57·1-61·4)	62·3 (60·5-64·3)	65·9 (64·2-67·7)	70·4 (68·8-72·0)	73·7 (71·8-75·4)	75·9 (73·8-77·4)
India	49·5 (48·5-50·5)	52·2 (51·3-53·0)	54·9 (54·3-55·5)	57·3 (56·7-57·9)	59·7 (59·0-60·3)	61·6 (61·1-62·2)	63·4 (62·9-64·0)	65·6 (65·0-66·2)	67·8 (67·2-68·4)	70·3 (69·6-71·0)
Nepal	49·4 (48·1-50·7)	50·8 (49·8-52·1)	52·5 (51·5-53·7)	54·9 (53·9-55·9)	57·7 (56·7-58·6)	60·8 (59·8-61·7)	64·6 (63·6-65·6)	66·7 (65·8-67·7)	69·1 (68·2-70·1)	71·9 (71·1-72·8)
Pakistan	57·3 (55·0-59·3)	59·0 (56·8-60·8)	60·6 (58·8-62·1)	61·9 (60·7-62·9)	62·5 (61·5-63·5)	61·8 (60·8-62·9)	62·9 (61·7-64·1)	63·4 (62·1-64·8)	66·3 (64·6-68·2)	68·9 (66·9-71·2)
Sub-Saharan Africa	50·7 (49·8-51·6)	52·3 (51·6-53·1)	53·9 (53·3-54·4)	54·9 (54·4-55·4)	55·4 (54·9-55·8)	55·0 (54·5-55·4)	54·2 (53·7-54·8)	55·8 (55·3-56·3)	59·5 (59·0-60·1)	64·6 (63·9-65·3)
Southern Sub-Saharan Africa	59·2 (57·4-60·9)	61·0 (59·7-62·4)	62·4 (61·4-63·6)	65·1 (64·3-65·8)	67·4 (66·6-68·1)	65·9 (64·5-67·2)	56·1 (54·6-57·5)	51·3 (50·0-52·6)	55·1 (54·1-56·2)	64·9 (63·8-66·0)
Botswana	58·3 (57·0-59·6)	61·5 (60·4-62·8)	64·0 (62·8-65·2)	66·4 (65·3-67·4)	67·6 (65·6-69·8)	60·1 (53·4-68·7)	48·7 (42·4-56·9)	49·2 (44·0-55·2)	60·2 (54·4-66·4)	69·2 (64·5-78·5)
Lesotho	58·4 (55·5-61·9)	59·8 (56·5-63·0)	61·6 (58·6-64·7)	63·0 (60·7-65·7)	64·2 (62·3-66·3)	61·9 (59·4-64·9)	51·8 (48·0-55·7)	45·4 (41·7-49·4)	49·1 (45·7-52·0)	53·7 (49·9-58·2)
Namibia	59·5 (57·2-61·7)	60·6 (58·9-62·3)	61·4 (60·0-62·8)	63·6 (62·3-64·9)	64·7 (63·6-65·9)	63·1 (60·8-65·7)	56·2 (52·3-61·3)	54·0 (50·1-58·1)	61·5 (57·7-65·5)	69·3 (65·5-75·2)
South Africa	59·2 (56·9-61·4)	61·0 (59·2-62·8)	62·3 (60·8-63·8)	64·9 (64·0-65·9)	68·1 (67·2-68·9)	68·7 (67·8-69·6)	58·4 (56·9-59·6)	52·5 (51·0-53·9)	55·8 (54·6-56·9)	65·5 (64·2-66·7)
Swaziland	53·5 (49·5-57·3)	57·2 (54·3-60·4)	60·3 (57·9-63·1)	63·0 (60·6-65·5)	65·4 (63·4-67·5)	63·1 (59·4-67·2)	50·1 (44·9-55·6)	43·7 (39·9-48·1)	49·2 (45·6-52·9)	62·2 (57·4-68·3)
Zimbabwe	59·4 (57·3-61·4)	61·2 (59·2-62·9)	62·5 (60·9-64·0)	65·0 (63·8-66·3)	64·4 (62·1-67·2)	56·8 (52·1-62·1)	48·6 (44·6-53·0)	46·6 (43·4-50·2)	51·3 (48·6-54·8)	61·9 (59·2-65·2)
Western Sub-Saharan Africa	50·0 (48·7-51·1)	52·0 (50·9-53·1)	54·0 (53·1-54·9)	55·2 (54·3-56·1)	55·6 (54·7-56·3)	55·5 (54·6-56·4)	55·9 (54·8-56·9)	57·7 (56·7-58·7)	60·8 (59·8-61·8)	64·8 (63·7-65·9)
Benin	48·8 (47·3-50·4)	51·4 (49·9-52·8)	53·4 (52·2-54·7)	55·7 (54·5-56·8)	57·6 (56·5-58·7)	58·6 (57·4-59·8)	59·7 (58·3-61·1)	61·6 (60·1-63·0)	63·8 (62·3-65·4)	66·3 (64·8-68·1)
Burkina Faso	45·8 (44·6-46·9)	47·4 (46·6-48·3)	49·6 (48·9-50·3)	51·3 (50·6-52·0)	52·1 (51·1-53·2)	52·3 (51·0-53·8)	53·7 (52·5-54·9)	56·3 (55·3-57·3)	59·1 (58·1-60·0)	62·1 (61·0-63·0)
Cameroon	52·1 (50·6-53·8)	53·9 (52·4-55·5)	55·8 (54·5-57·3)	58·4 (57·1-59·8)	59·6 (58·3-61·1)	58·0 (56·4-59·9)	55·8 (54·1-58·1)	56·1 (54·2-58·4)	58·6 (56·3-61·1)	62·0 (59·3-65·2)
Cape Verde	59·8 (57·2-62·5)	63·8 (61·6-66·0)	67·5 (66·1-69·0)	68·6 (67·7-69·6)	71·6 (70·5-72·8)	71·3 (69·4-73·6)	74·3 (72·0-76·7)	76·1 (74·3-78·2)	77·7 (76·5-79·2)	78·5 (77·2-80·2)
Chad	51·4 (49·8-53·1)	53·0 (51·6-54·4)	53·0 (51·5-54·3)	53·6 (52·3-54·8)	54·4 (53·3-55·7)	54·8 (53·5-56·0)	53·8 (52·2-55·4)	55·2 (53·6-57·0)	57·8 (56·2-59·6)	61·4 (59·6-63·2)
Cote d'Ivoire	53·1 (51·8-54·4)	55·6 (54·4-56·8)	57·7 (56·5-58·9)	58·4 (57·3-59·4)	57·7 (56·6-58·8)	55·9 (54·5-57·5)	55·1 (53·4-57·0)	55·7 (54·0-57·5)	58·3 (56·8-60·1)	62·3 (60·5-64·0)
The Gambia	56·5 (54·3-58·8)	58·2 (56·2-60·4)	59·9 (58·0-62·1)	61·3 (59·4-63·1)	62·3 (60·7-64·0)	63·3 (61·8-64·9)	64·4 (63·0-66·0)	66·0 (64·4-67·8)	67·3 (65·7-69·1)	69·2 (67·6-71·3)
Ghana	56·2 (54·5-58·1)	57·6 (56·0-59·5)	58·4 (56·7-60·2)	58·8 (57·2-60·4)	59·8 (58·4-61·4)	60·6 (59·1-62·3)	60·6 (58·8-62·7)	61·6 (59·8-63·6)	63·5 (61·9-65·4)	67·5 (66·0-69·7)
Guinea	44·9 (42·3-47·4)	45·9 (43·8-48·0)	47·8 (46·0-49·6)	49·5 (48·0-51·1)	51·9 (50·5-53·3)	53·8 (52·6-55·0)	55·5 (54·2-56·9)	57·0 (55·6-58·7)	58·9 (57·1-61·0)	61·6 (59·5-64·0)
Guinea-Bissau	44·5 (41·6-47·8)	48·2 (46·0-50·8)	49·7 (47·8-52·0)	50·4 (48·7-52·5)	51·9 (50·3-53·8)	53·5 (52·0-55·1)	54·7 (53·0-56·3)	55·6 (53·6-57·5)	57·8 (55·7-59·7)	61·4 (59·4-63·3)
Liberia	49·0 (47·5-50·6)	51·0 (49·4-52·7)	52·4 (50·9-53·9)	53·1 (51·7-54·4)	51·8 (50·5-53·1)	51·6 (50·3-52·9)	54·9 (53·5-56·5)	58·3 (57·0-59·8)	61·0 (59·8-62·3)	64·8 (63·6-66·1)
Mali	40·2 (38·3-42·7)	42·0 (40·4-44·0)	45·2 (43·6-46·9)	47·6 (46·3-49·0)	49·4 (48·2-50·7)	51·2 (50·0-52·6)	53·4 (52·1-54·8)	57·1 (55·7-58·8)	60·4 (58·5-62·4)	62·7 (60·0-65·5)
Mauritania	54·9 (51·8-58·4)	55·8 (52·7-58·9)	57·9 (55·3-60·5)	59·3 (57·6-61·2)	60·4 (59·1-61·7)	61·7 (60·6-63·1)	63·3 (61·9-64·9)	65·3 (63·7-67·3)	68·0 (65·9-70·3)	70·2 (67·6-73·3)
Niger	45·3 (43·1-47·5)	44·8 (43·0-46·6)	45·0 (43·5-46·6)	45·6 (44·1-47·1)	48·1 (46·6-49·5)	49·4 (47·9-50·8)	52·6 (51·2-54·0)	56·4 (54·7-57·9)	59·8 (57·6-61·9)	62·8 (60·2-65·5)
Nigeria	51·0 (48·3-53·4)	53·6 (51·4-55·7)	55·7 (53·8-57·6)	56·6 (54·7-58·4)	55·8 (53·9-57·4)	55·4 (53·6-57·4)	55·7 (53·7-57·9)	57·6 (55·6-59·7)	61·5 (59·6-63·7)	66·4 (64·3-69·1)
Sao Tome and Principe	60·7 (59·5-61·9)	63·7 (62·4-64·9)	65·0 (64·0-66·1)	64·3 (63·1-65·4)	64·6 (63·0-66·1)	64·3 (62·8-66·1)	65·1 (63·9-66·3)	67·3 (66·2-68·4)	70·0 (68·7-71·2)	72·1 (70·5-73·9)
Senegal	49·0 (47·6-50·4)	50·8 (49·4-52·3)	53·8 (52·6-55·2)	57·2 (56·0-58·4)	59·1 (57·9-60·3)	59·9 (58·8-61·0)	61·2 (60·1-62·4)	63·3 (62·1-64·5)	65·6 (64·6-66·6)	67·8 (66·6-68·8)
Sierra Leone	47·5 (44·5-50·2)	49·2 (46·7-51·6)	50·2 (48·1-52·2)	51·3 (49·6-52·9)	52·8 (51·4-54·1)	52·5 (51·1-53·9)	52·8 (51·5-54·2)	53·8 (52·4-55·2)	56·0 (54·4-57·6)	60·2 (58·4-62·1)
Togo	53·7 (52·1-55·4)	55·5 (54·0-57·1)	57·2 (55·7-58·8)	57·9 (56·5-59·3)	58·6 (57·5-59·9)	59·0 (57·7-60·4)	58·3 (56·5-60·7)	58·5 (56·4-61·0)	61·0 (59·3-63·1)	65·1 (63·6-66·8)
Eastern Sub-Saharan Africa	49·6 (48·6-50·6)	50·9 (49·8-51·8)	52·1 (51·2-52·9)	52·5 (51·8-53·2)	52·9 (52·4-53·5)	52·4 (51·8-53·0)	52·7 (52·0-53·4)	55·8 (55·1-56·5)	60·2 (59·4-61·0)	65·1 (64·1-66·1)
Burundi	45·7 (43·3-48·2)	46·3 (44·1-48·4)	47·4 (45·3-49·7)	48·7 (46·7-51·0)	49·3 (47·2-51·6)	48·5 (46·6-50·8)	48·9 (47·0-51·0)	54·0 (52·1-55·7)	58·7 (56·6-60·7)	61·5 (58·7-64·1)
Comoros	52·8 (50·4-55·5)	54·4 (52·2-56·9)	55·8 (53·7-58·2)	57·7 (55·6-60·0)	59·5 (57·8-61·5)	60·7 (59·2-62·5)	62·0 (60·5-63·5)	64·9 (63·3-66·6)	66·7 (65·1-68·7)	68·5 (66·8-70·9)

Appendix Table 13C: Estimates of life expectancy at birth by location, females, 1970, 1975, 1980, 1985, 1990, 1995, 2000, 2005, 2010, 2016

Location	1970	1975	1980	1985	1990	1995	2000	2005	2010	2016
Djibouti	62·4 (60·4-64·2)	63·4 (61·8-65·0)	63·7 (62·0-65·2)	63·7 (62·2-65·0)	64·1 (62·8-65·2)	64·0 (62·6-65·4)	63·8 (61·7-66·2)	64·3 (61·7-67·1)	65·8 (63·1-68·7)	68·8 (66·1-71·8)
Eritrea	47·4 (45·4-49·3)	49·2 (47·5-50·8)	50·6 (49·0-52·1)	51·8 (50·4-53·2)	53·7 (52·3-55·2)	57·5 (56·0-59·0)	59·4 (57·8-61·0)	61·2 (59·4-63·1)	62·4 (60·6-64·4)	64·5 (62·8-66·6)
Ethiopia	46·0 (43·7-48·3)	46·1 (43·8-48·2)	47·1 (45·0-49·2)	48·1 (46·5-49·8)	48·9 (47·6-50·2)	50·8 (49·6-52·1)	51·9 (50·5-53·3)	56·3 (54·7-58·0)	61·9 (60·0-64·0)	66·5 (64·2-68·9)
Kenya	58·2 (57·5-59·0)	60·1 (59·4-60·9)	62·0 (61·3-62·7)	63·1 (62·4-63·8)	62·6 (62·0-63·3)	58·9 (58·1-59·7)	56·7 (55·9-57·5)	58·8 (58·1-59·5)	64·3 (63·5-65·0)	69·0 (68·2-69·9)
Madagascar	51·1 (49·5-52·9)	53·8 (52·0-55·6)	55·9 (54·2-57·8)	55·1 (53·6-56·7)	56·8 (55·4-58·2)	57·8 (56·5-59·2)	59·3 (57·8-60·8)	61·0 (59·3-62·9)	61·9 (59·7-64·8)	63·9 (60·8-67·8)
Malawi	44·3 (41·9-46·6)	47·4 (45·5-49·4)	50·4 (48·8-52·1)	51·1 (49·4-52·6)	49·9 (48·1-51·9)	47·3 (44·5-50·2)	45·9 (42·8-49·6)	48·8 (45·7-52·4)	54·9 (52·0-58·1)	62·6 (59·8-65·9)
Mozambique	48·9 (46·8-51·0)	50·7 (49·0-52·3)	51·4 (50·1-52·8)	47·4 (45·4-49·6)	52·8 (51·7-53·9)	53·4 (51·7-55·0)	53·6 (51·1-56·3)	54·6 (51·5-57·8)	56·8 (53·9-59·8)	62·9 (60·4-65·4)
Rwanda	47·5 (45·3-49·8)	48·2 (46·2-50·4)	50·6 (48·6-52·8)	53·1 (51·4-54·9)	50·8 (49·2-52·3)	47·4 (45·7-49·3)	51·2 (49·5-53·1)	59·2 (57·3-61·1)	66·2 (64·3-68·3)	69·3 (67·2-71·5)
Somalia	51·4 (49·2-53·4)	49·6 (47·4-51·7)	52·2 (50·3-53·7)	51·4 (49·6-52·9)	51·7 (50·1-53·4)	52·1 (50·6-53·8)	52·6 (51·0-54·2)	54·3 (52·6-56·1)	53·8 (51·8-55·8)	57·7 (55·8-59·4)
South Sudan	50·6 (46·0-54·7)	51·8 (47·8-55·4)	52·7 (49·7-55·8)	52·9 (50·4-55·4)	53·3 (51·2-55·6)	54·7 (52·7-57·1)	55·8 (53·5-58·5)	57·6 (54·9-60·8)	59·1 (56·2-62·3)	60·7 (57·9-63·9)
Tanzania	53·0 (51·3-54·6)	55·0 (53·4-56·6)	56·8 (55·2-58·3)	57·2 (55·7-58·6)	56·0 (54·8-57·3)	54·0 (52·2-56·0)	53·7 (51·7-56·2)	56·0 (54·0-58·7)	60·3 (58·4-63·0)	66·0 (64·3-68·3)
Uganda	51·8 (50·6-53·2)	52·8 (51·6-54·2)	51·7 (50·2-53·3)	52·5 (50·9-53·8)	52·1 (50·4-53·4)	49·9 (47·6-52·3)	51·1 (49·2-53·0)	54·7 (53·2-56·3)	58·6 (56·8-60·6)	64·7 (62·9-67·0)
Zambia	55·5 (51·4-59·6)	57·5 (54·0-61·0)	57·6 (54·8-60·3)	55·9 (53·8-58·0)	52·3 (50·4-54·6)	47·1 (44·4-49·8)	45·0 (42·3-47·9)	47·4 (44·7-50·6)	54·0 (51·3-56·9)	61·9 (58·2-66·8)
Central Sub-Saharan Africa	50·9 (48·2-53·3)	52·1 (49·9-54·4)	53·2 (51·5-54·9)	54·7 (53·4-56·0)	54·4 (53·3-55·5)	54·0 (52·8-55·2)	54·1 (52·9-55·3)	55·9 (54·8-57·1)	58·5 (57·3-59·7)	62·8 (61·2-64·4)
Angola	51·7 (47·8-55·9)	52·5 (49·0-56·3)	52·7 (49·8-56·1)	52·9 (50·3-56·0)	52·2 (49·6-55·2)	53·1 (50·5-56·3)	54·0 (51·3-57·1)	56·7 (53·8-59·6)	60·4 (57·3-63·5)	65·4 (61·9-68·7)
Central African Republic	49·9 (47·0-53·2)	50·3 (47·4-53·2)	51·8 (49·3-54·3)	52·7 (50·6-55·0)	51·0 (48·8-53·3)	49·0 (46·3-51·8)	46·9 (43·8-50·3)	47·1 (43·9-50·7)	49·6 (46·1-53·4)	52·6 (48·8-56·6)
Congo	48·6 (45·2-52·1)	50·9 (47·6-54·1)	53·2 (50·1-56·4)	55·9 (53·6-58·6)	56·0 (53·8-58·1)	54·2 (52·4-56·0)	53·3 (51·6-55·3)	55·9 (54·0-57·8)	59·3 (57·2-61·8)	62·4 (59·6-65·6)
Democratic Republic of the Congo	50·9 (47·8-53·9)	52·3 (49·5-55·0)	53·4 (51·1-55·6)	55·3 (53·6-56·9)	55·3 (53·7-56·8)	54·6 (53·0-56·2)	54·8 (53·2-56·4)	56·4 (54·9-57·9)	58·4 (56·9-60·0)	62·7 (60·8-64·5)
Equatorial Guinea	45·8 (41·9-49·9)	47·7 (44·6-51·1)	50·4 (47·7-53·3)	51·3 (48·6-54·2)	49·8 (47·2-52·8)	49·2 (46·3-52·3)	55·2 (51·9-59·2)	58·7 (54·1-64·1)	61·2 (56·7-66·1)	66·6 (62·3-71·4)
Gabon	55·3 (52·4-58·8)	58·5 (55·8-61·7)	61·2 (59·1-64·1)	62·9 (60·9-65·2)	62·9 (61·2-64·5)	62·9 (61·3-64·3)	60·8 (59·0-62·5)	60·9 (59·2-62·8)	64·1 (62·0-66·3)	68·3 (65·9-71·1)

Appendix Table 14A. Estimates of life expectancy at age 65 by location, both sexes, 1970, 1975, 1980, 1985, 1990, 1995, 2000, 2005, 2010, 2016

Location	1970	1975	1980	1985	1990	1995	2000	2005	2010	2016
Global	13·6 (13·5-13·6)	13·9 (13·9-14·0)	14·2 (14·1-14·2)	14·4 (14·4-14·5)	14·7 (14·7-14·8)	14·9 (14·8-14·9)	15·3 (15·2-15·3)	15·8 (15·8-15·9)	16·6 (16·5-16·6)	17·2 (17·1-17·3)
High SDI	14·5 (14·4-14·5)	15·0 (15·0-15·1)	15·7 (15·7-15·8)	16·2 (16·2-16·3)	16·9 (16·8-16·9)	17·4 (17·4-17·5)	18·1 (18·1-18·1)	18·9 (18·9-19·0)	19·7 (19·7-19·8)	20·1 (20·0-20·2)
High-middle SDI	14·4 (14·3-14·6)	14·6 (14·5-14·8)	14·6 (14·5-14·8)	14·8 (14·7-15·0)	15·1 (14·9-15·3)	14·7 (14·5-14·9)	15·2 (15·0-15·3)	15·7 (15·5-15·8)	16·8 (16·6-16·9)	17·7 (17·1-18·1)
Middle SDI	13·3 (13·2-13·4)	13·6 (13·5-13·8)	13·6 (13·5-13·7)	13·8 (13·7-13·9)	13·9 (13·8-14·0)	14·2 (14·1-14·3)	14·6 (14·5-14·6)	15·2 (15·1-15·2)	15·9 (15·8-16·0)	16·7 (16·6-16·8)
Low-middle SDI	11·4 (11·2-11·5)	11·7 (11·6-11·8)	12·1 (12·0-12·2)	12·4 (12·2-12·5)	12·6 (12·5-12·7)	12·8 (12·7-12·9)	13·1 (13·0-13·2)	13·6 (13·5-13·7)	14·1 (13·9-14·2)	14·6 (14·5-14·8)
Low SDI	11·1 (10·9-11·3)	11·3 (11·1-11·4)	11·4 (11·3-11·6)	11·6 (11·4-11·7)	11·7 (11·5-11·8)	11·7 (11·5-11·9)	11·9 (11·7-12·1)	12·3 (12·1-12·5)	12·7 (12·4-12·9)	13·3 (13·1-13·5)
High-income	14·6 (14·5-14·6)	15·1 (15·1-15·2)	15·9 (15·8-15·9)	16·4 (16·3-16·4)	17·1 (17·0-17·1)	17·6 (17·6-17·7)	18·3 (18·2-18·3)	19·1 (19·1-19·1)	19·8 (19·8-19·9)	20·2 (20·2-20·3)
High-income North America	15·1 (15·0-15·1)	15·9 (15·8-15·9)	16·6 (16·5-16·6)	16·8 (16·8-16·9)	17·3 (17·3-17·4)	17·5 (17·5-17·6)	17·9 (17·9-17·9)	18·6 (18·6-18·7)	19·4 (19·4-19·5)	19·6 (19·4-19·7)
Canada	15·6 (15·5-15·8)	16·0 (15·9-16·2)	16·8 (16·6-16·9)	17·2 (17·1-17·4)	17·7 (17·6-17·9)	18·0 (17·9-18·2)	18·6 (18·5-18·8)	19·4 (19·3-19·6)	20·3 (20·1-20·4)	20·6 (20·4-20·9)
Greenland	11·0 (10·6-11·3)	10·1 (9·8-10·5)	10·1 (9·8-10·5)	9·8 (9·5-10·1)	10·1 (9·8-10·5)	10·5 (10·2-10·9)	10·8 (10·4-11·1)	10·9 (10·6-11·3)	12·1 (11·6-12·6)	12·9 (11·9-13·9)
United States	15·1 (15·0-15·1)	15·8 (15·8-15·9)	16·5 (16·5-16·6)	16·8 (16·8-16·9)	17·3 (17·2-17·3)	17·5 (17·4-17·5)	17·8 (17·8-17·9)	18·5 (18·5-18·6)	19·3 (19·3-19·4)	19·4 (19·3-19·5)
Australasia	14·2 (14·1-14·3)	15·0 (14·9-15·1)	15·8 (15·7-15·9)	16·4 (16·2-16·5)	17·2 (17·0-17·3)	17·9 (17·7-18·0)	18·9 (18·8-19·0)	19·8 (19·7-19·9)	20·4 (20·3-20·6)	20·8 (20·4-21·1)
Australia	14·2 (14·1-14·3)	15·1 (14·9-15·2)	16·0 (15·9-16·1)	16·5 (16·4-16·6)	17·2 (17·1-17·4)	18·0 (17·8-18·1)	19·0 (18·8-19·1)	19·9 (19·8-20·0)	20·5 (20·4-20·7)	20·9 (20·5-21·2)
New Zealand	14·4 (14·3-14·6)	14·8 (14·7-15·0)	15·2 (15·1-15·4)	15·7 (15·6-15·9)	16·7 (16·5-16·8)	17·4 (17·2-17·6)	18·3 (18·2-18·5)	19·2 (19·0-19·3)	19·9 (19·8-20·1)	20·2 (19·6-20·8)
High-income Asia Pacific	14·0 (13·7-14·3)	14·8 (14·6-15·0)	15·9 (15·8-16·0)	16·9 (16·8-17·0)	17·8 (17·7-17·9)	18·6 (18·4-18·7)	19·5 (19·4-19·6)	20·4 (20·3-20·5)	21·1 (21·0-21·2)	21·7 (21·3-22·0)
Brunei	13·7 (13·1-14·5)	14·5 (13·8-15·2)	13·9 (13·2-15·2)	14·9 (14·5-15·2)	15·6 (15·2-15·9)	16·1 (15·7-16·5)	16·9 (16·6-17·2)	17·1 (16·9-17·4)	17·3 (17·0-17·6)	17·3 (16·7-18·3)
Japan	14·2 (14·0-14·4)	15·2 (15·1-15·4)	16·3 (16·3-16·4)	17·4 (17·4-17·5)	18·3 (18·3-18·3)	19·0 (19·0-19·1)	20·0 (20·0-20·0)	20·8 (20·8-20·8)	21·4 (21·4-21·4)	22·0 (21·9-22·2)
Singapore	12·6 (12·0-13·2)	13·2 (12·6-13·8)	13·8 (13·2-14·5)	14·7 (14·1-15·6)	16·2 (15·5-16·9)	17·1 (16·4-17·8)	18·5 (17·7-19·3)	19·6 (18·9-20·3)	21·0 (20·2-21·7)	21·6 (20·3-22·9)
South Korea	12·7 (11·1-14·6)	12·4 (11·3-13·5)	13·3 (12·8-13·9)	14·1 (13·4-14·7)	14·8 (14·3-15·5)	15·8 (15·1-16·4)	16·7 (16·2-17·3)	18·1 (17·5-18·7)	19·3 (18·8-20·0)	19·8 (18·1-21·6)
Western Europe	14·4 (14·3-14·5)	14·8 (14·7-14·9)	15·5 (15·4-15·6)	16·1 (16·0-16·2)	16·8 (16·7-16·9)	17·4 (17·4-17·5)	18·2 (18·1-18·2)	19·0 (18·9-19·1)	19·8 (19·7-19·8)	20·2 (20·1-20·4)
Andorra	17·6 (15·7-19·4)	17·9 (16·4-19·6)	18·5 (17·3-19·8)	18·7 (17·5-20·0)	19·0 (17·9-20·1)	19·5 (18·3-20·6)	20·5 (19·4-21·4)	20·9 (20·0-21·7)	21·0 (20·0-21·8)	20·8 (19·7-21·9)
Austria	13·6 (13·5-13·7)	14·2 (14·1-14·3)	14·8 (14·7-15·0)	15·6 (15·5-15·7)	16·5 (16·4-16·6)	17·2 (17·0-17·3)	18·0 (17·9-18·1)	18·9 (18·7-19·0)	19·5 (19·4-19·7)	20·1 (19·7-20·4)
Belgium	13·8 (13·6-14·0)	14·2 (14·1-14·4)	14·9 (14·8-15·1)	15·6 (15·4-15·8)	16·6 (16·4-16·8)	17·2 (17·0-17·4)	17·8 (17·5-18·0)	18·6 (18·5-18·9)	19·3 (19·1-19·5)	19·8 (19·2-20·4)
Cyprus	12·9 (12·7-13·2)	12·6 (12·3-12·9)	13·3 (13·0-13·5)	14·2 (14·0-14·4)	14·9 (14·6-15·1)	15·7 (15·4-15·9)	16·6 (16·4-16·8)	17·5 (17·2-17·7)	18·5 (18·3-18·8)	18·8 (18·5-19·2)
Denmark	15·2 (14·9-15·5)	15·6 (15·3-15·9)	15·8 (15·4-16·1)	16·1 (15·7-16·4)	16·2 (15·9-16·6)	16·2 (15·8-16·6)	16·9 (16·5-17·3)	17·6 (17·2-18·0)	18·5 (18·2-18·9)	19·4 (18·8-20·2)
Finland	13·2 (13·1-13·4)	14·2 (14·0-14·4)	15·1 (15·0-15·3)	15·7 (15·5-15·8)	16·1 (15·9-16·3)	17·0 (16·8-17·1)	17·8 (17·6-17·9)	18·8 (18·7-19·0)	19·4 (19·3-19·6)	20·3 (19·9-20·8)
France	15·2 (15·1-15·3)	15·7 (15·5-15·8)	16·5 (16·3-16·6)	17·2 (17·0-17·3)	18·2 (18·0-18·3)	18·7 (18·6-18·9)	19·2 (19·1-19·4)	20·1 (19·9-20·2)	20·9 (20·7-21·0)	21·4 (21·1-21·7)
Germany	13·7 (13·4-13·9)	14·1 (13·9-14·4)	14·9 (14·6-15·1)	15·5 (15·2-15·8)	16·3 (16·0-16·5)	17·0 (16·6-17·2)	17·7 (17·5-18·0)	18·5 (18·3-18·8)	19·1 (18·9-19·4)	19·6 (19·1-20·1)
Greece	15·8 (15·7-16·0)	16·3 (16·1-16·4)	18·3 (18·1-18·5)	16·7 (16·6-16·9)	16·9 (16·7-17·0)	17·4 (17·3-17·6)	18·0 (17·8-18·1)	18·7 (18·6-18·9)	19·4 (19·2-19·5)	19·7 (19·3-20·2)
Iceland	15·9 (15·7-16·2)	16·7 (16·5-17·0)	17·4 (17·1-17·7)	17·5 (17·2-17·7)	17·7 (17·4-18·0)	18·0 (17·7-18·3)	18·7 (18·4-19·0)	19·5 (19·2-19·8)	20·0 (19·7-20·3)	20·2 (19·7-20·7)
Ireland	13·7 (13·5-14·0)	13·9 (13·6-14·1)	14·1 (13·9-14·4)	14·6 (14·3-14·9)	15·2 (14·9-15·5)	15·7 (15·4-15·9)	16·4 (16·1-16·7)	18·0 (17·7-18·3)	19·1 (18·9-19·4)	19·6 (18·8-20·4)
Israel	13·8 (13·2-14·4)	14·4 (13·9-14·9)	14·9 (14·3-15·5)	15·3 (14·7-15·8)	16·0 (15·4-16·5)	16·7 (16·1-17·3)	17·9 (17·3-18·6)	18·7 (18·1-19·3)	20·0 (19·4-20·5)	20·3 (19·2-21·4)
Italy	14·7 (14·6-14·8)	15·0 (14·8-15·1)	15·6 (15·4-15·7)	16·3 (16·1-16·4)	17·2 (17·1-17·3)	17·9 (17·7-18·0)	18·7 (18·6-18·8)	19·5 (19·4-19·6)	20·1 (19·9-20·2)	20·4 (20·0-20·8)
Luxembourg	13·5 (13·3-13·7)	13·9 (13·7-14·1)	14·6 (14·4-14·8)	15·3 (15·1-15·5)	16·2 (15·9-16·4)	17·1 (16·9-17·4)	18·1 (17·9-18·3)	19·1 (18·8-19·3)	19·9 (19·7-20·1)	20·3 (19·7-20·9)
Malta	12·4 (11·9-12·9)	12·7 (12·3-13·2)	13·0 (12·5-13·4)	14·2 (13·7-14·7)	15·5 (15·0-16·0)	16·4 (15·8-16·9)	16·6 (16·1-17·1)	18·0 (17·5-18·6)	19·0 (18·5-19·4)	19·8 (18·7-20·8)
Netherlands	15·1 (14·9-15·4)	15·6 (15·4-15·8)	16·3 (16·0-16·5)	16·5 (16·3-16·8)	16·9 (16·7-17·2)	17·1 (16·9-17·3)	17·4 (17·2-17·7)	18·4 (18·1-18·6)	19·3 (19·0-19·5)	19·8 (19·3-20·3)
Norway	15·3 (15·1-15·5)	15·8 (15·6-16·0)	16·3 (16·0-16·5)	16·6 (16·3-16·8)	16·9 (16·7-17·2)	17·4 (17·1-17·6)	18·0 (17·8-18·2)	19·0 (18·7-19·2)	19·6 (19·3-19·8)	20·2 (19·6-20·8)
Portugal	13·7 (13·6-13·9)	14·3 (14·1-14·5)	14·9 (14·8-15·1)	15·6 (15·4-15·8)	16·1 (15·9-16·2)	16·6 (16·4-16·8)	17·3 (17·1-17·4)	18·2 (18·1-18·4)	19·2 (19·0-19·3)	19·8 (19·5-20·2)
Spain	14·8 (14·7-14·9)	15·3 (15·2-15·4)	16·4 (16·3-16·5)	16·9 (16·8-17·0)	17·6 (17·5-17·7)	18·2 (18·1-18·3)	18·8 (18·7-18·9)	19·5 (19·4-19·6)	20·5 (20·4-20·6)	21·1 (20·8-21·4)
Sweden	15·5 (15·2-15·8)	15·7 (15·5-16·0)	16·2 (16·0-16·4)	16·8 (16·6-17·0)	17·3 (17·1-17·5)	17·9 (17·8-18·1)	18·4 (18·3-18·6)	19·0 (18·8-19·2)	19·7 (19·5-19·9)	20·1 (19·4-20·7)
Stockholm	15·3 (14·1-16·6)	15·7 (14·8-16·6)	16·4 (16·1-16·8)	17·0 (16·7-17·3)	17·3 (17·0-17·6)	18·0 (17·7-18·3)	18·5 (18·2-18·7)	19·2 (18·9-19·5)	19·9 (19·6-20·2)	20·2 (18·9-21·3)
Sweden except Stockholm	15·5 (15·2-15·8)	15·7 (15·5-16·0)	16·1 (15·9-16·3)	16·7 (16·5-16·9)	17·3 (17·1-17·5)	17·9 (17·7-18·1)	18·4 (18·2-18·6)	19·0 (18·8-19·2)	19·7 (19·5-19·9)	20·0 (19·3-20·8)
Switzerland	14·9 (14·5-15·2)	15·8 (15·5-16·2)	16·4 (16·0-16·8)	17·2 (16·8-17·6)	17·7 (17·3-18·0)	18·3 (17·9-18·6)	19·1 (18·7-19·4)	19·9 (19·6-20·2)	20·5 (20·2-20·9)	21·1 (19·8-22·4)
United Kingdom	14·3 (14·2-14·4)	14·5 (14·5-14·6)	15·0 (15·0-15·1)	15·4 (15·4-15·5)	16·1 (16·0-16·1)	16·6 (16·5-16·6)	17·4 (17·3-17·5)	18·3 (18·3-18·4)	19·3 (19·3-19·4)	19·6 (19·5-19·7)
England	14·4 (14·3-14·5)	14·6 (14·6-14·7)	15·1 (15·1-15·2)	15·5 (15·5-15·6)	16·2 (16·2-16·3)	16·7 (16·6-16·7)	17·5 (17·5-17·6)	18·5 (18·4-18·5)	19·5 (19·5-19·6)	19·8 (19·7-19·9)
East Midlands	14·2 (13·8-14·5)	14·5 (14·2-14·8)	15·0 (14·8-15·3)	15·5 (15·3-15·6)	16·1 (16·0-16·3)	16·6 (16·4-16·7)	17·5 (17·3-17·6)	18·3 (18·2-18·5)	19·3 (19·1-19·5)	19·6 (19·4-19·9)
East of England	14·7 (14·3-15·0)	15·0 (14·7-15·3)	15·5 (15·3-15·7)	15·9 (15·8-16·1)	16·7 (16·5-16·8)	17·2 (17·0-17·3)	17·9 (17·8-18·1)	19·0 (18·8-19·1)	20·0 (19·8-20·1)	20·2 (19·9-20·5)
Greater London	14·9 (14·7-15·1)	15·1 (14·9-15·2)	15·5 (15·3-15·6)	15·8 (15·7-15·9)	16·5 (16·4-16·6)	16·9 (16·8-17·0)	17·6 (17·5-17·7)	18·6 (18·5-18·7)	19·8 (19·7-19·9)	20·2 (20·0-20·3)
North East England	13·3 (13·0-13·6)	13·7 (13·4-13·9)	14·2 (14·0-14·4)	14·5 (14·3-14·7)	15·1 (15·0-15·3)	15·5 (15·3-15·6)	16·5 (16·4-16·7)	17·4 (17·3-17·6)	18·5 (18·4-18·7)	18·9 (18·6-19·1)
North West England	13·7 (13·5-13·9)	14·0 (13·8-14·2)	14·4 (14·3-14·6)	14·9 (14·7-15·0)	15·4 (15·3-15·5)	15·9 (15·8-16·0)	16·8 (16·7-16·9)	17·7 (17·6-17·8)	18·7 (18·6-18·8)	19·0 (18·9-19·2)
South East England	15·0 (14·7-15·3)	15·3 (15·0-15·5)	15·8 (15·6-15·9)	16·1 (16·0-16·3)	16·8 (16·7-17·0)	17·3 (17·1-17·4)	18·1 (18·0-18·2)	19·1 (19·0-19·2)	20·1 (20·0-20·2)	20·3 (20·1-20·5)
South West England	15·0 (14·8-15·3)	15·3 (15·1-15·6)	15·8 (15·6-16·0)	16·3 (16·2-16·4)	17·0 (16·8-17·1)	17·5 (17·4-17·6)	18·2 (18·1-18·4)	19·2 (19·1-19·3)	20·1 (20·0-20·2)	20·3 (20·1-20·5)
West Midlands	14·0 (13·7-14·3)	14·3 (14·0-14·6)	14·7 (14·5-14·9)	15·2 (15·0-15·3)	15·8 (15·7-16·0)	16·4 (16·2-16·5)	17·2 (17·1-17·4)	18·2 (18·1-18·3)	19·3 (19·2-19·5)	19·6 (19·4-19·8)
Yorkshire and the Humber	13·9 (13·7-14·2)	14·2 (14·0-14·4)	14·7 (14·5-14·9)	15·1 (15·0-15·3)	15·8 (15·7-15·9)	16·3 (16·2-16·4)	17·2 (17·0-17·3)	18·1 (18·0-18·3)	19·0 (18·9-19·1)	19·3 (19·1-19·5)
Northern Ireland	13·8 (13·5-14·2)	14·1 (13·7-14·4)	14·5 (14·2-14·8)	14·9 (14·5-15·4)	15·6 (15·2-16·1)	16·3 (15·8-16·8)	17·1 (16·6-17·6)	18·2 (17·7-18·6)	19·1 (18·6-19·5)	19·2 (18·5-20·1)
Scotland	13·7 (13·4-13·9)	13·9 (13·6-14·2)	14·4 (14·1-14·7)	14·6 (14·2-15·0)	15·1 (14·7-15·5)	15·6 (15·2-16·0)	16·5 (16·0-16·9)	17·2 (16·8-17·7)	18·2 (17·8-18·6)	18·5 (17·8-19·1)

Appendix Table 14A. Estimates of life expectancy at age 65 by location, both sexes, 1970, 1975, 1980, 1985, 1990, 1995, 2000, 2005, 2010, 2016

Location	1970	1975	1980	1985	1990	1995	2000	2005	2010	2016
Wales	13·9 (13·1-14·9)	14·2 (13·4-15·1)	14·8 (14·2-15·4)	15·4 (14·9-15·8)	15·9 (15·5-16·3)	16·3 (15·9-16·7)	17·1 (16·7-17·6)	18·1 (17·7-18·5)	18·9 (18·5-19·3)	19·1 (18·4-19·8)
Southern Latin America	14·4 (14·3-14·7)	15·0 (14·7-15·3)	15·3 (15·1-15·4)	15·6 (15·4-15·8)	16·0 (15·8-16·2)	16·6 (16·4-16·8)	17·0 (16·8-17·2)	17·4 (17·2-17·6)	17·8 (17·6-18·0)	18·4 (17·9-18·9)
Argentina	14·3 (14·0-14·5)	14·8 (14·4-15·2)	15·1 (14·9-15·4)	15·6 (15·4-15·8)	16·0 (15·7-16·2)	16·5 (16·3-16·7)	16·7 (16·5-16·9)	17·0 (16·8-17·2)	17·4 (17·1-17·6)	17·8 (17·4-18·3)
Chile	14·9 (14·6-15·3)	15·7 (15·3-16·0)	15·7 (15·3-16·1)	15·8 (15·4-16·1)	16·3 (15·9-16·7)	17·0 (16·6-17·4)	18·0 (17·6-18·3)	18·7 (18·3-19·0)	19·3 (19·0-19·7)	19·9 (18·4-21·5)
Uruguay	14·7 (14·5-14·9)	14·9 (14·8-15·1)	15·2 (15·0-15·4)	15·5 (15·3-15·6)	15·8 (15·6-16·0)	16·2 (16·0-16·4)	16·9 (16·7-17·0)	17·2 (17·1-17·4)	17·7 (17·5-17·9)	18·2 (17·9-18·6)
Central Europe, Eastern Europe, and Central Asi	14·3 (14·1-14·5)	14·4 (14·2-14·6)	14·3 (14·0-14·5)	14·2 (14·0-14·4)	14·6 (14·3-14·8)	13·7 (13·5-13·9)	14·1 (13·9-14·3)	14·3 (14·1-14·6)	15·4 (15·2-15·6)	16·3 (15·4-17·0)
Eastern Europe	14·7 (14·4-15·0)	14·6 (14·2-14·9)	14·4 (14·0-14·7)	14·2 (13·9-14·5)	14·6 (14·3-15·0)	13·3 (13·0-13·6)	13·6 (13·2-13·9)	13·8 (13·4-14·1)	14·9 (14·6-15·3)	15·8 (14·4-17·1)
Belarus	16·1 (15·8-16·4)	16·2 (15·9-16·4)	15·6 (15·4-15·9)	15·3 (15·0-15·5)	15·3 (15·0-15·5)	14·1 (13·9-14·4)	14·0 (13·8-14·3)	14·2 (13·9-14·4)	14·8 (14·6-15·0)	16·2 (15·2-17·4)
Estonia	14·4 (14·2-14·7)	14·5 (14·3-14·7)	14·3 (14·1-14·6)	14·3 (14·1-14·5)	14·7 (14·4-14·9)	14·3 (14·1-14·5)	15·3 (15·0-15·5)	16·1 (15·9-16·4)	17·6 (17·4-17·9)	18·4 (17·8-19·2)
Latvia	14·8 (14·5-15·1)	14·6 (14·3-14·9)	14·5 (14·2-14·7)	14·3 (14·0-14·5)	14·6 (14·3-14·9)	14·1 (13·9-14·4)	15·3 (15·0-15·6)	15·3 (15·0-15·5)	16·3 (16·0-16·6)	17·2 (16·4-18·0)
Lithuania	15·5 (15·3-15·7)	15·8 (15·6-16·0)	15·6 (15·4-15·9)	15·4 (15·2-15·6)	15·7 (15·5-15·9)	15·0 (14·8-15·2)	16·2 (16·0-16·4)	16·1 (15·9-16·3)	16·7 (16·5-17·0)	17·6 (17·2-18·1)
Moldova	12·6 (11·5-13·9)	13·0 (12·0-14·1)	13·3 (12·7-14·0)	12·9 (12·4-13·5)	13·5 (13·0-13·9)	12·1 (11·7-12·5)	13·4 (13·0-13·8)	13·3 (12·9-13·8)	13·9 (13·4-14·4)	15·1 (14·3-15·8)
Russia	14·5 (14·0-15·1)	14·4 (13·9-14·9)	14·2 (13·7-14·8)	14·1 (13·6-14·6)	14·6 (14·1-15·2)	13·2 (12·7-13·7)	13·4 (12·9-13·9)	13·6 (13·1-14·2)	14·9 (14·4-15·4)	15·7 (13·8-17·6)
Ukraine	14·9 (14·6-15·1)	14·7 (14·4-15·1)	14·5 (14·2-14·8)	14·3 (14·0-14·6)	14·6 (14·3-14·9)	13·3 (13·0-13·6)	13·7 (13·5-14·1)	13·7 (13·4-14·0)	14·8 (14·5-15·1)	15·7 (14·2-17·1)
Central Europe	13·7 (13·6-13·8)	14·0 (13·9-14·1)	13·9 (13·8-14·0)	14·1 (14·0-14·2)	14·4 (14·3-14·5)	14·5 (14·4-14·6)	15·2 (15·1-15·3)	15·8 (15·7-15·9)	16·5 (16·4-16·6)	17·3 (17·1-17·6)
Albania	16·7 (16·2-17·3)	17·0 (16·4-17·6)	17·2 (16·7-17·8)	17·1 (16·7-17·4)	16·6 (16·4-16·8)	16·9 (16·7-17·0)	16·4 (16·2-16·6)	16·2 (16·0-16·3)	16·9 (16·5-17·3)	17·8 (17·0-18·7)
Bosnia and Herzegovina	13·5 (12·3-14·5)	13·6 (12·7-14·7)	13·8 (12·9-14·7)	14·0 (13·2-14·8)	14·4 (13·7-15·2)	14·4 (13·7-15·1)	15·7 (15·0-16·4)	16·1 (15·4-16·8)	16·7 (15·9-17·4)	17·2 (16·3-18·2)
Bulgaria	14·0 (13·8-14·1)	13·8 (13·6-14·0)	13·8 (13·6-14·0)	13·8 (13·6-13·9)	14·1 (13·9-14·2)	14·0 (13·9-14·2)	14·2 (14·0-14·4)	14·7 (14·6-14·9)	15·5 (15·3-15·7)	16·1 (15·2-17·1)
Croatia	13·1 (11·7-14·4)	13·5 (12·2-14·9)	13·9 (12·7-15·2)	14·1 (13·6-14·7)	14·7 (14·2-15·1)	15·0 (14·6-15·4)	15·3 (14·9-15·7)	15·8 (15·4-16·2)	16·5 (16·1-16·9)	17·0 (16·3-17·9)
Czech Republic	12·8 (12·7-13·0)	13·2 (13·0-13·3)	13·1 (13·0-13·3)	13·3 (13·2-13·5)	13·8 (13·7-13·9)	14·7 (14·6-14·8)	15·6 (15·5-15·7)	16·4 (16·2-16·5)	17·2 (17·0-17·3)	18·2 (17·8-18·5)
Hungary	13·3 (13·1-13·6)	13·5 (13·3-13·8)	13·4 (13·1-13·6)	13·5 (13·2-13·8)	14·0 (13·7-14·2)	14·3 (14·0-14·6)	15·0 (14·7-15·3)	15·7 (15·4-15·9)	16·3 (16·0-16·6)	16·8 (16·2-17·5)
Macedonia	14·3 (13·8-14·7)	14·2 (13·7-14·6)	13·9 (13·6-14·3)	13·9 (13·6-14·2)	13·6 (13·2-13·9)	13·3 (13·0-13·6)	13·5 (13·2-13·8)	13·8 (13·5-14·1)	14·3 (14·0-14·6)	15·0 (14·7-15·4)
Montenegro	15·9 (15·3-16·5)	16·1 (15·4-16·8)	16·3 (15·7-16·9)	16·5 (15·7-17·0)	16·9 (16·5-17·4)	15·9 (15·4-16·3)	15·7 (15·4-16·1)	16·2 (15·9-16·6)	17·0 (16·4-17·5)	17·2 (16·6-17·9)
Poland	14·0 (13·8-14·2)	14·3 (14·1-14·5)	14·2 (14·0-14·4)	14·2 (14·0-14·4)	14·5 (14·3-14·7)	14·9 (14·7-15·1)	15·7 (15·5-15·9)	16·7 (16·5-17·0)	17·4 (17·2-17·6)	18·3 (17·8-18·9)
Romania	13·8 (13·5-14·0)	14·1 (13·9-14·4)	13·9 (13·6-14·1)	14·1 (13·8-14·3)	14·4 (14·1-14·7)	14·0 (13·8-14·3)	14·9 (14·7-15·2)	15·3 (15·1-15·6)	15·9 (15·6-16·2)	16·6 (16·0-17·2)
Serbia	14·6 (14·1-15·1)	15·0 (14·4-15·7)	15·2 (14·4-15·9)	15·2 (14·5-16·0)	14·9 (14·5-15·5)	14·1 (13·8-14·5)	14·2 (13·8-14·5)	14·5 (14·2-14·9)	15·4 (15·1-15·7)	16·5 (16·2-16·8)
Slovakia	13·5 (13·3-13·8)	13·9 (13·6-14·1)	13·8 (13·6-14·1)	13·9 (13·7-14·1)	14·0 (13·8-14·3)	14·6 (14·4-14·9)	15·0 (14·7-15·2)	15·5 (15·3-15·8)	16·3 (16·1-16·5)	17·2 (16·5-17·9)
Slovenia	13·1 (11·7-14·5)	13·5 (12·2-14·8)	13·9 (13·1-14·8)	14·5 (14·0-14·9)	15·5 (15·0-15·9)	15·9 (15·5-16·4)	16·7 (16·3-17·2)	17·6 (17·2-18·1)	18·8 (18·3-19·2)	19·6 (18·9-20·4)
Central Asia	13·9 (13·3-14·4)	14·3 (13·6-14·8)	14·7 (14·3-15·0)	14·8 (14·6-15·0)	14·7 (14·5-14·9)	13·7 (13·5-13·9)	13·7 (13·5-13·9)	13·6 (13·4-13·8)	14·3 (14·1-14·5)	15·3 (15·0-15·7)
Armenia	15·6 (14·6-16·9)	16·0 (14·8-17·2)	16·2 (15·6-16·8)	16·1 (15·7-16·4)	15·4 (15·1-15·8)	14·7 (14·4-15·1)	15·4 (15·1-15·8)	15·3 (14·9-15·7)	15·4 (15·0-15·7)	16·8 (16·2-17·5)
Azerbaijan	14·2 (13·4-15·2)	14·7 (13·9-15·6)	14·8 (14·2-15·5)	14·6 (14·1-15·0)	14·5 (14·2-14·9)	13·8 (13·4-14·2)	13·6 (13·2-14·0)	13·6 (13·1-14·1)	13·7 (13·2-14·2)	15·6 (14·6-16·7)
Georgia	14·8 (13·7-16·2)	15·2 (13·9-16·6)	15·6 (14·9-16·5)	15·2 (14·7-15·7)	15·1 (14·6-15·6)	15·5 (15·0-15·9)	15·6 (15·1-16·1)	15·4 (15·0-15·8)	16·0 (15·5-16·3)	16·4 (15·4-17·4)
Kazakhstan	13·2 (11·5-15·2)	13·7 (11·9-15·6)	14·2 (13·1-15·2)	14·6 (13·9-15·4)	14·6 (14·1-15·2)	13·0 (12·5-13·6)	13·2 (12·6-13·8)	13·1 (12·6-13·7)	14·0 (13·5-14·6)	15·4 (14·3-16·5)
Kyrgyzstan	13·7 (12·6-15·0)	14·2 (13·1-15·3)	14·9 (14·4-15·4)	14·7 (14·4-15·0)	14·6 (14·3-14·8)	13·2 (12·9-13·4)	14·0 (13·7-14·2)	13·3 (13·1-13·6)	14·0 (13·7-14·2)	15·3 (14·7-15·8)
Mongolia	11·5 (11·0-12·1)	12·8 (12·1-13·5)	12·8 (12·1-13·5)	13·0 (12·3-13·6)	13·3 (12·7-13·8)	12·2 (11·7-12·6)	12·1 (11·7-12·5)	12·3 (11·9-12·7)	13·0 (12·5-13·5)	14·0 (13·3-14·8)
Tajikistan	13·9 (13·4-14·6)	14·3 (13·7-15·0)	14·7 (14·1-15·4)	15·0 (14·4-15·6)	14·9 (14·4-15·5)	14·3 (13·8-14·9)	13·6 (13·1-14·2)	13·3 (12·8-13·9)	14·4 (13·8-15·0)	15·9 (14·8-16·6)
Turkmenistan	13·3 (12·9-13·8)	13·7 (13·3-14·0)	14·0 (13·7-14·3)	14·2 (14·0-14·4)	14·1 (13·9-14·2)	13·5 (13·4-13·7)	13·8 (13·6-14·0)	14·2 (14·0-14·4)	14·8 (14·6-15·0)	15·5 (15·1-15·9)
Uzbekistan	13·9 (13·1-14·5)	14·2 (13·3-15·0)	14·7 (14·1-15·2)	15·0 (14·6-15·4)	14·7 (14·4-15·1)	13·4 (13·1-13·8)	13·1 (12·8-13·4)	12·9 (12·5-13·2)	14·0 (13·7-14·3)	14·6 (14·0-15·1)
Latin America and Caribbean	14·4 (14·3-14·6)	15·0 (15·0-15·1)	15·5 (15·4-15·5)	15·4 (15·4-15·5)	16·0 (15·9-16·0)	16·2 (16·2-16·3)	16·9 (16·8-16·9)	17·4 (17·3-17·4)	17·7 (17·5-17·8)	18·2 (18·1-18·3)
Central Latin America	15·0 (14·9-15·2)	15·5 (15·4-15·6)	16·1 (16·0-16·2)	16·2 (16·1-16·3)	16·9 (16·8-17·0)	17·1 (17·0-17·2)	17·8 (17·7-17·9)	17·9 (17·8-18·0)	18·2 (18·1-18·3)	18·6 (18·4-18·8)
Colombia	16·4 (16·2-16·6)	15·5 (15·4-15·7)	16·0 (15·8-16·3)	15·7 (15·5-15·9)	16·7 (16·5-16·9)	16·8 (16·6-17·0)	17·2 (17·0-17·4)	17·5 (17·3-17·6)	18·1 (17·9-18·3)	19·0 (18·4-19·6)
Costa Rica	15·4 (15·2-15·6)	16·2 (15·9-16·4)	17·0 (16·8-17·2)	17·3 (17·1-17·6)	17·6 (17·4-17·9)	18·0 (17·7-18·2)	18·7 (18·4-18·9)	19·7 (19·5-19·9)	20·6 (20·4-20·8)	20·9 (20·5-21·4)
El Salvador	14·7 (14·4-15·0)	15·1 (14·8-15·4)	17·1 (16·7-17·4)	16·2 (15·9-16·5)	16·4 (16·2-16·7)	16·8 (16·6-17·1)	17·7 (17·4-18·0)	18·1 (17·8-18·3)	18·6 (18·3-18·9)	18·8 (18·2-19·5)
Guatemala	12·8 (12·0-13·5)	14·1 (13·2-14·9)	15·0 (14·1-15·9)	14·6 (13·9-15·3)	15·1 (14·5-15·9)	15·7 (15·0-16·5)	16·5 (15·7-17·4)	17·1 (16·2-17·9)	17·5 (16·7-18·3)	17·8 (16·4-19·4)
Honduras	13·5 (12·8-14·1)	14·4 (13·7-15·2)	14·8 (14·0-15·6)	14·2 (13·6-14·9)	14·7 (14·0-15·4)	14·8 (13·7-15·6)	14·9 (13·7-16·3)	15·2 (13·8-16·8)	15·5 (14·0-17·3)	15·9 (14·4-17·4)
Mexico	14·9 (14·7-15·1)	15·7 (15·5-15·9)	16·3 (16·2-16·4)	16·6 (16·5-16·7)	17·3 (17·2-17·4)	17·6 (17·5-17·6)	18·2 (18·1-18·3)	18·1 (18·0-18·2)	18·2 (18·1-18·3)	18·4 (18·2-18·6)
Nicaragua	15·4 (15·0-15·9)	17·4 (17·1-17·6)	17·7 (17·1-18·3)	17·8 (17·2-18·5)	19·7 (19·5-20·0)	19·0 (18·7-19·3)	19·4 (19·2-19·7)	19·4 (19·2-19·7)	19·5 (19·2-19·7)	19·8 (18·9-20·6)
Panama	15·7 (15·3-16·1)	16·8 (16·4-17·2)	17·6 (17·1-18·0)	17·3 (16·9-17·7)	17·5 (17·1-18·0)	17·9 (17·4-18·2)	18·9 (18·4-19·3)	19·2 (18·8-19·7)	19·6 (19·2-20·1)	20·2 (19·4-21·0)
Venezuela	14·6 (14·2-15·0)	14·7 (14·3-15·0)	15·2 (14·8-15·5)	15·8 (15·4-16·2)	16·2 (15·9-16·6)	16·6 (16·1-17·1)	17·4 (17·0-17·8)	18·1 (17·7-18·5)	18·5 (18·0-18·9)	18·5 (17·5-19·5)
Andean Latin America	14·2 (13·6-14·9)	15·6 (15·3-15·9)	15·8 (15·5-16·1)	15·9 (15·6-16·2)	16·5 (16·2-16·8)	16·7 (16·4-17·0)	17·7 (17·5-18·0)	18·6 (18·3-18·8)	18·8 (18·4-19·1)	19·3 (18·6-19·9)
Bolivia	12·6 (12·0-13·2)	12·9 (12·2-13·4)	13·1 (12·5-13·7)	13·3 (12·6-13·9)	13·8 (13·1-14·4)	14·4 (13·9-15·0)	15·5 (15·0-15·8)	16·2 (15·6-16·7)	16·4 (15·4-17·2)	16·8 (15·6-17·9)
Ecuador	17·3 (17·1-17·6)	16·8 (16·5-17·0)	16·9 (16·6-17·2)	17·0 (16·7-17·3)	17·2 (17·0-17·5)	17·8 (17·5-18·0)	18·4 (18·1-18·7)	19·0 (18·8-19·3)	19·3 (19·1-19·6)	19·8 (19·4-20·2)
Peru	13·4 (12·3-14·6)	16·2 (15·7-16·7)	16·3 (15·9-16·7)	16·4 (16·0-16·7)	17·3 (16·8-17·7)	17·1 (16·7-17·6)	18·3 (17·9-18·7)	19·2 (18·8-19·7)	19·4 (18·8-19·9)	20·0 (18·9-21·1)
Caribbean	14·6 (14·4-14·9)	15·1 (14·8-15·3)	15·6 (15·4-15·8)	15·7 (15·5-15·9)	15·8 (15·7-16·0)	16·0 (15·9-16·2)	16·6 (16·5-16·8)	17·0 (16·8-17·3)	16·2 (15·3-17·2)	17·6 (17·2-18·0)
Antigua and Barbuda	12·6 (12·0-13·1)	12·9 (12·4-13·4)	13·8 (13·3-14·4)	14·5 (13·9-15·1)	15·8 (15·3-16·3)	16·9 (16·4-17·4)	17·2 (16·7-17·6)	17·7 (17·2-18·2)	18·2 (17·7-18·8)	18·4 (17·7-19·1)
The Bahamas	13·4 (12·7-13·9)	14·2 (13·8-14·5)	14·8 (14·6-15·0)	14·9 (14·6-15·2)	15·6 (15·3-15·8)	15·5 (15·3-15·8)	15·8 (15·5-16·0)	16·6 (16·4-17·0)	17·0 (16·7-17·4)	16·8 (16·1-17·5)

Appendix Table 14A. Estimates of life expectancy at age 65 by location, both sexes, 1970, 1975, 1980, 1985, 1990, 1995, 2000, 2005, 2010, 2016

Location	1970	1975	1980	1985	1990	1995	2000	2005	2010	2016
Barbados	14·7 (14·3-15·0)	15·1 (14·7-15·4)	15·7 (15·3-16·0)	15·8 (15·4-16·2)	15·9 (15·5-16·2)	16·1 (15·6-16·5)	17·3 (16·9-17·7)	18·0 (17·6-18·3)	18·5 (18·2-18·9)	18·2 (17·6-18·7)
Belize	15·8 (15·5-16·2)	15·9 (15·6-16·3)	15·9 (15·4-16·5)	16·1 (15·5-16·6)	16·4 (15·6-17·0)	15·3 (14·7-15·9)	14·3 (13·8-14·7)	14·6 (14·1-15·0)	15·1 (14·6-15·6)	15·2 (14·5-16·1)
Bermuda	12·1 (11·7-12·4)	12·3 (11·9-12·6)	12·8 (12·4-13·2)	13·1 (12·7-13·5)	13·2 (12·8-13·6)	13·6 (13·2-14·0)	13·7 (13·3-14·2)	15·6 (15·1-16·1)	16·9 (16·5-17·4)	18·3 (17·5-19·2)
Cuba	15·7 (15·4-15·9)	16·4 (16·1-16·6)	16·9 (16·7-17·2)	17·1 (16·8-17·3)	16·9 (16·7-17·2)	17·0 (16·7-17·2)	17·7 (17·5-18·0)	18·1 (17·9-18·4)	18·4 (18·2-18·7)	18·8 (18·3-19·3)
Dominica	13·8 (13·5-14·2)	14·3 (14·0-14·7)	15·5 (15·1-15·9)	15·1 (14·6-15·5)	14·3 (13·9-14·8)	14·1 (13·7-14·5)	15·1 (14·6-15·6)	15·5 (15·0-16·0)	15·8 (15·3-16·2)	16·0 (15·2-16·7)
Dominican Republic	16·1 (15·7-16·6)	16·6 (16·1-17·1)	16·9 (16·4-17·5)	16·6 (16·2-17·3)	16·7 (16·3-17·1)	17·0 (16·6-17·4)	17·7 (17·2-18·4)	18·0 (17·4-19·0)	18·2 (17·5-19·2)	18·4 (17·7-19·6)
Grenada	14·3 (13·4-15·2)	14·8 (14·1-15·6)	14·9 (14·2-15·9)	14·5 (13·9-15·4)	14·8 (14·2-15·3)	15·1 (14·6-15·5)	15·1 (14·6-15·6)	15·0 (14·5-15·4)	15·2 (14·7-15·7)	15·2 (14·5-16·1)
Guyana	12·5 (12·1-12·9)	12·6 (12·2-13·1)	13·0 (12·5-13·4)	13·6 (13·1-14·1)	13·2 (12·8-13·6)	13·9 (13·6-14·2)	14·4 (14·0-14·8)	13·4 (13·0-13·8)	13·1 (12·7-13·5)	14·1 (13·5-14·8)
Haiti	11·1 (10·2-12·4)	11·1 (10·4-12·2)	11·2 (10·5-12·1)	11·3 (10·7-11·9)	11·5 (11·1-11·9)	11·8 (11·4-12·2)	12·3 (11·8-12·8)	12·6 (12·0-13·4)	9·2 (7·2-12·2)	13·3 (12·3-14·4)
Jamaica	15·1 (14·6-15·5)	15·9 (15·4-16·5)	16·0 (15·5-16·5)	16·3 (15·9-16·8)	17·4 (16·9-17·9)	17·1 (16·4-17·7)	17·1 (16·6-17·5)	17·6 (17·2-18·0)	17·2 (16·6-17·8)	17·1 (16·1-18·1)
Puerto Rico	16·3 (16·0-16·6)	16·1 (15·8-16·4)	17·0 (16·7-17·3)	17·1 (16·8-17·4)	17·4 (17·1-17·7)	17·5 (17·2-17·8)	18·5 (18·2-18·8)	18·9 (18·6-19·2)	19·3 (19·0-19·6)	19·5 (18·9-20·1)
Saint Lucia	14·2 (13·8-14·6)	13·5 (13·2-13·7)	14·0 (13·8-14·3)	14·7 (14·5-15·0)	16·1 (15·8-16·3)	17·0 (16·7-17·3)	17·7 (17·5-18·0)	17·4 (17·2-17·7)	18·5 (18·2-18·9)	18·6 (18·2-19·0)
Saint Vincent and the Grenadines	14·8 (13·9-15·3)	14·5 (14·0-15·1)	14·8 (14·3-15·4)	14·8 (14·3-15·6)	14·8 (14·5-15·3)	14·8 (14·5-15·2)	14·8 (14·3-15·1)	15·0 (14·8-15·3)	15·2 (14·8-15·6)	15·2 (14·6-15·8)
Suriname	13·4 (13·1-13·9)	13·6 (13·3-13·9)	13·9 (13·7-14·1)	15·2 (15·0-15·4)	16·1 (15·9-16·3)	16·0 (15·8-16·3)	15·5 (15·1-15·8)	15·2 (14·8-15·5)	15·5 (15·2-15·9)	15·9 (15·3-16·5)
Trinidad and Tobago	12·5 (12·3-12·8)	13·2 (13·0-13·5)	14·0 (13·7-14·2)	14·1 (13·8-14·3)	14·0 (13·7-14·2)	14·2 (13·9-14·4)	14·6 (14·3-14·8)	15·7 (15·5-16·0)	16·4 (16·1-16·7)	16·6 (16·1-17·1)
Virgin Islands, U.S.	13·4 (12·8-13·7)	13·9 (13·5-14·3)	14·5 (14·1-14·8)	15·2 (14·9-15·7)	15·6 (15·2-16·2)	15·7 (15·3-16·1)	16·0 (15·6-16·3)	16·2 (15·9-16·5)	16·3 (15·8-16·7)	16·6 (16·0-17·3)
Tropical Latin America	13·8 (13·6-14·1)	14·5 (14·3-14·7)	14·6 (14·5-14·7)	14·5 (14·4-14·6)	14·9 (14·8-15·0)	15·2 (15·1-15·3)	15·8 (15·7-15·9)	16·6 (16·5-16·7)	17·3 (17·2-17·4)	17·8 (17·6-17·9)
Brazil	13·8 (13·5-14·0)	14·4 (14·2-14·6)	14·6 (14·4-14·7)	14·4 (14·3-14·5)	14·8 (14·7-14·9)	15·2 (15·0-15·3)	15·8 (15·7-15·9)	16·6 (16·5-16·7)	17·3 (17·2-17·4)	17·8 (17·6-18·0)
Paraguay	16·5 (16·3-16·7)	16·7 (16·4-16·9)	17·2 (16·9-17·5)	17·2 (16·9-17·4)	17·6 (17·3-17·9)	17·4 (16·9-17·7)	17·4 (17·1-17·6)	17·1 (16·7-17·4)	16·8 (16·3-17·3)	16·8 (16·1-17·6)
Southeast Asia, East Asia, and Oceania	13·2 (13·1-13·3)	13·5 (13·3-13·6)	13·3 (13·1-13·4)	13·6 (13·5-13·7)	13·5 (13·4-13·6)	13·8 (13·7-13·9)	14·2 (14·2-14·3)	15·0 (14·9-15·1)	15·9 (15·8-16·0)	16·8 (16·6-16·9)
East Asia	13·4 (13·3-13·6)	13·7 (13·5-13·8)	13·2 (13·1-13·4)	13·5 (13·4-13·7)	13·4 (13·2-13·5)	13·7 (13·6-13·8)	14·2 (14·1-14·3)	15·0 (14·9-15·1)	16·0 (15·9-16·1)	17·0 (16·8-17·1)
China	13·4 (13·3-13·6)	13·6 (13·5-13·8)	13·2 (13·0-13·3)	13·5 (13·4-13·6)	13·3 (13·2-13·4)	13·7 (13·5-13·8)	14·1 (14·0-14·3)	15·0 (14·9-15·1)	16·0 (15·8-16·1)	17·0 (16·8-17·1)
North Korea	14·7 (13·8-15·4)	15·2 (14·4-16·0)	15·3 (14·5-16·1)	15·4 (14·6-16·2)	15·5 (14·7-16·4)	13·8 (12·6-15·3)	13·5 (12·4-14·8)	14·9 (14·4-15·5)	14·9 (14·5-15·4)	15·0 (14·6-15·5)
Taiwan (Province of China)	13·1 (12·8-13·3)	13·9 (13·7-14·2)	14·2 (14·0-14·5)	14·8 (14·5-15·0)	15·5 (15·3-15·8)	16·1 (15·9-16·4)	17·1 (16·8-17·4)	18·1 (17·8-18·3)	19·1 (18·8-19·3)	19·4 (18·6-20·4)
Southeast Asia	12·5 (12·3-12·8)	12·9 (12·7-13·1)	13·4 (13·2-13·6)	13·8 (13·6-14·0)	14·1 (13·9-14·3)	14·3 (14·1-14·5)	14·5 (14·3-14·7)	14·9 (14·7-15·1)	15·4 (15·3-15·6)	16·1 (15·9-16·3)
Cambodia	11·4 (10·8-12·0)	9·5 (8·0-11·3)	11·5 (11·0-12·0)	11·5 (11·1-12·0)	11·8 (11·5-12·3)	12·1 (11·8-12·4)	12·3 (12·0-12·6)	13·0 (12·6-13·3)	13·6 (13·3-14·0)	14·2 (13·9-14·6)
Indonesia	11·9 (11·7-12·2)	12·4 (12·1-12·6)	13·0 (12·7-13·2)	13·5 (13·2-13·7)	13·6 (13·4-13·8)	13·8 (13·6-14·0)	14·1 (13·9-14·2)	14·4 (14·2-14·6)	14·7 (14·5-14·9)	15·2 (14·9-15·6)
Laos	10·6 (10·3-10·9)	10·9 (10·5-11·1)	11·1 (10·8-11·3)	11·5 (11·2-11·7)	11·7 (11·5-12·0)	12·0 (11·8-12·3)	12·5 (12·3-12·8)	13·1 (12·8-13·4)	13·7 (13·5-14·0)	14·5 (14·2-14·9)
Malaysia	12·3 (12·2-12·5)	13·1 (12·9-13·2)	13·5 (13·3-13·7)	14·0 (13·8-14·2)	14·2 (14·1-14·4)	14·4 (14·3-14·5)	14·6 (14·5-14·8)	15·0 (14·9-15·1)	15·4 (15·2-15·6)	16·1 (15·8-16·4)
Maldives	12·5 (11·0-14·0)	12·2 (10·7-13·8)	12·2 (11·1-13·6)	12·7 (12·2-13·3)	13·4 (13·0-13·9)	13·9 (13·2-14·5)	14·8 (14·3-15·3)	16·2 (15·7-16·8)	17·2 (16·6-17·7)	18·2 (16·9-19·5)
Mauritius	10·5 (10·3-10·7)	10·7 (10·5-10·9)	11·8 (11·6-12·1)	12·4 (12·2-12·6)	13·2 (13·0-13·4)	13·8 (13·6-14·0)	14·5 (14·3-14·8)	15·1 (14·8-15·3)	16·1 (15·8-16·3)	16·5 (15·7-17·3)
Myanmar	10·5 (10·0-11·1)	11·0 (10·5-11·6)	11·3 (10·8-11·9)	11·7 (11·2-12·3)	11·9 (11·4-12·5)	12·1 (11·5-12·7)	12·5 (11·8-13·0)	13·2 (12·3-13·7)	13·9 (13·0-14·5)	14·7 (14·2-15·1)
Philippines	15·3 (15·0-15·7)	14·9 (14·6-15·2)	14·9 (14·6-15·2)	14·7 (14·4-15·0)	14·7 (14·4-15·0)	14·3 (14·0-14·6)	13·9 (13·6-14·2)	13·7 (13·4-14·0)	14·0 (13·7-14·3)	14·4 (13·7-15·1)
Sri Lanka	13·0 (12·7-13·3)	12·9 (12·6-13·3)	14·5 (14·2-14·8)	15·1 (14·8-15·4)	16·1 (15·8-16·5)	16·5 (16·1-17·0)	16·3 (15·9-16·8)	16·7 (16·2-17·2)	17·0 (16·4-17·6)	18·1 (16·8-19·6)
Seychelles	14·2 (13·4-14·8)	14·6 (14·0-15·0)	14·8 (14·3-15·3)	14·2 (13·8-14·6)	14·4 (14·1-14·7)	14·8 (14·4-15·2)	15·1 (14·8-15·4)	15·5 (15·2-15·8)	16·0 (15·6-16·4)	16·2 (15·5-16·8)
Thailand	15·2 (14·9-15·4)	15·8 (15·5-16·0)	15·6 (15·4-15·9)	15·5 (15·3-15·8)	15·9 (15·7-16·2)	16·5 (16·2-16·8)	17·1 (16·8-17·4)	17·6 (17·3-17·9)	18·8 (18·5-19·1)	19·4 (18·8-20·0)
Timor-Leste	11·1 (10·1-12·5)	11·5 (10·5-12·7)	12·4 (11·2-13·6)	13·0 (11·9-14·2)	13·4 (12·5-14·5)	13·9 (13·1-14·7)	14·3 (13·6-15·1)	14·7 (14·0-15·7)	16·0 (15·1-17·0)	16·3 (15·2-17·6)
Vietnam	12·5 (11·6-13·4)	12·9 (12·1-13·9)	13·4 (12·6-14·3)	14·1 (13·3-14·9)	14·5 (13·7-15·4)	14·7 (13·9-15·7)	14·9 (14·3-15·7)	15·2 (14·7-16·1)	15·9 (15·3-16·6)	16·5 (16·0-16·9)
Oceania	10·8 (10·2-11·6)	10·8 (10·3-11·5)	10·8 (10·3-11·5)	10·9 (10·4-11·5)	10·9 (10·5-11·5)	11·1 (10·6-11·7)	11·2 (10·6-11·8)	11·2 (10·6-11·8)	11·3 (10·7-12·0)	11·8 (11·1-12·4)
American Samoa	13·2 (12·7-13·7)	13·8 (13·3-14·4)	14·1 (13·5-14·6)	14·3 (13·7-14·8)	14·5 (14·1-15·0)	14·6 (14·1-15·2)	15·0 (14·4-15·5)	15·1 (14·5-15·6)	15·4 (14·8-16·0)	15·3 (14·4-16·3)
Federated States of Micronesia	11·6 (10·6-12·9)	11·5 (10·6-12·7)	11·6 (10·8-12·7)	11·7 (11·0-12·7)	11·9 (11·2-12·8)	12·2 (11·4-13·1)	12·5 (11·6-13·3)	12·5 (11·5-13·5)	12·4 (11·2-13·5)	12·4 (11·2-13·6)
Fiji	12·4 (11·5-13·4)	12·2 (11·5-13·1)	12·4 (11·7-13·1)	12·7 (12·1-13·2)	12·5 (11·4-13·6)	12·2 (11·4-13·1)	12·1 (11·5-12·7)	12·2 (11·6-12·8)	12·3 (11·4-13·1)	12·6 (11·4-14·1)
Guam	13·5 (13·1-13·9)	13·6 (13·2-14·1)	14·7 (14·2-15·2)	15·6 (15·1-16·1)	15·7 (15·3-16·1)	15·8 (15·3-16·3)	16·0 (15·6-16·4)	15·9 (15·5-16·3)	15·3 (14·9-15·8)	15·4 (14·8-16·2)
Kiribati	11·0 (10·3-12·0)	11·4 (10·7-12·4)	11·4 (10·7-12·2)	11·0 (10·5-11·5)	10·9 (10·5-11·2)	10·9 (10·7-11·2)	11·2 (10·9-11·5)	11·5 (11·0-11·9)	11·6 (11·1-12·2)	11·8 (11·2-12·5)
Marshall Islands	11·8 (10·9-13·1)	12·2 (11·4-13·3)	12·5 (11·8-13·5)	12·6 (12·1-13·3)	12·5 (12·2-12·8)	12·3 (11·9-12·7)	12·0 (11·5-12·4)	11·7 (11·2-12·3)	11·9 (11·2-12·6)	12·3 (11·5-13·2)
Northern Mariana Islands	13·7 (12·7-14·8)	14·3 (13·2-15·4)	14·8 (13·7-16·0)	15·1 (14·0-16·2)	15·7 (14·6-16·8)	16·5 (15·5-17·5)	16·9 (16·0-17·8)	16·9 (16·1-17·8)	16·8 (15·9-17·7)	16·6 (15·7-17·6)
Papua New Guinea	10·2 (9·5-11·5)	10·3 (9·5-11·4)	10·2 (9·5-11·1)	10·1 (9·5-10·9)	10·2 (9·5-11·0)	10·3 (9·7-11·2)	10·5 (9·8-11·3)	10·4 (9·7-11·3)	10·7 (9·9-11·6)	11·2 (10·4-12·1)
Samoa	13·2 (12·7-13·8)	12·9 (12·5-13·4)	13·4 (12·8-13·9)	13·6 (12·9-14·3)	13·9 (13·2-14·7)	14·2 (13·5-14·9)	14·5 (13·8-15·3)	14·8 (14·1-15·6)	14·9 (14·2-15·7)	15·1 (14·4-15·9)
Solomon Islands	10·1 (9·3-11·2)	9·9 (9·3-10·8)	10·5 (9·8-11·1)	10·6 (10·0-11·4)	10·7 (10·1-11·6)	11·0 (10·3-11·7)	11·1 (10·4-11·8)	10·9 (10·2-11·7)	11·1 (10·2-11·9)	11·5 (10·5-12·4)
Tonga	12·7 (11·9-13·6)	12·8 (12·0-13·6)	12·7 (12·0-13·6)	13·0 (12·2-13·8)	13·4 (12·7-14·3)	13·7 (12·9-14·5)	13·9 (13·2-14·5)	14·2 (13·5-14·9)	14·4 (13·7-15·2)	14·6 (13·8-15·5)
Vanuatu	10·5 (9·7-11·7)	10·6 (9·9-11·6)	10·9 (10·2-11·6)	11·1 (10·5-11·8)	11·3 (10·6-11·9)	11·4 (10·7-12·0)	11·4 (10·8-12·1)	11·4 (10·7-12·3)	11·7 (10·7-12·5)	12·0 (11·0-12·9)
North Africa and Middle East	13·9 (13·7-14·2)	14·2 (14·0-14·4)	14·7 (14·5-14·8)	14·9 (14·7-15·1)	15·3 (15·1-15·4)	15·2 (15·0-15·3)	15·6 (15·5-15·8)	15·9 (15·6-16·1)	16·3 (16·0-16·5)	16·7 (16·4-17·0)
North Africa and Middle East	13·9 (13·7-14·2)	14·2 (14·0-14·4)	14·7 (14·5-14·8)	14·9 (14·7-15·1)	15·3 (15·1-15·4)	15·2 (15·0-15·3)	15·6 (15·5-15·8)	15·9 (15·6-16·1)	16·3 (16·0-16·5)	16·7 (16·4-17·0)
Afghanistan	10·4 (9·9-10·8)	10·2 (9·8-10·6)	10·0 (9·6-10·4)	10·0 (9·5-10·5)	10·3 (9·9-10·8)	10·0 (9·6-10·5)	9·8 (9·4-10·3)	10·0 (9·6-10·4)	10·3 (10·0-10·8)	10·8 (10·4-11·2)
Algeria	14·1 (13·7-14·6)	13·9 (13·4-14·4)	14·2 (13·8-14·6)	15·6 (15·2-15·9)	16·1 (15·7-16·6)	16·5 (16·1-17·0)	17·0 (16·4-17·5)	17·6 (17·0-18·0)	18·1 (17·8-18·3)	18·2 (17·8-18·7)
Bahrain	13·8 (12·9-14·8)	13·6 (12·6-14·6)	13·5 (12·6-14·4)	13·0 (12·4-13·7)	12·3 (11·6-13·0)	12·2 (11·5-13·0)	13·2 (12·5-13·9)	14·0 (13·3-14·8)	15·3 (14·5-16·1)	16·0 (14·9-17·2)

Appendix Table 14A. Estimates of life expectancy at age 65 by location, both sexes, 1970, 1975, 1980, 1985, 1990, 1995, 2000, 2005, 2010, 2016										
**Location**	**1970**	**1975**	**1980**	**1985**	**1990**	**1995**	**2000**	**2005**	**2010**	**2016**
Egypt	13·0 (12·8-13·3)	13·5 (13·3-13·8)	14·1 (13·8-14·4)	13·2 (12·9-13·5)	14·3 (14·0-14·6)	14·6 (14·3-15·0)	15·3 (15·0-15·6)	15·0 (14·7-15·4)	14·8 (14·4-15·1)	15·4 (14·6-16·2)
Iran	16·1 (15·2-17·2)	16·6 (15·8-17·5)	16·7 (16·0-17·7)	16·9 (16·0-17·8)	15·8 (15·1-16·7)	15·9 (15·3-16·5)	15·8 (15·2-16·4)	15·6 (14·6-16·5)	16·4 (15·3-17·4)	17·1 (16·1-18·2)
Iraq	14·1 (13·7-14·4)	13·8 (13·2-14·4)	13·7 (13·0-14·6)	13·4 (12·7-14·4)	13·6 (12·8-14·3)	13·2 (12·5-13·8)	13·1 (12·5-13·8)	13·2 (12·5-13·9)	13·6 (12·7-14·4)	14·1 (13·2-15·4)
Jordan	13·8 (13·0-14·4)	14·2 (13·5-14·8)	14·2 (13·2-15·1)	14·6 (13·9-15·4)	15·7 (15·0-16·4)	15·9 (15·1-16·8)	15·9 (14·9-17·0)	15·4 (14·3-16·6)	17·1 (15·8-18·2)	17·7 (16·3-19·1)
Kuwait	15·0 (14·2-15·7)	14·9 (14·3-15·5)	15·2 (14·5-15·8)	15·5 (14·8-16·2)	16·7 (15·8-17·7)	16·5 (15·8-17·3)	16·8 (16·1-17·6)	17·3 (16·5-18·1)	17·7 (16·9-18·5)	18·6 (17·1-20·5)
Lebanon	14·4 (13·1-15·6)	14·2 (12·9-15·5)	13·6 (12·4-15·0)	14·3 (13·4-15·6)	14·8 (14·0-16·1)	16·2 (15·7-17·0)	17·6 (16·7-18·3)	18·3 (17·7-19·0)	19·2 (18·5-19·8)	19·3 (18·5-20·2)
Libya	16·4 (15·8-17·0)	16·6 (16·0-17·2)	16·8 (16·2-17·4)	16·7 (16·2-17·3)	16·7 (16·3-17·2)	16·9 (16·4-17·4)	16·9 (16·3-17·4)	17·1 (16·4-17·6)	17·2 (16·5-17·9)	16·8 (16·2-17·6)
Morocco	13·5 (13·1-13·8)	13·7 (13·4-14·0)	14·0 (13·7-14·3)	14·6 (14·3-14·9)	14·9 (14·5-15·3)	15·2 (14·8-15·6)	15·8 (15·3-16·2)	16·2 (15·7-16·6)	16·6 (16·1-17·1)	17·0 (16·5-17·6)
Palestine	14·2 (13·0-15·2)	14·6 (13·6-15·6)	15·1 (14·0-16·1)	14·8 (13·8-15·7)	14·7 (13·9-15·5)	14·3 (13·8-14·9)	13·6 (13·4-13·8)	13·7 (13·6-13·9)	13·6 (13·5-13·7)	13·3 (13·0-13·6)
Oman	14·2 (13·8-14·6)	14·6 (14·2-15·1)	15·0 (14·7-15·5)	15·5 (15·2-16·0)	15·8 (15·4-16·3)	16·0 (15·5-16·7)	16·2 (15·8-16·7)	16·7 (16·2-17·1)	16·4 (15·9-16·9)	17·3 (17·0-17·6)
Qatar	15·0 (13·4-16·8)	15·3 (13·5-17·0)	14·9 (13·7-16·5)	16·0 (14·7-16·9)	16·8 (15·9-17·9)	16·6 (15·6-17·5)	16·6 (15·5-17·6)	17·3 (16·1-18·5)	18·8 (17·7-19·9)	19·2 (17·3-21·1)
Saudi Arabia	17·0 (16·3-17·8)	17·4 (16·7-18·1)	17·4 (16·7-18·0)	16·7 (16·1-17·2)	15·9 (15·4-16·4)	15·2 (14·8-15·7)	14·3 (14·0-14·6)	14·5 (14·3-14·8)	15·1 (14·7-15·4)	16·5 (16·0-17·0)
Sudan	12·6 (12·3-12·8)	12·6 (12·3-12·8)	12·8 (12·5-13·1)	12·3 (11·8-12·8)	13·1 (12·8-13·5)	13·2 (12·9-13·5)	13·5 (13·2-13·9)	14·0 (13·7-14·4)	14·7 (14·4-15·1)	15·3 (14·9-15·7)
Syria	14·9 (14·6-15·3)	15·2 (14·9-15·5)	15·4 (15·2-15·7)	15·6 (15·3-15·9)	15·7 (15·3-16·0)	15·6 (14·9-16·1)	15·6 (15·3-16·1)	16·3 (16·0-16·7)	17·0 (16·7-17·4)	16·5 (16·0-17·2)
Tunisia	13·4 (13·0-13·8)	13·9 (13·5-14·2)	13·9 (13·4-14·4)	15·5 (15·0-16·0)	15·8 (15·3-16·3)	15·7 (15·2-16·2)	17·0 (16·5-17·5)	17·4 (16·9-18·1)	17·8 (16·8-18·7)	17·8 (16·8-18·9)
Turkey	14·8 (13·8-16·1)	15·4 (14·6-16·3)	16·2 (15·6-16·9)	17·1 (16·5-17·7)	17·3 (17·0-17·7)	16·7 (16·4-16·9)	17·6 (17·3-17·9)	18·5 (18·2-19·0)	19·0 (18·5-19·6)	19·4 (18·2-20·4)
United Arab Emirates	15·8 (14·5-17·1)	15·9 (14·6-17·2)	16·0 (14·6-17·4)	15·9 (14·7-17·2)	15·6 (14·3-16·7)	15·7 (14·8-16·6)	16·2 (15·4-17·0)	17·0 (16·6-17·4)	16·8 (16·0-17·5)	16·7 (15·6-17·5)
Yemen	11·7 (11·1-12·2)	12·2 (11·6-12·8)	12·6 (12·0-13·2)	12·8 (12·1-13·4)	12·8 (12·2-13·5)	12·9 (12·3-13·6)	13·1 (12·7-13·7)	13·5 (13·1-14·1)	14·2 (13·7-14·7)	14·3 (13·6-14·9)
South Asia	10·9 (10·8-11·1)	11·3 (11·1-11·4)	11·7 (11·6-11·9)	12·1 (11·9-12·2)	12·4 (12·3-12·6)	12·8 (12·6-12·9)	13·1 (13·0-13·3)	13·6 (13·5-13·8)	14·0 (13·8-14·1)	14·5 (14·3-14·7)
South Asia	10·9 (10·8-11·1)	11·3 (11·1-11·4)	11·7 (11·6-11·9)	12·1 (11·9-12·2)	12·4 (12·3-12·6)	12·8 (12·6-12·9)	13·1 (13·0-13·3)	13·6 (13·5-13·8)	14·0 (13·8-14·1)	14·5 (14·3-14·7)
Bangladesh	11·8 (11·0-12·8)	12·8 (12·0-13·5)	12·8 (12·2-13·4)	12·6 (12·2-13·1)	12·8 (12·4-13·3)	13·6 (13·2-14·1)	14·6 (14·2-15·1)	15·2 (14·9-15·5)	15·8 (15·4-16·1)	16·4 (15·7-16·9)
Bhutan	12·7 (11·8-13·5)	12·9 (12·0-13·7)	13·0 (12·1-13·9)	13·3 (12·4-14·1)	13·7 (12·9-14·5)	14·3 (13·6-14·9)	15·3 (14·7-15·9)	16·3 (15·6-16·9)	16·9 (16·2-17·5)	17·0 (16·2-17·8)
India	10·5 (10·4-10·7)	10·8 (10·7-11·0)	11·4 (11·2-11·5)	11·8 (11·6-11·9)	12·2 (12·0-12·4)	12·6 (12·4-12·8)	13·0 (12·8-13·1)	13·5 (13·3-13·6)	13·8 (13·7-14·0)	14·3 (14·1-14·5)
Nepal	12·3 (11·8-12·7)	12·3 (11·9-12·7)	12·3 (11·9-12·7)	12·4 (12·0-12·8)	12·5 (12·2-12·9)	12·8 (12·5-13·1)	13·5 (13·1-13·8)	13·9 (13·6-14·3)	14·3 (13·9-14·7)	15·0 (14·5-15·4)
Pakistan	13·8 (13·1-14·6)	14·1 (13·4-14·7)	14·3 (13·8-14·8)	14·4 (14·1-14·8)	14·2 (13·8-14·6)	13·3 (12·9-13·7)	13·3 (12·9-13·6)	13·3 (12·9-13·8)	13·9 (13·3-14·5)	14·4 (13·6-15·2)
Sub-Saharan Africa	12·2 (12·0-12·4)	12·4 (12·3-12·6)	12·6 (12·4-12·7)	12·7 (12·5-12·8)	12·7 (12·5-12·8)	12·6 (12·4-12·8)	12·4 (12·2-12·6)	12·8 (12·6-13·0)	13·4 (13·2-13·5)	14·1 (13·9-14·3)
Southern Sub-Saharan Africa	14·5 (14·0-14·9)	15·0 (14·6-15·4)	15·2 (14·9-15·5)	15·7 (15·4-15·9)	16·2 (15·9-16·7)	15·7 (15·3-16·2)	13·3 (12·9-13·7)	13·0 (12·7-13·3)	13·5 (13·2-13·8)	15·3 (15·0-15·6)
Botswana	10·7 (10·3-11·3)	11·4 (11·0-12·1)	12·0 (11·5-12·7)	12·8 (12·3-13·4)	13·4 (12·4-15·0)	13·1 (10·1-18·2)	11·3 (8·0-14·8)	11·6 (8·8-14·2)	13·5 (10·9-16·2)	14·4 (12·3-18·5)
Lesotho	11·1 (10·1-12·2)	11·4 (10·3-12·6)	11·9 (10·8-13·1)	12·2 (11·3-13·3)	12·3 (11·7-13·2)	12·1 (11·4-13·6)	10·7 (9·8-12·4)	9·8 (9·0-10·9)	10·3 (9·4-11·4)	10·9 (9·7-12·6)
Namibia	11·7 (11·1-12·4)	11·9 (11·3-12·6)	12·1 (11·5-12·6)	12·2 (11·8-12·7)	12·3 (12·0-12·8)	12·2 (11·6-13·4)	11·5 (10·3-13·7)	12·0 (10·6-13·8)	13·9 (12·5-15·8)	14·9 (13·4-17·3)
South Africa	15·6 (15·1-16·2)	16·1 (15·6-16·6)	16·2 (15·8-16·6)	16·6 (16·3-16·9)	17·1 (16·8-17·3)	16·6 (16·4-16·8)	14·0 (13·9-14·2)	13·9 (13·7-14·0)	14·2 (14·0-14·3)	16·2 (15·9-16·5)
Swaziland	11·5 (10·3-12·6)	11·9 (10·7-13·1)	12·1 (11·0-13·3)	12·2 (11·1-13·5)	12·4 (11·6-13·5)	13·0 (11·4-15·1)	11·3 (9·2-13·5)	10·2 (8·4-11·3)	11·2 (9·6-12·8)	12·9 (11·1-15·7)
Zimbabwe	11·5 (10·8-12·4)	12·0 (11·3-12·6)	12·3 (11·7-12·8)	13·1 (12·8-13·5)	14·1 (13·1-17·1)	13·8 (11·8-16·5)	11·3 (9·8-13·1)	10·5 (9·5-11·7)	11·1 (10·1-12·8)	12·4 (11·3-13·9)
Western Sub-Saharan Africa	12·9 (12·6-13·2)	13·1 (12·8-13·4)	13·3 (13·0-13·6)	13·4 (13·2-13·7)	13·3 (13·0-13·6)	13·3 (12·9-13·7)	13·3 (13·0-13·8)	13·7 (13·4-14·2)	14·3 (13·9-14·7)	14·8 (14·4-15·2)
Benin	12·5 (12·2-12·9)	12·6 (12·3-13·0)	12·7 (12·3-13·1)	13·0 (12·7-13·4)	13·3 (13·0-13·7)	13·3 (12·9-13·8)	13·3 (12·9-13·8)	13·5 (13·0-14·0)	13·6 (13·2-14·2)	13·9 (13·3-14·5)
Burkina Faso	12·0 (11·8-12·2)	12·1 (11·9-12·3)	12·2 (12·0-12·4)	12·4 (12·2-12·7)	12·3 (11·9-13·1)	12·4 (11·8-13·3)	12·5 (12·0-13·1)	12·9 (12·5-13·2)	13·0 (12·7-13·4)	13·2 (12·9-13·6)
Cameroon	12·0 (11·4-12·7)	12·2 (11·6-12·9)	12·6 (12·0-13·2)	13·1 (12·5-13·6)	13·3 (12·8-14·0)	13·0 (12·3-13·9)	12·4 (11·7-13·4)	12·6 (11·8-13·5)	13·1 (12·1-14·0)	13·5 (12·4-14·7)
Cape Verde	14·4 (13·5-15·6)	14·8 (14·0-15·6)	14·7 (14·1-15·3)	15·1 (14·7-15·5)	15·3 (15·0-15·7)	15·4 (14·8-16·1)	15·8 (14·8-16·9)	15·9 (15·1-17·0)	16·1 (15·7-16·7)	16·5 (15·9-17·3)
Chad	13·1 (12·7-13·4)	13·2 (12·8-13·5)	13·0 (12·6-13·4)	13·0 (12·7-13·4)	13·0 (12·6-13·3)	12·8 (12·4-13·3)	12·6 (12·1-13·2)	13·0 (12·4-13·6)	13·2 (12·6-13·8)	13·8 (13·1-14·4)
Cote d'Ivoire	12·4 (11·9-12·8)	12·5 (12·1-12·9)	12·8 (12·3-13·2)	12·7 (12·3-13·1)	12·2 (11·8-12·6)	11·7 (11·1-12·3)	11·7 (11·0-12·4)	11·9 (11·2-12·6)	12·3 (11·7-12·9)	12·8 (12·1-13·3)
The Gambia	13·2 (12·4-13·9)	13·4 (12·6-14·1)	13·5 (12·9-14·2)	13·7 (13·0-14·3)	13·7 (13·1-14·4)	13·8 (13·2-14·4)	13·8 (13·2-14·4)	14·1 (13·5-14·7)	14·3 (13·8-15·0)	14·7 (14·0-15·5)
Ghana	12·8 (12·1-13·5)	13·0 (12·3-13·7)	13·0 (12·3-13·7)	12·9 (12·2-13·7)	12·9 (12·3-13·6)	13·1 (12·5-14·0)	13·0 (12·4-14·0)	13·2 (12·6-14·1)	13·4 (12·9-14·1)	14·1 (13·6-15·0)
Guinea	12·5 (11·8-13·3)	12·6 (11·8-13·3)	12·8 (12·1-13·4)	12·9 (12·2-13·5)	13·0 (12·5-13·5)	12·9 (12·4-13·4)	12·7 (12·2-13·2)	12·8 (12·2-13·4)	12·8 (12·2-13·6)	13·0 (12·4-13·9)
Guinea-Bissau	10·8 (10·2-11·5)	11·1 (10·5-11·8)	11·2 (10·6-11·8)	11·0 (10·5-11·8)	11·0 (10·5-11·6)	11·1 (10·5-11·6)	11·1 (10·5-11·8)	11·3 (10·6-12·2)	11·4 (10·8-12·1)	11·8 (11·2-12·4)
Liberia	13·2 (12·8-13·7)	13·4 (13·0-13·9)	13·5 (13·0-13·9)	13·4 (12·9-13·8)	13·1 (12·6-13·5)	12·8 (12·3-13·3)	12·9 (12·3-13·6)	13·1 (12·5-13·9)	13·1 (12·7-13·7)	13·6 (13·3-14·0)
Mali	11·0 (10·2-11·8)	11·0 (10·3-11·7)	11·4 (10·8-12·1)	11·7 (11·3-12·3)	12·0 (11·5-12·5)	12·3 (11·8-12·9)	13·0 (12·5-13·5)	13·7 (13·1-14·3)	14·2 (13·5-14·9)	14·5 (13·4-15·4)
Mauritania	12·1 (11·0-13·3)	12·3 (11·2-13·5)	12·5 (11·7-13·5)	12·6 (12·0-13·3)	12·7 (12·2-13·3)	13·0 (12·6-13·7)	13·8 (13·2-14·4)	14·5 (13·8-15·4)	15·1 (14·2-16·2)	15·5 (14·5-16·8)
Niger	12·4 (11·7-13·0)	12·2 (11·7-12·7)	12·1 (11·6-12·6)	12·2 (11·7-12·6)	12·9 (12·5-13·4)	13·1 (12·6-13·6)	13·4 (12·9-13·8)	13·6 (13·0-14·1)	13·8 (13·1-14·4)	13·8 (12·9-14·7)
Nigeria	13·6 (12·9-14·3)	14·1 (13·3-14·8)	14·4 (13·7-15·1)	14·4 (13·8-15·1)	14·1 (13·3-14·7)	14·0 (13·3-15·0)	14·2 (13·3-15·2)	14·8 (14·0-16·1)	15·8 (14·9-17·0)	16·6 (15·6-17·7)
Sao Tome and Principe	13·7 (13·3-14·0)	14·1 (13·7-14·4)	14·6 (14·2-14·9)	14·6 (14·3-15·0)	14·4 (13·8-14·9)	13·9 (13·2-14·7)	13·4 (13·1-13·8)	13·8 (13·5-14·2)	14·5 (14·0-15·0)	14·9 (14·1-15·8)
Senegal	12·3 (11·9-12·7)	12·3 (11·9-12·6)	12·5 (12·1-12·8)	12·9 (12·5-13·3)	13·0 (12·5-13·4)	13·0 (12·5-13·4)	13·0 (12·5-13·4)	13·1 (12·8-13·6)	13·3 (13·1-13·6)	13·8 (13·4-14·1)
Sierra Leone	12·5 (12·0-13·0)	12·7 (12·2-13·2)	12·8 (12·3-13·2)	12·9 (12·4-13·3)	13·0 (12·6-13·4)	12·8 (12·4-13·1)	12·4 (12·0-12·8)	12·2 (11·7-12·7)	12·4 (11·9-12·9)	12·9 (12·4-13·6)
Togo	12·8 (12·3-13·3)	13·0 (12·5-13·5)	13·1 (12·6-13·6)	13·0 (12·5-13·4)	12·9 (12·4-13·4)	12·8 (12·4-13·4)	12·7 (12·0-13·7)	12·8 (12·0-13·8)	13·0 (12·3-13·8)	13·4 (12·7-14·1)
Eastern Sub-Saharan Africa	11·0 (10·7-11·2)	11·1 (10·9-11·4)	11·3 (11·0-11·5)	11·3 (11·1-11·5)	11·3 (11·1-11·5)	11·3 (11·1-11·5)	11·5 (11·3-11·8)	12·2 (11·9-12·4)	12·9 (12·6-13·1)	13·4 (13·1-13·8)
Burundi	9·7 (9·2-10·2)	9·7 (9·2-10·2)	9·6 (9·1-10·2)	9·4 (8·8-10·0)	9·5 (8·8-10·1)	9·4 (8·8-10·0)	9·8 (9·3-10·4)	11·0 (10·4-11·6)	11·9 (11·2-12·5)	12·2 (11·4-12·9)
Comoros	11·6 (10·9-12·4)	11·9 (11·2-12·7)	12·0 (11·3-12·9)	12·2 (11·5-13·0)	12·4 (11·9-13·2)	12·5 (12·1-13·1)	12·9 (12·5-13·4)	13·7 (13·2-14·3)	14·0 (13·5-14·8)	14·3 (13·7-15·1)

Appendix Table 14A. Estimates of life expectancy at age 65 by location, both sexes, 1970, 1975, 1980, 1985, 1990, 1995, 2000, 2005, 2010, 2016

Location	1970	1975	1980	1985	1990	1995	2000	2005	2010	2016
Djibouti	13·9 (13·5-14·3)	14·1 (13·6-14·5)	14·0 (13·5-14·5)	13·9 (13·4-14·3)	13·9 (13·6-14·3)	13·8 (13·4-14·3)	13·9 (13·0-14·8)	13·9 (12·8-15·0)	14·1 (13·1-15·3)	14·6 (13·6-16·2)
Eritrea	9·5 (9·1-9·9)	9·6 (9·2-10·1)	9·8 (9·4-10·2)	9·9 (9·6-10·3)	10·4 (10·0-10·8)	11·4 (10·9-11·9)	11·9 (11·4-12·5)	12·2 (11·6-12·8)	12·3 (11·7-12·9)	12·6 (11·9-13·3)
Ethiopia	9·2 (8·6-9·9)	9·1 (8·6-9·8)	9·2 (8·7-9·8)	9·3 (8·9-9·7)	9·7 (9·3-10·1)	10·2 (9·9-10·6)	10·8 (10·4-11·3)	11·7 (11·2-12·2)	12·6 (11·9-13·2)	13·3 (12·4-14·1)
Kenya	12·9 (12·7-13·2)	13·3 (13·0-13·5)	13·6 (13·4-13·9)	13·7 (13·5-14·0)	13·6 (13·3-13·8)	13·2 (12·8-13·5)	13·0 (12·7-13·3)	13·4 (13·2-13·7)	14·2 (13·9-14·5)	14·8 (14·5-15·1)
Madagascar	10·5 (10·0-10·9)	11·3 (10·8-11·8)	12·2 (11·7-12·8)	12·1 (11·6-12·5)	12·0 (11·7-12·4)	12·2 (11·8-12·6)	12·3 (11·9-12·7)	12·6 (12·1-13·2)	12·8 (12·1-13·8)	13·1 (12·0-14·3)
Malawi	12·3 (11·7-12·8)	12·6 (12·1-13·0)	12·9 (12·4-13·3)	12·9 (12·3-13·5)	12·6 (11·4-14·2)	11·6 (10·4-13·7)	11·3 (9·9-13·4)	11·9 (10·4-13·8)	12·8 (11·6-14·5)	13·5 (12·4-14·9)
Mozambique	11·9 (11·4-12·4)	12·2 (11·7-12·6)	12·3 (11·8-12·7)	11·6 (11·2-12·1)	12·3 (11·9-12·7)	12·5 (11·9-13·3)	12·7 (11·6-13·9)	13·3 (11·8-14·4)	13·6 (12·1-14·7)	14·0 (12·8-15·0)
Rwanda	10·8 (10·1-11·6)	11·0 (10·3-11·8)	11·2 (10·6-12·0)	11·2 (10·7-11·9)	10·4 (9·8-11·0)	9·5 (9·1-10·1)	10·9 (10·4-11·5)	13·3 (12·6-14·0)	14·3 (13·5-15·1)	14·7 (13·9-15·6)
Somalia	10·7 (10·0-11·4)	10·5 (9·9-11·2)	10·9 (10·2-11·5)	10·8 (10·1-11·4)	10·8 (10·1-11·4)	10·7 (10·0-11·4)	10·6 (10·0-11·3)	10·8 (10·1-11·5)	10·9 (10·1-11·6)	11·3 (10·4-12·0)
South Sudan	12·7 (11·8-13·4)	12·8 (11·9-13·6)	12·9 (12·0-13·7)	12·8 (11·9-13·7)	12·6 (11·6-13·7)	12·4 (11·3-13·7)	12·3 (11·3-13·9)	12·7 (11·6-14·2)	12·9 (11·8-14·5)	12·9 (11·8-14·3)
Tanzania	12·6 (12·1-13·1)	13·0 (12·4-13·5)	13·3 (12·7-13·8)	13·2 (12·7-13·8)	12·5 (12·0-13·3)	12·2 (11·5-13·3)	12·3 (11·6-13·6)	12·9 (12·1-14·0)	13·4 (12·7-14·5)	13·9 (13·1-15·0)
Uganda	11·1 (10·7-11·6)	11·3 (10·9-11·7)	11·2 (10·8-11·6)	11·3 (11·0-11·7)	11·1 (10·6-11·8)	11·0 (10·2-12·0)	11·2 (10·7-11·9)	11·8 (11·2-12·3)	12·6 (12·0-13·4)	13·2 (12·5-14·2)
Zambia	13·4 (11·7-15·1)	13·7 (12·2-15·1)	13·8 (12·6-14·9)	13·8 (12·6-15·2)	12·7 (11·8-13·9)	10·7 (10·0-11·5)	9·8 (9·1-10·8)	9·8 (9·2-11·1)	10·9 (10·0-11·9)	12·1 (10·9-13·6)
Central Sub-Saharan Africa	11·3 (10·9-11·6)	11·4 (11·1-11·8)	11·5 (11·1-11·8)	11·5 (11·2-11·9)	11·5 (11·2-11·9)	11·4 (11·1-11·8)	11·5 (11·1-11·8)	11·8 (11·4-12·1)	12·1 (11·8-12·5)	12·8 (12·5-13·2)
Angola	11·2 (10·1-12·6)	11·4 (10·2-12·7)	11·3 (10·1-12·6)	11·1 (10·0-12·5)	10·9 (9·8-12·3)	10·9 (9·7-12·3)	11·3 (10·1-12·6)	11·8 (10·6-13·3)	12·6 (11·2-14·2)	13·5 (11·8-14·9)
Central African Republic	9·8 (9·1-10·7)	9·9 (9·3-10·7)	10·1 (9·6-10·8)	10·2 (9·8-10·8)	9·8 (9·3-10·3)	9·5 (8·9-10·3)	9·4 (8·6-10·4)	9·5 (8·7-10·6)	9·9 (9·0-11·0)	10·1 (9·2-11·3)
Congo	9·6 (8·8-10·5)	9·8 (9·0-10·8)	10·0 (9·2-10·9)	10·2 (9·6-11·1)	10·1 (9·6-10·8)	10·1 (9·6-10·7)	10·6 (10·0-11·3)	11·7 (11·1-12·4)	12·4 (11·7-13·2)	13·0 (12·0-14·2)
Democratic Republic of the Congo	11·7 (11·4-12·2)	11·9 (11·5-12·3)	11·8 (11·4-12·3)	12·0 (11·6-12·4)	12·1 (11·7-12·5)	11·9 (11·6-12·3)	11·8 (11·5-12·2)	12·0 (11·6-12·3)	12·1 (11·8-12·5)	12·8 (12·5-13·2)
Equatorial Guinea	9·4 (8·6-10·6)	9·6 (8·8-10·7)	9·9 (9·0-11·2)	10·1 (9·2-11·4)	10·0 (9·1-11·2)	10·3 (9·3-11·5)	12·2 (10·8-14·2)	13·9 (11·8-16·5)	14·7 (12·4-17·3)	15·5 (13·2-18·7)
Gabon	11·1 (10·1-12·3)	11·5 (10·5-12·9)	11·9 (11·0-12·9)	11·9 (11·2-12·9)	11·7 (11·2-12·4)	11·9 (11·3-12·5)	12·0 (11·2-12·7)	12·4 (11·7-13·1)	13·4 (12·6-14·1)	14·3 (13·2-15·3)

Appendix Table 14B. Estimates of life expectancy at age 65 by location, males, 1970, 1975, 1980, 1985, 1990, 1995, 2000, 2005, 2010, 2016

Location	1970	1975	1980	1985	1990	1995	2000	2005	2010	2016
Global	12·5 (12·4-12·5)	12·7 (12·7-12·8)	12·9 (12·8-13·0)	13·1 (13·1-13·2)	13·3 (13·3-13·4)	13·5 (13·4-13·6)	13·9 (13·8-14·0)	14·5 (14·4-14·5)	15·1 (15·1-15·2)	15·7 (15·6-15·8)
High SDI	12·7 (12·6-12·8)	13·1 (13·0-13·2)	13·6 (13·6-13·7)	14·1 (14·1-14·2)	14·8 (14·7-14·8)	15·3 (15·3-15·4)	16·1 (16·0-16·1)	17·0 (17·0-17·1)	17·9 (17·8-17·9)	18·4 (18·2-18·5)
High-middle SDI	12·9 (12·7-13·1)	13·1 (12·9-13·2)	12·9 (12·8-13·1)	13·2 (13·0-13·4)	13·4 (13·2-13·6)	13·0 (12·8-13·2)	13·5 (13·3-13·7)	14·0 (13·8-14·2)	15·1 (14·9-15·3)	15·9 (15·4-16·3)
Middle SDI	12·9 (12·8-13·0)	13·0 (12·9-13·2)	12·8 (12·7-12·9)	12·9 (12·8-13·0)	12·7 (12·6-12·9)	13·0 (12·9-13·1)	13·3 (13·2-13·5)	13·9 (13·8-14·0)	14·5 (14·4-14·6)	15·1 (14·9-15·2)
Low-middle SDI	11·3 (11·1-11·5)	11·6 (11·4-11·7)	11·9 (11·8-12·1)	12·2 (12·0-12·3)	12·3 (12·2-12·5)	12·5 (12·3-12·6)	12·7 (12·5-12·8)	13·1 (12·9-13·2)	13·4 (13·3-13·6)	13·9 (13·7-14·1)
Low SDI	11·2 (11·0-11·5)	11·4 (11·1-11·6)	11·5 (11·2-11·8)	11·6 (11·4-11·9)	11·7 (11·5-12·0)	11·9 (11·6-12·1)	12·0 (11·8-12·3)	12·4 (12·2-12·6)	12·7 (12·4-13·0)	13·3 (12·9-13·6)
High-income	12·8 (12·7-12·8)	13·2 (13·1-13·2)	13·8 (13·7-13·9)	14·3 (14·2-14·3)	15·0 (14·9-15·0)	15·5 (15·4-15·5)	16·2 (16·2-16·3)	17·1 (17·1-17·2)	18·0 (17·9-18·0)	18·5 (18·3-18·6)
High-income North America	13·1 (13·0-13·2)	13·6 (13·6-13·7)	14·3 (14·2-14·3)	14·6 (14·5-14·7)	15·2 (15·1-15·2)	15·5 (15·5-15·6)	16·2 (16·1-16·3)	17·1 (17·0-17·2)	18·0 (17·9-18·0)	18·2 (18·1-18·3)
Canada	13·8 (13·6-14·0)	14·0 (13·8-14·2)	14·6 (14·4-14·8)	15·0 (14·8-15·2)	15·5 (15·3-15·7)	15·9 (15·7-16·1)	16·7 (16·5-16·9)	17·7 (17·5-17·9)	18·7 (18·5-18·9)	19·1 (18·8-19·4)
Greenland	10·1 (9·7-10·6)	9·7 (9·3-10·1)	9·1 (8·7-9·5)	8·9 (8·6-9·3)	8·9 (8·5-9·4)	9·7 (9·3-10·1)	10·2 (9·8-10·6)	10·3 (9·9-10·7)	11·4 (10·8-11·9)	11·9 (10·6-13·3)
United States	13·0 (13·0-13·1)	13·6 (13·5-13·7)	14·2 (14·2-14·3)	14·6 (14·5-14·6)	15·1 (15·1-15·2)	15·5 (15·4-15·6)	16·1 (16·1-16·2)	17·0 (17·0-17·1)	17·9 (17·8-18·0)	18·1 (17·9-18·2)
Australasia	12·3 (12·2-12·4)	13·0 (12·8-13·1)	13·7 (13·5-13·8)	14·2 (14·1-14·4)	15·1 (15·0-15·3)	15·9 (15·7-16·0)	17·0 (16·9-17·2)	18·2 (18·0-18·3)	19·0 (18·8-19·1)	19·4 (18·9-19·8)
Australia	12·2 (12·1-12·4)	13·0 (12·8-13·1)	13·8 (13·6-13·9)	14·4 (14·2-14·5)	15·2 (15·0-15·4)	15·9 (15·8-16·1)	17·1 (16·9-17·3)	18·2 (18·1-18·4)	19·0 (18·9-19·2)	19·5 (18·9-20·0)
New Zealand	12·6 (12·4-12·8)	12·9 (12·7-13·1)	13·2 (13·0-13·4)	13·7 (13·5-13·9)	14·7 (14·5-14·9)	15·4 (15·2-15·6)	16·5 (16·3-16·7)	17·7 (17·5-17·9)	18·6 (18·4-18·8)	18·9 (18·1-19·8)
High-income Asia Pacific	12·4 (12·0-12·7)	13·2 (12·9-13·5)	14·1 (14·0-14·2)	15·0 (14·8-15·1)	15·7 (15·5-15·8)	16·2 (16·1-16·3)	17·0 (16·9-17·1)	17·8 (17·7-17·9)	18·4 (18·3-18·6)	19·2 (18·8-19·6)
Brunei	13·8 (13·2-14·8)	14·4 (13·4-15·1)	13·3 (12·7-15·5)	14·0 (13·7-14·3)	14·5 (14·3-14·8)	15·1 (14·7-15·5)	15·7 (15·3-16·1)	15·8 (15·5-16·2)	15·9 (15·6-16·3)	15·9 (15·1-17·4)
Japan	12·7 (12·5-12·9)	13·7 (13·6-13·9)	14·7 (14·7-14·7)	15·6 (15·6-15·6)	16·2 (16·2-16·3)	16·6 (16·6-16·7)	17·4 (17·4-17·4)	18·1 (18·1-18·1)	18·7 (18·7-18·7)	19·5 (19·4-19·7)
Singapore	10·7 (9·9-11·5)	11·3 (10·6-12·1)	12·2 (11·4-13·1)	13·2 (12·2-14·1)	14·6 (13·7-15·6)	15·5 (14·5-16·4)	16·9 (15·7-18·0)	17·8 (16·9-18·8)	19·0 (18·0-20·0)	19·7 (17·9-21·5)
South Korea	10·3 (8·6-12·8)	10·0 (8·8-11·4)	10·6 (9·9-11·4)	11·4 (10·7-12·1)	12·4 (11·7-13·1)	13·5 (12·8-14·4)	14·6 (13·9-15·3)	16·0 (15·2-16·8)	17·0 (16·3-17·9)	17·5 (15·5-20·1)
Western Europe	12·7 (12·6-12·8)	12·9 (12·8-13·0)	13·5 (13·4-13·6)	14·0 (13·9-14·1)	14·7 (14·6-14·8)	15·3 (15·2-15·4)	16·1 (16·0-16·2)	17·0 (16·9-17·1)	17·9 (17·8-18·0)	18·5 (18·3-18·7)
Andorra	15·0 (12·7-17·3)	15·3 (14·0-17·9)	16·0 (15·0-17·8)	16·3 (15·3-18·1)	16·5 (15·6-18·0)	17·2 (16·0-18·3)	18·0 (16·9-19·0)	18·3 (17·5-19·2)	18·5 (17·6-19·6)	18·5 (17·4-20·1)
Austria	11·8 (11·6-11·9)	12·3 (12·1-12·4)	12·9 (12·7-13·0)	13·6 (13·4-13·7)	14·4 (14·2-14·5)	15·0 (14·8-15·2)	15·9 (15·8-16·1)	17·0 (16·8-17·1)	17·7 (17·6-17·9)	18·4 (18·0-18·9)
Belgium	12·1 (11·9-12·4)	12·4 (12·1-12·6)	12·8 (12·6-13·1)	13·4 (13·1-13·6)	14·3 (14·0-14·6)	14·8 (14·6-15·1)	15·6 (15·3-15·9)	16·6 (16·3-16·9)	17·4 (17·1-17·7)	18·0 (17·2-18·7)
Cyprus	12·2 (11·9-12·7)	12·0 (11·6-12·4)	12·6 (12·3-12·9)	13·5 (13·2-13·8)	14·0 (13·7-14·4)	14·6 (14·2-14·9)	15·4 (15·1-15·7)	16·1 (15·8-16·5)	17·1 (16·8-17·5)	17·4 (17·0-17·9)
Denmark	13·7 (13·2-14·2)	13·8 (13·4-14·3)	13·8 (13·3-14·2)	14·0 (13·6-14·5)	14·3 (13·8-14·8)	14·3 (13·9-14·8)	15·2 (14·7-15·7)	16·1 (15·6-16·6)	17·1 (16·7-17·6)	18·0 (17·0-18·9)
Finland	11·4 (11·3-11·6)	12·0 (11·9-12·2)	12·7 (12·5-12·9)	13·2 (13·0-13·4)	13·8 (13·6-14·0)	14·6 (14·4-14·8)	15·5 (15·3-15·7)	16·6 (16·4-16·8)	17·3 (17·1-17·6)	18·4 (17·8-19·0)
France	13·1 (12·9-13·3)	13·4 (13·2-13·6)	14·1 (13·9-14·3)	14·8 (14·6-15·0)	15·7 (15·5-15·9)	16·2 (16·0-16·4)	16·7 (16·6-17·0)	17·7 (17·5-17·9)	18·6 (18·4-18·8)	19·2 (18·9-19·6)
Germany	12·0 (11·7-12·4)	12·2 (11·9-12·6)	12·8 (12·5-13·2)	13·3 (13·0-13·7)	14·0 (13·7-14·4)	14·7 (14·3-15·0)	15·6 (15·2-15·9)	16·6 (16·2-16·9)	17·3 (17·0-17·7)	17·9 (17·3-18·6)
Greece	14·9 (14·7-15·1)	15·3 (15·0-15·5)	17·4 (17·1-17·6)	15·5 (15·3-15·7)	15·6 (15·4-15·8)	16·0 (15·8-16·2)	16·5 (16·3-16·7)	17·2 (17·0-17·5)	17·9 (17·7-18·1)	18·3 (17·7-19·0)
Iceland	14·7 (14·4-15·0)	15·3 (15·0-15·5)	15·8 (15·4-16·1)	15·8 (15·5-16·1)	16·2 (15·8-16·5)	16·6 (16·2-16·9)	17·3 (16·9-17·6)	18·2 (17·9-18·6)	18·7 (18·4-19·0)	19·0 (18·4-19·6)
Ireland	12·4 (12·0-12·8)	12·4 (12·1-12·8)	12·5 (12·1-12·9)	12·8 (12·4-13·2)	13·4 (13·0-13·8)	13·8 (13·5-14·2)	14·6 (14·3-15·0)	16·4 (16·0-16·8)	17·6 (17·2-18·0)	18·1 (17·1-19·1)
Israel	13·0 (12·3-13·8)	13·6 (12·8-14·4)	14·1 (13·3-15·0)	14·4 (13·7-15·3)	15·1 (14·4-15·9)	15·6 (14·8-16·3)	16·7 (15·9-17·6)	17·4 (16·7-18·2)	18·6 (17·8-19·3)	18·9 (17·4-20·5)
Italy	13·2 (13·0-13·4)	13·2 (13·0-13·4)	13·6 (13·4-13·8)	14·3 (14·1-14·4)	15·1 (14·9-15·2)	15·7 (15·5-15·9)	16·5 (16·4-16·7)	17·4 (17·2-17·6)	18·1 (17·9-18·2)	18·6 (18·1-19·1)
Luxembourg	11·8 (11·5-12·0)	12·1 (11·9-12·4)	12·5 (12·3-12·8)	13·2 (12·9-13·4)	14·0 (13·7-14·3)	14·8 (14·6-15·1)	15·8 (15·5-16·1)	17·1 (16·8-17·4)	18·2 (17·8-18·5)	18·9 (18·2-19·7)
Malta	11·6 (11·1-12·2)	11·9 (11·3-12·5)	12·1 (11·5-12·7)	13·2 (12·6-13·9)	14·3 (13·6-15·0)	14·9 (14·2-15·6)	15·0 (14·4-15·7)	16·4 (15·8-17·1)	17·4 (16·7-18·0)	18·0 (16·8-19·4)
Netherlands	13·6 (13·3-13·9)	13·7 (13·3-14·0)	14·0 (13·6-14·3)	14·1 (13·7-14·4)	14·4 (14·1-14·7)	14·8 (14·5-15·1)	15·3 (15·0-15·7)	16·4 (16·1-16·7)	17·6 (17·2-17·9)	18·1 (17·4-18·8)
Norway	13·8 (13·5-14·1)	14·1 (13·8-14·4)	14·3 (14·0-14·6)	14·4 (14·1-14·7)	14·8 (14·5-15·1)	15·3 (15·0-15·6)	16·1 (15·7-16·4)	17·2 (16·9-17·5)	17·9 (17·6-18·2)	18·7 (17·9-19·5)
Portugal	12·2 (12·0-12·4)	12·8 (12·5-13·0)	13·3 (13·0-13·5)	13·8 (13·6-14·0)	14·3 (14·0-14·5)	14·7 (14·5-14·9)	15·3 (15·1-15·6)	16·3 (16·0-16·5)	17·2 (17·0-17·4)	17·8 (17·3-18·4)
Spain	13·3 (13·1-13·4)	13·7 (13·5-13·8)	14·6 (14·4-14·7)	15·0 (14·9-15·2)	15·6 (15·4-15·7)	16·1 (15·9-16·2)	16·6 (16·5-16·8)	17·3 (17·2-17·5)	18·3 (18·2-18·5)	19·2 (18·8-19·6)
Sweden	14·1 (13·7-14·5)	14·0 (13·7-14·4)	14·3 (14·0-14·5)	14·8 (14·5-15·0)	15·3 (15·1-15·6)	16·0 (15·8-16·2)	16·7 (16·4-16·9)	17·4 (17·1-17·6)	18·2 (18·0-18·5)	18·6 (17·7-19·5)
Stockholm	14·0 (12·3-15·8)	13·8 (12·6-15·1)	14·0 (13·5-14·5)	14·5 (14·2-15·0)	15·1 (14·6-15·5)	15·9 (15·5-16·2)	16·6 (16·3-17·0)	17·5 (17·1-17·9)	18·3 (18·0-18·8)	18·7 (17·1-20·3)
Sweden except Stockholm	14·1 (13·7-14·5)	14·1 (13·7-14·4)	14·3 (14·1-14·6)	14·8 (14·5-15·1)	15·4 (15·1-15·7)	16·0 (15·7-16·3)	16·7 (16·4-17·0)	17·4 (17·1-17·6)	18·2 (17·9-18·5)	18·6 (17·6-19·6)
Switzerland	13·2 (12·8-13·7)	13·9 (13·4-14·4)	14·3 (13·9-14·8)	15·0 (14·5-15·5)	15·4 (14·9-15·8)	16·1 (15·6-16·5)	17·0 (16·6-17·5)	18·0 (17·5-18·5)	18·8 (18·4-19·3)	19·5 (17·5-21·3)
United Kingdom	12·1 (12·0-12·2)	12·4 (12·2-12·5)	12·8 (12·8-12·9)	13·3 (13·2-13·3)	14·0 (13·9-14·1)	14·6 (14·5-14·7)	15·6 (15·5-15·7)	16·8 (16·7-16·9)	17·9 (17·9-18·0)	18·3 (18·1-18·4)
England	12·2 (12·1-12·3)	12·4 (12·3-12·5)	12·9 (12·9-13·0)	13·4 (13·3-13·4)	14·1 (14·1-14·2)	14·7 (14·7-14·8)	15·7 (15·7-15·8)	16·9 (16·9-17·0)	18·1 (18·0-18·2)	18·4 (18·3-18·5)
East Midlands	12·1 (11·7-12·6)	12·4 (12·0-12·8)	12·9 (12·6-13·2)	13·4 (13·2-13·6)	14·1 (13·9-14·3)	14·6 (14·4-14·8)	15·8 (15·6-16·0)	16·8 (16·6-17·0)	17·9 (17·7-18·1)	18·3 (18·0-18·6)
East of England	12·6 (12·2-13·1)	12·9 (12·5-13·2)	13·4 (13·1-13·7)	13·9 (13·7-14·1)	14·7 (14·5-14·9)	15·3 (15·1-15·5)	16·2 (16·0-16·5)	17·5 (17·3-17·7)	18·6 (18·4-18·9)	18·9 (18·6-19·3)
Greater London	12·5 (12·3-12·7)	12·7 (12·5-12·9)	13·1 (12·9-13·2)	13·5 (13·4-13·6)	14·3 (14·2-14·4)	14·9 (14·8-15·0)	15·8 (15·6-15·9)	17·0 (16·8-17·1)	18·3 (18·2-18·4)	18·6 (18·4-18·9)
North East England	11·3 (10·9-11·6)	11·5 (11·2-11·8)	12·0 (11·8-12·2)	12·4 (12·2-12·6)	13·0 (12·8-13·2)	13·5 (13·4-13·7)	14·7 (14·5-14·9)	15·9 (15·7-16·1)	17·2 (17·0-17·4)	17·6 (17·3-17·9)
North West England	11·5 (11·3-11·8)	11·8 (11·5-12·0)	12·2 (12·1-12·4)	12·7 (12·5-12·8)	13·3 (13·2-13·5)	14·0 (13·8-14·1)	15·0 (14·9-15·2)	16·2 (16·0-16·3)	17·3 (17·2-17·5)	17·7 (17·5-18·0)
South East England	12·8 (12·5-13·2)	13·1 (12·7-13·4)	13·6 (13·3-13·8)	14·0 (13·8-14·1)	14·8 (14·6-15·0)	15·4 (15·2-15·5)	16·4 (16·2-16·6)	17·6 (17·4-17·8)	18·7 (18·5-18·9)	19·0 (18·7-19·2)
South West England	13·0 (12·6-13·4)	13·2 (12·9-13·5)	13·7 (13·5-13·9)	14·2 (14·0-14·4)	14·9 (14·7-15·0)	15·5 (15·4-15·7)	16·5 (16·3-16·6)	17·6 (17·5-17·8)	18·7 (18·5-18·8)	19·0 (18·7-19·2)
West Midlands	11·8 (11·5-12·2)	12·1 (11·7-12·4)	12·5 (12·3-12·7)	13·0 (12·8-13·2)	13·7 (13·5-13·9)	14·4 (14·2-14·6)	15·4 (15·2-15·6)	16·6 (16·4-16·8)	17·9 (17·7-18·1)	18·2 (17·9-18·5)
Yorkshire and the Humber	11·7 (11·4-12·1)	12·0 (11·7-12·3)	12·5 (12·3-12·7)	12·9 (12·7-13·1)	13·6 (13·5-13·8)	14·3 (14·2-14·5)	15·3 (15·1-15·4)	16·6 (16·4-16·8)	17·6 (17·4-17·7)	17·9 (17·7-18·2)
Northern Ireland	12·1 (11·7-12·5)	12·3 (11·8-12·7)	12·4 (12·1-12·9)	12·9 (12·3-13·6)	13·5 (13·0-14·2)	14·3 (13·7-14·9)	15·2 (14·6-15·8)	16·5 (15·9-17·1)	17·5 (16·9-18·2)	17·8 (16·7-18·9)
Scotland	11·6 (11·2-12·0)	11·8 (11·5-12·1)	12·2 (11·9-12·6)	12·5 (12·0-13·0)	13·0 (12·5-13·5)	13·6 (13·1-14·2)	14·7 (14·1-15·3)	15·7 (15·1-16·2)	16·8 (16·2-17·3)	17·2 (16·2-18·1)

Appendix Table 14B. Estimates of life expectancy at age 65 by location, males, 1970, 1975, 1980, 1985, 1990, 1995, 2000, 2005, 2010, 2016

Location	1970	1975	1980	1985	1990	1995	2000	2005	2010	2016
Wales	11·7 (10·7-12·8)	12·0 (11·0-13·0)	12·5 (11·8-13·3)	13·1 (12·6-13·7)	13·7 (13·1-14·2)	14·4 (13·8-14·9)	15·4 (14·8-16·0)	16·6 (16·0-17·1)	17·6 (16·9-18·2)	17·7 (16·7-18·7)
Southern Latin America	12·9 (12·6-13·2)	13·4 (13·0-13·8)	13·5 (13·3-13·7)	13·8 (13·6-14·0)	14·0 (13·8-14·3)	14·4 (14·2-14·7)	14·8 (14·6-15·0)	15·2 (15·0-15·5)	15·6 (15·4-15·9)	16·1 (15·5-16·8)
Argentina	12·7 (12·3-13·0)	13·2 (12·6-13·7)	13·3 (13·1-13·6)	13·7 (13·4-14·0)	13·9 (13·6-14·2)	14·3 (14·0-14·6)	14·4 (14·1-14·7)	14·7 (14·4-15·0)	15·1 (14·9-15·4)	15·6 (14·9-16·2)
Chile	13·7 (13·3-14·3)	14·4 (13·9-14·9)	14·3 (13·8-14·8)	14·2 (13·7-14·7)	14·7 (14·2-15·2)	15·2 (14·8-15·8)	16·2 (15·6-16·7)	16·9 (16·4-17·4)	17·3 (16·9-17·8)	17·9 (15·9-20·0)
Uruguay	13·0 (12·8-13·2)	13·2 (13·0-13·4)	13·4 (13·1-13·6)	13·5 (13·3-13·7)	13·7 (13·5-13·9)	13·8 (13·6-14·0)	14·4 (14·2-14·6)	14·7 (14·4-14·9)	15·0 (14·8-15·3)	15·5 (15·0-16·1)
Central Europe, Eastern Europe, and Central Asi	12·4 (12·2-12·7)	12·4 (12·2-12·7)	12·2 (11·9-12·4)	12·2 (12·0-12·4)	12·4 (12·2-12·7)	11·6 (11·3-11·9)	12·0 (11·7-12·3)	12·1 (11·9-12·4)	13·1 (12·9-13·4)	14·1 (13·2-15·0)
Eastern Europe	12·4 (12·0-12·9)	12·2 (11·8-12·6)	11·9 (11·5-12·3)	11·9 (11·5-12·2)	12·2 (11·8-12·7)	10·9 (10·4-11·4)	11·1 (10·7-11·6)	11·2 (10·8-11·7)	12·3 (11·9-12·8)	13·3 (11·9-14·9)
Belarus	14·1 (13·7-14·4)	13·8 (13·5-14·2)	13·2 (12·8-13·6)	13·0 (12·6-13·3)	12·9 (12·6-13·3)	11·7 (11·4-12·0)	11·5 (11·2-11·9)	11·4 (11·0-11·7)	11·8 (11·5-12·2)	13·3 (12·0-14·6)
Estonia	12·3 (11·9-12·5)	12·2 (11·9-12·5)	12·0 (11·7-12·3)	12·0 (11·7-12·3)	12·2 (11·9-12·5)	11·6 (11·4-11·9)	12·7 (12·4-13·0)	13·3 (13·0-13·6)	14·6 (14·3-15·0)	15·7 (14·9-16·6)
Latvia	12·9 (12·6-13·3)	12·4 (12·0-12·7)	12·2 (11·9-12·6)	12·1 (11·8-12·5)	12·2 (11·8-12·6)	11·5 (11·2-11·9)	12·6 (12·2-13·0)	12·4 (12·0-12·8)	13·4 (13·0-13·8)	14·2 (13·3-15·3)
Lithuania	13·9 (13·6-14·2)	14·1 (13·9-14·5)	13·6 (13·4-13·9)	13·5 (13·2-13·7)	13·5 (13·2-13·8)	12·6 (12·3-12·9)	13·6 (13·3-14·0)	13·3 (13·0-13·6)	13·8 (13·5-14·1)	14·7 (14·1-15·3)
Moldova	11·5 (10·2-13·2)	11·8 (10·5-13·1)	12·0 (11·1-12·9)	11·7 (11·1-12·4)	12·1 (11·5-12·7)	10·8 (10·3-11·3)	11·9 (11·3-12·5)	11·6 (11·1-12·3)	12·3 (11·6-12·9)	13·3 (12·4-14·4)
Russia	12·0 (11·4-12·7)	11·8 (11·2-12·5)	11·5 (10·9-12·2)	11·6 (11·0-12·1)	12·0 (11·4-12·8)	10·6 (10·0-11·4)	10·8 (10·2-11·5)	11·0 (10·5-11·7)	12·2 (11·5-12·9)	13·2 (11·3-15·7)
Ukraine	12·9 (12·5-13·3)	12·7 (12·3-13·1)	12·3 (11·9-12·7)	12·2 (11·9-12·6)	12·4 (12·0-12·8)	11·1 (10·8-11·5)	11·5 (11·1-11·9)	11·4 (11·0-11·8)	12·5 (12·0-12·9)	13·3 (11·6-15·5)
Central Europe	12·4 (12·3-12·5)	12·6 (12·5-12·8)	12·4 (12·3-12·5)	12·5 (12·3-12·6)	12·6 (12·5-12·8)	12·7 (12·6-12·8)	13·4 (13·2-13·5)	13·9 (13·8-14·0)	14·5 (14·4-14·6)	15·4 (15·1-15·7)
Albania	15·9 (15·1-16·7)	16·0 (15·2-17·0)	16·2 (15·4-17·0)	15·8 (15·3-16·2)	14·9 (14·7-15·1)	15·0 (14·8-15·2)	14·8 (14·6-15·0)	14·8 (14·6-15·0)	15·5 (15·1-15·9)	16·1 (14·9-17·3)
Bosnia and Herzegovina	12·3 (10·7-13·6)	12·4 (11·2-13·7)	12·4 (11·4-13·7)	12·5 (11·6-13·5)	12·8 (12·0-13·7)	12·9 (12·0-13·8)	14·2 (13·3-15·1)	14·6 (13·7-15·4)	15·2 (14·2-16·1)	15·6 (14·4-16·8)
Bulgaria	13·2 (12·9-13·4)	12·8 (12·6-13·1)	12·7 (12·5-13·0)	12·6 (12·3-12·8)	12·8 (12·6-13·0)	12·6 (12·3-12·8)	12·8 (12·6-13·1)	13·1 (12·9-13·4)	13·8 (13·5-14·0)	14·3 (13·1-15·6)
Croatia	11·7 (10·1-13·5)	12·1 (10·6-13·6)	12·3 (11·0-13·7)	12·3 (11·6-13·0)	12·7 (12·2-13·4)	13·0 (12·5-13·6)	13·3 (12·8-13·8)	13·8 (13·3-14·4)	14·5 (13·9-15·0)	15·1 (14·2-16·3)
Czech Republic	11·1 (10·9-11·2)	11·3 (11·1-11·4)	11·3 (11·2-11·5)	11·5 (11·3-11·7)	11·8 (11·7-12·0)	12·7 (12·6-12·9)	13·7 (13·5-13·9)	14·5 (14·3-14·6)	15·3 (15·1-15·5)	16·3 (15·9-16·8)
Hungary	12·0 (11·7-12·3)	12·1 (11·7-12·5)	11·7 (11·4-12·1)	11·8 (11·4-12·1)	12·1 (11·7-12·5)	12·2 (11·9-12·6)	12·9 (12·5-13·3)	13·4 (13·0-13·8)	14·0 (13·6-14·4)	14·7 (13·9-15·7)
Macedonia	13·3 (12·6-13·9)	13·2 (12·6-13·6)	13·1 (12·6-13·5)	12·9 (12·5-13·3)	12·6 (12·2-13·0)	12·3 (11·9-12·7)	12·4 (12·0-12·8)	12·7 (12·3-13·1)	13·0 (12·6-13·5)	13·7 (13·2-14·1)
Montenegro	14·4 (13·7-15·1)	14·8 (14·0-15·4)	14·5 (13·7-15·3)	14·6 (13·8-15·5)	15·4 (14·8-16·0)	14·3 (13·8-14·8)	14·0 (13·6-14·5)	14·6 (14·2-15·1)	15·4 (14·8-16·1)	15·7 (14·9-16·6)
Poland	12·3 (12·1-12·6)	12·6 (12·3-12·8)	12·2 (12·0-12·5)	12·3 (12·0-12·6)	12·4 (12·2-12·7)	12·7 (12·5-13·0)	13·5 (13·3-13·8)	14·4 (14·1-14·7)	15·0 (14·8-15·3)	16·0 (15·3-16·8)
Romania	12·9 (12·6-13·3)	13·3 (13·0-13·6)	12·9 (12·6-13·3)	12·9 (12·6-13·3)	13·2 (12·8-13·5)	12·6 (12·2-13·0)	13·6 (13·2-13·9)	13·7 (13·4-14·1)	14·0 (13·6-14·4)	14·8 (13·9-15·6)
Serbia	13·7 (13·0-14·4)	14·0 (13·0-14·8)	13·9 (12·9-14·9)	13·7 (12·8-14·9)	13·5 (12·9-14·2)	12·8 (12·3-13·3)	12·9 (12·5-13·3)	13·3 (12·9-13·7)	14·0 (13·6-14·4)	15·0 (14·6-15·4)
Slovakia	12·2 (11·9-12·5)	12·4 (12·1-12·7)	12·2 (11·9-12·5)	12·2 (12·0-12·5)	12·1 (11·8-12·5)	12·6 (12·3-13·0)	12·9 (12·6-13·2)	13·4 (13·0-13·7)	14·1 (13·8-14·5)	15·1 (14·2-16·0)
Slovenia	11·4 (9·8-13·3)	11·7 (10·1-13·4)	12·0 (11·0-13·1)	12·5 (11·9-13·1)	13·2 (12·7-13·9)	13·6 (13·0-14·2)	14·3 (13·7-14·9)	15·2 (14·6-15·9)	16·6 (16·0-17·2)	17·5 (16·5-18·6)
Central Asia	12·5 (11·8-13·1)	12·7 (12·0-13·3)	12·8 (12·4-13·3)	12·9 (12·6-13·2)	12·8 (12·5-13·1)	12·0 (11·8-12·3)	12·1 (11·9-12·4)	12·0 (11·7-12·2)	12·6 (12·4-12·9)	13·5 (13·1-14·0)
Armenia	14·2 (12·8-15·8)	14·3 (13·0-15·9)	14·5 (13·7-15·5)	14·7 (14·2-15·2)	14·0 (13·5-14·5)	12·9 (12·5-13·4)	13·9 (13·4-14·4)	13·8 (13·3-14·3)	14·0 (13·5-14·6)	14·8 (14·0-15·6)
Azerbaijan	12·6 (11·3-14·0)	12·8 (11·5-14·1)	12·6 (11·9-13·5)	12·3 (11·8-13·0)	12·4 (12·0-12·9)	12·1 (11·5-12·5)	12·2 (11·7-12·8)	12·3 (11·8-13·1)	12·4 (11·7-13·1)	13·8 (12·5-15·4)
Georgia	12·9 (11·4-14·6)	13·1 (11·5-15·0)	13·3 (12·3-14·4)	13·1 (12·5-13·9)	13·1 (12·5-13·9)	13·6 (13·0-14·3)	13·6 (13·0-14·4)	13·2 (12·7-13·7)	13·4 (12·8-14·0)	13·8 (12·6-15·1)
Kazakhstan	11·4 (9·5-13·7)	11·7 (9·7-13·8)	11·9 (10·7-13·3)	12·2 (11·4-13·2)	12·2 (11·5-13·0)	10·9 (10·2-11·6)	10·9 (10·3-11·6)	10·9 (10·2-11·7)	11·9 (11·2-12·6)	13·3 (11·9-14·7)
Kyrgyzstan	12·1 (10·7-13·6)	12·3 (11·0-13·7)	12·6 (12·0-13·5)	12·6 (12·2-12·9)	12·5 (12·1-12·8)	11·4 (11·1-11·7)	12·3 (12·0-12·6)	11·8 (11·4-12·1)	12·2 (11·9-12·6)	13·5 (12·9-14·3)
Mongolia	11·7 (10·9-12·3)	12·4 (11·8-13·1)	12·2 (11·6-12·9)	12·0 (11·3-12·8)	12·2 (11·5-12·8)	11·2 (10·7-11·9)	11·1 (10·6-11·7)	10·9 (10·4-11·5)	11·6 (11·0-12·4)	12·3 (11·5-13·2)
Tajikistan	13·4 (12·6-14·5)	13·7 (12·7-14·8)	14·0 (13·0-15·0)	13·7 (12·9-14·7)	13·6 (12·8-14·5)	13·3 (12·5-14·2)	12·8 (12·1-13·6)	12·6 (11·9-13·4)	13·3 (12·5-14·1)	14·9 (13·6-15·8)
Turkmenistan	12·1 (11·6-12·6)	12·2 (11·8-12·7)	12·4 (12·1-12·7)	12·5 (12·3-12·8)	12·4 (12·2-12·6)	12·0 (11·8-12·2)	12·3 (12·0-12·5)	12·6 (12·4-12·9)	13·2 (12·9-13·4)	13·7 (13·3-14·2)
Uzbekistan	12·8 (11·9-13·7)	13·0 (12·1-13·8)	13·3 (12·6-13·9)	13·4 (13·0-13·9)	13·2 (12·7-13·7)	12·2 (11·8-12·7)	11·9 (11·5-12·4)	11·7 (11·1-12·1)	12·7 (12·3-13·1)	13·1 (12·4-13·8)
Latin America and Caribbean	13·6 (13·5-13·8)	14·1 (14·0-14·2)	14·4 (14·3-14·5)	14·3 (14·2-14·4)	14·7 (14·6-14·8)	14·9 (14·8-15·0)	15·4 (15·3-15·5)	16·0 (15·9-16·0)	16·3 (16·1-16·4)	16·8 (16·6-16·9)
Central Latin America	14·4 (14·2-14·6)	14·8 (14·6-14·9)	15·2 (15·0-15·3)	15·1 (15·0-15·3)	15·8 (15·6-15·9)	15·9 (15·8-16·1)	16·5 (16·3-16·6)	16·7 (16·5-16·8)	16·9 (16·8-17·1)	17·3 (17·0-17·6)
Colombia	15·2 (14·9-15·4)	14·3 (14·0-14·6)	15·1 (14·8-15·4)	14·5 (14·2-14·7)	15·4 (15·2-15·7)	15·5 (15·2-15·7)	15·8 (15·6-16·1)	16·1 (15·9-16·4)	16·8 (16·6-17·0)	17·6 (16·9-18·3)
Costa Rica	15·1 (14·8-15·4)	15·7 (15·4-16·0)	16·1 (15·8-16·5)	16·2 (15·9-16·5)	16·4 (16·0-16·7)	16·6 (16·3-16·9)	17·3 (17·0-17·5)	18·3 (18·0-18·6)	19·2 (18·9-19·5)	19·5 (18·9-20·2)
El Salvador	14·0 (13·6-14·4)	14·0 (13·7-14·4)	16·9 (16·4-17·4)	15·2 (14·7-15·6)	15·3 (14·9-15·6)	15·9 (15·5-16·3)	16·6 (16·2-17·0)	17·4 (17·1-17·7)	17·7 (17·3-18·2)	18·0 (17·1-18·9)
Guatemala	12·5 (11·5-13·6)	13·6 (12·6-14·7)	14·7 (13·6-15·8)	14·3 (13·3-15·4)	14·8 (13·9-15·9)	15·0 (14·0-16·0)	15·6 (14·5-16·9)	16·2 (15·0-17·3)	16·7 (15·6-18·0)	16·9 (15·1-19·0)
Honduras	12·8 (12·4-13·6)	13·6 (13·0-14·5)	14·4 (13·4-15·6)	14·9 (14·0-16·1)	15·7 (14·1-16·9)	15·3 (13·2-16·9)	15·2 (13·2-17·1)	15·2 (13·2-17·5)	15·5 (13·4-17·7)	15·7 (13·5-17·9)
Mexico	14·4 (14·1-14·7)	15·1 (14·8-15·3)	15·2 (15·0-15·4)	15·4 (15·3-15·5)	16·0 (15·9-16·2)	16·3 (16·2-16·4)	16·8 (16·7-17·0)	16·8 (16·6-16·9)	16·9 (16·7-17·0)	17·2 (17·0-17·4)
Nicaragua	15·2 (14·7-15·8)	16·3 (15·9-16·6)	17·1 (16·3-18·0)	16·3 (15·5-17·3)	18·2 (17·9-18·6)	17·5 (17·1-17·9)	18·2 (17·9-18·5)	18·2 (17·9-18·5)	18·4 (18·0-18·7)	18·4 (17·1-19·7)
Panama	15·2 (14·6-15·8)	16·1 (15·5-16·7)	16·5 (15·9-17·1)	16·1 (15·5-16·7)	16·2 (15·6-16·8)	16·4 (15·8-16·9)	17·5 (16·9-18·2)	17·9 (17·4-18·6)	18·2 (17·6-18·9)	18·8 (17·6-19·9)
Venezuela	14·3 (13·8-14·9)	14·3 (13·8-14·8)	14·4 (13·9-15·0)	14·9 (14·3-15·5)	15·1 (14·5-15·6)	15·2 (14·6-15·9)	15·8 (15·3-16·4)	16·6 (16·0-17·1)	16·7 (16·2-17·3)	16·7 (15·3-18·0)
Andean Latin America	13·8 (13·0-14·5)	15·2 (14·8-15·6)	15·2 (14·9-15·6)	15·2 (14·9-15·6)	15·7 (15·3-16·1)	16·0 (15·6-16·4)	16·7 (16·4-17·1)	17·5 (17·1-17·9)	17·7 (17·3-18·2)	18·4 (17·5-19·2)
Bolivia	12·2 (11·7-12·9)	12·5 (12·0-13·2)	12·8 (12·3-13·4)	13·1 (12·6-13·9)	13·8 (13·1-14·4)	14·4 (13·7-15·0)	14·9 (14·4-15·4)	15·7 (15·0-16·5)	16·1 (15·3-17·4)	16·6 (15·5-18·0)
Ecuador	16·5 (16·2-16·9)	16·0 (15·7-16·4)	16·1 (15·7-16·5)	16·0 (15·7-16·4)	16·2 (15·8-16·5)	16·7 (16·4-17·1)	17·3 (16·9-17·7)	17·8 (17·5-18·2)	18·2 (17·8-18·6)	18·7 (18·0-19·3)
Peru	13·1 (11·9-14·4)	15·9 (15·1-16·6)	15·7 (15·2-16·4)	15·6 (15·1-16·2)	16·2 (15·6-16·8)	16·2 (15·6-16·8)	17·1 (16·6-17·7)	18·0 (17·3-18·7)	18·1 (17·4-18·8)	18·9 (17·4-20·5)
Caribbean	14·2 (13·9-14·4)	14·5 (14·3-14·8)	14·9 (14·7-15·1)	15·0 (14·8-15·2)	15·1 (14·9-15·3)	15·1 (14·9-15·3)	15·7 (15·5-15·9)	16·1 (15·9-16·4)	15·3 (14·3-16·1)	16·5 (16·1-16·9)
Antigua and Barbuda	11·6 (11·1-12·1)	11·2 (10·7-11·8)	12·1 (11·5-12·8)	13·0 (12·4-13·8)	14·1 (13·5-14·8)	15·0 (14·4-15·7)	15·3 (14·8-15·9)	16·3 (15·7-17·0)	16·6 (15·9-17·4)	16·8 (15·9-17·8)
The Bahamas	12·7 (12·3-13·1)	13·3 (13·0-13·7)	13·9 (13·6-14·2)	13·2 (12·9-13·5)	14·0 (13·6-14·3)	13·8 (13·4-14·1)	14·2 (13·9-14·5)	15·4 (15·0-15·7)	15·8 (15·5-16·2)	15·7 (15·1-16·5)

Appendix Table 14B. Estimates of life expectancy at age 65 by location, males, 1970, 1975, 1980, 1985, 1990, 1995, 2000, 2005, 2010, 2016

Location	1970	1975	1980	1985	1990	1995	2000	2005	2010	2016
Barbados	13·6 (13·1-14·1)	13·9 (13·4-14·4)	14·4 (14·0-15·0)	14·1 (13·6-14·5)	14·2 (13·7-14·6)	14·3 (13·8-14·9)	15·6 (15·0-16·2)	16·3 (15·8-16·8)	16·8 (16·3-17·4)	16·7 (16·0-17·5)
Belize	15·4 (15·0-16·0)	15·1 (14·8-15·5)	15·2 (14·7-15·5)	15·2 (14·8-15·8)	15·5 (14·3-16·2)	13·6 (12·8-14·4)	13·0 (12·2-13·6)	13·3 (12·4-14·1)	14·0 (13·0-14·6)	14·1 (13·1-15·2)
Bermuda	11·8 (11·4-12·3)	11·3 (10·9-11·8)	12·0 (11·5-12·6)	12·2 (11·7-12·8)	12·5 (11·9-13·0)	12·8 (12·3-13·3)	12·6 (12·2-13·2)	14·7 (14·2-15·2)	15·5 (14·9-16·1)	16·3 (15·3-17·2)
Cuba	14·9 (14·6-15·2)	15·5 (15·2-15·8)	15·8 (15·5-16·2)	15·8 (15·5-16·2)	15·7 (15·4-16·0)	15·7 (15·3-16·0)	16·4 (16·0-16·7)	16·8 (16·4-17·2)	17·1 (16·8-17·5)	17·3 (16·6-18·0)
Dominica	14·1 (13·6-14·6)	14·4 (14·0-14·9)	15·5 (15·0-16·0)	14·6 (14·0-15·3)	13·6 (13·1-14·3)	13·2 (12·7-13·9)	13·9 (13·4-14·7)	14·3 (13·7-15·0)	14·5 (14·0-15·2)	14·6 (13·7-15·8)
Dominican Republic	15·7 (15·4-16·3)	16·0 (15·5-16·6)	16·3 (15·7-16·9)	16·1 (15·7-16·7)	16·1 (15·5-16·6)	16·1 (15·7-16·7)	16·5 (16·0-17·3)	16·8 (16·3-17·8)	17·0 (16·5-18·1)	17·2 (16·3-18·5)
Grenada	13·3 (12·3-14·4)	13·3 (12·4-14·4)	13·7 (12·6-15·0)	13·4 (12·5-14·8)	13·7 (13·0-14·4)	13·9 (13·2-14·6)	13·8 (13·0-14·4)	13·8 (13·2-14·4)	13·9 (13·4-14·6)	14·0 (13·1-14·9)
Guyana	11·5 (10·9-12·1)	11·7 (11·0-12·3)	11·9 (11·3-12·6)	12·5 (11·8-13·1)	11·8 (11·2-12·4)	12·7 (12·2-13·2)	13·1 (12·6-13·6)	12·4 (11·9-12·9)	12·2 (11·7-12·8)	13·0 (12·3-13·7)
Haiti	11·7 (10·8-12·8)	11·8 (10·9-13·0)	11·9 (11·2-12·9)	12·0 (11·4-12·7)	12·3 (11·8-12·8)	12·5 (12·1-12·9)	12·8 (12·3-13·4)	13·2 (12·5-14·4)	9·1 (6·9-12·6)	13·8 (12·4-15·2)
Jamaica	14·0 (13·4-14·6)	14·9 (14·3-15·7)	15·1 (14·5-15·8)	15·5 (15·0-16·1)	16·8 (16·3-17·5)	16·6 (16·0-17·3)	16·3 (15·6-17·0)	17·1 (16·4-17·7)	16·4 (15·5-17·1)	16·3 (14·8-17·6)
Puerto Rico	15·1 (14·7-15·5)	14·6 (14·3-15·0)	15·6 (15·2-16·1)	15·8 (15·4-16·2)	15·9 (15·5-16·3)	15·8 (15·4-16·2)	16·8 (16·4-17·3)	17·2 (16·7-17·6)	17·4 (17·0-17·8)	17·6 (16·9-18·3)
Saint Lucia	13·4 (13·0-13·8)	12·8 (12·5-13·1)	13·3 (13·0-13·7)	13·8 (13·5-14·1)	15·0 (14·7-15·4)	15·9 (15·5-16·2)	16·7 (16·3-17·1)	16·1 (15·7-16·4)	17·0 (16·6-17·4)	17·2 (16·6-17·7)
Saint Vincent and the Grenadines	13·5 (12·9-14·3)	13·5 (13·0-14·2)	14·2 (13·2-15·1)	14·1 (13·1-15·5)	13·8 (13·1-15·1)	13·8 (13·2-14·4)	13·6 (12·8-14·2)	14·0 (13·4-14·5)	14·1 (13·5-14·6)	14·0 (13·1-14·6)
Suriname	13·2 (12·9-13·4)	13·0 (12·6-13·3)	13·1 (12·9-13·2)	14·3 (14·1-14·6)	15·2 (15·0-15·4)	15·1 (14·8-15·4)	14·6 (14·2-14·9)	14·1 (13·8-14·4)	14·1 (13·8-14·5)	14·5 (13·8-15·3)
Trinidad and Tobago	11·8 (11·5-12·1)	12·4 (12·1-12·7)	13·0 (12·7-13·3)	12·8 (12·4-13·1)	12·4 (12·1-12·7)	12·4 (12·1-12·7)	12·7 (12·4-13·0)	13·8 (13·5-14·1)	14·3 (13·9-14·7)	14·4 (13·8-15·0)
Virgin Islands, U.S.	12·1 (11·6-12·6)	12·5 (12·1-13·1)	13·0 (12·6-13·4)	13·7 (13·4-14·3)	14·0 (13·6-14·5)	14·0 (13·6-14·3)	14·2 (13·8-14·5)	14·5 (14·0-14·9)	14·4 (13·8-15·1)	14·6 (13·8-15·7)
Tropical Latin America	12·6 (12·3-13·0)	13·0 (12·8-13·3)	13·1 (12·9-13·2)	13·0 (12·8-13·1)	13·2 (13·0-13·3)	13·4 (13·3-13·6)	14·0 (13·9-14·2)	14·9 (14·7-15·0)	15·5 (15·4-15·7)	16·0 (15·7-16·2)
Brazil	12·6 (12·3-12·9)	13·0 (12·7-13·2)	13·0 (12·9-13·2)	12·9 (12·7-13·0)	13·1 (12·9-13·2)	13·3 (13·2-13·5)	14·0 (13·8-14·1)	14·8 (14·7-15·0)	15·5 (15·4-15·7)	16·0 (15·8-16·2)
Paraguay	15·9 (15·5-16·3)	16·0 (15·5-16·3)	16·4 (16·0-16·8)	16·3 (15·9-16·7)	16·6 (16·2-17·0)	16·4 (16·2-16·8)	16·4 (16·2-16·6)	16·3 (16·1-16·5)	16·0 (15·3-16·4)	15·7 (14·7-16·5)
Southeast Asia, East Asia, and Oceania	12·8 (12·7-13·0)	12·9 (12·8-13·0)	12·5 (12·3-12·6)	12·7 (12·6-12·8)	12·4 (12·3-12·6)	12·8 (12·7-12·9)	13·1 (13·0-13·2)	13·8 (13·6-13·9)	14·5 (14·3-14·6)	15·1 (14·9-15·2)
East Asia	13·0 (12·9-13·2)	13·1 (12·9-13·2)	12·3 (12·2-12·5)	12·5 (12·4-12·7)	12·2 (12·0-12·4)	12·6 (12·4-12·7)	12·9 (12·8-13·1)	13·7 (13·5-13·8)	14·5 (14·4-14·7)	15·2 (15·0-15·3)
China	13·0 (12·9-13·2)	13·1 (12·9-13·2)	12·3 (12·1-12·5)	12·5 (12·3-12·7)	12·1 (12·0-12·3)	12·5 (12·4-12·7)	12·9 (12·8-13·0)	13·6 (13·5-13·8)	14·5 (14·3-14·6)	15·1 (15·0-15·3)
North Korea	13·7 (13·2-14·3)	14·1 (13·2-14·8)	14·0 (13·1-15·2)	14·0 (13·2-15·3)	14·0 (13·2-15·3)	12·2 (11·0-14·0)	11·9 (10·8-13·4)	13·4 (13·0-14·0)	13·4 (13·0-14·0)	13·5 (13·1-14·0)
Taiwan (Province of China)	11·9 (11·6-12·2)	12·9 (12·5-13·2)	13·2 (12·8-13·5)	13·8 (13·5-14·1)	14·6 (14·3-15·0)	15·2 (14·8-15·5)	16·0 (15·7-16·4)	16·8 (16·4-17·2)	17·6 (17·2-18·0)	17·9 (16·9-19·0)
Southeast Asia	12·3 (12·0-12·5)	12·5 (12·3-12·8)	13·0 (12·7-13·2)	13·2 (13·0-13·4)	13·4 (13·2-13·6)	13·6 (13·3-13·8)	13·8 (13·6-14·0)	14·1 (13·8-14·3)	14·4 (14·2-14·7)	14·8 (14·5-15·2)
Cambodia	11·1 (10·1-11·6)	9·0 (7·4-11·0)	11·3 (10·5-12·0)	11·3 (10·6-12·0)	11·7 (11·2-12·3)	12·0 (11·5-12·4)	12·1 (11·5-12·5)	12·5 (11·9-13·1)	12·8 (12·2-13·4)	13·1 (12·6-13·7)
Indonesia	12·3 (12·0-12·6)	12·6 (12·4-12·9)	13·1 (12·8-13·3)	13·4 (13·2-13·6)	13·4 (13·2-13·6)	13·6 (13·5-13·8)	14·1 (13·9-14·3)	14·3 (14·0-14·6)	14·3 (13·9-14·8)	14·6 (14·0-15·3)
Laos	10·9 (10·4-11·3)	11·2 (10·7-11·5)	11·3 (11·0-11·6)	11·6 (11·4-11·8)	11·8 (11·6-11·9)	12·0 (11·8-12·1)	12·2 (12·1-12·4)	12·6 (12·4-12·8)	13·1 (12·8-13·3)	13·7 (13·3-14·2)
Malaysia	11·4 (11·2-11·7)	12·2 (12·0-12·5)	12·7 (12·5-13·0)	13·2 (13·0-13·4)	13·5 (13·3-13·7)	13·7 (13·6-13·9)	14·0 (13·9-14·2)	14·7 (14·6-14·9)	15·1 (14·9-15·4)	15·3 (14·8-15·8)
Maldives	12·8 (10·6-15·1)	12·6 (10·5-14·8)	12·7 (11·2-14·6)	13·2 (12·6-13·9)	13·6 (13·0-14·3)	13·9 (13·0-14·8)	14·8 (14·1-15·4)	15·9 (15·3-16·6)	16·5 (15·7-17·1)	17·2 (15·5-19·2)
Mauritius	8·6 (8·4-8·9)	8·3 (8·0-8·5)	9·6 (9·3-9·8)	10·3 (10·1-10·6)	11·0 (10·7-11·3)	11·6 (11·3-11·9)	12·9 (12·6-13·1)	13·4 (13·0-13·6)	14·4 (14·0-14·7)	14·8 (13·9-15·9)
Myanmar	10·3 (9·7-11·1)	10·7 (10·0-11·4)	11·0 (10·2-11·6)	11·5 (10·8-12·1)	11·6 (10·8-12·3)	11·7 (10·9-12·4)	12·1 (10·8-12·7)	12·6 (11·0-13·3)	13·0 (11·6-13·7)	13·4 (12·8-13·9)
Philippines	13·7 (13·3-14·2)	13·5 (13·1-13·9)	13·5 (13·1-13·9)	13·2 (12·9-13·6)	13·1 (12·7-13·5)	12·5 (12·1-12·9)	12·1 (11·7-12·5)	11·9 (11·5-12·3)	12·3 (11·9-12·7)	12·6 (11·6-13·6)
Sri Lanka	12·3 (11·9-12·8)	12·1 (11·7-12·6)	13·8 (13·3-14·2)	14·2 (13·7-14·7)	15·0 (14·4-15·5)	15·5 (14·8-16·2)	15·0 (14·5-15·6)	15·0 (14·2-15·7)	15·3 (14·5-16·1)	16·2 (14·4-18·1)
Seychelles	13·2 (12·4-13·5)	13·1 (12·6-13·7)	13·0 (12·7-13·4)	12·7 (12·2-13·3)	12·5 (12·1-12·8)	12·5 (12·2-12·9)	13·2 (12·9-13·5)	13·7 (13·4-13·9)	14·1 (13·7-14·7)	14·4 (13·6-15·4)
Thailand	14·2 (13·8-14·6)	14·6 (14·2-14·9)	14·2 (13·9-14·6)	14·2 (13·8-14·5)	14·7 (14·4-15·1)	15·5 (15·1-15·9)	16·1 (15·6-16·6)	16·6 (16·2-17·0)	17·8 (17·4-18·2)	18·3 (17·4-19·2)
Timor-Leste	12·5 (11·3-14·1)	12·7 (11·4-14·2)	13·5 (12·1-15·0)	14·2 (13·0-15·4)	14·4 (13·4-15·5)	14·5 (13·7-15·4)	14·7 (13·8-15·6)	14·8 (13·9-15·9)	15·9 (14·8-17·3)	16·1 (14·6-17·7)
Vietnam	12·0 (10·6-12·9)	12·5 (11·1-13·2)	12·9 (11·6-13·4)	13·3 (12·2-13·8)	13·5 (12·5-14·4)	13·4 (12·5-14·4)	13·3 (12·8-14·4)	13·5 (12·8-14·7)	14·0 (13·1-15·0)	14·5 (13·9-15·1)
Oceania	10·4 (9·9-11·3)	10·3 (9·8-11·0)	10·3 (9·9-10·9)	10·4 (10·0-10·9)	10·6 (10·2-11·1)	10·8 (10·3-11·3)	10·9 (10·4-11·4)	10·9 (10·4-11·5)	11·1 (10·5-11·7)	11·5 (10·9-12·1)
American Samoa	12·1 (11·4-12·9)	12·5 (11·8-13·1)	12·8 (12·1-13·4)	13·1 (12·5-13·7)	12·9 (12·4-13·5)	13·3 (12·6-14·0)	13·8 (13·2-14·3)	13·9 (13·2-14·5)	14·3 (13·5-15·1)	14·4 (13·3-15·5)
Federated States of Micronesia	11·2 (10·1-12·7)	10·9 (10·1-12·4)	10·9 (10·2-12·1)	11·0 (10·4-12·1)	11·2 (10·7-12·1)	11·5 (10·9-12·4)	11·9 (11·2-12·6)	12·2 (11·5-13·0)	12·0 (10·9-12·9)	12·0 (11·0-13·0)
Fiji	11·6 (10·4-13·3)	11·1 (10·3-12·4)	11·3 (10·5-12·4)	11·6 (10·9-12·3)	11·7 (10·7-12·9)	11·4 (10·5-12·5)	11·2 (10·6-11·9)	11·3 (10·6-12·3)	11·3 (10·4-12·5)	11·7 (10·2-13·6)
Guam	12·4 (11·8-12·9)	12·2 (11·5-12·8)	13·0 (12·3-13·7)	13·9 (13·2-14·5)	14·2 (13·7-14·7)	14·5 (13·7-15·1)	14·6 (14·0-15·1)	14·4 (13·8-14·8)	13·8 (13·2-14·5)	13·8 (13·0-14·8)
Kiribati	10·2 (9·3-11·6)	10·5 (9·6-11·9)	10·5 (9·7-11·7)	10·1 (9·6-11·0)	10·0 (9·6-10·4)	9·9 (9·7-10·2)	10·0 (9·7-10·3)	10·2 (9·7-10·6)	10·3 (9·7-11·0)	10·6 (9·9-11·3)
Marshall Islands	10·7 (9·6-12·5)	11·0 (10·0-12·6)	11·2 (10·5-12·7)	11·2 (10·7-12·2)	11·3 (10·9-11·8)	11·4 (11·0-12·0)	11·3 (10·8-12·0)	11·3 (10·7-12·0)	11·4 (10·8-12·2)	11·8 (10·9-12·8)
Northern Mariana Islands	13·4 (12·3-15·0)	13·9 (12·8-15·4)	14·4 (13·2-15·7)	14·7 (13·6-16·0)	15·3 (14·2-16·4)	15·9 (14·8-16·8)	16·3 (15·3-17·1)	16·2 (15·3-17·1)	16·1 (15·2-17·0)	15·8 (14·8-16·8)
Papua New Guinea	9·9 (9·2-11·4)	9·8 (9·1-11·1)	9·7 (9·1-10·6)	9·7 (9·2-10·5)	9·9 (9·3-10·6)	10·1 (9·5-10·9)	10·3 (9·7-11·2)	10·4 (9·7-11·3)	10·6 (9·9-11·6)	11·1 (10·3-12·0)
Samoa	12·5 (11·6-13·4)	11·8 (11·1-12·7)	12·1 (11·4-12·8)	12·4 (11·5-13·1)	12·7 (11·9-13·4)	13·0 (12·2-13·7)	13·4 (12·5-14·2)	13·7 (12·9-14·7)	13·9 (13·1-15·0)	14·1 (13·4-15·4)
Solomon Islands	9·9 (9·1-11·1)	9·6 (9·1-10·5)	10·2 (9·6-10·8)	10·4 (9·8-11·0)	10·6 (10·0-11·3)	10·9 (10·3-11·5)	11·1 (10·5-11·6)	11·0 (10·4-11·6)	11·2 (10·5-11·8)	11·5 (10·7-12·3)
Tonga	12·2 (11·0-13·4)	12·2 (11·1-13·4)	12·2 (11·1-13·4)	12·3 (11·4-13·4)	12·6 (11·7-13·6)	12·8 (11·9-13·6)	12·9 (12·1-13·7)	13·0 (12·3-13·9)	13·1 (12·3-14·0)	13·3 (12·5-14·3)
Vanuatu	10·2 (9·4-11·5)	10·2 (9·6-11·2)	10·4 (9·8-11·1)	10·7 (10·2-11·2)	10·9 (10·4-11·4)	11·1 (10·5-11·6)	11·1 (10·6-11·7)	11·2 (10·6-11·8)	11·4 (10·7-12·1)	11·7 (10·9-12·5)
North Africa and Middle East	13·0 (12·7-13·4)	13·3 (13·0-13·6)	13·7 (13·4-14·0)	13·9 (13·7-14·2)	14·4 (14·2-14·6)	14·3 (14·1-14·5)	14·7 (14·5-14·9)	15·0 (14·7-15·3)	15·4 (15·1-15·7)	15·7 (15·3-16·1)
North Africa and Middle East	13·0 (12·7-13·4)	13·3 (13·0-13·6)	13·7 (13·4-14·0)	13·9 (13·7-14·2)	14·4 (14·2-14·6)	14·3 (14·1-14·5)	14·7 (14·5-14·9)	15·0 (14·7-15·3)	15·4 (15·1-15·7)	15·7 (15·3-16·1)
Afghanistan	10·9 (10·2-11·5)	10·8 (10·2-11·4)	10·5 (9·8-11·1)	10·3 (9·6-11·1)	10·8 (10·1-11·6)	10·4 (9·7-11·1)	10·1 (9·6-10·9)	10·3 (9·7-11·0)	10·5 (10·0-11·2)	10·9 (10·4-11·5)
Algeria	13·6 (12·9-14·3)	13·4 (12·8-14·1)	13·8 (13·3-14·3)	14·5 (14·0-15·0)	15·3 (14·8-16·0)	15·8 (15·2-16·5)	16·3 (15·7-17·0)	17·0 (16·4-17·7)	17·6 (17·1-18·1)	17·8 (17·0-18·6)
Bahrain	12·9 (11·9-13·9)	12·7 (11·5-13·9)	12·1 (11·1-13·3)	11·9 (11·1-12·9)	11·7 (10·8-12·7)	11·9 (10·8-12·9)	12·7 (11·8-13·5)	13·6 (12·6-14·7)	15·0 (13·9-16·1)	15·5 (13·9-17·2)

Appendix Table 14B. Estimates of life expectancy at age 65 by location, males, 1970, 1975, 1980, 1985, 1990, 1995, 2000, 2005, 2010, 2016

Location	1970	1975	1980	1985	1990	1995	2000	2005	2010	2016
Egypt	11·8 (11·4-12·1)	12·0 (11·6-12·4)	12·6 (12·2-13·0)	11·8 (11·5-12·3)	12·9 (12·6-13·4)	13·2 (12·8-13·6)	13·8 (13·4-14·2)	13·6 (13·2-14·1)	13·5 (13·1-13·9)	14·0 (13·0-15·1)
Iran	15·3 (13·9-16·7)	15·6 (14·5-16·8)	15·5 (14·5-16·7)	15·7 (14·6-16·9)	14·8 (13·7-16·1)	14·9 (14·0-15·9)	15·0 (13·9-15·8)	15·1 (13·6-16·4)	16·0 (14·4-17·3)	16·3 (14·9-17·7)
Iraq	13·3 (12·8-13·9)	13·3 (12·8-13·9)	13·3 (12·8-14·2)	13·3 (12·3-14·2)	13·5 (12·1-14·5)	12·9 (11·6-14·0)	12·7 (11·6-13·7)	12·6 (11·5-13·6)	12·8 (11·8-14·2)	13·2 (12·1-15·7)
Jordan	12·8 (11·7-13·7)	13·1 (12·0-13·9)	13·4 (12·1-14·3)	14·5 (13·4-15·3)	15·6 (14·6-16·2)	15·8 (14·7-16·7)	15·9 (14·1-17·2)	15·6 (14·1-17·0)	16·5 (14·8-18·2)	16·9 (14·7-19·2)
Kuwait	13·7 (12·7-14·8)	14·0 (13·0-14·9)	14·8 (13·9-15·7)	15·5 (14·5-16·6)	16·7 (15·4-18·0)	16·7 (15·6-17·7)	17·2 (16·3-18·3)	17·9 (16·8-19·0)	19·0 (17·9-20·1)	19·8 (17·4-22·4)
Lebanon	13·8 (12·3-15·2)	13·4 (11·9-14·7)	12·6 (11·2-14·3)	13·1 (12·2-15·0)	13·8 (13·0-15·5)	16·1 (15·7-16·7)	17·4 (16·3-18·3)	18·1 (17·0-19·3)	18·8 (17·9-19·7)	18·6 (17·3-19·7)
Libya	15·9 (15·0-16·8)	16·0 (15·2-16·8)	16·1 (15·1-16·8)	15·9 (15·2-16·8)	15·9 (15·4-16·7)	16·3 (15·7-17·0)	16·4 (15·9-17·0)	16·5 (16·0-17·1)	16·3 (15·0-17·3)	15·4 (14·7-16·8)
Morocco	12·9 (12·5-13·4)	13·1 (12·7-13·6)	13·4 (13·0-13·8)	13·9 (13·6-14·3)	14·3 (13·9-14·6)	14·7 (14·4-15·0)	15·2 (14·9-15·5)	15·7 (15·3-16·0)	16·1 (15·8-16·5)	16·5 (16·0-16·9)
Palestine	13·6 (12·6-15·0)	14·0 (12·8-15·3)	14·4 (13·4-16·0)	14·3 (13·3-15·7)	14·3 (13·5-15·4)	14·0 (13·4-14·8)	12·9 (12·7-13·1)	12·8 (12·6-12·9)	13·0 (12·9-13·2)	12·9 (12·6-13·3)
Oman	14·1 (13·5-14·8)	14·5 (13·9-15·1)	14·7 (14·2-15·4)	14·9 (14·3-15·7)	15·2 (14·7-15·6)	15·7 (15·3-16·1)	15·3 (14·5-16·2)	15·4 (14·8-16·0)	15·3 (14·7-15·9)	16·0 (15·7-16·5)
Qatar	13·9 (11·6-16·4)	14·0 (11·9-16·3)	13·5 (11·9-15·5)	15·8 (13·7-16·8)	17·0 (15·7-18·4)	16·5 (14·9-17·9)	16·3 (14·6-17·9)	16·6 (14·9-18·5)	18·3 (16·6-20·0)	18·6 (16·1-21·3)
Saudi Arabia	16·2 (15·3-17·2)	16·6 (15·7-17·6)	16·8 (15·9-17·7)	16·3 (15·5-17·2)	15·7 (15·0-16·5)	15·6 (15·0-16·1)	15·2 (14·8-15·6)	15·1 (14·7-15·4)	15·4 (15·0-15·8)	16·2 (15·6-16·8)
Sudan	12·4 (12·0-12·6)	12·4 (12·1-12·6)	12·6 (12·3-12·8)	12·0 (11·5-12·6)	13·1 (12·8-13·4)	13·2 (12·9-13·6)	13·4 (13·1-13·8)	13·7 (13·4-14·2)	14·2 (13·8-14·6)	14·7 (14·2-15·1)
Syria	13·9 (13·4-14·4)	13·9 (13·5-14·4)	14·3 (13·9-14·7)	14·7 (14·4-15·1)	15·0 (14·7-15·3)	15·2 (14·0-15·7)	14·8 (14·3-15·6)	15·6 (15·1-16·1)	16·0 (15·5-16·7)	15·0 (14·3-15·9)
Tunisia	12·9 (12·4-13·4)	13·2 (12·7-13·7)	12·8 (12·2-13·5)	14·5 (13·8-15·3)	14·6 (14·0-15·3)	14·4 (13·8-15·1)	15·7 (15·1-16·6)	16·2 (15·5-17·3)	16·5 (14·9-17·7)	16·1 (14·6-18·0)
Turkey	13·2 (12·0-14·9)	13·8 (12·8-14·9)	14·6 (13·7-15·5)	15·6 (14·7-16·4)	15·8 (15·3-16·2)	15·0 (14·6-15·4)	15·8 (15·4-16·1)	16·7 (16·2-17·2)	17·1 (16·4-17·8)	17·3 (15·9-18·7)
United Arab Emirates	15·7 (14·1-17·7)	15·8 (14·2-18·0)	15·8 (14·4-17·8)	15·8 (13·8-17·5)	15·3 (13·4-17·0)	15·0 (13·9-16·6)	15·4 (14·5-16·4)	16·3 (15·8-16·6)	16·1 (15·2-16·8)	16·0 (14·8-17·0)
Yemen	11·3 (10·9-11·7)	11·9 (11·5-12·3)	12·4 (11·9-12·7)	12·8 (12·3-13·2)	13·1 (12·6-13·6)	13·2 (12·8-13·7)	13·4 (13·0-13·9)	13·8 (13·5-14·3)	14·6 (13·9-15·0)	14·5 (13·6-15·4)
South Asia	10·9 (10·7-11·1)	11·2 (11·0-11·4)	11·7 (11·5-11·8)	11·9 (11·7-12·1)	12·1 (12·0-12·3)	12·4 (12·2-12·5)	12·6 (12·5-12·8)	13·1 (12·9-13·3)	13·4 (13·2-13·5)	13·8 (13·6-14·0)
South Asia	10·9 (10·7-11·1)	11·2 (11·0-11·4)	11·7 (11·5-11·8)	11·9 (11·7-12·1)	12·1 (12·0-12·3)	12·4 (12·2-12·5)	12·6 (12·5-12·8)	13·1 (12·9-13·3)	13·4 (13·2-13·5)	13·8 (13·6-14·0)
Bangladesh	11·9 (10·9-13·1)	12·9 (12·1-13·9)	12·9 (12·3-13·7)	12·7 (12·2-13·2)	12·8 (12·3-13·4)	13·4 (12·9-14·1)	14·1 (13·6-14·7)	14·5 (14·0-14·9)	14·9 (14·4-15·4)	15·3 (14·5-16·1)
Bhutan	13·2 (12·2-14·4)	13·4 (12·4-14·6)	13·5 (12·4-14·7)	13·8 (12·7-15·0)	14·3 (13·1-15·3)	14·7 (13·7-15·6)	15·4 (14·5-16·2)	15·9 (15·0-16·8)	16·2 (15·2-17·1)	16·3 (15·2-17·4)
India	10·5 (10·3-10·7)	10·7 (10·5-11·0)	11·2 (11·1-11·5)	11·6 (11·4-11·8)	11·8 (11·6-12·0)	12·1 (11·9-12·3)	12·4 (12·2-12·6)	12·9 (12·7-13·1)	13·1 (12·9-13·3)	13·6 (13·4-13·8)
Nepal	12·3 (11·9-12·7)	12·4 (11·9-12·8)	12·4 (11·9-12·8)	12·5 (12·1-12·9)	12·7 (12·3-13·1)	12·8 (12·5-13·3)	13·2 (12·9-13·5)	13·9 (13·5-14·2)	14·2 (13·7-14·8)	14·7 (14·0-15·6)
Pakistan	13·6 (12·6-14·8)	13·9 (12·9-14·9)	14·1 (13·3-14·9)	14·3 (13·8-14·9)	14·1 (13·6-14·6)	13·4 (12·9-13·9)	13·2 (12·8-13·7)	13·2 (12·7-13·7)	13·7 (13·1-14·3)	14·1 (13·2-14·9)
Sub-Saharan Africa	12·3 (12·1-12·6)	12·5 (12·2-12·7)	12·6 (12·3-12·8)	12·6 (12·5-12·8)	12·6 (12·4-12·8)	12·5 (12·3-12·7)	12·3 (12·1-12·5)	12·6 (12·4-12·8)	13·1 (12·9-13·3)	13·7 (13·4-14·0)
Southern Sub-Saharan Africa	14·0 (13·4-14·6)	14·3 (13·8-14·9)	14·4 (14·0-14·9)	14·7 (14·4-15·0)	14·9 (14·5-15·5)	13·9 (13·4-14·4)	11·7 (11·4-12·1)	11·6 (11·4-11·8)	12·0 (11·8-12·3)	13·4 (13·1-13·8)
Botswana	10·3 (9·9-10·9)	10·9 (10·5-11·6)	11·3 (10·8-12·1)	12·1 (11·3-13·0)	12·5 (11·2-14·0)	11·5 (9·4-15·6)	10·0 (7·7-13·1)	10·3 (8·6-12·7)	11·6 (10·1-13·4)	12·8 (11·3-15·9)
Lesotho	10·2 (9·1-11·7)	10·4 (9·3-12·1)	10·8 (9·6-12·4)	11·0 (10·0-12·5)	11·0 (10·2-12·3)	10·6 (9·9-11·7)	9·3 (8·8-10·2)	8·6 (8·1-9·3)	9·0 (8·4-9·6)	9·4 (8·6-10·4)
Namibia	11·4 (10·5-12·5)	11·6 (10·7-12·6)	11·5 (10·7-12·3)	11·4 (10·8-12·1)	11·3 (10·8-12·1)	11·1 (10·6-12·1)	10·4 (9·7-11·5)	10·4 (9·7-11·9)	12·0 (11·0-13·4)	12·7 (11·7-14·2)
South Africa	15·0 (14·3-15·9)	15·3 (14·6-16·0)	15·3 (14·8-15·9)	15·4 (15·0-15·8)	15·4 (15·1-15·8)	14·4 (14·2-14·7)	12·3 (12·2-12·5)	12·4 (12·2-12·5)	12·7 (12·5-12·8)	14·2 (13·8-14·5)
Swaziland	10·9 (9·6-12·0)	11·2 (9·9-12·2)	11·4 (10·1-12·4)	11·5 (10·2-12·7)	11·5 (10·5-12·8)	11·5 (10·3-13·1)	9·8 (8·6-12·1)	9·0 (8·1-10·4)	9·6 (8·8-11·4)	11·1 (9·9-13·4)
Zimbabwe	11·3 (10·2-12·5)	11·8 (10·6-12·7)	12·2 (11·2-12·9)	13·1 (12·7-13·5)	14·2 (12·9-18·3)	13·2 (11·1-16·1)	10·5 (9·3-12·2)	9·6 (9·0-10·4)	10·2 (9·5-11·4)	11·6 (10·5-13·1)
Western Sub-Saharan Africa	13·0 (12·5-13·5)	13·2 (12·8-13·7)	13·5 (13·0-13·9)	13·5 (13·1-14·0)	13·4 (13·0-13·8)	13·4 (12·9-13·9)	13·3 (12·9-13·8)	13·6 (13·2-14·1)	14·0 (13·6-14·5)	14·6 (14·0-15·1)
Benin	12·1 (11·7-12·6)	12·2 (11·8-12·7)	12·3 (11·9-12·7)	12·6 (12·1-13·0)	12·7 (12·3-13·2)	12·6 (12·1-13·1)	12·5 (12·2-13·0)	12·7 (12·4-13·1)	12·9 (12·6-13·3)	13·3 (13·0-13·8)
Burkina Faso	11·7 (11·5-12·0)	11·8 (11·6-12·0)	11·9 (11·7-12·2)	12·3 (12·0-12·6)	12·2 (11·7-13·2)	12·2 (11·6-12·9)	12·3 (11·8-12·7)	12·7 (12·3-13·0)	12·9 (12·6-13·2)	13·2 (12·9-13·5)
Cameroon	12·6 (11·9-13·4)	12·8 (12·2-13·6)	13·1 (12·5-13·8)	13·3 (12·8-14·0)	13·2 (12·6-14·0)	12·7 (12·2-13·7)	12·2 (11·7-12·9)	12·3 (11·7-12·8)	12·6 (12·0-13·2)	12·9 (12·1-13·8)
Cape Verde	13·7 (12·4-15·4)	13·9 (12·8-15·1)	13·6 (12·9-14·5)	13·7 (13·1-14·5)	13·6 (13·3-14·2)	13·6 (12·8-14·4)	13·7 (12·4-15·0)	13·4 (12·5-15·4)	13·4 (12·9-14·3)	14·0 (13·2-15·0)
Chad	12·6 (12·1-13·1)	12·7 (12·2-13·3)	12·6 (12·2-13·1)	12·8 (12·3-13·3)	12·8 (12·4-13·2)	12·6 (12·0-13·0)	12·1 (11·7-12·7)	12·5 (11·9-13·1)	12·7 (12·2-13·3)	13·1 (12·6-14·0)
Cote d'Ivoire	12·2 (11·7-12·6)	12·3 (11·9-12·7)	12·8 (12·3-13·1)	12·6 (12·1-13·0)	12·0 (11·6-12·3)	11·4 (10·7-11·9)	11·5 (10·9-12·0)	11·8 (11·0-12·2)	12·1 (11·4-12·6)	12·6 (11·7-13·1)
The Gambia	13·4 (12·6-14·3)	13·5 (12·8-14·4)	13·7 (13·0-14·5)	13·9 (13·1-14·7)	14·0 (13·2-14·7)	14·0 (13·3-14·8)	13·8 (13·2-14·6)	13·7 (13·3-14·4)	14·1 (13·6-14·6)	14·5 (13·8-15·1)
Ghana	13·0 (12·3-13·8)	13·1 (12·4-14·0)	13·2 (12·5-14·0)	13·1 (12·4-14·0)	13·0 (12·5-13·8)	13·2 (12·6-14·0)	12·9 (12·5-13·5)	12·9 (12·6-13·3)	13·2 (12·8-13·6)	13·9 (13·2-14·6)
Guinea	13·0 (12·1-14·1)	13·1 (12·3-14·2)	13·4 (12·6-14·3)	13·7 (13·0-14·6)	13·7 (13·1-14·4)	13·3 (12·7-13·9)	12·8 (12·3-13·4)	12·8 (12·2-13·5)	12·8 (12·3-13·5)	13·1 (12·5-13·9)
Guinea-Bissau	10·4 (9·8-11·0)	10·7 (10·1-11·3)	10·8 (10·2-11·4)	10·8 (10·1-11·4)	10·8 (10·1-11·5)	10·8 (10·1-11·5)	10·8 (10·0-11·6)	10·8 (10·0-11·8)	10·9 (10·2-11·9)	11·1 (10·4-12·2)
Liberia	13·3 (12·7-14·1)	13·6 (13·0-14·3)	13·7 (13·1-14·3)	13·6 (13·0-14·2)	13·3 (12·8-13·9)	13·1 (12·6-13·7)	13·2 (12·7-13·9)	13·5 (12·8-14·1)	13·3 (12·9-13·9)	13·9 (13·5-14·3)
Mali	11·4 (10·6-12·3)	11·4 (10·7-12·1)	11·8 (11·2-12·5)	12·2 (11·7-12·7)	12·6 (12·2-13·2)	13·1 (12·5-13·7)	13·9 (13·3-14·5)	14·1 (13·4-14·8)	14·4 (13·4-15·2)	14·7 (13·4-16·0)
Mauritania	12·4 (11·3-13·9)	12·5 (11·5-13·8)	12·7 (11·9-13·8)	12·8 (12·3-13·6)	13·1 (12·7-13·9)	13·6 (12·9-14·3)	14·6 (13·8-15·4)	15·3 (14·5-16·3)	15·9 (14·8-17·2)	16·1 (14·8-17·9)
Niger	11·8 (11·0-12·7)	11·7 (11·1-12·4)	11·8 (11·2-12·4)	11·9 (11·4-12·5)	12·7 (12·1-13·5)	13·2 (12·6-13·9)	13·4 (12·8-14·1)	13·4 (12·8-14·1)	13·4 (12·6-14·2)	13·5 (12·7-14·5)
Nigeria	13·9 (12·8-15·0)	14·3 (13·3-15·5)	14·7 (13·6-15·9)	14·7 (13·7-15·7)	14·3 (13·3-15·4)	14·4 (13·4-16·0)	14·3 (13·3-15·6)	14·8 (13·9-16·1)	15·6 (14·6-16·9)	16·3 (15·0-17·8)
Sao Tome and Principe	12·8 (12·3-13·2)	13·2 (12·7-13·8)	13·8 (13·3-14·4)	14·1 (13·4-14·7)	14·0 (13·3-14·8)	14·1 (13·4-14·9)	13·7 (13·3-14·4)	13·9 (13·5-14·4)	14·6 (13·8-15·2)	14·6 (13·2-16·0)
Senegal	11·8 (11·4-12·3)	11·8 (11·4-12·3)	12·1 (11·7-12·5)	12·6 (12·1-12·9)	12·6 (12·3-13·1)	12·7 (12·4-13·1)	12·8 (12·5-13·2)	13·0 (12·8-13·4)	13·3 (13·1-13·6)	13·6 (13·0-14·3)
Sierra Leone	12·0 (11·4-12·7)	12·1 (11·5-12·8)	12·2 (11·6-13·0)	12·3 (11·7-12·9)	12·3 (11·8-13·0)	12·2 (11·6-12·6)	12·0 (11·6-12·5)	12·0 (11·7-12·4)	12·4 (12·0-12·9)	12·9 (12·4-13·5)
Togo	12·5 (12·0-13·1)	12·7 (12·2-13·4)	12·9 (12·3-13·4)	12·8 (12·3-13·3)	12·8 (12·3-13·3)	12·4 (12·2-13·0)	12·2 (11·6-12·6)	12·2 (11·5-12·6)	12·3 (11·5-12·8)	12·8 (11·9-13·4)
Eastern Sub-Saharan Africa	11·3 (10·9-11·7)	11·4 (11·0-11·8)	11·4 (11·0-11·8)	11·4 (11·2-11·7)	11·4 (11·2-11·7)	11·5 (11·2-11·8)	11·7 (11·5-12·0)	12·2 (11·9-12·4)	12·7 (12·3-13·0)	13·2 (12·7-13·6)
Burundi	9·7 (9·0-10·5)	9·7 (9·0-10·6)	9·7 (8·9-10·7)	9·4 (8·6-10·6)	9·5 (8·7-10·7)	9·3 (8·6-10·3)	10·1 (9·3-11·1)	11·2 (10·4-12·1)	12·1 (10·8-12·9)	12·3 (11·0-13·3)
Comoros	11·9 (11·1-12·7)	12·1 (11·3-12·9)	12·2 (11·4-12·9)	12·3 (11·7-13·1)	12·6 (12·1-13·3)	12·8 (12·3-13·3)	13·1 (12·8-13·7)	13·7 (13·2-14·5)	14·0 (13·4-14·7)	14·3 (13·4-15·1)

Appendix Table 14B. Estimates of life expectancy at age 65 by location, males, 1970, 1975, 1980, 1985, 1990, 1995, 2000, 2005, 2010, 2016

Location	1970	1975	1980	1985	1990	1995	2000	2005	2010	2016
Djibouti	13·1 (12·4-13·8)	13·2 (12·6-14·0)	13·1 (12·6-13·8)	13·0 (12·5-13·5)	12·9 (12·6-13·4)	12·9 (12·5-13·4)	13·0 (12·5-13·9)	13·1 (12·4-13·8)	13·4 (12·5-14·1)	13·9 (12·9-15·0)
Eritrea	9·5 (8·8-10·3)	9·6 (9·0-10·3)	9·7 (9·2-10·3)	9·8 (9·3-10·5)	10·6 (10·0-11·3)	11·9 (11·1-12·5)	12·4 (11·5-12·9)	12·6 (11·6-13·2)	12·6 (11·5-13·5)	12·8 (11·7-13·7)
Ethiopia	9·4 (8·5-10·5)	9·3 (8·4-10·3)	9·3 (8·5-10·2)	9·3 (8·7-10·1)	9·8 (9·2-10·5)	10·8 (10·1-11·4)	11·6 (10·9-12·1)	12·3 (11·6-12·7)	13·0 (11·9-13·6)	13·3 (12·0-14·8)
Kenya	13·4 (13·1-13·7)	13·7 (13·4-13·9)	14·0 (13·7-14·2)	14·1 (13·8-14·3)	13·7 (13·4-14·1)	13·1 (12·8-13·5)	12·8 (12·4-13·1)	13·0 (12·7-13·3)	13·7 (13·4-14·0)	14·1 (13·8-14·5)
Madagascar	11·3 (10·5-11·8)	11·9 (11·2-12·5)	12·4 (11·9-13·0)	12·2 (11·9-12·8)	12·1 (11·8-12·4)	12·3 (12·0-12·7)	12·5 (12·1-12·9)	12·7 (12·1-13·3)	12·9 (11·9-13·8)	13·0 (11·5-14·3)
Malawi	12·7 (12·1-13·4)	12·8 (12·2-13·5)	13·0 (12·4-13·6)	13·2 (12·4-14·2)	13·3 (11·8-16·2)	12·2 (10·9-14·0)	11·3 (10·1-13·0)	11·4 (10·2-13·2)	12·1 (10·8-13·1)	12·9 (11·4-14·1)
Mozambique	11·9 (11·5-12·4)	12·2 (11·6-12·6)	12·2 (11·7-12·6)	11·6 (11·1-12·1)	11·9 (11·5-12·3)	12·0 (11·5-12·7)	11·8 (11·1-12·9)	12·0 (11·0-12·9)	12·2 (11·0-13·1)	13·0 (11·8-14·0)
Rwanda	11·4 (10·5-12·3)	11·5 (10·6-12·4)	11·8 (11·1-12·6)	11·8 (11·2-12·5)	10·7 (9·8-11·6)	9·1 (8·6-9·8)	10·8 (10·0-11·5)	12·8 (12·4-13·6)	13·8 (13·4-14·3)	14·3 (13·2-15·2)
Somalia	11·4 (10·7-12·1)	11·1 (10·5-11·7)	11·6 (11·0-12·2)	11·6 (10·9-12·2)	11·6 (10·9-12·2)	11·5 (10·8-12·1)	11·4 (10·6-12·1)	11·6 (10·7-12·3)	11·5 (10·5-12·2)	11·9 (10·8-12·7)
South Sudan	12·6 (12·0-13·5)	12·8 (12·1-13·7)	12·9 (12·1-13·7)	12·9 (12·1-13·8)	12·7 (11·8-13·9)	12·6 (11·6-14·0)	12·5 (11·6-13·8)	12·7 (11·9-13·8)	12·9 (12·0-14·1)	12·9 (12·0-13·9)
Tanzania	12·6 (12·1-13·1)	12·8 (12·4-13·5)	13·0 (12·6-13·7)	13·2 (12·7-13·8)	12·7 (12·1-13·5)	12·4 (11·8-13·5)	12·4 (11·9-13·4)	12·7 (12·3-13·5)	13·1 (12·5-13·6)	13·6 (12·4-14·4)
Uganda	11·6 (11·0-12·1)	11·7 (11·3-12·2)	11·6 (11·2-12·1)	11·6 (11·3-12·0)	11·3 (10·6-12·1)	11·0 (10·1-11·7)	11·2 (10·6-11·8)	11·7 (10·8-12·3)	12·3 (11·5-12·9)	12·8 (11·7-13·5)
Zambia	14·0 (12·1-16·4)	14·1 (12·4-16·2)	14·3 (12·9-16·3)	15·0 (13·3-17·3)	14·3 (13·1-15·2)	11·8 (11·0-12·7)	10·2 (9·2-11·5)	9·4 (8·8-10·4)	10·1 (9·4-11·1)	11·0 (9·9-12·9)
Central Sub-Saharan Africa	11·1 (10·8-11·5)	11·3 (10·9-11·6)	11·3 (11·0-11·7)	11·4 (11·0-11·7)	11·3 (11·0-11·7)	11·3 (11·0-11·6)	11·5 (11·2-11·8)	12·0 (11·7-12·3)	12·4 (12·0-12·7)	12·9 (12·4-13·3)
Angola	10·6 (9·5-11·7)	10·8 (9·7-11·8)	10·8 (9·7-11·8)	10·7 (9·6-11·7)	10·8 (9·6-11·9)	11·1 (9·8-12·2)	11·8 (10·5-12·9)	12·4 (11·2-13·6)	13·0 (11·8-14·3)	13·6 (12·0-15·0)
Central African Republic	9·1 (8·5-10·3)	9·2 (8·6-10·2)	9·5 (8·8-10·3)	9·5 (8·9-10·1)	9·2 (8·6-9·8)	8·9 (8·3-10·0)	8·8 (8·1-10·0)	8·9 (8·2-10·2)	9·2 (8·4-10·7)	9·4 (8·6-10·8)
Congo	9·8 (8·8-11·4)	10·1 (9·0-11·7)	10·2 (9·2-11·8)	10·3 (9·4-11·6)	9·9 (9·3-10·9)	10·1 (9·4-11·1)	11·2 (10·2-12·3)	12·8 (12·0-13·2)	13·6 (12·8-14·7)	13·9 (12·4-15·5)
Democratic Republic of the Congo	11·9 (11·6-12·3)	12·0 (11·7-12·4)	12·0 (11·7-12·5)	12·1 (11·8-12·5)	12·1 (11·8-12·5)	11·9 (11·7-12·2)	11·8 (11·5-12·1)	12·2 (11·9-12·4)	12·4 (12·0-12·7)	12·9 (12·4-13·3)
Equatorial Guinea	9·3 (8·5-10·5)	9·4 (8·6-10·6)	9·7 (8·8-11·0)	9·9 (9·0-11·3)	10·0 (9·0-11·3)	10·6 (9·3-11·7)	12·4 (11·0-14·1)	14·0 (12·3-16·3)	14·8 (12·8-17·1)	15·3 (13·3-18·0)
Gabon	10·4 (9·1-11·9)	10·7 (9·3-12·2)	10·9 (9·7-12·4)	10·8 (9·9-12·0)	10·8 (10·1-11·8)	11·0 (10·3-12·2)	12·1 (10·7-13·1)	13·0 (11·7-13·6)	13·6 (12·6-14·6)	14·3 (12·6-16·0)

Appendix Table 14C. Estimates of life expectancy at age 65 by location, females, 1970, 1975, 1980, 1985, 1990, 1995, 2000, 2005, 2010, 2016

Location	1970	1975	1980	1985	1990	1995	2000	2005	2010	2016
Global	14·5 (14·4-14·5)	14·9 (14·8-15·0)	15·3 (15·2-15·4)	15·5 (15·5-15·6)	15·9 (15·8-16·0)	16·1 (16·0-16·2)	16·5 (16·4-16·6)	17·1 (17·0-17·2)	17·8 (17·8-17·9)	18·6 (18·4-18·7)
High SDI	16·0 (15·9-16·1)	16·6 (16·6-16·7)	17·4 (17·3-17·5)	17·9 (17·8-18·0)	18·6 (18·5-18·6)	19·1 (19·0-19·2)	19·7 (19·7-19·8)	20·5 (20·5-20·6)	21·3 (21·2-21·4)	21·7 (21·5-21·8)
High-middle SDI	15·4 (15·2-15·7)	15·7 (15·4-15·9)	15·8 (15·6-16·1)	16·0 (15·7-16·2)	16·3 (16·1-16·6)	16·0 (15·8-16·2)	16·6 (16·3-16·8)	17·1 (16·8-17·3)	18·2 (18·0-18·4)	19·2 (18·3-19·9)
Middle SDI	13·7 (13·5-13·9)	14·1 (13·9-14·3)	14·3 (14·1-14·5)	14·7 (14·5-14·8)	14·9 (14·8-15·1)	15·3 (15·1-15·4)	15·7 (15·6-15·9)	16·4 (16·3-16·5)	17·2 (17·1-17·4)	18·3 (18·1-18·4)
Low-middle SDI	11·4 (11·3-11·6)	11·8 (11·6-12·0)	12·2 (12·1-12·4)	12·5 (12·4-12·7)	12·9 (12·7-13·0)	13·1 (13·0-13·3)	13·5 (13·3-13·7)	14·1 (13·9-14·3)	14·7 (14·5-14·9)	15·4 (15·1-15·6)
Low SDI	11·0 (10·7-11·3)	11·2 (10·9-11·4)	11·4 (11·1-11·6)	11·5 (11·3-11·7)	11·6 (11·4-11·8)	11·6 (11·4-11·8)	11·8 (11·5-12·0)	12·2 (11·9-12·5)	12·6 (12·4-13·0)	13·3 (13·0-13·6)
High-income	16·1 (16·0-16·2)	16·7 (16·6-16·8)	17·5 (17·5-17·6)	18·1 (18·0-18·2)	18·8 (18·7-18·8)	19·3 (19·3-19·4)	19·9 (19·9-20·0)	20·7 (20·7-20·8)	21·4 (21·4-21·5)	21·8 (21·7-21·9)
High-income North America	16·9 (16·9-17·0)	17·8 (17·7-17·9)	18·5 (18·4-18·6)	18·7 (18·6-18·8)	19·1 (19·0-19·2)	19·2 (19·1-19·2)	19·3 (19·2-19·3)	19·9 (19·8-19·9)	20·6 (20·5-20·7)	20·7 (20·6-20·9)
Canada	17·5 (17·3-17·7)	18·0 (17·8-18·2)	18·8 (18·6-19·1)	19·2 (19·0-19·5)	19·7 (19·5-19·9)	19·9 (19·6-20·1)	20·3 (20·1-20·5)	20·9 (20·7-21·1)	21·6 (21·4-21·8)	22·0 (21·7-22·3)
Greenland	11·7 (11·2-12·3)	10·5 (10·1-11·1)	11·0 (10·5-11·5)	10·5 (10·1-11·0)	11·3 (10·7-11·9)	11·3 (10·8-12·0)	11·3 (10·9-11·9)	11·6 (11·1-12·2)	12·9 (12·2-13·6)	14·2 (12·8-15·9)
United States	16·9 (16·8-17·0)	17·8 (17·7-17·9)	18·5 (18·4-18·6)	18·6 (18·6-18·7)	19·0 (19·0-19·1)	19·1 (19·0-19·2)	19·2 (19·1-19·2)	19·8 (19·7-19·8)	20·5 (20·4-20·6)	20·6 (20·4-20·8)
Australasia	16·0 (15·9-16·2)	16·9 (16·7-17·0)	17·8 (17·7-18·0)	18·2 (18·1-18·4)	18·9 (18·8-19·1)	19·6 (19·5-19·8)	20·5 (20·3-20·7)	21·3 (21·1-21·4)	21·8 (21·7-22·0)	22·1 (21·7-22·6)
Australia	16·0 (15·8-16·2)	16·9 (16·7-17·1)	18·0 (17·8-18·2)	18·4 (18·2-18·5)	19·1 (18·9-19·2)	19·7 (19·6-19·9)	20·6 (20·4-20·8)	21·4 (21·2-21·6)	21·9 (21·8-22·1)	22·2 (21·7-22·7)
New Zealand	16·1 (15·9-16·3)	16·6 (16·4-16·8)	17·1 (16·9-17·4)	17·6 (17·3-17·8)	18·4 (18·2-18·7)	19·1 (18·8-19·3)	19·9 (19·7-20·2)	20·5 (20·2-20·7)	21·1 (20·9-21·3)	21·5 (20·7-22·4)
High-income Asia Pacific	15·4 (14·9-15·9)	16·2 (15·9-16·6)	17·4 (17·3-17·6)	18·5 (18·4-18·7)	19·5 (19·3-19·6)	20·4 (20·3-20·6)	21·6 (21·4-21·7)	22·6 (22·4-22·7)	23·3 (23·2-23·5)	23·8 (23·3-24·2)
Brunei	13·6 (12·9-15·1)	14·6 (14·0-15·9)	14·7 (13·6-15·4)	16·1 (15·3-16·6)	16·8 (16·0-17·5)	17·3 (16·4-18·0)	18·3 (17·9-18·8)	18·7 (18·2-19·0)	18·9 (18·4-19·2)	18·9 (18·2-20·2)
Japan	15·6 (15·3-15·8)	16·6 (16·4-16·8)	17·8 (17·7-17·8)	19·0 (18·9-19·0)	20·0 (19·9-20·0)	21·0 (20·9-21·0)	22·2 (22·1-22·2)	23·1 (23·0-23·1)	23·7 (23·7-23·8)	24·2 (24·1-24·4)
Singapore	14·4 (13·5-15·4)	15·0 (14·2-16·0)	15·3 (14·4-16·2)	16·2 (15·2-17·2)	17·6 (16·5-18·8)	18·6 (17·5-19·8)	19·9 (18·8-21·0)	21·2 (20·1-22·2)	22·6 (21·7-23·6)	23·3 (21·6-25·2)
South Korea	14·7 (12·2-18·0)	14·4 (12·6-16·4)	15·6 (14·8-16·5)	16·2 (15·2-17·1)	16·7 (15·8-17·6)	17·4 (16·5-18·2)	18·2 (17·5-19·0)	19·8 (19·0-20·6)	21·1 (20·4-21·9)	21·7 (19·4-23·9)
Western Europe	15·8 (15·7-15·9)	16·3 (16·2-16·4)	17·1 (17·0-17·2)	17·7 (17·6-17·8)	18·5 (18·4-18·6)	19·2 (19·0-19·3)	19·9 (19·7-19·9)	20·6 (20·5-20·7)	21·3 (21·2-21·4)	21·8 (21·6-22·0)
Andorra	19·7 (17·1-22·5)	19·9 (17·9-22·7)	20·4 (18·7-22·6)	20·5 (18·9-22·7)	20·8 (19·1-22·8)	21·3 (19·4-23·0)	22·5 (20·5-23·9)	23·1 (21·5-24·4)	23·1 (21·2-24·4)	23·1 (20·9-24·5)
Austria	15·0 (14·8-15·1)	15·6 (15·5-15·8)	16·2 (16·1-16·4)	16·9 (16·8-17·1)	17·9 (17·7-18·1)	18·6 (18·5-18·8)	19·5 (19·3-19·7)	20·3 (20·1-20·5)	21·0 (20·8-21·2)	21·5 (21·0-21·9)
Belgium	15·2 (15·0-15·5)	15·8 (15·5-16·1)	16·7 (16·4-17·0)	17·4 (17·2-17·7)	18·5 (18·2-18·7)	19·1 (18·8-19·3)	19·6 (19·3-19·9)	20·3 (20·1-20·6)	20·9 (20·6-21·2)	21·4 (20·6-22·2)
Cyprus	13·6 (13·3-13·9)	13·3 (12·9-13·7)	13·9 (13·5-14·2)	14·9 (14·6-15·2)	15·7 (15·3-16·0)	16·7 (16·4-17·0)	17·8 (17·4-18·1)	18·7 (18·3-19·0)	19·9 (19·5-20·3)	20·2 (19·7-20·6)
Denmark	16·6 (16·1-17·0)	17·2 (16·7-17·8)	17·6 (17·2-18·1)	17·8 (17·3-18·3)	18·0 (17·4-18·5)	17·8 (17·3-18·3)	18·3 (17·7-18·8)	19·0 (18·4-19·5)	19·8 (19·2-20·3)	20·8 (19·8-21·8)
Finland	14·5 (14·3-14·7)	15·8 (15·5-16·0)	16·9 (16·6-17·1)	17·4 (17·2-17·6)	17·7 (17·5-18·0)	18·6 (18·4-18·9)	19·4 (19·2-19·6)	20·6 (20·4-20·8)	21·2 (20·9-21·4)	22·0 (21·4-22·6)
France	16·8 (16·6-17·0)	17·5 (17·3-17·6)	18·4 (18·2-18·6)	19·1 (18·9-19·3)	20·1 (19·9-20·3)	20·8 (20·6-21·0)	21·3 (21·0-21·4)	22·0 (21·8-22·2)	22·8 (22·6-22·9)	23·2 (22·8-23·7)
Germany	14·9 (14·6-15·3)	15·5 (15·1-15·8)	16·3 (15·9-16·6)	16·9 (16·6-17·3)	17·7 (17·3-18·1)	18·5 (18·1-18·8)	19·3 (18·9-19·7)	20·1 (19·7-20·4)	20·6 (20·2-21·0)	21·1 (20·5-21·8)
Greece	16·6 (16·4-16·9)	17·2 (16·9-17·4)	19·1 (18·8-19·4)	17·8 (17·6-18·1)	18·0 (17·8-18·3)	18·7 (18·5-19·0)	19·3 (19·1-19·5)	20·1 (19·8-20·3)	20·7 (20·5-20·9)	21·0 (20·4-21·5)
Iceland	17·1 (16·7-17·5)	18·1 (17·7-18·5)	18·9 (18·5-19·4)	19·0 (18·6-19·5)	19·1 (18·7-19·5)	19·3 (18·9-19·7)	20·0 (19·6-20·4)	20·7 (20·2-21·1)	21·2 (20·8-21·6)	21·3 (20·5-22·1)
Ireland	15·1 (14·7-15·5)	15·3 (14·9-15·7)	15·8 (15·4-16·2)	16·4 (15·9-16·9)	16·9 (16·5-17·3)	17·4 (16·9-17·8)	18·0 (17·6-18·4)	19·5 (19·1-19·9)	20·5 (20·1-20·9)	21·0 (20·0-22·1)
Israel	14·5 (13·6-15·5)	15·1 (14·3-16·0)	15·7 (14·8-16·6)	16·1 (15·3-16·9)	16·8 (16·0-17·6)	17·6 (16·9-18·4)	19·0 (18·2-19·8)	19·8 (19·0-20·6)	21·1 (20·3-21·9)	21·5 (20·0-23·0)
Italy	16·0 (15·8-16·2)	16·4 (16·2-16·6)	17·2 (17·0-17·4)	17·9 (17·8-18·1)	18·9 (18·7-19·1)	19·7 (19·5-19·9)	20·5 (20·3-20·7)	21·2 (21·1-21·4)	21·7 (21·5-21·9)	21·9 (21·4-22·5)
Luxembourg	15·0 (14·7-15·4)	15·3 (15·0-15·7)	16·3 (16·0-16·6)	17·1 (16·7-17·4)	17·8 (17·4-18·1)	18·9 (18·5-19·2)	19·9 (19·5-20·2)	20·6 (20·2-20·9)	21·3 (20·9-21·7)	21·5 (20·6-22·5)
Malta	13·1 (12·4-13·8)	13·4 (12·8-14·1)	13·8 (13·1-14·5)	15·1 (14·4-15·8)	16·6 (15·7-17·4)	17·7 (16·9-18·5)	18·0 (17·2-18·7)	19·3 (18·5-20·0)	20·4 (19·6-21·1)	21·3 (19·9-22·9)
Netherlands	16·5 (16·2-16·9)	17·3 (17·0-17·7)	18·3 (18·0-18·7)	18·7 (18·4-19·0)	19·0 (18·6-19·3)	19·0 (18·7-19·3)	19·2 (18·9-19·5)	20·0 (19·6-20·3)	20·7 (20·4-21·1)	21·4 (20·7-22·1)
Norway	16·7 (16·4-17·0)	17·3 (17·0-17·7)	18·0 (17·7-18·4)	18·5 (18·2-18·8)	18·8 (18·5-19·2)	19·2 (18·9-19·5)	19·7 (19·4-20·0)	20·5 (20·1-20·9)	21·0 (20·6-21·3)	21·6 (20·7-22·5)
Portugal	14·9 (14·7-15·1)	15·5 (15·3-15·8)	16·3 (16·1-16·5)	17·1 (16·9-17·3)	17·6 (17·4-17·8)	18·2 (18·0-18·5)	19·0 (18·7-19·2)	19·9 (19·7-20·2)	20·9 (20·6-21·1)	21·5 (21·0-22·1)
Spain	16·0 (15·9-16·2)	16·6 (16·5-16·8)	17·8 (17·7-18·0)	18·5 (18·3-18·6)	19·3 (19·1-19·4)	20·0 (19·9-20·2)	20·7 (20·5-20·8)	21·3 (21·2-21·5)	22·3 (22·2-22·5)	22·8 (22·5-23·2)
Sweden	16·8 (16·3-17·3)	17·4 (16·9-17·8)	18·0 (17·7-18·2)	18·6 (18·4-18·9)	19·0 (18·8-19·3)	19·7 (19·4-19·9)	20·0 (19·7-20·2)	20·4 (20·2-20·7)	21·1 (20·8-21·3)	21·4 (20·4-22·3)
Stockholm	16·2 (14·6-18·1)	17·1 (15·8-18·5)	18·3 (17·8-18·9)	19·0 (18·6-19·4)	19·1 (18·7-19·5)	19·7 (19·3-20·0)	19·9 (19·5-20·3)	20·5 (20·2-21·0)	21·2 (20·7-21·6)	21·6 (20·0-23·3)
Sweden except Stockholm	16·9 (16·4-17·4)	17·4 (17·0-17·9)	17·9 (17·6-18·2)	18·5 (18·2-18·8)	19·0 (18·7-19·3)	19·7 (19·3-19·9)	20·0 (19·7-20·3)	20·4 (20·1-20·7)	21·0 (20·7-21·3)	21·3 (20·2-22·4)
Switzerland	16·2 (15·7-16·6)	17·4 (16·9-17·9)	18·1 (17·6-18·6)	19·0 (18·6-19·5)	19·6 (19·1-20·0)	20·1 (19·6-20·5)	20·7 (20·2-21·2)	21·5 (21·0-21·9)	21·9 (21·5-22·4)	22·5 (20·5-24·4)
United Kingdom	16·0 (15·8-16·1)	16·3 (16·2-16·4)	16·8 (16·7-16·9)	17·2 (17·1-17·3)	17·8 (17·7-17·9)	18·2 (18·1-18·3)	18·9 (18·8-19·0)	19·7 (19·6-19·8)	20·6 (20·5-20·7)	20·9 (20·7-21·0)
England	16·1 (16·0-16·2)	16·4 (16·3-16·5)	16·9 (16·8-17·0)	17·3 (17·3-17·4)	17·9 (17·9-18·0)	18·3 (18·3-18·4)	19·0 (19·0-19·1)	19·8 (19·7-19·9)	20·7 (20·7-20·8)	21·1 (20·9-21·2)
East Midlands	15·9 (15·4-16·4)	16·3 (15·9-16·7)	16·8 (16·5-17·1)	17·3 (17·1-17·6)	17·8 (17·6-18·1)	18·3 (18·0-18·5)	18·9 (18·7-19·2)	19·7 (19·4-19·9)	20·6 (20·4-20·8)	20·8 (20·5-21·2)
East of England	16·4 (15·9-16·9)	16·8 (16·4-17·2)	17·3 (17·0-17·6)	17·7 (17·5-17·9)	18·3 (18·1-18·6)	18·8 (18·6-19·1)	19·4 (19·2-19·6)	20·2 (20·0-20·5)	21·1 (20·9-21·3)	21·4 (21·0-21·7)
Greater London	16·7 (16·4-16·9)	16·9 (16·6-17·1)	17·4 (17·2-17·5)	17·7 (17·6-17·9)	18·3 (18·1-18·4)	18·6 (18·4-18·7)	19·2 (19·1-19·4)	20·1 (19·9-20·2)	21·2 (21·0-21·3)	21·6 (21·4-21·8)
North East England	15·1 (14·6-15·5)	15·5 (15·1-15·9)	16·1 (15·8-16·3)	16·2 (16·0-16·5)	16·9 (16·7-17·1)	17·1 (16·9-17·3)	18·1 (17·9-18·3)	18·7 (18·4-18·9)	19·7 (19·5-19·9)	20·0 (19·7-20·4)
North West England	15·3 (15·0-15·7)	15·7 (15·4-16·0)	16·2 (16·0-16·4)	16·6 (16·4-16·7)	17·1 (16·9-17·3)	17·5 (17·3-17·7)	18·3 (18·1-18·5)	19·0 (18·9-19·2)	19·9 (19·7-20·1)	20·2 (20·0-20·5)
South East England	16·7 (16·3-17·1)	17·0 (16·7-17·4)	17·5 (17·3-17·8)	17·9 (17·7-18·1)	18·5 (18·3-18·7)	18·8 (18·7-19·0)	19·6 (19·4-19·7)	20·4 (20·2-20·6)	21·3 (21·1-21·5)	21·6 (21·3-21·9)
South West England	16·7 (16·3-17·1)	17·0 (16·7-17·4)	17·5 (17·2-17·7)	18·0 (17·8-18·2)	18·7 (18·5-18·9)	19·1 (19·0-19·3)	19·7 (19·5-19·9)	20·5 (20·3-20·7)	21·4 (21·2-21·6)	21·6 (21·3-21·9)
West Midlands	15·7 (15·3-16·2)	16·1 (15·7-16·6)	16·6 (16·3-16·9)	17·0 (16·8-17·2)	17·7 (17·5-17·9)	18·1 (17·9-18·3)	18·8 (18·5-19·0)	19·6 (19·4-19·8)	20·6 (20·4-20·8)	21·0 (20·6-21·3)
Yorkshire and the Humber	15·7 (15·3-16·1)	16·0 (15·6-16·4)	16·5 (16·2-16·8)	17·0 (16·8-17·2)	17·6 (17·4-17·8)	17·9 (17·8-18·1)	18·8 (18·6-19·0)	19·4 (19·3-19·6)	20·3 (20·1-20·4)	20·6 (20·3-20·9)
Northern Ireland	15·3 (14·9-15·8)	15·6 (15·1-16·1)	16·2 (15·7-16·7)	16·6 (15·9-17·2)	17·4 (16·8-18·1)	17·9 (17·3-18·7)	18·6 (17·9-19·3)	19·5 (18·9-20·1)	20·3 (19·8-21·0)	20·6 (19·5-21·8)
Scotland	15·3 (14·9-15·7)	15·6 (15·2-16·0)	16·0 (15·6-16·5)	16·3 (15·7-16·9)	16·7 (16·1-17·4)	17·2 (16·6-17·8)	17·9 (17·2-18·5)	18·5 (17·9-19·2)	19·3 (18·8-19·9)	19·7 (18·7-20·7)

Appendix Table 14C. Estimates of life expectancy at age 65 by location, females, 1970, 1975, 1980, 1985, 1990, 1995, 2000, 2005, 2010, 2016

Location	1970	1975	1980	1985	1990	1995	2000	2005	2010	2016
Wales	15·8 (14·4-17·1)	16·1 (14·9-17·3)	16·7 (15·8-17·5)	17·3 (16·6-17·9)	17·7 (17·1-18·3)	18·0 (17·4-18·5)	18·6 (18·0-19·2)	19·5 (18·9-20·1)	20·1 (19·5-20·7)	20·4 (19·4-21·4)
Southern Latin America	16·0 (15·7-16·3)	16·5 (16·1-16·9)	16·9 (16·7-17·2)	17·2 (17·0-17·5)	17·7 (17·5-18·0)	18·5 (18·2-18·7)	18·9 (18·6-19·1)	19·2 (19·0-19·5)	19·6 (19·4-19·9)	20·2 (19·5-20·9)
Argentina	15·9 (15·5-16·3)	16·4 (15·8-17·0)	16·9 (16·6-17·2)	17·3 (17·0-17·6)	17·8 (17·5-18·1)	18·5 (18·1-18·8)	18·7 (18·4-19·0)	18·9 (18·7-19·3)	19·2 (18·9-19·5)	19·7 (19·1-20·4)
Chile	15·9 (15·4-16·4)	16·7 (16·2-17·3)	16·9 (16·4-17·5)	17·1 (16·6-17·6)	17·7 (17·2-18·3)	18·5 (18·0-19·1)	19·4 (19·0-19·9)	20·1 (19·5-20·6)	20·9 (20·4-21·4)	21·6 (19·4-23·9)
Uruguay	16·4 (16·1-16·7)	16·6 (16·4-16·9)	16·9 (16·7-17·2)	17·2 (17·0-17·5)	17·7 (17·4-18·0)	18·3 (18·1-18·6)	19·0 (18·8-19·2)	19·4 (19·1-19·6)	19·9 (19·6-20·2)	20·4 (19·9-21·0)
Central Europe, Eastern Europe, and Central Asi	15·4 (15·1-15·7)	15·6 (15·3-15·8)	15·5 (15·2-15·8)	15·4 (15·1-15·7)	15·8 (15·5-16·1)	15·1 (14·8-15·4)	15·5 (15·3-15·8)	15·9 (15·6-16·2)	17·0 (16·7-17·3)	17·8 (16·5-18·9)
Eastern Europe	15·9 (15·4-16·3)	15·8 (15·3-16·2)	15·6 (15·1-16·1)	15·4 (14·9-15·9)	15·8 (15·4-16·3)	14·7 (14·3-15·2)	15·1 (14·7-15·6)	15·4 (14·9-15·9)	16·6 (16·1-17·1)	17·4 (15·5-19·3)
Belarus	17·3 (16·9-17·7)	17·5 (17·2-17·9)	17·1 (16·7-17·4)	16·5 (16·2-16·9)	16·5 (16·1-16·9)	15·6 (15·2-16·0)	15·7 (15·4-16·1)	16·0 (15·7-16·4)	16·8 (16·5-17·1)	18·1 (16·7-19·7)
Estonia	15·6 (15·3-16·0)	15·8 (15·5-16·1)	15·7 (15·4-16·0)	15·6 (15·3-15·9)	16·1 (15·8-16·4)	16·0 (15·6-16·3)	16·9 (16·5-17·2)	18·0 (17·7-18·4)	19·6 (19·2-20·0)	20·1 (19·4-21·4)
Latvia	15·8 (15·4-16·2)	15·9 (15·5-16·4)	15·8 (15·5-16·2)	15·5 (15·2-15·9)	15·9 (15·6-16·3)	15·7 (15·3-16·1)	16·9 (16·6-17·3)	17·1 (16·7-17·5)	18·2 (17·8-18·6)	19·0 (18·0-20·1)
Lithuania	16·6 (16·3-16·9)	17·0 (16·7-17·4)	17·1 (16·8-17·3)	16·7 (16·4-17·0)	17·1 (16·8-17·4)	16·6 (16·2-16·9)	17·9 (17·6-18·1)	18·0 (17·7-18·3)	18·8 (18·5-19·1)	19·6 (19·0-20·3)
Moldova	13·4 (11·7-15·2)	13·9 (12·5-15·5)	14·3 (13·3-15·3)	13·8 (13·0-14·6)	14·4 (13·8-15·1)	13·0 (12·5-13·7)	14·4 (13·8-15·1)	14·5 (13·9-15·1)	15·2 (14·5-15·9)	16·3 (15·3-17·5)
Russia	15·7 (15·1-16·5)	15·6 (14·9-16·3)	15·5 (14·8-16·2)	15·3 (14·6-15·9)	15·8 (15·1-16·6)	14·7 (14·0-15·4)	15·0 (14·4-15·7)	15·3 (14·6-16·1)	16·6 (15·9-17·3)	17·3 (14·5-20·1)
Ukraine	16·0 (15·5-16·4)	15·9 (15·5-16·3)	15·7 (15·3-16·1)	15·4 (15·0-15·9)	15·8 (15·4-16·2)	14·6 (14·2-15·1)	15·2 (14·8-15·6)	15·3 (14·9-15·7)	16·3 (15·9-16·7)	17·2 (15·2-19·5)
Central Europe	14·8 (14·6-14·9)	15·2 (15·0-15·3)	15·2 (15·0-15·3)	15·3 (15·2-15·5)	15·7 (15·6-15·9)	16·0 (15·8-16·1)	16·6 (16·5-16·7)	17·3 (17·2-17·5)	18·1 (18·0-18·3)	18·9 (18·6-19·2)
Albania	17·5 (16·8-18·3)	17·9 (17·0-18·8)	18·2 (17·5-19·1)	18·3 (17·9-18·8)	18·3 (18·1-18·5)	18·6 (18·4-18·9)	18·0 (17·7-18·2)	17·6 (17·4-17·8)	18·4 (17·9-18·9)	19·6 (18·6-20·6)
Bosnia and Herzegovina	14·5 (12·8-16·1)	14·7 (13·5-16·4)	14·9 (13·7-16·4)	15·0 (13·9-16·3)	15·5 (14·6-16·6)	15·5 (14·6-16·6)	17·0 (15·9-18·1)	17·2 (16·3-18·3)	17·8 (16·9-18·8)	18·6 (17·2-19·9)
Bulgaria	14·7 (14·5-15·0)	14·7 (14·4-14·9)	14·8 (14·5-15·0)	14·9 (14·6-15·1)	15·2 (14·9-15·5)	15·3 (15·1-15·6)	15·4 (15·2-15·7)	16·1 (15·9-16·4)	17·0 (16·8-17·3)	17·6 (16·2-18·9)
Croatia	14·1 (12·2-16·2)	14·7 (12·7-16·8)	15·2 (13·3-17·1)	15·4 (14·6-16·2)	16·0 (15·3-16·6)	16·3 (15·7-16·9)	16·8 (16·2-17·3)	17·4 (16·8-17·9)	18·1 (17·6-18·6)	18·6 (17·5-19·8)
Czech Republic	14·3 (14·1-14·5)	14·6 (14·4-14·8)	14·6 (14·4-14·8)	14·8 (14·6-14·9)	15·3 (15·2-15·5)	16·2 (16·0-16·4)	17·1 (16·9-17·3)	17·8 (17·7-18·0)	18·7 (18·5-18·9)	19·7 (19·2-20·1)
Hungary	14·4 (14·0-14·9)	14·7 (14·4-15·1)	14·7 (14·3-15·1)	15·0 (14·6-15·4)	15·4 (15·0-15·9)	15·9 (15·5-16·3)	16·6 (16·2-17·0)	17·3 (16·9-17·8)	17·9 (17·6-18·3)	18·3 (17·5-19·2)
Macedonia	15·3 (14·8-15·8)	15·2 (14·6-15·8)	14·9 (14·4-15·4)	14·8 (14·4-15·4)	14·5 (14·0-15·1)	14·3 (13·8-14·7)	14·5 (14·1-14·9)	14·8 (14·4-15·3)	15·4 (15·0-15·9)	16·3 (15·8-16·8)
Montenegro	17·1 (16·4-18·1)	17·0 (16·0-18·3)	17·6 (16·7-18·5)	17·7 (16·4-18·3)	18·0 (17·2-18·7)	17·1 (16·5-17·8)	17·2 (16·7-17·7)	17·6 (17·2-18·1)	18·3 (17·5-19·0)	18·5 (17·6-19·7)
Poland	15·3 (15·0-15·6)	15·7 (15·4-16·0)	15·8 (15·5-16·0)	15·7 (15·4-16·0)	16·1 (15·8-16·4)	16·5 (16·2-16·8)	17·4 (17·1-17·7)	18·5 (18·2-18·8)	19·3 (19·0-19·6)	20·1 (19·3-20·9)
Romania	14·5 (14·1-14·8)	14·9 (14·5-15·3)	14·7 (14·3-15·0)	15·0 (14·6-15·3)	15·4 (15·0-15·8)	15·2 (14·8-15·6)	16·1 (15·7-16·5)	16·6 (16·3-17·0)	17·4 (17·0-17·8)	18·1 (17·3-18·9)
Serbia	15·3 (14·7-16·2)	15·9 (15·3-17·0)	16·2 (15·5-17·3)	16·4 (15·7-17·3)	16·1 (15·5-16·9)	15·3 (14·9-15·9)	15·2 (14·8-15·7)	15·6 (15·2-16·1)	16·6 (16·2-17·0)	17·9 (17·5-18·2)
Slovakia	14·6 (14·3-15·0)	15·2 (14·9-15·5)	15·3 (15·0-15·6)	15·3 (15·0-15·7)	15·6 (15·3-16·0)	16·2 (15·8-16·5)	16·7 (16·3-17·0)	17·2 (16·9-17·5)	18·0 (17·6-18·3)	18·8 (17·8-19·8)
Slovenia	14·4 (12·3-16·9)	14·9 (13·2-16·9)	15·4 (14·2-16·7)	15·9 (15·2-16·5)	17·0 (16·3-17·7)	17·5 (16·9-18·2)	18·5 (18·0-19·1)	19·4 (18·7-20·1)	20·4 (19·9-21·1)	21·3 (20·3-22·4)
Central Asia	14·9 (14·0-15·9)	15·4 (14·4-16·3)	15·9 (15·4-16·4)	16·0 (15·6-16·3)	15·9 (15·5-16·1)	14·8 (14·5-15·1)	14·9 (14·7-15·2)	14·9 (14·6-15·2)	15·6 (15·4-15·9)	16·8 (16·3-17·3)
Armenia	16·8 (15·4-18·7)	17·2 (15·9-18·9)	17·4 (16·5-18·3)	17·0 (16·5-17·5)	16·4 (15·9-16·8)	16·0 (15·6-16·5)	16·6 (16·1-17·1)	16·6 (16·0-17·1)	16·6 (15·9-17·1)	18·3 (17·4-19·3)
Azerbaijan	15·4 (14·1-17·0)	15·9 (14·7-17·3)	16·2 (15·2-17·3)	15·9 (15·3-16·6)	15·9 (15·4-16·5)	15·0 (14·5-15·6)	14·7 (14·1-15·3)	14·7 (13·9-15·5)	14·8 (14·0-15·6)	17·3 (15·7-18·6)
Georgia	16·4 (14·8-18·5)	16·8 (15·1-19·0)	17·1 (16·1-18·3)	16·4 (15·8-17·2)	16·3 (15·6-16·9)	16·7 (16·1-17·3)	17·0 (16·3-17·7)	17·2 (16·7-17·7)	18·1 (17·6-18·4)	18·5 (17·1-20·3)
Kazakhstan	14·4 (11·9-17·4)	14·8 (12·5-17·9)	15·5 (14·1-17·1)	15·9 (15·0-16·9)	15·9 (15·1-16·8)	14·4 (13·6-15·2)	14·8 (13·9-15·6)	14·7 (13·9-15·7)	15·6 (14·9-16·3)	16·9 (15·5-18·4)
Kyrgyzstan	14·9 (13·1-16·7)	15·5 (13·8-17·3)	16·3 (15·6-17·1)	15·9 (15·6-16·3)	15·8 (15·5-16·2)	14·4 (14·1-14·7)	15·2 (14·9-15·6)	14·6 (14·2-14·9)	15·4 (15·1-15·8)	16·6 (15·8-17·4)
Mongolia	11·4 (10·6-12·4)	13·1 (12·0-14·4)	13·3 (12·2-14·6)	13·8 (12·8-14·9)	14·2 (13·3-15·2)	13·0 (12·4-13·7)	13·0 (12·4-13·6)	13·6 (13·0-14·2)	14·2 (13·5-15·0)	15·6 (14·6-16·7)
Tajikistan	14·3 (13·7-15·1)	14·7 (14·0-15·6)	15·2 (14·6-16·1)	16·0 (15·2-17·0)	15·9 (15·1-16·7)	15·1 (14·3-16·0)	14·4 (13·5-15·3)	14·1 (13·2-14·9)	15·6 (14·6-16·5)	16·9 (15·7-17·9)
Turkmenistan	14·4 (13·9-15·0)	14·8 (14·3-15·3)	15·2 (14·8-15·5)	15·3 (15·1-15·6)	15·1 (14·8-15·4)	14·6 (14·3-14·8)	15·0 (14·7-15·3)	15·5 (15·2-15·8)	16·2 (15·8-16·4)	16·9 (16·3-17·5)
Uzbekistan	14·8 (13·5-15·8)	15·2 (13·7-16·4)	15·7 (14·8-16·6)	16·0 (15·4-16·6)	15·7 (15·2-16·2)	14·3 (13·8-14·8)	14·0 (13·6-14·5)	13·9 (13·4-14·4)	15·0 (14·7-15·5)	15·8 (14·9-16·7)
Latin America and Caribbean	15·1 (15·0-15·3)	15·9 (15·8-16·1)	16·5 (16·4-16·6)	16·5 (16·4-16·6)	17·1 (17·0-17·2)	17·4 (17·3-17·5)	18·2 (18·0-18·3)	18·6 (18·5-18·7)	18·9 (18·8-19·1)	19·5 (19·3-19·7)
Central Latin America	15·6 (15·4-15·8)	16·2 (16·0-16·4)	17·0 (16·8-17·1)	17·1 (17·0-17·3)	18·0 (17·8-18·1)	18·2 (18·1-18·3)	18·9 (18·8-19·1)	19·1 (18·9-19·2)	19·4 (19·2-19·5)	19·8 (19·5-20·1)
Colombia	17·5 (17·2-17·7)	16·6 (16·4-16·9)	16·8 (16·5-17·2)	16·8 (16·5-17·1)	17·9 (17·6-18·1)	18·0 (17·7-18·2)	18·4 (18·1-18·6)	18·6 (18·4-18·9)	19·3 (19·0-19·5)	20·2 (19·5-21·0)
Costa Rica	15·7 (15·4-16·1)	16·6 (16·3-17·0)	17·9 (17·5-18·2)	18·4 (18·1-18·8)	18·9 (18·5-19·2)	19·3 (19·0-19·6)	20·0 (19·7-20·3)	21·0 (20·7-21·4)	21·9 (21·6-22·3)	22·3 (21·6-22·9)
El Salvador	15·4 (15·0-15·8)	16·1 (15·7-16·5)	17·2 (16·7-17·6)	17·1 (16·7-17·6)	17·5 (17·1-17·9)	17·7 (17·3-18·0)	18·6 (18·3-19·0)	18·7 (18·3-19·0)	19·4 (19·0-19·8)	19·6 (18·6-20·5)
Guatemala	13·1 (12·0-14·3)	14·5 (13·4-15·8)	15·3 (14·1-16·5)	14·9 (14·0-16·0)	15·5 (14·5-16·4)	16·4 (15·4-17·5)	17·4 (16·2-18·7)	17·9 (16·8-19·1)	18·3 (17·0-19·6)	18·8 (16·6-21·2)
Honduras	14·0 (13·0-15·2)	15·1 (13·9-16·2)	15·2 (14·1-16·2)	13·7 (13·0-14·6)	14·0 (13·3-14·8)	14·4 (13·5-15·3)	14·8 (13·4-16·3)	15·1 (13·6-17·3)	15·6 (13·9-18·3)	16·0 (14·3-18·6)
Mexico	15·4 (15·1-15·8)	16·3 (16·0-16·6)	17·3 (17·1-17·5)	17·6 (17·4-17·7)	18·5 (18·3-18·6)	18·7 (18·5-18·8)	19·4 (19·3-19·6)	19·3 (19·1-19·4)	19·3 (19·2-19·5)	19·5 (19·2-19·7)
Nicaragua	15·7 (15·1-16·3)	18·3 (17·9-18·7)	18·2 (17·4-19·1)	19·0 (18·1-20·0)	21·0 (20·6-21·3)	20·3 (19·9-20·7)	20·5 (20·2-20·9)	20·5 (20·2-20·9)	20·5 (20·1-20·8)	21·0 (19·6-22·4)
Panama	16·2 (15·7-16·9)	17·6 (17·0-18·1)	18·7 (18·1-19·3)	18·5 (17·9-19·1)	18·9 (18·3-19·5)	19·4 (18·8-20·0)	20·3 (19·7-20·8)	20·6 (20·0-21·2)	21·1 (20·6-21·7)	21·7 (20·6-22·8)
Venezuela	14·9 (14·4-15·4)	15·1 (14·6-15·6)	15·9 (15·4-16·4)	16·7 (16·1-17·2)	17·3 (16·8-17·9)	17·8 (17·1-18·6)	18·9 (18·3-19·4)	19·5 (19·0-20·1)	20·0 (19·4-20·6)	20·2 (18·7-21·6)
Andean Latin America	14·6 (14·0-15·3)	16·0 (15·6-16·4)	16·3 (15·9-16·7)	16·5 (16·1-16·9)	17·2 (16·8-17·7)	17·4 (17·0-17·8)	18·7 (18·3-19·0)	19·5 (19·1-19·9)	19·7 (19·1-20·1)	20·1 (19·2-21·1)
Bolivia	12·9 (11·9-13·8)	13·2 (12·2-14·0)	13·4 (12·4-14·2)	13·4 (12·4-14·4)	13·9 (12·9-14·7)	14·5 (13·6-15·4)	15·9 (15·3-16·5)	16·6 (15·7-17·3)	16·7 (14·9-18·0)	16·9 (15·0-18·6)
Ecuador	18·1 (17·7-18·5)	17·4 (17·1-17·8)	17·6 (17·2-18·0)	17·9 (17·6-18·3)	18·3 (17·9-18·6)	18·7 (18·4-19·1)	19·5 (19·1-19·8)	20·2 (19·8-20·5)	20·4 (20·0-20·7)	20·8 (20·2-21·4)
Peru	13·7 (12·7-14·9)	16·4 (15·8-17·1)	16·8 (16·2-17·4)	17·0 (16·5-17·6)	18·2 (17·6-18·8)	18·0 (17·3-18·5)	19·4 (18·8-20·0)	20·3 (19·6-21·1)	20·5 (19·8-21·2)	21·1 (19·4-22·8)
Caribbean	15·1 (14·7-15·6)	15·6 (15·2-16·1)	16·2 (15·8-16·6)	16·4 (16·1-16·7)	16·5 (16·3-16·8)	16·8 (16·6-17·1)	17·5 (17·2-17·8)	17·9 (17·5-18·3)	17·1 (16·2-18·1)	18·6 (18·0-19·2)
Antigua and Barbuda	13·0 (12·4-13·7)	13·9 (13·2-14·5)	15·0 (14·3-15·9)	15·5 (14·6-16·4)	17·1 (16·3-17·9)	18·3 (17·6-19·0)	18·6 (17·9-19·3)	18·9 (18·2-19·6)	19·6 (18·7-20·4)	19·7 (18·7-20·9)
The Bahamas	14·0 (12·8-14·6)	14·9 (14·2-15·4)	15·6 (15·3-15·9)	16·3 (15·8-16·7)	16·9 (16·6-17·3)	17·0 (16·7-17·4)	17·1 (16·7-17·4)	17·6 (17·3-18·1)	17·9 (17·3-18·5)	17·6 (16·6-18·8)

Appendix Table 14C. Estimates of life expectancy at age 65 by location, females, 1970, 1975, 1980, 1985, 1990, 1995, 2000, 2005, 2010, 2016

Location	1970	1975	1980	1985	1990	1995	2000	2005	2010	2016
Barbados	15·3 (14·8-15·8)	15·8 (15·4-16·4)	16·7 (16·2-17·2)	17·1 (16·6-17·6)	17·2 (16·7-17·7)	17·5 (16·9-18·1)	18·7 (18·1-19·3)	19·3 (18·7-19·8)	19·9 (19·4-20·4)	19·4 (18·6-20·2)
Belize	16·2 (15·7-17·0)	16·6 (16·0-17·4)	16·6 (15·7-17·7)	17·0 (15·9-17·9)	17·2 (16·3-18·1)	17·2 (16·7-17·9)	15·8 (15·4-16·2)	16·0 (15·6-16·3)	16·6 (16·1-17·2)	16·6 (15·6-17·7)
Bermuda	12·4 (11·9-13·0)	13·3 (12·8-13·8)	13·6 (13·0-14·2)	14·1 (13·4-14·7)	13·9 (13·3-14·6)	14·4 (13·7-15·0)	14·8 (14·3-15·4)	16·5 (15·8-17·1)	18·3 (17·6-19·1)	20·4 (19·1-22·1)
Cuba	16·6 (16·3-16·8)	17·4 (17·1-17·7)	18·2 (17·8-18·5)	18·4 (18·0-18·8)	18·2 (17·8-18·5)	18·3 (17·9-18·7)	19·0 (18·6-19·4)	19·4 (19·0-19·7)	19·7 (19·4-20·1)	20·2 (19·5-21·0)
Dominica	13·6 (13·1-14·1)	14·3 (13·8-14·8)	15·6 (15·0-16·2)	15·5 (14·9-16·1)	14·9 (14·4-15·5)	14·9 (14·4-15·5)	16·2 (15·6-17·1)	16·7 (15·9-17·3)	16·9 (16·2-17·5)	17·3 (16·0-18·2)
Dominican Republic	16·4 (15·8-17·4)	17·2 (16·4-18·2)	17·6 (16·7-18·6)	17·2 (16·4-18·3)	17·4 (16·9-18·1)	18·0 (17·4-18·8)	19·0 (18·4-20·2)	19·4 (18·4-20·7)	19·6 (18·4-21·1)	19·6 (18·5-21·2)
Grenada	14·9 (13·8-16·2)	15·9 (14·7-17·1)	15·9 (14·8-17·3)	15·4 (14·6-16·6)	15·6 (15·0-16·2)	16·0 (15·4-16·5)	16·1 (15·5-16·8)	16·0 (15·3-16·7)	16·1 (15·4-16·8)	16·3 (15·2-17·6)
Guyana	13·3 (12·8-14·1)	13·5 (13·0-14·3)	14·1 (13·5-14·8)	14·8 (14·1-15·5)	14·7 (14·2-15·3)	15·0 (14·6-15·4)	15·7 (15·1-16·2)	14·4 (13·9-15·0)	13·9 (13·4-14·5)	15·2 (14·3-16·2)
Haiti	10·5 (9·3-12·7)	10·6 (9·4-12·5)	10·7 (9·6-12·0)	10·7 (9·9-11·6)	10·8 (10·2-11·4)	11·3 (10·7-11·9)	11·9 (11·1-12·6)	12·2 (11·2-13·4)	9·3 (7·4-12·2)	13·0 (11·7-14·5)
Jamaica	16·0 (15·4-16·7)	16·8 (16·0-17·6)	16·8 (15·9-17·7)	17·1 (16·4-17·8)	17·8 (17·0-18·6)	17·5 (16·4-18·8)	17·7 (17·1-18·5)	18·0 (17·4-18·8)	17·9 (17·1-18·8)	17·9 (16·5-19·4)
Puerto Rico	17·6 (17·2-18·0)	17·5 (17·1-17·9)	18·4 (18·0-18·8)	18·4 (18·0-18·8)	18·9 (18·5-19·3)	19·2 (18·8-19·6)	20·0 (19·5-20·4)	20·4 (20·0-20·8)	20·9 (20·5-21·3)	21·1 (20·3-22·0)
Saint Lucia	14·8 (14·2-15·4)	14·0 (13·7-14·3)	14·6 (14·3-15·0)	15·5 (15·2-15·9)	17·1 (16·7-17·4)	18·0 (17·7-18·4)	18·7 (18·3-19·1)	18·8 (18·3-19·2)	20·0 (19·6-20·5)	20·0 (19·5-20·6)
Saint Vincent and the Grenadines	15·6 (14·3-16·3)	15·2 (14·4-16·2)	15·3 (14·7-16·2)	15·5 (15·0-16·1)	15·7 (15·1-16·1)	15·7 (15·3-16·1)	15·8 (15·4-16·1)	16·0 (15·6-16·4)	16·2 (15·7-16·8)	16·4 (15·7-17·3)
Suriname	13·7 (13·1-14·6)	14·1 (13·6-14·8)	14·7 (14·4-15·1)	16·0 (15·7-16·4)	17·0 (16·6-17·4)	16·9 (16·5-17·3)	16·3 (15·8-16·7)	16·2 (15·6-16·6)	16·8 (16·2-17·2)	17·1 (16·4-17·7)
Trinidad and Tobago	13·2 (12·9-13·6)	14·0 (13·6-14·4)	14·9 (14·5-15·2)	15·3 (14·9-15·7)	15·4 (15·1-15·8)	15·9 (15·5-16·2)	16·3 (15·9-16·7)	17·4 (17·1-17·8)	18·4 (17·9-18·9)	18·7 (18·1-19·4)
Virgin Islands, U.S.	14·4 (13·6-15·0)	15·2 (14·5-15·6)	15·8 (15·4-16·2)	16·5 (16·1-17·2)	17·0 (16·6-18·1)	17·2 (16·7-17·9)	17·6 (17·2-18·3)	17·9 (17·5-18·3)	18·1 (17·7-18·6)	18·5 (17·9-19·1)
Tropical Latin America	14·9 (14·6-15·3)	15·8 (15·5-16·1)	16·1 (15·9-16·2)	15·8 (15·7-16·0)	16·4 (16·2-16·6)	16·8 (16·6-17·0)	17·4 (17·2-17·6)	18·1 (18·0-18·3)	18·8 (18·6-18·9)	19·3 (19·0-19·6)
Brazil	14·9 (14·5-15·2)	15·7 (15·5-16·1)	16·0 (15·8-16·2)	15·8 (15·6-16·0)	16·4 (16·2-16·5)	16·7 (16·6-16·9)	17·4 (17·2-17·6)	18·1 (18·0-18·3)	18·8 (18·6-19·0)	19·3 (19·1-19·6)
Paraguay	16·9 (16·6-17·1)	17·2 (17·0-17·5)	17·9 (17·4-18·2)	17·9 (17·5-18·3)	18·5 (17·9-18·9)	18·2 (17·4-18·6)	18·3 (17·7-18·7)	17·9 (17·1-18·5)	17·6 (17·1-18·4)	17·9 (17·0-18·9)
Southeast Asia, East Asia, and Oceania	13·5 (13·3-13·7)	13·9 (13·7-14·1)	13·9 (13·7-14·1)	14·4 (14·2-14·6)	14·5 (14·4-14·7)	14·8 (14·7-15·0)	15·4 (15·2-15·5)	16·2 (16·1-16·3)	17·3 (17·1-17·4)	18·5 (18·3-18·6)
East Asia	13·7 (13·5-14·0)	14·1 (13·9-14·4)	14·0 (13·8-14·2)	14·4 (14·2-14·7)	14·5 (14·3-14·7)	14·9 (14·7-15·1)	15·5 (15·3-15·6)	16·4 (16·3-16·6)	17·6 (17·5-17·8)	19·0 (18·8-19·2)
China	13·7 (13·5-14·0)	14·1 (13·8-14·4)	13·9 (13·7-14·2)	14·4 (14·2-14·6)	14·4 (14·2-14·7)	14·8 (14·6-15·0)	15·4 (15·3-15·6)	16·4 (16·2-16·6)	17·6 (17·5-17·8)	19·0 (18·8-19·2)
North Korea	15·1 (13·9-16·0)	15·7 (14·6-16·7)	15·9 (15·0-16·9)	16·0 (15·2-17·2)	16·2 (15·4-17·3)	14·6 (13·4-16·1)	14·2 (13·1-15·6)	15·6 (14·9-16·5)	15·7 (15·1-16·4)	15·9 (15·4-16·5)
Taiwan (Province of China)	14·2 (13·8-14·5)	15·0 (14·6-15·3)	15·3 (15·0-15·7)	15·8 (15·5-16·2)	16·6 (16·3-17·0)	17·4 (17·0-17·7)	18·4 (18·1-18·8)	19·5 (19·2-19·9)	20·6 (20·2-21·0)	21·0 (20·0-22·2)
Southeast Asia	12·7 (12·4-13·1)	13·1 (12·9-13·5)	13·7 (13·5-14·1)	14·3 (14·0-14·6)	14·6 (14·3-14·9)	14·9 (14·6-15·2)	15·1 (14·9-15·4)	15·6 (15·4-15·9)	16·3 (16·1-16·5)	17·1 (16·9-17·4)
Cambodia	11·6 (10·8-12·4)	9·9 (8·6-11·5)	11·6 (11·0-12·4)	11·7 (11·1-12·3)	11·9 (11·5-12·4)	12·2 (11·8-12·6)	12·6 (12·2-13·0)	13·3 (13·1-13·6)	14·2 (13·9-14·6)	15·1 (14·6-15·6)
Indonesia	11·6 (11·2-12·0)	12·2 (11·8-12·5)	12·9 (12·5-13·2)	13·5 (13·1-13·8)	13·7 (13·4-14·0)	13·9 (13·6-14·2)	14·1 (13·8-14·3)	14·5 (14·2-14·7)	15·0 (14·7-15·3)	15·8 (15·5-16·1)
Laos	10·4 (10·1-10·8)	10·7 (10·3-11·1)	10·9 (10·6-11·3)	11·4 (11·0-11·7)	11·7 (11·4-12·2)	12·1 (11·8-12·6)	12·7 (12·3-13·2)	13·5 (13·0-14·0)	14·3 (13·7-14·8)	15·2 (14·6-15·9)
Malaysia	13·4 (13·2-13·7)	14·1 (13·8-14·3)	14·4 (14·1-14·8)	14·8 (14·6-15·1)	14·9 (14·7-15·1)	15·0 (14·8-15·2)	15·2 (15·0-15·4)	15·3 (15·1-15·4)	15·7 (15·4-15·9)	16·9 (16·5-17·2)
Maldives	11·8 (9·7-13·8)	11·6 (9·6-13·7)	11·5 (9·9-13·3)	12·0 (11·3-12·7)	13·0 (12·4-13·7)	13·8 (12·9-14·7)	14·8 (14·1-15·6)	16·6 (15·8-17·3)	18·0 (17·2-18·8)	19·4 (17·4-21·5)
Mauritius	12·2 (11·8-12·5)	12·8 (12·5-13·1)	13·8 (13·5-14·2)	14·2 (13·9-14·5)	15·1 (14·7-15·4)	15·8 (15·4-16·1)	16·0 (15·7-16·4)	16·6 (16·2-16·9)	17·6 (17·2-17·9)	18·0 (16·8-19·1)
Myanmar	10·6 (10·2-11·2)	11·2 (10·8-11·8)	11·6 (11·2-12·1)	12·0 (11·5-12·5)	12·2 (11·8-12·7)	12·4 (12·0-12·9)	12·9 (12·5-13·3)	13·6 (13·2-14·2)	14·6 (14·1-15·2)	15·8 (15·4-16·3)
Philippines	16·8 (16·3-17·2)	16·3 (15·9-16·7)	16·3 (15·9-16·8)	16·1 (15·7-16·5)	16·2 (15·8-16·6)	16·0 (15·6-16·4)	15·6 (15·1-16·0)	15·3 (14·9-15·7)	15·5 (15·1-16·0)	16·0 (14·9-17·1)
Sri Lanka	13·7 (13·3-14·2)	14·0 (13·5-14·4)	15·4 (14·9-15·9)	16·2 (15·8-16·7)	17·5 (17·0-18·1)	17·7 (17·0-18·3)	17·7 (17·2-18·3)	18·4 (17·6-19·0)	18·4 (17·5-19·3)	19·7 (17·9-21·7)
Seychelles	15·0 (13·8-16·0)	15·8 (14·7-16·5)	16·3 (15·5-17·3)	15·5 (15·2-15·9)	16·1 (15·7-16·5)	16·8 (16·3-17·5)	16·8 (16·4-17·2)	16·9 (16·5-17·5)	17·6 (17·0-18·0)	17·7 (17·0-18·5)
Thailand	16·0 (15·6-16·4)	16·8 (16·5-17·2)	16·9 (16·5-17·2)	16·7 (16·4-17·2)	17·1 (16·7-17·4)	17·4 (17·0-17·8)	17·9 (17·5-18·4)	18·4 (18·1-18·8)	19·6 (19·2-20·0)	20·3 (19·5-21·1)
Timor-Leste	10·3 (9·0-12·2)	10·7 (9·3-12·6)	11·5 (10·0-13·3)	12·1 (10·6-13·8)	12·8 (11·4-14·3)	13·4 (12·2-14·6)	14·0 (12·9-15·1)	14·7 (13·5-16·0)	16·2 (14·6-17·3)	16·5 (14·8-18·1)
Vietnam	12·8 (11·8-14·4)	13·2 (12·4-15·0)	13·8 (13·0-15·4)	14·6 (13·6-16·0)	15·2 (14·2-16·5)	15·7 (14·7-17·2)	16·1 (15·3-17·8)	16·5 (15·8-18·2)	17·3 (16·4-18·4)	18·1 (17·3-18·5)
Oceania	11·1 (10·6-11·9)	11·3 (10·7-12·0)	11·4 (10·7-12·1)	11·3 (10·7-12·0)	11·3 (10·7-12·0)	11·4 (10·8-12·0)	11·4 (10·8-12·1)	11·4 (10·7-12·1)	11·5 (10·8-12·2)	12·0 (11·2-12·8)
American Samoa	14·2 (13·6-14·8)	15·2 (14·5-15·9)	15·5 (14·5-16·3)	15·6 (14·8-16·4)	16·4 (15·9-17·1)	16·1 (15·1-16·8)	16·1 (15·3-16·8)	16·2 (15·1-16·9)	16·3 (15·3-17·2)	16·0 (14·6-17·5)
Federated States of Micronesia	12·0 (11·0-13·0)	12·1 (11·2-13·0)	12·4 (11·5-13·3)	12·5 (11·7-13·4)	12·6 (11·7-13·6)	12·8 (11·8-13·8)	13·0 (11·9-14·1)	12·8 (11·4-14·1)	12·7 (11·3-14·1)	12·7 (11·4-14·2)
Fiji	13·4 (12·8-14·5)	13·5 (12·8-14·3)	13·7 (12·9-14·5)	13·9 (13·0-14·9)	13·4 (11·5-15·5)	13·1 (11·7-14·7)	12·9 (11·9-14·0)	13·2 (12·2-14·2)	13·2 (11·9-14·4)	13·5 (11·5-15·7)
Guam	14·6 (14·0-15·2)	15·1 (14·6-15·7)	16·5 (16·0-17·1)	17·3 (16·5-17·9)	17·1 (16·6-17·9)	17·1 (16·7-17·9)	17·5 (16·9-18·1)	17·4 (16·8-18·0)	16·9 (16·3-17·5)	17·1 (16·2-18·1)
Kiribati	11·7 (10·6-13·2)	12·2 (11·1-13·4)	12·2 (11·3-13·2)	11·8 (10·9-12·5)	11·6 (11·1-12·2)	11·8 (11·4-12·2)	12·1 (11·6-12·6)	12·4 (11·7-13·1)	12·6 (11·7-13·5)	12·8 (11·8-13·9)
Marshall Islands	13·0 (11·6-14·8)	13·5 (12·3-15·1)	13·9 (13·0-15·3)	14·0 (13·3-15·0)	13·6 (13·1-14·1)	13·1 (12·5-13·7)	12·5 (11·8-13·2)	12·1 (11·4-13·0)	12·2 (11·2-13·3)	12·7 (11·4-14·1)
Northern Mariana Islands	14·0 (12·4-15·7)	14·7 (13·0-16·5)	15·2 (13·5-16·9)	15·5 (13·8-17·1)	16·0 (14·5-17·6)	17·0 (15·5-18·4)	17·5 (16·1-18·8)	17·6 (16·2-18·9)	17·5 (16·1-18·9)	17·4 (15·9-19·0)
Papua New Guinea	10·5 (9·8-11·6)	10·6 (9·9-11·6)	10·6 (9·8-11·5)	10·4 (9·7-11·3)	10·4 (9·7-11·2)	10·5 (9·8-11·4)	10·6 (9·8-11·5)	10·5 (9·6-11·4)	10·7 (9·8-11·7)	11·3 (10·3-12·2)
Samoa	13·8 (13·3-14·6)	13·9 (13·4-14·5)	14·6 (14·0-15·3)	14·9 (14·2-15·9)	15·2 (14·3-16·2)	15·4 (14·4-16·5)	15·6 (14·5-16·7)	15·8 (14·8-16·9)	15·7 (14·8-16·8)	15·9 (14·8-17·1)
Solomon Islands	10·4 (9·6-11·4)	10·5 (9·7-11·4)	10·9 (10·0-11·9)	11·0 (10·1-12·0)	10·9 (10·1-11·9)	11·1 (10·2-12·0)	11·1 (10·3-12·0)	10·9 (10·0-11·9)	11·0 (10·0-12·1)	11·5 (10·3-12·6)
Tonga	13·2 (12·1-14·3)	13·4 (12·3-14·5)	13·3 (12·1-14·5)	13·7 (12·4-14·9)	14·2 (13·0-15·5)	14·5 (13·4-15·7)	14·7 (13·7-15·8)	15·2 (14·3-16·3)	15·5 (14·4-16·6)	15·7 (14·4-17·0)
Vanuatu	11·0 (10·1-12·0)	11·3 (10·5-12·2)	11·6 (10·7-12·5)	11·7 (10·8-12·6)	11·7 (10·9-12·7)	11·8 (10·9-12·7)	11·8 (10·9-12·7)	11·8 (10·7-12·8)	12·0 (10·8-13·1)	12·3 (11·0-13·4)
North Africa and Middle East	14·7 (14·3-15·0)	15·1 (14·8-15·4)	15·6 (15·3-15·8)	15·7 (15·5-16·0)	16·1 (15·9-16·3)	16·0 (15·8-16·2)	16·5 (16·3-16·7)	16·7 (16·3-16·9)	17·0 (16·7-17·4)	17·7 (17·3-18·2)
North Africa and Middle East	14·7 (14·3-15·0)	15·1 (14·8-15·4)	15·6 (15·3-15·8)	15·7 (15·5-16·0)	16·1 (15·9-16·3)	16·0 (15·8-16·2)	16·5 (16·3-16·7)	16·7 (16·3-16·9)	17·0 (16·7-17·4)	17·7 (17·3-18·2)
Afghanistan	9·9 (9·4-10·6)	9·8 (9·2-10·3)	9·6 (9·1-10·0)	9·6 (9·1-10·2)	10·0 (9·5-10·6)	9·7 (9·2-10·2)	9·6 (9·1-10·1)	9·8 (9·3-10·4)	10·2 (9·6-10·7)	10·7 (10·2-11·3)
Algeria	14·6 (14·1-15·2)	14·5 (13·7-15·0)	14·5 (13·8-15·1)	16·6 (16·1-17·2)	16·9 (16·1-17·5)	17·2 (16·4-17·9)	17·7 (16·8-18·3)	18·1 (17·1-18·6)	18·5 (18·1-18·6)	18·6 (18·1-19·0)
Bahrain	14·9 (13·2-16·3)	14·7 (13·0-16·6)	15·5 (14·0-17·0)	14·6 (13·5-15·8)	13·1 (12·0-14·2)	12·7 (11·6-13·9)	13·8 (12·7-14·8)	14·4 (13·4-15·6)	15·6 (14·7-16·8)	16·6 (15·2-18·3)

Appendix Table 14C. Estimates of life expectancy at age 65 by location, females, 1970, 1975, 1980, 1985, 1990, 1995, 2000, 2005, 2010, 2016

Location	1970	1975	1980	1985	1990	1995	2000	2005	2010	2016
Egypt	14·1 (13·8-14·5)	14·9 (14·5-15·3)	15·3 (14·9-15·8)	14·4 (14·0-14·9)	15·5 (15·1-15·9)	15·8 (15·4-16·3)	16·6 (16·1-17·1)	16·2 (15·7-16·8)	15·9 (15·3-16·4)	16·7 (15·5-18·0)
Iran	17·0 (15·6-18·5)	17·7 (16·5-19·0)	18·0 (16·9-19·2)	18·1 (17·0-19·4)	17·1 (16·2-18·3)	17·2 (16·3-18·3)	16·9 (15·9-17·8)	16·2 (14·6-17·6)	16·8 (15·2-18·5)	18·1 (16·9-19·6)
Iraq	14·8 (14·1-15·2)	14·3 (13·2-15·2)	14·1 (12·9-15·8)	13·5 (12·7-15·3)	13·7 (12·8-14·8)	13·4 (12·7-14·0)	13·6 (12·9-14·1)	13·7 (12·8-14·5)	14·2 (13·0-15·2)	14·9 (13·4-16·4)
Jordan	14·9 (13·7-15·8)	15·4 (14·2-16·3)	14·9 (13·5-16·4)	14·8 (14·0-16·1)	15·8 (14·7-17·3)	16·1 (14·8-17·6)	15·9 (14·5-17·5)	15·2 (13·7-17·1)	17·6 (15·8-19·4)	18·4 (16·5-20·4)
Kuwait	16·2 (15·3-17·2)	16·0 (15·1-17·1)	15·6 (14·7-16·8)	15·6 (14·5-16·7)	16·7 (15·4-18·2)	16·2 (15·2-17·3)	16·1 (15·1-17·2)	16·1 (15·0-17·3)	16·1 (15·0-17·1)	17·3 (15·0-20·0)
Lebanon	15·1 (13·1-16·9)	15·0 (13·1-16·8)	14·6 (13·1-16·5)	15·5 (14·2-17·3)	15·9 (14·8-17·4)	16·3 (15·4-17·9)	17·8 (16·5-18·5)	18·5 (18·2-19·3)	19·6 (18·7-20·4)	20·1 (19·0-21·2)
Libya	16·9 (16·1-17·9)	17·3 (16·6-18·2)	17·6 (16·9-18·5)	17·6 (17·1-18·4)	17·5 (16·6-18·0)	17·5 (16·5-18·2)	17·4 (16·2-18·2)	17·7 (16·4-18·4)	18·2 (17·2-18·5)	18·1 (17·3-18·7)
Morocco	14·0 (13·5-14·4)	14·3 (13·8-14·6)	14·6 (14·1-15·0)	15·3 (14·7-15·7)	15·5 (14·7-16·1)	15·7 (14·8-16·3)	16·3 (15·5-16·8)	16·5 (15·6-17·3)	17·0 (16·0-18·0)	17·5 (16·6-18·4)
Palestine	14·8 (12·7-16·1)	15·3 (13·3-16·9)	15·7 (14·1-17·1)	15·3 (13·6-16·6)	15·0 (13·7-16·3)	14·6 (13·7-15·5)	14·3 (14·0-14·6)	14·8 (14·6-14·9)	14·2 (14·0-14·4)	13·6 (13·2-14·1)
Oman	14·2 (13·7-14·9)	14·8 (14·3-15·4)	15·3 (14·9-15·9)	16·1 (15·6-16·5)	16·3 (15·4-17·1)	16·2 (15·5-17·4)	17·0 (16·5-17·9)	18·0 (17·6-18·7)	17·6 (16·8-18·4)	19·0 (18·6-19·5)
Qatar	16·4 (14·1-19·0)	16·9 (14·5-19·5)	17·0 (14·8-18·9)	16·3 (15·0-18·0)	16·6 (15·5-18·3)	16·6 (15·6-18·2)	17·2 (16·1-18·4)	18·3 (17·4-19·1)	19·6 (18·3-21·2)	20·3 (18·4-22·9)
Saudi Arabia	17·7 (16·7-18·8)	18·2 (17·1-19·2)	17·9 (16·9-18·9)	17·0 (16·1-17·8)	16·0 (15·2-16·8)	15·0 (14·3-15·7)	13·6 (13·2-14·1)	14·0 (13·7-14·4)	14·8 (14·3-15·2)	16·8 (16·1-17·5)
Sudan	12·8 (12·3-13·2)	12·7 (12·2-13·1)	13·0 (12·5-13·5)	12·5 (12·0-13·1)	13·2 (12·6-13·7)	13·2 (12·7-13·8)	13·6 (13·1-14·2)	14·3 (13·8-14·9)	15·2 (14·6-15·7)	15·9 (15·1-16·5)
Syria	16·2 (15·6-16·8)	16·6 (16·3-17·0)	16·7 (16·4-17·0)	16·4 (15·9-17·0)	16·4 (15·6-17·1)	16·0 (15·4-16·7)	16·5 (16·2-16·7)	17·2 (16·7-17·7)	18·1 (17·7-18·6)	18·1 (17·6-18·9)
Tunisia	13·9 (13·3-14·4)	14·6 (14·2-15·1)	15·2 (14·6-15·9)	16·6 (16·0-17·3)	17·1 (16·5-17·8)	17·3 (16·5-18·0)	18·4 (17·8-19·2)	18·7 (18·1-19·8)	19·0 (18·3-20·5)	19·4 (18·3-21·0)
Turkey	16·2 (14·6-18·0)	16·8 (15·7-18·1)	17·7 (16·8-18·7)	18·4 (17·6-19·3)	18·6 (18·0-19·2)	18·2 (17·7-18·7)	19·3 (18·8-19·8)	20·2 (19·6-20·9)	20·7 (19·9-21·5)	21·1 (19·6-22·6)
United Arab Emirates	15·8 (14·0-17·7)	16·1 (14·0-17·7)	16·2 (14·1-18·0)	16·0 (14·5-18·0)	15·9 (14·7-17·6)	16·5 (15·3-17·7)	17·3 (16·2-18·2)	18·3 (17·6-18·7)	18·3 (17·5-19·1)	18·4 (17·1-19·4)
Yemen	12·0 (11·3-12·6)	12·5 (11·7-13·2)	12·7 (12·0-13·5)	12·7 (12·0-13·5)	12·6 (11·9-13·5)	12·6 (11·9-13·4)	12·8 (12·3-13·6)	13·3 (12·8-13·9)	13·9 (13·4-14·5)	14·1 (13·6-14·5)
South Asia	10·9 (10·7-11·1)	11·3 (11·1-11·6)	11·8 (11·6-12·0)	12·2 (12·0-12·4)	12·7 (12·5-12·9)	13·2 (13·0-13·4)	13·7 (13·4-13·9)	14·1 (13·9-14·4)	14·6 (14·4-14·8)	15·2 (14·9-15·5)
South Asia	10·9 (10·7-11·1)	11·3 (11·1-11·6)	11·8 (11·6-12·0)	12·2 (12·0-12·4)	12·7 (12·5-12·9)	13·2 (13·0-13·4)	13·7 (13·4-13·9)	14·1 (13·9-14·4)	14·6 (14·4-14·8)	15·2 (14·9-15·5)
Bangladesh	11·8 (10·5-13·0)	12·5 (11·4-13·7)	12·5 (11·7-13·4)	12·5 (11·9-13·2)	12·9 (12·1-13·6)	13·8 (13·2-14·5)	15·2 (14·7-15·9)	16·1 (15·4-16·5)	16·8 (16·1-17·2)	17·6 (16·6-18·3)
Bhutan	12·2 (10·8-13·5)	12·4 (11·0-13·7)	12·5 (11·1-13·8)	12·7 (11·5-14·0)	13·2 (12·0-14·4)	13·8 (12·7-14·9)	15·3 (14·3-16·2)	16·7 (15·8-17·6)	17·8 (16·6-18·7)	18·1 (16·9-19·0)
India	10·5 (10·3-10·8)	11·0 (10·8-11·2)	11·5 (11·3-11·8)	12·0 (11·8-12·2)	12·6 (12·3-12·8)	13·1 (12·9-13·4)	13·5 (13·3-13·8)	14·0 (13·8-14·3)	14·5 (14·3-14·7)	15·0 (14·8-15·3)
Nepal	12·3 (11·6-12·9)	12·2 (11·6-12·9)	12·2 (11·6-12·9)	12·3 (11·7-12·8)	12·4 (11·9-12·9)	12·8 (12·3-13·3)	13·7 (13·2-14·3)	14·0 (13·5-14·6)	14·4 (14·0-14·9)	15·2 (14·8-15·7)
Pakistan	14·1 (12·9-14·9)	14·3 (13·3-15·1)	14·6 (13·8-15·2)	14·6 (14·0-15·0)	14·2 (13·7-14·7)	13·3 (12·7-13·9)	13·3 (12·7-14·0)	13·5 (12·8-14·3)	14·2 (13·2-15·4)	14·8 (13·6-16·3)
Sub-Saharan Africa	12·1 (11·9-12·3)	12·4 (12·2-12·6)	12·6 (12·4-12·8)	12·7 (12·5-12·9)	12·7 (12·6-12·9)	12·7 (12·5-12·9)	12·5 (12·3-12·7)	13·0 (12·7-13·2)	13·6 (13·4-13·9)	14·4 (14·1-14·7)
Southern Sub-Saharan Africa	14·8 (14·2-15·5)	15·5 (15·0-16·1)	15·8 (15·3-16·3)	16·5 (16·1-16·8)	17·3 (16·9-17·8)	17·3 (16·7-17·9)	14·5 (14·0-15·0)	14·1 (13·7-14·6)	14·6 (14·2-15·2)	16·7 (16·3-17·2)
Botswana	11·0 (10·5-11·5)	11·8 (11·4-12·4)	12·5 (12·1-13·0)	13·3 (12·9-13·7)	14·2 (13·3-15·7)	14·3 (10·5-20·2)	12·2 (8·1-16·4)	12·7 (8·9-15·8)	15·1 (11·0-19·3)	15·8 (12·7-23·0)
Lesotho	11·7 (10·3-13·8)	12·2 (10·6-14·2)	12·8 (11·2-14·7)	13·2 (12·0-14·9)	13·5 (12·5-14·9)	13·5 (12·4-15·8)	11·8 (10·4-14·8)	10·9 (9·6-12·7)	11·2 (9·8-13·1)	12·1 (10·1-15·7)
Namibia	12·0 (11·3-13·0)	12·2 (11·5-13·0)	12·5 (11·9-13·2)	12·9 (12·5-13·5)	13·2 (12·8-13·8)	13·2 (12·2-15·0)	12·5 (10·6-16·4)	13·5 (11·3-15·8)	15·6 (13·4-18·5)	16·8 (14·4-21·5)
South Africa	16·1 (15·2-17·0)	16·7 (16·0-17·6)	16·9 (16·3-17·6)	17·6 (17·1-18·0)	18·4 (18·0-18·8)	18·3 (18·0-18·6)	15·3 (15·1-15·5)	14·9 (14·7-15·1)	15·2 (15·0-15·4)	17·6 (17·3-18·1)
Swaziland	11·9 (10·6-13·5)	12·4 (11·0-14·1)	12·7 (11·4-14·2)	12·8 (11·7-14·4)	13·2 (12·3-14·3)	14·3 (12·2-17·2)	12·7 (9·6-15·2)	11·2 (8·6-12·6)	12·6 (10·1-14·8)	14·7 (11·7-19·1)
Zimbabwe	11·7 (10·7-12·8)	12·2 (11·2-13·1)	12·4 (11·7-13·2)	13·2 (12·7-13·9)	14·1 (13·1-16·5)	14·4 (12·1-17·2)	12·1 (9·9-14·3)	11·3 (9·6-13·5)	12·0 (10·4-15·9)	13·2 (11·8-15·6)
Western Sub-Saharan Africa	12·9 (12·4-13·2)	13·1 (12·6-13·4)	13·3 (12·9-13·6)	13·4 (13·0-13·7)	13·3 (12·9-13·6)	13·2 (12·8-13·6)	13·4 (12·9-13·9)	13·8 (13·4-14·4)	14·4 (14·0-15·0)	15·0 (14·5-15·6)
Benin	12·9 (12·3-13·4)	13·0 (12·4-13·6)	13·0 (12·4-13·6)	13·4 (12·8-13·9)	13·8 (13·3-14·3)	13·9 (13·2-14·4)	14·0 (13·2-14·7)	14·2 (13·3-15·0)	14·2 (13·4-15·2)	14·3 (13·4-15·4)
Burkina Faso	12·2 (12·0-12·5)	12·3 (12·1-12·6)	12·4 (12·1-12·7)	12·5 (12·2-12·8)	12·4 (11·9-13·2)	12·5 (11·8-13·6)	12·7 (12·0-13·5)	13·0 (12·4-13·5)	13·1 (12·7-13·5)	13·3 (12·9-13·8)
Cameroon	11·6 (10·7-12·7)	11·8 (11·0-12·9)	12·2 (11·4-13·1)	12·9 (12·1-13·7)	13·4 (12·6-14·3)	13·2 (12·1-14·5)	12·6 (11·4-14·1)	12·9 (11·5-14·5)	13·5 (12·0-15·2)	14·0 (12·4-16·1)
Cape Verde	15·0 (13·9-16·5)	15·4 (14·4-16·6)	15·6 (14·8-16·4)	16·2 (15·9-16·7)	16·8 (16·4-17·5)	17·1 (16·2-18·3)	17·8 (16·5-19·4)	18·4 (17·2-19·7)	18·8 (18·3-19·8)	18·8 (18·1-20·1)
Chad	13·5 (13·0-13·9)	13·5 (12·9-13·9)	13·4 (12·8-13·8)	13·3 (12·7-13·7)	13·1 (12·6-13·7)	13·1 (12·5-13·7)	13·0 (12·3-13·9)	13·4 (12·5-14·4)	13·7 (12·8-14·6)	14·3 (13·2-15·2)
Cote d'Ivoire	12·6 (11·8-13·3)	12·7 (11·9-13·4)	12·8 (12·1-13·5)	12·8 (12·2-13·5)	12·5 (11·9-13·2)	12·2 (11·4-13·1)	12·0 (11·0-13·4)	12·1 (11·2-13·5)	12·6 (11·8-13·9)	13·0 (12·2-14·0)
The Gambia	13·0 (11·9-14·1)	13·2 (12·1-14·4)	13·4 (12·2-14·5)	13·4 (12·3-14·5)	13·5 (12·4-14·6)	13·5 (12·6-14·6)	13·8 (12·9-14·9)	14·4 (13·3-15·7)	14·6 (13·6-15·8)	14·9 (14·0-16·3)
Ghana	12·6 (11·5-13·7)	12·8 (11·8-14·0)	12·9 (11·8-14·0)	12·7 (11·8-13·8)	12·7 (11·8-13·8)	13·1 (12·1-14·3)	13·2 (12·1-14·6)	13·5 (12·4-14·9)	13·5 (12·7-14·9)	14·3 (13·6-15·7)
Guinea	12·1 (11·0-13·1)	12·1 (11·0-13·1)	12·3 (11·3-13·1)	12·2 (11·4-13·0)	12·6 (11·8-13·2)	12·6 (11·9-13·2)	12·7 (11·9-13·4)	12·8 (11·9-13·9)	12·9 (11·9-14·1)	13·0 (12·0-14·4)
Guinea-Bissau	11·1 (10·2-12·2)	11·4 (10·5-12·7)	11·4 (10·5-12·6)	11·2 (10·4-12·4)	11·2 (10·4-12·2)	11·2 (10·5-12·1)	11·3 (10·6-12·2)	11·7 (10·8-13·0)	11·9 (11·0-13·0)	12·4 (11·6-13·3)
Liberia	13·2 (12·6-13·6)	13·3 (12·7-13·8)	13·3 (12·7-13·8)	13·2 (12·5-13·7)	12·9 (12·2-13·6)	12·5 (11·7-13·2)	12·7 (11·8-13·7)	12·8 (11·9-14·0)	12·9 (12·3-13·9)	13·4 (12·9-14·0)
Mali	10·7 (9·5-12·1)	10·7 (9·7-11·9)	11·1 (10·2-12·1)	11·4 (10·7-12·3)	11·4 (10·8-12·1)	11·7 (11·1-12·5)	12·3 (11·6-13·0)	13·4 (12·5-14·2)	14·1 (13·0-15·0)	14·3 (12·7-15·7)
Mauritania	11·9 (10·3-14·0)	12·2 (10·5-14·0)	12·4 (11·0-14·0)	12·4 (11·5-13·6)	12·4 (11·7-13·2)	12·6 (12·0-13·5)	13·2 (12·4-14·3)	13·9 (12·9-15·2)	14·6 (13·4-16·2)	15·1 (13·8-17·1)
Niger	12·8 (11·8-13·5)	12·6 (11·8-13·3)	12·4 (11·8-13·1)	12·4 (11·7-13·1)	13·1 (12·5-13·6)	13·0 (12·4-13·5)	13·4 (12·7-13·9)	13·9 (13·0-14·4)	14·1 (13·0-15·0)	14·1 (12·6-15·5)
Nigeria	13·5 (12·5-14·2)	13·8 (12·9-14·6)	14·2 (13·4-15·0)	14·2 (13·5-15·0)	13·9 (13·0-14·5)	13·8 (12·9-14·6)	14·1 (13·0-15·2)	14·8 (13·8-16·3)	16·0 (14·9-17·6)	16·8 (15·6-18·6)
Sao Tome and Principe	14·5 (14·0-14·8)	14·8 (14·3-15·3)	15·2 (14·8-15·6)	15·2 (14·7-15·6)	14·7 (13·7-15·4)	13·7 (12·8-14·9)	13·2 (12·8-13·9)	13·8 (13·4-14·3)	14·5 (14·0-15·0)	15·2 (14·5-16·2)
Senegal	12·8 (12·2-13·4)	12·7 (12·1-13·3)	12·8 (12·1-13·4)	13·2 (12·6-13·8)	13·3 (12·6-13·9)	13·2 (12·5-13·9)	13·1 (12·4-13·9)	13·3 (12·7-14·0)	13·3 (13·0-13·9)	13·9 (13·3-14·3)
Sierra Leone	13·1 (12·4-13·7)	13·3 (12·6-13·9)	13·4 (12·7-13·9)	13·5 (12·8-14·0)	13·6 (13·1-14·1)	13·4 (12·8-13·9)	12·8 (12·2-13·5)	12·4 (11·6-13·2)	12·4 (11·5-13·4)	12·9 (12·0-14·2)
Togo	13·1 (12·3-13·8)	13·2 (12·3-14·0)	13·3 (12·5-14·0)	13·1 (12·3-13·9)	13·0 (12·3-13·8)	13·2 (12·4-14·2)	13·2 (12·1-14·9)	13·5 (12·2-15·1)	13·7 (12·6-15·3)	13·8 (13·1-15·1)
Eastern Sub-Saharan Africa	10·7 (10·4-11·0)	10·9 (10·6-11·3)	11·1 (10·8-11·5)	11·1 (10·9-11·4)	11·1 (10·9-11·4)	11·1 (10·8-11·3)	11·3 (11·0-11·7)	12·1 (11·8-12·6)	13·0 (12·6-13·5)	13·7 (13·2-14·2)
Burundi	9·6 (9·0-10·3)	9·6 (9·0-10·3)	9·6 (8·9-10·3)	9·3 (8·6-10·2)	9·4 (8·7-10·4)	9·4 (8·8-10·2)	9·6 (9·0-10·4)	10·8 (10·0-11·5)	11·7 (10·9-12·6)	12·1 (11·2-13·0)
Comoros	11·3 (10·4-12·6)	11·7 (10·7-13·1)	11·9 (10·9-13·3)	12·0 (11·1-13·3)	12·2 (11·4-13·5)	12·4 (11·7-13·3)	12·7 (12·1-13·6)	13·7 (12·9-14·8)	14·1 (13·2-15·4)	14·3 (13·6-15·9)

Appendix Table 14C. Estimates of life expectancy at age 65 by location, females, 1970, 1975, 1980, 1985, 1990, 1995, 2000, 2005, 2010, 2016

Location	1970	1975	1980	1985	1990	1995	2000	2005	2010	2016
Djibouti	14·7<br>(14·0-15·2)	14·8<br>(14·2-15·4)	14·8<br>(14·0-15·3)	14·7<br>(14·0-15·3)	14·9<br>(14·2-15·3)	14·7<br>(14·0-15·3)	14·7<br>(13·3-15·9)	14·7<br>(12·9-16·4)	14·9<br>(13·2-16·7)	15·3<br>(13·9-17·5)
Eritrea	9·5<br>(9·0-10·1)	9·7<br>(9·1-10·3)	9·9<br>(9·4-10·4)	10·0<br>(9·5-10·5)	10·3<br>(9·8-10·8)	11·1<br>(10·5-11·7)	11·7<br>(11·0-12·4)	12·0<br>(11·3-12·9)	12·1<br>(11·4-13·0)	12·5<br>(11·7-13·4)
Ethiopia	9·0<br>(8·2-9·9)	9·0<br>(8·2-9·9)	9·1<br>(8·4-9·9)	9·2<br>(8·7-9·8)	9·5<br>(9·1-10·1)	9·8<br>(9·3-10·2)	10·2<br>(9·6-10·9)	11·2<br>(10·4-11·9)	12·3<br>(11·4-13·3)	13·3<br>(12·2-14·3)
Kenya	12·5<br>(12·2-12·9)	12·9<br>(12·6-13·3)	13·3<br>(13·0-13·6)	13·5<br>(13·1-13·8)	13·4<br>(13·1-13·7)	13·2<br>(12·7-13·6)	13·3<br>(12·9-13·7)	13·8<br>(13·4-14·2)	14·6<br>(14·2-15·1)	15·4<br>(14·9-15·8)
Madagascar	9·9<br>(9·4-10·5)	10·9<br>(10·3-11·7)	12·1<br>(11·2-13·1)	11·9<br>(11·2-12·8)	11·9<br>(11·3-12·6)	12·0<br>(11·4-12·7)	12·1<br>(11·5-12·8)	12·4<br>(11·6-13·5)	12·7<br>(11·7-14·5)	13·1<br>(11·7-15·5)
Malawi	11·9<br>(11·1-12·6)	12·4<br>(11·7-13·0)	12·8<br>(12·2-13·4)	12·7<br>(11·9-13·3)	12·1<br>(10·9-13·3)	11·2<br>(9·9-13·5)	11·2<br>(9·5-14·0)	12·3<br>(10·1-14·6)	13·4<br>(11·7-15·9)	14·0<br>(12·4-16·4)
Mozambique	11·9<br>(11·1-12·6)	12·2<br>(11·4-12·9)	12·3<br>(11·6-12·9)	11·6<br>(11·0-12·2)	12·7<br>(12·1-13·2)	12·9<br>(12·1-14·0)	13·3<br>(11·8-15·1)	14·4<br>(12·4-16·0)	14·8<br>(12·6-16·4)	14·8<br>(13·2-16·5)
Rwanda	10·5<br>(9·6-11·7)	10·7<br>(9·8-11·9)	10·8<br>(10·1-12·0)	10·9<br>(10·2-11·7)	10·2<br>(9·6-10·8)	9·9<br>(9·3-10·6)	11·1<br>(10·3-11·9)	13·7<br>(12·4-14·9)	14·7<br>(13·5-16·1)	15·0<br>(13·9-16·4)
Somalia	10·2<br>(9·5-10·9)	10·1<br>(9·4-10·8)	10·4<br>(9·7-11·0)	10·2<br>(9·5-10·8)	10·2<br>(9·5-10·9)	10·1<br>(9·5-10·8)	10·1<br>(9·5-10·8)	10·3<br>(9·6-11·0)	10·4<br>(9·7-11·2)	10·8<br>(10·1-11·5)
South Sudan	12·7<br>(11·6-13·5)	12·9<br>(11·8-13·7)	13·0<br>(11·9-13·8)	12·8<br>(11·7-13·7)	12·5<br>(11·4-13·5)	12·2<br>(11·0-13·5)	12·2<br>(11·0-14·0)	12·6<br>(11·3-14·5)	12·9<br>(11·6-14·8)	12·9<br>(11·6-14·7)
Tanzania	12·7<br>(11·7-13·6)	13·1<br>(12·1-13·9)	13·5<br>(12·5-14·2)	13·3<br>(12·5-14·1)	12·5<br>(11·8-13·3)	12·0<br>(11·2-13·4)	12·2<br>(11·2-14·1)	13·0<br>(11·8-15·0)	13·6<br>(12·6-15·8)	14·1<br>(13·3-16·0)
Uganda	10·7<br>(10·3-11·4)	10·9<br>(10·4-11·6)	10·9<br>(10·4-11·5)	11·1<br>(10·6-11·6)	11·0<br>(10·4-11·6)	11·0<br>(10·2-12·4)	11·3<br>(10·5-12·2)	11·9<br>(11·2-12·8)	12·8<br>(12·1-14·2)	13·6<br>(12·7-15·0)
Zambia	13·0<br>(10·7-14·9)	13·3<br>(11·3-14·9)	13·4<br>(11·7-14·6)	12·9<br>(11·7-14·2)	11·6<br>(10·7-13·2)	10·0<br>(9·1-11·0)	9·5<br>(8·7-10·7)	10·2<br>(9·1-12·0)	11·6<br>(10·0-13·3)	13·0<br>(11·1-16·0)
Central Sub-Saharan Africa	11·4<br>(10·9-11·9)	11·5<br>(11·1-12·0)	11·5<br>(11·1-12·0)	11·6<br>(11·2-12·1)	11·7<br>(11·2-12·2)	11·5<br>(11·1-12·0)	11·4<br>(11·0-12·0)	11·6<br>(11·2-12·1)	12·0<br>(11·5-12·5)	12·8<br>(12·3-13·2)
Angola	11·8<br>(10·5-13·4)	11·9<br>(10·6-13·5)	11·8<br>(10·5-13·3)	11·5<br>(10·4-13·1)	11·1<br>(10·0-12·7)	10·8<br>(9·6-12·4)	10·9<br>(9·8-12·5)	11·4<br>(10·2-13·2)	12·3<br>(10·8-14·3)	13·3<br>(11·7-15·1)
Central African Republic	10·4<br>(9·4-11·9)	10·6<br>(9·6-11·9)	10·8<br>(10·0-11·9)	11·0<br>(10·3-11·8)	10·3<br>(9·7-11·1)	10·1<br>(9·2-11·3)	10·0<br>(8·8-11·6)	10·1<br>(8·8-12·1)	10·5<br>(9·0-12·6)	10·7<br>(9·3-12·6)
Congo	9·4<br>(8·4-10·7)	9·6<br>(8·6-10·9)	9·8<br>(8·9-11·1)	10·2<br>(9·4-11·3)	10·2<br>(9·5-11·1)	10·1<br>(9·4-10·9)	10·2<br>(9·5-11·1)	10·9<br>(10·1-12·0)	11·6<br>(10·6-12·8)	12·4<br>(11·0-13·9)
Democratic Republic of the Congo	11·6<br>(11·0-12·3)	11·8<br>(11·1-12·4)	11·7<br>(11·2-12·3)	11·9<br>(11·3-12·5)	12·1<br>(11·6-12·8)	11·9<br>(11·4-12·6)	11·8<br>(11·3-12·5)	11·8<br>(11·4-12·5)	12·0<br>(11·5-12·5)	12·8<br>(12·3-13·3)
Equatorial Guinea	9·6<br>(8·8-10·7)	9·8<br>(9·0-10·9)	10·2<br>(9·2-11·3)	10·3<br>(9·4-11·6)	10·0<br>(9·1-11·1)	10·1<br>(9·2-11·4)	12·0<br>(10·5-14·3)	13·8<br>(11·2-16·8)	14·7<br>(12·0-17·5)	15·7<br>(13·1-19·3)
Gabon	11·8<br>(10·4-13·7)	12·4<br>(11·0-14·3)	12·9<br>(11·7-14·6)	13·0<br>(12·0-14·4)	12·6<br>(11·8-13·4)	12·6<br>(11·9-13·2)	11·9<br>(11·2-12·7)	12·1<br>(11·3-13·0)	13·2<br>(12·2-14·1)	14·2<br>(13·1-16·0)

Appendix Table 15A. Live births (in thousands) by location, both sexes combined, 1970, 1975, 1980, 1985, 1990, 1995, 2000, 2005, 2010, 2016

Country	1970	1975	1980	1985	1990	1995	2000	2005	2010	2016
Global	114039·51	115081·9	120500·14	127067·7	129806·24	126351·26	125778·47	128505·97	133282·62	128802·94
High-income	13780·08	12281·32	12441·32	11975·02	12378·57	11737·64	11773·67	11841·6	11847	11600·27
High-income North America	3962·54	3447·42	3969	4128·02	4548·69	4303·96	4377·95	4536·46	4332·73	4449·56
Canada	357·5	352·09	367·84	376·35	404·47	368·6	330·65	346·27	373·65	402·71
Greenland	1·52	1·01	1·02	1·04	1·14	1	0·85	0·75	0·71	0·7
United States	3603·52	3094·32	3600·14	3750·63	4143·08	3934·35	4046·45	4189·43	3958·37	4046·15
Australasia	327·83	282·05	278·37	294·89	317·63	310·45	302·59	317·39	355·61	357·55
Australia	265·1	227·19	228·03	242·9	258·57	253·22	246·05	258·93	293·71	300·49
New Zealand	62·73	54·86	50·33	52	59·06	57·22	56·53	58·46	61·9	57·06
High-income Asia Pacific	2979·97	2734·04	2484·77	2119·33	1968·69	1909·55	1782·38	1558·5	1579·16	1376·12
Brunei	3·67	4·53	5·78	6·25	7·09	7·68	8·07	7·15	6·68	6·54
Japan	1977·67	1832·96	1568·23	1430·34	1227·86	1189·95	1175·52	1087·81	1075·52	935·88
Singapore	45·73	39·48	41·05	42·21	48·12	47·87	42·8	36·26	35·2	34·33
South Korea	952·89	857·07	869·72	640·54	685·62	664·05	555·99	427·29	461·76	399·37
Western Europe	5722·38	4947·99	4795·29	4518·3	4584·61	4298·19	4317·64	4436·58	4553·01	4402·44
Andorra	0·16	0·22	0·28	0·4	0·5	0·6	0·6	0·61	0·64	0·6
Austria	110·06	90·4	91·54	86·13	91·51	88·81	77·41	78·63	78·42	82·43
Belgium	140·56	117·41	122·63	115·33	124·3	115·99	116·46	121·38	127·58	129·84
Cyprus	13·53	10·64	12·2	12·1	12	10·12	8·26	8·48	9·77	10·41
Denmark	72·07	68·57	55·54	54·38	63·53	68·41	66·64	65·15	61·77	59·73
Finland	61·81	66·09	63·34	61·67	65·77	62·31	56·75	58·41	60·42	59·55
France	854·71	721·92	799·99	771·26	760·41	731·46	774·61	788·62	798·46	792·44
Germany	1025·41	791·67	849·35	822·7	854	780·24	751·65	684·55	663·47	697·37
Greece	141·61	142·06	142·13	112·79	102·73	103·79	102·9	110·58	112·68	90·9
Iceland	4·08	4·29	4·4	3·79	4·62	4·31	4·18	4·32	4·71	4·25
Ireland	66·14	66·67	72·76	61·87	53·54	49·61	55·85	62·7	75·69	63·39
Israel	76·01	88·43	101·43	105·1	103·14	114·71	132·65	146·61	163·42	171·7
Italy	910·74	820·58	639·69	574·33	571·73	525·58	538·17	554·98	550·1	491·62
Luxembourg	4·35	3·88	4·26	4·16	4·93	5·5	5·59	5·42	5·69	6·51
Malta	5·46	5·98	5·71	5·7	5·83	5	4·02	3·63	3·9	3·77
Netherlands	229·52	174·83	179·77	180·65	197·4	188·86	204·61	188·65	183·54	177·48
Norway	63·25	53·82	50·56	51·57	60·78	60·59	58·14	57·71	60·51	62·21
Portugal	181·36	177·97	153·35	128·29	116·43	108·3	115·38	106·37	99·64	81·77
Spain	666·23	672·96	546·2	442·92	393·73	361·68	401·78	468·98	481·88	421·35
Sweden	110·96	100·72	96·14	100·41	123·84	99·06	91·17	104·21	115·34	118·31
Switzerland	96·92	76·7	73·64	74·99	83·56	82	75·55	73·57	80·23	85·55
United Kingdom	887·44	692·16	730·4	747·79	790·33	731·25	675·27	743·03	815·12	791·29
England	722·52	562·55	596·27	618·44	662·65	613·67	569·19	631·27	694·37	675·59
East Midlands	56·91	46·07	49·02	48·86	53·21	49·43	45·56	50·76	55·46	54·52
East of England	67·39	54·81	59·95	63·11	67·5	63·38	59·37	66·82	73·29	73·34
Greater London	115·14	89·8	94·09	101·3	112·41	106·57	105·74	118·52	137·03	132·86
North East England	43·96	32·51	33·84	33·58	34·18	30·77	26·93	28·59	30·1	29·1
North West England	118·18	87·49	91·05	92·51	96·71	85·09	76·62	83·73	90·41	85·39
South East England	98·87	80·42	87·79	93·41	100·63	94·12	86·91	96·76	107·8	103·19
South West England	56·9	46·54	50·25	52·63	56·9	54·53	49·26	54·79	60·99	60·06
West Midlands	87·76	65·9	68·07	69·59	73·57	67·18	61·96	68·3	73·66	71·72
Yorkshire and the Humber	77·41	59·01	62·22	63·46	67·54	62·6	56·83	62·99	65·62	65·42
Northern Ireland	30·42	28·22	29·13	28·38	26·11	23·99	21·83	22·92	25·48	23·06
Scotland	87·66	65·89	68·66	65·39	65·61	59·61	52·88	55·23	58·94	58·68
Wales	46·84	35·5	36·33	35·58	35·96	33·98	31·37	33·61	36·33	33·96
Southern Latin America	787·36	869·82	913·89	914·47	958·95	915·49	993·11	992·67	1026·49	1014·59
Argentina	484·92	582·78	628·57	614·89	620·52	585·33	687·64	707·46	734·22	725·79
Chile	245·61	233·27	238·62	251·82	287·74	273·49	251·75	236·97	245·31	240·37
Uruguay	56·83	53·77	46·7	47·76	50·69	56·67	53·72	48·24	46·97	48·44
Central Europe, Eastern Europe, and Central Asia	6306·91	6806·27	7002·7	7473·03	6529·09	5007·27	4493·59	4872·19	5570·89	5652·53
Eastern Europe	2988·41	3182·98	3281·42	3575·94	2891·75	2054·53	1872·11	2104·02	2514·56	2587·45
Belarus	147·96	147·43	156·14	167·91	141·29	102·43	93·24	95·39	110·68	115·39
Estonia	21·77	21·4	22·36	23·81	21·02	13·52	12·99	14·6	15·05	13·68
Latvia	34·71	34·34	35·69	41·29	36·57	20·98	19·85	21·89	19·58	22·38
Lithuania	56·02	51·9	50·92	58·56	56·46	39·87	33·23	29·63	31·71	29·41
Moldova	75·71	82·05	86·36	92·74	77·91	55·75	42·62	45·7	45·76	44·86
Russia	1922·91	2105·3	2181·54	2413·72	1910·51	1337	1283·43	1455·31	1785·1	1882·26
Ukraine	729·33	740·55	748·42	777·9	648	484·98	386·75	441·51	506·67	479·47
Central Europe	1907·61	2140·61	2053·27	1956·61	1675·57	1330·35	1175·25	1161·77	1164·39	1090·27
Albania	57·13	62·83	71·28	80·63	85·27	68·79	50·51	38·37	34·84	39·46
Bosnia and Herzegovina	84·01	76·67	72·46	79·33	71·59	50·28	33·51	33·09	34·59	31·58
Bulgaria	135·94	144·86	126·51	121·47	101·36	71·55	71·19	70·56	71·96	64·04
Croatia	65·16	65·52	66·51	63·5	54·76	50·37	40·76	40·67	41·11	37·34
Czech Republic	147·78	187·7	151·2	135·41	131·11	91·36	89·12	102·75	112·33	106·08
Hungary	150·56	188·61	147·83	130·16	127·26	108·04	96·48	97·95	89·79	88·85
Macedonia	41·48	40·65	40·7	38·03	34·98	32	27·82	22·84	23·63	21·63
Montenegro	10·71	10·64	10·65	10·86	9·72	9·02	8·17	7·61	7·61	7·16
Poland	537·54	647·14	683·46	659·17	529·84	408·19	370·59	370·18	401·48	365·83
Romania	421·29	431·17	405·4	373·73	289·52	232	215·86	212·51	175·73	172·89
Serbia	147·31	157·82	153·57	149·96	137·98	129·43	100·72	93·37	84·99	80·41
Slovakia	80·61	97·06	93·82	89·06	79·94	60·4	52·66	53·25	63·99	55·02
Slovenia	28·07	29·94	29·88	25·29	22·23	18·92	17·87	18·61	22·34	19·97
Central Asia	1410·89	1482·68	1668·01	1940·49	1961·77	1622·39	1446·22	1606·4	1891·95	1974·81
Armenia	61·58	69·54	84·9	89·32	78·36	47·27	37·08	38·72	41·71	44·95
Azerbaijan	146·94	144·93	163·46	185·13	188·57	154·56	125·62	157·84	200·23	187·31
Georgia	90·49	94·54	94·71	97·87	89·63	59·28	51·6	59·05	67·9	65·04
Kazakhstan	387·72	388·78	396·36	418·56	373·91	281·14	239·57	304·53	380·55	393·74
Kyrgyzstan	97·54	97·91	108·73	128·63	128·49	110·85	99·86	110	143·27	152·89
Mongolia	52·36	55·17	62·81	70·9	68·83	53·22	48·19	51·98	67·9	77·93
Tajikistan	111·37	120·62	142·65	180·34	198·42	172·44	158·1	168·99	206·55	251·42
Turkmenistan	69·06	76·1	89·37	111·55	125·58	115·78	100·68	103·31	114·2	120·95
Uzbekistan	393·83	435·09	525·03	658·18	709·97	627·84	585·52	611·98	669·63	680·58
Latin America and Caribbean	8937·75	9699·2	10519·57	10819·81	11086·83	11032·91	10788·85	10451·54	10324·77	9815·42
Central Latin America	3756·02	4153·35	4525·07	4805·11	5125·18	5212·6	5139·48	4972·8	4841·85	4501·11
Colombia	683·52	736·58	795·57	863·5	897·7	886·25	844·48	828·99	794·64	676·83
Costa Rica	58·34	59·29	70·03	81·17	79·74	76·38	74·69	70·77	67·38	57·96
El Salvador	145·92	162·16	160·34	150·66	154·4	154·93	145·18	131·6	117·77	103·01
Guatemala	245·39	267·62	285·57	315·66	352·52	387·64	409·88	413·59	402·6	413·58
Honduras	125·35	139·65	154·28	170·79	179·98	178·88	175·58	176·91	188·82	196·02
Mexico	1975·48	2205·65	2415·94	2552·61	2686·9	2771·36	2704·55	2546	2461·85	2304·77
Nicaragua	76·13	86·02	99·04	112·18	125·71	140·79	145·16	141·52	132·92	117·78
Panama	54·56	54·75	55·96	57·29	61·01	66·44	66·99	67·65	72·26	67·13
Venezuela	391·33	441·62	488·33	501·26	587·22	549·93	572·98	595·79	603·61	564·05
Andean Latin America	870·75	983·42	1092·25	1164·48	1197·76	1210·3	1188·14	1196·45	1286·65	1393·24
Bolivia	191·48	212·17	231·79	241·12	245·58	257·92	266·3	269·15	274·48	290·45
Ecuador	216·33	251·86	290·25	313·21	326·66	337·35	333·97	339·78	355·09	368·72
Peru	462·94	519·39	570·22	610·16	625·51	615·03	587·88	587·53	657·08	734·07
Caribbean	878·4	838·7	817·81	852·95	870·28	839·37	838·83	814·09	822·63	784·25
Antigua and Barbuda	1·86	1·62	1·42	1·21	1·08	1·13	1·32	1·33	1·26	1·22
The Bahamas	4·81	5·08	5·51	5·63	5·98	6·24	5·5	5·94	6·48	6·74
Barbados	5·13	4·84	4·7	4·44	3·89	3·32	3·12	3·27	3·14	2·87
Belize	5·51	5·75	6·24	7·57	7·49	6·39	7·71	8·53	9·11	10
Bermuda	1·06	0·78	0·7	0·76	0·81	0·84	0·85	0·87	0·88	0·92
Cuba	263·92	188·32	135·6	173·8	182·97	144·39	146·11	115·7	130·11	109·13
Dominica	2·92	2·14	1·91	1·81	1·69	1·55	1·25	1·17	1·11	1·11
Dominican Republic	168·92	189·2	202·03	184·71	200·44	215·67	213·1	209·66	202·7	185·07

Appendix Table 15A. Live births (in thousands) by location, both sexes combined, 1970, 1975, 1980, 1985, 1990, 1995, 2000, 2005, 2010, 2016

Country	1970	1975	1980	1985	1990	1995	2000	2005	2010	2016
Grenada	2·84	2·83	2·66	3·3	2·56	2·39	2·09	1·91	1·83	1·7
Guyana	22·02	23·66	24·33	21·89	22·55	21·65	19·16	16·04	13·5	13·63
Haiti	205·9	220·59	239·07	259·68	264·44	270·87	286·56	310·32	320·85	327·45
Jamaica	69·5	65·45	66·56	68·58	69·68	67·88	63·92	58·12	56·31	56·26
Puerto Rico	71·64	78·17	71·78	63·05	61·89	56·23	50·62	45·11	43·07	41·46
Saint Lucia	4·72	4·71	4·51	4·37	3·91	3·33	2·85	2·47	2·1	1·88
Saint Vincent and the Grenadines	3·93	3·8	3·67	3·4	2·95	2·6	2·19	1·96	1·92	1·78
Suriname	13·27	11·03	11·13	11·87	11·75	12·15	11·99	11·36	10·34	9·28
Trinidad and Tobago	27·96	28·13	33·55	34·55	24·02	20·85	18·92	18·87	16·72	12·67
Virgin Islands, U.S.	2·5	2·58	2·44	2·32	2·16	1·9	1·57	1·45	1·22	1·07
Tropical Latin America	3432·58	3723·74	4084·44	3997·26	3893·61	3770·65	3622·4	3468·2	3373·64	3136·82
Brazil	3348·39	3632·09	3983·99	3876·39	3753·99	3623·11	3476·91	3329·32	3240·94	3010·21
Paraguay	84·19	91·65	100·45	120·87	139·62	147·54	145·49	138·88	132·7	126·6
Southeast Asia, East Asia, and Oceania	37870·19	34657·63	33687·13	35143·25	34898·71	31362·73	28329·85	26670·02	26525·22	23579·73
East Asia	27729·88	24007·05	22420·3	23473·51	22938·99	19065·81	15939·2	14325·39	14136	12008·02
China	26759·08	23098·53	21542·31	22562·43	21981·14	17980·88	14890·3	13481·17	13339·4	11209·66
North Korea	588·27	531·01	479·35	593·29	631·21	758·7	763·78	637·81	611·31	606·75
Taiwan (Province of China)	382·52	377·52	398·63	317·79	326·64	326·23	285·12	206·41	185·29	191·61
Southeast Asia	9985·52	10476·66	11083·27	11472·55	11745·43	12058·61	12135·38	12080·36	12114·18	11277·65
Cambodia	258·39	273·08	254·65	266·86	285·91	310·78	325·95	364·82	387·62	391·47
Indonesia	4230·11	4287·71	4464·57	4461·71	4517·65	4785·55	4952·84	4939·77	4968·25	4409·86
Laos	126·41	139·26	132·23	145·75	165·31	184·63	187·82	194·66	221·98	252·66
Malaysia	350·63	378·65	418·19	490·7	509·43	539·08	534·02	487·07	526·99	508·07
Maldives	3·06	3·13	3·48	4·09	4·6	4·9	4·99	5·92	6·86	5·84
Mauritius	21·22	23·13	23·59	17·85	22·1	21·69	19·65	16·99	13·59	13·29
Myanmar	766·19	870·55	959·11	1045·67	1081·14	1083·1	1098·55	1095·5	1029·96	924·17
Philippines	1287·95	1448·66	1637·6	1785·39	1937·89	2066·3	2174·54	2258·28	2307·37	2423·92
Sri Lanka	360·55	373·14	426·85	382·36	365·72	349·56	349·57	369·5	353·08	272·19
Seychelles	1·91	1·79	1·89	1·87	1·56	1·59	1·42	1·68	1·74	1·56
Thailand	1394·27	1459·17	1377·29	1224·05	1193·59	1159·82	1025·51	851·57	710·62	556·29
Timor-Leste	22·62	24·8	23·46	25·23	25·69	26·55	19·02	26·28	35·36	33·03
Vietnam	1162·22	1193·6	1360·37	1621·02	1634·84	1525·05	1441·5	1468·33	1550·74	1485·29
Oceania	154·8	173·92	183·56	197·19	214·3	238·3	255·27	264·26	275·05	294·07
American Samoa	1·08	1·2	1·27	1·46	1·72	1·72	1·71	1·63	1·54	1·5
Federated States of Micronesia	3·07	2·88	3·51	3·72	3·54	3·27	2·79	2·19	1·96	1·97
Fiji	18·23	20·43	20·72	20·31	17·12	15·38	13·61	12·38	11·42	9·96
Guam	2·31	2·63	2·91	3·14	3·56	3·64	3·6	3·37	3·29	3·48
Kiribati	2·23	2·56	2·81	2·88	3·11	2·97	2·92	2·91	2·91	2·82
Marshall Islands	0·94	1·11	1·22	1·4	1·6	1·85	1·9	1·94	2·02	2·08
Northern Mariana Islands	0·43	0·52	0·6	1·08	1·9	2·41	2·66	2·83	2·96	3·18
Papua New Guinea	107·23	121·64	127·18	138·2	155·23	178·27	195·4	204·42	214·86	235·56
Samoa	5·65	5·66	5·78	5·77	5·6	5·6	5·55	5·67	5·7	5·42
Solomon Islands	6·73	7·74	9·19	10·23	11·32	13·27	15·12	16·27	17·18	16·99
Tonga	3·02	3·01	3·13	3·21	3·35	3·17	2·99	3·12	3·02	2·61
Vanuatu	3·88	4·55	5·25	5·79	6·24	6·76	7·03	7·51	8·19	8·51
North Africa and Middle East	8085·67	8958·17	9844·67	10606·62	10809·83	10825·82	11268·35	12013·2	13162·22	12621·29
North Africa and Middle East	8085·67	8958·17	9844·67	10606·62	10809·83	10825·82	11268·35	12013·2	13162·22	12621·29
Afghanistan	533·2	589·12	606·46	522·8	523·14	669·94	752·9	902·59	941·32	1160·01
Algeria	597·26	655·53	723·76	796·88	844·62	930·19	1034·1	1064·52	1007·9	870·3
Bahrain	7·29	8·7	10·86	13·6	14·1	13·28	13·41	12·71	19·12	18·77
Egypt	1466·25	1558·22	1640·14	1716·36	1706·71	1634·1	1738·97	1917·06	2203·45	2125·74
Iran	1340·86	1508·63	1744·84	1998·7	1951·36	1395·69	1280·31	1535·05	1722·58	1540·7
Iraq	449·03	508·05	571·41	630·1	667·63	751·85	851·9	1000·72	1413·25	1656·91
Jordan	92·41	98·95	93·02	105·02	110·36	143·81	143·07	156·04	186·9	197·32
Kuwait	37·94	46·6	53·02	55·09	42·93	45·28	41·91	47·89	59·05	53·66
Lebanon	93·8	98·73	89·9	80·58	69·19	65·57	56·79	53·28	55·46	68·65
Libya	76·09	82·99	91·54	101·58	111·15	124·65	136·07	137·73	122·19	78·01
Morocco	553·76	591·86	660·93	724·85	749·87	704·63	632·02	565·82	512·06	443·38
Palestine	55·05	60·03	63·28	72·09	85·87	103·42	115·98	128·67	192·55	232·96
Oman	39·43	46·23	55·86	66·78	73·52	81·06	92·72	91·83	86·37	90·73
Qatar	4	4·93	7·51	9·62	11·07	10·8	10·71	14·14	21·6	26·09
Saudi Arabia	293·1	363·58	442·3	590·66	642·77	648·97	703·02	664·29	587·01	449·88
Sudan	383·9	428·92	485·56	536·86	608·55	771·48	899·45	932·26	924·85	875·61
Syria	274·54	321·9	364·24	409·35	446·81	483·87	504·1	488·91	509·4	296·92
Tunisia	179·73	197·23	219·28	229·84	218·91	191·14	166·57	171·1	186·46	152·02
Turkey	1289·46	1438·19	1508·33	1450·19	1333·61	1338·68	1310·25	1270·05	1283·46	1188·41
United Arab Emirates	11·43	20·12	39·18	55·08	56·96	68·9	76·39	105·41	172·3	153·44
Yemen	307·16	329·66	373·25	440·59	540·7	648·48	707·69	753·12	954·96	941·79
South Asia	26028·67	28201·58	30826·45	33100·2	34068·74	34236·36	34379·78	34574·43	34209·61	30167·22
South Asia	26028·67	28201·58	30826·45	33100·2	34068·74	34236·36	34379·78	34574·43	34209·61	30167·22
Bangladesh	2653·97	2606·14	2841·34	3205·13	3422·78	3304·79	3235·34	3276·6	3163·71	2808·85
Bhutan	13·93	15·66	17·01	18·29	20·47	18·8	19·94	19·96	18·24	15·31
India	20267·82	22133·93	23933·97	25088·56	25674·35	26011·54	26412·06	26471·17	25404·63	21807·82
Nepal	500·79	551·56	606·58	667·36	727·02	751·25	777·49	715·79	762·88	828·76
Pakistan	2592·16	2894·28	3427·57	4120·87	4224·12	4149·99	3934·95	4090·91	4860·14	4706·49
Sub-Saharan Africa	13030·25	14477·73	16178·29	17949·77	20034·47	22148·53	24744·39	28082·99	31642·91	35366·48
Southern Sub-Saharan Africa	943·74	1058·17	1183·39	1335·16	1460·75	1508·16	1578·53	1672·54	1761·05	1852·63
Botswana	26·06	29·57	34·38	38·29	40·49	43·76	45·95	48·74	53·15	54·53
Lesotho	38·39	41·29	44·39	47·05	47·26	50·54	52·48	52·88	55·15	55·83
Namibia	30·82	34·45	36·67	41·1	53·6	58·68	61·35	59·42	64·36	72·07
South Africa	634·03	706·23	786·01	868·49	927·1	950·63	993·69	1048·3	1039·83	1072·2
Swaziland	17·81	19·76	22·2	25·4	30·3	30·77	33·75	36·24	43·99	44·34
Zimbabwe	196·64	226·86	259·74	314·83	362	373·77	391·29	426·96	504·56	553·65
Western Sub-Saharan Africa	5472·53	5972·66	6570·06	7228·2	8109·05	9063·3	10274·06	11785·65	13404·65	14748·41
Benin	135·33	148·13	166·34	187·68	209·7	243·1	276·24	326·92	372·85	438·87
Burkina Faso	277·97	298·1	323·12	361·97	410·75	452·85	518·96	603·87	677·62	735·65
Cameroon	311·54	335·45	370·4	420·61	477·95	529·92	591·35	676·36	781·88	877·4
Cape Verde	8·98	8·77	9·79	11·92	12·37	13·23	13·76	14·94	15·49	15·13
Chad	173·68	194·67	211·16	232·35	266·27	305·76	358·32	432·75	516·22	637·56
Cote d'Ivoire	233·53	279·59	341·91	426·48	472·59	514·16	587·26	641·36	710·94	823·29
The Gambia	25·2	28·46	32·53	36·21	44·71	51·74	59·06	65·41	71·89	80·69
Ghana	383·77	413·42	439·64	512·7	557·92	575·41	612·06	726·66	884·05	933·7
Guinea	181·33	187·3	191·71	210·79	243·24	307·65	338·62	369·73	412·52	467·91
Guinea-Bissau	30·29	33·74	36·47	38·86	41·77	45·59	50·54	56·53	62·61	69·24
Liberia	70·8	78·49	88·44	99·47	93·62	91·59	124·25	129·82	144·96	158·5
Mali	293·35	317·37	342·61	372·28	390·53	445·49	511·94	585·16	656·36	739·52
Mauritania	43·38	48·34	54·45	61·27	68·12	75·67	85·13	96·74	104·61	111·6
Niger	245·08	272·72	307·71	347·47	396·17	463·41	538·69	620·71	722·95	889·95
Nigeria	2659·96	2887·42	3173·61	3369·63	3817·86	4305·59	4912·85	5610·16	6329·15	6714·13
Sao Tome and Principe	3	3·36	3·8	4·15	4·47	4·92	5·45	5·89	6·46	7·05
Senegal	186·86	211·69	230·78	259·42	295·36	327·85	356·18	428·39	496·7	560·81
Sierra Leone	115·11	123·35	132·79	142·21	154·7	147·87	154·04	193·12	217·66	243·01
Togo	93·37	102·3	112·8	132·74	150·96	161·52	179·34	201·12	219·72	244·4
Eastern Sub-Saharan Africa	5150·04	5839·09	6627·13	7382·97	8215·21	8960·41	9911·87	11153·7	12445·78	13926·21
Burundi	170·8	179·03	208·9	235·04	255·61	259·35	262·96	327·31	410·85	494·24
Comoros	9·42	10·52	12·48	13·81	14·85	15·91	18·06	20·02	20·45	19·26
Djibouti	8·61	11·68	17·76	20·92	28·22	31·01	33·2	34·88	36·15	36·2
Eritrea	86·37	97·83	109·69	122·39	132·36	117·63	128·06	159·38	169·94	163·39
Ethiopia	1252·19	1405·14	1500·78	1681·69	1952·58	2222·09	2444·29	2706·48	2956·57	3422·08
Kenya	560·09	667·94	777·36	885·22	981·45	1074·76	1168·16	1271·44	1352·05	1392·06
Madagascar	324·9	370·54	419·89	470·67	523·54	593·89	665·97	730·64	792·05	888·11

Appendix Table 15A. Live births (in thousands) by location, both sexes combined, 1970, 1975, 1980, 1985, 1990, 1995, 2000, 2005, 2010, 2016										
**Country**	**1970**	**1975**	**1980**	**1985**	**1990**	**1995**	**2000**	**2005**	**2010**	**2016**
Malawi	263·68	289·08	320·16	355·35	462·45	450·66	495·19	543·73	628·83	719·85
Mozambique	429·78	484·44	556·82	573·8	543·28	666·96	768·14	891·23	1020·29	1104·39
Rwanda	190	226·83	272·44	311·06	327·42	271·69	326·31	377·51	392·33	400·04
Somalia	129·18	145·33	225·93	218·74	220·7	210·13	227·09	246·93	268·47	300·57
South Sudan	219·11	237·87	265·6	304·96	316·8	294·3	353·99	426·84	532·6	681·17
Tanzania	751·01	855·52	964·43	1075·69	1193·56	1355·85	1438·75	1610·25	1814·46	1977·41
Uganda	526·31	595·54	678·53	770·57	885·08	995·12	1135·4	1310·07	1491·38	1688·58
Zambia	228·59	261·81	296·36	343·04	377·33	401·05	446·29	496·99	559·35	638·87
Central Sub-Saharan Africa	1463·94	1607·81	1797·71	2003·44	2249·47	2616·67	2979·93	3471·1	4031·42	4839·23
Angola	301·42	341·82	396·8	468·5	528·11	615·75	704·17	820·36	955·63	1108·82
Central African Republic	71·94	77·05	85·6	98·74	106·68	120·85	132·93	141·08	156·68	178·57
Congo	57·49	64·81	72·51	82·1	93·25	105·87	120·5	131·98	152·29	161·48
Democratic Republic of the Congo	996	1091·7	1209·78	1312·29	1473·98	1722·48	1966·43	2315·86	2698·61	3314·51
Equatorial Guinea	12·43	7·84	7·47	14·01	16·3	16·87	16·47	17·44	19·56	22·67
Gabon	24·66	24·58	25·55	27·8	31·15	34·85	39·42	44·38	48·65	53·19

Appendix Table 15B. Live births (in thousands) by location, males, 1970, 1975, 1980, 1985, 1990, 1995, 2000, 2005, 2010, 2016										
**Country**	**1970**	**1975**	**1980**	**1985**	**1990**	**1995**	**2000**	**2005**	**2010**	**2016**
Global	58589·16	59101·7	61896·83	65407·08	67083·72	65388·99	65159·37	66555·63	68970·04	66507·94
High-income	7077·82	6307·06	6387·2	6154·82	6361·42	6024·93	6042·21	6072·42	6075·51	5948
High-income North America	2033·01	1767·72	2034·27	2114·54	2329·39	2202·93	2240·73	2321·8	2217·86	2277·69
Canada	183·95	180·84	188·93	192·94	207·75	189·15	169·67	177·61	191·91	206·84
Greenland	0·78	0·52	0·51	0·54	0·57	0·5	0·42	0·38	0·38	0·35
United States	1848·28	1586·37	1844·83	1921·05	2121·08	2013·28	2070·64	2143·81	2025·57	2070·49
Australasia	168·1	144·81	142·92	151·29	163·24	159·35	155·24	163·02	182·66	183·55
Australia	135·97	116·69	117·07	124·64	132·81	129·94	126·32	132·99	150·93	154·26
New Zealand	32·13	28·12	25·85	26·65	30·43	29·41	28·92	30·03	31·74	29·28
High-income Asia Pacific	1533·72	1407·18	1279·22	1100·96	1024·47	987·86	921·46	801·97	812·73	708·2
Brunei	1·89	2·33	2·97	3·21	3·64	3·94	4·14	3·67	3·43	3·36
Japan	1015·77	941·44	805·47	734·65	630·65	611·18	603·77	558·72	552·41	480·69
Singapore	23·51	20·39	21·22	21·89	24·94	24·9	22·32	18·71	18·21	17·72
South Korea	492·56	443·02	449·56	341·22	365·23	347·84	291·23	220·87	238·69	206·44
Western Europe	2941·46	2543·78	2464·77	2321·71	2355·32	2207·94	2218·36	2279·45	2338·83	2261·2
Andorra	0·08	0·11	0·15	0·21	0·26	0·31	0·31	0·31	0·33	0·31
Austria	56·5	46·41	46·99	44·22	46·98	45·59	39·74	40·37	40·26	42·32
Belgium	72·26	60·39	63·01	59·29	63·69	59·22	59·65	62·17	65·35	66·5
Cyprus	6·98	5·47	6·27	6·21	6·17	5·2	4·32	4·35	4·98	5·35
Denmark	37·02	35·25	28·45	27·98	32·61	35·13	34·16	33·45	31·73	30·68
Finland	31·69	33·85	32·41	31·54	33·53	31·84	29·07	29·84	30·91	30·46
France	438·18	370·11	410·13	395·4	389·84	375	397·12	404·3	409·35	406·26
Germany	527·15	406·99	436·64	422·94	439·03	401·11	386·42	351·92	341·09	358·51
Greece	73·17	73·47	73·66	58·22	53·03	53·46	53·09	57·11	58·03	46·88
Iceland	2·11	2·2	2·26	1·95	2·38	2·2	2·13	2·21	2·4	2·17
Ireland	34·19	34·46	37·61	31·98	27·67	25·64	28·87	32·41	39·12	32·62
Israel	39·02	45·36	52·1	53·86	52·98	58·97	68·07	75·2	83·82	88·07
Italy	469·28	422·82	329·61	295·93	294·59	270·82	277·3	285·97	283·45	253·32
Luxembourg	2·22	1·99	2·18	2·13	2·52	2·82	2·86	2·78	2·91	3·33
Malta	2·81	3·08	2·94	2·93	3	2·57	2·07	1·87	2·01	1·94
Netherlands	117·99	89·88	92·42	92·87	101·48	97·09	105·19	96·98	94·22	91·03
Norway	32·45	27·67	25·99	26·45	31·29	31·15	29·79	29·64	31·02	31·98
Portugal	93·32	91·58	78·91	66·01	59·91	55·73	59·37	54·73	51·27	42·07
Spain	343·44	346·91	281·57	228·33	202·97	186·45	207·12	241·76	248·41	217·21
Sweden	57·15	51·78	49·45	51·62	63·55	50·88	46·94	53·62	59·35	60·88
Switzerland	49·69	39·32	37·75	38·44	42·84	42·04	38·73	37·72	41·13	43·86
United Kingdom	454·75	354·69	374·28	383·19	404·99	374·72	346·03	380·75	417·7	405·48
England	370·04	288·16	305·57	317	339·48	314·54	291·36	323·25	356·09	346·2
East Midlands	29·14	23·59	25·11	25·24	27·39	25·49	23·37	26·12	28·21	27·45
East of England	34·53	28·09	30·73	32·21	34·82	32·34	30·44	34·3	37·67	36·3
Greater London	58·77	45·85	48·06	51·83	57·19	54·8	54·08	60·55	70·3	68·41
North East England	22·48	16·63	17·32	17·34	17·58	15·76	13·83	14·5	15·46	14·96
North West England	60·75	44·98	46·83	47·28	49·49	43·55	39·18	42·99	46·29	43·65
South East England	50·88	41·39	45·21	47·78	51·51	48·16	44·49	49·53	55·29	53·87
South West England	29·16	23·85	25·77	27·08	29·22	27·91	25·29	28·13	31·27	30·88
West Midlands	44·83	33·67	34·79	35·69	37·82	34·41	31·68	35·02	37·96	37·61
Yorkshire and the Humber	39·46	30·09	31·74	32·52	34·43	32·13	28·98	32·11	33·65	33·08
Northern Ireland	15·64	14·52	14·94	14·55	13·38	12·44	11·29	11·65	13	12·29
Scotland	45·09	33·88	35·21	33·42	33·74	30·41	27·1	28·54	29·94	29·58
Wales	23·91	18·12	18·56	18·22	18·39	17·34	16·29	17·32	18·67	17·35
Southern Latin America	401·54	443·57	466·02	466·32	489	466·86	506·42	506·18	523·42	517·36
Argentina	247·21	297·1	320·45	313·47	316·34	298·4	350·56	360·67	374·31	370·01
Chile	125·22	118·92	121·65	128·38	146·69	139·42	128·34	120·81	125·06	122·54
Uruguay	29·11	27·54	23·92	24·46	25·96	29·03	27·51	24·71	24·06	24·81
Central Europe, Eastern Europe, and Central Asia	3236·76	3492·92	3593·2	3836·88	3358·66	2579·91	2315·66	2509·75	2870·03	2907·68
Eastern Europe	1531·3	1631·04	1681·31	1835·43	1486·88	1057·76	964·27	1082·09	1293·68	1328·87
Belarus	76	75·62	80·05	86·4	72·87	52·85	48	49·15	56·98	59·4
Estonia	11·19	11	11·46	12·24	10·81	6·98	6·67	7·53	7·72	7·04
Latvia	17·83	17·65	18·32	21·2	18·72	10·82	10·18	11·13	10·05	11·48
Lithuania	28·61	26·55	26·12	30·11	28·97	20·54	17·07	15·15	16·26	15·08
Moldova	38·98	42·24	44·46	47·74	40·05	28·58	21·96	23·47	23·54	23·06
Russia	984·45	1078·33	1116·85	1238·02	982·18	687·97	661·01	748·16	917·7	965·87
Ukraine	374·25	379·66	384·05	399·73	333·28	250·01	199·37	227·49	261·43	246·94
Central Europe	980·54	1100·26	1055·33	1005·67	861·08	683·9	604·2	597·1	598·44	560·33
Albania	29·53	32·48	36·85	41·68	44·08	35·69	26·2	19·91	18·07	20·47
Bosnia and Herzegovina	43·33	39·54	37·37	40·91	36·92	25·93	17·28	17·06	17·84	16·23
Bulgaria	69·98	74·57	65·13	62·5	51·96	36·78	36·68	36·24	37·01	32·95
Croatia	33·51	33·7	34·21	32·66	28·17	25·91	20·97	20·92	21·14	19·2
Czech Republic	75·97	96·5	77·73	69·61	67·4	46·97	45·82	52·82	57·75	54·54
Hungary	77·47	97·05	76·07	66·98	65·48	55·59	49·64	50·4	46·2	45·72
Macedonia	21·29	20·86	20·88	19·51	17·95	16·42	14·28	11·72	12·12	11·1
Montenegro	5·56	5·52	5·53	5·64	5·05	4·68	4·24	3·95	3·93	3·68
Poland	276·09	332·38	351·04	338·56	272·13	209·65	190·34	190·13	206·21	187·9
Romania	216·58	221·66	208·41	192·13	148·84	119·27	110·97	109·25	90·34	88·88
Serbia	75·52	80·91	78·73	76·88	70·74	66·36	51·64	47·87	43·57	41·23
Slovakia	41·29	49·71	48·05	45·61	40·95	30·94	26·97	27·27	32·77	28·18
Slovenia	14·41	15·36	15·33	12·98	11·41	9·71	9·17	9·55	11·47	10·25
Central Asia	724·92	761·62	856·56	995·77	1010·7	838·25	747·2	830·56	977·91	1018·48
Armenia	31·64	35·65	43·49	45·92	40·63	25·29	20·02	20·72	22·2	23·84
Azerbaijan	75·68	74·65	84·19	95·35	97·7	82	67·73	84·97	107·45	99·17
Georgia	46·9	49	49·09	50·73	46·45	31·2	27·15	31·07	35·66	34·08
Kazakhstan	199·96	200·51	204·42	215·38	192·4	144·86	123·1	156·7	195·91	202·61
Kyrgyzstan	50·1	50·29	55·85	65·91	65·93	57·17	51·15	56·6	73·65	78·6
Mongolia	26·57	27·99	31·87	35·97	34·92	27	24·45	26·38	34·45	39·54
Tajikistan	57·04	61·78	73·06	92·37	101·63	88·32	80·98	86·56	105·79	128·78
Turkmenistan	35·3	38·9	45·69	57·03	64·2	59·19	51·47	52·81	58·38	61·83
Uzbekistan	201·72	222·85	268·92	337·12	366·82	323·21	301·15	314·76	344·41	350·04
Latin America and Caribbean	4578·28	4968·16	5388·25	5542·08	5678·9	5651·26	5526·24	5353·41	5288·48	5027·55
Central Latin America	1923·82	2127·32	2317·72	2461·16	2625·09	2669·87	2632·42	2547·05	2479·97	2305·45
Colombia	350·1	377·27	407·49	442·28	459·8	453·93	432·54	424·6	407·01	346·67
Costa Rica	29·88	30·37	35·87	41·57	40·84	39·12	38·26	36·25	34·51	29·68
El Salvador	74·74	83·06	82·13	77·17	79·08	79·36	74·36	67·41	60·32	52·76
Guatemala	125·69	137·08	146·27	161·68	180·56	198·55	209·94	211·84	206·21	211·83
Honduras	64·2	71·53	79·02	87·48	92·19	91·62	89·93	90·61	96·71	100·4
Mexico	1011·83	1129·72	1237·43	1307·43	1376·22	1419·48	1385·26	1304·05	1260·95	1180·49
Nicaragua	39	44·06	50·73	57·46	64·39	72·11	74·35	72·49	68·08	60·32
Panama	27·95	28·04	28·66	29·34	31·25	34·03	34·31	34·65	37·01	34·38
Venezuela	200·44	226·19	250·12	256·74	300·77	281·67	293·48	305·16	309·16	288·9
Andean Latin America	445·99	503·7	559·45	596·44	613·49	619·91	608·56	612·82	659·02	713·61
Bolivia	98·08	108·67	118·72	123·5	125·79	132·1	136·4	137·86	140·59	148·77
Ecuador	110·8	129	148·67	160·42	167·31	172·79	171·06	174·03	181·88	188·86
Peru	237·11	266·03	292·06	312·52	320·39	315·02	301·11	300·93	336·55	375·99
Caribbean	450·32	429·86	419·05	437·11	446·03	430·17	429·88	417·15	421·53	401·83
Antigua and Barbuda	0·94	0·82	0·72	0·61	0·55	0·57	0·67	0·67	0·64	0·62
The Bahamas	2·47	2·61	2·83	2·89	3·08	3·2	2·82	3·05	3·33	3·47
Barbados	2·61	2·46	2·39	2·26	1·98	1·69	1·59	1·66	1·6	1·46
Belize	2·8	2·92	3·16	3·84	3·8	3·24	3·91	4·33	4·62	5·07
Bermuda	0·53	0·39	0·35	0·38	0·41	0·44	0·43	0·44	0·44	0·46
Cuba	135·62	96·77	69·68	89·31	94·02	74·2	75·08	59·45	66·86	56·08
Dominica	1·49	1·1	0·98	0·93	0·86	0·79	0·64	0·6	0·57	0·57
Dominican Republic	86·52	96·91	103·48	94·61	102·67	110·47	109·15	107·39	103·82	94·79

Appendix Table 15B. Live births (in thousands) by location, males, 1970, 1975, 1980, 1985, 1990, 1995, 2000, 2005, 2010, 2016

Country	1970	1975	1980	1985	1990	1995	2000	2005	2010	2016
Grenada	1·46	1·45	1·36	1·69	1·31	1·22	1·07	0·98	0·94	0·87
Guyana	11·28	12·12	12·46	11·21	11·55	11·09	9·81	8·22	6·91	6·98
Haiti	105·46	112·99	122·45	133·01	135·45	138·74	146·77	158·94	164·34	167·72
Jamaica	35·6	33·52	34·09	35·12	35·69	34·77	32·74	29·77	28·84	28·82
Puerto Rico	36·74	40·1	36·82	32·34	31·74	28·84	25·96	23·14	22·09	21·27
Saint Lucia	2·4	2·39	2·29	2·22	1·99	1·69	1·45	1·25	1·06	0·95
Saint Vincent and the Grenadines	1·99	1·93	1·86	1·73	1·5	1·32	1·11	1	0·97	0·9
Suriname	6·89	5·73	5·78	6·16	6·1	6·31	6·22	5·9	5·36	4·8
Trinidad and Tobago	14·24	14·33	17·09	17·6	12·23	10·62	9·64	9·61	8·51	6·45
Virgin Islands, U.S.	1·29	1·33	1·25	1·19	1·11	0·98	0·81	0·75	0·63	0·55
Tropical Latin America	1758·15	1907·28	2092·03	2047·38	1994·29	1931·31	1855·38	1776·39	1727·96	1606·66
Brazil	1715·03	1860·34	2040·58	1985·47	1922·77	1855·74	1780·86	1705·26	1659·99	1541·82
Paraguay	43·12	46·94	51·45	61·91	71·51	75·57	74·52	71·13	67·97	64·85
Southeast Asia, East Asia, and Oceania	19526·09	17861·85	17358·63	18211·87	18237·45	16442·91	14892·22	14049·06	13947·58	12354·37
East Asia	14330·48	12406·04	11586·4	12234·48	12106·46	10135·49	8535·7	7701·75	7572·25	6405·75
China	13831·93	11939·75	11135·32	11766·59	11611·84	9577·57	7995·5	7267·4	7162·53	5994·84
North Korea	301·31	271·98	245·52	303·88	323·3	388·6	391·2	326·68	313·11	310·78
Taiwan (Province of China)	197·24	194·31	205·56	164·01	171·31	169·32	148·99	107·67	96·62	100·14
Southeast Asia	5115·42	5365·7	5677·11	5875·2	6019·91	6183·9	6224·15	6210·28	6232·71	5796·15
Cambodia	131·79	139·22	130·37	135·73	145·48	158·21	165·7	188·07	198·45	200·42
Indonesia	2166·64	2196·14	2286·73	2285·26	2313·92	2451·13	2536·82	2530·13	2544·71	2258·71
Laos	64·75	71·33	67·73	74·65	84·67	94·57	96·2	99·7	113·7	129·41
Malaysia	180·42	194·84	215·19	252·5	262·13	277·39	274·78	250·63	271·17	261·43
Maldives	1·57	1·61	1·79	2·1	2·39	2·53	2·57	3·05	3·6	3·04
Mauritius	10·83	11·8	12·04	9·11	11·28	11·07	10·03	8·67	6·94	6·78
Myanmar	388·76	441·71	486·64	530·56	548·56	549·55	557·39	555·84	522·59	468·91
Philippines	662·73	745·43	842·65	918·7	997·17	1063·24	1118·94	1162·03	1187·29	1247·26
Sri Lanka	183·63	190·23	218·12	195·48	186·36	178·54	178·63	188·73	180	138·96
Seychelles	0·98	0·92	0·97	0·96	0·8	0·82	0·73	0·86	0·9	0·8
Thailand	716·45	748·42	706·1	626·95	612·77	597·07	528·17	438·99	365·99	286·51
Timor-Leste	11·59	12·7	12·02	12·92	13·16	13·6	9·74	13·46	18·11	16·92
Vietnam	595·29	611·36	696·77	830·28	841·23	786·17	744·45	770·12	819·26	777
Oceania	80·19	90·1	95·12	102·19	111·08	123·52	132·37	137·03	142·63	152·47
American Samoa	0·56	0·62	0·66	0·75	0·89	0·88	0·9	0·85	0·8	0·78
Federated States of Micronesia	1·59	1·49	1·81	1·92	1·83	1·69	1·44	1·13	1·01	1·01
Fiji	9·38	10·51	10·66	10·45	8·81	7·91	7	6·37	5·88	5·12
Guam	1·19	1·35	1·5	1·61	1·83	1·87	1·85	1·73	1·69	1·79
Kiribati	1·14	1·31	1·45	1·49	1·61	1·54	1·51	1·5	1·5	1·45
Marshall Islands	0·48	0·57	0·62	0·72	0·82	0·95	0·97	0·99	1·04	1·07
Northern Mariana Islands	0·21	0·26	0·31	0·57	0·99	1·25	1·38	1·47	1·53	1·64
Papua New Guinea	55·68	63·16	66·03	71·76	80·6	92·56	101·46	106·14	111·56	122·31
Samoa	2·93	2·94	3	2·99	2·91	2·91	2·88	2·94	2·96	2·81
Solomon Islands	3·48	4	4·75	5·29	5·85	6·86	7·81	8·41	8·88	8·76
Tonga	1·55	1·54	1·6	1·64	1·72	1·62	1·53	1·6	1·54	1·33
Vanuatu	2	2·35	2·72	2·99	3·23	3·5	3·63	3·88	4·23	4·39
North Africa and Middle East	4145·44	4592·45	5046·38	5436·69	5541·43	5546·88	5777·32	6161·97	6754·26	6475·75
North Africa and Middle East	4145·44	4592·45	5046·38	5436·69	5541·43	5546·88	5777·32	6161·97	6754·26	6475·75
Afghanistan	274·37	303·14	312·06	269·01	269·19	344·73	387·42	464·44	484·37	596·9
Algeria	305·91	335·76	370·71	408·16	432·61	476·44	529·66	545·24	516·24	445·76
Bahrain	3·73	4·46	5·56	6·96	7·15	6·79	6·81	6·48	9·74	9·56
Egypt	754·48	801·8	843·95	883·17	878·21	840·85	897·26	989·15	1136·92	1093·83
Iran	686·78	772·72	893·7	1023·73	999·48	711·53	653·63	786·25	883·12	789·87
Iraq	231·58	262·02	294·7	324·96	344·32	387·76	439·36	516·11	728·87	854·53
Jordan	47·42	50·78	47·73	53·89	56·63	73·8	73·41	80·07	95·91	101·26
Kuwait	19·28	23·72	26·95	28·02	21·96	23·14	21·37	24·44	30·13	27·38
Lebanon	48·04	50·57	46·05	41·27	35·44	33·59	29·09	27·29	28·41	35·16
Libya	39·04	42·59	46·97	52·13	57·14	64·17	70·08	70·84	62·87	40·14
Morocco	283·63	303·15	338·53	371·95	385·5	362·74	325·51	291·02	263·49	228·15
Palestine	28·2	30·75	32·41	36·93	43·98	52·97	59·41	65·9	98·62	119·32
Oman	20·19	23·68	28·61	34·21	37·66	41·52	47·49	47·03	44·24	46·47
Qatar	2·05	2·52	3·85	4·94	5·69	5·5	5·49	7·21	11·04	13·33
Saudi Arabia	148·72	184·47	224·42	299·69	326·13	329·28	356·7	337·05	297·84	228·26
Sudan	195·71	218·66	247·54	273·69	310·24	393·3	458·55	475·27	471·49	446·39
Syria	140·62	164·87	186·56	209·67	228·85	247·83	258·2	250·42	260·91	152·08
Tunisia	92·05	101·02	112·31	117·72	112·13	97·9	85·32	87·63	95·51	77·86
Turkey	660·46	736·63	772·56	742·78	683·07	685·67	671·11	650·52	657·38	608·7
United Arab Emirates	5·84	10·28	20·02	28·13	29·11	35·23	39	53·86	88·05	78·41
Yemen	157·32	168·85	191·18	225·67	276·94	332·15	362·47	385·75	489·12	482·38
South Asia	13387·67	14506·07	15884·67	17085	17704·33	17863·98	18002·85	18104·39	17912·62	15777·31
South Asia	13387·67	14506·07	15884·67	17085	17704·33	17863·98	18002·85	18104·39	17912·62	15777·31
Bangladesh	1358·72	1334·23	1454·64	1640·89	1752·32	1691·91	1656·36	1677·48	1619·69	1438·01
Bhutan	7·1	7·98	8·67	9·32	10·43	9·58	10·16	10·18	9·3	7·81
India	10429·07	11389·3	12343·67	12968·48	13389·97	13619·19	13876·67	13919·64	13358·81	11452·73
Nepal	255·9	281·85	309·96	341·02	373·07	388·5	402·07	367·31	393·45	427·42
Pakistan	1336·88	1492·69	1767·73	2125·29	2178·54	2154·8	2057·59	2129·78	2531·37	2451·34
Sub-Saharan Africa	6637·09	7373·19	8238·5	9139·73	10201·52	11279·11	12602·86	14304·64	16121·56	18017·28
Southern Sub-Saharan Africa	478·54	536·03	599·5	676·42	740·1	764·2	800·18	847·68	892·38	938·74
Botswana	13·22	15	17·45	19·43	20·54	22·2	23·31	24·73	26·97	27·67
Lesotho	19·48	20·95	22·53	23·87	23·98	25·64	26·63	26·83	27·98	28·33
Namibia	15·64	17·48	18·6	20·85	27·2	29·77	31·13	30·15	32·66	36·57
South Africa	321·39	358·01	398·5	440·4	470·21	482·23	504·39	531·99	527·67	544·1
Swaziland	9·04	10·03	11·26	12·89	15·37	15·61	17·13	18·39	22·32	22·5
Zimbabwe	99·77	114·55	131·16	158·97	182·79	188·74	197·58	215·6	254·78	279·57
Western Sub-Saharan Africa	2801·3	3057·21	3362·94	3699·2	4149·98	4638·44	5258·36	6031·97	6860·57	7546·9
Benin	69·02	75·56	84·84	95·72	106·96	123·99	140·89	166·74	190·17	223·84
Burkina Faso	142·11	152·4	165·19	185·05	209·99	231·51	265·31	308·73	346·43	376·09
Cameroon	158·07	170·21	187·94	213·41	242·5	268·87	300·04	343·18	396·72	445·18
Cape Verde	4·56	4·45	4·97	6·05	6·27	6·71	6·98	7·58	7·86	7·68
Chad	88·12	98·77	107·14	117·89	135·1	155·14	181·81	219·57	261·92	323·49
Cote d'Ivoire	118·49	141·86	173·48	216·39	239·79	260·88	297·97	325·42	360·72	417·73
The Gambia	12·79	14·44	16·5	18·37	22·68	26·25	29·97	33·19	36·48	40·94
Ghana	196·84	212·05	225·5	262·97	286·16	295·13	313·93	372·71	453·44	478·9
Guinea	91·74	94·76	96·99	106·64	123·06	155·65	171·32	187·05	208·71	236·73
Guinea-Bissau	15·37	17·12	18·5	19·72	21·19	23·13	25·64	28·68	31·77	35·13
Liberia	36·33	40·28	45·38	51·04	48·04	47	63·76	66·61	74·39	81·33
Mali	150·25	162·55	175·49	190·68	200·03	228·18	262·21	299·72	336·19	378·78
Mauritania	22·22	24·76	27·89	31·38	34·89	38·76	43·6	49·55	53·58	57·16
Niger	125·53	139·68	157·61	177·97	202·91	237·36	275·92	317·93	370·29	455·83
Nigeria	1368·09	1485·08	1632·27	1733·09	1963·63	2214·48	2526·81	2885·46	3255·25	3453·26
Sao Tome and Principe	1·52	1·7	1·93	2·11	2·27	2·5	2·76	2·99	3·28	3·58
Senegal	95·08	107·72	117·43	132	150·29	166·82	181·24	217·98	252·74	285·36
Sierra Leone	58·07	62·23	66·99	71·74	78·04	74·59	77·71	97·42	109·8	122·59
Togo	47·1	51·61	56·9	66·96	76·15	81·48	90·47	101·46	110·84	123·29
Eastern Sub-Saharan Africa	2614·46	2964·17	3363·92	3747·59	4170·1	4548·8	5032·34	5663·78	6323·11	7076·27
Burundi	86·66	90·84	105·99	119·26	129·7	131·59	133·42	166·07	208·46	250·77
Comoros	4·82	5·39	6·39	7·08	7·61	8·15	9·25	10·26	10·48	9·86
Djibouti	4·39	5·95	9·05	10·66	14·38	15·8	16·92	17·78	18·42	18·45
Eritrea	44·28	50·15	56·24	62·74	67·86	60·31	65·65	81·71	87·12	83·76
Ethiopia	638·37	716·35	765·1	857·33	995·43	1132·83	1246·11	1379·77	1507·27	1744·59
Kenya	284·18	338·9	394·43	449·15	497·97	545·32	592·71	645·11	686·02	706·31
Madagascar	163·74	186·74	211·61	237·2	263·85	299·3	336·28	369·83	401·88	450·62

Appendix Table 15B. Live births (in thousands) by location, males, 1970, 1975, 1980, 1985, 1990, 1995, 2000, 2005, 2010, 2016

Country	1970	1975	1980	1985	1990	1995	2000	2005	2010	2016
Malawi	133·15	145·97	161·66	179·44	233·51	227·56	250·05	274·56	319·06	365·25
Mozambique	218·07	245·8	282·52	291·14	275·65	338·41	389·75	452·2	517·69	560·36
Rwanda	95·47	113·98	136·9	156·31	164·52	136·52	163·97	189·7	197·82	202·29
Somalia	65·54	73·74	114·63	110·99	111·98	106·62	115·22	125·29	136·22	152·5
South Sudan	111·7	121·27	135·41	155·47	161·5	150·04	180·46	217·61	271·52	347·26
Tanzania	381·06	434·08	489·34	545·79	605·6	687·94	730·01	817·03	920·64	1003·32
Uganda	267·05	302·17	344·28	390·98	449·08	504·91	576·09	664·71	756·71	856·77
Zambia	115·98	132·84	150·37	174·06	191·45	203·49	226·44	252·17	283·81	324·15
Central Sub-Saharan Africa	742·79	815·78	912·14	1016·52	1141·35	1327·67	1511·98	1761·2	2045·5	2455·37
Angola	152·94	173·44	201·33	237·71	267·96	312·42	357·29	416·24	484·88	562·6
Central African Republic	36·5	39·1	43·43	50·1	54·13	61·32	67·45	71·58	79·5	90·6
Congo	29·17	32·88	36·79	41·65	47·31	53·72	61·14	66·97	77·27	81·93
Democratic Republic of the Congo	505·36	553·92	613·83	665·84	747·88	873·97	997·75	1175·04	1369·24	1681·75
Equatorial Guinea	6·31	3·98	3·79	7·11	8·27	8·56	8·36	8·85	9·92	11·5
Gabon	12·51	12·47	12·97	14·11	15·81	17·68	20	22·52	24·69	26·99

Appendix Table 15C. Live births (in thousands) by location, females, 1970, 1975, 1980, 1985, 1990, 1995, 2000, 2005, 2010, 2016

Country	1970	1975	1980	1985	1990	1995	2000	2005	2010	2016
Global	55450·35	55980·2	58603·31	61660·62	62722·52	60962·28	60619·1	61950·34	64312·58	62295
High-income	6702·26	5974·26	6054·12	5820·2	6017·15	5712·71	5731·45	5769·18	5771·49	5652·27
High-income North America	1929·53	1679·7	1934·73	2013·48	2219·3	2101·03	2137·22	2214·66	2114·87	2171·88
Canada	173·54	171·25	178·91	183·41	196·73	179·46	160·98	168·67	181·74	195·87
Greenland	0·74	0·49	0·52	0·5	0·57	0·5	0·43	0·37	0·34	0·35
United States	1755·25	1507·95	1755·31	1829·57	2022	1921·07	1975·81	2045·62	1932·8	1975·66
Australasia	159·73	137·24	135·45	143·61	154·39	151·1	147·35	154·37	172·95	174
Australia	129·13	110·5	110·97	118·26	125·76	123·28	119·73	125·94	142·79	146·22
New Zealand	30·6	26·73	24·48	25·35	28·63	27·82	27·62	28·43	30·17	27·78
High-income Asia Pacific	1446·25	1326·86	1205·55	1018·37	944·23	921·69	860·91	756·53	766·42	667·91
Brunei	1·79	2·21	2·81	3·04	3·45	3·74	3·93	3·48	3·25	3·18
Japan	961·9	891·52	762·76	695·69	597·21	578·77	571·75	529·09	523·11	455·19
Singapore	22·23	19·1	19·83	20·32	23·19	22·97	20·48	17·54	16·99	16·6
South Korea	460·33	414·04	420·15	299·32	320·38	316·22	264·76	206·42	223·07	192·93
Western Europe	2780·92	2404·21	2330·52	2196·59	2229·28	2090·25	2099·28	2157·13	2214·17	2141·24
Andorra	0·08	0·11	0·14	0·2	0·23	0·29	0·29	0·3	0·31	0·29
Austria	53·56	43·99	44·54	41·91	44·53	43·22	37·67	38·26	38·16	40·11
Belgium	68·3	57·03	59·61	56·04	60·6	56·78	56·81	59·21	62·24	63·33
Cyprus	6·55	5·18	5·93	5·88	5·84	4·92	3·94	4·13	4·79	5·06
Denmark	35·05	33·32	27·09	26·4	30·91	33·27	32·47	31·7	30·04	29·05
Finland	30·12	32·24	30·93	30·13	32·24	30·47	27·68	28·58	29·52	29·09
France	416·52	351·81	389·86	375·86	370·57	356·46	377·49	384·32	389·11	386·18
Germany	498·25	384·68	412·7	399·75	414·97	379·13	365·23	332·63	322·39	338·86
Greece	68·44	68·6	68·46	54·57	49·7	50·34	49·81	53·47	54·65	44·02
Iceland	1·97	2·09	2·14	1·84	2·25	2·11	2·05	2·11	2·31	2·09
Ireland	31·95	32·21	35·15	29·89	25·86	23·97	26·98	30·29	36·56	30·77
Israel	36·99	43·07	49·33	51·24	50·17	55·74	64·58	71·41	79·6	83·63
Italy	441·47	397·76	310·08	278·4	277·13	254·77	260·87	269·02	266·65	238·31
Luxembourg	2·13	1·89	2·08	2·03	2·41	2·68	2·73	2·64	2·78	3·18
Malta	2·65	2·91	2·77	2·77	2·83	2·43	1·95	1·76	1·89	1·83
Netherlands	111·52	84·95	87·35	87·78	95·92	91·77	99·42	91·67	89·31	86·45
Norway	30·79	26·15	24·57	25·12	29·49	29·44	28·35	28·07	29·49	30·23
Portugal	88·04	86·39	74·44	62·28	56·52	52·57	56·01	51·63	48·37	39·69
Spain	322·78	326·04	264·63	214·59	190·76	175·23	194·66	227·22	233·47	204·14
Sweden	53·81	48·94	46·69	48·79	60·29	48·18	44·24	50·59	55·99	57·43
Switzerland	47·23	37·38	35·88	36·54	40·72	39·96	36·82	35·85	39·1	41·69
United Kingdom	432·69	337·47	356·12	364·6	385·34	356·53	329·24	362·28	397·43	385·81
England	352·48	274·39	290·7	301·44	323·17	299·13	277·83	308·02	338·28	329·39
East Midlands	27·77	22·47	23·9	23·62	25·81	23·94	22·19	24·63	27·25	27·07
East of England	32·87	26·73	29·22	30·9	32·68	31·04	28·93	32·53	35·63	37·04
Greater London	56·37	43·95	46·03	49·46	55·22	51·77	51·65	57·97	66·73	64·45
North East England	21·47	15·88	16·52	16·24	16·6	15·01	13·11	14·09	14·64	14·14
North West England	57·43	42·51	44·22	45·22	47·23	41·54	37·44	40·75	44·12	41·74
South East England	47·99	39·02	42·58	45·63	49·12	45·96	42·42	47·23	52·51	49·31
South West England	27·74	22·68	24·48	25·55	27·69	26·62	23·97	26·66	29·72	29·18
West Midlands	42·93	32·23	33·28	33·9	35·76	32·78	30·28	33·28	35·7	34·11
Yorkshire and the Humber	37·95	28·92	30·48	30·93	33·11	30·47	27·84	30·89	31·98	32·33
Northern Ireland	14·78	13·7	14·19	13·83	12·73	11·55	10·54	11·27	12·48	10·77
Scotland	42·56	32·01	33·45	31·97	31·87	29·2	25·78	26·69	29	29·1
Wales	22·94	17·38	17·78	17·36	17·57	16·64	15·08	16·29	17·66	16·61
Southern Latin America	385·83	426·25	447·87	448·16	469·95	448·64	486·69	486·49	503·07	497·23
Argentina	237·7	285·68	308·12	301·42	304·17	286·93	337·08	346·8	359·91	355·78
Chile	120·4	114·35	116·97	123·44	141·05	134·06	123·41	116·16	120·25	117·83
Uruguay	27·72	26·23	22·78	23·3	24·73	27·65	26·2	23·53	22·91	23·63
Central Europe, Eastern Europe, and Central Asia	3070·15	3313·35	3409·5	3636·15	3170·43	2427·36	2177·92	2362·43	2700·87	2744·84
Eastern Europe	1457·11	1551·93	1600·11	1740·5	1404·87	996·77	907·84	1021·93	1220·88	1258·57
Belarus	71·97	71·81	76·09	81·51	68·42	49·58	45·24	46·24	53·7	55·99
Estonia	10·58	10·4	10·9	11·57	10·21	6·54	6·31	7·08	7·33	6·64
Latvia	16·88	16·69	17·37	20·09	17·85	10·15	9·68	10·75	9·53	10·9
Lithuania	27·41	25·35	24·8	28·46	27·49	19·33	16·15	14·47	15·46	14·33
Moldova	36·73	39·81	41·9	45	37·86	27·17	20·66	22·23	22·23	21·8
Russia	938·46	1026·98	1064·68	1175·7	928·33	649·03	622·42	707·15	867·4	916·39
Ukraine	355·08	360·89	364·37	378·17	314·71	234·97	187·38	214·01	245·24	232·53
Central Europe	927·07	1040·36	997·94	950·94	814·49	646·45	571·05	564·66	565·95	529·94
Albania	27·6	30·35	34·43	38·95	41·2	33·1	24·3	18·46	16·76	18·99
Bosnia and Herzegovina	40·68	37·13	35·09	38·42	34·67	24·35	16·23	16·02	16·75	15·35
Bulgaria	65·96	70·29	61·38	58·97	49·4	34·77	34·51	34·32	34·95	31·09
Croatia	31·65	31·82	32·3	30·84	26·6	24·46	19·8	19·75	19·96	18·13
Czech Republic	71·81	91·21	73·47	65·8	63·71	44·39	43·3	49·93	54·58	51·55
Hungary	73·09	91·56	71·76	63·19	61·78	52·44	46·83	47·55	43·59	43·13
Macedonia	20·2	19·79	19·81	18·51	17·03	15·58	13·55	11·12	11·5	10·53
Montenegro	5·15	5·12	5·12	5·22	4·67	4·34	3·93	3·66	3·67	3·48
Poland	261·45	314·76	332·42	320·61	257·7	198·53	180·25	180·05	195·27	177·93
Romania	204·71	209·51	196·99	181·6	140·68	112·73	104·89	103·26	85·39	84·01
Serbia	71·79	76·91	74·84	73·08	67·24	63·08	49·09	45·5	41·42	39·19
Slovakia	39·32	47·35	45·76	43·44	39	29·46	25·69	25·98	31·21	26·84
Slovenia	13·67	14·57	14·55	12·31	10·82	9·21	8·7	9·06	10·88	9·72
Central Asia	685·97	721·06	811·45	944·71	951·07	784·13	699·03	775·84	914·04	956·33
Armenia	29·94	33·89	41·42	43·4	37·73	21·98	17·06	18	19·51	21·11
Azerbaijan	71·26	70·29	79·27	89·78	90·88	72·56	57·89	72·87	92·79	88·15
Georgia	43·59	45·54	45·62	47·14	43·17	28·08	24·46	27·99	32·24	30·96
Kazakhstan	187·76	188·27	191·94	203·19	181·51	136·28	116·46	147·83	184·65	191·14
Kyrgyzstan	47·44	47·62	52·88	62·71	62·56	53·68	48·71	53·4	69·62	74·29
Mongolia	25·79	27·18	30·94	34·92	33·91	26·22	23·74	25·61	33·45	38·39
Tajikistan	54·33	58·84	69·58	87·97	96·79	84·12	77·12	82·44	100·75	122·65
Turkmenistan	33·75	37·19	43·68	54·52	61·38	56·59	49·21	50·49	55·82	59·11
Uzbekistan	192·11	212·24	256·11	321·06	343·15	304·63	284·37	297·22	325·22	330·54
Latin America and Caribbean	4359·47	4731·04	5131·32	5277·73	5407·93	5381·65	5262·62	5098·14	5036·28	4787·87
Central Latin America	1832·21	2026·02	2207·35	2343·96	2500·09	2542·73	2507·06	2425·76	2361·88	2195·66
Colombia	333·42	359·31	388·08	421·22	437·9	432·32	411·94	404·38	387·63	330·16
Costa Rica	28·46	28·92	34·16	39·59	38·9	37·26	36·43	34·52	32·87	28·27
El Salvador	71·18	79·1	78·22	73·49	75·32	75·58	70·82	64·2	57·45	50·25
Guatemala	119·7	130·55	139·3	153·98	171·96	189·09	199·94	201·75	196·39	201·74
Honduras	61·15	68·12	75·26	83·31	87·8	87·26	85·65	86·3	92·11	95·62
Mexico	963·65	1075·93	1178·51	1245·18	1310·68	1351·88	1319·29	1241·95	1200·9	1124·28
Nicaragua	37·14	41·96	48·31	54·72	61·32	68·68	70·81	69·03	64·84	57·45
Panama	26·62	26·71	27·3	27·95	29·76	32·41	32·68	33	35·25	32·75
Venezuela	190·89	215·42	238·21	244·52	286·45	268·26	279·5	290·63	294·44	275·15
Andean Latin America	424·76	479·72	532·81	568·04	584·27	590·39	579·58	583·64	627·64	679·63
Bolivia	93·41	103·5	113·07	117·62	119·8	125·81	129·9	131·29	133·89	141·69
Ecuador	105·53	122·86	141·59	152·78	159·35	164·56	162·91	165·75	173·22	179·86
Peru	225·82	253·36	278·15	297·64	305·13	300·02	286·77	286·6	320·53	358·08
Caribbean	428·08	408·84	398·76	415·84	424·24	409·19	408·95	396·94	401·1	382·42
Antigua and Barbuda	0·91	0·8	0·7	0·59	0·53	0·55	0·65	0·65	0·62	0·6
The Bahamas	2·34	2·47	2·68	2·74	2·91	3·03	2·67	2·89	3·15	3·28
Barbados	2·52	2·38	2·31	2·18	1·91	1·63	1·53	1·61	1·54	1·41
Belize	2·71	2·83	3·07	3·73	3·69	3·15	3·8	4·2	4·49	4·92
Bermuda	0·53	0·39	0·35	0·38	0·4	0·4	0·42	0·43	0·44	0·46
Cuba	128·3	91·55	65·92	84·49	88·95	70·2	71·03	56·25	63·25	53·05
Dominica	1·42	1·04	0·93	0·88	0·82	0·75	0·61	0·57	0·54	0·54
Dominican Republic	82·4	92·29	98·55	90·1	97·78	105·21	103·95	102·27	98·88	90·28

Appendix Table 15C. Live births (in thousands) by location, females, 1970, 1975, 1980, 1985, 1990, 1995, 2000, 2005, 2010, 2016

Country	1970	1975	1980	1985	1990	1995	2000	2005	2010	2016
Grenada	1·39	1·38	1·3	1·61	1·25	1·16	1·02	0·93	0·89	0·83
Guyana	10·74	11·54	11·87	10·68	11	10·56	9·35	7·83	6·58	6·65
Haiti	100·44	107·61	116·62	126·67	129	132·13	139·79	151·37	156·51	159·73
Jamaica	33·9	31·93	32·47	33·45	33·99	33·11	31·18	28·35	27·47	27·44
Puerto Rico	34·89	38·08	34·96	30·71	30·15	27·39	24·66	21·97	20·98	20·2
Saint Lucia	2·33	2·32	2·22	2·15	1·93	1·64	1·41	1·22	1·03	0·93
Saint Vincent and the Grenadines	1·94	1·87	1·81	1·68	1·45	1·28	1·08	0·97	0·94	0·87
Suriname	6·38	5·31	5·35	5·71	5·65	5·84	5·76	5·46	4·98	4·48
Trinidad and Tobago	13·72	13·8	16·46	16·95	11·79	10·23	9·28	9·26	8·2	6·22
Virgin Islands, U.S.	1·21	1·25	1·18	1·12	1·05	0·92	0·76	0·71	0·59	0·52
Tropical Latin America	1674·43	1816·46	1992·41	1949·88	1899·32	1839·34	1767·02	1691·8	1645·68	1530·15
Brazil	1633·36	1771·75	1943·41	1890·92	1831·21	1767·37	1696·05	1624·06	1580·95	1468·4
Paraguay	41·07	44·71	49	58·96	68·11	71·97	70·97	67·75	64·73	61·76
Southeast Asia, East Asia, and Oceania	18344·1	16795·78	16328·5	16931·37	16661·26	14919·82	13437·63	12620·96	12577·64	11225·36
East Asia	13399·4	11601·01	10833·89	11239·03	10832·54	8930·33	7403·5	6623·64	6563·75	5602·27
China	12927·16	11158·77	10406·99	10795·84	10369·3	8403·32	6894·8	6213·77	6176·87	5214·82
North Korea	286·96	259·03	233·83	289·41	307·91	370·1	372·58	311·13	298·2	295·98
Taiwan (Province of China)	185·28	183·21	193·07	153·77	155·33	156·91	136·13	98·74	88·67	91·47
Southeast Asia	4870·09	5110·96	5406·17	5597·35	5725·51	5874·71	5911·22	5870·09	5881·47	5481·49
Cambodia	126·6	133·86	124·28	131·14	140·43	152·57	160·25	176·75	189·18	191·06
Indonesia	2063·47	2091·56	2177·84	2176·44	2203·73	2334·41	2416·02	2409·64	2423·54	2151·15
Laos	61·66	67·93	64·5	71·1	80·64	90·07	91·62	94·96	108·28	123·25
Malaysia	170·21	183·81	203·01	238·21	247·29	261·69	259·23	236·44	255·82	246·63
Maldives	1·49	1·52	1·69	1·99	2·21	2·38	2·43	2·87	3·26	2·8
Mauritius	10·39	11·33	11·55	8·74	10·82	10·62	9·62	8·32	6·66	6·51
Myanmar	377·43	428·84	472·47	515·11	532·58	533·55	541·16	539·65	507·37	455·26
Philippines	625·22	703·23	794·95	866·69	940·72	1003·06	1055·6	1096·25	1120·08	1176·66
Sri Lanka	176·91	182·91	208·73	186·88	179·36	171·02	170·94	180·77	173·08	133·23
Seychelles	0·93	0·87	0·92	0·91	0·76	0·77	0·69	0·81	0·84	0·76
Thailand	677·82	710·75	671·19	597·1	580·82	562·75	497·34	412·58	344·63	269·78
Timor-Leste	11·03	12·1	11·44	12·31	12·53	12·95	9·28	12·82	17·25	16·11
Vietnam	566·94	582·25	663·59	790·74	793·61	738·88	697·05	698·21	731·48	708·29
Oceania	74·61	83·81	88·44	95	103·21	114·78	122·9	127·23	132·42	141·6
American Samoa	0·52	0·58	0·61	0·7	0·83	0·85	0·81	0·79	0·74	0·72
Federated States of Micronesia	1·48	1·39	1·69	1·8	1·71	1·58	1·35	1·06	0·95	0·95
Fiji	8·85	9·92	10·06	9·86	8·31	7·46	6·61	6·01	5·55	4·83
Guam	1·12	1·28	1·41	1·52	1·73	1·77	1·75	1·64	1·6	1·69
Kiribati	1·09	1·25	1·36	1·39	1·5	1·44	1·41	1·41	1·41	1·37
Marshall Islands	0·46	0·54	0·59	0·68	0·78	0·9	0·92	0·95	0·99	1·02
Northern Mariana Islands	0·22	0·26	0·29	0·52	0·91	1·16	1·28	1·37	1·43	1·54
Papua New Guinea	51·55	58·48	61·14	66·44	74·63	85·7	93·94	98·28	103·3	113·25
Samoa	2·72	2·72	2·78	2·77	2·69	2·69	2·67	2·73	2·74	2·6
Solomon Islands	3·25	3·74	4·44	4·94	5·47	6·41	7·3	7·86	8·3	8·23
Tonga	1·48	1·47	1·52	1·56	1·64	1·55	1·46	1·52	1·47	1·27
Vanuatu	1·87	2·2	2·54	2·8	3·01	3·27	3·4	3·63	3·96	4·12
North Africa and Middle East	3940·23	4365·73	4798·3	5169·94	5268·4	5278·94	5491·03	5851·22	6407·96	6145·54
North Africa and Middle East	3940·23	4365·73	4798·3	5169·94	5268·4	5278·94	5491·03	5851·22	6407·96	6145·54
Afghanistan	258·84	285·98	294·4	253·78	253·95	325·22	365·49	438·15	456·95	563·11
Algeria	291·34	319·77	353·05	388·72	412·01	453·75	504·44	519·28	491·66	424·54
Bahrain	3·56	4·24	5·3	6·64	6·95	6·49	6·6	6·24	9·38	9·2
Egypt	711·77	756·42	796·18	833·18	828·5	793·25	841·71	927·91	1066·53	1031·91
Iran	654·08	735·92	851·14	974·98	951·88	684·16	626·68	748·81	839·46	750·83
Iraq	217·45	246·03	276·71	305·13	323·31	364·09	412·54	484·61	684·38	802·38
Jordan	44·99	48·17	45·29	51·13	53·73	70·02	69·65	75·97	90·99	96·07
Kuwait	18·65	22·88	26·07	27·07	20·97	22·14	20·55	23·45	28·92	26·28
Lebanon	45·76	48·16	43·85	39·31	33·75	31·99	27·7	25·99	27·05	33·49
Libya	37·04	40·4	44·57	49·46	54·01	60·48	65·99	66·89	59·32	37·87
Morocco	270·13	288·71	322·41	352·9	364·37	341·89	306·51	274·8	248·57	215·23
Palestine	26·85	29·28	30·87	35·17	41·89	50·45	56·58	62·76	93·93	113·64
Oman	19·23	22·55	27·25	32·58	35·86	39·54	45·23	44·79	42·13	44·26
Qatar	1·95	2·4	3·67	4·68	5·38	5·3	5·23	6·92	10·56	12·76
Saudi Arabia	144·38	179·1	217·88	290·96	316·63	319·69	346·31	327·24	289·17	221·62
Sudan	188·19	210·25	238·02	263·17	298·31	378·18	440·91	456·99	453·36	429·22
Syria	133·92	157·02	177·68	199·68	217·95	236·03	245·9	238·49	248·49	144·84
Tunisia	87·67	96·21	106·96	112·12	106·79	93·24	81·25	83·46	90·96	74·15
Turkey	629	701·56	735·77	707·41	650·54	653·02	639·15	619·54	626·08	579·71
United Arab Emirates	5·59	9·84	19·16	26·95	27·85	33·68	37·39	51·54	84·26	75·03
Yemen	149·83	160·81	182·08	214·92	263·75	316·33	345·21	367·38	465·83	459·41
South Asia	12641	13695·52	14941·78	16015·19	16364·41	16372·37	16376·92	16470·05	16296·99	14389·91
South Asia	12641	13695·52	14941·78	16015·19	16364·41	16372·37	16376·92	16470·05	16296·99	14389·91
Bangladesh	1295·25	1271·91	1386·69	1564·24	1670·46	1612·88	1578·99	1599·12	1544·03	1370·84
Bhutan	6·83	7·68	8·34	8·97	10·03	9·21	9·77	9·78	8·94	7·51
India	9838·75	10744·63	11590·3	12120·07	12284·38	12392·35	12535·39	12551·53	12045·82	10355·09
Nepal	244·88	269·71	296·61	326·34	353·96	362·75	375·42	348·49	369·43	401·34
Pakistan	1255·28	1401·59	1659·84	1995·58	2045·58	1995·19	1877·36	1961·12	2328·77	2255·14
Sub-Saharan Africa	6393·16	7104·54	7939·79	8810·04	9832·95	10869·43	12141·53	13778·35	15521·35	17349·2
Southern Sub-Saharan Africa	465·21	522·14	583·89	658·74	720·65	743·95	778·35	824·85	868·67	913·9
Botswana	12·84	14·57	16·94	18·86	19·94	21·56	22·64	24·01	26·18	26·86
Lesotho	18·91	20·34	21·87	23·18	23·28	24·9	25·85	26·05	27·17	27·5
Namibia	15·18	16·97	18·06	20·25	26·4	28·91	30·22	29·27	31·71	35·5
South Africa	312·64	348·22	387·5	428·09	456·89	468·4	489·3	516·31	512·16	528·1
Swaziland	8·78	9·74	10·93	12·51	14·93	15·16	16·63	17·85	21·67	21·84
Zimbabwe	96·87	112·31	128·58	155·85	179·21	185·03	193·71	211·37	249·78	274·09
Western Sub-Saharan Africa	2671·22	2915·45	3207·12	3529	3959·07	4424·86	5015·69	5753·67	6544·08	7201·51
Benin	66·31	72·58	81·5	91·95	102·74	119·11	135·34	160·18	182·68	215·03
Burkina Faso	135·86	145·7	157·93	176·91	200·76	221·33	253·65	295·15	331·19	359·55
Cameroon	153·47	165·25	182·46	207·2	235·44	261·04	291·31	333·18	385·16	432·22
Cape Verde	4·42	4·32	4·82	5·87	6·09	6·52	6·78	7·36	7·63	7·46
Chad	85·55	95·89	104·02	114·46	131·17	150·62	176·51	213·18	254·3	314·07
Cote d'Ivoire	115·04	137·73	168·43	210·09	232·8	253·28	289·29	315·94	350·22	405·56
The Gambia	12·42	14·02	16·02	17·84	22·02	25·49	29·09	32·22	35·41	39·75
Ghana	186·93	201·37	214·15	249·73	271·76	280·28	298·13	353·95	430·61	454·8
Guinea	89·59	92·54	94·72	104·14	120·18	152	167·3	182·67	203·82	231·18
Guinea-Bissau	14·92	16·62	17·96	19·14	20·58	22·46	24·9	27·85	30·84	34·11
Liberia	34·47	38·21	43·06	48·43	45·58	44·59	60·49	63·2	70·57	77·17
Mali	143·1	154·81	167·13	181·6	190·5	217·31	249·73	285·44	320·18	360·74
Mauritania	21·16	23·58	26·56	29·89	33·23	36·91	41·53	47·19	51·03	54·44
Niger	119·55	133·03	150·1	169·5	193·25	226·06	262·78	302·79	352·66	434·12
Nigeria	1291·87	1402·34	1541·33	1636·54	1854·23	2091·1	2386·04	2724·7	3073·89	3260·87
Sao Tome and Principe	1·48	1·65	1·87	2·04	2·2	2·42	2·68	2·9	3·18	3·47
Senegal	91·78	103·98	113·35	127·42	145·07	161·02	174·94	210·41	243·96	275·45
Sierra Leone	57·04	61·13	65·8	70·47	76·66	73·27	76·33	95·7	107·86	120·42
Togo	46·27	50·7	55·9	65·78	74·81	80·04	88·87	99·66	108·88	121·11
Eastern Sub-Saharan Africa	2535·58	2874·92	3263·21	3635·38	4045·11	4411·62	4879·54	5489·92	6122·67	6849·94
Burundi	84·14	88·19	102·9	115·78	125·92	127·76	129·54	161·23	202·39	243·47
Comoros	4·59	5·13	6·09	6·74	7·24	7·76	8·81	9·77	9·98	9·39
Djibouti	4·22	5·73	8·71	10·26	13·84	15·21	16·28	17·11	17·73	17·75
Eritrea	42·09	47·67	53·46	59·64	64·5	57·32	62·41	77·67	82·82	79·62
Ethiopia	613·82	688·79	735·67	824·36	957·15	1089·26	1198·18	1326·71	1449·3	1677·49
Kenya	275·9	329·03	382·94	436·07	483·47	529·44	575·45	626·33	666·04	685·74
Madagascar	161·16	183·8	208·28	233·47	259·69	294·59	329·69	360·81	390·17	437·49

Appendix Table 15C. Live births (in thousands) by location, females, 1970, 1975, 1980, 1985, 1990, 1995, 2000, 2005, 2010, 2016

Country	1970	1975	1980	1985	1990	1995	2000	2005	2010	2016
Malawi	130·54	143·11	158·49	175·92	228·93	223·1	245·15	269·17	309·77	354·61
Mozambique	211·71	238·64	274·29	282·66	267·62	328·55	378·4	439·03	502·61	544·04
Rwanda	94·53	112·85	135·54	154·76	162·89	135·17	162·34	187·82	194·51	197·75
Somalia	63·64	71·59	111·29	107·76	108·72	103·51	111·87	121·64	132·25	148·06
South Sudan	107·41	116·6	130·2	149·49	155·29	144·27	173·52	209·24	261·08	333·9
Tanzania	369·96	421·44	475·09	529·9	587·96	667·9	708·75	793·23	893·82	974·09
Uganda	259·27	293·37	334·25	379·59	436	490·21	559·31	645·35	734·67	831·81
Zambia	112·61	128·97	145·99	168·99	185·87	197·56	219·85	244·82	275·54	314·71
Central Sub-Saharan Africa	721·15	792·02	885·57	986·92	1108·11	1289	1467·95	1709·9	1985·92	2383·86
Angola	148·48	168·39	195·47	230·79	260·15	303·32	346·88	404·12	470·75	546·22
Central African Republic	35·44	37·96	42·17	48·64	52·55	59·53	65·48	69·5	77·18	87·96
Congo	28·32	31·93	35·72	40·44	45·93	52·15	59·36	65·02	75·02	79·54
Democratic Republic of the Congo	490·64	537·79	595·95	646·45	726·1	848·51	968·69	1140·82	1329·36	1632·76
Equatorial Guinea	6·12	3·86	3·68	6·9	8·03	8·31	8·11	8·59	9·64	11·17
Gabon	12·15	12·11	12·59	13·7	15·35	17·17	19·42	21·86	23·97	26·2

Appendix Table 16A. Under-5 populations (in thousands) by location, both sexes combined, 1970, 1975, 1980, 1985, 1990, 1995, 2000, 2005, 2010, 2016

Country	1970	1980	1985	1990	1995	2000	2005	2010	2016
Global	491538·8	530813·19	568300·87	598467·69	596764·32	591232·65	600221·69	626918·78	631970·98
High-income	67097·91	60377·57	60031·34	59980·65	59691·22	58351·88	58109·83	59849·66	57711·26
High-income North America	19030·77	18238·32	19907	21339·6	21913·98	21469·7	21894·1	22565·63	21613·47
Canada	1773·54	1771·24	1845·44	1891·07	1932·27	1715·76	1667·55	1833·59	1939·72
Greenland	8·13	4·64	4·9	5·24	5·22	4·51	3·98	3·64	3·41
United States	17236·43	16453·5	18046·88	19432·89	19965·96	19740·05	20214·02	20720·4	19663·32
Australasia	1462·05	1358·14	1420·59	1502·16	1568·57	1517·8	1521·45	1710·22	1776·22
Australia	1162·82	1100·77	1172·02	1223·9	1281·12	1236·2	1241·84	1400·08	1490·53
New Zealand	299·23	257·37	248·57	278·26	287·45	281·6	279·61	310·13	285·7
High-income Asia Pacific	13695·42	12718·39	11441·69	10008·75	9719·13	9159·49	8160·36	7918·51	7352·61
Brunei	15·54	25·07	29·58	33·1	36·34	39·13	37·6	33·77	32·97
Japan	8961·35	8464·35	7548·5	6541·91	5992·38	5895·18	5614·41	5461·16	5015·79
Singapore	231·99	190·52	204·42	242·86	242·03	221·89	186·52	179·83	176·87
South Korea	4486·54	4038·45	3659·19	3190·87	3448·38	3003·29	2321·83	2243·75	2126·98
Western Europe	29477·46	23840·13	22830·98	22598·7	21917·67	21479·48	21639·56	22669·25	21945·46
Andorra	0·67	1·23	1·74	2·24	2·84	3·03	2·95	3·13	3·05
Austria	595·6	427·93	443·84	431·57	459·03	409·55	389·22	386·82	401·95
Belgium	699·28	589·4	580·83	589·93	597·43	575·79	579·29	624·9	631·97
Cyprus	70·59	56·83	60·93	59·34	56·37	44·87	40·56	45·56	49·63
Denmark	374·33	303·82	261·6	291·6	334·15	332·63	323·18	320·05	288·4
Finland	346·02	319·38	321·45	311·7	324·55	291·99	284·02	296·64	290·46
France	4132·14	3678·29	3821·81	3799·69	3626·45	3700·05	3832·31	3964·22	3919·03
Germany	5854·45	4010·51	4102·71	4258·7	3948·23	3898·39	3550·48	3357·52	3429·58
Greece	727·91	706·13	633·78	526·16	512·2	513·64	524·79	576·91	472·55
Iceland	21·01	20·93	20·58	21·68	22·37	20·86	20·75	23·4	21·46
Ireland	305·34	345·05	330·62	279·12	248·44	263·66	298·95	363·04	336·37
Israel	334·59	476·41	509·86	506·11	533·36	616·96	696·08	775·04	837·75
Italy	4610·64	3549·96	3014·97	2811·83	2720·13	2651·55	2714·06	2796·98	2540·99
Luxembourg	22·72	19·61	20·73	22·42	26·08	27·43	26·84	27·62	30·21
Malta	24·49	28·71	28·42	29·2	26·71	23·35	18·53	18·9	19·08
Netherlands	1172·19	863·4	863·32	928·96	963·27	978·66	988·69	924·99	869·84
Norway	319·55	253·26	249·95	280·44	297·99	295·07	282·26	298·29	297·16
Portugal	922·35	807·62	699·69	596·94	554·98	562·66	555·03	511·41	423·7
Spain	3242·83	3066·09	2413·54	2054·52	1883·76	1867·18	2157·12	2456·44	2165·62
Sweden	560·14	477·26	470·56	553·52	573	453·55	489·84	548·2	571·94
Switzerland	507·68	361·14	367·58	390·95	413·6	395·52	363·38	380·69	416·73
United Kingdom	4608·8	3456·84	3592·6	3829·48	3769·78	3530·56	3477·88	3945·37	3904·71
England	3746·79	2820·61	2954·57	3197·12	3160·83	2966·96	2945·89	3352·7	3330·53
East Midlands	292·82	232·36	238·37	252·47	255·88	238·6	236·13	267·76	267·92
East of England	344·58	280·52	298·6	330·49	324·45	306·65	311·89	352·59	359·16
Greater London	600·69	448·04	470·05	537·91	536·62	534·1	547·23	649·44	652·49
North East England	232·01	161·33	165·4	166·25	162·74	144·24	137·05	147·37	144·39
North West England	621·41	432·38	449·46	467·84	450·36	405·03	392·34	443	423·62
South East England	504·79	411	438·91	490·12	482·14	453·86	450·06	517·7	513·98
South West England	290·44	236·19	250·21	273·99	277·92	260·22	254·96	292·97	295·19
West Midlands	458·56	324·67	336·35	355·03	346·65	324·92	320·9	359·5	352·49
Yorkshire and the Humber	401·49	294·13	307·23	323·02	324·07	299·34	295·34	322·36	321·29
Northern Ireland	148·48	140·88	138·65	135·62	122·89	115·74	109·15	123·33	119·77
Scotland	461·03	320·89	323·77	320·37	312·49	282·83	264·22	291·38	285·55
Wales	252·5	174·45	175·61	176·38	173·58	165·03	158·62	177·96	168·87
Southern Latin America	3432·22	4222·59	4431·08	4531·44	4571·87	4725·4	4894·36	4986·05	5023·48
Argentina	2033·33	2917·77	3000·79	2948·02	2921·4	3155·64	3432·23	3555·33	3581·64
Chile	1144·57	1059·57	1201·93	1345·24	1385·17	1296·59	1210·64	1197·56	1204·1
Uruguay	254·1	245·11	228·23	238·05	265·15	272·99	251·31	233	237·59
Central Europe, Eastern Europe, and Central Asia	29850·85	33231·77	35224·53	34984·64	27490·33	22651·94	22904·93	26064·48	28193·12
Eastern Europe	14209·09	15634·22	17013·55	16575·31	11580·3	9354·58	9969·29	11660·52	12958·71
Belarus	727·24	735·67	804·37	773·01	594·84	472·64	453·53	515·5	575·9
Estonia	97·56	106·95	114·96	118·45	80·64	63·29	66·85	76·67	68·54
Latvia	161·67	168·99	191·41	197·94	137·91	96·42	101·87	107·74	105·8
Lithuania	266·29	252·15	269·91	285·15	235·47	181·49	150·85	155·81	150·3
Moldova	358·15	401·58	432·55	422·53	322·5	232·86	212·58	226·17	224·9
Russia	9153·67	10383·69	11437·76	11235·09	7434·3	6224·66	6983·24	8194·62	9377·3
Ukraine	3444·51	3585·2	3762·6	3543·13	2774·64	2083·21	2000·36	2384·01	2455·97
Central Europe	9154·64	10282·03	9731·84	9025·9	7349·37	6128·02	5675·42	5926·37	5587·27
Albania	250·31	317·37	362·2	403·71	375·46	290·38	212·52	179·26	185·3
Bosnia and Herzegovina	421·19	353·27	374·07	373·09	298·37	207·21	164·54	173·84	170·89
Bulgaria	644·79	671·52	604·71	564·54	416·77	340·36	337·64	364·29	332·63
Croatia	317·86	322·36	323·32	293·15	247·94	226·58	198·05	206·64	196·75
Czech Republic	673·55	858·75	691·48	652·7	563·7	441·49	469·53	566·02	544·77
Hungary	717·02	818·55	651·82	616·64	582·16	487·72	475·27	478·75	452·63
Macedonia	202·22	194·42	189·44	171·3	165·22	143·38	121·08	115·14	112·23
Montenegro	57·64	51·89	53·15	50·65	46·24	42·25	38·71	38·53	36·42
Poland	2480·44	3244·99	3410·52	2887·74	2308·1	1905·76	1789·33	1993·01	1869·37
Romania	2163·03	2061·34	1746·84	1774·27	1242·23	1112·6	1034·82	982·2	878·38
Serbia	703	761·49	741·56	706·3	653·13	558·23	488·16	438·73	413·71
Slovakia	383·1	476·87	449·16	412·4	349·96	281·93	257·32	285·04	288·31
Slovenia	140·48	149·2	133·58	119·42	100·07	90·13	88·44	104·92	105·88
Central Asia	6487·12	7315·52	8479·14	9383·43	8560·66	7169·34	7260·22	8477·59	9647·13
Armenia	286·74	360·31	418·38	406·8	297·43	197·57	182·47	198·05	221·37
Azerbaijan	676·29	708·57	808·34	877·79	818·56	639·1	652·94	875·83	956·73
Georgia	412·05	446·41	460·42	458·72	354·54	257·59	265·83	312·01	333·2
Kazakhstan	1890·48	1882·58	1963·9	1955·65	1582·22	1221·81	1292·08	1689·37	1959·14
Kyrgyzstan	432·31	469·83	548·75	612	568·56	497·07	492·57	604·2	747·67
Mongolia	227·01	263·43	303·48	326·72	280·67	238·07	233·87	291·14	372·32
Tajikistan	468·02	572·64	723·5	880·39	853·04	763·68	763·8	889·76	1138·5
Turkmenistan	295·52	368·81	455·26	552·27	570·57	493·24	474·81	516·52	577·54
Uzbekistan	1798·69	2242·95	2797·1	3313·09	3235·07	2861·21	2901·86	3100·7	3340·68
Latin America and Caribbean	38654·8	46995·2	50310·16	52237·03	53118·07	53079·99	51753·22	50777·94	49604·99
Central Latin America	16419·37	20386·85	22199·91	23788·47	24929·45	25181·31	24743·79	24032·87	22843·84
Colombia	3014·33	3633·58	3981·19	4275·31	4362·89	4198·27	4085·03	3991·32	3573·32
Costa Rica	287·45	314·36	367·02	397·85	380·54	374·96	357·28	343·01	304·2
El Salvador	606·29	746·75	712·2	723·09	745·18	732·22	676	614·09	533·92
Guatemala	1001·97	1237·89	1367·34	1553·92	1746·7	1907·89	1986·13	1975·65	1971·72
Honduras	530·79	676·49	760·74	838·28	859·45	853·15	851·59	891·05	947·12
Mexico	8594·86	10851·18	11813·67	12570·12	13235·01	13354·25	12868·02	12254·88	11671·42
Nicaragua	309·24	413·53	486·02	557·54	635·1	693·64	699·52	673·14	609·14

Appendix Table 16A. Under-5 populations (in thousands) by location, both sexes combined, 1970, 1975, 1980, 1985, 1990, 1995, 2000, 2005, 2010, 2016									
**Country**	**1970**	**1980**	**1985**	**1990**	**1995**	**2000**	**2005**	**2010**	**2016**
Panama	247·05	267·89	273·81	286·22	311·18	330·79	325·01	345·2	346·73
Venezuela	1827·4	2245·18	2437·92	2586·15	2653·39	2736·14	2895·21	2944·52	2886·27
Andean Latin America	3500·79	4652·59	5155·56	5504·1	5675·68	5751·74	5720·04	6019·73	6662·74
Bolivia	738·88	960·92	1048·51	1094·47	1152·43	1228·44	1263·6	1294·51	1368·6
Ecuador	913·38	1264·2	1434·58	1524·25	1600·96	1626·68	1630·86	1700·48	1788·5
Peru	1848·53	2427·47	2672·47	2885·38	2922·29	2896·62	2825·58	3024·74	3505·64
Caribbean	4023·73	3896·28	4010·67	4173·3	4113·93	4135·84	4097·55	4033·45	3989·54
Antigua and Barbuda	9·57	7·55	6·41	5·59	5·38	6·1	6·54	6·36	6·1
The Bahamas	22·8	25·73	27·37	28·03	30·55	28·86	27·84	30·71	33
Barbados	26·38	23·02	22·8	20·31	17·64	15·7	15·76	15·74	14·79
Belize	24·26	28·17	33·14	36·82	33·17	34·29	39·91	43·23	47·51
Bermuda	4·78	3·51	3·59	3·91	4·1	4·21	4·33	4·36	4·39
Cuba	1272·22	767·93	788·63	894	770·93	736·96	669·14	603·98	599·52
Dominica	13·93	9·41	9·27	8·58	8·15	6·73	5·9	5·55	5·32
Dominican Republic	662·91	903·84	903·62	897·35	1000·23	1033·96	1025·59	994·68	941·19
Grenada	14·38	13·17	14·54	14·61	12·11	11·05	9·81	9·18	8·7
Guyana	99·88	113·66	108·92	105·13	105·45	99·02	84·78	71·5	65·34
Haiti	766·12	949·71	1063·62	1151·83	1179·68	1263·79	1376·93	1462·02	1519·37
Jamaica	349·61	319·04	323·75	337·07	336·01	322·49	299·72	278·41	276·67
Puerto Rico	357·4	380·63	324·94	312·92	288·79	265·01	235·85	216·96	209·58
Saint Lucia	22·77	22·17	22	20·58	17·8	15·11	13·06	11·18	9·63
Saint Vincent and the Grenadines	17·64	17·81	17·24	15·55	13·57	11·73	10·13	9·44	9·14
Suriname	62·19	51·68	55·23	56·31	57·1	57·9	56·32	52·3	46·46
Trinidad and Tobago	142·43	145·02	170·05	141·78	106·03	98·21	91·42	89·42	68·89
Tropical Latin America	14710·91	18059·49	18944·02	18771·15	18399·01	18011·11	17191·84	16691·89	16108·86
Brazil	14331·06	17604·36	18415·91	18138·97	17697·89	17293·93	16500·2	16029·23	15478·11
Paraguay	379·85	455·12	528·12	632·18	701·12	717·18	691·64	662·65	630·75
Southeast Asia, East Asia, and Oceania	171342·99	156218·95	162168·45	167848·76	157941·58	143236·79	132522·18	130287·6	123071·67
East Asia	127214·7	106094·87	108751·58	112425·92	100295·78	84080·71	72988·16	69958·16	64630·98
China	122576·94	101761·9	104343·41	107858·53	95335·76	79007·49	68493·85	65997	60657·92
North Korea	2770·64	2394·88	2576·97	2979·28	3354·28	3583·67	3333·53	2996·82	2958·99
Taiwan (Province of China)	1867·12	1938·09	1831·2	1588·11	1605·74	1489·55	1160·78	964·33	1014·07
Southeast Asia	43430·89	49241·45	52469·21	54392·86	56520·92	57928·24	58250·74	59008·36	57027·77
Cambodia	1058·95	1132·44	1143·27	1230·61	1353·71	1445·97	1603·97	1815·14	1883·8
Indonesia	17898·65	19507·39	20386·39	20545·77	21749·41	23099·4	23685·23	24088·26	22726·43
Laos	446·04	536·99	566·9	650·38	748·61	811·07	849·19	952·15	1130·32
Malaysia	1665·89	1893·48	2208·12	2444·54	2628·7	2649·28	2452·68	2522·84	2567·66
Maldives	10·56	12·88	15·97	19·61	22·36	23·85	26·22	32·32	31·34
Mauritius	108·27	115·73	96·74	101·51	110·59	100·94	91·31	73·99	66·16
Myanmar	3036·35	4042·6	4499	4859·16	4958·25	5043·84	5141·46	5088·57	4657·56
Philippines	5660·87	7209·84	8074·72	8823·13	9607·89	10242·1	10750·55	11100·98	11567·29
Sri Lanka	1676·7	1910·69	1982·22	1782·04	1769·95	1661·2	1792·47	1823·16	1511·47
Seychelles	8·93	8·68	8·95	8·19	7·97	7·2	7·62	8·56	8·35
Thailand	6237·14	6781·36	6222·79	5826·76	5768·03	5455·87	4634·89	3847·37	3093·82
Timor-Leste	80·06	94·58	101·52	110·17	118·09	102·36	100·23	151·81	165·53
Vietnam	5467·91	5937·33	7100·4	7923·47	7608·06	7214·38	7040·4	7432·26	7553·61
Oceania	697·4	882·63	947·65	1029·97	1124·88	1227·84	1283·28	1321·08	1412·92
Federated States of Micronesia	13·25	15·18	17·37	17·46	16·54	14·8	11·99	10·05	9·57
Fiji	82·26	99·52	100·69	90·81	78·94	70·31	63·38	57·61	50·83
Kiribati	9·56	12·44	13·18	14·01	14·3	13·85	13·79	13·88	13·52
Marshall Islands	3·97	5·55	6·27	7·26	8·25	9·19	9·23	9·7	9·91
Papua New Guinea	428·85	562·72	604·5	676·44	771·96	878·19	939·86	988·05	1077·6
Samoa	26·7	27·81	28·33	28·04	27·47	27·68	27·82	28·1	27·48
Solomon Islands	27·75	39·77	46·5	51·6	58·9	69·04	76·23	81·76	83·33
Tonga	14·38	14·87	15·31	16·11	16·06	15·04	15·11	15·1	13·46
Vanuatu	16·96	23·22	26·45	28·97	31·41	33·25	34·97	37·86	40·57
North Africa and Middle East	31616·38	41743·01	46535·69	49982·44	50886·04	52455·04	55678·17	60996·4	63190·72
North Africa and Middle East	31616·38	41743·01	46535·69	49982·44	50886·04	52455·04	55678·17	60996·4	63190·72
Afghanistan	1996·47	2464·34	2332·79	2192·9	2606·3	3153·36	3738·3	4228·55	4957·28
Algeria	2411·1	3095·15	3531·96	3901	4227·11	4757·93	5148·52	5085·43	4549·61
Bahrain	33·38	47·71	59·05	68·26	66·65	67·24	58·16	78·3	99·7
Egypt	5587·94	6855·91	7489·49	7917	7784·5	8000·77	8784·7	9971·82	10906·5
Iran	4854·43	7317·42	8604·69	9439·87	7939·44	6206·61	6776·42	8067·67	8064·69
Iraq	1873·71	2497·22	2809·87	3060·43	3369·97	3861·94	4320·38	5786·84	7705
Jordan	354·78	448·33	473·15	517·88	621·12	699·59	720·23	841·79	960·26
Kuwait	148	240·61	270·61	239·06	209·99	211·71	218·78	267·62	281·84
Lebanon	436·16	444·29	404·45	360·88	327·91	298·49	265·51	263·86	320·81
Libya	317·56	407·58	459·25	511·71	568·64	637·39	673·61	649·79	462·98
Morocco	2270·87	2779·71	3162·95	3450·78	3458·6	3191·68	2879·84	2606·96	2291·38
Palestine	270·74	291·01	318·29	380·21	458·77	538·68	581·64	777·05	1085·42
Oman	136·89	224·09	283·24	335·24	375·94	424·46	460·07	443·56	443·37
Qatar	16·18	29·97	41·87	51·53	54·78	51·62	61·62	87·53	123·81
Saudi Arabia	1150·53	1851·01	2441·65	2970·77	3139·06	3320·05	3370·63	3113·37	2492·07
Sudan	1528·17	1971·11	2150·32	2499·03	3093·86	3861·48	4282·88	4357·3	4193·42
Syria	1173·83	1626·94	1853·26	2067·84	2258·66	2436·22	2432·73	2509·98	1767
Tunisia	762·44	953·64	1060·52	1080·38	990·11	860·07	815·47	885·48	834·44
Turkey	5164·56	6586·52	6788·21	6474·5	6271·84	6374·48	6185·29	6233·64	6158·56
United Arab Emirates	44·54	142·01	231·58	274·35	307·9	356·73	435·67	691·38	815·47
Yemen	1070·93	1445·48	1739·26	2157·44	2721·14	3110	3422·72	3999·06	4626·73
South Asia	102321·28	127563·86	141286·82	151354·7	155801·9	158436·02	161162·96	162741·18	153610·26
South Asia	102321·28	127563·86	141286·82	151354·7	155801·9	158436·02	161162·96	162741·18	153610·26
Bangladesh	10208·71	11269·14	12905	14605·88	15155·4	14968·27	15251·58	15307·53	14329·41
Bhutan	49·63	67·19	74·72	85·75	88·76	88·63	94·24	91·17	78·07
India	79762·16	100042·56	108734·14	114509·49	118101·98	121291·83	123898·23	123008·28	112140·64
Nepal	1846·63	2381·42	2700·64	3062·58	3339·02	3548·55	3503·43	3469·43	3990·87
Pakistan	10454·16	13803·55	16872·32	19091	19116·74	18538·75	18415·48	20864·77	23071·27
Sub-Saharan Africa	50654·58	64682·83	72743·87	82079·48	91835·18	103020·99	118090·4	136201·53	156588·97
Southern Sub-Saharan Africa	3979·43	5109·09	5801·85	6550·71	6985·21	7195·91	7608·31	8073·04	8608·25
Botswana	112·62	150·18	172·26	189·11	200·74	213·19	225·7	248·05	264·73
Lesotho	168·15	195·44	209·6	218·47	226·11	236·63	239·8	247·57	258·22
Namibia	133·91	163·81	178·94	221·95	268·48	284·63	284·88	291·53	332·84
South Africa	2650·27	3386·18	3794·09	4181·85	4413·09	4528·93	4809·02	4932·45	5011·2
Swaziland	71·07	93·88	108·33	130·82	143·91	149·48	160·56	185·37	208·54
Zimbabwe	843·42	1119·6	1338·63	1608·52	1732·87	1783·04	1888·35	2168·06	2532·71
Western Sub-Saharan Africa	20869·65	25893·95	28953·16	32521·9	36816·47	41771·92	48393·68	56448·67	64619·76
Benin	502·96	643·92	745·64	850·32	984·32	1146·82	1357·29	1604·17	1910·74

Appendix Table 16A. Under-5 populations (in thousands) by location, both sexes combined, 1970, 1975, 1980, 1985, 1990, 1995, 2000, 2005, 2010, 2016

Country	1970	1980	1985	1990	1995	2000	2005	2010	2016
Burkina Faso	1027·18	1237·49	1399·25	1617·16	1829·13	2079·7	2458·34	2872·58	3194·76
Cameroon	1249·41	1520·02	1736·43	2005·02	2252·4	2493·83	2841·82	3312·03	3877·23
Cape Verde	37·96	41·89	50·35	57·54	60·63	64·52	69·35	74·73	75·08
Chad	656·78	836·03	916·21	1050·22	1221·99	1422·67	1711·53	2094·32	2641·51
Cote d'Ivoire	878·01	1331·58	1684·23	1989·16	2167·08	2430·84	2753·63	3052·14	3558·7
The Gambia	102·26	133·93	152·69	182·7	220·64	256·31	290·92	324·2	366·63
Ghana	1612·51	1873·44	2106·37	2433·85	2581·63	2679·42	3075·03	3739·15	4346·43
Guinea	659·58	738·69	802·34	922·41	1159·58	1393·68	1552·14	1745·14	2034·88
Guinea-Bissau	108·41	140·66	153·51	167·42	185·52	207·75	237·73	271·13	309·97
Liberia	250·33	330·68	385·88	403·34	379·78	463·08	573·05	632·5	712·82
Mali	1025·16	1268·7	1419·19	1550·62	1733·61	2023·54	2369·99	2734·63	3150·02
Mauritania	177·66	226·72	259·49	293·58	329·29	369·77	422·67	473·61	514·37
Niger	880·68	1107·54	1266·96	1452·52	1714·67	2071·4	2475·97	2953·91	3683·59
Nigeria	10224·36	12558·84	13718·6	15068·03	17251·5	19729·95	22774·68	26495·19	29533·86
Sao Tome and Principe	13·2	16·39	18·06	19·71	21·73	24·25	26·94	29·48	33·3
Senegal	698·53	928·18	1058·52	1228·4	1405·43	1534·81	1803·1	2172·96	2542
Sierra Leone	412·32	498·39	543·03	598·33	616·46	614·02	730·63	895·42	1039·95
Togo	351·6	460·23	535·77	631·02	700·58	765·09	868·56	971·18	1093·73
Eastern Sub-Saharan Africa	20039·21	26409·5	29756·2	33760·37	37420·56	41789·39	47698·06	54629·61	62566·28
Burundi	675·77	806·82	950·79	1063·63	1109·41	1130·13	1295·83	1689·48	2142·38
Comoros	37·54	49·31	57·78	64·09	69·48	77·27	88·62	94·99	92·78
Djibouti	32·81	66·35	87·29	111·81	135·69	147·25	158	167·03	172·42
Eritrea	334·71	444·59	504·05	565·16	563·27	562·19	669·15	789·18	795·34
Ethiopia	4819·82	5986·5	6431·22	7697·04	9045·09	10293·54	11673·98	13163·13	15284·55
Kenya	2282·94	3281·08	3814·25	4320·44	4720·43	5159·62	5696·16	6227·71	6573·53
Madagascar	1325·03	1719·32	1932·69	2174·85	2468·32	2841·78	3186·64	3497·25	3888·77
Malawi	933·73	1208·75	1361·33	1696·39	1912·81	2033·14	2300·42	2659·26	3201·34
Mozambique	1617·89	2131·26	2282·73	2274	2546·62	3088·25	3655·52	4350·63	4966·26
Rwanda	710·54	1035·62	1261·54	1427·25	1180·74	1291·22	1600·98	1812·75	1872·13
Somalia	508·29	790·04	958·91	937·41	926·99	952·94	1055·79	1170·48	1318·8
South Sudan	831·6	1016·83	1177·27	1312·37	1294·98	1406·21	1742·69	2171·08	2860·28
Tanzania	2930·2	3909·19	4422·74	4947·47	5639·81	6213·78	6921·41	7950·59	9002·88
Uganda	2071	2726·21	3102·71	3576·6	4095·82	4705·2	5510·46	6426·84	7517·63
Zambia	920·02	1226·41	1396·41	1573·72	1691·03	1861·85	2112·7	2424·23	2842·24
Central Sub-Saharan Africa	5766·29	7270·3	8232·66	9246·5	10612·94	12263·76	14390·35	17050·21	20794·68
Angola	1191·89	1564·85	1853·44	2133·29	2474	2898·1	3417·08	4060·52	4906·3
Central African Republic	288·58	346·49	396·99	441·14	489·84	545·36	590·47	643·28	742·05
Congo	234·89	307·88	353	405·14	459·48	518·11	583·92	668·56	745·16
Democratic Republic of the Congo	3900·66	4907·59	5461·16	6064·39	6964·68	8056·65	9525	11373·2	14057·2
Equatorial Guinea	47·92	30·95	46·15	65·77	70·67	71·86	75·28	84	99·13
Gabon	102·35	112·53	121·92	136·77	154·27	173·69	198·61	220·65	244·84

Appendix Table 16B. Under-5 population (in thousands) by location, males, 1970, 1975, 1980, 1985, 1990, 1995, 2000, 2005, 2010, 2016									
**Country**	**1970**	**1980**	**1985**	**1990**	**1995**	**2000**	**2005**	**2010**	**2016**
Global	251775·19	271788·42	291148·47	307337·84	307565·72	305149·38	310135·37	323926·41	326098·66
High-income	34358·52	30945·4	30777·24	30790·88	30646·58	29927·43	29796·56	30676·84	29580·98
High-income North America	9730·41	9336·03	10189·59	10918·34	11211·6	10981·12	11198·19	11542·32	11057·9
Canada	908·29	908·44	946·7	968·86	991·69	879·98	855·27	940·21	995·93
Greenland	4·13	2·37	2·51	2·64	2·64	2·29	2·02	1·87	1·76
United States	8811·56	8420·66	9235·38	9941·59	10211·92	10094·08	10336·54	10596·18	10056·59
Australasia	748·36	696·16	728·34	769·92	805·38	778·52	780·22	877·99	911·91
Australia	595·48	564·45	600·9	627·43	657·44	633·99	637·23	718·82	765·47
New Zealand	152·87	131·71	127·44	142·5	147·94	144·53	142·99	159·17	146·44
High-income Asia Pacific	7031·22	6537·32	5888·97	5199·12	5052·86	4734·63	4212·87	4074·17	3783·3
Brunei	7·93	12·84	15·16	16·98	18·64	20·07	19·29	17·32	16·91
Japan	4593·45	4342·58	3874·22	3358·39	3076·5	3026·92	2883·02	2804·46	2575·86
Singapore	118·71	98·63	106·13	125·69	124·93	115·22	96·36	92·86	91·28
South Korea	2311·13	2083·27	1893·46	1698·07	1832·78	1572·43	1214·21	1159·53	1099·25
Western Europe	15108·58	12232·56	11718·26	11599·3	11251·05	11028·21	11113·28	11642·93	11268·92
Andorra	0·35	0·63	0·9	1·16	1·46	1·58	1·5	1·61	1·58
Austria	304·67	219·19	227·48	221·31	235·47	210·14	199·73	198·51	206·29
Belgium	358·52	302·53	298·01	302·78	305·7	293·84	296·55	319·92	323·56
Cyprus	35·84	29·11	31·24	30·45	28·93	23·02	20·94	23·47	25·46
Denmark	192·2	155·91	133·9	149·86	171·43	170·73	165·64	164·26	148·1
Finland	176·64	163·39	164·36	159·26	165·41	149·18	145·38	151·5	148·54
France	2113·55	1882·8	1956·8	1945·82	1857·52	1895·84	1963·77	2031·57	2008·51
Germany	3000·53	2057·67	2106·17	2187·02	2028·19	2003·06	1824·47	1725·43	1762·59
Greece	375·23	364·49	327·85	271·33	264·18	264·45	270·71	297·79	243·41
Iceland	10·76	10·72	10·56	11·14	11·49	10·64	10·6	11·98	10·92
Ireland	157·45	178·08	170·72	144·15	128·33	136·21	154·46	187·6	173·6
Israel	171·88	244·01	261·59	259·24	273·81	316·95	357·05	397·42	429·61
Italy	2368·13	1825·56	1551·45	1447·47	1400·6	1365·63	1398·01	1440·79	1308·98
Luxembourg	11·58	10·02	10·58	11·48	13·33	14·06	13·74	14·17	15·44
Malta	12·56	14·74	14·6	15·01	13·73	12·01	9·53	9·72	9·81
Netherlands	601·45	443·29	443·33	477·12	494·82	502·81	508	475·27	446·29
Norway	164·3	130·03	128·33	143·75	153·28	151·57	144·62	153·13	152·44
Portugal	471·59	414·05	359·11	306·63	285·26	289·32	285·47	263·08	217·97
Spain	1665·67	1577·44	1242·51	1058·07	970·36	962·08	1111·6	1265·95	1116·16
Sweden	287·99	245·13	241·85	284·32	293·89	232·92	252·08	282·02	294·24
Switzerland	259·64	184·9	188·26	200·25	211·89	202·67	186·21	195·11	213·58
United Kingdom	2355·72	1768·37	1838·53	1960·13	1930·18	1807·95	1781·18	2020·74	2000·1
England	1914·22	1442·91	1512·39	1635·79	1619·17	1519·09	1508·26	1717·25	1706·36
East Midlands	149·58	118·84	122·01	129·33	131·46	122·36	121·38	137·08	135·84
East of England	176·13	143·58	152·9	169·28	166·32	157·32	160·12	180·83	180·48
Greater London	305·88	228·43	239·89	274·56	274·18	273·23	278·66	332·13	334·94
North East England	118·43	82·44	84·6	85·02	83·47	73·82	70·35	75·14	74·14
North West England	318·7	222·01	230·06	239·31	231·25	207·04	200·92	227·41	216·65
South East England	259·19	211·31	225·33	250·6	246·92	232·35	230·97	265·56	266·34
South West England	148·49	120·91	128·2	140·6	142·54	133·31	130·63	149·87	151·86
West Midlands	233·65	165·63	172·3	182·07	177·16	166·77	164·21	184·07	182·87
Yorkshire and the Humber	204·17	149·76	157·1	165·02	165·86	152·88	151·03	165·15	163·23
Northern Ireland	76·31	71·89	71·02	69·7	62·83	59·31	56·01	63·26	62·56
Scotland	236·69	164·66	165·46	164·22	159·57	144·94	135·32	148·95	145·02
Wales	128·51	88·9	89·65	90·42	88·62	84·61	81·59	91·29	86·16
Southern Latin America	1739·95	2143·32	2252·07	2304·2	2325·69	2404·95	2492·01	2539·42	2558·95
Argentina	1031·06	1480·66	1524·36	1498·11	1484·96	1604·97	1746·74	1810·08	1823·83
Chile	579·33	537·69	611·19	684·45	705·19	660·35	616·68	610·07	613·48
Uruguay	129·45	124·91	116·45	121·56	135·46	139·54	128·51	119·18	121·56
Central Europe, Eastern Europe, and Central Asia	15244·34	16979·16	18001·51	17897·89	14091·39	11630·15	11770·98	13397·96	14492·45
Eastern Europe	7260·13	7983·43	8691·38	8484·92	5939·42	4804·62	5124·44	5989·64	6657·07
Belarus	372·05	376·09	411·32	396·81	306·11	243·36	233·17	265·35	296·31
Estonia	49·9	54·81	58·8	60·76	41·4	32·61	34·33	39·49	35·19
Latvia	82·84	86·62	98·04	101·42	70·51	49·66	52·14	54·81	54·28
Lithuania	135·85	128·68	138·18	146·35	120·67	93·39	77·42	79·67	77·03
Moldova	184·2	206·52	222·46	217·36	165·76	119·48	109·54	116·23	115·71
Russia	4672·73	5299·74	5838·04	5746·56	3811·52	3195·44	3589·37	4207·74	4813·69
Ukraine	1762·56	1830·97	1924·54	1815·65	1423·47	1070·68	1028·46	1226·35	1264·86
Central Europe	4686·65	5268·8	4989·27	4628·91	3770·16	3145·76	2914·37	3043·43	2869·99
Albania	129·46	163·85	186·94	208·3	193·79	150·42	110·08	92·86	96
Bosnia and Herzegovina	216·12	181·53	192·33	191·96	153·69	106·71	84·77	89·59	88
Bulgaria	330·98	344·75	310·59	289·86	213·29	174·69	173·66	186·92	170·95
Croatia	162·79	165·3	165·88	150·52	127·39	116·43	101·8	106·23	101·16
Czech Republic	345·16	440·28	354·71	335·05	289·44	226·81	241·26	290·87	279·97
Hungary	367·4	419·85	334·59	316·69	299·14	250·71	244·39	246·23	232·8
Macedonia	103·49	99·52	96·96	87·69	84·61	73·47	62·07	59·03	57·54
Montenegro	29·87	26·9	27·56	26·26	23·98	21·9	20·07	19·98	18·79
Poland	1267·72	1660·96	1746·98	1479·86	1183·45	977·82	918·37	1023·1	959·82
Romania	1106·94	1056·63	895·66	909·4	636·87	570·56	530·88	504·2	451·1
Serbia	359·32	389·39	379·19	361·29	334·22	285·77	249·94	224·69	211·93
Slovakia	195·55	243·46	229·46	210·83	178·98	144·25	131·69	145·9	147·6
Slovenia	71·86	76·39	68·41	61·19	51·3	46·22	45·37	53·83	54·32
Central Asia	3297·56	3726·92	4320·85	4784·06	4381·81	3679·77	3732·18	4364·89	4965·39
Armenia	145·45	182·72	212·36	207·93	153·49	105·22	98	105·59	117·43
Azerbaijan	342·6	360·58	411·2	447·08	420·16	335·99	349·04	467·97	508·44
Georgia	210·77	229·23	236·79	236·11	182·71	134·56	138·92	163·25	174·17
Kazakhstan	970·27	966·78	1008·76	1002·56	810·44	626·37	661·62	866·98	1006·77
Kyrgyzstan	216·55	237·83	278·56	310·78	289·82	254·85	251·38	309·84	383·26
Mongolia	114·02	132·55	152·79	164·58	141·41	119·99	117·98	147·06	188·25
Tajikistan	235·76	289·69	366·64	446·45	432·75	387·64	388·36	453·16	580·31
Turkmenistan	149·37	186·87	230·83	280·09	289·35	250·13	240·98	262·46	293·9
Uzbekistan	912·79	1140·67	1422·92	1688·49	1661·68	1465·02	1485·89	1588·58	1712·86
Latin America and Caribbean	19700·73	23958·43	25658·36	26650·43	27110·67	27106·78	26440·1	25950·27	25361·68
Central Latin America	8362·3	10389·79	11323·17	12141·73	12730	12863·58	12644·75	12285·63	11682·08
Colombia	1536·94	1854·42	2033·58	2184·85	2229·67	2145·64	2088·08	2040·58	1827·64
Costa Rica	146·44	160·49	187·52	203·35	194·56	191·76	182·75	175·49	155·66
El Salvador	307·34	379·76	363·01	368·92	380·46	374·13	345·59	314·11	273·21
Guatemala	510·38	630·57	697	792·42	891·01	973·43	1013·76	1008·99	1007·44
Honduras	270·52	345·04	388·14	427·9	438·92	435·96	435·3	455·56	484·34
Mexico	4374·87	5526·62	6021·68	6412·74	6757·33	6821·59	6575·73	6264·88	5969·06
Nicaragua	156·81	210·51	247·64	284·23	323·78	353·95	357·22	343·93	311·43

Appendix Table 16B. Under-5 population (in thousands) by location, males, 1970, 1975, 1980, 1985, 1990, 1995, 2000, 2005, 2010, 2016

Country	1970	1980	1985	1990	1995	2000	2005	2010	2016
Panama	126·12	136·88	139·94	146·31	159·07	169·13	166·2	176·55	177·36
Venezuela	932·86	1145·51	1244·66	1321	1355·2	1398·01	1480·12	1505·54	1475·93
Andean Latin America	1782·51	2372·02	2628·17	2805·46	2894·41	2934·83	2919·73	3073·75	3403·99
Bolivia	375	489·14	534·15	557·76	587·36	626·29	644·47	660·53	698·87
Ecuador	466·77	645·1	731·51	776·65	816·39	829·63	831·91	867·95	913·48
Peru	940·74	1237·78	1362·52	1471·05	1490·67	1478·91	1443·35	1545·26	1791·65
Caribbean	2049·12	1984·6	2043·87	2128·91	2099·06	2111·16	2092·04	2059·37	2038·2
Antigua and Barbuda	4·84	3·82	3·24	2·83	2·72	3·08	3·31	3·21	3·09
The Bahamas	11·66	13·2	14·04	14·38	15·68	14·82	14·3	15·77	16·95
Barbados	13·36	11·68	11·57	10·32	8·96	7·98	8·01	8	7·52
Belize	12·08	14·11	16·63	18·54	16·74	17·34	20·21	21·9	24·08
Bermuda	2·4	1·76	1·8	1·96	2·06	2·09	2·18	2·2	2·21
Cuba	651·34	393·56	404·3	458·53	395·58	378·28	343·58	310·17	307·89
Dominica	7·11	4·82	4·74	4·39	4·17	3·44	3·02	2·84	2·72
Dominican Republic	336·99	459·98	460·21	457·41	510·32	527·99	523·96	508·26	481·03
Grenada	7·35	6·73	7·43	7·47	6·2	5·66	5·02	4·69	4·45
Guyana	50·68	57·74	55·38	53·46	53·64	50·41	43·18	36·43	33·31
Haiti	386·6	480·81	539·28	584·73	599·36	642·59	700·5	743·86	773·68
Jamaica	178·23	162·82	165·3	172·14	171·64	164·78	153·17	142·3	141·42
Puerto Rico	182·66	194·83	166·41	160·32	147·95	135·78	120·85	111·17	107·41
Saint Lucia	11·5	11·21	11·13	10·42	9·02	7·66	6·62	5·66	4·88
Saint Vincent and the Grenadines	8·93	9·01	8·73	7·87	6·87	5·94	5·13	4·78	4·63
Suriname	32·15	26·72	28·57	29·13	29·54	29·95	29·14	27·05	23·97
Trinidad and Tobago	72·26	73·68	86·46	72·09	53·91	49·93	46·48	45·47	35·03
Tropical Latin America	7506·81	9212·02	9663·14	9574·33	9387·21	9197·21	8783·58	8531·52	8237·41
Brazil	7312·83	8979·71	9393·62	9251·68	9029·34	8830·99	8430·28	8192·85	7914·85
Paraguay	193·98	232·32	269·52	322·64	357·86	366·21	353·31	338·67	322·56
Southeast Asia, East Asia, and Oceania	87920·7	80121·9	83238·06	86696·89	82188·37	74804·1	69441·43	68458·28	64523·1
East Asia	65526·64	54627	56052·51	58477·75	52795·95	44607·91	39022·99	37542·98	34540·72
China	63149·98	52403·78	53790·03	56130·03	50243·8	42004·18	36714·21	35508·02	32498·51
North Korea	1414·78	1223·64	1317	1522·8	1714·08	1827·68	1701·87	1531·59	1512·43
Taiwan (Province of China)	961·88	999·59	945·48	824·92	838·07	776·05	606·91	503·37	529·77
Southeast Asia	22035·96	25040·76	26697·58	27688·38	28812·39	29563·02	29756·43	30233·46	29253·1
Cambodia	531·68	570·4	578·16	619·52	682·53	729·87	811·02	930·2	960·91
Indonesia	9062·63	9912·93	10361·74	10444·58	11060·76	11754·45	12062·33	12278·86	11594·53
Laos	221·49	268·71	284·69	327·49	377·69	409·96	430·08	483·21	574·73
Malaysia	852·55	971·04	1133·52	1255·73	1350·98	1361·96	1261·21	1297·35	1320·53
Maldives	5·35	6·57	8·16	10·03	11·57	12·25	13·45	16·68	16·39
Mauritius	54·95	58·81	49·19	51·64	56·28	51·39	46·51	37·7	33·72
Myanmar	1524·1	2035·06	2266·04	2448·82	2499·53	2543·65	2594·23	2569·87	2355·19
Philippines	2890·09	3685·59	4129·14	4515·65	4921·92	5251·05	5515·06	5698·5	5940·9
Sri Lanka	849·49	970·34	1009·82	908·32	900·31	847·34	914·93	930·39	770·36
Seychelles	4·58	4·46	4·59	4·2	4·1	3·7	3·92	4·4	4·29
Thailand	3183·35	3459·91	3176·18	2975·38	2954·7	2803·93	2384·4	1981·42	1592·48
Timor-Leste	40·19	47·65	51·26	55·85	60·03	52·17	51·1	77·46	84·52
Vietnam	2778·33	3020·31	3613·67	4037·09	3896·99	3705·54	3630·43	3891·44	3971·86
Oceania	358·1	454·14	487·97	530·76	580·02	633·18	662·01	681·85	729·28
Federated States of Micronesia	6·8	7·8	8·93	8·99	8·52	7·63	6·18	5·18	4·93
Fiji	42·16	51·06	51·69	46·63	40·55	36·11	32·55	29·58	26·1
Kiribati	4·85	6·34	6·78	7·21	7·36	7·13	7·1	7·15	6·95
Marshall Islands	2·01	2·82	3·19	3·7	4·2	4·68	4·7	4·95	5·06
Papua New Guinea	220·51	289·92	311·7	348·97	398·35	453·39	485·33	510·33	556·96
Samoa	13·8	14·39	14·67	14·53	14·23	14·35	14·43	14·57	14·25
Solomon Islands	14·22	20·43	23·92	26·57	30·34	35·57	39·28	42·13	42·93
Tonga	7·33	7·59	7·82	8·23	8·2	7·68	7·72	7·71	6·88
Vanuatu	8·7	11·93	13·61	14·92	16·18	17·13	18·01	19·5	20·89
North Africa and Middle East	16166·21	21339·28	23789·03	25549·6	26009·75	26804·12	28478·45	31224·28	32372·05
North Africa and Middle East	16166·21	21339·28	23789·03	25549·6	26009·75	26804·12	28478·45	31224·28	32372·05
Afghanistan	1023·97	1265·86	1201·56	1127·57	1337·86	1618·63	1917·96	2169·39	2543·76
Algeria	1223·49	1573·95	1798·14	1989·39	2158·26	2431·12	2631·88	2600·43	2327·19
Bahrain	17·09	24·41	30·22	34·89	33·81	34·36	29·53	39·87	50·79
Egypt	2899·25	3541·66	3859·31	4073·58	4002·75	4111	4524·51	5136·95	5615·49
Iran	2487·36	3742·78	4402·08	4826·79	4056·94	3159·03	3455·74	4127·72	4130·68
Iraq	953·24	1276·52	1441·2	1571·91	1729·33	1983·1	2220·9	2976·3	3964·09
Jordan	182·22	229·84	242·5	265·41	318·27	358·45	369·03	431·37	492·22
Kuwait	75·11	122·21	137·34	121·5	107·33	108·08	111·47	136·47	143·73
Lebanon	223·62	228·72	207·85	185·42	168·12	153·02	136·11	135·25	164·44
Libya	162·68	208·62	235·08	262·13	291·89	327·68	346·46	333·91	238·19
Morocco	1152·78	1415·39	1611·72	1762·41	1770·43	1636·81	1478·21	1337·21	1176·4
Palestine	138·75	149·05	163·3	194·59	234·74	275·57	297·49	397·37	555·16
Oman	70·18	114·65	144·76	171·3	192·21	217·16	235·51	227·1	226·98
Qatar	8·26	15·32	21·41	26·41	28·1	26·26	31·51	44·64	63·22
Saudi Arabia	582·08	936·38	1235·35	1503·67	1589·61	1681·92	1708·28	1578·45	1263·85
Sudan	768·79	994·37	1084·22	1262·79	1564·32	1953·43	2168·54	2207·17	2124·47
Syria	598·26	829·86	946·03	1055·79	1153·66	1245·13	1244·19	1283·78	907·79
Tunisia	391·5	487·85	542·02	551·93	505·94	439·68	417·03	452·99	427·01
Turkey	2634·22	3360·5	3463·39	3303·4	3201·07	3254·92	3160·5	3186·65	3149·73
United Arab Emirates	22·62	72·26	117·97	139·81	157·09	182·14	222·28	353·11	416·41
Yemen	544·15	737·6	888·91	1103·13	1390·99	1589·21	1748·65	2043·18	2365·01
South Asia	52826·71	65768·17	72922·57	78255·06	81079·03	82783·9	84473·89	85280·93	80439·84
South Asia	52826·71	65768·17	72922·57	78255·06	81079·03	82783·9	84473·89	85280·93	80439·84
Bangladesh	5199·71	5756·59	6594·51	7462·07	7740·27	7642·46	7787·01	7818·23	7323·43
Bhutan	25·44	34·38	38·2	43·78	45·29	45·18	48·02	46·44	39·76
India	41234·84	51613·8	56183·26	59318·81	61700·74	63623·22	65205·6	64780·85	59014·32
Nepal	945·51	1218·65	1381·32	1565·9	1713·98	1833·31	1808·37	1779·04	2056·23
Pakistan	5421·21	7144·75	8725·27	9864·49	9878·75	9639·74	9624·89	10856·38	12006·09
Sub-Saharan Africa	25557·98	32676·07	36761·71	41497·09	46439·93	52092·89	59733·97	68937·86	79328·56
Southern Sub-Saharan Africa	1997·93	2567·79	2917·23	3296·47	3518·3	3626·17	3834·62	4068·56	4342·47
Botswana	56·57	75·56	86·73	95·29	101·25	107·56	113·95	125·45	134·04
Lesotho	84·86	98·5	105·49	109·73	113·45	118·67	120·2	124·22	129·93
Namibia	67·37	82·46	90·11	111·87	135·44	143·64	143·73	147·14	168·18
South Africa	1330·38	1703·32	1909·35	2106·17	2225·52	2285·93	2428·05	2490·2	2532·47
Swaziland	35·48	47·13	54·46	65·89	72·56	75·32	80·86	93·44	105·28
Zimbabwe	423·28	560·83	671·09	807·53	870·08	895·04	947·83	1088·12	1272·58
Western Sub-Saharan Africa	10555·14	13126·84	14691·05	16510·34	18693·78	21213·29	24584·16	28691·27	32861·21
Benin	252·4	324·92	377·26	431·07	499·48	582·2	689·1	814·43	970·19

Appendix Table 16B. Under-5 population (in thousands) by location, males, 1970, 1975, 1980, 1985, 1990, 1995, 2000, 2005, 2010, 2016

Country	1970	1980	1985	1990	1995	2000	2005	2010	2016
Burkina Faso	518·22	625·9	708·7	820·25	928·49	1056·1	1249·23	1460·47	1625·07
Cameroon	628·52	765·12	874·24	1010·1	1134·91	1256·54	1432·28	1669·9	1955·95
Cape Verde	19·13	21·14	25·42	29·05	30·61	32·59	35·06	37·8	38·01
Chad	329·68	420·37	460·84	528·74	615·36	716·33	861·86	1055·16	1331·67
Cote d'Ivoire	439·89	669·17	846·82	999·83	1088·58	1220·48	1382·41	1532·57	1788·23
The Gambia	51·53	67·55	77·05	92·25	111·47	129·52	147·05	163·92	185·45
Ghana	817·58	952·51	1072·1	1240·05	1315·9	1365·89	1567·78	1906·81	2218·04
Guinea	329·84	369·87	402·03	462·28	581·35	699·13	779·03	876·4	1022·78
Guinea-Bissau	54·25	70·56	77·04	83·93	93·06	104·29	119·49	136·43	156·21
Liberia	125·62	167	195·41	204·86	193·39	235·98	292·45	322·93	364·15
Mali	522·35	645·94	721·62	787·08	879·12	1026	1201·71	1386·86	1598·58
Mauritania	90·15	115·18	131·87	149·24	167·44	188·07	215·05	241·09	262·04
Niger	449·58	565·36	646·66	741	874·18	1055·81	1262·05	1505·81	1877·85
Nigeria	5187·5	6391·32	6991·82	7686·26	8801·31	10067·24	11625·11	13532·81	15094·38
Sao Tome and Principe	6·69	8·3	9·15	9·97	10·98	12·24	13·59	14·88	16·82
Senegal	352·31	468·58	534·6	620·55	710	775·21	910·99	1098·6	1286·5
Sierra Leone	204·57	247·99	270·42	298·22	307·6	306·77	365·18	448·05	521·02
Togo	174·96	229·74	267·68	315·34	350·27	382·66	434·61	486·25	548·2
Eastern Sub-Saharan Africa	10091·02	13306·44	14991·73	17016·88	18866·81	21061·76	24053·48	27573·71	31627·4
Burundi	340·41	406·47	479·21	536·07	559·1	568·84	652·5	851·55	1080·42
Comoros	19·03	25·03	29·34	32·56	35·33	39·34	45·16	48·44	47·34
Djibouti	16·61	33·62	44·22	56·65	68·74	74·59	80·07	84·68	87·49
Eritrea	168·52	224·84	255·28	286·7	286·23	286·38	340·86	402·27	405·83
Ethiopia	2428·36	3018·61	3240·97	3886·98	4566·93	5198·84	5899·66	6659·99	7749·2
Kenya	1155·32	1659·22	1927·95	2182·92	2383·29	2603·73	2875·35	3145·99	3323·32
Madagascar	662·52	859·96	966·54	1087·92	1235·5	1423·55	1600·26	1761	1962·73
Malawi	466·83	605·53	682·37	850·35	958·97	1019·52	1154·25	1335·84	1616·06
Mozambique	813·5	1072·92	1148·83	1145·71	1282·86	1556·04	1842·98	2194·49	2506·35
Rwanda	353·27	515·16	628·57	711·27	595·83	643·2	798·7	906·13	940·62
Somalia	255·84	397·91	483·08	472·48	467·19	480·22	532·3	590·35	665·6
South Sudan	423·95	517·89	599·41	668·03	658·45	713·74	883·88	1100·83	1450·33
Tanzania	1482·49	1977·72	2237·09	2501·82	2850·71	3139·57	3496·66	4016·84	4550·12
Uganda	1037·27	1367·41	1557·51	1795·44	2056·45	2364·84	2772·73	3236·65	3790·83
Zambia	463·41	618·49	704·02	792·79	851·08	936·66	1063·08	1220·94	1433·43
Central Sub-Saharan Africa	2913·88	3675	4161·69	4673·39	5361·04	6191·67	7261·7	8604·32	10497·48
Angola	607·41	796·7	943·16	1084·78	1256·91	1468·72	1727·01	2050·2	2477·52
Central African Republic	145·53	174·76	200·15	222·16	246·36	273·94	296·57	323·17	372·96
Congo	119·74	156·56	179·3	205·66	233·22	262·91	295·26	337·72	376·33
Democratic Republic of the Congo	1965·35	2474·49	2754·22	3058·56	3511·07	4062·24	4804·7	5739·54	7097·06
Equatorial Guinea	24·1	15·61	23·27	33·15	35·59	36·2	37·95	42·36	50·02
Gabon	51·76	56·88	61·6	69·08	77·88	87·65	100·21	111·33	123·59

Appendix Table 16C. Under-5 population (in thousands) by location, females, 1970, 1975, 1980, 1985, 1990, 1995, 2000, 2005, 2010, 2016									
Country	1970	1980	1985	1990	1995	2000	2005	2010	2016
Global	239763·62	259024·76	277152·4	291129·85	289198·6	286083·26	290086·32	302992·36	305872·32
High-income	32739·4	29432·17	29254·1	29189·77	29044·65	28424·44	28313·27	29172·82	28130·28
High-income North America	9300·35	8902·29	9717·41	10421·26	10702·38	10488·58	10695·92	11023·31	10555·57
Canada	865·25	862·8	898·74	922·21	940·59	835·79	812·28	893·38	943·79
Greenland	3·99	2·26	2·38	2·59	2·57	2·22	1·96	1·77	1·65
United States	8424·87	8032·85	8811·5	9491·3	9754·04	9645·97	9877·48	10124·22	9606·73
Australasia	713·69	661·98	692·24	732·24	763·19	739·28	741·24	832·22	864·31
Australia	567·34	536·32	571·12	596·48	623·68	602·21	604·61	681·26	725·06
New Zealand	146·35	125·66	121·13	135·76	139·51	137·07	136·62	150·96	139·26
High-income Asia Pacific	6664·2	6181·07	5552·72	4809·63	4666·27	4424·86	3947·5	3844·34	3569·32
Brunei	7·61	12·23	14·42	16·13	17·7	19·06	18·31	16·45	16·06
Japan	4367·9	4121·77	3674·28	3183·53	2915·87	2868·26	2731·39	2656·7	2439·94
Singapore	113·28	91·89	98·29	117·17	117·1	106·67	90·17	86·97	85·59
South Korea	2175·41	1955·18	1765·73	1492·8	1615·6	1430·87	1107·63	1084·22	1027·73
Western Europe	14368·88	11607·57	11112·72	10999·39	10666·62	10451·27	10526·28	11026·32	10676·54
Andorra	0·33	0·6	0·84	1·08	1·38	1·46	1·45	1·52	1·48
Austria	290·92	208·74	216·35	210·26	223·56	199·4	189·49	188·31	195·66
Belgium	340·76	286·87	282·82	287·15	291·73	281·95	282·73	304·98	308·41
Cyprus	34·74	27·73	29·69	28·89	27·43	21·85	19·62	22·09	24·17
Denmark	182·13	147·91	127·7	141·75	162·73	161·9	157·54	155·79	140·3
Finland	169·38	155·99	157·09	152·44	159·15	142·81	138·64	145·14	141·92
France	2018·58	1795·48	1865·01	1853·87	1768·94	1804·21	1868·54	1932·66	1910·52
Germany	2853·92	1952·84	1996·55	2071·68	1920·04	1895·32	1726·01	1632·09	1666·99
Greece	352·69	341·64	305·93	254·83	248·02	249·19	254·09	279·11	229·14
Iceland	10·25	10·21	10·03	10·55	10·89	10·22	10·16	11·42	10·53
Ireland	147·89	166·96	159·9	134·97	120·11	127·45	144·49	175·43	162·77
Israel	162·71	232·41	248·27	246·87	259·55	300·01	339·03	377·62	408·14
Italy	2242·52	1724·4	1463·52	1364·36	1319·53	1285·92	1316·04	1356·19	1232·01
Luxembourg	11·14	9·59	10·15	10·94	12·74	13·37	13·1	13·46	14·77
Malta	11·92	13·96	13·82	14·19	12·98	11·35	9	9·18	9·26
Netherlands	570·74	420·11	419·99	451·84	468·45	475·84	480·69	449·72	423·55
Norway	155·25	123·23	121·62	136·69	144·71	143·5	137·65	145·16	144·72
Portugal	450·76	393·57	340·58	290·31	269·72	273·34	269·56	248·34	205·74
Spain	1577·16	1488·66	1171·03	996·45	913·4	905·09	1045·52	1190·49	1049·46
Sweden	272·15	232·13	228·71	269·2	279·1	220·63	237·76	266·17	277·7
Switzerland	248·04	176·24	179·33	190·7	201·71	192·86	177·17	185·59	203·15
United Kingdom	2253·08	1688·47	1754·07	1869·35	1839·6	1722·61	1696·7	1924·62	1904·61
England	1832·58	1377·7	1442·18	1561·32	1541·66	1447·88	1437·62	1635·45	1624·17
East Midlands	143·24	113·51	116·36	123·14	124·41	116·24	114·75	130·68	132·08
East of England	168·45	136·95	145·7	161·21	158·13	149·33	151·76	171·76	178·69
Greater London	294·82	219·61	230·16	263·34	262·44	260·88	268·58	317·31	317·55
North East England	113·58	78·89	80·8	81·23	79·27	70·42	66·7	72·23	70·25
North West England	302·71	210·37	219·4	228·53	219·11	197·98	191·42	215·59	206·97
South East England	245·6	199·7	213·58	239·52	235·22	221·51	219·09	252·14	247·64
South West England	141·95	115·28	122·02	133·39	135·37	126·91	124·32	143·1	143·32
West Midlands	224·91	159·03	164·04	172·97	169·49	158·14	156·69	175·43	169·61
Yorkshire and the Humber	197·32	144·36	150·13	158	158·21	146·46	144·31	157·21	158·06
Northern Ireland	72·17	68·99	67·64	65·93	60·06	56·42	53·14	60·07	57·21
Scotland	224·34	156·23	158·3	156·15	152·92	137·89	128·9	142·43	140·53
Wales	123·99	85·55	85·95	85·95	84·95	80·41	77·03	86·67	82·71
Southern Latin America	1692·27	2079·26	2179·01	2227·25	2246·19	2320·46	2402·34	2446·63	2464·53
Argentina	1002·26	1437·11	1476·43	1449·9	1436·44	1550·68	1685·49	1745·25	1757·81
Chile	565·24	521·88	590·74	660·79	679·98	636·23	593·96	587·49	590·62
Uruguay	124·65	120·2	111·78	116·49	129·69	133·45	122·81	113·81	116·03
Central Europe, Eastern Europe, and Central Asia	14606·52	16252·62	17223·02	17086·75	13398·94	11021·79	11133·95	12666·52	13700·67
Eastern Europe	6948·97	7650·79	8322·17	8090·39	5640·88	4549·95	4844·86	5670·89	6301·64
Belarus	355·18	359·58	393·04	376·2	288·73	229·29	220·36	250·15	279·59
Estonia	47·67	52·15	56·16	57·69	39·24	30·68	32·52	37·18	33·34
Latvia	78·83	82·37	93·37	96·53	67·4	46·76	49·73	52·93	51·52
Lithuania	130·44	123·47	131·73	138·8	114·81	88·1	73·44	76·14	73·27
Moldova	173·95	195·06	210·1	205·17	156·75	113·38	103·04	109·94	109·19
Russia	4480·94	5083·95	5599·71	5488·53	3622·78	3029·22	3393·87	3986·88	4563·61
Ukraine	1681·95	1754·22	1838·05	1727·47	1351·17	1012·53	971·9	1157·66	1191·11
Central Europe	4467·99	5013·23	4742·56	4396·99	3579·21	2982·26	2761·05	2882·94	2717·29
Albania	120·85	153·52	175·26	195·41	181·67	139·96	102·44	86·4	89·3
Bosnia and Herzegovina	205·07	171·74	181·75	181·13	144·68	100·5	79·76	84·25	82·89
Bulgaria	313·81	326·77	294·12	274·68	203·49	165·68	163·98	177·37	161·68
Croatia	155·07	157·06	157·43	142·62	120·55	110·15	96·25	100·4	95·59
Czech Republic	328·39	418·47	336·77	317·65	274·26	214·68	228·27	275·15	264·8
Hungary	349·62	398·71	317·23	299·95	283·02	237·01	230·88	232·52	219·82
Macedonia	98·73	94·9	92·47	83·6	80·61	69·91	59·01	56·11	54·69
Montenegro	27·77	24·99	25·59	24·39	22·26	20·35	18·64	18·55	17·63
Poland	1212·72	1584·03	1663·53	1407·88	1124·65	927·94	870·95	969·91	909·56
Romania	1056·1	1004·72	851·18	864·88	605·36	542·04	503·94	478	427·28
Serbia	343·69	372·1	362·37	345·01	318·92	272·46	238·22	214·04	201·79
Slovakia	187·55	233·41	219·7	201·57	170·97	137·68	125·63	139·14	140·71
Slovenia	68·62	72·81	65·17	58·23	48·76	43·91	43·07	51·09	51·55
Central Asia	3189·56	3588·6	4158·29	4599·37	4178·85	3489·58	3528·04	4112·69	4681·74
Armenia	141·29	177·59	206·02	198·87	143·95	92·34	84·47	92·46	103·94
Azerbaijan	333·7	347·99	397·15	430·71	398·4	303·11	303·9	407·86	448·29
Georgia	201·29	217·18	223·63	222·62	171·83	123·03	126·91	148·76	159·03
Kazakhstan	920·22	915·79	955·14	953·09	771·78	595·44	630·46	822·39	952·37
Kyrgyzstan	215·76	232	270·19	301·21	278·74	242·21	241·19	294·36	364·41
Mongolia	112·99	130·88	150·69	162·14	139·26	118·09	115·88	144·08	184·06
Tajikistan	232·26	282·95	356·86	433·95	420·29	376·05	375·43	436·6	558·19
Turkmenistan	146·15	181·94	224·44	272·18	281·21	243·12	233·84	254·06	283·64
Uzbekistan	885·9	1102·28	1374·18	1624·6	1573·39	1396·19	1415·97	1512·13	1627·82
Latin America and Caribbean	18954·07	23036·77	24651·8	25586·6	26007·4	25973·21	25313·12	24827·67	24243·32
Central Latin America	8057·08	9997·05	10876·74	11646·75	12199·45	12317·73	12099·05	11747·23	11161·77
Colombia	1477·39	1779·16	1947·61	2090·45	2133·22	2052·63	1996·95	1950·74	1745·68
Costa Rica	141	153·87	179·5	194·5	185·98	183·2	174·53	167·53	148·54
El Salvador	298·95	366·99	349·18	354·17	364·72	358·09	330·41	299·98	260·72
Guatemala	491·59	607·32	670·34	761·5	855·69	934·46	972·37	966·66	964·28
Honduras	260·26	331·46	372·61	410·38	420·53	417·19	416·3	435·49	462·77
Mexico	4219·99	5324·56	5791·99	6157·38	6477·68	6532·66	6292·29	5989·99	5702·36
Nicaragua	152·43	203·01	238·37	273·3	311·32	339·69	342·3	329·21	297·71

Appendix Table 16C. Under-5 population (in thousands) by location, females, 1970, 1975, 1980, 1985, 1990, 1995, 2000, 2005, 2010, 2016									
**Country**	**1970**	**1980**	**1985**	**1990**	**1995**	**2000**	**2005**	**2010**	**2016**
Panama	120·93	131·01	133·87	139·91	152·11	161·66	158·81	168·65	169·36
Venezuela	894·54	1099·67	1193·26	1265·15	1298·19	1338·13	1415·09	1438·99	1410·34
Andean Latin America	1718·28	2280·57	2527·39	2698·64	2781·27	2816·9	2800·31	2945·98	3258·75
Bolivia	363·88	471·79	514·36	536·71	565·07	602·15	619·13	633·98	669·73
Ecuador	446·61	619·1	703·07	747·6	784·57	797·05	798·95	832·53	875·02
Peru	907·79	1189·69	1309·96	1414·33	1431·63	1417·71	1382·23	1479·47	1714
Caribbean	1974·61	1911·68	1966·8	2044·39	2014·87	2024·68	2005·51	1974·08	1951·34
Antigua and Barbuda	4·73	3·73	3·17	2·76	2·66	3·02	3·24	3·14	3·02
The Bahamas	11·13	12·53	13·33	13·65	14·87	14·05	13·54	14·94	16·05
Barbados	13·02	11·34	11·22	10	8·68	7·72	7·75	7·74	7·27
Belize	12·18	14·06	16·51	18·28	16·43	16·94	19·7	21·32	23·43
Bermuda	2·38	1·74	1·79	1·94	2·04	2·12	2·15	2·16	2·18
Cuba	620·88	374·37	384·33	435·47	375·35	358·68	325·56	293·81	291·63
Dominica	6·82	4·6	4·53	4·19	3·98	3·28	2·88	2·71	2·59
Dominican Republic	325·92	443·86	443·41	439·94	489·91	505·97	501·63	486·43	460·17
Grenada	7·04	6·44	7·1	7·13	5·91	5·4	4·79	4·48	4·25
Guyana	49·2	55·92	53·55	51·67	51·8	48·61	41·6	35·07	32·03
Haiti	379·52	468·9	524·34	567·1	580·32	621·2	676·42	718·16	745·69
Jamaica	171·38	156·22	158·45	164·93	164·37	157·71	146·55	136·12	135·25
Puerto Rico	174·74	185·81	158·53	152·59	140·83	129·23	115	105·78	102·16
Saint Lucia	11·27	10·96	10·87	10·16	8·78	7·45	6·44	5·51	4·75
Saint Vincent and the Grenadines	8·72	8·8	8·51	7·68	6·7	5·79	5	4·66	4·52
Suriname	30·04	24·95	26·66	27·18	27·56	27·95	27·18	25·24	22·48
Trinidad and Tobago	70·17	71·34	83·59	69·68	52·12	48·28	44·94	43·95	33·86
Tropical Latin America	7204·1	8847·46	9280·88	9196·83	9011·8	8813·9	8408·26	8160·37	7871·46
Brazil	7018·23	8624·65	9022·28	8887·29	8668·55	8462·93	8069·92	7836·39	7563·26
Paraguay	185·87	222·81	258·59	309·54	343·26	350·97	338·34	323·98	308·2
Southeast Asia, East Asia, and Oceania	83422·28	76097·04	78930·39	81151·87	75753·21	68432·68	63080·75	61829·31	58548·57
East Asia	61688·06	51467·86	52699·07	53948·17	47499·83	39472·8	33965·17	32415·18	30090·26
China	59426·96	49358·12	50553·38	51728·5	45091·96	37003·3	31779·64	30488·98	28159·41
North Korea	1355·86	1171·24	1259·97	1456·48	1640·2	1756	1631·67	1465·24	1446·56
Taiwan (Province of China)	905·24	938·5	885·72	763·19	767·67	713·5	553·87	460·96	484·29
Southeast Asia	21394·93	24200·69	25771·64	26704·48	27708·53	28365·22	28494·31	28774·91	27774·67
Cambodia	527·27	562·03	565·11	611·09	671·18	716·11	792·95	884·94	922·9
Indonesia	8836·02	9594·46	10024·65	10101·19	10688·65	11344·95	11622·9	11809·4	11131·9
Laos	224·55	268·28	282·22	322·89	370·92	401·11	419·12	468·95	555·59
Malaysia	813·34	922·44	1074·6	1188·81	1277·71	1287·31	1191·47	1225·49	1247·13
Maldives	5·21	6·31	7·82	9·58	10·79	11·6	12·76	15·65	14·95
Mauritius	53·32	56·92	47·54	49·87	54·3	49·55	44·8	36·29	32·44
Myanmar	1512·24	2007·54	2232·96	2410·34	2458·72	2500·19	2547·23	2518·69	2302·37
Philippines	2770·78	3524·26	3945·58	4307·49	4685·97	4991·05	5235·48	5402·48	5626·39
Sri Lanka	827·21	940·35	972·4	873·72	869·64	813·85	877·54	892·77	741·11
Seychelles	4·35	4·22	4·35	3·98	3·88	3·5	3·7	4·16	4·06
Thailand	3053·8	3321·45	3046·61	2851·37	2813·32	2651·95	2250·49	1865·95	1501·34
Timor-Leste	39·87	46·93	50·27	54·32	58·06	50·19	49·13	74·35	81·01
Vietnam	2689·57	2917·01	3486·73	3886·38	3711·06	3508·85	3409·97	3540·82	3581·75
Oceania	339·3	428·49	459·68	499·22	544·86	594·67	621·27	639·23	683·64
Federated States of Micronesia	6·45	7·37	8·43	8·47	8·02	7·18	5·81	4·87	4·64
Fiji	40·1	48·47	49	44·17	38·4	34·2	30·83	28·03	24·74
Kiribati	4·7	6·1	6·4	6·8	6·94	6·72	6·69	6·73	6·57
Marshall Islands	1·95	2·72	3·08	3·56	4·04	4·5	4·52	4·75	4·85
Papua New Guinea	208·34	272·8	292·8	327·47	373·61	424·81	454·53	477·72	520·65
Samoa	12·9	13·42	13·66	13·52	13·23	13·33	13·4	13·53	13·23
Solomon Islands	13·54	19·33	22·57	25·03	28·56	33·47	36·95	39·62	40·39
Tonga	7·05	7·28	7·49	7·88	7·85	7·36	7·39	7·38	6·58
Vanuatu	8·26	11·28	12·84	14·05	15·23	16·12	16·96	18·36	19·68
North Africa and Middle East	15450·17	20403·72	22746·65	24432·84	24876·29	25650·92	27199·72	29772·12	30818·67
North Africa and Middle East	15450·17	20403·72	22746·65	24432·84	24876·29	25650·92	27199·72	29772·12	30818·67
Afghanistan	972·5	1198·49	1131·23	1065·33	1268·44	1534·72	1820·34	2059·16	2413·52
Algeria	1187·6	1521·2	1733·82	1911·61	2068·85	2326·81	2516·64	2485	2222·42
Bahrain	16·29	23·29	28·83	33·37	32·84	32·88	28·62	38·43	48·91
Egypt	2688·69	3314·24	3630·18	3843·42	3781·76	3889·78	4260·19	4834·87	5291·01
Iran	2367·07	3574·64	4202·6	4613·08	3882·5	3047·58	3320·68	3939·95	3934·01
Iraq	920·47	1220·7	1368·67	1488·52	1640·63	1878·84	2099·48	2810·55	3740·91
Jordan	172·56	218·5	230·65	252·47	302·85	341·13	351·2	410·42	468·04
Kuwait	72·88	118·4	133·27	117·56	102·66	103·63	107·31	131·14	138·11
Lebanon	212·54	215·57	196·6	175·46	159·79	145·47	129·4	128·6	156·37
Libya	154·88	198·96	224·17	249·58	276·75	309·71	327·14	315·88	224·79
Morocco	1118·09	1364·32	1551·23	1688·36	1688·16	1554·87	1401·64	1269·74	1114·98
Palestine	131·99	141·96	154·99	185·61	224·03	263·11	284·15	379·67	530·26
Oman	66·71	109·43	138·48	163·94	183·72	207·3	224·57	216·46	216·39
Qatar	7·92	14·66	20·46	25·12	26·68	25·35	30·12	42·89	60·58
Saudi Arabia	568·45	914·63	1206·3	1467·11	1549·45	1638·13	1662·35	1534·92	1228·22
Sudan	759·38	976·74	1066·1	1236·24	1529·54	1908·04	2114·34	2150·12	2068·95
Syria	575·56	797·08	907·24	1012·05	1105	1191·1	1188·55	1226·2	859·21
Tunisia	370·95	465·8	518·5	528·46	484·16	420·39	398·44	432·49	407·43
Turkey	2530·34	3226·02	3324·82	3171·1	3070·76	3119·56	3024·79	3046·99	3008·83
United Arab Emirates	21·93	69·75	113·61	134·54	150·81	174·59	213·39	338·26	399·06
Yemen	526·78	707·88	850·34	1054·31	1330·15	1520·79	1674·07	1955·88	2261·72
South Asia	49494·57	61795·68	68364·26	73099·64	74722·87	75652·12	76689·07	77460·25	73170·42
South Asia	49494·57	61795·68	68364·26	73099·64	74722·87	75652·12	76689·07	77460·25	73170·42
Bangladesh	5009	5512·56	6310·49	7143·81	7415·13	7325·81	7464·57	7489·3	7005·98
Bhutan	24·19	32·8	36·52	41·96	43·48	43·45	46·22	44·73	38·3
India	38527·31	48428·76	52550·88	55190·68	56401·24	57668·61	58692·63	58227·43	53126·33
Nepal	901·12	1162·77	1319·32	1496·68	1625·03	1715·24	1695·06	1690·39	1934·64
Pakistan	5032·95	6658·8	8147·05	9226·5	9237·99	8899·01	8790·59	10008·39	11065·17
Sub-Saharan Africa	25096·6	32006·76	35982·17	40582·39	45395·25	50928·1	58356·43	67263·67	77260·4
Southern Sub-Saharan Africa	1981·5	2541·29	2884·62	3254·24	3466·9	3569·74	3773·69	4004·48	4265·77
Botswana	56·05	74·61	85·53	93·82	99·49	105·64	111·75	122·6	130·69
Lesotho	83·29	96·94	104·1	108·74	112·65	117·96	119·6	123·35	128·3
Namibia	66·55	81·35	88·83	110·08	133·04	140·99	141·15	144·39	164·66
South Africa	1319·89	1682·86	1884·74	2075·68	2187·58	2243	2380·97	2442·25	2478·73
Swaziland	35·59	46·75	53·87	64·93	71·35	74·15	79·7	91·94	103·26
Zimbabwe	420·14	558·77	667·54	800·99	862·79	887·99	940·52	1079·95	1260·14
Western Sub-Saharan Africa	10314·51	12767·11	14262·11	16011·56	18122·69	20558·64	23809·52	27757·41	31758·55
Benin	250·55	319	368·39	419·26	484·84	564·62	668·19	789·74	940·56

Appendix Table 16C. Under-5 population (in thousands) by location, females, 1970, 1975, 1980, 1985, 1990, 1995, 2000, 2005, 2010, 2016

Country	1970	1980	1985	1990	1995	2000	2005	2010	2016
Burkina Faso	508·96	611·59	690·55	796·92	900·64	1023·61	1209·11	1412·11	1569·69
Cameroon	620·89	754·9	862·19	994·92	1117·48	1237·29	1409·55	1642·12	1921·28
Cape Verde	18·83	20·76	24·93	28·5	30·01	31·93	34·3	36·92	37·07
Chad	327·11	415·66	455·37	521·48	606·62	706·33	849·68	1039·16	1309·85
Cote d'Ivoire	438·12	662·41	837·41	989·33	1078·5	1210·36	1371·22	1519·57	1770·47
The Gambia	50·74	66·37	75·64	90·44	109·18	126·79	143·87	160·28	181·18
Ghana	794·94	920·93	1034·27	1193·8	1265·73	1313·53	1507·25	1832·35	2128·39
Guinea	329·74	368·82	400·3	460·13	578·23	694·55	773·11	868·74	1012·1
Guinea-Bissau	54·16	70·11	76·48	83·49	92·46	103·46	118·24	134·7	153·76
Liberia	124·71	163·67	190·47	198·48	186·39	227·09	280·6	309·57	348·67
Mali	502·8	622·76	697·56	763·55	854·49	997·54	1168·28	1347·78	1551·45
Mauritania	87·51	111·54	127·62	144·33	161·84	181·7	207·62	232·51	252·34
Niger	431·1	542·18	620·3	711·52	840·5	1015·59	1213·92	1448·1	1805·75
Nigeria	5036·86	6167·52	6726·78	7381·77	8450·18	9662·71	11149·57	12962·38	14439·48
Sao Tome and Principe	6·51	8·09	8·91	9·74	10·75	12·01	13·34	14·6	16·48
Senegal	346·22	459·61	523·92	607·86	695·43	759·6	892·11	1074·36	1255·5
Sierra Leone	207·75	250·4	272·61	300·11	308·85	307·25	365·45	447·37	518·93
Togo	176·64	230·49	268·09	315·67	350·31	382·43	433·95	484·94	545·53
Eastern Sub-Saharan Africa	9948·19	13103·06	14764·47	16743·49	18553·75	20727·63	23644·58	27055·89	30938·88
Burundi	335·36	400·35	471·58	527·56	550·31	561·29	643·34	837·93	1061·95
Comoros	18·52	24·28	28·44	31·53	34·15	37·93	43·46	46·55	45·44
Djibouti	16·2	32·73	43·07	55·16	66·95	72·65	77·94	82·35	84·93
Eritrea	166·19	219·75	248·77	278·46	277·04	275·8	328·29	386·92	389·51
Ethiopia	2391·45	2967·89	3190·25	3810·06	4478·16	5094·7	5774·32	6503·14	7535·35
Kenya	1127·63	1621·86	1886·3	2137·51	2337·14	2555·89	2820·81	3081·72	3250·21
Madagascar	662·51	859·37	966·15	1086·93	1232·82	1418·23	1586·38	1736·26	1926·04
Malawi	466·9	603·23	678·96	846·04	953·84	1013·62	1146·17	1323·42	1585·28
Mozambique	804·39	1058·34	1133·91	1128·28	1263·76	1532·21	1812·55	2156·14	2459·91
Rwanda	357·27	520·45	632·97	715·98	584·92	648·02	802·28	906·63	931·51
Somalia	252·45	392·13	475·83	464·93	459·81	472·72	523·49	580·12	653·19
South Sudan	407·65	498·94	577·86	644·34	636·54	692·46	858·81	1070·24	1409·95
Tanzania	1447·71	1931·47	2185·65	2445·65	2789·1	3074·21	3424·75	3933·76	4452·76
Uganda	1033·73	1358·8	1545·2	1781·16	2039·37	2340·36	2737·72	3190·19	3726·81
Zambia	456·61	607·92	692·39	780·93	839·95	925·19	1049·62	1203·29	1408·8
Central Sub-Saharan Africa	2852·41	3595·3	4070·97	4573·11	5251·9	6072·09	7128·65	8445·9	10297·2
Angola	584·48	768·14	910·28	1048·5	1217·1	1429·37	1690·06	2010·32	2428·78
Central African Republic	143·05	171·73	196·84	218·99	243·47	271·42	293·9	320·11	369·08
Congo	115·15	151·33	173·7	199·48	226·25	255·21	288·66	330·85	368·83
Democratic Republic of the Congo	1935·31	2433·1	2706·95	3005·84	3453·61	3994·41	4720·29	5633·66	6960·14
Equatorial Guinea	23·83	15·34	22·88	32·62	35·08	35·66	37·33	41·64	49·11
Gabon	50·59	55·66	60·32	67·69	76·39	86·03	98·4	109·32	121·26

Appendix Table 17A. Stillbirth rates (per 1,000 live births) by location, 1970-1979

	1970	1971	1972	1973	1974	1975	1976	1977	1978	1979
**Global**	41·52 (37·96 to 45·65)	40·65 (37·14 to 44·58)	39·91 (36·57 to 43·83)	39·06 (35·93 to 42·8)	38·24 (35·17 to 41·8)	37·45 (34·52 to 40·83)	36·62 (33·83 to 39·82)	35·82 (33·13 to 38·86)	34·94 (32·37 to 37·77)	34·07 (31·62 to 36·72)
**High SDI**	9·73 (8·51 to 11·28)	9·41 (8·26 to 10·88)	9·2 (8·12 to 10·63)	8·95 (7·9 to 10·27)	8·61 (7·66 to 9·8)	8·35 (7·48 to 9·43)	7·97 (7·18 to 8·92)	7·61 (6·87 to 8·48)	7·28 (6·61 to 8·09)	7·02 (6·42 to 7·75)
**High-middle SDI**	26·29 (23·26 to 29·91)	25·35 (22·41 to 28·76)	24·51 (21·73 to 27·86)	23·64 (21·07 to 26·79)	22·78 (20·37 to 25·73)	21·97 (19·65 to 24·74)	21·18 (18·97 to 23·79)	20·47 (18·35 to 22·91)	19·73 (17·69 to 21·97)	19·03 (17·14 to 21·2)
**Middle SDI**	40·34 (36·64 to 44·62)	39·17 (35·55 to 43·22)	38·09 (34·65 to 42·08)	36·98 (33·74 to 40·77)	35·94 (32·84 to 39·56)	34·88 (31·92 to 38·31)	33·86 (31·04 to 37·11)	32·83 (30·14 to 35·82)	31·74 (29·2 to 34·57)	30·68 (28·25 to 33·3)
**Low-middle SDI**	73·12 (67·69 to 79·27)	71·56 (66·2 to 77·46)	70·14 (64·99 to 75·92)	68·64 (63·7 to 74·35)	67·19 (62·42 to 72·59)	65·75 (61·17 to 70·94)	64·36 (59·94 to 69·36)	62·96 (58·6 to 67·74)	61·54 (57·35 to 66·07)	60·21 (56·18 to 64·44)
**Low SDI**	51·16 (48·77 to 53·85)	50·51 (48·16 to 53·1)	49·87 (47·6 to 52·43)	49·25 (47·01 to 51·78)	48·64 (46·47 to 51·12)	48·1 (45·99 to 50·55)	47·5 (45·49 to 49·87)	46·85 (44·92 to 49·11)	46·22 (44·32 to 48·39)	45·57 (43·71 to 47·62)
**High-income**	9·66 (8·19 to 11·62)	9·45 (8·02 to 11·34)	9·16 (7·81 to 10·99)	8·92 (7·59 to 10·63)	8·51 (7·31 to 10·04)	8·12 (7·03 to 9·49)	7·61 (6·61 to 8·82)	7·16 (6·28 to 8·26)	6·82 (6·01 to 7·84)	6·48 (5·74 to 7·42)
**High-income North America**	6·27 (5·69 to 6·93)	6·07 (5·49 to 6·73)	5·87 (5·32 to 6·51)	5·69 (5·19 to 6·28)	5·47 (4·98 to 6)	5·26 (4·79 to 5·77)	5·02 (4·58 to 5·51)	4·81 (4·38 to 5·21)	4·63 (4·26 to 5)	4·45 (4·13 to 4·78)
Canada	5·71 (5·61 to 5·82)	5·48 (5·38 to 5·58)	5·33 (5·24 to 5·43)	5·02 (4·93 to 5·11)	4·84 (4·75 to 4·93)	4·62 (4·54 to 4·71)	4·37 (4·3 to 4·45)	4·13 (4·06 to 4·2)	3·93 (3·87 to 4·01)	3·67 (3·61 to 3·74)
Greenland	15·7 (14·69 to 16·81)	14·7 (13·76 to 15·71)	13·9 (13 to 14·86)	13·67 (12·78 to 14·61)	13·51 (12·67 to 14·47)	13·55 (12·69 to 14·53)	13·91 (13·01 to 14·89)	13·57 (12·7 to 14·48)	13·27 (12·37 to 14·17)	13·56 (12·61 to 14·48)
United States	6·32 (5·69 to 7·04)	6·12 (5·5 to 6·85)	5·92 (5·33 to 6·62)	5·76 (5·22 to 6·4)	5·54 (4·99 to 6·12)	5·33 (4·81 to 5·88)	5·09 (4·6 to 5·63)	4·88 (4·41 to 5·32)	4·71 (4·29 to 5·11)	4·53 (4·18 to 4·89)
**Australasia**	8·27 (7·13 to 9·61)	8·11 (7·01 to 9·41)	7·82 (6·76 to 9·09)	7·6 (6·63 to 8·75)	7·5 (6·54 to 8·57)	7·02 (6·11 to 7·98)	6·6 (5·78 to 7·51)	6·16 (5·38 to 7·06)	5·81 (5·06 to 6·68)	5·42 (4·68 to 6·24)
Australia	8·21 (7 to 9·65)	8·05 (6·88 to 9·46)	7·74 (6·61 to 9·08)	7·52 (6·44 to 8·76)	7·45 (6·39 to 8·62)	6·92 (5·94 to 7·99)	6·51 (5·6 to 7·54)	6·05 (5·18 to 7·03)	5·72 (4·88 to 6·67)	5·32 (4·52 to 6·2)
New Zealand	8·5 (7·69 to 9·41)	8·36 (7·57 to 9·25)	8·16 (7·4 to 9·02)	7·95 (7·22 to 8·77)	7·68 (6·98 to 8·45)	7·41 (6·73 to 8·13)	6·98 (6·32 to 7·66)	6·62 (6·01 to 7·25)	6·22 (5·65 to 6·81)	5·86 (5·34 to 6·43)
**High-income Asia Pacific**	14·26 (11·07 to 18·58)	13·48 (10·5 to 17·67)	12·92 (10·11 to 16·84)	12·6 (9·8 to 16·23)	11·97 (9·4 to 15·41)	11·21 (8·8 to 14·19)	10·51 (8·33 to 13·16)	9·89 (7·86 to 12·5)	9·39 (7·51 to 11·82)	9 (7·23 to 11·21)
Brunei	24·1 (19·13 to 30·3)	22·67 (17·97 to 28·45)	21·04 (16·8 to 26·36)	19·53 (15·68 to 24·4)	17·66 (14·2 to 21·88)	16·96 (13·6 to 20·97)	15·52 (12·47 to 19·19)	14·6 (11·71 to 17·82)	13·5 (10·83 to 16·46)	12·56 (10·15 to 15·46)
Japan	13·64 (9·38 to 19·52)	12·91 (8·92 to 18·6)	12·38 (8·62 to 17·72)	12·27 (8·64 to 17·31)	11·63 (8·11 to 16·29)	10·83 (7·57 to 14·93)	10·13 (7·09 to 13·72)	9·58 (6·8 to 13·21)	9·12 (6·5 to 12·56)	8·75 (6·29 to 12·04)
Singapore	13·3 (12·71 to 13·95)	12·76 (12·19 to 13·38)	12·36 (11·81 to 12·99)	11·98 (11·45 to 12·58)	11·14 (10·63 to 11·69)	10·2 (9·71 to 10·7)	9·46 (9 to 9·91)	9·06 (8·62 to 9·48)	8·83 (8·41 to 9·23)	8·61 (8·21 to 9·02)
South Korea	15·55 (14·45 to 16·78)	14·68 (13·64 to 15·84)	14·06 (13·02 to 15·18)	13·33 (12·35 to 14·35)	12·73 (11·79 to 13·71)	12·05 (11·18 to 13)	11·36 (10·53 to 12·25)	10·61 (9·82 to 11·42)	9·97 (9·2 to 10·73)	9·45 (8·74 to 10·15)
**Western Europe**	8·88 (8·2 to 9·72)	8·74 (8·08 to 9·53)	8·35 (7·74 to 9·09)	8·07 (7·5 to 8·78)	7·71 (7·18 to 8·35)	7·5 (7·01 to 8·08)	6·99 (6·55 to 7·52)	6·6 (6·19 to 7·07)	6·32 (5·94 to 6·75)	6·01 (5·66 to 6·39)
Andorra	10·7 (10·61 to 10·79)	10·05 (9·97 to 10·13)	9·28 (9·21 to 9·36)	8·46 (8·39 to 8·53)	7·67 (7·61 to 7·74)	6·98 (6·92 to 7·04)	6·46 (6·41 to 6·52)	6·13 (6·08 to 6·18)	5·84 (5·79 to 5·89)	5·49 (5·45 to 5·54)
Austria	6·17 (5·67 to 6·74)	6·18 (5·68 to 6·75)	6·08 (5·6 to 6·62)	5·74 (5·29 to 6·25)	5·73 (5·27 to 6·23)	5·34 (4·92 to 5·81)	4·82 (4·44 to 5·25)	4·48 (4·13 to 4·87)	4·18 (3·85 to 4·54)	4 (3·69 to 4·34)
Belgium	13·02 (8·96 to 18·63)	12·66 (8·79 to 17·95)	12·15 (8·5 to 17·16)	11·57 (8·03 to 16·37)	11·12 (7·62 to 15·63)	10·59 (7·25 to 14·82)	10·11 (6·95 to 14·06)	9·5 (6·57 to 13·15)	9·04 (6·25 to 12·49)	8·58 (5·98 to 11·73)
Cyprus	12·7 (12·16 to 13·29)	12·03 (11·52 to 12·59)	11·63 (11·14 to 12·17)	11·15 (10·68 to 11·67)	10·95 (10·5 to 11·47)	10·3 (9·87 to 10·77)	10·14 (9·7 to 10·6)	9·18 (8·79 to 9·59)	8·74 (8·36 to 9·14)	8·25 (7·88 to 8·63)
Denmark	5·31 (4·38 to 6·43)	5·11 (4·24 to 6·15)	4·78 (3·99 to 5·71)	4·41 (3·71 to 5·25)	4·13 (3·48 to 4·88)	3·99 (3·39 to 4·66)	3·81 (3·26 to 4·42)	3·56 (3·07 to 4·09)	3·44 (2·99 to 3·92)	3·38 (2·98 to 3·8)
Finland	4·39 (4·23 to 4·57)	4·18 (4·02 to 4·35)	4 (3·85 to 4·17)	3·61 (3·48 to 3·77)	3·68 (3·54 to 3·83)	3·49 (3·36 to 3·63)	3·16 (3·04 to 3·29)	3·04 (2·93 to 3·17)	2·74 (2·64 to 2·86)	2·64 (2·55 to 2·75)
France	10·79 (10·54 to 11·06)	10·42 (10·18 to 10·69)	9·82 (9·59 to 10·07)	9·42 (9·2 to 9·66)	9·09 (8·88 to 9·32)	9·66 (9·44 to 9·91)	8·84 (8·65 to 9·06)	8·38 (8·19 to 8·59)	7·78 (7·6 to 7·97)	7·21 (7·05 to 7·39)
Germany	5·06 (5·01 to 5·11)	5·19 (5·15 to 5·24)	5 (4·96 to 5·05)	4·88 (4·84 to 4·93)	4·79 (4·75 to 4·84)	4·69 (4·65 to 4·74)	4·36 (4·32 to 4·4)	4·01 (3·97 to 4·05)	3·87 (3·84 to 3·91)	3·65 (3·62 to 3·68)
Greece	9·75 (9·54 to 9·97)	9·12 (8·93 to 9·32)	8·99 (8·8 to 9·2)	8·47 (8·29 to 8·66)	8·32 (8·15 to 8·51)	8·13 (7·96 to 8·32)	7·91 (7·75 to 8·09)	7·47 (7·32 to 7·64)	7·16 (7·01 to 7·32)	6·91 (6·77 to 7·07)
Iceland	4·26 (3·78 to 4·83)	4·18 (3·7 to 4·73)	4·05 (3·59 to 4·59)	3·92 (3·47 to 4·43)	3·8 (3·37 to 4·3)	3·7 (3·28 to 4·19)	3·48 (3·09 to 3·93)	3·35 (2·99 to 3·79)	3·24 (2·88 to 3·66)	3·02 (2·7 to 3·41)
Ireland	7·57 (7·11 to 8·07)	7·32 (6·88 to 7·79)	7·25 (6·82 to 7·73)	7·22 (6·81 to 7·72)	7·16 (6·75 to 7·66)	7·05 (6·65 to 7·52)	6·66 (6·28 to 7·09)	6·5 (6·12 to 6·91)	6·26 (5·91 to 6·66)	5·76 (5·44 to 6·12)
Israel	5·99 (4·95 to 7·24)	5·82 (4·84 to 7·03)	5·8 (4·81 to 6·97)	5·97 (4·95 to 7·16)	6·14 (5·1 to 7·36)	6·21 (5·15 to 7·43)	5·85 (4·84 to 6·97)	5·29 (4·39 to 6·35)	4·94 (4·09 to 5·95)	4·67 (3·89 to 5·61)
Italy	9·75 (8·67 to 10·98)	9·62 (8·59 to 10·81)	9·14 (8·13 to 10·23)	9 (8·01 to 10·1)	8·23 (7·34 to 9·19)	7·79 (6·94 to 8·71)	7·3 (6·52 to 8·11)	6·87 (6·16 to 7·64)	6·64 (5·93 to 7·37)	6·19 (5·53 to 6·87)
Luxembourg	4·75 (3·7 to 6·08)	4·67 (3·66 to 5·99)	4·27 (3·34 to 5·47)	4·2 (3·31 to 5·4)	3·98 (3·12 to 5·08)	3·76 (2·9 to 4·8)	3·69 (2·84 to 4·69)	3·26 (2·5 to 4·16)	3·16 (2·43 to 4·01)	3·16 (2·43 to 4)
Malta	8·26 (8·17 to 8·35)	7·83 (7·75 to 7·92)	7·32 (7·25 to 7·4)	7·37 (7·29 to 7·45)	6·89 (6·82 to 6·97)	6·55 (6·49 to 6·63)	6·31 (6·25 to 6·39)	5·84 (5·78 to 5·91)	5·7 (5·64 to 5·77)	5·54 (5·48 to 5·6)
Netherlands	9·75 (6·33 to 14·65)	9·42 (6·1 to 14·02)	9·12 (5·84 to 13·72)	8·83 (5·8 to 13·3)	8·58 (5·6 to 12·59)	8·16 (5·4 to 11·84)	7·85 (5·26 to 11·2)	7·3 (4·85 to 10·55)	7·16 (4·75 to 10·37)	6·71 (4·4 to 9·87)
Norway	5·9 (5·49 to 6·36)	5·73 (5·35 to 6·17)	5·42 (5·06 to 5·82)	5·21 (4·86 to 5·58)	4·89 (4·57 to 5·23)	4·74 (4·44 to 5·06)	4·5 (4·23 to 4·78)	4·07 (3·83 to 4·3)	3·85 (3·63 to 4·05)	3·65 (3·45 to 3·83)
Portugal	14·75 (14·38 to 15·15)	14·03 (13·68 to 14·41)	12·95 (12·62 to 13·29)	12·24 (11·94 to 12·58)	11·57 (11·3 to 11·88)	10·71 (10·45 to 11)	10·08 (9·83 to 10·36)	9·68 (9·46 to 9·93)	9·07 (8·86 to 9·32)	8·65 (8·45 to 8·87)
Spain	8·31 (7·49 to 9·25)	7·99 (7·22 to 8·9)	7·36 (6·64 to 8·17)	7·03 (6·34 to 7·83)	6·66 (6·03 to 7·41)	6·29 (5·68 to 6·99)	5·85 (5·27 to 6·47)	5·55 (4·99 to 6·14)	5·38 (4·86 to 5·96)	5·08 (4·59 to 5·61)
Sweden	3·64 (2·87 to 4·61)	3·58 (2·85 to 4·49)	3·5 (2·77 to 4·36)	3·26 (2·59 to 4·02)	3·18 (2·54 to 3·87)	2·99 (2·39 to 3·61)	2·85 (2·34 to 3·41)	2·78 (2·36 to 3·26)	2·68 (2·33 to 3·08)	2·66 (2·36 to 2·98)
Switzerland	4·67 (4·24 to 5·15)	4·54 (4·14 to 4·99)	4·36 (3·96 to 4·79)	4·2 (3·83 to 4·61)	3·98 (3·65 to 4·36)	3·64 (3·34 to 3·97)	3·4 (3·11 to 3·71)	3·18 (2·92 to 3·46)	3·01 (2·78 to 3·26)	2·9 (2·69 to 3·1)
**United Kingdom**	11·3 (11·04 to 11·58)	11·26 (11 to 11·53)	11·01 (10·76 to 11·28)	10·78 (10·54 to 11·05)	10·56 (10·32 to 10·83)	10·12 (9·9 to 10·39)	9·4 (9·19 to 9·64)	8·96 (8·76 to 9·18)	8·68 (8·48 to 8·9)	8·51 (8·32 to 8·72)
England	10·15 (9·91 to 10·4)	10·1 (9·87 to 10·35)	9·91 (9·69 to 10·16)	9·64 (9·42 to 9·88)	9·41 (9·2 to 9·65)	9·02 (8·82 to 9·25)	8·34 (8·15 to 8·55)	7·93 (7·75 to 8·13)	7·76 (7·59 to 7·95)	7·64 (7·48 to 7·83)
Northern Ireland	17·6 (17·2 to 18·04)	17·53 (17·14 to 17·97)	16·49 (16·12 to 16·91)	16·44 (16·06 to 16·87)	15·95 (15·56 to 16·37)	15·29 (14·91 to 15·7)	14·87 (14·51 to 15·27)	13·8 (13·47 to 14·17)	13·07 (12·78 to 13·4)	12·58 (12·32 to 12·88)
Scotland	16·51 (16·14 to 16·91)	16·5 (16·15 to 16·9)	16·11 (15·75 to 16·51)	16·17 (15·8 to 16·59)	16·08 (15·7 to 16·51)	15·33 (14·95 to 15·77)	14·26 (13·89 to 14·7)	14·11 (13·72 to 14·57)	12·91 (12·53 to 13·34)	12·38 (12·01 to 12·81)
Wales	15·16 (14·81 to 15·54)	15·15 (14·8 to 15·52)	14·94 (14·59 to 15·33)	14·57 (14·23 to 14·95)	14·26 (13·91 to 14·65)	13·82 (13·47 to 14·21)	12·95 (12·6 to 13·33)	12·37 (12·01 to 12·77)	12·15 (11·78 to 12·57)	12 (11·61 to 12·43)
**Southern Latin America**	15·37 (10·2 to 23·04)	15·17 (10·05 to 22·72)	14·94 (9·99 to 22·03)	14·57 (10·02 to 21·13)	14·09 (9·76 to 20·02)	13·57 (9·56 to 18·98)	12·99 (9·24 to 17·92)	12·35 (8·87 to 17·14)	11·59 (8·36 to 15·92)	10·87 (7·69 to 14·99)
Argentina	14·49 (10·47 to 19·86)	14·56 (10·48 to 19·88)	14·41 (10·34 to 19·66)	14·04 (9·99 to 19·09)	13·52 (9·77 to 18·13)	12·97 (9·33 to 17·39)	12·47 (9 to 16·7)	11·9 (8·54 to 16·09)	11·32 (8·23 to 15·2)	10·75 (7·75 to 14·55)
Chile	16·64 (8·69 to 29·77)	16·02 (8·3 to 28·56)	15·45 (8·06 to 27·47)	14·92 (7·88 to 25·93)	14·35 (7·84 to 24·35)	13·84 (7·56 to 23·05)	13·05 (7·18 to 21·75)	12·04 (6·56 to 20·38)	11·02 (6·02 to 18·77)	10·1 (5·59 to 16·88)
Uruguay	17·31 (14·42 to 20·79)	16·77 (13·95 to 20·2)	17·65 (14·75 to 21·25)	18·12 (15·17 to 21·67)	18·76 (15·77 to 22·26)	18·84 (15·88 to 22·2)	18·35 (15·49 to 21·53)	18·48 (15·62 to 21·62)	17·12 (14·35 to 20·09)	16·1 (13·43 to 19·08)
**Central Europe, Eastern Europe, and Central Asia**	12·69 (11·84 to 13·73)	11·99 (11·19 to 12·94)	11·82 (11·06 to 12·74)	11·38 (10·66 to 12·23)	11·11 (10·46 to 11·92)	11 (10·34 to 11·79)	10·82 (10·19 to 11·55)	10·63 (10·03 to 11·36)	10·35 (9·76 to 11·03)	10·27 (9·74 to 10·92)
**Eastern Europe**	9·04 (8·83 to 9·26)	8·55 (8·36 to 8·75)	8·77 (8·58 to 8·98)	8·5 (8·31 to 8·69)	8·48 (8·29 to 8·67)	8·5 (8·31 to 8·7)	8·62 (8·43 to 8·82)	8·45 (8·26 to 8·64)	8·27 (8·08 to 8·45)	8·3 (8·11 to 8·48)
Belarus	11·25 (11·08 to 11·45)	10·9 (10·74 to 11·08)	10·61 (10·45 to 10·78)	10·31 (10·15 to 10·48)	10·03 (9·87 to 10·19)	9·79 (9·64 to 9·95)	9·56 (9·41 to 9·71)	9·34 (9·2 to 9·49)	9·13 (8·99 to 9·27)	8·93 (8·79 to 9·07)
Estonia	6·74 (6·27 to 7·26)	6·53 (6·08 to 7·03)	6·78 (6·32 to 7·29)	6·2 (5·78 to 6·66)	6·67 (6·24 to 7·16)	6·51 (6·08 to 6·97)	6·7 (6·28 to 7·18)	6·62 (6·21 to 7·1)	6·24 (5·86 to 6·69)	6·53 (6·13 to 7)

Appendix Table 17A. Stillbirth rates (per 1,000 live births) by location, 1970-1979

	1970	1971	1972	1973	1974	1975	1976	1977	1978	1979
Latvia	6·72 (6·47 to 6·99)	6·48 (6·24 to 6·73)	6·32 (6·09 to 6·57)	6·28 (6·06 to 6·53)	6·75 (6·51 to 7·02)	7·22 (6·97 to 7·51)	7·14 (6·9 to 7·42)	6·67 (6·43 to 6·91)	6·58 (6·34 to 6·81)	6·74 (6·51 to 6·98)
Lithuania	6·66 (6·39 to 6·96)	5·82 (5·58 to 6·08)	6·21 (5·96 to 6·49)	6·26 (6·01 to 6·54)	6·41 (6·16 to 6·69)	6·43 (6·18 to 6·7)	6·2 (5·96 to 6·47)	6·02 (5·78 to 6·26)	6·18 (5·94 to 6·43)	6·04 (5·81 to 6·29)
Moldova	16·87 (16·02 to 17·82)	16·4 (15·58 to 17·29)	15·94 (15·15 to 16·82)	15·53 (14·77 to 16·41)	15·08 (14·34 to 15·93)	14·69 (13·97 to 15·54)	14·35 (13·63 to 15·15)	13·99 (13·29 to 14·79)	13·69 (13·02 to 14·45)	13·4 (12·74 to 14·13)
Russia	8·16 (7·94 to 8·39)	7·6 (7·4 to 7·82)	8·06 (7·84 to 8·28)	7·77 (7·55 to 7·98)	7·86 (7·64 to 8·08)	7·98 (7·76 to 8·2)	8·25 (8·02 to 8·47)	8·07 (7·85 to 8·29)	7·86 (7·64 to 8·06)	7·98 (7·76 to 8·19)
Ukraine	10·46 (10·33 to 10·6)	10·14 (10·02 to 10·27)	9·92 (9·79 to 10·05)	9·69 (9·57 to 9·82)	9·48 (9·36 to 9·61)	9·3 (9·18 to 9·43)	9·14 (9·02 to 9·26)	9·04 (8·92 to 9·16)	8·95 (8·83 to 9·06)	8·79 (8·68 to 8·9)
**Central Europe**	11·94 (10·25 to 14·13)	11·13 (9·54 to 13·14)	10·72 (9·24 to 12·63)	10·24 (8·83 to 11·99)	9·9 (8·59 to 11·51)	9·87 (8·55 to 11·5)	9·43 (8·19 to 11·04)	9·36 (8·12 to 10·95)	8·94 (7·7 to 10·48)	8·8 (7·6 to 10·29)
Albania	11·68 (11·5 to 11·88)	11·14 (10·97 to 11·32)	10·59 (10·43 to 10·77)	10·05 (9·89 to 10·22)	9·55 (9·39 to 9·71)	9·08 (8·93 to 9·23)	8·67 (8·53 to 8·82)	8·29 (8·15 to 8·42)	7·94 (7·81 to 8·08)	7·69 (7·56 to 7·82)
Bosnia and Herzegovina	16·71 (13·98 to 20·01)	16·02 (13·44 to 18·98)	15·38 (12·88 to 18·21)	14·76 (12·45 to 17·54)	14·14 (11·75 to 16·78)	13·54 (11·29 to 16·14)	12·99 (10·84 to 15·43)	12·45 (10·33 to 14·76)	11·9 (9·85 to 14·11)	11·34 (9·41 to 13·38)
Bulgaria	7·07 (5·8 to 8·61)	6·67 (5·49 to 8·13)	6·66 (5·49 to 8·12)	6·69 (5·56 to 8·15)	6·72 (5·59 to 8·18)	6·24 (5·16 to 7·63)	6·27 (5·16 to 7·61)	6·38 (5·27 to 7·67)	6·07 (5·03 to 7·25)	5·7 (4·73 to 6·81)
Croatia	8·79 (8·25 to 9·39)	8·49 (7·97 to 9·07)	8·21 (7·71 to 8·77)	7·95 (7·47 to 8·51)	7·71 (7·24 to 8·25)	7·51 (7·04 to 8·02)	7·32 (6·87 to 7·84)	7·12 (6·67 to 7·59)	6·92 (6·49 to 7·37)	6·73 (6·31 to 7·17)
Czech Republic	5·58 (4·82 to 6·47)	5·57 (4·82 to 6·44)	5·33 (4·6 to 6·15)	5·35 (4·65 to 6·19)	5·31 (4·61 to 6·11)	5·24 (4·54 to 6·03)	5·2 (4·53 to 5·95)	5·15 (4·5 to 5·88)	4·84 (4·23 to 5·51)	4·58 (3·96 to 5·23)
Hungary	12·09 (11·52 to 12·71)	11·74 (11·2 to 12·35)	11·16 (10·65 to 11·74)	11·06 (10·58 to 11·64)	11·81 (11·28 to 12·4)	11·38 (10·85 to 11·96)	10·22 (9·75 to 10·74)	9·03 (8·61 to 9·47)	8·35 (7·97 to 8·76)	8·1 (7·74 to 8·47)
Macedonia	19·61 (15·26 to 25·12)	18·98 (14·72 to 24·22)	18·42 (14·4 to 23·59)	17·85 (14·01 to 22·74)	17·32 (13·61 to 21·84)	16·89 (13·28 to 21·15)	16·61 (13·05 to 20·75)	16·46 (12·88 to 20·6)	16·36 (12·74 to 20·67)	16·31 (12·72 to 20·79)
Montenegro	9·82 (8·85 to 10·91)	9·58 (8·64 to 10·67)	9·36 (8·45 to 10·39)	9·15 (8·23 to 10·21)	8·92 (8·02 to 9·92)	8·72 (7·82 to 9·67)	8·51 (7·64 to 9·4)	8·3 (7·45 to 9·18)	8·08 (7·26 to 8·94)	7·88 (7·05 to 8·75)
Poland	13·98 (9·99 to 19·35)	12·81 (9·2 to 17·57)	12·36 (8·91 to 16·8)	11·71 (8·32 to 15·73)	10·76 (7·72 to 14·66)	11·25 (8·09 to 15·27)	10·85 (7·89 to 14·7)	11·11 (8·13 to 15·01)	10·55 (7·66 to 14·24)	10·02 (7·37 to 13·45)
Romania	12·64 (12·55 to 12·73)	11·27 (11·19 to 11·35)	10·95 (10·87 to 11·03)	10·21 (10·14 to 10·28)	10·06 (9·99 to 10·13)	9·86 (9·79 to 9·93)	9·05 (8·98 to 9·11)	9·2 (9·14 to 9·26)	8·65 (8·59 to 8·72)	9·19 (9·13 to 9·26)
Serbia	13 (11·77 to 14·39)	12·61 (11·4 to 13·93)	12·25 (11·08 to 13·54)	11·92 (10·82 to 13·14)	11·63 (10·53 to 12·8)	11·36 (10·29 to 12·49)	11·11 (10·07 to 12·19)	10·89 (9·85 to 11·96)	10·68 (9·68 to 11·68)	10·51 (9·56 to 11·52)
Slovakia	7·7 (7·36 to 8·07)	7·42 (7·09 to 7·77)	7·36 (7·03 to 7·71)	7·41 (7·09 to 7·76)	7·04 (6·73 to 7·36)	7 (6·7 to 7·32)	7·18 (6·88 to 7·51)	6·58 (6·31 to 6·87)	6·61 (6·33 to 6·9)	6·41 (6·14 to 6·68)
Slovenia	7·9 (7·04 to 8·89)	7·62 (6·79 to 8·55)	7·36 (6·56 to 8·27)	7·13 (6·35 to 8)	6·88 (6·14 to 7·72)	6·64 (5·9 to 7·44)	6·41 (5·71 to 7·2)	6·2 (5·49 to 6·95)	5·97 (5·3 to 6·68)	5·75 (5·09 to 6·43)
**Central Asia**	21·33 (20·27 to 22·52)	20·46 (19·44 to 21·6)	19·78 (18·82 to 20·85)	19·1 (18·2 to 20·12)	18·49 (17·65 to 19·45)	17·94 (17·16 to 18·87)	17·38 (16·63 to 18·24)	16·85 (16·13 to 17·65)	16·39 (15·71 to 17·13)	16·06 (15·4 to 16·75)
Armenia	24·12 (19·92 to 29·22)	22·75 (18·71 to 27·53)	21·57 (17·81 to 26·06)	20·42 (16·97 to 24·47)	19·38 (16·21 to 23·15)	18·48 (15·43 to 22·03)	17·54 (14·73 to 20·78)	16·74 (14·05 to 19·68)	16·11 (13·6 to 18·79)	15·62 (13·21 to 18·25)
Azerbaijan	27·39 (26·41 to 28·45)	25·96 (25·04 to 26·93)	24·73 (23·85 to 25·66)	23·46 (22·59 to 24·35)	22·27 (21·45 to 23·11)	21·21 (20·42 to 22·01)	20·09 (19·35 to 20·86)	19·6 (18·89 to 20·35)	19·4 (18·68 to 20·13)	19·4 (18·67 to 20·15)
Georgia	27·16 (24·76 to 29·88)	25·69 (23·41 to 28·23)	24·36 (22·22 to 26·78)	23·01 (21·01 to 25·32)	21·77 (19·81 to 23·95)	20·68 (18·84 to 22·69)	19·61 (17·81 to 21·5)	18·6 (16·87 to 20·39)	17·64 (15·97 to 19·35)	16·79 (15·18 to 18·43)
Kazakhstan	14·38 (13·4 to 15·47)	13·91 (12·94 to 14·94)	13·6 (12·68 to 14·62)	13·34 (12·45 to 14·32)	13·13 (12·28 to 14·08)	12·94 (12·1 to 13·86)	12·74 (11·92 to 13·61)	12·51 (11·72 to 13·34)	12·24 (11·46 to 13·02)	11·98 (11·22 to 12·76)
Kyrgyzstan	33·51 (32·31 to 34·81)	32·14 (30·99 to 33·35)	30·93 (29·82 to 32·1)	29·83 (28·74 to 30·96)	28·83 (27·82 to 29·94)	27·87 (26·89 to 28·98)	26·95 (25·97 to 27·99)	26·1 (25·16 to 27·06)	25·28 (24·32 to 26·22)	24·58 (23·62 to 25·49)
Mongolia	23·21 (22·38 to 24·12)	22·68 (21·87 to 23·56)	22·22 (21·43 to 23·1)	21·79 (21·01 to 22·65)	21·42 (20·65 to 22·26)	21·09 (20·33 to 21·93)	20·76 (20·01 to 21·56)	20·4 (19·66 to 21·2)	20·07 (19·33 to 20·84)	19·75 (19 to 20·5)
Tajikistan	29·79 (29·05 to 30·58)	28·39 (27·71 to 29·14)	28·05 (27·36 to 28·79)	27·63 (26·94 to 28·37)	27·2 (26·52 to 27·92)	26·58 (25·91 to 27·27)	25·94 (25·28 to 26·61)	24·94 (24·32 to 25·58)	23·7 (23·13 to 24·32)	22·57 (22·01 to 23·14)
Turkmenistan	27·08 (26·66 to 27·57)	26·09 (25·68 to 26·55)	25·23 (24·83 to 25·67)	24·34 (23·94 to 24·76)	23·5 (23·12 to 23·91)	22·76 (22·39 to 23·14)	21·99 (21·63 to 22·36)	21·25 (20·91 to 21·6)	20·57 (20·23 to 20·9)	19·91 (19·59 to 20·23)
Uzbekistan	17·33 (16·71 to 18)	16·72 (16·12 to 17·36)	16·17 (15·6 to 16·81)	15·57 (15·01 to 16·18)	15·08 (14·54 to 15·68)	14·69 (14·16 to 15·27)	14·23 (13·72 to 14·79)	13·77 (13·27 to 14·32)	13·49 (13 to 14·01)	13·43 (12·92 to 13·94)
**Latin America and Caribbean**	28·51 (24·23 to 33·81)	27·68 (23·51 to 32·74)	26·9 (22·93 to 31·79)	26·04 (22·31 to 30·69)	25·18 (21·75 to 29·49)	24·36 (21·06 to 28·43)	23·51 (20·38 to 27·31)	22·64 (19·68 to 26·16)	21·76 (18·96 to 25·03)	20·91 (18·27 to 23·95)
**Central Latin America**	18·6 (17·37 to 19·99)	18·25 (17·05 to 19·59)	17·87 (16·7 to 19·19)	17·41 (16·29 to 18·71)	16·88 (15·81 to 18·16)	16·31 (15·26 to 17·55)	15·74 (14·75 to 16·91)	15·19 (14·24 to 16·29)	14·65 (13·74 to 15·69)	14·16 (13·29 to 15·14)
Colombia	21·36 (19·28 to 23·73)	20·98 (18·96 to 23·23)	20·59 (18·66 to 22·87)	20·1 (18·21 to 22·4)	19·49 (17·65 to 21·75)	18·83 (17·06 to 21·01)	18·2 (16·49 to 20·28)	17·61 (15·91 to 19·57)	17·03 (15·38 to 18·95)	16·58 (14·98 to 18·41)
Costa Rica	13·2 (11·83 to 14·77)	12·86 (11·58 to 14·31)	12·5 (11·3 to 13·85)	12·11 (10·96 to 13·36)	11·76 (10·68 to 12·93)	11·43 (10·43 to 12·52)	11·08 (10·17 to 12·04)	10·7 (9·85 to 11·54)	10·36 (9·61 to 11·13)	10·06 (9·39 to 10·73)
El Salvador	28·39 (27·69 to 29·13)	27·4 (26·73 to 28·11)	26·35 (25·72 to 27·05)	25·22 (24·61 to 25·89)	24·2 (23·61 to 24·84)	23·27 (22·7 to 23·89)	22·36 (21·81 to 22·95)	21·47 (20·94 to 22·04)	20·64 (20·12 to 21·17)	19·9 (19·39 to 20·42)
Guatemala	30·74 (26·77 to 35·39)	30·13 (26·2 to 34·57)	29·63 (25·81 to 34·03)	29·17 (25·56 to 33·55)	28·57 (25·05 to 32·76)	27·83 (24·38 to 31·81)	27·17 (23·78 to 30·99)	26·64 (23·33 to 30·3)	26·09 (22·83 to 29·65)	25·56 (22·33 to 28·91)
Honduras	28·69 (28·54 to 28·84)	27·83 (27·69 to 27·98)	26·99 (26·85 to 27·13)	26·15 (26·01 to 26·29)	25·38 (25·25 to 25·52)	24·67 (24·54 to 24·8)	23·84 (23·71 to 23·96)	22·94 (22·82 to 23·06)	21·99 (21·88 to 22·11)	21·11 (21 to 21·23)
Mexico	14·95 (14·26 to 15·71)	14·61 (13·94 to 15·33)	14·24 (13·6 to 14·97)	13·85 (13·24 to 14·59)	13·47 (12·86 to 14·18)	13·07 (12·48 to 13·75)	12·68 (12·11 to 13·33)	12·29 (11·73 to 12·92)	11·88 (11·34 to 12·48)	11·45 (10·93 to 12·03)
Nicaragua	28·86 (28·46 to 29·29)	27·78 (27·4 to 28·2)	26·79 (26·42 to 27·19)	25·79 (25·43 to 26·17)	24·65 (24·31 to 25·02)	23·66 (23·33 to 24)	22·62 (22·3 to 22·96)	21·52 (21·22 to 21·84)	20·58 (20·29 to 20·89)	19·96 (19·67 to 20·26)
Panama	11·76 (10·63 to 13·04)	11·31 (10·2 to 12·53)	10·89 (9·83 to 12·06)	10·55 (9·54 to 11·69)	10·25 (9·31 to 11·31)	9·98 (9·05 to 10·99)	9·75 (8·85 to 10·72)	9·56 (8·69 to 10·48)	9·37 (8·51 to 10·28)	9·2 (8·36 to 10·08)
Venezuela	17·2 (15·8 to 18·77)	17·59 (16·17 to 19·19)	17·75 (16·33 to 19·4)	17·46 (16·04 to 19·07)	16·76 (15·39 to 18·34)	15·89 (14·6 to 17·38)	14·95 (13·74 to 16·34)	14·04 (12·94 to 15·34)	13·29 (12·22 to 14·48)	12·73 (11·71 to 13·88)
**Andean Latin America**	43·81 (36·68 to 52·4)	42·19 (35·32 to 50·23)	40·73 (34·25 to 48·53)	39·35 (33·16 to 46·83)	38·02 (32·13 to 45·05)	36·82 (31·23 to 43·45)	35·66 (30·42 to 41·88)	34·48 (29·43 to 40·38)	33·33 (28·49 to 39·01)	32·21 (27·7 to 37·48)
Bolivia	56·71 (47·26 to 68·11)	54·78 (45·75 to 65·28)	52·89 (44·06 to 63·28)	50·98 (42·19 to 60·97)	49·07 (40·67 to 58·89)	47·3 (39·25 to 56·79)	45·59 (37·75 to 54·6)	43·91 (36·61 to 52·55)	42·39 (35·19 to 50·74)	41·01 (33·92 to 49·09)
Ecuador	29·2 (23·55 to 36·18)	28 (22·7 to 34·27)	27·07 (21·85 to 33·31)	26·23 (21·06 to 32·23)	25·5 (20·43 to 31·28)	24·86 (19·9 to 30·42)	24·11 (19·33 to 29·44)	23·13 (18·51 to 28·26)	22·11 (17·74 to 26·9)	21·26 (17·05 to 25·92)
Peru	45·13 (38·31 to 53·25)	43·49 (36·85 to 51·28)	42·04 (35·68 to 49·51)	40·69 (34·55 to 47·89)	39·38 (33·49 to 46·25)	38·21 (32·55 to 44·94)	37·14 (31·78 to 43·48)	36·13 (30·82 to 42·13)	35·14 (29·99 to 40·72)	34·05 (29·1 to 39·43)
**Caribbean**	38·9 (31·57 to 48·89)	37·83 (30·72 to 47·65)	37·18 (30·55 to 46·31)	36·47 (30·08 to 44·8)	35·78 (29·85 to 43·42)	35·09 (29·42 to 41·97)	34·41 (29·02 to 40·86)	33·95 (28·75 to 40·21)	33·34 (28·18 to 39·37)	32·47 (27·39 to 38·36)
Antigua and Barbuda	14·8 (12·06 to 18·16)	14·28 (11·66 to 17·46)	13·89 (11·32 to 17·04)	13·47 (10·96 to 16·42)	13·56 (10·96 to 16·56)	13·6 (10·99 to 16·61)	13·56 (10·98 to 16·58)	13·37 (10·84 to 16·34)	13·16 (10·65 to 16·07)	12·61 (10·17 to 15·45)
The Bahamas	23·76 (14·54 to 37·51)	22·63 (13·91 to 35·7)	21·46 (13·31 to 33·77)	20·12 (12·57 to 31·64)	19·53 (12·33 to 30·19)	20·33 (12·73 to 30·89)	19·2 (12·04 to 29·36)	18·59 (11·55 to 27·63)	18·12 (11·27 to 26·98)	17·96 (11·35 to 27·11)
Barbados	20·76 (16·91 to 25·48)	19·5 (15·92 to 23·72)	18·51 (15·07 to 22·62)	17·55 (14·22 to 21·46)	16·72 (13·54 to 20·51)	15·86 (12·86 to 19·46)	15·16 (12·25 to 18·54)	14·49 (11·81 to 17·71)	13·93 (11·3 to 17·03)	13·34 (10·79 to 16·3)
Belize	27·1 (22·08 to 33·26)	26·14 (21·34 to 31·96)	25·08 (20·43 to 30·77)	24 (19·53 to 29·25)	22·99 (18·58 to 28·08)	22·17 (17·92 to 27·08)	21·46 (17·38 to 26·23)	20·6 (16·7 to 25·19)	19·8 (16·02 to 24·17)	19·26 (15·53 to 23·58)
Bermuda	18·4 (14·1 to 23·9)	18·15 (13·9 to 23·55)	17·53 (13·49 to 22·67)	16·44 (12·68 to 21·21)	16·01 (12·28 to 20·62)	15·88 (12·23 to 20·38)	16·28 (12·47 to 20·84)	16·22 (12·51 to 20·78)	15·7 (12·07 to 19·87)	15·18 (11·66 to 19·22)
Cuba	21·65 (11·9 to 37·24)	21·06 (11·64 to 36·56)	20·22 (11·34 to 34·3)	19·56 (10·99 to 32·52)	19·02 (10·62 to 31·03)	18·46 (10·33 to 30·31)	17·84 (9·93 to 29·92)	17·31 (9·56 to 29·48)	16·71 (9·36 to 28·55)	15·99 (9·01 to 27·42)
Dominica	14·3 (11·65 to 17·55)	13·48 (11 to 16·48)	12·7 (10·34 to 15·57)	11·97 (9·74 to 14·59)	11·35 (9·17 to 13·87)	10·82 (8·74 to 13·21)	10·37 (8·4 to 12·68)	10·01 (8·12 to 12·24)	9·61 (7·78 to 11·74)	9·29 (7·49 to 11·38)
Dominican Republic	34·51 (31·09 to 38·8)	33·62 (30·19 to 37·66)	32·56 (29·18 to 36·53)	31·36 (28·14 to 35·11)	30·38 (27·27 to 33·99)	29·71 (26·65 to 33·3)	28·99 (25·98 to 32·52)	27·92 (25·04 to 31·21)	26·78 (24·02 to 29·94)	25·77 (23·07 to 28·76)
Grenada	19·25 (15·68 to 23·63)	18·89 (15·43 to 22·98)	18·56 (15·12 to 22·69)	18·23 (14·76 to 22·29)	17·88 (14·48 to 21·93)	17·41 (14·11 to 21·35)	16·94 (13·69 to 20·72)	16·52 (13·47 to 20·2)	16·11 (13·07 to 19·69)	15·56 (12·6 to 19·02)

Appendix Table 17A. Stillbirth rates (per 1,000 live births) by location, 1970-1979

	1970	1971	1972	1973	1974	1975	1976	1977	1978	1979
Guyana	33·9 (31·51 to 36·56)	33·12 (30·8 to 35·69)	32·4 (30·12 to 34·93)	31·68 (29·5 to 34·16)	30·93 (28·81 to 33·37)	29·91 (27·84 to 32·21)	28·65 (26·62 to 30·86)	27·7 (25·74 to 29·82)	26·77 (24·88 to 28·85)	26·05 (24·11 to 28·09)
Haiti	81·27 (66·2 to 99·74)	79·49 (64·89 to 97·18)	77·47 (63·11 to 95·02)	74·64 (60·73 to 90·96)	71·59 (57·85 to 87·42)	69·4 (56·09 to 84·75)	67·7 (54·83 to 82·77)	66·22 (53·69 to 80·97)	64·59 (52·29 to 78·88)	62·29 (50·23 to 76·29)
Jamaica	24·04 (21·31 to 27·19)	23·59 (20·91 to 26·61)	22·86 (20·29 to 25·79)	22·22 (19·8 to 25·12)	21·76 (19·38 to 24·49)	21·31 (18·97 to 23·9)	20·67 (18·44 to 23·18)	20·03 (17·87 to 22·39)	19·56 (17·42 to 21·84)	19·23 (17·11 to 21·43)
Puerto Rico	18·04 (17·22 to 18·94)	17·38 (16·6 to 18·22)	16·73 (15·99 to 17·54)	15·98 (15·26 to 16·76)	15·26 (14·58 to 15·99)	14·64 (13·98 to 15·34)	14·15 (13·51 to 14·82)	13·81 (13·18 to 14·45)	13·57 (12·96 to 14·2)	13·42 (12·81 to 14·03)
Saint Lucia	19·79 (17·56 to 22·36)	18·99 (16·89 to 21·41)	18·23 (16·2 to 20·6)	17·54 (15·52 to 19·81)	16·89 (14·93 to 19·09)	16·45 (14·47 to 18·57)	15·94 (14·01 to 17·95)	15·5 (13·62 to 17·44)	15·22 (13·37 to 17·12)	15 (13·19 to 16·88)
Saint Vincent and the Grenadines	13·48 (8·96 to 19·86)	13 (8·59 to 19·17)	12·48 (8·27 to 18·41)	12·03 (7·99 to 17·49)	11·66 (7·82 to 16·83)	11·41 (7·67 to 16·41)	11·11 (7·49 to 15·94)	10·87 (7·37 to 15·39)	10·62 (7·19 to 15)	10·37 (7·01 to 14·7)
Suriname	24·9 (21·39 to 29·04)	24·53 (21·06 to 28·53)	23·99 (20·66 to 28·02)	23·79 (20·65 to 27·77)	23·82 (20·64 to 27·85)	23·77 (20·56 to 27·71)	23·65 (20·47 to 27·41)	23·38 (20·2 to 26·88)	23·02 (19·9 to 26·41)	22·65 (19·56 to 25·96)
Trinidad and Tobago	20·82 (17·95 to 24·54)	20·57 (17·86 to 24·12)	20·27 (17·56 to 23·63)	19·99 (17·36 to 23·18)	19·48 (16·95 to 22·5)	19·01 (16·67 to 21·72)	18·47 (16·31 to 21·01)	17·89 (15·87 to 20·22)	17·25 (15·37 to 19·29)	16·61 (14·93 to 18·43)
Virgin Islands, U.S.	13·56 (11·04 to 16·64)	13·32 (10·88 to 16·21)	13·08 (10·65 to 15·98)	12·85 (10·41 to 15·72)	12·61 (10·21 to 15·46)	12·4 (10·05 to 15·2)	12·19 (9·85 to 14·91)	11·99 (9·77 to 14·66)	11·8 (9·57 to 14·42)	11·58 (9·37 to 14·15)
**Tropical Latin America**	32·49 (26·55 to 39·75)	31·4 (25·56 to 38·39)	30·39 (24·84 to 37·06)	29·34 (24·15 to 35·79)	28·34 (23·26 to 34·49)	27·4 (22·66 to 33·28)	26·34 (21·71 to 31·81)	25·2 (20·72 to 30·38)	24·01 (19·77 to 28·74)	22·84 (18·75 to 27·33)
Brazil	32·72 (26·79 to 39·96)	31·61 (25·78 to 38·58)	30·6 (25·07 to 37·25)	29·53 (24·35 to 35·94)	28·53 (23·46 to 34·62)	27·58 (22·84 to 33·39)	26·49 (21·89 to 31·96)	25·34 (20·88 to 30·48)	24·13 (19·91 to 28·83)	22·94 (18·88 to 27·4)
Paraguay	23·16 (16·86 to 31·53)	22·74 (16·54 to 30·82)	22·23 (16·16 to 30·27)	21·66 (16·06 to 29·42)	21·08 (15·63 to 28·89)	20·55 (15·16 to 27·93)	20·05 (14·68 to 26·89)	19·55 (14·32 to 25·86)	19·05 (14·04 to 25·14)	18·55 (13·65 to 24·44)
**Southeast Asia, East Asia, and Oceania**	29·4 (26·98 to 32·15)	28·33 (26·03 to 30·85)	27·37 (25·17 to 29·89)	26·45 (24·4 to 28·89)	25·63 (23·7 to 27·94)	24·81 (22·95 to 27·02)	24·05 (22·3 to 26·08)	23·36 (21·69 to 25·21)	22·61 (21 to 24·32)	21·92 (20·39 to 23·54)
**East Asia**	27·79 (24·96 to 31·03)	26·72 (24·01 to 29·73)	25·76 (23·17 to 28·78)	24·83 (22·41 to 27·73)	24·01 (21·7 to 26·85)	23·17 (20·92 to 25·87)	22·44 (20·25 to 24·94)	21·79 (19·68 to 24·08)	21·09 (19·05 to 23·22)	20·44 (18·44 to 22·53)
China	28·24 (25·36 to 31·53)	27·16 (24·41 to 30·22)	26·19 (23·55 to 29·27)	25·25 (22·79 to 28·19)	24·43 (22·08 to 27·31)	23·6 (21·31 to 26·35)	22·88 (20·64 to 25·43)	22·21 (20·06 to 24·55)	21·51 (19·43 to 23·7)	20·84 (18·79 to 22·99)
North Korea	15·17 (13·2 to 17·47)	14·36 (12·53 to 16·5)	13·63 (11·85 to 15·66)	13 (11·27 to 14·93)	12·46 (10·82 to 14·33)	12·04 (10·44 to 13·87)	11·66 (10·11 to 13·39)	11·27 (9·81 to 12·95)	11·01 (9·55 to 12·65)	10·72 (9·28 to 12·32)
Taiwan (Province of China)	15·32 (14·52 to 16·2)	14·65 (13·88 to 15·48)	13·62 (12·89 to 14·41)	13·45 (12·74 to 14·22)	12·82 (12·15 to 13·58)	12·22 (11·57 to 12·93)	11·41 (10·82 to 12·08)	10·98 (10·42 to 11·62)	10·42 (9·87 to 11·02)	10·05 (9·51 to 10·62)
**Southeast Asia**	33·76 (32·49 to 35·16)	32·56 (31·34 to 33·91)	31·46 (30·28 to 32·76)	30·38 (29·25 to 31·63)	29·36 (28·29 to 30·55)	28·39 (27·38 to 29·5)	27·44 (26·44 to 28·5)	26·52 (25·58 to 27·53)	25·56 (24·69 to 26·5)	24·71 (23·86 to 25·6)
Cambodia	48·7 (47·33 to 50·17)	48·05 (46·7 to 49·49)	47·45 (46·11 to 48·88)	47·82 (46·51 to 49·26)	47·87 (46·57 to 49·33)	47·04 (45·74 to 48·43)	46·16 (44·85 to 47·52)	45·27 (44 to 46·61)	44·12 (42·87 to 45·44)	42·99 (41·72 to 44·29)
Indonesia	40·93 (39·87 to 42·07)	39·54 (38·52 to 40·64)	38·14 (37·14 to 39·2)	36·71 (35·78 to 37·74)	35·31 (34·43 to 36·31)	34·12 (33·25 to 35·06)	33·02 (32·15 to 33·92)	31·91 (31·08 to 32·78)	30·9 (30·09 to 31·75)	29·89 (29·07 to 30·72)
Laos	74·54 (73·36 to 75·8)	73·09 (71·95 to 74·35)	71·67 (70·55 to 72·89)	70·16 (69·07 to 71·36)	68·65 (67·57 to 69·84)	67·1 (66·03 to 68·25)	65·47 (64·42 to 66·58)	64·28 (63·25 to 65·38)	63 (61·97 to 64·06)	61·77 (60·73 to 62·79)
Malaysia	12·89 (10·65 to 15·62)	12·46 (10·27 to 15·07)	11·86 (9·82 to 14·34)	11·44 (9·45 to 13·8)	11·08 (9·23 to 13·3)	10·79 (8·98 to 12·91)	10·41 (8·68 to 12·43)	10·7 (8·92 to 12·73)	9·79 (8·17 to 11·56)	9·27 (7·73 to 11)
Maldives	126·24 (101·99 to 156·14)	121·65 (97·88 to 149·74)	117·15 (94·43 to 145·44)	113·18 (91·05 to 139·87)	109·29 (88·54 to 134·21)	105·05 (85·27 to 129·05)	102·76 (83·86 to 125·47)	100·52 (81·75 to 121·93)	97·37 (79·32 to 117·65)	93·95 (76·28 to 113·57)
Mauritius	36·2 (32·33 to 40·62)	34·65 (31·03 to 38·83)	32·93 (29·56 to 36·86)	31·04 (27·82 to 34·76)	29·1 (25·94 to 32·51)	27·46 (24·59 to 30·75)	25·78 (22·98 to 28·87)	24·32 (21·69 to 27·21)	23·02 (20·54 to 25·63)	21·94 (19·69 to 24·39)
Myanmar	41·45 (40·94 to 42)	39·28 (38·79 to 39·79)	37·42 (36·95 to 37·91)	35·79 (35·35 to 36·26)	34·3 (33·89 to 34·76)	32·99 (32·59 to 33·42)	31·77 (31·38 to 32·18)	30·69 (30·3 to 31·08)	29·67 (29·3 to 30·06)	28·72 (28·34 to 29·1)
Philippines	18·98 (18·68 to 19·3)	18·64 (18·34 to 18·95)	18·3 (18·01 to 18·61)	17·93 (17·66 to 18·23)	17·57 (17·3 to 17·87)	17·22 (16·95 to 17·5)	16·89 (16·62 to 17·16)	16·55 (16·28 to 16·82)	16·2 (15·94 to 16·47)	15·86 (15·6 to 16·13)
Sri Lanka	21·58 (20·47 to 22·79)	20·6 (19·57 to 21·75)	20·86 (19·81 to 22·05)	20·55 (19·51 to 21·75)	21·02 (19·96 to 22·23)	20·29 (19·29 to 21·45)	19·48 (18·53 to 20·56)	18·3 (17·42 to 19·29)	16·13 (15·33 to 17·02)	15·66 (14·87 to 16·52)
Seychelles	23·79 (21·88 to 25·93)	22·79 (20·95 to 24·87)	21·93 (20·17 to 23·92)	21·12 (19·5 to 23·02)	20·53 (18·98 to 22·39)	19·81 (18·26 to 21·66)	18·88 (17·37 to 20·64)	17·94 (16·47 to 19·62)	16·8 (15·49 to 18·33)	15·61 (14·39 to 17)
Thailand	24·14 (21·61 to 27·05)	22·98 (20·57 to 25·75)	21·93 (19·67 to 24·51)	20·88 (18·7 to 23·36)	19·92 (17·84 to 22·23)	19·02 (16·98 to 21·23)	18·13 (16·22 to 20·18)	17·24 (15·41 to 19·19)	16·37 (14·63 to 18·23)	15·56 (13·89 to 17·35)
Timor-Leste	73·03 (72·43 to 73·66)	70·55 (69·97 to 71·15)	68·07 (67·52 to 68·66)	65·56 (65·04 to 66·13)	62·9 (62·4 to 63·45)	60·44 (59·96 to 60·96)	58·79 (58·3 to 59·29)	57·98 (57·5 to 58·47)	57·63 (57·15 to 58·12)	56·73 (56·24 to 57·23)
Vietnam	30·67 (28·93 to 32·58)	29·92 (28·23 to 31·77)	29·18 (27·53 to 30·99)	28·48 (26·9 to 30·25)	27·76 (26·24 to 29·51)	27·1 (25·6 to 28·76)	26·33 (24·83 to 27·94)	25·46 (24·02 to 27·01)	24·69 (23·29 to 26·22)	23·96 (22·53 to 25·45)
**Oceania**	33·46 (30·84 to 36·4)	33·19 (30·58 to 36)	32·94 (30·34 to 35·84)	32·7 (30·1 to 35·6)	32·45 (29·84 to 35·38)	32·31 (29·76 to 35·24)	32·18 (29·69 to 35·11)	32·15 (29·66 to 35·07)	32·31 (29·84 to 35·16)	32·41 (29·9 to 35·24)
American Samoa	14·73 (13·55 to 16·06)	14·29 (13·13 to 15·54)	13·92 (12·79 to 15·18)	13·52 (12·41 to 14·75)	13·12 (12·03 to 14·34)	12·72 (11·68 to 13·91)	12·33 (11·34 to 13·5)	11·98 (11·02 to 13·1)	11·63 (10·71 to 12·7)	11·31 (10·4 to 12·32)
Federated States of Micronesia	25 (22·99 to 27·26)	24·53 (22·55 to 26·71)	24·13 (22·17 to 26·36)	23·76 (21·82 to 25·97)	23·4 (21·47 to 25·56)	23·16 (21·27 to 25·33)	22·94 (21·09 to 25·11)	22·63 (20·81 to 24·75)	22·38 (20·61 to 24·43)	21·95 (20·19 to 23·92)
Fiji	12·03 (11·69 to 12·4)	12·16 (11·82 to 12·53)	12·24 (11·89 to 12·61)	12·2 (11·87 to 12·58)	12·11 (11·78 to 12·48)	12·17 (11·83 to 12·54)	11·78 (11·44 to 12·13)	11·53 (11·2 to 11·87)	11·59 (11·26 to 11·94)	11·08 (10·75 to 11·42)
Guam	19·13 (18·47 to 19·85)	18·7 (18·06 to 19·38)	18·2 (17·6 to 18·88)	17·79 (17·2 to 18·47)	17·29 (16·71 to 17·96)	16·68 (16·13 to 17·33)	15·97 (15·44 to 16·58)	15·16 (14·64 to 15·73)	14·38 (13·89 to 14·93)	13·5 (13·04 to 14)
Kiribati	34·94 (32·13 to 38·09)	34·64 (31·84 to 37·71)	34·23 (31·45 to 37·4)	33·6 (30·86 to 36·73)	32·28 (29·62 to 35·26)	31·11 (28·56 to 34·02)	30·68 (28·2 to 33·58)	30·38 (27·94 to 33·23)	30·06 (27·69 to 32·81)	29·82 (27·43 to 32·49)
Marshall Islands	19·61 (18·03 to 21·38)	19·26 (17·71 to 20·98)	18·99 (17·45 to 20·75)	18·56 (17·04 to 20·28)	18·16 (16·66 to 19·84)	17·95 (16·48 to 19·63)	17·78 (16·35 to 19·47)	17·59 (16·18 to 19·24)	17·41 (16·04 to 19·01)	17·23 (15·85 to 18·77)
Northern Mariana Islands	11·45 (10·53 to 12·48)	11·03 (10·16 to 12·02)	10·66 (9·81 to 11·65)	10·25 (9·43 to 11·19)	9·71 (8·94 to 10·6)	9·08 (8·35 to 9·91)	8·6 (7·9 to 9·38)	8·23 (7·54 to 8·97)	7·93 (7·25 to 8·65)	7·64 (6·98 to 8·32)
Papua New Guinea	39·44 (36·27 to 43·01)	39·04 (35·88 to 42·44)	38·69 (35·55 to 42·2)	38·47 (35·32 to 41·97)	38·3 (35·12 to 41·87)	38·27 (35·14 to 41·85)	38·39 (35·32 to 42·02)	38·63 (35·53 to 42·25)	38·94 (35·87 to 42·51)	39·28 (36·14 to 42·8)
Samoa	16·57 (15·24 to 18·07)	16·26 (14·94 to 17·67)	15·98 (14·68 to 17·43)	15·88 (14·58 to 17·33)	15·68 (14·38 to 17·14)	15·17 (13·93 to 16·59)	14·53 (13·37 to 15·9)	14·01 (12·88 to 15·32)	13·46 (12·39 to 14·69)	13·24 (12·18 to 14·43)
Solomon Islands	33·55 (30·85 to 36·58)	32·57 (29·93 to 35·4)	31·63 (29·06 to 34·49)	30·83 (28·3 to 33·63)	29·74 (27·27 to 32·51)	28·93 (26·56 to 31·63)	27·93 (25·69 to 30·57)	26·9 (24·74 to 29·42)	25·91 (23·86 to 28·28)	24·66 (22·69 to 26·87)
Tonga	14·68 (13·5 to 16)	14·54 (13·36 to 15·8)	14·43 (13·26 to 15·74)	14·42 (13·24 to 15·74)	14·31 (13·12 to 15·64)	14·26 (13·09 to 15·6)	14·25 (13·11 to 15·6)	14·26 (13·12 to 15·6)	14·27 (13·14 to 15·58)	14·34 (13·19 to 15·62)
Vanuatu	28·88 (26·56 to 31·49)	28·26 (25·97 to 30·72)	27·7 (25·45 to 30·21)	27·01 (24·8 to 29·47)	25·88 (23·74 to 28·3)	25·03 (22·98 to 27·37)	24·27 (22·33 to 26·56)	23·7 (21·8 to 25·92)	23·07 (21·25 to 25·18)	22·46 (20·67 to 24·47)
**North Africa and Middle East**	52·59 (45·22 to 61·41)	50·53 (43·43 to 58·76)	48·51 (41·79 to 56·48)	46·51 (40·26 to 53·99)	44·34 (38·49 to 51·29)	42·24 (36·77 to 48·81)	40·24 (35·07 to 46·29)	38·36 (33·53 to 43·93)	36·66 (32·22 to 41·81)	35·07 (30·89 to 39·86)
**North Africa and Middle East**	52·59 (45·22 to 61·41)	50·53 (43·43 to 58·76)	48·51 (41·79 to 56·48)	46·51 (40·26 to 53·99)	44·34 (38·49 to 51·29)	42·24 (36·77 to 48·81)	40·24 (35·07 to 46·29)	38·36 (33·53 to 43·93)	36·66 (32·22 to 41·81)	35·07 (30·89 to 39·86)
Afghanistan	44·51 (38·31 to 51·82)	44·21 (38·15 to 51·29)	44·14 (37·93 to 51·22)	43·95 (37·83 to 50·96)	43·66 (37·53 to 50·65)	43·26 (37·1 to 50·17)	42·76 (36·77 to 49·67)	42·41 (36·51 to 49·23)	41·96 (36·26 to 48·45)	41·51 (35·66 to 47·84)
Algeria	61·71 (48·93 to 77·69)	59·88 (47·74 to 75·32)	57·66 (45·77 to 72·49)	55·47 (44·16 to 68·95)	53·03 (42·38 to 65·66)	50·85 (41 to 62·27)	48·51 (39·05 to 58·88)	46·04 (37·01 to 56·03)	43·69 (35·13 to 53·52)	41·18 (33·04 to 50·68)
Bahrain	33·39 (32·83 to 33·99)	30·94 (30·42 to 31·49)	28·64 (28·16 to 29·15)	26·26 (25·83 to 26·73)	24 (23·61 to 24·43)	22·29 (21·92 to 22·68)	20·59 (20·25 to 20·95)	19·11 (18·79 to 19·44)	17·8 (17·51 to 18·12)	16·54 (16·26 to 16·84)
Egypt	64·62 (55·22 to 75·77)	63·71 (54·54 to 74·5)	62·51 (53·47 to 73·16)	60·95 (52·07 to 71·22)	58·88 (50·08 to 68·73)	56·56 (48·03 to 66·05)	54·2 (46·08 to 63·4)	51·63 (44 to 60·27)	49·07 (41·79 to 57·28)	46·88 (39·68 to 54·8)
Iran	60·57 (53·4 to 68·86)	55·3 (48·69 to 62·76)	50·28 (44·37 to 57)	45·25 (40·17 to 51·36)	40·21 (35·61 to 45·49)	35·98 (31·81 to 40·57)	32·72 (29·05 to 36·86)	30·37 (26·93 to 34·13)	28·83 (25·5 to 32·32)	27·51 (24·34 to 30·9)
Iraq	34·84 (29·76 to 40·85)	33·56 (28·72 to 39·23)	32·46 (27·73 to 38·04)	31·25 (26·68 to 36·44)	30·01 (25·49 to 35·05)	28·63 (24·31 to 33·43)	27·14 (23·09 to 31·73)	25·85 (22·01 to 30·22)	24·55 (20·87 to 28·67)	23·15 (19·63 to 27·1)
Jordan	26·67 (24·57 to 29·02)	25·78 (23·77 to 28·03)	24·78 (22·83 to 26·96)	23·97 (22·13 to 26·11)	23·12 (21·35 to 25·17)	22·31 (20·59 to 24·23)	21·3 (19·63 to 23·13)	20·19 (18·6 to 21·91)	19 (17·52 to 20·65)	17·88 (16·42 to 19·44)
Kuwait	15·5 (12·55 to 19·14)	14·75 (11·96 to 18·2)	15·44 (12·51 to 19·04)	16·2 (13·17 to 19·91)	16·3 (13·14 to 19·94)	15·36 (12·49 to 18·59)	13·96 (11·38 to 16·87)	15·37 (12·53 to 18·57)	13·88 (11·29 to 16·86)	13·14 (10·68 to 16·05)

Appendix Table 17A. Stillbirth rates (per 1,000 live births) by location, 1970-1979

	1970	1971	1972	1973	1974	1975	1976	1977	1978	1979
Lebanon	29·84 (28·17 to 31·67)	29·62 (27·98 to 31·39)	28·73 (27·09 to 30·38)	27·4 (25·86 to 28·96)	25·76 (24·26 to 27·32)	24·31 (22·88 to 25·75)	23·36 (21·97 to 24·75)	22·27 (20·98 to 23·57)	21·39 (20·13 to 22·64)	20·6 (19·41 to 21·81)
Libya	25·92 (22·15 to 30·4)	24·34 (20·83 to 28·46)	23·03 (19·68 to 26·99)	21·87 (18·67 to 25·51)	20·8 (17·66 to 24·29)	19·82 (16·84 to 23·15)	18·79 (15·99 to 21·97)	17·76 (15·12 to 20·77)	16·83 (14·31 to 19·66)	15·92 (13·5 to 18·64)
Morocco	52·05 (46·93 to 57·88)	50·64 (45·6 to 56·25)	49·36 (44·49 to 54·99)	48·05 (43·55 to 53·24)	46·62 (42·27 to 51·52)	45·13 (40·85 to 49·83)	43·51 (39·41 to 47·83)	41·89 (38·01 to 46)	40·31 (36·64 to 44·18)	38·75 (35·34 to 42·57)
Palestine	14·17 (12·51 to 16·08)	13·55 (11·99 to 15·31)	13·01 (11·55 to 14·76)	12·86 (11·42 to 14·65)	12·98 (11·52 to 14·83)	13·13 (11·68 to 15)	13·06 (11·6 to 14·86)	12·88 (11·41 to 14·61)	12·27 (10·87 to 13·91)	11·66 (10·33 to 13·2)
Oman	102·46 (95·57 to 110·12)	97·31 (90·84 to 104·47)	92·38 (86·01 to 99·23)	88·46 (82·25 to 94·81)	83·21 (77·64 to 89·37)	77·81 (72·6 to 83·39)	71·88 (67·11 to 76·84)	66·99 (62·64 to 71·41)	62·67 (58·67 to 67·01)	58·48 (54·7 to 62·32)
Qatar	12·6 (9·08 to 17·3)	12·78 (9·21 to 17·38)	12·94 (9·34 to 17·76)	13·23 (9·42 to 18·19)	12·32 (8·63 to 17·07)	11·53 (8·16 to 15·84)	10·45 (7·28 to 14·29)	9·54 (6·71 to 13)	8·79 (6·23 to 11·89)	7·99 (5·72 to 10·68)
Saudi Arabia	46·18 (34·28 to 61·75)	44·55 (32·88 to 59·23)	42·12 (31·32 to 56·29)	39·96 (29·58 to 53·12)	37·64 (28·07 to 49·88)	36·01 (27·02 to 47·78)	34·48 (25·83 to 45·42)	32·74 (24·57 to 42·65)	31·41 (23·58 to 40·68)	30·3 (22·66 to 39·36)
Sudan	46·65 (42·16 to 51·75)	46·16 (41·68 to 51·17)	45·95 (41·54 to 50·93)	45·99 (41·66 to 50·91)	45·89 (41·52 to 50·86)	45·55 (41·18 to 50·34)	44·94 (40·56 to 49·54)	44·15 (39·87 to 48·76)	43·56 (39·37 to 47·99)	43·18 (38·97 to 47·59)
Syria	22·99 (19·64 to 26·95)	22·35 (19·13 to 26·13)	21·56 (18·44 to 25·23)	20·95 (17·9 to 24·49)	20·21 (17·18 to 23·58)	19·36 (16·44 to 22·61)	18·6 (15·81 to 21·76)	18 (15·34 to 21·01)	17·43 (14·84 to 20·34)	16·93 (14·33 to 19·79)
Tunisia	25·43 (16·37 to 38·5)	24·39 (15·97 to 36·48)	23·27 (15·32 to 34·61)	22·31 (14·64 to 32·63)	21·3 (14·05 to 31·1)	20·29 (13·5 to 29·08)	19·36 (12·89 to 27·69)	18·43 (12·3 to 26·78)	17·53 (11·73 to 25·11)	16·67 (11·27 to 24·12)
Turkey	51·02 (43·6 to 59·82)	48·79 (41·75 to 57·03)	46·99 (40·16 to 55·08)	45·58 (38·91 to 53·15)	44·02 (37·38 to 51·42)	42·32 (35·94 to 49·43)	40·44 (34·41 to 47·28)	38·57 (32·84 to 45·09)	36·77 (31·27 to 42·95)	34·89 (29·58 to 40·84)
United Arab Emirates	22·67 (20·8 to 24·78)	21·52 (19·71 to 23·52)	20·36 (18·62 to 22·27)	19·43 (17·77 to 21·21)	18·33 (16·78 to 20·04)	17·25 (15·79 to 18·85)	16·27 (14·95 to 17·73)	15·29 (14·02 to 16·71)	14·39 (13·19 to 15·74)	13·46 (12·35 to 14·73)
Yemen	69·48 (65·16 to 74·26)	67·04 (62·88 to 71·61)	64·63 (60·6 to 69·06)	62·19 (58·42 to 66·49)	59·93 (56·32 to 64·08)	57·74 (54·22 to 61·64)	55·15 (51·71 to 58·87)	52·45 (49·19 to 55·98)	49·94 (46·83 to 53·35)	47·74 (44·61 to 51·02)
**South Asia**	77·02 (71·76 to 82·89)	75·21 (69·99 to 80·85)	73·57 (68·63 to 79·13)	71·86 (67·01 to 77·3)	70·18 (65·48 to 75·3)	68·41 (63·85 to 73·33)	66·71 (62·4 to 71·4)	65 (60·82 to 69·47)	63·27 (59·22 to 67·52)	61·72 (57·77 to 65·79)
**South Asia**	77·02 (71·76 to 82·89)	75·21 (69·99 to 80·85)	73·57 (68·63 to 79·13)	71·86 (67·01 to 77·3)	70·18 (65·48 to 75·3)	68·41 (63·85 to 73·33)	66·71 (62·4 to 71·4)	65 (60·82 to 69·47)	63·27 (59·22 to 67·52)	61·72 (57·77 to 65·79)
Bangladesh	82·28 (77·39 to 87·68)	80·88 (76·05 to 86·19)	79·92 (75·27 to 85·28)	79·27 (74·76 to 84·59)	78·39 (74·01 to 83·52)	77·61 (73·24 to 82·64)	76·49 (72·19 to 81·27)	75·24 (71 to 79·74)	73·85 (69·68 to 78·17)	72·27 (68·2 to 76·49)
Bhutan	93·75 (90·79 to 96·95)	91·19 (88·35 to 94·24)	88·96 (86·14 to 91·95)	87 (84·17 to 89·91)	85·11 (82·38 to 87·99)	83·56 (80·86 to 86·41)	81·87 (79·18 to 84·61)	80·1 (77·57 to 82·79)	78·28 (75·76 to 80·9)	76·42 (73·94 to 78·97)
India	76·32 (70·65 to 82·65)	74·41 (68·79 to 80·54)	72·66 (67·3 to 78·69)	70·78 (65·5 to 76·59)	68·96 (63·9 to 74·51)	67 (62·14 to 72·46)	65·16 (60·56 to 70·3)	63·32 (58·79 to 68·15)	61·47 (57·05 to 66·03)	59·84 (55·6 to 64·23)
Nepal	81·99 (77·58 to 86·85)	80·64 (76·22 to 85·36)	79·26 (75·02 to 83·94)	78·04 (73·81 to 82·6)	76·65 (72·59 to 81·08)	75·34 (71·36 to 79·72)	74·04 (70·22 to 78·21)	72·66 (68·81 to 76·65)	71·17 (67·41 to 74·97)	69·51 (65·9 to 73·18)
Pakistan	76·07 (73·41 to 78·95)	74·55 (71·99 to 77·3)	73·18 (70·6 to 75·89)	71·84 (69·33 to 74·48)	70·55 (68·07 to 73·16)	69·4 (66·92 to 71·96)	68·18 (65·79 to 70·73)	67·02 (64·67 to 69·49)	65·81 (63·58 to 68·17)	64·66 (62·37 to 66·94)
**Sub-Saharan Africa**	50·68 (47·13 to 54·76)	49·95 (46·47 to 53·89)	49·18 (45·8 to 53·06)	48·37 (45·15 to 52·2)	47·55 (44·48 to 51·26)	46·87 (43·9 to 50·52)	46·2 (43·28 to 49·73)	45·44 (42·67 to 48·77)	44·72 (41·98 to 47·9)	44·05 (41·4 to 47·01)
**Southern Sub-Saharan Africa**	29·75 (26·2 to 33·88)	29·12 (25·62 to 33·1)	28·5 (25·14 to 32·54)	27·92 (24·66 to 31·67)	27·33 (24·22 to 30·98)	26·85 (23·82 to 30·37)	26·44 (23·48 to 29·91)	26·1 (23·24 to 29·38)	25·7 (23 to 28·7)	25·25 (22·66 to 28·17)
Botswana	33·57 (28·1 to 40·13)	32·42 (27·23 to 38·61)	31·23 (26·1 to 37·21)	30·1 (25·2 to 35·82)	28·97 (24·21 to 34·51)	27·87 (23·24 to 33·18)	26·87 (22·47 to 32·04)	26·03 (21·77 to 30·98)	25·27 (21·26 to 29·9)	24·49 (20·45 to 28·93)
Lesotho	30·85 (28·25 to 33·77)	30·83 (28·21 to 33·68)	30·68 (28·15 to 33·61)	30·28 (27·83 to 33·25)	29·93 (27·53 to 32·85)	29·78 (27·44 to 32·68)	29·64 (27·29 to 32·48)	29·39 (27·08 to 32·14)	28·95 (26·65 to 31·65)	28·83 (26·56 to 31·5)
Namibia	25·59 (21·24 to 30·84)	25·33 (21·08 to 30·46)	25·1 (20·88 to 30·38)	24·9 (20·71 to 30·13)	24·71 (20·54 to 29·88)	24·56 (20·38 to 29·71)	24·51 (20·29 to 29·58)	24·46 (20·17 to 29·45)	24·5 (20·08 to 29·59)	24·6 (20·12 to 29·58)
South Africa	29·6 (26·55 to 33·08)	28·9 (25·88 to 32·27)	28·23 (25·31 to 31·51)	27·59 (24·8 to 30·74)	26·96 (24·22 to 29·96)	26·43 (23·79 to 29·43)	25·98 (23·38 to 28·87)	25·59 (23·06 to 28·27)	25·12 (22·65 to 27·75)	24·58 (22·15 to 27·26)
Swaziland	35·49 (30·12 to 41·89)	33·99 (28·8 to 40·05)	32·44 (27·54 to 38·22)	31·06 (26·45 to 36·53)	29·79 (25·32 to 35·1)	28·4 (24·12 to 33·31)	27·09 (22·93 to 31·6)	25·91 (21·96 to 30·37)	24·86 (21·1 to 29·02)	23·9 (20·24 to 27·91)
Zimbabwe	29·62 (24·83 to 35·37)	29·21 (24·49 to 34·86)	28·76 (24·13 to 34·36)	28·39 (23·79 to 33·86)	28 (23·47 to 33·47)	27·69 (23·28 to 33·2)	27·45 (23·07 to 32·85)	27·37 (23·14 to 32·7)	27·21 (22·92 to 32·48)	26·99 (22·76 to 32·13)
**Western Sub-Saharan Africa**	56·28 (50·5 to 63·01)	55·46 (49·82 to 62·04)	54·6 (49·09 to 61·07)	53·62 (48·31 to 60·04)	52·55 (47·62 to 58·82)	51·66 (46·75 to 57·77)	50·77 (45·94 to 56·77)	49·74 (45·08 to 55·28)	48·74 (44·15 to 53·95)	47·87 (43·5 to 52·75)
Benin	47·88 (44·2 to 52)	46·95 (43·33 to 50·95)	45·86 (42·36 to 49·73)	44·6 (41·06 to 48·34)	43·38 (39·86 to 47·02)	42·48 (39 to 46·05)	41·58 (38·15 to 45·08)	40·65 (37·37 to 44·1)	39·75 (36·56 to 43·17)	38·76 (35·69 to 42·07)
Burkina Faso	40·74 (37·2 to 44·74)	40·31 (36·85 to 44·15)	39·66 (36·27 to 43·3)	39·13 (35·7 to 42·75)	38·5 (35·14 to 42·04)	37·87 (34·47 to 41·36)	37·26 (33·91 to 40·69)	36·53 (33·23 to 39·86)	35·56 (32·37 to 38·7)	34·54 (31·46 to 37·65)
Cameroon	42·48 (39·75 to 45·5)	41·51 (38·84 to 44·42)	40·58 (37·99 to 43·55)	39·7 (37·2 to 42·62)	38·79 (36·37 to 41·66)	37·89 (35·48 to 40·69)	37·16 (34·84 to 39·91)	36·34 (34·03 to 39·05)	35·4 (33·09 to 38·03)	34·54 (32·31 to 37·04)
Cape Verde	27·92 (24·14 to 32·76)	28·65 (24·87 to 33·48)	25·74 (22·4 to 30·1)	24·17 (21·08 to 28·22)	22·03 (19·19 to 25·53)	21·16 (18·46 to 24·39)	19·72 (17·31 to 22·6)	18·33 (16·16 to 20·74)	16·98 (15·02 to 19·04)	15·78 (14·03 to 17·58)
Chad	65·93 (63·94 to 68·08)	64 (62·09 to 66·08)	63·21 (61·29 to 65·26)	62·75 (60·9 to 64·82)	62·03 (60·22 to 64·07)	61·06 (59·26 to 63·02)	60·04 (58·23 to 61·95)	59·09 (57·3 to 60·96)	58·18 (56·42 to 60·05)	58·04 (56·2 to 59·95)
Cote d'Ivoire	50·22 (49·05 to 51·47)	48·76 (47·63 to 49·97)	47·3 (46·2 to 48·47)	45·76 (44·72 to 46·9)	44·24 (43·24 to 45·35)	42·91 (41·93 to 43·96)	41·47 (40·5 to 42·49)	39·97 (39·04 to 40·95)	38·56 (37·66 to 39·51)	37·31 (36·39 to 38·24)
The Gambia	60·79 (58·99 to 62·73)	59·43 (57·68 to 61·31)	58·07 (56·35 to 59·91)	56·96 (55·32 to 58·78)	55·73 (54·14 to 57·52)	54·46 (52·88 to 56·17)	53·25 (51·66 to 54·92)	52·02 (50·49 to 53·64)	50·8 (49·3 to 52·41)	49·53 (47·98 to 51·11)
Ghana	37·4 (33·43 to 41·94)	36·55 (32·72 to 40·92)	35·86 (32 to 40·14)	35·1 (31·42 to 39·23)	34·25 (30·7 to 38·22)	33·58 (29·93 to 37·49)	33·07 (29·5 to 36·94)	32·47 (28·9 to 36·29)	31·77 (28·21 to 35·56)	31·23 (27·74 to 34·89)
Guinea	47·96 (46·4 to 49·65)	47·74 (46·19 to 49·4)	47·43 (45·88 to 49·09)	47·1 (45·6 to 48·75)	46·67 (45·2 to 48·32)	46·19 (44·72 to 47·78)	45·55 (44·06 to 47·12)	44·98 (43·52 to 46·53)	44·27 (42·82 to 45·81)	43·59 (42·1 to 45·13)
Guinea-Bissau	65·51 (61·3 to 70·16)	64·47 (60·34 to 68·99)	63·42 (59·32 to 68·04)	62·46 (58·37 to 67·11)	61·52 (57·51 to 65·96)	60·58 (56·63 to 65·04)	59·42 (55·58 to 63·85)	58·68 (54·92 to 63·01)	57·68 (54·05 to 61·83)	56·75 (53·16 to 60·74)
Liberia	67·75 (63·41 to 72·57)	66·31 (62·06 to 70·95)	64·93 (60·73 to 69·66)	63·59 (59·43 to 68·33)	62·18 (58·13 to 66·67)	60·84 (56·87 to 65·32)	59·48 (55·63 to 63·91)	58·24 (54·5 to 62·54)	56·99 (53·4 to 61·09)	55·73 (52·2 to 59·65)
Mali	76·09 (71·2 to 81·5)	74·94 (70·14 to 80·21)	73·84 (69·13 to 79·23)	72·74 (68·16 to 78·1)	71·77 (67·29 to 77·08)	70·54 (66·05 to 75·76)	68·98 (64·68 to 74·1)	67·39 (63·1 to 72·41)	65·96 (61·65 to 70·86)	64·27 (60·12 to 68·93)
Mauritania	38·62 (37·65 to 39·66)	38·09 (37·15 to 39·11)	37·74 (36·79 to 38·75)	37·47 (36·56 to 38·48)	37·13 (36·21 to 38·11)	36·69 (35·79 to 37·65)	36·14 (35·23 to 37·09)	35·58 (34·69 to 36·52)	35·13 (34·25 to 36·07)	34·6 (33·68 to 35·53)
Niger	52·58 (50·89 to 54·41)	52·35 (50·69 to 54·12)	52·3 (50·68 to 54·11)	52·53 (50·91 to 54·42)	52·53 (50·9 to 54·44)	52·67 (51·04 to 54·57)	52·64 (51·01 to 54·53)	52·34 (50·67 to 54·17)	51·82 (50·16 to 53·64)	51·11 (49·47 to 52·88)
Nigeria	62·51 (54·12 to 72·35)	61·74 (53·5 to 71·38)	60·85 (52·81 to 70·54)	59·62 (51·95 to 69·12)	58·3 (50·95 to 67·5)	57·28 (49·94 to 66·44)	56·31 (49 to 65·11)	55·11 (47·86 to 63·33)	54·02 (46·97 to 61·69)	53·18 (46·23 to 60·7)
Sao Tome and Principe	22·05 (20·64 to 23·62)	21·12 (19·77 to 22·6)	20·37 (19·05 to 21·86)	19·67 (18·38 to 21·13)	19·06 (17·82 to 20·43)	18·49 (17·29 to 19·85)	18·01 (16·84 to 19·35)	17·5 (16·37 to 18·79)	17·25 (16·16 to 18·49)	17·31 (16·22 to 18·53)
Senegal	33·61 (26·48 to 42·55)	33·33 (26·19 to 42·14)	32·93 (25·95 to 41·57)	32·55 (25·68 to 41·06)	32·05 (25·33 to 40·27)	31·44 (24·9 to 39·59)	30·71 (24·48 to 38·43)	29·97 (23·84 to 37·27)	29·34 (23·3 to 36·2)	28·69 (22·86 to 35·31)
Sierra Leone	61·87 (57·9 to 66·26)	61·06 (57·14 to 65·34)	60·31 (56·42 to 64·71)	59·61 (55·7 to 64·05)	58·94 (55·1 to 63·19)	58·36 (54·56 to 62·66)	57·82 (54·07 to 62·13)	57·35 (53·67 to 61·59)	56·98 (53·39 to 61·08)	56·67 (53·09 to 60·66)
Togo	51·78 (48·93 to 54·92)	50·57 (47·82 to 53·63)	49·41 (46·72 to 52·39)	48·41 (45·92 to 51·35)	47·17 (44·71 to 50·05)	46·31 (43·85 to 49·17)	45·52 (43·07 to 48·23)	44·55 (42·16 to 47·09)	43·52 (41·2 to 45·87)	42·5 (40·26 to 44·83)
**Eastern Sub-Saharan Africa**	47·39 (45·69 to 49·24)	46·78 (45·12 to 48·58)	46·07 (44·44 to 47·88)	45·45 (43·87 to 47·22)	44·89 (43·34 to 46·63)	44·4 (42·92 to 46·12)	43·91 (42·43 to 45·57)	43·41 (41·94 to 45·03)	42·93 (41·5 to 44·5)	42·45 (41·02 to 43·98)
Burundi	36·1 (35·59 to 36·66)	35·77 (35·26 to 36·31)	35·58 (35·07 to 36·12)	35·41 (34·91 to 35·95)	35·29 (34·8 to 35·83)	35·24 (34·74 to 35·77)	35·01 (34·5 to 35·54)	34·47 (33·97 to 34·98)	33·89 (33·4 to 34·4)	33·25 (32·75 to 33·76)
Comoros	75·72 (71·7 to 80·13)	74·42 (70·48 to 78·72)	73·29 (69·4 to 77·55)	72·19 (68·44 to 76·39)	70·99 (67·33 to 75·16)	69·85 (66·21 to 73·85)	68·8 (65·12 to 72·73)	67·65 (64·06 to 71·5)	66·29 (62·76 to 70·11)	64·66 (61·03 to 68·43)
Djibouti	44·15 (43·32 to 45·03)	43·49 (42·69 to 44·35)	43 (42·19 to 43·85)	42·27 (41·5 to 43·11)	41·85 (41·09 to 42·69)	41·73 (40·97 to 42·55)	40·78 (40·01 to 41·57)	40·38 (39·63 to 41·17)	40·18 (39·43 to 40·97)	40 (39·21 to 40·8)
Eritrea	45·74 (43·31 to 48·41)	44·91 (42·53 to 47·5)	44·06 (41·72 to 46·62)	43·19 (40·95 to 45·7)	42·32 (40·15 to 44·81)	41·57 (39·41 to 43·95)	40·79 (38·61 to 43·12)	40·13 (38 to 42·41)	39·57 (37·46 to 41·85)	39 (36·82 to 41·28)
Ethiopia	43·95 (42·86 to 45·11)	43·77 (42·7 to 44·92)	43·4 (42·33 to 44·54)	43·05 (42·01 to 44·19)	42·73 (41·71 to 43·87)	42·34 (41·32 to 43·44)	42·05 (41·01 to 43·14)	41·98 (40·94 to 43·06)	41·94 (40·91 to 43·04)	41·8 (40·72 to 42·91)

Appendix Table 17A. Stillbirth rates (per 1,000 live births) by location, 1970-1979

	1970	1971	1972	1973	1974	1975	1976	1977	1978	1979
Kenya	44·14<br>(41·46 to 47·1)	43·47<br>(40·84 to 46·36)	42·68<br>(40·13 to 45·58)	41·87<br>(39·43 to 44·71)	40·96<br>(38·63 to 43·71)	40·2<br>(37·88 to 42·94)	39·5<br>(37·18 to 42·13)	38·66<br>(36·35 to 41·12)	37·85<br>(35·61 to 40·14)	37·15<br>(34·95 to 39·4)
Madagascar	38·51<br>(36·47 to 40·76)	37·75<br>(35·75 to 39·93)	36·78<br>(34·82 to 38·91)	36·37<br>(34·48 to 38·49)	36·43<br>(34·56 to 38·57)	36·93<br>(35·01 to 39·04)	37·43<br>(35·43 to 39·57)	37·42<br>(35·43 to 39·55)	37·1<br>(35·13 to 39·24)	36·77<br>(34·71 to 38·92)
Malawi	60·11<br>(58·76 to 61·56)	58·79<br>(57·48 to 60·19)	57·32<br>(56·04 to 58·7)	55·97<br>(54·75 to 57·32)	54·56<br>(53·38 to 55·88)	53·1<br>(51·93 to 54·36)	51·69<br>(50·53 to 52·92)	50·28<br>(49·16 to 51·47)	48·69<br>(47·59 to 49·85)	47·07<br>(45·95 to 48·2)
Mozambique	61·69<br>(58·41 to 65·28)	60·8<br>(57·62 to 64·23)	59·86<br>(56·78 to 63·3)	59·09<br>(55·93 to 62·43)	58·4<br>(55·23 to 61·72)	57·95<br>(54·75 to 61·24)	57·54<br>(54·36 to 60·81)	56·92<br>(53·82 to 60·09)	56·5<br>(53·44 to 59·71)	56·19<br>(53·02 to 59·37)
Rwanda	48·46<br>(44·4 to 53·04)	48·29<br>(44·25 to 52·87)	48·04<br>(44·1 to 52·48)	47·7<br>(43·73 to 52·15)	47·51<br>(43·56 to 51·83)	47·28<br>(43·23 to 51·59)	46·6<br>(42·7 to 50·75)	45·7<br>(41·82 to 49·76)	44·51<br>(40·74 to 48·48)	42·67<br>(39·02 to 46·51)
Somalia	46·36<br>(43·9 to 49·06)	46·26<br>(43·84 to 48·87)	45·84<br>(43·48 to 48·48)	45·65<br>(43·21 to 48·23)	45·79<br>(43·3 to 48·39)	45·42<br>(42·91 to 48)	45·27<br>(42·77 to 47·85)	44·84<br>(42·4 to 47·35)	44·46<br>(42·05 to 46·98)	44·4<br>(41·89 to 46·91)
South Sudan	93·01<br>(91·03 to 95·13)	92·18<br>(90·23 to 94·27)	91·45<br>(89·5 to 93·52)	90·68<br>(88·79 to 92·75)	89·79<br>(87·94 to 91·86)	89·11<br>(87·25 to 91·11)	88·41<br>(86·52 to 90·4)	87·66<br>(85·79 to 89·61)	86·98<br>(85·13 to 88·95)	86·31<br>(84·37 to 88·28)
Tanzania	42·26<br>(41·11 to 43·5)	41·5<br>(40·37 to 42·71)	40·74<br>(39·63 to 41·94)	40·04<br>(38·97 to 41·21)	39·34<br>(38·3 to 40·5)	38·62<br>(37·59 to 39·73)	37·83<br>(36·79 to 38·91)	37·14<br>(36·13 to 38·21)	36·54<br>(35·54 to 37·61)	35·96<br>(34·93 to 37·02)
Uganda	40·45<br>(40·03 to 40·9)	40·01<br>(39·59 to 40·45)	39·48<br>(39·06 to 39·92)	38·99<br>(38·59 to 39·43)	38·64<br>(38·25 to 39·07)	38·33<br>(37·93 to 38·75)	37·98<br>(37·58 to 38·4)	37·61<br>(37·21 to 38·02)	37·33<br>(36·94 to 37·75)	37·25<br>(36·84 to 37·67)
Zambia	38·55<br>(36·5 to 40·8)	37·67<br>(35·7 to 39·8)	36·66<br>(34·77 to 38·77)	35·76<br>(33·85 to 37·78)	35·01<br>(33·11 to 37·01)	34·43<br>(32·54 to 36·39)	33·82<br>(31·95 to 35·74)	33·59<br>(31·76 to 35·47)	33·45<br>(31·64 to 35·35)	33·13<br>(31·26 to 35)
**Central Sub-Saharan Africa**	54·34<br>(52·71 to 56·1)	53·62<br>(52·01 to 55·31)	53·05<br>(51·51 to 54·74)	52·27<br>(50·77 to 53·96)	51·39<br>(49·92 to 53·07)	50·88<br>(49·48 to 52·52)	50·26<br>(48·88 to 51·84)	49·42<br>(48·08 to 50·92)	48·71<br>(47·37 to 50·19)	47·95<br>(46·65 to 49·39)
Angola	57·44<br>(54·95 to 60·15)	56·87<br>(54·43 to 59·48)	56·41<br>(54·06 to 59·07)	55·83<br>(53·52 to 58·56)	55·3<br>(52·98 to 58·03)	55·2<br>(52·91 to 57·92)	55·34<br>(53·02 to 58·04)	55·01<br>(52·65 to 57·63)	54·57<br>(52·22 to 57·18)	54·12<br>(51·79 to 56·68)
Central African Republic	61·24<br>(57·57 to 65·3)	61·2<br>(57·5 to 65·14)	61·51<br>(57·77 to 65·54)	61·64<br>(57·91 to 65·68)	61·78<br>(58·03 to 65·95)	61·76<br>(58·1 to 65·85)	61·37<br>(57·71 to 65·45)	60·58<br>(57·03 to 64·62)	59·68<br>(56·21 to 63·57)	58·87<br>(55·46 to 62·63)
Congo	44·29<br>(41·63 to 47·22)	43·35<br>(40·75 to 46·18)	42·4<br>(39·84 to 45·28)	41·36<br>(38·83 to 44·21)	40·17<br>(37·72 to 42·87)	39·02<br>(36·64 to 41·7)	37·88<br>(35·58 to 40·5)	36·89<br>(34·68 to 39·43)	35·96<br>(33·85 to 38·38)	34·94<br>(32·87 to 37·23)
Democratic Republic of the Congo	53·25<br>(52·17 to 54·4)	52·49<br>(51·43 to 53·62)	51·91<br>(50·85 to 53·03)	51·05<br>(50·04 to 52·16)	50·05<br>(49·07 to 51·15)	49·46<br>(48·48 to 50·52)	48·67<br>(47·67 to 49·71)	47·68<br>(46·71 to 48·69)	46·91<br>(45·95 to 47·92)	46·06<br>(45·07 to 47·06)
Equatorial Guinea	72·64<br>(68·28 to 77·45)	71·52<br>(67·19 to 76·13)	70·4<br>(66·13 to 75·01)	69<br>(64·82 to 73·52)	67·38<br>(63·29 to 71·93)	65·28<br>(61·41 to 69·61)	63·09<br>(59·33 to 67·28)	60·85<br>(57·29 to 64·9)	58·96<br>(55·53 to 62·81)	57·46<br>(54·14 to 61·14)
Gabon	53·78<br>(50·56 to 57·34)	52·2<br>(49·07 to 55·6)	50·65<br>(47·59 to 54·09)	49<br>(45·99 to 52·36)	46·77<br>(43·93 to 49·92)	44·73<br>(42 to 47·79)	42·45<br>(39·88 to 45·39)	40·86<br>(38·41 to 43·67)	39·67<br>(37·34 to 42·34)	38·43<br>(36·16 to 40·95)

Appendix Table 17B. Stillbirth rates (per 1,000 live births) by location, 1980-1995

Location	1980	1981	1982	1983	1984	1985	1986	1987	1988	1989	1990	1991	1992	1993	1994	1995
**Global**	33·2 (30·88 to 35·72)	32·28 (30·07 to 34·68)	31·39 (29·28 to 33·7)	30·59 (28·58 to 32·82)	29·82 (27·88 to 32·03)	29·04 (27·15 to 31·18)	28·25 (26·44 to 30·36)	27·52 (25·77 to 29·57)	26·82 (25·11 to 28·79)	26·17 (24·53 to 28·07)	25·6 (24·08 to 27·44)	25·14 (23·62 to 26·88)	24·73 (23·25 to 26·44)	24·31 (22·86 to 25·93)	23·91 (22·53 to 25·45)	23·5 (22·14 to 25)
**High SDI**	6·77 (6·21 to 7·44)	6·49 (5·97 to 7·11)	6·21 (5·74 to 6·8)	6·05 (5·61 to 6·57)	5·82 (5·43 to 6·27)	5·63 (5·29 to 6·02)	5·39 (5·09 to 5·72)	5·22 (4·97 to 5·49)	5·05 (4·83 to 5·28)	4·84 (4·64 to 5·03)	4·68 (4·5 to 4·85)	4·55 (4·4 to 4·71)	4·41 (4·26 to 4·56)	4·32 (4·18 to 4·46)	4·24 (4·11 to 4·38)	4·09 (3·97 to 4·22)
**High-middle SDI**	18·32 (16·53 to 20·31)	17·66 (15·95 to 19·62)	17 (15·35 to 18·92)	16·36 (14·78 to 18·19)	15·75 (14·24 to 17·48)	15·18 (13·75 to 16·86)	14·64 (13·3 to 16·25)	14·22 (12·95 to 15·77)	13·8 (12·59 to 15·28)	13·42 (12·28 to 14·81)	13·08 (11·99 to 14·37)	12·74 (11·7 to 13·93)	12·44 (11·44 to 13·54)	12·13 (11·18 to 13·19)	11·91 (11·03 to 12·89)	11·67 (10·84 to 12·58)
**Middle SDI**	29·64 (27·36 to 32·07)	28·54 (26·42 to 30·87)	27·48 (25·49 to 29·66)	26·45 (24·54 to 28·54)	25·49 (23·68 to 27·52)	24·55 (22·85 to 26·48)	23·62 (21·98 to 25·53)	22·76 (21·19 to 24·53)	21·95 (20·42 to 23·66)	21·22 (19·74 to 22·87)	20·59 (19·18 to 22·12)	20·08 (18·77 to 21·55)	19·54 (18·29 to 21)	18·98 (17·77 to 20·37)	18·4 (17·26 to 19·71)	17·85 (16·74 to 19·09)
**Low-middle SDI**	58·76 (54·9 to 62·78)	57·19 (53·51 to 61·11)	55·63 (52·06 to 59·45)	54·13 (50·71 to 57·88)	52·73 (49·39 to 56·47)	51·28 (47·96 to 55·06)	49·92 (46·67 to 53·56)	48·62 (45·42 to 52·3)	47·21 (44·13 to 50·76)	45·88 (42·93 to 49·33)	44·64 (41·76 to 47·93)	43·49 (40·72 to 46·59)	42·41 (39·73 to 45·44)	41·39 (38·77 to 44·36)	40·38 (37·8 to 43·29)	39·43 (36·93 to 42·22)
**Low SDI**	44·94 (43·1 to 46·89)	44·3 (42·52 to 46·14)	43·64 (41·9 to 45·43)	42·92 (41·19 to 44·63)	42·15 (40·5 to 43·77)	41·48 (39·92 to 43·03)	40·78 (39·29 to 42·31)	40·1 (38·67 to 41·62)	39·45 (38·06 to 40·95)	38·8 (37·43 to 40·28)	38·21 (36·85 to 39·65)	37·68 (36·34 to 39·13)	37·17 (35·84 to 38·63)	36·64 (35·35 to 38·09)	36·15 (34·85 to 37·59)	35·58 (34·31 to 37·04)
**High-income**	6·19 (5·5 to 7·06)	5·9 (5·25 to 6·74)	5·64 (5·05 to 6·39)	5·4 (4·86 to 6·1)	5·16 (4·67 to 5·77)	4·99 (4·55 to 5·52)	4·83 (4·44 to 5·29)	4·67 (4·33 to 5·04)	4·53 (4·25 to 4·85)	4·38 (4·14 to 4·65)	4·27 (4·06 to 4·49)	4·16 (3·99 to 4·34)	4·03 (3·88 to 4·19)	3·95 (3·82 to 4·09)	3·86 (3·74 to 3·97)	3·73 (3·62 to 3·84)
**High-income North America**	4·31 (4·04 to 4·57)	4·1 (3·89 to 4·31)	3·96 (3·77 to 4·15)	3·81 (3·65 to 3·98)	3·67 (3·51 to 3·84)	3·63 (3·47 to 3·78)	3·55 (3·39 to 3·69)	3·49 (3·35 to 3·63)	3·48 (3·33 to 3·62)	3·48 (3·34 to 3·62)	3·4 (3·27 to 3·55)	3·38 (3·24 to 3·52)	3·32 (3·18 to 3·47)	3·37 (3·22 to 3·53)	3·38 (3·23 to 3·53)	3·33 (3·18 to 3·48)
Canada	3·46 (3·4 to 3·52)	3·27 (3·21 to 3·33)	3·12 (3·07 to 3·17)	2·98 (2·93 to 3·03)	2·89 (2·84 to 2·94)	2·81 (2·77 to 2·86)	2·78 (2·73 to 2·83)	2·7 (2·65 to 2·75)	2·65 (2·61 to 2·7)	2·63 (2·59 to 2·67)	2·58 (2·54 to 2·62)	2·49 (2·45 to 2·53)	2·42 (2·38 to 2·46)	2·41 (2·38 to 2·45)	2·43 (2·39 to 2·46)	2·41 (2·37 to 2·44)
Greenland	13·1 (12·21 to 14)	13·09 (12·22 to 14·01)	13·19 (12·25 to 14·1)	12·9 (11·98 to 13·76)	12·58 (11·7 to 13·43)	12·14 (11·26 to 12·99)	12·11 (11·28 to 12·98)	12·23 (11·35 to 13·1)	12·17 (11·29 to 13·03)	12·04 (11·2 to 12·93)	12·13 (11·29 to 13·03)	11·57 (10·77 to 12·4)	11·56 (10·8 to 12·39)	11·05 (10·36 to 11·83)	10·38 (9·7 to 11·1)	9·69 (9·08 to 10·38)
United States	4·4 (4·1 to 4·67)	4·18 (3·95 to 4·41)	4·05 (3·84 to 4·25)	3·9 (3·71 to 4·08)	3·75 (3·57 to 3·93)	3·71 (3·54 to 3·87)	3·62 (3·45 to 3·78)	3·56 (3·4 to 3·72)	3·55 (3·39 to 3·71)	3·56 (3·4 to 3·71)	3·48 (3·33 to 3·64)	3·46 (3·31 to 3·62)	3·41 (3·25 to 3·57)	3·46 (3·3 to 3·63)	3·47 (3·31 to 3·64)	3·42 (3·25 to 3·58)
**Australasia**	5·1 (4·4 to 5·88)	4·82 (4·17 to 5·53)	4·69 (4·09 to 5·4)	4·5 (3·92 to 5·22)	4·35 (3·81 to 5·02)	4·28 (3·75 to 4·89)	4·03 (3·56 to 4·57)	3·9 (3·45 to 4·39)	3·77 (3·35 to 4·23)	3·73 (3·31 to 4·16)	3·62 (3·25 to 4·01)	3·43 (3·1 to 3·76)	3·39 (3·08 to 3·73)	3·27 (2·99 to 3·56)	3·2 (2·95 to 3·45)	3·12 (2·88 to 3·37)
Australia	4·99 (4·24 to 5·82)	4·72 (4·04 to 5·49)	4·62 (3·98 to 5·38)	4·43 (3·81 to 5·21)	4·29 (3·72 to 5·03)	4·24 (3·66 to 4·92)	3·95 (3·44 to 4·56)	3·82 (3·34 to 4·38)	3·69 (3·23 to 4·2)	3·66 (3·19 to 4·13)	3·55 (3·14 to 3·98)	3·33 (2·95 to 3·7)	3·31 (2·95 to 3·7)	3·19 (2·86 to 3·52)	3·11 (2·83 to 3·42)	3·04 (2·76 to 3·34)
New Zealand	5·56 (5·08 to 6·11)	5·27 (4·83 to 5·8)	5·02 (4·61 to 5·51)	4·83 (4·44 to 5·28)	4·63 (4·27 to 5·03)	4·46 (4·14 to 4·83)	4·36 (4·06 to 4·68)	4·22 (3·95 to 4·5)	4·14 (3·89 to 4·39)	4·05 (3·83 to 4·27)	3·94 (3·74 to 4·15)	3·85 (3·67 to 4·03)	3·75 (3·59 to 3·92)	3·67 (3·51 to 3·82)	3·56 (3·42 to 3·71)	3·47 (3·34 to 3·61)
**High-income Asia Pacific**	8·6 (7 to 10·72)	8·24 (6·73 to 10·29)	7·88 (6·44 to 9·73)	7·58 (6·22 to 9·3)	7·31 (6·03 to 8·81)	6·92 (5·87 to 8·19)	6·53 (5·63 to 7·55)	6·14 (5·38 to 6·98)	5·73 (5·14 to 6·37)	5·3 (4·86 to 5·78)	4·98 (4·66 to 5·35)	4·61 (4·37 to 4·88)	4·37 (4·17 to 4·59)	4·1 (3·91 to 4·3)	3·88 (3·7 to 4·09)	3·66 (3·49 to 3·85)
Brunei	11·6 (9·36 to 14·27)	10·68 (8·67 to 13·09)	9·57 (7·81 to 11·74)	8·84 (7·26 to 10·77)	8·36 (6·92 to 10·17)	7·92 (6·6 to 9·59)	7·44 (6·23 to 8·94)	6·92 (5·85 to 8·22)	6·38 (5·44 to 7·47)	6·23 (5·37 to 7·2)	6 (5·23 to 6·82)	5·97 (5·28 to 6·7)	5·74 (5·16 to 6·34)	5·55 (5·06 to 6·07)	5·37 (4·95 to 5·8)	5·18 (4·82 to 5·56)
Japan	8·41 (6·12 to 11·49)	8·12 (5·99 to 11·05)	7·77 (5·8 to 10·34)	7·51 (5·73 to 9·82)	7·39 (5·76 to 9·38)	7·01 (5·63 to 8·66)	6·68 (5·49 to 8·01)	6·3 (5·34 to 7·37)	5·91 (5·16 to 6·74)	5·45 (4·88 to 6·06)	5·14 (4·73 to 5·64)	4·71 (4·4 to 5·07)	4·47 (4·19 to 4·75)	4·12 (3·88 to 4·36)	3·85 (3·63 to 4·08)	3·58 (3·39 to 3·79)
Singapore	8·17 (7·8 to 8·57)	7·71 (7·36 to 8·07)	7·3 (6·98 to 7·64)	6·81 (6·5 to 7·13)	6·38 (6·1 to 6·67)	6 (5·75 to 6·28)	5·64 (5·4 to 5·89)	5·29 (5·07 to 5·51)	5·08 (4·88 to 5·3)	4·9 (4·7 to 5·1)	4·82 (4·63 to 5·01)	4·59 (4·41 to 4·77)	4·34 (4·18 to 4·51)	4·06 (3·91 to 4·22)	3·8 (3·65 to 3·94)	3·56 (3·43 to 3·69)
South Korea	8·96 (8·32 to 9·63)	8·46 (7·86 to 9·09)	8·11 (7·53 to 8·74)	7·76 (7·19 to 8·37)	7·16 (6·63 to 7·72)	6·76 (6·26 to 7·3)	6·27 (5·82 to 6·77)	5·86 (5·44 to 6·35)	5·41 (5·02 to 5·86)	5·03 (4·65 to 5·45)	4·7 (4·36 to 5·08)	4·42 (4·11 to 4·78)	4·2 (3·91 to 4·54)	4·05 (3·77 to 4·36)	3·93 (3·67 to 4·23)	3·8 (3·54 to 4·09)
**Western Europe**	5·78 (5·46 to 6·14)	5·51 (5·21 to 5·83)	5·25 (4·98 to 5·56)	5·03 (4·78 to 5·33)	4·78 (4·54 to 5·05)	4·62 (4·4 to 4·87)	4·52 (4·31 to 4·76)	4·38 (4·18 to 4·61)	4·28 (4·09 to 4·49)	4·12 (3·94 to 4·3)	4·04 (3·87 to 4·22)	3·96 (3·8 to 4·12)	3·82 (3·68 to 3·98)	3·72 (3·58 to 3·86)	3·61 (3·49 to 3·73)	3·44 (3·34 to 3·55)
Andorra	5·21 (5·17 to 5·26)	5·06 (5·02 to 5·1)	5·1 (5·06 to 5·15)	5·06 (5·01 to 5·1)	4·84 (4·8 to 4·88)	4·67 (4·63 to 4·71)	4·54 (4·5 to 4·58)	4·44 (4·4 to 4·48)	4·36 (4·32 to 4·39)	4·28 (4·25 to 4·32)	4·21 (4·17 to 4·24)	4·16 (4·13 to 4·19)	4·25 (4·22 to 4·29)	3·94 (3·91 to 3·97)	3·59 (3·56 to 3·62)	3·43 (3·4 to 3·46)
Austria	3·91 (3·61 to 4·24)	3·6 (3·33 to 3·9)	3·48 (3·23 to 3·76)	3·23 (3 to 3·48)	3·16 (2·95 to 3·41)	3·06 (2·87 to 3·29)	2·86 (2·68 to 3·07)	2·7 (2·53 to 2·88)	2·44 (2·3 to 2·59)	2·43 (2·3 to 2·57)	2·37 (2·25 to 2·49)	2·3 (2·2 to 2·42)	2·3 (2·2 to 2·4)	2·16 (2·08 to 2·25)	2·14 (2·07 to 2·22)	2·04 (1·98 to 2·11)
Belgium	8·2 (5·79 to 11·19)	7·81 (5·59 to 10·53)	7·35 (5·31 to 9·8)	7·04 (5·18 to 9·26)	6·73 (5·07 to 8·72)	6·41 (4·93 to 8·09)	6·17 (4·85 to 7·6)	5·98 (4·82 to 7·26)	5·76 (4·72 to 6·85)	5·51 (4·61 to 6·5)	5·29 (4·52 to 6·08)	5·1 (4·42 to 5·78)	4·83 (4·25 to 5·42)	4·61 (4·11 to 5·11)	4·34 (3·89 to 4·77)	4·03 (3·63 to 4·43)
Cyprus	7·91 (7·55 to 8·28)	7·59 (7·22 to 7·94)	7·31 (6·96 to 7·65)	6·85 (6·52 to 7·16)	6·59 (6·27 to 6·9)	6·18 (5·88 to 6·47)	5·93 (5·64 to 6·2)	5·7 (5·42 to 5·96)	5·55 (5·28 to 5·8)	5·49 (5·24 to 5·74)	5·23 (4·99 to 5·46)	5·08 (4·85 to 5·31)	4·91 (4·69 to 5·13)	4·74 (4·54 to 4·96)	4·57 (4·38 to 4·79)	4·45 (4·27 to 4·66)
Denmark	3·27 (2·92 to 3·65)	3·15 (2·82 to 3·46)	3·08 (2·8 to 3·36)	3·03 (2·77 to 3·28)	3 (2·77 to 3·24)	3·01 (2·8 to 3·23)	3·04 (2·84 to 3·25)	3·1 (2·89 to 3·31)	3·06 (2·87 to 3·27)	3·07 (2·87 to 3·27)	3·02 (2·82 to 3·23)	2·97 (2·78 to 3·18)	2·87 (2·69 to 3·07)	2·73 (2·55 to 2·91)	2·69 (2·52 to 2·87)	2·62 (2·45 to 2·8)
Finland	2·62 (2·52 to 2·72)	2·42 (2·33 to 2·51)	2·28 (2·2 to 2·37)	2·29 (2·21 to 2·37)	2·32 (2·23 to 2·4)	2·26 (2·18 to 2·35)	2·17 (2·09 to 2·25)	2·19 (2·12 to 2·27)	2·2 (2·12 to 2·28)	2·2 (2·12 to 2·28)	2·13 (2·06 to 2·21)	2·18 (2·1 to 2·25)	2·12 (2·05 to 2·19)	1·98 (1·91 to 2·04)	1·95 (1·89 to 2·02)	1·85 (1·79 to 1·91)
France	7·28 (7·11 to 7·45)	7·01 (6·85 to 7·18)	6·78 (6·62 to 6·95)	6·5 (6·35 to 6·67)	6·12 (5·97 to 6·27)	6 (5·85 to 6·15)	5·95 (5·81 to 6·09)	5·73 (5·6 to 5·87)	5·8 (5·67 to 5·94)	5·63 (5·5 to 5·76)	5·56 (5·43 to 5·69)	5·61 (5·48 to 5·74)	5·51 (5·38 to 5·63)	5·43 (5·31 to 5·56)	5·29 (5·17 to 5·41)	4·83 (4·72 to 4·93)
Germany	3·56 (3·53 to 3·59)	3·36 (3·33 to 3·39)	3·21 (3·18 to 3·24)	3·01 (2·98 to 3·03)	2·84 (2·82 to 2·87)	2·73 (2·7 to 2·75)	2·69 (2·67 to 2·72)	2·61 (2·59 to 2·64)	2·53 (2·51 to 2·56)	2·43 (2·41 to 2·46)	2·48 (2·45 to 2·5)	2·44 (2·41 to 2·46)	2·31 (2·29 to 2·33)	2·29 (2·27 to 2·31)	2·26 (2·24 to 2·28)	2·19 (2·17 to 2·21)
Greece	6·68 (6·54 to 6·83)	6·22 (6·09 to 6·36)	5·86 (5·74 to 5·99)	5·65 (5·53 to 5·78)	5·47 (5·36 to 5·59)	5·29 (5·18 to 5·4)	4·88 (4·78 to 4·98)	4·51 (4·42 to 4·6)	4·22 (4·13 to 4·31)	3·91 (3·83 to 3·99)	3·75 (3·68 to 3·83)	3·56 (3·49 to 3·63)	3·42 (3·35 to 3·49)	3·33 (3·27 to 3·4)	3·23 (3·16 to 3·29)	3·13 (3·07 to 3·2)
Iceland	2·94 (2·62 to 3·3)	2·8 (2·49 to 3·15)	2·74 (2·44 to 3·08)	2·68 (2·38 to 3·01)	2·61 (2·33 to 2·92)	2·59 (2·31 to 2·9)	2·52 (2·25 to 2·82)	2·51 (2·25 to 2·81)	2·48 (2·22 to 2·76)	2·41 (2·17 to 2·69)	2·38 (2·14 to 2·66)	2·34 (2·1 to 2·61)	2·3 (2·06 to 2·55)	2·24 (2·01 to 2·48)	2·16 (1·93 to 2·39)	2·15 (1·92 to 2·37)
Ireland	5·26 (4·97 to 5·59)	4·91 (4·64 to 5·21)	4·8 (4·54 to 5·08)	4·61 (4·36 to 4·87)	4·36 (4·13 to 4·61)	4·19 (3·97 to 4·42)	4·09 (3·88 to 4·31)	3·97 (3·77 to 4·18)	4·02 (3·83 to 4·25)	3·96 (3·76 to 4·18)	3·89 (3·7 to 4·09)	3·78 (3·59 to 3·98)	3·61 (3·43 to 3·8)	3·49 (3·32 to 3·68)	3·4 (3·23 to 3·58)	3·43 (3·25 to 3·6)
Israel	4·39 (3·68 to 5·24)	4·29 (3·62 to 5·14)	4·14 (3·5 to 4·96)	4·17 (3·53 to 4·97)	3·93 (3·32 to 4·68)	3·65 (3·06 to 4·34)	3·52 (2·98 to 4·12)	3·44 (2·93 to 3·99)	3·28 (2·83 to 3·76)	3·23 (2·83 to 3·67)	3·24 (2·85 to 3·64)	3·18 (2·83 to 3·57)	3·17 (2·85 to 3·51)	3 (2·74 to 3·3)	2·84 (2·61 to 3·1)	2·76 (2·54 to 2·99)
Italy	5·84 (5·23 to 6·51)	5·68 (5·08 to 6·36)	5·36 (4·81 to 6·02)	5·13 (4·6 to 5·75)	4·77 (4·28 to 5·36)	4·49 (4·02 to 5·03)	4·25 (3·81 to 4·76)	4·14 (3·72 to 4·64)	4·04 (3·63 to 4·52)	3·75 (3·38 to 4·17)	3·6 (3·26 to 4)	3·56 (3·23 to 3·92)	3·54 (3·21 to 3·87)	3·37 (3·07 to 3·7)	3·18 (2·9 to 3·47)	3·02 (2·77 to 3·28)
Luxembourg	3·06 (2·37 to 3·83)	3·17 (2·46 to 3·96)	3·11 (2·45 to 3·88)	2·91 (2·31 to 3·6)	2·9 (2·33 to 3·56)	2·74 (2·22 to 3·33)	2·58 (2·11 to 3·1)	2·69 (2·22 to 3·21)	2·53 (2·1 to 2·98)	2·55 (2·13 to 3·02)	2·47 (2·08 to 2·88)	2·5 (2·13 to 2·9)	2·44 (2·09 to 2·82)	2·37 (2·04 to 2·73)	2·33 (2·03 to 2·65)	2·15 (1·88 to 2·43)
Malta	5·46 (5·41 to 5·52)	4·88 (4·83 to 4·93)	5·22 (5·17 to 5·28)	5·07 (5·02 to 5·13)	4·7 (4·65 to 4·75)	4·73 (4·68 to 4·78)	4·21 (4·17 to 4·26)	3·82 (3·78 to 3·86)	3·87 (3·83 to 3·91)	3·8 (3·76 to 3·84)	3·72 (3·68 to 3·76)	3·64 (3·61 to 3·68)	3·67 (3·64 to 3·71)	3·51 (3·47 to 3·54)	3·52 (3·48 to 3·55)	3·42 (3·38 to 3·45)
Netherlands	6·51 (4·25 to 9·45)	6·37 (4·2 to 9·25)	6·25 (4·21 to 9·2)	6·18 (4·19 to 9·1)	6·13 (4·14 to 8·92)	5·97 (3·99 to 8·66)	5·89 (3·95 to 8·48)	5·79 (4 to 8·17)	5·52 (3·88 to 7·58)	5·41 (3·87 to 7·24)	5·5 (4·02 to 7·26)	5·34 (4·01 to 6·93)	5·18 (3·97 to 6·58)	5·1 (4·07 to 6·34)	4·93 (4·04 to 5·97)	4·81 (4·14 to 5·55)
Norway	3·43 (3·26 to 3·61)	3·28 (3·13 to 3·43)	3·27 (3·12 to 3·42)	3·17 (3·04 to 3·31)	3·19 (3·06 to 3·32)	3·16 (3·03 to 3·28)	3·05 (2·93 to 3·16)	3·04 (2·93 to 3·15)	3·01 (2·89 to 3·11)	2·92 (2·81 to 3·02)	2·79 (2·68 to 2·89)	2·71 (2·61 to 2·81)	2·64 (2·54 to 2·74)	2·53 (2·44 to 2·62)	2·45 (2·36 to 2·53)	2·28 (2·2 to 2·36)
Portugal	8·16 (7·97 to 8·36)	7·67 (7·49 to 7·85)	7·26 (7·09 to 7·43)	6·93 (6·76 to 7·09)	6·55 (6·4 to 6·71)	6·32 (6·18 to 6·46)	6 (5·87 to 6·14)	5·63 (5·51 to 5·75)	5·29 (5·17 to 5·41)	4·92 (4·8 to 5·03)	4·53 (4·42 to 4·63)	4·27 (4·18 to 4·37)	3·94 (3·85 to 4·03)	3·71 (3·63 to 3·8)	3·47 (3·4 to 3·55)	3·29 (3·22 to 3·37)
Spain	4·52 (4·09 to 4·97)	4·42 (4 to 4·86)	4·03 (3·67 to 4·44)	3·87 (3·53 to 4·25)	3·56 (3·24 to 3·89)	3·26 (2·98 to 3·55)	3·22 (2·96 to 3·5)	3·09 (2·85 to 3·34)	2·9 (2·68 to 3·11)	2·8 (2·61 to 3)	2·75 (2·56 to 2·92)	2·59 (2·43 to 2·75)	2·54 (2·4 to 2·7)	2·47 (2·33 to 2·61)	2·32 (2·2 to 2·46)	2·16 (2·05 to 2·29)
Sweden	2·44 (2·21 to 2·7)	2·31 (2·11 to 2·52)	2·25 (2·07 to 2·43)	2·23 (2·06 to 2·4)	2·13 (1·98 to 2·29)	2·2 (2·03 to 2·39)	2·14 (1·97 to 2·32)	2·21 (2·05 to 2·38)	2·21 (2·05 to 2·37)	2·19 (2·04 to 2·35)	2·2 (2·05 to 2·36)	2·27 (2·11 to 2·43)	2·22 (2·07 to 2·38)	2·19 (2·04 to 2·35)	2·16 (2·01 to 2·32)	2·11 (1·97 to 2·27)
Switzerland	2·86 (2·68 to 3·05)	2·74 (2·58 to 2·91)	2·66 (2·51 to 2·81)	2·6 (2·47 to 2·75)	2·54 (2·4 to 2·68)	2·46 (2·33 to 2·6)	2·43 (2·3 to 2·56)	2·39 (2·26 to 2·52)	2·39 (2·26 to 2·53)	2·4 (2·27 to 2·54)	2·38 (2·25 to 2·52)	2·32 (2·19 to 2·45)	2·28 (2·17 to 2·41)	2·21 (2·09 to 2·33)	2·13 (2·02 to 2·25)	2·07 (1·96 to 2·17)
**United Kingdom**	8·14 (7·96 to 8·35)	7·57 (7·39 to 7·76)	7·25 (7·08 to 7·42)	6·88 (6·71 to 7·04)	6·59 (6·43 to 6·74)	6·48 (6·33 to 6·63)	6·4 (6·25 to 6·55)	6·27 (6·12 to 6·42)	6·17 (6·03 to 6·32)	5·95 (5·81 to 6·09)	5·73 (5·6 to 5·87)	5·51 (5·38 to 5·64)	5·2 (5·08 to 5·32)	5·04 (4·93 to 5·16)	4·94 (4·83 to 5·06)	4·87 (4·75 to 4·99)
England	7·33 (7·17 to 7·51)	6·73 (6·58 to 6·89)	6·48 (6·33 to 6·63)	6·16 (6·03 to 6·31)	5·89 (5·75 to 6·03)	5·84 (5·71 to 5·98)	5·83 (5·69 to 5·96)	5·74 (5·61 to 5·87)	5·7 (5·57 to 5·84)	5·45 (5·33 to 5·58)	5·25 (5·13 to 5·38)	5·03 (4·91 to 5·15)	4·7 (4·59 to 4·82)	4·55 (4·44 to 4·66)	4·48 (4·37 to 4·58)	4·38 (4·28 to 4·49)
Northern Ireland	12·08 (11·83 to 12·36)	12·27 (12·02 to 12·55)	11·95 (11·7 to 12·22)	11·15 (10·91 to 11·41)	10·32 (10·09 to 10·57)	9·63 (9·41 to 9·87)	9·43 (9·21 to 9·66)	9·03 (8·81 to 9·26)	9·05 (8·83 to 9·28)	8·41 (8·21 to 8·63)	8·31 (8·11 to 8·51)	8·17 (7·99 to 8·37)	7·65 (7·49 to 7·83)	7·97 (7·8 to 8·16)	7·68 (7·5 to 7·86)	7·85 (7·66 to 8·05)
Scotland	11·7 (11·34 to 12·1)	11·28 (10·93 to 11·66)	10·85 (10·5 to 11·22)	10·24 (9·92 to 10·58)	10·31 (10 to 10·64)	9·78 (9·48 to 10·07)	9·34 (9·06 to 9·61)	9·08 (8·82 to 9·33)	8·8 (8·55 to 9·02)	9·02 (8·79 to 9·24)	8·65 (8·45 to 8·85)	8·35 (8·18 to 8·52)	8·18 (8·03 to 8·35)	7·92 (7·77 to 8·08)	7·72 (7·55 to 7·89)	7·64 (7·47 to 7·82)
Wales	11·54 (11·17 to 11·96)	10·76 (10·4 to 11·16)	9·78 (9·45 to 10·14)	9·3 (8·99 to 9·63)	8·74 (8·45 to 9·04)	8·84 (8·55 to 9·13)	8·69 (8·41 to 8·97)	8·52 (8·25 to 8·78)	7·82 (7·6 to 8·04)	7·8 (7·59 to 8·01)	7·42 (7·24 to 7·62)	7·1 (6·93 to 7·28)	6·97 (6·81 to 7·13)	6·71 (6·56 to 6·87)	6·63 (6·47 to 6·79)	6·65 (6·48 to 6·82)
**Southern Latin America**	10·28 (7·29 to 14·41)	9·82 (6·92 to 13·83)	9·41 (6·8 to 13·05)	9·1 (6·66 to 12·58)	8·88 (6·64 to 11·89)	8·75 (6·67 to 11·51)	8·61 (6·72 to 11·13)	8·45 (6·71 to 10·6)	8·32 (6·76 to 10·17)	8·24 (6·83 to 9·81)	8·19 (6·93 to 9·72)	8·06 (6·93 to 9·42)	7·86 (6·89 to 8·95)	7·66 (6·86 to 8·57)	7·43 (6·8 to 8·17)	7·25 (6·68 to 7·87)
Argentina	10·26 (7·45 to 13·79)	9·96 (7·26 to 13·45)	9·74 (7·18 to 13·11)	9·52 (7·14 to 12·75)	9·33 (7·04 to 12·24)	9·25 (6·93 to 12·14)	9·17 (7 to 11·97)	9·11 (7·08 to 11·56)	9·07 (7·09 to 11·36)	9·04 (7·16 to 11·18)	9·02 (7·27 to 11·06)	8·94 (7·34 to 10·82)	8·79 (7·38 to 10·42)	8·61 (7·47 to 9·87)	8·39 (7·48 to 9·35)	8·18 (7·47 to 8·95)
Chile	9·29 (5·3 to 15·75)	8·54 (4·88 to 14·07)	7·85 (4·62 to 12·65)	7·36 (4·35 to 11·66)	6·96 (4·3 to 10·74)	6·71 (4·29 to 10·02)	6·55 (4·37 to 9·32)	6·4 (4·5 to 8·79)	6·29 (4·7 to 8·24)	6·15 (4·83 to 7·68)	5·99 (4·94 to 7·21)	5·75 (4·84 to 6·76)	5·46 (4·63 to 6·34)	5·16 (4·37 to 5·95)	4·92 (4·2 to 5·75)	4·74 (4·08 to 5·54)
Uruguay	15·57 (13·03 to 18·47)	14·63 (12·25 to 17·31)	13·22 (11·17 to 15·72)	12·61 (10·7 to 14·98)	12·99 (11·08 to 15·35)	13·04 (11·06 to 15·35)	12·6 (10·81 to 14·72)	11·66 (10·09 to 13·52)	10·83 (9·35 to 12·51)	10·46 (9·09 to 11·98)	10·47 (9·17 to 11·87)	10·35 (9·13 to 11·62)	9·91 (8·86 to 11·08)	10 (8·98 to 11·05)	9·8 (8·89 to 10·81)	9·78 (8·89 to 10·75)
**Central Europe, Eastern Europe, and Central Asia**	10·04 (9·52 to 10·64)	9·86 (9·37 to 10·42)	9·56 (9·09 to 10·1)	9·45 (9·01 to 9·94)	9·29 (8·88 to 9·74)	9·13 (8·77 to 9·53)	8·77 (8·45 to 9·12)	8·66 (8·36 to 8·98)	8·49 (8·22 to 8·77)	8·33 (8·08 to 8·61)	8·24 (7·98 to 8·5)	8·2 (7·93 to 8·46)	8·2 (7·94 to 8·48)	8·21 (7·96 to 8·49)	8·26 (8 to 8·55)	8·15 (7·9 to 8·42)
**Eastern Europe**	7·95 (7·77 to 8·13)	7·81 (7·63 to 7·99)	7·36 (7·19 to 7·53)	7·46 (7·28 to 7·62)	7·25 (7·08 to 7·4)	7·13 (6·96 to 7·28)	6·8 (6·64 to 6·94)	6·69 (6·54 to 6·84)	6·59 (6·44 to 6·74)	6·34 (6·19 to 6·48)	6·18 (6·04 to 6·31)	6·21 (6·07 to 6·35)	6·18 (6·05 to 6·32)	6·33 (6·19 to 6·47)	6·27 (6·14 to 6·41)	6·26 (6·13 to 6·39)
Belarus	8·75 (8·61 to 8·88)	8·58 (8·45 to 8·71)	8·4 (8·28 to 8·53)	8·22 (8·1 to 8·36)	8·06 (7·94 to 8·18)	7·9 (7·79 to 8·03)	7·75 (7·64 to 7·87)	7·61 (7·5 to 7·73)	7·43 (7·32 to 7·54)	7·25 (7·15 to 7·37)	7·1 (7 to 7·21)	6·93 (6·83 to 7·04)	6·78 (6·68 to 6·89)	6·65 (6·55 to 6·75)	6·56 (6·46 to 6·66)	6·48 (6·38 to 6·57)
Estonia	6·45 (6·06 to 6·9)	6·44 (6·04 to 6·89)	6·09 (5·71 to 6·5)	6·25 (5·87 to 6·67)	5·38 (5·05 to 5·72)	5·86 (5·51 to 6·23)	5·4 (5·09 to 5·73)	5·73 (5·39 to 6·09)	5 (4·7 to 5·31)	5·56 (5·24 to 5·91)	5·05 (4·75 to 5·36)	4·82 (4·54 to 5·12)	5·61 (5·29 to 5·97)	5·28 (4·97 to 5·61)	4·78 (4·5 to 5·08)	5·39 (5·08 to 5·72)
Latvia	6·33 (6·1 to 6·55)	5·99 (5·78 to 6·21)	5·88 (5·68 to 6·1)	6·07 (5·86 to 6·3)	5·59 (5·39 to 5·8)	5·27 (5·09 to 5·48)	5·23 (5·04 to 5·43)	4·9 (4·73 to 5·09)	4·61 (4·46 to 4·79)	4·69 (4·53 to 4·87)	5·25 (5·08 to 5·44)	5·76 (5·57 to 5·97)	6·05 (5·86 to 6·27)	5·91 (5·73 to 6·12)	5·73 (5·56 to 5·94)	5·97 (5·79 to 6·17)
Lithuania	5·31 (5·1 to 5·53)	5·76 (5·54 to 6·01)	5·51 (5·3 to 5·75)	5·25 (5·05 to 5·48)	5·05 (4·85 to 5·28)	5·24 (5·03 to 5·47)	4·51 (4·34 to 4·71)	4·46 (4·28 to 4·65)	4·33 (4·15 to 4·52)	4·04 (3·87 to 4·22)	3·88 (3·73 to 4·04)	4·73 (4·55 to 4·93)	5·37 (5·17 to 5·59)	5·1 (4·91 to 5·3)	4·81 (4·64 to 5)	4·44 (4·27 to 4·61)
Moldova	13·3 (12·64 to 14·05)	12·96 (12·3 to 13·71)	12·48 (11·84 to 13·17)	11·96 (11·34 to 12·63)	11·37 (10·79 to 11·99)	10·68 (10·13 to 11·26)	10·04 (9·53 to 10·61)	9·62 (9·12 to 10·17)	9·26 (8·78 to 9·79)	8·94 (8·45 to 9·44)	8·62 (8·16 to 9·1)	8·38 (7·94 to 8·84)	8·38 (7·96 to 8·85)	8·51 (8·08 to 8·97)	8·85 (8·4 to 9·34)	9·06 (8·59 to 9·56)
Russia	7·58 (7·37 to 7·78)	7·51 (7·3 to 7·71)	7·01 (6·81 to 7·2)	7·29 (7·08 to 7·48)	7·13 (6·94 to 7·32)	7·07 (6·87 to 7·25)	6·72 (6·53 to 6·89)	6·66 (6·48 to 6·83)	6·58 (6·41 to 6·75)	6·25 (6·09 to 6·42)	6·07 (5·92 to 6·23)	6·16 (6 to 6·31)	6·12 (5·97 to 6·29)	6·37 (6·22 to 6·53)	6·24 (6·08 to 6·39)	6·17 (6·02 to 6·32)
Ukraine	8·53 (8·42 to 8·64)	8·21 (8·11 to 8·32)	7·87 (7·76 to 7·98)	7·54 (7·44 to 7·64)	7·25 (7·15 to 7·35)	6·98 (6·88 to 7·07)	6·75 (6·65 to 6·84)	6·56 (6·46 to 6·65)	6·47 (6·38 to 6·55)	6·38 (6·29 to 6·46)	6·27 (6·19 to 6·36)	6·13 (6·05 to 6·21)	6·02 (5·94 to 6·1)	6·04 (5·97 to 6·12)	6·19 (6·11 to 6·27)	6·31 (6·23 to 6·4)
**Central Europe**	8·68 (7·48 to 10·23)	8·42 (7·28 to 9·84)	8·33 (7·19 to 9·79)	7·96 (6·89 to 9·22)	7·84 (6·9 to 9·01)	7·67 (6·79 to 8·64)	7·19 (6·44 to 8)	7·22 (6·6 to 7·93)	6·83 (6·3 to 7·39)	6·73 (6·32 to 7·19)	6·63 (6·23 to 7·05)	6·37 (5·95 to 6·82)	6·19 (5·78 to 6·63)	5·91 (5·52 to 6·35)	6·15 (5·73 to 6·65)	5·73 (5·36 to 6·17)
Albania	7·52 (7·39 to 7·64)	7·4 (7·26 to 7·52)	7·32 (7·19 to 7·44)	7·25 (7·12 to 7·37)	7·05 (6·93 to 7·18)	6·74 (6·61 to 6·85)	6·4 (6·28 to 6·51)	6·12 (6·02 to 6·23)	5·93 (5·83 to 6·04)	5·75 (5·65 to 5·85)	5·59 (5·5 to 5·69)	5·53 (5·43 to 5·62)	5·55 (5·46 to 5·65)	5·62 (5·53 to 5·71)	5·68 (5·59 to 5·78)	5·71 (5·62 to 5·81)
Bosnia and Herzegovina	10·84 (9 to 12·87)	10·38 (8·63 to 12·21)	9·94 (8·29 to 11·75)	9·51 (7·97 to 11·33)	9·12 (7·73 to 10·89)	8·79 (7·45 to 10·43)	8·08 (6·86 to 9·62)	7·68 (6·54 to 9·12)	7·31 (6·22 to 8·56)	6·9 (5·9 to 8·04)	6·33 (5·46 to 7·28)	6·09 (5·3 to 6·95)	6·07 (5·31 to 6·86)	6·08 (5·36 to 6·8)	6·11 (5·45 to 6·83)	6·11 (5·5 to 6·73)
Bulgaria	5·75 (4·76 to 6·85)	5·53 (4·55 to 6·59)	5·45 (4·47 to 6·53)	5·11 (4·19 to 6·14)	5·06 (4·16 to 6·1)	4·77 (3·92 to 5·74)	4·56 (3·75 to 5·54)	4·57 (3·76 to 5·62)	4·47 (3·69 to 5·49)	4·49 (3·7 to 5·46)	4·49 (3·72 to 5·46)	4·88 (4·06 to 5·93)	4·79 (3·99 to 5·79)	4·61 (3·85 to 5·55)	4·87 (4·07 to 5·86)	4·44 (3·72 to 5·31)
Croatia	6·53 (6·11 to 6·97)	6·33 (5·94 to 6·77)	6·16 (5·78 to 6·59)	5·98 (5·63 to 6·39)	5·77 (5·43 to 6·17)	5·57 (5·24 to 5·95)	5·31 (5 to 5·67)	4·99 (4·69 to 5·31)	4·67 (4·39 to 4·97)	4·34 (4·09 to 4·62)	4·13 (3·9 to 4·39)	4·06 (3·84 to 4·31)	4·04 (3·82 to 4·28)	3·95 (3·74 to 4·17)	3·85 (3·65 to 4·05)	3·71 (3·53 to 3·9)
Czech Republic	4·68 (4·05 to 5·38)	4·42 (3·81 to 5·07)	4·33 (3·74 to 5·03)	4·23 (3·68 to 4·88)	4·14 (3·61 to 4·77)	3·77 (3·29 to 4·3)	3·57 (3·12 to 4·08)	3·42 (2·98 to 3·88)	3·23 (2·83 to 3·67)	3·01 (2·64 to 3·4)	3·11 (2·77 to 3·49)	3·08 (2·75 to 3·42)	2·91 (2·64 to 3·22)	2·72 (2·48 to 3·01)	2·68 (2·45 to 2·92)	2·61 (2·4 to 2·83)
Hungary	7·74 (7·39 to 8·1)	7·15 (6·82 to 7·48)	6·84 (6·51 to 7·16)	6·48 (6·17 to 6·79)	6·56 (6·23 to 6·88)	6·69 (6·37 to 7·02)	6·36 (6·05 to 6·67)	5·9 (5·62 to 6·2)	5·43 (5·18 to 5·71)	5·28 (5·03 to 5·56)	5·06 (4·83 to 5·34)	5·2 (4·96 to 5·46)	4·81 (4·58 to 5·05)	4·35 (4·15 to 4·57)	4·03 (3·85 to 4·23)	3·8 (3·63 to 3·99)
Macedonia	16·3 (12·67 to 20·96)	16·28 (12·7 to 20·89)	16·15 (12·69 to 20·68)	15·6 (12·36 to 19·78)	14·71 (11·74 to 18·67)	14·01 (11·22 to 17·57)	13·67 (11 to 17·02)	13·75 (11·1 to 16·96)	14·03 (11·4 to 17·12)	13·93 (11·43 to 16·72)	13·06 (10·93 to 15·4)	12·41 (10·6 to 14·4)	12·68 (10·98 to 14·49)	11·78 (10·29 to 13·34)	11·74 (10·41 to 13·23)	11·77 (10·57 to 13·08)
Montenegro	7·67 (6·86 to 8·52)	7·47 (6·69 to 8·31)	7·27 (6·58 to 8·06)	7·06 (6·42 to 7·74)	6·84 (6·2 to 7·48)	6·62 (6·03 to 7·26)	6·41 (5·86 to 7·01)	6·19 (5·69 to 6·73)	5·97 (5·5 to 6·46)	5·77 (5·33 to 6·23)	5·54 (5·15 to 5·94)	5·37 (5 to 5·74)	5·3 (4·95 to 5·67)	5·34 (5 to 5·71)	5·44 (5·1 to 5·79)	5·54 (5·2 to 5·92)
Poland	10·16 (7·61 to 13·59)	9·71 (7·36 to 12·83)	9·59 (7·35 to 12·48)	9·32 (7·2 to 11·88)	9·25 (7·31 to 11·59)	9·07 (7·26 to 11·11)	8·59 (7·08 to 10·35)	8·54 (7·18 to 10·16)	8·06 (6·9 to 9·29)	7·92 (7·01 to 8·99)	7·88 (6·97 to 8·92)	7·49 (6·52 to 8·6)	7·02 (5·93 to 8·22)	6·33 (5·26 to 7·58)	6·86 (5·64 to 8·3)	5·95 (4·87 to 7·3)
Romania	8·47 (8·41 to 8·53)	8·42 (8·36 to 8·48)	8·5 (8·44 to 8·55)	7·45 (7·4 to 7·5)	7·39 (7·34 to 7·44)	7·55 (7·5 to 7·6)	6·84 (6·79 to 6·88)	7·76 (7·7 to 7·81)	7·24 (7·19 to 7·29)	7·56 (7·51 to 7·61)	7·75 (7·69 to 7·8)	7·02 (6·97 to 7·06)	7·12 (7·07 to 7·17)	7·15 (7·1 to 7·2)	7·49 (7·44 to 7·54)	6·91 (6·87 to 6·96)
Serbia	10·35 (9·4 to 11·37)	10·24 (9·35 to 11·25)	10·14 (9·28 to 11·15)	9·96 (9·13 to 10·97)	9·97 (9·15 to 10·94)	9·63 (8·83 to 10·53)	9·22 (8·5 to 10·03)	8·84 (8·18 to 9·58)	8·49 (7·89 to 9·11)	8·23 (7·69 to 8·76)	8·04 (7·55 to 8·54)	7·68 (7·26 to 8·13)	7·8 (7·38 to 8·22)	7·88 (7·48 to 8·3)	7·95 (7·54 to 8·34)	8·02 (7·62 to 8·41)
Slovakia	6·34 (6·07 to 6·6)	5·96 (5·71 to 6·22)	5·7 (5·46 to 5·96)	5·56 (5·32 to 5·81)	5·47 (5·24 to 5·72)	5·27 (5·05 to 5·52)	4·92 (4·7 to 5·16)	4·74 (4·54 to 4·97)	4·52 (4·33 to 4·73)	4·41 (4·22 to 4·61)	4·07 (3·91 to 4·26)	4·29 (4·12 to 4·48)	4·05 (3·89 to 4·23)	3·66 (3·52 to 3·82)	3·6 (3·46 to 3·75)	3·54 (3·41 to 3·69)
Slovenia	5·55 (4·94 to 6·19)	5·38 (4·78 to 5·98)	5·21 (4·66 to 5·8)	5·11 (4·6 to 5·67)	4·92 (4·45 to 5·44)	4·67 (4·24 to 5·14)	4·39 (3·99 to 4·79)	4·08 (3·72 to 4·44)	3·75 (3·44 to 4·07)	3·47 (3·19 to 3·75)	3·28 (3·02 to 3·53)	3·15 (2·92 to 3·4)	3·05 (2·83 to 3·26)	2·91 (2·71 to 3·1)	2·8 (2·62 to 2·98)	2·7 (2·53 to 2·87)
**Central Asia**	15·79 (15·16 to 16·45)	15·52 (14·93 to 16·14)	15·21 (14·64 to 15·8)	14·9 (14·35 to 15·46)	14·59 (14·07 to 15·13)	14·26 (13·76 to 14·77)	13·9 (13·41 to 14·39)	13·51 (13·05 to 14)	13·16 (12·7 to 13·63)	12·86 (12·44 to 13·31)	12·62 (12·21 to 13·04)	12·44 (12·05 to 12·83)	12·43 (12·05 to 12·8)	12·45 (12·1 to 12·81)	12·5 (12·16 to 12·85)	12·49 (12·16 to 12·84)
Armenia	15·16 (12·88 to 17·73)	14·53 (12·4 to 16·97)	13·89 (11·94 to 16·21)	13·4 (11·6 to 15·66)	12·99 (11·28 to 15·03)	12·5 (10·89 to 14·38)	11·84 (10·36 to 13·44)	11·12 (9·8 to 12·56)	10·64 (9·4 to 11·98)	10·38 (9·23 to 11·61)	10·15 (9·08 to 11·31)	9·99 (8·98 to 11·06)	10·07 (9·09 to 11·09)	10·05 (9·11 to 11·04)	9·82 (8·93 to 10·76)	9·4 (8·59 to 10·26)
Azerbaijan	19·53 (18·77 to 20·28)	19·77 (18·97 to 20·54)	19·69 (18·89 to 20·44)	19·43 (18·65 to 20·18)	19·09 (18·31 to 19·84)	18·73 (17·96 to 19·46)	18·4 (17·64 to 19·11)	18·11 (17·38 to 18·81)	17·73 (17·01 to 18·4)	17·36 (16·68 to 18·01)	17·18 (16·53 to 17·82)	17·11 (16·47 to 17·75)	17·18 (16·55 to 17·82)	17·23 (16·62 to 17·89)	17·24 (16·64 to 17·91)	17·12 (16·53 to 17·8)
Georgia	15·99 (14·48 to 17·54)	15·3 (13·87 to 16·76)	14·69 (13·35 to 16·06)	14·15 (12·92 to 15·48)	13·71 (12·52 to 14·95)	13·33 (12·21 to 14·54)	13·01 (11·95 to 14·17)	12·74 (11·72 to 13·82)	12·49 (11·54 to 13·5)	12·27 (11·32 to 13·24)	12·03 (11·16 to 13)	11·81 (11 to 12·72)	11·79 (11·01 to 12·62)	11·84 (11·12 to 12·63)	11·87 (11·17 to 12·64)	11·84 (11·19 to 12·56)
Kazakhstan	11·74 (11 to 12·52)	11·47 (10·76 to 12·23)	11·17 (10·49 to 11·89)	10·9 (10·26 to 11·61)	10·69 (10·09 to 11·38)	10·5 (9·94 to 11·14)	10·31 (9·78 to 10·89)	10·14 (9·63 to 10·71)	10 (9·52 to 10·53)	9·89 (9·43 to 10·39)	9·78 (9·35 to 10·27)	9·8 (9·39 to 10·26)	9·87 (9·47 to 10·31)	9·97 (9·59 to 10·39)	10·1 (9·72 to 10·5)	10·2 (9·82 to 10·56)
Kyrgyzstan	23·91 (23 to 24·81)	23·25 (22·38 to 24·15)	22·62 (21·72 to 23·47)	21·99 (21·11 to 22·79)	21·35 (20·51 to 22·14)	20·73 (19·88 to 21·52)	20·07 (19·3 to 20·86)	19·43 (18·64 to 20·18)	18·81 (18·05 to 19·54)	18·11 (17·4 to 18·83)	17·37 (16·7 to 18·08)	16·68 (16·03 to 17·33)	16·13 (15·54 to 16·77)	15·65 (15·11 to 16·25)	15·29 (14·72 to 15·86)	14·99 (14·45 to 15·56)
Mongolia	19·39 (18·64 to 20·14)	19·06 (18·29 to 19·81)	18·68 (17·92 to 19·4)	18·29 (17·54 to 18·99)	17·89 (17·15 to 18·59)	17·52 (16·82 to 18·21)	17·15 (16·46 to 17·83)	16·77 (16·11 to 17·45)	16·36 (15·72 to 17·02)	15·99 (15·37 to 16·63)	15·48 (14·88 to 16·08)	15·04 (14·47 to 15·61)	14·66 (14·12 to 15·22)	14·34 (13·83 to 14·89)	14 (13·51 to 14·53)	13·6 (13·12 to 14·13)
Tajikistan	21·59 (21·04 to 22·13)	20·86 (20·32 to 21·38)	20·26 (19·75 to 20·77)	19·75 (19·25 to 20·25)	19·34 (18·85 to 19·82)	18·97 (18·47 to 19·45)	18·55 (18·08 to 19·03)	18·05 (17·59 to 18·52)	17·5 (17·05 to 17·96)	16·93 (16·5 to 17·38)	16·44 (16·02 to 16·87)	15·96 (15·56 to 16·39)	15·92 (15·53 to 16·35)	15·94 (15·55 to 16·38)	15·88 (15·49 to 16·32)	15·68 (15·29 to 16·11)
Turkmenistan	19·32 (19 to 19·63)	18·76 (18·46 to 19·06)	18·24 (17·95 to 18·54)	17·83 (17·55 to 18·13)	17·35 (17·07 to 17·63)	16·91 (16·65 to 17·19)	16·54 (16·29 to 16·8)	16·11 (15·86 to 16·37)	15·8 (15·56 to 16·06)	15·47 (15·23 to 15·72)	15·12 (14·89 to 15·37)	14·89 (14·66 to 15·14)	14·77 (14·55 to 15·02)	14·65 (14·42 to 14·88)	14·7 (14·47 to 14·93)	14·7 (14·46 to 14·94)
Uzbekistan	13·41 (12·89 to 13·93)	13·35 (12·81 to 13·87)	13·2 (12·66 to 13·71)	12·99 (12·46 to 13·49)	12·74 (12·22 to 13·24)	12·39 (11·89 to 12·87)	11·95 (11·47 to 12·42)	11·46 (11·01 to 11·92)	11·05 (10·62 to 11·49)	10·77 (10·35 to 11·2)	10·56 (10·15 to 10·97)	10·42 (10·03 to 10·82)	10·46 (10·08 to 10·86)	10·54 (10·17 to 10·95)	10·71 (10·33 to 11·12)	10·84 (10·46 to 11·26)
**Latin America and Caribbean**	20·05 (17·56 to 22·88)	19·26 (16·93 to 21·89)	18·54 (16·35 to 21·02)	17·93 (15·85 to 20·38)	17·33 (15·35 to 19·63)	16·72 (14·87 to 18·98)	16·08 (14·3 to 18·26)	15·47 (13·77 to 17·47)	14·87 (13·26 to 16·73)	14·33 (12·82 to 16·06)	13·85 (12·44 to 15·47)	13·4 (12·06 to 14·96)	12·99 (11·7 to 14·45)	12·57 (11·35 to 13·91)	12·14 (11·03 to 13·4)	11·72 (10·68 to 12·89)
**Central Latin America**	13·7 (12·86 to 14·62)	13·24 (12·46 to 14·12)	12·81 (12·06 to 13·63)	12·44 (11·72 to 13·21)	12·06 (11·38 to 12·79)	11·68 (11·03 to 12·36)	11·34 (10·71 to 11·99)	11·03 (10·43 to 11·65)	10·74 (10·18 to 11·35)	10·47 (9·94 to 11·05)	10·23 (9·7 to 10·8)	10 (9·48 to 10·55)	9·78 (9·26 to 10·32)	9·57 (9·05 to 10·1)	9·34 (8·84 to 9·87)	9·1 (8·61 to 9·62)
Colombia	16·19 (14·62 to 18·01)	15·85 (14·31 to 17·65)	15·52 (14 to 17·18)	15·21 (13·71 to 16·79)	14·88 (13·4 to 16·42)	14·55 (13·13 to 16·05)	14·25 (12·85 to 15·74)	14 (12·6 to 15·48)	13·77 (12·42 to 15·26)	13·56 (12·27 to 15·01)	13·39 (12·11 to 14·8)	13·25 (12·02 to 14·63)	13·1 (11·87 to 14·47)	12·95 (11·7 to 14·33)	12·75 (11·5 to 14·06)	12·51 (11·27 to 13·86)
Costa Rica	9·78 (9·23 to 10·36)	9·59 (9·09 to 10·1)	9·44 (8·97 to 9·91)	9·31 (8·89 to 9·74)	9·2 (8·79 to 9·61)	9·11 (8·72 to 9·51)	9·02 (8·65 to 9·38)	8·93 (8·57 to 9·29)	8·84 (8·49 to 9·18)	8·71 (8·37 to 9·07)	8·61 (8·29 to 8·95)	8·52 (8·19 to 8·87)	8·41 (8·08 to 8·75)	8·27 (7·94 to 8·62)	8·09 (7·76 to 8·43)	7·89 (7·56 to 8·23)
El Salvador	19·37 (18·86 to 19·88)	18·9 (18·38 to 19·4)	18·32 (17·81 to 18·8)	17·49 (17 to 17·94)	16·51 (16·05 to 16·95)	15·49 (15·07 to 15·9)	14·57 (14·17 to 14·96)	13·8 (13·43 to 14·18)	13·22 (12·87 to 13·58)	12·75 (12·41 to 13·09)	12·29 (11·97 to 12·61)	11·82 (11·52 to 12·13)	11·36 (11·07 to 11·65)	10·9 (10·64 to 11·19)	10·56 (10·31 to 10·83)	10·25 (10 to 10·52)
Guatemala	24·74 (21·64 to 28·05)	23·75 (20·73 to 27·06)	23·02 (20·04 to 26·41)	22·3 (19·48 to 25·61)	21·44 (18·82 to 24·59)	20·5 (18·03 to 23·52)	19·59 (17·17 to 22·49)	18·78 (16·52 to 21·49)	17·95 (15·81 to 20·49)	17·18 (15·18 to 19·52)	16·63 (14·72 to 18·86)	16·24 (14·37 to 18·41)	15·82 (14·05 to 17·84)	15·3 (13·63 to 17·2)	14·78 (13·21 to 16·59)	14·23 (12·79 to 15·89)
Honduras	20·33 (20·22 to 20·44)	19·6 (19·49 to 19·71)	18·87 (18·76 to 18·97)	18·25 (18·14 to 18·35)	17·63 (17·53 to 17·73)	17·05 (16·95 to 17·14)	16·51 (16·42 to 16·6)	16·02 (15·93 to 16·11)	15·54 (15·45 to 15·63)	15·11 (15·03 to 15·19)	14·76 (14·68 to 14·84)	14·43 (14·36 to 14·51)	14·11 (14·03 to 14·18)	13·79 (13·72 to 13·87)	13·5 (13·43 to 13·58)	13·21 (13·15 to 13·29)
Mexico	11·01 (10·5 to 11·55)	10·56 (10·07 to 11·07)	10·14 (9·67 to 10·63)	9·8 (9·35 to 10·27)	9·49 (9·05 to 9·94)	9·18 (8·77 to 9·62)	8·92 (8·52 to 9·34)	8·67 (8·28 to 9·08)	8·44 (8·06 to 8·84)	8·2 (7·84 to 8·58)	7·96 (7·61 to 8·33)	7·73 (7·39 to 8·07)	7·5 (7·18 to 7·83)	7·29 (6·98 to 7·61)	7·07 (6·77 to 7·37)	6·88 (6·59 to 7·17)
Nicaragua	19·25 (18·96 to 19·54)	18·51 (18·22 to 18·79)	17·79 (17·51 to 18·05)	17·04 (16·77 to 17·29)	16·35 (16·09 to 16·6)	15·69 (15·44 to 15·93)	15·1 (14·86 to 15·33)	14·5 (14·27 to 14·72)	14·04 (13·82 to 14·25)	13·6 (13·4 to 13·79)	13·16 (12·96 to 13·35)	12·71 (12·52 to 12·9)	12·28 (12·11 to 12·46)	11·89 (11·73 to 12·07)	11·51 (11·35 to 11·68)	11·13 (10·98 to 11·3)
Panama	8·99 (8·18 to 9·87)	8·78 (7·99 to 9·63)	8·55 (7·78 to 9·41)	8·36 (7·64 to 9·19)	8·2 (7·49 to 9·03)	8·07 (7·39 to 8·86)	7·91 (7·25 to 8·66)	7·77 (7·15 to 8·48)	7·68 (7·09 to 8·37)	7·59 (7·02 to 8·25)	7·49 (6·95 to 8·11)	7·36 (6·86 to 7·95)	7·22 (6·77 to 7·77)	7·1 (6·68 to 7·59)	6·99 (6·61 to 7·43)	6·9 (6·55 to 7·3)

Appendix Table 17B. Stillbirth rates (per 1,000 live births) by location, 1980-1995

Location	1980	1981	1982	1983	1984	1985	1986	1987	1988	1989	1990	1991	1992	1993	1994	1995
Venezuela	12·33 (11·34 to 13·43)	12·12 (11·12 to 13·18)	11·74 (10·78 to 12·78)	11·42 (10·49 to 12·4)	11·08 (10·17 to 12·01)	10·78 (9·9 to 11·69)	10·51 (9·67 to 11·4)	10·26 (9·47 to 11·12)	10·03 (9·28 to 10·86)	9·9 (9·17 to 10·66)	9·81 (9·09 to 10·53)	9·67 (8·97 to 10·37)	9·59 (8·92 to 10·23)	9·56 (8·9 to 10·18)	9·55 (8·91 to 10·16)	9·37 (8·78 to 9·95)
**Andean Latin America**	31·13 (26·86 to 36·09)	30·1 (25·96 to 34·89)	29·13 (25·18 to 33·63)	28·23 (24·42 to 32·69)	27·31 (23·58 to 31·51)	26·35 (22·76 to 30·37)	25·36 (21·97 to 29·43)	24·3 (21·05 to 28·14)	23·25 (20·12 to 26·95)	22·36 (19·34 to 25·91)	21·5 (18·65 to 24·88)	20·62 (17·94 to 23·92)	19·74 (17·17 to 22·92)	18·85 (16·38 to 21·82)	17·92 (15·54 to 20·77)	17·02 (14·78 to 19·64)
Bolivia	39·69 (32·62 to 47·66)	38·44 (31·33 to 46·24)	37·3 (30·3 to 44·75)	36·33 (29·56 to 43·64)	35·33 (28·64 to 42·52)	34·31 (27·91 to 41·16)	33·37 (27·22 to 40·06)	32·46 (26·58 to 39·21)	31·57 (25·94 to 38·16)	30·65 (25·16 to 36·97)	29·73 (24·43 to 35·69)	28·77 (23·71 to 34·46)	27·8 (23·09 to 33·25)	26·82 (22·39 to 32·11)	25·8 (21·55 to 30·77)	24·76 (20·61 to 29·61)
Ecuador	20·51 (16·54 to 25·09)	19·87 (15·88 to 24·46)	19·2 (15·25 to 23·53)	18·54 (14·83 to 22·72)	17·94 (14·47 to 21·96)	17·37 (14·01 to 21·33)	16·86 (13·58 to 20·91)	16·38 (13·22 to 20·37)	15·79 (12·77 to 19·6)	15·24 (12·34 to 18·93)	14·73 (11·98 to 18·28)	14·24 (11·56 to 17·7)	13·78 (11·2 to 17·12)	13·33 (10·87 to 16·5)	12·88 (10·52 to 15·93)	12·46 (10·24 to 15·38)
Peru	32·96 (28·18 to 38·08)	31·87 (27·14 to 37·05)	30·86 (26·3 to 36·02)	29·9 (25·51 to 35·05)	28·86 (24·63 to 33·98)	27·74 (23·7 to 32·63)	26·49 (22·54 to 31·41)	25·1 (21·43 to 29·61)	23·78 (20·29 to 28·02)	22·75 (19·47 to 26·77)	21·75 (18·66 to 25·57)	20·71 (17·78 to 24·23)	19·64 (16·93 to 23·01)	18·53 (15·99 to 21·61)	17·37 (15·02 to 20·15)	16·24 (14·06 to 18·77)
**Caribbean**	31·48 (26·33 to 37·18)	30·15 (25·04 to 35·78)	28·95 (23·97 to 34·45)	28·2 (23·26 to 33·51)	27·36 (22·66 to 32·54)	26·69 (22·25 to 31·69)	25·99 (21·76 to 30·82)	25·04 (21·07 to 29·48)	24·23 (20·6 to 28·27)	23·72 (20·44 to 27·36)	23·49 (20·26 to 27·04)	23·42 (20·26 to 27·01)	23·23 (20·13 to 26·8)	22·87 (19·86 to 26·3)	22·47 (19·54 to 25·99)	22·05 (19·25 to 25·55)
Antigua and Barbuda	12·1 (9·69 to 14·86)	11·56 (9·18 to 14·21)	10·92 (8·65 to 13·36)	10·15 (8·05 to 12·44)	9·95 (7·89 to 12·23)	9·57 (7·56 to 11·75)	9 (7·11 to 11·03)	9·07 (7·2 to 11·1)	9·44 (7·49 to 11·52)	9·65 (7·75 to 11·76)	9·95 (8·02 to 12·1)	9·92 (8·01 to 12·08)	10·26 (8·34 to 12·49)	10·93 (8·93 to 13·37)	11·32 (9·28 to 13·89)	11·62 (9·54 to 14·31)
The Bahamas	17·26 (10·96 to 26·11)	16·74 (10·64 to 25·34)	16·03 (10·4 to 24·08)	15·47 (10·16 to 23·1)	14·93 (9·95 to 22·15)	14·78 (9·94 to 21·87)	14·54 (9·94 to 21·25)	13·84 (9·66 to 19·64)	13·12 (9·26 to 18·2)	12·87 (9·33 to 17·33)	12·62 (9·44 to 16·44)	12·09 (9·3 to 15·34)	11·44 (9·09 to 14·09)	11·21 (9·18 to 13·42)	11·38 (9·67 to 13·25)	10·94 (9·59 to 12·46)
Barbados	12·79 (10·25 to 15·69)	12·32 (9·79 to 15·15)	11·91 (9·42 to 14·58)	11·75 (9·32 to 14·42)	11·51 (9·09 to 14·16)	11·39 (9·03 to 13·96)	11·18 (8·89 to 13·71)	10·99 (8·77 to 13·57)	10·75 (8·61 to 13·27)	10·53 (8·43 to 12·99)	10·34 (8·29 to 12·69)	10·2 (8·2 to 12·48)	10·11 (8·2 to 12·34)	10·01 (8·17 to 12·24)	10·05 (8·22 to 12·24)	10·05 (8·18 to 12·28)
Belize	18·84 (15·08 to 23·12)	18·53 (14·72 to 22·79)	18·06 (14·31 to 22·1)	17·39 (13·8 to 21·31)	16·62 (13·17 to 20·44)	15·94 (12·58 to 19·56)	15·43 (12·18 to 18·89)	15·1 (11·99 to 18·49)	14·87 (11·79 to 18·14)	14·76 (11·85 to 17·99)	14·71 (11·86 to 17·9)	14·7 (11·86 to 17·9)	14·57 (11·85 to 17·74)	14·25 (11·63 to 17·43)	13·81 (11·32 to 16·94)	13·34 (10·95 to 16·43)
Bermuda	15·13 (11·56 to 19·16)	13·94 (10·73 to 17·73)	13·18 (10·13 to 16·76)	12·47 (9·72 to 15·88)	11·37 (8·86 to 14·67)	10·89 (8·55 to 13·93)	10·25 (8·1 to 13·07)	9·39 (7·46 to 11·89)	8·73 (6·96 to 10·99)	8·73 (7 to 10·9)	9·05 (7·26 to 11·24)	9·24 (7·47 to 11·52)	9·61 (7·82 to 11·86)	9·45 (7·79 to 11·61)	9·07 (7·55 to 11·06)	8·31 (6·99 to 10·02)
Cuba	15·39 (8·83 to 26·47)	14·73 (8·52 to 24·64)	14·12 (8·22 to 22·76)	13·49 (8·19 to 20·9)	12·81 (8·23 to 18·81)	12·34 (8·33 to 17·47)	11·7 (8·57 to 15·55)	11·27 (8·82 to 14·2)	10·91 (9·18 to 12·8)	10·7 (9·72 to 11·77)	10·76 (10·15 to 11·41)	10·98 (10·41 to 11·56)	11·11 (10·58 to 11·69)	11·16 (10·62 to 11·73)	11·08 (10·51 to 11·65)	10·93 (10·41 to 11·49)
Dominica	9·01 (7·22 to 11·06)	8·69 (6·91 to 10·69)	8·4 (6·65 to 10·28)	8·26 (6·56 to 10·13)	8·23 (6·52 to 10·12)	8·23 (6·49 to 10·1)	8·22 (6·49 to 10·06)	8·24 (6·54 to 10·08)	8·26 (6·55 to 10·08)	8·4 (6·74 to 10·24)	8·58 (6·91 to 10·43)	8·83 (7·13 to 10·75)	9·08 (7·38 to 11·05)	9·3 (7·59 to 11·38)	9·56 (7·84 to 11·73)	9·8 (8·04 to 12·07)
Dominican Republic	24·82 (22·25 to 27·74)	23·84 (21·44 to 26·54)	22·91 (20·63 to 25·41)	22·16 (19·97 to 24·56)	21·7 (19·47 to 24·11)	21·4 (19·22 to 23·79)	21·1 (18·92 to 23·43)	20·66 (18·56 to 22·96)	20·1 (18·05 to 22·35)	19·5 (17·54 to 21·69)	18·99 (17·05 to 21·12)	18·45 (16·61 to 20·44)	17·84 (16·11 to 19·69)	17·27 (15·6 to 19·05)	16·76 (15·17 to 18·5)	16·34 (14·81 to 18·04)
Grenada	15·07 (12·08 to 18·48)	14·62 (11·61 to 17·97)	14·2 (11·23 to 17·38)	13·8 (10·95 to 16·94)	13·41 (10·59 to 16·5)	12·97 (10·28 to 15·9)	12·65 (10·06 to 15·51)	12·24 (9·78 to 15·12)	11·83 (9·48 to 14·61)	11·39 (9·12 to 14·05)	11·05 (8·86 to 13·55)	10·63 (8·55 to 13)	10·33 (8·38 to 12·62)	10·16 (8·3 to 12·43)	10·09 (8·26 to 12·29)	10·04 (8·17 to 12·26)
Guyana	25·37 (23·44 to 27·37)	24·7 (22·76 to 26·66)	24·11 (22·2 to 25·96)	23·74 (21·86 to 25·6)	23·5 (21·61 to 25·37)	23·18 (21·29 to 24·97)	22·83 (20·99 to 24·6)	22·44 (20·67 to 24·18)	22·1 (20·35 to 23·8)	21·78 (20·14 to 23·41)	21·43 (19·84 to 23·02)	21·04 (19·48 to 22·63)	20·54 (19·07 to 22·08)	20·03 (18·64 to 21·57)	19·49 (18·14 to 21·02)	18·97 (17·68 to 20·48)
Haiti	59·49 (47·63 to 73·02)	56·86 (45·18 to 69·92)	54·61 (43·25 to 66·81)	52·73 (41·84 to 64·63)	51·07 (40·47 to 62·79)	49·37 (38·96 to 60·6)	47·61 (37·57 to 58·3)	45·85 (36·39 to 56·12)	44·17 (35·02 to 53·9)	43·14 (34·63 to 52·58)	42·53 (34·28 to 51·74)	41·88 (33·79 to 50·98)	41·18 (33·51 to 50·15)	40·3 (32·91 to 49·3)	39·32 (32·23 to 48·25)	38·22 (31·37 to 47·06)
Jamaica	19·07 (16·99 to 21·32)	18·94 (16·86 to 21·24)	18·81 (16·76 to 21·14)	18·56 (16·51 to 20·89)	18·21 (16·2 to 20·55)	17·86 (15·94 to 20·2)	17·56 (15·71 to 19·83)	17·34 (15·54 to 19·49)	17·17 (15·43 to 19·28)	17·1 (15·38 to 19·16)	17·02 (15·31 to 19)	16·93 (15·27 to 18·8)	16·89 (15·28 to 18·67)	16·77 (15·25 to 18·48)	16·6 (15·12 to 18·14)	16·41 (15·03 to 17·86)
Puerto Rico	13·26 (12·66 to 13·85)	13·02 (12·43 to 13·62)	12·69 (12·12 to 13·27)	12·28 (11·71 to 12·83)	11·8 (11·25 to 12·33)	11·33 (10·81 to 11·84)	10·93 (10·42 to 11·42)	10·69 (10·18 to 11·17)	10·51 (10·03 to 10·98)	10·41 (9·95 to 10·89)	10·35 (9·89 to 10·83)	10·31 (9·85 to 10·78)	10·2 (9·75 to 10·65)	10·01 (9·58 to 10·46)	10·12 (9·7 to 10·59)	9·86 (9·44 to 10·3)
Saint Lucia	14·84 (13·02 to 16·67)	14·71 (12·88 to 16·56)	14·55 (12·73 to 16·38)	14·41 (12·67 to 16·2)	14·29 (12·63 to 16·05)	14·22 (12·66 to 15·97)	14·12 (12·58 to 15·82)	14·04 (12·5 to 15·72)	13·87 (12·39 to 15·43)	13·73 (12·31 to 15·26)	13·59 (12·25 to 15·11)	13·52 (12·28 to 14·95)	13·47 (12·25 to 14·77)	13·4 (12·22 to 14·65)	13·31 (12·19 to 14·54)	13·21 (12·16 to 14·4)
Saint Vincent and the Grenadines	10·15 (6·91 to 14·36)	9·96 (6·78 to 14·28)	9·77 (6·71 to 14·03)	9·59 (6·63 to 13·84)	9·46 (6·57 to 13·58)	9·36 (6·55 to 13·34)	9·31 (6·59 to 13·27)	9·3 (6·59 to 12·97)	9·27 (6·62 to 12·77)	9·3 (6·72 to 12·69)	9·29 (6·84 to 12·49)	9·3 (6·91 to 12·2)	9·27 (7·01 to 11·93)	9·25 (7·17 to 11·71)	9·28 (7·33 to 11·52)	9·29 (7·52 to 11·32)
Suriname	22·44 (19·36 to 25·74)	22·15 (19·05 to 25·5)	21·91 (18·87 to 25·23)	21·69 (18·72 to 25·16)	21·5 (18·59 to 25)	21·26 (18·32 to 24·87)	21·07 (18·17 to 24·63)	20·98 (18·18 to 24·45)	20·85 (18·08 to 24·19)	20·67 (17·95 to 23·95)	20·47 (17·81 to 23·58)	20·33 (17·72 to 23·43)	20·18 (17·61 to 23·15)	20·16 (17·65 to 23·05)	20·17 (17·71 to 23·01)	20·14 (17·77 to 22·89)
Trinidad and Tobago	15·97 (14·42 to 17·56)	15·36 (13·94 to 16·76)	14·76 (13·5 to 16·09)	14·33 (13·12 to 15·59)	14·02 (12·9 to 15·26)	13·71 (12·64 to 14·9)	13·45 (12·41 to 14·57)	13·31 (12·27 to 14·39)	13·3 (12·29 to 14·35)	13·28 (12·29 to 14·3)	13·32 (12·32 to 14·39)	13·34 (12·33 to 14·34)	13·41 (12·42 to 14·47)	13·47 (12·49 to 14·52)	13·55 (12·52 to 14·63)	13·61 (12·59 to 14·71)
Virgin Islands, U.S.	11·5 (9·22 to 14·11)	11·29 (8·97 to 13·89)	11·08 (8·77 to 13·57)	10·81 (8·57 to 13·26)	10·49 (8·28 to 12·9)	10·18 (8·07 to 12·48)	9·89 (7·87 to 12·13)	9·79 (7·82 to 12·1)	9·67 (7·75 to 11·94)	9·62 (7·7 to 11·86)	9·54 (7·65 to 11·7)	9·47 (7·62 to 11·58)	9·38 (7·61 to 11·46)	9·29 (7·59 to 11·37)	9·21 (7·54 to 11·22)	9·03 (7·34 to 11·03)
**Tropical Latin America**	21·67 (17·79 to 26·06)	20·71 (17·03 to 25·14)	19·86 (16·36 to 24·12)	19·13 (15·81 to 23·2)	18·44 (15·24 to 22·58)	17·71 (14·65 to 21·6)	16·88 (14·09 to 20·45)	16·11 (13·51 to 19·55)	15·36 (12·9 to 18·41)	14·63 (12·29 to 17·44)	13·98 (11·78 to 16·62)	13·35 (11·29 to 15·8)	12·81 (10·92 to 14·96)	12·27 (10·55 to 14·19)	11·74 (10·22 to 13·43)	11·21 (9·86 to 12·7)
Brazil	21·76 (17·9 to 26·1)	20·8 (17·15 to 25·17)	19·94 (16·47 to 24·15)	19·21 (15·92 to 23·22)	18·51 (15·35 to 22·59)	17·77 (14·76 to 21·6)	16·94 (14·16 to 20·42)	16·16 (13·58 to 19·51)	15·41 (12·98 to 18·38)	14·67 (12·37 to 17·42)	14·01 (11·84 to 16·6)	13·38 (11·37 to 15·74)	12·83 (10·99 to 14·9)	12·29 (10·59 to 14·15)	11·74 (10·26 to 13·37)	11·2 (9·89 to 12·59)
Paraguay	18·01 (13·12 to 23·86)	17·42 (12·69 to 23·17)	16·94 (12·37 to 22·66)	16·55 (12·22 to 22·42)	16·13 (11·87 to 21·85)	15·68 (11·57 to 21·46)	15·22 (11·14 to 20·91)	14·72 (10·87 to 20·03)	14·16 (10·5 to 19·12)	13·59 (10·11 to 18·24)	13·04 (9·72 to 17·44)	12·58 (9·41 to 16·73)	12·24 (9·23 to 16·28)	11·97 (9·13 to 15·59)	11·72 (8·99 to 15·19)	11·5 (8·92 to 14·82)
**Southeast Asia, East Asia, and Oceania**	21·24 (19·76 to 22·78)	20·56 (19·16 to 22·03)	19·91 (18·59 to 21·33)	19·3 (18·02 to 20·75)	18·7 (17·47 to 20·12)	18·14 (16·93 to 19·47)	17·57 (16·43 to 18·88)	17·06 (15·95 to 18·36)	16·56 (15·5 to 17·8)	16·1 (15·1 to 17·28)	15·69 (14·72 to 16·76)	15·34 (14·4 to 16·36)	14·93 (14·04 to 15·92)	14·5 (13·66 to 15·44)	14·09 (13·31 to 14·97)	13·71 (12·94 to 14·53)
**East Asia**	19·82 (17·87 to 21·87)	19·21 (17·3 to 21·19)	18·57 (16·73 to 20·48)	17·97 (16·21 to 19·93)	17·37 (15·69 to 19·33)	16·83 (15·18 to 18·74)	16·26 (14·68 to 18·17)	15·75 (14·23 to 17·52)	15·25 (13·83 to 16·9)	14·79 (13·46 to 16·37)	14·41 (13·12 to 15·88)	14·09 (12·83 to 15·55)	13·7 (12·47 to 15·1)	13·3 (12·14 to 14·61)	12·92 (11·81 to 14·18)	12·57 (11·52 to 13·77)
China	20·21 (18·19 to 22·32)	19·58 (17·62 to 21·62)	18·93 (17·04 to 20·9)	18·32 (16·51 to 20·32)	17·71 (15·95 to 19·72)	17·14 (15·44 to 19·13)	16·56 (14·94 to 18·49)	16·04 (14·46 to 17·86)	15·52 (14·06 to 17·23)	15·06 (13·67 to 16·71)	14·68 (13·34 to 16·19)	14·36 (13·07 to 15·89)	13·97 (12·7 to 15·43)	13·58 (12·37 to 14·96)	13·19 (12·04 to 14·5)	12·83 (11·73 to 14·11)
North Korea	10·41 (8·97 to 12)	10·3 (8·82 to 11·88)	10·12 (8·64 to 11·65)	10 (8·54 to 11·52)	9·87 (8·41 to 11·39)	9·79 (8·36 to 11·26)	9·66 (8·27 to 11·13)	9·62 (8·26 to 11·13)	9·4 (8·09 to 10·88)	9·26 (7·97 to 10·71)	9·1 (7·84 to 10·48)	9·06 (7·82 to 10·4)	9·04 (7·85 to 10·38)	8·95 (7·8 to 10·29)	8·99 (7·84 to 10·3)	9·16 (7·96 to 10·51)
Taiwan (Province of China)	9·95 (9·41 to 10·51)	9·38 (8·87 to 9·91)	8·68 (8·21 to 9·18)	8·12 (7·67 to 8·59)	7·57 (7·16 to 8·01)	7·52 (7·12 to 7·96)	7·3 (6·91 to 7·7)	6·68 (6·33 to 7·05)	6·67 (6·32 to 7·05)	6·68 (6·32 to 7·06)	6·61 (6·26 to 7)	6·43 (6·08 to 6·79)	6·4 (6·07 to 6·76)	6·17 (5·85 to 6·52)	5·96 (5·65 to 6·3)	6·3 (5·95 to 6·67)
**Southeast Asia**	23·88 (23·05 to 24·76)	23·08 (22·26 to 23·91)	22·41 (21·62 to 23·17)	21·76 (20·98 to 22·48)	21·13 (20·37 to 21·83)	20·51 (19·83 to 21·2)	19·93 (19·25 to 20·59)	19·44 (18·77 to 20·06)	18·9 (18·27 to 19·5)	18·39 (17·82 to 18·96)	17·85 (17·33 to 18·39)	17·31 (16·83 to 17·83)	16·78 (16·32 to 17·27)	16·2 (15·76 to 16·67)	15·68 (15·26 to 16·14)	15·16 (14·76 to 15·62)
Cambodia	41·87 (40·59 to 43·14)	40·74 (39·46 to 41·99)	39·54 (38·3 to 40·72)	38·38 (37·16 to 39·53)	37·27 (36·08 to 38·42)	36·24 (35·06 to 37·34)	35·37 (34·23 to 36·43)	34·46 (33·37 to 35·48)	33·47 (32·41 to 34·46)	32·59 (31·61 to 33·53)	31·91 (30·96 to 32·82)	31·27 (30·35 to 32·18)	30·73 (29·85 to 31·63)	30·33 (29·49 to 31·23)	30·01 (29·18 to 30·92)	29·61 (28·81 to 30·52)
Indonesia	28·86 (28·04 to 29·67)	27·87 (27·06 to 28·66)	26·97 (26·18 to 27·71)	26·2 (25·43 to 26·93)	25·48 (24·73 to 26·2)	24·76 (24·02 to 25·45)	24·09 (23·37 to 24·76)	23·44 (22·75 to 24·08)	22·8 (22·13 to 23·43)	22·19 (21·57 to 22·79)	21·53 (20·94 to 22·1)	20·84 (20·27 to 21·41)	20·13 (19·59 to 20·67)	19·38 (18·88 to 19·91)	18·63 (18·15 to 19·16)	17·88 (17·43 to 18·39)
Laos	60·42 (59·39 to 61·44)	58·82 (57·78 to 59·83)	57·33 (56·3 to 58·29)	55·77 (54·76 to 56·7)	54·06 (53·07 to 54·97)	52·88 (51·94 to 53·78)	51·9 (50·98 to 52·8)	50·99 (50·11 to 51·88)	50·18 (49·32 to 51·06)	49·23 (48·39 to 50·09)	48·07 (47·24 to 48·88)	46·83 (46·04 to 47·61)	45·51 (44·77 to 46·26)	44·22 (43·53 to 44·96)	42·96 (42·29 to 43·67)	41·71 (41·06 to 42·42)
Malaysia	8·84 (7·36 to 10·49)	8·46 (7·04 to 10·07)	8·13 (6·79 to 9·68)	7·81 (6·58 to 9·3)	7·48 (6·35 to 8·88)	7·23 (6·16 to 8·58)	6·81 (5·83 to 7·96)	6·51 (5·59 to 7·59)	6·24 (5·4 to 7·23)	5·97 (5·19 to 6·86)	5·7 (5 to 6·46)	5·4 (4·79 to 6·05)	5·22 (4·68 to 5·79)	5·08 (4·61 to 5·57)	4·89 (4·47 to 5·31)	4·7 (4·36 to 5·05)
Maldives	89·17 (72·47 to 108·47)	84·33 (68·67 to 103·75)	79·04 (64·12 to 97)	73·65 (59·88 to 90·88)	68·02 (55·61 to 83·55)	62·46 (51·09 to 76·68)	57·16 (47·22 to 69·73)	52·12 (43·25 to 63·5)	47·4 (39·48 to 57·21)	42·58 (35·53 to 51·1)	37·86 (31·66 to 45·29)	33·59 (28·22 to 39·99)	29·74 (25·11 to 35·06)	26·39 (22·48 to 30·81)	23·5 (20·2 to 27·08)	20·99 (18·23 to 24·01)
Mauritius	21·17 (19·01 to 23·56)	20·45 (18·44 to 22·81)	19·65 (17·79 to 21·86)	18·96 (17·15 to 21·05)	18·3 (16·56 to 20·21)	17·73 (16·13 to 19·51)	17·14 (15·69 to 18·77)	16·5 (15·2 to 17·99)	15·94 (14·73 to 17·24)	15·43 (14·32 to 16·59)	14·94 (13·94 to 15·95)	14·5 (13·62 to 15·4)	14·14 (13·32 to 14·99)	13·86 (13·07 to 14·65)	13·62 (12·86 to 14·39)	13·38 (12·68 to 14·11)
Myanmar	28·03 (27·65 to 28·4)	27·34 (26·95 to 27·7)	26·69 (26·32 to 27·04)	26·16 (25·79 to 26·5)	25·56 (25·2 to 25·91)	25 (24·63 to 25·33)	24·38 (24·03 to 24·7)	23·85 (23·52 to 24·16)	23·45 (23·12 to 23·76)	23·14 (22·83 to 23·44)	22·81 (22·51 to 23·09)	22·47 (22·18 to 22·76)	22·1 (21·82 to 22·38)	21·68 (21·41 to 21·96)	21·19 (20·93 to 21·48)	20·71 (20·46 to 20·99)
Philippines	15·49 (15·23 to 15·75)	15·2 (14·94 to 15·46)	14·86 (14·6 to 15·11)	14·42 (14·17 to 14·67)	14·06 (13·81 to 14·3)	13·77 (13·51 to 14)	13·45 (13·2 to 13·67)	13·08 (12·85 to 13·3)	12·7 (12·47 to 12·91)	12·29 (12·09 to 12·49)	11·91 (11·71 to 12·09)	11·53 (11·34 to 11·72)	11·23 (11·05 to 11·41)	10·94 (10·77 to 11·12)	10·64 (10·48 to 10·82)	10·36 (10·2 to 10·53)
Sri Lanka	14·32 (13·61 to 15·1)	12·71 (12·08 to 13·38)	12·92 (12·28 to 13·6)	12·55 (11·94 to 13·19)	12·21 (11·61 to 12·82)	11·01 (10·49 to 11·56)	10·15 (9·68 to 10·65)	11·57 (11·04 to 12·12)	11·25 (10·74 to 11·78)	11·09 (10·6 to 11·57)	10·71 (10·25 to 11·17)	10·4 (9·95 to 10·86)	10·36 (9·92 to 10·8)	8·16 (7·82 to 8·5)	7·75 (7·43 to 8·07)	7·65 (7·33 to 7·96)
Seychelles	14·68 (13·48 to 16·05)	13·99 (12·88 to 15·24)	13·28 (12·25 to 14·45)	12·48 (11·49 to 13·53)	11·88 (10·96 to 12·89)	11·46 (10·54 to 12·4)	11·1 (10·21 to 12)	10·76 (9·95 to 11·68)	10·39 (9·59 to 11·29)	9·99 (9·18 to 10·83)	9·58 (8·84 to 10·41)	9·26 (8·52 to 10·04)	8·95 (8·24 to 9·74)	8·7 (8 to 9·44)	8·46 (7·75 to 9·16)	8·31 (7·63 to 9)
Thailand	14·8 (13·2 to 16·55)	14·09 (12·57 to 15·73)	13·4 (11·95 to 14·91)	12·75 (11·35 to 14·2)	12·15 (10·82 to 13·53)	11·61 (10·34 to 12·94)	11·1 (9·88 to 12·41)	10·64 (9·48 to 11·93)	10·18 (9·1 to 11·38)	9·72 (8·7 to 10·89)	9·28 (8·3 to 10·44)	8·85 (7·93 to 9·95)	8·44 (7·56 to 9·47)	8·04 (7·2 to 9·03)	7·64 (6·86 to 8·59)	7·27 (6·53 to 8·16)
Timor-Leste	54·51 (54·02 to 54·98)	51·08 (50·61 to 51·53)	47·7 (47·26 to 48·11)	44·96 (44·55 to 45·35)	42·92 (42·52 to 43·3)	41·01 (40·62 to 41·36)	39·34 (38·97 to 39·68)	38·16 (37·81 to 38·49)	37·18 (36·84 to 37·5)	36·38 (36·06 to 36·68)	35·56 (35·25 to 35·85)	34·49 (34·19 to 34·78)	33·29 (33·01 to 33·57)	32·05 (31·8 to 32·33)	30·97 (30·72 to 31·25)	30·27 (30·03 to 30·54)
Vietnam	23·39 (21·95 to 24·85)	22·64 (21·21 to 24·06)	22·05 (20·64 to 23·39)	21·38 (20·02 to 22·71)	20·71 (19·38 to 22·02)	20·06 (18·75 to 21·29)	19·43 (18·17 to 20·63)	18·83 (17·63 to 19·98)	18·22 (17·05 to 19·33)	17·6 (16·54 to 18·65)	16·96 (15·95 to 17·96)	16·32 (15·35 to 17·3)	15·67 (14·77 to 16·61)	15·06 (14·22 to 15·98)	14·48 (13·68 to 15·38)	13·91 (13·15 to 14·79)
**Oceania**	32·62 (30·12 to 35·55)	32·74 (30·17 to 35·71)	33·01 (30·41 to 35·97)	33·24 (30·57 to 36·17)	33·44 (30·72 to 36·41)	33·67 (30·95 to 36·76)	33·71 (30·95 to 36·75)	33·58 (30·9 to 36·5)	33·26 (30·63 to 36·13)	32·92 (30·35 to 35·79)	32·63 (30·1 to 35·46)	32·27 (29·75 to 35·06)	31·78 (29·24 to 34·54)	31·07 (28·64 to 33·8)	30·35 (27·89 to 32·99)	29·86 (27·4 to 32·44)
American Samoa	10·98 (10·11 to 11·99)	10·62 (9·77 to 11·62)	10·35 (9·51 to 11·29)	10·01 (9·18 to 10·91)	9·65 (8·84 to 10·53)	9·57 (8·78 to 10·47)	9·44 (8·65 to 10·31)	9·38 (8·62 to 10·22)	9·33 (8·58 to 10·15)	9·24 (8·51 to 10·06)	9·24 (8·51 to 10·05)	9·05 (8·33 to 9·84)	8·87 (8·15 to 9·65)	8·76 (8·06 to 9·54)	8·61 (7·9 to 9·37)	8·47 (7·76 to 9·22)
Federated States of Micronesia	22·07 (20·34 to 24·12)	22·16 (20·37 to 24·24)	22·29 (20·48 to 24·31)	21·93 (20·11 to 23·91)	21·52 (19·72 to 23·49)	21·08 (19·33 to 23·05)	20·64 (18·91 to 22·55)	20·16 (18·52 to 21·96)	19·68 (18·09 to 21·41)	19·22 (17·69 to 20·92)	18·71 (17·24 to 20·37)	18·28 (16·83 to 19·89)	17·8 (16·36 to 19·37)	17·28 (15·9 to 18·81)	16·9 (15·51 to 18·4)	16·33 (14·97 to 17·77)
Fiji	11·05 (10·71 to 11·39)	11·07 (10·72 to 11·41)	10·88 (10·53 to 11·2)	10·66 (10·33 to 10·99)	10·55 (10·21 to 10·88)	10·64 (10·29 to 10·96)	10·76 (10·41 to 11·08)	10·73 (10·39 to 11·05)	10·79 (10·45 to 11·12)	10·87 (10·54 to 11·19)	10·78 (10·46 to 11·09)	10·82 (10·51 to 11·14)	10·76 (10·45 to 11·07)	10·99 (10·68 to 11·31)	11·14 (10·83 to 11·48)	11·21 (10·9 to 11·55)
Guam	12·82 (12·38 to 13·31)	12·06 (11·65 to 12·52)	11·58 (11·18 to 12)	11·19 (10·8 to 11·58)	10·86 (10·48 to 11·24)	10·6 (10·23 to 10·97)	10·32 (9·96 to 10·69)	10·2 (9·83 to 10·56)	10·05 (9·71 to 10·42)	10·07 (9·73 to 10·43)	9·96 (9·63 to 10·32)	9·92 (9·6 to 10·27)	9·86 (9·53 to 10·21)	9·79 (9·46 to 10·14)	9·73 (9·39 to 10·06)	9·58 (9·25 to 9·92)
Kiribati	30·19 (27·82 to 32·99)	30·66 (28·19 to 33·53)	31·14 (28·61 to 33·96)	31·63 (29·01 to 34·49)	32·01 (29·34 to 34·93)	32·32 (29·64 to 35·35)	32·36 (29·65 to 35·35)	32·31 (29·67 to 35·19)	31·86 (29·29 to 34·66)	31·28 (28·78 to 34·04)	30·51 (28·11 to 33·21)	29·8 (27·43 to 32·43)	29·16 (26·8 to 31·74)	28·55 (26·28 to 31·09)	27·79 (25·5 to 30·25)	27·19 (24·91 to 29·57)
Marshall Islands	17·15 (15·8 to 18·74)	17·04 (15·67 to 18·64)	17·3 (15·9 to 18·87)	17·44 (16 to 19·01)	17·51 (16·05 to 19·11)	17·6 (16·14 to 19·25)	17·46 (16 to 19·08)	17·07 (15·67 to 18·59)	16·73 (15·38 to 18·2)	16·38 (15·07 to 17·83)	16·15 (14·88 to 17·58)	15·84 (14·58 to 17·24)	15·49 (14·23 to 16·85)	15·04 (13·85 to 16·38)	14·63 (13·43 to 15·93)	14·27 (13·07 to 15·52)
Northern Mariana Islands	7·37 (6·72 to 8·03)	7·17 (6·53 to 7·83)	6·93 (6·3 to 7·56)	6·71 (6·1 to 7·31)	6·52 (5·93 to 7·12)	6·31 (5·73 to 6·89)	6·13 (5·57 to 6·69)	5·96 (5·43 to 6·52)	5·84 (5·33 to 6·39)	5·74 (5·24 to 6·28)	5·65 (5·16 to 6·16)	5·55 (5·07 to 6·05)	5·44 (5 to 5·93)	5·34 (4·91 to 5·81)	5·25 (4·83 to 5·71)	5·17 (4·75 to 5·63)
Papua New Guinea	39·66 (36·54 to 43·31)	39·91 (36·69 to 43·64)	40·37 (37·1 to 44·03)	40·69 (37·32 to 44·35)	40·93 (37·51 to 44·66)	41·14 (37·73 to 45)	41·06 (37·61 to 44·85)	40·75 (37·43 to 44·38)	40·16 (36·91 to 43·69)	39·58 (36·43 to 43·11)	39·12 (36·04 to 42·58)	38·54 (35·48 to 41·94)	37·81 (34·75 to 41·14)	36·75 (33·83 to 40·02)	35·7 (32·75 to 38·86)	34·99 (32·06 to 38·06)
Samoa	12·8 (11·8 to 13·98)	12·63 (11·62 to 13·82)	12·57 (11·55 to 13·7)	12·48 (11·45 to 13·61)	12·35 (11·32 to 13·48)	12·23 (11·22 to 13·38)	12·07 (11·06 to 13·19)	11·84 (10·87 to 12·89)	11·63 (10·69 to 12·65)	11·36 (10·46 to 12·37)	11·1 (10·22 to 12·08)	10·83 (9·97 to 11·78)	10·55 (9·7 to 11·48)	10·24 (9·43 to 11·15)	9·96 (9·14 to 10·84)	9·65 (8·84 to 10·5)
Solomon Islands	23·75 (21·88 to 25·94)	22·87 (21·02 to 25·01)	22·58 (20·75 to 24·63)	22·32 (20·48 to 24·33)	22·02 (20·18 to 24·03)	21·89 (20·07 to 23·94)	21·75 (19·93 to 23·76)	21·49 (19·74 to 23·4)	21·24 (19·52 to 23·1)	20·92 (19·26 to 22·79)	20·56 (18·94 to 22·38)	20·26 (18·65 to 22·05)	19·85 (18·24 to 21·6)	19·44 (17·89 to 21·17)	19·05 (17·48 to 20·74)	18·64 (17·08 to 20·28)
Tonga	14·35 (13·22 to 15·67)	14·38 (13·22 to 15·72)	14·42 (13·25 to 15·73)	14·26 (13·08 to 15·54)	13·97 (12·8 to 15·24)	13·74 (12·6 to 15·02)	13·5 (12·37 to 14·75)	13·27 (12·19 to 14·45)	13·1 (12·04 to 14·25)	12·96 (11·92 to 14·11)	12·83 (11·81 to 13·96)	12·67 (11·67 to 13·79)	12·5 (11·49 to 13·6)	12·49 (11·5 to 13·6)	12·45 (11·43 to 13·55)	12·39 (11·36 to 13·48)
Vanuatu	22·08 (20·34 to 24·11)	21·68 (19·93 to 23·71)	21·46 (19·72 to 23·41)	21·19 (19·44 to 23·1)	20·87 (19·13 to 22·77)	20·56 (18·85 to 22·49)	20·33 (18·62 to 22·21)	20·08 (18·45 to 21·87)	19·86 (18·26 to 21·61)	19·6 (18·04 to 21·34)	19·24 (17·72 to 20·94)	18·86 (17·36 to 20·53)	18·53 (17·03 to 20·16)	18·3 (16·85 to 19·93)	18·12 (16·63 to 19·72)	18 (16·49 to 19·58)
**North Africa and Middle East**	33·64 (29·58 to 38·01)	32·27 (28·4 to 36·5)	30·83 (27·14 to 34·77)	29·3 (25·82 to 32·94)	27·82 (24·56 to 31·32)	26·4 (23·33 to 29·74)	25·12 (22·3 to 28·26)	23·98 (21·36 to 26·93)	23·03 (20·57 to 25·79)	22·27 (19·97 to 24·87)	21·56 (19·42 to 24)	20·97 (18·95 to 23·26)	20·47 (18·55 to 22·69)	20·03 (18·18 to 22·19)	19·66 (17·91 to 21·73)	19·29 (17·62 to 21·29)
**North Africa and Middle East**	33·64 (29·58 to 38·01)	32·27 (28·4 to 36·5)	30·83 (27·14 to 34·77)	29·3 (25·82 to 32·94)	27·82 (24·56 to 31·32)	26·4 (23·33 to 29·74)	25·12 (22·3 to 28·26)	23·98 (21·36 to 26·93)	23·03 (20·57 to 25·79)	22·27 (19·97 to 24·87)	21·56 (19·42 to 24)	20·97 (18·95 to 23·26)	20·47 (18·55 to 22·69)	20·03 (18·18 to 22·19)	19·66 (17·91 to 21·73)	19·29 (17·62 to 21·29)
Afghanistan	41·08 (35·09 to 47·36)	40·48 (34·63 to 46·8)	39·79 (34·08 to 45·98)	38·52 (33 to 44·47)	37·1 (31·76 to 42·84)	35·6 (30·51 to 41·29)	33·97 (29 to 39·4)	32·74 (28 to 37·94)	31·81 (27·19 to 36·86)	30·91 (26·49 to 35·93)	30·18 (25·9 to 35·23)	29·64 (25·5 to 34·66)	29·17 (25·24 to 34·13)	28·96 (25·06 to 33·89)	29·01 (24·98 to 33·95)	28·62 (24·71 to 33·49)
Algeria	39·12 (31·91 to 47·73)	37·06 (30·32 to 45·7)	34·95 (28·73 to 42·59)	32·66 (27 to 39·6)	30·51 (25·49 to 36·47)	28·41 (23·79 to 33·53)	26·73 (22·58 to 31·38)	25·26 (21·76 to 29·25)	24·24 (21·11 to 27·74)	23·5 (20·87 to 26·63)	23·12 (20·56 to 26)	23·01 (20·35 to 25·97)	23·01 (20·35 to 26·11)	23·19 (20·39 to 26·54)	23·43 (20·7 to 26·61)	23·63 (20·84 to 26·71)
Bahrain	15·43 (15·15 to 15·71)	14·25 (13·99 to 14·51)	13·63 (13·38 to 13·88)	13·35 (13·1 to 13·59)	13·12 (12·87 to 13·36)	12·67 (12·42 to 12·9)	12·3 (12·06 to 12·51)	12·47 (12·24 to 12·69)	12·59 (12·35 to 12·8)	12·4 (12·18 to 12·61)	12·2 (11·99 to 12·4)	12·16 (11·95 to 12·37)	12·2 (11·99 to 12·4)	11·95 (11·75 to 12·16)	11·31 (11·13 to 11·52)	10·38 (10·21 to 10·56)
Egypt	44·94 (37·86 to 52·68)	43·17 (36·18 to 50·69)	41·13 (34·45 to 48·16)	38·79 (32·47 to 45·5)	36·42 (30·31 to 42·72)	34 (28·4 to 39·87)	31·55 (26·44 to 37·02)	29·32 (24·56 to 34·31)	27·63 (23·17 to 32·21)	26·46 (22·31 to 30·85)	25·54 (21·57 to 29·85)	24·76 (20·94 to 28·94)	23·92 (20·38 to 27·91)	22·94 (19·64 to 26·83)	21·78 (18·67 to 25·5)	20·49 (17·55 to 24·07)
Iran	26·54 (23·51 to 29·88)	25·57 (22·7 to 28·85)	24·25 (21·53 to 27·37)	22·77 (20·23 to 25·74)	21·44 (19·09 to 24·23)	20·37 (18·19 to 22·98)	19·6 (17·56 to 22·06)	18·82 (16·87 to 21·07)	18·11 (16·3 to 20·26)	17·27 (15·54 to 19·22)	16·32 (14·73 to 18·07)	15·37 (13·93 to 16·92)	14·59 (13·3 to 15·95)	13·93 (12·8 to 15·11)	13·45 (12·46 to 14·49)	13·09 (12·21 to 14·01)
Iraq	21·91 (18·48 to 25·7)	21·03 (17·63 to 24·69)	20·24 (16·92 to 23·68)	19·64 (16·44 to 23)	19·05 (15·93 to 22·36)	18·5 (15·42 to 21·69)	17·98 (14·99 to 21·05)	17·41 (14·58 to 20·37)	16·93 (14·16 to 19·76)	16·53 (13·96 to 19·27)	15·99 (13·55 to 18·62)	16·04 (13·61 to 18·7)	16·04 (13·69 to 18·69)	15·91 (13·62 to 18·61)	15·81 (13·57 to 18·53)	15·81 (13·59 to 18·58)
Jordan	16·81 (15·39 to 18·31)	15·79 (14·42 to 17·18)	14·93 (13·63 to 16·21)	14·2 (12·97 to 15·43)	13·53 (12·33 to 14·73)	12·91 (11·76 to 14·04)	12·36 (11·26 to 13·44)	11·9 (10·86 to 12·91)	11·49 (10·49 to 12·45)	11·2 (10·26 to 12·13)	11 (10·11 to 11·9)	10·82 (9·94 to 11·71)	10·59 (9·76 to 11·48)	10·38 (9·58 to 11·26)	10·18 (9·41 to 11·07)	10 (9·27 to 10·89)
Kuwait	12·67 (10·34 to 15·61)	11·73 (9·57 to 14·49)	11·57 (9·49 to 14·25)	10·41 (8·54 to 12·78)	10·02 (8·26 to 12·28)	9·79 (8·07 to 11·89)	9·03 (7·48 to 10·91)	9·78 (8·16 to 11·71)	9·65 (8·09 to 11·4)	9·23 (7·81 to 10·8)	6·99 (6·01 to 8·07)	5·24 (4·56 to 5·98)	5·99 (5·29 to 6·74)	6·86 (6·15 to 7·66)	6·65 (6·04 to 7·32)	6·25 (5·77 to 6·79)
Lebanon	19·82 (18·7 to 20·96)	18·95 (17·85 to 20·08)	18·45 (17·38 to 19·53)	17·91 (16·84 to 18·97)	17·22 (16·22 to 18·21)	16·47 (15·55 to 17·44)	16·05 (15·13 to 16·99)	15·66 (14·76 to 16·62)	15·42 (14·54 to 16·38)	15·32 (14·49 to 16·3)	15·09 (14·26 to 16·05)	14·61 (13·81 to 15·51)	14·15 (13·4 to 15·03)	13·77 (13·04 to 14·6)	13·37 (12·68 to 14·17)	12·87 (12·2 to 13·62)
Libya	15·08 (12·72 to 17·69)	14·52 (12·17 to 17·05)	13·92 (11·64 to 16·28)	13·38 (11·2 to 15·67)	12·92 (10·8 to 15·16)	12·47 (10·39 to 14·62)	12·09 (10·08 to 14·15)	11·82 (9·9 to 13·83)	11·58 (9·69 to 13·52)	11·36 (9·6 to 13·25)	11·11 (9·41 to 12·94)	10·79 (9·15 to 12·57)	10·49 (8·95 to 12·22)	10·24 (8·77 to 11·98)	10 (8·59 to 11·72)	9·8 (8·42 to 11·52)
Morocco	37·22 (33·99 to 40·89)	35·87 (32·76 to 39·36)	34·39 (31·49 to 37·64)	33·02 (30·41 to 36·05)	31·73 (29·32 to 34·56)	30·43 (28·24 to 33·05)	29·11 (27·05 to 31·46)	27·96 (26·1 to 29·97)	26·75 (25·07 to 28·51)	25·69 (24·24 to 27·19)	24·72 (23·47 to 25·94)	23·75 (22·69 to 24·83)	22·96 (22·07 to 23·83)	22·29 (21·63 to 23·01)	21·58 (21·04 to 22·16)	21·05 (20·61 to 21·53)
Palestine	11·11 (9·84 to 12·58)	10·64 (9·44 to 12·08)	10·13 (8·96 to 11·43)	9·6 (8·48 to 10·78)	9·08 (8·04 to 10·16)	8·65 (7·7 to 9·7)	8·29 (7·35 to 9·3)	8·07 (7·15 to 9·06)	8·01 (7·11 to 9)	7·96 (7·08 to 8·92)	7·86 (6·99 to 8·8)	7·73 (6·91 to 8·65)	7·5 (6·7 to 8·39)	7·27 (6·48 to 8·13)	6·98 (6·2 to 7·8)	6·73 (5·97 to 7·56)
Oman	54·14 (50·62 to 57·81)	49·9 (46·77 to 53·28)	46·35 (43·39 to 49·68)	42·61 (39·79 to 45·68)	39·36 (36·63 to 42·1)	36·05 (33·57 to 38·63)	33·09 (30·91 to 35·6)	30·64 (28·54 to 32·93)	28·73 (26·81 to 30·95)	26·93 (25·17 to 29)	25·01 (23·46 to 26·9)	23·49 (21·96 to 25·25)	22·11 (20·68 to 23·78)	20·63 (19·32 to 22·12)	19·56 (18·35 to 20·94)	18·39 (17·26 to 19·62)
Qatar	7·82 (5·74 to 10·45)	7·45 (5·52 to 9·9)	7·18 (5·37 to 9·41)	6·93 (5·29 to 8·94)	6·68 (5·16 to 8·43)	6·67 (5·22 to 8·25)	6·49 (5·22 to 7·87)	6·3 (5·2 to 7·5)	6·13 (5·17 to 7·13)	5·9 (5·08 to 6·69)	5·78 (5·13 to 6·45)	5·66 (5·06 to 6·25)	5·48 (4·95 to 6·05)	5·37 (4·88 to 5·92)	5·26 (4·76 to 5·76)	5·19 (4·72 to 5·68)
Saudi Arabia	28·78 (21·48 to 37·77)	27·15 (20·28 to 36·05)	26·08 (19·43 to 34·63)	25·02 (18·74 to 33·18)	24·06 (18·19 to 31·98)	23·06 (17·48 to 30·46)	22·07 (16·88 to 29·02)	21·12 (16·21 to 27·85)	20·11 (15·44 to 26·15)	19·71 (15·21 to 25·46)	19·31 (15·01 to 24·85)	18·71 (14·65 to 23·78)	18·01 (14·23 to 22·47)	17·34 (13·83 to 21·41)	16·67 (13·48 to 20·2)	16·17 (13·33 to 19·35)
Sudan	42·71 (38·51 to 47·15)	42·11 (37·98 to 46·56)	41·34 (37·2 to 45·7)	40·73 (36·7 to 45)	40·08 (36·05 to 44·38)	39·31 (35·38 to 43·43)	38·21 (34·47 to 42·19)	36·85 (33·26 to 40·74)	35·43 (32·02 to 39·22)	34·16 (30·91 to 37·98)	33·14 (30·01 to 36·84)	32·21 (29·2 to 35·82)	31·44 (28·47 to 35·05)	30·7 (27·76 to 34·16)	30 (27·15 to 33·34)	29·3 (26·56 to 32·45)
Syria	16·43 (13·84 to 19·26)	15·99 (13·4 to 18·77)	15·57 (13·04 to 18·22)	15·19 (12·71 to 17·81)	14·84 (12·35 to 17·4)	14·47 (12·08 to 16·96)	14·15 (11·85 to 16·6)	13·84 (11·59 to 16·19)	13·46 (11·29 to 15·69)	13·1 (11·04 to 15·27)	12·68 (10·71 to 14·82)	12·19 (10·31 to 14·25)	11·66 (9·93 to 13·6)	11·09 (9·49 to 12·97)	10·52 (9·02 to 12·31)	9·94 (8·52 to 11·68)
Tunisia	15·81 (10·9 to 22·88)	15 (10·39 to 21·65)	14·26 (9·99 to 19·68)	13·51 (9·69 to 18·4)	12·83 (9·35 to 16·93)	12·17 (9·2 to 15·71)	11·64 (9·02 to 14·73)	11·1 (8·84 to 13·51)	10·62 (8·97 to 12·49)	10·24 (9·05 to 11·48)	10·04 (9·18 to 10·99)	9·93 (9·14 to 10·76)	10 (9·3 to 10·71)	10·23 (9·51 to 10·94)	10·34 (9·65 to 11·11)	10·18 (9·51 to 10·96)
Turkey	33·13 (27·93 to 38·85)	31·5 (26·4 to 36·98)	29·96 (25·05 to 35·05)	28·44 (23·81 to 33·31)	26·85 (22·46 to 31·52)	25·27 (21·07 to 29·63)	23·88 (19·91 to 27·95)	22·79 (19·09 to 26·67)	21·91 (18·33 to 25·57)	21·04 (17·78 to 24·54)	20 (16·95 to 23·29)	18·98 (16·1 to 22·12)	18·01 (15·37 to 20·98)	17·21 (14·74 to 20·13)	16·83 (14·45 to 19·73)	16·59 (14·26 to 19·5)
United Arab Emirates	12·41 (11·41 to 13·54)	11·49 (10·55 to 12·6)	10·6 (9·72 to 11·58)	10 (9·15 to 10·94)	9·44 (8·63 to 10·26)	8·86 (8·09 to 9·63)	8·47 (7·78 to 9·22)	8 (7·37 to 8·69)	7·71 (7·07 to 8·41)	7·3 (6·72 to 7·95)	6·89 (6·32 to 7·5)	6·5 (5·97 to 7·06)	6·1 (5·59 to 6·62)	5·79 (5·3 to 6·27)	5·45 (5 to 5·89)	5·09 (4·68 to 5·5)
Yemen	45·86 (42·78 to 49·03)	44·07 (41·01 to 47·13)	42·48 (39·52 to 45·35)	40·96 (38·1 to 43·77)	39·66 (36·86 to 42·43)	38·53 (35·77 to 41·17)	37·53 (34·86 to 40·08)	36·65 (34·1 to 39·13)	35·81 (33·31 to 38·22)	35·08 (32·76 to 37·38)	34·29 (32·05 to 36·52)	33·57 (31·4 to 35·8)	32·91 (30·83 to 35·07)	32·33 (30·36 to 34·5)	31·71 (29·79 to 33·89)	31·04 (29·18 to 33·19)
**South Asia**	59·98 (56·17 to 63·84)	58·09 (54·47 to 61·94)	56·26 (52·69 to 60·03)	54·56 (51·13 to 58·39)	53·04 (49·65 to 56·88)	51·5 (48·23 to 55·29)	49·97 (46·75 to 53·68)	48·47 (45·35 to 51·93)	46·84 (43·88 to 50·23)	45·25 (42·45 to 48·48)	43·74 (41·03 to 46·8)	42·38 (39·81 to 45·27)	41·1 (38·63 to 43·96)	39·87 (37·5 to 42·54)	38·7 (36·42 to 41·26)	37·59 (35·46 to 39·97)
**South Asia**	59·98 (56·17 to 63·84)	58·09 (54·47 to 61·94)	56·26 (52·69 to 60·03)	54·56 (51·13 to 58·39)	53·04 (49·65 to 56·88)	51·5 (48·23 to 55·29)	49·97 (46·75 to 53·68)	48·47 (45·35 to 51·93)	46·84 (43·88 to 50·23)	45·25 (42·45 to 48·48)	43·74 (41·03 to 46·8)	42·38 (39·81 to 45·27)	41·1 (38·63 to 43·96)	39·87 (37·5 to 42·54)	38·7 (36·42 to 41·26)	37·59 (35·46 to 39·97)
Bangladesh	70·71 (66·76 to 74·86)	69·08 (65·15 to 73·25)	67·44 (63·57 to 71·68)	65·74 (61·93 to 69·87)	63·87 (60·2 to 67·9)	62·01 (58·37 to 65·92)	60·17 (56·67 to 64·04)	58·27 (54·98 to 62·01)	56·21 (53·11 to 59·72)	54·02 (51·08 to 57·38)	51·83 (49·11 to 54·9)	49·73 (47·14 to 52·61)	47·64 (45·19 to 50·43)	45·58 (43·31 to 48·22)	43·63 (41·51 to 46·07)	41·72 (39·74 to 44·03)
Bhutan	74·62 (72·09 to 77·17)	72·39 (69·83 to 74·88)	70·05 (67·53 to 72·42)	67·44 (65·04 to 69·75)	64·9 (62·54 to 67·14)	62·46 (60·23 to 64·59)	60·2 (58·08 to 62·25)	57·66 (55·67 to 59·69)	55·12 (53·25 to 57·06)	52·67 (50·88 to 54·52)	50·25 (48·54 to 51·96)	48·07 (46·46 to 49·68)	46·04 (44·56 to 47·58)	44·14 (42·76 to 45·62)	42·25 (40·95 to 43·64)	40·29 (39·01 to 41·63)
India	57·98 (53·87 to 62·14)	55·96 (51·9 to 60·15)	54·01 (50·11 to 58·21)	52·22 (48·47 to 56·41)	50·68 (47·04 to 54·8)	49·14 (45·64 to 53·11)	47·61 (44·13 to 51·64)	46·15 (42·87 to 49·94)	44·56 (41·39 to 48·18)	43·03 (40·02 to 46·5)	41·59 (38·72 to 44·89)	40·32 (37·58 to 43·41)	39·18 (36·53 to 42·25)	38·04 (35·46 to 40·93)	36·98 (34·48 to 39·71)	35·96 (33·62 to 38·52)
Nepal	68·06 (64·51 to 71·63)	66·41 (62·96 to 69·95)	64·65 (61·28 to 68·25)	63·02 (59·73 to 66·7)	61·18 (57·96 to 64·81)	59·25 (56·18 to 62·79)	57·19 (54·13 to 60·77)	55·12 (52·18 to 58·48)	53·06 (50·31 to 56·27)	51·11 (48·47 to 54·11)	49·2 (46·68 to 52)	47·26 (44·88 to 49·94)	45·45 (43·15 to 48·03)	43·68 (41·48 to 46·1)	41·84 (39·82 to 44·06)	40·16 (38·25 to 42·27)
Pakistan	63·33 (61 to 65·57)	61·9 (59·66 to 64·14)	60·44 (58·27 to 62·62)	59·03 (56·91 to 61·15)	57·72 (55·64 to 59·79)	56·28 (54·26 to 58·36)	54·84 (52·81 to 56·86)	53·39 (51·43 to 55·34)	51·85 (49·96 to 53·75)	50·43 (48·62 to 52·31)	49·19 (47·45 to 51·09)	47·99 (46·31 to 49·86)	46·78 (45·2 to 48·58)	45·77 (44·2 to 47·55)	44·81 (43·26 to 46·56)	43·91 (42·43 to 45·63)
**Sub-Saharan Africa**	43·43 (40·97 to 46·23)	42·81 (40·44 to 45·44)	42·2 (39·93 to 44·69)	41·57 (39·39 to 43·98)	40·94 (38·84 to 43·29)	40·3 (38·27 to 42·6)	39·83 (37·77 to 42·13)	39·42 (37·4 to 41·73)	38·99 (36·97 to 41·35)	38·57 (36·57 to 41·01)	38·17 (36·11 to 40·57)	37·83 (35·79 to 40·25)	37·51 (35·49 to 39·91)	37·19 (35·15 to 39·6)	36·86 (34·78 to 39·23)	36·51 (34·46 to 38·88)
**Southern Sub-Saharan Africa**	24·72 (22·25 to 27·47)	24·09 (21·76 to 26·67)	23·48 (21·24 to 25·95)	22·92 (20·75 to 25·24)	22·38 (20·3 to 24·66)	21·76 (19·8 to 24·04)	21·13 (19·26 to 23·32)	20·49 (18·62 to 22·58)	19·82 (18·04 to 21·85)	19·13 (17·39 to 21·1)	18·49 (16·78 to 20·42)	17·91 (16·25 to 19·77)	17·45 (15·79 to 19·28)	17·05 (15·46 to 18·82)	16·73 (15·19 to 18·41)	16·48 (14·97 to 18·13)
Botswana	23·75 (19·68 to 28·1)	23·04 (19·14 to 27·34)	22·38 (18·63 to 26·54)	21·73 (18·07 to 25·76)	21·11 (17·54 to 25·02)	20·43 (17 to 24·33)	19·72 (16·34 to 23·53)	19·03 (15·78 to 22·65)	18·28 (15·16 to 21·75)	17·46 (14·56 to 20·81)	16·75 (13·99 to 20·14)	16·17 (13·56 to 19·49)	15·82 (13·27 to 19·06)	15·53 (13·01 to 18·64)	15·25 (12·76 to 18·38)	15·12 (12·71 to 18·24)
Lesotho	28·81 (26·54 to 31·47)	28·78 (26·49 to 31·38)	28·81 (26·44 to 31·39)	28·93 (26·53 to 31·51)	29·07 (26·65 to 31·67)	29·07 (26·58 to 31·78)	29·05 (26·51 to 31·62)	29·05 (26·51 to 31·59)	28·84 (26·36 to 31·4)	28·4 (25·99 to 30·97)	27·86 (25·51 to 30·42)	27·35 (25·06 to 29·91)	26·85 (24·64 to 29·4)	26·42 (24·25 to 28·83)	26·08 (23·91 to 28·44)	25·85 (23·67 to 28·23)

Appendix Table 17B. Stillbirth rates (per 1,000 live births) by location, 1980-1995

	1980	1981	1982	1983	1984	1985	1986	1987	1988	1989	1990	1991	1992	1993	1994	1995
Namibia	24·45 (19·92 to 29·45)	24·14 (19·6 to 29·15)	23·55 (19·05 to 28·37)	22·74 (18·41 to 27·41)	22·02 (17·84 to 26·66)	21·29 (17·19 to 25·73)	20·7 (16·74 to 24·99)	20·14 (16·42 to 24·42)	19·7 (16·13 to 23·94)	19·15 (15·63 to 23·2)	18·43 (15·11 to 22·23)	17·56 (14·37 to 21·15)	16·63 (13·77 to 20·01)	15·77 (13·12 to 18·95)	14·97 (12·46 to 17·97)	14·27 (11·81 to 17·17)
South Africa	23·95 (21·5 to 26·58)	23·26 (20·84 to 25·83)	22·63 (20·25 to 25·24)	22·12 (19·83 to 24·7)	21·6 (19·37 to 24·21)	20·99 (18·86 to 23·54)	20·35 (18·24 to 22·8)	19·63 (17·63 to 22·02)	18·86 (16·91 to 21·1)	18·07 (16·2 to 20·15)	17·31 (15·6 to 19·3)	16·61 (14·96 to 18·42)	15·99 (14·45 to 17·75)	15·43 (14 to 17·1)	14·93 (13·6 to 16·41)	14·53 (13·28 to 15·94)
Swaziland	22·99 (19·43 to 26·91)	22·02 (18·63 to 25·85)	21·08 (17·75 to 24·72)	20·13 (17 to 23·6)	19·24 (16·2 to 22·62)	18·38 (15·49 to 21·54)	17·51 (14·81 to 20·5)	16·57 (14·03 to 19·44)	15·66 (13·3 to 18·44)	14·74 (12·53 to 17·45)	13·88 (11·81 to 16·43)	13·18 (11·22 to 15·6)	12·62 (10·73 to 15·01)	12·24 (10·4 to 14·51)	11·98 (10·22 to 14·19)	11·83 (10·11 to 13·97)
Zimbabwe	26·65 (22·46 to 31·68)	26·06 (21·85 to 30·9)	25·41 (21·34 to 30·08)	24·65 (20·64 to 29·19)	24 (20·2 to 28·38)	23·27 (19·67 to 27·56)	22·61 (19·09 to 26·7)	22·08 (18·71 to 26·09)	21·6 (18·32 to 25·44)	21·18 (18·02 to 24·93)	20·87 (17·82 to 24·53)	20·63 (17·52 to 24·19)	20·63 (17·58 to 24·06)	20·68 (17·62 to 24)	20·84 (17·72 to 24·18)	21·03 (17·9 to 24·32)
**Western Sub-Saharan Africa**	47·17 (42·94 to 51·74)	46·47 (42·31 to 50·93)	45·78 (41·66 to 50·16)	45·14 (41·09 to 49·38)	44·58 (40·57 to 48·76)	43·84 (39·99 to 48·14)	43·49 (39·52 to 47·96)	43·23 (39·42 to 47·64)	42·96 (39·13 to 47·39)	42·69 (38·91 to 47·25)	42·43 (38·65 to 46·99)	42·21 (38·45 to 46·73)	42·07 (38·35 to 46·57)	41·91 (38·14 to 46·25)	41·74 (38·01 to 46·11)	41·58 (37·79 to 46·1)
Benin	37·72 (34·76 to 40·9)	36·68 (33·84 to 39·73)	35·87 (33·14 to 38·82)	35·14 (32·49 to 38·03)	34·37 (31·8 to 37·27)	33·54 (31·01 to 36·36)	32·78 (30·35 to 35·51)	32·15 (29·74 to 34·86)	31·52 (29·18 to 34·22)	31·06 (28·76 to 33·72)	30·67 (28·42 to 33·31)	30·21 (28·01 to 32·82)	29·71 (27·55 to 32·22)	29·13 (27·07 to 31·57)	28·58 (26·59 to 30·94)	28·01 (26·13 to 30·16)
Burkina Faso	33·59 (30·67 to 36·68)	32·65 (29·74 to 35·73)	31·8 (28·88 to 34·74)	31·06 (28·25 to 33·97)	30·34 (27·68 to 33·11)	29·45 (26·9 to 32·21)	28·56 (26·06 to 31·35)	27·82 (25·41 to 30·6)	27·07 (24·75 to 29·74)	26·41 (24·16 to 28·99)	25·84 (23·7 to 28·38)	25·23 (23·1 to 27·7)	24·72 (22·68 to 27·1)	24·21 (22·2 to 26·54)	23·82 (21·87 to 26·1)	23·43 (21·58 to 25·66)
Cameroon	33·82 (31·64 to 36·28)	32·95 (30·84 to 35·38)	32·07 (30 to 34·32)	31·17 (29·16 to 33·37)	30·23 (28·21 to 32·43)	29·32 (27·35 to 31·46)	28·5 (26·62 to 30·54)	27·84 (26·03 to 29·79)	27·35 (25·57 to 29·28)	26·96 (25·23 to 28·9)	26·65 (24·96 to 28·54)	26·45 (24·77 to 28·29)	26·36 (24·67 to 28·23)	26·37 (24·69 to 28·21)	26·4 (24·69 to 28·24)	26·41 (24·68 to 28·28)
Cape Verde	15·16 (13·61 to 16·83)	15·07 (13·6 to 16·6)	15·17 (13·8 to 16·54)	15·29 (14·02 to 16·63)	15·25 (14·09 to 16·55)	15·26 (14·2 to 16·46)	14·83 (13·83 to 15·93)	14·29 (13·33 to 15·31)	13·78 (12·88 to 14·71)	13·33 (12·5 to 14·21)	12·99 (12·2 to 13·8)	12·78 (12·03 to 13·57)	12·95 (12·18 to 13·74)	13·28 (12·45 to 14·12)	13·65 (12·8 to 14·55)	14 (13·1 to 14·97)
Chad	57·82 (55·94 to 59·73)	57·7 (55·75 to 59·61)	57·46 (55·51 to 59·31)	56·64 (54·72 to 58·48)	55·72 (53·8 to 57·56)	54·52 (52·62 to 56·3)	53·37 (51·51 to 55·1)	52·26 (50·48 to 53·94)	50·96 (49·21 to 52·57)	49·74 (48·13 to 51·31)	48·67 (47·11 to 50·18)	47·64 (46·15 to 49·13)	46·73 (45·31 to 48·21)	46·14 (44·77 to 47·62)	45·57 (44·23 to 47·06)	45·15 (43·83 to 46·66)
Cote d'Ivoire	36·54 (35·61 to 37·45)	35·82 (34·89 to 36·73)	35·2 (34·28 to 36·06)	34·81 (33·89 to 35·67)	34·5 (33·59 to 35·37)	34·13 (33·21 to 34·98)	33·79 (32·89 to 34·63)	33·52 (32·65 to 34·35)	33·23 (32·36 to 34·04)	32·91 (32·1 to 33·7)	32·71 (31·9 to 33·49)	32·53 (31·73 to 33·32)	32·39 (31·63 to 33·18)	32·29 (31·55 to 33·08)	32·2 (31·47 to 33·01)	32 (31·28 to 32·81)
The Gambia	48·26 (46·71 to 49·81)	47·17 (45·61 to 48·69)	45·83 (44·3 to 47·27)	44·64 (43·15 to 46·06)	43·82 (42·34 to 45·24)	43·2 (41·71 to 44·56)	42·57 (41·12 to 43·92)	41·91 (40·51 to 43·23)	41·21 (39·83 to 42·5)	40·62 (39·34 to 41·86)	40·02 (38·77 to 41·24)	39·46 (38·23 to 40·68)	38·85 (37·68 to 40·05)	38·2 (37·08 to 39·4)	37·57 (36·48 to 38·78)	36·89 (35·83 to 38·09)
Ghana	30·76 (27·25 to 34·25)	30·2 (26·75 to 33·67)	29·78 (26·53 to 33·23)	29·38 (26·31 to 32·61)	28·8 (25·9 to 31·96)	28·12 (25·39 to 31·14)	27·41 (24·88 to 30·2)	26·75 (24·42 to 29·33)	26·03 (23·84 to 28·42)	25·35 (23·2 to 27·55)	24·76 (22·79 to 26·86)	24·24 (22·44 to 26·19)	23·85 (22·18 to 25·69)	23·58 (21·99 to 25·33)	23·43 (21·92 to 25·09)	23·36 (21·97 to 24·83)
Guinea	42·9 (41·38 to 44·42)	42·29 (40·75 to 43·79)	41·68 (40·15 to 43·12)	41·07 (39·56 to 42·51)	40·46 (38·96 to 41·9)	39·74 (38·24 to 41·12)	38·99 (37·53 to 40·34)	38·2 (36·8 to 39·52)	37·32 (35·95 to 38·61)	36·44 (35·18 to 37·67)	35·57 (34·34 to 36·76)	34·73 (33·54 to 35·91)	33·98 (32·85 to 35·14)	33·21 (32·14 to 34·36)	32·43 (31·39 to 33·58)	31·61 (30·61 to 32·74)
Guinea-Bissau	56·24 (52·71 to 60·26)	55·91 (52·32 to 60·03)	55·4 (51·77 to 59·32)	54·87 (51·23 to 58·75)	54·29 (50·67 to 58·18)	53·71 (50·21 to 57·64)	52·76 (49·23 to 56·54)	51·88 (48·5 to 55·51)	51·04 (47·73 to 54·56)	50·19 (47·03 to 53·67)	49·34 (46·24 to 52·77)	48·49 (45·37 to 51·87)	47·79 (44·72 to 51·15)	47·18 (44·19 to 50·48)	46·56 (43·48 to 49·75)	45·83 (42·77 to 48·99)
Liberia	54·62 (51·2 to 58·53)	53·58 (50·13 to 57·52)	52·52 (49·08 to 56·24)	51·28 (47·88 to 54·91)	50·1 (46·76 to 53·69)	48·83 (45·65 to 52·4)	47·52 (44·33 to 50·92)	46·11 (43·11 to 49·34)	44·52 (41·63 to 47·58)	42·98 (40·27 to 45·96)	42·14 (39·49 to 45·07)	41·47 (38·8 to 44·37)	41·01 (38·37 to 43·89)	40·86 (38·27 to 43·72)	40·94 (38·22 to 43·74)	40·98 (38·25 to 43·81)
Mali	62·97 (58·91 to 67·56)	61·76 (57·79 to 66·31)	60·68 (56·78 to 64·95)	59·72 (55·87 to 63·93)	58·87 (54·94 to 63·15)	57·87 (54 to 62·1)	56·9 (53·14 to 60·99)	56·05 (52·41 to 59·98)	55·48 (51·87 to 59·4)	54·78 (51·25 to 58·72)	53·94 (50·52 to 57·76)	52·99 (49·63 to 56·68)	52·27 (48·91 to 55·98)	51·43 (48·17 to 55·03)	50·58 (47·3 to 54·11)	49·84 (46·58 to 53·38)
Mauritania	34·04 (33·11 to 34·96)	33·41 (32·48 to 34·32)	32·88 (31·95 to 33·75)	32·33 (31·42 to 33·19)	31·88 (30·97 to 32·75)	31·31 (30·4 to 32·15)	30·77 (29·89 to 31·59)	30·17 (29·32 to 30·97)	29·66 (28·82 to 30·44)	29·22 (28·45 to 29·98)	28·82 (28·06 to 29·56)	28·51 (27·76 to 29·25)	28·23 (27·51 to 28·96)	27·93 (27·24 to 28·67)	27·69 (27·02 to 28·45)	27·38 (26·72 to 28·13)
Niger	50·4 (48·79 to 52·19)	49·87 (48·27 to 51·65)	49·38 (47·77 to 51·04)	48·87 (47·28 to 50·48)	48·78 (47·18 to 50·37)	48·47 (46·9 to 50·05)	48·03 (46·45 to 49·62)	47·55 (45·98 to 49·15)	46·88 (45·36 to 48·48)	46·27 (44·79 to 47·82)	45·7 (44·22 to 47·2)	45·07 (43·66 to 46·56)	44·47 (43·05 to 45·96)	43·75 (42·33 to 45·22)	42·88 (41·47 to 44·31)	42·02 (40·62 to 43·47)
Nigeria	52·58 (45·85 to 59·89)	52·01 (45·3 to 59·26)	51·42 (44·68 to 58·72)	50·92 (44·23 to 58·14)	50·58 (43·96 to 58)	49·92 (43·38 to 57·32)	50·05 (43·54 to 57·26)	50·28 (43·64 to 57·76)	50·43 (43·65 to 58·29)	50·49 (43·72 to 58·56)	50·47 (43·79 to 58·78)	50·54 (43·93 to 58·61)	50·66 (44·09 to 58·8)	50·71 (44·24 to 58·68)	50·72 (44·22 to 58·74)	50·73 (44·18 to 58·59)
Sao Tome and Principe	17·5 (16·4 to 18·75)	17·85 (16·71 to 19·17)	18·2 (17 to 19·49)	18·57 (17·34 to 19·89)	18·93 (17·66 to 20·28)	18·56 (17·35 to 19·92)	18 (16·8 to 19·29)	17·47 (16·33 to 18·69)	17·18 (16·06 to 18·36)	17·05 (15·97 to 18·23)	17·02 (15·95 to 18·2)	16·94 (15·85 to 18·12)	16·87 (15·78 to 18·05)	16·78 (15·72 to 17·96)	16·74 (15·63 to 17·88)	16·68 (15·57 to 17·83)
Senegal	28·16 (22·46 to 34·52)	27·65 (21·94 to 34·21)	27·09 (21·54 to 33·72)	26·59 (21·19 to 33·23)	26·25 (20·92 to 32·98)	25·92 (20·74 to 32·56)	25·63 (20·36 to 32·51)	25·31 (20·22 to 31·9)	25·07 (20·05 to 31·52)	24·92 (20·01 to 31·24)	24·85 (20·01 to 31·07)	24·86 (20·05 to 30·91)	24·95 (20·25 to 31·05)	25·13 (20·37 to 31·12)	25·36 (20·6 to 31·22)	25·56 (20·76 to 31·36)
Sierra Leone	56·41 (52·88 to 60·45)	56·11 (52·5 to 60·24)	55·68 (52·03 to 59·62)	55·27 (51·61 to 59·18)	54·78 (51·13 to 58·71)	54·35 (50·81 to 58·32)	53·9 (50·29 to 57·75)	53·24 (49·78 to 56·97)	52·48 (49·07 to 56·09)	51·62 (48·36 to 55·19)	50·6 (47·42 to 54·12)	49·64 (46·45 to 53·11)	49·14 (45·97 to 52·59)	48·43 (45·36 to 51·82)	47·52 (44·37 to 50·77)	46·84 (43·72 to 50·07)
Togo	41·49 (39·28 to 43·71)	40·66 (38·44 to 42·85)	40 (37·77 to 42·23)	39·53 (37·31 to 41·78)	39·02 (36·87 to 41·26)	38·41 (36·32 to 40·62)	37·82 (35·69 to 40·13)	37·35 (35·34 to 39·66)	36·75 (34·78 to 39·07)	36·15 (34·2 to 38·43)	35·49 (33·61 to 37·67)	34·89 (33·03 to 37·05)	34·34 (32·49 to 36·4)	34·11 (32·31 to 36·13)	33·7 (31·95 to 35·69)	33·16 (31·49 to 35)
**Eastern Sub-Saharan Africa**	41·94 (40·51 to 43·45)	41·45 (40·03 to 42·92)	40·97 (39·52 to 42·39)	40·44 (38·98 to 41·8)	39·9 (38·48 to 41·21)	39·42 (38·07 to 40·74)	38·88 (37·57 to 40·17)	38·25 (36·97 to 39·55)	37·57 (36·37 to 38·83)	36·89 (35·76 to 38·1)	36·25 (35·13 to 37·42)	35·72 (34·61 to 36·87)	35·28 (34·19 to 36·45)	34·77 (33·71 to 35·93)	34·25 (33·2 to 35·42)	33·67 (32·63 to 34·86)
Burundi	32·59 (32·08 to 33·09)	31·76 (31·24 to 32·25)	30·98 (30·47 to 31·44)	30·2 (29·71 to 30·66)	29·49 (29·01 to 29·95)	28·96 (28·48 to 29·4)	28·62 (28·15 to 29·05)	28·47 (28·01 to 28·91)	28·42 (27·96 to 28·85)	28·47 (28·03 to 28·89)	28·59 (28·15 to 29·01)	28·76 (28·32 to 29·19)	28·97 (28·55 to 29·4)	29·28 (28·87 to 29·72)	29·41 (28·99 to 29·86)	29·42 (29·01 to 29·88)
Comoros	62·85 (59·22 to 66·53)	60·95 (57·33 to 64·55)	58·88 (55·36 to 62·25)	56·76 (53·36 to 60·05)	54·68 (51·38 to 57·92)	52·64 (49·41 to 55·68)	50·69 (47·61 to 53·62)	48·97 (46·05 to 51·79)	47·4 (44·57 to 50·1)	46·13 (43·52 to 48·71)	45·1 (42·57 to 47·61)	44·2 (41·73 to 46·69)	43·15 (40·81 to 45·58)	42·1 (39·9 to 44·52)	41·26 (39·12 to 43·71)	40·23 (38·16 to 42·62)
Djibouti	40 (39·19 to 40·8)	39·95 (39·11 to 40·75)	39·81 (38·97 to 40·58)	39·91 (39·07 to 40·7)	39·72 (38·88 to 40·53)	39·54 (38·68 to 40·32)	39·29 (38·45 to 40·07)	38·98 (38·17 to 39·75)	38·74 (37·92 to 39·49)	38·47 (37·71 to 39·21)	38·36 (37·6 to 39·09)	38·32 (37·57 to 39·06)	38·21 (37·49 to 38·95)	37·97 (37·27 to 38·71)	37·87 (37·18 to 38·64)	37·71 (37·03 to 38·48)
Eritrea	38·46 (36·24 to 40·71)	37·93 (35·68 to 40·17)	37·36 (35·13 to 39·5)	36·8 (34·59 to 38·93)	36·16 (33·98 to 38·3)	35·54 (33·35 to 37·59)	34·88 (32·76 to 36·9)	34·2 (32·16 to 36·17)	33·48 (31·48 to 35·38)	32·72 (30·87 to 34·55)	32 (30·2 to 33·78)	31·1 (29·36 to 32·86)	30·05 (28·43 to 31·75)	28·96 (27·44 to 30·62)	27·69 (26·25 to 29·33)	26·48 (25·12 to 28·06)
Ethiopia	41·31 (40·2 to 42·41)	40·83 (39·7 to 41·92)	40·29 (39·18 to 41·34)	39·5 (38·4 to 40·54)	38·71 (37·62 to 39·75)	38·25 (37·15 to 39·26)	37·65 (36·59 to 38·64)	36·98 (35·95 to 37·95)	36·42 (35·41 to 37·37)	35·83 (34·89 to 36·74)	35·14 (34·23 to 36·03)	34·62 (33·73 to 35·52)	34·21 (33·35 to 35·09)	33·62 (32·8 to 34·5)	32·99 (32·19 to 33·88)	32·19 (31·42 to 33·05)
Kenya	36·5 (34·38 to 38·68)	35·94 (33·84 to 38·1)	35·42 (33·32 to 37·58)	35 (32·91 to 37·12)	34·59 (32·53 to 36·77)	34·21 (32·18 to 36·38)	33·8 (31·8 to 35·9)	33·45 (31·43 to 35·59)	33·09 (31·05 to 35·31)	32·77 (30·76 to 35·01)	32·58 (30·62 to 34·89)	32·44 (30·5 to 34·67)	32·49 (30·56 to 34·74)	32·63 (30·72 to 34·85)	32·69 (30·76 to 34·93)	32·84 (30·89 to 35·06)
Madagascar	36·72 (34·6 to 38·87)	37·16 (34·95 to 39·35)	37·67 (35·41 to 39·82)	37·92 (35·65 to 40·12)	38·02 (35·73 to 40·27)	37·92 (35·59 to 40·11)	37·55 (35·27 to 39·72)	36·85 (34·66 to 38·98)	35·86 (33·72 to 37·9)	34·83 (32·87 to 36·79)	34·03 (32·11 to 35·92)	33·59 (31·71 to 35·49)	33·31 (31·5 to 35·19)	33·06 (31·33 to 34·96)	32·61 (30·92 to 34·55)	31·91 (30·27 to 33·81)
Malawi	45·65 (44·54 to 46·76)	44·43 (43·31 to 45·51)	43·47 (42·37 to 44·49)	42·82 (41·73 to 43·84)	42·33 (41·25 to 43·37)	41·81 (40·72 to 42·82)	41·31 (40·25 to 42·3)	40·63 (39·6 to 41·59)	39·75 (38·74 to 40·69)	38·68 (37·76 to 39·57)	37·56 (36·66 to 38·42)	36·34 (35·48 to 37·19)	35·3 (34·5 to 36·12)	34·12 (33·37 to 34·93)	33·11 (32·38 to 33·92)	32·14 (31·45 to 32·93)
Mozambique	55·76 (52·49 to 59·01)	55·21 (51·93 to 58·46)	54·81 (51·54 to 57·96)	54·71 (51·46 to 57·93)	54·56 (51·19 to 57·75)	54·49 (51·21 to 57·7)	54·27 (50·99 to 57·4)	53·9 (50·69 to 57·01)	53·36 (50·24 to 56·4)	52·65 (49·61 to 55·59)	51·92 (48·96 to 54·88)	51 (48·15 to 53·91)	50·29 (47·57 to 53·16)	49·36 (46·77 to 52·19)	48·24 (45·72 to 51·04)	47·17 (44·74 to 49·98)
Rwanda	40·45 (36·97 to 44·19)	38·11 (34·83 to 41·6)	36·13 (33 to 39·34)	34·56 (31·52 to 37·65)	33·49 (30·56 to 36·48)	32·71 (29·86 to 35·65)	31·89 (29·08 to 34·83)	31·11 (28·4 to 34·07)	30·5 (27·93 to 33·33)	30·19 (27·64 to 33·04)	30·13 (27·6 to 33·09)	30·35 (27·82 to 33·3)	30·44 (27·91 to 33·38)	30·37 (27·85 to 33·32)	30·81 (28·27 to 33·81)	30·8 (28·29 to 33·74)
Somalia	44·55 (41·94 to 47·15)	44·57 (41·92 to 47·2)	44·5 (41·85 to 47·06)	44·33 (41·7 to 46·94)	44·31 (41·57 to 46·9)	44·34 (41·67 to 46·95)	43·91 (41·25 to 46·43)	43·29 (40·71 to 45·79)	42·82 (40·32 to 45·26)	42·23 (39·79 to 44·59)	41·77 (39·39 to 44·16)	41·49 (39·17 to 43·85)	41·24 (39 to 43·59)	40·81 (38·67 to 43·15)	40·51 (38·4 to 42·87)	40·19 (38·12 to 42·58)
South Sudan	85·63 (83·65 to 87·6)	85·02 (82·99 to 86·98)	84·43 (82·4 to 86·33)	83·76 (81·74 to 85·66)	82·78 (80·77 to 84·7)	81·67 (79·65 to 83·52)	80·51 (78·54 to 82·33)	79·14 (77·24 to 80·93)	77·59 (75·72 to 79·33)	76·03 (74·31 to 77·7)	74·49 (72·8 to 76·11)	72·86 (71·23 to 74·47)	71·18 (69·64 to 72·75)	69·46 (68 to 71·02)	68·03 (66·61 to 69·6)	66·83 (65·45 to 68·38)
Tanzania	35·48 (34·43 to 36·53)	35·17 (34·1 to 36·22)	34·85 (33·78 to 35·86)	34·46 (33·4 to 35·47)	33·97 (32·91 to 34·98)	33·35 (32·3 to 34·33)	32·96 (31·92 to 33·92)	32·6 (31·6 to 33·55)	32·06 (31·07 to 32·98)	31·56 (30·65 to 32·45)	31·03 (30·14 to 31·9)	30·53 (29·65 to 31·39)	30·09 (29·26 to 30·95)	29·68 (28·88 to 30·53)	29·27 (28·49 to 30·15)	28·83 (28·07 to 29·69)
Uganda	37·21 (36·79 to 37·63)	37·06 (36·62 to 37·47)	36·64 (36·2 to 37·04)	36 (35·57 to 36·4)	35·38 (34·96 to 35·78)	34·79 (34·36 to 35·18)	34·18 (33·77 to 34·56)	33·51 (33·11 to 33·88)	32·78 (32·39 to 33·14)	32·06 (31·7 to 32·4)	31·4 (31·05 to 31·74)	30·87 (30·53 to 31·2)	30·38 (30·06 to 30·71)	29·91 (29·6 to 30·24)	29·36 (29·06 to 29·69)	28·84 (28·55 to 29·17)
Zambia	33·2 (31·25 to 35·14)	32·94 (30·98 to 34·88)	32·87 (30·91 to 34·76)	32·96 (31 to 34·9)	33·12 (31·08 to 35·06)	33·29 (31·29 to 35·26)	33·49 (31·46 to 35·42)	33·34 (31·35 to 35·26)	32·9 (30·98 to 34·77)	32·6 (30·72 to 34·43)	32·45 (30·6 to 34·3)	32·36 (30·55 to 34·21)	32·37 (30·61 to 34·21)	32·21 (30·52 to 34·06)	31·94 (30·27 to 33·8)	31·47 (29·85 to 33·34)
**Central Sub-Saharan Africa**	47·26 (45·96 to 48·63)	46·58 (45·26 to 47·92)	45·81 (44·5 to 47·16)	44·91 (43·62 to 46·23)	43·64 (42·33 to 44·97)	42·8 (41·51 to 44·11)	42·4 (41·13 to 43·73)	42·3 (41·06 to 43·64)	42·29 (41·02 to 43·66)	42·3 (41·01 to 43·67)	42·29 (41 to 43·69)	42·05 (40·75 to 43·44)	41·43 (40·17 to 42·78)	40·9 (39·65 to 42·23)	40·4 (39·17 to 41·71)	39·84 (38·63 to 41·13)
Angola	53·67 (51·35 to 56·25)	53·42 (51·1 to 56·01)	53·34 (50·99 to 55·78)	52·82 (50·5 to 55·18)	52·05 (49·75 to 54·36)	51·29 (49·05 to 53·58)	51·27 (49·01 to 53·59)	51·48 (49·19 to 53·83)	51·66 (49·4 to 54·06)	51·76 (49·53 to 54·11)	51·35 (49·12 to 53·65)	50·45 (48·33 to 52·73)	49·11 (46·99 to 51·35)	47·84 (45·75 to 50·03)	46·27 (44·22 to 48·37)	44·67 (42·66 to 46·77)
Central African Republic	58·34 (54·95 to 62·18)	57·62 (54·16 to 61·57)	57·22 (53·74 to 61·1)	57·22 (53·76 to 61·03)	57·08 (53·64 to 60·89)	56·95 (53·41 to 60·74)	56·96 (53·31 to 60·8)	57·12 (53·56 to 60·95)	57·01 (53·49 to 60·81)	56·54 (53·03 to 60·29)	55·91 (52·5 to 59·57)	55·02 (51·64 to 58·64)	54·31 (51·12 to 57·86)	53·93 (50·73 to 57·44)	53·7 (50·46 to 57·17)	53·63 (50·35 to 57·09)
Congo	33·7 (31·73 to 35·95)	32·49 (30·54 to 34·72)	31·35 (29·44 to 33·42)	30·3 (28·42 to 32·29)	29·23 (27·41 to 31·18)	28·41 (26·67 to 30·33)	27·84 (26·1 to 29·7)	27·39 (25·73 to 29·18)	26·96 (25·33 to 28·7)	26·51 (24·95 to 28·22)	26·21 (24·67 to 27·91)	25·98 (24·42 to 27·66)	25·85 (24·29 to 27·54)	25·78 (24·25 to 27·46)	25·85 (24·26 to 27·52)	25·93 (24·31 to 27·59)
Democratic Republic of the Congo	45·3 (44·3 to 46·29)	44·47 (43·46 to 45·45)	43·42 (42·42 to 44·34)	42·25 (41·28 to 43·17)	40·63 (39·69 to 41·53)	39·66 (38·73 to 40·51)	39·08 (38·17 to 39·92)	38·88 (37·99 to 39·71)	38·87 (37·97 to 39·69)	38·93 (38·09 to 39·75)	39·16 (38·32 to 39·96)	39·22 (38·39 to 40·04)	38·84 (38·04 to 39·65)	38·54 (37·77 to 39·36)	38·36 (37·6 to 39·2)	38·12 (37·37 to 38·95)
Equatorial Guinea	56·13 (52·86 to 59·83)	55·05 (51·75 to 58·83)	54·35 (51·04 to 58·03)	53·68 (50·43 to 57·26)	53·16 (49·97 to 56·72)	53·07 (49·77 to 56·61)	53·29 (49·88 to 56·88)	53·63 (50·29 to 57·22)	53·81 (50·49 to 57·4)	54·01 (50·66 to 57·6)	54·48 (51·16 to 58·06)	55·12 (51·73 to 58·74)	55·06 (51·83 to 58·66)	54·81 (51·57 to 58·39)	54·56 (51·27 to 58·1)	53·91 (50·61 to 57·39)
Gabon	37·16 (34·98 to 39·64)	35·98 (33·81 to 38·44)	34·97 (32·84 to 37·28)	33·94 (31·84 to 36·18)	32·85 (30·8 to 35·04)	31·82 (29·87 to 33·97)	30·87 (28·94 to 32·93)	30·21 (28·37 to 32·18)	29·65 (27·85 to 31·55)	28·93 (27·22 to 30·8)	28·19 (26·54 to 30·02)	27·45 (25·81 to 29·23)	26·83 (25·22 to 28·59)	26·26 (24·71 to 27·97)	25·72 (24·14 to 27·39)	25·19 (23·62 to 26·81)

Appendix Table 17C. Stillbirth rates (per 1,000 live births) by location, 1996-2005	1996	1997	1998	1999	2000	2001	2002	2003	2004	2005
**Global**	23·03 (21·71 to 24·46)	22·59 (21·31 to 23·98)	22·17 (20·96 to 23·51)	21·76 (20·61 to 23·04)	21·37 (20·26 to 22·57)	20·99 (19·92 to 22·12)	20·6 (19·57 to 21·69)	20·21 (19·22 to 21·27)	19·79 (18·86 to 20·82)	19·28 (18·39 to 20·27)
**High SDI**	3·95 (3·83 to 4·07)	3·82 (3·72 to 3·94)	3·72 (3·61 to 3·82)	3·62 (3·53 to 3·72)	3·51 (3·43 to 3·6)	3·45 (3·37 to 3·53)	3·34 (3·27 to 3·41)	3·25 (3·18 to 3·32)	3·16 (3·09 to 3·23)	3·08 (3·01 to 3·15)
**High-middle SDI**	11·42 (10·62 to 12·27)	11·15 (10·41 to 11·95)	10·86 (10·15 to 11·58)	10·56 (9·92 to 11·23)	10·28 (9·7 to 10·87)	10·05 (9·52 to 10·62)	9·85 (9·36 to 10·37)	9·68 (9·23 to 10·16)	9·54 (9·11 to 9·99)	9·34 (8·93 to 9·78)
**Middle SDI**	17·29 (16·25 to 18·46)	16·78 (15·79 to 17·89)	16·34 (15·41 to 17·36)	15·93 (15·05 to 16·87)	15·53 (14·71 to 16·38)	15·16 (14·38 to 15·98)	14·82 (14·08 to 15·6)	14·53 (13·84 to 15·27)	14·25 (13·59 to 14·98)	13·88 (13·26 to 14·59)
**Low-middle SDI**	38·42 (35·99 to 41·1)	37·48 (35·16 to 40·06)	36·58 (34·36 to 39·04)	35·74 (33·63 to 38·13)	34·98 (32·96 to 37·24)	34·26 (32·28 to 36·38)	33·57 (31·67 to 35·59)	32·93 (31·09 to 34·88)	32·18 (30·45 to 34·07)	31·3 (29·62 to 33·09)
**Low SDI**	34·94 (33·68 to 36·35)	34·27 (33·04 to 35·65)	33·6 (32·39 to 34·94)	32·85 (31·69 to 34·15)	32·05 (30·93 to 33·29)	31·18 (30·07 to 32·39)	30·27 (29·2 to 31·44)	29·37 (28·31 to 30·52)	28·38 (27·36 to 29·5)	27·43 (26·45 to 28·48)
**High-income**	3·63 (3·53 to 3·73)	3·53 (3·43 to 3·64)	3·45 (3·35 to 3·55)	3·36 (3·28 to 3·46)	3·3 (3·22 to 3·38)	3·26 (3·18 to 3·34)	3·2 (3·12 to 3·29)	3·16 (3·08 to 3·25)	3·11 (3·03 to 3·19)	3·08 (2·99 to 3·16)
**High-income North America**	3·29 (3·14 to 3·44)	3·23 (3·09 to 3·39)	3·2 (3·05 to 3·35)	3·15 (3·01 to 3·29)	3·07 (2·95 to 3·22)	3·01 (2·88 to 3·15)	2·97 (2·84 to 3·11)	2·93 (2·79 to 3·07)	2·88 (2·75 to 3·01)	2·88 (2·75 to 3·01)
Canada	2·37 (2·33 to 2·4)	2·33 (2·29 to 2·36)	2·27 (2·23 to 2·3)	2·24 (2·21 to 2·27)	2·2 (2·17 to 2·23)	2·2 (2·16 to 2·23)	2·19 (2·16 to 2·22)	2·18 (2·15 to 2·21)	2·16 (2·13 to 2·19)	2·18 (2·15 to 2·21)
Greenland	9·18 (8·62 to 9·79)	8·82 (8·27 to 9·38)	8·18 (7·67 to 8·72)	7·81 (7·31 to 8·33)	7·33 (6·87 to 7·84)	7·31 (6·84 to 7·82)	7·2 (6·73 to 7·69)	6·97 (6·52 to 7·45)	6·78 (6·34 to 7·24)	6·75 (6·29 to 7·22)
United States	3·37 (3·21 to 3·53)	3·31 (3·15 to 3·48)	3·28 (3·12 to 3·44)	3·22 (3·07 to 3·37)	3·14 (3·01 to 3·3)	3·08 (2·94 to 3·23)	3·04 (2·89 to 3·19)	2·99 (2·84 to 3·15)	2·94 (2·8 to 3·08)	2·93 (2·8 to 3·08)
**Australasia**	3·06 (2·83 to 3·3)	2·97 (2·76 to 3·21)	2·88 (2·67 to 3·09)	2·88 (2·68 to 3·1)	2·83 (2·64 to 3·05)	2·84 (2·64 to 3·05)	2·88 (2·68 to 3·08)	2·95 (2·74 to 3·17)	3·04 (2·82 to 3·28)	3·16 (2·93 to 3·4)
Australia	2·97 (2·71 to 3·25)	2·88 (2·63 to 3·16)	2·78 (2·53 to 3·04)	2·78 (2·54 to 3·04)	2·72 (2·5 to 2·97)	2·72 (2·49 to 2·97)	2·77 (2·53 to 3·01)	2·85 (2·6 to 3·1)	2·95 (2·69 to 3·24)	3·11 (2·82 to 3·4)
New Zealand	3·42 (3·29 to 3·55)	3·39 (3·26 to 3·52)	3·33 (3·21 to 3·45)	3·32 (3·2 to 3·44)	3·35 (3·22 to 3·47)	3·36 (3·23 to 3·48)	3·4 (3·26 to 3·52)	3·39 (3·26 to 3·52)	3·4 (3·27 to 3·52)	3·36 (3·24 to 3·49)
**High-income Asia Pacific**	3·38 (3·22 to 3·55)	3·18 (3·03 to 3·35)	3·06 (2·92 to 3·23)	2·9 (2·77 to 3·05)	2·81 (2·68 to 2·94)	2·74 (2·62 to 2·85)	2·67 (2·56 to 2·79)	2·63 (2·52 to 2·74)	2·53 (2·42 to 2·64)	2·41 (2·31 to 2·51)
Brunei	5·03 (4·69 to 5·37)	4·93 (4·63 to 5·25)	4·83 (4·56 to 5·13)	4·71 (4·46 to 4·99)	4·73 (4·48 to 5)	4·62 (4·39 to 4·87)	4·52 (4·28 to 4·76)	4·43 (4·21 to 4·67)	4·27 (4·05 to 4·49)	4·09 (3·88 to 4·3)
Japan	3·23 (3·05 to 3·42)	3·01 (2·85 to 3·2)	2·92 (2·75 to 3·09)	2·72 (2·56 to 2·88)	2·57 (2·43 to 2·72)	2·45 (2·31 to 2·59)	2·39 (2·26 to 2·53)	2·35 (2·22 to 2·49)	2·3 (2·17 to 2·44)	2·25 (2·12 to 2·39)
Singapore	3·38 (3·26 to 3·5)	3·23 (3·12 to 3·35)	3·15 (3·04 to 3·26)	3·01 (2·91 to 3·12)	2·88 (2·78 to 2·98)	2·77 (2·67 to 2·87)	2·7 (2·6 to 2·79)	2·61 (2·52 to 2·71)	2·53 (2·43 to 2·62)	2·48 (2·39 to 2·57)
South Korea	3·65 (3·41 to 3·93)	3·46 (3·23 to 3·71)	3·31 (3·09 to 3·55)	3·23 (3·02 to 3·46)	3·27 (3·07 to 3·49)	3·38 (3·17 to 3·59)	3·34 (3·13 to 3·55)	3·28 (3·08 to 3·46)	3·09 (2·91 to 3·25)	2·76 (2·61 to 2·9)
**Western Europe**	3·37 (3·28 to 3·47)	3·3 (3·21 to 3·38)	3·18 (3·11 to 3·27)	3·09 (3·01 to 3·16)	3·02 (2·95 to 3·1)	2·98 (2·92 to 3·05)	2·86 (2·8 to 2·93)	2·8 (2·74 to 2·86)	2·72 (2·66 to 2·78)	2·63 (2·57 to 2·68)
Andorra	3·24 (3·21 to 3·27)	3·04 (3·02 to 3·07)	2·91 (2·89 to 2·94)	2·8 (2·78 to 2·83)	2·71 (2·68 to 2·73)	2·61 (2·58 to 2·63)	2·46 (2·44 to 2·48)	2·6 (2·58 to 2·62)	2·7 (2·68 to 2·72)	2·78 (2·75 to 2·8)
Austria	1·97 (1·92 to 2·04)	1·89 (1·84 to 1·95)	1·88 (1·83 to 1·93)	1·81 (1·77 to 1·86)	1·83 (1·78 to 1·88)	1·82 (1·78 to 1·87)	1·75 (1·7 to 1·79)	1·79 (1·75 to 1·83)	1·77 (1·73 to 1·82)	1·75 (1·71 to 1·79)
Belgium	3·71 (3·36 to 4·08)	3·46 (3·14 to 3·79)	3·22 (2·92 to 3·52)	2·97 (2·71 to 3·26)	2·81 (2·56 to 3·07)	2·65 (2·43 to 2·88)	2·5 (2·29 to 2·72)	2·39 (2·19 to 2·61)	2·27 (2·08 to 2·48)	2·21 (2·02 to 2·43)
Cyprus	4·32 (4·14 to 4·52)	4·2 (4·02 to 4·39)	4·07 (3·9 to 4·25)	3·9 (3·73 to 4·07)	3·75 (3·59 to 3·91)	3·58 (3·43 to 3·74)	3·53 (3·37 to 3·69)	3·41 (3·26 to 3·57)	3·32 (3·18 to 3·48)	3·31 (3·16 to 3·46)
Denmark	2·58 (2·41 to 2·77)	2·49 (2·33 to 2·67)	2·36 (2·2 to 2·52)	2·21 (2·06 to 2·36)	2·15 (2 to 2·3)	2·07 (1·93 to 2·23)	1·98 (1·84 to 2·13)	1·91 (1·77 to 2·06)	1·82 (1·68 to 1·97)	1·72 (1·58 to 1·87)
Finland	1·78 (1·72 to 1·84)	1·79 (1·73 to 1·85)	1·77 (1·71 to 1·82)	1·71 (1·65 to 1·76)	1·63 (1·58 to 1·69)	1·53 (1·49 to 1·58)	1·48 (1·43 to 1·52)	1·55 (1·5 to 1·6)	1·62 (1·57 to 1·67)	1·58 (1·53 to 1·63)
France	4·7 (4·59 to 4·8)	4·64 (4·54 to 4·75)	4·48 (4·38 to 4·58)	4·28 (4·19 to 4·37)	4·37 (4·28 to 4·47)	4·44 (4·34 to 4·54)	4·13 (4·03 to 4·22)	4·08 (3·98 to 4·17)	3·96 (3·87 to 4·05)	3·7 (3·61 to 3·79)
Germany	2·11 (2·09 to 2·13)	2·12 (2·1 to 2·14)	2·03 (2·01 to 2·05)	1·97 (1·95 to 1·99)	1·94 (1·92 to 1·95)	1·91 (1·9 to 1·93)	1·87 (1·86 to 1·89)	1·87 (1·85 to 1·88)	1·85 (1·83 to 1·86)	1·78 (1·76 to 1·79)
Greece	3 (2·94 to 3·06)	2·87 (2·81 to 2·93)	2·81 (2·76 to 2·87)	2·73 (2·67 to 2·79)	2·63 (2·58 to 2·69)	2·55 (2·49 to 2·6)	2·5 (2·45 to 2·56)	2·33 (2·28 to 2·38)	2·24 (2·2 to 2·29)	2·13 (2·08 to 2·17)
Iceland	2·04 (1·83 to 2·26)	1·97 (1·77 to 2·19)	1·87 (1·67 to 2·07)	1·74 (1·57 to 1·93)	1·71 (1·54 to 1·89)	1·65 (1·49 to 1·82)	1·62 (1·46 to 1·79)	1·57 (1·42 to 1·73)	1·53 (1·38 to 1·69)	1·5 (1·36 to 1·66)
Ireland	3·41 (3·23 to 3·58)	3·37 (3·2 to 3·54)	3·32 (3·15 to 3·49)	3·29 (3·13 to 3·46)	3·24 (3·08 to 3·4)	3·23 (3·08 to 3·39)	3·13 (2·98 to 3·29)	3·06 (2·91 to 3·21)	2·89 (2·75 to 3·03)	2·67 (2·54 to 2·8)
Israel	2·68 (2·47 to 2·91)	2·7 (2·5 to 2·93)	2·7 (2·49 to 2·92)	2·72 (2·51 to 2·94)	2·71 (2·52 to 2·93)	2·68 (2·49 to 2·88)	2·71 (2·51 to 2·92)	2·7 (2·51 to 2·92)	2·62 (2·44 to 2·82)	2·56 (2·38 to 2·77)
Italy	3 (2·77 to 3·27)	2·9 (2·67 to 3·15)	2·72 (2·49 to 2·96)	2·64 (2·43 to 2·87)	2·43 (2·24 to 2·62)	2·42 (2·24 to 2·61)	2·31 (2·13 to 2·48)	2·08 (1·93 to 2·23)	1·94 (1·79 to 2·08)	1·84 (1·71 to 1·97)
Luxembourg	2·16 (1·9 to 2·45)	2·08 (1·84 to 2·35)	2·17 (1·92 to 2·45)	2·1 (1·86 to 2·36)	1·98 (1·75 to 2·23)	2·17 (1·92 to 2·45)	2·13 (1·88 to 2·4)	2·1 (1·87 to 2·37)	2·01 (1·78 to 2·26)	1·92 (1·7 to 2·17)
Malta	3·45 (3·41 to 3·49)	3·19 (3·16 to 3·23)	2·98 (2·95 to 3·01)	2·99 (2·95 to 3·02)	2·9 (2·87 to 2·93)	2·81 (2·78 to 2·84)	2·83 (2·8 to 2·86)	2·77 (2·74 to 2·8)	2·84 (2·81 to 2·87)	2·79 (2·76 to 2·82)
Netherlands	4·78 (4·21 to 5·41)	4·51 (4·01 to 5·01)	4·24 (3·82 to 4·72)	3·96 (3·54 to 4·37)	3·56 (3·2 to 3·93)	3·18 (2·88 to 3·51)	2·88 (2·59 to 3·19)	2·65 (2·39 to 2·93)	2·43 (2·2 to 2·69)	2·38 (2·13 to 2·63)
Norway	2·23 (2·15 to 2·31)	2·22 (2·14 to 2·3)	2·21 (2·14 to 2·29)	2·19 (2·12 to 2·27)	2·14 (2·07 to 2·21)	2·11 (2·04 to 2·18)	2·05 (1·97 to 2·12)	2·01 (1·94 to 2·08)	1·94 (1·87 to 2·01)	1·86 (1·8 to 1·93)
Portugal	3·16 (3·09 to 3·23)	3·05 (2·98 to 3·12)	2·96 (2·89 to 3·03)	2·86 (2·8 to 2·93)	2·82 (2·75 to 2·88)	2·7 (2·63 to 2·76)	2·63 (2·57 to 2·69)	2·44 (2·39 to 2·5)	2·35 (2·3 to 2·4)	2·24 (2·19 to 2·29)
Spain	2·16 (2·04 to 2·28)	2·05 (1·94 to 2·16)	1·92 (1·82 to 2·03)	1·81 (1·72 to 1·91)	1·75 (1·66 to 1·85)	1·65 (1·57 to 1·74)	1·65 (1·57 to 1·74)	1·64 (1·55 to 1·72)	1·6 (1·52 to 1·68)	1·54 (1·46 to 1·62)
Sweden	2·16 (2 to 2·33)	2·17 (2·01 to 2·34)	2·18 (2·02 to 2·34)	2·16 (2·01 to 2·32)	2·15 (2 to 2·3)	2·18 (2·03 to 2·34)	2·1 (1·96 to 2·26)	2·05 (1·91 to 2·2)	2·02 (1·87 to 2·18)	1·9 (1·75 to 2·05)
Switzerland	2 (1·9 to 2·11)	1·96 (1·86 to 2·07)	1·92 (1·82 to 2·03)	1·91 (1·81 to 2·03)	1·93 (1·82 to 2·05)	1·95 (1·83 to 2·07)	1·91 (1·81 to 2·03)	1·86 (1·76 to 1·97)	1·86 (1·75 to 1·97)	1·84 (1·72 to 1·95)
**United Kingdom**	4·84 (4·72 to 4·96)	4·75 (4·63 to 4·87)	4·68 (4·57 to 4·8)	4·67 (4·55 to 4·78)	4·6 (4·48 to 4·71)	4·52 (4·41 to 4·63)	4·44 (4·33 to 4·54)	4·43 (4·33 to 4·54)	4·34 (4·24 to 4·44)	4·3 (4·2 to 4·4)
[truncated: 600,624 more chars]
